# The Role of Programmed Cell Death Regulator *LSD1* in Nematode-Induced Syncytium Formation

**DOI:** 10.3389/fpls.2018.00314

**Published:** 2018-03-19

**Authors:** Mateusz Matuszkiewicz, Miroslaw Sobczak, Javier Cabrera, Carolina Escobar, Stanislaw Karpiński, Marcin Filipecki

**Affiliations:** ^1^Department of Plant Genetics, Breeding, and Biotechnology, Warsaw University of Life Sciences – SGGW, Warsaw, Poland; ^2^Department of Botany, Warsaw University of Life Sciences – SGGW, Warsaw, Poland; ^3^Facultad de Ciencias Ambientales y Bioquímica, Universidad de Castilla-La Mancha, Toledo, Spain

**Keywords:** programmed cell death, PCD, *lsd1*, plant-parasitic nematode, RNA-sequencing, *Arabidopsis*

## Abstract

Cyst-forming plant-parasitic nematodes are common pests of many crops. They inject secretions into host cells to induce the developmental and metabolic reprogramming that leads to the formation of a syncytium, which is the sole food source for growing nematodes. As in other host-parasite models, avirulence leads to rapid and local programmed cell death (PCD) known as the hypersensitive response (HR), whereas in the case of virulence, PCD is still observed but is limited to only some cells. Several regulators of PCD were analyzed to understand the role of PCD in compatible plant–nematode interactions. Thus, *Arabidopsis* plants carrying recessive mutations in *LESION SIMULATING DISEASE1* (*LSD1*) family genes were subjected to nematode infection assays with juveniles of *Heterodera schachtii*. LSD1 is a negative and conditional regulator of PCD, and fewer and smaller syncytia were induced in the roots of *lsd1* mutants than in wild-type Col-0 plants. Mutation in *LSD ONE LIKE2* (*LOL2*) revealed a pattern of susceptibility to *H. schachtii* antagonistic to *lsd1.* Syncytia induced on *lsd1* roots compared to Col0 showed significantly retarded growth, modified cell wall structure, increased vesiculation, and some myelin-like bodies present at 7 and 12 days post-infection. To place these data in a wider context, RNA-sequencing analysis of infected and uninfected roots was conducted. During nematode infection, the number of transcripts with changed expression in *lsd1* was approximately three times smaller than in wild-type plants (1440 vs. 4206 differentially expressed genes, respectively). LSD1-dependent PCD in roots is thus a highly regulated process in compatible plant–nematode interactions. Two genes identified in this analysis, coding for AUTOPHAGY-RELATED PROTEIN 8F and 8H were down-regulated in syncytia in the presence of LSD1 and showed an increased susceptibility to nematode infection contrasting with *lsd1* phenotype. Our data indicate that molecular regulators belonging to the *LSD1* family play an important role in precise balancing of diverse PCD players during syncytium development required for successful nematode parasitism.

## Introduction

The development of feeding structures such as syncytia and giant cells, which are induced in plant roots upon infection with cyst-forming and root-knot nematodes, respectively, is a very complex process. Successful parasitism by nematodes consists of several steps, including plant localization, root invasion, migration, suppression of plant defense responses, feeding site induction and development, and nematode reproduction. Plant cells, however, react to nematode invasion either by activating defense response pathways, which limit the parasite by the hypersensitive response (HR), or by allowing parasite development through formation of the feeding structure. Programmed cell death (PCD) is involved in both processes but its intensity and timing is different (Sobczak et al., 2005; Sobczak and Golinowski, 2011).

In the case of compatible plant–nematode interactions, the development of a syncytium begins with the selection of an initial syncytial cell (ISC) located in the procambium or in the pericycle (Sobczak and Golinowski, 2009). The criteria by which nematodes select ISCs are still largely unknown. If an ISC is successfully selected, the juvenile nematode uses a hollow stylet to inject it with a protein cocktail produced in the esophageal gland cells. Nematode secretions are injected directly into the cytoplasm of the ISC and by diverse molecular mechanisms provoke a profound developmental reprogramming that leads to the formation of a feeding structure. A number of effectors have been described for both cyst and root-knot nematodes (Mitchum et al., 2013; Quentin et al., 2013; Eves-van den Akker et al., 2014; Niu et al., 2016; Mantelin et al., 2017); for example, infective juveniles of *Heterodera schachtii* secrete HsCLE1 and two effectors (CLAVATA 1 and 2-like genes) that modify hormone homeostasis (Wang et al., 2011), Hs4F01, which changes the defense response by manipulation of an oxidoreductase of the 2OG-Fe(II) oxygenase family (Patel et al., 2010); and Hs25A01, which modifies plant growth by interacting with chalcone synthase and the translation initiation factor eIF-2β subunit (eIF-2bs) (Pogorelko et al., 2016).

A specific set of anatomical and ultrastructural changes occurs in cells modified by juvenile nematodes during the development of the feeding site (syncytium or giant cell). Starting from the onset of nematode parasitism, selected ISCs or induced giant cells grow intensively and become hypertrophied. Mitochondria, plastids, endoplasmic reticulum, lipid bodies, and ribosomes proliferate. Cell walls uniformly thicken and are locally dissolved in syncytia. The volume of the nuclei also increases and the central vacuole is replaced with numerous smaller ones. In *Arabidopsis*, a syncytium is formed inside the vascular cylinder only by fusion of several tens of modified parenchymatous cells (Sobczak and Golinowski, 2009).

Although nematodes are widely known as common plant parasites that cause tremendous yield losses, methods for their control are neither highly efficient nor commonly used. Plant breeding for resistance is the most effective and sustainable method of control, although the repertoire of nematode resistance loci is rather limited (Fuller et al., 2008; Tomczak et al., 2009). The decline in use and withdrawal of pesticides requires the development of new strategies for nematode control and management. In this context, better understanding of plant reactions to nematode infection is crucial. The current widespread use of genomics will identify novel control targets or allow the verification of older models (Williamson and Kumar, 2006; Jones et al., 2013).

Transcriptomic studies revealed massive modifications of gene expression underlying the developmental switch to syncytium formation. These studies indicate the engagement of pathways involved in cell cycle regulation, cell wall rearrangement, hormonal biosynthesis, and signaling during plant–nematode interactions (Barcala et al., 2010; Cabrera et al., 2014). Interesting functional classes of genes responding to nematode infections are those involved in reactive oxygen species (ROS) homeostasis and PCD. The roles and functions of these genes are usually analyzed with respect to the HR of plants during incompatible (resistant) interactions with avirulent pathogens, where plant defense mechanisms surround pathogens with a layer of dead cells and thus limit their spread through plant tissues. The quantity and amplitude of ROS production differ in compatible plant-cyst nematode interactions. Large increments in ROS production can damage cells and inhibit nematode development, but ROS also act as signaling molecules to orchestrate cellular events essential for cell growth, development, and stress responses (Feng and Shan, 2014). Nematodes trigger ROS production in infected root tissue with a dualistic role. ROS generated by respiratory burst oxidase homologs (Rbohs) positively regulate infection processes and promote nematode growth during the early infection stages. Cell death adjacent to the site of nematode infection is enhanced in the *atrbohD/F* mutant, suggesting that nematodes stimulate ROS, limiting the activation of plant defense responses and controling the balance of ROS production toward the development of syncytium (Siddique et al., 2014). Hence, PCD seems to play different roles during the migratory phase of nematode parasitism, syncytium induction, and its successive growth.

The discovery of the molecular, physiological, and genetic mechanisms of PCD involved at different hierarchical levels of biological organization was facilitated by the identification of *Arabidopsis thaliana* mutants with deregulated PCD (Dietrich et al., 1994; Lorrain et al., 2003; Vandenabeele et al., 2004; Moeder and Yoshioka, 2008; Bruggeman et al., 2015). One of the best-studied mutants, in terms of PCD, is *lsd1*, which lacks functional LSD1, a protein of yet unknown molecular function. The *lsd1* phenotype is characterized by so-called runaway cell death (RCD) in leaves, which manifests as an inability to restrict PCD propagation once it has been initiated. The uncontrolled systemic spread of foliar RCD in *lsd1* plants can be provoked by either abiotic factors, such as excess light or red light (Mateo et al., 2004; Chai et al., 2015), root hypoxia, impeded stomatal conductance (Mühlenbock et al., 2007, 2008), low temperature (Huang et al., 2010), drought (Wituszyńska et al., 2013; Szechyńska-Hebda et al., 2016), and UV radiation (Wituszyńska et al., 2015), or by biotic factors, such as bacterial infection (Dietrich et al., 1994; Rustérucci et al., 2001). Therefore, LSD1 was proposed to function as a negative PCD regulator that integrates various signaling pathways in response to both biotic and abiotic stresses (Karpiński et al., 2013). Importantly, the *lsd1* RCD phenotype depends on ENHANCED DISEASE SUSCEPTIBILITY 1 (EDS1) and PHYTOALEXIN DEFICIENT 4 (PAD4), two proteins that were originally described as components of basal disease resistance (Parker et al., 1996; Glazebrook et al., 1997). It was proven that they are essential for RCD in *lsd1* plants, since RCD was reverted in the double mutants *eds1*/*lsd1* and *pad4*/*lsd1* (Rustérucci et al., 2001; Mateo et al., 2004). Moreover, LSD1 and EDS1/PAD4 elicit opposite effects on foliar ROS and salicylic acid (SA) levels (Mühlenbock et al., 2008; Huang et al., 2010; Wituszyńska et al., 2013). Therefore, LSD1 is considered as a negative regulator of EDS1- and PAD4-dependent RCD.

Our main objective was to increase the knowledge on the role of PCD during syncytium formation. We analyzed several lines of *Arabidopsis* with mutations in PCD-related genes following infection with beet cyst nematode (*H. schachtii*). One of these *Arabidopsis* mutants, *lsd1*, where PCD is impaired, was used for the RNA-sequencing analysis of infected and uninfected roots. We correlated dramatic changes in transcriptome profiles with the ultrastructural changes occurring during syncytium development.

## Materials and Methods

### Plant Material and Growing Conditions

Seeds of *A. thaliana* L. Heynh. ecotypes Columbia (Col0) and Wassilewskaja (Ws0), and mutant lines *lsd1*(Col0 background), *lsd1*(Ws0 background), *lol1*(Ws0 background) (Dietrich et al., 1997), *eds1*(Ws0 background), *pad4*(Ws0 background) (Parker et al., 1996; Zhou et al., 1998), *lol2*(Col0 background) (Sail_1288_E09; N879262), *atg8 f* (Col0 background) (Salk_057021C; N653221), and *atg8 h* (Col0 background) (Salk_119920C; N671962) were used in experiments. Seeds were surface-sterilized in 0.7% NaClO for 5 min and 70% ethanol (EtOH) for 1 min. Afterward they were rinsed five times in ddH_2_O. Two seeds were placed on modified KNOP medium supplemented with 2% sucrose in a 90 mm diameter Petri dish (Sijmons et al., 1991) and grown under a light and temperature regime of 8 h light:16 h dark (SD 8:16) and 22:20°C. Light intensity was 90 μmol m^-2^ s^-1^, although the irradiance was increased to 155 μmol m^-2^ s^-1^ (non-permissive light intensities) to induce RCD on leaves.

### Nematode Infection Assay

*Heterodera schachtii* Schmidt cysts were harvested from *in vitro* stock cultures produced aseptically on white mustard (*Sinapis alba* cv. *Albatros*) roots grown on 0.2 KNOP medium. Hatching of juveniles was stimulated by incubating the cysts in 3 mM ZnCl_2_ (Sijmons et al., 1991). Second stage juveniles (J2s) were collected 6–7 days later, sterilized in 0.05% HgCl_2_ for 5 min, and immediately washed five times in dH_2_O. Fourteen-day-old *Arabidopsis* plants were inoculated with 80–100 J2s under sterile conditions. Inoculated plates were kept in the dark for 24 h, and thereafter transferred into a growth chamber under SD 8:16 photoperiods. The experiments were repeated three times with 10 plants per genotype in one replicate.

The numbers of males and females per plant, and sizes of syncytia, and the associated female nematodes were counted and measured at 14 days post-inoculation (dpi). For each line, 50 syncytia associated with females were randomly selected and photographed using a Leica M165C stereomicroscope (Leica Microsystems, Wetzlar, Germany) equipped with a Leica DFC 425 digital camera. The syncytia and females were outlined using the Leica Application Suite software (V3.8). The individual measurements were used to calculate the average size of each syncytium and female.

Data were analyzed using *t*-tests (*p* < 0.05) or single-factor ANOVA (*p* < 0.05). In the case of ANOVA, if the *F*-statistic was higher than *F*-critical, Fisher’s Least Significant Difference (LSD) test was applied.

### Ultrastructural Analysis

*Arabidopsis* plants were grown and inoculated with *H. schachtii* as described above. Samples of uninfected roots and roots containing syncytia were collected from wild-type (Col0) and *lsd1* mutant plants at 7 and 12 dpi, and processed for light and transmission electron microscopy examinations, as described by Golinowski et al. (1996) and Sobczak et al. (1997). Sections were cut using a Leica UCT ultramicrotome, stained with uranyl acetate and lead citrate, and examined using a FEI 268 D ‘Morgagni’ (FEI Comp., Hillsboro, OR, United States) transmission electron microscope equipped with an SIS ‘Morada’ (Olympus SIS, Münster, Germany) digital camera.

### RNA Extraction for Transcriptomic Analysis

Total RNA was isolated at 12 dpi from root segments containing syncytia and from uninfected roots of wild-type (Col0) and *lsd1* plants using the Universal RNA Purification Kit (Eurx, Gdańsk, Poland), according to the manufacturer’s protocol, with on-column digestion of DNA. RNA yield and purity were estimated using the NanoDrop ND-1000 (NanoDrop Products, Wilmington, DE, United States) and the Experion (Bio-Rad, Miasto, CA, United States). Total RNA with RQI values ≥9.0 and 28S:18S ratios ≥1.2 was used in the RNA-sequencing analysis.

### RNA-Sequencing Analysis

The Illumina HiSeq2500 platform (Illumina Inc., San Diego, CA, United States) was used for RNA-sequencing (RNA-seq) analysis. To obtain a comprehensive overview of the *A. thaliana* root transcriptome and transcript profiles under *H. schachtii* parasitism, the libraries were constructed in three biological replicates and paired-end sequencing was carried out by Genomed SA (Warsaw, Poland). Quality control of sequencing was evaluated using FastQC software (V0.10.1). High quality reads were mapped to the *A. thaliana* TAIR10 reference genome^1^ by using TopHat2 software tools with the default values (Kim et al., 2013). Reads per gene were counted using the HTSeq framework (Anders et al., 2015). Gene expression analysis was performed using R software (R Core Team, 2014). Bioconductor (Gentleman et al., 2004), and DESeq2 packages (Love et al., 2014) from the SARTools package (Varet et al., 2016). Normalization and differential analysis were carried out according to the DESeq2 model (Love et al., 2014). The sequencing data are accessible in SRA database under accession number PRJNA407426.

Genes differentially expressed between Col0 and *lsd1* were classified into MapMan BINs against the *Arabidopsis* TAIR10 database, and their annotated functions were visualized using the MapMan tool (Thimm et al., 2004). GO enrichment analyses were performed using GOrilla software (Eden et al., 2009). Amino acid sequences of differentially expressed NB-LRR sequences were downloaded from the NCBI database, and subjected to phylogenetic analysis by the Neighbor-Joining method using the CLC Genomics Workbench (Qiagen GmbH, Hilden, Germany).

### Quantitative Real-Time RT-PCR (qRT-PCR)

Total RNA was isolated using the same procedure as for RNA-seq analysis. Total RNA (1 μg) was reverse transcribed using random hexamer primers following the manufacturer’s protocol for the QuantiTect Reverse Transcription Kit (Qiagen GmbH, Hilden, Germany). Quantitative RT-PCR was performed in triplicate using the Bio-Rad CFX96 Touch^TM^ Real-Time PCR Detection System (Bio-Rad, Hercules, CA, United States) with the primers listed in Supplementary Figure S5, according to the QuantiTect SYBR Green PCR Kit manual (Qiagen).

Real-time PCR cycling conditions were as follows: 5 min denaturation at 95°C and 40 cycles of amplification (15 s at 95°C, 30 s at 58°C, and 30 s at 72°C). Relative expression levels were calculated using the expression of *actin 2* as an internal reference, according to the ΔΔ*C*t method (Livak and Schmittgen, 2001). Significant differences in expression in comparison to the control were revealed using the REST tool (Pfaffl et al., 2002). Product melting curves were generated following PCR to ensure purity of the amplification products. A list of all primers used is included in the supplementary materials (Supplementary Figure S5).

## Results

### Light Intensity Influences Nematode Parasitism in the Roots of *lsd1* Mutants

To determine if PCD regulators were involved in the development of feeding structures in *Arabidopsis* roots, infection tests were performed in loss of function lines of the *LSD1* gene family. The assumption that the deregulation of PCD in *lsd1* may influence nematode infection and syncytium development on the root systems was verified under permissive and non-permissive light intensities in a short-day growth regime. The non-permissive conditions, which produced the visible RCD symptoms on *lsd1* mutant leaves, differed depending on the genetic background of the mutant and were 120 μmol m^-2^ s^-1^ for the Col0 background and 155 μmol m^-2^ s^-1^ for the Ws0 background.

**FIGURE 1:**
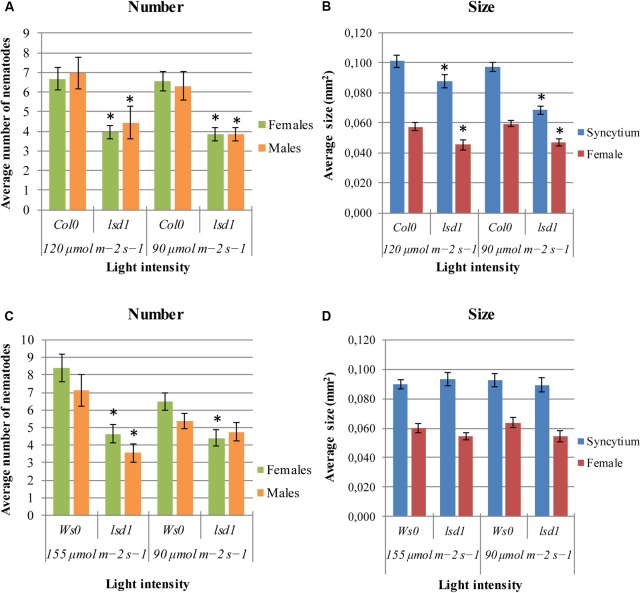
Development of *Heterodera schachtii* and nematode-induced syncytia in roots of *lsd1* mutants and wild-type (Col0 and Ws0) *Arabidopsis* plants grown under different light conditions. **(A,C)** Average numbers of females and males that developed by 14 dpi. **(B,D)** Average sizes of syncytia and associated females measured at 14 dpi. Data represent means (±SEM) from three independent experiments, each containing 10 plants per genotype. Data were analyzed using *t*-tests. Asterisk: significant difference from wild-type plants (*p* < 0.05).

The *lsd1*(Col0) mutants were less susceptible to nematodes under any of two conditions of applied irradiation (**Figure 1A**). The significant differences between genotypes were also observed in syncytium size, which was 30% lower in *lsd1* than in Col0, and in female size, by 21% (**Figure 1B**). The differences in susceptibility between Ws0 wild-type and *lsd1* plants were observed following irradiation with 155 μmol m^-2^ s^-1^ and overlapped with clear signs of RCD on the rosette. A slight effect was seen in Ws0 background plants at 90 μmol m^-2^ s^-1^ (permissive conditions for both genotypes) which was best visualized as a comparison of female/male proportion, which was 1.21 in Ws0 and 0.93 in *lsd1* mutants. The light intensity of 155 μmol m^-2^ s^-1^ gave quite strong differences – approximately 40% reduction in susceptibility in the mutant. The differences in susceptibility in the Ws0 background were not reflected by changes in syncytium size irrespective of light intensity (**Figures 1C,D**).

### Nematode Parasitism Changes in *lsd1*-Related Mutants

**FIGURE 2:**
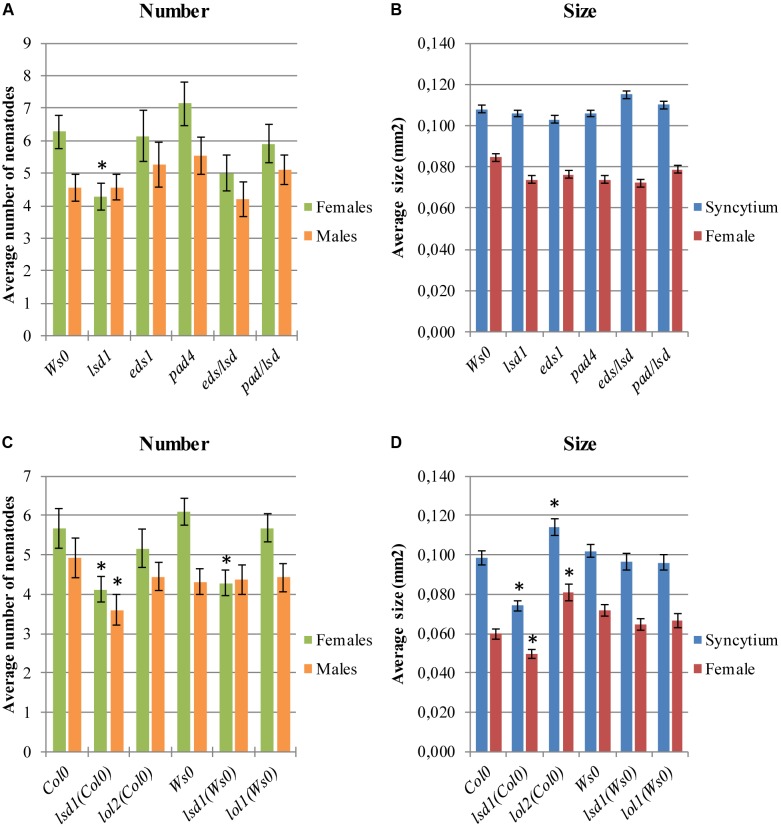
Development of *H. schachtii* and nematode-induced syncytia in roots of LSD1 dependent genes *lsd1*, *eds1*, *pad4*, *eds/lsd1*, *pad4/lsd1*, *lol1*, and *lol2* mutants, and wild-type (Col0 and Ws0). Mutants and wild-type (Col0 and Ws0) *Arabidopsis* plants were grown under 90 μmol m^-2^ s^-1^ light intensity. **(A,C)** Average numbers of females and males that developed by 14 dpi. **(B,D)** Average sizes of syncytia and associated females at 14 dpi. Data represent means (±SEM) from three independent experiments, each containing 10 plants per genotype. Data were analyzed using ANOVA. Asterisk: significant difference from wild-type plants (*p* < 0.05). Fisher’s Least Significant Difference (LSD) test was used for *post hoc* analysis.

The *lsd1* phenotype depends on EDS1 and PAD4, two components of basal disease resistance (Parker et al., 1996; Glazebrook et al., 1997). The *eds1* and *pad4* single mutants in a Ws0 background showed similar susceptibility to nematode infection as wild-type plants. Both the *eds1/lsd1* and *pad4/lsd1* double mutants reversed the effect of the single *lsd1* mutation (**Figures 2A,B**), confirming the mentioned dependency.

LSD1 is a zinc-finger protein with three zinc-finger motifs. The *Arabidopsis* genome contains two close homologs, LOL1 and LOL2 (Dietrich et al., 1997). Analyses of *lol1* and *lol2* mutants show LOL1 is negatively regulated by LOL2 (Epple et al., 2003). Thus, cell death appears to be regulated by the balance between LSD1, LOL1, and LOL2, with LSD1 acting as a negative regulator of PCD and LOL1 as a positive regulator that is itself negatively regulated by LOL2 (Coll et al., 2011). In nematode infection tests, *lol2* mutants behaved similarly to wild-type plants in terms of susceptibility, expressed as the number of developed females and the female/male ratio (**Figure 2C**). The average size of syncytia and females found in *lol2* mutants, however, showed opposite trends to those of *lsd1* plants (**Figure 2D**). The *lol1* mutant did not show significant differences to the control in terms of susceptibility and syncytium/female size.

### Ultrastructural Changes in *lsd1* Syncytia

**FIGURE 3:**
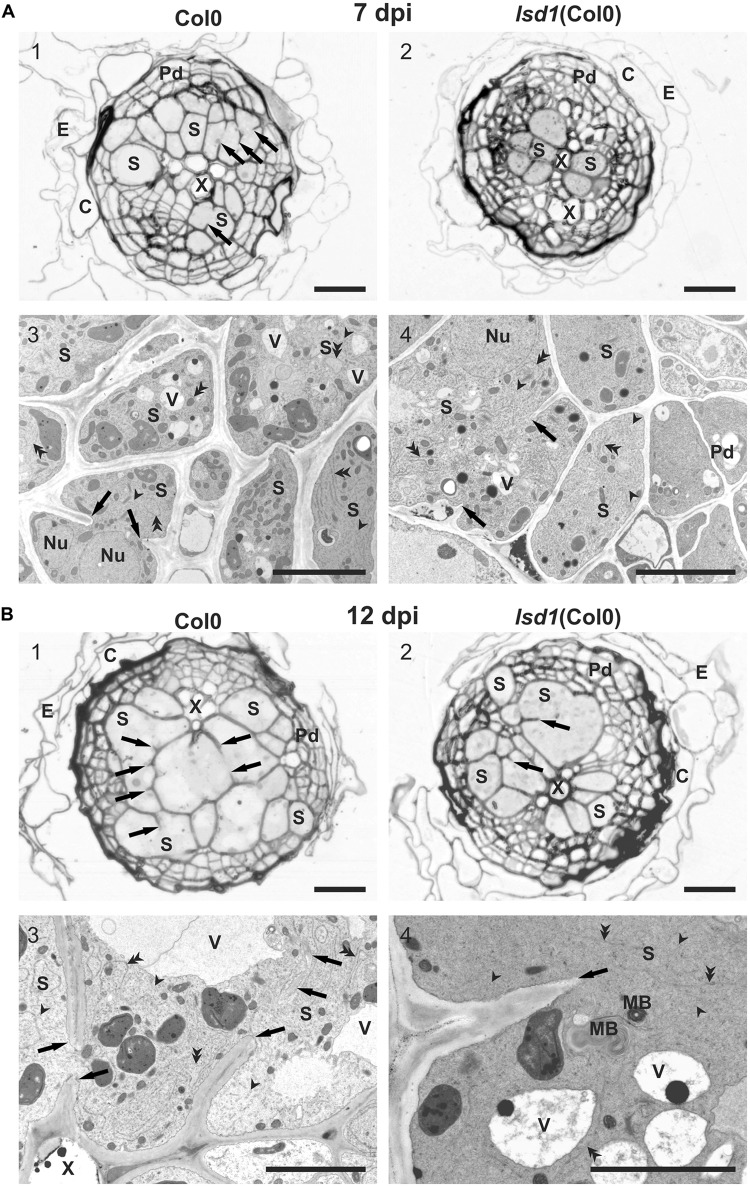
Anatomy and ultrastructure of syncytia induced in wild-type (Col0) and *lsd1* mutant roots. **(A)** Light **(A1,A2)** and transmission electron microscopy **(A3,A4)** images of cross-sections of syncytia at 7 dpi induced in Col0 **(A1,A3)** and *lsd1* **(A2,A4)** roots. **(B)** Light **(B1,B2)** and transmission electron microscopy **(B3,B4)** images of cross-sections of syncytia at 12 dpi induced in Col0 **(B1,B3)** and *lsd1* **(B2,B4)** roots. Arrowheads point to selected tubular structures of the ER. Double arrowheads indicate selected cisternae of the ER. C, cortex; E, epidermis; Nu, nucleus; MB, myelin-like body; Pd, periderm-like tissue; S, syncytial element with dense electron cytoplasm; X, xylem vessel; V, vacuole. Arrowheads indicate cell wall stubs flanking cell wall openings. Scale bars: 20 μm **(A1,A2,B1,B2)**; 5 μm **(A3,A4,B3,B4)**.

Infective J2s were able to induce syncytia in the roots of wild-type (Col0) and *lsd1*(Col0) plants. Syncytium anatomy was typical of that described for *Arabidopsis* (Golinowski et al., 1996). Syncytia were localized in the center of the vascular cylinder and surrounded by dividing pericyclic cells forming a periderm-like cover tissue. The cortex and epidermis were degraded and shed out (**Figure 3**). The anatomy of syncytia induced in both genotypes was similar at 7 dpi, but the feeding structures induced in *lsd1* roots were smaller (**Figures 3A1,A2**). The wild-type and *lsd1* syncytia were composed of distinctly enlarged procambial cells with only a few cell wall openings (**Figures 3A1,A2**). Syncytial protoplasts had a typical organization with an electron dense cytoplasm, numerous tubular and cisternal structures of the endoplasmic reticulum, and hypertrophied nuclei (**Figures 3A3,A4**). The central vacuoles in syncytial elements had re-differentiated into small vesicles (vacuoles). At 12 dpi, syncytia induced in roots of the *lsd1* mutant were clearly smaller in transverse section (**Figures 3B1,B2**). They were composed of fewer, less hypertrophied elements derived from cambial or procambial cells. In addition, the number and extent of cell wall openings were lower (**Figures 3B1,B2** and Supplementary Figure S8). The ultrastructural organization was similar to, and typical of, syncytia induced in wild-type *Arabidopsis* plants; however, syncytia induced in *lsd1* roots contained smaller, more numerous vacuoles and other vesicles (**Figures 3B3,B4**). The most characteristic was the presence of numerous myelin-like (multilamellar) bodies of different sizes in *lsd1* syncytia (**Figure 3B4**).

### The Transcriptome Is Extensively Deregulated in Nematode-Infected *lsd1*

**FIGURE 4:**
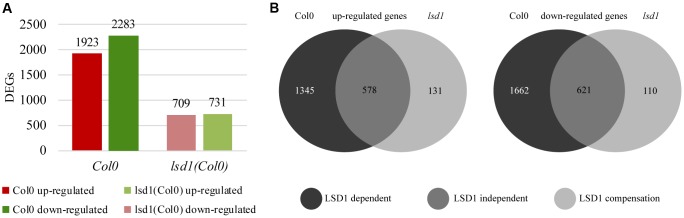
Number of differentially expressed genes (DEGs; infected vs. uninfected) at 12 dpi in syncytia induced in wild-type (Col0) and *lsd1* mutant roots. **(A)** Total number of DEGs showing reduction in gene regulation complexity on nematode infection. **(B)** Venn diagrams showing DEGs grouped according to changes in their expression relative to uninfected roots. **(B)** Left diagram: up-regulated genes. **(B)** Right diagram: down-regulated genes. The Venn diagrams classify DEGs into three functional groups based on *LSD1* activity. The “LSD1-dependent group” encompasses transcripts differentially expressed in Col0 only (in which *LSD1* is active); the “LSD1-independent group” encompasses transcripts up- or down-regulated irrespective of the *LSD1*mutation; the “LSD1-compensation group” encompasses transcripts with changed expression only when *LSD1* is mutated.

A transcriptomic approach was used to investigate the functions of LSD1 in PCD-related regulatory networks active in nematode-infected roots, and the RNA-seq data were validated by RT-qPCR of 11 genes showing a high degree of concordance (Supplementary Figure S6). The first striking observation was that, despite the quite mild phenotype in terms of nematode infection (**Figures 1**–**3**), the number of differentially expressed transcripts in syncytia at 12 dpi was almost three times lower in infected *lsd1* than in infected wild-type roots [1440 vs. 4206 differentially expressed genes (DEGs), respectively] (**Figure 4A**).

RNA-seq analysis of syncytia induced in control Col0 and *lsd1* mutant plants allowed us to divide the DEGs into three distinct groups (**Figure 4B**). The largest group contained “LSD1-dependent” genes, which encompassed genes up- or down-regulated in syncytia induced in wild-type plants only. This group consisted of 1345 up-regulated and 1662 down-regulated genes. The second group, “LSD1-independent,” contained genes that showed similar changes in expression in both *lsd1* and wild-type (Col0) plants. This group consisted of 578 up-regulated and 621 down-regulated genes. The third group consisted of genes whose expression changed only in syncytia induced in the mutant plants. As their expression could be related to the compensation effect due to the lack of LSD1 activity, we named this group the “LSD1-compensation group.” It consisted of 131 up- and 110 down-regulated genes. All these results are displayed in Venn diagrams (**Figure 4B**). These data indicate that the presence of a functional *LSD1* gene increases the complexity of the plant responses to nematode parasitism.

Interestingly, expression of only a few genes changed upon nematode infection from up- to down-regulated (or vice versa) depending on the genetic background of the plants. These were WAT1-related protein and serine carboxypeptidase-like 30, changing from 0.9 log2FC to -1.2 log2FC and 2.0 log2FC to -1.7 log2FC, respectively, and a glycosyl hydrolase family protein with chitinase interaction domain, changing from -1.0 log2FC to 1.6 log2FC (Supplementary Tables S1, S2). The analysis of expression dynamics within defined gene groups shows quite a high range of regulation. Changes in gene expression levels in the “LSD1-dependent group” ranged from 6.5 log2FC (for myo-inositol oxygenase 5) to -5.5 log2FC (mannose-binding lectin superfamily protein) (Supplementary Table S1). Our analyses identified some genes not previously associated with nematode parasitism among the most strongly regulated transcripts (**Table 1**; Szakasits et al., 2009). These include alternative oxidase 1D (AOX1D; 1.8 log2FC) and nuclear transport factor 2 (NTF2; -4.3log2FC), which may be more directly linked to PCD-related processes. The DEGs also include a number of genes that were previously known as important players in plant–nematode interactions (Supplementary Tables S1, S2).

**Table 1:** Changes in the level of expression of transcripts newly identified as involved in nematode parasitism.

Group	Gene_ID	Description	log2FC	*p*-value
**LSD1 dependent**	AT3G54700	*PHT1:7*	5.53	3.34E-35
	AT4G13260	*YUC2*	4.32	1.66E-14
	AT1G50960	*GA2OX7*	3.50	1.77E-09
	AT1G07430	*HAI2*	3.49	4.37E-24
	AT1G25410	*IPT6*	3.22	1.71E-07
	AT4G39000	*GH9B17*	-4.11	1.45E-13
	AT4G15360	*CYP705A3*	-3.96	2.19E-12
	AT3G47710	*BNQ3*	-3.29	1.06E-15
	AT2G45050	*GATA2*	-3.19	2.28E-32
	AT4G37160	*SKS15*	-3.17	2.19E-13

**LSD1 independent**	AT1G68460	*IPT1*	5.05	1.73E-46
	AT1G08440	*ALMT2*	4.50	6.90E-39
	AT1G06030	*TPS7*	4.19	6.27E-17
	AT1G71890	*SUC5*	4.09	5.56E-21
	AT4G15210	*BAM5*	3.59	1.66E-10
	AT5G01740	*NTF2*	-4.32	8.68E-30
	AT4G15370	*BARS1*	-4.17	4.76E-16
	AT1G80340	*YUC9*	-3.84	2.93E-17
	AT1G65570	*RNA HELICASE*	-3.77	2.54E-15
	AT3G29430	*GGPS11*	-3.06	1.03E-34

**LSD1 compensation**	AT2G41240	*BHLH100*	4.01	1.68E-21
	AT2G44460	*BGLU28*	2.33	3.37E-06
	AT1G32350	*AOX1D*	1.83	2.43E-05
	AT5G43360	*PHT1:3*	1.71	1.07E-03
	AT5G56970	*CKX3*	1.62	1.80E-04
	AT2G47560	*ATL64*	-2.46	3.05E-12
	AT4G25960	*ABCB2*	-2.17	3.75E-08
	AT3G62740	*BGLU7*	-1.99	1.26E-05
	AT3G02493	*RTFL19*	-1.64	5.35E-04
	AT5G07030	*TUDOR1*	-1.63	6.71E-06


**Genes were divided into three groups based on the presence of functional LSD1*.*

Similarly, the “LSD1-independent” group included some genes not previously identified as involved in nematode parasitism (**Table 1**). This group encompassed genes whose expression changed upon nematode infection both in the presence and absence of functional *LSD1*. Some widely accepted PCD markers appeared in this group; however, the expression levels were often lower in mutants than in wild-type plants. This tendency was most evident among up-regulated genes; for example, the transcription of cytochrome P450 family 707, subfamily A, polypeptide 4 (CYP707A4) increased at 5.7 log2FC in infected Col0, but at only 2.2 log2FC in infected *lsd1*, and expression of mannose-binding lectin superfamily protein was strongly suppressed to -4.5 log2FC in infected Col0, but more weakly suppressed to -1.8 log2FC in infected *lsd1*. In some cases, however, the change in expression was greater in the mutant; for example, *NTF2* was strongly down-regulated in Col0 (-3.2 log2FC), but its expression was inhibited even more strongly in *lsd1* syncytia (-4.3 log2FC).

In the “LSD1-compensation” group, the range of changes in expression was between 4.0 and -2.4 log2FC (**Table 1**). Upon nematode infection, the most strongly up-regulated gene was that encoding a transcription factor, bHLH100, containing a basic helix-loop-helix protein motif. Nematode infection induced an opposite reaction in *ATL64*, which was the most strongly down-regulated gene in this group. In both cases, the change of expression in these genes has not been demonstrated in previous microarray-transcriptomic studies linked to plant-parasitic nematodes in *Arabidopsis* (Cabrera et al., 2014).

### Functional Classification of Nematode-Regulated Genes

The number of genes changing expression in response to nematode parasitism was quite large and covered a substantial portion of the genome. A functional classification of 5646 DEGs was carried out using the MapMan hierarchical ontology system (version 3.6.0RC1; Thimm et al., 2004). This allowed visualization of regulatory trends in biological pathways. The observed reduction in complexity of up- and down-regulated genes in syncytia induced in *lsd1* mutants appeared to be distributed proportionally between the functional categories (BINs) defined by MapManin a manner suggesting a pleiotropic effect (Supplementary Figures S1, S2).

**FIGURE 5:**
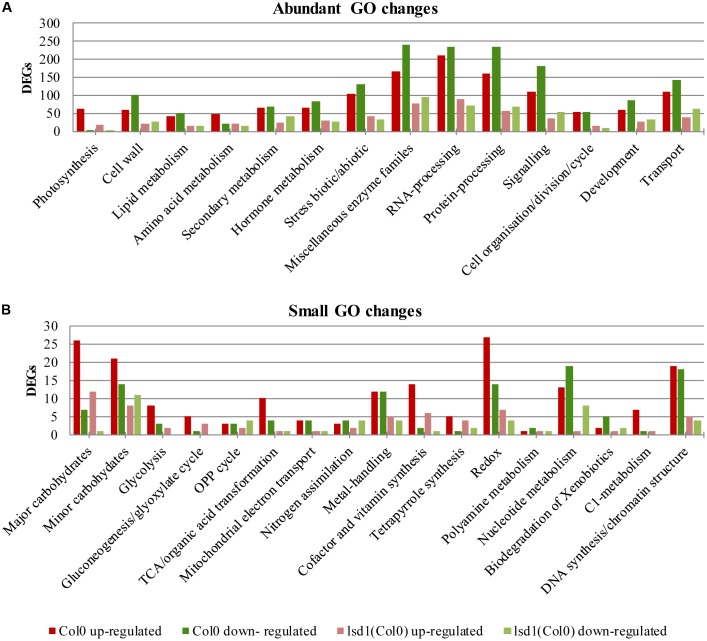
Overview of functional GO classification of transcripts differentially expressed in syncytia induced in wild-type (Col0) and *lsd1* mutant plants (classification produced using MapMan). **(A)** The most numerous categories of DEGs. **(B)** The least numerous categories of DEGs.

Comparing the numbers of up- and down-regulated genes in infected and uninfected wild-type and mutant roots across the functional categories showed that the most significant reductions in gene number occurred in categories such as “protein processing,” “RNA processing,” “signaling,” “biotic/abiotic stress,” and “cell wall modification.” The ratio between up- and down-regulated genes usually remained similar between the wild-type and mutant lines, except for “hormone metabolism,” “stress,” and “RNA processing.” In these categories, more genes were down-regulated than up-regulated in wild-type plants, but in *lsd1*mutants slightly greater numbers of genes were up-regulated (**Figures 5A,B**).

Because of the specific focus of the research conducted, we manually created the stress hormone related categories containing SA, JA, and ET markers, signaling components, and genes involved in hormone biosynthesis. Interestingly these categories are strongly biased toward gene up-regulation upon nematode parasitism. Moreover, the number of DEGs in the wild-type and mutant plants was almost equal, distinguishing these groups from the general trend of gene expression complexity reduction in *lsd1* plants (**Table 2** and Supplementary Figure S7).

**Table 2:** The nematode induced changes in salicylic acid (SA) related genes in 12 dpi syncytia of Col0 and *lsd1*(Col0) plants.

Gene_ID	Description	Log2FC		Log2FC	
			
		Col0	*p*-value	*lsd1*(Col0)	*p*-value
**SA related genes**					
At2g14610	*PR1*	-0.74	2.00E-01	**1.63**	**2.51E-05**
At3g57260	*PR2*	0.66	1.69E-01	**1.97**	**2.11E-04**
At1g75040	*PR5*	**1.29**	**2.65E-03**	**2.39**	**7.20E-06**
At3g48090	*EDS1*	-0.24	2.82E-01	0.11	6.85E-01
At4g39030	*EDS5*	**1.18**	**2.99E-06**	**0.73**	**8.05E-03**
At4g26120	*NPR1*	-0.06	8.44E-01	0.00	9.94E-01
At5g45110	*NPR3*	**0.64**	**2.15E-02**	0.45	2.43E-01
At4g19660	*NPR4*	-0.43	6.64E-02	-0.28	2.57E-01
At5g13320	*PBS3*	0.32	4.25E-01	0.79	1.24E-01
At5g67160	*EPS1*	**0.79**	**5.43E-03**	0.30	3.19E-01
At1g74710	*ICS1/EDS16*	-0.32	3.65E-01	0.46	1.23E-01
At1g18870	*ICS2*	**-0.82**	**6.08E-03**	**-1.80**	**8.40E-05**
At2g37040	*PAL1*	**0.90**	**5.99E-05**	**1.32**	**2.48E-03**
At3g53260	*PAL2*	**0.47**	**4.08E-02**	**1.25**	**2.84E-03**
At5g04230	*PAL3*	-0.27	5.97E-01	-0.25	6.33E-01
At3g10340	*PAL4*	**-0.44**	**4.09E-02**	-0.01	9.86E-01


**Genes are listed in the order: SA marker genes – PR1, PR2, PR5; SA signaling – EDS1, EDS5, NPR1, NPR3, NPR4, PBS3; SA biosynthesis – EPS1, ISC1/EDS16, ICS2, PAL1, PAL2, PAL3. Statistically significant gene expression changes in transcriptome analyses (p < 0.05) are in bold font*.*

Additionally, to pinpoint the biological processes affected by the *LSD1* mutation, gene ontology enrichment analysis was performed using the GOrilla tool (Eden et al., 2009). This revealed that “cell wall organization” was the most significantly enriched category of genes in syncytia induced in wild-type plants. Other categories such as “hydrogen peroxide metabolic processes,” “protein ubiquitination,” “lipid transport,” and “response to oxidative stress” were also enriched in syncytia induced in wild-type plants. These groupings were not enriched in syncytia in mutant plants; instead there were slight enrichments in the “amino acid transport,” “polysaccharide catabolic process,” and “amino acid homeostasis” categories (Supplementary Figure S3).

### Reaction of Host Autophagy- or PCD-Related Genes to Nematode Parasitism

The diverse and pleiotropic functions of genes involved in PCD makes it difficult to specifically filter GO terms and create such a category. “Protein degradation” is one of the most characteristic molecular features of PCD. The MapMan classification identified 222 and 68 DEGs in syncytia induced in Col0 and *lsd1* plants, respectively, in this category. We found there were six genes linked to selective autophagy (five encoded isoforms of ATG8 and one encoded ATG12a); all were down-regulated in wild-type but not in *lsd1* (**Table 3**). The “protein degradation” category also contains metacaspases, which are homologs of animal caspases. Most of the detected genes were down-regulated (*MC1*, *MC2*, *MC3*, *MC5*, *MC6*, *MC8*, and *MC9* in wild-type; *MC2*, *MC3, MC6*, and *MC9* in *lsd1* plants). Only *MC7* was up-regulated in infected wild-type and *lsd1* plants (**Table 3**, Supplementary Figure S6 and Supplementary Tables S1, S2).

**Table 3:** The nematode induced changes in protein degradation related genes in 12 dpi syncytia of Col0 and *lsd1*(Col0) plants.

Gene_ID	Description	Log2 FC		Log2FC	
			
		Col0	*p*-value	*lsd1*(Col0)	*p*-value
**Protein degradation related genes**					
AT4G21980	*ATG 8A*	**-1.22**	**3.91E-06**	**-0.50**	**3.17E-02**
AT2G45170	*ATG 8E*	**-0.98**	**5.70E-05**	**-**0.36	2.12E-01
AT4G16520	*ATG 8F*	**-0.81**	**2.74E-04**	**-**0.37	9.22E-02
AT3G06420	*ATG 8H*	**-1.16**	**1.60E-06**	**-**0.53	1.41E-01
AT3G15580	*ATG 8I*	**-1.16**	**1.60E-06**	**-**0.20	3.85E-01
AT1G02170	*MC 1*	**-0.47**	**3.48E-02**	0.09	7.15E-01
AT4G25110	*MC 2*	**-1.51**	**8.49E-09**	**-0.74**	**4.15E-02**
AT5G64240	*MC 3*	**-0.86**	**2.79E-04**	**-1.11**	**2.23E-06**
AT1G79340	*MC 4*	**-**0.32	1.19E-01	**-**0.16	5.84E-01
AT1G79330	*MC 5*	**-0.81**	**4.43E-03**	**-**0.22	4.91E-01
AT1G79320	*MC 6*	**-1.85**	**3.08E-10**	**-0.87**	**2.01E-02**
AT1G79310	*MC 7*	**1.56**	**1.89E-10**	**0.54**	**3.33E-02**
AT1G16420	*MC 8*	**-0.89**	**3.41E-02**	**-**0.69	1.62E-01
AT5G04200	*MC 9*	**-1.33**	**3.91E-07**	**-0.84**	**7.50E-04**


**Statistically significant gene expression changes in transcriptome analyses (p < 0.05) are in bold font.**

**FIGURE 6:**
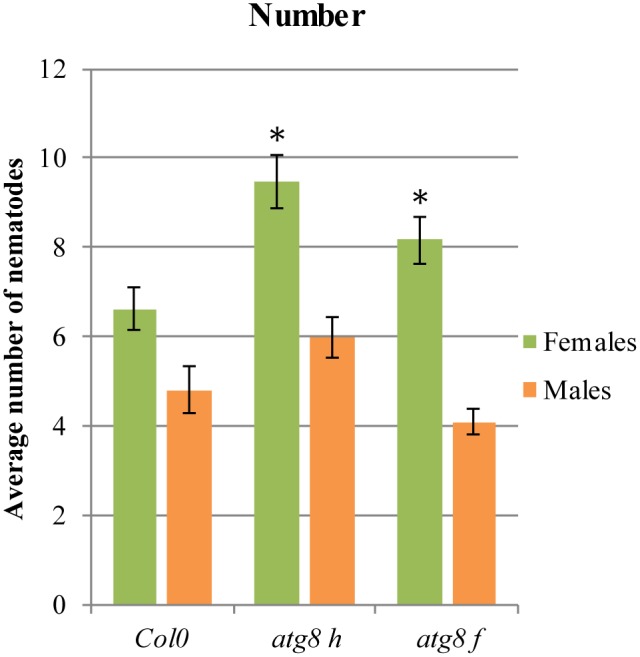
Development of *H. schachtii* and nematode-induced syncytia in roots of *atg8 h* and *atg8 f* mutants, and wild-type (Col0). Mutants and wild-type (Col0) *Arabidopsis* plants were grown under 90 μmol m^-2^ s^-1^ light intensity. Average numbers of females and males that developed by 14 dpi. Data represent means (±SEM) from three independent experiments, each containing 10 plants per genotype. Data were analyzed using ANOVA. Asterisk: significant difference from wild-type plants (*p* < 0.05). Fisher’s Least Significant Difference (LSD) test was used for *post hoc* analysis.

In order to validate the *LSD1*-dependent function of PCD related genes during cyst nematode parasitism, we tested *Arabidopsis* mutants of two genes coding for ATG8F and H isoforms. As shown in **Figure 6**, in roots of both mutant lines a significantly greater number of females could develop. The number of males did not change significantly when compared to the wild-type control.

### Involvement of Nucleotide Binding Domain-Leucine Rich Repeat Domain (NB-LRR) Genes in Compatible Plant–Nematode Interactions

Although the *Arabidopsis* genome lacks beet cyst nematode resistance genes, it contains homologs of other resistance genes (R-genes), and these provide an intriguing topic for many biotic stress studies. Their involvement in the HR in other pathogens suggests they also play a role in PCD in general. The most numerous and best studied group of R-genes is the Nucleotide Binding domain-Leucine Rich Repeat domain (NB-LRR) family. There are 149 genes encoding NB-LRR proteins in the *Arabidopsis* genome. These can be divided into two groups, the CNL and TNL subfamilies, according to whether they contain the coiled-coil domain (CC; 51 genes) or the Toll-interleukin receptor domain (TIR; 98 genes) at the N-terminus, respectively. These domains are responsible for the transmission of signals during defense responses (Meyers et al., 2003, 2005).

**FIGURE 7:**
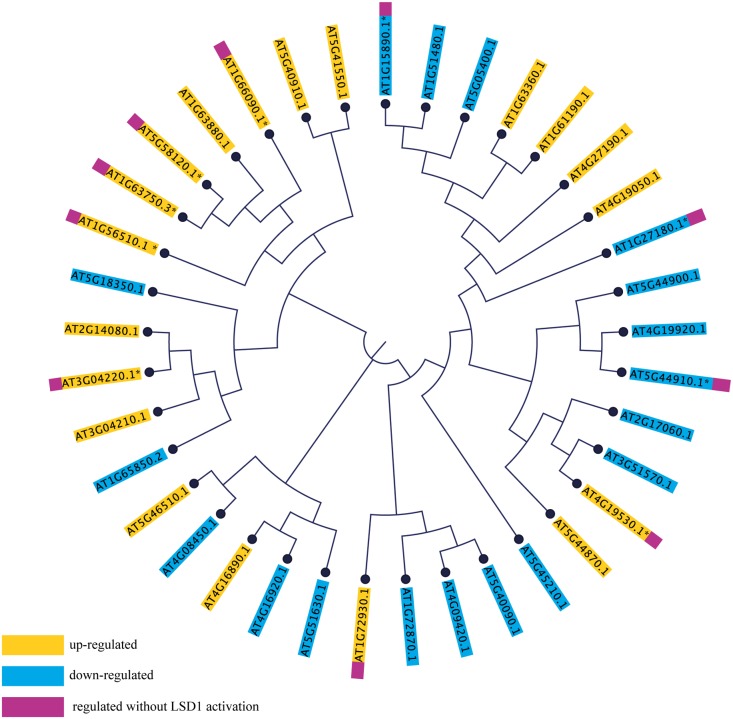
Phylogenetic tree based on amino acid sequences of *NB-LRR* genes differentially expressed in syncytia induced in wild-type (Col0) and *lsd1* mutant plants. The amino acid sequences were aligned in CLC Workbench Genomics and the phylogenetic tree was constructed using the Neighbor-Joining method.

Transcriptome screening showed that, of the 147 R-genes transcripts detected, as many as 37 were differentially regulated during syncytium development in wild-type plants; 19 genes were induced and 18 repressed (**Figure 7**). These numbers were reduced to 7 and 3, respectively, in *lsd1* mutants. Interestingly, no genes from the CNL and TNL subfamilies changed their expression exclusively in *lsd1* relative to wild-type plants (i.e., the “LSD1-compensation group”).

To determine the extent of sequence correlation among TNL and CNL genes responding to nematode parasitism, they were phylogenetically arranged on a cladogram. This indicated a strong bias toward a reaction by TNLs (31 differentially regulated TNL genes vs. 6 CNL genes). Most of these were distributed across the cladogram; however, the majority of the “LSD1-independent” R-genes were grouped on one branch, whereas the “LSD1-dependent”genes were grouped on the other branch (**Figure 7**). This may reflect the existence of conserved and partially separated regulatory pathways for these genes which are hypothetically associated with PCD regulation.

## Discussion

### The LSD1 Regulon

LESION SIMULATING DISEASE (LSD1) is a factor integrating diverse signaling pathways in response to biotic and abiotic stresses (e.g., Rustérucci et al., 2001; Mateo et al., 2004, respectively). It is a negative conditional regulator of PCD. In the current study, analyses of mutant lines with impaired LSD1 activity indicated that PCD regulators are involved in the development and functioning of nematode-induced feeding structures. Almost all existing data point to the involvement of LSD1 in the inhibition of the spread of rapid cell death in above-ground organs. *lsd1* mutants under high light intensities or challenged with avirulent pathogens exhibited the RCD phenotype in rosette leaves; this phenotype is ROS- and SA dependent (Dietrich et al., 1994).

Torres et al. (2005) proposed that LSD1, AtrbohD, and AtrbohF respond to signals released from cells undergoing the HR. In this model, AtrbohD and AtrbohF interact, and together with LSD1, fine-tune the spatial control of reactive oxygen intermediates (ROI) production and the HR in cells in and around infection sites. Torres et al. (2005) suggested that Rboh-derived ROS antagonize SA-dependent death signals to limit the spread of cell death during successful recognition of above-ground avirulent pathogens. It was postulated that LSD1 also acts in roots to inhibit hypoxia-induced PCD during aerenchyma formation (Mühlenbock et al., 2007). Our study is the first to show directly the role of LSD1 in the regulation of biotic stress responses in roots. Taken together with the report of Mühlenbock et al. (2007), the results of the present study indicate also that rosette signals clearly influence infected root processes. We found that the light intensity capable of inducing RCD in leaves decreased the susceptibility of *lsd1* mutants to beet cyst nematode infection. Thus, the results of the present study indicate a strong correlation between the rosette response and that to cyst nematodes in roots. This is in line with other studies, which showed that hypoxia-induced lysigenous aerenchyma formation intensifies when foliar RCD is induced. To eliminate potential signaling noise, further analyses of this interaction were conducted under permissive illumination conditions.

Nematode and feeding site development were influenced by genetic background in both light regimes. The mutant vs. wild-type significant differences were expressed both in nematodes number and syncytium and female size in Col0 background, whereas in Ws0 background higher susceptibility was reflected only by more females in wild-type than in *lsd1*. Such discrepancy may be related to the fact that successful nematode parasitism is a complex process extended in time. This complexity involves hormonal defense- and development-related signaling pathways regulated in stage-dependent manner (e.g., Świȩcicka et al., 2009). It starts from host finding where ethylene seems to be a key modulator of root attractiveness (Hu et al., 2017). The same hormone positively regulates later syncytium growth and functioning (for review, see Goverse and Bird, 2011). When a nematode migrates and initiates syncytium development JA is a main player in building up the host defense (Kammerhofer et al., 2015). Later stages of nematode parasitism are additionally negatively regulated by SA pathway (Hamamouch et al., 2011). The defense signaling overlaps with developmental processes which are under temporal auxin and cytokinin control (Dowd et al., 2017). Therefore a simplified assessment of *Arabidopsis* ecotypes susceptibility usually measured in female numbers per root system indicates only a summarized effect of many factors (Sijmons et al., 1991; Anwer et al., 2018). There is a growing evidence on *Arabidopsis* ecotype variation on genomic (1001 Genomes Consortium, 2016), transcriptomic (Kuśnierczyk et al., 2007) and metabolomic (Sotelo-Silveira et al., 2015) levels. This variation corresponds to numerous signaling pathways involving such hormones as auxin, JA, ethylene and SA (Kuśnierczyk et al., 2007). Moreover the Ws0 ecotype has a point mutation in *FLS2*, silencing its reaction to flg22 (Gómez-Gómez and Boller, 2002). The potential for FLS2 crosstalk with active signaling pathways involved in nematode infection may partially explain the different reactions of the ecotypes tested. In light of presented considerations the ecotype Col0 was chosen for microscopic examination and RNA-seq analysis.

There are two close homologs of *LSD1* in the *Arabidopsis* genome, *LOL1* and *LOL2*, and both are positive PCD regulators. *LOL1* overexpression is sufficient to induce PCD in wild-type plants and *lsd1* mutants (Epple et al., 2003). All three proteins share a common, functionally relevant domain (the zinc-finger domain), and they may collaborate to integrate the many signals impinging on ROI homeostasis in plants, including signals from pathogen infection (Epple et al., 2003). Infection tests with cyst nematodes were performed because the roles of these genes in plant–nematode interactions were unknown as well as the engagement of PCD. The *lsd1* and *lol2* showed opposite effects, supporting the importance of this regulatory module. The lack of effect of the *LOL1* mutation on nematode parasitism can be explained by its low expression level in roots. Cooperation between *LSD1* and other genes linked the processes analyzed here with R-gene expression, even in the case of compatible plant–nematode interactions. Generally, in many plant-pathogen systems during avirulent interactions, the detection of effectors may engage NDR1 and CC-NB-LRR or EDS1, PAD4, and SAG101, together with TIR-NB-LRRs. These complexes integrate redox signaling downstream of NADPH oxidase (Torres et al., 2005) and lead to the accumulation of SA and induction of ethylene production, which have a central role in the plant defense response. ROS and SA act synergistically to drive the HR (Coll et al., 2011). LSD1 together with EDS1 and PAD4 forms a feedback module regulating RCD that has been studied in abiotic stress responses (Mateo et al., 2004; Wituszyńska et al., 2013), as well as in the response to pathogen infection (Rustérucci et al., 2001). These proteins were defined as an interpretation module of ROI-derived signals working upstream of ethylene and SA to regulate defense responses (Mühlenbock et al., 2008).

Analysis of *lsd1*, *eds1*, and *pad4* mutants in the Ws0 background reveals the *eds1* and *pad4* mutations can partially reverse the *lsd1* RCD phenotype (Wituszyńska et al., 2013). Similar effects were also described in our study (**Figure 2**), and suggest analogous engagement of the PCD molecular machinery in plant responses to root infection by nematodes. Interestingly, the *pad4-1* mutant in the Col0 background studied by Wubben et al. (2008) was more susceptible to *H. schachtii*, as in our analyses of a Ws0 background *pad4* mutant; however, recent experiments conducted by Nguyen et al. (2016) showed lower susceptibility of *pad4-1* in the Col0 background and of *eds1-2* mutants to *H. schachtii* infection, in contrast to our results. These inconsistencies may result from differences in the experimental set-up. Relatively large differences may result, for instance, due to different light intensities and/or its spectral composition (Mateo et al., 2004), the type of container, or choice of sealing tape. In line with our results, increased resistance to *H. glycines* and *Meloidogyne incognita* infection was found in soybean overexpressing heterologous *AtPAD4* (Youssef et al., 2013). By contrast, overexpression of *Arabidopsis EDS1* increased susceptibility of soybean roots to *H. glycines* (Matthews et al., 2014). Taken together, these results indicate that the regulation of ROS and SA biosynthesis and signaling are of key importance in plant–nematode interactions.

### Anatomy of Syncytia in the *lsd1* Mutant

Plants that are more susceptible to infection by cyst nematodes often form larger syncytia, and female nematodes grow larger and develop faster. Additionally, the male:female ratio could be shifted toward females (Müller et al., 1981; Siddique et al., 2009; Anwer et al., 2018). It is difficult to indicate precisely why *lsd1* mutants show lower susceptibility to infection (**Figure 1**). It may be related to lower rates of success during the earliest infection events (root recognition, invasion, migration, selection of the ISC, and early syncytium development). Smaller sizes of syncytia and associated females may also result from changes in syncytium metabolism that were not examined in this study (Sobczak et al., 1997; Siddique et al., 2014).

The effects of the *lsd1* recessive mutation on syncytium development were observed using light and transmission electron microscopy. The differences between *lsd1* and wild-type in size and the cell wall structure (cell wall openings) suggested that syncytia development was delayed as compared to Col0 (**Figure 3** and Supplementary Figure S8). The shape and number of organelles were similar, but the clear-cut increase in syncytium vesiculation and formation of myelin-like bodies in *lsd1* syncytia suggested that some processes related to autophagy might be initiated (**Figure 3**). Autophagy cannot be simply regarded as an initial stage of cell death. In fact, it plays multiple and often antagonistic roles in plants and it is often observed upstream of the “point of no return” leading to cell death (Minina et al., 2014). Myelin-like bodies are membrane-bound cellular organelles present both in animal and plant cells. Multilamellar bodies are composed of concentric circles of membrane layers which vary in size from 100 to 2400 nm and frequently exhibit an electron-dense core. Papini and van Doorn (unpublished) suggested the autophagic/mitochondrial/plastidial origin of multilamellar bodies. Moreover, structures similar to myelin-like bodies are found in the autophagous structures in degenerating chloroplasts (van Doorn and Papini, 2013). The presence of an increased number of vesicles may also directly influence cytoplasm fluidity, and consequently impede nematode feeding, thus affecting the sizes of females.

### Transcriptomic Analysis of *lsd1* in Plant–Nematode Interactions

Transcriptomic analysis revealed that quite a mild mutant phenotype was accompanied by a massive reprogramming of gene expression. The mutation of a single gene reduced the number of DEGs from 4026 in wild-type Col0 plants to 1440. To our knowledge, this is the first report of RNA-seq usage to monitor *A. thaliana* transcriptome dynamics in cyst nematode infected roots. These results partially correspond to microarray transcriptomic analysis of *lsd1* and wild-type plants grown under laboratory and field conditions. In a field environment saturated with diverse stress factors, the mutation caused deregulation of only 105 genes, with no significant differences in seed yield, by contrast, a comparison of mutant vs. wild-type plants in the laboratory found the deregulation of 2100 genes (Wituszyńska et al., 2013). These results indicate that LSD1 is a conditional regulator of PCD. Overall, the complexity of gene expression (number of genes expressed at any level) resembled microarray data published by Szakasits et al. (2009); however, we found a substantially lower number of DEGs, and this might result from not only the transcriptomic platform used but also differences in mRNA isolation strategies (root segments containing syncytia or microaspirated syncytial protoplasts, respectively) and/or different time points for sample collection (12 dpi vs. a mixture of 5 and 15 dpi). Our sampling time, although quite late, reflected the point at which the growth dynamics of syncytium were highest (Supplementary Figure S4). Moreover, dissecting syncytia together with a small amount of root tissue ensures profiling of gene expression also in the cells surrounding the feeding structures where extensive signal exchange is expected. This region is of particular importance for our examination due to the common occurrence of dead cells abutting syncytium.

### Functional Classification of Differentially Transcribed Genes

We found the classification system provided by MapMan the most suitable for defining functional categories of genes. These categories differed in the number of genes up- and down-regulated, and it was not surprising to find the strong involvement of genes related to hormone reaction, cell wall activity, transcription regulation, and stress responses during syncytium formation and maintenance. These categories were also the ones most affected by the *lsd1* mutation, which changed the proportions of up- and down-regulated genes.

Genes related to hormone biosynthesis, perception, and signaling are especially interesting as they are involved in developmental processes, stress responses, and PCD. Kammerhofer et al. (2015) postulate that SA is a negative regulator during later syncytium and female development, whereas JA seems to be responsible for triggering early defense response against *H. schachtii*. SA plays a critical role in transcriptional reprograming during defense responses and PCD. According to Wituszyńska et al. (2013) the foliar SA content in an *lsd1* mutant was three–fivefold elevated as compared to the wild-type and conditionally regulated. Consequently, the transcription of many genes was changed. We observed this effect also in root syncytia. The number of SA-related marker, signaling, and biosynthesis genes changed their expression in a *LSD1* dependent manner. Although the group of genes classified by us as “SA related” is not very numerous (16 members), it shows a clear overrepresentation of genes reacting more severely to nematode parasitism in mutant than in the wild-type, which is not so evident among the JA and ET related genes (**Table 2** and Supplementary Figure S7). This observation supports the regulatory linkage of PCD regulators, SA signaling, and syncytium development. The observed slight retardation in syncytium development in *lsd1* may contribute to an observed increased gene transcription amplitude, however, the dynamics of SA marker gene expression through syncytium development presented in other studies cannot fully explain our observations (Hamamouch et al., 2011).

There is no convenient gene ontology category that automatically annotates and groups genes involved in autophagy or PCD. Since protein degradation is one of the most characteristic molecular features of PCD, this category should reflect the process we focus on. The ubiquitin-like protein ATG8 is a central player in the autophagy network that is required for autophagosome formation and binds to numerous cargo receptors (Kellner et al., 2016). ATG12a is important during induced autophagy (Chung et al., 2010). The *LSD1* dependent expression changes of such genes may explain the increased vesiculation of syncytial protoplasts and the presence of myelin-like bodies in syncytia induced in *lsd1* roots. The role of *ATG* genes was further documented by the analysis of two mutants, *atg8 f* and *atg8 h*, which were significantly more susceptible when number of developing females was considered (**Figure 6**). Together with the fact that these genes were down-regulated in wild-type syncytia, it indicates that precisely regulated balance of PCD players is required for successful nematode parasitism.

The “protein degradation” category also contains metacaspases, which are homologs of animal caspases. In plants, metacaspases cooperate with autophagy to regulate cell fate. These proteases are responsible for aging, immune responses, and cell differentiation (Minina et al., 2017). In our transcriptomic data seven out of nine metacaspase genes were down-regulated, and three of them in a *LSD1*-dependent manner. Only *MC7* was up-regulated in wild-type and mutant plants. Besides the expression changes, metacaspases may act antagonistically, which was shown by Coll et al. (2010) in *Pseudomonas syringae* induced foliar PCD. It was described that MC1 was a positive regulator of cell death and interacted with LSD1 via zinc-finger domains, whereas MC2 inhibited MC1-dependent cell death controlled by analogous plant NB-LRR innate immune receptors. This suggests an ancient link between the control of cell death by divergent metacaspase/caspase proteases and innate immune receptor function governed by NB-LRR proteins (Coll et al., 2011). Such numerous molecular symptoms in our data of the deregulation of PCD machinery reflects the complexity of the processes required for the HR threshold to be reached.

Besides, our experimental analysis applies to the compatible interaction, and we considered the behavior of the *NB-LRR* immune receptor genes. We speculate that tight regulation of NB-LRR proteins by the host is essential to prevent inappropriate signaling and induction of PCD in the absence of pathogens or in the case of virulence. Many NB-LRRs were subjected to transcriptional (this study) and posttranscriptional regulation (Zhang et al., 2016). Although it is difficult to hypothesize their roles and possible interactions in PCD during syncytium development and maintenance, this gene family is transcriptionally very dynamic. Given that many published models of PCD classify it as developmental and stress-related, in our results we can find premises supporting a mixed nature of these processes. For instance, Olvera-Carrillo et al. (2015) conducted a meta-analysis of published data on PCD in plants specifying the list of markers for developmental (dPCD) and biotic PCD (bPCD). Our RNA-seq data revealed differentially expressed dPCD markers for the SAUR-like auxin-responsive protein family and *Ca^2^*^+^ *DEPENDENT NUCLEASE* (*CAN*). Markers for bPCD were also present on our list, including *PHYTOALEXIN DEFICIENT 3* (*PAD3*) and *ENHANCED DISEASE SUSCEPTIBILITY 5* (*EDS5*).

Programmed cell death cannot be separated from the regulation of cellular ROS homeostasis. ROS play dual roles in plant biology. One function lies in cell signaling but, at higher concentrations, ROS can be toxic by-products of aerobic metabolism that cause oxidative stress and activate PCD (Mittler, 2017). Thus, pro- and anti-apoptotic levels of ROS are crucial for multiple plant processes. Plants have evolved several pathways for ROS generation and scavenging to maintain an appropriate balance. Many of these involve a central regulatory role for LSD1; for example, LSD1 regulates photorespiratory ROS and CAT activity (Mateo et al., 2004). It is also required for the accumulation of a MnSOD during the spread of cell death in response to high light stress, suggesting that AtrbohD and LSD1 might ultimately protect cells by regulating ROS metabolism (Torres et al., 2005, 2006). Other studies show that LSD1 interacts with catalase genes to regulate light-dependent RCD and hypersensitive-type cell death (Mateo et al., 2004; Li et al., 2013). Similar LSD1-dependent regulation was observed in the case of CuZnSOD to limit the spread of cell death (Kliebenstein et al., 1999). We observed up-regulation of catalase 3 (CAT3) and two superoxide dismutases, MnSOD and FeSOD, in the transcriptomic data of infected Col0 and *lsd1* plants. Li et al. (2013) postulated that *LSD1* may act as a house-keeping gene interacting with proteins with diverse functions and subcellular localization patterns to regulate PCD-associated signaling pathways. In this context, LSD1 may function as a molecular chaperone to monitor or sense reduced activity, distorted structure, or mis-localization of target proteins induced by the cellular ROS and/or the cellular redox status.

The LSD1-dependent transcriptomic dynamics point many other genes which are apparently co-regulated with PCD molecular machinery driving syncytium development. They encode, e.g., transcription regulators such as WRKY factors (Supplementary Tables S1, S2) or cell wall modifying enzymes (Supplementary Tables S1, S2 and Supplementary Figure S3) which were described previously but their correlation to autophagy or PCD might be an interesting feature worth further research.

In this study, we have shown that negative and positive regulators of PCD play a role in compatible plant–nematode interactions and dramatically deregulate gene expression during syncytium development. This is strongly correlated to susceptibility changes to cyst nematodes and morphological features, including reduced size of the feeding sites. Symptoms of enhanced autophagy were apparent in the transcriptomic and ultrastructural changes, but they did not reach a detrimental level. More experiments involving other mutants and markers of autophagy or PCD are required. These should include mutants with genetic backgrounds that allow examination of responses to avirulent infection. It is also becoming clear that changes in the balance of pro- and anti-PCD signals are required at different stages of nematode parasitism.

## Author Contributions

MM, MS, and MF: conceived and designed the experiments. MM and MS: performed the experiments. MM, MS, JC, and MF: analyzed the results. MM, MF, MS, CE, and SK: contributed to the writing of the manuscript.

## Conflict of Interest Statement

The authors declare that the research was conducted in the absence of any commercial or financial relationships that could be construed as a potential conflict of interest.

## Footnotes

1 http://www.ncbi.nlm.nih.gov/genome/4
