# Supplementary material for: The Role of Programmed Cell Death Regulator LSD1 in Nematode-Induced Syncytium Formation
Source: Front Plant Sci. 2018 Mar 19;9:314. doi: 10.3389/fpls.2018.00314 (PMC5868158; doi:10.3389/fpls.2018.00314)
Supplement: Supplementary file 1 [file Table_1.PDF]

Supplementary Table 1 - RNA-seq analysis of syncytia induced in wild-type (Col0)

Col0 infected vs Col0 uninfected			
**Locus ID**	**log2FC**	**Primary Gene Symbol**	**Gene Model Description**
AT5G56640	*6,563*	MYO-INOSITOL OXYGENASE 5 (MIOX5)	Myo-Inositol Oxygenase gene family
AT2G27240	*6,18*		Aluminium activated malate transporter family protein; INVOLVED IN: response to aluminum ion; EXPRESSED IN: embryo, sepal, male gametophyte, flower, seed; EXPRESSED DURING: 6 growth stages; CONTAINS InterPro DOMAIN/s: Malate transporter, aliminium toerance (InterPro:IPR020966); BEST Arabidopsis thaliana protein match is: Aluminium activated malate transporter family protein (TAIR:AT1G08440.1)
AT3G19270	*5,724*	CYTOCHROME P450, FAMILY 707, SUBFAMILY A, POLYPEPTIDE 4 (CYP707A4)	Encodes a protein with ABA 8'-hydroxylase activity, involved in ABA catabolism. Member of the CYP707A gene family.
AT3G54700	*5,526*	PHOSPHATE TRANSPORTER 1;7 (PHT1;7)	Encodes Pht1;7, a member of the Pht1 family of phosphate transporters which include: Pht1;1/At5g43350, Pht1;2/At5g43370, Pht1;3/At5g43360, Pht1;4/At2g38940, Pht1;5/At2g32830, Pht1;6/At5g43340, Pht1;7/At3g54700, Pht1;8/At1g20860, Pht1;9/At1g76430 (Plant Journal 2002, 31:341).
AT5G19700	*5,469*	EARLY LEAF SENESCENCE 1 (ELS1)	Encodes a MATE transporter involved in leaf senescence and iron homeostasis.
AT5G05340	*5,27*	PEROXIDASE 52 (PRX52)	Encodes a protein with sequence similarity to peroxidases that is involved in lignin biosynthesis. Loss of function mutations show abnormal development of xylem fibers and reduced levels of lignin biosynthetic enxymes.
AT3G55500	*5,24*	EXPANSIN A16 (EXPA16)	expansin-like protein. Naming convention from the Expansin Working Group (Kende et al, 2004. Plant Mol Bio). Involved in the formation of nematode-induced syncytia in roots of Arabidopsis thaliana.
AT4G26260	*5,2*	MYO-INOSITOL OXYGENASE 4 (MIOX4)	Encodes a myo-inositol oxygenase.
AT5G52390	*5,132*		PAR1 protein; FUNCTIONS IN: molecular_function unknown; INVOLVED IN: biological_process unknown; LOCATED IN: endomembrane system; EXPRESSED IN: 13 plant structures; EXPRESSED DURING: 8 growth stages; CONTAINS InterPro DOMAIN/s: PAR1 (InterPro:IPR009489); BEST Arabidopsis thaliana protein match is: PAR1 protein (TAIR:AT3G54040.1)
AT1G68460	*5,048*	ISOPENTENYLTRANSFERASE 1 (IPT1)	Encodes a putative adenylate isopentenyltransferase. It catalyzes the formation of isopentenyladenosine 5'-monophosphate (iPMP) from AMP and dimethylallylpyrophosphate (DMAPP), but it has a lower Km for ADP and likely works using ADP or ATP in plants. It is involved in cytokinin biosynthesis.
AT1G08080	*4,949*	ALPHA CARBONIC ANHYDRASE 7 (ACA7)	alpha carbonic anhydrase 7 (ACA7); FUNCTIONS IN: carbonate dehydratase activity, zinc ion binding; INVOLVED IN: one-carbon metabolic process; LOCATED IN: endomembrane system; CONTAINS InterPro DOMAIN/s: Carbonic anhydrase, alpha-class, catalytic domain (InterPro:IPR001148), Carbonic anhydrase, CAH1-like (InterPro:IPR018340), Carbonic anhydrase, alpha-class, conserved site (InterPro:IPR018338); BEST Arabidopsis thaliana protein match is: alpha carbonic anhydrase 5 (TAIR:AT1G08065.1)
AT5G20740	*4,568*		Plant invertase/pectin methylesterase inhibitor superfamily protein; FUNCTIONS IN: enzyme inhibitor activity, pectinesterase inhibitor activity, pectinesterase activity; INVOLVED IN: biological_process unknown; LOCATED IN: endomembrane system; EXPRESSED IN: 13 plant structures; EXPRESSED DURING: 7 growth stages; CONTAINS InterPro DOMAIN/s: Pectinesterase inhibitor (InterPro:IPR006501); BEST Arabidopsis thaliana protein match is: Plant invertase/pectin methylesterase inhibitor superfamily protein (TAIR:AT1G62770.1)
AT1G08440	*4,497*		Aluminium activated malate transporter family protein; INVOLVED IN: response to aluminum ion; CONTAINS InterPro DOMAIN/s: Malate transporter, aliminium toerance (InterPro:IPR020966); BEST Arabidopsis thaliana protein match is: aluminum-activated malate transporter 1 (TAIR:AT1G08430.1)
AT4G13260	*4,315*	YUCCA2 (YUC2)	Encodes YUC2. Catalyzes conversion of IPA (indole-3-pyruvic acid) to IAA (indole-3-acetic acid) in auxin biosynthesis pathway.

AT4G24040	*4,24*	TREHALASE 1 (TRE1)	Encodes a trehalase, member of Glycoside Hydrolase Family 37.
AT5G56510	*4,215*	PUMILIO 12 (PUM12)	Encodes a member of the Arabidopsis Pumilio (APUM) proteins containing PUF domain (eight repeats of approximately 36 amino acids each). PUF proteins regulate both mRNA stability and translation through sequence-specific binding to the 3' UTR of target mRNA transcripts.
AT1G06030	*4,193*		pfkB-like carbohydrate kinase family protein; FUNCTIONS IN: kinase activity, ribokinase activity; INVOLVED IN: D-ribose metabolic process, acetate fermentation, sucrose biosynthetic process, sucrose catabolic process, using beta-fructofuranosidase; EXPRESSED IN: guard cell; CONTAINS InterPro DOMAIN/s: Carbohydrate/purine kinase (InterPro:IPR011611), Ribokinase (InterPro:IPR002139), Carbohydrate/puine kinase, PfkB, conserved site (InterPro:IPR002173); BEST Arabidopsis thaliana protein match is: pfkB-like carbohydrate kinase family protein (TAIR:AT1G06020.1)
AT1G69930	*4,175*	GLUTATHIONE S-TRANSFERASE TAU 11	Encodes glutathione transferase belonging to the tau class of GSTs. Naming convention according to Wagner et al. (2002).
AT1G54870	*4,162*		ChlADR is an aldehyde reductase that catalyzes the reduction of the aldehyde carbonyl groups on saturated and alpha,beta-unsaturated aldehydes with more than 5 carbons in vitro. The N-terminal region of this protein directs GFP to the chloroplast where where ChlADR likely helps to maintain the photosynthetic process by detoxifying reactive carbonyls formed during lipid peroxidation. In addition, this enzyme can also reduce cis-3-hexenal, a major plant volatile compound that contributes to green leaf odor, as well as methylglyoxal in vitro.
AT4G25580	*4,108*		CAP160 protein; CONTAINS InterPro DOMAIN/s: CAP160 (InterPro:IPR012418); BEST Arabidopsis thaliana protein match is: CAP160 protein (TAIR:AT5G52300.1)
AT1G71890	*4,092*	(SUC5)	Encodes a sucrose transporter that is expressed in the endosperm. Mutants have delayed accumulation of fatty acids and embryo maturation.
AT1G10010	*4,088*	AMINO ACID PERMEASE 8 (AAP8)	Encodes a high affinity amino acid transporter that is probably responsible for import of organic nitrogen into developing seeds. One of eight gene family members that encode amino acid permeases. Most closely related to AAP1 (75%) identity.
AT5G50360	*4,059*		unknown protein; BEST Arabidopsis thaliana protein match is: unknown protein (TAIR:AT3G48510.1)
AT5G43340	*4,058*	PHOSPHATE TRANSPORTER 1;6 (PHT1;6)	Encodes Pht1;6, a member of the Pht1 family of phosphate transporters which include: Pht1;1/At5g43350, Pht1;2/At5g43370, Pht1;3/At5g43360, Pht1;4/At2g38940, Pht1;5/At2g32830, Pht1;6/At5g43340, Pht1;7/At3g54700, Pht1;8/At1g20860, Pht1;9/At1g76430 (Plant Journal 2002, 31:341).
AT2G36970	*4,04*		UDP-Glycosyltransferase superfamily protein; FUNCTIONS IN: UDP-glycosyltransferase activity, transferase activity, transferring glycosyl groups; INVOLVED IN: metabolic process; LOCATED IN: endomembrane system; EXPRESSED IN: 20 plant structures; EXPRESSED DURING: 13 growth stages; CONTAINS InterPro DOMAIN/s: UDP-glucuronosyl/UDP-glucosyltransferase (InterPro:IPR002213); BEST Arabidopsis thaliana protein match is: UDP-Glycosyltransferase superfamily protein (TAIR:AT2G28080.1)
AT1G49330	*3,98*		hydroxyproline-rich glycoprotein family protein; BEST Arabidopsis thaliana protein match is: unknown protein (TAIR:AT2G16190.1)
AT1G61610	*3,954*		S-locus lectin protein kinase family protein; FUNCTIONS IN: in 6 functions; INVOLVED IN: protein amino acid phosphorylation, recognition of pollen; LOCATED IN: endomembrane system; EXPRESSED IN: 7 plant structures; EXPRESSED DURING: 6 growth stages; CONTAINS InterPro DOMAIN/s: Curculin-like (mannose-binding) lectin (InterPro:IPR001480), Protein kinase, ATP binding site (InterPro:IPR017441), PAN-2 domain (InterPro:IPR013227), Apple-like (InterPro:IPR003609), EGF-like, type 3 (InterPro:IPR000742), Serine-threonine/tyrosine-protein kinase (InterPro:IPR001245), Protein kinase-like domain (InterPro:IPR011009), Serine/threonine-protein kinase, active site (InterPro:IPR008271), Protein kinase, catalytic domain (InterPro:IPR000719), S-locus glycoprotein (InterPro:IPR000858), EGF-like (InterPro:IPR006210); BEST Arabidopsis thaliana protein match is: S-locus lectin protein kinase family protein (TAIR:AT4G21390.1)
AT2G47780	*3,922*	LD-ASSOCIATED PROTEIN 2 (LDAP2)	Encodes a small rubber particle protein homolog. Plays dual roles as positive factors for tissue growth and development and in drought stress responses.

AT2G44070	*3,885*		NagB/RpiA/CoA transferase-like superfamily protein; FUNCTIONS IN: GTP binding, translation initiation factor activity; INVOLVED IN: translational initiation, cellular metabolic process; LOCATED IN: eukaryotic translation initiation factor 2B complex; CONTAINS InterPro DOMAIN/s: Initiation factor 2B related (InterPro:IPR000649); BEST Arabidopsis thaliana protein match is: NagB/RpiA/CoA transferase-like superfamily protein (TAIR:AT5G38640.1)
AT2G40100	*3,836*	LIGHT HARVESTING COMPLEX PHOTOSYSTEM II (LHCB4.3)	Lhcb4:3 protein (Lhcb4.3, light harvesting complex of photosystem II The mRNA is cell-to-cell mobile.
AT5G61890	*3,794*		encodes a member of the ERF (ethylene response factor) subfamily B-4 of ERF/AP2 transcription factor family. The protein contains one AP2 domain. There are 7 members in this subfamily.
AT1G64110	*3,778*	DUO1-ACTIVATED ATPASE 1 (DAA1)	Target promoter of the male germline-specific transcription factor DUO1.
AT5G52300	*3,743*	LOW-TEMPERATURE-INDUCED 65 (LTI65)	Encodes a protein that is induced in expression in response to water deprivation such as cold, high-salt, and desiccation. The response appears to be via abscisic acid. The promoter region contains two ABA-responsive elements (ABREs) that are required for the dehydration-responsive expression of rd29B as cis-acting elements. Protein is a member of a gene family with other members found plants, animals and fungi.
AT2G16190	*3,69*		BEST Arabidopsis thaliana protein match is: hydroxyproline-rich glycoprotein family protein (TAIR:AT1G49330.1)
AT4G17600	*3,656*	LIGHT-HARVESTING-LIKE 3:1 (LIL3:1)	Encodes Lil3:1 (light-harvesting-like) protein. Belongs to the Lhc super-gene family encodes the light-harvesting chlorophyll a/b-binding (LHC) proteins that constitute the antenna system of the photosynthetic apparatus. A generic LHC motif is present in Lil3:1. The mRNA is cell-to-cell mobile.
AT5G19110	*3,639*		Eukaryotic aspartyl protease family protein; FUNCTIONS IN: aspartic-type endopeptidase activity; INVOLVED IN: proteolysis, N-terminal protein myristoylation; LOCATED IN: endomembrane system; EXPRESSED IN: shoot apex, leaf apex, hypocotyl, root, leaf; CONTAINS InterPro DOMAIN/s: Peptidase aspartic (InterPro:IPR021109), Peptidase aspartic, catalytic (InterPro:IPR009007), Peptidase A1 (InterPro:IPR001461); BEST Arabidopsis thaliana protein match is: Eukaryotic aspartyl protease family protein (TAIR:AT1G03220.1)
AT2G40200	*3,632*		basic helix-loop-helix (bHLH) DNA-binding superfamily protein; FUNCTIONS IN: DNA binding, sequence-specific DNA binding transcription factor activity; INVOLVED IN: regulation of transcription; LOCATED IN: nucleus; EXPRESSED IN: 21 plant structures; EXPRESSED DURING: 13 growth stages; CONTAINS InterPro DOMAIN/s: Helix-loop-helix DNA-binding domain (InterPro:IPR001092), Helix-loop-helix DNA-binding (InterPro:IPR011598); BEST Arabidopsis thaliana protein match is: basic helix-loop-helix (bHLH) DNA-binding superfamily protein (TAIR:AT3G56770.1)
AT5G13220	*3,621*	JASMONATE-ZIM-DOMAIN PROTEIN 10 (JAZ10)	Plants overexpressing At5g13220.3, but not At5g13220.1 showed enhanced insensitivity to MeJa.
AT5G01300	*3,615*		PEBP (phosphatidylethanolamine-binding protein) family protein; FUNCTIONS IN: phosphatidylethanolamine binding; INVOLVED IN: biological_process unknown; LOCATED IN: cellular_component unknown; EXPRESSED IN: 7 plant structures; EXPRESSED DURING: LP.04 four leaves visible, 4 anthesis, 4 leaf senescence stage, petal differentiation and expansion stage, LP.08 eight leaves visible; CONTAINS InterPro DOMAIN/s: YbhB/YbcL (InterPro:IPR005247), Phosphatidylethanolamine-binding protein PEBP (InterPro:IPR008914)
AT4G15210	*3,592*	BETA-AMYLASE 5 (BAM5)	cytosolic beta-amylase expressed in rosette leaves and inducible by sugar. RAM1 mutants have reduced beta amylase in leaves and stems.
AT3G55970	*3,578*	JASMONATE-REGULATED GENE 21 (JRG21)	jasmonate-regulated gene 21 (JRG21); FUNCTIONS IN: oxidoreductase activity, iron ion binding; INVOLVED IN: oxidation reduction; LOCATED IN: cellular_component unknown; EXPRESSED IN: 10 plant structures; EXPRESSED DURING: LP.06 six leaves visible, LP.04 four leaves visible, 4 anthesis, C globular stage, petal differentiation and expansion stage; CONTAINS InterPro DOMAIN/s: Isopenicillin N synthase (InterPro:IPR002283), Oxoglutarate/iron-dependent oxygenase (InterPro:IPR005123); BEST Arabidopsis thaliana protein match is: 2-oxoglutarate (2OG) and Fe(II)-dependent oxygenase superfamily protein (TAIR:AT5G05600.1)

AT4G16690	*3,571*	METHYL ESTERASE 16 (MES16)	Encodes a protein shown to have carboxylesterase activity, methyl IAA esterase activity, and methyl jasmonate esterase activity in vitro. This protein does not act on MeSA, MeGA4, or MEGA9 in vitro. Although MES16 is similar to MES17, a MeIAA hydrolase, two mes16 mutant lines (SALK_151578) and (SALK_139756) do not show altered sensitivity to MeIAA in root growth assays. MES16 transcripts appear to be more than 10-fold less abundant than those of MES17 in roots.
AT1G71770	*3,565*	POLY(A)-BINDING PROTEIN 5 (PAB5)	Encodes a Class I polyA-binding protein. Expressed in floral organs. Binds polyA sepharose in vitro.
AT4G39650	*3,556*	GAMMA-GLUTAMYL TRANSPEPTIDASE 2 (GGT2)	The gene encodes a gamma-glutamyltransferase (AKA gamma-glutamyl transpeptidase, EC 2.3.2.2) that is located in the apoplast of young siliques (within the ovules of the carpel) and is involved in the degradation of glutathione. The encoded enzyme also acts as part of a GSH pumping gamma-glutamyl cycle in this tissue and may also be involved in gamma-glutamyl amino acid formation.
AT1G65500	*3,519*		unknown protein; FUNCTIONS IN: molecular_function unknown; INVOLVED IN: biological_process unknown; LOCATED IN: endomembrane system; EXPRESSED IN: 14 plant structures; EXPRESSED DURING: 9 growth stages; BEST Arabidopsis thaliana protein match is: unknown protein (TAIR:AT1G65486.1)
AT1G12180	*3,505*		BEST Arabidopsis thaliana protein match is: HSP20-like chaperones superfamily protein (TAIR:AT5G47600.1)
AT1G50960	*3,501*	GIBBERELLIN 2-OXIDASE 7 (GA2OX7)	Encodes a protein with gibberellin 2-oxidase activity which acts specifically on C-20 gibberellins. DDF1 binds to GA2OX7 and regulates its expression in response to salt stress.
AT5G49630	*3,494*	AMINO ACID PERMEASE 6 (AAP6)	Is a high affinity amino acid transporter capable of transporting aspartate and tryptophan. May be involved in the amino acid uptake from xylem.
AT1G18710	*3,491*	MYB DOMAIN PROTEIN 47 (MYB47)	Member of the R2R3 factor gene family.
AT1G07430	*3,486*	HIGHLY ABA-INDUCED PP2C GENE 2 (HAI2)	Encodes a member of the group A protein phosphatase 2C (PP2C) family that is responsible for negatively regulating seed dormancy.
AT1G62290	*3,484*	PUTATIVE ASPARTIC PROTEINASE A2 (PASPA2)	Saposin-like aspartyl protease family protein; FUNCTIONS IN: aspartic-type endopeptidase activity; INVOLVED IN: proteolysis, lipid metabolic process; LOCATED IN: vacuole; EXPRESSED IN: 10 plant structures; EXPRESSED DURING: LP.06 six leaves visible, LP.04 four leaves visible, seedling growth, petal differentiation and expansion stage, LP.08 eight leaves visible; CONTAINS InterPro DOMAIN/s: Saposin-like (InterPro:IPR011001), Peptidase aspartic (InterPro:IPR021109), Peptidase aspartic, catalytic (InterPro:IPR009007), Saposin-like type B, 1 (InterPro:IPR007856), Saposin-like type B, 2 (InterPro:IPR008138), Saposin B (InterPro:IPR008139), Peptidase A1 (InterPro:IPR001461), Peptidase aspartic, active site (InterPro:IPR001969); BEST Arabidopsis thaliana protein match is: aspartic proteinase A1 (TAIR:AT1G11910.1)
AT4G23990	*3,481*	CELLULOSE SYNTHASE LIKE G3 (CSLG3)	encodes a protein similar to cellulose synthase
AT1G14520	*3,464*	MYO-INOSITOL OXYGENASE 1 (MIOX1)	Encodes MIOX1. Belongs to myo-inositol oxygenase gene family.
AT2G27690	*3,435*	CYTOCHROME P450, FAMILY 94, SUBFAMILY C, POLYPEPTIDE 1 (CYP94C1)	Encodes a CYP94C1. Has highest omega-hydroxylase activity with 9,10-epoxystearic acid, while also metabolized lauric acid (C12:0) and C18 unsaturated fatty acids. Gene expression is induced in response to wounding and jasmonic acid treatment.
AT3G04510	*3,411*	LIGHT SENSITIVE HYPOCOTYLS 2 (LSH2)	LIGHT SENSITIVE HYPOCOTYLS 2 (LSH2); FUNCTIONS IN: molecular_function unknown; INVOLVED IN: biological_process unknown; LOCATED IN: chloroplast; EXPRESSED IN: 12 plant structures; EXPRESSED DURING: 4 leaf senescence stage, petal differentiation and expansion stage; CONTAINS InterPro DOMAIN/s: Protein of unknown function DUF640 (InterPro:IPR006936); BEST Arabidopsis thaliana protein match is: Protein of unknown function (DUF640) (TAIR:AT5G28490.1)
AT4G13480	*3,406*	MYB DOMAIN PROTEIN 79 (MYB79)	Member of the R2R3 factor gene family.
AT4G18920	*3,393*		Protein of unknown function (DUF1264); CONTAINS InterPro DOMAIN/s: Protein of unknown function DUF1264 (InterPro:IPR010686); BEST Arabidopsis thaliana protein match is: Protein of unknown function (DUF1264) (TAIR:AT5G45690.1)

AT5G61740	*3,388*	ATP-BINDING CASSETTE A10 (ABCA10)	member of ATH subfamily
AT5G44310	*3,37*		Late embryogenesis abundant protein (LEA) family protein; INVOLVED IN: embryo development ending in seed dormancy; EXPRESSED IN: cotyledon; BEST Arabidopsis thaliana protein match is: Late embryogenesis abundant protein (LEA) family protein (TAIR:AT4G21020.1)
AT1G26730	*3,332*		EXS (ERD1/XPR1/SYG1) family protein; LOCATED IN: integral to membrane, plasma membrane; EXPRESSED IN: 22 plant structures; EXPRESSED DURING: 13 growth stages; CONTAINS InterPro DOMAIN/s: EXS, C-terminal (InterPro:IPR004342), SPX, N-terminal (InterPro:IPR004331); BEST Arabidopsis thaliana protein match is: EXS (ERD1/XPR1/SYG1) family protein (TAIR:AT1G35350.1)
AT1G56510	*3,302*	WHITE RUST RESISTANCE 4 (WRR4)	TIR-NB-LRR protein that confers resistance to four races of Albugo candida. The mRNA is cell-to-cell mobile.
AT2G30830	*3,291*		encodes a protein whose sequence is similar to 2-oxoglutarate-dependent dioxygenase
AT2G43360	*3,283*	BIOTIN AUXOTROPH 2 (BIO2)	Catalyzes the conversion of dethiobiotin to biotin.
AT5G02030	*3,281*	REPLUMLESS (RPL)	Mutant has additional lateral organs and phyllotaxy defect. Encodes a homeodomain transcription factor. Has sequence similarity to the Arabidopsis ovule development regulator Bell1. Binds directly to the AGAMOUS cis-regulatory element. Its localization to the nucleus is dependent on the coexpression of either STM or BP.
AT5G21150	*3,28*	ARGONAUTE 9 (AGO9)	AGO9-dependent sRNA silencing is crucial to specify cell fate in the Arabidopsis ovule. AGO9 is expressed in reproductive companion cells but not in the associated male or female gametes or their precursors. Therefore, AGO9 acts non-cell autonomously to silencing the activity of TEs activity in the female gametophyte.Loss of function mutants produce ectopic megaspore mother cell and supernumary female gametophytes.
AT5G16980	*3,266*		Zinc-binding dehydrogenase family protein; FUNCTIONS IN: oxidoreductase activity, binding, zinc ion binding, catalytic activity; INVOLVED IN: response to oxidative stress, response to cyclopentenone; EXPRESSED IN: 11 plant structures; EXPRESSED DURING: LP.06 six leaves visible, LP.04 four leaves visible, 4 anthesis, petal differentiation and expansion stage; CONTAINS InterPro DOMAIN/s: GroES-like (InterPro:IPR011032), Polyketide synthase, enoylreductase (InterPro:IPR020843), NAD(P)-binding domain (InterPro:IPR016040), Alcohol dehydrogenase, C-terminal (InterPro:IPR013149), Alcohol dehydrogenase superfamily, zinc-containing (InterPro:IPR002085); BEST Arabidopsis thaliana protein match is: alkenal reductase (TAIR:AT5G16970.1).
AT3G60140	*3,265*	DARK INDUCIBLE 2 (DIN2)	Encodes a protein similar to beta-glucosidase and is a member of glycoside hydrolase family 1. Expression is induced after 24 hours of dark treatment, in senescing leaves and treatment with exogenous photosynthesis inhibitor. Induction of gene expression was suppressed in excised leaves supplied with sugar. The authors suggest that the gene's expression pattern is responding to the level of sugar in the cell. The mRNA is cell-to-cell mobile.
AT2G20350	*3,243*		encodes a member of the ERF (ethylene response factor) subfamily B-6 of ERF/AP2 transcription factor family. The protein contains one AP2 domain. There are 12 members in this subfamily including RAP2.11.
AT4G27560	*3,242*	(UGT79B2)	UDP-Glycosyltransferase superfamily protein; FUNCTIONS IN: transferase activity, transferring glycosyl groups; INVOLVED IN: response to salt stress, N-terminal protein myristoylation; LOCATED IN: cellular_component unknown; EXPRESSED IN: guard cell; CONTAINS InterPro DOMAIN/s: UDP-glucuronosyl/UDP-glucosyltransferase (InterPro:IPR002213); BEST Arabidopsis thaliana protein match is: UDP-Glycosyltransferase superfamily protein (TAIR:AT4G27570.1)
AT5G62800	*3,229*		Protein with RING/U-box and TRAF-like domains; FUNCTIONS IN: ubiquitin-protein ligase activity, zinc ion binding; INVOLVED IN: multicellular organismal development, protein ubiquitination, ubiquitin-dependent protein catabolic process; LOCATED IN: nucleus; EXPRESSED IN: stem; CONTAINS InterPro DOMAIN/s: TRAF-like (InterPro:IPR008974), Seven-in-absentia protein, TRAF-like domain (InterPro:IPR018121), Zinc finger, SIAH-type (InterPro:IPR013010), Seven In Absentia Homolog-type (InterPro:IPR013323), Seven-in-absentia protein, sina (InterPro:IPR004162); BEST Arabidopsis thaliana protein match is: Protein with RING/U-box and TRAF-like domains (TAIR:AT5G37890.1)
AT1G05800	*3,223*	DONGLE (DGL)	Encodes a galactolipase. Located in the chloroplast. Involved in the initial step of jasmonic acid biosynthesis. Expressed in vegetative tissues and is necessary for the biosynthesis of basal-level JAs in vegetative tissues.

AT5G24540	*3,223*	BETA GLUCOSIDASE 31 (BGLU31)	beta glucosidase 31 (BGLU31); FUNCTIONS IN: cation binding, hydrolase activity, hydrolyzing O-glycosyl compounds, catalytic activity; INVOLVED IN: response to other organism; LOCATED IN: endomembrane system; EXPRESSED IN: stem, sepal; EXPRESSED DURING: 4 anthesis; CONTAINS InterPro DOMAIN/s: Glycoside hydrolase, family 1 (InterPro:IPR001360), Glycoside hydrolase, family 1, active site (InterPro:IPR018120), Glycoside hydrolase, catalytic core (InterPro:IPR017853), Glycoside hydrolase, subgroup, catalytic core (InterPro:IPR013781); BEST Arabidopsis thaliana protein match is: beta glucosidase 32 (TAIR:AT5G24550.1)
AT1G25410	*3,222*	ISOPENTENYLTRANSFERASE 6 (IPT6)	AB061404 Arabidopsis thaliana AtIPT6 mRNA for cytokinin synthase, complete cds
AT1G32960	*3,213*	(SBT3.3)	SBT3.3; FUNCTIONS IN: identical protein binding, serine-type endopeptidase activity; INVOLVED IN: proteolysis, negative regulation of catalytic activity; LOCATED IN: apoplast, plant-type cell wall; EXPRESSED IN: 7 plant structures; EXPRESSED DURING: 4 anthesis; CONTAINS InterPro DOMAIN/s: Protease-associated PA (InterPro:IPR003137), Proteinase inhibitor, propeptide (InterPro:IPR009020), Peptidase S8/S53, subtilisin/kexin/sedolisin (InterPro:IPR000209), Peptidase S8, subtilisin-related (InterPro:IPR015500), Proteinase inhibitor I9, subtilisin propeptide (InterPro:IPR010259), Peptidase S8/S53, subtilisin, active site (InterPro:IPR022398); BEST Arabidopsis thaliana protein match is: Subtilase family protein (TAIR:AT1G32950.1)
AT1G52700	*3,209*		alpha/beta-Hydrolases superfamily protein; FUNCTIONS IN: hydrolase activity, carboxylesterase activity; INVOLVED IN: biological_process unknown; EXPRESSED IN: 11 plant structures; EXPRESSED DURING: 7 growth stages; CONTAINS InterPro DOMAIN/s: Phospholipase/carboxylesterase (InterPro:IPR003140); BEST Arabidopsis thaliana protein match is: alpha/beta-Hydrolases superfamily protein (TAIR:AT3G15650.2)
AT2G05400	*3,207*		Ubiquitin-specific protease family C19-related protein; BEST Arabidopsis thaliana protein match is: TRAF-like family protein (TAIR:AT2G05410.1)
AT4G18170	*3,193*	WRKY DNA-BINDING PROTEIN 28 (WRKY28)	member of WRKY Transcription Factor; Group II-c. Involved in the activation of salicylic acid biosynthesis genes ICS1 and PBS3.
AT2G42540	*3,176*	COLD-REGULATED 15A (COR15A)	A cold-regulated gene whose product is targeted to the chloroplast. Cor15am protects stromal proteins from aggregation under various stress conditions. Constitutive expression increases freezing tolerance in protoplasts in vitro and chloroplasts in vivo. NMR and x-ray diffraction studies suggest that COR15a alters the intrinsic curvature of the inner membrane of chloroplast envelope. Late Embryogenesis abundant protein (LEA). Protects chloroplast membranes during freezing.
AT2G26740	*3,162*	SOLUBLE EPOXIDE HYDROLASE (SEH)	Encodes a soluble epoxide hydrolase whose expression is induced by auxin and water stress.
AT3G25760	*3,143*	ALLENE OXIDE CYCLASE 1 (AOC1)	encodes allene oxide cyclase. One of four genes in Arabidopsis that encode this enzyme, which catalyzes an essential step in jasmonic acid biosynthesis. Gene expression is induced during senescence, a process that involves jasmonic acid signalling pathway. The mRNA is cell-to-cell mobile.
AT2G46420	*3,142*		Plant protein 1589 of unknown function; CONTAINS InterPro DOMAIN/s: Conserved hypothetical protein CHP01589, plant (InterPro:IPR006476); BEST Arabidopsis thaliana protein match is: Plant protein 1589 of unknown function (TAIR:AT3G61700.1)
AT1G78490	*3,131*	CYTOCHROME P450, FAMILY 708, SUBFAMILY A, POLYPEPTIDE 3 (CYP708A3)	member of CYP708A The mRNA is cell-to-cell mobile.
AT1G19630	*3,108*	CYTOCHROME P450, FAMILY 722, SUBFAMILY A, POLYPEPTIDE 1 (CYP722A1)	member of CYP722A
AT5G13370	*3,108*		Auxin-responsive GH3 family protein; CONTAINS InterPro DOMAIN/s: GH3 auxin-responsive promoter (InterPro:IPR004993); BEST Arabidopsis thaliana protein match is: Auxin-responsive GH3 family protein (TAIR:AT5G13360.3)
AT4G29770	*3,087*	HEAT-INDUCED TAS1 TARGET 1 (HTT1)	Target of trans acting-siR480/255. Testing.

AT1G71140	*3,058*		MATE efflux family protein; FUNCTIONS IN: antiporter activity, drug transmembrane transporter activity, transporter activity; INVOLVED IN: drug transmembrane transport, transmembrane transport; LOCATED IN: plasma membrane, membrane; EXPRESSED IN: 13 plant structures; EXPRESSED DURING: 8 growth stages; CONTAINS InterPro DOMAIN/s: MATE family transporter related protein (InterPro:IPR015521), Multi antimicrobial extrusion protein MatE (InterPro:IPR002528); BEST Arabidopsis thaliana protein match is: MATE efflux family protein (TAIR:AT1G15150.1)
AT1G62420	*3,048*		Protein of unknown function (DUF506) ; CONTAINS InterPro DOMAIN/s: Protein of unknown function DUF506, plant (InterPro:IPR006502); BEST Arabidopsis thaliana protein match is: Protein of unknown function (DUF506) (TAIR:AT1G12030.1)
AT5G11410	*3,048*		Protein kinase superfamily protein; FUNCTIONS IN: protein kinase activity, kinase activity, ATP binding; INVOLVED IN: protein amino acid phosphorylation; EXPRESSED IN: 12 plant structures; EXPRESSED DURING: 8 growth stages; CONTAINS InterPro DOMAIN/s: Protein kinase, catalytic domain (InterPro:IPR000719), Serine/threonine-protein kinase-like domain (InterPro:IPR017442), Protein kinase-like domain (InterPro:IPR011009); BEST Arabidopsis thaliana protein match is: Protein kinase superfamily protein (TAIR:AT5G11400.2)
AT5G39110	*3,041*		RmlC-like cupins superfamily protein; FUNCTIONS IN: manganese ion binding, nutrient reservoir activity; INVOLVED IN: biological_process unknown; LOCATED IN: endomembrane system, apoplast; CONTAINS InterPro DOMAIN/s: Cupin, RmlC-type (InterPro:IPR011051), Cupin 1 (InterPro:IPR006045), RmlC-like jelly roll fold (InterPro:IPR014710), Germin (InterPro:IPR001929), Germin, manganese binding site (InterPro:IPR019780); BEST Arabidopsis thaliana protein match is: RmlC-like cupins superfamily protein (TAIR:AT5G39150.1)
AT1G74430	*3,037*	MYB DOMAIN PROTEIN 95 (MYB95)	Encodes a putative transcription factor (MYB95). The mRNA is cell-to-cell mobile.
AT4G25920	*3,03*		Protein of unknown function (DUF295); CONTAINS InterPro DOMAIN/s: Protein of unknown function DUF295 (InterPro:IPR005174); BEST Arabidopsis thaliana protein match is: Protein of unknown function (DUF295) (TAIR:AT4G14260.1)
AT2G43520	*3,027*	TRYPSIN INHIBITOR PROTEIN 2 (TI2)	Encodes putative trypsin inhibitor protein which may function in defense against herbivory. Member of the defensin-like (DEFL) family.
AT4G17660	*3,016*		Protein kinase superfamily protein; FUNCTIONS IN: protein serine/threonine kinase activity, protein kinase activity, kinase activity, ATP binding; INVOLVED IN: protein amino acid phosphorylation; LOCATED IN: cellular_component unknown; EXPRESSED IN: 6 plant structures; EXPRESSED DURING: LP.06 six leaves visible, 4 anthesis, C globular stage; CONTAINS InterPro DOMAIN/s: Protein kinase, ATP binding site (InterPro:IPR017441), Protein kinase, catalytic domain (InterPro:IPR000719), Serine/threonine-protein kinase domain (InterPro:IPR002290), Tyrosine-protein kinase, catalytic domain (InterPro:IPR020635), Serine/threonine-protein kinase-like domain (InterPro:IPR017442), Protein kinase-like domain (InterPro:IPR011009), Serine/threonine-protein kinase, active site (InterPro:IPR008271); BEST Arabidopsis thaliana protein match is: Protein kinase superfamily protein (TAIR:AT5G47070.1)
AT3G02480	*3,01*	ABA-RESPONSE PROTEIN (ABR)	Late embryogenesis abundant protein (LEA) family protein; BEST Arabidopsis thaliana protein match is: Late embryogenesis abundant protein (LEA) family protein (TAIR:AT5G38760.1)
AT5G10340	*3,005*		F-box family protein; CONTAINS InterPro DOMAIN/s: F-box domain, cyclin-like (InterPro:IPR001810), F-box domain, Skp2-like (InterPro:IPR022364), F-box associated domain, type 1 (InterPro:IPR006527), F-box associated interaction domain (InterPro:IPR017451); BEST Arabidopsis thaliana protein match is: F-box and associated interaction domains-containing protein (TAIR:AT2G34280.1)
AT1G70130	*2,98*	L-TYPE LECTIN RECEPTOR KINASE V.2 (LECRK-V.2)	Concanavalin A-like lectin protein kinase family protein; FUNCTIONS IN: kinase activity; INVOLVED IN: protein amino acid phosphorylation; LOCATED IN: endomembrane system; EXPRESSED IN: hypocotyl, male gametophyte, root; CONTAINS InterPro DOMAIN/s: Legume lectin, beta chain (InterPro:IPR001220), Protein kinase, ATP binding site (InterPro:IPR017441), Serine/threonine-protein kinase-like domain (InterPro:IPR017442), Concanavalin A-like lectin/glucanase, subgroup (InterPro:IPR013320), Protein kinase-like domain (InterPro:IPR011009), Serine/threonine-protein kinase, active site (InterPro:IPR008271), Protein kinase, catalytic domain (InterPro:IPR000719), Concanavalin A-like lectin/glucanase (InterPro:IPR008985); BEST Arabidopsis thaliana protein match is: Concanavalin A-like lectin protein kinase family protein (TAIR:AT4G29050.1)

AT4G32540	*2,974*	YUCCA 1 (YUC1)	Mutant has elevated levels of free IAA in dominant mutant allele; Flavin Monooxygenase-Like Enzyme; Auxin Biosynthesis
AT1G69920	*2,946*	GLUTATHIONE S-TRANSFERASE TAU 12	Encodes glutathione transferase belonging to the tau class of GSTs. Naming convention according to Wagner et al. (2002).
AT2G03020	*2,941*		Heat shock protein HSP20/alpha crystallin family; FUNCTIONS IN: aspartic-type endopeptidase activity; INVOLVED IN: proteolysis; LOCATED IN: cellular_component unknown; CONTAINS InterPro DOMAIN/s: Heat shock protein Hsp20 (InterPro:IPR002068), Small heat shock protein, predicted, plant (InterPro:IPR016952), Peptidase A1 (InterPro:IPR001461), HSP20-like chaperone (InterPro:IPR008978); BEST Arabidopsis thaliana protein match is: Heat shock protein HSP20/alpha crystallin family (TAIR:AT4G16540.1)
AT5G64870	*2,939*		SPFH/Band 7/PHB domain-containing membrane-associated protein family; FUNCTIONS IN: molecular_function unknown; INVOLVED IN: biological_process unknown; LOCATED IN: plasma membrane, vacuole; EXPRESSED IN: 18 plant structures; EXPRESSED DURING: 11 growth stages; CONTAINS InterPro DOMAIN/s: Band 7 protein (InterPro:IPR001107); BEST Arabidopsis thaliana protein match is: SPFH/Band 7/PHB domain-containing membrane-associated protein family (TAIR:AT5G25250.1)
AT5G38120	*2,927*	(4CL8)	AMP-dependent synthetase and ligase family protein; FUNCTIONS IN: 4-coumarate-CoA ligase activity; INVOLVED IN: metabolic process; EXPRESSED IN: 17 plant structures; EXPRESSED DURING: 9 growth stages; CONTAINS InterPro DOMAIN/s: AMP-binding, conserved site (InterPro:IPR020845), AMP-dependent synthetase/ligase (InterPro:IPR000873); BEST Arabidopsis thaliana protein match is: AMP-dependent synthetase and ligase family protein (TAIR:AT1G20500.1)
AT2G19780	*2,925*		Leucine-rich repeat (LRR) family protein; INVOLVED IN: signal transduction; LOCATED IN: endomembrane system; EXPRESSED IN: 14 plant structures; EXPRESSED DURING: 7 growth stages; CONTAINS InterPro DOMAIN/s: Leucine-rich repeat-containing N-terminal domain, type 2 (InterPro:IPR013210), Leucine-rich repeat (InterPro:IPR001611); BEST Arabidopsis thaliana protein match is: Leucine-rich repeat (LRR) family protein (TAIR:AT4G29240.1)
AT4G12290	*2,911*		Copper amine oxidase family protein; FUNCTIONS IN: quinone binding, primary amine oxidase activity, copper ion binding; INVOLVED IN: oxidation reduction, amine metabolic process; LOCATED IN: vacuole; CONTAINS InterPro DOMAIN/s: Copper amine oxidase, N-terminal (InterPro:IPR016182), Copper amine oxidase, N2-terminal (InterPro:IPR015800), Copper amine oxidase, N2/N3-terminal (InterPro:IPR015801), Copper amine oxidase, N3-terminal (InterPro:IPR015802), Copper amine oxidase (InterPro:IPR000269), Copper amine oxidase, C-terminal (InterPro:IPR015798); BEST Arabidopsis thaliana protein match is: Copper amine oxidase family protein (TAIR:AT1G62810.1)
AT1G44350	*2,909*	IAA-LEUCINE RESISTANT (ILR)-LIKE GENE 6 (ILL6)	encodes a protein similar to IAA amino acid conjugate hydrolase.
AT1G02800	*2,902*	CELLULASE 2 (CEL2)	Encodes a protein with similarity to endo-1,4-b-glucanases and is a member of Glycoside Hydrolase Family 9. CEL2 is induced by nemotodes and is expressed in syncitia induced by Heterodera schachtii.May be involved in the development and function of syncitia.
AT1G22600	*2,9*		Late embryogenesis abundant protein (LEA) family protein; FUNCTIONS IN: molecular_function unknown; INVOLVED IN: biological_process unknown; LOCATED IN: endomembrane system; EXPRESSED IN: stem, embryo, sperm cell, hypocotyl, flower; EXPRESSED DURING: petal differentiation and expansion stage, D bilateral stage; BEST Arabidopsis thaliana protein match is: late embryogenesis abundant domain-containing protein / LEA domain-containing protein (TAIR:AT1G72100.1)
AT3G04000	*2,899*		ChlADR is an aldehyde reductase that catalyzes the reduction of the aldehyde carbonyl groups on saturated and alpha,beta-unsaturated aldehydes with more than 5 carbons in vitro. The N-terminal region of this protein directs GFP to the chloroplast where where ChlADR likely helps to maintain the photosynthetic process by detoxifying reactive carbonyls formed during lipid peroxidation. In addition, this enzyme can also reduce cis-3-hexenal, a major plant volatile compound that contributes to green leaf odor, as well as methylglyoxal in vitro.
AT5G03790	*2,889*	HOMEOBOX 51 (HB51)	Encodes a homeodomain leucine zipper class I (HD-Zip I) meristem identity regulator that acts together with LFY to induce CAL expression. It binds to the CAL promoter proximal CAATNATTG element. LMI1 acts primarily downstream of LFY in meristem identity regulation. The interaction between LFY, LMI1 and CAL resembles a feed-forward loop transcriptional network motif. The gene also had additional LFY-independent roles in leaf morphogenesis and bract formation.

AT2G29450	*2,887*	GLUTATHIONE S-TRANSFERASE TAU 5 (GSTU5)	Encodes a member of the TAU glutathione S-transferase gene family. Gene expression is induced by exposure to auxin, pathogen and herbicides. Naming convention according to Wagner et al. (2002)
AT4G36950	*2,885*	MITOGEN-ACTIVATED PROTEIN KINASE KINASE KINASE 21 (MAPKKK21)	member of MEKK subfamily
AT4G02670	*2,883*	INDETERMINATE(ID)-DOMAIN 12 (IDD12)	indeterminate(ID)-domain 12 (IDD12); FUNCTIONS IN: sequence-specific DNA binding transcription factor activity, zinc ion binding, nucleic acid binding; INVOLVED IN: regulation of transcription; LOCATED IN: intracellular, chloroplast; EXPRESSED IN: shoot apex, embryo, flower, pedicel, seed; EXPRESSED DURING: 4 anthesis, C globular stage, petal differentiation and expansion stage, E expanded cotyledon stage, D bilateral stage; CONTAINS InterPro DOMAIN/s: Zinc finger, C2H2-like (InterPro:IPR015880), Zinc finger, C2H2-type (InterPro:IPR007087), Zinc finger, double-stranded RNA binding (InterPro:IPR022755); BEST Arabidopsis thaliana protein match is: C2H2-like zinc finger protein (TAIR:AT5G66730.1)
AT2G21130	*2,863*		Cyclophilin-like peptidyl-prolyl cis-trans isomerase family protein; FUNCTIONS IN: peptidyl-prolyl cis-trans isomerase activity; INVOLVED IN: protein folding; LOCATED IN: plasma membrane; EXPRESSED IN: 26 plant structures; EXPRESSED DURING: 15 growth stages; CONTAINS InterPro DOMAIN/s: Cyclophilin-like (InterPro:IPR015891), Peptidyl-prolyl cis-trans isomerase, cyclophilin-type (InterPro:IPR002130), Peptidyl-prolyl cis-trans isomerase, cyclophilin-type, conserved site (InterPro:IPR020892); BEST Arabidopsis thaliana protein match is: rotamase CYP 1 (TAIR:AT4G38740.1)
AT4G15530	*2,855*	PYRUVATE ORTHOPHOSPHATE DIKINASE (PPDK)	Encodes a dual-targeted protein believed to act as a pyruvate, orthophosphate dikinase. These enzymes are normally associated with C4 photosynthesis which does not occur in Arabidopsis. However, PPDK may play a role in remobilizing nitrogen during leaf senescence in Arabidopsis. The product of the long transcript (.1 gene model) was shown to be targeted to the chloroplast, whereas the shorter transcript (no targeting sequence) accumulates in the cytosol. The two proteins were also found to be expressed in slightly different tissues.
AT5G57550	*2,855*	XYLOGLUCAN ENDOTRANSGLUCOSYLASE/HYDROLASE 25 (XTH25)	xyloglucan endotransglycosylase-related protein (XTR3)
AT2G42560	*2,848*		late embryogenesis abundant domain-containing protein / LEA domain-containing protein; FUNCTIONS IN: molecular_function unknown; INVOLVED IN: embryo development ending in seed dormancy; LOCATED IN: cellular_component unknown; EXPRESSED IN: seed; EXPRESSED DURING: seedling growth; CONTAINS InterPro DOMAIN/s: Late embryogenesis abundant protein, group 4 (InterPro:IPR004238); BEST Arabidopsis thaliana protein match is: embryonic cell protein 63 (TAIR:AT2G36640.1)
AT5G52320	*2,847*	CYTOCHROME P450, FAMILY 96, SUBFAMILY A, POLYPEPTIDE 4 (CYP96A4)	member of CYP96A
AT5G39120	*2,817*		RmlC-like cupins superfamily protein; FUNCTIONS IN: manganese ion binding, nutrient reservoir activity; INVOLVED IN: biological_process unknown; LOCATED IN: endomembrane system, apoplast; CONTAINS InterPro DOMAIN/s: Cupin, RmlC-type (InterPro:IPR011051), Cupin 1 (InterPro:IPR006045), RmlC-like jelly roll fold (InterPro:IPR014710), Germin (InterPro:IPR001929), Germin, manganese binding site (InterPro:IPR019780); BEST Arabidopsis thaliana protein match is: RmlC-like cupins superfamily protein (TAIR:AT5G39150.1)
AT1G52400	*2,815*	BETA GLUCOSIDASE 18 (BGLU18)	encodes a member of glycosyl hydrolase family 1, located in inducible ER bodies which were formed after wounding, required in inducible ER body formation The mRNA is cell-to-cell mobile.
AT4G14250	*2,812*		This locus is annotated as a protein-coding gene in TAIR10. Based on communication with Jean-Luc GALLOIS (April 2013), this gene is split into two UBX domain-containing pseudogenes: one retains the original name: AT4G14250 (Chr4:8213237..8211984), one given a new locus identifier AT4G14245 (Chr4:8210231..8208985). Note that the Map Detail Image on the locus detial page and in GBrowse will not be updated until after the next genome release.

AT5G06870	*2,811*	POLYGALACTURONASE INHIBITING PROTEIN 2 (PGIP2)	Encodes a polygalacturonase inhibiting protein involved in plant defense response. PGIPs inhibit the activity of pectin degrading enzymes such as those produced by fungal pathogens. PGIP2 is induced by fungal infection and methyl jasmonate.Suppressed in the proton sensitive stop1-mutant, but the transcription level was recovered by transformation of STOP2. Knockout mutant showed severe damage in the root tip in low Ca and low pH medium.
AT1G21890	*2,805*	USUALLY MULTIPLE ACIDS MOVE IN AND OUT TRANSPORTERS 19 (UMAMIT19)	nodulin MtN21-like transporter family protein
AT4G37720	*2,797*	PHYTOSULFOKINE 6 PRECURSOR (PSK6)	Probable phytosulfokines 6 precursor, coding for a unique plant peptide growth factor.
AT4G34400	*2,796*		AP2/B3-like transcriptional factor family protein; FUNCTIONS IN: DNA binding, sequence-specific DNA binding transcription factor activity; INVOLVED IN: regulation of transcription, DNA-dependent; LOCATED IN: endomembrane system; EXPRESSED IN: 17 plant structures; EXPRESSED DURING: 9 growth stages; CONTAINS InterPro DOMAIN/s: Transcriptional factor B3 (InterPro:IPR003340); BEST Arabidopsis thaliana protein match is: AP2/B3-like transcriptional factor family protein (TAIR:AT3G06160.2)
AT2G37870	*2,79*		Bifunctional inhibitor/lipid-transfer protein/seed storage 2S albumin superfamily protein; FUNCTIONS IN: lipid binding; INVOLVED IN: lipid transport; LOCATED IN: endomembrane system; EXPRESSED IN: 11 plant structures; EXPRESSED DURING: LP.06 six leaves visible, LP.04 four leaves visible, 4 anthesis, C globular stage, petal differentiation and expansion stage; CONTAINS InterPro DOMAIN/s: Bifunctional inhibitor/plant lipid transfer protein/seed storage (InterPro:IPR016140), Plant lipid transfer protein/seed storage/trypsin-alpha amylase inhibitor (InterPro:IPR003612), Plant lipid transfer protein/Par allergen (InterPro:IPR000528); BEST Arabidopsis thaliana protein match is: Bifunctional inhibitor/lipid-transfer protein/seed storage 2S albumin superfamily protein (TAIR:AT5G05960.1)
AT5G66110	*2,785*	HEAVY METAL ASSOCIATED ISOPRENYLATED PLANT PROTEIN 27 (HIPP27)	Heavy metal transport/detoxification superfamily protein ; FUNCTIONS IN: metal ion binding; INVOLVED IN: metal ion transport; LOCATED IN: plasma membrane; EXPRESSED IN: cultured cell, leaf; EXPRESSED DURING: LP.04 four leaves visible; CONTAINS InterPro DOMAIN/s: Heavy metal transport/detoxification protein (InterPro:IPR006121); BEST Arabidopsis thaliana protein match is: farnesylated protein 6 (TAIR:AT4G38580.1)
AT2G41470	*2,767*		unknown protein; FUNCTIONS IN: molecular_function unknown; INVOLVED IN: biological_process unknown; LOCATED IN: vacuole; BEST Arabidopsis thaliana protein match is: unknown protein (TAIR:AT2G41440.1)
AT1G72520	*2,766*	LIPOXYGENASE 4 (LOX4)	PLAT/LH2 domain-containing lipoxygenase family protein; FUNCTIONS IN: oxidoreductase activity, acting on single donors with incorporation of molecular oxygen, incorporation of two atoms of oxygen, lipoxygenase activity, iron ion binding, metal ion binding; INVOLVED IN: growth, jasmonic acid biosynthetic process, response to wounding, defense response; LOCATED IN: chloroplast; EXPRESSED IN: 17 plant structures; EXPRESSED DURING: 8 growth stages; CONTAINS InterPro DOMAIN/s: Lipoxygenase, iron binding site (InterPro:IPR020833), Lipoxygenase, C-terminal (InterPro:IPR013819), Lipoxygenase, LH2 (InterPro:IPR001024), Lipase/lipooxygenase, PLAT/LH2 (InterPro:IPR008976), Lipoxygenase, conserved site (InterPro:IPR020834), Lipoxygenase (InterPro:IPR000907), Lipoxygenase, plant (InterPro:IPR001246); BEST Arabidopsis thaliana protein match is: lipoxygenase 3 (TAIR:AT1G17420.1)
AT4G37710	*2,748*	VQ MOTIF-CONTAINING PROTEIN 29 (VQ29)	VQ motif-containing protein; FUNCTIONS IN: molecular_function unknown; INVOLVED IN: biological_process unknown; LOCATED IN: chloroplast; EXPRESSED IN: pollen tube; CONTAINS InterPro DOMAIN/s: VQ (InterPro:IPR008889); BEST Arabidopsis thaliana protein match is: VQ motif-containing protein (TAIR:AT2G22880.1)
AT1G03445	*2,744*	BRI1 SUPPRESSOR 1 (BSU1)	encodes a serine?threonine protein phosphatase with an N-terminal Kelch-repeat domain, which is nuclear localized and expressed preferentially in elongating cells. Genetic evidence suggest that this gene plays a redundant role (along with other members of the same gene family) in modulating growth in response to brassinosteroid.
AT2G29440	*2,738*	GLUTATHIONE S-TRANSFERASE TAU 6 (GSTU6)	Encodes glutathione transferase belonging to the tau class of GSTs. Naming convention according to Wagner et al. (2002).
AT2G41300	*2,738*	STRICTOSIDINE SYNTHASE-LIKE 1 (SSL1)	Although this enzyme is predicted to encode a strictosidine synthase (SS), it lacks a conserved catalytic glutamate residue found in active SS enzymes and it is not expected to have SS activity.

AT3G24650	*2,736*	ABA INSENSITIVE 3 (ABI3)	Homologous to the maize transcription factor Viviparous-1. Full length ABI3 protein binds to the highly conserved RY motif [DNA motif CATGCA(TG)], present in many seed-specific promoters, and the B3 domains of this transcription factor is necessary for the specific interaction with the RY element. Transcriptional activity of ABI3 requires the B3 DNA-binding domain and an activation domain. In addition to the known N-terminal-located activation domain, a second transcription activation domain was found in the B1 region of ABI3. ABI3 is essential for seed maturation. Regulator of the transition between embryo maturation and early seedling development. Putative seed-specific transcriptional activator. ABI3 is a central regulator in ABA signaling and is unstable in vivo. It interacts with and can by polyubiquitinated by AIP2 in vivo. Based on double mutant analyses, ABI3 interacts genetically with both FUS3 and LEC1 and is involved in controlling accumulation of chlorophyll and anthocyanins, sensitivity to abscisic acid, and expression of the members of the 12S storage protein gene family. In addition, both FUS3 and LEC1 regulate positively the abundance of the ABI3 protein in the seed. Alternative splicing of ABI3 is developmentally regulated by SUA (AT3G54230).
AT3G12145	*2,734*	FLOR1 (FLR1)	A novel leucine-rich repeat protein. Interacts directly with MADS domain transcription factor.
AT2G32830	*2,731*	PHOSPHATE TRANSPORTER 1;5 (PHT1;5)	Encodes Pht1;5, a member of the Pht1 family of phosphate transporters which include: Pht1;1/At5g43350, Pht1;2/At5g43370, Pht1;3/At5g43360, Pht1;4/At2g38940, Pht1;5/At2g32830, Pht1;6/At5g43340, Pht1;7/At3g54700, Pht1;8/At1g20860, Pht1;9/At1g76430 (Plant Journal 2002, 31:341).
AT1G32900	*2,729*	GRANULE BOUND STARCH SYNTHASE 1 (GBSS1)	UDP-Glycosyltransferase superfamily protein; FUNCTIONS IN: protein binding, transferase activity, transferring glycosyl groups; INVOLVED IN: biosynthetic process, glucan biosynthetic process; LOCATED IN: chloroplast; EXPRESSED IN: 21 plant structures; EXPRESSED DURING: 13 growth stages; CONTAINS InterPro DOMAIN/s: Glycogen/starch synthases, ADP-glucose type (InterPro:IPR011835), Starch synthase, catalytic domain (InterPro:IPR013534), Glycosyl transferase, group 1 (InterPro:IPR001296); BEST Arabidopsis thaliana protein match is: starch synthase 2 (TAIR:AT3G01180.1)
AT5G52310	*2,729*	LOW-TEMPERATURE-INDUCED 78 (LTI78)	cold regulated gene, the 5' region of cor78 has cis-acting regulatory elements that can impart cold-regulated gene expression The mRNA is cell-to-cell mobile.
AT2G40610	*2,716*	EXPANSIN A8 (EXPA8)	member of Alpha-Expansin Gene Family. Naming convention from the Expansin Working Group (Kende et al, 2004. Plant Mol Bio). Involved in the formation of nematode-induced syncytia in roots of Arabidopsis thaliana.
AT4G35650	*2,71*	ISOCITRATE DEHYDROGENASE III (IDH-III)	Encodes a regulatory subunit of the mitochondrially-localized NAD+- dependent isocitrate dehydrogenase. In contrast to the broadly expressed other regulatory (IDH-I and IDH-II) and catalytic (IDH-V and IDH-VI) subunits of this enzyme, IDH-III expression appears to be restricted largely to pollen.
AT5G07800	*2,706*		Flavin-binding monooxygenase family protein; FUNCTIONS IN: NADP or NADPH binding, monooxygenase activity, FAD binding, flavin-containing monooxygenase activity; INVOLVED IN: oxidation reduction; LOCATED IN: cellular_component unknown; EXPRESSED IN: 16 plant structures; EXPRESSED DURING: 6 growth stages; CONTAINS InterPro DOMAIN/s: Flavin-containing monooxygenase FMO (InterPro:IPR000960), Flavin-containing monooxygenase-like (InterPro:IPR020946); BEST Arabidopsis thaliana protein match is: Flavin-binding monooxygenase family protein (TAIR:AT5G61290.1)
AT3G14450	*2,702*	CTC-INTERACTING DOMAIN 9 (CID9)	RNA-binding protein, putative, contains Pfam profile: PF00076 RNA recognition motif. (a.k.a. RRM, RBD, or RNP domain) (2 copies). Contains PAM PABC binding domain.
AT2G39330	*2,7*	JACALIN-RELATED LECTIN 23 (JAL23)	jacalin-related lectin 23 (JAL23); FUNCTIONS IN: molecular_function unknown; INVOLVED IN: biological_process unknown; LOCATED IN: vacuole; EXPRESSED IN: 24 plant structures; EXPRESSED DURING: 14 growth stages; CONTAINS InterPro DOMAIN/s: Mannose-binding lectin (InterPro:IPR001229); BEST Arabidopsis thaliana protein match is: jacalin-related lectin 22 (TAIR:AT2G39310.3)
AT2G18050	*2,699*	HISTONE H1-3 (HIS1-3)	encodes a structurally divergent linker histone whose gene expression is induced by dehydration and ABA. The mRNA is cell-to-cell mobile.
AT4G12830	*2,698*		alpha/beta-Hydrolases superfamily protein; FUNCTIONS IN: hydrolase activity, catalytic activity; LOCATED IN: chloroplast; EXPRESSED IN: 15 plant structures; EXPRESSED DURING: 9 growth stages; CONTAINS InterPro DOMAIN/s: Epoxide hydrolase-like (InterPro:IPR000639), Alpha/beta hydrolase fold-1 (InterPro:IPR000073); BEST Arabidopsis thaliana protein match is: alpha/beta-Hydrolases superfamily protein (TAIR:AT1G52510.1)

AT2G47950	*2,695*		unknown protein; FUNCTIONS IN: molecular_function unknown; INVOLVED IN: biological_process unknown; LOCATED IN: endomembrane system; EXPRESSED IN: root, flower; EXPRESSED DURING: petal differentiation and expansion stage; BEST Arabidopsis thaliana protein match is: unknown protein (TAIR:AT3G62990.1)
AT1G19610	*2,686*	(PDF1.4)	Predicted to encode a PR (pathogenesis-related) protein. Belongs to the plant defensin (PDF) family with the following members: At1g75830/PDF1.1, At5g44420/PDF1.2a, At2g26020/PDF1.2b, At5g44430/PDF1.2c, At2g26010/PDF1.3, At1g19610/PDF1.4, At1g55010/PDF1.5, At2g02120/PDF2.1, At2g02100/PDF2.2, At2g02130/PDF2.3, At1g61070/PDF2.4, At5g63660/PDF2.5, At2g02140/PDF2.6, At5g38330/PDF3.1 and At4g30070/PDF3.2.
AT2G30760	*2,683*		unknown protein
AT5G01900	*2,68*	WRKY DNA-BINDING PROTEIN 62 (WRKY62)	member of WRKY Transcription Factor; Group III
AT5G06730	*2,673*		Peroxidase superfamily protein; FUNCTIONS IN: peroxidase activity, heme binding; INVOLVED IN: response to oxidative stress, oxidation reduction; LOCATED IN: plasma membrane; EXPRESSED IN: root, cultured cell; CONTAINS InterPro DOMAIN/s: Haem peroxidase (InterPro:IPR010255), Plant peroxidase (InterPro:IPR000823), Peroxidases heam-ligand binding site (InterPro:IPR019793), Haem peroxidase, plant/fungal/bacterial (InterPro:IPR002016), Peroxidase, active site (InterPro:IPR019794); BEST Arabidopsis thaliana protein match is: peroxidase 2 (TAIR:AT5G06720.1)
AT5G60250	*2,673*		zinc finger (C3HC4-type RING finger) family protein; FUNCTIONS IN: zinc ion binding, nucleic acid binding; EXPRESSED IN: 14 plant structures; EXPRESSED DURING: 8 growth stages; CONTAINS InterPro DOMAIN/s: Zinc finger, RING-type, conserved site (InterPro:IPR017907), Zinc finger, C6HC-type (InterPro:IPR002867), Zinc finger, RING-type (InterPro:IPR001841), Polynucleotidyl transferase, ribonuclease H fold (InterPro:IPR012337); BEST Arabidopsis thaliana protein match is: RING/U-box superfamily protein (TAIR:AT2G25370.1)
AT3G53200	*2,667*	MYB DOMAIN PROTEIN 27 (MYB27)	Member of the R2R3 factor gene family.
AT5G28490	*2,667*	LIGHT-DEPENDENT SHORT HYPOCOTYLS 1 (LSH1)	Encodes a nuclear protein that mediates light regulation of seedling development in a phytochrome-dependent manner.
AT2G36110	*2,664*		Polynucleotidyl transferase, ribonuclease H-like superfamily protein; FUNCTIONS IN: 3'-5' exonuclease activity, nucleic acid binding; INVOLVED IN: nucleobase, nucleoside, nucleotide and nucleic acid metabolic process; LOCATED IN: intracellular; CONTAINS InterPro DOMAIN/s: Polynucleotidyl transferase, ribonuclease H fold (InterPro:IPR012337), 3'-5' exonuclease (InterPro:IPR002562); BEST Arabidopsis thaliana protein match is: Polynucleotidyl transferase, ribonuclease H-like superfamily protein (TAIR:AT3G12460.1)
AT2G21260	*2,663*		NAD(P)-linked oxidoreductase superfamily protein; FUNCTIONS IN: oxidoreductase activity; INVOLVED IN: oxidation reduction; CONTAINS InterPro DOMAIN/s: Aldo/keto reductase (InterPro:IPR001395), Aldo/keto reductase subgroup (InterPro:IPR020471), Aldo/keto reductase, conserved site (InterPro:IPR018170); BEST Arabidopsis thaliana protein match is: NAD(P)-linked oxidoreductase superfamily protein (TAIR:AT2G21250.1)
AT2G33420	*2,663*		CONTAINS InterPro DOMAIN/s: Munc13 homology 1 (InterPro:IPR014770), Protein of unknown function DUF810 (InterPro:IPR008528), Mammalian uncoordinated homology 13, domain 2 (InterPro:IPR014772); BEST Arabidopsis thaliana protein match is: Protein of unknown function (DUF810) (TAIR:AT1G04470.1)
AT2G37900	*2,661*		Major facilitator superfamily protein; FUNCTIONS IN: transporter activity; INVOLVED IN: oligopeptide transport; LOCATED IN: membrane; EXPRESSED IN: 15 plant structures; EXPRESSED DURING: 7 growth stages; CONTAINS InterPro DOMAIN/s: PTR2 family proton/oligopeptide symporter, conserved site (InterPro:IPR018456), Oligopeptide transporter (InterPro:IPR000109), Major facilitator superfamily, general substrate transporter (InterPro:IPR016196); BEST Arabidopsis thaliana protein match is: Major facilitator superfamily protein (TAIR:AT3G53960.1)
AT2G23590	*2,653*	METHYL ESTERASE 8 (MES8)	Encodes a protein shown to have carboxylesterase activity in vitro. It has similarity to the SABP2 methyl salicylate esterase from tobacco. This protein does not act on methyl IAA, methyl JA, MeSA, MeGA4, or MEGA9 in vitro.
AT5G61730	*2,647*	ATP-BINDING CASSETTE A9 (ABCA9)	Encodes an ER-localized ABC transporter with a role in the supply of fatty acid substrates for TAG biosynthesis at the ER during the seed-filling stage.

AT1G17420	*2,641*	LIPOXYGENASE 3 (LOX3)	LOX3 encode a Lipoxygenase. Lipoxygenases (LOXs) catalyze the oxygenation of fatty acids (FAs).
AT1G69880	*2,631*	THIOREDOXIN H-TYPE 8 (TH8)	thioredoxin H-type 8 (TH8); INVOLVED IN: N-terminal protein myristoylation, cell redox homeostasis; LOCATED IN: cellular_component unknown; EXPRESSED IN: 15 plant structures; EXPRESSED DURING: 6 growth stages; CONTAINS InterPro DOMAIN/s: Thioredoxin fold (InterPro:IPR012335), Thioredoxin, core (InterPro:IPR015467), Thioredoxin-like (InterPro:IPR017936), Thioredoxin domain (InterPro:IPR013766), Thioredoxin-like fold (InterPro:IPR012336), Thioredoxin, conserved site (InterPro:IPR017937); BEST Arabidopsis thaliana protein match is: thioredoxin H-type 7 (TAIR:AT1G59730.1)
AT1G09950	*2,629*	RESPONSE TO ABA AND SALT 1 (RAS1)	RESPONSE TO ABA AND SALT 1 (RAS1); FUNCTIONS IN: molecular_function unknown; INVOLVED IN: biological_process unknown; LOCATED IN: endomembrane system; EXPRESSED IN: leaf apex, root, flower, leaf; EXPRESSED DURING: petal differentiation and expansion stage; BEST Arabidopsis thaliana protein match is: transcription factor-related (TAIR:AT1G58330.1)
AT1G16850	*2,627*		unknown protein; FUNCTIONS IN: molecular_function unknown; INVOLVED IN: response to salt stress; LOCATED IN: endomembrane system; EXPRESSED IN: leaf apex, leaf whorl, male gametophyte, flower, leaf; EXPRESSED DURING: LP.06 six leaves visible, LP.04 four leaves visible, LP.10 ten leaves visible, petal differentiation and expansion stage, LP.08 eight leaves visible; BEST Arabidopsis thaliana protein match is: unknown protein (TAIR:AT5G64820.1)
AT4G11480	*2,602*	CYSTEINE-RICH RLK (RECEPTOR-LIKE PROTEIN KINASE) 32 (CRK32)	Encodes a cysteine-rich receptor-like protein kinase.
AT1G26770	*2,588*	EXPANSIN A10 (EXPA10)	Encodes an expansin. Naming convention from the Expansin Working Group (Kende et al, Plant Mol Bio). Involved in the formation of nematode-induced syncytia in roots of Arabidopsis thaliana.
AT1G77380	*2,585*	AMINO ACID PERMEASE 3 (AAP3)	Amino acid permease which transports basic amino acids.
AT4G14690	*2,578*	EARLY LIGHT-INDUCIBLE PROTEIN 2 (ELIP2)	Encodes an early light-induced protein. ELIPs are thought not to be directly involved in the synthesis and assembly of specific photosynthetic complexes, but rather affect the biogenesis of all chlorophyll-binding complexes. A study (PMID 17553115) has shown that the chlorophyll synthesis pathway was downregulated as a result of constitutive ELIP2 expression, leading to decreased chlorophyll availability for the assembly of pigment-binding proteins for photosynthesis.
AT2G16890	*2,574*		UDP-Glycosyltransferase superfamily protein; FUNCTIONS IN: transferase activity, transferring hexosyl groups, UDP-glycosyltransferase activity, transferase activity, transferring glycosyl groups; INVOLVED IN: metabolic process; LOCATED IN: endomembrane system; EXPRESSED IN: 17 plant structures; EXPRESSED DURING: 7 growth stages; CONTAINS InterPro DOMAIN/s: UDP-glucuronosyl/UDP-glucosyltransferase (InterPro:IPR002213), Tudor subgroup (InterPro:IPR018351); BEST Arabidopsis thaliana protein match is: UDP-Glycosyltransferase superfamily protein (TAIR:AT5G14860.1)
AT1G54020	*2,572*		GDSL-motif esterase/acyltransferase/lipase. Enzyme group with broad substrate specificity that may catalyze acyltransfer or hydrolase reactions with lipid and non-lipid substrates.
AT3G25620	*2,572*	ATP-BINDING CASSETTE G21 (ABCG21)	ABC-2 type transporter family protein; FUNCTIONS IN: ATPase activity, coupled to transmembrane movement of substances; LOCATED IN: chloroplast, membrane; EXPRESSED IN: 17 plant structures; EXPRESSED DURING: 9 growth stages; CONTAINS InterPro DOMAIN/s: ATPase, AAA+ type, core (InterPro:IPR003593), ABC transporter-like (InterPro:IPR003439), ABC transporter, conserved site (InterPro:IPR017871), ABC-2 type transporter (InterPro:IPR013525); BEST Arabidopsis thaliana protein match is: ATP-binding cassette 14 (TAIR:AT1G31770.1).
AT2G37640	*2,57*	(EXP3)	member of Alpha-Expansin Gene Family. Naming convention from the Expansin Working Group (Kende et al, 2004. Plant Mol Bio). Involved in the formation of nematode-induced syncytia in roots of Arabidopsis thaliana.
AT4G33280	*2,559*		AP2/B3-like transcriptional factor family protein; FUNCTIONS IN: DNA binding, sequence-specific DNA binding transcription factor activity; INVOLVED IN: regulation of transcription, DNA-dependent; LOCATED IN: vacuole; CONTAINS InterPro DOMAIN/s: Transcriptional factor B3 (InterPro:IPR003340); BEST Arabidopsis thaliana protein match is: AP2/B3-like transcriptional factor family protein (TAIR:AT3G18990.1)

AT3G05630	*2,55*	PHOSPHOLIPASE D P2 (PLDP2)	Encodes a member of the PXPH-PLD subfamily of phospholipase D proteins. Regulates vesicle trafficking. Required for auxin transport and distribution and hence auxin responses. This subfamily is novel structurally different from the majority of plant PLDs by having phox homology (PX) and pleckstrin homology (PH) domains. Involved regulating root development in response to nutrient limitation. Plays a major role in phosphatidic acid production during phosphate deprivation. Induced upon Pi starvation in both shoots and roots. Involved in hydrolyzing phosphatidylcholine and phosphatidylethanolamine to produce diacylglycerol for digalactosyldiacylglycerol synthesis and free Pi to sustain other Pi-requiring processes. Does not appear to be involved in root hair patterning. Expression is upregulated in the shoot of cax1/cax3 mutant and is responsive to phosphate (Pi) and not phosphite (Phi) in roots and shoots.
AT1G56150	*2,546*	SMALL AUXIN UPREGULATED 71 (SAUR71)	SAUR-like auxin-responsive protein family ; CONTAINS InterPro DOMAIN/s: Auxin responsive SAUR protein (InterPro:IPR003676); BEST Arabidopsis thaliana protein match is: SAUR-like auxin-responsive protein family (TAIR:AT3G12830.1)
AT2G06050	*2,545*	OXOPHYTODIENOATE-REDUCTASE 3 (OPR3)	Encodes a 12-oxophytodienoate reductase that is required for jasmonate biosynthesis. Mutants are male sterile and defective in pollen dehiscence. Shows activity towards 2,4,6-trinitrotoluene. CFA-Ile, CFA-Leu, CFA-Val, CFA-Met and CFA-Ala can restore the fertility of opr3 plants by inducing filament elongation and anther dehiscence.
AT3G55515	*2,544*	ROTUNDIFOLIA LIKE 7 (RTFL7)	ROTUNDIFOLIA like 7 (RTFL7); CONTAINS InterPro DOMAIN/s: DVL (InterPro:IPR012552); BEST Arabidopsis thaliana protein match is: ROTUNDIFOLIA like 8 (TAIR:AT2G39705.1)
AT4G02280	*2,54*	SUCROSE SYNTHASE 3 (SUS3)	Encodes a protein with sucrose synthase activity (SUS3). It appears to be important for sucrose metabolism in developing seeds, especially during the late maturation phase, about 18 days after flowering.
AT5G43330	*2,537*	CYTOSOLIC-NAD-DEPENDENT MALATE DEHYDROGENASE 2 (c-NAD-MDH2)	predicted to encode a cytosolic malate dehydrogenase. The mRNA is cell-to-cell mobile.
AT2G45600	*2,53*		alpha/beta-Hydrolases superfamily protein; FUNCTIONS IN: hydrolase activity; INVOLVED IN: metabolic process; LOCATED IN: cellular_component unknown; EXPRESSED IN: 20 plant structures; EXPRESSED DURING: 8 growth stages; CONTAINS InterPro DOMAIN/s: Lipase, GDXG, active site (InterPro:IPR002168), Alpha/beta hydrolase fold-3 (InterPro:IPR013094); BEST Arabidopsis thaliana protein match is: alpha/beta-Hydrolases superfamily protein (TAIR:AT2G45610.1)
AT5G40380	*2,528*	CYSTEINE-RICH RLK (RECEPTOR-LIKE PROTEIN KINASE) 42 (CRK42)	Encodes a cysteine-rich receptor-like protein kinase.
AT4G23220	*2,524*	CYSTEINE-RICH RLK (RECEPTOR-LIKE PROTEIN KINASE) 14 (CRK14)	Encodes a cysteine-rich receptor-like protein kinase.
AT3G50440	*2,515*	METHYL ESTERASE 10 (MES10)	Encodes a protein shown to have methyl jasmonate esterase activity in vitro. This protein does not act on methyl IAA, MeSA, MeGA4, or MEGA9 in vitro.
AT4G30430	*2,507*	TETRASPANIN9 (TET9)	Member of TETRASPANIN family
AT4G15440	*2,506*	HYDROPEROXIDE LYASE 1 (HPL1)	Encodes a hydroperoxide lyase. Also a member of the CYP74B cytochrome p450 family. In the ecotype Columbia (Col) the gene contains a 10-nucleotide deletion in its first exon that causes it to code for a truncated protein that results in a non-functional hydroperoxide lyase.
AT5G01380	*2,505*		Homeodomain-like superfamily protein; CONTAINS InterPro DOMAIN/s: SANT, DNA-binding (InterPro:IPR001005), MYB-like (InterPro:IPR017877); BEST Arabidopsis thaliana protein match is: Homeodomain-like superfamily protein (TAIR:AT2G38250.1)
AT5G24600	*2,5*		Protein of unknown function, DUF599; INVOLVED IN: biological_process unknown; LOCATED IN: plasma membrane; EXPRESSED IN: 15 plant structures; EXPRESSED DURING: 6 growth stages; CONTAINS InterPro DOMAIN/s: Protein of unknown function DUF599 (InterPro:IPR006747); BEST Arabidopsis thaliana protein match is: Protein of unknown function, DUF599 (TAIR:AT3G18215.1)
AT1G67855	*2,498*		unknown protein
AT4G03620	*2,49*		myosin heavy chain-related

AT4G18810	*2,476*		NAD(P)-binding Rossmann-fold superfamily protein; FUNCTIONS IN: binding, catalytic activity; INVOLVED IN: metabolic process; EXPRESSED IN: 22 plant structures; EXPRESSED DURING: 13 growth stages; CONTAINS InterPro DOMAIN/s: NAD(P)-binding domain (InterPro:IPR016040), NADH:ubiquinone oxidoreductase intermediate-associated protein 30 (InterPro:IPR013857), NmrA-like (InterPro:IPR008030); BEST Arabidopsis thaliana protein match is: high chlorophyll fluorescence phenotype 173 (TAIR:AT1G16720.1).
AT5G56700	*2,47*		FBD / Leucine Rich Repeat domains containing protein; CONTAINS InterPro DOMAIN/s: FBD (InterPro:IPR013596), Leucine-rich repeat 2 (InterPro:IPR013101); BEST Arabidopsis thaliana protein match is: F-box/RNI-like superfamily protein (TAIR:AT5G60610.1)
AT4G12320	*2,456*	CYTOCHROME P450, FAMILY 706, SUBFAMILY A, POLYPEPTIDE 6 (CYP706A6)	member of CYP706A
AT5G53190	*2,452*	(SWEET3)	Nodulin MtN3 family protein; INVOLVED IN: biological_process unknown; LOCATED IN: integral to membrane, membrane; EXPRESSED IN: 8 plant structures; EXPRESSED DURING: 4 anthesis, C globular stage, petal differentiation and expansion stage, E expanded cotyledon stage; CONTAINS InterPro DOMAIN/s: MtN3/saliva-related transmembrane protein, conserved region (InterPro:IPR018169), RAG1-activating protein 1 homologue (InterPro:IPR018179), RAG1-activating protein-1-related (InterPro:IPR004316); BEST Arabidopsis thaliana protein match is: Nodulin MtN3 family protein (TAIR:AT1G21460.1)
AT1G70830	*2,443*	MLP-LIKE PROTEIN 28 (MLP28)	MLP-like protein 28 (MLP28); FUNCTIONS IN: molecular_function unknown; INVOLVED IN: response to biotic stimulus, defense response; LOCATED IN: cellular_component unknown; EXPRESSED IN: 22 plant structures; EXPRESSED DURING: 14 growth stages; CONTAINS InterPro DOMAIN/s: Bet v I allergen (InterPro:IPR000916); BEST Arabidopsis thaliana protein match is: MLP-like protein 34 (TAIR:AT1G70850.3)
AT2G19920	*2,441*		RNA-dependent RNA polymerase family protein; CONTAINS InterPro DOMAIN/s: RNA-dependent RNA polymerase, eukaryotic-type (InterPro:IPR007855); BEST Arabidopsis thaliana protein match is: RNA-dependent RNA polymerase family protein (TAIR:AT2G19910.1)
AT2G33020	*2,44*	RECEPTOR LIKE PROTEIN 24 (RLP24)	receptor like protein 24 (RLP24); FUNCTIONS IN: kinase activity; INVOLVED IN: signal transduction; LOCATED IN: endomembrane system; EXPRESSED IN: root, cultured cell; CONTAINS InterPro DOMAIN/s: Leucine-rich repeat (InterPro:IPR001611); BEST Arabidopsis thaliana protein match is: receptor like protein 23 (TAIR:AT2G32680.1)
AT2G45570	*2,436*	CYTOCHROME P450, FAMILY 76, SUBFAMILY C, POLYPEPTIDE 2 (CYP76C2)	member of CYP76C
AT3G17690	*2,435*	CYCLIC NUCLEOTIDE GATED CHANNEL 19 (CNGC19)	member of Cyclic nucleotide gated channel family
AT5G25970	*2,424*		Core-2/I-branching beta-1,6-N-acetylglucosaminyltransferase family protein; CONTAINS InterPro DOMAIN/s: Core-2/I-Branching enzyme (InterPro:IPR021141); BEST Arabidopsis thaliana protein match is: Core-2/I-branching beta-1,6-N-acetylglucosaminyltransferase family protein (TAIR:AT5G11730.1)
AT2G22590	*2,419*		UDP-Glycosyltransferase superfamily protein; FUNCTIONS IN: transferase activity, transferring glycosyl groups; INVOLVED IN: metabolic process; LOCATED IN: cellular_component unknown; CONTAINS InterPro DOMAIN/s: UDP-glucuronosyl/UDP-glucosyltransferase (InterPro:IPR002213); BEST Arabidopsis thaliana protein match is: UDP-Glycosyltransferase superfamily protein (TAIR:AT5G65550.1)
AT5G24820	*2,418*		Eukaryotic aspartyl protease family protein; FUNCTIONS IN: aspartic-type endopeptidase activity; INVOLVED IN: proteolysis; LOCATED IN: endomembrane system; EXPRESSED IN: leaf whorl, flower, seed; EXPRESSED DURING: petal differentiation and expansion stage, E expanded cotyledon stage; CONTAINS InterPro DOMAIN/s: Peptidase aspartic (InterPro:IPR021109), Peptidase aspartic, catalytic (InterPro:IPR009007), Peptidase A1 (InterPro:IPR001461); BEST Arabidopsis thaliana protein match is: Eukaryotic aspartyl protease family protein (TAIR:AT2G35615.1)

AT1G65890	*2,411*	ACYL ACTIVATING ENZYME 12 (AAE12)	acyl activating enzyme 12 (AAE12); FUNCTIONS IN: catalytic activity; INVOLVED IN: metabolic process; EXPRESSED IN: leaf apex, hypocotyl, flower; EXPRESSED DURING: petal differentiation and expansion stage; CONTAINS InterPro DOMAIN/s: AMP-dependent synthetase/ligase (InterPro:IPR000873); BEST Arabidopsis thaliana protein match is: benzoyloxyglucosinolate 1 (TAIR:AT1G65880.1)
AT4G17090	*2,402*	CHLOROPLAST BETA-AMYLASE (CT-BMY)	Encodes a beta-amylase targeted to the chloroplast. Transgenic BMY8 RNAi lines fail to accumulate maltose during cold shock suggesting that maltose accumulation coincides with BMY8 expression. Apart from maltose, the sugar content of the RNAi lines were similar to wildtype (glucose and sucrose unaffected).
AT3G52770	*2,401*	LITTLE ZIPPER 3 (ZPR3)	ZPR3 is a small-leucine zipper containing protein that is involved in the establishment of leaf polarity.
AT1G31290	*2,4*	ARGONAUTE 3 (AGO3)	ARGONAUTE 3 (AGO3); FUNCTIONS IN: nucleic acid binding; INVOLVED IN: biological_process unknown; LOCATED IN: cellular_component unknown; EXPRESSED IN: 11 plant structures; EXPRESSED DURING: LP.06 six leaves visible, LP.04 four leaves visible, 4 anthesis, C globular stage, petal differentiation and expansion stage; CONTAINS InterPro DOMAIN/s: Domain of unknown function DUF1785 (InterPro:IPR014811), Stem cell self-renewal protein Piwi (InterPro:IPR003165), Argonaute/Dicer protein, PAZ (InterPro:IPR003100), Polynucleotidyl transferase, ribonuclease H fold (InterPro:IPR012337); BEST Arabidopsis thaliana protein match is: Argonaute family protein (TAIR:AT1G31280.1)
AT4G17970	*2,393*	ALUMINUM-ACTIVATED, MALATE TRANSPORTER 12 (ALMT12)	Anion transporter involved in stomatal closure. Gene has 3 splicing variants.
AT5G62160	*2,391*	ZINC TRANSPORTER 12 PRECURSOR (ZIP12)	member of Fe(II) transporter isolog family
AT5G64060	*2,39*	NAC DOMAIN CONTAINING PROTEIN 103 (NAC103)	NAC domain containing protein 103 (NAC103); FUNCTIONS IN: sequence-specific DNA binding transcription factor activity; INVOLVED IN: multicellular organismal development, regulation of transcription; LOCATED IN: cellular_component unknown; EXPRESSED IN: 9 plant structures; EXPRESSED DURING: 6 growth stages; CONTAINS InterPro DOMAIN/s: No apical meristem (NAM) protein (InterPro:IPR003441); BEST Arabidopsis thaliana protein match is: NAC domain containing protein 82 (TAIR:AT5G09330.4)
AT4G39410	*2,388*	WRKY DNA-BINDING PROTEIN 13 (WRKY13)	Encodes a member of the Group II-c WRKY Transcription Factor family that is involved in stem development and has been shown to directly bind to the promoter of NST2. Mutants show a weak stem phenotype and show decreased expression of lignin-synthesis-related genes.
AT5G60470	*2,387*	EGRET (EGRET)	C2H2 and C2HC zinc fingers superfamily protein; FUNCTIONS IN: sequence-specific DNA binding transcription factor activity, zinc ion binding, nucleic acid binding; INVOLVED IN: regulation of transcription; LOCATED IN: intracellular; EXPRESSED IN: stem, sepal, carpel, stamen; EXPRESSED DURING: 4 anthesis; CONTAINS InterPro DOMAIN/s: Zinc finger, C2H2-like (InterPro:IPR015880), Zinc finger, C2H2-type (InterPro:IPR007087), Zinc finger, double-stranded RNA binding (InterPro:IPR022755); BEST Arabidopsis thaliana protein match is: C2H2-like zinc finger protein (TAIR:AT3G45260.1)
AT1G31550	*2,379*		GDSL-motif esterase/acyltransferase/lipase. Enzyme group with broad substrate specificity that may catalyze acyltransfer or hydrolase reactions with lipid and non-lipid substrates.
AT5G39130	*2,378*		RmlC-like cupins superfamily protein; FUNCTIONS IN: manganese ion binding, nutrient reservoir activity; INVOLVED IN: biological_process unknown; LOCATED IN: endomembrane system, apoplast; CONTAINS InterPro DOMAIN/s: Cupin, RmlC-type (InterPro:IPR011051), Cupin 1 (InterPro:IPR006045), RmlC-like jelly roll fold (InterPro:IPR014710), Germin (InterPro:IPR001929), Germin, manganese binding site (InterPro:IPR019780); BEST Arabidopsis thaliana protein match is: germin-like protein 2 (TAIR:AT5G39190.1)
AT2G18590	*2,371*		Major facilitator superfamily protein; FUNCTIONS IN: carbohydrate transmembrane transporter activity, sugar:hydrogen symporter activity; INVOLVED IN: transmembrane transport; LOCATED IN: endomembrane system, membrane; EXPRESSED IN: 12 plant structures; EXPRESSED DURING: 4 anthesis, F mature embryo stage, petal differentiation and expansion stage, E expanded cotyledon stage, D bilateral stage; CONTAINS InterPro DOMAIN/s: Major facilitator superfamily (InterPro:IPR020846), Major facilitator superfamily MFS-1 (InterPro:IPR011701), Major facilitator superfamily, general substrate transporter (InterPro:IPR016196); BEST Arabidopsis thaliana protein match is: Major facilitator superfamily protein (TAIR:AT4G36790.1)

AT4G35480	*2,37*	RING-H2 FINGER A3B (RHA3B)	Encodes a putative RING-H2 finger protein RHA3b.
AT3G21720	*2,369*	ISOCITRATE LYASE (ICL)	Encodes a glyoxylate cycle enzyme isocitrate lyase (ICL).
AT4G23260	*2,369*	CYSTEINE-RICH RLK (RECEPTOR-LIKE PROTEIN KINASE) 18 (CRK18)	Encodes a cysteine-rich receptor-like protein kinase.
AT2G39030	*2,367*	N-ACETYLTRANSFERASE ACTIVITY 1 (NATA1)	Encodes a protein that acts as an ornithine N-delta-acetyltransferase, leading to the formation of N-delta-actetylornithine. This compound is likely used in plant defense and levels of it are increased in Arabidopsis plants in response to MeJA and ABA. The mRNA is cell-to-cell mobile.
AT3G05260	*2,364*		NAD(P)-binding Rossmann-fold superfamily protein; FUNCTIONS IN: oxidoreductase activity, binding, catalytic activity; INVOLVED IN: oxidation reduction, metabolic process; LOCATED IN: cellular_component unknown; EXPRESSED IN: seed; CONTAINS InterPro DOMAIN/s: Short-chain dehydrogenase/reductase, conserved site (InterPro:IPR020904), NAD(P)-binding domain (InterPro:IPR016040), Glucose/ribitol dehydrogenase (InterPro:IPR002347), Short-chain dehydrogenase/reductase SDR (InterPro:IPR002198); BEST Arabidopsis thaliana protein match is: NAD(P)-binding Rossmann-fold superfamily protein (TAIR:AT1G54870.1)
AT5G61090	*2,363*		Polynucleotidyl transferase, ribonuclease H-like superfamily protein; FUNCTIONS IN: nucleic acid binding; INVOLVED IN: biological_process unknown; LOCATED IN: chloroplast; EXPRESSED IN: 19 plant structures; EXPRESSED DURING: 11 growth stages; CONTAINS InterPro DOMAIN/s: Polynucleotidyl transferase, ribonuclease H fold (InterPro:IPR012337); BEST Arabidopsis thaliana protein match is: unknown protein (TAIR:AT5G61120.1)
AT1G24070	*2,361*	CELLULOSE SYNTHASE-LIKE A10 (CSLA10)	encodes a gene similar to cellulose synthase
AT2G26380	*2,36*		Leucine-rich repeat (LRR) family protein; INVOLVED IN: signal transduction, defense response; LOCATED IN: endomembrane system; EXPRESSED IN: hypocotyl, root; CONTAINS InterPro DOMAIN/s: Leucine-rich repeat-containing N-terminal domain, type 2 (InterPro:IPR013210), Leucine-rich repeat (InterPro:IPR001611); BEST Arabidopsis thaliana protein match is: Leucine-rich repeat (LRR) family protein (TAIR:AT1G33590.1)
AT2G32770	*2,36*	PURPLE ACID PHOSPHATASE 13 (PAP13)	purple acid phosphatase 13 (PAP13); FUNCTIONS IN: acid phosphatase activity; INVOLVED IN: biological_process unknown; LOCATED IN: endomembrane system; EXPRESSED IN: 6 plant structures; EXPRESSED DURING: 4 anthesis, C globular stage; CONTAINS InterPro DOMAIN/s: Purple acid phosphatase, N-terminal (InterPro:IPR015914), Metallophosphoesterase (InterPro:IPR004843), Purple acid phosphatase-like, N-terminal (InterPro:IPR008963); BEST Arabidopsis thaliana protein match is: purple acid phosphatase 15 (TAIR:AT3G07130.1)
AT1G34460	*2,359*	CYCLIN B1;5 (CYCB1;5)	B1 type cyclin
AT2G28500	*2,358*	LOB DOMAIN-CONTAINING PROTEIN 11 (LBD11)	LOB domain-containing protein 11 (LBD11); INVOLVED IN: biological_process unknown; LOCATED IN: chloroplast; CONTAINS InterPro DOMAIN/s: Lateral organ boundaries, LOB (InterPro:IPR004883); BEST Arabidopsis thaliana protein match is: LOB domain-containing protein 1 (TAIR:AT1G07900.1)
AT1G30040	*2,354*	GIBBERELLIN 2-OXIDASE (GA2OX2)	Encodes a gibberellin 2-oxidase that acts on C-19 gibberellins. AtGA2OX2 expression is responsive to cytokinin and KNOX activities.
AT1G70700	*2,336*	(TIFY7)	JAZ9 is a protein presumed to be involved in jasmonate signaling. JAZ9 transcript levels rise in response to a jasmonate stimulus. JAZ9 can interact with the COI1 F-box subunit of an SCF E3 ubiquitin ligase in a yeast-two-hybrid assay only in the presence of jasmonate-isoleucine (JA-ILE) or coronatine. The Jas domain appears to be important for JAZ9-COI1 interactions in the presence of coronatine. Two positive residues (R205 and R206) in the Jas domain shown to be important for coronatine -dependent COI1 binding are not required for binding AtMYC2. The mRNA is cell-to-cell mobile.

AT4G31800	*2,336*	WRKY DNA-BINDING PROTEIN 18 (WRKY18)	Pathogen-induced transcription factor. Binds W-box sequences in vitro. Forms protein complexes with itself and with WRKY40 and WRKY60. Constitutive expression of WRKY18 enhanced resistance to P. syringae, but its coexpression with WRKY40 or WRKY60 made plants more susceptible to both P. syringae and B. cinerea. WRKY18, WRKY40, and WRKY60 have partially redundant roles in response to the hemibiotrophic bacterial pathogen Pseudomonas syringae and the necrotrophic fungal pathogen Botrytis cinerea, with WRKY18 playing a more important role than the other two. The mRNA is cell-to-cell mobile.
AT1G51090	*2,333*	(ATHMAD1)	Heavy metal associated domain containing protein involved in plant immunity.Mutants show an increase in root length under NO stress and reduction in root length under H2O2 stress conditions. Mutants show increases in defense responses to pathogens including hypersensitive lesions, increased resistance and induction of SAR genes.
AT4G15280	*2,332*	UDP-GLUCOSYL TRANSFERASE 71B5 (UGT71B5)	UDP-glucosyl transferase 71B5 (UGT71B5); FUNCTIONS IN: quercetin 3-O-glucosyltransferase activity, UDP-glycosyltransferase activity, transferase activity, transferring glycosyl groups; INVOLVED IN: metabolic process; LOCATED IN: cellular_component unknown; CONTAINS InterPro DOMAIN/s: UDP-glucuronosyl/UDP-glucosyltransferase (InterPro:IPR002213); BEST Arabidopsis thaliana protein match is: UDP-Glycosyltransferase superfamily protein (TAIR:AT4G15260.1)
AT4G36700	*2,33*		RmlC-like cupins superfamily protein; FUNCTIONS IN: nutrient reservoir activity; INVOLVED IN: biological_process unknown; LOCATED IN: endomembrane system; EXPRESSED IN: seed; EXPRESSED DURING: seedling growth; CONTAINS InterPro DOMAIN/s: Cupin, RmlC-type (InterPro:IPR011051), Cupin 1 (InterPro:IPR006045), RmlC-like jelly roll fold (InterPro:IPR014710); BEST Arabidopsis thaliana protein match is: RmlC-like cupins superfamily protein (TAIR:AT2G18540.1)
AT1G71380	*2,324*	CELLULASE 3 (CEL3)	cellulase 3 (CEL3); FUNCTIONS IN: hydrolase activity, hydrolyzing O-glycosyl compounds, catalytic activity; INVOLVED IN: carbohydrate metabolic process; LOCATED IN: cell wall, plasma membrane, plant-type cell wall; EXPRESSED IN: 7 plant structures; EXPRESSED DURING: L mature pollen stage, M germinated pollen stage, 4 anthesis, petal differentiation and expansion stage; CONTAINS InterPro DOMAIN/s: Six-hairpin glycosidase (InterPro:IPR012341), Six-hairpin glycosidase-like (InterPro:IPR008928), Glycoside hydrolase, family 9, active site (InterPro:IPR018221), Glycoside hydrolase, family 9 (InterPro:IPR001701); BEST Arabidopsis thaliana protein match is: cellulase 5 (TAIR:AT1G22880.1)
AT3G56350	*2,324*		Iron/manganese superoxide dismutase family protein; FUNCTIONS IN: superoxide dismutase activity, metal ion binding; INVOLVED IN: oxidation reduction, superoxide metabolic process, removal of superoxide radicals; LOCATED IN: mitochondrion, endomembrane system; EXPRESSED IN: male gametophyte, seed; EXPRESSED DURING: seedling growth; CONTAINS InterPro DOMAIN/s: Manganese/iron superoxide dismutase, N-terminal (InterPro:IPR019831), Manganese/iron superoxide dismutase (InterPro:IPR001189), Manganese/iron superoxide dismutase, C-terminal (InterPro:IPR019832), Manganese/iron superoxide dismutase, binding site (InterPro:IPR019833); BEST Arabidopsis thaliana protein match is: manganese superoxide dismutase 1 (TAIR:AT3G10920.1)
AT5G59570	*2,323*	BROTHER OF LUX ARRHYTHMO (BOA)	Encodes BOA (BROTHER OF LUX ARRHYTHMO), a component of the circadian clock. The mRNA is cell-to-cell mobile.
AT4G22620	*2,321*	SMALL AUXIN UPREGULATED RNA 34 (SAUR34)	SAUR-like auxin-responsive protein family ; CONTAINS InterPro DOMAIN/s: Auxin responsive SAUR protein (InterPro:IPR003676); BEST Arabidopsis thaliana protein match is: SAUR-like auxin-responsive protein family (TAIR:AT4G12410.1)
AT3G51820	*2,32*	(G4)	Encodes a protein with chlorophyll synthase activity. This enzyme has been shown to perform the esterification of chlorophyllide (a and b), the last step of chlorophyll biosynthesis. Although it can use either geranylgeranyl pyrophosphate (GGPP) or phytyl pyrophosphate (PhyPP) as substrates, the esterification reaction was faster with GGPP than with PhyPP.
AT1G54860	*2,319*		Glycoprotein membrane precursor GPI-anchored; BEST Arabidopsis thaliana protein match is: Glycoprotein membrane precursor GPI-anchored (TAIR:AT3G06035.1)
AT3G49110	*2,318*	PEROXIDASE CA (PRXCA)	Class III peroxidase Perx33. Expressed in roots. Located in the cell wall. Involved in cell elongation. Expression activated by light. May play a role in generating H2O2 during defense response. The mRNA is cell-to-cell mobile.
AT5G20230	*2,316*	BLUE-COPPER-BINDING PROTEIN (BCB)	Encodes a Al-stress-induced gene. Along with TCF, it promotes lignin biosynthesis in response to cold stress. The mRNA is cell-to-cell mobile.

AT4G21760	*2,313*	BETA-GLUCOSIDASE 47 (BGLU47)	beta-glucosidase 47 (BGLU47); FUNCTIONS IN: cation binding, hydrolase activity, hydrolyzing O-glycosyl compounds, catalytic activity; INVOLVED IN: carbohydrate metabolic process; LOCATED IN: endomembrane system; EXPRESSED IN: 19 plant structures; EXPRESSED DURING: 13 growth stages; CONTAINS InterPro DOMAIN/s: Glycoside hydrolase, family 1 (InterPro:IPR001360), Glycoside hydrolase, family 1, active site (InterPro:IPR018120), Glycoside hydrolase, catalytic core (InterPro:IPR017853), Glycoside hydrolase, subgroup, catalytic core (InterPro:IPR013781); BEST Arabidopsis thaliana protein match is: beta-glucosidase 45 (TAIR:AT1G61810.1)
AT4G22880	*2,313*	LEUCOANTHOCYANIDIN DIOXYGENASE (LDOX)	encodes leucoanthocyanidin dioxygenase, which is involved in proanthocyanin biosynthesis. Mutant analysis suggests that this gene is also involved in vacuole formation.
AT4G22050	*2,312*		Eukaryotic aspartyl protease family protein; FUNCTIONS IN: aspartic-type endopeptidase activity; INVOLVED IN: proteolysis; LOCATED IN: endomembrane system; EXPRESSED IN: 6 plant structures; EXPRESSED DURING: LP.04 four leaves visible, 4 anthesis, C globular stage, petal differentiation and expansion stage; CONTAINS InterPro DOMAIN/s: Peptidase aspartic (InterPro:IPR021109), Peptidase aspartic, catalytic (InterPro:IPR009007), Peptidase A1 (InterPro:IPR001461), Peptidase aspartic, active site (InterPro:IPR001969); BEST Arabidopsis thaliana protein match is: Eukaryotic aspartyl protease family protein (TAIR:AT1G69100.1)
AT1G05100	*2,298*	MITOGEN-ACTIVATED PROTEIN KINASE KINASE KINASE 18 (MAPKKK18)	member of MEKK subfamily
AT5G49740	*2,296*	FERRIC REDUCTION OXIDASE 7 (FRO7)	Encodes a chloroplast ferric chelate reductase. Shows differential splicing and has three different mRNA products. Expressed in the shoot, flower and cotyledon.
AT1G64160	*2,294*	DIRIGENT PROTEIN 5 (DIR5)	Encodes a dirigent protein involved in the synthesis of (-)pinoresinol. Dirigent proteins impart stereoselectivity on the phenoxy radical coupling reaction yielding optically active lignans from two molecules of coniferyl alcohol.
AT4G24120	*2,293*	YELLOW STRIPE LIKE 1 (YSL1)	Member of a small family of oligopeptide transporters similar to the yellow stripe locus of maize (ZmYS1).
AT5G39180	*2,293*		RmlC-like cupins superfamily protein; FUNCTIONS IN: manganese ion binding, nutrient reservoir activity; INVOLVED IN: biological_process unknown; LOCATED IN: endomembrane system, apoplast; CONTAINS InterPro DOMAIN/s: Cupin, RmlC-type (InterPro:IPR011051), Cupin 1 (InterPro:IPR006045), RmlC-like jelly roll fold (InterPro:IPR014710), Germin (InterPro:IPR001929), Germin, manganese binding site (InterPro:IPR019780); BEST Arabidopsis thaliana protein match is: RmlC-like cupins superfamily protein (TAIR:AT5G39150.1)
AT3G25770	*2,292*	ALLENE OXIDE CYCLASE 2 (AOC2)	Encodes allene oxide cyclase. One of four genes in Arabidopsis that encode this enzyme, which catalyzes an essential step in jasmonic acid biosynthesis. Gene expression is induced during senescence, a process that involves jasmonic acid signalling pathway. Note: Nomenclature for Arabidopsis allene oxide cyclase 2 (AOC2, AT3G25770) gene is based on Stenzel et al. 2003 Plant Molecular Biology 51:895-911. AOC2 (AT3G25770) is also referred to as AOC3 in He et al. 2002 Plant Physiology, 128:876-884. The mRNA is cell-to-cell mobile.
AT1G27480	*2,291*		alpha/beta-Hydrolases superfamily protein; FUNCTIONS IN: phosphatidylcholine-sterol O-acyltransferase activity; INVOLVED IN: lipid metabolic process; LOCATED IN: plasma membrane, vacuole; EXPRESSED IN: 21 plant structures; EXPRESSED DURING: 13 growth stages; CONTAINS InterPro DOMAIN/s: Lecithin:cholesterol acyltransferase (InterPro:IPR003386)
AT4G22390	*2,284*		F-box associated ubiquitination effector family protein; CONTAINS InterPro DOMAIN/s: F-box associated interaction domain (InterPro:IPR017451); BEST Arabidopsis thaliana protein match is: F-box and associated interaction domains-containing protein (TAIR:AT4G12560.2)
AT5G50160	*2,277*	FERRIC REDUCTION OXIDASE 8 (FRO8)	Encodes a ferric chelate reductase that is expressed in shoots and flowers.

AT2G32860	*2,276*	BETA GLUCOSIDASE 33 (BGLU33)	beta glucosidase 33 (BGLU33); FUNCTIONS IN: cation binding, hydrolase activity, hydrolyzing O-glycosyl compounds, catalytic activity; INVOLVED IN: carbohydrate metabolic process; LOCATED IN: endomembrane system; EXPRESSED IN: 20 plant structures; EXPRESSED DURING: 10 growth stages; CONTAINS InterPro DOMAIN/s: Glycoside hydrolase, family 1 (InterPro:IPR001360), Glycoside hydrolase, family 1, active site (InterPro:IPR018120), Glycoside hydrolase, catalytic core (InterPro:IPR017853), Glycoside hydrolase, subgroup, catalytic core (InterPro:IPR013781); BEST Arabidopsis thaliana protein match is: Glycosyl hydrolase superfamily protein (TAIR:AT3G60140.1)
AT4G29930	*2,276*		basic helix-loop-helix (bHLH) DNA-binding superfamily protein; FUNCTIONS IN: DNA binding, sequence-specific DNA binding transcription factor activity; INVOLVED IN: regulation of transcription; LOCATED IN: nucleus; CONTAINS InterPro DOMAIN/s: Helix-loop-helix DNA-binding domain (InterPro:IPR001092), Helix-loop-helix DNA-binding (InterPro:IPR011598); BEST Arabidopsis thaliana protein match is: basic helix-loop-helix (bHLH) DNA-binding superfamily protein (TAIR:AT5G57150.1)
AT3G51895	*2,264*	SULFATE TRANSPORTER 3;1 (SULTR3;1)	Encodes a chloroplast-localized sulfate transporter.
AT5G66780	*2,262*		unknown protein
AT2G33160	*2,26*	NIMNA (SANSKRIT FOR "'SUNKEN" OR "LOW") (NMA)	Gene structure annotation for AT2G33160.1 is inaccurate in TAIR10, see PMID:23709666 and Comments field on the locus page for updated annotation.
AT1G54280	*2,257*	AMINOPHOSPHOLIPID ATPASE 6 (ALA6)	Encodes a member of the P4 subfamily of P-type ATPases expressed in the pollen plasma membrane. Double mutants with ALA7 display pollen and pollen tube defects.
AT5G59100	*2,256*		Subtilisin-like serine endopeptidase family protein; FUNCTIONS IN: identical protein binding, serine-type endopeptidase activity; INVOLVED IN: proteolysis, negative regulation of catalytic activity; LOCATED IN: endomembrane system; CONTAINS InterPro DOMAIN/s: Protease-associated PA (InterPro:IPR003137), Peptidase S8/S53, subtilisin/kexin/sedolisin (InterPro:IPR000209), Peptidase S8/S53, subtilisin, active site (InterPro:IPR022398), Peptidase S8, subtilisin-related (InterPro:IPR015500), Proteinase inhibitor I9, subtilisin propeptide (InterPro:IPR010259); BEST Arabidopsis thaliana protein match is: Subtilase family protein (TAIR:AT3G46850.1)
AT1G17710	*2,249*	PHOSPHOETHANOLAMINE/PHOSPHOCHOLINE PHOSPHATASE 1 (PEPC1)	Encodes a phosphoethanolamine/phosphocholine phosphatase. It is likely to be involved in the liberation of inorganic phosphate from intracellular sources. Expression is upregulated in the shoot of cax1/cax3 mutant.
AT2G47770	*2,244*	TSPO(OUTER MEMBRANE TRYPTOPHAN-RICH SENSORY PROTEIN)-RELATED (TSPO)	Encodes a membrane-bound protein designated AtTSPO (Arabidopsis thaliana TSPO-related). AtTSPO is related to the bacterial outer membrane tryptophan-rich sensory protein (TspO) and the mammalian mitochondrial 18 kDa Translocator Protein (18 kDa TSPO), members of the TspO/MBR domain-containing membrane proteins. Mainly detected in dry seeds, but can be induced in vegetative tissues by osmotic or salt stress or abscisic acid treatment. Located in endoplasmic reticulum and the Golgi stacks. It is degraded through the autophagy pathway.
AT2G43530	*2,227*		Encodes a defensin-like (DEFL) family protein. The mRNA is cell-to-cell mobile.
AT2G07708	*2,222*		unknown protein; BEST Arabidopsis thaliana protein match is: unknown protein (TAIR:ATMG00500.1)
AT2G42530	*2,22*	COLD REGULATED 15B (COR15B)	Encodes COR15B, a protein that protects chloroplast membranes during freezing.
AT4G08870	*2,22*	ARGININE AMIDOHYDROLASE 2 (ARGAH2)	Encodes one of the two arginase in the genome. Gene expression is enhanced by methyl jasmonate treatment. It is involved in the defense response to B.cinerea.
AT5G52760	*2,218*		Copper transport protein family; BEST Arabidopsis thaliana protein match is: Heavy metal transport/detoxification superfamily protein (TAIR:AT5G52750.1)
AT2G27880	*2,217*	ARGONAUTE 5 (AGO5)	AGO5.Required for antiviral RNA silencing.Confers resistance to Potato virus X.
AT1G10980	*2,216*		Lung seven transmembrane receptor family protein; INVOLVED IN: biological_process unknown; LOCATED IN: vacuole; EXPRESSED IN: 8 plant structures; EXPRESSED DURING: 6 growth stages; CONTAINS InterPro DOMAIN/s: Transmembrane receptor, eukaryota (InterPro:IPR009637); BEST Arabidopsis thaliana protein match is: Lung seven transmembrane receptor family protein (TAIR:AT1G61670.1)

AT5G57690	*2,216*	DIACYLGLYCEROL KINASE 4 (DGK4)	diacylglycerol kinase 4 (DGK4); FUNCTIONS IN: diacylglycerol kinase activity; INVOLVED IN: activation of protein kinase C activity by G-protein coupled receptor protein signaling pathway; LOCATED IN: chloroplast; EXPRESSED IN: 7 plant structures; EXPRESSED DURING: L mature pollen stage, M germinated pollen stage, 4 anthesis, petal differentiation and expansion stage; CONTAINS InterPro DOMAIN/s: Diacylglycerol kinase, catalytic domain (InterPro:IPR001206), Diacylglycerol kinase, accessory domain (InterPro:IPR000756), Diacylglycerol kinase, plant (InterPro:IPR016961); BEST Arabidopsis thaliana protein match is: diacylglycerol kinase 3 (TAIR:AT2G18730.1)
AT2G43510	*2,213*	TRYPSIN INHIBITOR PROTEIN 1 (TI1)	Member of the defensin-like (DEFL) family. Encodes putative trypsin inhibitor protein which may function in defense against herbivory.
AT1G17380	*2,209*	JASMONATE-ZIM-DOMAIN PROTEIN 5 (JAZ5)	jasmonate-zim-domain protein 5 (JAZ5); CONTAINS InterPro DOMAIN/s: Tify (InterPro:IPR010399), CCT domain-like (InterPro:IPR018467); BEST Arabidopsis thaliana protein match is: jasmonate-zim-domain protein 6 (TAIR:AT1G72450.1)
AT5G61520	*2,209*		Major facilitator superfamily protein; FUNCTIONS IN: carbohydrate transmembrane transporter activity, sugar:hydrogen symporter activity; INVOLVED IN: transport, transmembrane transport; LOCATED IN: integral to membrane, membrane; EXPRESSED IN: 15 plant structures; EXPRESSED DURING: 8 growth stages; CONTAINS InterPro DOMAIN/s: Sugar transporter, conserved site (InterPro:IPR005829), Major facilitator superfamily (InterPro:IPR020846), General substrate transporter (InterPro:IPR005828), Sugar/inositol transporter (InterPro:IPR003663), Major facilitator superfamily, general substrate transporter (InterPro:IPR016196); BEST Arabidopsis thaliana protein match is: Major facilitator superfamily protein (TAIR:AT1G34580.1)
AT4G35180	*2,202*	LYS/HIS TRANSPORTER 7 (LHT7)	LYS/HIS transporter 7 (LHT7); CONTAINS InterPro DOMAIN/s: Amino acid transporter, transmembrane (InterPro:IPR013057); BEST Arabidopsis thaliana protein match is: Transmembrane amino acid transporter family protein (TAIR:AT1G47670.1)
AT4G24830	*2,201*		arginosuccinate synthase family; FUNCTIONS IN: argininosuccinate synthase activity, ATP binding; INVOLVED IN: arginine biosynthetic process; LOCATED IN: chloroplast stroma, chloroplast; EXPRESSED IN: 25 plant structures; EXPRESSED DURING: 15 growth stages; CONTAINS InterPro DOMAIN/s: Rossmann-like alpha/beta/alpha sandwich fold (InterPro:IPR014729), Argininosuccinate synthase, conserved site (InterPro:IPR018223), Argininosuccinate synthase (InterPro:IPR001518)
AT3G44860	*2,2*	FARNESOIC ACID CARBOXYL-O-METHYLTRANSFERASE (FAMT)	Encodes a farnesoic acid carboxyl-O-methyltransferase. The mRNA is cell-to-cell mobile.
AT4G24960	*2,198*	HVA22 HOMOLOGUE D (HVA22D)	Homologous to a eukaryote specific ABA- and stress-inducible gene first isolated from barley. Groups in one subfamily with ATHVA22E. Along with other members of the ATHVA22 family, it may be involved in regulation of autophagy during development. The mRNA is cell-to-cell mobile.
AT5G59170	*2,19*		Proline-rich extensin-like family protein; LOCATED IN: endomembrane system
AT4G12100	*2,183*		Cullin family protein; FUNCTIONS IN: ubiquitin protein ligase binding; INVOLVED IN: ubiquitin-dependent protein catabolic process; LOCATED IN: cullin-RING ubiquitin ligase complex; CONTAINS InterPro DOMAIN/s: Cullin, N-terminal (InterPro:IPR001373), Cullin repeat-like-containing domain (InterPro:IPR016159); BEST Arabidopsis thaliana protein match is: cullin4 (TAIR:AT5G46210.1)
AT2G33380	*2,182*	RESPONSIVE TO DESICCATION 20 (RD20)	Encodes a calcium binding protein whose mRNA is induced upon treatment with NaCl, ABA and in response to desiccation. mRNA expression under drought conditions is apparent particularly in leaves and flowers. Isoform of caleosin with a role as a peroxygenase involved in oxylipin metabolism during biotic and abiotic stress. Involved in the production of 2-hydroxy-octadecatrienoic acid. The peroxygenase has a narrow substrate specificity thus acting as a fatty acid hydroperoxide reductase in vivo.
AT4G14090	*2,179*		The At4g14090 encodes a anthocyanidin 5-O-glucosyltransferase specifically glucosylating the 5-position of the flavonoid A-ring.
AT5G46730	*2,175*		glycine-rich protein; FUNCTIONS IN: molecular_function unknown; INVOLVED IN: biological_process unknown; LOCATED IN: endomembrane system; EXPRESSED IN: 21 plant structures; EXPRESSED DURING: 13 growth stages

AT1G61550	2,174		S-locus lectin protein kinase family protein; FUNCTIONS IN: in 6 functions; INVOLVED IN: protein amino acid phosphorylation, recognition of pollen; LOCATED IN: endomembrane system; EXPRESSED IN: sperm cell, root; CONTAINS InterPro DOMAIN/s: Curculin-like (mannose-binding) lectin (InterPro:IPR001480), PAN-2 domain (InterPro:IPR013227), Apple-like (InterPro:IPR003609), S-locus receptor kinase, C-terminal (InterPro:IPR021820), EGF-like, type 3 (InterPro:IPR000742), Serine-threonine/tyrosine-protein kinase (InterPro:IPR001245), Protein kinase-like domain (InterPro:IPR011009), Serine/threonine-protein kinase, active site (InterPro:IPR008271), Protein kinase, catalytic domain (InterPro:IPR000719), S-locus glycoprotein (InterPro:IPR000858); BEST Arabidopsis thaliana protein match is: S-locus lectin protein kinase family protein (TAIR:AT1G61490.1)
AT3G22840	2,172	EARLY LIGHT-INDUCABLE PROTEIN (ELIP1)	Encodes an early light-inducible protein.
AT4G00050	2,166	UNFERTILIZED EMBRYO SAC 10 (UNE10)	unfertilized embryo sac 10 (UNE10); FUNCTIONS IN: DNA binding, sequence-specific DNA binding transcription factor activity; INVOLVED IN: double fertilization forming a zygote and endosperm, regulation of transcription; LOCATED IN: nucleus; EXPRESSED IN: 20 plant structures; EXPRESSED DURING: 13 growth stages; CONTAINS InterPro DOMAIN/s: Helix-loop-helix DNA-binding domain (InterPro:IPR001092), Helix-loop-helix DNA-binding (InterPro:IPR011598); BEST Arabidopsis thaliana protein match is: phytochrome-interacting factor7 (TAIR:AT5G61270.1)
AT3G22120	2,165	CELL WALL-PLASMA MEMBRANE LINKER PROTEIN (CWLP)	cell wall-plasma membrane linker protein homolog (CWLP)
AT2G36800	2,162	DON-GLUCOSYLTRANSFERASE 1 (DOGT1)	Encodes a DON-Glucosyltransferase. The UGT73C5 glucosylates both brassinolide and castasterone in the 23-O position. The enzyme is presumably involved in the homeostasis of those steroid hormones hence regulating BR activity. Transgenic plants overexpressing UGT73C5 show a typical BR-deficient phenotype.
AT5G22300	2,16	NITRILASE 4 (NIT4)	encodes a nitrilase isomer. The purified enzyme shows a strong substrate specificity for beta-cyano-L-alanine, a intermediate product of the cyanide detoxification pathway. The mRNA is cell-to-cell mobile.
AT1G50780	2,158		2Fe-2S ferredoxin-like superfamily protein; FUNCTIONS IN: electron carrier activity, iron-sulfur cluster binding; INVOLVED IN: biological_process unknown; LOCATED IN: endomembrane system; CONTAINS InterPro DOMAIN/s: Ferredoxin (InterPro:IPR001041), 2Fe-2S ferredoxin, iron-sulphur binding site (InterPro:IPR006058), Beta-grasp fold, ferredoxin-type (InterPro:IPR012675); BEST Arabidopsis thaliana protein match is: MADS-box transcription factor family protein (TAIR:AT1G48150.1)
AT2G36660	2,152	POLY(A) BINDING PROTEIN 7 (PAB7)	polyadenylate-binding protein, putative / PABP, putative. Member of the class III family of PABP proteins.
AT1G19670	2,151	CHLOROPHYLLASE 1 (CLH1)	Chlorophyllase is the first enzyme involved in chlorophyll degradation. It catalyzes the hydrolysis of the ester bond to yield chlorophyllide and phytol. AtCLH1 lacks a typical signal sequence for the chloroplast. Its expression is induced rapidly by methyljasmonate, a known promoter of senescence and chlorophyll degradation.
AT1G68250	2,15		unknown protein
AT1G04660	2,149		glycine-rich protein; FUNCTIONS IN: molecular_function unknown; INVOLVED IN: biological_process unknown; LOCATED IN: endomembrane system; EXPRESSED IN: 8 plant structures; EXPRESSED DURING: 4 anthesis, F mature embryo stage, petal differentiation and expansion stage, E expanded cotyledon stage, D bilateral stage
AT1G70640	2,146		octicosapeptide/Phox/Bem1p (PB1) domain-containing protein; CONTAINS InterPro DOMAIN/s: Octicosapeptide/Phox/Bem1p (InterPro:IPR000270); BEST Arabidopsis thaliana protein match is: Octicosapeptide/Phox/Bem1p family protein (TAIR:AT3G26510.4)
AT5G22530	2,146		unknown protein; BEST Arabidopsis thaliana protein match is: unknown protein (TAIR:AT5G22520.1)
AT5G13330	2,144	RELATED TO AP2 6L (Rap2.6L)	encodes a member of the ERF (ethylene response factor) subfamily B-4 of ERF/AP2 transcription factor family. The protein contains one AP2 domain. There are 7 members in this subfamily.
AT5G50790	2,144	(SWEET10)	Encodes a member of the SWEET sucrose efflux transporter family proteins.

AT3G54820	*2,143*	PLASMA MEMBRANE INTRINSIC PROTEIN 2;5 (PIP2;5)	plasma membrane intrinsic protein 2;5 (PIP2;5); FUNCTIONS IN: water channel activity; INVOLVED IN: transport, transmembrane transport; LOCATED IN: integral to membrane, membrane; EXPRESSED IN: 17 plant structures; EXPRESSED DURING: 10 growth stages; CONTAINS InterPro DOMAIN/s: Major intrinsic protein, conserved site (InterPro:IPR022357), Aquaporin (InterPro:IPR012269), Major intrinsic protein (InterPro:IPR000425); BEST Arabidopsis thaliana protein match is: plasma membrane intrinsic protein 2 (TAIR:AT2G37170.1)
AT5G22540	*2,141*		Plant protein of unknown function (DUF247); CONTAINS InterPro DOMAIN/s: Protein of unknown function DUF247, plant (InterPro:IPR004158); BEST Arabidopsis thaliana protein match is: Plant protein of unknown function (DUF247) (TAIR:AT5G22550.2)
AT2G42610	*2,14*	LIGHT SENSITIVE HYPOCOTYLS 10 (LSH10)	LIGHT SENSITIVE HYPOCOTYLS 10 (LSH10); CONTAINS InterPro DOMAIN/s: Protein of unknown function DUF640 (InterPro:IPR006936); BEST Arabidopsis thaliana protein match is: Protein of unknown function (DUF640) (TAIR:AT1G07090.1)
AT5G67310	*2,137*	CYTOCHROME P450, FAMILY 81, SUBFAMILY G, POLYPEPTIDE 1 (CYP81G1)	member of CYP81G
AT5G07430	*2,134*		Pectin lyase-like superfamily protein; FUNCTIONS IN: pectinesterase activity; INVOLVED IN: cell wall modification; LOCATED IN: endomembrane system, cell wall, plant-type cell wall; EXPRESSED IN: 9 plant structures; EXPRESSED DURING: L mature pollen stage, M germinated pollen stage, 4 anthesis, C globular stage, petal differentiation and expansion stage; CONTAINS InterPro DOMAIN/s: Pectin lyase fold/virulence factor (InterPro:IPR011050), Pectinesterase, catalytic (InterPro:IPR000070), Pectin lyase fold (InterPro:IPR012334); BEST Arabidopsis thaliana protein match is: Pectin lyase-like superfamily protein (TAIR:AT5G07420.1)
AT5G46330	*2,132*	FLAGELLIN-SENSITIVE 2 (FLS2)	Encodes a leucine-rich repeat serine/threonine protein kinase that is expressed ubiquitously. FLS2 is involved in MAP kinase signalling relay involved in innate immunity. Essential in the perception of flagellin, a potent elicitor of the defense response. FLS2 is directed for degradation by the bacterial ubiquitin ligase AvrPtoB. The mRNA is cell-to-cell mobile.
AT2G12190	*2,129*		Cytochrome P450 superfamily protein; FUNCTIONS IN: electron carrier activity, monooxygenase activity, iron ion binding, oxygen binding, heme binding; INVOLVED IN: oxidation reduction; EXPRESSED IN: stem, leaf whorl, male gametophyte, flower, stamen; EXPRESSED DURING: 4 anthesis; CONTAINS InterPro DOMAIN/s: Cytochrome P450 (InterPro:IPR001128), Cytochrome P450, conserved site (InterPro:IPR017972), Cytochrome P450, E-class, group I (InterPro:IPR002401); BEST Arabidopsis thaliana protein match is: cytochrome P450, family 87, subfamily A, polypeptide 6 (TAIR:AT1G64940.1)
AT3G18610	*2,128*	NUCLEOLIN LIKE 2 (NUC-L2)	Encodes ATNUC-L2 (NUCLEOLIN LIKE 2).
AT4G21680	*2,125*	NRT1/ PTR FAMILY 7.2 (NPF7.2)	Encodes a nitrate transporter (NRT1.8). Functions in nitrate removal from the xylem sap. Mediates cadmium tolerance.
AT5G45530	*2,123*		Protein of unknown function (DUF594); FUNCTIONS IN: molecular_function unknown; INVOLVED IN: biological_process unknown; LOCATED IN: endomembrane system; EXPRESSED IN: 13 plant structures; EXPRESSED DURING: C globular stage, F mature embryo stage, petal differentiation and expansion stage; CONTAINS InterPro DOMAIN/s: Protein of unknown function DUF594 (InterPro:IPR007658); BEST Arabidopsis thaliana protein match is: Protein of unknown function (DUF594) (TAIR:AT5G45470.1)
AT3G26180	*2,116*	CYTOCHROME P450, FAMILY 71, SUBFAMILY B, POLYPEPTIDE 20 (CYP71B20)	putative cytochrome P450
AT1G50830	*2,115*		Aminotransferase-like, plant mobile domain family protein; CONTAINS InterPro DOMAIN/s: Aminotransferase-like, plant mobile domain (InterPro:IPR019557); BEST Arabidopsis thaliana protein match is: Aminotransferase-like, plant mobile domain family protein (TAIR:AT5G18510.1)
AT3G05650	*2,113*	RECEPTOR LIKE PROTEIN 32 (RLP32)	receptor like protein 32 (RLP32); FUNCTIONS IN: kinase activity; INVOLVED IN: signal transduction, defense response; LOCATED IN: endomembrane system; EXPRESSED IN: 22 plant structures; EXPRESSED DURING: 13 growth stages; CONTAINS InterPro DOMAIN/s: Leucine-rich repeat-containing N-terminal domain, type 2 (InterPro:IPR013210), Leucine-rich repeat (InterPro:IPR001611); BEST Arabidopsis thaliana protein match is: receptor like protein 35 (TAIR:AT3G11080.1)

AT1G02770	*2,106*		Protein of unknown function (DUF626); CONTAINS InterPro DOMAIN/s: Protein of unknown function DUF626, Arabidopsis thaliana (InterPro:IPR006462); BEST Arabidopsis thaliana protein match is: Protein of unknown function (DUF626) (TAIR:AT1G19060.1)
AT5G04310	*2,104*		Pectin lyase-like superfamily protein; CONTAINS InterPro DOMAIN/s: Pectin lyase fold/virulence factor (InterPro:IPR011050), AmbAllergen (InterPro:IPR018082), Pectate lyase/Amb allergen (InterPro:IPR002022), Pectin lyase fold (InterPro:IPR012334); BEST Arabidopsis thaliana protein match is: Pectin lyase-like superfamily protein (TAIR:AT3G54920.1)
AT4G32800	*2,103*		encodes a member of the DREB subfamily A-4 of ERF/AP2 transcription factor family. The protein contains one AP2 domain. There are 17 members in this subfamily including TINY.
AT4G09820	*2,102*	TRANSPARENT TESTA 8 (TT8)	TT8 is a regulation factor that acts in a concerted action with TT1, PAP1 and TTG1 on the regulation of flavonoid pathways, namely proanthocyanidin and anthocyanin biosynthesis. Affects dihydroflavonol 4-reductase gene expression. It is thought that a ternary complex composed of TT2, TT8 and TTG1 is necessary for correct expression of BAN in seed endothelium. Also important for important for marginal trichome development. It binds the promoter of both AT3G26790 and AT1G28300.TT8 interacts with JAZ proteins to regulate anthocyanin accumulation.
AT1G11230	*2,099*		Protein of unknown function (DUF761); FUNCTIONS IN: molecular_function unknown; INVOLVED IN: biological_process unknown; LOCATED IN: endomembrane system; CONTAINS InterPro DOMAIN/s: Protein of unknown function DUF761, plant (InterPro:IPR008480); BEST Arabidopsis thaliana protein match is: Protein of unknown function (DUF761) (TAIR:AT1G11220.1).
AT1G13370	*2,094*		Histone superfamily protein; FUNCTIONS IN: DNA binding; INVOLVED IN: nucleosome assembly; LOCATED IN: chloroplast, nucleosome; EXPRESSED IN: root, inflorescence, flower, leaf; EXPRESSED DURING: seedling growth; CONTAINS InterPro DOMAIN/s: Histone H3 (InterPro:IPR000164), Histone-fold (InterPro:IPR009072), Histone core (InterPro:IPR007125); BEST Arabidopsis thaliana protein match is: Histone superfamily protein (TAIR:AT4G40030.2)
AT2G20800	*2,091*	NAD(P)H DEHYDROGENASE B4 (NDB4)	NAD(P)H dehydrogenase B4 (NDB4); FUNCTIONS IN: NADH dehydrogenase activity; INVOLVED IN: oxidation reduction; LOCATED IN: extrinsic to mitochondrial inner membrane, mitochondrion, plastid; EXPRESSED IN: 7 plant structures; CONTAINS InterPro DOMAIN/s: Pyridine nucleotide-disulphide oxidoreductase, class-II (InterPro:IPR000103), FAD-dependent pyridine nucleotide-disulphide oxidoreductase (InterPro:IPR013027), Pyridine nucleotide-disulphide oxidoreductase, NAD-binding region (InterPro:IPR001327), EF-HAND 2 (InterPro:IPR018249); BEST Arabidopsis thaliana protein match is: NAD(P)H dehydrogenase B3 (TAIR:AT4G21490.1)
AT5G06760	*2,089*	LATE EMBRYOGENESIS ABUNDANT 4-5 (LEA4-5)	Encodes LEA4-5, a member of the Late Embryogenesis Abundant (LEA) proteins which typically accumulate in response to low water availability conditions imposed during development or by the environment. Most of the diverse set of LEA proteins can be grouped according to properties such as high hydrophilicity and high content of glycine or other small amino acids in what has been termed hydrophilins. LEA4-5 protects enzyme activities from the adverse effects induced by freeze-thaw cycles in vitro.
AT5G45120	*2,089*		Eukaryotic aspartyl protease family protein; FUNCTIONS IN: aspartic-type endopeptidase activity; INVOLVED IN: proteolysis; CONTAINS InterPro DOMAIN/s: Peptidase aspartic (InterPro:IPR021109), Peptidase aspartic, catalytic (InterPro:IPR009007), Peptidase A1 (InterPro:IPR001461), Peptidase aspartic, active site (InterPro:IPR001969); BEST Arabidopsis thaliana protein match is: Eukaryotic aspartyl protease family protein (TAIR:AT4G16563.1)
AT1G48470	*2,086*	GLUTAMINE SYNTHETASE 1;5 (GLN1;5)	Encodes cytosolic glutamine synthase isozyme. Expression of mRNA is not detectable in roots.
AT1G76780	*2,079*		HSP20-like chaperones superfamily protein; CONTAINS InterPro DOMAIN/s: Heat shock protein Hsp20 (InterPro:IPR002068), HSP20-like chaperone (InterPro:IPR008978); BEST Arabidopsis thaliana protein match is: HSP20-like chaperones superfamily protein (TAIR:AT1G76770.1)
AT1G60970	*2,078*		SNARE-like superfamily protein; INVOLVED IN: intracellular protein transport, transport; LOCATED IN: clathrin vesicle coat; EXPRESSED IN: leaf whorl, sperm cell, pedicel; EXPRESSED DURING: 4 anthesis; CONTAINS InterPro DOMAIN/s: Longin-like (InterPro:IPR011012); BEST Arabidopsis thaliana protein match is: SNARE-like superfamily protein (TAIR:AT3G09800.1)

AT2G21450	*2,076*	CHROMATIN REMODELING 34 (CHR34)	chromatin remodeling 34 (CHR34); FUNCTIONS IN: helicase activity, DNA binding, nucleic acid binding, ATP binding; EXPRESSED IN: 8 plant structures; EXPRESSED DURING: 4 anthesis, C globular stage, petal differentiation and expansion stage, E expanded cotyledon stage, D bilateral stage; CONTAINS InterPro DOMAIN/s: DEAD-like helicase, N-terminal (InterPro:IPR014001), DNA/RNA helicase, C-terminal (InterPro:IPR001650), Helicase, superfamily 1/2, ATP-binding domain (InterPro:IPR014021), SNF2-related (InterPro:IPR000330); BEST Arabidopsis thaliana protein match is: SNF2 domain-containing protein / helicase domain-containing protein (TAIR:AT2G16390.1)
AT2G21590	*2,075*	(APL4)	Encodes the large subunit of ADP-glucose pyrophosphorylase, the enzyme which catalyzes the first and limiting step in starch biosynthesis. The large subunit plays a regulatory role whereas the small subunit (ApS) is the catalytic isoform. Four isoforms of the large subunit (ApL1-4) have been described.
AT1G22150	*2,068*	SULFATE TRANSPORTER 1;3 (SULTR1;3)	sulfate transporter Sultr1;3
AT2G05510	*2,065*		Glycine-rich protein family; FUNCTIONS IN: molecular_function unknown; INVOLVED IN: biological_process unknown; LOCATED IN: endomembrane system; CONTAINS InterPro DOMAIN/s: Glycine rich protein (InterPro:IPR010800); BEST Arabidopsis thaliana protein match is: GLYCINE RICH PROTEIN 9 (TAIR:AT2G05440.1)
AT4G25010	*2,065*	(SWEET14)	Encodes a member of the SWEET sucrose efflux transporter family proteins.
AT1G33770	*2,058*		Protein kinase superfamily protein; FUNCTIONS IN: protein serine/threonine kinase activity, protein kinase activity, kinase activity, ATP binding; INVOLVED IN: protein amino acid phosphorylation, N-terminal protein myristoylation; LOCATED IN: cellular_component unknown; EXPRESSED IN: 7 plant structures; EXPRESSED DURING: 6 growth stages; CONTAINS InterPro DOMAIN/s: Protein kinase, ATP binding site (InterPro:IPR017441), Protein kinase, catalytic domain (InterPro:IPR000719), Serine/threonine-protein kinase domain (InterPro:IPR002290), Serine/threonine-protein kinase-like domain (InterPro:IPR017442), Protein kinase-like domain (InterPro:IPR011009), Serine/threonine-protein kinase, active site (InterPro:IPR008271); BEST Arabidopsis thaliana protein match is: Protein kinase superfamily protein (TAIR:AT4G10010.1)
AT5G40000	*2,056*		P-loop containing nucleoside triphosphate hydrolases superfamily protein; FUNCTIONS IN: nucleoside-triphosphatase activity, ATPase activity, nucleotide binding, ATP binding; INVOLVED IN: biological_process unknown; LOCATED IN: endomembrane system; CONTAINS InterPro DOMAIN/s: ATPase, AAA+ type, core (InterPro:IPR003593), ATPase, AAA-type, core (InterPro:IPR003959), ATPase, AAA-type, conserved site (InterPro:IPR003960); BEST Arabidopsis thaliana protein match is: P-loop containing nucleoside triphosphate hydrolases superfamily protein (TAIR:AT3G28600.1)
AT2G26400	*2,054*	ACIREDUCTONE DIOXYGENASE 3 (ARD3)	Encodes a protein predicted to belong to the acireductone dioxygenase (ARD/ARD?)family.
AT1G52000	*2,053*		Mannose-binding lectin superfamily protein; FUNCTIONS IN: molecular_function unknown; INVOLVED IN: response to salt stress; LOCATED IN: cellular_component unknown; EXPRESSED IN: 22 plant structures; EXPRESSED DURING: 14 growth stages; CONTAINS InterPro DOMAIN/s: Mannose-binding lectin (InterPro:IPR001229); BEST Arabidopsis thaliana protein match is: myrosinase-binding protein 1 (TAIR:AT1G52040.1)
AT3G47290	*2,053*	PHOSPHATIDYLINOSITOL-SPECIWC PHOSPHOLIPASE C8 (PLC8)	phosphatidylinositol-speciwc phospholipase C8 (PLC8); FUNCTIONS IN: phospholipase C activity, phosphoinositide phospholipase C activity, phosphoric diester hydrolase activity; INVOLVED IN: signal transduction, intracellular signaling pathway, lipid metabolic process; CONTAINS InterPro DOMAIN/s: Phospholipase C, phosphatidylinositol-specific , X domain (InterPro:IPR000909), PLC-like phosphodiesterase, TIM beta/alpha-barrel domain (InterPro:IPR017946), C2 membrane targeting protein (InterPro:IPR018029), C2 calcium/lipid-binding domain, CaLB (InterPro:IPR008973), Phospholipase C, phosphoinositol-specific (InterPro:IPR001192), C2 calcium-dependent membrane targeting (InterPro:IPR000008), Phospholipase C, phosphatidylinositol-specific, Y domain (InterPro:IPR001711); BEST Arabidopsis thaliana protein match is: phosphatidylinositol-speciwc phospholipase C9 (TAIR:AT3G47220.1)

AT5G59480	*2,051*		Haloacid dehalogenase-like hydrolase (HAD) superfamily protein; FUNCTIONS IN: hydrolase activity; LOCATED IN: cellular_component unknown; EXPRESSED IN: 24 plant structures; EXPRESSED DURING: 15 growth stages; CONTAINS InterPro DOMAIN/s: Pyrimidine 5-nucleotidase (InterPro:IPR010237), HAD-superfamily hydrolase, subfamily IA, variant 3 (InterPro:IPR006402); BEST Arabidopsis thaliana protein match is: Haloacid dehalogenase-like hydrolase (HAD) superfamily protein (TAIR:AT5G02230.2)
AT5G63530	*2,049*	FARNESYLATED PROTEIN 3 (FP3)	Farnesylated protein that binds metals.
AT4G23520	*2,046*		Cysteine proteinases superfamily protein; FUNCTIONS IN: cysteine-type endopeptidase activity, cysteine-type peptidase activity; INVOLVED IN: proteolysis; LOCATED IN: endomembrane system; CONTAINS InterPro DOMAIN/s: Peptidase C1A, papain (InterPro:IPR013128), Proteinase inhibitor I29, cathepsin propeptide (InterPro:IPR013201), Peptidase C1A, papain C-terminal (InterPro:IPR000668), Peptidase, cysteine peptidase active site (InterPro:IPR000169); BEST Arabidopsis thaliana protein match is: Granulin repeat cysteine protease family protein (TAIR:AT5G43060.1)
AT4G27970	*2,042*	SLAC1 HOMOLOGUE 2 (SLAH2)	Encodes a protein with ten predicted transmembrane helices. The SLAH2 protein has similarity to the SLAC1 protein involved in ion homeostasis in guard cells. But, it is not expressed in guard cells and cannot complement a slac1-2 mutant suggesting that it performs a different function. SLAH2:GFP localizes to the plasma membrane.
AT1G32910	*2,04*		HXXXD-type acyl-transferase family protein; FUNCTIONS IN: transferase activity, transferring acyl groups other than amino-acyl groups, transferase activity; INVOLVED IN: biological_process unknown; LOCATED IN: cellular_component unknown; CONTAINS InterPro DOMAIN/s: Transferase (InterPro:IPR003480); BEST Arabidopsis thaliana protein match is: HXXXD-type acyl-transferase family protein (TAIR:AT1G78990.1)
AT4G29600	*2,04*		Cytidine/deoxycytidylate deaminase family protein; FUNCTIONS IN: hydrolase activity, cytidine deaminase activity, catalytic activity, zinc ion binding; INVOLVED IN: cytidine deamination, cytidine metabolic process; LOCATED IN: cellular_component unknown; CONTAINS InterPro DOMAIN/s: Cytidine deaminase, homodimeric (InterPro:IPR006263), CMP/dCMP deaminase, zinc-binding (InterPro:IPR002125), Cytidine deaminase-like (InterPro:IPR016193), Cytidine/deoxycytidylate deaminase, zinc-binding domain (InterPro:IPR013171); BEST Arabidopsis thaliana protein match is: Cytidine/deoxycytidylate deaminase family protein (TAIR:AT4G29620.1)
AT2G43620	*2,038*		Chitinase family protein; FUNCTIONS IN: chitin binding, chitinase activity; INVOLVED IN: response to salt stress; LOCATED IN: apoplast; EXPRESSED IN: 12 plant structures; EXPRESSED DURING: 11 growth stages; CONTAINS InterPro DOMAIN/s: Chitin-binding, type 1, conserved site (InterPro:IPR018371), Glycoside hydrolase, family 19 (InterPro:IPR016283), Chitin-binding, type 1 (InterPro:IPR001002), Glycoside hydrolase, family 19, catalytic (InterPro:IPR000726); BEST Arabidopsis thaliana protein match is: Chitinase family protein (TAIR:AT2G43610.1)
AT3G09922	*2,029*	INDUCED BY PHOSPHATE STARVATION1 (IPS1)	Encodes a gene product whose expression is responsive to both phosphate (Pi) and phosphite (Phi) in both roots and shoots.
AT4G24540	*2,029*	AGAMOUS-LIKE 24 (AGL24)	Encodes a MADS-box protein involved in flowering. Regulates the expression of SOC1 and is also upregulated by SOC1. Binds with IMK3 kinase domain. Phosphorylated by IMK3; likely to be a target for IMK3 kinase domain.
AT3G20520	*2,025*	SHV3-LIKE 3 (SVL3)	Encodes a member of the glycerophosphodiester phosphodiesterase like (GDPD-like) family.
AT4G22753	*2,024*	STEROL 4-ALPHA METHYL OXIDASE 1-3 (SMO1-3)	Encodes a member of the SMO1 family of sterol 4alpha-methyl oxidases.
AT2G29460	*2,023*	GLUTATHIONE S-TRANSFERASE TAU 4 (GSTU4)	Encodes glutathione transferase belonging to the tau class of GSTs. Naming convention according to Wagner et al. (2002).
AT5G65890	*2,023*	ACT DOMAIN REPEAT 1 (ACR1)	Member of ACT domain containing protein family. ACT domains are amino acid binding domains. Shows strongest expression in flowers and siliques.
AT4G28290	*2,022*		unknown protein; FUNCTIONS IN: molecular_function unknown; INVOLVED IN: biological_process unknown; LOCATED IN: chloroplast; EXPRESSED IN: 23 plant structures; EXPRESSED DURING: 15 growth stages

AT4G15100	*2,021*	SERINE CARBOXYPEPTIDASE-LIKE 30 (scpl30)	serine carboxypeptidase-like 30 (scpl30); FUNCTIONS IN: serine-type carboxypeptidase activity; INVOLVED IN: proteolysis; LOCATED IN: endomembrane system; EXPRESSED IN: root, flower; EXPRESSED DURING: petal differentiation and expansion stage; CONTAINS InterPro DOMAIN/s: Peptidase S10, serine carboxypeptidase (InterPro:IPR001563), Peptidase S10, serine carboxypeptidase, active site (InterPro:IPR018202); BEST Arabidopsis thaliana protein match is: serine carboxypeptidase-like 31 (TAIR:AT1G11080.2)
AT3G29590	*2,015*	(AT5MAT)	At3g29590 (At5MAT) encodes a malonyl-CoA:anthocyanidin 5-O-glucoside-6"-O-malonyltransferase that is coordinately expressed with a epistatic 5-O-anthocyanidin glucosyltransferase (At4g14090). The enzyme is involved in the malonylation of anthocyanins in Arabidopsis.
AT1G26540	*2,014*		Agenet domain-containing protein; FUNCTIONS IN: RNA binding; INVOLVED IN: biological_process unknown; LOCATED IN: vacuole; EXPRESSED IN: 13 plant structures; EXPRESSED DURING: 8 growth stages; CONTAINS InterPro DOMAIN/s: Tudor-like, plant (InterPro:IPR014002), Agenet (InterPro:IPR008395), Protein of unknown function DUF724 (InterPro:IPR007930); BEST Arabidopsis thaliana protein match is: DOMAIN OF UNKNOWN FUNCTION 724 6 (TAIR:AT2G47230.1)
AT3G16530	*2,01*		Lectin like protein whose expression is induced upon treatment with chitin oligomers.
AT5G24420	*2,007*	6-PHOSPHOGLUCONOLACTONASE 5 (PGL5)	Encodes a cytosolic 6-phosphogluconolactonase (PGL) thought to be involved in the oxidative pentose-phosphate pathway (OPPP).
AT1G08630	*2,006*	THREONINE ALDOLASE 1 (THA1)	Encodes a threonine aldolase, involved in threonine degradation to glycine. Primarily expressed in seeds and seedlings.
AT2G21330	*2,005*	FRUCTOSE-BISPHOSPHATE ALDOLASE 1 (FBA1)	fructose-bisphosphate aldolase 1 (FBA1); FUNCTIONS IN: fructose-bisphosphate aldolase activity, catalytic activity; INVOLVED IN: response to cadmium ion, pentose-phosphate shunt; LOCATED IN: in 8 components; EXPRESSED IN: 25 plant structures; EXPRESSED DURING: 15 growth stages; CONTAINS InterPro DOMAIN/s: Aldolase-type TIM barrel (InterPro:IPR013785), Fructose-bisphosphate aldolase, class-I (InterPro:IPR000741); BEST Arabidopsis thaliana protein match is: fructose-bisphosphate aldolase 2 (TAIR:AT4G38970.1)
AT4G25750	*2,005*	ATP-BINDING CASSETTE G4 (ABCG4)	ABC-2 type transporter family protein; FUNCTIONS IN: ATPase activity, coupled to transmembrane movement of substances; LOCATED IN: membrane; EXPRESSED IN: 8 plant structures; EXPRESSED DURING: 6 growth stages; CONTAINS InterPro DOMAIN/s: ATPase, AAA+ type, core (InterPro:IPR003593), ABC transporter-like (InterPro:IPR003439), ABC-2 type transporter (InterPro:IPR013525), ABC transporter, conserved site (InterPro:IPR017871); BEST Arabidopsis thaliana protein match is: ABC-2 type transporter family protein (TAIR:AT5G52860.1)
AT5G17220	*2*	GLUTATHIONE S-TRANSFERASE PHI 12 (GSTF12)	Encodes glutathione transferase belonging to the phi class of GSTs. Naming convention according to Wagner et al. (2002). Mutants display no pigments on leaves and stems. Likely to function as a carrier to transport anthocyanin from the cytosol to tonoplasts.
AT5G19880	*2*		Peroxidase superfamily protein; FUNCTIONS IN: peroxidase activity, heme binding; INVOLVED IN: response to ethylene stimulus, oxidation reduction, response to oxidative stress, response to virus, N-terminal protein myristoylation; LOCATED IN: endomembrane system; EXPRESSED IN: 6 plant structures; EXPRESSED DURING: 4 anthesis, C globular stage; CONTAINS InterPro DOMAIN/s: Haem peroxidase (InterPro:IPR010255), Plant peroxidase (InterPro:IPR000823), Peroxidases heam-ligand binding site (InterPro:IPR019793), Peroxidase, active site (InterPro:IPR019794), Haem peroxidase, plant/fungal/bacterial (InterPro:IPR002016); BEST Arabidopsis thaliana protein match is: peroxidase 2 (TAIR:AT5G06720.1)
AT3G09520	*1,997*	EXOCYST SUBUNIT EXO70 FAMILY PROTEIN H4 (EXO70H4)	A member of EXO70 gene family, putative exocyst subunits, conserved in land plants. Arabidopsis thaliana contains 23 putative EXO70 genes, which can be classified into eight clusters on the phylogenetic tree.
AT1G32570	*1,995*		unknown protein
AT2G04050	*1,994*		MATE efflux family protein; FUNCTIONS IN: antiporter activity, drug transmembrane transporter activity, transporter activity; INVOLVED IN: drug transmembrane transport, transmembrane transport; LOCATED IN: membrane; EXPRESSED IN: stem, leaf whorl, stamen; EXPRESSED DURING: 4 anthesis, petal differentiation and expansion stage; CONTAINS InterPro DOMAIN/s: MATE family transporter related protein (InterPro:IPR015521), Multi antimicrobial extrusion protein MatE (InterPro:IPR002528); BEST Arabidopsis thaliana protein match is: MATE efflux family protein (TAIR:AT2G04070.1)

AT4G22710	*1,992*	CYTOCHROME P450, FAMILY 706, SUBFAMILY A, POLYPEPTIDE 2 (CYP706A2)	member of CYP706A
AT4G29760	*1,991*	HEAT-INDUCED TAS1 TARGET 4 (HTT4)	unknown protein; BEST Arabidopsis thaliana protein match is: unknown protein (TAIR:AT4G29770.2)
AT5G22570	*1,99*	WRKY DNA-BINDING PROTEIN 38 (WRKY38)	member of WRKY Transcription Factor; Group III
AT4G10060	*1,989*		Beta-glucosidase, GBA2 type family protein; FUNCTIONS IN: catalytic activity, glucosylceramidase activity; INVOLVED IN: glucosylceramide catabolic process, sphingolipid metabolic process; LOCATED IN: vacuole; EXPRESSED IN: 21 plant structures; EXPRESSED DURING: 13 growth stages; CONTAINS InterPro DOMAIN/s: Glucosylceramidase (InterPro:IPR006775), Six-hairpin glycosidase-like (InterPro:IPR008928), Beta-glucosidase, GBA2 type (InterPro:IPR014551); BEST Arabidopsis thaliana protein match is: Beta-glucosidase, GBA2 type family protein (TAIR:AT1G33700.2)
AT5G10140	*1,988*	FLOWERING LOCUS C (FLC)	MADS-box protein encoded by FLOWERING LOCUS C - transcription factor that functions as a repressor of floral transition and contributes to temperature compensation of the circadian clock. Expression is downregulated during cold treatment. Vernalization, FRI and the autonomous pathway all influence the state of FLC chromatin. Both maternal and paternal alleles are reset by vernalization, but their earliest activation differs in timing and location. Histone H3 trimethylation at lysine 4 and histone acetylation are associated with active FLC expression, whereas histone deacetylation and histone H3 dimethylation at lysines 9 and 27 are involved in FLC repression. Expression is also repressed by two small RNAs (30- and 24-nt) complementary to the FLC sense strand 3? to the polyA site. The small RNAs are most likely derived from an antisense transcript of FLC. Interacts with SOC1 and FT chromatin in vivo. Member of a protein complex.
AT2G26010	*1,987*	PLANT DEFENSIN 1.3 (PDF1.3)	Predicted to encode a PR (pathogenesis-related) protein. Belongs to the plant defensin (PDF) family with the following members: At1g75830/PDF1.1, At5g44420/PDF1.2a, At2g26020/PDF1.2b, At5g44430/PDF1.2c, At2g26010/PDF1.3, At1g19610/PDF1.4, At1g55010/PDF1.5, At2g02120/PDF2.1, At2g02100/PDF2.2, At2g02130/PDF2.3, At1g61070/PDF2.4, At5g63660/PDF2.5, At2g02140/PDF2.6, At5g38330/PDF3.1 and At4g30070/PDF3.2.
AT5G26340	*1,987*	(MSS1)	Encodes a protein with high affinity, hexose-specific/H+ symporter activity. The activity of the transporter appears to be negatively regulated by phosphorylation. Importantly, microarray analysis, as well as the study of the expression of this gene in mutants involved in programmed cell death (PCD) demonstrated a tight correlation between this gene's expression and PCD.
AT1G79680	*1,986*	WALL ASSOCIATED KINASE (WAK)-LIKE 10 (WAKL10)	Encodes a twin-domain, kinase-GC signaling molecule that may function in biotic stress responses that is critically dependent on the second messenger cGMP.
AT5G06720	*1,985*	PEROXIDASE 2 (PA2)	Encodes a peroxidase with diverse roles in the wound response, flower development, and syncytium formation.
AT4G02690	*1,983*	LIFEGUARD 3 (LFG3)	Bax inhibitor-1 family protein; CONTAINS InterPro DOMAIN/s: Inhibitor of apoptosis-promoting Bax1 related (InterPro:IPR006214); BEST Arabidopsis thaliana protein match is: Bax inhibitor-1 family protein (TAIR:AT1G03070.2)
AT5G38710	*1,974*		Methylenetetrahydrofolate reductase family protein; FUNCTIONS IN: proline dehydrogenase activity; INVOLVED IN: oxidation reduction, proline catabolic process, glutamate biosynthetic process; EXPRESSED IN: 21 plant structures; EXPRESSED DURING: 12 growth stages; CONTAINS InterPro DOMAIN/s: Proline dehydrogenase (InterPro:IPR002872), Proline oxidase (InterPro:IPR015659); BEST Arabidopsis thaliana protein match is: Methylenetetrahydrofolate reductase family protein (TAIR:AT3G30775.1)
AT2G18150	*1,973*		Peroxidase superfamily protein; FUNCTIONS IN: peroxidase activity, heme binding; INVOLVED IN: response to oxidative stress, oxidation reduction, response to nematode; LOCATED IN: cell wall; CONTAINS InterPro DOMAIN/s: Haem peroxidase (InterPro:IPR010255), Plant peroxidase (InterPro:IPR000823), Peroxidases heam-ligand binding site (InterPro:IPR019793), Haem peroxidase, plant/fungal/bacterial (InterPro:IPR002016), Peroxidase, active site (InterPro:IPR019794); BEST Arabidopsis thaliana protein match is: Peroxidase superfamily protein (TAIR:AT2G18140.1)
AT1G07540	*1,967*	TRF-LIKE 2 (TRFL2)	Arabidopsis thaliana telomere-binding protein, putative (At1g07540)
AT2G34600	*1,965*	JASMONATE-ZIM-DOMAIN PROTEIN 7 (JAZ7)	jasmonate-zim-domain protein 7 (JAZ7); CONTAINS InterPro DOMAIN/s: Tify (InterPro:IPR010399); BEST Arabidopsis thaliana protein match is: jasmonate-zim-domain protein 8 (TAIR:AT1G30135.1)

AT2G37240	*1,964*		Thioredoxin superfamily protein; FUNCTIONS IN: oxidoreductase activity, antioxidant activity; INVOLVED IN: biological_process unknown; LOCATED IN: chloroplast; EXPRESSED IN: 21 plant structures; EXPRESSED DURING: 14 growth stages; CONTAINS InterPro DOMAIN/s: Thioredoxin fold (InterPro:IPR012335), Alkyl hydroperoxide reductase/ Thiol specific antioxidant/ Mal allergen (InterPro:IPR000866), Thioredoxin-like fold (InterPro:IPR012336); BEST Arabidopsis thaliana protein match is: Thioredoxin superfamily protein (TAIR:AT5G65840.1)
AT2G30770	*1,962*	CYTOCHROME P450, FAMILY 71, SUBFAMILY A, POLYPEPTIDE 13 (CYP71A13)	putative cytochrome P450
AT1G15040	*1,961*	GLUTAMINE AMIDOTRANSFERASE 1_2.1 (GAT1_2.1)	Encodes a nitrogen regulated putative glutamine amidotransferase that represses shoot branching.
AT1G44890	*1,961*		BEST Arabidopsis thaliana protein match is: homolog of yeast oxidase assembly 1 (OXA1) (TAIR:AT5G62050.1)
AT5G43650	*1,961*	(BHLH92)	BHLH92; FUNCTIONS IN: DNA binding, sequence-specific DNA binding transcription factor activity; INVOLVED IN: regulation of transcription; LOCATED IN: nucleus; CONTAINS InterPro DOMAIN/s: Helix-loop-helix DNA-binding domain (InterPro:IPR001092), Helix-loop-helix DNA-binding (InterPro:IPR011598); BEST Arabidopsis thaliana protein match is: basic helix-loop-helix (bHLH) DNA-binding superfamily protein (TAIR:AT4G09820.1)
AT2G46110	*1,956*	KETOPANTOATE HYDROXYMETHYLTRANSFERASE 1 (KPHMT1)	Encodes a ketopentoate hydroxymethyltransferase that appears to localize to the mitochondria. This protein is expected to play a role in pantothenate (vitamin B5) biosynthesis.
AT5G42120	*1,95*	L-TYPE LECTIN RECEPTOR KINASE S.6 (LECRK-S.6)	Concanavalin A-like lectin protein kinase family protein; FUNCTIONS IN: kinase activity; INVOLVED IN: protein amino acid phosphorylation; LOCATED IN: endomembrane system; CONTAINS InterPro DOMAIN/s: Legume lectin, beta chain (InterPro:IPR001220), Protein kinase, ATP binding site (InterPro:IPR017441), Serine/threonine-protein kinase-like domain (InterPro:IPR017442), Concanavalin A-like lectin/glucanase, subgroup (InterPro:IPR013320), Protein kinase-like domain (InterPro:IPR011009), Serine/threonine-protein kinase, active site (InterPro:IPR008271), Protein kinase, catalytic domain (InterPro:IPR000719), Concanavalin A-like lectin/glucanase (InterPro:IPR008985), Legume lectin, beta chain, Mn/Ca-binding site (InterPro:IPR019825); BEST Arabidopsis thaliana protein match is: Concanavalin A-like lectin protein kinase family protein (TAIR:AT5G55830.1)
AT2G35820	*1,947*		ureidoglycolate hydrolases; FUNCTIONS IN: ureidoglycolate hydrolase activity; INVOLVED IN: allantoin catabolic process; LOCATED IN: cellular_component unknown; EXPRESSED IN: 17 plant structures; EXPRESSED DURING: 13 growth stages; CONTAINS InterPro DOMAIN/s: Ureidoglycolate hydrolase (InterPro:IPR007247); BEST Arabidopsis thaliana protein match is: unknown protein (TAIR:AT2G35810.1)
AT2G32765	*1,945*	SMALL UBIQUITINRELATED MODIFIER 5 (SUMO5)	Encodes a small ubiquitin-like modifier (SUMO) protein that becomes covalently attached to various intracellular protein targets through an isopeptide bond. SUMOylation typically has a post-translational effect on the behavior of the target protein.
AT4G13800	*1,944*		Protein of unknown function (DUF803); INVOLVED IN: biological_process unknown; LOCATED IN: plasma membrane; EXPRESSED IN: 18 plant structures; EXPRESSED DURING: 12 growth stages; CONTAINS InterPro DOMAIN/s: Protein of unknown function DUF803 (InterPro:IPR008521); BEST Arabidopsis thaliana protein match is: Protein of unknown function (DUF803) (TAIR:AT3G23870.1)
AT5G37240	*1,942*		unknown protein
AT5G39150	*1,941*		RmlC-like cupins superfamily protein; FUNCTIONS IN: manganese ion binding, nutrient reservoir activity; INVOLVED IN: biological_process unknown; LOCATED IN: endomembrane system, apoplast; CONTAINS InterPro DOMAIN/s: Cupin, RmlC-type (InterPro:IPR011051), Cupin 1 (InterPro:IPR006045), RmlC-like jelly roll fold (InterPro:IPR014710), Germin (InterPro:IPR001929), Germin, manganese binding site (InterPro:IPR019780); BEST Arabidopsis thaliana protein match is: RmlC-like cupins superfamily protein (TAIR:AT5G39180.1)

AT4G27590	*1,934*		Heavy metal transport/detoxification superfamily protein ; FUNCTIONS IN: copper ion binding, metal ion binding; INVOLVED IN: copper ion transport, metal ion transport; LOCATED IN: cellular_component unknown; EXPRESSED IN: 8 plant structures; EXPRESSED DURING: 6 growth stages; CONTAINS InterPro DOMAIN/s: Heavy metal transport/detoxification protein (InterPro:IPR006121); BEST Arabidopsis thaliana protein match is: Heavy metal transport/detoxification superfamily protein (TAIR:AT3G56891.1)
AT1G15960	*1,93*	NRAMP METAL ION TRANSPORTER 6 (NRAMP6)	member of Nramp2 family
AT4G23720	*1,926*		Protein of unknown function (DUF1191); FUNCTIONS IN: molecular_function unknown; INVOLVED IN: biological_process unknown; LOCATED IN: plasma membrane; EXPRESSED IN: 16 plant structures; EXPRESSED DURING: 7 growth stages; CONTAINS InterPro DOMAIN/s: Protein of unknown function DUF1191 (InterPro:IPR010605); BEST Arabidopsis thaliana protein match is: Protein of unknown function (DUF1191) (TAIR:AT4G01140.1)
AT2G24850	*1,92*	TYROSINE AMINOTRANSFERASE 3 (TAT3)	Encodes a tyrosine aminotransferase that is responsive to treatment with jasmonic acid.
AT1G67000	*1,918*		Protein kinase superfamily protein; FUNCTIONS IN: protein serine/threonine kinase activity, protein kinase activity, kinase activity, ATP binding; INVOLVED IN: protein amino acid phosphorylation; LOCATED IN: endomembrane system; EXPRESSED IN: stem; CONTAINS InterPro DOMAIN/s: Protein kinase, ATP binding site (InterPro:IPR017441), Protein kinase, catalytic domain (InterPro:IPR000719), Serine-threonine/tyrosine-protein kinase (InterPro:IPR001245), Protein kinase-like domain (InterPro:IPR011009), Serine/threonine-protein kinase, active site (InterPro:IPR008271); BEST Arabidopsis thaliana protein match is: Protein kinase superfamily protein (TAIR:AT1G66930.1)
AT3G28360	*1,918*	ATP-BINDING CASSETTE B16 (ABCB16)	P-glycoprotein 16 (PGP16); FUNCTIONS IN: ATPase activity, coupled to transmembrane movement of substances; INVOLVED IN: transport, transmembrane transport; LOCATED IN: integral to membrane; EXPRESSED IN: flower; EXPRESSED DURING: petal differentiation and expansion stage; CONTAINS InterPro DOMAIN/s: ATPase, AAA+ type, core (InterPro:IPR003593), ABC transporter-like (InterPro:IPR003439), ABC transporter, transmembrane domain, type 1 (InterPro:IPR011527), ABC transporter integral membrane type 1 (InterPro:IPR017940), ABC transporter, transmembrane domain (InterPro:IPR001140), ABC transporter, conserved site (InterPro:IPR017871); BEST Arabidopsis thaliana protein match is: P-glycoprotein 18 (TAIR:AT3G28390.1)
AT5G40990	*1,918*	GDSL LIPASE 1 (GLIP1)	Component of plant resistance. Contains lipase signature motif and GDSL domain. Directly interferes with the fungal infection process by acting on fungal cell walls through its action as a antimicrobial compound. Critical component for both local and systemic resistance responses in the incompatible interaction with Alternaria brassicicola in the ethylene-dependent pathway.
AT2G02930	*1,906*	GLUTATHIONE S-TRANSFERASE F3 (GSTF3)	Encodes glutathione transferase belonging to the phi class of GSTs. Naming convention according to Wagner et al. (2002).
AT4G22600	*1,903*	INAPERTURATE POLLEN1 (INP1)	unknown protein
AT5G38240	*1,901*		Protein kinase family protein; FUNCTIONS IN: protein serine/threonine kinase activity, protein kinase activity, kinase activity, ATP binding; INVOLVED IN: protein amino acid phosphorylation; LOCATED IN: endomembrane system; CONTAINS InterPro DOMAIN/s: Protein kinase, ATP binding site (InterPro:IPR017441), Serine/threonine-protein kinase domain (InterPro:IPR002290), Serine-threonine/tyrosine-protein kinase (InterPro:IPR001245), Protein kinase-like domain (InterPro:IPR011009), Serine/threonine-protein kinase, active site (InterPro:IPR008271), Protein kinase, catalytic domain (InterPro:IPR000719), Tyrosine-protein kinase, catalytic domain (InterPro:IPR020635); BEST Arabidopsis thaliana protein match is: Protein kinase family protein (TAIR:AT5G38250.1)
AT1G13790	*1,9*	FACTOR OF DNA METHYLATION 4 (FDM4)	Belongs to a subgroup of SGS3-like proteins that act redundantly in RNA-directed DNA methylation: AT1G15910 (FDM1), AT4G00380 (FDM2), AT3G12550 (FDM3), AT1G13790 (FDM4), AT1G80790 (FDM5).
AT3G26570	*1,899*	PHOSPHATE TRANSPORTER 2;1 (PHT2;1)	low affinity phosphate transporter

AT2G05520	*1,896*	GLYCINE-RICH PROTEIN 3 (GRP-3)	Encodes a glycine-rich protein that is expressed mainly in stems and leaves. AtGRP3 functions in root size determination during development and in Al stress. mRNA levels are upregulated in response to ABA, salicylic acid and ethylene but downregulated in response to desiccation. The mRNA is cell-to-cell mobile.
AT1G04560	*1,895*		AWPM-19-like family protein; FUNCTIONS IN: molecular_function unknown; INVOLVED IN: biological_process unknown; LOCATED IN: endomembrane system; CONTAINS InterPro DOMAIN/s: AWPM-19-like (InterPro:IPR008390); BEST Arabidopsis thaliana protein match is: AWPM-19-like family protein (TAIR:AT5G46530.1)
AT2G07727	*1,894*		Di-haem cytochrome, transmembrane;Cytochrome b/b6, C-terminal; FUNCTIONS IN: electron carrier activity, oxidoreductase activity; INVOLVED IN: respiratory electron transport chain; LOCATED IN: membrane; CONTAINS InterPro DOMAIN/s: Cytochrome b/b6, C-terminal (InterPro:IPR005798), Cytochrome b/b6 (InterPro:IPR016175), Di-haem cytochrome, transmembrane (InterPro:IPR016174), Cytochrome b/b6, N-terminal (InterPro:IPR005797); BEST Arabidopsis thaliana protein match is: apocytochrome b (TAIR:ATMG00220.1)
AT5G08000	*1,892*	GLUCAN ENDO-1,3-BETA-GLUCOSIDASE-LIKE PROTEIN 3 (E13L3)	Encodes a member of the X8-GPI family of proteins. It localizes to the plasmodesmata and binds callose.
AT5G14760	*1,892*	L-ASPARTATE OXIDASE (AO)	At5g14760 encodes for L-aspartate oxidase involved in the early steps of NAD biosynthesis. In contrary to the EC 1.4.3.16 (l-aspartate oxidase - deaminating) the enzyme catalyzes the reaction L-aspartate + O2 = iminoaspartate (alpha-iminosuccinate) + H2O2
AT1G80660	*1,89*	H(+)-ATPASE 9 (HA9)	H(+)-ATPase 9 (HA9); FUNCTIONS IN: hydrogen-exporting ATPase activity, phosphorylative mechanism; INVOLVED IN: proton transport; LOCATED IN: plasma membrane; EXPRESSED IN: 12 plant structures; EXPRESSED DURING: L mature pollen stage, M germinated pollen stage, 4 anthesis, petal differentiation and expansion stage; CONTAINS InterPro DOMAIN/s: ATPase, P-type, ATPase-associated domain (InterPro:IPR008250), ATPase, P-type cation-transporter, N-terminal (InterPro:IPR004014), Haloacid dehalogenase-like hydrolase (InterPro:IPR005834), ATPase, P-type, H+ transporting proton pump (InterPro:IPR000695), ATPase, P-type, K/Mg/Cd/Cu/Zn/Na/Ca/Na/H-transporter (InterPro:IPR001757), ATPase, P-type, plasma-membrane proton-efflux (InterPro:IPR006534), ATPase, P-type phosphorylation site (InterPro:IPR018303); BEST Arabidopsis thaliana protein match is: H(+)-ATPase 8 (TAIR:AT3G42640.1)
AT1G33030	*1,888*		O-methyltransferase family protein; CONTAINS InterPro DOMAIN/s: Winged helix-turn-helix transcription repressor DNA-binding (InterPro:IPR011991), Plant methyltransferase dimerisation (InterPro:IPR012967), O-methyltransferase, family 2 (InterPro:IPR001077), O-methyltransferase, COMT, eukaryota (InterPro:IPR016461); BEST Arabidopsis thaliana protein match is: O-methyltransferase 1 (TAIR:AT5G54160.1)
AT5G14700	*1,886*		NAD(P)-binding Rossmann-fold superfamily protein; FUNCTIONS IN: coenzyme binding, binding, cinnamoyl-CoA reductase activity, catalytic activity; INVOLVED IN: lignin biosynthetic process, cellular metabolic process, metabolic process; LOCATED IN: cellular_component unknown; EXPRESSED IN: 22 plant structures; EXPRESSED DURING: 13 growth stages; CONTAINS InterPro DOMAIN/s: NAD-dependent epimerase/dehydratase (InterPro:IPR001509), NAD(P)-binding domain (InterPro:IPR016040); BEST Arabidopsis thaliana protein match is: NAD(P)-binding Rossmann-fold superfamily protein (TAIR:AT2G23910.1)
AT1G80580	*1,882*		encodes a member of the ERF (ethylene response factor) subfamily B-1 of ERF/AP2 transcription factor family. The protein contains one AP2 domain. There are 15 members in this subfamily including ATERF-3, ATERF-4, ATERF-7, and leafy petiole.
AT1G29860	*1,878*	WRKY DNA-BINDING PROTEIN 71 (WRKY71)	member of WRKY Transcription Factor; Group II-c
AT5G17860	*1,878*	CALCIUM EXCHANGER 7 (CAX7)	calcium exchanger 7 (CAX7); FUNCTIONS IN: cation:cation antiporter activity, calcium:sodium antiporter activity; INVOLVED IN: cation transport, transmembrane transport; LOCATED IN: endomembrane system, integral to membrane; EXPRESSED IN: 19 plant structures; EXPRESSED DURING: 10 growth stages; CONTAINS InterPro DOMAIN/s: Sodium/calcium exchanger membrane region (InterPro:IPR004837); BEST Arabidopsis thaliana protein match is: cation calcium exchanger 4 (TAIR:AT1G54115.1)

AT5G64750	*1,875*	ABA REPRESSOR1 (ABR1)	Encodes a putative transcription factor containing an AP2 domain. Is a member of the ERF (ethylene response factor) subfamily B-4 of ERF/AP2 transcription factor family. Expressed in response to ABA, osmotic stress, sugar stress and drought. Mutants are hypersensitive to these stresses. May be involved in regulation of ABA mediated stress response. The mRNA is cell-to-cell mobile.
AT2G23970	*1,872*		Class I glutamine amidotransferase-like superfamily protein; FUNCTIONS IN: catalytic activity; INVOLVED IN: defense response; LOCATED IN: endomembrane system; EXPRESSED IN: 6 plant structures; EXPRESSED DURING: 4 anthesis, petal differentiation and expansion stage; CONTAINS InterPro DOMAIN/s: Glutamine amidotransferase class-I, C-terminal (InterPro:IPR000991), Glutamine amidotransferase type 1 (InterPro:IPR017926); BEST Arabidopsis thaliana protein match is: Class I glutamine amidotransferase-like superfamily protein (TAIR:AT4G30550.1)
AT1G08430	*1,871*	ALUMINUM-ACTIVATED MALATE TRANSPORTER 1 (ALMT1)	Encodes a Al-activated malate efflux transporter. It is essential for aluminum tolerance but does not represent the major Al tolerance QTL. Staurosporine and calyculin A both block all changes in AtALMT1 gene expression (as a result malate release is totally inhibited). AtALMT1 transcription was clearly induced by indole-3-acetic acid, abscisic acid, low pH, hydrogen peroxide and flg22. STOP1 and CAMTA2 transcription factors are involved in Al-inducible expression of AtALMT1 and both proteins bind to the AtALMT1 promoter.
AT5G45520	*1,87*		Leucine-rich repeat (LRR) family protein; FUNCTIONS IN: molecular_function unknown; INVOLVED IN: biological_process unknown; LOCATED IN: chloroplast; BEST Arabidopsis thaliana protein match is: RNI-like superfamily protein (TAIR:AT5G45500.2)
AT2G19800	*1,865*	MYO-INOSITOL OXYGENASE 2 (MIOX2)	Encodes a myo-inositol oxygenase family gene.
AT3G10450	*1,865*	SERINE CARBOXYPEPTIDASE-LIKE 7 (SCPL7)	serine carboxypeptidase-like 7 (SCPL7); FUNCTIONS IN: serine-type carboxypeptidase activity; INVOLVED IN: proteolysis; LOCATED IN: endomembrane system; EXPRESSED IN: 18 plant structures; EXPRESSED DURING: 11 growth stages; CONTAINS InterPro DOMAIN/s: Peptidase S10, serine carboxypeptidase (InterPro:IPR001563); BEST Arabidopsis thaliana protein match is: serine carboxypeptidase-like 3 (TAIR:AT1G73280.1)
AT5G41610	*1,865*	CATION/H+ EXCHANGER 18 (CHX18)	member of Putative Na+/H+ antiporter family
AT1G09180	*1,86*	SECRETION-ASSOCIATED RAS SUPER FAMILY 1 (SARA1A)	A member of ARF-like GTPase family. A thaliana has 21 members, in two subfamilies, ARF and ARF-like (ARL) GTPases.
AT1G62760	*1,859*	(ATPMEI10)	Plant invertase/pectin methylesterase inhibitor superfamily protein; FUNCTIONS IN: enzyme inhibitor activity, pectinesterase inhibitor activity, pectinesterase activity; EXPRESSED IN: stem, sepal, male gametophyte, carpel, stamen; EXPRESSED DURING: 4 anthesis; CONTAINS InterPro DOMAIN/s: Pectinesterase inhibitor (InterPro:IPR006501); BEST Arabidopsis thaliana protein match is: Plant invertase/pectin methylesterase inhibitor superfamily protein (TAIR:AT4G25260.1)
AT4G24260	*1,859*	GLYCOSYL HYDROLASE 9A3 (GH9A3)	Encodes a protein with similarity to endo-1,4-b-glucanases. KOR3 is induced by nemotodes and is expressed in syncitia induced by Heterodera schachtii.May be involved in the development and function of syncitia.
AT1G63210	*1,858*		SPT6L encodes a putative WG/GW-repeat protein involved in the regulation of apical?basal polarity of embryo
AT2G04080	*1,858*		MATE efflux family protein; FUNCTIONS IN: antiporter activity, drug transmembrane transporter activity, transporter activity; INVOLVED IN: drug transmembrane transport, transmembrane transport; LOCATED IN: membrane; CONTAINS InterPro DOMAIN/s: MATE family transporter related protein (InterPro:IPR015521), Multi antimicrobial extrusion protein MatE (InterPro:IPR002528); BEST Arabidopsis thaliana protein match is: MATE efflux family protein (TAIR:AT2G04070.1)
AT1G04920	*1,857*	SUCROSE PHOSPHATE SYNTHASE 3F (SPS3F)	Encodes a protein with putative sucrose-phosphate synthase activity.
AT1G12320	*1,857*		Protein of unknown function (DUF1442); CONTAINS InterPro DOMAIN/s: Protein of unknown function DUF1442 (InterPro:IPR009902); BEST Arabidopsis thaliana protein match is: Protein of unknown function (DUF1442) (TAIR:AT1G62840.1)

AT2G34850	*1,857*	MATERNAL EFFECT EMBRYO ARREST 25 (MEE25)	maternal effect embryo arrest 25 (MEE25); FUNCTIONS IN: coenzyme binding, UDP-glucose 4-epimerase activity, catalytic activity; INVOLVED IN: nucleotide-sugar metabolic process, pollen tube development, embryo development ending in seed dormancy; LOCATED IN: cellular_component unknown; EXPRESSED IN: embryo, leaf whorl, flower, seed; EXPRESSED DURING: F mature embryo stage, petal differentiation and expansion stage, E expanded cotyledon stage, D bilateral stage; CONTAINS InterPro DOMAIN/s: NAD-dependent epimerase/dehydratase (InterPro:IPR001509), UDP-glucose 4-epimerase (InterPro:IPR005886); BEST Arabidopsis thaliana protein match is: NAD(P)-binding Rossmann-fold superfamily protein (TAIR:AT1G30620.2)
AT3G60120	*1,856*	BETA GLUCOSIDASE 27 (BGLU27)	beta glucosidase 27 (BGLU27); FUNCTIONS IN: cation binding, hydrolase activity, hydrolyzing O-glycosyl compounds, catalytic activity; INVOLVED IN: carbohydrate metabolic process; LOCATED IN: cellular_component unknown; EXPRESSED IN: stem, hypocotyl, sepal, stamen; EXPRESSED DURING: 4 anthesis; CONTAINS InterPro DOMAIN/s: Glycoside hydrolase, family 1 (InterPro:IPR001360), Glycoside hydrolase, family 1, active site (InterPro:IPR018120), Glycoside hydrolase, catalytic core (InterPro:IPR017853), Glycoside hydrolase, subgroup, catalytic core (InterPro:IPR013781); BEST Arabidopsis thaliana protein match is: Glycosyl hydrolase superfamily protein (TAIR:AT2G44490.1)
AT5G61270	*1,853*	PHYTOCHROME-INTERACTING FACTOR7 (PIF7)	Basic helix-loop-helix (bHLH) phytochrome interacting factor. Interacts specifically with the far-red light?absorbing Pfr form of phyB through a conserved domain called the active phyB binding motif. Upon light exposure, PIF7 rapidly migrates to intranuclear speckles, where it colocalizes with phyB. Role as negative regulator of phyB-mediated seedling deetiolation.
AT5G63750	*1,853*	ARIADNE 13 (ARI13)	ARIADNE 13 (ARI13); FUNCTIONS IN: zinc ion binding; LOCATED IN: intracellular; EXPRESSED IN: 6 plant structures; EXPRESSED DURING: 4 anthesis, petal differentiation and expansion stage; CONTAINS InterPro DOMAIN/s: Zinc finger, C6HC-type (InterPro:IPR002867), Zinc finger, RING-type (InterPro:IPR001841), Zinc finger, RanBP2-type (InterPro:IPR001876); BEST Arabidopsis thaliana protein match is: IBR domain-containing protein (TAIR:AT5G63730.1)
AT5G42680	*1,85*		Protein of unknown function, DUF617; CONTAINS InterPro DOMAIN/s: Protein of unknown function DUF617, plant (InterPro:IPR006460); BEST Arabidopsis thaliana protein match is: Protein of unknown function, DUF617 (TAIR:AT4G39610.1)
AT3G63140	*1,849*	CHLOROPLAST STEM-LOOP BINDING PROTEIN OF 41 KDA (CSP41A)	Encodes a protein with ribonuclease activity that is involved in plastid rRNA maturation.
AT1G68470	*1,846*		Exostosin family protein; FUNCTIONS IN: catalytic activity; INVOLVED IN: biological_process unknown; LOCATED IN: membrane; EXPRESSED IN: 18 plant structures; EXPRESSED DURING: 13 growth stages; CONTAINS InterPro DOMAIN/s: Exostosin-like (InterPro:IPR004263); BEST Arabidopsis thaliana protein match is: Exostosin family protein (TAIR:AT2G20370.1)
AT4G27190	*1,843*		NB-ARC domain-containing disease resistance protein; FUNCTIONS IN: nucleoside-triphosphatase activity, nucleotide binding, ATP binding; INVOLVED IN: apoptosis, defense response; LOCATED IN: cellular_component unknown; CONTAINS InterPro DOMAIN/s: ATPase, AAA+ type, core (InterPro:IPR003593), NB-ARC (InterPro:IPR002182), Disease resistance protein (InterPro:IPR000767); BEST Arabidopsis thaliana protein match is: NB-ARC domain-containing disease resistance protein (TAIR:AT4G27220.1)
AT1G63360	*1,841*		Disease resistance protein (CC-NBS-LRR class) family; FUNCTIONS IN: ATP binding; INVOLVED IN: N-terminal protein myristoylation, defense response; LOCATED IN: cellular_component unknown; CONTAINS InterPro DOMAIN/s: NB-ARC (InterPro:IPR002182), Disease resistance protein (InterPro:IPR000767); BEST Arabidopsis thaliana protein match is: Disease resistance protein (CC-NBS-LRR class) family (TAIR:AT1G62630.1)
AT1G10680	*1,84*	P-GLYCOPROTEIN 10 (PGP10)	P-glycoprotein 10 (PGP10); FUNCTIONS IN: ATPase activity, coupled to transmembrane movement of substances; INVOLVED IN: transport, transmembrane transport; LOCATED IN: mitochondrion; EXPRESSED IN: sperm cell, male gametophyte; EXPRESSED DURING: L mature pollen stage, M germinated pollen stage; CONTAINS InterPro DOMAIN/s: ATPase, AAA+ type, core (InterPro:IPR003593), ABC transporter-like (InterPro:IPR003439), ABC transporter, transmembrane domain, type 1 (InterPro:IPR011527), ABC transporter integral membrane type 1 (InterPro:IPR017940), ABC transporter, transmembrane domain (InterPro:IPR001140), ABC transporter, conserved site (InterPro:IPR017871); BEST Arabidopsis thaliana protein match is: P-glycoprotein 2 (TAIR:AT4G25960.1)

AT1G71450	*1,84*	FYF UP-REGULATING 321 FACTOR 1 (FUF1)	encodes a member of the DREB subfamily A-4 of ERF/AP2 transcription factor family. The protein contains one AP2 domain. There are 17 members in this subfamily including TINY.
AT5G61660	*1,84*		glycine-rich protein; FUNCTIONS IN: molecular_function unknown; INVOLVED IN: biological_process unknown; LOCATED IN: endomembrane system; EXPRESSED IN: 19 plant structures; EXPRESSED DURING: 9 growth stages
AT4G08170	*1,839*		Inositol 1,3,4-trisphosphate 5/6-kinase family protein; FUNCTIONS IN: magnesium ion binding, inositol-1,3,4-trisphosphate 5/6-kinase activity, catalytic activity, ATP binding, inositol tetrakisphosphate 1-kinase activity; INVOLVED IN: response to wounding; LOCATED IN: nucleus, cytoplasm; EXPRESSED IN: 22 plant structures; EXPRESSED DURING: 11 growth stages; CONTAINS InterPro DOMAIN/s: Inositol-tetrakisphosphate 1-kinase (InterPro:IPR017427), ATP-grasp fold (InterPro:IPR011761), Inositol 1, 3, 4-trisphosphate 56-kinase (InterPro:IPR008656); BEST Arabidopsis thaliana protein match is: Inositol 1,3,4-trisphosphate 5/6-kinase family protein (TAIR:AT4G33770.1)
AT5G07990	*1,837*	TRANSPARENT TESTA 7 (TT7)	Required for flavonoid 3' hydroxylase activity. Enzyme abundance relative to CHS determines Quercetin/Kaempferol metabolite ratio. The mRNA is cell-to-cell mobile.
AT3G44300	*1,836*	NITRILASE 2 (NIT2)	Encodes an enzyme that catalyzes the hydrolysis of indole-3-acetonitrile (IAN) to indole-3-acetic acid (IAA) (nitrile aminohydrolase, EC 3.5.5.1) and IAN to indole-3-acetamide (IAM) at lower levels. Mutants have reduced sensitivity to IAN and are sensitive to IAA. This enzyme likely participates in other non-auxin-related metabolic pathways. The mRNA is cell-to-cell mobile.
AT1G12030	*1,834*		Protein of unknown function (DUF506) ; CONTAINS InterPro DOMAIN/s: Protein of unknown function DUF506, plant (InterPro:IPR006502); BEST Arabidopsis thaliana protein match is: Protein of unknown function (DUF506) (TAIR:AT1G62420.1)
AT1G48530	*1,832*		CONTAINS InterPro DOMAIN/s: Proteasome Inhibitor PI31 (InterPro:IPR021625); BEST Arabidopsis thaliana protein match is: proteasome inhibitor-related (TAIR:AT3G53970.2)
AT4G12410	*1,832*	SMALL AUXIN UPREGULATED RNA 35 (SAUR35)	SAUR-like auxin-responsive protein family ; CONTAINS InterPro DOMAIN/s: Auxin responsive SAUR protein (InterPro:IPR003676); BEST Arabidopsis thaliana protein match is: SAUR-like auxin-responsive protein family (TAIR:AT4G22620.1)
AT4G23250	*1,832*	EMBRYO DEFECTIVE 1290 (emb1290)	EMBRYO DEFECTIVE 1290 (EMB1290); FUNCTIONS IN: protein kinase activity, kinase activity; INVOLVED IN: protein amino acid autophosphorylation, embryo development ending in seed dormancy; LOCATED IN: plasma membrane; EXPRESSED IN: stem; CONTAINS InterPro DOMAIN/s: Protein kinase, ATP binding site (InterPro:IPR017441), Serine/threonine-protein kinase domain (InterPro:IPR002290), Protein of unknown function DUF26 (InterPro:IPR002902), Serine-threonine/tyrosine-protein kinase (InterPro:IPR001245), Protein kinase-like domain (InterPro:IPR011009), Serine/threonine-protein kinase, active site (InterPro:IPR008271), Protein kinase, catalytic domain (InterPro:IPR000719), Tyrosine-protein kinase, catalytic domain (InterPro:IPR020635), Concanavalin A-like lectin/glucanase (InterPro:IPR008985); BEST Arabidopsis thaliana protein match is: cysteine-rich RLK (RECEPTOR-like protein kinase) 18 (TAIR:AT4G23260.1)
AT5G24160	*1,816*	SQUALENE MONOXYGENASE 6 (SQE6)	squalene monoxygenase 6 (SQE6); FUNCTIONS IN: squalene monooxygenase activity, oxidoreductase activity, FAD binding; INVOLVED IN: sterol biosynthetic process; LOCATED IN: endomembrane system, integral to membrane; EXPRESSED IN: 12 plant structures; EXPRESSED DURING: 9 growth stages; CONTAINS InterPro DOMAIN/s: Squalene epoxidase (InterPro:IPR013698), FAD dependent oxidoreductase (InterPro:IPR006076); BEST Arabidopsis thaliana protein match is: FAD/NAD(P)-binding oxidoreductase family protein (TAIR:AT5G24150.1)
AT1G66490	*1,815*		F-box and associated interaction domains-containing protein; CONTAINS InterPro DOMAIN/s: F-box domain, cyclin-like (InterPro:IPR001810), F-box domain, Skp2-like (InterPro:IPR022364), F-box associated domain, type 1 (InterPro:IPR006527), F-box associated interaction domain (InterPro:IPR017451); BEST Arabidopsis thaliana protein match is: F-box and associated interaction domains-containing protein (TAIR:AT3G13680.1)

AT4G15660	*1,814*	(GRXS8)	Thioredoxin superfamily protein; FUNCTIONS IN: electron carrier activity, arsenate reductase (glutaredoxin) activity, protein disulfide oxidoreductase activity; INVOLVED IN: cell redox homeostasis; LOCATED IN: endomembrane system; EXPRESSED IN: 16 plant structures; EXPRESSED DURING: 10 growth stages; CONTAINS InterPro DOMAIN/s: Glutaredoxin-like, plant II (InterPro:IPR011905), Glutaredoxin (InterPro:IPR002109), Thioredoxin fold (InterPro:IPR012335), Glutaredoxin subgroup (InterPro:IPR014025), Thioredoxin-like fold (InterPro:IPR012336); BEST Arabidopsis thaliana protein match is: Thioredoxin superfamily protein (TAIR:AT4G15670.1)
AT5G11930	*1,813*		Thioredoxin superfamily protein; FUNCTIONS IN: electron carrier activity, protein disulfide oxidoreductase activity; INVOLVED IN: cell redox homeostasis; EXPRESSED IN: 11 plant structures; EXPRESSED DURING: 6 growth stages; CONTAINS InterPro DOMAIN/s: Glutaredoxin-like, plant II (InterPro:IPR011905), Thioredoxin fold (InterPro:IPR012335), Glutaredoxin (InterPro:IPR002109), Thioredoxin-like fold (InterPro:IPR012336); BEST Arabidopsis thaliana protein match is: Thioredoxin superfamily protein (TAIR:AT4G33040.1)
AT4G17980	*1,812*	NAC DOMAIN CONTAINING PROTEIN 71 (NAC071)	Encodes ANAC071, a transcription factor involved in cell proliferation in incised inflorescence stems.
AT5G43870	*1,802*		FUNCTIONS IN: phosphoinositide binding; INVOLVED IN: signal transduction; LOCATED IN: plasma membrane; EXPRESSED IN: 21 plant structures; EXPRESSED DURING: 13 growth stages; CONTAINS InterPro DOMAIN/s: Pleckstrin-like, plant (InterPro:IPR013666), Protein of unknown function DUF828 (InterPro:IPR008546); BEST Arabidopsis thaliana protein match is: Plant protein of unknown function (DUF828) with plant pleckstrin homology-like region (TAIR:AT3G22810.1)
AT1G74470	*1,797*		Encodes for a multifunctional protein with geranylgeranyl reductase activity shown to catalyze the reduction of prenylated geranylgeranyl-chlorophyll a to phytyl-chlorophyll a (chlorophyll a) and free geranylgeranyl pyrophosphate to phytyl pyrophosphate. The mRNA is cell-to-cell mobile.
AT2G07719	*1,797*		Putative membrane lipoprotein; FUNCTIONS IN: molecular_function unknown; INVOLVED IN: biological_process unknown; LOCATED IN: endomembrane system; BEST Arabidopsis thaliana protein match is: Putative membrane lipoprotein (TAIR:ATMG00610.1)
AT4G27520	*1,796*	EARLY NODULIN-LIKE PROTEIN 2 (ENODL2)	early nodulin-like protein 2 (ENODL2); FUNCTIONS IN: electron carrier activity, copper ion binding; LOCATED IN: in 6 components; EXPRESSED IN: 24 plant structures; EXPRESSED DURING: 13 growth stages; CONTAINS InterPro DOMAIN/s: Plastocyanin-like (InterPro:IPR003245), Cupredoxin (InterPro:IPR008972); BEST Arabidopsis thaliana protein match is: early nodulin-like protein 1 (TAIR:AT5G53870.1)
AT5G24280	*1,795*	GAMMA-IRRADIATION AND MITOMYCIN C INDUCED 1	Encodes GMI1, a structural-maintenance-of-chromosomes-hinge domain-containing protein. Involved in somatic homologous recombination.
AT1G30160	*1,793*		Protein of unknown function (DUF295); CONTAINS InterPro DOMAIN/s: Protein of unknown function DUF295 (InterPro:IPR005174); BEST Arabidopsis thaliana protein match is: Protein of unknown function (DUF295) (TAIR:AT1G05540.1)
AT2G17780	*1,793*	MID1-COMPLEMENTING ACTIVITY 2 (MCA2)	Encodes a mechanosensitive channel candidate MCA2. The three-dimensional structure of MCA2 was reconstructed and appears to comprise a small transmembrane region and large cytoplasmic region.
AT5G52570	*1,792*	BETA-CAROTENE HYDROXYLASE 2 (BETA-OHASE 2)	Converts β-carotene to zeaxanthin via cryptoxanthin.
AT1G66300	*1,791*		F-box/RNI-like/FBD-like domains-containing protein; CONTAINS InterPro DOMAIN/s: F-box domain, cyclin-like (InterPro:IPR001810), FBD (InterPro:IPR013596), F-box domain, Skp2-like (InterPro:IPR022364), FBD-like (InterPro:IPR006566), Leucine-rich repeat 2 (InterPro:IPR013101); BEST Arabidopsis thaliana protein match is: F-box/RNI-like/FBD-like domains-containing protein (TAIR:AT1G66310.1)
AT1G68320	*1,791*	MYB DOMAIN PROTEIN 62 (MYB62)	putative transcription factor: R2R3-MYB transcription family. Involved in regulation of phosphate starvation responses and gibberellic acid biosynthesis.

AT2G37770	*1,787*	CHLOROPLASTIC ALDO-KETO REDUCTASE (ChlAKR)	Encodes an NADPH-dependent aldo-keto reductase that can act on a wide variety of substrates in vitro including saturated and unsaturated aldehydes, steroids, and sugars. GFP-tagged AKR4C9 localizes to the chloroplast where it may play a role in detoxifying reactive carbonyl compounds that threaten to impair the photosynthetic process. Transcript levels for this gene are up-regulated in response to cold, salt, and drought stress.
AT1G26560	*1,786*	BETA GLUCOSIDASE 40 (BGLU40)	beta glucosidase 40 (BGLU40); FUNCTIONS IN: cation binding, hydrolase activity, hydrolyzing O-glycosyl compounds, catalytic activity; INVOLVED IN: carbohydrate metabolic process; LOCATED IN: apoplast, chloroplast; EXPRESSED IN: 23 plant structures; EXPRESSED DURING: 13 growth stages; CONTAINS InterPro DOMAIN/s: Glycoside hydrolase, family 1 (InterPro:IPR001360), Glycoside hydrolase, family 1, active site (InterPro:IPR018120), Glycoside hydrolase, catalytic core (InterPro:IPR017853), Glycoside hydrolase, subgroup, catalytic core (InterPro:IPR013781); BEST Arabidopsis thaliana protein match is: beta glucosidase 41 (TAIR:AT5G54570.1)
AT2G41360	*1,784*		Galactose oxidase/kelch repeat superfamily protein; CONTAINS InterPro DOMAIN/s: F-box domain, cyclin-like (InterPro:IPR001810), Galactose oxidase/kelch, beta-propeller (InterPro:IPR011043), Kelch-type beta propeller (InterPro:IPR015915); BEST Arabidopsis thaliana protein match is: Galactose oxidase/kelch repeat superfamily protein (TAIR:AT2G44630.1)
AT2G40520	*1,783*		Nucleotidyltransferase family protein; FUNCTIONS IN: nucleotidyltransferase activity; INVOLVED IN: biological_process unknown; LOCATED IN: cellular_component unknown; CONTAINS InterPro DOMAIN/s: Nucleotidyl transferase domain (InterPro:IPR002934); BEST Arabidopsis thaliana protein match is: PAP/OAS1 substrate-binding domain superfamily (TAIR:AT3G56320.1)
AT2G15760	*1,782*		Protein of unknown function (DUF1645); FUNCTIONS IN: calmodulin binding; INVOLVED IN: biological_process unknown; LOCATED IN: plasma membrane; EXPRESSED IN: 19 plant structures; EXPRESSED DURING: 10 growth stages; CONTAINS InterPro DOMAIN/s: Protein of unknown function DUF1645 (InterPro:IPR012442); BEST Arabidopsis thaliana protein match is: Protein of unknown function (DUF1645) (TAIR:AT2G26530.2)
AT2G22760	*1,781*		basic helix-loop-helix (bHLH) DNA-binding superfamily protein; FUNCTIONS IN: DNA binding, sequence-specific DNA binding transcription factor activity; INVOLVED IN: regulation of transcription; LOCATED IN: nucleus; EXPRESSED IN: root, cultured cell; CONTAINS InterPro DOMAIN/s: Helix-loop-helix DNA-binding domain (InterPro:IPR001092), Helix-loop-helix DNA-binding (InterPro:IPR011598); BEST Arabidopsis thaliana protein match is: basic helix-loop-helix (bHLH) DNA-binding superfamily protein (TAIR:AT2G22770.1)
AT1G23070	*1,78*		Protein of unknown function (DUF300); CONTAINS InterPro DOMAIN/s: Protein of unknown function DUF300 (InterPro:IPR005178); BEST Arabidopsis thaliana protein match is: Protein of unknown function (DUF300) (TAIR:AT4G38360.2)
AT4G38970	*1,78*	FRUCTOSE-BISPHOSPHATE ALDOLASE 2 (FBA2)	Protein is tyrosine-phosphorylated and its phosphorylation state is modulated in response to ABA in Arabidopsis thaliana seeds.
AT1G50450	*1,779*		Saccharopine dehydrogenase ; FUNCTIONS IN: binding, catalytic activity; INVOLVED IN: metabolic process; LOCATED IN: chloroplast; EXPRESSED IN: 21 plant structures; EXPRESSED DURING: 13 growth stages; CONTAINS InterPro DOMAIN/s: Saccharopine dehydrogenase / Homospermidine synthase (InterPro:IPR005097), NAD(P)-binding domain (InterPro:IPR016040)
AT4G37770	*1,776*	1-AMINO-CYCLOPROPANE-1-CARBOXYLATE SYNTHASE 8 (ACS8)	Encodes an auxin inducible ACC synthase.
AT1G62500	*1,775*		Bifunctional inhibitor/lipid-transfer protein/seed storage 2S albumin superfamily protein; FUNCTIONS IN: lipid binding; INVOLVED IN: lipid transport; LOCATED IN: endomembrane system; EXPRESSED IN: 17 plant structures; EXPRESSED DURING: 8 growth stages; CONTAINS InterPro DOMAIN/s: Bifunctional inhibitor/plant lipid transfer protein/seed storage (InterPro:IPR016140), Plant lipid transfer protein/seed storage/trypsin-alpha amylase inhibitor (InterPro:IPR003612), Plant lipid transfer protein/hydrophobic protein, helical domain (InterPro:IPR013770); BEST Arabidopsis thaliana protein match is: Bifunctional inhibitor/lipid-transfer protein/seed storage 2S albumin superfamily protein (TAIR:AT3G22142.1)

AT5G12910	*1,773*		Histone superfamily protein; FUNCTIONS IN: DNA binding; INVOLVED IN: nucleosome assembly; LOCATED IN: nucleosome; EXPRESSED IN: shoot apex, root, inflorescence, flower, leaf; EXPRESSED DURING: seedling growth, LP.02 two leaves visible, petal differentiation and expansion stage; CONTAINS InterPro DOMAIN/s: Histone H3 (InterPro:IPR000164), Histone-fold (InterPro:IPR009072), Histone core (InterPro:IPR007125); BEST Arabidopsis thaliana protein match is: Histone superfamily protein (TAIR:AT5G10400.1)
AT4G38400	*1,77*	EXPANSIN-LIKE A2 (EXLA2)	member of EXPANSIN-LIKE. Naming convention from the Expansin Working Group (Kende et al, 2004. Plant Mol Bio)
AT5G20240	*1,77*	PISTILLATA (PI)	Floral homeotic gene encoding a MADS domain transcription factor. Required for the specification of petal and stamen identities.
AT2G43660	*1,769*		Carbohydrate-binding X8 domain superfamily protein; FUNCTIONS IN: molecular_function unknown; INVOLVED IN: biological_process unknown; LOCATED IN: endomembrane system; EXPRESSED IN: inflorescence meristem, hypocotyl, root, flower; EXPRESSED DURING: petal differentiation and expansion stage; CONTAINS InterPro DOMAIN/s: X8 (InterPro:IPR012946); BEST Arabidopsis thaliana protein match is: Carbohydrate-binding X8 domain superfamily protein (TAIR:AT2G43670.1)
AT1G24470	*1,768*	BETA-KETOACYL REDUCTASE 2 (KCR2)	Encodes one of the two Arabidopsis homologues to YBR159w encoding a S. cerevisiae beta-ketoacyl reductase (KCR), which catalyzes the first reduction during VLCFA (very long chain fatty acids, >18 carbon) elongation: KCR1 (At1g67730), KCR2 (At1g24470). Complementation of the yeast ybr159Delta mutant demonstrated that the two KCR proteins are divergent and that only AtKCR1 can restore heterologous elongase activity similar to the native yeast KCR gene.
AT4G26460	*1,768*		S-adenosyl-L-methionine-dependent methyltransferases superfamily protein; FUNCTIONS IN: methyltransferase activity; INVOLVED IN: biological_process unknown; LOCATED IN: cellular_component unknown; EXPRESSED IN: sperm cell; CONTAINS InterPro DOMAIN/s: SAM dependent carboxyl methyltransferase (InterPro:IPR005299); BEST Arabidopsis thaliana protein match is: S-adenosyl-L-methionine-dependent methyltransferases superfamily protein (TAIR:AT4G26420.2)
AT5G05390	*1,768*	LACCASE 12 (LAC12)	putative laccase, a member of laccase family of genes (17 members in Arabidopsis).
AT1G65060	*1,767*	4-COUMARATE:COA LIGASE 3 (4CL3)	encodes an isoform of 4-coumarate:CoA ligase (4CL), which is involved in the last step of the general phenylpropanoid pathway. mRNA levels are not induced in response to wounding or to fungal infection by P. parasitica. mRNA is expressed in flowers, to a lesser degree in mature leaves and siliques and marginally in seedling roots and bolting stems of mature plants. The catalytic efficiency was in the following (descending) order: p-coumaric acid, caffeic acid, ferulic acid, cinnamic acid and 5-OH-ferulic acid. At4CL3 was unable to use sinapic acid as substrate.
AT2G36270	*1,766*	ABA INSENSITIVE 5 (ABI5)	Encodes a member of the basic leucine zipper transcription factor family, involved in ABA signalling during seed maturation and germination. The Arabidopsis abscisic acid (ABA)-insensitive abi5 mutants have pleiotropic defects in ABA response, including decreased sensitivity to ABA inhibition of germination and altered expression of some ABA-regulated genes. Comparison of seed and ABA-inducible vegetative gene expression in wild-type and abi5-1 plants indicates that ABI5 regulates a subset of late embryogenesis-abundant genes during both developmental stages.
AT3G25780	*1,765*	ALLENE OXIDE CYCLASE 3 (AOC3)	Encodes allene oxide cyclase, one of the enzymes involved in jasmonic acid biosynthesis. One of four genes in Arabidopsis that encode this enzyme. mRNA expression is upregulated in senescing leaves. Note: Nomenclature for Arabidopsis allene oxide cyclase 3 (AOC3, AT3G25780) gene is based on Stenzel et al. 2003 Plant Molecular Biology 51:895-911. AOC3 (AT3G25780) is also referred to as AOC2 in He et al. 2002 Plant Physiology, 128:876-884. The mRNA is cell-to-cell mobile.
AT2G17280	*1,764*		Phosphoglycerate mutase family protein; FUNCTIONS IN: catalytic activity; INVOLVED IN: metabolic process; LOCATED IN: cellular_component unknown; EXPRESSED IN: 24 plant structures; EXPRESSED DURING: 13 growth stages; CONTAINS InterPro DOMAIN/s: Histidine phosphatase superfamily, clade-1 (InterPro:IPR013078); BEST Arabidopsis thaliana protein match is: Phosphoglycerate mutase family protein (TAIR:AT5G64460.8)
AT1G74140	*1,763*		Rhomboid-related intramembrane serine protease family protein; FUNCTIONS IN: serine-type endopeptidase activity; INVOLVED IN: biological_process unknown; LOCATED IN: integral to membrane, chloroplast; CONTAINS InterPro DOMAIN/s: Peptidase S54, rhomboid (InterPro:IPR002610); BEST Arabidopsis thaliana protein match is: Rhomboid-related intramembrane serine protease family protein (TAIR:AT1G74130.1)

AT5G11160	*1,762*	ADENINE PHOSPHORIBOSYLTRANSFERASE 5 (APT5)	adenine phosphoribosyltransferase 5 (APT5); FUNCTIONS IN: adenine phosphoribosyltransferase activity; INVOLVED IN: nucleoside metabolic process, adenine salvage, anaerobic respiration, nucleotide metabolic process; LOCATED IN: cytoplasm; EXPRESSED IN: 23 plant structures; EXPRESSED DURING: 13 growth stages; CONTAINS InterPro DOMAIN/s: Adenine phosphoribosyl transferase (InterPro:IPR005764), Phosphoribosyltransferase (InterPro:IPR000836); BEST Arabidopsis thaliana protein match is: adenine phosphoribosyl transferase 2 (TAIR:AT1G80050.1)
AT1G10960	*1,761*	FERREDOXIN 1 (FD1)	ferredoxin 1 (FD1); FUNCTIONS IN: electron carrier activity, iron-sulfur cluster binding, 2 iron, 2 sulfur cluster binding; INVOLVED IN: response to karrikin; LOCATED IN: chloroplast stroma, chloroplast; EXPRESSED IN: 22 plant structures; EXPRESSED DURING: 13 growth stages; CONTAINS InterPro DOMAIN/s: 2Fe-2S ferredoxin, iron-sulphur binding site (InterPro:IPR006058), Ferredoxin (InterPro:IPR001041), Ferredoxin [2Fe-2S], plant (InterPro:IPR010241), Beta-grasp fold, ferredoxin-type (InterPro:IPR012675); BEST Arabidopsis thaliana protein match is: 2Fe-2S ferredoxin-like superfamily protein (TAIR:AT1G60950.1)
AT1G76640	*1,76*	CALMODULIN LIKE 39 (CML39)	Calcium-binding EF-hand family protein; CONTAINS InterPro DOMAIN/s: EF-Hand 1, calcium-binding site (InterPro:IPR018247), EF-HAND 2 (InterPro:IPR018249), EF-hand-like domain (InterPro:IPR011992), Calcium-binding EF-hand (InterPro:IPR002048), EF-hand (InterPro:IPR018248); BEST Arabidopsis thaliana protein match is: calmodulin-like 38 (TAIR:AT1G76650.3)
AT2G38240	*1,759*		2-oxoglutarate (2OG) and Fe(II)-dependent oxygenase superfamily protein; FUNCTIONS IN: oxidoreductase activity; INVOLVED IN: response to salt stress; EXPRESSED IN: 11 plant structures; EXPRESSED DURING: 6 growth stages; CONTAINS InterPro DOMAIN/s: Oxoglutarate/iron-dependent oxygenase (InterPro:IPR005123); BEST Arabidopsis thaliana protein match is: 2-oxoglutarate (2OG) and Fe(II)-dependent oxygenase superfamily protein (TAIR:AT5G05600.1)
AT1G77110	*1,758*	PIN-FORMED 6 (PIN6)	Rate-limiting factor in saturable efflux of auxins. PINs are directly involved of in catalyzing cellular auxin efflux.
AT2G45940	*1,757*		Protein of unknown function (DUF295); CONTAINS InterPro DOMAIN/s: Protein of unknown function DUF295 (InterPro:IPR005174); BEST Arabidopsis thaliana protein match is: Protein of unknown function (DUF295) (TAIR:AT4G16080.1)
AT3G21780	*1,757*	UDP-GLUCOSYL TRANSFERASE 71B6 (UGT71B6)	Encodes a protein with UDP-glucosyl transferase activity that was shown to preferentially glucosylates abscisic acid (ABA), and not its catabolites. Moreover, UGT71B6 was shown to have a strict preference for the naturally-occurring ABA enantiomer, (+)-ABA, and not its 'unnatural' relative, (-)-ABA. This is in contrast to the other identified UGT genes catalyzing the glucosylation of ABA which were shown to accept both stereoisomers as substrates.
AT5G65990	*1,756*	(ATAVT3)	Transmembrane amino acid transporter family protein; CONTAINS InterPro DOMAIN/s: Amino acid transporter, transmembrane (InterPro:IPR013057); BEST Arabidopsis thaliana protein match is: Transmembrane amino acid transporter family protein (TAIR:AT2G42005.1)
AT1G31920	*1,755*		Tetratricopeptide repeat (TPR)-like superfamily protein; CONTAINS InterPro DOMAIN/s: Pentatricopeptide repeat (InterPro:IPR002885); BEST Arabidopsis thaliana protein match is: Tetratricopeptide repeat (TPR)-like superfamily protein (TAIR:AT5G66520.1)
AT4G21940	*1,754*	CALCIUM-DEPENDENT PROTEIN KINASE 15 (CPK15)	member of Calcium Dependent Protein Kinase
AT5G67180	*1,754*	TARGET OF EARLY ACTIVATION TAGGED (EAT) 3 (TOE3)	target of early activation tagged (EAT) 3 (TOE3); CONTAINS InterPro DOMAIN/s: DNA-binding, integrase-type (InterPro:IPR016177), Pathogenesis-related transcriptional factor/ERF, DNA-binding (InterPro:IPR001471); BEST Arabidopsis thaliana protein match is: Integrase-type DNA-binding superfamily protein (TAIR:AT4G36920.2)
AT3G16770	*1,752*	ETHYLENE-RESPONSIVE ELEMENT BINDING PROTEIN (EBP)	Encodes a member of the ERF (ethylene response factor) subfamily B-2 of the plant specific ERF/AP2 transcription factor family (RAP2.3). The protein contains one AP2 domain. There are 5 members in this subfamily including RAP2.2 AND RAP2.12.It is localized to the nucleus and acts as a transcriptional activator through the GCC-box. It has been identified as a suppressor of Bax-induced cell death by functional screening in yeast and can also suppress Bax-induced cell death in tobacco plants. Overexpression of this gene in tobacco BY-2 cells confers resistance to H2O2 and heat stresses. Overexpression in Arabidopsis causes upregulation of PDF1.2 and GST6. It is part of the ethylene signaling pathway and is predicted to act downstream of EIN2 and CTR1, but not under EIN3. The mRNA is cell-to-cell mobile.

AT1G28480	*1,748*	(GRX480)	Encodes GRX480, a member of the glutaredoxin family that regulates protein redox state. GRX480 interacts with TGA factors and suppresses JA-responsive PDF1.2 transcription. GRX480 transcription is SA-inducible and requires NPR1. Maybe involved in SA/JA cross-talk.
AT3G58790	*1,748*	GALACTURONOSYLTRANSFERASE 15 (GAUT15)	Encodes a protein with putative galacturonosyltransferase activity.
AT1G79180	*1,744*	MYB DOMAIN PROTEIN 63 (MYB63)	Member of the R2R3 factor gene family.
AT1G49990	*1,742*		F-box family protein; CONTAINS InterPro DOMAIN/s: F-box domain, cyclin-like (InterPro:IPR001810), F-box domain, Skp2-like (InterPro:IPR022364); BEST Arabidopsis thaliana protein match is: F-box family protein (TAIR:AT4G22430.1)
AT2G18720	*1,742*		Translation elongation factor EF1A/initiation factor IF2gamma family protein; FUNCTIONS IN: serine-type peptidase activity, translation factor activity, nucleic acid binding, GTP binding, GTPase activity; LOCATED IN: integral to membrane; EXPRESSED IN: male gametophyte; CONTAINS InterPro DOMAIN/s: Translation elongation factor EFTu/EF1A, domain 2 (InterPro:IPR004161), Initiation factor eIF2 gamma, C-terminal (InterPro:IPR015256), Translation elongation factor EF1A/initiation factor IF2gamma, C-terminal (InterPro:IPR009001), Protein synthesis factor, GTP-binding (InterPro:IPR000795), Peptidase S26A, signal peptidase I, serine active site (InterPro:IPR019756), Translation elongation/initiation factor/Ribosomal, beta-barrel (InterPro:IPR009000); BEST Arabidopsis thaliana protein match is: Translation elongation factor EF1A/initiation factor IF2gamma family protein (TAIR:AT4G18330.2)
AT1G07010	*1,74*	SHEWENELLA-LIKE PROTEIN PHOSPHATASE 1 (SLP1)	Calcineurin-like metallo-phosphoesterase superfamily protein; BEST Arabidopsis thaliana protein match is: Calcineurin-like metallo-phosphoesterase superfamily protein (TAIR:AT1G18480.1)
AT5G26270	*1,74*		unknown protein
AT5G59580	*1,74*	UDP-GLUCOSYL TRANSFERASE 76E1 (UGT76E1)	UDP-glucosyl transferase 76E1 (UGT76E1); FUNCTIONS IN: quercetin 3-O-glucosyltransferase activity, UDP-glycosyltransferase activity, quercetin 7-O-glucosyltransferase activity; INVOLVED IN: metabolic process; CONTAINS InterPro DOMAIN/s: UDP-glucuronosyl/UDP-glucosyltransferase (InterPro:IPR002213); BEST Arabidopsis thaliana protein match is: UDP-glucosyl transferase 76E2 (TAIR:AT5G59590.1)
AT2G41190	*1,739*		Transmembrane amino acid transporter family protein; FUNCTIONS IN: amino acid transmembrane transporter activity; INVOLVED IN: amino acid transport; LOCATED IN: plasma membrane, membrane; EXPRESSED IN: 24 plant structures; EXPRESSED DURING: 13 growth stages; CONTAINS InterPro DOMAIN/s: Amino acid transporter, transmembrane (InterPro:IPR013057); BEST Arabidopsis thaliana protein match is: Transmembrane amino acid transporter family protein (TAIR:AT2G39130.1)
AT5G53870	*1,739*	EARLY NODULIN-LIKE PROTEIN 1 (ENODL1)	early nodulin-like protein 1 (ENODL1); FUNCTIONS IN: electron carrier activity, copper ion binding; LOCATED IN: plasma membrane, anchored to membrane; EXPRESSED IN: 22 plant structures; EXPRESSED DURING: 9 growth stages; CONTAINS InterPro DOMAIN/s: Plastocyanin-like (InterPro:IPR003245), Cupredoxin (InterPro:IPR008972); BEST Arabidopsis thaliana protein match is: early nodulin-like protein 2 (TAIR:AT4G27520.1)
AT1G11370	*1,737*		Pectin lyase-like superfamily protein; FUNCTIONS IN: pectinesterase activity; INVOLVED IN: cell wall modification; LOCATED IN: endomembrane system, cell wall, plant-type cell wall; EXPRESSED IN: flower; CONTAINS InterPro DOMAIN/s: Pectinesterase, active site (InterPro:IPR018040), Pectin lyase fold/virulence factor (InterPro:IPR011050), Pectinesterase, catalytic (InterPro:IPR000070), Pectin lyase fold (InterPro:IPR012334); BEST Arabidopsis thaliana protein match is: methylesterase PCR A (TAIR:AT1G11580.1)
AT2G01990	*1,737*		unknown protein; BEST Arabidopsis thaliana protein match is: unknown protein (TAIR:AT1G14630.1)
AT2G38250	*1,737*		Homeodomain-like superfamily protein; FUNCTIONS IN: sequence-specific DNA binding transcription factor activity; INVOLVED IN: regulation of transcription; LOCATED IN: nucleolus; EXPRESSED IN: stamen; EXPRESSED DURING: 4 anthesis, petal differentiation and expansion stage; CONTAINS InterPro DOMAIN/s: SANT, DNA-binding (InterPro:IPR001005), MYB-like (InterPro:IPR017877); BEST Arabidopsis thaliana protein match is: Homeodomain-like superfamily protein (TAIR:AT5G01380.1)
AT1G69530	*1,736*	EXPANSIN A1 (EXPA1)	Member of Alpha-Expansin Gene Family. Naming convention from the Expansin Working Group (Kende et al, Plant Mol Bio). Involved in the formation of nematode-induced syncytia in roots of Arabidopsis thaliana.

AT1G54930	*1,735*		GRF zinc finger / Zinc knuckle protein; FUNCTIONS IN: zinc ion binding, nucleic acid binding; INVOLVED IN: biological_process unknown; CONTAINS InterPro DOMAIN/s: Zinc finger, CCHC-type (InterPro:IPR001878), Zinc finger, GRF-type (InterPro:IPR010666); BEST Arabidopsis thaliana protein match is: GRF zinc finger / Zinc knuckle protein (TAIR:AT5G13920.1)
AT5G07330	*1,734*		unknown protein; FUNCTIONS IN: molecular_function unknown; INVOLVED IN: biological_process unknown; LOCATED IN: mitochondrion; BEST Arabidopsis thaliana protein match is: unknown protein (TAIR:AT1G63060.1)
AT5G16970	*1,733*	ALKENAL REDUCTASE (AER)	encodes a 2-alkenal reductase (EC 1.3.1.74), plays a key role in the detoxification of reactive carbonyls
AT2G18540	*1,732*		RmlC-like cupins superfamily protein; FUNCTIONS IN: nutrient reservoir activity; INVOLVED IN: biological_process unknown; LOCATED IN: endomembrane system; CONTAINS InterPro DOMAIN/s: Cupin, RmlC-type (InterPro:IPR011051), Cupin 1 (InterPro:IPR006045), RmlC-like jelly roll fold (InterPro:IPR014710); BEST Arabidopsis thaliana protein match is: RmlC-like cupins superfamily protein (TAIR:AT4G36700.1)
AT4G21930	*1,732*		Protein of unknown function, DUF584; CONTAINS InterPro DOMAIN/s: Protein of unknown function DUF584 (InterPro:IPR007608); BEST Arabidopsis thaliana protein match is: Protein of unknown function, DUF584 (TAIR:AT1G61930.1)
AT2G26750	*1,731*		alpha/beta-Hydrolases superfamily protein; FUNCTIONS IN: epoxide hydrolase activity, catalytic activity; INVOLVED IN: biological_process unknown; LOCATED IN: cellular_component unknown; EXPRESSED IN: cultured cell; CONTAINS InterPro DOMAIN/s: Epoxide hydrolase-like (InterPro:IPR000639), Alpha/beta hydrolase fold-1 (InterPro:IPR000073); BEST Arabidopsis thaliana protein match is: soluble epoxide hydrolase (TAIR:AT2G26740.1)
AT1G74890	*1,728*	RESPONSE REGULATOR 15 (ARR15)	Encodes a nuclear response regulator that acts as a negative regulator in cytokinin-mediated signal transduction. Transcript accumulates in leaves and roots in response to cytokinin treatment.
AT1G52030	*1,727*	MYROSINASE-BINDING PROTEIN 2 (MBP2)	Similar to myrosinase binding proteins which may be involved in metabolizing glucosinolates and forming defense compounds to protect against herbivory. Also similar to lectins and other agglutinating factors. Expressed only in flowers.
AT5G39100	*1,724*	GERMIN-LIKE PROTEIN 6 (GLP6)	germin-like protein (GLP6)
AT4G01080	*1,723*	TRICHOME BIREFRINGENCE-LIKE 26 (TBL26)	Encodes a member of the TBL (TRICHOME BIREFRINGENCE-LIKE) gene family containing a plant-specific DUF231 (domain of unknown function) domain. TBL gene family has 46 members, two of which (TBR/AT5G06700 and TBL3/AT5G01360) have been shown to be involved in the synthesis and deposition of secondary wall cellulose, presumably by influencing the esterification state of pectic polymers. A nomenclature for this gene family has been proposed (Volker Bischoff & Wolf Scheible, 2010, personal communication).
AT5G14370	*1,722*		CCT motif family protein; LOCATED IN: chloroplast; EXPRESSED IN: 21 plant structures; EXPRESSED DURING: 13 growth stages; CONTAINS InterPro DOMAIN/s: CCT domain (InterPro:IPR010402); BEST Arabidopsis thaliana protein match is: CCT motif family protein (TAIR:AT4G25990.1)
AT4G18280	*1,719*		glycine-rich cell wall protein-related
AT5G53820	*1,717*		Late embryogenesis abundant protein (LEA) family protein; BEST Arabidopsis thaliana protein match is: Late embryogenesis abundant protein (LEA) family protein (TAIR:AT5G38760.1)
AT2G07687	*1,715*		Cytochrome c oxidase, subunit III; FUNCTIONS IN: cytochrome-c oxidase activity; INVOLVED IN: mitochondrial electron transport, cytochrome c to oxygen; LOCATED IN: membrane; CONTAINS InterPro DOMAIN/s: Cytochrome c oxidase, subunit III (InterPro:IPR000298); BEST Arabidopsis thaliana protein match is: cytochrome c oxidase subunit 3 (TAIR:ATMG00730.1)
AT4G37780	*1,715*	MYB DOMAIN PROTEIN 87 (MYB87)	encoded by the Myb-like transcription factor MYB87, regulates axillary meristem formation, expressed throughout the plant. Member of the R2R3 factor gene family.
AT1G62560	*1,714*	FLAVIN-MONOOXYGENASE GLUCOSINOLATE S-OXYGENASE 3 (FMO GS-OX3)	belongs to the flavin-monooxygenase (FMO) family, encodes a glucosinolate S-oxygenase that catalyzes the conversion of methylthioalkyl glucosinolates to methylsulfinylalkyl glucosinolates The mRNA is cell-to-cell mobile.
AT4G31780	*1,713*	MONOGALACTOSYL DIACYLGLYCEROL SYNTHASE 1 (MGD1)	Encodes an A-type monogalactosyldiacylglycerol (MGDG) synthase. It represents the isoform responsible for the bulk of MGDG synthesis in Arabidopsis.

AT5G47130	*1,71*		Bax inhibitor-1 family protein; INVOLVED IN: negative regulation of apoptosis, anti-apoptosis; LOCATED IN: integral to membrane; EXPRESSED IN: 6 plant structures; EXPRESSED DURING: 4 anthesis; CONTAINS InterPro DOMAIN/s: Inhibitor of apoptosis-promoting Bax1 related (InterPro:IPR006214), Bax inhibitor 1, conserved site (InterPro:IPR006213); BEST Arabidopsis thaliana protein match is: BAX inhibitor 1 (TAIR:AT5G47120.1)
AT1G29395	*1,708*	COLD REGULATED 314 INNER MEMBRANE 1 (COR413IM1)	Integral membrane protein in the inner envelope of chloroplasts. Provide freezing tolerance. Expression is induced by short-term cold-treatment, water deprivation, and abscisic acid treatment.
AT2G39850	*1,708*		Subtilisin-like serine endopeptidase family protein; FUNCTIONS IN: identical protein binding, serine-type endopeptidase activity; INVOLVED IN: proteolysis, negative regulation of catalytic activity; LOCATED IN: plant-type cell wall; EXPRESSED IN: 20 plant structures; EXPRESSED DURING: 10 growth stages; CONTAINS InterPro DOMAIN/s: Peptidase S8/S53, subtilisin/kexin/sedolisin (InterPro:IPR000209), Proteinase inhibitor I9, subtilisin propeptide (InterPro:IPR010259), Peptidase S8, subtilisin-related (InterPro:IPR015500), Peptidase S8/S53, subtilisin, active site (InterPro:IPR022398); BEST Arabidopsis thaliana protein match is: Subtilase family protein (TAIR:AT3G46850.1)
AT3G01440	*1,708*	PHOTOSYNTHETIC NDH SUBCOMPLEX L 3 (PnsL3)	Encodes a subunit of the NAD(P)H complex located in the chloroplast thylakoid lumen.
AT1G63960	*1,705*		Copper transport protein family; BEST Arabidopsis thaliana protein match is: Heavy metal transport/detoxification superfamily protein (TAIR:AT1G63950.1)
AT4G02420	*1,704*	L-TYPE LECTIN RECEPTOR KINASE IV.4 (LECRK-IV.4)	Concanavalin A-like lectin protein kinase family protein; FUNCTIONS IN: kinase activity; INVOLVED IN: protein amino acid phosphorylation; LOCATED IN: endomembrane system; EXPRESSED IN: 21 plant structures; EXPRESSED DURING: 13 growth stages; CONTAINS InterPro DOMAIN/s: Legume lectin, beta chain (InterPro:IPR001220), Protein kinase, ATP binding site (InterPro:IPR017441), Serine/threonine-protein kinase-like domain (InterPro:IPR017442), Concanavalin A-like lectin/glucanase, subgroup (InterPro:IPR013320), Protein kinase-like domain (InterPro:IPR011009), Serine/threonine-protein kinase, active site (InterPro:IPR008271), Protein kinase, catalytic domain (InterPro:IPR000719), Concanavalin A-like lectin/glucanase (InterPro:IPR008985); BEST Arabidopsis thaliana protein match is: Concanavalin A-like lectin protein kinase family protein (TAIR:AT4G02410.1)
AT3G46090	*1,701*	(ZAT7)	ZAT7; FUNCTIONS IN: sequence-specific DNA binding transcription factor activity, zinc ion binding, nucleic acid binding; INVOLVED IN: response to chitin, regulation of transcription; LOCATED IN: intracellular; CONTAINS InterPro DOMAIN/s: Zinc finger, C2H2-like (InterPro:IPR015880), Zinc finger, C2H2-type (InterPro:IPR007087); BEST Arabidopsis thaliana protein match is: C2H2-type zinc finger family protein (TAIR:AT3G46080.1)
AT5G19100	*1,693*		Eukaryotic aspartyl protease family protein; FUNCTIONS IN: aspartic-type endopeptidase activity; INVOLVED IN: proteolysis; LOCATED IN: endomembrane system; EXPRESSED IN: root; CONTAINS InterPro DOMAIN/s: Peptidase aspartic (InterPro:IPR021109), Peptidase aspartic, catalytic (InterPro:IPR009007), Peptidase A1 (InterPro:IPR001461); BEST Arabidopsis thaliana protein match is: Eukaryotic aspartyl protease family protein (TAIR:AT1G03220.1)
AT4G11470	*1,692*	CYSTEINE-RICH RLK (RECEPTOR-LIKE PROTEIN KINASE) 31 (CRK31)	Encodes a cysteine-rich receptor-like protein kinase.
AT1G19960	*1,691*		BEST Arabidopsis thaliana protein match is: transmembrane receptors (TAIR:AT2G32140.1)
AT2G32400	*1,691*	GLUTAMATE RECEPTOR 5 (GLR5)	Glr5
AT4G21326	*1,691*	SUBTILASE 3.12 (SBT3.12)	subtilase 3.12 (SBT3.12); FUNCTIONS IN: identical protein binding, serine-type endopeptidase activity; INVOLVED IN: proteolysis, negative regulation of catalytic activity; LOCATED IN: endomembrane system; EXPRESSED IN: 17 plant structures; EXPRESSED DURING: 8 growth stages; CONTAINS InterPro DOMAIN/s: Peptidase S8/S53, subtilisin/kexin/sedolisin (InterPro:IPR000209), Peptidase S8, subtilisin-related (InterPro:IPR015500), Peptidase S8/S53, subtilisin, active site (InterPro:IPR022398), Proteinase inhibitor I9, subtilisin propeptide (InterPro:IPR010259); BEST Arabidopsis thaliana protein match is: Subtilase family protein (TAIR:AT4G10550.1)

AT5G54170	*1,691*		Polyketide cyclase/dehydrase and lipid transport superfamily protein; CONTAINS InterPro DOMAIN/s: Lipid-binding START (InterPro:IPR002913); BEST Arabidopsis thaliana protein match is: Polyketide cyclase/dehydrase and lipid transport superfamily protein (TAIR:AT1G64720.1)
AT1G29090	*1,688*		Cysteine proteinases superfamily protein; FUNCTIONS IN: cysteine-type endopeptidase activity, cysteine-type peptidase activity; INVOLVED IN: proteolysis; LOCATED IN: endomembrane system; EXPRESSED IN: sperm cell; CONTAINS InterPro DOMAIN/s: Peptidase C1A, papain (InterPro:IPR013128), Proteinase inhibitor I29, cathepsin propeptide (InterPro:IPR013201), Peptidase C1A, papain C-terminal (InterPro:IPR000668), Peptidase, cysteine peptidase active site (InterPro:IPR000169); BEST Arabidopsis thaliana protein match is: Cysteine proteinases superfamily protein (TAIR:AT2G34080.1)
AT3G23240	*1,688*	ETHYLENE RESPONSE FACTOR 1 (ERF1)	encodes a member of the ERF (ethylene response factor) subfamily B-3 of ERF/AP2 transcription factor family (ERF1). The protein contains one AP2 domain. There are 18 members in this subfamily including ATERF-1, ATERF-2, AND ATERF-5. EREBP like protein that binds GCC box of ethylene regulated promoters such as basic chitinases. Constitutive expression of ERF1 phenocopies ethylene over production. Involved in ethylene signaling cascade,downstream of EIN2 and EIN3.
AT4G25020	*1,688*		D111/G-patch domain-containing protein; FUNCTIONS IN: nucleic acid binding; INVOLVED IN: biological_process unknown; LOCATED IN: intracellular; EXPRESSED IN: 16 plant structures; EXPRESSED DURING: 8 growth stages; CONTAINS InterPro DOMAIN/s: D111/G-patch (InterPro:IPR000467), KOW (InterPro:IPR005824); BEST Arabidopsis thaliana protein match is: D111/G-patch domain-containing protein (TAIR:AT1G33520.1)
AT1G66830	*1,687*		Leucine-rich repeat protein kinase family protein; FUNCTIONS IN: protein serine/threonine kinase activity, kinase activity, ATP binding; INVOLVED IN: transmembrane receptor protein tyrosine kinase signaling pathway, protein amino acid phosphorylation; LOCATED IN: plasma membrane; EXPRESSED IN: stem, hypocotyl, sepal, leaf; EXPRESSED DURING: LP.04 four leaves visible, 4 anthesis; CONTAINS InterPro DOMAIN/s: Protein kinase, catalytic domain (InterPro:IPR000719), Leucine-rich repeat-containing N-terminal domain, type 2 (InterPro:IPR013210), Leucine-rich repeat (InterPro:IPR001611), Serine-threonine/tyrosine-protein kinase (InterPro:IPR001245), Protein kinase-like domain (InterPro:IPR011009); BEST Arabidopsis thaliana protein match is: Leucine-rich repeat protein kinase family protein (TAIR:AT2G01210.1)
AT1G74950	*1,684*	(TIFY10B)	TIFY10B; CONTAINS InterPro DOMAIN/s: Tify (InterPro:IPR010399), CCT domain-like (InterPro:IPR018467); BEST Arabidopsis thaliana protein match is: jasmonate-zim-domain protein 1 (TAIR:AT1G19180.1)
AT5G39760	*1,683*	HOMEOBOX PROTEIN 23 (HB23)	homeobox protein 23 (HB23); FUNCTIONS IN: DNA binding, sequence-specific DNA binding transcription factor activity; INVOLVED IN: regulation of transcription; EXPRESSED IN: 21 plant structures; EXPRESSED DURING: 12 growth stages; CONTAINS InterPro DOMAIN/s: Homeobox domain, ZF-HD class (InterPro:IPR006455), ZF-HD homeobox protein, Cys/His-rich dimerisation domain (InterPro:IPR006456), Homeodomain-related (InterPro:IPR012287); BEST Arabidopsis thaliana protein match is: homeobox protein 34 (TAIR:AT3G28920.1)
AT2G45130	*1,682*	SPX DOMAIN GENE 3 (SPX3)	Expression is upregulated in the shoot of cax1/cax3 mutant.
AT1G66090	*1,679*		Disease resistance protein (TIR-NBS class); FUNCTIONS IN: transmembrane receptor activity, nucleoside-triphosphatase activity, nucleotide binding, ATP binding; INVOLVED IN: signal transduction, defense response, apoptosis, innate immune response; LOCATED IN: intrinsic to membrane, chloroplast; EXPRESSED IN: 22 plant structures; EXPRESSED DURING: 10 growth stages; CONTAINS InterPro DOMAIN/s: ATPase, AAA+ type, core (InterPro:IPR003593), NB-ARC (InterPro:IPR002182), Disease resistance protein (InterPro:IPR000767), Toll-Interleukin receptor (InterPro:IPR000157); BEST Arabidopsis thaliana protein match is: Disease resistance protein (TIR-NBS-LRR class) family (TAIR:AT1G56540.1)
AT5G16010	*1,679*		3-oxo-5-alpha-steroid 4-dehydrogenase family protein; FUNCTIONS IN: oxidoreductase activity, acting on the CH-CH group of donors, 3-oxo-5-alpha-steroid 4-dehydrogenase activity; INVOLVED IN: lipid metabolic process; LOCATED IN: chloroplast, chloroplast envelope; EXPRESSED IN: 23 plant structures; EXPRESSED DURING: 15 growth stages; CONTAINS InterPro DOMAIN/s: 3-oxo-5-alpha-steroid 4-dehydrogenase, C-terminal (InterPro:IPR001104); BEST Arabidopsis thaliana protein match is: 3-oxo-5-alpha-steroid 4-dehydrogenase family protein (TAIR:AT3G55360.1)

AT5G23850	*1,676*		FUNCTIONS IN: molecular_function unknown; INVOLVED IN: biological_process unknown; LOCATED IN: cellular_component unknown; EXPRESSED IN: 23 plant structures; EXPRESSED DURING: 13 growth stages; CONTAINS InterPro DOMAIN/s: Lipopolysaccharide-modifying protein (InterPro:IPR006598), Protein of unknown function DUF821, CAP10-like (InterPro:IPR008539); BEST Arabidopsis thaliana protein match is: Arabidopsis thaliana protein of unknown function (DUF821) (TAIR:AT3G48980.1)
AT5G40040	*1,673*		60S acidic ribosomal protein family; FUNCTIONS IN: structural constituent of ribosome; INVOLVED IN: translational elongation; LOCATED IN: cytosolic ribosome, ribosome; EXPRESSED IN: 7 plant structures; EXPRESSED DURING: 4 anthesis, F mature embryo stage, petal differentiation and expansion stage, D bilateral stage; CONTAINS InterPro DOMAIN/s: Ribosomal protein 60S (InterPro:IPR001813); BEST Arabidopsis thaliana protein match is: 60S acidic ribosomal protein family (TAIR:AT3G28500.1)
AT5G43570	*1,673*		Predicted to encode a PR (pathogenesis-related) peptide that belongs to the PR-6 proteinase inhibitor family. Six putative PR-6-type protein encoding genes are found in Arabidopsis: At2g38900, At2g38870, At5g43570, At5g43580, At3g50020 and At3g46860.
AT1G64260	*1,671*		MuDR family transposase; FUNCTIONS IN: zinc ion binding; INVOLVED IN: biological_process unknown; LOCATED IN: cellular_component unknown; EXPRESSED IN: shoot apex, embryo, leaf whorl, flower, seed; EXPRESSED DURING: F mature embryo stage, petal differentiation and expansion stage, E expanded cotyledon stage, D bilateral stage; CONTAINS InterPro DOMAIN/s: MULE transposase, conserved domain (InterPro:IPR018289), Zinc finger, PMZ-type (InterPro:IPR006564), Zinc finger, SWIM-type (InterPro:IPR007527), Transposase, MuDR, plant (InterPro:IPR004332); BEST Arabidopsis thaliana protein match is: MuDR family transposase (TAIR:AT1G64255.1)
AT1G01060	*1,668*	LATE ELONGATED HYPOCOTYL (LHY)	LHY encodes a myb-related putative transcription factor involved in circadian rhythm along with another myb transcription factor CCA1
AT5G42290	*1,668*		transcription activator-related
AT1G30530	*1,667*	UDP-GLUCOSYL TRANSFERASE 78D1 (UGT78D1)	The At1g30530 gene encodes a UDP-rhamnose:flavonol-3-O-rhamnosyltransferase (UGT78D1) attaching a rhamnosyl residue to the 3-O-position of the flavonols kaempferol and quercetin
AT3G49620	*1,667*	DARK INDUCIBLE 11 (DIN11)	encodes a protein similar to 2-oxoacid-dependent dioxygenase. Expression is induced after 24 hours of dark treatment, in senescing leaves and treatment with exogenous photosynthesis inhibitor. Induction of gene expression was suppressed in excised leaves supplied with sugar. The authors suggest that the gene's expression pattern is responding to the level of sugar in the cell.
AT2G19910	*1,665*		RNA-dependent RNA polymerase family protein; CONTAINS InterPro DOMAIN/s: RNA-dependent RNA polymerase, eukaryotic-type (InterPro:IPR007855); BEST Arabidopsis thaliana protein match is: RNA-dependent RNA polymerase family protein (TAIR:AT2G19920.1)
AT4G39210	*1,663*	(APL3)	Encodes the large subunit of ADP-Glucose Pyrophosphorylase which catalyzes the first, rate limiting step in starch biosynthesis. The large subunit plays a regulatory role whereas the small subunit (ApS) is the catalytic isoform. Four isoforms (ApL1-4) have been identified. ApL3 is the major large subunit isoform present in inflorescences, fruits and roots.
AT5G39080	*1,661*		HXXXD-type acyl-transferase family protein; FUNCTIONS IN: transferase activity, transferring acyl groups other than amino-acyl groups, transferase activity; INVOLVED IN: biological_process unknown; LOCATED IN: cellular_component unknown; EXPRESSED IN: 16 plant structures; EXPRESSED DURING: 9 growth stages; CONTAINS InterPro DOMAIN/s: Transferase (InterPro:IPR003480); BEST Arabidopsis thaliana protein match is: HXXXD-type acyl-transferase family protein (TAIR:AT5G39050.1)
AT5G43980	*1,661*	PLASMODESMATA-LOCATED PROTEIN 1 (PDLP1)	Encodes a plasmodesmal protein that affects the intercellular movement of molecules through the plasmodesmata. The cytoplasmic C-terminal portion of the protein is connected to the apoplastic N-terminal portion of the protein by a single transmembrane domain (TMD). It is transported to the plasmodesmata through the secretory pathway. PDLP1 has two DUF26 domains and a signal peptide, but the proper localization of the protein appears to depend on the TMD.

AT1G56120	*1,66*		Leucine-rich repeat transmembrane protein kinase; FUNCTIONS IN: kinase activity; INVOLVED IN: protein amino acid phosphorylation; CONTAINS InterPro DOMAIN/s: Protein kinase, ATP binding site (InterPro:IPR017441), Serine/threonine-protein kinase domain (InterPro:IPR002290), Leucine-rich repeat (InterPro:IPR001611), Serine-threonine/tyrosine-protein kinase (InterPro:IPR001245), Protein kinase-like domain (InterPro:IPR011009), Serine/threonine-protein kinase, active site (InterPro:IPR008271), Protein kinase, catalytic domain (InterPro:IPR000719), Malectin/receptor-like protein kinase (InterPro:IPR021720), Tyrosine-protein kinase, catalytic domain (InterPro:IPR020635); BEST Arabidopsis thaliana protein match is: Leucine-rich repeat transmembrane protein kinase (TAIR:AT1G56130.1)
AT3G25730	*1,66*	ETHYLENE RESPONSE DNA BINDING FACTOR 3 (EDF3)	ethylene response DNA binding factor 3 (EDF3); FUNCTIONS IN: sequence-specific DNA binding transcription factor activity; INVOLVED IN: regulation of transcription, DNA-dependent; LOCATED IN: chloroplast; EXPRESSED IN: cotyledon, hypocotyl, root, flower, seed; EXPRESSED DURING: petal differentiation and expansion stage; CONTAINS InterPro DOMAIN/s: DNA-binding, integrase-type (InterPro:IPR016177), Transcriptional factor B3 (InterPro:IPR003340), Pathogenesis-related transcriptional factor/ERF, DNA-binding (InterPro:IPR001471); BEST Arabidopsis thaliana protein match is: related to ABI3/VP1 1 (TAIR:AT1G13260.1)
AT4G30830	*1,659*		Protein of unknown function, DUF593; CONTAINS InterPro DOMAIN/s: Protein of unknown function DUF593 (InterPro:IPR007656); BEST Arabidopsis thaliana protein match is: Protein of unknown function, DUF593 (TAIR:AT2G24140.1)
AT1G71000	*1,657*		Chaperone DnaJ-domain superfamily protein; FUNCTIONS IN: heat shock protein binding; INVOLVED IN: protein folding; LOCATED IN: cellular_component unknown; EXPRESSED IN: 7 plant structures; EXPRESSED DURING: 4 anthesis, petal differentiation and expansion stage; CONTAINS InterPro DOMAIN/s: Molecular chaperone, heat shock protein, Hsp40, DnaJ (InterPro:IPR015609), Heat shock protein DnaJ, N-terminal (InterPro:IPR001623), Heat shock protein DnaJ, conserved site (InterPro:IPR018253); BEST Arabidopsis thaliana protein match is: Chaperone DnaJ-domain superfamily protein (TAIR:AT1G56300.1)
AT2G24270	*1,656*	ALDEHYDE DEHYDROGENASE 11A3 (ALDH11A3)	Encodes a protein with non-phosphorylating NADP-dependent glyceraldehyde-3-phosphate dehydrogenase activity. The activity of the enzyme was determined from leaf extracts; the enzyme has not been purified to confirm activity.
AT2G21900	*1,655*	WRKY DNA-BINDING PROTEIN 59 (WRKY59)	member of WRKY Transcription Factor; Group II-c
AT5G54060	*1,655*	UDP-GLUCOSE:FLAVONOID 3-O-GLUCOSYLTRANSFERASE (UF3GT)	Encodes a anthocyanin 3-O-glucoside: 2''-O-xylosyl-transferase involved in anthocyanin modification that converts cyanidin 3-O-glucoside to cyanidin 3-O-xylosyl(1->2)glucoside. Its preferred sugar donor is UDP-xylose.
AT5G58120	*1,652*		Disease resistance protein (TIR-NBS-LRR class) family; FUNCTIONS IN: transmembrane receptor activity, nucleoside-triphosphatase activity, nucleotide binding, ATP binding; INVOLVED IN: signal transduction, apoptosis, defense response, innate immune response; LOCATED IN: intrinsic to membrane; EXPRESSED IN: 16 plant structures; EXPRESSED DURING: 9 growth stages; CONTAINS InterPro DOMAIN/s: ATPase, AAA+ type, core (InterPro:IPR003593), NB-ARC (InterPro:IPR002182), Leucine-rich repeat (InterPro:IPR001611), Toll-Interleukin receptor (InterPro:IPR000157), Disease resistance protein (InterPro:IPR000767); BEST Arabidopsis thaliana protein match is: Disease resistance protein (TIR-NBS-LRR class) family (TAIR:AT1G63750.3)
AT5G39160	*1,651*		RmlC-like cupins superfamily protein; FUNCTIONS IN: manganese ion binding, nutrient reservoir activity; INVOLVED IN: biological_process unknown; LOCATED IN: endomembrane system, apoplast; CONTAINS InterPro DOMAIN/s: Cupin, RmlC-type (InterPro:IPR011051), Cupin 1 (InterPro:IPR006045), Germin (InterPro:IPR001929), RmlC-like jelly roll fold (InterPro:IPR014710), Germin, manganese binding site (InterPro:IPR019780); BEST Arabidopsis thaliana protein match is: germin-like protein 2 (TAIR:AT5G39190.1)
AT5G11110	*1,646*	SUCROSE PHOSPHATE SYNTHASE 2F (SPS2F)	Encodes a protein with putative sucrose-phosphate synthase activity.Involved in pollen exine formation.

AT1G17100	*1,644*	HAEM-BINDING PROTEIN 1 (HBP1)	SOUL heme-binding family protein; FUNCTIONS IN: binding; INVOLVED IN: biological_process unknown; LOCATED IN: plasma membrane, vacuole; EXPRESSED IN: 24 plant structures; EXPRESSED DURING: 14 growth stages; CONTAINS InterPro DOMAIN/s: SOUL haem-binding protein (InterPro:IPR006917); BEST Arabidopsis thaliana protein match is: SOUL heme-binding family protein (TAIR:AT1G78450.1)
AT4G38420	*1,644*	SKU5 SIMILAR 9 (sks9)	SKU5 similar 9 (sks9); FUNCTIONS IN: oxidoreductase activity, copper ion binding; INVOLVED IN: oxidation reduction; LOCATED IN: plant-type cell wall; EXPRESSED IN: 12 plant structures; EXPRESSED DURING: 9 growth stages; CONTAINS InterPro DOMAIN/s: Multicopper oxidase, type 3 (InterPro:IPR011707), Cupredoxin (InterPro:IPR008972), Multicopper oxidase, type 2 (InterPro:IPR011706), Multicopper oxidase, type 1 (InterPro:IPR001117); BEST Arabidopsis thaliana protein match is: SKU5 similar 10 (TAIR:AT4G28090.1)
AT5G42900	*1,643*	COLD REGULATED GENE 27 (COR27)	cold regulated gene 27 (COR27); BEST Arabidopsis thaliana protein match is: unknown protein (TAIR:AT4G33980.1)
AT2G07715	*1,642*		Nucleic acid-binding, OB-fold-like protein; FUNCTIONS IN: structural constituent of ribosome; INVOLVED IN: translation; LOCATED IN: mitochondrion, ribosome, intracellular, large ribosomal subunit; CONTAINS InterPro DOMAIN/s: Nucleic acid-binding, OB-fold (InterPro:IPR012340), Nucleic acid-binding, OB-fold-like (InterPro:IPR016027), Ribosomal Proteins L2, RNA binding domain (InterPro:IPR022666), Ribosomal protein L2 (InterPro:IPR002171); BEST Arabidopsis thaliana protein match is: Nucleic acid-binding, OB-fold-like protein (TAIR:ATMG00560.1)
AT1G07900	*1,641*	LOB DOMAIN-CONTAINING PROTEIN 1 (LBD1)	LOB domain-containing protein 1 (LBD1); CONTAINS InterPro DOMAIN/s: Lateral organ boundaries, LOB (InterPro:IPR004883); BEST Arabidopsis thaliana protein match is: LOB domain-containing protein 11 (TAIR:AT2G28500.1)
AT2G32200	*1,641*		unknown protein; BEST Arabidopsis thaliana protein match is: unknown protein (TAIR:AT2G32210.1)
AT2G18660	*1,639*	PLANT NATRIURETIC PEPTIDE A (PNP-A)	Encodes PNP-A (Plant Natriuretic Peptide A). PNPs are a class of systemically mobile molecules distantly related to expansins; their biological role has remained elusive. PNP-A contains a signal peptide domain and is secreted into the extracellular space. Co-expression analyses using microarray data suggest that PNP-A may function as a component of plant defence response and SAR in particular, and could be classified as a newly identified PR protein. It is stress responsive and can enhance its own expression.
AT4G36520	*1,639*		Chaperone DnaJ-domain superfamily protein; FUNCTIONS IN: heat shock protein binding; CONTAINS InterPro DOMAIN/s: Heat shock protein DnaJ, N-terminal (InterPro:IPR001623); BEST Arabidopsis thaliana protein match is: Chaperone DnaJ-domain superfamily protein (TAIR:AT4G12770.2)
AT5G04010	*1,639*		F-box family protein; CONTAINS InterPro DOMAIN/s: F-box domain, Skp2-like (InterPro:IPR022364); BEST Arabidopsis thaliana protein match is: unknown protein (TAIR:AT5G03920.1)
AT5G13790	*1,639*	AGAMOUS-LIKE 15 (AGL15)	AGL15 (AGAMOUS-Like 15) is a member of the MADS domain family of regulatory factors. Although AGL15 is preferentially expressed during embryogenesis, AGL15 is also expressed in leaf primordia, shoot apical meristems and young floral buds, suggesting that AGL15 may play a role during post-germinative development. Transgenic plants that ectopically express AGL15 show delays in the transition to flowering, perianth abscission and senescence and fruit and seed maturation. Role in embryogenesis and gibberellic acid catabolism. Targets B3 domain transcription factors that are key regulators of embryogenesis.AGL15 binds the HAE promoter in floral receptacles and represses HAE expression. AGL15 is phosphorylated in a MKK4/5 dependent manner in floral receptacles.
AT5G22520	*1,639*		unknown protein; BEST Arabidopsis thaliana protein match is: unknown protein (TAIR:AT5G22530.1)
AT5G51480	*1,639*	SKU5 SIMILAR 2 (SKS2)	SKU5 similar 2 (SKS2); FUNCTIONS IN: oxidoreductase activity, copper ion binding; INVOLVED IN: oxidation reduction; LOCATED IN: anchored to plasma membrane, plasma membrane, anchored to membrane; EXPRESSED IN: 11 plant structures; EXPRESSED DURING: M germinated pollen stage, 4 anthesis, C globular stage, petal differentiation and expansion stage, E expanded cotyledon stage; CONTAINS InterPro DOMAIN/s: Multicopper oxidase, type 3 (InterPro:IPR011707), Cupredoxin (InterPro:IPR008972), Multicopper oxidase, type 2 (InterPro:IPR011706), Multicopper oxidase, type 1 (InterPro:IPR001117); BEST Arabidopsis thaliana protein match is: SKU5 similar 1 (TAIR:AT4G25240.1)
AT1G16750	*1,638*		Protein of unknown function, DUF547; CONTAINS InterPro DOMAIN/s: Protein of unknown function DUF547 (InterPro:IPR006869); BEST Arabidopsis thaliana protein match is: Protein of unknown function, DUF547 (TAIR:AT3G13000.2)

AT1G18280	*1,638*	GLYCOSYLPHOSPHATIDYLINOSITOL-ANCHORED LIPID PROTEIN TRANSFER 3 (LTPG3)	Bifunctional inhibitor/lipid-transfer protein/seed storage 2S albumin superfamily protein; CONTAINS InterPro DOMAIN/s: Bifunctional inhibitor/plant lipid transfer protein/seed storage (InterPro:IPR016140), Plant lipid transfer protein/seed storage/trypsin-alpha amylase inhibitor (InterPro:IPR003612); BEST Arabidopsis thaliana protein match is: Bifunctional inhibitor/lipid-transfer protein/seed storage 2S albumin superfamily protein (TAIR:AT1G73560.1)
AT2G20720	*1,638*		Pentatricopeptide repeat (PPR) superfamily protein; BEST Arabidopsis thaliana protein match is: Tetratricopeptide repeat (TPR)-like superfamily protein (TAIR:AT2G20710.1)
AT4G13820	*1,637*		Leucine-rich repeat (LRR) family protein; FUNCTIONS IN: kinase activity; INVOLVED IN: signal transduction; LOCATED IN: endomembrane system; EXPRESSED IN: stem, leaf whorl, hypocotyl, root, leaf; EXPRESSED DURING: LP.06 six leaves visible; CONTAINS InterPro DOMAIN/s: Leucine-rich repeat-containing N-terminal domain, type 2 (InterPro:IPR013210), Leucine-rich repeat (InterPro:IPR001611); BEST Arabidopsis thaliana protein match is: receptor like protein 47 (TAIR:AT4G13810.1)
AT4G11720	*1,635*	HAPLESS 2 (HAP2)	Encodes HAP2 with the following predicted motifs: an N-terminal secretion signal, a single transmembrane domain and a C-terminal histidine-rich domain. HAP2 is expressed only in the haploid sperm and is required for pollen tube guidance and fertilization. Predominantly localized to sperm endoplasmic reticulum membranes. May also reside in other endomembranes, including the plasma membrane. Target promoter of the male germline-specific transcription factor DUO1.
AT4G00970	*1,634*	CYSTEINE-RICH RLK (RECEPTOR-LIKE PROTEIN KINASE) 41 (CRK41)	Encodes a cysteine-rich receptor-like protein kinase.
AT2G24700	*1,63*		Transcriptional factor B3 family protein; FUNCTIONS IN: DNA binding, sequence-specific DNA binding transcription factor activity; INVOLVED IN: regulation of transcription, DNA-dependent; LOCATED IN: cellular_component unknown; CONTAINS InterPro DOMAIN/s: Transcriptional factor B3 (InterPro:IPR003340); BEST Arabidopsis thaliana protein match is: Transcriptional factor B3 family protein (TAIR:AT4G00260.1)
AT3G53130	*1,629*	LUTEIN DEFICIENT 1 (LUT1)	Lutein-deficient 1 (LUT1) required for lutein biosynthesis, member of the xanthophyll class of carotenoids. Involved in epsilon ring hydroxylation. Maps at 67.3 cM on chromosome 3.
AT4G25780	*1,627*	(ATCAPE2)	CAP (Cysteine-rich secretory proteins, Antigen 5, and Pathogenesis-related 1 protein) superfamily protein; FUNCTIONS IN: molecular_function unknown; INVOLVED IN: biological_process unknown; LOCATED IN: endomembrane system, extracellular region; EXPRESSED IN: 14 plant structures; EXPRESSED DURING: 11 growth stages; CONTAINS InterPro DOMAIN/s: Allergen V5/Tpx-1 related, conserved site (InterPro:IPR018244), Allergen V5/Tpx-1 related (InterPro:IPR001283), Ves allergen (InterPro:IPR002413), SCP-like extracellular (InterPro:IPR014044); BEST Arabidopsis thaliana protein match is: CAP (Cysteine-rich secretory proteins, Antigen 5, and Pathogenesis-related 1 protein) superfamily protein (TAIR:AT5G57625.1)
AT2G43440	*1,626*		F-box and associated interaction domains-containing protein; CONTAINS InterPro DOMAIN/s: F-box domain, cyclin-like (InterPro:IPR001810), F-box domain, Skp2-like (InterPro:IPR022364), F-box associated domain, type 1 (InterPro:IPR006527), F-box associated interaction domain (InterPro:IPR017451); BEST Arabidopsis thaliana protein match is: F-box and associated interaction domains-containing protein (TAIR:AT2G43445.1)
AT5G65140	*1,623*	TREHALOSE-6-PHOSPHATE PHOSPHATASE J (TPPJ)	Haloacid dehalogenase-like hydrolase (HAD) superfamily protein; FUNCTIONS IN: catalytic activity, trehalose-phosphatase activity; INVOLVED IN: response to cadmium ion, trehalose biosynthetic process; EXPRESSED IN: 21 plant structures; EXPRESSED DURING: 14 growth stages; CONTAINS InterPro DOMAIN/s: HAD-superfamily hydrolase, subfamily IIB (InterPro:IPR006379), Trehalose-phosphatase (InterPro:IPR003337); BEST Arabidopsis thaliana protein match is: Haloacid dehalogenase-like hydrolase (HAD) superfamily protein (TAIR:AT5G10100.1)
AT2G01810	*1,622*		RING/FYVE/PHD zinc finger superfamily protein; FUNCTIONS IN: DNA binding, zinc ion binding; INVOLVED IN: regulation of transcription, DNA-dependent; LOCATED IN: cellular_component unknown; EXPRESSED IN: sperm cell, male gametophyte, flower, pollen tube; EXPRESSED DURING: M germinated pollen stage, 4 anthesis, petal differentiation and expansion stage; CONTAINS InterPro DOMAIN/s: Zinc finger, PHD-type, conserved site (InterPro:IPR019786), Zinc finger, PHD-type (InterPro:IPR001965), Zinc finger, FYVE/PHD-type (InterPro:IPR011011), Zinc finger, PHD-finger (InterPro:IPR019787); BEST Arabidopsis thaliana protein match is: RING/FYVE/PHD zinc finger superfamily protein (TAIR:AT1G66170.1)

AT1G52890	*1,621*	NAC DOMAIN CONTAINING PROTEIN 19 (NAC019)	encodes a NAC transcription factor whose expression is induced by drought, high salt, and abscisic acid. This gene binds to ERD1 promoter in vitro.
AT3G17820	*1,618*	GLUTAMINE SYNTHETASE 1.3 (GLN1.3)	encodes a cytosolic glutamine synthetase, the enzyme has low affinity with substrate ammonium The mRNA is cell-to-cell mobile.
AT2G45450	*1,616*	LITTLE ZIPPER 1 (ZPR1)	ZPR1, a small leucine zipper-containing protein that interacts with REV HD-ZIPIII and is involved in the establishment of leaf polarity.
AT2G46510	*1,615*	ABA-INDUCIBLE BHLH-TYPE TRANSCRIPTION FACTOR (AIB)	Encodes a nuclear localized BLH domain containing transcriptional activator involved in response to ABA. Overexpression confers enhanced ABA responsiveness while loss of function mutants are ABA sensitive.bHLH17 interacts with JAZ proteins, and functions redundantly with bHLH3, bHLH13 and bHLH14 to negatively regulate jasmonate responses.
AT4G29140	*1,615*	ACTIVATED DISEASE SUSCEPTIBILITY 1 (ADS1)	Encodes Activated Disease Susceptibility 1 (ADS1), a putative MATE (multidrug and toxic compound extrusion) transport protein that negatively regulates plant disease resistance.
AT3G11170	*1,613*	FATTY ACID DESATURASE 7 (FAD7)	Chloroplastic enzyme responsible for the synthesis of 16:3 and 18:3 fatty acids from galactolipids, sulpholipids and phosphatidylglycerol. Uses ferredoxin as electron donor. Gene expression is induced by wounding in shoot and root. The wound-response in shoot is independent of jasmonic acid mediated pathway whereas the root response is mediated by jasmonic acid. The mRNA is cell-to-cell mobile.
AT4G28490	*1,613*	HAESA (HAE)	Member of Receptor kinase-like protein family. Controls the separation step of floral organ abscission. The mRNA is cell-to-cell mobile.
AT5G24130	*1,604*		unknown protein; FUNCTIONS IN: molecular_function unknown; INVOLVED IN: biological_process unknown; LOCATED IN: mitochondrion; EXPRESSED IN: petal, leaf whorl, flower; EXPRESSED DURING: 4 anthesis, petal differentiation and expansion stage
AT1G20870	*1,6*	INCREASED DNA METHYLATION 3 (IDM3)	Encodes an anti-silencing factor that prevents gene repression and DNA hypermethylation.
AT2G30140	*1,6*	UDP-GLUCOSYL TRANSFERASE 87A2 (UGT87A2)	Encodes a putative glycosyltransferase. Regulates flowering time via FLOWERING LOCUS C.
AT4G23030	*1,599*		MATE efflux family protein; FUNCTIONS IN: antiporter activity, drug transmembrane transporter activity, transporter activity; INVOLVED IN: drug transmembrane transport, transmembrane transport; LOCATED IN: membrane; EXPRESSED IN: stem, male gametophyte, flower; EXPRESSED DURING: 4 anthesis; CONTAINS InterPro DOMAIN/s: Multi antimicrobial extrusion protein MatE (InterPro:IPR002528); BEST Arabidopsis thaliana protein match is: MATE efflux family protein (TAIR:AT5G52050.1)
AT5G65510	*1,599*	AINTEGUMENTA-LIKE 7 (AIL7)	Encodes one of three PLETHORA transcription factors required to maintain high levels of PIN1 expression at the periphery of the meristem and modulate local auxin production in the central region of the SAM which underlies phyllotactic transitions.
AT4G02320	*1,597*		Plant invertase/pectin methylesterase inhibitor superfamily; FUNCTIONS IN: enzyme inhibitor activity, pectinesterase activity; INVOLVED IN: cell wall modification; LOCATED IN: endomembrane system, cell wall, plant-type cell wall; EXPRESSED IN: flower; CONTAINS InterPro DOMAIN/s: Pectinesterase, active site (InterPro:IPR018040), Pectin lyase fold/virulence factor (InterPro:IPR011050), Pectinesterase, catalytic (InterPro:IPR000070), Pectinesterase inhibitor (InterPro:IPR006501), Pectin lyase fold (InterPro:IPR012334); BEST Arabidopsis thaliana protein match is: Plant invertase/pectin methylesterase inhibitor superfamily (TAIR:AT4G02300.1)
AT1G65240	*1,594*	(A39)	Eukaryotic aspartyl protease family protein; FUNCTIONS IN: aspartic-type endopeptidase activity; INVOLVED IN: proteolysis; LOCATED IN: anchored to plasma membrane, plasma membrane, anchored to membrane; EXPRESSED IN: 10 plant structures; EXPRESSED DURING: L mature pollen stage, M germinated pollen stage, 4 anthesis, C globular stage, petal differentiation and expansion stage; CONTAINS InterPro DOMAIN/s: Peptidase aspartic (InterPro:IPR021109), Peptidase aspartic, catalytic (InterPro:IPR009007), Peptidase A1 (InterPro:IPR001461); BEST Arabidopsis thaliana protein match is: Eukaryotic aspartyl protease family protein (TAIR:AT5G36260.1)
AT4G09600	*1,594*	GAST1 PROTEIN HOMOLOG 3 (GASA3)	One of GASA gene family which is related to a GA-stimulated transcript (GAST) from tomato.

AT1G64100	*1,593*		pentatricopeptide (PPR) repeat-containing protein; CONTAINS InterPro DOMAIN/s: Pentatricopeptide repeat (InterPro:IPR002885); BEST Arabidopsis thaliana protein match is: Tetratricopeptide repeat (TPR)-like superfamily protein (TAIR:AT1G12300.1)
AT1G60270	*1,592*	BETA GLUCOSIDASE 6 (BGLU6)	beta glucosidase 6 (BGLU6); FUNCTIONS IN: cation binding, hydrolase activity, hydrolyzing O-glycosyl compounds, catalytic activity; INVOLVED IN: carbohydrate metabolic process; LOCATED IN: endomembrane system; EXPRESSED IN: 16 plant structures; EXPRESSED DURING: 10 growth stages; CONTAINS InterPro DOMAIN/s: Glycoside hydrolase, family 1 (InterPro:IPR001360), Glycoside hydrolase, family 1, active site (InterPro:IPR018120), Glycoside hydrolase, catalytic core (InterPro:IPR017853), Glycoside hydrolase, subgroup, catalytic core (InterPro:IPR013781); BEST Arabidopsis thaliana protein match is: beta glucosidase 4 (TAIR:AT1G60090.1).
AT1G68500	*1,592*		unknown protein; BEST Arabidopsis thaliana protein match is: unknown protein (TAIR:AT1G25422.1)
AT2G07696	*1,592*		Ribosomal protein S7p/S5e family protein; FUNCTIONS IN: structural constituent of ribosome; INVOLVED IN: translation; LOCATED IN: ribosome, small ribosomal subunit, intracellular; CONTAINS InterPro DOMAIN/s: Ribosomal protein S7, bacterial-type (InterPro:IPR005717), Ribosomal protein S7 (InterPro:IPR000235); BEST Arabidopsis thaliana protein match is: mitochondrial ribosomal protein S7 (TAIR:ATMG01270.1)
AT4G10490	*1,59*	DMR6-LIKE OXYGENASE 2 (DLO2)	2-oxoglutarate (2OG) and Fe(II)-dependent oxygenase superfamily protein; FUNCTIONS IN: oxidoreductase activity, acting on paired donors, with incorporation or reduction of molecular oxygen, 2-oxoglutarate as one donor, and incorporation of one atom each of oxygen into both donors, oxidoreductase activity; INVOLVED IN: secondary metabolic process; LOCATED IN: cellular_component unknown; CONTAINS InterPro DOMAIN/s: Oxoglutarate/iron-dependent oxygenase (InterPro:IPR005123); BEST Arabidopsis thaliana protein match is: 2-oxoglutarate (2OG) and Fe(II)-dependent oxygenase superfamily protein (TAIR:AT4G10500.1)
AT5G62210	*1,589*		Embryo-specific protein 3, (ATS3); CONTAINS InterPro DOMAIN/s: Lipase/lipooxygenase, PLAT/LH2 (InterPro:IPR008976), Embryo-specific 3 (InterPro:IPR010417); BEST Arabidopsis thaliana protein match is: seed gene 3 (TAIR:AT5G07190.1)
AT5G64080	*1,589*	XYLOGEN PROTEIN 1 (XYP1)	Bifunctional inhibitor/lipid-transfer protein/seed storage 2S albumin superfamily protein; FUNCTIONS IN: lipid binding; INVOLVED IN: lipid transport; LOCATED IN: anchored to plasma membrane, anchored to membrane; EXPRESSED IN: 25 plant structures; EXPRESSED DURING: 14 growth stages; CONTAINS InterPro DOMAIN/s: Bifunctional inhibitor/plant lipid transfer protein/seed storage (InterPro:IPR016140), Plant lipid transfer protein/seed storage/trypsin-alpha amylase inhibitor (InterPro:IPR003612), Plant lipid transfer protein/Par allergen (InterPro:IPR000528), Plant lipid transfer protein/hydrophobic protein, helical domain (InterPro:IPR013770); BEST Arabidopsis thaliana protein match is: Bifunctional inhibitor/lipid-transfer protein/seed storage 2S albumin superfamily protein (TAIR:AT2G13820.1)
AT1G02700	*1,58*		unknown protein; FUNCTIONS IN: molecular_function unknown; INVOLVED IN: biological_process unknown; LOCATED IN: mitochondrion; BEST Arabidopsis thaliana protein match is: unknown protein (TAIR:AT4G02140.1)
AT4G15490	*1,579*	(UGT84A3)	Encodes a protein that might have sinapic acid:UDP-glucose glucosyltransferase activity.
AT5G01660	*1,579*		CONTAINS InterPro DOMAIN/s: Galactose oxidase/kelch, beta-propeller (InterPro:IPR011043), Kelch repeat type 1 (InterPro:IPR006652), Development/cell death domain (InterPro:IPR013989), Kelch related (InterPro:IPR013089), Kelch-type beta propeller (InterPro:IPR015915); BEST Arabidopsis thaliana protein match is: DCD (Development and Cell Death) domain protein (TAIR:AT3G11000.1)
AT5G44400	*1,578*	(ATBBE26)	FAD-binding Berberine family protein; FUNCTIONS IN: electron carrier activity, oxidoreductase activity, FAD binding, catalytic activity; LOCATED IN: cell wall; EXPRESSED IN: 18 plant structures; EXPRESSED DURING: 10 growth stages; CONTAINS InterPro DOMAIN/s: FAD-linked oxidase, FAD-binding, subdomain 2 (InterPro:IPR016168), FAD-binding, type 2 (InterPro:IPR016166), Berberine/berberine-like (InterPro:IPR012951), FAD linked oxidase, N-terminal (InterPro:IPR006094); BEST Arabidopsis thaliana protein match is: FAD-binding Berberine family protein (TAIR:AT5G44390.1)
AT5G50770	*1,577*	HYDROXYSTEROID DEHYDROGENASE 6 (HSD6)	Encodes a putative hydroxysteroid dehydrogenase (HSD). Genes that encode HSD include: At5g50600 and At5g50700 (HSD1), At3g47350(HSD2), At3g47360(HSD3), At5g50590 and At5g50690(HSD4), At5g50770(HSD6) (Plant Cell Physiology 50:1463). Two copies of HSD1 and HSD4 exist due to a gene duplication event. In Plant Physiology 145:87, At5g50690 is HSD7, At4g10020 is HSD5.

AT5G54990	*1,573*		RING/U-box superfamily protein; FUNCTIONS IN: zinc ion binding; CONTAINS InterPro DOMAIN/s: Zinc finger, RING-type (InterPro:IPR001841), Zinc finger, C3HC4 RING-type (InterPro:IPR018957); BEST Arabidopsis thaliana protein match is: RING/U-box superfamily protein (TAIR:AT4G18110.1)
AT1G65370	*1,572*		TRAF-like family protein; CONTAINS InterPro DOMAIN/s: TRAF-like (InterPro:IPR008974), MATH (InterPro:IPR002083); BEST Arabidopsis thaliana protein match is: TRAF-like family protein (TAIR:AT1G65150.2)
AT1G72100	*1,571*		late embryogenesis abundant domain-containing protein / LEA domain-containing protein; INVOLVED IN: embryo development ending in seed dormancy; LOCATED IN: endomembrane system; EXPRESSED IN: sperm cell; CONTAINS InterPro DOMAIN/s: Late embryogenesis abundant protein, group 4 (InterPro:IPR004238); BEST Arabidopsis thaliana protein match is: Late embryogenesis abundant protein (LEA) family protein (TAIR:AT1G22600.1)
AT5G62360	*1,569*		Plant invertase/pectin methylesterase inhibitor superfamily protein; FUNCTIONS IN: enzyme inhibitor activity, pectinesterase inhibitor activity, pectinesterase activity; INVOLVED IN: biological_process unknown; LOCATED IN: cellular_component unknown; EXPRESSED IN: 22 plant structures; EXPRESSED DURING: 13 growth stages; CONTAINS InterPro DOMAIN/s: Pectinesterase inhibitor (InterPro:IPR006501); BEST Arabidopsis thaliana protein match is: Plant invertase/pectin methylesterase inhibitor superfamily protein (TAIR:AT5G62350.1)
AT1G21400	*1,568*		Thiamin diphosphate-binding fold (THDP-binding) superfamily protein; FUNCTIONS IN: oxidoreductase activity, acting on the aldehyde or oxo group of donors, disulfide as acceptor, 3-methyl-2-oxobutanoate dehydrogenase (2-methylpropanoyl-transferring) activity; INVOLVED IN: metabolic process; EXPRESSED IN: guard cell; CONTAINS InterPro DOMAIN/s: Dehydrogenase, E1 component (InterPro:IPR001017); BEST Arabidopsis thaliana protein match is: Thiamin diphosphate-binding fold (THDP-binding) superfamily protein (TAIR:AT5G09300.1)
AT3G21760	*1,565*	HYPOSTATIN RESISTANCE 1 (HYR1)	Encodes HYR1, a UDP glycosyltransferase (UGT). HYR1 glucosylates hypostatin, an inhibitor of cell expansion in vivo to form a bioactive glucoside.
AT5G52050	*1,565*	DETOXIFICATION EFFLUX CARRIER 50 (DTX50)	MATE efflux family protein; FUNCTIONS IN: antiporter activity, drug transmembrane transporter activity; INVOLVED IN: drug transmembrane transport, transmembrane transport; LOCATED IN: membrane; EXPRESSED IN: 7 plant structures; EXPRESSED DURING: 7 growth stages; CONTAINS InterPro DOMAIN/s: Multi antimicrobial extrusion protein MatE (InterPro:IPR002528); BEST Arabidopsis thaliana protein match is: MATE efflux family protein (TAIR:AT4G23030.1)
AT2G21510	*1,564*		DNAJ heat shock N-terminal domain-containing protein; FUNCTIONS IN: unfolded protein binding, heat shock protein binding; INVOLVED IN: protein folding; LOCATED IN: cellular_component unknown; EXPRESSED IN: 8 plant structures; EXPRESSED DURING: 4 anthesis, petal differentiation and expansion stage; CONTAINS InterPro DOMAIN/s: Molecular chaperone, heat shock protein, Hsp40, DnaJ (InterPro:IPR015609), Heat shock protein DnaJ, N-terminal (InterPro:IPR001623), Heat shock protein DnaJ (InterPro:IPR003095), Heat shock protein DnaJ, conserved site (InterPro:IPR018253); BEST Arabidopsis thaliana protein match is: DNAJ heat shock N-terminal domain-containing protein (TAIR:AT4G39150.2)
AT2G36630	*1,564*		Sulfite exporter TauE/SafE family protein; INVOLVED IN: N-terminal protein myristoylation; LOCATED IN: endomembrane system, integral to membrane; EXPRESSED IN: 22 plant structures; EXPRESSED DURING: 15 growth stages; CONTAINS InterPro DOMAIN/s: Protein of unknown function DUF81 (InterPro:IPR002781); BEST Arabidopsis thaliana protein match is: Sulfite exporter TauE/SafE family protein (TAIR:AT2G25737.1)
AT4G28140	*1,564*		encodes a member of the DREB subfamily A-6 of ERF/AP2 transcription factor family. The protein contains one AP2 domain. There are 8 members in this subfamily including RAP2.4.
AT3G01600	*1,561*	NAC DOMAIN CONTAINING PROTEIN 44 (NAC044)	NAC domain containing protein 44 (NAC044); FUNCTIONS IN: sequence-specific DNA binding transcription factor activity; INVOLVED IN: multicellular organismal development, regulation of transcription; CONTAINS InterPro DOMAIN/s: No apical meristem (NAM) protein (InterPro:IPR003441); BEST Arabidopsis thaliana protein match is: NAC domain containing protein 85 (TAIR:AT5G14490.1)
AT1G79310	*1,555*	METACASPASE 7 (MC7)	Encodes a putative metacaspase. Arabidopsis contains three type I MCP genes (MCP1a-c) and six type II MCP genes (MCP2a?f): AtMCP1a/At5g64240, AtMCP1b/At1g02170, AtMCP1c/At4g25110, AtMCP2a/At1g79310, AtMCP2b/At1g79330, AtMCP2c/At1g79320, AtMCP2d/At1g79340, AtMCP2e/At1g16420, AtMCP2f/At5g04200.

AT5G53730	*1,553*	NDR1/HIN1-LIKE 26 (NHL26)	Late embryogenesis abundant (LEA) hydroxyproline-rich glycoprotein family; CONTAINS InterPro DOMAIN/s: Late embryogenesis abundant protein, group 2 (InterPro:IPR004864); BEST Arabidopsis thaliana protein match is: Late embryogenesis abundant (LEA) hydroxyproline-rich glycoprotein family (TAIR:AT3G44220.1)
AT1G72450	*1,55*	JASMONATE-ZIM-DOMAIN PROTEIN 6 (JAZ6)	JAZ6 transcript levels rise in response to a jasmonate stimulus and a GFP:JAZ6 fusion protein localizes to the nucleus. Application of jasmonate methyl ester to Arabidopsis roots reduces the levels of a JAZ6:GUS fusion protein, presumably by stimulating ubiquitin-proteasome-mediated degradation.
AT3G25882	*1,549*	NIM1-INTERACTING 2 (NIMIN-2)	encodes a kinase that physically interacts with NPR1/NIM1
AT4G34230	*1,547*	CINNAMYL ALCOHOL DEHYDROGENASE 5 (CAD5)	Encodes a catalytically active cinnamyl alcohol dehydrogenase which uses p-coumaryl aldehyde as a preferred substrate. It can also use sinapyl, caffeyl, coniferyl and d-hydroxyconiferyl aldehydes as substrates.
AT4G33110	*1,546*		S-adenosyl-L-methionine-dependent methyltransferases superfamily protein; FUNCTIONS IN: (S)-coclaurine-N-methyltransferase activity; INVOLVED IN: lipid biosynthetic process; LOCATED IN: plasma membrane; EXPRESSED IN: cultured cell; CONTAINS InterPro DOMAIN/s: Cyclopropane-fatty-acyl-phospholipid/mycolic acid synthase (InterPro:IPR003333); BEST Arabidopsis thaliana protein match is: S-adenosyl-L-methionine-dependent methyltransferases superfamily protein (TAIR:AT4G33120.1)
AT2G46830	*1,543*	CIRCADIAN CLOCK ASSOCIATED 1 (CCA1)	Encodes a transcriptional repressor that performs overlapping functions with LHY in a regulatory feedback loop that is closely associated with the circadian oscillator of Arabidopsis. Binds to the evening element in the promoter of TOC1 and represses TOC1 transcription. CCA1 and LHY colocalize in the nucleus and form heterodimers in vivo. CCA1 and LHY function synergistically in regulating circadian rhythms of Arabidopsis.
AT5G16290	*1,543*	VALINE-TOLERANT 1 (VAT1)	Encodes a regulatory subunit of acetohydroxy acid synthase (AHAS), the ﬁrst committed enzyme in the branched chain amino acid biosynthesis pathway.
AT1G56250	*1,541*	PHLOEM PROTEIN 2-B14 (PP2-B14)	Encodes an F-box protein that can functionally replace VirF, regulating levels of the VirE2 and VIP1 proteins via a VBF-containing SCF complex. It is thought to be involved in DNA integration and T-DNA degradation.
AT1G72490	*1,54*	DEEPER ROOTING 1 (DRO1)	unknown protein; BEST Arabidopsis thaliana protein match is: unknown protein (TAIR:AT1G17400.1)
AT5G57130	*1,538*	SMAX1-LIKE 5 (SMXL5)	Encodes a member of an eight-gene family (SMAX1 and SMAX1-like) that has weak similarity to AtHSP101, a ClpB chaperonin required for thermotolerance.
AT2G11810	*1,536*	MONOGALACTOSYLDIACYLGLYCEROL SYNTHASE TYPE C (MGDC)	MGD3 is the major enzyme for galactolipid metabolism during phosphate starvation. Does not contribute to galactolipid synthesis under P1-sufficient conditions.
AT1G51670	*1,535*	HEAT-INDUCED TAS1 TARGET 5 (HTT5)	unknown protein; BEST Arabidopsis thaliana protein match is: unknown protein (TAIR:AT1G48180.1)
AT5G58680	*1,532*		ARM repeat superfamily protein; FUNCTIONS IN: binding; INVOLVED IN: biological_process unknown; LOCATED IN: cellular_component unknown; EXPRESSED IN: stem, root; CONTAINS InterPro DOMAIN/s: Armadillo-like helical (InterPro:IPR011989), Armadillo (InterPro:IPR000225), Armadillo-type fold (InterPro:IPR016024); BEST Arabidopsis thaliana protein match is: ARM repeat superfamily protein (TAIR:AT3G01400.1)
AT1G78410	*1,531*		VQ motif-containing protein; CONTAINS InterPro DOMAIN/s: VQ (InterPro:IPR008889); BEST Arabidopsis thaliana protein match is: VQ motif-containing protein (TAIR:AT1G17147.1)
AT3G03980	*1,529*		NAD(P)-binding Rossmann-fold superfamily protein; FUNCTIONS IN: oxidoreductase activity, binding, catalytic activity; INVOLVED IN: oxidation reduction, metabolic process; EXPRESSED IN: 25 plant structures; EXPRESSED DURING: 15 growth stages; CONTAINS InterPro DOMAIN/s: Short-chain dehydrogenase/reductase, conserved site (InterPro:IPR020904), NAD(P)-binding domain (InterPro:IPR016040), Glucose/ribitol dehydrogenase (InterPro:IPR002347), Short-chain dehydrogenase/reductase SDR (InterPro:IPR002198); BEST Arabidopsis thaliana protein match is: NAD(P)-binding Rossmann-fold superfamily protein (TAIR:AT3G04000.1)

AT4G24860	*1,529*		P-loop containing nucleoside triphosphate hydrolases superfamily protein; FUNCTIONS IN: nucleoside-triphosphatase activity, nucleotide binding, ATP binding; LOCATED IN: chloroplast; EXPRESSED IN: shoot apex, leaf whorl, sperm cell, sepal, flower; EXPRESSED DURING: petal differentiation and expansion stage; CONTAINS InterPro DOMAIN/s: ATPase, AAA-type, core (InterPro:IPR003959), ATPase, AAA+ type, core (InterPro:IPR003593), ATPase, AAA-type, conserved site (InterPro:IPR003960); BEST Arabidopsis thaliana protein match is: AAA-type ATPase family protein (TAIR:AT1G62130.1)
AT3G27660	*1,528*	OLEOSIN 4 (OLEO4)	Encodes oleosin4 (Plant Cell, 2006, 18:1961), a protein found in oil bodies, involved in seed lipid accumulation. Functions in freezing tolerance of seeds. Note: also referred to as OLE3 in Plant Journal 2008, 55:798.
AT5G23000	*1,528*	MYB DOMAIN PROTEIN 37 (MYB37)	Putative homolog of the Blind gene in tomato. Together with RAX2 and RAX3 belong to the class R2R3 MYB genes; encoded by the Myb-like transcription factor MYB37, regulates axillary meristem formation. RAX1 is expressed in a small central domain within the boundary zone separating SAM and leaf primordia during early leaf primordium development and is currently the earliest spatial marker for future axillary meristems. Member of the R2R3 factor gene family.
AT3G15170	*1,527*	CUP-SHAPED COTYLEDON1 (CUC1)	Encodes a transcription factor involved in shoot apical meristem formation and auxin-mediated lateral root formation. The gene is thought not to be involved in stress responses (NaCl, auxins, ethylene). <i>Cuc</i> mutant was first recognized at the heart stage, where embryos lacking two distinct bulges of cotyledonary primordia were observed.
AT5G16080	*1,527*	CARBOXYESTERASE 17 (CXE17)	carboxyesterase 17 (CXE17); FUNCTIONS IN: hydrolase activity; INVOLVED IN: metabolic process; LOCATED IN: cellular_component unknown; EXPRESSED IN: 11 plant structures; EXPRESSED DURING: 4 anthesis, petal differentiation and expansion stage; CONTAINS InterPro DOMAIN/s: Lipase, GDXG, active site (InterPro:IPR002168), Alpha/beta hydrolase fold-3 (InterPro:IPR013094); BEST Arabidopsis thaliana protein match is: alpha/beta-Hydrolases superfamily protein (TAIR:AT1G68620.1)
AT2G38230	*1,523*	PYRIDOXINE BIOSYNTHESIS 1.1 (PDX1.1)	Encodes a protein predicted to function in tandem with PDX2 to form glutamine amidotransferase complex with involved in vitamin B6 biosynthesis. The mRNA is cell-to-cell mobile.
AT5G60260	*1,522*		unknown protein
AT1G76930	*1,521*	EXTENSIN 4 (EXT4)	Encodes an Arabidopsis extensin gene that belongs to cell-wall hydroxyproline-rich glycoproteins. The cross-link of extensins enforces cell wall strength. Transgenic plants overexpressing this gene show an increase in stem thickness.
AT5G05600	*1,519*		Encodes a protein with similarity to flavonol synthases that is involved in the detoxifcation polycyclic aromatic hydrocarbons.
AT4G24930	*1,518*		thylakoid lumenal 17.9 kDa protein, chloroplast; FUNCTIONS IN: molecular_function unknown; INVOLVED IN: biological_process unknown; LOCATED IN: thylakoid, thylakoid lumen, chloroplast thylakoid lumen, chloroplast; EXPRESSED IN: 22 plant structures; EXPRESSED DURING: 13 growth stages
AT5G09660	*1,518*	PEROXISOMAL NAD-MALATE DEHYDROGENASE 2 (PMDH2)	encodes a microbody NAD-dependent malate dehydrogenase encodes an peroxisomal NAD-malate dehydrogenase that is involved in fatty acid beta-oxidation through providing NAD to the process of converting fatty acyl CoA to acetyl CoA.
AT5G62840	*1,518*		Phosphoglycerate mutase family protein; FUNCTIONS IN: catalytic activity; INVOLVED IN: metabolic process; LOCATED IN: chloroplast; EXPRESSED IN: 20 plant structures; EXPRESSED DURING: 13 growth stages; CONTAINS InterPro DOMAIN/s: Histidine phosphatase superfamily, clade-1 (InterPro:IPR013078), Twin-arginine translocation pathway, signal sequence (InterPro:IPR006311)
AT4G04710	*1,517*	CALCIUM-DEPENDENT PROTEIN KINASE 22 (CPK22)	member of Calcium Dependent Protein Kinase
AT5G10380	*1,515*	(RING1)	Encodes a RING finger domain protein with E3 ligase activity that is localized to the lipid rafts of the plasma membrane. Expression is increased in response to fungal pathogen. May be involved in regulation of programmed cell death by facilitating degredation of regulation of PDC activators. The mRNA is cell-to-cell mobile.
AT5G53750	*1,513*		CBS domain-containing protein; CONTAINS InterPro DOMAIN/s: Cystathionine beta-synthase, core (InterPro:IPR000644); BEST Arabidopsis thaliana protein match is: Cystathionine beta-synthase (CBS) family protein (TAIR:AT4G27460.1)
AT5G25920	*1,508*		BEST Arabidopsis thaliana protein match is: Eukaryotic aspartyl protease family protein (TAIR:AT3G29750.1)
AT3G50410	*1,506*	OBF BINDING PROTEIN 1 (OBP1)	Arabidopsis Dof protein containing a single 51-amino acid zinc finger DNA-binding domain, which may play an important roles in plant growth and development.

AT5G03210	*1,505*	DBP-INTERACTING PROTEIN 2 (DIP2)	Encodes a small polypeptide contributing to resistance to potyvirus.
AT1G29980	*1,503*		INVOLVED IN: biological_process unknown; LOCATED IN: plasma membrane, anchored to membrane; EXPRESSED IN: 24 plant structures; EXPRESSED DURING: 13 growth stages; CONTAINS InterPro DOMAIN/s: Protein of unknown function DUF642 (InterPro:IPR006946), Galactose-binding domain-like (InterPro:IPR008979); BEST Arabidopsis thaliana protein match is: Protein of unknown function, DUF642 (TAIR:AT2G34510.1)
AT3G61530	*1,5*	(PANB2)	Encodes a ketopentoate hydroxymethyltransferase that appears to localize to the mitochondria. This protein is expected to play a role in pantothenate (vitamin B5) biosynthesis.
AT5G15840	*1,5*	CONSTANS (CO)	Encodes a protein showing similarities to zinc finger transcription factors, involved in regulation of flowering under long days. Acts upstream of FT and SOC1.
AT3G21750	*1,498*	UDP-GLUCOSYL TRANSFERASE 71B1 (UGT71B1)	Encodes a glucosyltransferase that can attach glucose to a number of hydroxylated phenolic compounds as well as quercetins in vitro
AT5G53880	*1,497*		unknown protein; FUNCTIONS IN: molecular_function unknown; INVOLVED IN: biological_process unknown; LOCATED IN: mitochondrion, plasma membrane; EXPRESSED IN: 23 plant structures; EXPRESSED DURING: 13 growth stages
AT1G58360	*1,489*	AMINO ACID PERMEASE 1 (AAP1)	Encodes AAP1 (amino acid permease 1), a neutral amino acid transporter expressed in seeds. Functions in amino acid uptake into embryos. The transporter also functions in acquisition of glutamate and neutral amino acids by the root.
AT3G09390	*1,489*	METALLOTHIONEIN 2A (MT2A)	metallothionein, binds to and detoxifies excess copper and other metals, limiting oxidative damage
AT3G51030	*1,488*	THIOREDOXIN H-TYPE 1 (TRX1)	encodes a cytosolic thioredoxin that reduces disulfide bridges of target proteins by the reversible formation of a disulfide bridge between two neighboring Cys residues present in the active site. Thioredoxins have been found to regulate a variety of biological reactions in prokaryotic and eukaryotic cells.
AT1G63020	*1,487*	NUCLEAR RNA POLYMERASE D1A (NRPD1A)	Encodes one of two alternative largest subunits of a putative plant-specific RNA polymerase IV (aka RNA polymerase D). Required for posttranscriptional gene silencing.
AT5G51920	*1,487*		Pyridoxal phosphate (PLP)-dependent transferases superfamily protein; FUNCTIONS IN: pyridoxal phosphate binding, catalytic activity; INVOLVED IN: metabolic process; LOCATED IN: chloroplast; CONTAINS InterPro DOMAIN/s: Pyridoxal phosphate-dependent transferase, major domain (InterPro:IPR015424), Aminotransferase, class V/Cysteine desulfurase (InterPro:IPR000192), Pyridoxal phosphate-dependent transferase, major region, subdomain 1 (InterPro:IPR015421); BEST Arabidopsis thaliana protein match is: unknown protein (TAIR:AT4G22980.1)
AT5G11880	*1,485*		Pyridoxal-dependent decarboxylase family protein; FUNCTIONS IN: diaminopimelate decarboxylase activity; INVOLVED IN: lysine biosynthetic process via diaminopimelate; LOCATED IN: chloroplast; EXPRESSED IN: guard cell, cultured cell; CONTAINS InterPro DOMAIN/s: Alanine racemase/group IV decarboxylase, C-terminal (InterPro:IPR009006), Ornithine/DAP/Arg decarboxylase (InterPro:IPR000183), Orn/DAP/Arg decarboxylase 2, N-terminal (InterPro:IPR022644), Orn/DAP/Arg decarboxylase 2, C-terminal (InterPro:IPR022643), Diaminopimelate decarboxylase (InterPro:IPR002986), Orn/DAP/Arg decarboxylase 2, conserved site (InterPro:IPR022657), Orn/DAP/Arg decarboxylase 2, pyridoxal-phosphate binding site (InterPro:IPR022653); BEST Arabidopsis thaliana protein match is: Pyridoxal-dependent decarboxylase family protein (TAIR:AT3G14390.1)
AT1G20120	*1,483*		GDSL-motif esterase/acyltransferase/lipase. Enzyme group with broad substrate specificity that may catalyze acyltransfer or hydrolase reactions with lipid and non-lipid substrates.
AT4G33270	*1,482*	CELL DIVISION CYCLE 20.1 (CDC20.1)	Encodes a CDC20 protein that interacts with APC subunits, components of the mitochondrial checkpoint complex and mitotic cyclin substrates and is indispensable for normal plant development and fertility.
AT1G09420	*1,481*	GLUCOSE-6-PHOSPHATE DEHYDROGENASE 4 (G6PD4)	Encodes a protein similar to glucose-6-phosphate dehydrogenase but, based on amino acid differences in the active site and lack of activity, does not encode a functional G6PDH. The amino acid sequence for the consensus sequence of the G6PDH active site (DHYLGKE) differs in three places in this protein. gc exon splice site at 20574 is based on protein alignment, and is not confirmed experimentally.
AT1G69310	*1,48*	WRKY DNA-BINDING PROTEIN 57 (WRKY57)	Encodes WRKY57, a member of the WRKY Transcription Factor. Activation of WRKY57 confers drought tolerance.

AT4G31620	*1,48*	REPRODUCTIVE MERISTEM 36 (REM36)	Transcriptional factor B3 family protein; FUNCTIONS IN: DNA binding, sequence-specific DNA binding transcription factor activity; INVOLVED IN: regulation of transcription, DNA-dependent; LOCATED IN: cellular_component unknown; CONTAINS InterPro DOMAIN/s: Transcriptional factor B3 (InterPro:IPR003340); BEST Arabidopsis thaliana protein match is: Transcriptional factor B3 family protein (TAIR:AT4G31615.1)
AT5G20190	*1,479*		Tetratricopeptide repeat (TPR)-like superfamily protein; FUNCTIONS IN: binding; INVOLVED IN: biological_process unknown; LOCATED IN: chloroplast; CONTAINS InterPro DOMAIN/s: Tetratricopeptide-like helical (InterPro:IPR011990), Tetratricopeptide repeat-containing (InterPro:IPR013026); BEST Arabidopsis thaliana protein match is: Tetratricopeptide repeat (TPR)-like superfamily protein (TAIR:AT1G80130.1)
AT1G80160	*1,478*	GLYOXYLASE I 7 (GLYI7)	Lactoylglutathione lyase / glyoxalase I family protein; CONTAINS InterPro DOMAIN/s: Glyoxalase/bleomycin resistance protein/dioxygenase (InterPro:IPR004360); BEST Arabidopsis thaliana protein match is: Lactoylglutathione lyase / glyoxalase I family protein (TAIR:AT1G15380.2)
AT4G36430	*1,476*		Peroxidase superfamily protein; FUNCTIONS IN: peroxidase activity, heme binding; INVOLVED IN: response to other organism; LOCATED IN: cell wall; EXPRESSED IN: 8 plant structures; EXPRESSED DURING: 4 anthesis, C globular stage, 4 leaf senescence stage; CONTAINS InterPro DOMAIN/s: Haem peroxidase (InterPro:IPR010255), Plant peroxidase (InterPro:IPR000823), Peroxidases heam-ligand binding site (InterPro:IPR019793), Haem peroxidase, plant/fungal/bacterial (InterPro:IPR002016), Peroxidase, active site (InterPro:IPR019794); BEST Arabidopsis thaliana protein match is: Peroxidase superfamily protein (TAIR:AT2G18150.1)
AT4G26520	*1,475*	FRUCTOSE-BISPHOSPHATE ALDOLASE 7 (FBA7)	Aldolase superfamily protein; CONTAINS InterPro DOMAIN/s: Aldolase-type TIM barrel (InterPro:IPR013785), Fructose-bisphosphate aldolase, class-I (InterPro:IPR000741); BEST Arabidopsis thaliana protein match is: Aldolase superfamily protein (TAIR:AT4G26530.2)
AT5G10300	*1,475*	METHYL ESTERASE 5 (MES5)	Encodes a protein with R-selective hydroxynitrile lyase activity. It has similarity to the SABP2 methyl salicylate esterase from tobacco. This protein does not act on methyl IAA, methyl JA, MeSA, MeGA4, or MEGA9 in vitro.
AT5G04620	*1,474*	BIOTIN F (BIOF)	The cDNA encoding 7-keto-8-aminopelargonic acid (KAPA) synthase, the first committed enzyme of the biotin synthesis pathway has been cloned and its molecular function confirmed (functional complementation of an E. coli mutant). The subcellular localization of the enzyme (cytosol) proves that the biotin biosynthesis in plants takes place in different compartments which differs from the biosynthetic route found in microorganisms.
AT1G56240	*1,473*	PHLOEM PROTEIN 2-B13 (PP2-B13)	phloem protein 2-B13 (PP2-B13); FUNCTIONS IN: carbohydrate binding; LOCATED IN: endomembrane system; CONTAINS InterPro DOMAIN/s: F-box domain, cyclin-like (InterPro:IPR001810), F-box domain, Skp2-like (InterPro:IPR022364); BEST Arabidopsis thaliana protein match is: phloem protein 2-B14 (TAIR:AT1G56250.1)
AT5G49690	*1,473*		UDP-Glycosyltransferase superfamily protein; FUNCTIONS IN: transferase activity, transferring glycosyl groups; INVOLVED IN: metabolic process; CONTAINS InterPro DOMAIN/s: UDP-glucuronosyl/UDP-glucosyltransferase (InterPro:IPR002213); BEST Arabidopsis thaliana protein match is: UDP-Glycosyltransferase superfamily protein (TAIR:AT5G65550.1)
AT2G20825	*1,472*	ULTRAPETALA 2 (ULT2)	ULTRAPETALA 2 (ULT2); FUNCTIONS IN: DNA binding; INVOLVED IN: biological_process unknown; LOCATED IN: cytosol, nucleus; EXPRESSED IN: 7 plant structures; EXPRESSED DURING: 6 growth stages; CONTAINS InterPro DOMAIN/s: Developmental regulator, ULTRAPETALA (InterPro:IPR020533), SAND domain (InterPro:IPR000770); BEST Arabidopsis thaliana protein match is: Developmental regulator, ULTRAPETALA (TAIR:AT4G28190.1)
AT5G49280	*1,472*		hydroxyproline-rich glycoprotein family protein

AT2G34650	*1,471*	PINOID (PID)	Encodes a protein serine/threonine kinase that may act as a positive regulator of cellular auxin efflux, as a a binary switch for PIN polarity, and as a negative regulator of auxin signaling. Recessive mutants exhibit similar phenotypes as pin-formed mutants in flowers and inflorescence but distinct phenotypes in cotyledons and leaves. Expressed in the vascular tissue proximal to root and shoot meristems, shoot apex, and embryos. Expression is induced by auxin. Overexpression of the gene results in phenotypes in the root and shoot similar to those found in auxin-insensitive mutants. The protein physically interacts with TCH3 (TOUCH3) and PID-BINDING PROTEIN 1 (PBP1), a previously uncharacterized protein containing putative EF-hand calcium-binding motifs. Acts together with ENP (ENHANCER OF PINOID) to instruct precursor cells to elaborate cotyledons in the transition stage embryo. Interacts with PDK1. PID autophosphorylation is required for the ability of PID to phosphorylate an exogenous substrate. PID activation loop is required for PDK1-dependent PID phosphorylation and requires the PIF domain. Negative regulator of root hair growth. PID kinase activity is critical for the inhibition of root hair growth and for maintaining the proper subcellular localization of PID.
AT2G15080	*1,469*	RECEPTOR LIKE PROTEIN 19 (RLP19)	receptor like protein 19 (RLP19); FUNCTIONS IN: kinase activity; INVOLVED IN: signal transduction, defense response; LOCATED IN: endomembrane system; EXPRESSED IN: 10 plant structures; EXPRESSED DURING: 9 growth stages; CONTAINS InterPro DOMAIN/s: Leucine-rich repeat-containing N-terminal domain, type 2 (InterPro:IPR013210), Leucine-rich repeat (InterPro:IPR001611); BEST Arabidopsis thaliana protein match is: receptor like protein 53 (TAIR:AT5G27060.1)
AT3G59010	*1,469*	PECTIN METHYLESTERASE 61 (PME61)	Encodes PME35, a pectin methylesterase. PME35-mediated demethylesterification of the primary cell wall regulates the mechanical strength of the supporting tissue.
AT3G01660	*1,467*		S-adenosyl-L-methionine-dependent methyltransferases superfamily protein; FUNCTIONS IN: methyltransferase activity; INVOLVED IN: metabolic process; LOCATED IN: cellular_component unknown; EXPRESSED IN: 21 plant structures; EXPRESSED DURING: 13 growth stages; CONTAINS InterPro DOMAIN/s: Methyltransferase type 11 (InterPro:IPR013216); BEST Arabidopsis thaliana protein match is: S-adenosyl-L-methionine-dependent methyltransferases superfamily protein (TAIR:AT4G29590.1)
AT4G00380	*1,467*	FACTOR OF DNA METHYLATION 2 (FDM2)	Belongs to a subgroup of SGS3-like proteins that act redundantly in RNA-directed DNA methylation: AT1G15910 (FDM1), AT4G00380 (FDM2), AT3G12550 (FDM3), AT1G13790 (FDM4), AT1G80790 (FDM5).
AT5G57240	*1,467*	OSBP(OXYSTEROL BINDING PROTEIN)-RELATED PROTEIN 4C (ORP4C)	OSBP(oxysterol binding protein)-related protein 4C (ORP4C); FUNCTIONS IN: oxysterol binding; INVOLVED IN: steroid metabolic process; LOCATED IN: cellular_component unknown; EXPRESSED IN: 9 plant structures; EXPRESSED DURING: L mature pollen stage, M germinated pollen stage, 4 anthesis, petal differentiation and expansion stage; CONTAINS InterPro DOMAIN/s: Oxysterol-binding protein, conserved site (InterPro:IPR018494), Oxysterol-binding protein (InterPro:IPR000648); BEST Arabidopsis thaliana protein match is: OSBP(oxysterol binding protein)-related protein 4B (TAIR:AT4G25850.1)
AT3G23120	*1,465*	RECEPTOR LIKE PROTEIN 38 (RLP38)	receptor like protein 38 (RLP38); FUNCTIONS IN: kinase activity; INVOLVED IN: signal transduction, defense response; LOCATED IN: endomembrane system; CONTAINS InterPro DOMAIN/s: Leucine-rich repeat-containing N-terminal domain, type 2 (InterPro:IPR013210), Leucine-rich repeat (InterPro:IPR001611); BEST Arabidopsis thaliana protein match is: receptor like protein 37 (TAIR:AT3G23110.1)
AT2G42170	*1,464*		Actin family protein; FUNCTIONS IN: structural constituent of cytoskeleton, ATP binding; INVOLVED IN: biological_process unknown; LOCATED IN: actin cytoskeleton; EXPRESSED IN: vascular bundle; CONTAINS InterPro DOMAIN/s: Actin, conserved site (InterPro:IPR004001), Actin/actin-like (InterPro:IPR004000); BEST Arabidopsis thaliana protein match is: Actin-like ATPase superfamily protein (TAIR:AT2G42100.1)
AT4G24780	*1,463*	(PLL19)	Encodes a pectate lyase involved in response to nematodes.
AT5G50750	*1,463*	REVERSIBLY GLYCOSYLATED POLYPEPTIDE 4 (RGP4)	RGP4 is a reversibly glycosylated polypeptide. Analyses using tagged RGP4 suggest that it is present in the cytosol and in association with the Golgi apparatus. Recombinant RGP4 does not have UDP-arabinose mutase activity based on an in vitro assay even though the related RGP1, RGP2, and RGP3 proteins do have activity in the same assay. RGP4 can form complexes with RGP1 and RGP2. RGP4 is expressed during seed development.

AT4G34710	*1,462*	ARGININE DECARBOXYLASE 2 (ADC2)	Encodes a arginine decarboxylase (ADC), a rate-limiting enzyme that catalyzes the first step of polyamine (PA) biosynthesis via ADC pathway in Arabidopsis thaliana. Arabidopsis genome has two ADC paralogs, ADC1 and ADC2. ADC2 is stress-inducible (osmotic stress). Double mutant analysis showed that ADC genes are essential for the production of PA, and are required for normal seed development. Overexpression causes phenotypes similar to GA-deficient plants and these plants show reduced levels of GA due to lower expression levels of AtGA20ox1, AtGA3ox3 and AtGA3ox1.
AT1G29560	*1,461*		Zinc finger C-x8-C-x5-C-x3-H type family protein; FUNCTIONS IN: zinc ion binding, nucleic acid binding; LOCATED IN: cellular_component unknown; CONTAINS InterPro DOMAIN/s: Zinc finger, CCCH-type (InterPro:IPR000571); BEST Arabidopsis thaliana protein match is: Zinc finger C-x8-C-x5-C-x3-H type family protein (TAIR:AT1G29570.1)
AT5G42370	*1,461*		Calcineurin-like metallo-phosphoesterase superfamily protein; FUNCTIONS IN: molecular_function unknown; INVOLVED IN: biological_process unknown; LOCATED IN: endomembrane system; EXPRESSED IN: 22 plant structures; EXPRESSED DURING: 13 growth stages; CONTAINS InterPro DOMAIN/s: Alkaline phosphatase D-related (InterPro:IPR018946).
AT1G28610	*1,459*		GDSL-motif esterase/acyltransferase/lipase. Enzyme group with broad substrate specificity that may catalyze acyltransfer or hydrolase reactions with lipid and non-lipid substrates.
AT2G14290	*1,457*		CONTAINS InterPro DOMAIN/s: F-box domain, cyclin-like (InterPro:IPR001810), F-box domain, Skp2-like (InterPro:IPR022364), Protein of unknown function DUF295 (InterPro:IPR005174); BEST Arabidopsis thaliana protein match is: F-box family protein with a domain of unknown function (DUF295) (TAIR:AT5G25290.1)
AT4G24420	*1,454*		RNA-binding (RRM/RBD/RNP motifs) family protein
AT5G03680	*1,454*	PETAL LOSS (PTL)	Recessive mutations are defective in organ initiation and orientation in the second whorl. This gene encodes a trihelix transcription factor whose expression is limited to margins of floral and vegetative organs. Overexpression and double mutant analyses suggest that this gene is involved in limiting lateral growth of organs.
AT3G22420	*1,453*	WITH NO LYSINE (K) KINASE 2 (WNK2)	Encodes a member of the WNK family (9 members in all) of protein kinases, the structural design of which is clearly distinct from those of other known protein kinases, such as receptor-like kinases and mitogen-activated protein kinases. Its transcription is under the control of circadian rhythms.
AT5G42800	*1,451*	DIHYDROFLAVONOL 4-REDUCTASE (DFR)	dihydroflavonol reductase. Catalyzes the conversion of dihydroquercetin to leucocyanidin in the biosynthesis of anthocyanins. Not expressed in roots (qRT-PCR). The mRNA is cell-to-cell mobile.
AT1G17360	*1,447*		BEST Arabidopsis thaliana protein match is: COP1-interacting protein-related (TAIR:AT1G72410.1)
AT5G15850	*1,447*	CONSTANS-LIKE 1 (COL1)	Homologous to the flowering-time gene CONSTANS.
AT1G52290	*1,446*	PROLINE-RICH EXTENSIN-LIKE RECEPTOR KINASE 15 (PERK15)	Encodes a member of the proline-rich extensin-like receptor kinase (PERK) family. This family consists of 15 predicted receptor kinases (PMID: 15653807).
AT4G13345	*1,446*	MATERNAL EFFECT EMBRYO ARREST 55 (MEE55)	maternal effect embryo arrest 55 (MEE55); INVOLVED IN: embryo development ending in seed dormancy; LOCATED IN: membrane; EXPRESSED IN: 14 plant structures; EXPRESSED DURING: 7 growth stages; CONTAINS InterPro DOMAIN/s: TMS membrane protein/tumour differentially expressed protein (InterPro:IPR005016); BEST Arabidopsis thaliana protein match is: Serinc-domain containing serine and sphingolipid biosynthesis protein (TAIR:AT3G24460.1)
AT5G55930	*1,446*	OLIGOPEPTIDE TRANSPORTER 1 (OPT1)	oligopeptide transporter
AT1G48100	*1,445*		Pectin lyase-like superfamily protein; FUNCTIONS IN: polygalacturonase activity; INVOLVED IN: carbohydrate metabolic process; EXPRESSED IN: 19 plant structures; EXPRESSED DURING: 10 growth stages; CONTAINS InterPro DOMAIN/s: Pectin lyase fold/virulence factor (InterPro:IPR011050), Pectin lyase fold (InterPro:IPR012334), Glycoside hydrolase, family 28 (InterPro:IPR000743), Parallel beta-helix repeat (InterPro:IPR006626); BEST Arabidopsis thaliana protein match is: Pectin lyase-like superfamily protein (TAIR:AT1G02460.1)
AT2G02060	*1,443*		Homeodomain-like superfamily protein; CONTAINS InterPro DOMAIN/s: Homeodomain-like (InterPro:IPR009057), Myb, DNA-binding (InterPro:IPR014778), HTH transcriptional regulator, Myb-type, DNA-binding (InterPro:IPR017930), Myb-like DNA-binding domain, SHAQKYF class (InterPro:IPR006447), Homeodomain-related (InterPro:IPR012287); BEST Arabidopsis thaliana protein match is: Homeodomain-like superfamily protein (TAIR:AT1G14600.1)

AT2G35760	*1,44*	CASP-LIKE PROTEIN 2B2 (CASPL2B2)	Uncharacterised protein family (UPF0497); CONTAINS InterPro DOMAIN/s: Uncharacterised protein family UPF0497, trans-membrane plant (InterPro:IPR006702), Uncharacterised protein family UPF0497, trans-membrane plant subgroup (InterPro:IPR006459); BEST Arabidopsis thaliana protein match is: Uncharacterised protein family (UPF0497) (TAIR:AT4G16442.1)
AT1G42970	*1,439*	GLYCERALDEHYDE-3-PHOSPHATE DEHYDROGENASE B SUBUNIT (GAPB)	Encodes chloroplast localized glyceraldehyde-3-phosphate dehydrogenase that can use both NADH and NADPH to reduce 1,3-diphosphate glycerate. It forms A2B2 heterotetramers with GapA forms of the GADPH enzyme. These complexes are active in the light under reducing conditions, but show reduced NADPH-dependent activity in response to oxidized thioredoxins and increased NAD(H)/NADP(H) ratios due to the formation of inactive A8B8 hexadecamers. The mRNA is cell-to-cell mobile.
AT1G66840	*1,438*	PLASTID MOVEMENT IMPAIRED 2 (PMI2)	Encodes a coiled-coil protein WEB2 (weak chloroplast movement under blue light 2, also named PMI2/plastid movement impaired 2). Involved in chloroplast avoidance movement under intermediate and high light intensities. WEB2, together with another coiled-coil protein WEB1 (AT2G26570), maintains the chloroplast photorelocation movement velocity.
AT1G30135	*1,435*	JASMONATE-ZIM-DOMAIN PROTEIN 8 (JAZ8)	jasmonate-zim-domain protein 8 (JAZ8); CONTAINS InterPro DOMAIN/s: Tify (InterPro:IPR010399), CCT domain-like (InterPro:IPR018467); BEST Arabidopsis thaliana protein match is: jasmonate-zim-domain protein 7 (TAIR:AT2G34600.1)
AT1G63630	*1,434*		Tetratricopeptide repeat (TPR)-like superfamily protein; CONTAINS InterPro DOMAIN/s: Pentatricopeptide repeat (InterPro:IPR002885); BEST Arabidopsis thaliana protein match is: Tetratricopeptide repeat (TPR)-like superfamily protein (TAIR:AT1G63230.1)
AT1G67090	*1,429*	RIBULOSE BISPHOSPHATE CARBOXYLASE SMALL CHAIN 1A (RBCS1A)	Encodes a member of the Rubisco small subunit (RBCS) multigene family: RBCS1A (At1g67090), RBCS1B (At5g38430), RBCS2B (At5g38420), and RBCS3B (At5g38410). Functions to yield sufficient Rubisco content for leaf photosynthetic capacity.
AT2G47190	*1,429*	MYB DOMAIN PROTEIN 2 (MYB2)	Encodes a MYB transcription factor that possesses an R2R3 MYB DNA binding domain and is known to regulate the expression of salt- and dehydration-responsive genes. Has been shown to bind calmodulin.
AT1G18210	*1,428*		Calcium-binding EF-hand family protein; FUNCTIONS IN: calcium ion binding; INVOLVED IN: biological_process unknown; LOCATED IN: plasma membrane, vacuole; EXPRESSED IN: 25 plant structures; EXPRESSED DURING: 13 growth stages; CONTAINS InterPro DOMAIN/s: EF-Hand 1, calcium-binding site (InterPro:IPR018247), EF-HAND 2 (InterPro:IPR018249), EF-hand-like domain (InterPro:IPR011992), Calcium-binding EF-hand (InterPro:IPR002048), EF-hand (InterPro:IPR018248); BEST Arabidopsis thaliana protein match is: EF hand calcium-binding protein family (TAIR:AT1G73630.1)
AT4G15670	*1,426*	MONOTHIOL GLUTAREDOXIN-S7 (GRXS7)	Thioredoxin superfamily protein; FUNCTIONS IN: electron carrier activity, arsenate reductase (glutaredoxin) activity, protein disulfide oxidoreductase activity; INVOLVED IN: cell redox homeostasis; LOCATED IN: cellular_component unknown; CONTAINS InterPro DOMAIN/s: Glutaredoxin-like, plant II (InterPro:IPR011905), Thioredoxin fold (InterPro:IPR012335), Glutaredoxin (InterPro:IPR002109), Glutaredoxin subgroup (InterPro:IPR014025), Thioredoxin-like fold (InterPro:IPR012336); BEST Arabidopsis thaliana protein match is: Thioredoxin superfamily protein (TAIR:AT4G15680.1)
AT5G08370	*1,424*	ALPHA-GALACTOSIDASE 2 (AGAL2)	alpha-galactosidase 2 (AGAL2); FUNCTIONS IN: alpha-galactosidase activity, hydrolase activity, hydrolyzing O-glycosyl compounds, catalytic activity; INVOLVED IN: positive regulation of flower development, leaf morphogenesis; LOCATED IN: plant-type cell wall; EXPRESSED IN: 22 plant structures; EXPRESSED DURING: 13 growth stages; CONTAINS InterPro DOMAIN/s: Aldolase-type TIM barrel (InterPro:IPR013785), Glycoside hydrolase, family 27 (InterPro:IPR002241), Glycoside hydrolase, clan GH-D (InterPro:IPR000111), Glycoside hydrolase, catalytic core (InterPro:IPR017853); BEST Arabidopsis thaliana protein match is: alpha-galactosidase 1 (TAIR:AT5G08380.1)
AT1G43910	*1,423*		P-loop containing nucleoside triphosphate hydrolases superfamily protein; FUNCTIONS IN: nucleoside-triphosphatase activity, ATPase activity, nucleotide binding, ATP binding; LOCATED IN: endomembrane system; EXPRESSED IN: 23 plant structures; EXPRESSED DURING: 13 growth stages; CONTAINS InterPro DOMAIN/s: ATPase, AAA-type, core (InterPro:IPR003959), ATPase, AAA+ type, core (InterPro:IPR003593); BEST Arabidopsis thaliana protein match is: P-loop containing nucleoside triphosphate hydrolases superfamily protein (TAIR:AT4G05380.1)
AT4G04570	*1,423*	CYSTEINE-RICH RLK (RECEPTOR-LIKE PROTEIN KINASE) 40 (CRK40)	Encodes a cysteine-rich receptor-like protein kinase. The mRNA is cell-to-cell mobile.

AT2G39705	*1,422*	ROTUNDIFOLIA LIKE 8 (RTFL8)	ROTUNDIFOLIA like 8 (RTFL8); FUNCTIONS IN: molecular_function unknown; INVOLVED IN: shoot development; LOCATED IN: chloroplast; EXPRESSED IN: 23 plant structures; EXPRESSED DURING: 13 growth stages; CONTAINS InterPro DOMAIN/s: DVL (InterPro:IPR012552); BEST Arabidopsis thaliana protein match is: ROTUNDIFOLIA like 7 (TAIR:AT3G55515.1)
AT1G74420	*1,421*	FUCOSYLTRANSFERASE 3 (FUT3)	Predicted fucosyltransferase, based on similarity to FUT1, but not functionally redundantwith FUT1.
AT2G01610	*1,421*		Plant invertase/pectin methylesterase inhibitor superfamily protein; FUNCTIONS IN: enzyme inhibitor activity, pectinesterase inhibitor activity, pectinesterase activity; INVOLVED IN: biological_process unknown; LOCATED IN: endomembrane system; EXPRESSED IN: 22 plant structures; EXPRESSED DURING: 13 growth stages; CONTAINS InterPro DOMAIN/s: Pectinesterase inhibitor (InterPro:IPR006501); BEST Arabidopsis thaliana protein match is: Plant invertase/pectin methylesterase inhibitor superfamily protein (TAIR:AT1G14890.1)
AT5G10180	*1,42*	SULFATE TRANSPORTER 2;1 (SULTR2;1)	Encodes a low-affinity sulfate transporter expressed in the root cap and central cylinder, where it is induced by sulfur starvation. Expression in the shoot vascular system is not induced by sulfur starvation.
AT2G24030	*1,419*		zinc ion binding;nucleic acid binding; FUNCTIONS IN: zinc ion binding, nucleic acid binding; INVOLVED IN: biological_process unknown; LOCATED IN: intracellular; EXPRESSED IN: cultured cell; CONTAINS InterPro DOMAIN/s: Zinc finger, C2H2-like (InterPro:IPR015880), Zinc finger, U1-type (InterPro:IPR003604), Zinc finger, double-stranded RNA binding (InterPro:IPR022755)
AT3G48420	*1,419*		Haloacid dehalogenase-like hydrolase (HAD) superfamily protein; FUNCTIONS IN: hydrolase activity, catalytic activity; INVOLVED IN: metabolic process; LOCATED IN: chloroplast, chloroplast stroma, chloroplast envelope; EXPRESSED IN: 22 plant structures; EXPRESSED DURING: 14 growth stages; CONTAINS InterPro DOMAIN/s: Haloacid dehalogenase-like hydrolase (InterPro:IPR005834), HAD-superfamily hydrolase, subfamily IA, variant 3 (InterPro:IPR006402); BEST Arabidopsis thaliana protein match is: Haloacid dehalogenase-like hydrolase (HAD) superfamily protein (TAIR:AT4G39970.1)
AT5G52720	*1,418*		Copper transport protein family; BEST Arabidopsis thaliana protein match is: Copper transport protein family (TAIR:AT5G52670.1)
AT1G10480	*1,417*	ZINC FINGER PROTEIN 5 (ZFP5)	Encodes a zinc finger protein containing only a single zinc finger that acts downstream of ZFP6 in regulating trichome development by integrating GA and cytokinin signaling.
AT1G03800	*1,416*	ERF DOMAIN PROTEIN 10 (ERF10)	encodes a member of the ERF (ethylene response factor) subfamily B-1 of ERF/AP2 transcription factor family (ATERF-10). The protein contains one AP2 domain. There are 15 members in this subfamily including ATERF-3, ATERF-4, ATERF-7, and leafy petiole.
AT4G37370	*1,416*	CYTOCHROME P450, FAMILY 81, SUBFAMILY D, POLYPEPTIDE 8 (CYP81D8)	member of CYP81D
AT5G55740	*1,416*	CHLORORESPIRATORY REDUCTION 21 (CRR21)	Encodes a member of the E+ subgroup of the PPR protein family, containing the E and E+ motifs following a tandem array of PPR motifs. It also contains an unknown motif consisting of 15 aa, which is highly conserved in some PPR proteins, including CRR4. CRR21 is involved in RNA editing of the site 2 of ndhD (ndhD-2),which encodes a subunit of the NDH complex. The RNA editing changes aa 128 from Ser to Leu. Mutants have impaired NDH complex activity.
AT5G59390	*1,415*		XH/XS domain-containing protein; CONTAINS InterPro DOMAIN/s: Domain of unknown function XS (InterPro:IPR005380), Domain of unknown function XH (InterPro:IPR005379); BEST Arabidopsis thaliana protein match is: XH/XS domain-containing protein (TAIR:AT4G01180.1)
AT3G04210	*1,414*		Disease resistance protein (TIR-NBS class); FUNCTIONS IN: transmembrane receptor activity, ATP binding; INVOLVED IN: signal transduction, apoptosis, defense response, innate immune response; LOCATED IN: intrinsic to membrane, endomembrane system; EXPRESSED IN: 18 plant structures; EXPRESSED DURING: 11 growth stages; CONTAINS InterPro DOMAIN/s: NB-ARC (InterPro:IPR002182), Disease resistance protein (InterPro:IPR000767), Toll-Interleukin receptor (InterPro:IPR000157); BEST Arabidopsis thaliana protein match is: Disease resistance protein (TIR-NBS-LRR class) family (TAIR:AT5G38340.1)
AT1G07880	*1,413*	(ATMPK13)	member of MAP Kinase

AT1G67460	*1,413*		Minichromosome maintenance (MCM2/3/5) family protein; FUNCTIONS IN: GTP binding, GTPase activity; INVOLVED IN: biological_process unknown; EXPRESSED IN: petal, hypocotyl, flower, leaf; EXPRESSED DURING: 4 anthesis, LP.10 ten leaves visible, petal differentiation and expansion stage, LP.08 eight leaves visible; CONTAINS InterPro DOMAIN/s: EngC GTPase (InterPro:IPR010914), Nucleic acid-binding, OB-fold-like (InterPro:IPR016027), GTPase EngC (InterPro:IPR004881); BEST Arabidopsis thaliana protein match is: Minichromosome maintenance (MCM2/3/5) family protein (TAIR:AT1G67440.1)
AT2G45930	*1,411*		unknown protein; FUNCTIONS IN: molecular_function unknown; INVOLVED IN: biological_process unknown; LOCATED IN: mitochondrion; BEST Arabidopsis thaliana protein match is: unknown protein (TAIR:AT4G16090.1)
AT1G29100	*1,41*		Heavy metal transport/detoxification superfamily protein ; FUNCTIONS IN: copper ion binding, metal ion binding; INVOLVED IN: copper ion transport, metal ion transport; CONTAINS InterPro DOMAIN/s: Heavy metal transport/detoxification protein (InterPro:IPR006121); BEST Arabidopsis thaliana protein match is: Heavy metal transport/detoxification superfamily protein (TAIR:AT1G06330.1)
AT3G26170	*1,41*	CYTOCHROME P450, FAMILY 71, SUBFAMILY B, POLYPEPTIDE 19 (CYP71B19)	putative cytochrome P450
AT5G02120	*1,41*	ONE HELIX PROTEIN (OHP)	Encodes a one helix protein homologous to cyanobacterial high-light inducible proteins. The protein is localized to the thylakoid membrane and its transcript is transiently induced by exposure to high light conditions. The mRNA is cell-to-cell mobile.
AT5G16990	*1,41*		molecular function has not been defined, was shown involved in oxidative stress tolerance. The mRNA is cell-to-cell mobile.
AT2G07698	*1,408*		ATPase, F1 complex, alpha subunit protein; FUNCTIONS IN: hydrogen ion transporting ATP synthase activity, rotational mechanism, poly(U) RNA binding, zinc ion binding; INVOLVED IN: proton transport, ATP metabolic process, ATP synthesis coupled proton transport; LOCATED IN: in 7 components; EXPRESSED IN: male gametophyte, juvenile leaf, cultured cell, leaf; EXPRESSED DURING: seedling growth, seed development stages; CONTAINS InterPro DOMAIN/s: ATPase, F1 complex, alpha subunit (InterPro:IPR005294), ATPase, F1/V1/A1 complex, alpha/beta subunit, C-terminal (InterPro:IPR000793), ATPase, alpha/beta subunit, nucleotide-binding domain, active site (InterPro:IPR020003), ATPase, F1/V1/A1 complex, alpha/beta subunit, N-terminal (InterPro:IPR004100), ATPase, F1/A1 complex, alpha/beta subunit, N-terminal (InterPro:IPR018118), ATPase, alpha/beta subunit, nucleotide-binding domain (InterPro:IPR000194); BEST Arabidopsis thaliana protein match is: ATP synthase subunit 1 (TAIR:ATMG01190.1)
AT1G10770	*1,406*		Encodes a putative pectin methylesterase/invertase inhibitor. Anti-sense reduction of this gene's transcript results in pollen tube growth retardation and then partial male sterility and reduced seed set.
AT1G56060	*1,403*		unknown protein; BEST Arabidopsis thaliana protein match is: unknown protein (TAIR:AT2G32210.1)
AT1G15980	*1,4*	PHOTOSYNTHETIC NDH SUBCOMPLEX B 1 (PnsB1)	encodes a novel subunit of the chloroplast NAD(P)H dehydrogenase complex, involved in cyclic electron flow around photosystem I to produce ATP.
AT3G14620	*1,4*	CYTOCHROME P450, FAMILY 72, SUBFAMILY A, POLYPEPTIDE 8 (CYP72A8)	putative cytochrome P450 The mRNA is cell-to-cell mobile.
AT1G77920	*1,399*	TGACG SEQUENCE-SPECIFIC BINDING PROTEIN 7 (TGA7)	bZIP transcription factor family protein; CONTAINS InterPro DOMAIN/s: Basic-leucine zipper (bZIP) transcription factor (InterPro:IPR004827), bZIP transcription factor, bZIP-1 (InterPro:IPR011616); BEST Arabidopsis thaliana protein match is: TGA1A-related gene 3 (TAIR:AT1G22070.1)
AT3G05400	*1,399*		Major facilitator superfamily protein; FUNCTIONS IN: carbohydrate transmembrane transporter activity, sugar:hydrogen symporter activity; INVOLVED IN: transport, transmembrane transport; LOCATED IN: integral to membrane, membrane; EXPRESSED IN: 16 plant structures; EXPRESSED DURING: 8 growth stages; CONTAINS InterPro DOMAIN/s: Sugar transporter, conserved site (InterPro:IPR005829), Major facilitator superfamily (InterPro:IPR020846), General substrate transporter (InterPro:IPR005828), Sugar/inositol transporter (InterPro:IPR003663), Major facilitator superfamily, general substrate transporter (InterPro:IPR016196); BEST Arabidopsis thaliana protein match is: Major facilitator superfamily protein (TAIR:AT5G27350.1)

AT3G21520	*1,399*	DUF679 DOMAIN MEMBRANE PROTEIN 1 (DMP1)	Encodes a protein is directly or indirectly involved in membrane fission during breakdown of the ER and the tonoplast during leaf senescence and in membrane fusion during vacuole biogenesis in roots. The mRNA is cell-to-cell mobile.
AT5G17170	*1,399*	ENHANCER OF SOS3-1 (ENH1)	enhancer of sos3-1 (ENH1); FUNCTIONS IN: electron carrier activity, metal ion binding; LOCATED IN: chloroplast thylakoid membrane, chloroplast, chloroplast envelope; EXPRESSED IN: 22 plant structures; EXPRESSED DURING: 14 growth stages; CONTAINS InterPro DOMAIN/s: Rubredoxin-type Fe(Cys)4 protein (InterPro:IPR004039), PDZ/DHR/GLGF (InterPro:IPR001478); BEST Arabidopsis thaliana protein match is: Rubredoxin-like superfamily protein (TAIR:AT5G51010.1)
AT2G18480	*1,398*		Major facilitator superfamily protein; FUNCTIONS IN: carbohydrate transmembrane transporter activity, sugar:hydrogen symporter activity; INVOLVED IN: transport, transmembrane transport; LOCATED IN: integral to membrane, membrane; EXPRESSED IN: 12 plant structures; EXPRESSED DURING: 6 growth stages; CONTAINS InterPro DOMAIN/s: Sugar transporter, conserved site (InterPro:IPR005829), Major facilitator superfamily (InterPro:IPR020846), General substrate transporter (InterPro:IPR005828), Sugar/inositol transporter (InterPro:IPR003663), Major facilitator superfamily, general substrate transporter (InterPro:IPR016196); BEST Arabidopsis thaliana protein match is: Major facilitator superfamily protein (TAIR:AT4G36670.1)
AT5G64530	*1,398*	XYLEM NAC DOMAIN 1 (XND1)	xylem NAC domain 1 (XND1); CONTAINS InterPro DOMAIN/s: No apical meristem (NAM) protein (InterPro:IPR003441); BEST Arabidopsis thaliana protein match is: NAC domain containing protein 25 (TAIR:AT1G61110.1)
AT3G47430	*1,397*	PEROXIN 11B (PEX11B)	member of the peroxin11 (PEX11) gene family, located on the peroxisome membrane, controls peroxisome proliferation. The mRNA is cell-to-cell mobile.
AT5G05580	*1,397*	FATTY ACID DESATURASE 8 (FAD8)	Encodes a temperature sensitive plastidic fatty acid desaturase.
AT1G14640	*1,396*		SWAP (Suppressor-of-White-APricot)/surp domain-containing protein; FUNCTIONS IN: RNA binding; INVOLVED IN: RNA processing; EXPRESSED IN: male gametophyte, flower, seed, pollen tube; EXPRESSED DURING: L mature pollen stage, M germinated pollen stage, 4 anthesis, E expanded cotyledon stage; CONTAINS InterPro DOMAIN/s: SWAP/Surp (InterPro:IPR000061), Pre-mRNA splicing factor PRP21 like protein (InterPro:IPR022030); BEST Arabidopsis thaliana protein match is: SWAP (Suppressor-of-White-APricot)/surp domain-containing protein / ubiquitin family protein (TAIR:AT1G14650.2)
AT4G03270	*1,396*	CYCLIN D6;1 (CYCD6;1)	Cyclin D6;1 (CYCD6;1); FUNCTIONS IN: cyclin-dependent protein kinase activity; INVOLVED IN: regulation of cell cycle; LOCATED IN: nucleus; EXPRESSED IN: 11 plant structures; EXPRESSED DURING: F mature embryo stage, 4 leaf senescence stage, petal differentiation and expansion stage, E expanded cotyledon stage, D bilateral stage; CONTAINS InterPro DOMAIN/s: Cyclin, C-terminal (InterPro:IPR004367), Cyclin D (InterPro:IPR015451), Cyclin-like (InterPro:IPR011028), Cyclin-related (InterPro:IPR013763), Cyclin, N-terminal (InterPro:IPR006671), Cyclin (InterPro:IPR006670); BEST Arabidopsis thaliana protein match is: CYCLIN D4;1 (TAIR:AT5G65420.1)
AT5G17780	*1,396*		alpha/beta-Hydrolases superfamily protein; FUNCTIONS IN: hydrolase activity; LOCATED IN: endomembrane system; EXPRESSED IN: 13 plant structures; EXPRESSED DURING: 8 growth stages; CONTAINS InterPro DOMAIN/s: Alpha/beta hydrolase fold-1 (InterPro:IPR000073); BEST Arabidopsis thaliana protein match is: alpha/beta-Hydrolases superfamily protein (TAIR:AT5G17720.1)
AT4G38050	*1,395*		Xanthine/uracil permease family protein; FUNCTIONS IN: transmembrane transporter activity; INVOLVED IN: transport, transmembrane transport; LOCATED IN: plasma membrane, membrane; EXPRESSED IN: 23 plant structures; EXPRESSED DURING: 13 growth stages; CONTAINS InterPro DOMAIN/s: Xanthine/uracil/vitamin C permease (InterPro:IPR006043); BEST Arabidopsis thaliana protein match is: nucleobase-ascorbate transporter 12 (TAIR:AT2G27810.1)
AT5G21482	*1,395*	CYTOKININ OXIDASE 7 (CKX7)	This gene used to be called AtCKX5. It encodes a protein whose sequence is similar to cytokinin oxidase/dehydrogenase, which catalyzes the degradation of cytokinins.
AT5G52600	*1,395*	MYB DOMAIN PROTEIN 82 (MYB82)	Encodes a nuclear-localized transcription activator that is a member of the R2R3 factor gene family. MYB82 and GL1 can form homodimers and heterodimers at R2R3-MYB domains. At least one of the two introns in MYB82 is essential to the protein?s trichome developmental function.

AT2G18500	*1,394*	OVATE FAMILY PROTEIN 7 (OFP7)	ovate family protein 7 (OFP7); INVOLVED IN: biological_process unknown; LOCATED IN: plasma membrane; EXPRESSED IN: shoot apex, embryo, hypocotyl, flower, seed; EXPRESSED DURING: F mature embryo stage, petal differentiation and expansion stage, E expanded cotyledon stage, D bilateral stage; CONTAINS InterPro DOMAIN/s: Protein of unknown function DUF623 (InterPro:IPR006458); BEST Arabidopsis thaliana protein match is: ovate family protein 8 (TAIR:AT5G19650.1)
AT5G38510	*1,392*		Rhomboid-related intramembrane serine protease family protein; FUNCTIONS IN: serine-type endopeptidase activity; INVOLVED IN: biological_process unknown; LOCATED IN: integral to membrane; EXPRESSED IN: 22 plant structures; EXPRESSED DURING: 13 growth stages; CONTAINS InterPro DOMAIN/s: Peptidase S54, rhomboid (InterPro:IPR002610); BEST Arabidopsis thaliana protein match is: RHOMBOID-like protein 10 (TAIR:AT1G25290.2)
AT1G12200	*1,391*	FLAVIN MONOOXYGENASE (FMO)	Putative flavin monooxygenase.
AT3G11050	*1,389*	FERRITIN 2 (FER2)	ferritin 2 (FER2); FUNCTIONS IN: oxidoreductase activity, ferric iron binding, binding, transition metal ion binding; INVOLVED IN: response to oxidative stress, cellular iron ion homeostasis, response to abscisic acid stimulus, iron ion transport; LOCATED IN: chloroplast; EXPRESSED IN: 12 plant structures; EXPRESSED DURING: 7 growth stages; CONTAINS InterPro DOMAIN/s: Ferritin, N-terminal (InterPro:IPR001519), Ferritin-related (InterPro:IPR012347), Ferritin-like (InterPro:IPR009040), Ferritin, conserved site (InterPro:IPR014034), Ferritin/ribonucleotide reductase-like (InterPro:IPR009078), Ferritin/Dps protein (InterPro:IPR008331); BEST Arabidopsis thaliana protein match is: ferritin 4 (TAIR:AT2G40300.1)
AT1G22570	*1,387*		Major facilitator superfamily protein; FUNCTIONS IN: transporter activity; INVOLVED IN: oligopeptide transport; LOCATED IN: membrane; EXPRESSED IN: 17 plant structures; EXPRESSED DURING: 10 growth stages; CONTAINS InterPro DOMAIN/s: PTR2 family proton/oligopeptide symporter, conserved site (InterPro:IPR018456), Oligopeptide transporter (InterPro:IPR000109), Major facilitator superfamily, general substrate transporter (InterPro:IPR016196); BEST Arabidopsis thaliana protein match is: Major facilitator superfamily protein (TAIR:AT1G22550.1)
AT2G40130	*1,385*	SMAX1-LIKE 8 (SMXL8)	Encodes a member of an eight-gene family (SMAX1 and SMAX1-like) that has weak similarity to AtHSP101, a ClpB chaperonin required for thermotolerance.
AT1G14600	*1,384*		Homeodomain-like superfamily protein; CONTAINS InterPro DOMAIN/s: Myb, DNA-binding (InterPro:IPR014778), Homeodomain-like (InterPro:IPR009057), Homeodomain-related (InterPro:IPR012287), HTH transcriptional regulator, Myb-type, DNA-binding (InterPro:IPR017930), Myb-like DNA-binding domain, SHAQKYF class (InterPro:IPR006447); BEST Arabidopsis thaliana protein match is: Homeodomain-like superfamily protein (TAIR:AT2G02060.1)
AT4G08150	*1,383*	KNOTTED-LIKE FROM ARABIDOPSIS THALIANA (KNAT1)	A member of class I knotted1-like homeobox gene family (together with KNAT2). Similar to the knotted1 (kn1) homeobox gene of maize. Normally expressed in the peripheral and rib zone of shoot apical meristem but not in the leaf primordia. It is also expressed in the fourth floral whorl, in the region that would become style, particularly in the cell surrounding the transmitting tissue. No expression was detected in the first three floral whorls. Expression is repressed by auxin and AS1 which results in the promotion of leaf fate.
AT3G23410	*1,382*	FATTY ALCOHOL OXIDASE 3 (FAO3)	Encodes a fatty alcohol oxidase.
AT5G54400	*1,382*		S-adenosyl-L-methionine-dependent methyltransferases superfamily protein; FUNCTIONS IN: methyltransferase activity; INVOLVED IN: metabolic process; LOCATED IN: plasma membrane; EXPRESSED IN: 18 plant structures; EXPRESSED DURING: 6 growth stages; CONTAINS InterPro DOMAIN/s: Methyltransferase type 11 (InterPro:IPR013216); BEST Arabidopsis thaliana protein match is: S-adenosyl-L-methionine-dependent methyltransferases superfamily protein (TAIR:AT3G15530.2)
AT2G07706	*1,381*		unknown protein; BEST Arabidopsis thaliana protein match is: unknown protein (TAIR:ATMG00470.1)
AT3G26830	*1,38*	PHYTOALEXIN DEFICIENT 3 (PAD3)	Mutations in pad3 are defective in biosynthesis of the indole derived phytoalexin camalexin. Encodes a cytochrome P450 enzyme that catalyzes the conversion of dihydrocamalexic acid to camalexin. The mRNA is cell-to-cell mobile.
AT4G39230	*1,38*	PHENYLCOUMARAN BENZYLIC ETHER REDUCTASE 1 (PCBER1)	encodes a protein whose sequence is similar to phenylcoumaran benzylic ether reductase (PCBER), which catalyzes NADPH-dependent reduction of 8-5' linked lignans such as dehydrodiconiferyl alcohol to give isodihydrodehydrodiconiferyl alcohol.
AT5G01370	*1,38*	ALC-INTERACTING PROTEIN 1 (ACI1)	Nuclear protein with a lysine-rich domain and a C-terminal serine-rich domain. Interacts with Alcatraz (ALC). ACI1 is mainly expressed in the vascular system. Involved in cell separation during fruit dehiscence.

AT1G09795	*1,379*	ATP PHOSPHORIBOSYL TRANSFERASE 2 (ATP-PRT2)	ATP phosphoribosyl transferase, catalyses first step of histidine biosynthesis
AT4G33040	*1,376*		Thioredoxin superfamily protein; FUNCTIONS IN: electron carrier activity, protein disulfide oxidoreductase activity; INVOLVED IN: cell redox homeostasis; LOCATED IN: cellular_component unknown; EXPRESSED IN: 23 plant structures; EXPRESSED DURING: 13 growth stages; CONTAINS InterPro DOMAIN/s: Glutaredoxin-like, plant II (InterPro:IPR011905), Thioredoxin fold (InterPro:IPR012335), Glutaredoxin (InterPro:IPR002109), Thioredoxin-like fold (InterPro:IPR012336); BEST Arabidopsis thaliana protein match is: Thioredoxin superfamily protein (TAIR:AT5G11930.1)
AT4G33930	*1,374*		Encodes a protein with 14.6% glycine residues, similar to hyphally regulated protein from Candida albicans, PIR2:S58135
AT5G39050	*1,373*	PHENOLIC GLUCOSIDE MALONYLTRANSFERASE 1 (PMAT1)	Encodes a malonyltransferase that may play a role in phenolic xenobiotic detoxification.
AT4G10270	*1,371*		Wound-responsive family protein; CONTAINS InterPro DOMAIN/s: Protein of unknown function wound-induced (InterPro:IPR022251); BEST Arabidopsis thaliana protein match is: Wound-responsive family protein (TAIR:AT4G10265.1)
AT4G24972	*1,371*	TAPETUM DETERMINANT 1 (TPD1)	Encodes a novel small protein which is similar to proteins of unknown function from other plant species. TPD1 is involved in cell specification during anther and pollen development. Identified in a screen for male steriles. Mutants lack tapetal cells and have an increased number of microsporocytes. Expressed in flower buds, leaves and young seedlings. In anthers, TPD1 is expressed throughout pollen development in parietal cells and sporocytes. Physically interacts with the LRR kinase EMS1 and that interaction results in phosphorylation of TPD1.
AT4G18050	*1,369*	ATP-BINDING CASSETTE B9 (ABCB9)	P-glycoprotein 9 (PGP9); FUNCTIONS IN: ATPase activity, coupled to transmembrane movement of substances; INVOLVED IN: transport, transmembrane transport; LOCATED IN: integral to membrane; EXPRESSED IN: 13 plant structures; EXPRESSED DURING: L mature pollen stage, M germinated pollen stage, 4 anthesis, petal differentiation and expansion stage; CONTAINS InterPro DOMAIN/s: ATPase, AAA+ type, core (InterPro:IPR003593), ABC transporter-like (InterPro:IPR003439), ABC transporter, transmembrane domain, type 1 (InterPro:IPR011527), ABC transporter integral membrane type 1 (InterPro:IPR017940), ABC transporter, transmembrane domain (InterPro:IPR001140), ABC transporter, conserved site (InterPro:IPR017871); BEST Arabidopsis thaliana protein match is: P-glycoprotein 7 (TAIR:AT5G46540.1)
AT3G48610	*1,368*	NON-SPECIFIC PHOSPHOLIPASE C6 (NPC6)	non-specific phospholipase C6 (NPC6); FUNCTIONS IN: hydrolase activity, acting on ester bonds; LOCATED IN: endomembrane system; EXPRESSED IN: 22 plant structures; EXPRESSED DURING: 13 growth stages; CONTAINS InterPro DOMAIN/s: Phosphoesterase (InterPro:IPR007312); BEST Arabidopsis thaliana protein match is: non-specific phospholipase C1 (TAIR:AT1G07230.1)
AT5G17980	*1,368*		C2 calcium/lipid-binding plant phosphoribosyltransferase family protein; LOCATED IN: endoplasmic reticulum, plasma membrane; EXPRESSED IN: 18 plant structures; EXPRESSED DURING: 7 growth stages; CONTAINS InterPro DOMAIN/s: C2 membrane targeting protein (InterPro:IPR018029), C2 calcium/lipid-binding domain, CaLB (InterPro:IPR008973), Phosphoribosyltransferase C-terminal (InterPro:IPR013583), C2 calcium-dependent membrane targeting (InterPro:IPR000008); BEST Arabidopsis thaliana protein match is: C2 calcium/lipid-binding plant phosphoribosyltransferase family protein (TAIR:AT1G74720.1)
AT5G04360	*1,365*	LIMIT DEXTRINASE (LDA)	Encodes an enzyme thought to be involved in the hydrolysis of the α-1,6 linkages during starch degradation in seed endosperm. However, a knockout mutant of Arabidopsis lacking limit dextrinase has normal rates of starch degradation in the leaf at night, indicating that more than one isoamylases might be involved in this process.
AT3G44880	*1,364*	ACCELERATED CELL DEATH 1 (ACD1)	Encodes a pheide a oxygenase (PAO). Accelerated cell death (acd1) mutants show rapid, spreading necrotic responses to both virulent and avirulent Pseudomonas syringae pv. maculicola or pv. tomato pathogens and to ethylene.
AT2G39230	*1,362*	LATERAL ORGAN JUNCTION (LOJ)	Encodes a pentatricopeptide protein (LOJ) that is specifically expressed in lateral organ junctions.
AT5G04140	*1,358*	GLUTAMATE SYNTHASE 1 (GLU1)	Encodes a gene whose sequence is similar to ferredoxin dependent glutamate synthase (Fd-GOGAT). Expression in leaves is induced by light and sucrose. Proposed to be involved in photorespiration and nitrogen assimilation. The mRNA is cell-to-cell mobile.

AT4G30090	*1,355*		FUNCTIONS IN: molecular_function unknown; INVOLVED IN: embryo development ending in seed dormancy; LOCATED IN: endomembrane system; EXPRESSED IN: 8 plant structures; EXPRESSED DURING: 4 anthesis, F mature embryo stage, petal differentiation and expansion stage, E expanded cotyledon stage, D bilateral stage; BEST Arabidopsis thaliana protein match is: myosin heavy chain-related (TAIR:AT4G31340.1)
AT4G31870	*1,353*	GLUTATHIONE PEROXIDASE 7 (GPX7)	Encodes glutathione peroxidase.
AT1G23740	*1,352*	ALKENAL/ONE OXIDOREDUCTASE (AOR)	AOR is an alkenal/one oxidoreductase that acts on compounds with unsaturated alpha,beta-carbonyls. The activity of this enzyme with a number of substrates, including acrolein and 3-buten-2-one, was demonstrated in vitro using a truncated form of the protein that lacked approximately 80 of the first amino acids. This protein appears to localize to the chloroplast where it likely helps to maintain the photosynthetic process by detoxifying reactive carbonyls formed during lipid peroxidation.
AT1G73020	*1,352*		unknown protein; CONTAINS InterPro DOMAIN/s: Protein of unknown function DUF590 (InterPro:IPR007632)
AT4G25810	*1,35*	XYLOGLUCAN ENDOTRANSGLYCOSYLASE 6 (XTR6)	xyloglucan endotransglycosylase-related protein (XTR6)
AT1G03820	*1,348*		unknown protein; FUNCTIONS IN: molecular_function unknown; INVOLVED IN: biological_process unknown; LOCATED IN: endomembrane system; EXPRESSED IN: 21 plant structures; EXPRESSED DURING: 13 growth stages
AT2G35960	*1,348*	NDR1/HIN1-LIKE 12 (NHL12)	Encodes a protein whose sequence is similar to tobacco hairpin-induced gene (HIN1) and Arabidopsis non-race specific disease resistance gene (NDR1). Expression is not altered in response to cucumber mosaic virus or spermine.
AT2G24600	*1,347*		Ankyrin repeat family protein; CONTAINS InterPro DOMAIN/s: Ankyrin repeat-containing domain (InterPro:IPR020683), Ankyrin repeat (InterPro:IPR002110); BEST Arabidopsis thaliana protein match is: Ankyrin repeat family protein (TAIR:AT1G10340.1)
AT3G02150	*1,347*	PLASTID TRANSCRIPTION FACTOR 1 (PTF1)	a chloroplast trans-acting factor of the psbD light-responsive promoter.TCP gene involved in heterochronic control of leaf differentiation.
AT2G41100	*1,345*	TOUCH 3 (TCH3)	encodes a calmodulin-like protein, with six potential calcium binding domains. Calcium binding shown by Ca(2+)-specific shift in electrophoretic mobility. Expression induced by touch and darkness. Expression may also be developmentally controlled. Expression in growing regions of roots, vascular tissue, root/shoot junctions, trichomes, branch points of the shoot, and regions of siliques and flowers. The mRNA is cell-to-cell mobile.
AT5G54770	*1,345*	(THI1)	Encodes a thiamine biosynthetic gene that has a dual function in thiamine biosynthesis and mitochondrial DNA damage tolerance. It appears to be involved in producing the thiazole portion of thiamine (vitamin B1). A crystal structure of the protein reveals that it forms a 2-ring homo-octamer. The mRNA is cell-to-cell mobile.
AT1G66480	*1,343*		Involved in chloroplast avoidance movement under intermediate and high light intensities
AT5G58330	*1,343*	NADP-DEPENDENT MALATE DEHYDROGENASE (NADP-MDH)	lactate/malate dehydrogenase family protein; FUNCTIONS IN: oxidoreductase activity, binding, malate dehydrogenase activity, catalytic activity, malate dehydrogenase (NADP+) activity; INVOLVED IN: oxidation reduction, malate metabolic process, carbohydrate metabolic process, metabolic process; LOCATED IN: in 6 components; EXPRESSED IN: 23 plant structures; EXPRESSED DURING: 14 growth stages; CONTAINS InterPro DOMAIN/s: Malate dehydrogenase, NAD/NADP (InterPro:IPR010945), Lactate/malate dehydrogenase, C-terminal (InterPro:IPR022383), Malate dehydrogenase, NADP-dependent, plants (InterPro:IPR011273), NAD(P)-binding domain (InterPro:IPR016040), Lactate/malate dehydrogenase, N-terminal (InterPro:IPR001236), Malate dehydrogenase, active site (InterPro:IPR001252), Lactate dehydrogenase/glycoside hydrolase, family 4, C-terminal (InterPro:IPR015955); BEST Arabidopsis thaliana protein match is: Lactate/malate dehydrogenase family protein (TAIR:AT5G56720.1)
AT2G01290	*1,342*	RIBOSE-5-PHOSPHATE ISOMERASE 2 (RPI2)	Cytosolic ribose-5-phosphate isomerase. Knockout mutation causes chloroplast dysfunction, late flowering and premature cell death.
AT1G66350	*1,341*	RGA-LIKE 1 (RGL1)	Negative regulator of GA responses, member of GRAS family of transcription factors. Also belongs to the DELLA proteins that restrain the cell proliferation and expansion that drives plant growth. RGL1 may be involved in reducing ROS accumulation in response to stress by up-regulating the transcription of superoxide dismutases. Rapidly degraded in response to GA. Involved in flower and fruit development.

AT4G18020	*1,34*	(APRR2)	Encodes pseudo-response regulator 2 (APRR2) that interacts with a calcium sensor (CML9).
AT4G22570	*1,339*	ADENINE PHOSPHORIBOSYL TRANSFERASE 3 (APT3)	Encodes an adenine phosphoribosyltransferase (APT; EC 2.4.2.7), which is a constitutively expressed enzyme involved in the one-step salvage of adenine to AMP. APT3 has higher affinity for zeatin, isopentenyladenine and benzyladenine than APT1 but lower Vmax than APT1.
AT4G34990	*1,339*	MYB DOMAIN PROTEIN 32 (MYB32)	Member of the R2R3 factor gene family.
AT5G51720	*1,338*	NEET GROUP PROTEIN (NEET)	Encodes a protein with biochemical, structural, and biophysical characteristics of a NEET protein. It plays a key role in plant development, senescence, reactive oxygen homeostasis, and Fe metabolism.
AT1G71360	*1,337*	SUN-DOMAIN PROTEIN 4 (SUN4)	Encodes a member of the mid-SUN subfamily of SUN-domain proteins that is localized to both the nuclear envelope and the ER. It is involved in early seed development and nuclear morphology.
AT3G04220	*1,335*		Disease resistance protein (TIR-NBS-LRR class) family; FUNCTIONS IN: transmembrane receptor activity, nucleoside-triphosphatase activity, nucleotide binding, ATP binding; INVOLVED IN: signal transduction, defense response, apoptosis, innate immune response; LOCATED IN: intrinsic to membrane; EXPRESSED IN: 21 plant structures; EXPRESSED DURING: 13 growth stages; CONTAINS InterPro DOMAIN/s: ATPase, AAA+ type, core (InterPro:IPR003593), NB-ARC (InterPro:IPR002182), Leucine-rich repeat (InterPro:IPR001611), Disease resistance protein (InterPro:IPR000767), Toll-Interleukin receptor (InterPro:IPR000157); BEST Arabidopsis thaliana protein match is: Disease resistance protein (TIR-NBS-LRR class) (TAIR:AT5G11250.1)
AT3G30210	*1,334*	MYB DOMAIN PROTEIN 121 (MYB121)	Encodes a putative transcription factor, member of the R2R3 factor gene family (MYB121).
AT5G20280	*1,334*	SUCROSE PHOSPHATE SYNTHASE 1F (SPS1F)	Encodes a protein with putative sucrose-phosphate synthase activity. When the gene was expressed in transgenic tobacco plants, a clear trend for increased SPS activity was noted.
AT3G15990	*1,333*	SULFATE TRANSPORTER 3;4 (SULTR3;4)	Encodes sulfate transporter Sultr3;4.
AT1G11330	*1,332*		S-locus lectin protein kinase family protein; FUNCTIONS IN: in 6 functions; INVOLVED IN: protein amino acid phosphorylation, recognition of pollen; LOCATED IN: plasma membrane; EXPRESSED IN: 21 plant structures; EXPRESSED DURING: 13 growth stages; CONTAINS InterPro DOMAIN/s: Curculin-like (mannose-binding) lectin (InterPro:IPR001480), Apple-like (InterPro:IPR003609), PAN-2 domain (InterPro:IPR013227), Serine/threonine-protein kinase domain (InterPro:IPR002290), Serine/threonine-protein kinase-like domain (InterPro:IPR017442), Protein kinase-like domain (InterPro:IPR011009), Serine/threonine-protein kinase, active site (InterPro:IPR008271), Protein kinase, catalytic domain (InterPro:IPR000719), S-locus glycoprotein (InterPro:IPR000858), Tyrosine-protein kinase, catalytic domain (InterPro:IPR020635); BEST Arabidopsis thaliana protein match is: S-domain-1 13 (TAIR:AT1G11350.1).
AT1G52510	*1,332*		alpha/beta-Hydrolases superfamily protein; FUNCTIONS IN: hydrolase activity, catalytic activity; LOCATED IN: chloroplast, chloroplast envelope; EXPRESSED IN: 21 plant structures; EXPRESSED DURING: 13 growth stages; CONTAINS InterPro DOMAIN/s: Epoxide hydrolase-like (InterPro:IPR000639), Alpha/beta hydrolase fold-1 (InterPro:IPR000073); BEST Arabidopsis thaliana protein match is: alpha/beta-Hydrolases superfamily protein (TAIR:AT4G12830.1)
AT1G76820	*1,332*		eukaryotic translation initiation factor 2 (eIF-2) family protein; FUNCTIONS IN: GTP binding, GTPase activity; INVOLVED IN: biological_process unknown; LOCATED IN: cellular_component unknown; EXPRESSED IN: sperm cell, male gametophyte; EXPRESSED DURING: M germinated pollen stage; CONTAINS InterPro DOMAIN/s: Small GTP-binding protein (InterPro:IPR005225), Translation elongation factor EFTu/EF1A, domain 2 (InterPro:IPR004161), Translation initiation factor 2 related (InterPro:IPR015760), Protein synthesis factor, GTP-binding (InterPro:IPR000795), Translation elongation/initiation factor/Ribosomal, beta-barrel (InterPro:IPR009000); BEST Arabidopsis thaliana protein match is: eukaryotic translation initiation factor 2 (eIF-2) family protein (TAIR:AT1G76720.1)

AT5G53830	*1,332*	MPK3/6-TARGETED VQP 3 (MVQ3)	VQ motif-containing protein; FUNCTIONS IN: molecular_function unknown; INVOLVED IN: biological_process unknown; LOCATED IN: cellular_component unknown; EXPRESSED IN: 19 plant structures; EXPRESSED DURING: 11 growth stages; CONTAINS InterPro DOMAIN/s: VQ (InterPro:IPR008889); BEST Arabidopsis thaliana protein match is: VQ motif-containing protein (TAIR:AT3G15300.1)
AT1G72030	*1,329*		Acyl-CoA N-acyltransferases (NAT) superfamily protein; FUNCTIONS IN: N-acetyltransferase activity; INVOLVED IN: metabolic process; EXPRESSED IN: 22 plant structures; EXPRESSED DURING: 13 growth stages; CONTAINS InterPro DOMAIN/s: GCN5-related N-acetyltransferase, C-terminal (InterPro:IPR022610), GCN5-related N-acetyltransferase (InterPro:IPR000182), Acyl-CoA N-acyltransferase (InterPro:IPR016181)
AT2G21770	*1,328*	CELLULOSE SYNTHASE A9 (CESA9)	cellulose synthase, related to CESA6.
AT1G72930	*1,326*	TOLL/INTERLEUKIN-1 RECEPTOR-LIKE (TIR)	Toll/interleukin-1 receptor-like protein (TIR) mRNA,
AT1G33760	*1,325*	ETHYLENE RESPONSE FACTOR022 (ERF022)	encodes a member of the DREB subfamily A-4 of ERF/AP2 transcription factor family. The protein contains one AP2 domain. There are 17 members in this subfamily including TINY.
AT1G73690	*1,325*	CYCLIN-DEPENDENT KINASE D1;1 (CDKD1;1)	cyclin dependent kinase activator CDKD;1. Nuclear localization. Involved in cell cycle regulation and cell differentiation.
AT5G02240	*1,325*		Protein is tyrosine-phosphorylated and its phosphorylation state is modulated in response to ABA in Arabidopsis thaliana seeds. The mRNA is cell-to-cell mobile.
AT5G40340	*1,325*		Tudor/PWWP/MBT superfamily protein; FUNCTIONS IN: molecular_function unknown; LOCATED IN: nucleolus; EXPRESSED IN: 24 plant structures; EXPRESSED DURING: 14 growth stages; CONTAINS InterPro DOMAIN/s: PWWP (InterPro:IPR000313); BEST Arabidopsis thaliana protein match is: Tudor/PWWP/MBT superfamily protein (TAIR:AT3G27860.1)
AT5G65900	*1,325*		DEA(D/H)-box RNA helicase family protein; FUNCTIONS IN: helicase activity, ATP-dependent helicase activity, nucleic acid binding, ATP binding; LOCATED IN: cellular_component unknown; EXPRESSED IN: 23 plant structures; EXPRESSED DURING: 13 growth stages; CONTAINS InterPro DOMAIN/s: RNA helicase, DEAD-box type, Q motif (InterPro:IPR014014), DNA/RNA helicase, DEAD/DEAH box type, N-terminal (InterPro:IPR011545), RNA helicase, ATP-dependent, DEAD-box, conserved site (InterPro:IPR000629), DEAD-like helicase, N-terminal (InterPro:IPR014001), DNA/RNA helicase, C-terminal (InterPro:IPR001650), Helicase, superfamily 1/2, ATP-binding domain (InterPro:IPR014021); BEST Arabidopsis thaliana protein match is: P-loop containing nucleoside triphosphate hydrolases superfamily protein (TAIR:AT3G18600.1)
AT1G73210	*1,324*		Protein of unknown function (DUF789); CONTAINS InterPro DOMAIN/s: Protein of unknown function DUF789 (InterPro:IPR008507); BEST Arabidopsis thaliana protein match is: Protein of unknown function (DUF789) (TAIR:AT1G17830.1)
AT1G51950	*1,322*	INDOLE-3-ACETIC ACID INDUCIBLE 18 (IAA18)	indole-3-acetic acid inducible 18 (IAA18); FUNCTIONS IN: sequence-specific DNA binding transcription factor activity; INVOLVED IN: response to auxin stimulus; LOCATED IN: nucleus; EXPRESSED IN: 22 plant structures; EXPRESSED DURING: 13 growth stages; CONTAINS InterPro DOMAIN/s: Aux/IAA-ARF-dimerisation (InterPro:IPR011525), AUX/IAA protein (InterPro:IPR003311); BEST Arabidopsis thaliana protein match is: phytochrome-associated protein 1 (TAIR:AT3G16500.1)
AT3G01060	*1,322*		unknown protein
AT5G37820	*1,322*	NOD26-LIKE INTRINSIC PROTEIN 4;2 (NIP4;2)	NOD26-like intrinsic protein 4;2 (NIP4;2); FUNCTIONS IN: water channel activity; INVOLVED IN: transport, transmembrane transport; LOCATED IN: integral to membrane, membrane; EXPRESSED IN: root, flower, cultured cell, leaf; CONTAINS InterPro DOMAIN/s: Major intrinsic protein, conserved site (InterPro:IPR022357), Aquaporin (InterPro:IPR012269), Major intrinsic protein (InterPro:IPR000425); BEST Arabidopsis thaliana protein match is: NOD26-like intrinsic protein 4;1 (TAIR:AT5G37810.1)
AT5G57390	*1,322*	AINTEGUMENTA-LIKE 5 (AIL5)	Encodes a member of the AP2 family of transcriptional regulators. May be involved in germination and seedling growth. Mutants are resistant to ABA analogs and are resistant to high nitrogen concentrations.essential for the developmental transition between the embryonic and vegetative phases in plants. Overexpression results in the formation of somatic embryos on cotyledons. It is also required to maintain high levels of PIN1 expression at the periphery of the meristem and modulate local auxin production in the central region of the SAM which underlies phyllotactic transitions.

AT4G12430	*1,32*	TREHALOSE-6-PHOSPHATE PHOSPHATASE F (TPPF)	Haloacid dehalogenase-like hydrolase (HAD) superfamily protein; FUNCTIONS IN: catalytic activity, trehalose-phosphatase activity; INVOLVED IN: trehalose biosynthetic process, metabolic process; EXPRESSED IN: sperm cell, male gametophyte, pollen tube; EXPRESSED DURING: L mature pollen stage, M germinated pollen stage; CONTAINS InterPro DOMAIN/s: HAD-superfamily hydrolase, subfamily IIB (InterPro:IPR006379), Trehalose-phosphatase (InterPro:IPR003337); BEST Arabidopsis thaliana protein match is: Haloacid dehalogenase-like hydrolase (HAD) superfamily protein (TAIR:AT4G22590.1)
AT1G49750	*1,319*		Leucine-rich repeat (LRR) family protein; LOCATED IN: chloroplast; EXPRESSED IN: 22 plant structures; EXPRESSED DURING: 13 growth stages; CONTAINS InterPro DOMAIN/s: Leucine-rich repeat (InterPro:IPR001611); BEST Arabidopsis thaliana protein match is: Leucine-rich repeat (LRR) family protein (TAIR:AT3G19320.1)
AT4G34410	*1,317*	REDOX RESPONSIVE TRANSCRIPTION FACTOR 1 (RRTF1)	encodes a member of the ERF (ethylene response factor) subfamily B-3 of ERF/AP2 transcription factor family. The protein contains one AP2 domain. There are 18 members in this subfamily including ATERF-1, ATERF-2, AND ATERF-5.
AT1G62870	*1,316*		unknown protein; BEST Arabidopsis thaliana protein match is: unknown protein (TAIR:AT1G12380.1)
AT5G02490	*1,316*	(Hsp70-2)	Heat shock protein 70 (Hsp 70) family protein; FUNCTIONS IN: protein binding; INVOLVED IN: protein folding, response to cadmium ion, response to heat, response to bacterium; LOCATED IN: cytosol, cell wall, plasma membrane; EXPRESSED IN: 19 plant structures; EXPRESSED DURING: 10 growth stages; CONTAINS InterPro DOMAIN/s: Heat shock protein 70, conserved site (InterPro:IPR018181), Heat shock protein Hsp70 (InterPro:IPR001023), Heat shock protein 70 (InterPro:IPR013126); BEST Arabidopsis thaliana protein match is: heat shock cognate protein 70-1 (TAIR:AT5G02500.1)
AT1G02820	*1,315*	LATE EMBRYOGENESIS ABUNDANT 3 (LEA3)	Late embryogenesis abundant 3 (LEA3) family protein; FUNCTIONS IN: molecular_function unknown; INVOLVED IN: embryo development, response to stress; EXPRESSED IN: 16 plant structures; EXPRESSED DURING: 8 growth stages; CONTAINS InterPro DOMAIN/s: Late embryogenesis abundant protein, group 3 (InterPro:IPR004926); BEST Arabidopsis thaliana protein match is: senescence-associated gene 21 (TAIR:AT4G02380.1)
AT4G21620	*1,315*		glycine-rich protein; FUNCTIONS IN: molecular_function unknown; INVOLVED IN: biological_process unknown; LOCATED IN: endomembrane system; EXPRESSED IN: 22 plant structures; EXPRESSED DURING: 13 growth stages; BEST Arabidopsis thaliana protein match is: unknown protein (TAIR:AT2G19000.1)
AT2G35830	*1,312*		unknown protein; FUNCTIONS IN: molecular_function unknown; INVOLVED IN: biological_process unknown; LOCATED IN: cellular_component unknown; BEST Arabidopsis thaliana protein match is: unknown protein (TAIR:AT2G35810.1).
AT5G17230	*1,312*	PHYTOENE SYNTHASE (PSY)	Encodes phytoene synthase that is the rate-limiting enzyme in the carotenoid biosynthetic pathway and that interacts with the ORANGE (OR) protein. PSY's expression is posttransciptionally regulated by OR.
AT1G56170	*1,311*	NUCLEAR FACTOR Y, SUBUNIT C2 (NF-YC2)	Encodes a protein with similarity to a subunit of the CCAAT promoter motif binding complex of yeast.One of two members of this class (HAP5B) and expressed in vegetative and reproductive tissues
AT3G28150	*1,311*	TRICHOME BIREFRINGENCE-LIKE 22 (TBL22)	Encodes a member of the TBL (TRICHOME BIREFRINGENCE-LIKE) gene family containing a plant-specific DUF231 (domain of unknown function) domain. A putative xyloglucan O-acetyltransferase. TBL gene family has 46 members, two of which (TBR/AT5G06700 and TBL3/AT5G01360) have been shown to be involved in the synthesis and deposition of secondary wall cellulose, presumably by influencing the esterification state of pectic polymers. A nomenclature for this gene family has been proposed (Volker Bischoff & Wolf Scheible, 2010, personal communication).
AT4G18910	*1,31*	NOD26-LIKE INTRINSIC PROTEIN 1;2 (NIP1;2)	Encodes an aquaporin homolog. Functions in arsenite transport and tolerance.When expressed in yeast cells can conduct hydrogen peroxide into those cells.
AT5G64940	*1,308*	ABC2 HOMOLOG 13 (ATH13)	ABC1K8 is a member of an atypical protein kinase family that is induced by heavy metals. Loss of function mutations affect the metabolic profile of chloroplast lipids. It appears to function along with ABC1K7 in mediating lipid membrane changes in response to stress. The mRNA is cell-to-cell mobile.
AT3G49580	*1,307*	RESPONSE TO LOW SULFUR 1 (LSU1)	RESPONSE TO LOW SULFUR 1 (LSU1); BEST Arabidopsis thaliana protein match is: response to low sulfur 3 (TAIR:AT3G49570.1)
AT3G46530	*1,306*	RECOGNITION OF PERONOSPORA PARASITICA 13 (RPP13)	Confers resistance to the biotrophic oomycete, Peronospora parasitica. Encodes an NBS-LRR type R protein with a putative amino-terminal leucine zipper. Fungal protein ATR13 induces RPP13 gene expression and disease resistance. The mRNA is cell-to-cell mobile.

AT5G62620	*1,306*	(GALT6)	Encodes a Golgi-localized hydroxyproline-O-galactosyltransferase. Mutants display multiple phenotypes including reduced seed coat mucilage and accelerated leaf senescence.
AT1G27020	*1,305*		unknown protein; BEST Arabidopsis thaliana protein match is: unknown protein (TAIR:AT1G27030.1)
AT5G56600	*1,303*	PROFILIN 3 (PRF3)	Encodes profilin3, a low-molecular weight, actin monomer-binding protein that regulates the organization of actin cytoskeleton. Originally known as profilin5, and later named profilin3. Expressed in vegetative organs. Mutants have slightly elongated petioles.
AT1G77270	*1,302*		unknown protein; BEST Arabidopsis thaliana protein match is: unknown protein (TAIR:AT3G07730.1)
AT5G46590	*1,301*	NAC DOMAIN CONTAINING PROTEIN 96 (NAC096)	NAC domain containing protein 96 (NAC096); FUNCTIONS IN: sequence-specific DNA binding transcription factor activity; INVOLVED IN: multicellular organismal development, regulation of transcription; LOCATED IN: cellular_component unknown; EXPRESSED IN: 9 plant structures; EXPRESSED DURING: 6 growth stages; CONTAINS InterPro DOMAIN/s: No apical meristem (NAM) protein (InterPro:IPR003441); BEST Arabidopsis thaliana protein match is: NAC domain containing protein 71 (TAIR:AT4G17980.1)
AT1G12900	*1,3*	GLYCERALDEHYDE 3-PHOSPHATE DEHYDROGENASE A SUBUNIT 2 (GAPA-2)	glyceraldehyde 3-phosphate dehydrogenase A subunit 2 (GAPA-2); FUNCTIONS IN: NAD or NADH binding, glyceraldehyde-3-phosphate dehydrogenase activity; INVOLVED IN: oxidation reduction, glycolysis, glucose metabolic process; LOCATED IN: apoplast, chloroplast stroma, chloroplast, membrane, chloroplast envelope; EXPRESSED IN: 24 plant structures; EXPRESSED DURING: 14 growth stages; CONTAINS InterPro DOMAIN/s: Glyceraldehyde 3-phosphate dehydrogenase subfamily (InterPro:IPR000173), Glyceraldehyde 3-phosphate dehydrogenase family (InterPro:IPR020831), Glyceraldehyde-3-phosphate dehydrogenase, type I (InterPro:IPR006424), Glyceraldehyde 3-phosphate dehydrogenase, active site (InterPro:IPR020830), Glyceraldehyde 3-phosphate dehydrogenase, catalytic domain (InterPro:IPR020829), Glyceraldehyde 3-phosphate dehydrogenase, catalytic domain, subgroup (InterPro:IPR020832), Glyceraldehyde 3-phosphate dehydrogenase, NAD(P) binding domain (InterPro:IPR020828); BEST Arabidopsis thaliana protein match is: glyceraldehyde 3-phosphate dehydrogenase A subunit (TAIR:AT3G26650.1)
AT2G02010	*1,3*	GLUTAMATE DECARBOXYLASE 4 (GAD4)	glutamate decarboxylase 4 (GAD4); FUNCTIONS IN: calmodulin binding; INVOLVED IN: carboxylic acid metabolic process, glutamate metabolic process, glutamate decarboxylation to succinate; LOCATED IN: cytosol, nucleus; CONTAINS InterPro DOMAIN/s: Pyridoxal phosphate-dependent transferase, major domain (InterPro:IPR015424), Pyridoxal phosphate-dependent decarboxylase (InterPro:IPR002129), Glutamate decarboxylase (InterPro:IPR010107), Pyridoxal phosphate-dependent transferase, major region, subdomain 1 (InterPro:IPR015421); BEST Arabidopsis thaliana protein match is: glutamate decarboxylase 3 (TAIR:AT2G02000.1)
AT2G40020	*1,298*		Nucleolar histone methyltransferase-related protein; FUNCTIONS IN: molecular_function unknown; INVOLVED IN: biological_process unknown; LOCATED IN: cellular_component unknown; EXPRESSED IN: 22 plant structures; EXPRESSED DURING: 13 growth stages; CONTAINS InterPro DOMAIN/s: WIYLD domain (InterPro:IPR018848); BEST Arabidopsis thaliana protein match is: Nucleolar histone methyltransferase-related protein (TAIR:AT1G45248.4)
AT2G47680	*1,298*		zinc finger (CCCH type) helicase family protein; FUNCTIONS IN: helicase activity, zinc ion binding, nucleic acid binding, ATP binding, ATP-dependent helicase activity; INVOLVED IN: nucleobase, nucleoside, nucleotide and nucleic acid metabolic process; LOCATED IN: chloroplast; EXPRESSED IN: 19 plant structures; EXPRESSED DURING: 13 growth stages; CONTAINS InterPro DOMAIN/s: Zinc finger, CCCH-type (InterPro:IPR000571), DNA/RNA helicase, DEAD/DEAH box type, N-terminal (InterPro:IPR011545), DEAD-like helicase, N-terminal (InterPro:IPR014001), DNA/RNA helicase, C-terminal (InterPro:IPR001650), Helicase, superfamily 1/2, ATP-binding domain (InterPro:IPR014021); BEST Arabidopsis thaliana protein match is: DEA(D/H)-box RNA helicase family protein (TAIR:AT2G01130.1)
AT5G15970	*1,297*	(KIN2)	Encodes a gene that can be induced by cold and abscisic acid and may be involved in cold acclimation and salt tolerance. The mRNA is cell-to-cell mobile.
AT5G43860	*1,293*	CHLOROPHYLLASE 2 (CLH2)	Encodes a chlorophyllase, the first enzyme in chlorophyll degradation. It catalyzes the hydrolysis of the ester bond to chlorophyllide and phytol. AtCLH2 has a typical signal sequence for the chloroplast. Gene expression does not respond to methyljasmonate, a known promoter of senescence and chlorophyll degradation.

AT1G52570	*1,291*	PHOSPHOLIPASE D ALPHA 2 (PLDALPHA2)	member of C2-PLD subfamily
AT1G75040	*1,29*	PATHOGENESIS-RELATED GENE 5 (PR5)	Thaumatin-like protein involved in response to pathogens. mRNA level of the PR-5 gene (At1g75040)is significantly changed after cutting the inflorescence stem indicating the existence of a network of signal transducing pathways as other stress-regulated genes (At5g01410, At3g17800, At1g29930)do not response to the treatment. The mRNA is cell-to-cell mobile.
AT3G13640	*1,29*	ATP-BINDING CASSETTE E1 (ABCE1)	member of RLI subfamily
AT5G62350	*1,29*		Plant invertase/pectin methylesterase inhibitor superfamily protein; FUNCTIONS IN: enzyme inhibitor activity, pectinesterase inhibitor activity, pectinesterase activity; INVOLVED IN: response to salt stress; LOCATED IN: endomembrane system; EXPRESSED IN: 22 plant structures; EXPRESSED DURING: 13 growth stages; CONTAINS InterPro DOMAIN/s: Pectinesterase inhibitor (InterPro:IPR006501); BEST Arabidopsis thaliana protein match is: Plant invertase/pectin methylesterase inhibitor superfamily protein (TAIR:AT3G47380.1)
AT1G80600	*1,286*	HOPW1-1-INTERACTING 1 (WIN1)	Encodes HopW1-1-Interacting protein 1 (WIN1). Interacts with the P. syringae effector HopW1-1. WIN1 is a putative acetylornithine transaminase. Modulates plant defenses against bacteria. Three WIN proteins are identified so far (WIN1: AT1G80600; WIN2: AT4G31750; WIN3: AT5G13320). Mediates red-light inhibition of seed germination.
AT4G15680	*1,282*	(GRXS4)	Thioredoxin superfamily protein; FUNCTIONS IN: electron carrier activity, arsenate reductase (glutaredoxin) activity, protein disulfide oxidoreductase activity; INVOLVED IN: cell redox homeostasis; LOCATED IN: endomembrane system; EXPRESSED IN: 14 plant structures; EXPRESSED DURING: 8 growth stages; CONTAINS InterPro DOMAIN/s: Glutaredoxin-like, plant II (InterPro:IPR011905), Thioredoxin fold (InterPro:IPR012335), Glutaredoxin (InterPro:IPR002109), Glutaredoxin subgroup (InterPro:IPR014025), Thioredoxin-like fold (InterPro:IPR012336); BEST Arabidopsis thaliana protein match is: Thioredoxin superfamily protein (TAIR:AT4G15670.1)
AT4G11100	*1,281*		unknown protein; BEST Arabidopsis thaliana protein match is: unknown protein (TAIR:AT5G03060.1)
AT4G36870	*1,281*	BEL1-LIKE HOMEODOMAIN 2 (BLH2)	Encodes a member of the BEL family of homeodomain proteins. Plants doubly mutant for saw1/saw2 (blh2/blh4) have serrated leaves. BP is expressed in the serrated leaves, therefore saw1/saw2 may act redundantly to repress BP in leaves.
AT1G07440	*1,28*		NAD(P)-binding Rossmann-fold superfamily protein; FUNCTIONS IN: oxidoreductase activity, binding, catalytic activity; INVOLVED IN: response to cadmium ion, response to karrikin; LOCATED IN: cellular_component unknown; EXPRESSED IN: 22 plant structures; EXPRESSED DURING: 13 growth stages; CONTAINS InterPro DOMAIN/s: Short-chain dehydrogenase/reductase, conserved site (InterPro:IPR020904), NAD(P)-binding domain (InterPro:IPR016040), Glucose/ribitol dehydrogenase (InterPro:IPR002347), Short-chain dehydrogenase/reductase SDR (InterPro:IPR002198); BEST Arabidopsis thaliana protein match is: NAD-dependent epimerase/dehydratase family protein (TAIR:AT2G29340.1)
AT2G19440	*1,28*		O-Glycosyl hydrolases family 17 protein; FUNCTIONS IN: cation binding, hydrolase activity, hydrolyzing O-glycosyl compounds, catalytic activity; INVOLVED IN: carbohydrate metabolic process; LOCATED IN: plasma membrane, anchored to membrane; EXPRESSED IN: male gametophyte, pollen tube; EXPRESSED DURING: M germinated pollen stage; CONTAINS InterPro DOMAIN/s: X8 (InterPro:IPR012946), Glycoside hydrolase, catalytic core (InterPro:IPR017853), Glycoside hydrolase, family 17 (InterPro:IPR000490), Glycoside hydrolase, subgroup, catalytic core (InterPro:IPR013781); BEST Arabidopsis thaliana protein match is: O-Glycosyl hydrolases family 17 protein (TAIR:AT1G64760.2)
AT1G78970	*1,279*	LUPEOL SYNTHASE 1 (LUP1)	Lupeol synthase. Converts oxidosqualene to multiple triterpene alcohols and a triterpene diols. This conversion proceeds through the formation of a 17β-dammarenyl cation.
AT2G39730	*1,279*	RUBISCO ACTIVASE (RCA)	Rubisco activase, a nuclear-encoded chloroplast protein that consists of two isoforms arising from alternative splicing in most plants. Required for the light activation of rubisco. Involved in jasmonate-induced leaf senescence.
AT1G70220	*1,278*		RNA-processing, Lsm domain; CONTAINS InterPro DOMAIN/s: RNA-processing, Lsm domain (InterPro:IPR019181); BEST Arabidopsis thaliana protein match is: RNA-processing, Lsm domain (TAIR:AT1G24050.1)
AT1G66980	*1,276*	SUPPRESSOR OF NPR1-1 CONSTITUTIVE 4 (SNC4)	Encodes SNC4 (suppressor of npr1-1, constitutive 4), an atypical receptor-like kinase with two predicted extracellular glycerophosphoryl diester phosphodiesterase domains.
AT4G32190	*1,276*		Myosin heavy chain-related protein

AT5G04690	*1,274*		Ankyrin repeat family protein; CONTAINS InterPro DOMAIN/s: Ankyrin repeat-containing domain (InterPro:IPR020683), Ankyrin repeat (InterPro:IPR002110); BEST Arabidopsis thaliana protein match is: Ankyrin repeat family protein (TAIR:AT5G04700.1)
AT5G52190	*1,274*		Sugar isomerase (SIS) family protein; FUNCTIONS IN: sugar binding; INVOLVED IN: carbohydrate metabolic process; EXPRESSED IN: 17 plant structures; EXPRESSED DURING: 10 growth stages; CONTAINS InterPro DOMAIN/s: Sugar isomerase (SIS) (InterPro:IPR001347)
AT3G61150	*1,273*	HOMEODOMAIN GLABROUS 1 (HDG1)	Encodes a homeobox-leucine zipper family protein belonging to the HD-ZIP IV family.
AT5G63090	*1,273*	LATERAL ORGAN BOUNDARIES (LOB)	Involved in lateral organ development
AT5G66670	*1,273*		Protein of unknown function (DUF677); FUNCTIONS IN: molecular_function unknown; INVOLVED IN: biological_process unknown; LOCATED IN: chloroplast; EXPRESSED IN: inflorescence meristem, hypocotyl, root; CONTAINS InterPro DOMAIN/s: Protein of unknown function DUF677 (InterPro:IPR007749); BEST Arabidopsis thaliana protein match is: Protein of unknown function (DUF677) (TAIR:AT5G66660.1)
AT1G15310	*1,268*	SIGNAL RECOGNITION PARTICLE 54 KDA SUBUNIT	54 kDa protein subunit of SRP that interacts with the signal peptide of secreted proteins
AT4G36910	*1,268*	LOSS OF THE TIMING OF ET AND JA BIOSYNTHESIS 2 (LEJ2)	Encodes a single cystathionine beta-synthase domain-containing protein. Modulates development by regulating the thioredoxin system.
AT5G01040	*1,268*	LACCASE 8 (LAC8)	putative laccase, knockout mutant showed early flowering
AT1G70140	*1,267*	FORMIN 8 (FH8)	Encodes a group I formin. Binds to F-actin barbed ends. Has severing actin filaments activity. Binds profilin. Involved in the initiation and tip growth of root hairs through regulation of actin cytoskeleton.
AT2G29360	*1,264*		NAD(P)-binding Rossmann-fold superfamily protein; FUNCTIONS IN: oxidoreductase activity, binding, catalytic activity; INVOLVED IN: oxidation reduction, metabolic process; LOCATED IN: cellular_component unknown; EXPRESSED IN: 22 plant structures; EXPRESSED DURING: 14 growth stages; CONTAINS InterPro DOMAIN/s: Short-chain dehydrogenase/reductase, conserved site (InterPro:IPR020904), NAD(P)-binding domain (InterPro:IPR016040), Glucose/ribitol dehydrogenase (InterPro:IPR002347), Short-chain dehydrogenase/reductase SDR (InterPro:IPR002198); BEST Arabidopsis thaliana protein match is: NAD(P)-binding Rossmann-fold superfamily protein (TAIR:AT2G29150.1)
AT4G33560	*1,264*		Wound-responsive family protein; CONTAINS InterPro DOMAIN/s: Protein of unknown function wound-induced (InterPro:IPR022251); BEST Arabidopsis thaliana protein match is: wound-responsive protein-related (TAIR:AT2G14070.1)
AT5G49520	*1,264*	WRKY DNA-BINDING PROTEIN 48 (WRKY48)	Encodes WRKY48, a member of the WRKY Transcription Factor. WRKY48 is a stress- and pathogen-induced transcriptional activator that represses plant basal defense. The mRNA is cell-to-cell mobile.
AT2G28950	*1,263*	EXPANSIN A6 (EXPA6)	Encodes an expansin. Naming convention from the Expansin Working Group (Kende et al, 2004. Plant Mol Bio). Involved in the formation of nematode-induced syncytia in roots of Arabidopsis thaliana.
AT3G29810	*1,262*	COBRA-LIKE PROTEIN 2 PRECURSOR (COBL2)	During the course of seed coat epidermal cell differentiation, COBRA-LIKE 2 plays a role in cellulose deposition into mucilage secretory cells of Arabidopsis seeds. COBRA-LIKE 2 affects mucilage solubility and cellulosic ray formation.
AT5G62940	*1,262*	HIGH CAMBIAL ACTIVITY2 (HCA2)	HCA2 induces the formation of interfascicular cambium and regulates vascular tissue development in the aerial parts of the plant. Evidence from both gain of function and dominant negative alleles.
AT5G17490	*1,261*	RGA-LIKE PROTEIN 3 (RGL3)	DELLA subfamily member involved in GA signal transduction
AT3G49690	*1,26*	MYB DOMAIN PROTEIN 84 (MYB84)	Putative homolog of the Blind gene in tomato. Together with RAX1 and RAX3 belong to the class R2R3 MYB genes; encoded by the Myb-like transcription factor MYB84, regulates axillary meristem formation.
AT1G12610	*1,259*	DWARF AND DELAYED FLOWERING 1 (DDF1)	Encodes a member of the DREB subfamily A-1 of ERF/AP2 transcription factor family (DDF1). The protein contains one AP2 domain. There are six members in this subfamily, including CBF1, CBF2, and CBF3. Overexpression of this gene results in delayed flowering and dwarfism, reduction of gibberellic acid biosynthesis, and increased tolerance to high levels of salt. This gene is expressed in all tissues examined, but most abundantly expressed in upper stems. Overexpression of this gene is also correlated with increased expression of GA biosynthetic genes and RD29A (a cold and drought responsive gene). Under salt stress it induces the expression of GAOX7, which encodes ad C20-GA inhibitor.

AT1G21810	*1,259*	VESICLE TETHERING 2 (VETH2)	Plant protein of unknown function (DUF869); CONTAINS InterPro DOMAIN/s: Protein of unknown function DUF869, plant (InterPro:IPR008587); BEST Arabidopsis thaliana protein match is: Plant protein of unknown function (DUF869) (TAIR:AT1G77580.1)
AT1G02850	*1,258*	BETA GLUCOSIDASE 11 (BGLU11)	beta glucosidase 11 (BGLU11); FUNCTIONS IN: cation binding, hydrolase activity, hydrolyzing O-glycosyl compounds, catalytic activity; INVOLVED IN: carbohydrate metabolic process; LOCATED IN: endomembrane system; EXPRESSED IN: 24 plant structures; EXPRESSED DURING: 13 growth stages; CONTAINS InterPro DOMAIN/s: Glycoside hydrolase, family 1 (InterPro:IPR001360), Glycoside hydrolase, family 1, active site (InterPro:IPR018120), Glycoside hydrolase, catalytic core (InterPro:IPR017853), Glycoside hydrolase, subgroup, catalytic core (InterPro:IPR013781); BEST Arabidopsis thaliana protein match is: beta glucosidase 10 (TAIR:AT4G27830.1)
AT4G01026	*1,255*	PYR1-LIKE 7 (PYL7)	Encodes a member of the PYR (pyrabactin resistance)/PYL(PYR1-like)/RCAR (regulatory components of ABA receptor) family proteins with 14 members. PYR/PYL/RCAR family proteins function as abscisic acid sensors. Mediate ABA-dependent regulation of protein phosphatase 2Cs ABI1 and ABI2. PYL/RCAR family proteins function as abscisic acid sensors. Mediate ABA-dependent regulation of ABI1 and ABI2.
AT5G15960	*1,252*	(KIN1)	cold and ABA inducible protein kin1, possibly functions as an anti-freeze protein. Transcript level of this gene is induced by cold, ABA, dehydration and osmoticum (mannitol). However, protein activity of GUS fused to the promoter of this gene is inhibited by cold treatment, suggesting an inhibition of the protein by increased transcript level.
AT3G49120	*1,251*	PEROXIDASE CB (PRXCB)	Class III peroxidase Perx34. Expressed in roots, leaves and stems. Located in the cell wall. Involved in cell elongation. Expression activated by light. May play a role in generating H2O2 during defense response. The mRNA is cell-to-cell mobile.
AT4G30020	*1,249*		PA-domain containing subtilase family protein; FUNCTIONS IN: identical protein binding, serine-type endopeptidase activity; INVOLVED IN: proteolysis, negative regulation of catalytic activity; LOCATED IN: endomembrane system, cell wall, membrane; EXPRESSED IN: 20 plant structures; EXPRESSED DURING: 13 growth stages; CONTAINS InterPro DOMAIN/s: Protease-associated PA (InterPro:IPR003137), Proteinase inhibitor, propeptide (InterPro:IPR009020), Peptidase S8A, DUF1034 C-terminal (InterPro:IPR010435), Peptidase S8/S53, subtilisin/kexin/sedolisin (InterPro:IPR000209), Peptidase S8, subtilisin-related (InterPro:IPR015500), Peptidase S8/S53, subtilisin, active site (InterPro:IPR022398), Proteinase inhibitor I9, subtilisin propeptide (InterPro:IPR010259); BEST Arabidopsis thaliana protein match is: subtilisin-like serine protease 3 (TAIR:AT2G19170.1)
AT4G22690	*1,248*	CYTOCHROME P450, FAMILY 706, SUBFAMILY A, POLYPEPTIDE 1 (CYP706A1)	member of CYP706A The mRNA is cell-to-cell mobile.
AT4G24110	*1,248*		unknown protein
AT5G44350	*1,244*		ethylene-responsive nuclear protein -related; BEST Arabidopsis thaliana protein match is: ethylene-responsive nuclear protein / ethylene-regulated nuclear protein (ERT2) (TAIR:AT4G20880.1)
AT2G37620	*1,241*	ACTIN 1 (ACT1)	Member of the actin gene family. Expressed in mature pollen.
AT4G19390	*1,241*		Uncharacterised protein family (UPF0114); FUNCTIONS IN: molecular_function unknown; INVOLVED IN: biological_process unknown; LOCATED IN: chloroplast; EXPRESSED IN: 23 plant structures; EXPRESSED DURING: 13 growth stages; CONTAINS InterPro DOMAIN/s: Uncharacterised protein family UPF0114, plant (InterPro:IPR016804), Uncharacterised protein family UPF0114 (InterPro:IPR005134); BEST Arabidopsis thaliana protein match is: Uncharacterised protein family (UPF0114) (TAIR:AT5G13720.1)
AT1G71690	*1,239*		Protein of unknown function (DUF579); CONTAINS InterPro DOMAIN/s: Protein of unknown function DUF579 (InterPro:IPR021148), Conserved hypothetical protein CHP01627 (InterPro:IPR006514); BEST Arabidopsis thaliana protein match is: Protein of unknown function (DUF579) (TAIR:AT4G09990.1)
AT5G43450	*1,239*		encodes a protein whose sequence is similar to ACC oxidase
AT1G51440	*1,238*	DAD1-LIKE LIPASE 2 (DALL2)	Encodes a lipase that hydrolyzes phosphatidylcholine, glycolipids as well as triacylglycerols.

AT1G24330	*1,234*		ARM repeat superfamily protein; FUNCTIONS IN: ubiquitin-protein ligase activity, binding; INVOLVED IN: protein ubiquitination; LOCATED IN: chloroplast; EXPRESSED IN: 23 plant structures; EXPRESSED DURING: 15 growth stages; CONTAINS InterPro DOMAIN/s: U box domain (InterPro:IPR003613), Armadillo-like helical (InterPro:IPR011989), Armadillo (InterPro:IPR000225), Armadillo-type fold (InterPro:IPR016024); BEST Arabidopsis thaliana protein match is: ARM repeat superfamily protein (TAIR:AT1G67530.2)
AT1G71110	*1,234*		unknown protein; FUNCTIONS IN: molecular_function unknown; INVOLVED IN: biological_process unknown; LOCATED IN: endomembrane system; BEST Arabidopsis thaliana protein match is: unknown protein (TAIR:AT2G12400.1)
AT5G66460	*1,234*	ENDO-BETA-MANNASE 7 (MAN7)	Encodes a endo-beta-mannanase involved in seed germination.
AT2G47750	*1,233*	PUTATIVE INDOLE-3-ACETIC ACID-AMIDO SYNTHETASE GH3.9 (GH3.9)	Encodes GH3.9, a member of the GH3 family auxin-responsive genes. gh3.9-1 mutants had greater primary root length, increased sensitivity to indole-3-acetic acid (IAA)-mediated root growth inhibition, but no obvious effects on apical dominance or leaf morphology.
AT3G56090	*1,233*	FERRITIN 3 (FER3)	Encodes FERRITIN 3, AtFER3. Ferritins are a class of 24-mer multi-meric proteins found in all kingdoms of life. Function as the main iron store in mammals. Evidence suggests that Arabidopsis ferritins are essential to protect cells against oxidative damage, but they do not constitute the major iron pool.
AT3G02030	*1,232*		transferases, transferring acyl groups other than amino-acyl groups;acyltransferases; FUNCTIONS IN: transferase activity, transferring acyl groups other than amino-acyl groups, acyltransferase activity; INVOLVED IN: metabolic process; CONTAINS InterPro DOMAIN/s: Diacylglycerol acyltransferase (InterPro:IPR007130), Alpha/beta hydrolase fold-1 (InterPro:IPR000073); BEST Arabidopsis thaliana protein match is: Esterase/lipase/thioesterase family protein (TAIR:AT5G41130.1)
AT4G02710	*1,231*	NETWORKED 1C (NET1C)	Kinase interacting (KIP1-like) family protein; FUNCTIONS IN: molecular_function unknown; INVOLVED IN: biological_process unknown; LOCATED IN: plasma membrane; EXPRESSED IN: 22 plant structures; EXPRESSED DURING: 10 growth stages; CONTAINS InterPro DOMAIN/s: KIP1-like (InterPro:IPR011684); BEST Arabidopsis thaliana protein match is: kinase interacting (KIP1-like) family protein (TAIR:AT1G03080.1)
AT3G26060	*1,229*	PEROXIREDOXIN Q (PRXQ)	encodes periredoxin Q which decomposes peroxides and plays a role in the protection of the photosynthetic apparatus
AT2G47520	*1,226*	ETHYLENE RESPONSE FACTOR 71 (ERF71)	encodes a member of the ERF (ethylene response factor) subfamily B-2 of ERF/AP2 transcription factor family. The protein contains one AP2 domain. There are 5 members in this subfamily including RAP2.2 AND RAP2.12.
AT1G56660	*1,224*		unknown protein
AT5G17050	*1,223*	UDP-GLUCOSYL TRANSFERASE 78D2 (UGT78D2)	The At5g17050 encodes a anthocyanidin 3-O-glucosyltransferase which specifically glucosylates the 3-position of the flavonoid C-ring. Anthocyanidins such as cyanidin and pelargonidin as well as flavonols such as kaempferol and quercetin are accepted substrates.
AT1G27910	*1,222*	PLANT U-BOX 45 (PUB45)	Encodes a protein containing a UND, a U-box, and an ARM domain. This protein has E3 ubiquitin ligase activity based on in vitro assays.
AT3G61890	*1,222*	HOMEOBOX 12 (HB-12)	Encodes a homeodomain leucine zipper class I (HD-Zip I) protein. Loss of function mutant has abnormally shaped leaves and stems.
AT4G27660	*1,221*		unknown protein; BEST Arabidopsis thaliana protein match is: unknown protein (TAIR:AT5G54150.1)
AT3G55800	*1,219*	SEDOHEPTULOSE-BISPHOSPHATASE (SBPASE)	Encodes the chloroplast enzyme sedoheptulose-1,7-bisphosphatase (SBPase), involved in the carbon reduction of the Calvin cycle. Increase in SBPase activity in transgenic lines accumulate up to 50% more sucrose and starch than wild-type. The mRNA is cell-to-cell mobile.
AT1G69840	*1,218*	HYPERSENSITIVE INDUCED REACTION 1 (ATHIR1)	SPFH/Band 7/PHB domain-containing membrane-associated protein family; INVOLVED IN: biological_process unknown; LOCATED IN: plasma membrane, chloroplast, vacuole; EXPRESSED IN: 26 plant structures; EXPRESSED DURING: 15 growth stages; CONTAINS InterPro DOMAIN/s: Band 7 protein (InterPro:IPR001107); BEST Arabidopsis thaliana protein match is: SPFH/Band 7/PHB domain-containing membrane-associated protein family (TAIR:AT5G62740.1)

AT1G16640	*1,217*		AP2/B3-like transcriptional factor family protein; FUNCTIONS IN: DNA binding, sequence-specific DNA binding transcription factor activity; INVOLVED IN: regulation of transcription, DNA-dependent; LOCATED IN: cellular_component unknown; EXPRESSED IN: 16 plant structures; EXPRESSED DURING: 11 growth stages; CONTAINS InterPro DOMAIN/s: Transcriptional factor B3 (InterPro:IPR003340); BEST Arabidopsis thaliana protein match is: AP2/B3-like transcriptional factor family protein (TAIR:AT3G06220.1)
AT1G30490	*1,217*	PHAVOLUTA (PHV)	Dominant PHV mutations cause transformation of abaxial leaf fates into adaxial leaf fates. Has overlapping functions with PHABULOSA, REVOLUTA and CORONA/ATHB15 in patterning the apical portion of the embryo. Encodes a member of HD-Zip family which contains homeodomain-leucine zipper domains and domain similar to a mammalian sterol binding domain.
AT1G74670	*1,217*	GA-STIMULATED ARABIDOPSIS 6 (GASA6)	Gibberellin-regulated family protein; FUNCTIONS IN: molecular_function unknown; INVOLVED IN: in 6 processes; LOCATED IN: endomembrane system; EXPRESSED IN: 21 plant structures; EXPRESSED DURING: 13 growth stages; CONTAINS InterPro DOMAIN/s: Gibberellin regulated protein (InterPro:IPR003854); BEST Arabidopsis thaliana protein match is: GAST1 protein homolog 4 (TAIR:AT5G15230.1)
AT3G48730	*1,217*	GLUTAMATE-1-SEMIALDEHYDE 2,1-AMINOMUTASE 2 (GSA2)	glutamate-1-semialdehyde 2,1-aminomutase 2 (GSA2); FUNCTIONS IN: glutamate-1-semialdehyde 2,1-aminomutase activity, pyridoxal phosphate binding, transaminase activity, catalytic activity; INVOLVED IN: porphyrin biosynthetic process; LOCATED IN: chloroplast stroma, chloroplast, chloroplast envelope; EXPRESSED IN: 23 plant structures; EXPRESSED DURING: 15 growth stages; CONTAINS InterPro DOMAIN/s: Pyridoxal phosphate-dependent transferase, major domain (InterPro:IPR015424), Aminotransferase class-III (InterPro:IPR005814), Tetrapyrrole biosynthesis, glutamate-1-semialdehyde aminotransferase (InterPro:IPR004639), Pyridoxal phosphate-dependent transferase, major region, subdomain 1 (InterPro:IPR015421); BEST Arabidopsis thaliana protein match is: glutamate-1-semialdehyde-2,1-aminomutase (TAIR:AT5G63570.1)
AT4G33010	*1,217*	GLYCINE DECARBOXYLASE P-PROTEIN 1 (GLDP1)	glycine decarboxylase P-protein 1 (GLDP1); FUNCTIONS IN: glycine dehydrogenase (decarboxylating) activity, protein binding; INVOLVED IN: glycine catabolic process, response to cadmium ion, glycine decarboxylation via glycine cleavage system; LOCATED IN: mitochondrion, apoplast, glycine cleavage complex, chloroplast, chloroplast envelope; EXPRESSED IN: 31 plant structures; EXPRESSED DURING: 16 growth stages; CONTAINS InterPro DOMAIN/s: Pyridoxal phosphate-dependent transferase, major domain (InterPro:IPR015424), Glycine cleavage system P-protein (InterPro:IPR003437), Glycine cleavage system P-protein-like (InterPro:IPR020581), Pyridoxal phosphate-dependent transferase, major region, subdomain 1 (InterPro:IPR015421), Glycine cleavage system P-protein, N-terminal (InterPro:IPR020580); BEST Arabidopsis thaliana protein match is: glycine decarboxylase P-protein 2 (TAIR:AT2G26080.1)
AT1G12380	*1,216*		unknown protein; BEST Arabidopsis thaliana protein match is: unknown protein (TAIR:AT1G62870.1)
AT1G61890	*1,216*		MATE efflux family protein; FUNCTIONS IN: antiporter activity, drug transmembrane transporter activity, transporter activity; INVOLVED IN: response to karrikin; LOCATED IN: membrane; EXPRESSED IN: 22 plant structures; EXPRESSED DURING: 13 growth stages; CONTAINS InterPro DOMAIN/s: Multi antimicrobial extrusion protein MatE (InterPro:IPR002528); BEST Arabidopsis thaliana protein match is: MATE efflux family protein (TAIR:AT1G11670.1)
AT2G14080	*1,216*		Disease resistance protein (TIR-NBS-LRR class) family; FUNCTIONS IN: transmembrane receptor activity, nucleoside-triphosphatase activity, nucleotide binding, ATP binding; INVOLVED IN: signal transduction, apoptosis, defense response, innate immune response; LOCATED IN: intrinsic to membrane, endomembrane system; EXPRESSED IN: 19 plant structures; EXPRESSED DURING: 13 growth stages; CONTAINS InterPro DOMAIN/s: ATPase, AAA+ type, core (InterPro:IPR003593), NB-ARC (InterPro:IPR002182), Leucine-rich repeat (InterPro:IPR001611), Disease resistance protein (InterPro:IPR000767), Toll-Interleukin receptor (InterPro:IPR000157); BEST Arabidopsis thaliana protein match is: Disease resistance protein (TIR-NBS-LRR class) (TAIR:AT5G11250.1)
AT3G02870	*1,215*	(VTC4)	Encodes a L-galactose-1-phosphate phosphatase, involved in ascorbate biosynthesis.
AT1G21670	*1,213*		LOCATED IN: cell wall, plant-type cell wall; EXPRESSED IN: 23 plant structures; EXPRESSED DURING: 14 growth stages; CONTAINS InterPro DOMAIN/s: WD40-like Beta Propeller (InterPro:IPR011659), Six-bladed beta-propeller, TolB-like (InterPro:IPR011042); BEST Arabidopsis thaliana protein match is: DPP6 N-terminal domain-like protein (TAIR:AT1G21680.1)

AT2G44920	*1,211*		Tetratricopeptide repeat (TPR)-like superfamily protein; FUNCTIONS IN: molecular_function unknown; LOCATED IN: thylakoid, thylakoid lumen, chloroplast thylakoid membrane, chloroplast thylakoid lumen, chloroplast; EXPRESSED IN: 24 plant structures; EXPRESSED DURING: 13 growth stages; CONTAINS InterPro DOMAIN/s: Pentapeptide repeat (InterPro:IPR001646); BEST Arabidopsis thaliana protein match is: Pentapeptide repeat-containing protein (TAIR:AT1G12250.1)
AT1G56500	*1,21*	SUPPRESSOR OF QUENCHING 1 (SOQ1)	Encodes a thylakoid membrane protein with thioredoxin-like and beta-propeller domains located in the lumen and a haloacid-dehalogenase domain exposed to the chloroplast stroma. The protein's role may be to prevent formation of a slowly reversible form of antenna quenching, thereby maintaining the efficiency of light harvesting. The mRNA is cell-to-cell mobile.
AT2G19900	*1,21*	NADP-MALIC ENZYME 1 (NADP-ME1)	The malic enzyme (EC 1.1.1.40) encoded by AtNADP-ME1 is expressed in response to developmental and cell-specific signals. The enzyme is active in vitro and appears to function as a homohexamer or homooctamer. It is believed to be a cytosolic protein.
AT5G50950	*1,21*	FUMARASE 2 (FUM2)	Encodes a fumarase enzyme initially shown to be in the mitochondria through proteomic studies but later shown to be present in the cytosol using an RFP fluorescent protein tag. It appears to be important for the accumulation of fumarate from malate in leaves in the light, and helps to promote nitrogen assimilation under high nitrogen conditions. It does not appear to be necessary for lipid metabolism and seedling growth. Inhibition of fumarate accumulation results in an overall shift in the cold response of leaves, with a complete inhibition of cold acclimation of photosynthesis.
AT5G67080	*1,21*	MITOGEN-ACTIVATED PROTEIN KINASE KINASE KINASE 19 (MAPKKK19)	member of MEKK subfamily
AT5G48450	*1,209*	SKU5 SIMILAR 3 (sks3)	Encodes a protein with two DUF26 domains and a signal peptide for secretion. The protein is transported to the apoplast when it is expressed as a GFP fusion protein.
AT1G62780	*1,207*		unknown protein; FUNCTIONS IN: molecular_function unknown; INVOLVED IN: biological_process unknown; LOCATED IN: chloroplast, chloroplast stroma; EXPRESSED IN: 22 plant structures; EXPRESSED DURING: 14 growth stages
AT5G13770	*1,207*		Pentatricopeptide repeat (PPR-like) superfamily protein; FUNCTIONS IN: molecular_function unknown; INVOLVED IN: biological_process unknown; LOCATED IN: chloroplast; EXPRESSED IN: 22 plant structures; EXPRESSED DURING: 15 growth stages; CONTAINS InterPro DOMAIN/s: Pentatricopeptide repeat (InterPro:IPR002885); BEST Arabidopsis thaliana protein match is: Pentatricopeptide repeat (PPR-like) superfamily protein (TAIR:AT5G42310.1)
AT3G28930	*1,206*	AVRRPT2-INDUCED GENE 2 (AIG2)	avrRpt2-induced gene that exhibits RPS2- and avrRpt2-dependent induction early after infection with Pseudomonas syringae pv maculicola strain ES4326 carrying avrRpt2
AT4G15700	*1,205*	GLUTAREDOXIN 3 (GRXS3)	Thioredoxin superfamily protein; FUNCTIONS IN: electron carrier activity, arsenate reductase (glutaredoxin) activity, protein disulfide oxidoreductase activity; INVOLVED IN: cell redox homeostasis; LOCATED IN: endomembrane system; EXPRESSED IN: 16 plant structures; EXPRESSED DURING: 9 growth stages; CONTAINS InterPro DOMAIN/s: Glutaredoxin-like, plant II (InterPro:IPR011905), Thioredoxin fold (InterPro:IPR012335), Glutaredoxin (InterPro:IPR002109), Glutaredoxin subgroup (InterPro:IPR014025), Thioredoxin-like fold (InterPro:IPR012336); BEST Arabidopsis thaliana protein match is: Thioredoxin superfamily protein (TAIR:AT4G15690.1)
AT4G31820	*1,204*	ENHANCER OF PINOID (ENP)	A member of the NPY family genes (NPY1/AT4G31820, NPY2/AT2G14820, NPY3/AT5G67440, NPY4/AT2G23050, NPY5/AT4G37590). Encodes a protein with similarity to NHP3. Contains BTB/POZ domain. Promoter region has canonical auxin response element binding site and Wus binding site. Co-localizes to the late endosome with PID. Regulates cotyledon development through control of PIN1 polarity in concert with PID. Also involved in sepal and gynoecia development.
AT1G15830	*1,203*		unknown protein
AT5G45540	*1,203*		Protein of unknown function (DUF594); CONTAINS InterPro DOMAIN/s: Protein of unknown function DUF594 (InterPro:IPR007658); BEST Arabidopsis thaliana protein match is: Protein of unknown function (DUF594) (TAIR:AT5G45480.1)

AT1G64760	*1,2*		O-Glycosyl hydrolases family 17 protein; FUNCTIONS IN: cation binding, hydrolase activity, hydrolyzing O-glycosyl compounds, catalytic activity; INVOLVED IN: carbohydrate metabolic process; LOCATED IN: anchored to plasma membrane, plasma membrane, anchored to membrane; EXPRESSED IN: guard cell, cultured cell; CONTAINS InterPro DOMAIN/s: X8 (InterPro:IPR012946), Glycoside hydrolase, catalytic core (InterPro:IPR017853), Glycoside hydrolase, family 17 (InterPro:IPR000490), Glycoside hydrolase, subgroup, catalytic core (InterPro:IPR013781); BEST Arabidopsis thaliana protein match is: O-Glycosyl hydrolases family 17 protein (TAIR:AT2G19440.1)
AT1G73110	*1,199*		P-loop containing nucleoside triphosphate hydrolases superfamily protein; FUNCTIONS IN: ATPase activity, ATP binding; LOCATED IN: chloroplast thylakoid membrane, chloroplast; EXPRESSED IN: 21 plant structures; EXPRESSED DURING: 13 growth stages; CONTAINS InterPro DOMAIN/s: ATPase, AAA-type, core (InterPro:IPR003959); BEST Arabidopsis thaliana protein match is: rubisco activase (TAIR:AT2G39730.1)
AT2G32210	*1,199*		unknown protein; BEST Arabidopsis thaliana protein match is: unknown protein (TAIR:AT2G32190.1)
AT1G49530	*1,198*	GERANYLGERANYL PYROPHOSPHATE SYNTHASE 6 (GGPS6)	encodes a mitochondria-targeted geranylgeranyl pyrophosphate synthase
AT2G41230	*1,198*	ORGAN SIZE RELATED 1 (OSR1)	Encodes an ER-localized plant hormone-responsive gene and appears to act redundantly with ARGOS and ARL during organ growth. Over-expression modifies plant sensitivity to ethylene, leading to improved drought tolerance.
AT2G21640	*1,197*		Encodes a protein of unknown function that is a marker for oxidative stress response.Expression in rosette leaves is activated by high concentration of boron.
AT4G18250	*1,197*		receptor serine/threonine kinase, putative; FUNCTIONS IN: transmembrane receptor protein serine/threonine kinase activity, kinase activity; INVOLVED IN: protein amino acid phosphorylation; EXPRESSED IN: 8 plant structures; EXPRESSED DURING: LP.06 six leaves visible, LP.04 four leaves visible, LP.10 ten leaves visible, LP.02 two leaves visible, LP.08 eight leaves visible; CONTAINS InterPro DOMAIN/s: Protein kinase, ATP binding site (InterPro:IPR017441), Serine/threonine-protein kinase domain (InterPro:IPR002290), Serine-threonine/tyrosine-protein kinase (InterPro:IPR001245), Protein kinase-like domain (InterPro:IPR011009), Serine/threonine-protein kinase, active site (InterPro:IPR008271), Protein kinase, catalytic domain (InterPro:IPR000719), Tyrosine-protein kinase, catalytic domain (InterPro:IPR020635), Thaumatin, conserved site (InterPro:IPR017949), Thaumatin, pathogenesis-related (InterPro:IPR001938); BEST Arabidopsis thaliana protein match is: receptor serine/threonine kinase, putative (TAIR:AT1G70250.1)
AT5G46510	*1,196*	VARIATION IN COMPOUND TRIGGERED ROOT GROWTH RESPONSE-LIKE (VICTL)	Disease resistance protein (TIR-NBS-LRR class) family; FUNCTIONS IN: transmembrane receptor activity, nucleoside-triphosphatase activity, nucleotide binding, ATP binding; INVOLVED IN: signal transduction, defense response, apoptosis, innate immune response; LOCATED IN: intrinsic to membrane; EXPRESSED IN: 21 plant structures; EXPRESSED DURING: 12 growth stages; CONTAINS InterPro DOMAIN/s: ATPase, AAA+ type, core (InterPro:IPR003593), NB-ARC (InterPro:IPR002182), Toll-Interleukin receptor (InterPro:IPR000157), Disease resistance protein (InterPro:IPR000767); BEST Arabidopsis thaliana protein match is: Disease resistance protein (TIR-NBS-LRR class) family (TAIR:AT5G46520.1)
AT2G45850	*1,193*	AT-HOOK MOTIF NUCLEAR LOCALIZED PROTEIN 9 (AHL9)	AT hook motif DNA-binding family protein; FUNCTIONS IN: DNA binding; INVOLVED IN: biological_process unknown; LOCATED IN: nucleus; EXPRESSED IN: 21 plant structures; EXPRESSED DURING: 13 growth stages; CONTAINS InterPro DOMAIN/s: Protein of unknown function DUF296 (InterPro:IPR005175), AT hook, DNA-binding motif (InterPro:IPR017956); BEST Arabidopsis thaliana protein match is: AT hook motif DNA-binding family protein (TAIR:AT3G61310.1)
AT5G56790	*1,193*		Protein kinase superfamily protein; FUNCTIONS IN: protein tyrosine kinase activity, protein kinase activity, kinase activity, ATP binding; INVOLVED IN: protein amino acid phosphorylation; EXPRESSED IN: 22 plant structures; EXPRESSED DURING: 13 growth stages; CONTAINS InterPro DOMAIN/s: Protein kinase, catalytic domain (InterPro:IPR000719), Tyrosine-protein kinase, active site (InterPro:IPR008266), Serine-threonine/tyrosine-protein kinase (InterPro:IPR001245), Protein kinase-like domain (InterPro:IPR011009); BEST Arabidopsis thaliana protein match is: Protein kinase protein with adenine nucleotide alpha hydrolases-like domain (TAIR:AT3G13690.1)
AT1G80300	*1,191*	NUCLEOTIDE TRANSPORTER 1 (NTT1)	Encodes an ATP/ADP transporter. The mRNA is cell-to-cell mobile.

AT1G55480	*1,19*	PROTEIN CONTAINING PDZ DOMAIN, A K-BOX DOMAIN, AND A TPR REGION (ZKT)	Encodes a member of a novel plant protein family containing a PDZ, a K-box, and a TPR motif. mRNA but not protein levels decrease after wounding. ZKT is phosphorylated at Thr and Ser residues after wounding. The mRNA is cell-to-cell mobile.
AT2G38070	*1,19*		Protein of unknown function (DUF740); FUNCTIONS IN: molecular_function unknown; INVOLVED IN: biological_process unknown; LOCATED IN: plasma membrane; CONTAINS InterPro DOMAIN/s: Protein of unknown function DUF740 (InterPro:IPR008004); BEST Arabidopsis thaliana protein match is: Protein of unknown function (DUF740) (TAIR:AT3G09070.1)
AT5G20420	*1,19*	CHROMATIN REMODELING 42 (CHR42)	chromatin remodeling 42 (CHR42); FUNCTIONS IN: helicase activity, DNA binding, ATP binding, nucleic acid binding; EXPRESSED IN: shoot apex, embryo, flower, seed; EXPRESSED DURING: F mature embryo stage, petal differentiation and expansion stage, E expanded cotyledon stage, D bilateral stage; CONTAINS InterPro DOMAIN/s: DEAD-like helicase, N-terminal (InterPro:IPR014001), DNA/RNA helicase, C-terminal (InterPro:IPR001650), Helicase, superfamily 1/2, ATP-binding domain (InterPro:IPR014021), SNF2-related (InterPro:IPR000330); BEST Arabidopsis thaliana protein match is: chromatin remodeling 38 (TAIR:AT3G42670.1)
AT2G29970	*1,188*	SMAX1-LIKE 7 (SMXL7)	Encodes a member of an eight-gene family (SMAX1 and SMAX1-like) that has weak similarity to AtHSP101, a ClpB chaperonin required for thermotolerance. The mRNA is cell-to-cell mobile.
AT2G32640	*1,188*		Encodes a lycopene beta cyclase that catalyzes the addition of beta-ionone end groups to the end of lycopene molecules.
AT4G33070	*1,188*	PYRUVATE DECARBOXYLASE 1 (PDC1)	Thiamine pyrophosphate dependent pyruvate decarboxylase family protein; FUNCTIONS IN: in 6 functions; LOCATED IN: membrane; EXPRESSED IN: 11 plant structures; EXPRESSED DURING: 4 anthesis, C globular stage, F mature embryo stage, petal differentiation and expansion stage, E expanded cotyledon stage; CONTAINS InterPro DOMAIN/s: TPP-binding enzyme, conserved site (InterPro:IPR000399), Thiamine pyrophosphate enzyme, central domain (InterPro:IPR012000), Pyruvate decarboxylase/indolepyruvate decarboxylase (InterPro:IPR012110), Thiamine pyrophosphate enzyme, N-terminal TPP-binding domain (InterPro:IPR012001), Thiamine pyrophosphate enzyme, C-terminal TPP-binding (InterPro:IPR011766); BEST Arabidopsis thaliana protein match is: Thiamine pyrophosphate dependent pyruvate decarboxylase family protein (TAIR:AT5G01320.1)
AT3G50820	*1,187*	PHOTOSYSTEM II SUBUNIT O-2 (PSBO2)	Encodes a protein which is an extrinsic subunit of photosystem II and which has been proposed to play a central role in stabilization of the catalytic manganese cluster. In <i>Arabidopsis thaliana</i> the PsbO proteins are encoded by two genes: <i>psbO1</i> and <i>psbO2</i>. PsbO2 is the minor isoform in the wild-type. Mutants defective in this gene have been shown to be affected in the dephosphorylation of the D1 protein of PSII.
AT4G34480	*1,185*		O-Glycosyl hydrolases family 17 protein; FUNCTIONS IN: cation binding, hydrolase activity, hydrolyzing O-glycosyl compounds, catalytic activity; INVOLVED IN: carbohydrate metabolic process; LOCATED IN: endomembrane system; EXPRESSED IN: 22 plant structures; EXPRESSED DURING: 13 growth stages; CONTAINS InterPro DOMAIN/s: X8 (InterPro:IPR012946), Glycoside hydrolase, catalytic core (InterPro:IPR017853), Glycoside hydrolase, family 17 (InterPro:IPR000490), Glycoside hydrolase, subgroup, catalytic core (InterPro:IPR013781); BEST Arabidopsis thaliana protein match is: O-Glycosyl hydrolases family 17 protein (TAIR:AT2G16230.1)
AT1G57770	*1,182*		FAD/NAD(P)-binding oxidoreductase family protein; FUNCTIONS IN: oxidoreductase activity; LOCATED IN: chloroplast; EXPRESSED IN: 21 plant structures; EXPRESSED DURING: 13 growth stages; CONTAINS InterPro DOMAIN/s: Amine oxidase (InterPro:IPR002937); BEST Arabidopsis thaliana protein match is: carotenoid isomerase (TAIR:AT1G06820.1)
AT3G23820	*1,182*	UDP-D-GLUCURONATE 4-EPIMERASE 6 (GAE6)	Encodes a UDP-D-glucuronate 4-epimerase involved in pectin biosynthesis in the cell wall and affects cell wall integrity and immunity to fungi and bacteria. The mRNA is cell-to-cell mobile.
AT4G22470	*1,182*		protease inhibitor/seed storage/lipid transfer protein (LTP) family protein; FUNCTIONS IN: lipid binding; INVOLVED IN: lipid transport; LOCATED IN: endomembrane system; EXPRESSED IN: embryo, hypocotyl, sepal, stamen; EXPRESSED DURING: 4 anthesis, C globular stage, petal differentiation and expansion stage; CONTAINS InterPro DOMAIN/s: Bifunctional inhibitor/plant lipid transfer protein/seed storage (InterPro:IPR016140), Plant lipid transfer protein/hydrophobic protein, helical domain (InterPro:IPR013770); BEST Arabidopsis thaliana protein match is: Bifunctional inhibitor/lipid-transfer protein/seed storage 2S albumin superfamily protein (TAIR:AT4G22485.1)

AT4G39030	*1,182*	ENHANCED DISEASE SUSCEPTIBILITY 5 (EDS5)	Encodes an orphan multidrug and toxin extrusion transporter. Essential component of salicylic acid-dependent signaling for disease resistance. Member of the MATE-transporter family. Expression induced by salicylic acid. Mutants are salicylic acid-deficient.
AT5G20790	*1,182*		unknown protein; FUNCTIONS IN: molecular_function unknown; INVOLVED IN: biological_process unknown; LOCATED IN: endomembrane system; EXPRESSED IN: 11 plant structures; EXPRESSED DURING: LP.04 four leaves visible, 4 anthesis, petal differentiation and expansion stage; BEST Arabidopsis thaliana protein match is: unknown protein (TAIR:AT3G43110.1)
AT2G32510	*1,181*	MITOGEN-ACTIVATED PROTEIN KINASE KINASE KINASE 17 (MAPKKK17)	member of MEKK subfamily
AT1G74150	*1,179*		Galactose oxidase/kelch repeat superfamily protein; CONTAINS InterPro DOMAIN/s: Kelch repeat type 1 (InterPro:IPR006652), Galactose oxidase/kelch, beta-propeller (InterPro:IPR011043), Kelch repeat type 2 (InterPro:IPR011498), Kelch-type beta propeller (InterPro:IPR015915); BEST Arabidopsis thaliana protein match is: Galactose oxidase/kelch repeat superfamily protein (TAIR:AT1G18610.1)
AT4G36710	*1,179*	(HAM4)	GRAS family transcription factor; CONTAINS InterPro DOMAIN/s: Transcription factor GRAS (InterPro:IPR005202); BEST Arabidopsis thaliana protein match is: GRAS family transcription factor (TAIR:AT4G00150.1)
AT1G80840	*1,178*	WRKY DNA-BINDING PROTEIN 40 (WRKY40)	Pathogen-induced transcription factor. Binds W-box sequences in vitro. Forms protein complexes with itself and with WRKY40 and WRKY60. Coexpression with WRKY18 or WRKY60 made plants more susceptible to both P. syringae and B. cinerea. WRKY18, WRKY40, and WRKY60 have partially redundant roles in response to the hemibiotrophic bacterial pathogen Pseudomonas syringae and the necrotrophic fungal pathogen Botrytis cinerea, with WRKY18 playing a more important role than the other two. The mRNA is cell-to-cell mobile.
AT3G19830	*1,178*	(NTMC2T5.2)	NTMC2T5.2; FUNCTIONS IN: molecular_function unknown; INVOLVED IN: biological_process unknown; LOCATED IN: chloroplast; CONTAINS InterPro DOMAIN/s: Protein of unknown function DUF2404, transmembrane (InterPro:IPR019411), C2 membrane targeting protein (InterPro:IPR018029), C2 calcium/lipid-binding domain, CaLB (InterPro:IPR008973), C2 calcium-dependent membrane targeting (InterPro:IPR000008); BEST Arabidopsis thaliana protein match is: N-terminal-transmembrane-C2 domain type 5.1 (TAIR:AT1G50260.1)
AT5G48250	*1,178*	B-BOX DOMAIN PROTEIN 8 (BBX8)	B-box type zinc finger protein with CCT domain; FUNCTIONS IN: sequence-specific DNA binding transcription factor activity, zinc ion binding; LOCATED IN: plasma membrane; EXPRESSED IN: 23 plant structures; EXPRESSED DURING: 13 growth stages; CONTAINS InterPro DOMAIN/s: CCT domain (InterPro:IPR010402), Zinc finger, B-box (InterPro:IPR000315); BEST Arabidopsis thaliana protein match is: CONSTANS-like 9 (TAIR:AT3G07650.4)
AT5G48300	*1,177*	ADP GLUCOSE PYROPHOSPHORYLASE 1 (ADG1)	Encodes the small subunit of ADP-glucose pyrophosphorylase. The small subunit is the catalytic isoform responsible for ADP-glucose pyrophosphorylase activity. The presence of the small subunit is required for large subunit stability. Two isoforms of the small subunit (ApS1 and ApS2) have been described. ApS1 is the major small subunit isoform present in all plant tissues tested. The mRNA is cell-to-cell mobile.
AT2G35340	*1,176*	MATERNAL EFFECT EMBRYO ARREST 29 (MEE29)	maternal effect embryo arrest 29 (MEE29); FUNCTIONS IN: helicase activity, ATP-dependent RNA helicase activity, ATP binding, ATP-dependent helicase activity, nucleic acid binding; INVOLVED IN: embryo development ending in seed dormancy; LOCATED IN: endomembrane system; EXPRESSED IN: 22 plant structures; EXPRESSED DURING: 13 growth stages; CONTAINS InterPro DOMAIN/s: Helicase-associated domain (InterPro:IPR007502), DNA/RNA helicase, DEAD/DEAH box type, N-terminal (InterPro:IPR011545), Domain of unknown function DUF1605 (InterPro:IPR011709), DNA/RNA helicase, ATP-dependent, DEAH-box type, conserved site (InterPro:IPR002464), DEAD-like helicase, N-terminal (InterPro:IPR014001), DNA/RNA helicase, C-terminal (InterPro:IPR001650), Helicase, superfamily 1/2, ATP-binding domain (InterPro:IPR014021); BEST Arabidopsis thaliana protein match is: RNA helicase family protein (TAIR:AT1G32490.1)
AT1G21520	*1,174*		unknown protein

AT2G19940	*1,174*		oxidoreductases, acting on the aldehyde or oxo group of donors, NAD or NADP as acceptor;copper ion binding; FUNCTIONS IN: oxidoreductase activity, acting on the aldehyde or oxo group of donors, NAD or NADP as acceptor, copper ion binding; INVOLVED IN: response to cadmium ion, cellular amino acid metabolic process; LOCATED IN: nucleolus, chloroplast stroma, chloroplast, membrane; EXPRESSED IN: 25 plant structures; EXPRESSED DURING: 14 growth stages; CONTAINS InterPro DOMAIN/s: Semialdehyde dehydrogenase, dimerisation domain (InterPro:IPR012280), Semialdehyde dehydrogenase, NAD-binding (InterPro:IPR000534), N-acetyl-gamma-glutamyl-phosphate reductase (InterPro:IPR000706)
AT1G06520	*1,172*	GLYCEROL-3-PHOSPHATE SN-2-ACYLTRANSFERASE 1 (GPAT1)	sn-glycerol-3-phosphate 2-O-acyltransferase. Expressed in flower buds and siliques. Homozygous mutant plants are male sterile.
AT2G01340	*1,171*	(At17.1)	Encodes a protein whose expression is responsive to nematode infection.
AT2G37025	*1,171*	TRF-LIKE 8 (TRFL8)	TRF-like 8 (TRFL8); FUNCTIONS IN: DNA binding; INVOLVED IN: regulation of transcription; CONTAINS InterPro DOMAIN/s: SANT, DNA-binding (InterPro:IPR001005), Homeodomain-like (InterPro:IPR009057), HTH transcriptional regulator, Myb-type, DNA-binding (InterPro:IPR017930), Homeodomain-related (InterPro:IPR012287); BEST Arabidopsis thaliana protein match is: TRF-like 6 (TAIR:AT1G72650.2)
AT4G16860	*1,169*	RECOGNITION OF PERONOSPORA PARASITICA 4 (RPP4)	Confers resistance to Peronospora parasitica. RPP4 is coordinately regulated by transcriptional activation and RNA silencing.
AT4G35070	*1,166*		SBP (S-ribonuclease binding protein) family protein; CONTAINS InterPro DOMAIN/s: S-ribonuclease binding protein, SBP1, pollen (InterPro:IPR017066); BEST Arabidopsis thaliana protein match is: S-ribonuclease binding protein 1 (TAIR:AT1G45976.1)
AT3G20500	*1,165*	PURPLE ACID PHOSPHATASE 18 (PAP18)	purple acid phosphatase 18 (PAP18); FUNCTIONS IN: protein serine/threonine phosphatase activity, acid phosphatase activity; INVOLVED IN: biological_process unknown; LOCATED IN: endomembrane system; EXPRESSED IN: 24 plant structures; EXPRESSED DURING: 13 growth stages; CONTAINS InterPro DOMAIN/s: Metallophosphoesterase (InterPro:IPR004843), Purple acid phosphatase-like, N-terminal (InterPro:IPR008963); BEST Arabidopsis thaliana protein match is: purple acid phosphatase 22 (TAIR:AT3G52820.1)
AT3G49670	*1,165*	BARELY ANY MERISTEM 2 (BAM2)	Encodes a CLAVATA1-related receptor kinase-like protein required for both shoot and flower meristem function. Very similar to BAM1,with more than 85% a.a. identity. It has a broad expression pattern and is involved in vascular strand development in the leaf, control of leaf shape, size and symmetry, male gametophyte development and ovule specification and function. Anthers of double mutants (bam1bam2) appeared abnormal at a very early stage and lack the endothecium, middle, and tapetum layers. Further analyses revealed that cells interior to the epidermis (in anther tissue) acquire some characteristics of pollen mother cells (PMCs), suggesting defects in cell fate specification. The pollen mother-like cells degenerate before the completion of meiosis, suggesting that these cells are defective. In addition, the BAM2 expression pattern supports both an early role in promoting somatic cell fates and a subsequent function in the PMCs. The mRNA is cell-to-cell mobile.
AT4G23100	*1,163*	GLUTAMATE-CYSTEINE LIGASE (GSH1)	Encodes the enzyme glutamate-cysteine ligase catalyzing the first, and rate-limiting, step of glutathione biosynthesis. Required for cell proliferation at the root tip. Involved in susceptibility to the bacterial pathogen Pseudomonas syringae. Mutants are phytoalexin defective.
AT4G37670	*1,162*	N-ACETYL-L-GLUTAMATE SYNTHASE 2 (NAGS2)	N-acetyl-l-glutamate synthase 2 (NAGS2); FUNCTIONS IN: acetyl-CoA:L-glutamate N-acetyltransferase activity, N-acetyltransferase activity; INVOLVED IN: cellular amino acid biosynthetic process, arginine biosynthetic process, metabolic process; LOCATED IN: cytoplasm; EXPRESSED IN: 20 plant structures; EXPRESSED DURING: 12 growth stages; CONTAINS InterPro DOMAIN/s: GCN5-related N-acetyltransferase, C-terminal (InterPro:IPR022610), GCN5-related N-acetyltransferase (InterPro:IPR000182), Aspartate/glutamate/uridylate kinase (InterPro:IPR001048), Acyl-CoA N-acyltransferase (InterPro:IPR016181), Amino-acid N-acetyltransferase (ArgA) (InterPro:IPR010167); BEST Arabidopsis thaliana protein match is: N-acetyl-l-glutamate synthase 1 (TAIR:AT2G22910.1)
AT1G73080	*1,16*	PEP1 RECEPTOR 1 (PEPR1)	Encodes a leucine-rich repeat receptor kinase. Functions as a receptor for AtPep1 to amplify innate immunity response to pathogen attacks. The mRNA is cell-to-cell mobile.
AT2G22860	*1,16*	PHYTOSULFOKINE 2 PRECURSOR (PSK2)	Phytosulfokine 2 precursor, coding for a unique plant peptide growth factor. The mRNA is cell-to-cell mobile.

AT2G32990	*1,16*	GLYCOSYL HYDROLASE 9B8 (GH9B8)	glycosyl hydrolase 9B8 (GH9B8); FUNCTIONS IN: hydrolase activity, hydrolyzing O-glycosyl compounds, catalytic activity; INVOLVED IN: carbohydrate metabolic process; LOCATED IN: endomembrane system; EXPRESSED IN: 17 plant structures; EXPRESSED DURING: 11 growth stages; CONTAINS InterPro DOMAIN/s: Six-hairpin glycosidase (InterPro:IPR012341), Glycoside hydrolase, family 9, active site (InterPro:IPR018221), Six-hairpin glycosidase-like (InterPro:IPR008928), Glycoside hydrolase, family 9 (InterPro:IPR001701); BEST Arabidopsis thaliana protein match is: glycosyl hydrolase 9C2 (TAIR:AT1G64390.1)
AT2G34930	*1,16*		disease resistance family protein / LRR family protein; INVOLVED IN: signal transduction, defense response to fungus, defense response; LOCATED IN: cell wall; EXPRESSED IN: 10 plant structures; EXPRESSED DURING: 7 growth stages; CONTAINS InterPro DOMAIN/s: Leucine-rich repeat-containing N-terminal domain, type 2 (InterPro:IPR013210), Leucine-rich repeat (InterPro:IPR001611); BEST Arabidopsis thaliana protein match is: Leucine-rich repeat transmembrane protein kinase (TAIR:AT4G20140.1)
AT2G44840	*1,159*	ETHYLENE-RESPONSIVE ELEMENT BINDING FACTOR 13 (ERF13)	encodes a member of the ERF (ethylene response factor) subfamily B-3 of ERF/AP2 transcription factor family. The protein contains one AP2 domain. There are 18 members in this subfamily including ATERF-1, ATERF-2, AND ATERF-5. The mRNA is cell-to-cell mobile.
AT1G60260	*1,158*	BETA GLUCOSIDASE 5 (BGLU5)	beta glucosidase 5 (BGLU5); FUNCTIONS IN: cation binding, hydrolase activity, hydrolyzing O-glycosyl compounds, catalytic activity; INVOLVED IN: carbohydrate metabolic process; LOCATED IN: endomembrane system; CONTAINS InterPro DOMAIN/s: Glycoside hydrolase, family 1 (InterPro:IPR001360), Glycoside hydrolase, family 1, active site (InterPro:IPR018120), Glycoside hydrolase, catalytic core (InterPro:IPR017853), Glycoside hydrolase, subgroup, catalytic core (InterPro:IPR013781); BEST Arabidopsis thaliana protein match is: beta glucosidase 4 (TAIR:AT1G60090.1).
AT1G02730	*1,157*	CELLULOSE SYNTHASE-LIKE D5 (CSLD5)	Encodes a gene similar to cellulose synthase. Knock-out mutant has reduced growth, reduced xylan level and reduced xylan synthase activity in stems.It's expression is cell cycle dependent and it appears to function in cell plate formation.
AT1G49520	*1,157*		SWIB complex BAF60b domain-containing protein; CONTAINS InterPro DOMAIN/s: SWIB/MDM2 domain (InterPro:IPR003121), SWIB domain (InterPro:IPR019835), DEK, C-terminal (InterPro:IPR014876); BEST Arabidopsis thaliana protein match is: SWIB complex BAF60b domain-containing protein (TAIR:AT3G19080.1)
AT2G20250	*1,157*		unknown protein
AT1G26570	*1,155*	UDP-GLUCOSE DEHYDROGENASE 1 (UGD1)	UDP-glucose dehydrogenase 1 (UGD1); FUNCTIONS IN: in 7 functions; INVOLVED IN: oxidation reduction, metabolic process; LOCATED IN: endomembrane system; EXPRESSED IN: 23 plant structures; EXPRESSED DURING: 13 growth stages; CONTAINS InterPro DOMAIN/s: UDP-glucose/GDP-mannose dehydrogenase, N-terminal (InterPro:IPR001732), 6-phosphogluconate dehydrogenase, C-terminal-like (InterPro:IPR008927), Dehydrogenase, multihelical (InterPro:IPR013328), UDP-glucose/GDP-mannose dehydrogenase, dimerisation and substrate-binding domain (InterPro:IPR014028), UDP-glucose/GDP-mannose dehydrogenase, C-terminal (InterPro:IPR014027), NAD(P)-binding domain (InterPro:IPR016040), UDP-glucose/GDP-mannose dehydrogenase, dimerisation (InterPro:IPR014026), Nucleotide sugar dehydrogenase (InterPro:IPR017476); BEST Arabidopsis thaliana protein match is: UDP-glucose 6-dehydrogenase family protein (TAIR:AT3G29360.2)
AT4G03470	*1,155*		Ankyrin repeat family protein; EXPRESSED IN: cultured cell; CONTAINS InterPro DOMAIN/s: Ankyrin repeat-containing domain (InterPro:IPR020683), Ankyrin repeat (InterPro:IPR002110); BEST Arabidopsis thaliana protein match is: ankyrin repeat family protein (TAIR:AT4G05040.4)
AT1G17750	*1,152*	PEP1 RECEPTOR 2 (PEPR2)	Encodes PEPR2, a plasma membrane leucine-rich repeat receptor kinase functioning as a receptor for the Pep1 and Pep2 peptides. Pep1 and Pep2 are amino acids that induce the transcription of defense-related genes.
AT1G64540	*1,152*		F-box/FBD-like domains containing protein; CONTAINS InterPro DOMAIN/s: F-box domain, cyclin-like (InterPro:IPR001810), F-box domain, Skp2-like (InterPro:IPR022364), FBD-like (InterPro:IPR006566), Leucine-rich repeat 2 (InterPro:IPR013101); BEST Arabidopsis thaliana protein match is: F-box/RNI-like superfamily protein (TAIR:AT5G35995.2)
AT1G05000	*1,151*	PLANT AND FUNGI ATYPICAL DUAL-SPECIﬁCITY PHOSPHATASE 1 (PFA-DSP1)	Encodes an atypical dual-speciﬁcity phosphatase.
AT1G18250	*1,15*	(ATLP-1)	encodes a thaumatin-like protein

AT5G50890	*1,15*		alpha/beta-Hydrolases superfamily protein; BEST Arabidopsis thaliana protein match is: alpha/beta-Hydrolases superfamily protein (TAIR:AT2G05260.1)
AT1G30080	*1,149*		Glycosyl hydrolase superfamily protein; FUNCTIONS IN: cation binding, hydrolase activity, hydrolyzing O-glycosyl compounds, catalytic activity; INVOLVED IN: carbohydrate metabolic process; LOCATED IN: endomembrane system; EXPRESSED IN: 9 plant structures; EXPRESSED DURING: 4 anthesis, F mature embryo stage, petal differentiation and expansion stage, E expanded cotyledon stage, D bilateral stage; CONTAINS InterPro DOMAIN/s: Glycoside hydrolase, family 17 (InterPro:IPR000490), Glycoside hydrolase, catalytic core (InterPro:IPR017853), Glycoside hydrolase, subgroup, catalytic core (InterPro:IPR013781); BEST Arabidopsis thaliana protein match is: Glycosyl hydrolase superfamily protein (TAIR:AT4G18340.1)
AT5G41020	*1,149*		myb family transcription factor; FUNCTIONS IN: DNA binding, sequence-specific DNA binding transcription factor activity; CONTAINS InterPro DOMAIN/s: SANT, DNA-binding (InterPro:IPR001005), Homeodomain-like (InterPro:IPR009057), Myb, DNA-binding (InterPro:IPR014778), MYB-like (InterPro:IPR017877), Myb transcription factor (InterPro:IPR015495)
AT5G08400	*1,148*		Protein of unknown function (DUF3531); CONTAINS InterPro DOMAIN/s: Protein of unknown function DUF3531 (InterPro:IPR021920); BEST Arabidopsis thaliana protein match is: Protein of unknown function (DUF3531) (TAIR:AT4G29400.1)
AT5G27630	*1,148*	ACYL-COA BINDING PROTEIN 5 (ACBP5)	Acyl-CoA binding protein with high affinity for oleoyl-CoA. Expressed in all plant organs. Involved in fatty acid transport.
AT5G60610	*1,148*		F-box/RNI-like superfamily protein; CONTAINS InterPro DOMAIN/s: F-box domain, cyclin-like (InterPro:IPR001810), FBD (InterPro:IPR013596), F-box domain, Skp2-like (InterPro:IPR022364), Leucine-rich repeat 2 (InterPro:IPR013101); BEST Arabidopsis thaliana protein match is: FBD, F-box and Leucine Rich Repeat domains containing protein (TAIR:AT2G26860.1)
AT2G39490	*1,147*		F-box family protein; FUNCTIONS IN: molecular_function unknown; INVOLVED IN: biological_process unknown; LOCATED IN: endomembrane system; CONTAINS InterPro DOMAIN/s: F-box domain, cyclin-like (InterPro:IPR001810), F-box domain, Skp2-like (InterPro:IPR022364)
AT5G38520	*1,147*	CHLOROPHYLL DEPHYTYLASE1 (CLD1)	CLD1 is involved in steady-state chlorophyll turnover; CLD1 dephytylates chlorophyll a, chlorophyll b, and pheophytin a in vitro; CLD1 and CHLG form a salvage cycle in recycling chlorophyll. Suppression of CLD1 expression results in reduced tolerance to moderately high temperature.
AT1G69430	*1,146*		unknown protein; BEST Arabidopsis thaliana protein match is: unknown protein (TAIR:AT1G26650.1)
AT2G04230	*1,145*		FBD, F-box and Leucine Rich Repeat domains containing protein; CONTAINS InterPro DOMAIN/s: F-box domain, cyclin-like (InterPro:IPR001810), FBD (InterPro:IPR013596), F-box domain, Skp2-like (InterPro:IPR022364), FBD-like (InterPro:IPR006566), Leucine-rich repeat 2 (InterPro:IPR013101); BEST Arabidopsis thaliana protein match is: FBD, F-box and Leucine Rich Repeat domains containing protein (TAIR:AT3G49030.1)
AT4G32980	*1,145*	HOMEOBOX GENE 1 (ATH1)	Encodes transcription factor involved in photomorphogenesis. Regulates gibberellin biosynthesis. Activated by AGAMOUS in a cal-1, ap1-1 background. Expressed at low levels in developing stamens. Increased levels of ATH1 severely delay flowering in the C24 accession. Most remarkably, ectopically expressed ATH1 hardly had an effect on flowering time in the Col-0 and Ler accessions. ATH1 physically interacts with STM, BP and KNAT6 and enhances the shoot apical meristem defect of some of these genes suggesting a role in SAM maintenance. Nuclear localization is dependent upon interaction with STM.
AT2G29500	*1,144*		HSP20-like chaperones superfamily protein; CONTAINS InterPro DOMAIN/s: Heat shock protein Hsp20 (InterPro:IPR002068), HSP20-like chaperone (InterPro:IPR008978); BEST Arabidopsis thaliana protein match is: HSP20-like chaperones superfamily protein (TAIR:AT1G07400.1)
AT4G20050	*1,144*	QUARTET 3 (QRT3)	Encodes a polygalacturonase that plays a direct role in degrading the pollen mother cell wall during microspore development.

AT2G31010	*1,143*		Protein kinase superfamily protein; FUNCTIONS IN: protein serine/threonine kinase activity, protein kinase activity, kinase activity, ATP binding; INVOLVED IN: protein amino acid phosphorylation; LOCATED IN: cellular_component unknown; EXPRESSED IN: 19 plant structures; EXPRESSED DURING: 15 growth stages; CONTAINS InterPro DOMAIN/s: Protein kinase, ATP binding site (InterPro:IPR017441), Serine/threonine-protein kinase domain (InterPro:IPR002290), Serine-threonine/tyrosine-protein kinase (InterPro:IPR001245), Protein kinase-like domain (InterPro:IPR011009), Serine/threonine-protein kinase, active site (InterPro:IPR008271), Protein kinase, catalytic domain (InterPro:IPR000719), Tyrosine-protein kinase, catalytic domain (InterPro:IPR020635); BEST Arabidopsis thaliana protein match is: Mitogen activated protein kinase kinase kinase-related (TAIR:AT3G58640.2)
AT3G44720	*1,141*	AROGENATE DEHYDRATASE 4 (ADT4)	Encodes a plastid-localized arogenate dehydratase involved in phenylalanine biosynthesis. Not less than six genes encoding ADT were identiﬁed in the Arabidopsis genome: ADT1 [At1g11790]; ADT2 [At3g07630]; ADT3 [At2g27820]; ADT4 [At3g44720]; ADT5 [At5g22630]; and ADT6 [At1g08250]. The mRNA is cell-to-cell mobile.
AT5G02570	*1,141*		Histone superfamily protein; FUNCTIONS IN: DNA binding; INVOLVED IN: nucleosome assembly; LOCATED IN: nucleolus; EXPRESSED IN: 8 plant structures; EXPRESSED DURING: L mature pollen stage, M germinated pollen stage, 4 anthesis, petal differentiation and expansion stage; CONTAINS InterPro DOMAIN/s: Histone H2B (InterPro:IPR000558), Histone-fold (InterPro:IPR009072), Histone core (InterPro:IPR007125); BEST Arabidopsis thaliana protein match is: Histone superfamily protein (TAIR:AT2G37470.1)
AT1G56430	*1,139*	NICOTIANAMINE SYNTHASE 4 (NAS4)	Encodes a protein with nicotianamine synthase activity.
AT3G03530	*1,137*	NON-SPECIFIC PHOSPHOLIPASE C4 (NPC4)	PHOSPHOESTERASE FAMILY PROTEIN, NPC4 is significantly induced upon phosphate starvation and plays an important role in the supply of inorganic phosphate and diacylglycerol from membrane-phospholipids during phosphate deprivation.
AT4G21820	*1,137*		binding;calmodulin binding; FUNCTIONS IN: calmodulin binding, binding; LOCATED IN: chloroplast; EXPRESSED IN: 13 plant structures; EXPRESSED DURING: 6 growth stages; CONTAINS InterPro DOMAIN/s: Calponin-homology (InterPro:IPR016146), Armadillo (InterPro:IPR000225), Calponin-like actin-binding (InterPro:IPR001715), Armadillo-type fold (InterPro:IPR016024), IQ calmodulin-binding region (InterPro:IPR000048); BEST Arabidopsis thaliana protein match is: myosin 2 (TAIR:AT5G43900.1)
AT4G13310	*1,136*	CYTOCHROME P450, FAMILY 71, SUBFAMILY A, POLYPEPTIDE 20 (CYP71A20)	putative cytochrome P450
AT4G21780	*1,136*		unknown protein
AT1G19720	*1,135*		Pentatricopeptide repeat (PPR-like) superfamily protein; INVOLVED IN: biological_process unknown; LOCATED IN: nucleus, chloroplast, cytoplasm; CONTAINS InterPro DOMAIN/s: Pentatricopeptide repeat (InterPro:IPR002885); BEST Arabidopsis thaliana protein match is: Tetratricopeptide repeat (TPR)-like superfamily protein (TAIR:AT3G57430.1)
AT2G33580	*1,135*	LYSM-CONTAINING RECEPTOR-LIKE KINASE 5 (LYK5)	Encodes a putative LysM-containing receptor-like kinase. LYK5 is a major chitin receptor and forms a chitin-induced complex with related kinase CERK1. Based on protein sequence alignment analysis, it was determined as a pseudo kinase due to a lack of the ATP-binding P-loop in the kinase domain.
AT4G29670	*1,135*	ATYPICAL CYS HIS RICH THIOREDOXIN 2 (ACHT2)	Encodes a member of the thioredoxin family protein. Located in the chloroplast. Shows high activity towards the chloroplast 2-Cys peroxiredoxin A, and poor activity towards the chloroplast NADP-malate dehydrogenase.
AT1G03040	*1,134*		basic helix-loop-helix (bHLH) DNA-binding superfamily protein; FUNCTIONS IN: DNA binding, sequence-specific DNA binding transcription factor activity; INVOLVED IN: regulation of transcription, acetyl-CoA biosynthetic process from pyruvate; LOCATED IN: nucleus; EXPRESSED IN: 22 plant structures; EXPRESSED DURING: 13 growth stages; CONTAINS InterPro DOMAIN/s: Helix-loop-helix DNA-binding domain (InterPro:IPR001092), Helix-loop-helix DNA-binding (InterPro:IPR011598); BEST Arabidopsis thaliana protein match is: basic helix-loop-helix (bHLH) DNA-binding superfamily protein (TAIR:AT4G02590.2)

AT1G32220	1,134		NAD(P)-binding Rossmann-fold superfamily protein; FUNCTIONS IN: coenzyme binding, binding, catalytic activity; INVOLVED IN: response to oxidative stress; LOCATED IN: thylakoid, chloroplast, plastoglobule; EXPRESSED IN: 24 plant structures; EXPRESSED DURING: 15 growth stages; CONTAINS InterPro DOMAIN/s: NAD-dependent epimerase/dehydratase (InterPro:IPR001509), NAD(P)-binding domain (InterPro:IPR016040); BEST Arabidopsis thaliana protein match is: NAD(P)-binding Rossmann-fold superfamily protein (TAIR:AT5G10730.1)
AT5G65280	1,132	GCR2-LIKE 1 (GCL1)	Encodes a protein with reported similarity to GCR2 a putative G protein coupled receptor thought to be an ABA receptor. Loss of function mutations in GCL1 show no ABA response defects based on assays of seed germination and seedling development.GCL1 also has similarity to LANCL1 and LANCL2, human homologs of bacterial lanthionine synthetase.
AT1G72900	1,131		Toll-Interleukin-Resistance (TIR) domain-containing protein; FUNCTIONS IN: transmembrane receptor activity, ATP binding; INVOLVED IN: signal transduction, defense response, apoptosis, innate immune response; LOCATED IN: intrinsic to membrane; EXPRESSED IN: 20 plant structures; EXPRESSED DURING: 10 growth stages; CONTAINS InterPro DOMAIN/s: NB-ARC (InterPro:IPR002182), Toll-Interleukin receptor (InterPro:IPR000157); BEST Arabidopsis thaliana protein match is: Toll-Interleukin-Resistance (TIR) domain-containing protein (TAIR:AT1G72940.1)
AT2G30010	1,129	TRICHOME BIREFRINGENCE-LIKE 45 (TBL45)	Encodes a member of the TBL (TRICHOME BIREFRINGENCE-LIKE) gene family containing a plant-specific DUF231 (domain of unknown function) domain. TBL gene family has 46 members, two of which (TBR/AT5G06700 and TBL3/AT5G01360) have been shown to be involved in the synthesis and deposition of secondary wall cellulose, presumably by influencing the esterification state of pectic polymers. A nomenclature for this gene family has been proposed (Volker Bischoff & Wolf Scheible, 2010, personal communication).
AT3G08860	1,129	PYRIMIDINE 4 (PYD4)	Encodes a protein that is predicted to have beta-alanine aminotransferase activity.
AT1G04600	1,127	MYOSIN XI A (XIA)	member of Myosin-like proteins
AT5G53050	1,127		alpha/beta-Hydrolases superfamily protein; FUNCTIONS IN: hydrolase activity, catalytic activity; EXPRESSED IN: 24 plant structures; EXPRESSED DURING: 15 growth stages; CONTAINS InterPro DOMAIN/s: Epoxide hydrolase-like (InterPro:IPR000639), Alpha/beta hydrolase fold-1 (InterPro:IPR000073); BEST Arabidopsis thaliana protein match is: alpha/beta-Hydrolases superfamily protein (TAIR:AT4G02340.1)
AT2G39010	1,125	PLASMA MEMBRANE INTRINSIC PROTEIN 2E (PIP2E)	plasma membrane intrinsic protein 2E (PIP2E); FUNCTIONS IN: water channel activity; INVOLVED IN: transport, response to nematode; LOCATED IN: plasma membrane, membrane; EXPRESSED IN: 23 plant structures; EXPRESSED DURING: 13 growth stages; CONTAINS InterPro DOMAIN/s: Major intrinsic protein, conserved site (InterPro:IPR022357), Aquaporin (InterPro:IPR012269), Major intrinsic protein (InterPro:IPR000425); BEST Arabidopsis thaliana protein match is: plasma membrane intrinsic protein 2;5 (TAIR:AT3G54820.1)
AT3G45960	1,124	EXPANSIN-LIKE A3 (EXLA3)	member of EXPANSIN-LIKE. Naming convention from the Expansin Working Group (Kende et al, 2004. Plant Mol Bio)
AT3G47420	1,124	GLYCEROL-3-PHOSPHATE PERMEASE 1 (G3Pp1)	Encodes a Pi starvation-responsive protein AtPS3. A member of the phosphate starvation-induced glycerol-3-phosphate permease gene family: AT3G47420(G3Pp1), AT4G25220(G3Pp2), AT1G30560(G3Pp3), AT4G17550(G3Pp4) and AT2G13100(G3Pp5). Its expression is responsive to phosphate (Pi) and not phosphite (Phi) in roots and shoots.
AT1G20020	1,123	FERREDOXIN-NADP(+)-OXIDOREDUCTASE 2 (FNR2)	Encodes a leaf-type ferredoxin:NADP(H) oxidoreductase. It is present in both chloroplast stroma and thylakoid membranes but is more abundant in the stroma The mRNA is cell-to-cell mobile.
AT2G43500	1,123	NIN-LIKE PROTEIN 8 (NLP8)	Plant regulator RWP-RK family protein; CONTAINS InterPro DOMAIN/s: Octicosapeptide/Phox/Bem1p (InterPro:IPR000270), Plant regulator RWP-RK (InterPro:IPR003035); BEST Arabidopsis thaliana protein match is: Plant regulator RWP-RK family protein (TAIR:AT3G59580.2)
AT4G39980	1,123	3-DEOXY-D-ARABINO-HEPTULOSONATE 7-PHOSPHATE SYNTHASE 1 (DHS1)	Encodes a 2-deoxy-D-arabino-heptulosonate 7-phosphate (DAHP) synthase, which catalyzes the first committed step in aromatic amino acid biosynthesis. Gene expression is induced by wounding and pathogenic bacteria Pseudomonas syringae. The mRNA is cell-to-cell mobile.

AT5G59000	*1,121*		RING/FYVE/PHD zinc finger superfamily protein; FUNCTIONS IN: zinc ion binding; INVOLVED IN: biological_process unknown; LOCATED IN: cellular_component unknown; EXPRESSED IN: 21 plant structures; EXPRESSED DURING: 13 growth stages; CONTAINS InterPro DOMAIN/s: Zinc finger, C3HC4 RING-type (InterPro:IPR018957), Zinc finger, RING-CH-type (InterPro:IPR011016); BEST Arabidopsis thaliana protein match is: RING/FYVE/PHD zinc finger superfamily protein (TAIR:AT5G05830.1)
AT5G41550	*1,12*		Disease resistance protein (TIR-NBS-LRR class) family; FUNCTIONS IN: transmembrane receptor activity, nucleoside-triphosphatase activity, nucleotide binding, ATP binding; INVOLVED IN: signal transduction, defense response, apoptosis, innate immune response; LOCATED IN: intrinsic to membrane; EXPRESSED IN: root; CONTAINS InterPro DOMAIN/s: ATPase, AAA+ type, core (InterPro:IPR003593), NB-ARC (InterPro:IPR002182), Leucine-rich repeat (InterPro:IPR001611), Toll-Interleukin receptor (InterPro:IPR000157), Disease resistance protein (InterPro:IPR000767); BEST Arabidopsis thaliana protein match is: Disease resistance protein (TIR-NBS-LRR class) family (TAIR:AT5G41750.2)
AT5G41620	*1,12*		FUNCTIONS IN: molecular_function unknown; INVOLVED IN: biological_process unknown; LOCATED IN: chloroplast, plasma membrane; EXPRESSED IN: 9 plant structures; EXPRESSED DURING: 6 growth stages; BEST Arabidopsis thaliana protein match is: intracellular protein transport protein USO1-related (TAIR:AT1G64180.1)
AT4G38370	*1,118*		Phosphoglycerate mutase family protein; CONTAINS InterPro DOMAIN/s: Histidine phosphatase superfamily, clade-1 (InterPro:IPR013078)
AT1G60470	*1,117*	GALACTINOL SYNTHASE 4 (GolS4)	Predicted to encode a galactinol synthase.
AT4G19710	*1,116*	ASPARTATE KINASE-HOMOSERINE DEHYDROGENASE II (AK-HSDH II)	Encodes a bifunctional aspartate kinase/homoserine dehydrogenase. These two activities catalyze the first and the third steps toward the synthesis of the essential amino acids threonine, isoleucine and methionine.
AT5G20150	*1,114*	SPX DOMAIN GENE 1 (SPX1)	Expression is upregulated in the shoot of cax1/cax3 mutant. Additionally, its expression is responsive to both phosphate (Pi) and phosphite (Phi) in both roots and shoots. The mRNA is cell-to-cell mobile.
AT1G32860	*1,113*		Glycosyl hydrolase superfamily protein; FUNCTIONS IN: cation binding, hydrolase activity, hydrolyzing O-glycosyl compounds, catalytic activity; INVOLVED IN: carbohydrate metabolic process; LOCATED IN: anchored to plasma membrane, plasma membrane, anchored to membrane; EXPRESSED IN: 18 plant structures; EXPRESSED DURING: 12 growth stages; CONTAINS InterPro DOMAIN/s: Glycoside hydrolase, catalytic core (InterPro:IPR017853), Glycoside hydrolase, family 17 (InterPro:IPR000490), Glycoside hydrolase, subgroup, catalytic core (InterPro:IPR013781); BEST Arabidopsis thaliana protein match is: beta-1,3-glucanase_putative (TAIR:AT5G42100.1)
AT4G32810	*1,113*	CAROTENOID CLEAVAGE DIOXYGENASE 8 (CCD8)	Encodes a protein with similarity to carotenoid cleaving deoxygenases, the enzymes that cleave beta-carotene. Involved in the production of a graft transmissable signal to suppress axillary branching. Protein is localized to chloroplast stroma and expressed primarily in root tip. Mutants in the gene exhibit increased shoot branching, and light-dependent defects in hook opening and hypocotyl/root elongation. Only upregulated by auxin in the root and hypocotyl, and this is not required for the inhibition of shoot branching.
AT1G69490	*1,111*	NAC-LIKE, ACTIVATED BY AP3/PI (NAP)	Encodes a member of the NAC transcription factor gene family. It is expressed in floral primordia and upregulated by AP3 and PI. Its expression is associated with leaf senescence. The mRNA is cell-to-cell mobile.
AT4G34830	*1,111*	MATURATION OF RBCL 1 (MRL1)	Encodes MRL1, a conserved pentatricopeptide repeat protein, required for stabilization of rbcL mRNA.
AT2G39510	*1,11*	USUALLY MULTIPLE ACIDS MOVE IN AND OUT TRANSPORTERS 14 (UMAMIT14)	Encodes a plasma membrane-localized amino acid transporter likely involved in amino acid export in the developing seed.
AT2G36230	*1,108*	ALBINO AND PALE GREEN 10 (APG10)	Encodes a BBMII isomerase involved in histidine biosynthesis.

AT4G04020	*1,106*	FIBRILLIN (FIB)	Fibrillin precursor protein. The fibrillin preprotein, but not the mature protein interacts with ABI2. Regulated by abscisic acid response regulators. Involved in abscisic acid-mediated photoprotection. The mRNA is cell-to-cell mobile.
AT4G14330	*1,106*		P-loop containing nucleoside triphosphate hydrolases superfamily protein; FUNCTIONS IN: microtubule motor activity, ATP binding; INVOLVED IN: microtubule-based movement; LOCATED IN: phragmoplast; EXPRESSED IN: 13 plant structures; EXPRESSED DURING: 7 growth stages; CONTAINS InterPro DOMAIN/s: Kinesin, motor domain (InterPro:IPR001752); BEST Arabidopsis thaliana protein match is: kinesin 1 (TAIR:AT4G21270.1)
AT4G17000	*1,103*		unknown protein
AT1G51110	*1,102*		Plastid-lipid associated protein PAP / fibrillin family protein; FUNCTIONS IN: structural molecule activity; INVOLVED IN: tryptophan biosynthetic process; LOCATED IN: chloroplast thylakoid membrane, chloroplast, membrane; EXPRESSED IN: 21 plant structures; EXPRESSED DURING: 13 growth stages; CONTAINS InterPro DOMAIN/s: Plastid lipid-associated protein/fibrillin (InterPro:IPR006843); BEST Arabidopsis thaliana protein match is: Plastid-lipid associated protein PAP / fibrillin family protein (TAIR:AT3G26070.1)
AT3G61450	*1,101*	SYNTAXIN OF PLANTS 73 (SYP73)	syntaxin of plants 73 (SYP73)
AT1G07280	*1,098*		Tetratricopeptide repeat (TPR)-like superfamily protein; FUNCTIONS IN: binding; INVOLVED IN: biological_process unknown; LOCATED IN: chloroplast; EXPRESSED IN: 21 plant structures; EXPRESSED DURING: 13 growth stages; CONTAINS InterPro DOMAIN/s: Tetratricopeptide-like helical (InterPro:IPR011990), Tetratricopeptide repeat-containing (InterPro:IPR013026); BEST Arabidopsis thaliana protein match is: Tetratricopeptide repeat (TPR)-like superfamily protein (TAIR:AT2G29670.1)
AT1G14890	*1,098*		Plant invertase/pectin methylesterase inhibitor superfamily protein; FUNCTIONS IN: enzyme inhibitor activity, pectinesterase inhibitor activity, pectinesterase activity; INVOLVED IN: biological_process unknown; EXPRESSED IN: 22 plant structures; EXPRESSED DURING: 13 growth stages; CONTAINS InterPro DOMAIN/s: Pectinesterase inhibitor (InterPro:IPR006501); BEST Arabidopsis thaliana protein match is: Plant invertase/pectin methylesterase inhibitor superfamily protein (TAIR:AT2G01610.1)
AT1G19850	*1,098*	MONOPTEROS (MP)	Encodes a transcription factor (IAA24) mediating embryo axis formation and vascular development. Similar to AUXIN RESPONSIVE FACTOR 1 (ARF1) shown to bind to auxin responsive elements (AREs), and to the maize transcriptional activator VIVIPAROUS 1(VP1). In situ hybridization shows expression in provascular tissue of embryos, the emerging shoot primordia, then is restricted to provascular tissue, and in the root central vascular cylinder.
AT3G53160	*1,098*	UDP-GLUCOSYL TRANSFERASE 73C7 (UGT73C7)	UDP-glucosyl transferase 73C7 (UGT73C7); CONTAINS InterPro DOMAIN/s: UDP-glucuronosyl/UDP-glucosyltransferase (InterPro:IPR002213); BEST Arabidopsis thaliana protein match is: UDP-Glycosyltransferase superfamily protein (TAIR:AT2G36770.1)
AT3G26650	*1,095*	GLYCERALDEHYDE 3-PHOSPHATE DEHYDROGENASE A SUBUNIT (GAPA)	Encodes one of the two subunits forming the photosynthetic glyceraldehyde-3-phosphate dehydrogenase (GAPDH) and as such a constituent of the supramolecular complex with phosphoribulokinase (PRK) thought to be linked by a small peptide encoded by CP12-2. GapA-1 is coordinately expressed by light with PRK and CP12-2. The enzyme activity, tested in leaf protein extracts dropped significantly after external sucrose treatment for the photosynthetic GAPDH (NADPH-dependent) but not for the cytosolic GAPDH (NADH-dependent).
AT5G43420	*1,095*		RING/U-box superfamily protein; FUNCTIONS IN: zinc ion binding; CONTAINS InterPro DOMAIN/s: Zinc finger, RING-type (InterPro:IPR001841), Zinc finger, C3HC4 RING-type (InterPro:IPR018957); BEST Arabidopsis thaliana protein match is: RING/U-box superfamily protein (TAIR:AT1G04360.1)
AT5G63770	*1,095*	DIACYLGLYCEROL KINASE 2 (DGK2)	a member of the diacylglycerol kinase gene family. Encodes a functional diacylglycerol kinase. Involved in root elongation and plant development. Gene expression is induced by wounding or cold.
AT4G30110	*1,093*	HEAVY METAL ATPASE 2 (HMA2)	encodes a protein similar to Zn-ATPase, a P1B-type ATPases transport zinc
AT5G18470	*1,093*		Curculin-like (mannose-binding) lectin family protein; FUNCTIONS IN: sugar binding; INVOLVED IN: response to karrikin; LOCATED IN: plant-type cell wall; EXPRESSED IN: 22 plant structures; EXPRESSED DURING: 12 growth stages; CONTAINS InterPro DOMAIN/s: Curculin-like (mannose-binding) lectin (InterPro:IPR001480); BEST Arabidopsis thaliana protein match is: lectin protein kinase family protein (TAIR:AT1G67520.1)

AT5G21100	*1,091*		Plant L-ascorbate oxidase; FUNCTIONS IN: oxidoreductase activity, copper ion binding, L-ascorbate oxidase activity; INVOLVED IN: oxidation reduction; LOCATED IN: plant-type cell wall; EXPRESSED IN: 21 plant structures; EXPRESSED DURING: 13 growth stages; CONTAINS InterPro DOMAIN/s: Multicopper oxidase, type 3 (InterPro:IPR011707), Multicopper oxidase, type 2 (InterPro:IPR011706), Cupredoxin (InterPro:IPR008972), Multicopper oxidase, copper-binding site (InterPro:IPR002355), Multicopper oxidase, type 1 (InterPro:IPR001117), L-ascorbate oxidase, plants (InterPro:IPR017760); BEST Arabidopsis thaliana protein match is: Plant L-ascorbate oxidase (TAIR:AT5G21105.1)
AT3G07970	*1,09*	QUARTET 2 (QRT2)	Required for pollen separation during normal development. In qrt mutants, the outer walls of the four meiotic products of the pollen mother cell are fused, and pollen grains are released in tetrads.May be required for cell type-specific pectin degradation.
AT4G28320	*1,09*	ENDO-BETA-MANNASE 5 (MAN5)	Encodes a endo-beta-mannanase involved in seed germination.
AT4G19230	*1,089*	CYTOCHROME P450, FAMILY 707, SUBFAMILY A, POLYPEPTIDE 1 (CYP707A1)	Encodes a protein with ABA 8'-hydroxylase activity, involved in ABA catabolism. Member of the CYP707A gene family. CYP707A1 appears to play an important role in determining the ABA levels in dry seeds. Gene involved in postgermination growth. Overexpression of CYP707A1 leads to a decrease in ABA levels and a reduction in after-ripening period to break dormancy.
AT5G24655	*1,089*	RESPONSE TO LOW SULFUR 4 (LSU4)	RESPONSE TO LOW SULFUR 4 (LSU4); BEST Arabidopsis thaliana protein match is: response to low sulfur 2 (TAIR:AT5G24660.1)
AT2G04530	*1,088*	(CPZ)	Encodes a protein with RNAse Z activity suggesting a role in tRNA processing. Protein contains a signal sequence for import into the chloroplast.
AT4G34220	*1,088*	RECEPTOR DEAD KINASE1 (RDK1)	Encodes a receptor like kinase involved in ABA-mediated seedling development and drought tolerance.RDK1 is an atypical or pseudokinase and has no phosphorylation activity. Its expression is upregulated in response to ABA.interacts with ABI1 and other PP2C phosphatases.
AT3G03770	*1,087*		Leucine-rich repeat protein kinase family protein; FUNCTIONS IN: protein serine/threonine kinase activity, protein kinase activity, ATP binding; INVOLVED IN: transmembrane receptor protein tyrosine kinase signaling pathway, protein amino acid phosphorylation; LOCATED IN: endomembrane system; EXPRESSED IN: 22 plant structures; EXPRESSED DURING: 13 growth stages; CONTAINS InterPro DOMAIN/s: Protein kinase, catalytic domain (InterPro:IPR000719), Leucine-rich repeat (InterPro:IPR001611), Serine/threonine-protein kinase-like domain (InterPro:IPR017442), Protein kinase-like domain (InterPro:IPR011009); BEST Arabidopsis thaliana protein match is: Leucine-rich repeat protein kinase family protein (TAIR:AT5G14210.1)
AT4G28430	*1,087*		Reticulon family protein; INVOLVED IN: biological_process unknown; LOCATED IN: endoplasmic reticulum, chloroplast; EXPRESSED IN: 11 plant structures; EXPRESSED DURING: 6 growth stages; CONTAINS InterPro DOMAIN/s: Reticulon (InterPro:IPR003388); BEST Arabidopsis thaliana protein match is: Reticulon family protein (TAIR:AT2G20590.1)
AT5G05740	*1,087*	ETHYLENE-DEPENDENT GRAVITROPISM-DEFICIENT AND YELLOW-GREEN-LIKE 2 (EGY2)	S2P-like putative metalloprotease, also contain transmembrane helices near their C-termini and many of them, five of seven, contain a conserved zinc-binding motif HEXXH. Homolog of EGY1. Each of the EGY1 and EGY-like proteins share two additional highly conserved motifs, the previously reported NPDG motif (aa 442?454 in EGY1, Rudner et al., 1999) and a newly defined GNLR motif (aa 171?179 in EGY1). The GNLR motif is a novel signature motif unique to EGY1 and EGY-like proteins as well as other EGY1 orthologs found in cyanobacteria.
AT5G24010	*1,086*		Protein kinase superfamily protein; FUNCTIONS IN: protein serine/threonine kinase activity, protein kinase activity, kinase activity, ATP binding; INVOLVED IN: protein amino acid phosphorylation; LOCATED IN: plasma membrane; EXPRESSED IN: 23 plant structures; EXPRESSED DURING: 13 growth stages; CONTAINS InterPro DOMAIN/s: Protein kinase, ATP binding site (InterPro:IPR017441), Protein kinase, catalytic domain (InterPro:IPR000719), Serine-threonine/tyrosine-protein kinase (InterPro:IPR001245), Protein kinase-like domain (InterPro:IPR011009), Serine/threonine-protein kinase, active site (InterPro:IPR008271); BEST Arabidopsis thaliana protein match is: hercules receptor kinase 1 (TAIR:AT3G46290.1)

AT1G17230	*1,085*		Leucine-rich receptor-like protein kinase family protein; FUNCTIONS IN: protein serine/threonine kinase activity, protein kinase activity, ATP binding; INVOLVED IN: protein amino acid phosphorylation; LOCATED IN: endomembrane system; EXPRESSED IN: 11 plant structures; EXPRESSED DURING: 6 growth stages; CONTAINS InterPro DOMAIN/s: Serine/threonine-protein kinase domain (InterPro:IPR002290), Leucine-rich repeat-containing N-terminal domain, type 2 (InterPro:IPR013210), Leucine-rich repeat (InterPro:IPR001611), Serine/threonine-protein kinase-like domain (InterPro:IPR017442), Serine/threonine-protein kinase, active site (InterPro:IPR008271), Protein kinase-like domain (InterPro:IPR011009), Protein kinase, catalytic domain (InterPro:IPR000719), Leucine-rich repeat, typical subtype (InterPro:IPR003591), Tyrosine-protein kinase, catalytic domain (InterPro:IPR020635); BEST Arabidopsis thaliana protein match is: Leucine-rich repeat protein kinase family protein (TAIR:AT5G63930.1)
AT1G28190	*1,085*		unknown protein; BEST Arabidopsis thaliana protein match is: unknown protein (TAIR:AT5G12340.1)
AT3G44310	*1,085*	NITRILASE 1 (NIT1)	Mutants are resistant to indole-3-acetonitrile (IAN). NIT1 catalyzes the terminal activation step in indole-acetic acid biosynthesis. Predominantly expressed isoform of nitrilase isoenzyme family. Aggregation of NIT1 in cells directly abutting wound sites is one of the earliest events associated with wound and herbicide-induced cell death. The protein undergoes thiolation following treatment with the oxidant tert-butylhydroperoxide. It is also involved in the conversion of IAN to IAM (indole-3-acetamide) and other non-auxin-related metabolic processes. The mRNA is cell-to-cell mobile.
AT3G02930	*1,084*		Encodes a microtubule-associated protein.
AT1G11420	*1,083*	DOMAIN OF UNKNOWN FUNCTION 724 2 (DUF2)	Member of the plant-specific DUF724 protein family. Arabidopsis has 10 DUF724 proteins.
AT1G51700	*1,083*	DOF ZINC FINGER PROTEIN 1 (DOF1)	Encodes dof zinc finger protein (adof1). The mRNA is cell-to-cell mobile.
AT1G06690	*1,082*		NAD(P)-linked oxidoreductase superfamily protein; FUNCTIONS IN: oxidoreductase activity, aldo-keto reductase activity; INVOLVED IN: oxidation reduction; LOCATED IN: chloroplast thylakoid membrane, chloroplast, plastoglobule, chloroplast envelope; EXPRESSED IN: 22 plant structures; EXPRESSED DURING: 14 growth stages; CONTAINS InterPro DOMAIN/s: Aldo/keto reductase (InterPro:IPR001395), Aldo/keto reductase subgroup (InterPro:IPR020471), Aldo/keto reductase, conserved site (InterPro:IPR018170); BEST Arabidopsis thaliana protein match is: NAD(P)-linked oxidoreductase superfamily protein (TAIR:AT5G53580.1)
AT5G17850	*1,081*		Sodium/calcium exchanger family protein; FUNCTIONS IN: cation:cation antiporter activity, calcium:sodium antiporter activity; INVOLVED IN: cation transport, transmembrane transport; LOCATED IN: endomembrane system, integral to membrane; EXPRESSED IN: 19 plant structures; EXPRESSED DURING: 12 growth stages; CONTAINS InterPro DOMAIN/s: Sodium/calcium exchanger membrane region (InterPro:IPR004837); BEST Arabidopsis thaliana protein match is: calcium exchanger 7 (TAIR:AT5G17860.1)
AT5G20480	*1,08*	EF-TU RECEPTOR (EFR)	Encodes a predicted leucine-rich repeat receptor kinase (LRR-RLK). Functions as the receptor for bacterial PAMP (pathogen associated molecular patterns) EF-Tu.
AT5G06860	*1,079*	POLYGALACTURONASE INHIBITING PROTEIN 1 (PGIP1)	Encodes a polygalacturonase inhibiting protein involved in defense response. PGIPs inhibit the function of cell wall pectin degrading enzymes such as those produced by fungal pathogens. PGIP1 is induced by fungal infection. Suppressed in the proton sensitive stop1-mutant, but the transcription level was recovered by transformation of STOP2. Knockout mutant showed severe damage in the root tip in low Ca and low pH medium.
AT5G13280	*1,079*	ASPARTATE KINASE 1 (AK-LYS1)	Asp kinase inhibited by Lys and S-adenosylmethionine. Contains regulatory domains that belong to the ACT domain family, which allow binding to a extreme variety of ligands. Can function as a monomer or as a dimer with acetohydroxyacid synthase (HSDH).
AT5G38980	*1,078*		unknown protein; FUNCTIONS IN: molecular_function unknown; INVOLVED IN: biological_process unknown; LOCATED IN: endomembrane system; EXPRESSED IN: 22 plant structures; EXPRESSED DURING: 13 growth stages
AT5G66840	*1,077*		SAP domain-containing protein; FUNCTIONS IN: DNA binding, nucleic acid binding; LOCATED IN: nucleus; CONTAINS InterPro DOMAIN/s: DNA-binding SAP (InterPro:IPR003034)

AT5G22630	*1,076*	AROGENATE DEHYDRATASE 5 (ADT5)	Encodes a plastid-localized arogenate dehydratase involved in phenylalanine biosynthesis. Not less than six genes encoding ADT were identiﬁed in the Arabidopsis genome: ADT1 [At1g11790]; ADT2 [At3g07630]; ADT3 [At2g27820]; ADT4 [At3g44720]; ADT5 [At5g22630]; and ADT6 [At1g08250]. The mRNA is cell-to-cell mobile.
AT5G42310	*1,076*		Pentatricopeptide repeat (PPR-like) superfamily protein; LOCATED IN: chloroplast; EXPRESSED IN: 23 plant structures; EXPRESSED DURING: 13 growth stages; CONTAINS InterPro DOMAIN/s: Pentatricopeptide repeat (InterPro:IPR002885); BEST Arabidopsis thaliana protein match is: Pentatricopeptide repeat (PPR) superfamily protein (TAIR:AT3G22470.1)
AT1G77390	*1,074*	CYCLIN A1;2 (CYCA1;2)	Encodes a core cell cycle gene involved in meiosis II during microsporogenesis. Recessive mutants exhibit delayed and asynchronous meiosis in pollen mother cell populations and uncoordinated nuclear division and cytokinesis resulting in dyad microspores.
AT3G12915	*1,074*		Ribosomal protein S5/Elongation factor G/III/V family protein; FUNCTIONS IN: GTP binding, GTPase activity; INVOLVED IN: biological_process unknown; LOCATED IN: cellular_component unknown; CONTAINS InterPro DOMAIN/s: Translation elongation factor EFTu/EF1A, domain 2 (InterPro:IPR004161), Translation elongation factor EFG/EF2, C-terminal (InterPro:IPR000640), Ribosomal protein S5 domain 2-type fold (InterPro:IPR020568), Protein synthesis factor, GTP-binding (InterPro:IPR000795), Elongation factor G/III/V (InterPro:IPR009022), Translation elongation/initiation factor/Ribosomal, beta-barrel (InterPro:IPR009000), Translation elongation factor EFG/EF2, domain IV (InterPro:IPR005517); BEST Arabidopsis thaliana protein match is: Ribosomal protein S5/Elongation factor G/III/V family protein (TAIR:AT1G56070.1)
AT4G11350	*1,074*		Protein of unknown function (DUF604); CONTAINS InterPro DOMAIN/s: Protein of unknown function DUF604 (InterPro:IPR006740); BEST Arabidopsis thaliana protein match is: Protein of unknown function (DUF604) (TAIR:AT4G23490.1)
AT5G01340	*1,074*	MITOCHONDRIAL SUCCINATE-FUMARATE CARRIER 1 (mSFC1)	Mitochondrial substrate carrier family protein; FUNCTIONS IN: transporter activity, binding; INVOLVED IN: transport, mitochondrial transport, transmembrane transport; LOCATED IN: mitochondrial inner membrane, membrane; EXPRESSED IN: 23 plant structures; EXPRESSED DURING: 15 growth stages; CONTAINS InterPro DOMAIN/s: Mitochondrial substrate carrier (InterPro:IPR001993), Mitochondrial substrate/solute carrier (InterPro:IPR018108), Adenine nucleotide translocator 1 (InterPro:IPR002113); BEST Arabidopsis thaliana protein match is: Mitochondrial substrate carrier family protein (TAIR:AT2G37890.1)
AT1G49700	*1,073*		Plant protein 1589 of unknown function; CONTAINS InterPro DOMAIN/s: Conserved hypothetical protein CHP01589, plant (InterPro:IPR006476); BEST Arabidopsis thaliana protein match is: unknown protein (TAIR:AT4G07675.1)
AT1G24735	*1,072*		S-adenosyl-L-methionine-dependent methyltransferases superfamily protein; FUNCTIONS IN: O-methyltransferase activity; LOCATED IN: cytosol; EXPRESSED IN: stem, leaf apex, root, flower; EXPRESSED DURING: petal differentiation and expansion stage; CONTAINS InterPro DOMAIN/s: O-methyltransferase, family 3 (InterPro:IPR002935); BEST Arabidopsis thaliana protein match is: caffeoyl-CoA 3-O-methyltransferase (TAIR:AT1G67980.1).
AT1G53060	*1,072*		Legume lectin family protein; FUNCTIONS IN: carbohydrate binding, binding; INVOLVED IN: biological_process unknown; LOCATED IN: cellular_component unknown; EXPRESSED IN: 21 plant structures; EXPRESSED DURING: 13 growth stages; CONTAINS InterPro DOMAIN/s: Legume lectin, beta chain (InterPro:IPR001220), Concanavalin A-like lectin/glucanase, subgroup (InterPro:IPR013320), Concanavalin A-like lectin/glucanase (InterPro:IPR008985), Lectin (InterPro:IPR016363); BEST Arabidopsis thaliana protein match is: Legume lectin family protein (TAIR:AT1G53080.1)
AT4G16680	*1,072*		P-loop containing nucleoside triphosphate hydrolases superfamily protein; FUNCTIONS IN: RNA helicase activity, helicase activity, nucleic acid binding, ATP binding, ATP-dependent helicase activity; LOCATED IN: cellular_component unknown; EXPRESSED IN: stem, sepal, male gametophyte, carpel, stamen; EXPRESSED DURING: 4 anthesis, petal differentiation and expansion stage; CONTAINS InterPro DOMAIN/s: DNA/RNA helicase, DEAD/DEAH box type, N-terminal (InterPro:IPR011545), DNA/RNA helicase, ATP-dependent, DEAH-box type, conserved site (InterPro:IPR002464), DEAD-like helicase, N-terminal (InterPro:IPR014001), DNA/RNA helicase, C-terminal (InterPro:IPR001650), Helicase, superfamily 1/2, ATP-binding domain (InterPro:IPR014021); BEST Arabidopsis thaliana protein match is: RNA helicase family protein (TAIR:AT1G32490.1)
AT5G12010	*1,072*		unknown protein; INVOLVED IN: response to salt stress; LOCATED IN: chloroplast, plasma membrane, membrane; EXPRESSED IN: 23 plant structures; EXPRESSED DURING: 13 growth stages; BEST Arabidopsis thaliana protein match is: unknown protein (TAIR:AT4G29780.1)

AT1G54570	*1,07*	PHYTYL ESTER SYNTHASE 1 (PES1)	Encodes a protein with phytyl ester synthesis and diacylglycerol acyltransferase activities that is involved in the deposition of free phytol and free fatty acids in the form of phytyl esters in chloroplasts, a process involved in maintaining the integrity of the photosynthetic membrane during abiotic stress and senescence.
AT1G22650	*1,068*	ALKALINE/NEUTRAL INVERTASE D (A/N-InvD)	Plant neutral invertase family protein; FUNCTIONS IN: catalytic activity, beta-fructofuranosidase activity; INVOLVED IN: sucrose catabolic process, using beta-fructofuranosidase; LOCATED IN: cellular_component unknown; EXPRESSED IN: 19 plant structures; EXPRESSED DURING: 14 growth stages; CONTAINS InterPro DOMAIN/s: Plant neutral invertase (InterPro:IPR006937), Six-hairpin glycosidase-like (InterPro:IPR008928); BEST Arabidopsis thaliana protein match is: cytosolic invertase 2 (TAIR:AT4G09510.1)
AT2G33770	*1,066*	PHOSPHATE 2 (PHO2)	Encodes a ubiquitin-conjugating E2 enzyme. UBC24 mRNA accumulation is suppressed by miR399f, miR399b and miR399c. Involved in phosphate starvation response and mediates degradation of PHO1 and PHT1s at endomembrane. Its expression is responsive to phosphate (Pi) and not phosphite (Phi) in roots and shoots. The mRNA is cell-to-cell mobile.
AT1G74440	*1,065*		Protein of unknown function (DUF962); CONTAINS InterPro DOMAIN/s: Protein of unknown function DUF962 (InterPro:IPR009305); BEST Arabidopsis thaliana protein match is: Protein of unknown function (DUF962) (TAIR:AT1G18720.1)
AT3G06630	*1,064*		protein kinase family protein; FUNCTIONS IN: two-component sensor activity, protein serine/threonine kinase activity, protein kinase activity, signal transducer activity, ATP binding; INVOLVED IN: signal transduction, protein amino acid phosphorylation, regulation of transcription, DNA-dependent, two-component signal transduction system (phosphorelay); EXPRESSED IN: sperm cell; CONTAINS InterPro DOMAIN/s: PAC motif (InterPro:IPR001610), PAS fold (InterPro:IPR013767), PAS (InterPro:IPR000014), Serine-threonine/tyrosine-protein kinase (InterPro:IPR001245), PAS-associated, C-terminal (InterPro:IPR000700), Protein kinase-like domain (InterPro:IPR011009), Serine/threonine-protein kinase, active site (InterPro:IPR008271), Protein kinase, catalytic domain (InterPro:IPR000719); BEST Arabidopsis thaliana protein match is: PAS domain-containing protein tyrosine kinase family protein (TAIR:AT3G06620.1)
AT5G27110	*1,064*		Tetratricopeptide repeat (TPR)-like superfamily protein; CONTAINS InterPro DOMAIN/s: Pentatricopeptide repeat (InterPro:IPR002885); BEST Arabidopsis thaliana protein match is: Pentatricopeptide repeat (PPR) superfamily protein (TAIR:AT4G18750.1)
AT2G23670	*1,062*	HOMOLOG OF SYNECHOCYSTIS YCF37 (YCF37)	homolog of Synechocystis YCF37 (YCF37); FUNCTIONS IN: molecular_function unknown; INVOLVED IN: biological_process unknown; LOCATED IN: thylakoid, chloroplast thylakoid membrane, chloroplast thylakoid lumen, chloroplast; EXPRESSED IN: 20 plant structures; EXPRESSED DURING: 13 growth stages
AT5G24300	*1,062*	STARCH SYNTHASE 1 (SS1)	SSI is a plastidial enzyme and crucial for the synthesis of normal amylopectin in the leaves of Arabidopsis. The absence of SSI results in a deficiency in the number of shorter glucans which in turn affect the formation and connection of the amylopectin clusters in starch.
AT2G35040	*1,061*		AICARFT/IMPCHase bienzyme family protein; FUNCTIONS IN: phosphoribosylaminoimidazolecarboxamide formyltransferase activity, IMP cyclohydrolase activity, catalytic activity; INVOLVED IN: response to cold, purine nucleotide biosynthetic process; LOCATED IN: stromule, chloroplast, chloroplast stroma; EXPRESSED IN: 23 plant structures; EXPRESSED DURING: 14 growth stages; CONTAINS InterPro DOMAIN/s: AICARFT/IMPCHase bienzyme, transformylase domain (InterPro:IPR013982), AICARFT/IMPCHase bienzyme (InterPro:IPR002695), MGS-like (InterPro:IPR011607)
AT4G18890	*1,061*	BES1/BZR1 HOMOLOG 3 (BEH3)	BES1/BZR1 homolog 3 (BEH3); CONTAINS InterPro DOMAIN/s: BZR1, transcriptional repressor (InterPro:IPR008540); BEST Arabidopsis thaliana protein match is: BES1/BZR1 homolog 4 (TAIR:AT1G78700.1)
AT4G23000	*1,061*		Calcineurin-like metallo-phosphoesterase superfamily protein; FUNCTIONS IN: hydrolase activity, protein serine/threonine phosphatase activity; INVOLVED IN: biological_process unknown; LOCATED IN: cellular_component unknown; EXPRESSED IN: 22 plant structures; EXPRESSED DURING: 13 growth stages; CONTAINS InterPro DOMAIN/s: Metallophosphoesterase (InterPro:IPR004843); BEST Arabidopsis thaliana protein match is: Calcineurin-like metallo-phosphoesterase superfamily protein (TAIR:AT4G11800.1)

AT2G24820	*1,06*	TRANSLOCON AT THE INNER ENVELOPE MEMBRANE OF CHLOROPLASTS 55-II (TIC55-II)	translocon at the inner envelope membrane of chloroplasts 55-II (TIC55-II); FUNCTIONS IN: oxidoreductase activity, 2 iron, 2 sulfur cluster binding, chlorophyllide a oxygenase [overall] activity; INVOLVED IN: protein targeting to chloroplast; LOCATED IN: chloroplast, chloroplast envelope; EXPRESSED IN: 23 plant structures; EXPRESSED DURING: 13 growth stages; CONTAINS InterPro DOMAIN/s: Rieske [2Fe-2S] iron-sulphur domain (InterPro:IPR017941), Pheophorbide a oxygenase (InterPro:IPR013626); BEST Arabidopsis thaliana protein match is: Pheophorbide a oxygenase family protein with Rieske [2Fe-2S] domain (TAIR:AT3G44880.1)
AT2G41180	*1,06*	SIGMA FACTOR BINDING PROTEIN 2 (SIB2)	VQ motif-containing protein; FUNCTIONS IN: molecular_function unknown; INVOLVED IN: biological_process unknown; LOCATED IN: chloroplast; EXPRESSED IN: 22 plant structures; EXPRESSED DURING: 13 growth stages; CONTAINS InterPro DOMAIN/s: VQ (InterPro:IPR008889); BEST Arabidopsis thaliana protein match is: sigma factor binding protein 1 (TAIR:AT3G56710.1)
AT2G38940	*1,059*	PHOSPHATE TRANSPORTER 1;4 (PHT1;4)	Encodes Pht1;4, a member of the Pht1 family of phosphate transporters which include: Pht1;1/At5g43350, Pht1;2/At5g43370, Pht1;3/At5g43360, Pht1;4/At2g38940, Pht1;5/At2g32830, Pht1;6/At5g43340, Pht1;7/At3g54700, Pht1;8/At1g20860, Pht1;9/At1g76430 (Plant Journal 2002, 31:341). Expression is upregulated in the shoot of cax1/cax3 mutant and is responsive to phosphate (Pi) and not phosphite (Phi) in roots and shoots. The mRNA is cell-to-cell mobile.
AT4G03230	*1,059*		S-locus lectin protein kinase family protein; FUNCTIONS IN: sugar binding, protein serine/threonine kinase activity, protein kinase activity, kinase activity, ATP binding; INVOLVED IN: protein amino acid phosphorylation, recognition of pollen; LOCATED IN: endomembrane system; EXPRESSED IN: 9 plant structures; EXPRESSED DURING: 4 anthesis, F mature embryo stage, petal differentiation and expansion stage, E expanded cotyledon stage, D bilateral stage; CONTAINS InterPro DOMAIN/s: Curculin-like (mannose-binding) lectin (InterPro:IPR001480), PAN-2 domain (InterPro:IPR013227), Apple-like (InterPro:IPR003609), S-locus receptor kinase, C-terminal (InterPro:IPR021820), Serine/threonine-protein kinase domain (InterPro:IPR002290), EGF-like, type 3 (InterPro:IPR000742), Serine/threonine-protein kinase-like domain (InterPro:IPR017442), Serine/threonine-protein kinase, active site (InterPro:IPR008271), Protein kinase-like domain (InterPro:IPR011009), Protein kinase, catalytic domain (InterPro:IPR000719), S-locus glycoprotein (InterPro:IPR000858), Tyrosine-protein kinase, catalytic domain (InterPro:IPR020635); BEST Arabidopsis thaliana protein match is: S-locus lectin protein kinase family protein (TAIR:AT1G11340.1)
AT4G33260	*1,059*	CELL DIVISION CYCLE 20.2 (CDC20.2)	Encodes a CDC20 protein that interacts with APC subunits, components of the mitochondrial checkpoint complex and mitotic cyclin substrates and is indispensable for normal plant development and fertility.
AT2G42220	*1,056*		Rhodanese/Cell cycle control phosphatase superfamily protein; FUNCTIONS IN: molecular_function unknown; INVOLVED IN: biological_process unknown; LOCATED IN: chloroplast thylakoid membrane, chloroplast; EXPRESSED IN: 22 plant structures; EXPRESSED DURING: 13 growth stages; CONTAINS InterPro DOMAIN/s: Rhodanese-like (InterPro:IPR001763); BEST Arabidopsis thaliana protein match is: Rhodanese/Cell cycle control phosphatase superfamily protein (TAIR:AT3G08920.1)
AT4G28660	*1,056*	PHOTOSYSTEM II REACTION CENTER PSB28 PROTEIN (PSB28)	Similar to PsbW subunit of photosystem II.
AT2G37420	*1,055*		ATP binding microtubule motor family protein; FUNCTIONS IN: microtubule motor activity, ATP binding; INVOLVED IN: microtubule-based movement; LOCATED IN: chloroplast; EXPRESSED IN: 19 plant structures; EXPRESSED DURING: 9 growth stages; CONTAINS InterPro DOMAIN/s: Kinesin, motor region, conserved site (InterPro:IPR019821), Kinesin, motor domain (InterPro:IPR001752); BEST Arabidopsis thaliana protein match is: P-loop containing nucleoside triphosphate hydrolases superfamily protein (TAIR:AT3G45850.2)
AT1G68010	*1,054*	HYDROXYPYRUVATE REDUCTASE (HPR)	Encodes hydroxypyruvate reductase.

AT4G27480	*1,053*		Core-2/I-branching beta-1,6-N-acetylglucosaminyltransferase family protein; FUNCTIONS IN: transferase activity, transferring glycosyl groups, acetylglucosaminyltransferase activity; INVOLVED IN: carbohydrate biosynthetic process, N-terminal protein myristoylation; LOCATED IN: endomembrane system, membrane; EXPRESSED IN: 12 plant structures; EXPRESSED DURING: 8 growth stages; CONTAINS InterPro DOMAIN/s: Glycosyl transferase, family 14 (InterPro:IPR003406), Core-2/I-Branching enzyme (InterPro:IPR021141); BEST Arabidopsis thaliana protein match is: Core-2/I-branching beta-1,6-N-acetylglucosaminyltransferase family protein (TAIR:AT3G15350.2)
AT3G04040	*1,052*		unknown protein; BEST Arabidopsis thaliana protein match is: unknown protein (TAIR:AT5G18250.1)
AT4G11990	*1,051*		Cell cycle regulated microtubule associated protein; CONTAINS InterPro DOMAIN/s: Cell cycle regulated microtubule associated protein (InterPro:IPR022021); BEST Arabidopsis thaliana protein match is: Cell cycle regulated microtubule associated protein (TAIR:AT4G22860.1)
AT4G19120	*1,05*	EARLY-RESPONSIVE TO DEHYDRATION 3 (ERD3)	early-responsive to dehydration 3 (ERD3); CONTAINS InterPro DOMAIN/s: Protein of unknown function DUF248, methyltransferase putative (InterPro:IPR004159); BEST Arabidopsis thaliana protein match is: S-adenosyl-L-methionine-dependent methyltransferases superfamily protein (TAIR:AT1G31850.2)
AT1G52590	*1,049*		Putative thiol-disulphide oxidoreductase DCC; FUNCTIONS IN: molecular_function unknown; INVOLVED IN: biological_process unknown; LOCATED IN: plastoglobule; EXPRESSED IN: 22 plant structures; EXPRESSED DURING: 13 growth stages; CONTAINS InterPro DOMAIN/s: Putative thiol-disulphide oxidoreductase DCC (InterPro:IPR007263); BEST Arabidopsis thaliana protein match is: Putative thiol-disulphide oxidoreductase DCC (TAIR:AT1G24095.1)
AT4G33030	*1,049*	SULFOQUINOVOSYLDIACYLGLYCEROL 1 (SQD1)	involved in sulfolipid biosynthesis The mRNA is cell-to-cell mobile.
AT1G76100	*1,048*	PLASTOCYANIN 1 (PETE1)	One of two Arabidopsis plastocyanin genes. Expressed at 1/10th level of PETE2. Does not respond to increased copper levels and is thought to be the isoform that participates in electron transport under copper-limiting conditions. Mutation of this gene does not have obvious effect on photosynthesis.
AT3G14810	*1,046*	MECHANOSENSITIVE CHANNEL OF SMALL CONDUCTANCE-LIKE 5 (MSL5)	mechanosensitive channel of small conductance-like 5 (MSL5); INVOLVED IN: transmembrane transport; LOCATED IN: membrane; CONTAINS InterPro DOMAIN/s: Membrane protein, At2g17000, predicted (InterPro:IPR016688), Mechanosensitive ion channel MscS (InterPro:IPR006685), Like-Sm ribonucleoprotein (LSM)-related domain (InterPro:IPR010920); BEST Arabidopsis thaliana protein match is: mechanosensitive channel of small conductance-like 4 (TAIR:AT1G53470.1)
AT4G05430	*1,045*		Carbohydrate-binding X8 domain superfamily protein; CONTAINS InterPro DOMAIN/s: X8 (InterPro:IPR012946); BEST Arabidopsis thaliana protein match is: Carbohydrate-binding X8 domain superfamily protein (TAIR:AT1G09460.1)
AT1G64660	*1,042*	METHIONINE GAMMA-LYASE (MGL)	Encodes a functional methionine gamma-lyase, a cytosolic enzyme catalyzes the degradation of methionine into methanethiol, alpha-ketobutyrate and ammonia. The catabolism of excess methionine is important to methionine homeostasis. The mRNA is cell-to-cell mobile.
AT2G29630	*1,042*	THIAMINC (THIC)	Encodes a protein involved in thiamin biosynthesis. The protein is an iron-sulfur cluster protein predicted to catalyze the conversion of 5-aminoimidazole ribonucleotide (AIR) to hydroxymethylpyrimidine (HMP) or hydroxymethylpyrimidine phosphate (HMP-P). A severe reduction of THIC levels in plants decreases vitamin B1 (thiamin diphosphate (TPP)) levels and also leads to changes in the levels of numerous other metabolites since so many primary metabolic enzymes require a TPP co-factor. thiC mutants are chlorotic and arrest in their development at the cotyledon stage. A N-terminal targeting sequence directs the THIC protein to the chloroplast stroma. A conserved TPP-binding site is located in the 3' UTR of the At2g29630.2 gene model, and is predicted to function as a riboswitch. The riboswitch controls the formation of transcripts with alternative 3' UTR lengths, which affect mRNA accumulation and protein production. THIC transcripts are observed in seedlings 5 or more days after germination, and light promotes the expression of this gene. Recessive mutant isolated by Redei. Leaves but not cotyledons white, lethal; restored to normal by thiamine or 2,5-dimethyl-4-aminopyrimidine.
AT5G23800	*1,042*	DOMAIN OF UNKNOWN FUNCTION 724 10 (DUF 10)	Member of the plant-specific DUF724 protein family. Arabidopsis has 10 DUF724 proteins. Loss of function mutant has a WT phenotype
AT2G35290	*1,041*	SMALL AUXIN UPREGULATED RNA 79 (SAUR79)	unknown protein

AT5G06120	*1,041*		ARM repeat superfamily protein; FUNCTIONS IN: protein transporter activity, binding; INVOLVED IN: intracellular protein transport, protein import into nucleus, docking; LOCATED IN: nucleus, nuclear pore, cytoplasm; EXPRESSED IN: 22 plant structures; EXPRESSED DURING: 13 growth stages; CONTAINS InterPro DOMAIN/s: Importin-beta, N-terminal (InterPro:IPR001494), Armadillo-type fold (InterPro:IPR016024); BEST Arabidopsis thaliana protein match is: unknown protein (TAIR:AT3G04490.1)
AT5G19380	*1,041*	CRT (CHLOROQUINE-RESISTANCE TRANSPORTER)-LIKE TRANSPORTER 1 (CLT1)	Encodes one of the CRT-Like transporters (CLT1/AT5G19380, CLT2/AT4G24460, CLT3/AT5G12170). Required for glutathione homeostasis and stress responses. Mutants lacking these transporters are heavy metal-sensitive, glutathione(GSH)-deficient, and hypersensitive to Phytophthora infection.
AT5G20140	*1,041*	HAEM-BINDING PROTEIN 5 (HBP5)	Encodes a haem-binding protein, HBP5. HBP5 binds haem and interacts with the haem oxygenase, HY1. Disrupting the binding of HBP5 to HY1 leads to oxidative stress.
AT5G41790	*1,041*	COP1-INTERACTIVE PROTEIN 1 (CIP1)	encodes a protein that physically interacts specifically with the putative coiled-coil region of COP1 in vitro. In hypocotyl and cotyledon protoplasts, it is associated to the cytoskeleton, but not in the root. expression is not regulated by light. The mRNA is cell-to-cell mobile.
AT5G62380	*1,041*	NAC-DOMAIN PROTEIN 101 (NAC101)	Encodes a NAC-domain transcription factor involved in xylem formation. Induces transdifferentiation of various cells into metaxylem vessel elements. Located in the nucleus. Expression induced in the presence of auxin, cytokinin and brassinosteroids.
AT1G45191	*1,04*	BETA GLUCOSIDASE 1 (BGLU1)	beta-glucosidase related protein, similar to beta-glucosidase GI:3820531 from (Pinus contorta); contains Pfam profile: PF00232 Glycosyl hydrolase family 1
AT3G07390	*1,04*	AUXIN-INDUCED IN ROOT CULTURES 12 (AIR12)	isolated from differential screening of a cDNA library from auxin-treated root culture. sequence does not show homology to any known proteins and is predicted to be extracellular. The mRNA is cell-to-cell mobile.
AT4G27720	*1,04*		Major facilitator superfamily protein; LOCATED IN: plasma membrane; EXPRESSED IN: 21 plant structures; EXPRESSED DURING: 13 growth stages; CONTAINS InterPro DOMAIN/s: Protein of unknown function DUF791 (InterPro:IPR008509), Major facilitator superfamily, general substrate transporter (InterPro:IPR016196); BEST Arabidopsis thaliana protein match is: Major facilitator superfamily protein (TAIR:AT3G49310.1)
AT4G13950	*1,039*	RALF-LIKE 31 (RALFL31)	Member of a diversely expressed predicted peptide family showing sequence similarity to tobacco Rapid Alkalinization Factor (RALF), and is believed to play an essential role in the physiology of Arabidopsis. Consists of a single exon and is characterized by a conserved C-terminal motif and N-terminal signal peptide.
AT4G10340	*1,038*	LIGHT HARVESTING COMPLEX OF PHOTOSYSTEM II 5 (LHCB5)	photosystem II encoding the light-harvesting chlorophyll a/b binding protein CP26 of the antenna system of the photosynthetic apparatus The mRNA is cell-to-cell mobile.
AT5G03545	*1,037*	(AT4)	Expressed in response to phosphate starvation, this response is enhanced by the presence of IAA. Additionally, its expression is responsive to both phosphate (Pi) and phosphite (Phi) in shoots. The mRNA is cell-to-cell mobile.
AT5G05730	*1,037*	ANTHRANILATE SYNTHASE ALPHA SUBUNIT 1 (ASA1)	ASA1 encodes the alpha subunit of anthranilate synthase, which catalyzes the rate-limiting step of tryptophan synthesis. ASA1 is induced by ethylene, and forms a link between ethylene signalling and auxin synthesis in roots.
AT5G62960	*1,036*		unknown protein; BEST Arabidopsis thaliana protein match is: unknown protein (TAIR:AT1G10660.1)
AT1G01320	*1,035*	REDUCED CHLOROPLAST COVERAGE (REC1)	Encodes REDUCED CHLOROPLAST COVERAGE 1 (REC1). Located in the nucleus and cytosol. Contributes to establishing the size of the chloroplast compartment.
AT3G12750	*1,035*	ZINC TRANSPORTER 1 PRECURSOR (ZIP1)	A member of Zrt- and Irt-related protein (ZIP) family. transcript is induced in response to zinc deficiency in the root.
AT1G72500	*1,034*		LOCATED IN: plasma membrane; EXPRESSED IN: 23 plant structures; EXPRESSED DURING: 13 growth stages; CONTAINS InterPro DOMAIN/s: von Willebrand factor, type A (InterPro:IPR002035); BEST Arabidopsis thaliana protein match is: inter-alpha-trypsin inhibitor heavy chain-related (TAIR:AT1G19110.1)
AT4G05010	*1,034*	F-BOX STRESS INDUCED 3 (FBS3)	F-box family protein; CONTAINS InterPro DOMAIN/s: F-box domain, cyclin-like (InterPro:IPR001810); BEST Arabidopsis thaliana protein match is: F-box family protein (TAIR:AT4G21510.1)

AT5G59760	*1,034*		Protein of unknown function (DUF1635); CONTAINS InterPro DOMAIN/s: Protein of unknown function DUF1635 (InterPro:IPR012862); BEST Arabidopsis thaliana protein match is: Protein of unknown function (DUF1635) (TAIR:AT2G28690.1)
AT2G19400	*1,033*		AGC (cAMP-dependent, cGMP-dependent and protein kinase C) kinase family protein; FUNCTIONS IN: kinase activity; INVOLVED IN: protein amino acid phosphorylation; LOCATED IN: cellular_component unknown; EXPRESSED IN: 9 plant structures; EXPRESSED DURING: 6 growth stages; CONTAINS InterPro DOMAIN/s: Protein kinase, ATP binding site (InterPro:IPR017441), Serine/threonine-protein kinase domain (InterPro:IPR002290), Serine/threonine-protein kinase-like domain (InterPro:IPR017442), Protein kinase, C-terminal (InterPro:IPR017892), Protein kinase-like domain (InterPro:IPR011009), Serine/threonine-protein kinase, active site (InterPro:IPR008271), AGC-kinase, C-terminal (InterPro:IPR000961), Protein kinase, catalytic domain (InterPro:IPR000719); BEST Arabidopsis thaliana protein match is: AGC (cAMP-dependent, cGMP-dependent and protein kinase C) kinase family protein (TAIR:AT4G33080.1)
AT3G13080	*1,033*	ATP-BINDING CASSETTE C3 (ABCC3)	encodes an ATP-dependent MRP-like ABC transporter able to transport glutathione-conjugates as well as chlorophyll catabolites. The expression of this gene is upregulated by herbicide safeners such as benoxacor and fenclorim.
AT4G15540	*1,032*	USUALLY MULTIPLE ACIDS MOVE IN AND OUT TRANSPORTERS 38 (UMAMIT38)	nodulin MtN21-like transporter family protein The mRNA is cell-to-cell mobile.
AT4G02590	*1,031*	UNFERTILIZED EMBRYO SAC 12 (UNE12)	unfertilized embryo sac 12 (UNE12); FUNCTIONS IN: DNA binding, sequence-specific DNA binding transcription factor activity; INVOLVED IN: double fertilization forming a zygote and endosperm, regulation of transcription; LOCATED IN: nucleus; EXPRESSED IN: 22 plant structures; EXPRESSED DURING: 13 growth stages; CONTAINS InterPro DOMAIN/s: Helix-loop-helix DNA-binding domain (InterPro:IPR001092), Helix-loop-helix DNA-binding (InterPro:IPR011598); BEST Arabidopsis thaliana protein match is: basic helix-loop-helix (bHLH) DNA-binding superfamily protein (TAIR:AT1G03040.1)
AT1G66230	*1,027*	MYB DOMAIN PROTEIN 20 (MYB20)	Encodes a putative transcription factor (MYB20).
AT5G03270	*1,027*	LONELY GUY 6 (LOG6)	lysine decarboxylase family protein; FUNCTIONS IN: molecular_function unknown; INVOLVED IN: lysine biosynthetic process via diaminopimelate; EXPRESSED IN: 13 plant structures; EXPRESSED DURING: 8 growth stages; CONTAINS InterPro DOMAIN/s: Conserved hypothetical protein CHP00730 (InterPro:IPR005269); BEST Arabidopsis thaliana protein match is: lysine decarboxylase family protein (TAIR:AT2G37210.1)
AT5G12890	*1,026*		UDP-Glycosyltransferase superfamily protein; FUNCTIONS IN: UDP-glycosyltransferase activity, transferase activity, transferring glycosyl groups; INVOLVED IN: metabolic process; EXPRESSED IN: 22 plant structures; EXPRESSED DURING: 12 growth stages; CONTAINS InterPro DOMAIN/s: UDP-glucuronosyl/UDP-glucosyltransferase (InterPro:IPR002213); BEST Arabidopsis thaliana protein match is: UDP-Glycosyltransferase superfamily protein (TAIR:AT2G36780.1)
AT2G44690	*1,024*	ARABIDOPSIS RAC-LIKE 9 (ARAC9)	A member of ROP GTPase gene family.
AT5G20040	*1,024*	ISOPENTENYLTRANSFERASE 9 (IPT9)	Encodes tRNA isopentenyltransferase AtIPT9.
AT1G74730	*1,023*	(RIQ2)	Encodes a grana core localized protein, is homologous to RIG1. Mutant plants have reduced NPQ, affected organization of light-havesting complex II and an enhanced grana stacking.
AT5G64980	*1,02*		unknown protein; BEST Arabidopsis thaliana protein match is: unknown protein (TAIR:AT4G02880.1)
AT1G47960	*1,019*	CELL WALL / VACUOLAR INHIBITOR OF FRUCTOSIDASE 1 (C/VIF1)	Plant cell wall (CWI) and vacuolar invertases (VI) play important roles in carbohydrate metabolism, stress responses and sugar signaling. This protein may inhibit their activity.

AT1G76810	*1,019*		eukaryotic translation initiation factor 2 (eIF-2) family protein; FUNCTIONS IN: translation factor activity, nucleic acid binding, GTP binding, GTPase activity; LOCATED IN: plasma membrane; EXPRESSED IN: 25 plant structures; EXPRESSED DURING: 15 growth stages; CONTAINS InterPro DOMAIN/s: Small GTP-binding protein (InterPro:IPR005225), Translation elongation factor EFTu/EF1A, domain 2 (InterPro:IPR004161), Translation initiation factor 2 related (InterPro:IPR015760), Protein synthesis factor, GTP-binding (InterPro:IPR000795), Translation elongation/initiation factor/Ribosomal, beta-barrel (InterPro:IPR009000); BEST Arabidopsis thaliana protein match is: eukaryotic translation initiation factor 2 (eIF-2) family protein (TAIR:AT1G76720.1)
AT1G56690	*1,018*		Pentatricopeptide repeat (PPR) superfamily protein; CONTAINS InterPro DOMAIN/s: Pentatricopeptide repeat (InterPro:IPR002885); BEST Arabidopsis thaliana protein match is: pentatricopeptide (PPR) repeat-containing protein (TAIR:AT1G09410.1)
AT1G03620	*1,017*		ELMO/CED-12 family protein; FUNCTIONS IN: molecular_function unknown; INVOLVED IN: phagocytosis; LOCATED IN: cytoskeleton; EXPRESSED IN: 16 plant structures; EXPRESSED DURING: 6 growth stages; CONTAINS InterPro DOMAIN/s: Engulfment/cell motility, ELMO (InterPro:IPR006816); BEST Arabidopsis thaliana protein match is: ELMO/CED-12 family protein (TAIR:AT3G03610.1)
AT1G55610	*1,017*	BRI1 LIKE (BRL1)	mutant has Altered vascular cell differentiation; LRR Receptor Kinase
AT5G43380	*1,017*	TYPE ONE SERINE/THREONINE PROTEIN PHOSPHATASE 6 (TOPP6)	encodes a type I serine/threonine protein phosphatase expressed in expressed in roots, rosettes and flowers.
AT5G54390	*1,016*	HAL2-LIKE (HL)	Encodes a 3'-phosphoadenosine-5'-phosphate (PAP) phosphatase that is sensitive to physiological concentrations of Na+. It does not also act as inositol polyphosphate 1-phosphatases, which other members of the HAL2-like family do. It is proposed that AHL acts in concert with sulphotransferases to prevent both the toxicity of PAP on RNA processing enzymes as well as the product inhibition of PAP on sulphate conjugation. The mRNA is cell-to-cell mobile.
AT4G36010	*1,013*		Pathogenesis-related thaumatin superfamily protein; CONTAINS InterPro DOMAIN/s: Thaumatin, pathogenesis-related (InterPro:IPR001938); BEST Arabidopsis thaliana protein match is: Pathogenesis-related thaumatin superfamily protein (TAIR:AT2G17860.1)
AT5G57790	*1,012*	ABORTED GAMETOPHYTE 1 (AOG1)	Encodes a nuclear localized protein of unknown function that is involved in pollen and embryo sac development.
AT1G05710	*1,011*		basic helix-loop-helix (bHLH) DNA-binding superfamily protein; FUNCTIONS IN: sequence-specific DNA binding transcription factor activity; INVOLVED IN: response to ethylene stimulus, regulation of transcription; LOCATED IN: nucleus; EXPRESSED IN: 22 plant structures; EXPRESSED DURING: 13 growth stages; CONTAINS InterPro DOMAIN/s: Helix-loop-helix DNA-binding domain (InterPro:IPR001092), Helix-loop-helix DNA-binding (InterPro:IPR011598); BEST Arabidopsis thaliana protein match is: basic helix-loop-helix (bHLH) DNA-binding superfamily protein (TAIR:AT2G31730.1)
AT1G10750	*1,011*		Protein of Unknown Function (DUF239); INVOLVED IN: N-terminal protein myristoylation; EXPRESSED IN: 13 plant structures; EXPRESSED DURING: 6 growth stages; CONTAINS InterPro DOMAIN/s: Protein of unknown function DUF239, plant (InterPro:IPR004314); BEST Arabidopsis thaliana protein match is: Protein of Unknown Function (DUF239) (TAIR:AT1G23340.2)
AT5G14780	*1,011*	FORMATE DEHYDROGENASE (FDH)	Encodes a NAD-dependent formate dehydrogenase.
AT1G50460	*1,009*	HEXOKINASE-LIKE 1 (HKL1)	Involved in glucose-ethylene crosstalk.
AT2G18620	*1,009*	GERANYLGERANYL PYROPHOSPHATE SYNTHASE 2 (GGPPS2)	Terpenoid synthases superfamily protein; INVOLVED IN: isoprenoid biosynthetic process; EXPRESSED IN: root; CONTAINS InterPro DOMAIN/s: Polyprenyl synthetase-related (InterPro:IPR017446), Terpenoid synthase (InterPro:IPR008949), Polyprenyl synthetase (InterPro:IPR000092); BEST Arabidopsis thaliana protein match is: geranylgeranyl pyrophosphate synthase 1 (TAIR:AT4G36810.1)
AT2G36650	*1,009*		unknown protein

AT1G74010	*1,008*		Calcium-dependent phosphotriesterase superfamily protein; FUNCTIONS IN: strictosidine synthase activity; INVOLVED IN: biosynthetic process; LOCATED IN: plant-type cell wall; EXPRESSED IN: 11 plant structures; EXPRESSED DURING: L mature pollen stage, M germinated pollen stage, 4 anthesis, C globular stage, petal differentiation and expansion stage; CONTAINS InterPro DOMAIN/s: Strictosidine synthase, conserved region (InterPro:IPR018119), Strictosidine synthase (InterPro:IPR004141), Six-bladed beta-propeller, TolB-like (InterPro:IPR011042); BEST Arabidopsis thaliana protein match is: strictosidine synthase 2 (TAIR:AT1G74020.1)
AT5G13630	*1,008*	GENOMES UNCOUPLED 5 (GUN5)	Encodes magnesium chelatase involved in plastid-to-nucleus signal transduction.
AT1G06420	*1,007*		unknown protein
AT1G75090	*1,007*		DNA glycosylase superfamily protein; FUNCTIONS IN: DNA-3-methyladenine glycosylase I activity, catalytic activity; INVOLVED IN: DNA repair, base-excision repair; EXPRESSED IN: 22 plant structures; EXPRESSED DURING: 9 growth stages; CONTAINS InterPro DOMAIN/s: DNA glycosylase (InterPro:IPR011257), Methyladenine glycosylase (InterPro:IPR005019); BEST Arabidopsis thaliana protein match is: DNA glycosylase superfamily protein (TAIR:AT5G57970.2)
AT5G62070	*1,007*	IQ-DOMAIN 23 (IQD23)	IQ-domain 23 (IQD23); CONTAINS InterPro DOMAIN/s: IQ calmodulin-binding region (InterPro:IPR000048); BEST Arabidopsis thaliana protein match is: IQ-domain 24 (TAIR:AT5G07240.1)
AT5G64620	*1,006*	CELL WALL / VACUOLAR INHIBITOR OF FRUCTOSIDASE 2 (C/VIF2)	Plant cell wall (CWI) and vacuolar invertases (VI) play important roles in carbohydrate metabolism, stress responses and sugar signaling.
AT3G52340	*1,005*	SUCROSE-6F-PHOSPHATE PHOSPHOHYDROLASE 2 (SPP2)	sucrose-phosphatase (SPP2)
AT4G00950	*1,005*	MATERNAL EFFECT EMBRYO ARREST 47 (MEE47)	maternal effect embryo arrest 47 (MEE47); CONTAINS InterPro DOMAIN/s: Protein of unknown function DUF688 (InterPro:IPR007789)
AT1G80560	*1,004*	ISOPROPYLMALATE DEHYDROGENASE 2 (IMD2)	The AtIMD2 is one out of 3 genes encoding the enzyme 3-isopropylmalate dehydrogenase involved in leucine biosynthesis in Arabidopsis. Its subcellular location has been targeted to plastids. The mRNA is cell-to-cell mobile.
AT4G10500	*1,003*	DMR6-LIKE OXYGENASE 1 (DLO1)	2-oxoglutarate (2OG) and Fe(II)-dependent oxygenase superfamily protein; FUNCTIONS IN: oxidoreductase activity, acting on paired donors, with incorporation or reduction of molecular oxygen, 2-oxoglutarate as one donor, and incorporation of one atom each of oxygen into both donors, oxidoreductase activity; INVOLVED IN: secondary metabolic process; EXPRESSED IN: 12 plant structures; EXPRESSED DURING: 9 growth stages; CONTAINS InterPro DOMAIN/s: Oxoglutarate/iron-dependent oxygenase (InterPro:IPR005123); BEST Arabidopsis thaliana protein match is: 2-oxoglutarate (2OG) and Fe(II)-dependent oxygenase superfamily protein (TAIR:AT4G10490.1)
AT2G34510	*1,002*		FUNCTIONS IN: molecular_function unknown; INVOLVED IN: biological_process unknown; LOCATED IN: anchored to membrane; EXPRESSED IN: 20 plant structures; EXPRESSED DURING: 14 growth stages; CONTAINS InterPro DOMAIN/s: Protein of unknown function DUF642 (InterPro:IPR006946), Galactose-binding domain-like (InterPro:IPR008979); BEST Arabidopsis thaliana protein match is: Protein of unknown function, DUF642 (TAIR:AT1G29980.1)
AT4G15450	*1,002*		Senescence/dehydration-associated protein-related; CONTAINS InterPro DOMAIN/s: Senescence/spartin-associated (InterPro:IPR009686); BEST Arabidopsis thaliana protein match is: Senescence/dehydration-associated protein-related (TAIR:AT3G21600.1)
AT5G54180	*1,001*	PLASTID TRANSCRIPTIONALLY ACTIVE 15 (PTAC15)	plastid transcriptionally active 15 (PTAC15); FUNCTIONS IN: molecular_function unknown; INVOLVED IN: biological_process unknown; LOCATED IN: plastid chromosome, chloroplast, nucleoid; EXPRESSED IN: 21 plant structures; EXPRESSED DURING: 13 growth stages; CONTAINS InterPro DOMAIN/s: Mitochodrial transcription termination factor-related (InterPro:IPR003690); BEST Arabidopsis thaliana protein match is: Mitochondrial transcription termination factor family protein (TAIR:AT4G14605.1)
AT2G30790	*1*	PHOTOSYSTEM II SUBUNIT P-2 (PSBP-2)	Encodes a 23 kD extrinsic protein that is part of photosystem II and participates in the regulation of oxygen evolution.

AT2G41050	*1*		PQ-loop repeat family protein / transmembrane family protein; FUNCTIONS IN: molecular_function unknown; INVOLVED IN: biological_process unknown; LOCATED IN: endomembrane system, membrane; EXPRESSED IN: 8 plant structures; EXPRESSED DURING: 4 anthesis, F mature embryo stage, petal differentiation and expansion stage, E expanded cotyledon stage, D bilateral stage; CONTAINS InterPro DOMAIN/s: Cystinosin/ERS1p repeat (InterPro:IPR006603); BEST Arabidopsis thaliana protein match is: PQ-loop repeat family protein / transmembrane family protein (TAIR:AT4G36850.1)
AT3G03780	*1*	METHIONINE SYNTHASE 2	Encodes a cytosolic methionine synthase, involved in methionine regeneration via the activated methyl cycle (or SAM cycle)
AT2G43010	*0,999*	PHYTOCHROME INTERACTING FACTOR 4 (PIF4)	Isolated as a semidominant mutation defective in red -light responses. Encodes a nuclear localized bHLH protein that interacts with active PhyB protein. Negatively regulates phyB mediated red light responses. Involved in shade avoidance response. Protein abundance is negatively regulated by PhyB.
AT4G17695	*0,999*	KANADI 3 (KAN3)	KANADI 3 (KAN3); CONTAINS InterPro DOMAIN/s: Homeodomain-like (InterPro:IPR009057), Myb, DNA-binding (InterPro:IPR014778), Myb-like DNA-binding domain, SHAQKYF class (InterPro:IPR006447), Homeodomain-related (InterPro:IPR012287); BEST Arabidopsis thaliana protein match is: Homeodomain-like superfamily protein (TAIR:AT1G32240.1)
AT5G02460	*0,999*		Dof-type zinc finger DNA-binding family protein; CONTAINS InterPro DOMAIN/s: Zinc finger, Dof-type (InterPro:IPR003851); BEST Arabidopsis thaliana protein match is: DNA binding with one finger 2.4 (TAIR:AT2G37590.1)
AT5G65690	*0,999*	PHOSPHOENOLPYRUVATE CARBOXYKINASE 2 (PCK2)	Encodes a putative phosphoenolpyruvate carboxykinase (ATP-dependent). The mRNA is cell-to-cell mobile.
AT4G19050	*0,998*		NB-ARC domain-containing disease resistance protein; FUNCTIONS IN: ATP binding; INVOLVED IN: defense response, apoptosis; LOCATED IN: cellular_component unknown; EXPRESSED IN: 20 plant structures; EXPRESSED DURING: 9 growth stages; CONTAINS InterPro DOMAIN/s: NB-ARC (InterPro:IPR002182), Disease resistance protein (InterPro:IPR000767); BEST Arabidopsis thaliana protein match is: Leucine-rich repeat (LRR) family protein (TAIR:AT5G45510.2)
AT2G26500	*0,997*		cytochrome b6f complex subunit (petM), putative; FUNCTIONS IN: plastoquinol-plastocyanin reductase activity; LOCATED IN: chloroplast thylakoid membrane; EXPRESSED IN: 22 plant structures; EXPRESSED DURING: 13 growth stages; CONTAINS InterPro DOMAIN/s: PetM of cytochrome b6/f complex subunit 7 (InterPro:IPR012595)
AT4G02510	*0,997*	TRANSLOCON AT THE OUTER ENVELOPE MEMBRANE OF CHLOROPLASTS 159 (TOC159)	An integral membrane GTPase that functions as a transit-sequence receptor required for the import of proteins necessary for chloroplast biogenesis. Located in the outer chloroplast membrane. Phosphorylation of the G-domains regulate translocon assembly. The mRNA is cell-to-cell mobile.
AT4G10390	*0,992*		Protein kinase superfamily protein; FUNCTIONS IN: protein serine/threonine kinase activity, protein kinase activity, kinase activity, ATP binding; INVOLVED IN: response to wounding; LOCATED IN: cellular_component unknown; EXPRESSED IN: 18 plant structures; EXPRESSED DURING: 11 growth stages; CONTAINS InterPro DOMAIN/s: Protein kinase, ATP binding site (InterPro:IPR017441), Protein kinase, catalytic domain (InterPro:IPR000719), Serine/threonine-protein kinase-like domain (InterPro:IPR017442), Protein kinase-like domain (InterPro:IPR011009), Serine/threonine-protein kinase, active site (InterPro:IPR008271); BEST Arabidopsis thaliana protein match is: Protein kinase superfamily protein (TAIR:AT1G33260.1)
AT1G51760	*0,991*	IAA-ALANINE RESISTANT 3 (IAR3)	encodes a member of the six Arabidopsis IAA-amino acid conjugate hydrolase subfamily and conjugates and conjugates IAA-Ala in vitro. Gene is expressed most strongly in roots, stems, and flowers. The mRNA is cell-to-cell mobile.
AT5G56850	*0,991*		unknown protein; EXPRESSED IN: 21 plant structures; EXPRESSED DURING: 13 growth stages
AT2G28660	*0,989*		Chloroplast-targeted copper chaperone protein; FUNCTIONS IN: copper ion binding, metal ion binding; INVOLVED IN: copper ion transport, metal ion transport; LOCATED IN: chloroplast; EXPRESSED IN: 21 plant structures; EXPRESSED DURING: 13 growth stages; CONTAINS InterPro DOMAIN/s: Heavy metal transport/detoxification protein (InterPro:IPR006121), Copper chaperone, chloroplast-targeted, predicted (InterPro:IPR016578); BEST Arabidopsis thaliana protein match is: Chloroplast-targeted copper chaperone protein (TAIR:AT3G53530.1)
AT1G74390	*0,988*	NAC45/86-DEPENDENT EXONUCLEASE-DOMAIN PROTEIN 3 (NEN3)	Polynucleotidyl transferase, ribonuclease H-like superfamily protein; FUNCTIONS IN: exonuclease activity, nucleic acid binding; LOCATED IN: intracellular; EXPRESSED IN: 12 plant structures; EXPRESSED DURING: 6 growth stages; CONTAINS InterPro DOMAIN/s: Exonuclease (InterPro:IPR006055), Polynucleotidyl transferase, ribonuclease H fold (InterPro:IPR012337), Exonuclease, RNase T/DNA polymerase III (InterPro:IPR013520); BEST Arabidopsis thaliana protein match is: Polynucleotidyl transferase, ribonuclease H-like superfamily protein (TAIR:AT5G61390.1)

AT2G28510	*0,988*		Dof-type zinc finger DNA-binding family protein; FUNCTIONS IN: DNA binding, sequence-specific DNA binding transcription factor activity; INVOLVED IN: regulation of transcription; LOCATED IN: nucleolus; EXPRESSED IN: 22 plant structures; EXPRESSED DURING: 13 growth stages; CONTAINS InterPro DOMAIN/s: Zinc finger, Dof-type (InterPro:IPR003851); BEST Arabidopsis thaliana protein match is: TARGET OF MONOPTEROS 6 (TAIR:AT5G60200.1)
AT3G12780	*0,988*	PHOSPHOGLYCERATE KINASE 1 (PGK1)	nuclear phosphoglycerate kinase (PGK1)
AT4G22590	*0,988*	TREHALOSE-6-PHOSPHATE PHOSPHATASE G (TPPG)	Haloacid dehalogenase-like hydrolase (HAD) superfamily protein; FUNCTIONS IN: catalytic activity, trehalose-phosphatase activity; INVOLVED IN: trehalose biosynthetic process, metabolic process; LOCATED IN: chloroplast; EXPRESSED IN: sperm cell, male gametophyte, pollen tube; EXPRESSED DURING: L mature pollen stage, M germinated pollen stage; CONTAINS InterPro DOMAIN/s: HAD-superfamily hydrolase, subfamily IIB (InterPro:IPR006379), Trehalose-phosphatase (InterPro:IPR003337); BEST Arabidopsis thaliana protein match is: Haloacid dehalogenase-like hydrolase (HAD) superfamily protein (TAIR:AT4G12430.1)
AT4G24380	*0,988*		INVOLVED IN: 10-formyltetrahydrofolate biosynthetic process, folic acid and derivative biosynthetic process; EXPRESSED IN: 24 plant structures; EXPRESSED DURING: 15 growth stages; CONTAINS InterPro DOMAIN/s: Serine hydrolase (InterPro:IPR005645); BEST Arabidopsis thaliana protein match is: alpha/beta-Hydrolases superfamily protein (TAIR:AT5G65400.1)
AT5G65400	*0,988*		alpha/beta-Hydrolases superfamily protein; CONTAINS InterPro DOMAIN/s: Serine hydrolase (InterPro:IPR005645); BEST Arabidopsis thaliana protein match is: unknown protein (TAIR:AT4G24380.1)
AT2G27040	*0,987*	ARGONAUTE 4 (AGO4)	AGO4 is a member of a class of PAZ/PIWI domain containing proteins involved in siRNA mediated gene silencing.Loss of function mutations have reduced site specific CpNpG and CpHpH methylation and increased susceptibility to bacterial pathogens.
AT2G18780	*0,985*		F-box and associated interaction domains-containing protein; CONTAINS InterPro DOMAIN/s: F-box domain, cyclin-like (InterPro:IPR001810), F-box domain, Skp2-like (InterPro:IPR022364), F-box associated domain, type 1 (InterPro:IPR006527), F-box associated interaction domain (InterPro:IPR017451); BEST Arabidopsis thaliana protein match is: F-box and associated interaction domains-containing protein (TAIR:AT3G16740.1)
AT2G23530	*0,985*		Zinc-finger domain of monoamine-oxidase A repressor R1; CONTAINS InterPro DOMAIN/s: Cell division cycle-associated protein (InterPro:IPR018866); BEST Arabidopsis thaliana protein match is: Zinc-finger domain of monoamine-oxidase A repressor R1 (TAIR:AT4G37110.1)
AT2G42760	*0,984*		unknown protein; CONTAINS InterPro DOMAIN/s: Protein of unknown function DUF1685 (InterPro:IPR012881)
AT4G19460	*0,984*		UDP-Glycosyltransferase superfamily protein; FUNCTIONS IN: transferase activity, transferring glycosyl groups; INVOLVED IN: biosynthetic process; LOCATED IN: cellular_component unknown; EXPRESSED IN: 10 plant structures; EXPRESSED DURING: 4 anthesis, C globular stage, petal differentiation and expansion stage; CONTAINS InterPro DOMAIN/s: Glycosyl transferase, group 1 (InterPro:IPR001296); BEST Arabidopsis thaliana protein match is: UDP-Glycosyltransferase superfamily protein (TAIR:AT1G73160.1)
AT4G37870	*0,984*	PHOSPHOENOLPYRUVATE CARBOXYKINASE 1 (PCK1)	Encodes a phosphoenolpyruvate carboxykinase that localizes to the cytosol.
AT5G46110	*0,984*	ACCLIMATION OF PHOTOSYNTHESIS TO ENVIRONMENT 2 (APE2)	Encodes a chloroplast triose phosphate / 3-phosphoglycerate translocator that transports triose phosphates derived from the Calvin cycle in the stroma to the cytosol for use in sucrose synthesis and other biosynthetic processes. A tpt mutant has altered acclimation responses. The mRNA is cell-to-cell mobile.
AT1G72250	*0,983*		Di-glucose binding protein with Kinesin motor domain; FUNCTIONS IN: microtubule motor activity, ATP binding; INVOLVED IN: microtubule-based movement; EXPRESSED IN: 18 plant structures; EXPRESSED DURING: 7 growth stages; CONTAINS InterPro DOMAIN/s: Kinesin, motor region, conserved site (InterPro:IPR019821), Malectin/receptor-like protein kinase (InterPro:IPR021720), Kinesin, motor domain (InterPro:IPR001752); BEST Arabidopsis thaliana protein match is: Di-glucose binding protein with Kinesin motor domain (TAIR:AT2G22610.2).

AT1G27720	*0,982*	TBP-ASSOCIATED FACTOR 4B (TAF4b)	TBP-associated factor 4B (TAF4B); FUNCTIONS IN: transcription initiation factor activity; INVOLVED IN: transcription initiation; LOCATED IN: transcription factor TFIID complex; EXPRESSED IN: 6 plant structures; EXPRESSED DURING: L mature pollen stage, M germinated pollen stage; CONTAINS InterPro DOMAIN/s: Transcription initiation factor TFIID component TAF4 (InterPro:IPR007900), RST domain of plant C-terminal (InterPro:IPR022003); BEST Arabidopsis thaliana protein match is: TBP-associated factor 4 (TAIR:AT5G43130.1)
AT4G28330	*0,982*		unknown protein; BEST Arabidopsis thaliana protein match is: unknown protein (TAIR:AT4G28340.1)
AT5G66610	*0,981*	DA1-RELATED PROTEIN 7 (DAR7)	DA1-related protein 7 (DAR7); FUNCTIONS IN: zinc ion binding; LOCATED IN: cellular_component unknown; EXPRESSED IN: 16 plant structures; EXPRESSED DURING: 7 growth stages; CONTAINS InterPro DOMAIN/s: Zinc finger, LIM-type (InterPro:IPR001781), Protein of unknown function DUF3633 (InterPro:IPR022087); BEST Arabidopsis thaliana protein match is: DA1-related protein 6 (TAIR:AT5G66620.1).
AT1G05370	*0,98*		Sec14p-like phosphatidylinositol transfer family protein; CONTAINS InterPro DOMAIN/s: Cellular retinaldehyde-binding/triple function, C-terminal (InterPro:IPR001251), Phosphatidylinositol transfer protein-like, N-terminal (InterPro:IPR011074); BEST Arabidopsis thaliana protein match is: Sec14p-like phosphatidylinositol transfer family protein (TAIR:AT3G22410.1)
AT1G68360	*0,98*	GLABROUS INFLORESCENCE STEMS 3 (GIS3)	Encodes a nuclear localized member of the C2H2 family of TFIIIA transcription factors.GIS3 is involved in trichome initiation and development downstream of GA and cytokinin signaling. GIS regulates the expression GIS and GIS2.
AT5G20220	*0,979*		zinc knuckle (CCHC-type) family protein; FUNCTIONS IN: unfolded protein binding, heat shock protein binding, zinc ion binding, nucleic acid binding; INVOLVED IN: protein folding; EXPRESSED IN: 23 plant structures; EXPRESSED DURING: 13 growth stages; CONTAINS InterPro DOMAIN/s: Zinc finger, CCHC-type (InterPro:IPR001878), Heat shock protein DnaJ, cysteine-rich domain (InterPro:IPR001305)
AT4G12740	*0,977*		HhH-GPD base excision DNA repair family protein; FUNCTIONS IN: hydrolase activity, catalytic activity; INVOLVED IN: DNA repair, base-excision repair; EXPRESSED IN: 10 plant structures; EXPRESSED DURING: 4 anthesis, F mature embryo stage, petal differentiation and expansion stage, E expanded cotyledon stage, D bilateral stage; CONTAINS InterPro DOMAIN/s: NUDIX hydrolase domain-like (InterPro:IPR015797), DNA glycosylase (InterPro:IPR011257), NUDIX hydrolase domain (InterPro:IPR000086), HhH-GPD domain (InterPro:IPR003265)
AT4G31000	*0,977*		Calmodulin-binding protein; CONTAINS InterPro DOMAIN/s: Calmodulin binding protein-like (InterPro:IPR012416); BEST Arabidopsis thaliana protein match is: Calmodulin-binding protein (TAIR:AT2G24300.2)
AT2G22890	*0,976*		Kua-ubiquitin conjugating enzyme hybrid localisation domain; LOCATED IN: chloroplast; EXPRESSED IN: 21 plant structures; EXPRESSED DURING: 13 growth stages; CONTAINS InterPro DOMAIN/s: Kua-ubiquitin conjugating enzyme hybrid, localisation (InterPro:IPR019547); BEST Arabidopsis thaliana protein match is: fatty acid desaturase A (TAIR:AT4G27030.1)
AT3G26220	*0,976*	CYTOCHROME P450, FAMILY 71, SUBFAMILY B, POLYPEPTIDE 3 (CYP71B3)	cytochrome P450 monooxygenase
AT3G50980	*0,976*	DEHYDRIN XERO 1 (XERO1)	dehydrin xero 1 (XERO1); FUNCTIONS IN: molecular_function unknown; INVOLVED IN: response to water, response to stress; LOCATED IN: cellular_component unknown; EXPRESSED IN: shoot apex, inflorescence meristem, petal, leaf whorl, seed; EXPRESSED DURING: F mature embryo stage, petal differentiation and expansion stage, E expanded cotyledon stage; CONTAINS InterPro DOMAIN/s: Dehydrin (InterPro:IPR000167); BEST Arabidopsis thaliana protein match is: Dehydrin family protein (TAIR:AT5G66400.1)
AT2G20850	*0,975*	STRUBBELIG-RECEPTOR FAMILY 1 (SRF1)	STRUBBELIG-receptor family 1 (SRF1); FUNCTIONS IN: kinase activity; INVOLVED IN: protein amino acid phosphorylation; LOCATED IN: plasma membrane; EXPRESSED IN: 22 plant structures; EXPRESSED DURING: 13 growth stages; CONTAINS InterPro DOMAIN/s: Serine/threonine-protein kinase domain (InterPro:IPR002290), Leucine-rich repeat-containing N-terminal domain, type 2 (InterPro:IPR013210), Leucine-rich repeat (InterPro:IPR001611), Serine-threonine/tyrosine-protein kinase (InterPro:IPR001245), Protein kinase-like domain (InterPro:IPR011009), Protein kinase, catalytic domain (InterPro:IPR000719), Tyrosine-protein kinase, catalytic domain (InterPro:IPR020635); BEST Arabidopsis thaliana protein match is: STRUBBELIG-receptor family 3 (TAIR:AT4G03390.1)

AT3G61880	*0,975*	CYTOCHROME P450 78A9 (CYP78A9)	Encodes a cytochrome p450 monooxygenase. Overexpression of this gene allows fruit growth independently of fertilization. The gene is normally expressed only in floral organs(during the Arabidopsis stage 14 flower) and in the funiculus at anthesis.
AT2G01320	*0,973*	ATP-BINDING CASSETTE G7 (ABCG7)	ABC-2 type transporter family protein; FUNCTIONS IN: ATPase activity, coupled to transmembrane movement of substances; LOCATED IN: chloroplast; EXPRESSED IN: 22 plant structures; EXPRESSED DURING: 14 growth stages; CONTAINS InterPro DOMAIN/s: ATPase, AAA+ type, core (InterPro:IPR003593), ABC transporter-like (InterPro:IPR003439), ABC-2 type transporter (InterPro:IPR013525), ABC transporter, conserved site (InterPro:IPR017871); BEST Arabidopsis thaliana protein match is: ABC-2 type transporter family protein (TAIR:AT3G25620.2)
AT3G01970	*0,973*	WRKY DNA-BINDING PROTEIN 45 (WRKY45)	member of WRKY Transcription Factor; Group I
AT5G18570	*0,973*	EMBRYO DEFECTIVE 269 (EMB269)	Encodes AtObgC, a plant ortholog of bacterial Obg. AtObgC is a chloroplast-targeting GTPase essential for early embryogenesis. Mutations in this locus result in embryo lethality. The protein is dually localized in the stroma and the inner envelope membrane and is involved in thylakoid membrane biogenesis and functions primarily in plastid ribosome biogenesis during chloroplast development.
AT5G08380	*0,972*	ALPHA-GALACTOSIDASE 1 (AGAL1)	alpha-galactosidase 1 (AGAL1); FUNCTIONS IN: alpha-galactosidase activity, hydrolase activity, hydrolyzing O-glycosyl compounds, catalytic activity; INVOLVED IN: carbohydrate metabolic process, metabolic process, lactose catabolic process; LOCATED IN: apoplast, cell wall, plant-type cell wall; EXPRESSED IN: 21 plant structures; EXPRESSED DURING: 13 growth stages; CONTAINS InterPro DOMAIN/s: Glycoside hydrolase, family 27 (InterPro:IPR002241), Aldolase-type TIM barrel (InterPro:IPR013785), Glycoside hydrolase, clan GH-D (InterPro:IPR000111), Glycoside hydrolase, catalytic core (InterPro:IPR017853); BEST Arabidopsis thaliana protein match is: alpha-galactosidase 2 (TAIR:AT5G08370.1)
AT3G04760	*0,97*		Pentatricopeptide repeat (PPR-like) superfamily protein; INVOLVED IN: biological_process unknown; LOCATED IN: chloroplast; EXPRESSED IN: 21 plant structures; EXPRESSED DURING: 13 growth stages; CONTAINS InterPro DOMAIN/s: Pentatricopeptide repeat (InterPro:IPR002885); BEST Arabidopsis thaliana protein match is: Pentatricopeptide repeat (PPR-like) superfamily protein (TAIR:AT1G09900.1)
AT3G06360	*0,97*	ARABINOGALACTAN PROTEIN 27 (AGP27)	Encodes an arabinogalactan-protein (AGP27).
AT1G69910	*0,969*		Protein kinase superfamily protein; FUNCTIONS IN: kinase activity; INVOLVED IN: protein amino acid phosphorylation; EXPRESSED IN: 20 plant structures; EXPRESSED DURING: 13 growth stages; CONTAINS InterPro DOMAIN/s: Protein kinase, ATP binding site (InterPro:IPR017441), Protein kinase, catalytic domain (InterPro:IPR000719), Serine/threonine-protein kinase domain (InterPro:IPR002290), Tyrosine-protein kinase, active site (InterPro:IPR008266), Tyrosine-protein kinase, catalytic domain (InterPro:IPR020635), Serine/threonine-protein kinase-like domain (InterPro:IPR017442), Protein kinase-like domain (InterPro:IPR011009); BEST Arabidopsis thaliana protein match is: Protein kinase superfamily protein (TAIR:AT1G18390.2)
AT4G12310	*0,969*	CYTOCHROME P450, FAMILY 706, SUBFAMILY A, POLYPEPTIDE 5 (CYP706A5)	member of CYP706A
AT5G13360	*0,968*		Auxin-responsive GH3 family protein; INVOLVED IN: response to auxin stimulus; LOCATED IN: cellular_component unknown; CONTAINS InterPro DOMAIN/s: GH3 auxin-responsive promoter (InterPro:IPR004993); BEST Arabidopsis thaliana protein match is: Auxin-responsive GH3 family protein (TAIR:AT5G13370.1).
AT5G52750	*0,968*		Heavy metal transport/detoxification superfamily protein ; FUNCTIONS IN: metal ion binding; INVOLVED IN: metal ion transport; LOCATED IN: cellular_component unknown; EXPRESSED IN: 20 plant structures; EXPRESSED DURING: 12 growth stages; CONTAINS InterPro DOMAIN/s: Heavy metal transport/detoxification protein (InterPro:IPR006121); BEST Arabidopsis thaliana protein match is: Copper transport protein family (TAIR:AT5G52760.1)
AT1G04760	*0,967*	VESICLE-ASSOCIATED MEMBRANE PROTEIN 726 (VAMP726)	member of Synaptobrevin -like protein family

AT1G31650	*0,967*	ROP (RHO OF PLANTS) GUANINE NUCLEOTIDE EXCHANGE FACTOR 14 (ROPGEF14)	Encodes a member of KPP-like gene family, homolog of KPP (kinase partner protein) gene in tomato. Also a member of the RopGEF (guanine nucleotide exchange factor) family, containing the novel PRONE domain (plant-specific Rop nucleotide exchanger), which is exclusively active towards members of the Rop subfamily.
AT2G44300	*0,967*		Bifunctional inhibitor/lipid-transfer protein/seed storage 2S albumin superfamily protein; FUNCTIONS IN: lipid binding; INVOLVED IN: lipid transport; LOCATED IN: plasma membrane, anchored to membrane; EXPRESSED IN: 21 plant structures; EXPRESSED DURING: 13 growth stages; CONTAINS InterPro DOMAIN/s: Bifunctional inhibitor/plant lipid transfer protein/seed storage (InterPro:IPR016140), Plant lipid transfer protein/seed storage/trypsin-alpha amylase inhibitor (InterPro:IPR003612), Plant lipid transfer protein/Par allergen (InterPro:IPR000528), Plant lipid transfer protein/hydrophobic protein, helical domain (InterPro:IPR013770); BEST Arabidopsis thaliana protein match is: Bifunctional inhibitor/lipid-transfer protein/seed storage 2S albumin superfamily protein (TAIR:AT2G44290.1)
AT1G19180	*0,966*	JASMONATE-ZIM-DOMAIN PROTEIN 1 (JAZ1)	JAZ1 is a nuclear-localized protein involved in jasmonate signaling. JAZ1 transcript levels rise in response to a jasmonate stimulus. JAZ1 can interact with the COI1 F-box subunit of an SCF E3 ubiquitin ligase in a yeast-two-hybrid assay only in the presence of jasmonate-isoleucine (JA-ILE) or coronatine. Application of jasmonate methyl ester to Arabidopsis roots reduces the levels of a JAZ1:GUS fusion protein, presumably by stimulating ubiquitin-proteasome-mediated degradation. The Jas domain appears to be important for JAZ1-COI1 interactions in the presence of coronatine. Two positive residues (R205 and R206) in the Jas domain shown to be important for coronatine -dependent COI1 binding are not required for binding AtMYC2. The mRNA is cell-to-cell mobile.
AT1G77490	*0,966*	THYLAKOIDAL ASCORBATE PEROXIDASE (TAPX)	Encodes a chloroplastic thylakoid ascorbate peroxidase tAPX. Ascorbate peroxidases are enzymes that scavenge hydrogen peroxide in plant cells. Eight types of APX have been described for Arabidopsis: three cytosolic (APX1, APX2, APX6), two chloroplastic types (stromal sAPX, thylakoid tAPX), and three microsomal (APX3, APX4, APX5) isoforms.
AT2G33530	*0,966*	SERINE CARBOXYPEPTIDASE-LIKE 46 (scpl46)	serine carboxypeptidase-like 46 (scpl46); FUNCTIONS IN: serine-type carboxypeptidase activity; INVOLVED IN: proteolysis; LOCATED IN: plant-type cell wall; EXPRESSED IN: 21 plant structures; EXPRESSED DURING: 13 growth stages; CONTAINS InterPro DOMAIN/s: Peptidase S10, serine carboxypeptidase (InterPro:IPR001563), Peptidase S10, serine carboxypeptidase, active site (InterPro:IPR018202); BEST Arabidopsis thaliana protein match is: serine carboxypeptidase-like 45 (TAIR:AT1G28110.2)
AT4G32520	*0,965*	SERINE HYDROXYMETHYLTRANSFERAS E 3 (SHM3)	Encodes a serine hydroxymethyltransferase SHMT3 located in the plastid.
AT1G63750	*0,964*		Disease resistance protein (TIR-NBS-LRR class) family; FUNCTIONS IN: transmembrane receptor activity, nucleoside-triphosphatase activity, nucleotide binding, ATP binding; INVOLVED IN: signal transduction, apoptosis, defense response, innate immune response; LOCATED IN: intrinsic to membrane; EXPRESSED IN: 7 plant structures; EXPRESSED DURING: 7 growth stages; CONTAINS InterPro DOMAIN/s: ATPase, AAA+ type, core (InterPro:IPR003593), NB-ARC (InterPro:IPR002182), Leucine-rich repeat (InterPro:IPR001611), Toll-Interleukin receptor (InterPro:IPR000157), Disease resistance protein (InterPro:IPR000767); BEST Arabidopsis thaliana protein match is: Disease resistance protein (TIR-NBS-LRR class) family (TAIR:AT5G58120.1)
AT2G37280	*0,964*	ATP-BINDING CASSETTE G33 (ABCG33)	Encodes an ATP-binding cassette (ABC) transporter. Expressed in the vascular tissue of primary stem.
AT5G64550	*0,962*		loricrin-related; BEST Arabidopsis thaliana protein match is: loricrin-related (TAIR:AT5G09670.1)
AT2G40080	*0,961*	EARLY FLOWERING 4 (ELF4)	Encodes a novel nuclear 111 amino-acid phytochrome-regulated component of a negative feedback loop involving the circadian clock central oscillator components CCA1 and LHY. ELF4 is necessary for light-induced expression of both CCA1 and LHY, and conversely, CCA1 and LHY act negatively on light-induced ELF4 expression. ELF4 promotes clock accuracy and is required for sustained rhythms in the absence of daily light/dark cycles. It is involved in the phyB-mediated constant red light induced seedling de-etiolation process and may function to coregulate the expression of a subset of phyB-regulated genes.

AT2G34140	*0,96*	CYCLING DOF FACTOR 4 (CDF4)	CDF4 is member of the group II DOF transcription factor family is involved in regulation of differentiation root columella cells. It is a direct target of the transcriptional repressor WOX5. CDF4 itself is a transcriptional repressor that appears to repress root columella stem cell identity. Ectopic expression of CDF leads to premature differentiation of root columella cells.
AT4G02330	*0,96*	(ATPMEPCRB)	Encodes a pectin methylesterase that is sensitive to chilling stress and brassinosteroid regulation.
AT5G01670	*0,96*		NAD(P)-linked oxidoreductase superfamily protein; FUNCTIONS IN: oxidoreductase activity; INVOLVED IN: oxidation reduction; EXPRESSED IN: 18 plant structures; EXPRESSED DURING: 11 growth stages; CONTAINS InterPro DOMAIN/s: Aldo/keto reductase (InterPro:IPR001395), Aldo/keto reductase subgroup (InterPro:IPR020471), Aldo/keto reductase, conserved site (InterPro:IPR018170); BEST Arabidopsis thaliana protein match is: NAD(P)-linked oxidoreductase superfamily protein (TAIR:AT2G37770.2)
AT5G54510	*0,959*	DWARF IN LIGHT 1 (DFL1)	Encodes an IAA-amido synthase that conjugates Ala, Asp, Phe, and Trp to auxin. Lines overexpressing this gene accumulate IAA-ASP and are hypersensitive to several auxins. Identified as a dominant mutation that displays shorter hypocotyls in light grown plants when compared to wild type siblings. Protein is similar to auxin inducible gene from pea (GH3).
AT4G38780	*0,958*		Pre-mRNA-processing-splicing factor; FUNCTIONS IN: molecular_function unknown; INVOLVED IN: nuclear mRNA splicing, via spliceosome; LOCATED IN: spliceosomal complex; EXPRESSED IN: 19 plant structures; EXPRESSED DURING: 8 growth stages; CONTAINS InterPro DOMAIN/s: Mov34/MPN/PAD-1 (InterPro:IPR000555), Pre-mRNA-processing-splicing factor 8, U5-snRNA-binding (InterPro:IPR019581), Pre-mRNA-processing-splicing factor 8 (InterPro:IPR012591), Pre-mRNA-processing-splicing factor 8, U6-snRNA-binding (InterPro:IPR019580), PROCN (InterPro:IPR012592), PRP8 domain IV core (InterPro:IPR021983), PRO, C-terminal (InterPro:IPR012984), RNA recognition motif, spliceosomal PrP8 (InterPro:IPR019582); BEST Arabidopsis thaliana protein match is: Pre-mRNA-processing-splicing factor (TAIR:AT1G80070.1)
AT5G52970	*0,958*		thylakoid lumen 15.0 kDa protein; FUNCTIONS IN: molecular_function unknown; INVOLVED IN: biological_process unknown; LOCATED IN: thylakoid, thylakoid lumen, chloroplast thylakoid lumen, chloroplast; EXPRESSED IN: 20 plant structures; EXPRESSED DURING: 13 growth stages
AT1G17480	*0,957*	IQ-DOMAIN 7 (IQD7)	IQ-domain 7 (IQD7); CONTAINS InterPro DOMAIN/s: IQ calmodulin-binding region (InterPro:IPR000048); BEST Arabidopsis thaliana protein match is: IQ-domain 8 (TAIR:AT1G72670.1)
AT1G65910	*0,957*	NAC DOMAIN CONTAINING PROTEIN 28 (NAC028)	NAC domain containing protein 28 (NAC028); FUNCTIONS IN: sequence-specific DNA binding transcription factor activity; INVOLVED IN: multicellular organismal development, regulation of transcription; LOCATED IN: cellular_component unknown; EXPRESSED IN: 19 plant structures; EXPRESSED DURING: 10 growth stages; CONTAINS InterPro DOMAIN/s: No apical meristem (NAM) protein (InterPro:IPR003441); BEST Arabidopsis thaliana protein match is: NAC domain containing protein 86 (TAIR:AT5G17260.1)
AT5G49700	*0,957*	AT-HOOK MOTIF NUCLEAR LOCALIZED PROTEIN 17 (AHL17)	Predicted AT-hook DNA-binding family protein; CONTAINS InterPro DOMAIN/s: Protein of unknown function DUF296 (InterPro:IPR005175), Predicted AT-hook DNA-binding (InterPro:IPR014476); BEST Arabidopsis thaliana protein match is: Predicted AT-hook DNA-binding family protein (TAIR:AT1G14490.1)
AT1G67560	*0,956*	LIPOXYGENASE 6 (LOX6)	PLAT/LH2 domain-containing lipoxygenase family protein; FUNCTIONS IN: oxidoreductase activity, acting on single donors with incorporation of molecular oxygen, incorporation of two atoms of oxygen, lipoxygenase activity, iron ion binding, metal ion binding; INVOLVED IN: oxidation reduction, growth; LOCATED IN: chloroplast; EXPRESSED IN: 22 plant structures; EXPRESSED DURING: 13 growth stages; CONTAINS InterPro DOMAIN/s: Lipoxygenase, iron binding site (InterPro:IPR020833), Lipoxygenase, C-terminal (InterPro:IPR013819), Lipoxygenase, LH2 (InterPro:IPR001024), Lipase/lipooxygenase, PLAT/LH2 (InterPro:IPR008976), Lipoxygenase, conserved site (InterPro:IPR020834), Lipoxygenase (InterPro:IPR000907), Lipoxygenase, plant (InterPro:IPR001246); BEST Arabidopsis thaliana protein match is: PLAT/LH2 domain-containing lipoxygenase family protein (TAIR:AT1G72520.1)
AT1G32200	*0,955*	(ATS1)	Encodes a chloroplast glycerol-3-phosphate acyltransferase.Involved in the biosynthesis of chloroplast phosphatidylglycerol.
AT1G63240	*0,954*		unknown protein

AT2G39240	*0,954*		RNA polymerase I specific transcription initiation factor RRN3 protein; FUNCTIONS IN: RNA polymerase I transcription factor activity, binding; CONTAINS InterPro DOMAIN/s: RNA polymerase I specific transcription initiation factor RRN3 (InterPro:IPR007991), Armadillo-type fold (InterPro:IPR016024); BEST Arabidopsis thaliana protein match is: RNA polymerase I specific transcription initiation factor RRN3 protein (TAIR:AT2G34750.1)
AT2G46400	*0,954*	WRKY DNA-BINDING PROTEIN 46 (WRKY46)	Encodes a WRKY transcription factor that contributes to the feedforward inhibition of osmotic/salt stress-dependent LR inhibition via regulation of ABA signaling and auxin homeostasis.
AT5G47910	*0,954*	RESPIRATORY BURST OXIDASE HOMOLOGUE D (RBOHD)	NADPH/respiratory burst oxidase protein D (RbohD).Interacts with AtrbohF gene to fine tune the spatial control of ROI production and hypersensitive response to cell in and around infection site. The mRNA is cell-to-cell mobile.
AT4G25150	*0,953*		HAD superfamily, subfamily IIIB acid phosphatase ; FUNCTIONS IN: acid phosphatase activity; INVOLVED IN: biological_process unknown; LOCATED IN: endomembrane system; EXPRESSED IN: 21 plant structures; EXPRESSED DURING: 9 growth stages; CONTAINS InterPro DOMAIN/s: Acid phosphatase (Class B) (InterPro:IPR005519), Vegetative storage protein/acid phosphatase (InterPro:IPR014403), Acid phosphatase, plant (InterPro:IPR010028); BEST Arabidopsis thaliana protein match is: HAD superfamily, subfamily IIIB acid phosphatase (TAIR:AT5G51260.1)
AT5G43440	*0,953*		encodes a protein whose sequence is similar to ACC oxidase
AT5G65470	*0,953*		O-fucosyltransferase family protein; CONTAINS InterPro DOMAIN/s: GDP-fucose protein O-fucosyltransferase (InterPro:IPR019378); BEST Arabidopsis thaliana protein match is: O-fucosyltransferase family protein (TAIR:AT4G24530.1)
AT2G39220	*0,952*	PATATIN-LIKE PROTEIN 6 (PLP6)	PATATIN-like protein 6 (PLP6); FUNCTIONS IN: nutrient reservoir activity; INVOLVED IN: metabolic process, lipid metabolic process; EXPRESSED IN: 22 plant structures; EXPRESSED DURING: 13 growth stages; CONTAINS InterPro DOMAIN/s: Acyl transferase/acyl hydrolase/lysophospholipase (InterPro:IPR016035), Patatin (InterPro:IPR002641); BEST Arabidopsis thaliana protein match is: patatin-like protein 6 (TAIR:AT3G54950.1)
AT3G25690	*0,952*	CHLOROPLAST UNUSUAL POSITIONING 1 (CHUP1)	actin binding protein required for normal chloroplast positioning The mRNA is cell-to-cell mobile.
AT3G50970	*0,952*	LOW TEMPERATURE-INDUCED 30 (LTI30)	Belongs to the dehydrin protein family, which contains highly conserved stretches of 7-17 residues that are repetitively scattered in their sequences, the K-, S-, Y- and lysine rich segments. LTI29 and LTI30 double overexpressors confer freeze tolerance. Located in membranes. mRNA upregulated by water deprivation and abscisic acid. The mRNA is cell-to-cell mobile.
AT4G19170	*0,952*	NINE-CIS-EPOXYCAROTENOID DIOXYGENASE 4 (NCED4)	chloroplast-targeted member of a family of enzymes similar to nine-cis-epoxycarotenoid dioxygenase The mRNA is cell-to-cell mobile.
AT4G35290	*0,952*	GLUTAMATE RECEPTOR 2 (GLUR2)	Encodes a putative glutamate receptor like-protein, member of Putative ligand-gated ion channel subunit family
AT2G17230	*0,949*	EXORDIUM LIKE 5 (EXL5)	EXORDIUM like 5 (EXL5); FUNCTIONS IN: molecular_function unknown; INVOLVED IN: biological_process unknown; LOCATED IN: endomembrane system; EXPRESSED IN: 22 plant structures; EXPRESSED DURING: 13 growth stages; CONTAINS InterPro DOMAIN/s: Phosphate-induced protein 1 (InterPro:IPR006766); BEST Arabidopsis thaliana protein match is: EXORDIUM like 1 (TAIR:AT2G35150.1)
AT4G32300	*0,948*	S-DOMAIN-2 5 (SD2-5)	S-domain-2 5 (SD2-5); FUNCTIONS IN: carbohydrate binding, protein kinase activity, kinase activity; INVOLVED IN: protein amino acid autophosphorylation; LOCATED IN: plasma membrane; EXPRESSED IN: 25 plant structures; EXPRESSED DURING: 15 growth stages; CONTAINS InterPro DOMAIN/s: Curculin-like (mannose-binding) lectin (InterPro:IPR001480), Protein kinase, ATP binding site (InterPro:IPR017441), Protein kinase, catalytic domain (InterPro:IPR000719), Serine/threonine-protein kinase-like domain (InterPro:IPR017442), Protein kinase-like domain (InterPro:IPR011009), Serine/threonine-protein kinase, active site (InterPro:IPR008271); BEST Arabidopsis thaliana protein match is: S-locus lectin protein kinase family protein (TAIR:AT5G35370.1)

AT5G02790	*0,948*	GLUTATHIONE TRANSFERASE L3 (GSTL3)	Glutathione transferase L3 (GSTL3); INVOLVED IN: response to cadmium ion; EXPRESSED IN: 24 plant structures; EXPRESSED DURING: 14 growth stages; CONTAINS InterPro DOMAIN/s: Thioredoxin fold (InterPro:IPR012335), Glutathione S-transferase, C-terminal (InterPro:IPR004046), Glutathione S-transferase, C-terminal-like (InterPro:IPR010987), Glutathione S-transferase/chloride channel, C-terminal (InterPro:IPR017933), Glutathione S-transferase, N-terminal (InterPro:IPR004045), Thioredoxin-like fold (InterPro:IPR012336); BEST Arabidopsis thaliana protein match is: glutathione transferase lambda 1 (TAIR:AT5G02780.1)
AT1G62750	*0,947*	SNOWY COTYLEDON 1 (SCO1)	Nuclear encoded protein consists of the five domains conserved in EF-G proteins, with two GTP-binding sites in the first domain, and an additional transit peptide at the N-terminus. Localized in chloroplasts. Point mutation results in a delay in the onset of germination. At early developmental stage embryos still contain undifferentiated proplastids. The greening of cotyledons is severely impaired in light-grown mutant sco1 seedlings, whereas the following true leaves develop normally as in wild-type plants.
AT2G39700	*0,946*	EXPANSIN A4 (EXPA4)	putative expansin. Naming convention from the Expansin Working Group (Kende et al, 2004. Plant Mol Bio). Involved in the formation of nematode-induced syncytia in roots of Arabidopsis thaliana.
AT4G03960	*0,946*	PLANT AND FUNGI ATYPICAL DUAL-SPECIﬁCITY PHOSPHATASE 4 (PFA-DSP4)	Encodes an atypical dual-speciﬁcity phosphatase involved in the negative regulation of defense response to a bacterial pathogen, P. syringae pv. tomato.
AT5G20860	*0,946*		Plant invertase/pectin methylesterase inhibitor superfamily; FUNCTIONS IN: enzyme inhibitor activity, pectinesterase activity; INVOLVED IN: cell wall modification; LOCATED IN: endomembrane system, cell wall, plant-type cell wall; EXPRESSED IN: flower; CONTAINS InterPro DOMAIN/s: Pectinesterase, active site (InterPro:IPR018040), Pectin lyase fold/virulence factor (InterPro:IPR011050), Pectinesterase, catalytic (InterPro:IPR000070), Pectinesterase inhibitor (InterPro:IPR006501), Pectin lyase fold (InterPro:IPR012334); BEST Arabidopsis thaliana protein match is: methylesterase PCR A (TAIR:AT1G11580.1)
AT2G33620	*0,944*	AT-HOOK MOTIF NUCLEAR LOCALIZED PROTEIN 10 (AHL10)	AT hook motif DNA-binding family protein; FUNCTIONS IN: DNA binding; LOCATED IN: cytosol; EXPRESSED IN: 25 plant structures; EXPRESSED DURING: 15 growth stages; CONTAINS InterPro DOMAIN/s: Protein of unknown function DUF296 (InterPro:IPR005175), AT hook, DNA-binding motif (InterPro:IPR017956); BEST Arabidopsis thaliana protein match is: AT-hook motif nuclear-localized protein 1 (TAIR:AT4G12080.1)
AT1G66760	*0,943*		MATE efflux family protein; FUNCTIONS IN: antiporter activity, drug transmembrane transporter activity, transporter activity; INVOLVED IN: response to wounding; LOCATED IN: membrane; EXPRESSED IN: 17 plant structures; EXPRESSED DURING: 11 growth stages; CONTAINS InterPro DOMAIN/s: MATE family transporter related protein (InterPro:IPR015521), Multi antimicrobial extrusion protein MatE (InterPro:IPR002528); BEST Arabidopsis thaliana protein match is: MATE efflux family protein (TAIR:AT1G66780.1)
AT4G32620	*0,943*		Enhancer of polycomb-like transcription factor protein; FUNCTIONS IN: nucleic acid binding; INVOLVED IN: biological_process unknown; LOCATED IN: cellular_component unknown; EXPRESSED IN: 24 plant structures; EXPRESSED DURING: 14 growth stages; CONTAINS InterPro DOMAIN/s: Tudor domain (InterPro:IPR002999), Enhancer of polycomb-like (InterPro:IPR019542); BEST Arabidopsis thaliana protein match is: Enhancer of polycomb-like transcription factor protein (TAIR:AT5G04670.1)
AT5G01410	*0,943*	REDUCED SUGAR RESPONSE 4 (RSR4)	Encodes a protein predicted to function in tandem with PDX2 to form glutamine amidotransferase complex with involved in vitamin B6 biosynthesis.
AT2G22610	*0,942*		Di-glucose binding protein with Kinesin motor domain; FUNCTIONS IN: microtubule motor activity, ATP binding; INVOLVED IN: microtubule-based movement; EXPRESSED IN: 20 plant structures; EXPRESSED DURING: 10 growth stages; CONTAINS InterPro DOMAIN/s: Kinesin, motor region, conserved site (InterPro:IPR019821), Malectin/receptor-like protein kinase (InterPro:IPR021720), Kinesin, motor domain (InterPro:IPR001752); BEST Arabidopsis thaliana protein match is: Di-glucose binding protein with Kinesin motor domain (TAIR:AT1G72250.2)
AT5G19500	*0,942*		Encodes a putative amino acid transporter that localizes to the chloroplast inner envelope membrane.
AT1G28360	*0,941*	ERF DOMAIN PROTEIN 12 (ERF12)	encodes a member of the ERF (ethylene response factor) subfamily B-1 of ERF/AP2 transcription factor family (ERF12). The protein contains one AP2 domain. There are 15 members in this subfamily including ATERF-3, ATERF-4, ATERF-7, and leafy petiole.

AT3G16520	*0,941*	UDP-GLUCOSYL TRANSFERASE 88A1 (UGT88A1)	UDP-glucosyl transferase 88A1 (UGT88A1); FUNCTIONS IN: in 6 functions; INVOLVED IN: metabolic process; LOCATED IN: cellular_component unknown; EXPRESSED IN: 22 plant structures; EXPRESSED DURING: 13 growth stages; CONTAINS InterPro DOMAIN/s: UDP-glucuronosyl/UDP-glucosyltransferase (InterPro:IPR002213); BEST Arabidopsis thaliana protein match is: UDP-glucosyl transferase 72B3 (TAIR:AT1G01420.1)
AT1G67850	*0,94*		Protein of unknown function (DUF707); CONTAINS InterPro DOMAIN/s: Protein of unknown function DUF707 (InterPro:IPR007877); BEST Arabidopsis thaliana protein match is: Protein of unknown function (DUF707) (TAIR:AT1G13000.2)
AT1G11170	*0,938*		Protein of unknown function (DUF707); CONTAINS InterPro DOMAIN/s: Protein of unknown function DUF707 (InterPro:IPR007877); BEST Arabidopsis thaliana protein match is: Protein of unknown function (DUF707) (TAIR:AT1G61240.4)
AT1G76730	*0,938*	CLUSTERS OF ORTHOLOGOUS GROUP 212 (COG0212)	Encodes a paralog of ATP-dependent folate salvage enzyme 5-formyltetrahydrofolate cycloligase (5-FCL) that is targeted to chloroplasts and to be required for embryo viability and lacks 5-FCL activity.
AT2G21960	*0,937*		unknown protein; LOCATED IN: chloroplast; EXPRESSED IN: 22 plant structures; EXPRESSED DURING: 13 growth stages; BEST Arabidopsis thaliana protein match is: unknown protein (TAIR:AT1G56180.1)
AT4G23180	*0,937*	CYSTEINE-RICH RLK (RECEPTOR-LIKE PROTEIN KINASE) 10 (CRK10)	Encodes a receptor-like protein kinase. Naming convention from Chen et al 2003 (PMID 14756307) The mRNA is cell-to-cell mobile.
AT4G32770	*0,937*	VITAMIN E DEFICIENT 1 (VTE1)	Tocopherol cyclase involved in tocopherol (vitamin E)synthesis. VTE1 over-expressing plants have increased tocopherol indicating VTE1 is a major limiting factor in tocopherol synthesis. Mutants defective in this gene accumulate high amounts of zeaxanthin in conditions of high light or low temperature. Plays a role in the adaptation to low temperature stress, notably phloem loading.
AT1G58170	*0,936*		Disease resistance-responsive (dirigent-like protein) family protein; FUNCTIONS IN: molecular_function unknown; INVOLVED IN: N-terminal protein myristoylation, lignan biosynthetic process, defense response; LOCATED IN: endomembrane system; EXPRESSED IN: 17 plant structures; EXPRESSED DURING: 6 growth stages; CONTAINS InterPro DOMAIN/s: Plant disease resistance response protein (InterPro:IPR004265); BEST Arabidopsis thaliana protein match is: Disease resistance-responsive (dirigent-like protein) family protein (TAIR:AT5G49040.1)
AT2G16700	*0,936*	ACTIN DEPOLYMERIZING FACTOR 5 (ADF5)	Encodes actin depolymerizing factor 5 (ADF5).
AT2G25270	*0,936*		unknown protein; LOCATED IN: plasma membrane; EXPRESSED IN: 18 plant structures; EXPRESSED DURING: 13 growth stages; BEST Arabidopsis thaliana protein match is: unknown protein (TAIR:AT2G12400.1)
AT5G61370	*0,936*		Pentatricopeptide repeat (PPR) superfamily protein; FUNCTIONS IN: molecular_function unknown; INVOLVED IN: biological_process unknown; LOCATED IN: mitochondrion; EXPRESSED IN: 19 plant structures; EXPRESSED DURING: 8 growth stages; CONTAINS InterPro DOMAIN/s: Pentatricopeptide repeat (InterPro:IPR002885); BEST Arabidopsis thaliana protein match is: Pentatricopeptide repeat (PPR) superfamily protein (TAIR:AT5G60960.1)
AT3G23810	*0,935*	S-ADENOSYL-L-HOMOCYSTEINE (SAH) HYDROLASE 2 (SAHH2)	S-adenosyl-l-homocysteine (SAH) hydrolase 2 (SAHH2); FUNCTIONS IN: adenosylhomocysteinase activity; INVOLVED IN: one-carbon metabolic process; LOCATED IN: plasma membrane, vacuole, membrane; EXPRESSED IN: 33 plant structures; EXPRESSED DURING: 15 growth stages; CONTAINS InterPro DOMAIN/s: S-adenosyl-L-homocysteine hydrolase (InterPro:IPR000043), S-adenosyl-L-homocysteine hydrolase, conserved site (InterPro:IPR020082), S-adenosyl-L-homocysteine hydrolase, NAD binding (InterPro:IPR015878); BEST Arabidopsis thaliana protein match is: S-adenosyl-L-homocysteine hydrolase (TAIR:AT4G13940.1)
AT1G43650	*0,934*	USUALLY MULTIPLE ACIDS MOVE IN AND OUT TRANSPORTERS 22 (UMAMIT22)	nodulin MtN21-like transporter family protein
AT1G79900	*0,934*	(BAC2)	encodes a mitochondrial ornithine transporter that exports ornithine from the mitochondria to the cytosol
AT4G23060	*0,934*	IQ-DOMAIN 22 (IQD22)	IQ-domain 22 (IQD22); CONTAINS InterPro DOMAIN/s: IQ calmodulin-binding region (InterPro:IPR000048); BEST Arabidopsis thaliana protein match is: IQ-domain 26 (TAIR:AT3G16490.1)

AT4G29840	0,934	METHIONINE OVER-ACCUMULATOR 2 (MTO2)	threonine synthase
AT1G59620	0,933	(CW9)	Encodes CW9.
AT1G78070	0,933		Transducin/WD40 repeat-like superfamily protein; CONTAINS InterPro DOMAIN/s: WD40 repeat 2 (InterPro:IPR019782), WD40 repeat-like-containing domain (InterPro:IPR011046), WD40 repeat, conserved site (InterPro:IPR019775), WD40-repeat-containing domain (InterPro:IPR017986), WD40/YVTN repeat-like-containing domain (InterPro:IPR015943), WD40 repeat (InterPro:IPR001680), WD40 repeat, subgroup (InterPro:IPR019781); BEST Arabidopsis thaliana protein match is: Transducin/WD40 repeat-like superfamily protein (TAIR:AT1G36070.1)
AT2G28900	0,933	OUTER PLASTID ENVELOPE PROTEIN 16-1 (OEP16-1)	Encodes AtOEP16, a 16-KDa plastid outer membrane protein involved in plastid import of protochlorophyllide oxidoreductase A. Predominantly expressed in leaves and is also inducible by cold treatment.
AT5G57030	0,933	LUTEIN DEFICIENT 2 (LUT2)	Lutein-deficient 2 (LUT2) required for lutein biosynthesis, member of the xanthophyll class of carotenoids. Encodes lycopene epsilon cyclase
AT1G31180	0,932	ISOPROPYLMALATE DEHYDROGENASE 3 (IMD3)	The AtIMD3 is one out of 3 genes encoding the enzyme 3-isopropylmalate dehydrogenase involved in leucine biosynthesis in Arabidopsis. Its subcellular location has been targeted to plastids.
AT2G30550	0,932	DAD1-LIKE LIPASE 3 (DALL3)	Encodes a lipase that hydrolyzes phosphatidylcholine, glycolipids as well as triacylglycerols.
AT4G28950	0,932	RHO-RELATED PROTEIN FROM PLANTS 9 (ROP9)	A member of ROP GTPase gene family.
AT5G16200	0,932		50S ribosomal protein-related; BEST Arabidopsis thaliana protein match is: unknown protein (TAIR:AT1G66890.1)
AT5G54290	0,931	(CcdA)	Encodes CcdA, a thylakoid membrane protein required for the transfer of reducing equivalents from stroma to thylakoid lumen.
AT5G62530	0,931	ALDEHYDE DEHYDROGENASE 12A1 (ALDH12A1)	Encodes mitochondrial Delta-pyrroline-5- carboxylate dehydrogenase. Involved in the catabolism of proline to glutamate. Involved in protection from proline toxicity. Induced at pathogen infection sites. P5CDH and SRO5 (an overlapping gene in the sense orientation) generate 24-nt and 21-nt siRNAs, which together are components of a regulatory loop controlling reactive oxygen species (ROS) production and stress response.
AT5G64310	0,931	ARABINOGALACTAN PROTEIN 1 (AGP1)	Encodes arabinogalactan-protein (AGP1). The mRNA is cell-to-cell mobile.
AT5G66120	0,931		3-dehydroquinate synthase, putative; FUNCTIONS IN: 3-dehydroquinate synthase activity; INVOLVED IN: aromatic amino acid family biosynthetic process; LOCATED IN: chloroplast; EXPRESSED IN: 23 plant structures; EXPRESSED DURING: 14 growth stages; CONTAINS InterPro DOMAIN/s: 3-dehydroquinate synthase AroB, subgroup (InterPro:IPR016037), 3-dehydroquinate synthase AroB (InterPro:IPR002658)
AT1G21270	0,93	WALL-ASSOCIATED KINASE 2 (WAK2)	cytoplasmic serine/threonine protein kinase induced by salicylic acid. mutant plants exhibit a loss of cell expansion and dependence on sugars and salts for seedling growth, affecting the expression and activity of vacuolar invertase.
AT1G61190	0,929		Putative CC-NB-LRR resistance gene. It's ortholog in ecotype Wei-0 RPP39 (R_190-Wei-0, accession number JQ045573, PMID:22359513) has been shown experimentally to recognize the effector ATR39-1 produced by Hyaloperonospora arabidopsidis (Hpa). The mRNA is cell-to-cell mobile.
AT1G77120	0,929	ALCOHOL DEHYDROGENASE 1 (ADH1)	Catalyzes the reduction of acetaldehyde using NADH as reductant. Requires zinc for activity. Dimer. Anaerobic response polypeptide (ANP). Fermentation. The protein undergoes thiolation following treatment with the oxidant tert-butylhydroperoxide. The mRNA is cell-to-cell mobile.
AT3G17040	0,928	HIGH CHLOROPHYLL FLUORESCENT 107 (HCF107)	It is a RNA tetratricopeptide repeat-containing protein required for normal processing of transcripts from the polycistronic chloroplast psbB-psbT-psbH-petB-petD operon coding for proteins of the photosystem II and cytochrome b6/f complexes. Localizes to the chloroplast membrane. Involved in regulating plastidial gene expression and biogenesis. It binds in the psbT?psbH intercistronic region and blocks the progression of 5′ → 3′ exoribonucleases, which defines the 5′ end of processed psbH transcripts and also stabilizes the downstream RNA segment. In addition, HCF107 binding remodels the structure of the psbH 5′ UTR in a way that can account for its ability to enhance psbH translation.

AT4G39710	*0,928*	PHOTOSYNTHETIC NDH SUBCOMPLEX L 4 (PnsL4)	FK506-binding protein 16-2 (FKBP16-2); FUNCTIONS IN: FK506 binding, peptidyl-prolyl cis-trans isomerase activity; INVOLVED IN: protein folding; LOCATED IN: thylakoid, chloroplast thylakoid membrane, chloroplast thylakoid lumen, chloroplast; EXPRESSED IN: 20 plant structures; EXPRESSED DURING: 13 growth stages; CONTAINS InterPro DOMAIN/s: Peptidyl-prolyl cis-trans isomerase, FKBP-type (InterPro:IPR001179); BEST Arabidopsis thaliana protein match is: FK506-binding protein 13 (TAIR:AT5G45680.1)
AT4G06746	*0,927*	RELATED TO AP2 9 (RAP2.9)	encodes a member of the DREB subfamily A-5 of ERF/AP2 transcription factor family (RAP2.9). The protein contains one AP2 domain. There are 16 members in this subfamily including RAP2.1 and RAP2.10.
AT1G28050	*0,926*	B-BOX DOMAIN PROTEIN 13 (BBX13)	B-box type zinc finger protein with CCT domain; FUNCTIONS IN: sequence-specific DNA binding transcription factor activity, zinc ion binding; INVOLVED IN: regulation of transcription; LOCATED IN: intracellular; EXPRESSED IN: 21 plant structures; EXPRESSED DURING: 13 growth stages; CONTAINS InterPro DOMAIN/s: CCT domain (InterPro:IPR010402), Zinc finger, B-box (InterPro:IPR000315); BEST Arabidopsis thaliana protein match is: B-box type zinc finger protein with CCT domain (TAIR:AT2G33500.2)
AT1G75280	*0,926*		isoflavone reductase, putative, identical to SP:P52577 Isoflavone reductase homolog P3 (EC 1.3.1.-) {Arabidopsis thaliana}; contains Pfam profile PF02716: isoflavone reductase. Involved in response to oxidative stress. The mRNA is cell-to-cell mobile.
AT4G21570	*0,926*		Protein of unknown function (DUF300); INVOLVED IN: biological_process unknown; LOCATED IN: endomembrane system; CONTAINS InterPro DOMAIN/s: Protein of unknown function DUF300 (InterPro:IPR005178); BEST Arabidopsis thaliana protein match is: Protein of unknown function (DUF300) (TAIR:AT1G11200.1)
AT4G03280	*0,925*	PHOTOSYNTHETIC ELECTRON TRANSFER C (PETC)	Encodes the Rieske FeS center of cytochrome b6f complex. Gene is expressed in shoot but not in root. Mutant has reduced electron transport at saturating light intensities and Q-cycle activity is hypersensitive to acidification of the thylakoid lumen. The mRNA is cell-to-cell mobile.
AT5G65600	*0,925*	L-TYPE LECTIN RECEPTOR KINASE IX.2 (LECRK-IX.2)	Concanavalin A-like lectin protein kinase family protein; FUNCTIONS IN: kinase activity; INVOLVED IN: protein amino acid phosphorylation; LOCATED IN: endomembrane system; EXPRESSED IN: stem; CONTAINS InterPro DOMAIN/s: Legume lectin, beta chain (InterPro:IPR001220), Protein kinase, ATP binding site (InterPro:IPR017441), Serine/threonine-protein kinase-like domain (InterPro:IPR017442), Concanavalin A-like lectin/glucanase, subgroup (InterPro:IPR013320), Protein kinase-like domain (InterPro:IPR011009), Serine/threonine-protein kinase, active site (InterPro:IPR008271), Protein kinase, catalytic domain (InterPro:IPR000719), Concanavalin A-like lectin/glucanase (InterPro:IPR008985); BEST Arabidopsis thaliana protein match is: Concanavalin A-like lectin protein kinase family protein (TAIR:AT5G10530.1)
AT1G11580	*0,924*	METHYLESTERASE PCR A (PMEPCRA)	methylesterase PCR A (PMEPCRA); FUNCTIONS IN: enzyme inhibitor activity, pectinesterase activity; INVOLVED IN: cell wall modification; LOCATED IN: cell wall, plasma membrane, plant-type cell wall; EXPRESSED IN: 25 plant structures; EXPRESSED DURING: 13 growth stages; CONTAINS InterPro DOMAIN/s: Pectinesterase, active site (InterPro:IPR018040), Pectin lyase fold/virulence factor (InterPro:IPR011050), Pectinesterase inhibitor (InterPro:IPR006501), Pectinesterase, catalytic (InterPro:IPR000070), Pectin lyase fold (InterPro:IPR012334); BEST Arabidopsis thaliana protein match is: Plant invertase/pectin methylesterase inhibitor superfamily (TAIR:AT1G11590.1)
AT1G65920	*0,924*		Regulator of chromosome condensation (RCC1) family with FYVE zinc finger domain; FUNCTIONS IN: zinc ion binding; LOCATED IN: plasma membrane, vacuole; EXPRESSED IN: 11 plant structures; EXPRESSED DURING: 6 growth stages; CONTAINS InterPro DOMAIN/s: Regulator of chromosome condensation, RCC1 (InterPro:IPR000408), Disease resistance/zinc finger/chromosome condensation-like region (InterPro:IPR013591), Pleckstrin homology (InterPro:IPR001849), Zinc finger, FYVE-type (InterPro:IPR000306), Regulator of chromosome condensation/beta-lactamase-inhibitor protein II (InterPro:IPR009091), Zinc finger, FYVE-related (InterPro:IPR017455), Pleckstrin homology-type (InterPro:IPR011993), Zinc finger, FYVE/PHD-type (InterPro:IPR011011); BEST Arabidopsis thaliana protein match is: Regulator of chromosome condensation (RCC1) family with FYVE zinc finger domain (TAIR:AT5G42140.1)
AT5G17700	*0,924*		MATE efflux family protein; FUNCTIONS IN: antiporter activity, drug transmembrane transporter activity, transporter activity; INVOLVED IN: drug transmembrane transport, transmembrane transport; LOCATED IN: membrane; EXPRESSED IN: 22 plant structures; EXPRESSED DURING: 10 growth stages; CONTAINS InterPro DOMAIN/s: Multi antimicrobial extrusion protein MatE (InterPro:IPR002528); BEST Arabidopsis thaliana protein match is: MATE efflux family protein (TAIR:AT3G03620.1)

AT1G23850	*0,922*		unknown protein; BEST Arabidopsis thaliana protein match is: unknown protein (TAIR:AT1G23840.1)
AT3G56400	*0,922*	WRKY DNA-BINDING PROTEIN 70 (WRKY70)	member of WRKY Transcription Factor; Group III. Function as activator of SA-dependent defense genes and a repressor of JA-regulated genes. WRKY70-controlled suppression of JA-signaling is partly executed by NPR1.
AT1G60730	*0,921*		NAD(P)-linked oxidoreductase superfamily protein; FUNCTIONS IN: oxidoreductase activity, aldo-keto reductase activity; INVOLVED IN: oxidation reduction; EXPRESSED IN: 7 plant structures; EXPRESSED DURING: LP.06 six leaves visible, LP.04 four leaves visible, 4 anthesis, petal differentiation and expansion stage; CONTAINS InterPro DOMAIN/s: Aldo/keto reductase (InterPro:IPR001395); BEST Arabidopsis thaliana protein match is: NAD(P)-linked oxidoreductase superfamily protein (TAIR:AT1G60710.1).
AT2G22880	*0,92*	(VQ12)	VQ motif-containing protein; FUNCTIONS IN: molecular_function unknown; INVOLVED IN: biological_process unknown; LOCATED IN: chloroplast; EXPRESSED IN: 8 plant structures; EXPRESSED DURING: LP.06 six leaves visible, LP.04 four leaves visible, LP.10 ten leaves visible, LP.08 eight leaves visible, LP.12 twelve leaves visible; CONTAINS InterPro DOMAIN/s: VQ (InterPro:IPR008889); BEST Arabidopsis thaliana protein match is: VQ motif-containing protein (TAIR:AT4G37710.1)
AT5G24660	*0,919*	RESPONSE TO LOW SULFUR 2 (LSU2)	RESPONSE TO LOW SULFUR 2 (LSU2); BEST Arabidopsis thaliana protein match is: response to low sulfur 4 (TAIR:AT5G24655.1)
AT1G01300	*0,918*		Eukaryotic aspartyl protease family protein; FUNCTIONS IN: aspartic-type endopeptidase activity; INVOLVED IN: proteolysis, response to karrikin; LOCATED IN: membrane, plant-type cell wall; EXPRESSED IN: 22 plant structures; EXPRESSED DURING: 13 growth stages; CONTAINS InterPro DOMAIN/s: Peptidase aspartic (InterPro:IPR021109), Peptidase aspartic, catalytic (InterPro:IPR009007), Peptidase A1 (InterPro:IPR001461); BEST Arabidopsis thaliana protein match is: Eukaryotic aspartyl protease family protein (TAIR:AT3G61820.1)
AT1G14590	*0,917*		Nucleotide-diphospho-sugar transferase family protein; CONTAINS InterPro DOMAIN/s: Nucleotide-diphospho-sugar transferase, predicted (InterPro:IPR005069); BEST Arabidopsis thaliana protein match is: Nucleotide-diphospho-sugar transferase family protein (TAIR:AT2G02061.1)
AT1G16220	*0,917*		Protein phosphatase 2C family protein; FUNCTIONS IN: protein serine/threonine phosphatase activity, catalytic activity; INVOLVED IN: N-terminal protein myristoylation; LOCATED IN: plasma membrane; EXPRESSED IN: 10 plant structures; EXPRESSED DURING: 6 growth stages; CONTAINS InterPro DOMAIN/s: Protein phosphatase 2C-related (InterPro:IPR001932), Protein phosphatase 2C (InterPro:IPR015655), Protein phosphatase 2C, N-terminal (InterPro:IPR014045); BEST Arabidopsis thaliana protein match is: Protein phosphatase 2C family protein (TAIR:AT1G79630.1)
AT2G30990	*0,917*		Protein of unknown function (DUF688); CONTAINS InterPro DOMAIN/s: Protein of unknown function DUF688 (InterPro:IPR007789); BEST Arabidopsis thaliana protein match is: Protein of unknown function (DUF688) (TAIR:AT1G29240.1)
AT4G37970	*0,916*	CINNAMYL ALCOHOL DEHYDROGENASE 6 (CAD6)	cinnamyl alcohol dehydrogenase 6 (CAD6); FUNCTIONS IN: oxidoreductase activity, zinc ion binding; INVOLVED IN: oxidation reduction; LOCATED IN: cellular_component unknown; EXPRESSED IN: 9 plant structures; EXPRESSED DURING: petal differentiation and expansion stage; CONTAINS InterPro DOMAIN/s: GroES-like (InterPro:IPR011032), Alcohol dehydrogenase GroES-like (InterPro:IPR013154), Alcohol dehydrogenase, zinc-containing, conserved site (InterPro:IPR002328), Alcohol dehydrogenase, C-terminal (InterPro:IPR013149), Alcohol dehydrogenase superfamily, zinc-containing (InterPro:IPR002085); BEST Arabidopsis thaliana protein match is: cinnamyl alcohol dehydrogenase 9 (TAIR:AT4G39330.1)
AT5G41460	*0,915*		Protein of unknown function (DUF604); CONTAINS InterPro DOMAIN/s: Protein of unknown function DUF604 (InterPro:IPR006740); BEST Arabidopsis thaliana protein match is: Protein of unknown function (DUF604) (TAIR:AT4G23490.1)
AT1G49870	*0,914*		unknown protein; LOCATED IN: chloroplast; EXPRESSED IN: 10 plant structures; EXPRESSED DURING: F mature embryo stage, petal differentiation and expansion stage, E expanded cotyledon stage, D bilateral stage
AT2G40230	*0,914*		HXXXD-type acyl-transferase family protein; FUNCTIONS IN: transferase activity, transferring acyl groups other than amino-acyl groups, transferase activity; INVOLVED IN: biological_process unknown; LOCATED IN: cellular_component unknown; EXPRESSED IN: 16 plant structures; EXPRESSED DURING: 7 growth stages; CONTAINS InterPro DOMAIN/s: Transferase (InterPro:IPR003480); BEST Arabidopsis thaliana protein match is: HXXXD-type acyl-transferase family protein (TAIR:AT1G27620.1)

AT2G46610	*0,914*	ARGININE/SERINE-RICH SPLICING FACTOR 31A (RS31a)	Barta et al (2010) have proposed a nomenclature for Serine/Arginine-Rich Protein Splicing Factors (SR proteins): Plant Cell. 2010, 22:2926.
AT3G55340	*0,913*	PHRAGMOPLASTIN INTERACTING PROTEIN 1	Plant-specific protein. Interacts with phragmoplastin, Rop1 and Rop2. Involved in cell plate formation.
AT3G55630	*0,913*	DHFS-FPGS HOMOLOG D (DFD)	Encodes one of the three folylpolyglutamate synthetase isoforms (FPGSs): FPGS1 (At5g05980, plastidic), FPGS2 (At3g10160, mitochondrial) and FPGS3 (At3g55630, cytosolic).
AT5G57320	*0,912*	VILLIN 5 (VLN5)	villin, putative; FUNCTIONS IN: actin binding; INVOLVED IN: cytoskeleton organization; LOCATED IN: cellular_component unknown; EXPRESSED IN: petal, leaf whorl, male gametophyte, flower, pollen tube; EXPRESSED DURING: L mature pollen stage, M germinated pollen stage, 4 anthesis, petal differentiation and expansion stage; CONTAINS InterPro DOMAIN/s: Gelsolin (InterPro:IPR007122), Villin headpiece (InterPro:IPR003128), Gelsolin domain (InterPro:IPR007123); BEST Arabidopsis thaliana protein match is: villin 4 (TAIR:AT4G30160.1)
AT5G44870	*0,911*	LAZARUS 5 (LAZ5)	Encodes LAZ5, a TIR-class NB-LRR R protein of unknown pathogen specificity with sequence similarity to RPS4, an R protein conferring resistance to Pseudomonas syringae expressing the effector AvrRPS4. Overexpression of LAZ5 results in hypersensitive cell death (plants did not survive to set seeds).
AT2G32220	*0,91*		Ribosomal L27e protein family; FUNCTIONS IN: structural constituent of ribosome; INVOLVED IN: translation; LOCATED IN: ribosome, cytosolic large ribosomal subunit, intracellular, membrane; EXPRESSED IN: 20 plant structures; EXPRESSED DURING: 13 growth stages; CONTAINS InterPro DOMAIN/s: Ribosomal protein L27e, conserved site (InterPro:IPR018262), Ribosomal protein L27e (InterPro:IPR001141); BEST Arabidopsis thaliana protein match is: Ribosomal L27e protein family (TAIR:AT3G22230.1)
AT1G20920	*0,909*	REGULATOR OF CBF GENE EXPRESSION 1 (RCF1)	P-loop containing nucleoside triphosphate hydrolases superfamily protein; FUNCTIONS IN: helicase activity, nucleic acid binding, ATP-dependent helicase activity, ATP binding; EXPRESSED IN: 25 plant structures; EXPRESSED DURING: 14 growth stages; CONTAINS InterPro DOMAIN/s: DNA/RNA helicase, DEAD/DEAH box type, N-terminal (InterPro:IPR011545), RNA helicase, DEAD-box type, Q motif (InterPro:IPR014014), RNA helicase, ATP-dependent, DEAD-box, conserved site (InterPro:IPR000629), DEAD-like helicase, N-terminal (InterPro:IPR014001), DNA/RNA helicase, C-terminal (InterPro:IPR001650), Helicase, superfamily 1/2, ATP-binding domain (InterPro:IPR014021); BEST Arabidopsis thaliana protein match is: P-loop containing nucleoside triphosphate hydrolases superfamily protein (TAIR:AT3G09620.1)
AT2G30660	*0,909*		ATP-dependent caseinolytic (Clp) protease/crotonase family protein; FUNCTIONS IN: catalytic activity; INVOLVED IN: metabolic process; EXPRESSED IN: 7 plant structures; EXPRESSED DURING: F mature embryo stage, petal differentiation and expansion stage, LP.12 twelve leaves visible; CONTAINS InterPro DOMAIN/s: Crotonase, core (InterPro:IPR001753); BEST Arabidopsis thaliana protein match is: beta-hydroxyisobutyryl-CoA hydrolase 1 (TAIR:AT5G65940.1)
AT2G40240	*0,909*		Tetratricopeptide repeat (TPR)-like superfamily protein; CONTAINS InterPro DOMAIN/s: Pentatricopeptide repeat (InterPro:IPR002885); BEST Arabidopsis thaliana protein match is: Tetratricopeptide repeat (TPR)-like superfamily protein (TAIR:AT3G56030.1)
AT4G27850	*0,909*		Glycine-rich protein family
AT2G41720	*0,908*	EMBRYO DEFECTIVE 2654 (EMB2654)	EMBRYO DEFECTIVE 2654 (EMB2654); INVOLVED IN: embryo development ending in seed dormancy; EXPRESSED IN: 23 plant structures; EXPRESSED DURING: 13 growth stages; CONTAINS InterPro DOMAIN/s: Pentatricopeptide repeat (InterPro:IPR002885); BEST Arabidopsis thaliana protein match is: Pentatricopeptide repeat (PPR) superfamily protein (TAIR:AT5G02860.1)
AT4G22730	*0,907*		Leucine-rich repeat protein kinase family protein; FUNCTIONS IN: protein serine/threonine kinase activity, protein kinase activity, ATP binding; INVOLVED IN: transmembrane receptor protein tyrosine kinase signaling pathway, protein amino acid phosphorylation; LOCATED IN: plasma membrane; EXPRESSED IN: 20 plant structures; EXPRESSED DURING: 7 growth stages; CONTAINS InterPro DOMAIN/s: Protein kinase, catalytic domain (InterPro:IPR000719), Leucine-rich repeat-containing N-terminal domain, type 2 (InterPro:IPR013210), Leucine-rich repeat (InterPro:IPR001611), Serine-threonine/tyrosine-protein kinase (InterPro:IPR001245), Protein kinase-like domain (InterPro:IPR011009); BEST Arabidopsis thaliana protein match is: Leucine-rich repeat protein kinase family protein (TAIR:AT5G51560.1)

AT4G32920	*0,906*		glycine-rich protein; FUNCTIONS IN: molecular_function unknown; INVOLVED IN: biological_process unknown; LOCATED IN: vacuole; EXPRESSED IN: 21 plant structures; EXPRESSED DURING: 11 growth stages; BEST Arabidopsis thaliana protein match is: unknown protein (TAIR:AT5G11700.1)
AT5G04680	*0,906*		Ankyrin repeat family protein; CONTAINS InterPro DOMAIN/s: Ankyrin repeat-containing domain (InterPro:IPR020683), Ankyrin repeat (InterPro:IPR002110); BEST Arabidopsis thaliana protein match is: Ankyrin repeat family protein (TAIR:AT5G04700.1)
AT1G76740	*0,905*		unknown protein; BEST Arabidopsis thaliana protein match is: unknown protein (TAIR:AT1G76840.1)
AT4G19450	*0,905*		Major facilitator superfamily protein; FUNCTIONS IN: molecular_function unknown; INVOLVED IN: biological_process unknown; LOCATED IN: endomembrane system; EXPRESSED IN: 22 plant structures; EXPRESSED DURING: 13 growth stages; CONTAINS InterPro DOMAIN/s: Nodulin-like (InterPro:IPR010658), Major facilitator superfamily, general substrate transporter (InterPro:IPR016196); BEST Arabidopsis thaliana protein match is: Major facilitator superfamily protein (TAIR:AT5G45275.1)
AT5G05180	*0,903*		unknown protein; BEST Arabidopsis thaliana protein match is: unknown protein (TAIR:AT3G10880.1)
AT5G57350	*0,903*	H(+)-ATPASE 3 (HA3)	member of Plasma membrane H+-ATPase family
AT5G36000	*0,902*		BEST Arabidopsis thaliana protein match is: reduced male fertility (TAIR:AT3G61730.1)
AT4G10630	*0,901*		Glutaredoxin family protein; FUNCTIONS IN: electron carrier activity, protein disulfide oxidoreductase activity; INVOLVED IN: N-terminal protein myristoylation, cell redox homeostasis; LOCATED IN: cellular_component unknown; CONTAINS InterPro DOMAIN/s: Thioredoxin fold (InterPro:IPR012335), Glutaredoxin (InterPro:IPR002109), Thioredoxin-like fold (InterPro:IPR012336); BEST Arabidopsis thaliana protein match is: Glutaredoxin family protein (TAIR:AT1G32760.1)
AT1G67820	*0,9*		Protein phosphatase 2C family protein; FUNCTIONS IN: protein serine/threonine phosphatase activity, catalytic activity; INVOLVED IN: protein amino acid dephosphorylation; LOCATED IN: protein serine/threonine phosphatase complex; EXPRESSED IN: 19 plant structures; EXPRESSED DURING: 7 growth stages; CONTAINS InterPro DOMAIN/s: Protein phosphatase 2C, manganese/magnesium aspartate binding site (InterPro:IPR000222), Protein phosphatase 2C-related (InterPro:IPR001932), Protein phosphatase 2C, N-terminal (InterPro:IPR014045); BEST Arabidopsis thaliana protein match is: Protein phosphatase 2C family protein (TAIR:AT2G30020.1)
AT5G63350	*0,9*		unknown protein; BEST Arabidopsis thaliana protein match is: unknown protein (TAIR:AT3G48510.1)
AT2G37040	*0,899*	PHE AMMONIA LYASE 1 (pal1)	Encodes PAL1, a phenylalanine ammonia-lyase. Arabidopsis has four PALs: AT2G37040 (PAL1), AT3G53260 (PAL2), AT5G04230 (PAL3) and AT3G10340 (PAL4).
AT1G75060	*0,898*		unknown protein; BEST Arabidopsis thaliana protein match is: unknown protein (TAIR:AT1G19330.2)
AT2G36870	*0,898*	XYLOGLUCAN ENDOTRANSGLUCOSYLASE/HYDROLASE 32 (XTH32)	Encodes a xyloglucan endotransglycosylase/hydrolase. Protein sequence and phylogenetic analysis indicates that this enzyme resides in Group III-A of the XTH family, with high similarity to Tropaeolum majus (nasturtium) xyloglucanase 1 (TmNXG1). By sequence similarity to XTH31 (At3g44990) and in vivo analysis, likely to exhibit predominant xyloglucan endo-hydrolase activity (EC 3.2.1.151) with only limited potential to act as a xyloglucan endo-transglycosylase (EC 2.4.1.207).
AT2G45440	*0,898*	DIHYDRODIPICOLINATE SYNTHASE 2 (DHDPS2)	Encodes a protein that likely has dihydropicolinate synthase activity based on its mutant phenotype of decreased lysine levels and increased aspartate levels. The mutant also has increased levels of threonine. The enzyme is predicted to localize to the chloroplast.
AT1G66970	*0,897*	SHV3-LIKE 2 (SVL2)	Encodes a member of the glycerophosphodiester phosphodiesterase like (GDPD-like) family.
AT1G75860	*0,897*		unknown protein; BEST Arabidopsis thaliana protein match is: unknown protein (TAIR:AT1G20100.1)
AT4G05180	*0,897*	PHOTOSYSTEM II SUBUNIT Q-2 (PSBQ-2)	Encodes the PsbQ subunit of the oxygen evolving complex of photosystem II.
AT5G65750	*0,897*		2-oxoglutarate dehydrogenase, E1 component; FUNCTIONS IN: oxoglutarate dehydrogenase (succinyl-transferring) activity, cobalt ion binding, zinc ion binding; INVOLVED IN: response to cadmium ion; LOCATED IN: mitochondrion; EXPRESSED IN: 23 plant structures; EXPRESSED DURING: 11 growth stages; CONTAINS InterPro DOMAIN/s: 2-oxoglutarate dehydrogenase, E1 component (InterPro:IPR011603), Dehydrogenase, E1 component (InterPro:IPR001017), Transketolase-like, pyrimidine-binding domain (InterPro:IPR005475); BEST Arabidopsis thaliana protein match is: 2-oxoglutarate dehydrogenase, E1 component (TAIR:AT3G55410.1)

AT1G20450	*0,896*	EARLY RESPONSIVE TO DEHYDRATION 10 (ERD10)	Encodes a gene induced by low temperature and dehydration. Inhibits e.coli growth while overexpressed. Belongs to the dehydrin protein family, which contains highly conserved stretches of 7-17 residues that are repetitively scattered in their sequences, the K-, S-, Y- and lysine rich segments. LTI29 and LTI30 double overexpressors confer cold tolerance. Localized to membranes and cytoplasm.
AT1G46480	*0,896*	WUSCHEL RELATED HOMEOBOX 4 (WOX4)	Encodes WOX4, a WUSCHEL-related homeobox gene family member with 65 amino acids in its homeodomain. Proteins in this family contain a sequence of eight residues (TLPLFPMH) downstream of the homeodomain called the WUS box. This protein also contains an acidic domain approximately 10 residues upstream of the WUS box. Part of the TDIF-TDR-WOX4 signaling pathway that plays a crucial role in the maintenance of the vascular meristem organization during secondary growth. WOX4 and WOX14 act downstream of the PXY receptor kinase to regulate plant vascular proliferation independently of any role in vascular organisation.
AT1G68890	*0,896*	PHYLLO (PHYLLO)	Homologous to the four eubacterial men genes involved in menanoquinone biosynthesis. Studies of mutants defective in this gene demonstrated its involvement in phylloquinone biosynthesis in Arabidopsis. The mRNA is cell-to-cell mobile.
AT1G76720	*0,896*		eukaryotic translation initiation factor 2 (eIF-2) family protein; FUNCTIONS IN: GTP binding, GTPase activity, translation initiation factor activity; INVOLVED IN: translational initiation; LOCATED IN: cellular_component unknown; EXPRESSED IN: guard cell, cultured cell; CONTAINS InterPro DOMAIN/s: Small GTP-binding protein (InterPro:IPR005225), Translation elongation factor EFTu/EF1A, domain 2 (InterPro:IPR004161), Translation initiation factor 2 related (InterPro:IPR015760), Protein synthesis factor, GTP-binding (InterPro:IPR000795), Translation elongation/initiation factor/Ribosomal, beta-barrel (InterPro:IPR009000); BEST Arabidopsis thaliana protein match is: eukaryotic translation initiation factor 2 (eIF-2) family protein (TAIR:AT1G76820.1)
AT3G55950	*0,896*	CRINKLY4 RELATED 3 (CCR3)	CRINKLY4 related 3 (CCR3); FUNCTIONS IN: kinase activity; INVOLVED IN: protein amino acid phosphorylation; LOCATED IN: endomembrane system; EXPRESSED IN: 23 plant structures; EXPRESSED DURING: 10 growth stages; CONTAINS InterPro DOMAIN/s: Protein kinase, ATP binding site (InterPro:IPR017441), Regulator of chromosome condensation/beta-lactamase-inhibitor protein II (InterPro:IPR009091), Protein kinase, catalytic domain (InterPro:IPR000719), Serine/threonine-protein kinase-like domain (InterPro:IPR017442), Protein kinase-like domain (InterPro:IPR011009), Serine/threonine-protein kinase, active site (InterPro:IPR008271); BEST Arabidopsis thaliana protein match is: CRINKLY4 related 4 (TAIR:AT5G47850.1)
AT5G12330	*0,896*	LATERAL ROOT PRIMORDIUM 1 (LRP1)	A member of SHI gene family. Arabidopsis thaliana has ten members that encode proteins with a RING finger-like zinc finger motif. Despite being highly divergent in sequence, many of the SHI-related genes are partially redundant in function and synergistically promote gynoecium, stamen and leaf development in Arabidopsis. Expressed in lateral root primordia and induced by auxin. SWP1 is involved in the repression of LRP1 via histone deacetylation.
AT5G38900	*0,896*		Thioredoxin superfamily protein; FUNCTIONS IN: protein disulfide oxidoreductase activity; INVOLVED IN: defense response to fungus, incompatible interaction; LOCATED IN: cellular_component unknown; EXPRESSED IN: 16 plant structures; EXPRESSED DURING: 10 growth stages; CONTAINS InterPro DOMAIN/s: DSBA oxidoreductase (InterPro:IPR001853), Thioredoxin-like fold (InterPro:IPR012336)
AT1G50950	*0,895*		INVOLVED IN: cell redox homeostasis; EXPRESSED IN: 20 plant structures; EXPRESSED DURING: 11 growth stages; CONTAINS InterPro DOMAIN/s: Thioredoxin fold (InterPro:IPR012335), Thioredoxin-like (InterPro:IPR017936), Thioredoxin-like fold (InterPro:IPR012336), Thioredoxin domain (InterPro:IPR013766), Protein of unknown function DUF1692 (InterPro:IPR012936); BEST Arabidopsis thaliana protein match is: PDI-like 5-3 (TAIR:AT3G20560.1)
AT1G78280	*0,895*		transferases, transferring glycosyl groups; CONTAINS InterPro DOMAIN/s: Transcription factor jumonji/aspartyl beta-hydroxylase (InterPro:IPR003347), F-box domain, cyclin-like (InterPro:IPR001810), F-box domain, Skp2-like (InterPro:IPR022364), Transcription factor jumonji (InterPro:IPR013129), Aminoglycoside phosphotransferase (InterPro:IPR002575), Protein kinase-like domain (InterPro:IPR011009); BEST Arabidopsis thaliana protein match is: unknown protein (TAIR:AT5G06550.1)
AT5G05940	*0,894*	ROP (RHO OF PLANTS) GUANINE NUCLEOTIDE EXCHANGE FACTOR 5 (ROPGEF5)	Encodes a member of KPP-like gene family, homolog of KPP (kinase partner protein) gene in tomato. Also a member of the RopGEF (guanine nucleotide exchange factor) family, containing the novel PRONE domain (plant-specific Rop nucleotide exchanger), which is exclusively active towards members of the Rop subfamily.

AT3G25020	*0,893*	RECEPTOR LIKE PROTEIN 42 (RLP42)	receptor like protein 42 (RLP42); INVOLVED IN: signal transduction, defense response; LOCATED IN: endomembrane system; EXPRESSED IN: 13 plant structures; EXPRESSED DURING: 11 growth stages; CONTAINS InterPro DOMAIN/s: Leucine-rich repeat (InterPro:IPR001611); BEST Arabidopsis thaliana protein match is: receptor like protein 40 (TAIR:AT3G24982.1)
AT1G69610	*0,892*		FUNCTIONS IN: structural constituent of ribosome; INVOLVED IN: N-terminal protein myristoylation, translation; LOCATED IN: ribosome, intracellular; EXPRESSED IN: 20 plant structures; EXPRESSED DURING: 12 growth stages; CONTAINS InterPro DOMAIN/s: Ribosomal protein L34e (InterPro:IPR008195), Protein of unknown function DUF1666 (InterPro:IPR012870); BEST Arabidopsis thaliana protein match is: Protein of unknown function (DUF1666) (TAIR:AT5G39785.1)
AT5G25900	*0,892*	GA REQUIRING 3 (GA3)	Encodes a member of the CYP701A cytochrome p450 family that is involved in later steps of the gibberellin biosynthetic pathway.
AT1G33590	*0,891*		Leucine-rich repeat (LRR) family protein; INVOLVED IN: signal transduction, response to karrikin, defense response; LOCATED IN: cell wall, chloroplast, plant-type cell wall; EXPRESSED IN: 22 plant structures; EXPRESSED DURING: 14 growth stages; CONTAINS InterPro DOMAIN/s: Leucine-rich repeat, typical subtype (InterPro:IPR003591), Leucine-rich repeat-containing N-terminal domain, type 2 (InterPro:IPR013210), Leucine-rich repeat (InterPro:IPR001611); BEST Arabidopsis thaliana protein match is: Leucine-rich repeat (LRR) family protein (TAIR:AT1G33600.1)
AT1G71480	*0,891*		Nuclear transport factor 2 (NTF2) family protein; FUNCTIONS IN: protein transporter activity; INVOLVED IN: transport, protein import into nucleus; LOCATED IN: chloroplast thylakoid membrane, intracellular, nucleus, chloroplast; EXPRESSED IN: 22 plant structures; EXPRESSED DURING: 13 growth stages; CONTAINS InterPro DOMAIN/s: Nuclear transport factor 2 (InterPro:IPR002075); BEST Arabidopsis thaliana protein match is: Nuclear transport factor 2 (NTF2) family protein (TAIR:AT5G41470.1)
AT2G28140	*0,891*		Protein of unknown function (DUF1635); CONTAINS InterPro DOMAIN/s: Protein of unknown function DUF1635 (InterPro:IPR012862); BEST Arabidopsis thaliana protein match is: Protein of unknown function (DUF1635) (TAIR:AT5G22930.1)
AT5G15210	*0,891*	HOMEOBOX PROTEIN 30 (HB30)	Encodes ZFHD3, a member of the zinc finger homeodomain transcriptional factor family.
AT5G60100	*0,891*	PSEUDO-RESPONSE REGULATOR 3 (PRR3)	Encodes pseudo-response regulator 3 (APRR3/PRR3). PRR3 transcript levels vary in a circadian pattern with peak expression at dusk under long and short day conditions. PRR3 affects the period of the circadian clock and seedlings with reduced levels of PRR3 have shorter periods, based on transcriptional assays of clock-regulated genes. PRR3 is expressed in the vasculature of cotyledons and leaves where it may help stabilize the TOC1 protein by preventing interactions between TOC1 and the F-box protein ZTL.
AT1G64620	*0,89*	(DOF1.8)	Dof-type zinc finger DNA-binding family protein; CONTAINS InterPro DOMAIN/s: Zinc finger, Dof-type (InterPro:IPR003851); BEST Arabidopsis thaliana protein match is: Dof-type zinc finger DNA-binding family protein (TAIR:AT4G24060.1)
AT1G04680	*0,889*		Pectin lyase-like superfamily protein; FUNCTIONS IN: lyase activity, pectate lyase activity; INVOLVED IN: biological_process unknown; LOCATED IN: membrane; EXPRESSED IN: 23 plant structures; EXPRESSED DURING: 13 growth stages; CONTAINS InterPro DOMAIN/s: Pectin lyase fold/virulence factor (InterPro:IPR011050), AmbAllergen (InterPro:IPR018082), Pectate lyase/Amb allergen (InterPro:IPR002022), Pectin lyase fold (InterPro:IPR012334); BEST Arabidopsis thaliana protein match is: Pectin lyase-like superfamily protein (TAIR:AT4G13710.1)
AT2G16270	*0,888*		unknown protein; FUNCTIONS IN: molecular_function unknown; INVOLVED IN: biological_process unknown; LOCATED IN: chloroplast; EXPRESSED IN: 16 plant structures; EXPRESSED DURING: 9 growth stages; BEST Arabidopsis thaliana protein match is: unknown protein (TAIR:AT1G16630.1)
AT3G14720	*0,887*	MAP KINASE 19 (MPK19)	member of MAP Kinase The mRNA is cell-to-cell mobile.
AT4G13250	*0,887*	NON-YELLOW COLORING 1 (NYC1)	Encodes a chlorophyll b reductase involved in the degradation of chlorophyll b and LHCII (light harvesting complex II).
AT1G70800	*0,886*	ENHANCED BENDING 1 (EHB1)	Encodes a novel NPH3/phototropin binding factor with a calcium binding domain that negatively affects hypocotyl bending under blue light conditions in Arabidopsis thaliana and may regulate phototropism.

AT1G78940	*0,883*		Protein kinase protein with adenine nucleotide alpha hydrolases-like domain; FUNCTIONS IN: kinase activity; INVOLVED IN: protein amino acid phosphorylation, response to stress; EXPRESSED IN: 12 plant structures; EXPRESSED DURING: 8 growth stages; CONTAINS InterPro DOMAIN/s: UspA (InterPro:IPR006016), Serine/threonine-protein kinase domain (InterPro:IPR002290), Serine-threonine/tyrosine-protein kinase (InterPro:IPR001245), Serine/threonine-protein kinase, active site (InterPro:IPR008271), Protein kinase-like domain (InterPro:IPR011009), Rossmann-like alpha/beta/alpha sandwich fold (InterPro:IPR014729), Protein kinase, catalytic domain (InterPro:IPR000719), Tyrosine-protein kinase, catalytic domain (InterPro:IPR020635); BEST Arabidopsis thaliana protein match is: Protein kinase protein with adenine nucleotide alpha hydrolases-like domain (TAIR:AT1G16760.1).
AT2G05940	*0,882*	RPM1-INDUCED PROTEIN KINASE (RIPK)	Encodes a receptor-like cytoplasmic kinase that phosphorylates the host target RIN4, leading to the activation of a plant innate immune receptor RPM1.
AT2G16660	*0,882*		Major facilitator superfamily protein; INVOLVED IN: response to karrikin; LOCATED IN: endomembrane system; EXPRESSED IN: 23 plant structures; EXPRESSED DURING: 13 growth stages; CONTAINS InterPro DOMAIN/s: Nodulin-like (InterPro:IPR010658), Major facilitator superfamily, general substrate transporter (InterPro:IPR016196); BEST Arabidopsis thaliana protein match is: Major facilitator superfamily protein (TAIR:AT4G34950.1)
AT4G00340	*0,879*	RECEPTOR-LIKE PROTEIN KINASE 4 (RLK4)	Encodes a receptor-like protein kinase that is expressed in roots.
AT5G03780	*0,879*	TRF-LIKE 10 (TRFL10)	Encodes a protein whose sequence is similar to human telomere proteins. This belongs to TRFL family 2, which do not show DNA binding in vitro.
AT2G47230	*0,877*	DOMAIN OF UNKNOWN FUNCTION 724 6 (DUF6)	Member of the plant-specific DUF724 protein family. Arabidopsis has 10 DUF724 proteins.
AT2G35350	*0,876*	POLTERGEIST LIKE 1 (PLL1)	Encodes a protein most similar to the POLTERGEIST locus. Double mutant analysis of loss of function alleles indicate PLL1 functions redundantly with POL to regulate meristem size and pedicel length. Acts in a dose dependent manner with POL to suppress the clv1, clv2 and clv3 phenotypes.
AT1G51680	*0,875*	4-COUMARATE:COA LIGASE 1 (4CL1)	encodes an isoform of 4-coumarate:CoA ligase (4CL), which is involved in the last step of the general phenylpropanoid pathway. In addition to 4-coumarate, it also converts ferulate. The catalytic efficiency was in the following (descending) order: p-coumaric acid, ferulic acid, caffeic acid and 5-OH-ferulic acid. At4CL1 was unable to use sinapic acid as substrate.
AT1G52450	*0,875*		Ubiquitin carboxyl-terminal hydrolase-related protein; FUNCTIONS IN: ubiquitin thiolesterase activity; INVOLVED IN: ubiquitin-dependent protein catabolic process; LOCATED IN: cellular_component unknown; CONTAINS InterPro DOMAIN/s: Protein of unknown function DUF627 (InterPro:IPR006866), Peptidase C19, ubiquitin carboxyl-terminal hydrolase 2 (InterPro:IPR001394), Protein of unknown function DUF629 (InterPro:IPR006865); BEST Arabidopsis thaliana protein match is: Ubiquitin carboxyl-terminal hydrolase-related protein (TAIR:AT1G52430.1)
AT1G60950	*0,875*	(FED A)	encodes a major leaf ferredoxin
AT1G69480	*0,875*		EXS (ERD1/XPR1/SYG1) family protein; FUNCTIONS IN: molecular_function unknown; INVOLVED IN: biological_process unknown; LOCATED IN: integral to membrane, plasma membrane; EXPRESSED IN: stem, sepal, stamen, seed; EXPRESSED DURING: 4 anthesis, petal differentiation and expansion stage, E expanded cotyledon stage; CONTAINS InterPro DOMAIN/s: EXS, C-terminal (InterPro:IPR004342), SPX, N-terminal (InterPro:IPR004331); BEST Arabidopsis thaliana protein match is: EXS (ERD1/XPR1/SYG1) family protein (TAIR:AT1G14040.1)
AT4G36270	*0,875*		ATP binding; BEST Arabidopsis thaliana protein match is: Histidine kinase-, DNA gyrase B-, and HSP90-like ATPase family protein (TAIR:AT4G36280.1)
AT5G52410	*0,875*		INVOLVED IN: biological_process unknown; LOCATED IN: chloroplast; EXPRESSED IN: 13 plant structures; EXPRESSED DURING: 6 growth stages; CONTAINS InterPro DOMAIN/s: S-layer homology domain (InterPro:IPR001119); BEST Arabidopsis thaliana protein match is: unknown protein (TAIR:AT5G23890.1)

AT1G20510	*0,872*	OPC-8:0 COA LIGASE1 (OPCL1)	OPC-8:0 CoA ligase1 (OPCL1); FUNCTIONS IN: 4-coumarate-CoA ligase activity; INVOLVED IN: phenylpropanoid metabolic process, jasmonic acid biosynthetic process, response to wounding; LOCATED IN: peroxisome; EXPRESSED IN: 22 plant structures; EXPRESSED DURING: 13 growth stages; CONTAINS InterPro DOMAIN/s: AMP-binding, conserved site (InterPro:IPR020845), AMP-dependent synthetase/ligase (InterPro:IPR000873); BEST Arabidopsis thaliana protein match is: AMP-dependent synthetase and ligase family protein (TAIR:AT1G20500.1)
AT1G72540	*0,872*		Protein kinase superfamily protein; FUNCTIONS IN: protein serine/threonine kinase activity, protein kinase activity, kinase activity, ATP binding; INVOLVED IN: protein amino acid phosphorylation; LOCATED IN: chloroplast; EXPRESSED IN: stem, stamen; EXPRESSED DURING: 4 anthesis; CONTAINS InterPro DOMAIN/s: Protein kinase, ATP binding site (InterPro:IPR017441), Protein kinase, catalytic domain (InterPro:IPR000719), Serine/threonine-protein kinase-like domain (InterPro:IPR017442), Protein kinase-like domain (InterPro:IPR011009), Serine/threonine-protein kinase, active site (InterPro:IPR008271); BEST Arabidopsis thaliana protein match is: Protein kinase superfamily protein (TAIR:AT2G05940.1)
AT3G11700	*0,872*	FASCICLIN-LIKE ARABINOGALACTAN PROTEIN 18 PRECURSOR (FLA18)	FASCICLIN-like arabinogalactan protein 18 precursor (FLA18); FUNCTIONS IN: molecular_function unknown; INVOLVED IN: response to cyclopentenone, cell adhesion; LOCATED IN: vacuole; EXPRESSED IN: 23 plant structures; EXPRESSED DURING: 15 growth stages; CONTAINS InterPro DOMAIN/s: FAS1 domain (InterPro:IPR000782); BEST Arabidopsis thaliana protein match is: FASCICLIN-like arabinogalactan protein 17 precursor (TAIR:AT5G06390.1)
AT2G38010	*0,871*	NEUTRAL CERAMIDASE 2 (ATNCER2)	Neutral/alkaline non-lysosomal ceramidase; FUNCTIONS IN: ceramidase activity; LOCATED IN: endomembrane system; EXPRESSED IN: 22 plant structures; EXPRESSED DURING: 13 growth stages; CONTAINS InterPro DOMAIN/s: Neutral/alkaline nonlysosomal ceramidase (InterPro:IPR006823); BEST Arabidopsis thaliana protein match is: Neutral/alkaline non-lysosomal ceramidase (TAIR:AT1G07380.1)
AT5G04480	*0,87*	BURSTING POLLEN (BUP)	Encodes a protein with sequence similarity to glycosyltransferases that is localized to the golgi apparatus and is involved in pollen tube development.
AT2G02230	*0,869*	PHLOEM PROTEIN 2-B1 (PP2-B1)	phloem protein 2-B1 (PP2-B1); CONTAINS InterPro DOMAIN/s: F-box domain, cyclin-like (InterPro:IPR001810), F-box domain, Skp2-like (InterPro:IPR022364); BEST Arabidopsis thaliana protein match is: F-box family protein (TAIR:AT2G02240.1)
AT3G18110	*0,869*	EMBRYO DEFECTIVE 1270 (EMB1270)	embryo defective 1270 (EMB1270); INVOLVED IN: embryo development ending in seed dormancy; LOCATED IN: chloroplast; EXPRESSED IN: 21 plant structures; EXPRESSED DURING: 13 growth stages; CONTAINS InterPro DOMAIN/s: Pentatricopeptide repeat (InterPro:IPR002885); BEST Arabidopsis thaliana protein match is: proton gradient regulation 3 (TAIR:AT4G31850.1)
AT3G63180	*0,868*	TIC-LIKE (TKL)	TIC-like (TKL); BEST Arabidopsis thaliana protein match is: time for coffee (TAIR:AT3G22380.2)
AT1G61260	*0,866*		Protein of unknown function (DUF761); FUNCTIONS IN: molecular_function unknown; INVOLVED IN: biological_process unknown; LOCATED IN: endomembrane system; EXPRESSED IN: 12 plant structures; EXPRESSED DURING: 7 growth stages; CONTAINS InterPro DOMAIN/s: Protein of unknown function DUF761, plant (InterPro:IPR008480); BEST Arabidopsis thaliana protein match is: Protein of unknown function (DUF761) (TAIR:AT1G11220.1)
AT5G65590	*0,866*	STOMATAL CARPENTER 1 (SCAP1)	Encodes a plant-specific Dof-type transcription factor expressed in maturing guard cells, but not in guard mother cells. It regulates essential processes of stomatal guard cell maturation and functions as a key transcription factor regulating the final stages of guard cell differentiation.
AT1G06490	*0,865*	CALLOSE SYNTHASE 7 (CalS7)	Encodes Callose Synthase 7 (CalS7), a phloem-specific callose synthase responsible for callose deposition in developing sieve elements during phloem formation and in mature phloem induced by wounding.
AT4G14150	*0,864*	PHRAGMOPLAST-ASSOCIATED KINESIN-RELATED PROTEIN 1 (PAKRP1)	Microtubule motor kinesin PAKRP1/Kinesin-12A. Together with PAKRP1L/Kinesin-12B, serve as linkers of the plus ends of antiparallel microtubules in the phragmoplast.
AT5G01030	*0,864*		Protein of unknown function (DUF3527); FUNCTIONS IN: molecular_function unknown; INVOLVED IN: biological_process unknown; LOCATED IN: cellular_component unknown; EXPRESSED IN: 24 plant structures; EXPRESSED DURING: 13 growth stages; CONTAINS InterPro DOMAIN/s: Protein of unknown function DUF3527 (InterPro:IPR021916); BEST Arabidopsis thaliana protein match is: Protein of unknown function (DUF3527) (TAIR:AT2G37930.1)
AT2G05310	*0,863*		unknown protein; FUNCTIONS IN: molecular_function unknown; INVOLVED IN: biological_process unknown; LOCATED IN: chloroplast; BEST Arabidopsis thaliana protein match is: unknown protein (TAIR:AT4G13500.1)

AT5G06670	*0,863*	KINESIN 7.5 (KIN7.5)	P-loop containing nucleoside triphosphate hydrolases superfamily protein; FUNCTIONS IN: microtubule motor activity, ATP binding; INVOLVED IN: microtubule-based movement; LOCATED IN: chloroplast; EXPRESSED IN: 14 plant structures; EXPRESSED DURING: 7 growth stages; CONTAINS InterPro DOMAIN/s: Kinesin, motor region, conserved site (InterPro:IPR019821), Kinesin, motor domain (InterPro:IPR001752); BEST Arabidopsis thaliana protein match is: P-loop containing nucleoside triphosphate hydrolases superfamily protein (TAIR:AT3G12020.1)
AT3G63110	*0,862*	ISOPENTENYLTRANSFERASE 3 (IPT3)	Encodes cytokinin synthase involved in cytokinin biosynthesis. IPT3 subcellular localization is modulated by farnesylation- when farnesylated it is localized to the nucleus, otherwise to the chloroplast.
AT5G59750	*0,862*	HOMOLOG OF RIBA 3 (RIBA3)	DHBP synthase RibB-like alpha/beta domain;GTP cyclohydrolase II; FUNCTIONS IN: 3,4-dihydroxy-2-butanone-4-phosphate synthase activity, GTP cyclohydrolase II activity; INVOLVED IN: riboflavin biosynthetic process; EXPRESSED IN: 23 plant structures; EXPRESSED DURING: 13 growth stages; CONTAINS InterPro DOMAIN/s: GTP cyclohydrolase II (InterPro:IPR000926), DHBP synthase RibB (InterPro:IPR000422), DHBP synthase RibB-like alpha/beta domain (InterPro:IPR017945); BEST Arabidopsis thaliana protein match is: GTP cyclohydrolase II (TAIR:AT5G64300.1).
AT1G04710	*0,861*	PEROXISOMAL 3-KETOACYL-COA THIOLASE 4 (PKT4)	EC2.3.1.16 thiolase. Its transcript levels change after inducing MUTE expression in a mute background.
AT1G05560	*0,861*	UDP-GLUCOSYLTRANSFERASE 75B1 (UGT75B1)	A UDP-glucose transferase localized in the phragmoplast. It has been co-purified with the callose synthase complex and may transfer UDP-glucose from sucrose synthase to the callose synthase and thus help form a substrate channel for the synthesis of callose at the forming cell plate. Induced by salicylic acid. Independent of NPR1 for their induction by salicylic acid. UGT1 encodes a protein with glucosyltransferase activity with high sequence homology to UGT2 (AT1G05530). It belongs to an UGT subfamily that binds UDP-glucose but not UDP-glucuronate, UDP-galactose, or UDP-rhamnose as the glycosyl donor. UGT1 was shown to be able to use abscisic acid as glycosylation substrate in the presence of UDP-glucose. UGT1/UGT75B1 catalyzes the formation of the p-aminobenzoate-glucose ester in vitro and in vivo. It appears to be the enzyme predominantly responsible for pABA-Glc formation in Arabidopsis based on assays in leaves, flowers, and siliques.
AT2G34460	*0,86*		NAD(P)-binding Rossmann-fold superfamily protein; FUNCTIONS IN: binding, catalytic activity; INVOLVED IN: metabolic process; LOCATED IN: chloroplast thylakoid membrane, chloroplast, plastoglobule, chloroplast envelope; EXPRESSED IN: 21 plant structures; EXPRESSED DURING: 13 growth stages; CONTAINS InterPro DOMAIN/s: NAD(P)-binding domain (InterPro:IPR016040), NmrA-like (InterPro:IPR008030); BEST Arabidopsis thaliana protein match is: NAD(P)-binding Rossmann-fold superfamily protein (TAIR:AT3G18890.1)
AT3G61820	*0,86*		Eukaryotic aspartyl protease family protein; FUNCTIONS IN: aspartic-type endopeptidase activity; INVOLVED IN: proteolysis; LOCATED IN: plant-type cell wall; EXPRESSED IN: 22 plant structures; EXPRESSED DURING: 13 growth stages; CONTAINS InterPro DOMAIN/s: Peptidase aspartic (InterPro:IPR021109), Peptidase aspartic, catalytic (InterPro:IPR009007), Peptidase A1 (InterPro:IPR001461); BEST Arabidopsis thaliana protein match is: Eukaryotic aspartyl protease family protein (TAIR:AT1G01300.1)
AT3G23790	*0,859*	ACYL ACTIVATING ENZYME 16 (AAE16)	acyl activating enzyme 16 (AAE16); FUNCTIONS IN: catalytic activity; INVOLVED IN: metabolic process; LOCATED IN: chloroplast envelope; EXPRESSED IN: 21 plant structures; EXPRESSED DURING: 9 growth stages; CONTAINS InterPro DOMAIN/s: AMP-binding (InterPro:IPR020459), AMP-binding, conserved site (InterPro:IPR020845), AMP-dependent synthetase/ligase (InterPro:IPR000873); BEST Arabidopsis thaliana protein match is: acyl-activating enzyme 15 (TAIR:AT4G14070.1)
AT2G45680	*0,858*	TCP DOMAIN PROTEIN 9 (TCP9)	TCP family transcription factor ; CONTAINS InterPro DOMAIN/s: Transcription factor, TCP (InterPro:IPR005333), Transcription factor TCP subgroup (InterPro:IPR017887); BEST Arabidopsis thaliana protein match is: TCP family transcription factor (TAIR:AT5G51910.2)
AT1G29930	*0,857*	CHLOROPHYLL A/B BINDING PROTEIN 1 (CAB1)	Subunit of light-harvesting complex II (LHCII),which absorbs light and transfers energy to the photosynthetic reaction center. The mRNA is cell-to-cell mobile.
AT1G77630	*0,857*	LYSIN-MOTIF (LYSM) DOMAIN PROTEIN 3 (LYM3)	Encodes a lysin-motif protein mediating bacterial peptidoglycan sensing and immunity to bacterial infection. Induction of chitin-responsive genes by chitin treatment is not blocked in the mutant. It contains a C-terminal GPI anchor signal and is an ortholog of OsLYP4 and OsLYP6.

AT2G44190	*0,857*	ENDOSPERM DEFECTIVE 1 (EDE1)	Encodes a novel microtubule binding protein that preferentially associates with nuclear microtubules during mitosis and is essential during the mitotic and cytokinetic stages that generate the endosperm and embryo.
AT5G02830	*0,857*		Tetratricopeptide repeat (TPR)-like superfamily protein; INVOLVED IN: biological_process unknown; LOCATED IN: chloroplast; EXPRESSED IN: 22 plant structures; EXPRESSED DURING: 13 growth stages; CONTAINS InterPro DOMAIN/s: Pentatricopeptide repeat (InterPro:IPR002885); BEST Arabidopsis thaliana protein match is: Pentatricopeptide repeat (PPR-like) superfamily protein (TAIR:AT1G09900.1)
AT1G54780	*0,856*	THYLAKOID LUMEN PROTEIN 18.3 (TLP18.3)	Encodes a thylakoid lumen protein regulating photosystem II repair cycle. Has acid phosphatase activity. The mRNA is cell-to-cell mobile.
AT3G16140	*0,855*	PHOTOSYSTEM I SUBUNIT H-1 (PSAH-1)	Encodes subunit H of photosystem I reaction center subunit VI.
AT3G46640	*0,855*	PHYTOCLOCK 1 (PCL1)	Encodes a myb family transcription factor with a single Myb DNA-binding domain (type SHAQKYF) that is unique to plants and is essential for circadian rhythms, specifically for transcriptional regulation within the circadian clock. LUX is required for normal rhythmic expression of multiple clock outputs in both constant light and darkness. It is coregulated with TOC1 and seems to be repressed by CCA1 and LHY by direct binding of these proteins to the evening element in the LUX promoter. The mRNA is cell-to-cell mobile.
AT5G52520	*0,855*	OVULE ABORTION 6 (OVA6)	Encodes a chloroplast and mitochondria localized prolyl-tRNA synthetase.
AT5G64560	*0,855*	MAGNESIUM TRANSPORTER 9 (MGT9)	Transmembrane magnesium transporter that is located in plasma membrane of microspores to take up Mg from the locule. One of 9 family members.
AT1G06460	*0,854*	ALPHA-CRYSTALLIN DOMAIN 32.1 (ACD32.1)	ACD32.1 encodes an alpha-crystallin domain containing protein with homology to small heat shock proteins.
AT1G09960	*0,854*	SUCROSE TRANSPORTER 4 (SUT4)	low affinity (10mM) sucrose transporter in sieve elements (phloem)
AT3G49780	*0,854*	PHYTOSULFOKINE 4 PRECURSOR (PSK4)	Phytosulfokine 3 precursor, coding for a unique plant peptide growth factor. Plants overexpressing this gene (under a 35S promoter), develop normal cotyledons and hypocotyls but their growth, in particular that of their roots, was faster than that of wildtype.
AT5G23400	*0,854*		Leucine-rich repeat (LRR) family protein; INVOLVED IN: signal transduction, defense response; LOCATED IN: cell wall; EXPRESSED IN: 17 plant structures; EXPRESSED DURING: 10 growth stages; CONTAINS InterPro DOMAIN/s: Leucine-rich repeat-containing N-terminal domain, type 2 (InterPro:IPR013210), Leucine-rich repeat (InterPro:IPR001611); BEST Arabidopsis thaliana protein match is: Leucine-rich repeat (LRR) family protein (TAIR:AT2G26380.1)
AT2G36570	*0,852*	PXY/TDR-CORRELATED 1 (PXC1)	Leucine-rich repeat protein kinase family protein; FUNCTIONS IN: protein serine/threonine kinase activity, kinase activity, ATP binding; INVOLVED IN: transmembrane receptor protein tyrosine kinase signaling pathway, protein amino acid phosphorylation; LOCATED IN: plasma membrane; EXPRESSED IN: 21 plant structures; EXPRESSED DURING: 13 growth stages; CONTAINS InterPro DOMAIN/s: Protein kinase, catalytic domain (InterPro:IPR000719), Serine/threonine-protein kinase-like domain (InterPro:IPR017442), Protein kinase-like domain (InterPro:IPR011009); BEST Arabidopsis thaliana protein match is: leucine-rich repeat transmembrane protein kinase family protein (TAIR:AT1G68400.1)
AT4G16140	*0,852*		proline-rich family protein
AT3G22830	*0,851*	HEAT SHOCK TRANSCRIPTION FACTOR A6B (HSFA6B)	member of Heat Stress Transcription Factor (Hsf) family
AT2G32280	*0,85*	VASCULATURE COMPLEXITY AND CONNECTIVITY (VCC)	Encodes a member of a plant-specific gene family that is required for embryo provasculature development. The gene product regulates vascular network complexity and connectivity in cotyledons.
AT2G40030	*0,85*	NUCLEAR RNA POLYMERASE D1B (NRPD1B)	Encodes the unique largest subunit of nuclear DNA-dependent RNA polymerase V; homologous to budding yeast RPB1 and the E. coli RNA polymerase beta prime subunit. Required for normal RNA-directed DNA methylation at non-CG methylation sites and transgene silencing. The nrpe1 mutant is more resistant to biotrophic pathogens and is primed to activate salicylic acid-dependent defence genes.

AT5G23120	*0,85*	HIGH CHLOROPHYLL FLUORESCENCE 136 (HCF136)	encodes a stability and/or assembly factor of photosystem II The mRNA is cell-to-cell mobile.
AT1G04620	*0,849*	7-HYDROXYMETHYL CHLOROPHYLL A (HMCHL) REDUCTASE (HCAR)	Encodes a 7-hydroxymethyl chlorophyll a reductase, an enzyme of the chlorophyll cycle that converts 7-hydroxymethyl chlorophyll a to chlorophyll a.
AT2G18230	*0,849*	PYROPHOSPHORYLASE 2 (PPa2)	Encodes a protein that might have inorganic pyrophosphatase activity.
AT5G55660	*0,849*		DEK domain-containing chromatin associated protein; CONTAINS InterPro DOMAIN/s: DEK, C-terminal (InterPro:IPR014876); BEST Arabidopsis thaliana protein match is: DEK domain-containing chromatin associated protein (TAIR:AT4G26630.2)
AT1G14270	*0,848*		CAAX amino terminal protease family protein; INVOLVED IN: proteolysis; LOCATED IN: membrane; EXPRESSED IN: 24 plant structures; EXPRESSED DURING: 15 growth stages; CONTAINS InterPro DOMAIN/s: Abortive infection protein (InterPro:IPR003675); BEST Arabidopsis thaliana protein match is: CAAX amino terminal protease family protein (TAIR:AT5G60750.1)
AT1G32060	*0,848*	PHOSPHORIBULOKINASE (PRK)	phosphoribulokinase (PRK); FUNCTIONS IN: protein binding, phosphoribulokinase activity, ATP binding; INVOLVED IN: response to cold, defense response to bacterium, biosynthetic process, peptidyl-cysteine S-nitrosylation; LOCATED IN: in 8 components; EXPRESSED IN: 24 plant structures; EXPRESSED DURING: 15 growth stages; CONTAINS InterPro DOMAIN/s: Phosphoribulokinase/uridine kinase (InterPro:IPR006083), Phosphoribulokinase (InterPro:IPR006082); BEST Arabidopsis thaliana protein match is: uridine kinase-like 5 (TAIR:AT3G27440.1)
AT3G62410	*0,848*	CP12 DOMAIN-CONTAINING PROTEIN 2 (CP12-2)	CP12-2 encodes a small peptide found in the chloroplast stroma. It belongs to the CP12 gene family thought to be involved in the formation of a supramolecular complex with glyceraldehyde-3-phosphate dehydrogenase (GAPDH) and phosphoribulokinase (PRK) embedded in the Calvin cycle. CP12-2 is coordinately regulated by light with the photosynthetic GAPDH and PRK. The annotation of this gene is based on article 32494. The mRNA is cell-to-cell mobile.
AT5G18550	*0,848*		Zinc finger C-x8-C-x5-C-x3-H type family protein; FUNCTIONS IN: zinc ion binding, nucleic acid binding; INVOLVED IN: biological_process unknown; LOCATED IN: cellular_component unknown; EXPRESSED IN: 17 plant structures; EXPRESSED DURING: 8 growth stages; CONTAINS InterPro DOMAIN/s: Zinc finger, CCCH-type (InterPro:IPR000571); BEST Arabidopsis thaliana protein match is: Zinc finger C-x8-C-x5-C-x3-H type family protein (TAIR:AT3G06410.1)
AT4G25434	*0,847*	NUDIX HYDROLASE HOMOLOG 10 (NUDT10)	nudix hydrolase homolog 10 (NUDT10); FUNCTIONS IN: ADP-ribose diphosphatase activity, NAD or NADH binding, catalytic activity; INVOLVED IN: metabolic process; LOCATED IN: cytosol; EXPRESSED IN: 6 plant structures; EXPRESSED DURING: petal differentiation and expansion stage; CONTAINS InterPro DOMAIN/s: NUDIX hydrolase domain-like (InterPro:IPR015797), NUDIX hydrolase, conserved site (InterPro:IPR020084), NUDIX hydrolase domain (InterPro:IPR000086); BEST Arabidopsis thaliana protein match is: nudix hydrolase homolog 2 (TAIR:AT5G47650.1)
AT5G64570	*0,847*	BETA-D-XYLOSIDASE 4 (XYL4)	Encodes a beta-d-xylosidase that belongs to family 3 of glycoside hydrolases.
AT2G37660	*0,846*		NAD(P)-binding Rossmann-fold superfamily protein; FUNCTIONS IN: copper ion binding; INVOLVED IN: defense response to bacterium; LOCATED IN: thylakoid, apoplast, chloroplast stroma, chloroplast; EXPRESSED IN: 24 plant structures; EXPRESSED DURING: 15 growth stages; CONTAINS InterPro DOMAIN/s: 3-beta hydroxysteroid dehydrogenase/isomerase (InterPro:IPR002225), NAD(P)-binding domain (InterPro:IPR016040); BEST Arabidopsis thaliana protein match is: NAD(P)-binding Rossmann-fold superfamily protein (TAIR:AT5G02240.1)
AT1G72210	*0,845*	(BHLH096)	basic helix-loop-helix (bHLH) DNA-binding superfamily protein; FUNCTIONS IN: DNA binding, sequence-specific DNA binding transcription factor activity; INVOLVED IN: regulation of transcription; LOCATED IN: nucleus; CONTAINS InterPro DOMAIN/s: Helix-loop-helix DNA-binding domain (InterPro:IPR001092), Helix-loop-helix DNA-binding (InterPro:IPR011598); BEST Arabidopsis thaliana protein match is: basic helix-loop-helix (bHLH) DNA-binding superfamily protein (TAIR:AT1G22490.1)

AT2G27130	*0,845*		Bifunctional inhibitor/lipid-transfer protein/seed storage 2S albumin superfamily protein; FUNCTIONS IN: lipid binding; INVOLVED IN: lipid transport; LOCATED IN: anchored to plasma membrane, anchored to membrane; EXPRESSED IN: 22 plant structures; EXPRESSED DURING: 13 growth stages; CONTAINS InterPro DOMAIN/s: Bifunctional inhibitor/plant lipid transfer protein/seed storage (InterPro:IPR016140), Plant lipid transfer protein/seed storage/trypsin-alpha amylase inhibitor (InterPro:IPR003612); BEST Arabidopsis thaliana protein match is: Bifunctional inhibitor/lipid-transfer protein/seed storage 2S albumin superfamily protein (TAIR:AT3G43720.2)
AT3G47690	*0,845*	MICROTUBULE END BINDING PROTEIN EB1A (EB1A)	encodes a homolog of animal microtubule-end-binding protein. There are two other members of this family. EB1 forms foci at regions where the minus ends of microtubules are gathered during mitosis and early cytokinesis.
AT2G35370	*0,843*	GLYCINE DECARBOXYLASE COMPLEX H (GDCH)	Encodes glycine decarboxylase complex H protein. Involved in photorespiration. The mRNA is cell-to-cell mobile.
AT5G22270	*0,842*	SALT-INDUCED AND EIN3/EIL1-DEPENDENT 1 (SIED1)	unknown protein; BEST Arabidopsis thaliana protein match is: unknown protein (TAIR:AT3G11600.1)
AT1G15820	*0,841*	LIGHT HARVESTING COMPLEX PHOTOSYSTEM II SUBUNIT 6 (LHCB6)	Lhcb6 protein (Lhcb6), light harvesting complex of photosystem II.
AT1G55210	*0,841*		Disease resistance-responsive (dirigent-like protein) family protein; FUNCTIONS IN: molecular_function unknown; INVOLVED IN: lignan biosynthetic process, defense response; LOCATED IN: endomembrane system; EXPRESSED IN: 22 plant structures; EXPRESSED DURING: 13 growth stages; CONTAINS InterPro DOMAIN/s: Plant disease resistance response protein (InterPro:IPR004265); BEST Arabidopsis thaliana protein match is: Disease resistance-responsive (dirigent-like protein) family protein (TAIR:AT5G49040.1)
AT1G09470	*0,84*	(ATNEAP3)	NEAP3 is a member of a small family containing coiled-coil domains, a nuclear localization signal and a C-terminal predicted transmembrane domain. It localizes to the nuclear periphery. Mutants have altered nuclear morphology and chromatin structure.
AT1G32640	*0,839*	(MYC2)	Encodes a MYC-related transcriptional activator with a typical DNA binding domain of a basic helix-loop-helix leucine zipper motif. Binds to an extended G-Box promoter motif and interacts with Jasmonate ZIM-domain proteins. MYC2 interacts with EIN3 and EIL1 to repress hook curvature and resistance to Botrytis cinera.Its transcription is induced by dehydration stress and ABA treatment. Negative regulator of blue light?mediated photomorphogenic growth and blue and far-red-light?regulated gene expression. Positive regulator of lateral root formation. Regulates diverse JA-dependent functions. Negatively regulates Trp metabolism and biosynthesis of Trp-derived secondary metabolites. Positively regulates flavonoid biosynthesis, resistance to insects, and response to oxidative stress. Regulates other transcription factors, and negatively regulates its own expression. Its stability is modulated by PUB10 through polyubiquitination.
AT3G03090	*0,839*	VACUOLAR GLUCOSE TRANSPORTER 1 (VGT1)	Encodes a vacuolar membrane-localized glucose transporter that can also transport fructose. Mutations in these gene have effects on seed germination and time to flowering.
AT5G61480	*0,838*	PHLOEM INTERCALATED WITH XYLEM (PXY)	Encodes PXY, a receptor-like kinase essential for maintaining polarity during plant vascular-tissue development.
AT1G60870	*0,837*	MATERNAL EFFECT EMBRYO ARREST 9 (MEE9)	maternal effect embryo arrest 9 (MEE9); FUNCTIONS IN: molecular_function unknown; INVOLVED IN: embryo development ending in seed dormancy, pollen development; LOCATED IN: plasma membrane; EXPRESSED IN: 22 plant structures; EXPRESSED DURING: 13 growth stages
AT1G76952	*0,837*	INFLORESCENCE DEFICIENT IN ABSCISSION (IDA)-LIKE 5 (IDL5)	Similar to Inflorescence deficient in abscission (IDA). Involved in floral organ abscission.
AT5G03650	*0,837*	STARCH BRANCHING ENZYME 2.2 (SBE2.2)	Encodes starch branching enzyme (E.C.2.4.1.18) similar to SBE2 from maize and rice. Expressed throughout the plant and highest in seedlings and cauline leaves.
AT1G16070	*0,836*	TUBBY LIKE PROTEIN 8 (TLP8)	Member of TLP family

AT2G24190	*0,835*	SHORT-CHAIN DEHYDROGENASE/REDUCTASE 2 (SDR2)	Encodes an aldehyde reductase that catalyzes the reduction of the aldehyde carbonyl groups on saturated and alpha,beta-unsaturated aldehydes with more than 5 carbons in vitro. In addition, this enzyme can reduce methylglyoxal in vitro. It is believed that this enzyme localizes to the cytosol like the closely related protein encoded by AT3G61220.
AT3G25500	*0,835*	FORMIN HOMOLOGY 1 (AFH1)	Poly-L-proline-containing (PLP) protein that form part of the signal-transduction cascade that leads to rearrangement of the actin cytoskeleton. AFH1 is a nonprocessive formin that moves from the barbered end to the side of an actin filament after the nucleation event.
AT4G03440	*0,835*		Ankyrin repeat family protein; CONTAINS InterPro DOMAIN/s: Ankyrin repeat-containing domain (InterPro:IPR020683), Ankyrin repeat (InterPro:IPR002110); BEST Arabidopsis thaliana protein match is: Ankyrin repeat family protein (TAIR:AT4G03480.1)
AT5G24930	*0,834*	CONSTANS-LIKE 4 (COL4)	CONSTANS-like 4 (COL4); FUNCTIONS IN: sequence-specific DNA binding transcription factor activity, zinc ion binding; INVOLVED IN: regulation of transcription; LOCATED IN: intracellular, chloroplast; EXPRESSED IN: 21 plant structures; EXPRESSED DURING: 13 growth stages; CONTAINS InterPro DOMAIN/s: CCT domain (InterPro:IPR010402), Zinc finger, B-box (InterPro:IPR000315); BEST Arabidopsis thaliana protein match is: CONSTANS-like 3 (TAIR:AT2G24790.1)
AT4G13920	*0,829*	RECEPTOR LIKE PROTEIN 50 (RLP50)	receptor like protein 50 (RLP50); FUNCTIONS IN: kinase activity; INVOLVED IN: signal transduction, defense response; LOCATED IN: endomembrane system; CONTAINS InterPro DOMAIN/s: Leucine-rich repeat-containing N-terminal domain, type 2 (InterPro:IPR013210), Leucine-rich repeat (InterPro:IPR001611); BEST Arabidopsis thaliana protein match is: receptor like protein 47 (TAIR:AT4G13810.1)
AT4G25450	*0,829*	ATP-BINDING CASSETTE B28 (ABCB28)	member of NAP subfamily
AT5G38280	*0,827*	PR5-LIKE RECEPTOR KINASE (PR5K)	putative receptor serine/threonine kinase PR5K (PR5K) mRNA, PR5-like receptor kinase
AT1G33260	*0,825*		Protein kinase superfamily protein; FUNCTIONS IN: kinase activity; INVOLVED IN: protein amino acid phosphorylation; LOCATED IN: cellular_component unknown; EXPRESSED IN: 16 plant structures; EXPRESSED DURING: 7 growth stages; CONTAINS InterPro DOMAIN/s: Protein kinase, ATP binding site (InterPro:IPR017441), Protein kinase, catalytic domain (InterPro:IPR000719), Serine/threonine-protein kinase-like domain (InterPro:IPR017442), Protein kinase-like domain (InterPro:IPR011009), Serine/threonine-protein kinase, active site (InterPro:IPR008271); BEST Arabidopsis thaliana protein match is: Protein kinase superfamily protein (TAIR:AT4G10390.1)
AT4G01050	*0,825*	THYLAKOID RHODANESE-LIKE (TROL)	hydroxyproline-rich glycoprotein family protein, contains a rhodanese homology domain. Required for anchoring the FNR flavoenzyme to the thylakoid membranes and sustaining high efficiency photosynthetic linear electron flow. The mRNA is cell-to-cell mobile.
AT5G09870	*0,825*	CELLULOSE SYNTHASE 5 (CESA5)	Encodes a cellulose synthase CESA5 that produces seed mucilage cellulose.Mutants are defective in seed coat mucilage.Involved in the regulation of mucilage composition and/or mucilage synthesis.
AT1G69870	*0,824*	NRT1/ PTR FAMILY 2.13 (NPF2.13)	Encodes a low affinity nitrate transporter NRT1.7. Expressed in phloem. Responsible for source-to-sink remobilization of nitrate. The mRNA is cell-to-cell mobile.
AT3G07770	*0,824*	HEAT SHOCK PROTEIN 89.1 (Hsp89.1)	HEAT SHOCK PROTEIN 89.1 (Hsp89.1); FUNCTIONS IN: ATP binding; INVOLVED IN: protein folding, response to stress; LOCATED IN: mitochondrion, cell wall; EXPRESSED IN: 24 plant structures; EXPRESSED DURING: 14 growth stages; CONTAINS InterPro DOMAIN/s: Chaperone protein htpG (InterPro:IPR001404), Heat shock protein Hsp90, C-terminal (InterPro:IPR020576), Heat shock protein Hsp90, conserved site (InterPro:IPR019805), Ribosomal protein S5 domain 2-type fold (InterPro:IPR020568), Heat shock protein Hsp90, N-terminal (InterPro:IPR020575), ATPase-like, ATP-binding domain (InterPro:IPR003594); BEST Arabidopsis thaliana protein match is: Chaperone protein htpG family protein (TAIR:AT2G04030.1)
AT3G20100	*0,823*	CYTOCHROME P450, FAMILY 705, SUBFAMILY A, POLYPEPTIDE 19 (CYP705A19)	member of CYP705A The mRNA is cell-to-cell mobile.

AT4G15830	*0,823*		ARM repeat superfamily protein; FUNCTIONS IN: binding; INVOLVED IN: biological_process unknown; LOCATED IN: cellular_component unknown; EXPRESSED IN: 21 plant structures; EXPRESSED DURING: 14 growth stages; CONTAINS InterPro DOMAIN/s: Armadillo-type fold (InterPro:IPR016024); BEST Arabidopsis thaliana protein match is: ARM repeat superfamily protein (TAIR:AT3G01450.1)
AT4G35730	*0,823*	IST1-LIKE 3 (ISTL3)	Regulator of Vps4 activity in the MVB pathway protein; CONTAINS InterPro DOMAIN/s: Protein of unknown function DUF292, eukaryotic (InterPro:IPR005061); BEST Arabidopsis thaliana protein match is: Regulator of Vps4 activity in the MVB pathway protein (TAIR:AT1G34220.2)
AT5G43900	*0,822*	MYOSIN 2 (MYA2)	Encodes a member of the type XI myosin protein family that binds F-actin and co-localizes with actin filaments and peroxisomes. Homozygous mutants are reported to have pleiotropic effects in growth and fertility and may also be lethal. This protein is also involved in root hair growth and organelle trafficking. This protein interacts with RabC2a and RabD1 in a GTP-dependent manner.
AT1G60600	*0,82*	ABERRANT CHLOROPLAST DEVELOPMENT 4 (ABC4)	Encodes a protein similar to 1,4-dihydroxy-2-naphthoic acid phytyltransferase involved in phylloquinone and plastoquinone biosynthesis. Mutants are pale green and heterotrophic with defects in photosynthetic electron transport.
AT3G15790	*0,82*	METHYL-CPG-BINDING DOMAIN 11 (MBD11)	Protein containing methyl-CpG-binding domain.Has sequence similarity to human MBD proteins.
AT1G06620	*0,818*		encodes a protein whose sequence is similar to a 2-oxoglutarate-dependent dioxygenase The mRNA is cell-to-cell mobile.
AT4G30650	*0,818*		Low temperature and salt responsive protein family; INVOLVED IN: response to salt stress, response to cold, hyperosmotic salinity response, defense response to fungus; LOCATED IN: endomembrane system, integral to membrane; EXPRESSED IN: 21 plant structures; EXPRESSED DURING: 13 growth stages; CONTAINS InterPro DOMAIN/s: Uncharacterised protein family UPF0057 (InterPro:IPR000612); BEST Arabidopsis thaliana protein match is: Low temperature and salt responsive protein family (TAIR:AT4G30660.2)
AT5G11510	*0,818*	MYB DOMAIN PROTEIN 3R-4 (MYB3R-4)	Arabidopsis thaliana putative c-myb-like transcription factor MYB3R-4. Functions in powdery mildew induced host endoreduplication at the site of infection.
AT5G42570	*0,818*		B-cell receptor-associated 31-like; FUNCTIONS IN: molecular_function unknown; INVOLVED IN: intracellular protein transport; LOCATED IN: endoplasmic reticulum, plasma membrane; EXPRESSED IN: 26 plant structures; EXPRESSED DURING: 13 growth stages; CONTAINS InterPro DOMAIN/s: B-cell receptor-associated 31-like (InterPro:IPR008417); BEST Arabidopsis thaliana protein match is: B-cell receptor-associated protein 31-like (TAIR:AT1G11905.1)
AT5G42650	*0,818*	ALLENE OXIDE SYNTHASE (AOS)	Encodes a member of the cytochrome p450 CYP74 gene family that functions as an allene oxide synthase. This enzyme catalyzes dehydration of the hydroperoxide to an unstable allene oxide in the JA biosynthetic pathway. It shows a dual catalytic activity, the major one being a 13-AOS but also expressing a 9-AOS activity. CFA-Leu, CFA-Val, CFA-Met and CFA-Ala can induce the expression of AOS.
AT5G53590	*0,818*	SMALL AUXIN UPREGULATED RNA 30 (SAUR30)	SAUR-like auxin-responsive protein family ; CONTAINS InterPro DOMAIN/s: Auxin responsive SAUR protein (InterPro:IPR003676); BEST Arabidopsis thaliana protein match is: SAUR-like auxin-responsive protein family (TAIR:AT4G00880.1)
AT3G01410	*0,816*		Polynucleotidyl transferase, ribonuclease H-like superfamily protein; FUNCTIONS IN: ribonuclease H activity, nuclease activity, nucleic acid binding; INVOLVED IN: biological_process unknown; LOCATED IN: cellular_component unknown; EXPRESSED IN: 12 plant structures; EXPRESSED DURING: 7 growth stages; CONTAINS InterPro DOMAIN/s: Polynucleotidyl transferase, ribonuclease H fold (InterPro:IPR012337), Ribonuclease H (InterPro:IPR002156); BEST Arabidopsis thaliana protein match is: RNase H family protein (TAIR:AT1G24090.1)
AT3G54090	*0,816*	FRUCTOKINASE-LIKE 1 (FLN1)	Encodes a fructokinase-like protein (AT3G54090/FLN1, AT1G69200/FLN2), a member of the pfkB-carbohydrate kinase family. FLN1 and FLN2 are potential plastidial thioredoxin z (TRX z) targets. Mutants display mutant chloroplast development, general plant growth and development defects and defects in PEP-dependent transcription.
AT3G61470	*0,816*	PHOTOSYSTEM I LIGHT HARVESTING COMPLEX GENE 2 (LHCA2)	Encodes a component of the light harvesting antenna complex of photosystem I. The mRNA is cell-to-cell mobile.

AT1G66410	*0,815*	CALMODULIN 4 (CAM4)	encodes a calmodulin
AT2G20590	*0,815*		Reticulon family protein; INVOLVED IN: biological_process unknown; LOCATED IN: endoplasmic reticulum, chloroplast; CONTAINS InterPro DOMAIN/s: Reticulon (InterPro:IPR003388); BEST Arabidopsis thaliana protein match is: Reticulon family protein (TAIR:AT4G28430.1)
AT4G22770	*0,815*	AT-HOOK MOTIF NUCLEAR LOCALIZED PROTEIN 2 (AHL2)	AT hook motif DNA-binding family protein; FUNCTIONS IN: DNA binding; EXPRESSED IN: 14 plant structures; EXPRESSED DURING: 4 anthesis, F mature embryo stage, petal differentiation and expansion stage, E expanded cotyledon stage, D bilateral stage; CONTAINS InterPro DOMAIN/s: Protein of unknown function DUF296 (InterPro:IPR005175), AT hook, DNA-binding motif (InterPro:IPR017956); BEST Arabidopsis thaliana protein match is: AT-hook motif nuclear-localized protein 1 (TAIR:AT4G12080.1)
AT4G38530	*0,814*	PHOSPHOLIPASE C1 (PLC1)	Encodes a putative phosphoinositide-specific phospholipase C. There are two genes called ATPLC1, one corresponding to AT4g38530 (this one) and one corresponding to AT5g58670.
AT2G40530	*0,812*		unknown protein; FUNCTIONS IN: molecular_function unknown; INVOLVED IN: biological_process unknown; LOCATED IN: endomembrane system; EXPRESSED IN: leaf apex, inflorescence meristem, petal, flower; EXPRESSED DURING: 4 anthesis, petal differentiation and expansion stage
AT5G47110	*0,812*	LIGHT-HARVESTING-LIKE 3:2 (LIL3:2)	Encodes a light-harvesting-like protein that is involved in chlorophyll and tocopherol biosynthesis anchoring geranylgeranyl reductase in the thylakoid membrane.
AT1G78610	*0,811*	MECHANOSENSITIVE CHANNEL OF SMALL CONDUCTANCE-LIKE 6 (MSL6)	mechanosensitive channel of small conductance-like 6 (MSL6); INVOLVED IN: transmembrane transport; LOCATED IN: plasma membrane, membrane; EXPRESSED IN: 23 plant structures; EXPRESSED DURING: 13 growth stages; CONTAINS InterPro DOMAIN/s: Membrane protein, At2g17000, predicted (InterPro:IPR016688), Mechanosensitive ion channel MscS (InterPro:IPR006685), Like-Sm ribonucleoprotein (LSM)-related domain (InterPro:IPR010920); BEST Arabidopsis thaliana protein match is: mechanosensitive channel of small conductance-like 4 (TAIR:AT1G53470.1)
AT2G35260	*0,811*		unknown protein; FUNCTIONS IN: molecular_function unknown; INVOLVED IN: biological_process unknown; LOCATED IN: chloroplast; EXPRESSED IN: 21 plant structures; EXPRESSED DURING: 13 growth stages; BEST Arabidopsis thaliana protein match is: unknown protein (TAIR:AT4G17840.1)
AT2G41130	*0,811*	(BHLH106)	Encodes STC8 (salt tolerant callus 8)/bHLH106 (basic helix-loop-helix transcription factor bHLH106). Knockout lines are more sensitive to NaCl, KCl, LiCl, ABA, and low temperatures than the wild-type.
AT4G20320	*0,81*		CTP synthase family protein; FUNCTIONS IN: CTP synthase activity, catalytic activity; INVOLVED IN: pyrimidine ribonucleotide metabolic process, pyrimidine nucleotide biosynthetic process; LOCATED IN: cellular_component unknown; EXPRESSED IN: 18 plant structures; EXPRESSED DURING: 8 growth stages; CONTAINS InterPro DOMAIN/s: Glutamine amidotransferase class-I, C-terminal (InterPro:IPR000991), CTP synthase (InterPro:IPR004468), CTP synthase, N-terminal (InterPro:IPR017456), Glutamine amidotransferase type 1 (InterPro:IPR017926); BEST Arabidopsis thaliana protein match is: CTP synthase family protein (TAIR:AT1G30820.1)
AT4G02770	*0,809*	PHOTOSYSTEM I SUBUNIT D-1 (PSAD-1)	Encodes a protein predicted by sequence similarity with spinach PsaD to be photosystem I reaction center subunit II (PsaD1)
AT2G17480	*0,807*	MILDEW RESISTANCE LOCUS O 8 (MLO8)	A member of a large family of seven-transmembrane domain proteins specific to plants, homologs of the barley mildew resistance locus o (MLO) protein. The Arabidopsis genome contains 15 genes encoding MLO proteins, with localization in plasma membrane. Phylogenetic analysis revealed four clades of closely-related AtMLO genes. ATMLO8 belongs to the clade III, with AtMLO5, AtMLO7, AtMLO9, and AtMLO10. The gene is expressed during seedling growth, in cotyledons and hypocotyl, and in fruit abscission zone, as shown by GUS activity patterns. The expression of several phylogenetically closely-related AtMLO genes showed similar or overlapping tissue specificity and analogous responsiveness to external stimuli, suggesting functional redundancy, co-function, or antagonistic function(s).

AT3G02400	*0,807*		SMAD/FHA domain-containing protein ; FUNCTIONS IN: DNA binding; INVOLVED IN: regulation of transcription, DNA-dependent; LOCATED IN: nucleus; EXPRESSED IN: 21 plant structures; EXPRESSED DURING: 11 growth stages; CONTAINS InterPro DOMAIN/s: AT hook, DNA-binding motif (InterPro:IPR017956), SMAD/FHA domain (InterPro:IPR008984), Forkhead-associated (FHA) domain (InterPro:IPR000253); BEST Arabidopsis thaliana protein match is: SMAD/FHA domain-containing protein (TAIR:AT4G14490.1)
AT1G56130	*0,803*		Leucine-rich repeat transmembrane protein kinase; FUNCTIONS IN: protein serine/threonine kinase activity, protein kinase activity, ATP binding; INVOLVED IN: protein amino acid phosphorylation; LOCATED IN: plasma membrane, membrane; EXPRESSED IN: cultured cell; CONTAINS InterPro DOMAIN/s: Serine/threonine-protein kinase domain (InterPro:IPR002290), Leucine-rich repeat (InterPro:IPR001611), Serine-threonine/tyrosine-protein kinase (InterPro:IPR001245), Serine/threonine-protein kinase, active site (InterPro:IPR008271), Protein kinase-like domain (InterPro:IPR011009), Protein kinase, catalytic domain (InterPro:IPR000719), Malectin/receptor-like protein kinase (InterPro:IPR021720), Tyrosine-protein kinase, catalytic domain (InterPro:IPR020635); BEST Arabidopsis thaliana protein match is: Leucine-rich repeat transmembrane protein kinase (TAIR:AT1G56140.1)
AT2G46450	*0,803*	CYCLIC NUCLEOTIDE-GATED CHANNEL 12 (CNGC12)	Member of Cyclic nucleotide gated channel family.Positive regulator of resistance against avirulent fungal pathogen.Suppresses the phenotype conferred by cpr22 in a dosage-dependent manner.
AT3G15730	*0,802*	PHOSPHOLIPASE D ALPHA 1 (PLDALPHA1)	Encodes phospholipase D alpha 1 (PLD alpha 1). Positive regulator of abscisic acid (ABA) mediated stomatal movements. PLD alpha 1 plays an important role in seed deterioration and aging in Arabidopsis. The mRNA is cell-to-cell mobile.
AT3G29320	*0,802*	ALPHA-GLUCAN PHOSPHORYLASE 1 (PHS1)	Encodes a plastidic alpha-glucan phosphorylase. In vitro, the enzyme has a preference for maltooligosaccharides, such as maltoheptaose. The mRNA is cell-to-cell mobile.
AT5G37530	*0,802*		NAD(P)-binding Rossmann-fold superfamily protein; FUNCTIONS IN: binding, catalytic activity; INVOLVED IN: metabolic process; LOCATED IN: endomembrane system; EXPRESSED IN: 13 plant structures; EXPRESSED DURING: 7 growth stages; CONTAINS InterPro DOMAIN/s: UBA/THIF-type NAD/FAD binding fold (InterPro:IPR000594), Molybdenum cofactor biosynthesis, MoeB (InterPro:IPR009036), NAD(P)-binding domain (InterPro:IPR016040); BEST Arabidopsis thaliana protein match is: co-factor for nitrate, reductase and xanthine dehydrogenase 5 (TAIR:AT5G55130.1)
AT5G49620	*0,802*	MYB DOMAIN PROTEIN 78 (MYB78)	Member of the R2R3 factor gene family.
AT3G21240	*0,799*	4-COUMARATE:COA LIGASE 2 (4CL2)	encodes an isoform of 4-coumarate:CoA ligase (4CL), which is involved in the last step of the general phenylpropanoid pathway. The catalytic efficiency was in the following (descending) order: p-coumaric acid, caffeic acid, ferulic acid, 5-OH-ferulic acid and cinnamic acid. At4CL2 was unable to use sinapic acid as substrate.
AT5G44740	*0,799*	Y-FAMILY DNA POLYMERASE H (POLH)	Y-family DNA polymerase. Catalyses translesion synthesis in response to UV damage. Functionally interacts with PCNA2. Has a ubiquitin binding motif.
AT4G38380	*0,798*		MATE efflux family protein; FUNCTIONS IN: antiporter activity, drug transmembrane transporter activity; INVOLVED IN: drug transmembrane transport, transmembrane transport; LOCATED IN: chloroplast; EXPRESSED IN: 13 plant structures; EXPRESSED DURING: 7 growth stages; CONTAINS InterPro DOMAIN/s: Multi antimicrobial extrusion protein MatE (InterPro:IPR002528); BEST Arabidopsis thaliana protein match is: MATE efflux family protein (TAIR:AT2G38330.1)
AT5G57015	*0,798*	CASEIN KINASE I-LIKE 12 (ckl12)	casein kinase I-like 12 (ckl12); FUNCTIONS IN: protein serine/threonine kinase activity, protein kinase activity, kinase activity, ATP binding; INVOLVED IN: protein amino acid phosphorylation; LOCATED IN: cytoplasm; CONTAINS InterPro DOMAIN/s: Protein kinase, ATP binding site (InterPro:IPR017441), Protein kinase, catalytic domain (InterPro:IPR000719), Serine/threonine-protein kinase-like domain (InterPro:IPR017442), Protein kinase-like domain (InterPro:IPR011009), Serine/threonine-protein kinase, active site (InterPro:IPR008271); BEST Arabidopsis thaliana protein match is: casein kinase 1 (TAIR:AT4G26100.1)
AT1G20340	*0,797*	DNA-DAMAGE-REPAIR/TOLERATION PROTEIN 112 (DRT112)	recombination and DNA-damage resistance protein (DRT112) One of two Arabidopsis plastocyanin genes. Predominant form, expressed 10x higher than PETE1. PETE2 is thought to be post-transcriptionally regulated via copper accumulation and is involved in copper homeostasis. Mutation of this gene does not have obvious effect on photosynthesis. In plsp1-1 mutant plastids, the nonmature form of the protein localizes in the membrane.

AT5G64640	*0,797*		Plant invertase/pectin methylesterase inhibitor superfamily; FUNCTIONS IN: enzyme inhibitor activity, pectinesterase activity; INVOLVED IN: cell wall modification; LOCATED IN: plasma membrane, plant-type cell wall; EXPRESSED IN: 24 plant structures; EXPRESSED DURING: 15 growth stages; CONTAINS InterPro DOMAIN/s: Pectin lyase fold/virulence factor (InterPro:IPR011050), Pectinesterase, catalytic (InterPro:IPR000070), Pectinesterase inhibitor (InterPro:IPR006501), Pectin lyase fold (InterPro:IPR012334); BEST Arabidopsis thaliana protein match is: Plant invertase/pectin methylesterase inhibitor superfamily (TAIR:AT5G09760.1)
AT3G04260	*0,796*	PLASTID TRANSCRIPTIONALLY ACTIVE 3 (PTAC3)	plastid transcriptionally active 3 (PTAC3); FUNCTIONS IN: DNA binding, nucleic acid binding; LOCATED IN: plastid chromosome, nucleus, chloroplast, nucleoid, membrane; EXPRESSED IN: 22 plant structures; EXPRESSED DURING: 13 growth stages; CONTAINS InterPro DOMAIN/s: DNA-binding SAP (InterPro:IPR003034); BEST Arabidopsis thaliana protein match is: genomes uncoupled 1 (TAIR:AT2G31400.1)
AT4G32400	*0,796*	SODIUM HYPERSENSITIVE 1 (SHS1)	Encodes a plastidial nucleotide uniport carrier protein required to export newly synthesized adenylates into the cytosol.
AT5G24770	*0,796*	VEGETATIVE STORAGE PROTEIN 2 (VSP2)	Has acid phosphatase activity dependent on the presence of divalent cations (Mg2+, Co2+, Zn2+, Mn2+) and anti-insect activity. Insects fed with the protein show a retarded development. Induced in response to abscisic acid, jasmonic acid, salt, water deficiency and wounding.
AT1G03300	*0,795*	DOMAIN OF UNKNOWN FUNCTION 724 1 (ATDUF1)	Member of the plant-specific DUF724 protein family. Arabidopsis has 10 DUF724 proteins. Loss of function mutant has a WT phenotype
AT1G60000	*0,795*		RNA-binding (RRM/RBD/RNP motifs) family protein; FUNCTIONS IN: RNA binding, nucleotide binding, nucleic acid binding; INVOLVED IN: biological_process unknown; LOCATED IN: chloroplast; EXPRESSED IN: 22 plant structures; EXPRESSED DURING: 13 growth stages; CONTAINS InterPro DOMAIN/s: RNA recognition motif, RNP-1 (InterPro:IPR000504), Nucleotide-binding, alpha-beta plait (InterPro:IPR012677); BEST Arabidopsis thaliana protein match is: chloroplast RNA-binding protein 31B (TAIR:AT5G50250.1)
AT1G16780	*0,794*	(VHP2;2)	Encodes a type II H+-PPases that localizes to and function as a proton pump of the Golgi apparatus in most tissues except for mature leaves.
AT1G50970	*0,794*		Membrane trafficking VPS53 family protein; FUNCTIONS IN: transporter activity; INVOLVED IN: transport; LOCATED IN: endomembrane system; EXPRESSED IN: 18 plant structures; EXPRESSED DURING: 10 growth stages; CONTAINS InterPro DOMAIN/s: Vps53-like, N-terminal (InterPro:IPR007234); BEST Arabidopsis thaliana protein match is: Membrane trafficking VPS53 family protein (TAIR:AT1G50500.1)
AT2G44480	*0,794*	BETA GLUCOSIDASE 17 (BGLU17)	beta glucosidase 17 (BGLU17); FUNCTIONS IN: cation binding, hydrolase activity, hydrolyzing O-glycosyl compounds, catalytic activity; INVOLVED IN: carbohydrate metabolic process; LOCATED IN: endomembrane system; EXPRESSED IN: stem, hypocotyl, sepal, male gametophyte, root; EXPRESSED DURING: 4 anthesis; CONTAINS InterPro DOMAIN/s: Glycoside hydrolase, family 1 (InterPro:IPR001360), Glycoside hydrolase, family 1, active site (InterPro:IPR018120), Glycoside hydrolase, catalytic core (InterPro:IPR017853), Glycoside hydrolase, subgroup, catalytic core (InterPro:IPR013781); BEST Arabidopsis thaliana protein match is: beta glucosidase 15 (TAIR:AT2G44450.1)
AT3G53750	*0,794*	ACTIN 3 (ACT3)	Member of the Actin gene family. Expressed in mature pollen.
AT3G46970	*0,793*	ALPHA-GLUCAN PHOSPHORYLASE 2 (PHS2)	Encodes a cytosolic alpha-glucan phosphorylase. In vitro, the enzyme has a preference for branched polysaccharides, such as glycogen.
AT3G62150	*0,792*	ATP-BINDING CASSETTE B21 (ABCB21)	Encodes a facultative transporter controlling auxin concentrations in plant cells.
AT5G48930	*0,792*	HYDROXYCINNAMOYL-COA SHIKIMATE/QUINATE HYDROXYCINNAMOYL TRANSFERASE (HCT)	At5g48930 has been shown to encode for the hydroxycinnamoyl-Coenzyme A shikimate/quinate hydroxycinnamoyltransferase (HCT) both synthesizing and catabolizing the hydroxycinnamoylesters (coumaroyl/caffeoyl shikimate and quinate) involved in the phenylpropanoid pathway. Influence on the accumulation of flavonoids which in turn inhibit auxin transport and reduce plant growth. The mRNA is cell-to-cell mobile.

AT3G02790	*0,79*	METHYLENE BLUE SENSITIVITY 1 (MBS1)	zinc finger (C2H2 type) family protein; FUNCTIONS IN: sequence-specific DNA binding transcription factor activity; INVOLVED IN: regulation of transcription; LOCATED IN: intracellular; EXPRESSED IN: 22 plant structures; EXPRESSED DURING: 14 growth stages; CONTAINS InterPro DOMAIN/s: Zinc finger, C2H2-like (InterPro:IPR015880); BEST Arabidopsis thaliana protein match is: zinc finger (C2H2 type) family protein (TAIR:AT5G16470.1)
AT3G18750	*0,79*	WITH NO LYSINE (K) KINASE 6 (WNK6)	Encodes a member of the WNK family (9 members in all) of protein kinases, the structural design of which is clearly distinct from those of other known protein kinases, such as receptor-like kinases and mitogen-activated protein kinases. Its transcription is under the control of circadian rhythms.
AT2G35940	*0,789*	BEL1-LIKE HOMEODOMAIN 1 (BLH1)	Encodes a member of the BEL-like homeodomain protein family. Ecotopic expression in the embryo sac leads to defects in nuclear migration and cellularization and embryo sacs with multiple egg cells. Loss of function alleles have no female gametophyte defects. The ecotopic expression phenotype requires KNAT3 because it can be suppressed by loss of KNAT3 function alleles. Localized to the nucleus but interaction with OFP1 relocates it to the cytoplasm.
AT5G01170	*0,789*		Protein of unknown function (DUF740); CONTAINS InterPro DOMAIN/s: Protein of unknown function DUF740 (InterPro:IPR008004); BEST Arabidopsis thaliana protein match is: Protein of unknown function (DUF740) (TAIR:AT3G09070.1)
AT1G67740	*0,788*	PHOTOSYSTEM II BY (PSBY)	PsbY precursor (psbY) mRNA. This single nuclear gene is imported into the chloroplasts where it is processed into two integral membrane proteins with identical topology (PsbY-1 and PsbY-2). The protein appears to bind manganese. Important for the redox control of cytochrome b559.
AT4G12800	*0,788*	PHOTOSYSTEM I SUBUNIT L (PSAL)	Encodes subunit L of photosystem I reaction center.
AT4G16370	*0,788*	OLIGOPEPTIDE TRANSPORTER (OPT3)	Encodes a phloem-specific iron transporter that is essential for systemic iron signaling and redistribution of iron and cadmium. It loads iron into the phloem, facilitates iron recirculation from the xylem to the phloem, and regulates both shoot-to-root iron signaling and iron redistribution from mature to developing tissues.
AT5G67160	*0,788*	ENHANCED PSEUDOMONAS SUSCEPTIBILTY 1 (EPS1)	Encodes a member of the BAHD acyltransferase superfamily. Mutants have enhanced susceptibility to virulent and avirulent pathogens and are defective in pathogen induced SA biosynthesis. EPS1 may act upstream of SA biosynthesis as application of SA can rescue the mutant phenotype.
AT1G74720	*0,787*	QUIRKY (QKY)	Encodes a putative transmembrane protein carrying four C(2) domains, suggesting that QKY may function in membrane trafficking in a Ca(2+)-dependent fashion. Mutant analysis shows that this gene is involved in organ development.
AT1G10990	*0,786*		unknown protein
AT1G70090	*0,786*	GLUCOSYL TRANSFERASE FAMILY 8 (LGT8)	Encodes a protein with putative galacturonosyltransferase activity.
AT2G28930	*0,785*	PROTEIN KINASE 1B (PK1B)	protein kinase 1B (PK1B); FUNCTIONS IN: protein serine/threonine kinase activity, protein kinase activity, kinase activity, ATP binding; INVOLVED IN: protein amino acid phosphorylation, N-terminal protein myristoylation; LOCATED IN: plasma membrane; EXPRESSED IN: 22 plant structures; EXPRESSED DURING: 13 growth stages; CONTAINS InterPro DOMAIN/s: Protein kinase, ATP binding site (InterPro:IPR017441), Protein kinase, catalytic domain (InterPro:IPR000719), Serine-threonine/tyrosine-protein kinase (InterPro:IPR001245), Protein kinase-like domain (InterPro:IPR011009), Serine/threonine-protein kinase, active site (InterPro:IPR008271); BEST Arabidopsis thaliana protein match is: Protein kinase superfamily protein (TAIR:AT1G07570.1)
AT1G13220	*0,783*	LITTLE NUCLEI2 (LINC2)	Encodes a nuclear coiled-coil protein related to the carrot peripheral nuclear protein NMCP1 that is involved in the determination of plant nuclear structure.
AT3G02730	*0,783*	THIOREDOXIN F-TYPE 1 (TRXF1)	Encodes a type-f thioredoxin. Has a role in the short-term activation of carbon metabolism. Loss affects growth under short-day conditions.
AT3G26980	*0,783*	MEMBRANE-ANCHORED UBIQUITIN-FOLD PROTEIN 4 PRECURSOR (MUB4)	membrane-anchored ubiquitin-fold protein 4 precursor (MUB4); CONTAINS InterPro DOMAIN/s: Membrane-anchored ubiquitin-fold protein, HCG-1 (InterPro:IPR017000), Ubiquitin (InterPro:IPR000626), Ubiquitin supergroup (InterPro:IPR019955); BEST Arabidopsis thaliana protein match is: Ubiquitin family protein (TAIR:AT4G24990.1)
AT3G58160	*0,783*	(XIJ)	Class XI myosin gene expressed in flowers from 4-6 week old plants and leaves from 3 week old plants

AT4G03390	*0,783*	STRUBBELIG-RECEPTOR FAMILY 3 (SRF3)	STRUBBELIG-receptor family 3 (SRF3); FUNCTIONS IN: protein serine/threonine kinase activity, kinase activity, ATP binding; INVOLVED IN: transmembrane receptor protein tyrosine kinase signaling pathway, protein amino acid phosphorylation; LOCATED IN: plasma membrane; EXPRESSED IN: 23 plant structures; EXPRESSED DURING: 13 growth stages; CONTAINS InterPro DOMAIN/s: Protein kinase, catalytic domain (InterPro:IPR000719), Serine/threonine-protein kinase domain (InterPro:IPR002290), Leucine-rich repeat-containing N-terminal domain, type 2 (InterPro:IPR013210), Leucine-rich repeat (InterPro:IPR001611), Tyrosine-protein kinase, catalytic domain (InterPro:IPR020635), Serine-threonine/tyrosine-protein kinase (InterPro:IPR001245), Protein kinase-like domain (InterPro:IPR011009); BEST Arabidopsis thaliana protein match is: STRUBBELIG-receptor family 1 (TAIR:AT2G20850.1)
AT2G47590	*0,782*	PHOTOLYASE/BLUE-LIGHT RECEPTOR 2 (PHR2)	photolyase/blue light photoreceptor PHR2 (PHR2) mRNA,
AT2G43710	*0,781*	SUPPRESSOR OF SA INSENSITIVE 2 (SSI2)	Encodes a stearoyl-ACP desaturase, involved in fatty acid desaturation. The ssi2 mutants have increased 18:0 and reduced 18:1 fatty acids. Exogenous application of glycerol to wild type plants mimics the ssi2 mutant phenotype. The altered 18:1 fatty acid content in the ssi2 mutants has an impact on SA- and JA-mediated defense signaling. ssi2 mutants resulted in hyper-resistance to green peach aphid and antibiosis activity in petiole exudates.
AT4G23740	*0,781*		Leucine-rich repeat protein kinase family protein; FUNCTIONS IN: protein serine/threonine kinase activity, kinase activity, ATP binding; INVOLVED IN: transmembrane receptor protein tyrosine kinase signaling pathway, protein amino acid phosphorylation; LOCATED IN: plasma membrane; EXPRESSED IN: 22 plant structures; EXPRESSED DURING: 13 growth stages; CONTAINS InterPro DOMAIN/s: Protein kinase, ATP binding site (InterPro:IPR017441), Serine/threonine-protein kinase domain (InterPro:IPR002290), Leucine-rich repeat-containing N-terminal domain, type 2 (InterPro:IPR013210), Leucine-rich repeat (InterPro:IPR001611), Serine/threonine-protein kinase-like domain (InterPro:IPR017442), Protein kinase-like domain (InterPro:IPR011009), Protein kinase, catalytic domain (InterPro:IPR000719), Tyrosine-protein kinase, catalytic domain (InterPro:IPR020635); BEST Arabidopsis thaliana protein match is: Leucine-rich repeat protein kinase family protein (TAIR:AT1G64210.1)
AT5G11740	*0,781*	ARABINOGALACTAN PROTEIN 15 (AGP15)	Encodes arabinogalactan protein (AGP15). The mRNA is cell-to-cell mobile.
AT5G59730	*0,781*	EXOCYST SUBUNIT EXO70 FAMILY PROTEIN H7 (EXO70H7)	A member of EXO70 gene family, putative exocyst subunits, conserved in land plants. Arabidopsis thaliana contains 23 putative EXO70 genes, which can be classified into eight clusters on the phylogenetic tree. The mRNA is cell-to-cell mobile.
AT5G24580	*0,78*		Heavy metal transport/detoxification superfamily protein ; FUNCTIONS IN: copper ion binding, metal ion binding; INVOLVED IN: copper ion transport, metal ion transport; LOCATED IN: cellular_component unknown; EXPRESSED IN: 20 plant structures; EXPRESSED DURING: 13 growth stages; CONTAINS InterPro DOMAIN/s: Heavy metal transport/detoxification protein (InterPro:IPR006121); BEST Arabidopsis thaliana protein match is: Heavy metal transport/detoxification superfamily protein (TAIR:AT5G50740.1)
AT1G28400	*0,779*		unknown protein; FUNCTIONS IN: molecular_function unknown; INVOLVED IN: biological_process unknown; LOCATED IN: endomembrane system; EXPRESSED IN: 22 plant structures; EXPRESSED DURING: 13 growth stages; BEST Arabidopsis thaliana protein match is: unknown protein (TAIR:AT2G33850.1)
AT5G37770	*0,779*	TOUCH 2 (TCH2)	Encodes a protein with 40% similarity to calmodulin. Binds Ca(2+) and, as a consequence, undergoes conformational changes. CML24 expression occurs in all major organs, and transcript levels are increased from 2- to 15-fold in plants subjected to touch, darkness, heat, cold, hydrogen peroxide, abscisic acid (ABA), and indole-3-acetic acid. However, CML24 protein accumulation changes were not detectable. The putative CML24 regulatory region confers reporter expression at sites of predicted mechanical stress; in regions undergoing growth; in vascular tissues and various floral organs; and in stomata, trichomes, and hydathodes. CML24-underexpressing transgenics are resistant to ABA inhibition of germination and seedling growth, are defective in long-day induction of flowering, and have enhanced tolerance to CoCl(2), molybdic acid, ZnSO(4), and MgCl(2). Also regulates nitric oxide levels.

AT1G12550	*0,778*	HYDROXYPYRUVATE REDUCTASE 3 (HPR3)	Encodes a hydroxypyruvate reductase that reduces HP to glycerate and shows even more activity with glyoxylate, a more upstream intermediate of the photorespiratory cycle. It is likely targeted to the chloroplast where it could provide a compensatory bypass for the reduction of HP and glyoxylate within this compartment.
AT3G25740	*0,778*	METHIONINE AMINOPEPTIDASE 1C (MAP1B)	Encodes a plastid localized methionine aminopeptidase. Formerly called MAP1C, now called MAP1B.
AT4G37750	*0,778*	AINTEGUMENTA (ANT)	ANT is required for control of cell proliferation and encodes a putative transcriptional regulator similar to AP2. Loss of function alleles have reduced fertility, abnormal ovules and abnormal lateral organs. Expressed specifically in the chalaza and in floral organ primordia.
AT5G13650	*0,777*	SUPPRESSOR OF VARIEGATION 3 (SVR3)	Encodes SVR3, a putative chloroplast TypA translation elongation GTPase. Loss of SVR3 suppresses variegation mediated by var2. SVR3 is essential for plants? ability to develop functional chloroplasts under chilling stress (8C), but not at normal temperature (22C).
AT5G44050	*0,777*		MATE efflux family protein; FUNCTIONS IN: antiporter activity, drug transmembrane transporter activity, transporter activity; INVOLVED IN: drug transmembrane transport, transmembrane transport; LOCATED IN: membrane; EXPRESSED IN: 10 plant structures; EXPRESSED DURING: 7 growth stages; CONTAINS InterPro DOMAIN/s: Multi antimicrobial extrusion protein MatE (InterPro:IPR002528); BEST Arabidopsis thaliana protein match is: MATE efflux family protein (TAIR:AT5G65380.1)
AT1G26270	*0,775*		Phosphatidylinositol 3- and 4-kinase family protein; FUNCTIONS IN: inositol or phosphatidylinositol kinase activity, phosphotransferase activity, alcohol group as acceptor; LOCATED IN: cellular_component unknown; EXPRESSED IN: 23 plant structures; EXPRESSED DURING: 13 growth stages; CONTAINS InterPro DOMAIN/s: Phosphatidylinositol 3-/4-kinase, catalytic (InterPro:IPR000403); BEST Arabidopsis thaliana protein match is: phosphoinositide 4-kinase gamma 7 (TAIR:AT2G03890.1)
AT2G25530	*0,774*		AFG1-like ATPase family protein; FUNCTIONS IN: ATPase activity, ATP binding; INVOLVED IN: biological_process unknown; LOCATED IN: cellular_component unknown; EXPRESSED IN: 23 plant structures; EXPRESSED DURING: 14 growth stages; CONTAINS InterPro DOMAIN/s: ATPase, AFG1-like (InterPro:IPR005654); BEST Arabidopsis thaliana protein match is: AFG1-like ATPase family protein (TAIR:AT4G28070.2)
AT2G25690	*0,774*		Protein of unknown function (DUF581); CONTAINS InterPro DOMAIN/s: Protein of unknown function DUF581 (InterPro:IPR007650); BEST Arabidopsis thaliana protein match is: Protein of unknown function (DUF581) (TAIR:AT5G11460.1)
AT4G29060	*0,774*	EMBRYO DEFECTIVE 2726 (emb2726)	embryo defective 2726 (emb2726); FUNCTIONS IN: RNA binding, translation elongation factor activity; INVOLVED IN: translational elongation, response to cadmium ion, embryo development ending in seed dormancy; LOCATED IN: chloroplast stroma, chloroplast, chloroplast envelope; EXPRESSED IN: 24 plant structures; EXPRESSED DURING: 14 growth stages; CONTAINS InterPro DOMAIN/s: Nucleic acid-binding, OB-fold (InterPro:IPR012340), Ubiquitin-associated/translation elongation factor EF1B, N-terminal (InterPro:IPR000449), Ribosomal protein S1, RNA-binding domain (InterPro:IPR003029), Translation elongation factor EFTs/EF1B (InterPro:IPR001816), Translation elongation factor EFTs/EF1B, dimerisation (InterPro:IPR014039), Nucleic acid-binding, OB-fold-like (InterPro:IPR016027), Translation elongation factor Ts, conserved site (InterPro:IPR018101), UBA-like (InterPro:IPR009060); BEST Arabidopsis thaliana protein match is: translation elongation factor Ts (EF-Ts), putative (TAIR:AT4G11120.1)
AT4G35440	*0,774*	CHLORIDE CHANNEL E (CLC-E)	Enclodes a choride channel protein that is localized to the thlakoid membrane.
AT5G03390	*0,774*		Protein of unknown function (DUF295); FUNCTIONS IN: molecular_function unknown; INVOLVED IN: biological_process unknown; LOCATED IN: chloroplast; EXPRESSED IN: 10 plant structures; EXPRESSED DURING: 7 growth stages; CONTAINS InterPro DOMAIN/s: Protein of unknown function DUF295 (InterPro:IPR005174); BEST Arabidopsis thaliana protein match is: Protein of unknown function (DUF295) (TAIR:AT1G68960.1)
AT1G09415	*0,773*	NIM1-INTERACTING 3 (NIMIN-3)	encodes a kinase that physically interacts with NPR1/NIM1
AT4G19530	*0,773*		Encodes a TIR-NB-LRR resistance protein. Transient expression in tobacco induces cell death.
AT1G78430	*0,772*	ROP INTERACTIVE PARTNER 4 (RIP4)	Encodes RIP4 (ROP interactive partner 4), a putative Rho protein effector, interacting specifically with the active form of ROPs (Rho proteins of plants).
AT5G65170	*0,772*		VQ motif-containing protein; CONTAINS InterPro DOMAIN/s: VQ (InterPro:IPR008889); BEST Arabidopsis thaliana protein match is: VQ motif-containing protein (TAIR:AT1G35830.1)

AT4G36580	*0,771*		AAA-type ATPase family protein; FUNCTIONS IN: ATPase activity, zinc ion binding; LOCATED IN: chloroplast; EXPRESSED IN: 14 plant structures; EXPRESSED DURING: 6 growth stages; CONTAINS InterPro DOMAIN/s: ATPase, AAA-type, core (InterPro:IPR003959), ATPase, AAA+ type, core (InterPro:IPR003593), ATPase, AAA-type, conserved site (InterPro:IPR003960), Protein of unknown function DUF3523 (InterPro:IPR021911); BEST Arabidopsis thaliana protein match is: AAA-type ATPase family protein (TAIR:AT2G18330.1)
AT1G31850	*0,77*		S-adenosyl-L-methionine-dependent methyltransferases superfamily protein; CONTAINS InterPro DOMAIN/s: Protein of unknown function DUF248, methyltransferase putative (InterPro:IPR004159); BEST Arabidopsis thaliana protein match is: S-adenosyl-L-methionine-dependent methyltransferases superfamily protein (TAIR:AT4G19120.2)
AT4G21280	*0,77*	PHOTOSYSTEM II SUBUNIT QA (PSBQA)	Encodes the PsbQ subunit of the oxygen evolving complex of photosystem II.
AT5G23910	*0,77*		ATP binding microtubule motor family protein; FUNCTIONS IN: microtubule motor activity, ATP binding; INVOLVED IN: microtubule-based movement; EXPRESSED IN: 14 plant structures; EXPRESSED DURING: 8 growth stages; CONTAINS InterPro DOMAIN/s: Kinesin, motor domain (InterPro:IPR001752); BEST Arabidopsis thaliana protein match is: ATP binding microtubule motor family protein (TAIR:AT5G02370.1)
AT3G24430	*0,769*	HIGH-CHLOROPHYLL-FLUORESCENCE 101 (HCF101)	Encodes chloroplast protein HCF101 (high chlorophyll fluorescence 101). Serves as a chloroplast scaffold protein that specifically assembles [4Fe-4S] clusters and transfers them to the chloroplast membrane and soluble target proteins.
AT3G55400	*0,768*	OVULE ABORTION 1 (OVA1)	OVULE ABORTION 1 (OVA1); FUNCTIONS IN: methionine-tRNA ligase activity, aminoacyl-tRNA ligase activity, ATP binding; INVOLVED IN: tRNA aminoacylation for protein translation, ovule development; LOCATED IN: mitochondrion, chloroplast; EXPRESSED IN: 23 plant structures; EXPRESSED DURING: 15 growth stages; CONTAINS InterPro DOMAIN/s: Rossmann-like alpha/beta/alpha sandwich fold (InterPro:IPR014729), Valyl/Leucyl/Isoleucyl-tRNA synthetase, class I, anticodon-binding (InterPro:IPR013155), Methionyl-tRNA synthetase, class Ia, N-terminal (InterPro:IPR014758), Aminoacyl-tRNA synthetase, class I (M) (InterPro:IPR015413), Methionyl-tRNA synthetase, class Ia (InterPro:IPR002304), Aminoacyl-tRNA synthetase, class 1a, anticodon-binding (InterPro:IPR009080); BEST Arabidopsis thaliana protein match is: methionine--tRNA ligase, putative / methionyl-tRNA synthetase, putative / MetRS, putative (TAIR:AT4G13780.1)
AT4G20430	*0,767*		Subtilase family protein; FUNCTIONS IN: identical protein binding, serine-type endopeptidase activity; INVOLVED IN: proteolysis, negative regulation of catalytic activity; LOCATED IN: endomembrane system; EXPRESSED IN: 19 plant structures; EXPRESSED DURING: 13 growth stages; CONTAINS InterPro DOMAIN/s: Peptidase S8/S53, subtilisin/kexin/sedolisin (InterPro:IPR000209), Peptidase S8, subtilisin-related (InterPro:IPR015500), Peptidase S8/S53, subtilisin, active site (InterPro:IPR022398), Proteinase inhibitor I9, subtilisin propeptide (InterPro:IPR010259); BEST Arabidopsis thaliana protein match is: Subtilase family protein (TAIR:AT5G44530.1)
AT4G04640	*0,766*	(ATPC1)	One of two genes (with ATPC2) encoding the gamma subunit of Arabidopsis chloroplast ATP synthase.
AT5G07240	*0,766*	IQ-DOMAIN 24 (IQD24)	IQ-domain 24 (IQD24); CONTAINS InterPro DOMAIN/s: IQ calmodulin-binding region (InterPro:IPR000048); BEST Arabidopsis thaliana protein match is: IQ-domain 23 (TAIR:AT5G62070.1)
AT2G16380	*0,765*		Sec14p-like phosphatidylinositol transfer family protein; FUNCTIONS IN: transporter activity; INVOLVED IN: transport; LOCATED IN: intracellular; EXPRESSED IN: 10 plant structures; EXPRESSED DURING: 7 growth stages; CONTAINS InterPro DOMAIN/s: Cellular retinaldehyde-binding/triple function, C-terminal (InterPro:IPR001251), Cellular retinaldehyde-binding/triple function, N-terminal (InterPro:IPR008273), Cellular retinaldehyde binding/alpha-tocopherol transport (InterPro:IPR001071), Phosphatidylinositol transfer protein-like, N-terminal (InterPro:IPR011074); BEST Arabidopsis thaliana protein match is: Sec14p-like phosphatidylinositol transfer family protein (TAIR:AT4G34580.1)
AT5G01530	*0,764*	LIGHT HARVESTING COMPLEX PHOTOSYSTEM II (LHCB4.1)	light harvesting complex photosystem II (LHCB4.1); FUNCTIONS IN: chlorophyll binding; INVOLVED IN: response to blue light, response to red light, response to far red light, photosynthesis; LOCATED IN: in 6 components; EXPRESSED IN: 27 plant structures; EXPRESSED DURING: 14 growth stages; CONTAINS InterPro DOMAIN/s: Chlorophyll A-B binding protein (InterPro:IPR001344); BEST Arabidopsis thaliana protein match is: light harvesting complex photosystem II (TAIR:AT3G08940.2)

AT5G28500	*0,764*		unknown protein; FUNCTIONS IN: molecular_function unknown; INVOLVED IN: biological_process unknown; LOCATED IN: chloroplast stroma, chloroplast; EXPRESSED IN: 23 plant structures; EXPRESSED DURING: 13 growth stages; BEST Arabidopsis thaliana protein match is: unknown protein (TAIR:AT3G04550.1)
AT3G27690	*0,763*	PHOTOSYSTEM II LIGHT HARVESTING COMPLEX GENE 2.3 (LHCB2.3)	Encodes Lhcb2.4. Belongs to the Lhc super-gene family encodes the light-harvesting chlorophyll a/b-binding (LHC) proteins that constitute the antenna system of the photosynthetic apparatus. The mRNA is cell-to-cell mobile.
AT3G52750	*0,763*	(FTSZ2-2)	Nuclear gene that encodes a plastidial division protein (FtsZ2-2). FtsZ2-2 is involved in chloroplast morphology and internal organisation in addition to participating in chloroplast partition
AT4G04850	*0,76*	K+ EFFLUX ANTIPORTER 3 (KEA3)	Encodes a potassium efflux antiporter; has three splice forms KEA3.1, KEA3.2, and KEA3.3, KEA3.2 is the most abundant splice form in all plant organs (silique, flower, leaf and root). KEA3.1 and KEA3.3 are minor variants that can be found in flowers and in leaves. KEA3 is localized to the thylakoid membrane and enriched in the stromal lamellae. It allows proton efflux from the thylakoid lumen by proton/potassium antiport.
AT5G09590	*0,76*	MITOCHONDRIAL HSO70 2 (MTHSC70-2)	heat shock protein 70 (Hsc70-5); nuclear
AT5G57710	*0,76*	SUPPRESSOR OF MAX2 1 (SMAX1)	SMAX1 (SUPPRESSOR OF MAX2 1) is a member of an eight-gene family in Arabidopsis that has weak similarity to AtHSP101, a ClpB chaperonin required for thermotolerance. SMAX1 is an important component of KAR/SL signaling during seed germination and seedling growth, but is not necessary for all MAX2-dependent responses. The mRNA is cell-to-cell mobile.
AT1G29550	*0,759*	(EIF4E1B)	Eukaryotic initiation factor 4E protein; FUNCTIONS IN: RNA binding, translation initiation factor activity; INVOLVED IN: translational initiation; LOCATED IN: cytoplasm; EXPRESSED IN: male gametophyte, pollen tube; EXPRESSED DURING: L mature pollen stage, M germinated pollen stage; CONTAINS InterPro DOMAIN/s: Eukaryotic translation initiation factor 4E (eIF-4E) (InterPro:IPR001040), Eukaryotic translation initiation factor 4E (eIF-4E), conserved site (InterPro:IPR019770); BEST Arabidopsis thaliana protein match is: Eukaryotic initiation factor 4E protein (TAIR:AT1G29590.1)
AT1G68790	*0,759*	LITTLE NUCLEI3 (LINC3)	LITTLE NUCLEI3 (LINC3); INVOLVED IN: biological_process unknown; LOCATED IN: nucleolus; EXPRESSED IN: 23 plant structures; EXPRESSED DURING: 13 growth stages; BEST Arabidopsis thaliana protein match is: nuclear matrix constituent protein-related (TAIR:AT1G13220.2)
AT2G41290	*0,759*	STRICTOSIDINE SYNTHASE-LIKE 2 (SSL2)	Although this enzyme is predicted to encode a strictosidine synthase (SS), it lacks a conserved catalytic glutamate residue found in active SS enzymes and it is not expected to have SS activity.
AT4G03550	*0,759*	GLUCAN SYNTHASE-LIKE 5 (GSL05)	Encodes a callose synthase that is required for wound and papillary callose formation in response to fungal pathogens Erysiphe and Blumeria. Mutants are resistant to P. parasitica and exhibit an exaggerated PR1 response.Contributes to PAMP-induced basal defense. The mRNA is cell-to-cell mobile.
AT2G22500	*0,758*	UNCOUPLING PROTEIN 5	Encodes one of the mitochondrial dicarboxylate carriers (DIC): DIC1 (AT2G22500), DIC2 (AT4G24570), DIC3 (AT5G09470).
AT3G10270	*0,758*	DNA GYRASE B1 (GYRB1)	Protein targeting to mitochondria is influenced by UTR sequences.
AT5G50720	*0,758*	HVA22 HOMOLOGUE E (HVA22E)	Encodes one of five HVA22 homologs in Arabidopsis. HVA22 is an ABA- and stress-inducible gene first isolated from barley. Members of this gene family have only been found in eukaryotes. AtHVA22e mRNA is upregulated to varying degrees in response to cold stress, salt stress, ABA treatment or dehydration.
AT2G47010	*0,757*		unknown protein; FUNCTIONS IN: molecular_function unknown; INVOLVED IN: biological_process unknown; LOCATED IN: endomembrane system; EXPRESSED IN: 17 plant structures; EXPRESSED DURING: 10 growth stages; BEST Arabidopsis thaliana protein match is: unknown protein (TAIR:AT1G17030.1)
AT2G29650	*0,756*	PHOSPHATE TRANSPORTER 4;1 (PHT4;1)	Encodes an inorganic phosphate transporter (PHT4;1) that is localized to the thylakoid membrane.
AT5G53420	*0,756*		CCT motif family protein; FUNCTIONS IN: molecular_function unknown; INVOLVED IN: biological_process unknown; LOCATED IN: cellular_component unknown; EXPRESSED IN: 23 plant structures; EXPRESSED DURING: 13 growth stages; CONTAINS InterPro DOMAIN/s: CCT domain (InterPro:IPR010402); BEST Arabidopsis thaliana protein match is: CCT motif family protein (TAIR:AT4G27900.2)

AT5G67200	*0,755*		Leucine-rich repeat protein kinase family protein; FUNCTIONS IN: protein serine/threonine kinase activity, protein kinase activity, ATP binding; INVOLVED IN: transmembrane receptor protein tyrosine kinase signaling pathway, protein amino acid phosphorylation; LOCATED IN: plasma membrane; EXPRESSED IN: 22 plant structures; EXPRESSED DURING: 10 growth stages; CONTAINS InterPro DOMAIN/s: Protein kinase, catalytic domain (InterPro:IPR000719), Leucine-rich repeat (InterPro:IPR001611), Serine/threonine-protein kinase-like domain (InterPro:IPR017442), Protein kinase-like domain (InterPro:IPR011009); BEST Arabidopsis thaliana protein match is: Leucine-rich repeat protein kinase family protein (TAIR:AT3G50230.1)
AT1G65230	*0,754*		Uncharacterized conserved protein (DUF2358); FUNCTIONS IN: molecular_function unknown; INVOLVED IN: biological_process unknown; LOCATED IN: chloroplast; EXPRESSED IN: 21 plant structures; EXPRESSED DURING: 13 growth stages; CONTAINS InterPro DOMAIN/s: Protein of unknown function DUF2358 (InterPro:IPR018790)
AT2G45460	*0,754*		SMAD/FHA domain-containing protein ; LOCATED IN: chloroplast; EXPRESSED IN: 22 plant structures; EXPRESSED DURING: 12 growth stages; CONTAINS InterPro DOMAIN/s: SMAD/FHA domain (InterPro:IPR008984), Forkhead-associated (FHA) domain (InterPro:IPR000253).
AT4G34290	*0,754*		SWIB/MDM2 domain superfamily protein; FUNCTIONS IN: molecular_function unknown; INVOLVED IN: biological_process unknown; LOCATED IN: chloroplast; EXPRESSED IN: 21 plant structures; EXPRESSED DURING: 13 growth stages; CONTAINS InterPro DOMAIN/s: SWIB/MDM2 domain (InterPro:IPR003121), SWIB domain (InterPro:IPR019835); BEST Arabidopsis thaliana protein match is: SWIB/MDM2 domain superfamily protein (TAIR:AT2G14880.1)
AT5G26570	*0,754*	PHOSPHOGLUCAN WATER DIKINASE (PWD)	chloroplastidic phosphoglucan, water dikinase (PWD) which is required for normal degradation of leaf starch in Arabidopsis. NMR analysis of the mutants, suggests that the gene is specifically involved in the phosphorylation of the glucosyl residues of starch at the C3 position.
AT5G43470	*0,754*	RECOGNITION OF PERONOSPORA PARASITICA 8 (RPP8)	Confers resistance to Peronospora parasitica. In arabidopsis ecotype Dijon-17, HRT-mediated signaling is dependent on light for the induction of hypersensitive response and resistance to turnip crinkle virus.
AT1G54200	*0,753*		unknown protein; BEST Arabidopsis thaliana protein match is: unknown protein (TAIR:AT3G13980.1)
AT4G31390	*0,752*	ABC1-LIKE KINASE RELATED TO CHLOROPHYLL DEGRADATION AND OXIDATIVE STRESS 1 (ACDO1)	Protein kinase superfamily protein; FUNCTIONS IN: protein kinase activity, ATP binding; INVOLVED IN: protein amino acid phosphorylation; LOCATED IN: chloroplast, plastoglobule; EXPRESSED IN: 22 plant structures; EXPRESSED DURING: 13 growth stages; CONTAINS InterPro DOMAIN/s: ABC-1 (InterPro:IPR004147), Protein kinase, catalytic domain (InterPro:IPR000719), Protein kinase-like domain (InterPro:IPR011009); BEST Arabidopsis thaliana protein match is: Protein kinase superfamily protein (TAIR:AT1G79600.1)
AT3G20330	*0,751*	PYRIMIDINE B (PYRB)	encodes aspartate carbamoyltransferase catalyzing the second step in the de novo pyrimidine ribonucleotide biosynthesis
AT3G63250	*0,751*	HOMOCYSTEINE METHYLTRANSFERASE 2 (HMT2)	Encodes a homocysteine methyltransferase (HMT). Among the three HMT coding genes in the genome, HMT2 is responsible for a significant proportion of HMT activity in the flower stalks and silique hulls. However, HMT2 does not significantly contribute to the total HMT activity in seeds.
AT2G21860	*0,75*		violaxanthin de-epoxidase-related; BEST Arabidopsis thaliana protein match is: non-photochemical quenching 1 (TAIR:AT1G08550.2)
AT5G08350	*0,75*		GRAM domain-containing protein / ABA-responsive protein-related; CONTAINS InterPro DOMAIN/s: GRAM (InterPro:IPR004182); BEST Arabidopsis thaliana protein match is: GRAM domain-containing protein / ABA-responsive protein-related (TAIR:AT5G23370.1)
AT5G11060	*0,75*	KNOTTED1-LIKE HOMEOBOX GENE 4 (KNAT4)	A member of Class II KN1-like homeodomain transcription factors (together with KNAT3 and KNAT5), with greatest homology to the maize knox1 homeobox protein. Expression regulated by light. Detected in all tissues examined, but most prominent in leaves and young siliques. Transient expression of GFP translational fusion protein suggests bipartite localization in nucleus and cytoplasm. KNAT4 promoter activity showed cell-type specific pattern along longitudinal root axis; GUS expression pattern started at the elongation zone, predominantly in the phloem and pericycle cells, extending to endodermis toward the base of the root.

AT5G66570	*0,749*	PS II OXYGEN-EVOLVING COMPLEX 1 (PSBO1)	Encodes a protein which is an extrinsic subunit of photosystem II and which has been proposed to play a central role in stabilization of the catalytic manganese cluster. In <i>Arabidopsis thaliana</i> the PsbO proteins are encoded by two genes: <i>psbO1</i> and <i>psbO2</i>. PsbO1 is the major isoform in the wild-type. In plsp1-1 mutant plastids, the nonmature form of the protein localizes in the membrane. The mRNA is cell-to-cell mobile.
AT1G26150	*0,748*	PROLINE-RICH EXTENSIN-LIKE RECEPTOR KINASE 10 (PERK10)	Encodes a member of the proline-rich extensin-like receptor kinase (PERK) family. This family consists of 15 predicted receptor kinases (PMID: 15653807).
AT3G42670	*0,748*	CHROMATIN REMODELING 38 (CHR38)	Encodes a nuclear localized SNF domain containing protein involved in RNA silencing. Mutants were identified in a screen for defects in the spread of RNA silencing. CLSY1 may affect production of dsRNA from the locus to be silenced.
AT1G26090	*0,747*		P-loop containing nucleoside triphosphate hydrolases superfamily protein; LOCATED IN: chloroplast, plastoglobule; EXPRESSED IN: 22 plant structures; EXPRESSED DURING: 13 growth stages
AT1G52220	*0,745*	CURVATURE THYLAKOID 1C (CURT1C)	FUNCTIONS IN: molecular_function unknown; INVOLVED IN: biological_process unknown; LOCATED IN: chloroplast thylakoid membrane, chloroplast; EXPRESSED IN: 23 plant structures; EXPRESSED DURING: 13 growth stages; BEST Arabidopsis thaliana protein match is: photosystem I P subunit (TAIR:AT2G46820.2)
AT1G79280	*0,745*	NUCLEAR PORE ANCHOR (NUA)	Encodes a 237-kDA protein with similarity to vertebrate Tpr, a long coiled-coil proteins of nuclear pore inner basket filaments. It is localized to the inner surface of the nuclear envelope and is a component of the nuclear pore-associated steps of sumoylation and mRNA export in plants. Mutations affect flowering time regulation and other developmental processes. Probably acts in the same pathway as ESD4 in affecting flowering time, vegetative and inflorescence development.
AT3G10050	*0,745*	L-O-METHYLTHREONINE RESISTANT 1 (OMR1)	first enzyme in the biosynthetic pathway of isoleucine
AT4G17550	*0,744*	GLYCEROL-3-PHOSPHATE PERMEASE 4 (G3Pp4)	Encodes a member of the phosphate starvation-induced glycerol-3-phosphate permease gene family: AT3G47420(G3Pp1), AT4G25220(G3Pp2), AT1G30560(G3Pp3), AT4G17550(G3Pp4) and AT2G13100(G3Pp5).
AT1G03310	*0,743*	DEBRANCHING ENZYME 1 (DBE1)	Encodes a protein with strong similarity to isoamylase (EC:3.2.1.68) however lacks critical residues known to be important for activity. Appears to co localize with ISA1 in the chloroplast isoamylase complex. Mutations in this gene cause the loss of detectable isoamylase activity and the disruption of normal starch structure. It has been postulated that AtISA2 interacts with AtISA1 to form the Iso1 complex.
AT4G26270	*0,743*	PHOSPHOFRUCTOKINASE 3 (PFK3)	phosphofructokinase 3 (PFK3); CONTAINS InterPro DOMAIN/s: Pyrophosphate-dependent phosphofructokinase TP0108 (InterPro:IPR012004), Phosphofructokinase (InterPro:IPR000023); BEST Arabidopsis thaliana protein match is: phosphofructokinase 7 (TAIR:AT5G56630.1)
AT5G39850	*0,743*		Ribosomal protein S4; FUNCTIONS IN: structural constituent of ribosome; INVOLVED IN: translation; LOCATED IN: cytosolic small ribosomal subunit, membrane; EXPRESSED IN: 22 plant structures; EXPRESSED DURING: 13 growth stages; CONTAINS InterPro DOMAIN/s: Ribosomal protein S4 (InterPro:IPR001912), Ribosomal protein S4, conserved site (InterPro:IPR018079), Ribosomal protein S4/S9, eukaryotic/archaeal (InterPro:IPR005710), RNA-binding S4 (InterPro:IPR002942); BEST Arabidopsis thaliana protein match is: Ribosomal protein S4 (TAIR:AT5G15200.1)
AT2G43800	*0,742*		Actin-binding FH2 (formin homology 2) family protein; FUNCTIONS IN: actin binding; INVOLVED IN: cellular component organization, actin cytoskeleton organization; LOCATED IN: plasma membrane; EXPRESSED IN: 21 plant structures; EXPRESSED DURING: 13 growth stages; CONTAINS InterPro DOMAIN/s: Actin-binding FH2/DRF autoregulatory (InterPro:IPR003104), Actin-binding FH2 (InterPro:IPR015425); BEST Arabidopsis thaliana protein match is: formin homology 1 (TAIR:AT3G25500.1)
AT2G29550	*0,74*	TUBULIN BETA-7 CHAIN (TUB7)	Encodes a beta-tubulin that is expressed in leaves, roots and flowers.
AT5G66210	*0,74*	CALCIUM-DEPENDENT PROTEIN KINASE 28 (CPK28)	member of Calcium Dependent Protein Kinase
AT2G29720	*0,739*	(CTF2B)	Encodes CTF2B.
AT3G03020	*0,739*		unknown protein; FUNCTIONS IN: molecular_function unknown; INVOLVED IN: biological_process unknown; LOCATED IN: chloroplast; EXPRESSED IN: 21 plant structures; EXPRESSED DURING: 13 growth stages

AT4G13990	*0,739*		Exostosin family protein; FUNCTIONS IN: catalytic activity; INVOLVED IN: biological_process unknown; LOCATED IN: cell wall, membrane; EXPRESSED IN: 18 plant structures; EXPRESSED DURING: 7 growth stages; CONTAINS InterPro DOMAIN/s: Exostosin-like (InterPro:IPR004263); BEST Arabidopsis thaliana protein match is: Exostosin family protein (TAIR:AT2G20370.1)
AT5G25930	*0,739*		Protein kinase family protein with leucine-rich repeat domain; FUNCTIONS IN: kinase activity; INVOLVED IN: protein amino acid phosphorylation; LOCATED IN: plasma membrane, membrane; EXPRESSED IN: 23 plant structures; EXPRESSED DURING: 14 growth stages; CONTAINS InterPro DOMAIN/s: Protein kinase, ATP binding site (InterPro:IPR017441), Protein kinase, catalytic domain (InterPro:IPR000719), Leucine-rich repeat-containing N-terminal domain, type 2 (InterPro:IPR013210), Leucine-rich repeat (InterPro:IPR001611), Serine/threonine-protein kinase-like domain (InterPro:IPR017442), Protein kinase-like domain (InterPro:IPR011009), Serine/threonine-protein kinase, active site (InterPro:IPR008271); BEST Arabidopsis thaliana protein match is: receptor like protein 52 (TAIR:AT5G25910.1)
AT1G71720	*0,738*	PIGMENT DEFECTIVE 338 (PDE338)	Encodes a chloroplast localized protein that regulates the translation of Ycf1 by binding to its mRNA. It is involved in the biogenesis of photosynthetic complexes.
AT1G77060	*0,738*		Phosphoenolpyruvate carboxylase family protein; FUNCTIONS IN: catalytic activity; INVOLVED IN: metabolic process; LOCATED IN: chloroplast, chloroplast stroma; EXPRESSED IN: 22 plant structures; EXPRESSED DURING: 13 growth stages; CONTAINS InterPro DOMAIN/s: Pyruvate/Phosphoenolpyruvate kinase, catalytic core (InterPro:IPR015813), Isocitrate lyase/phosphorylmutase, conserved site (InterPro:IPR018523), Isocitrate lyase/phosphorylmutase (InterPro:IPR000918); BEST Arabidopsis thaliana protein match is: Phosphoenolpyruvate carboxylase family protein (TAIR:AT1G21440.1)
AT2G40280	*0,738*		S-adenosyl-L-methionine-dependent methyltransferases superfamily protein; CONTAINS InterPro DOMAIN/s: Protein of unknown function DUF248, methyltransferase putative (InterPro:IPR004159); BEST Arabidopsis thaliana protein match is: S-adenosyl-L-methionine-dependent methyltransferases superfamily protein (TAIR:AT3G56080.1)
AT5G51050	*0,738*	ATP/PHOSPHATE CARRIER 2 (APC2)	Encodes an APC isoform in Arabidopsis, a calcium-dependent mitochondrial ATP-Mg/Pi transporter.
AT1G12800	*0,737*	S1 DOMAIN-CONTAINING RBP (SDP)	SDP is a chloroplast localized RNA binding protein that is required for plastid rRNA processing. Plants harboring a mutation in SDP have numerous defects including reduced chlorophyll content, poor growth, yellow leaves and abnormal chloroplasts.
AT1G15940	*0,737*	(PDS5E)	One of 5 PO76/PDS5 cohesion cofactor orthologs of Arabidopsis.
AT1G64180	*0,737*		intracellular protein transport protein USO1-related; FUNCTIONS IN: molecular_function unknown; INVOLVED IN: biological_process unknown; LOCATED IN: plasma membrane; EXPRESSED IN: 14 plant structures; EXPRESSED DURING: 9 growth stages; BEST Arabidopsis thaliana protein match is: unknown protein (TAIR:AT5G41620.1)
AT1G31970	*0,736*	STRESS RESPONSE SUPPRESSOR 1 (STRS1)	STRESS RESPONSE SUPPRESSOR 1 (STRS1); FUNCTIONS IN: helicase activity, nucleic acid binding, ATP-dependent helicase activity, ATP binding; EXPRESSED IN: 24 plant structures; EXPRESSED DURING: 13 growth stages; CONTAINS InterPro DOMAIN/s: RNA helicase, DEAD-box type, Q motif (InterPro:IPR014014), DNA/RNA helicase, DEAD/DEAH box type, N-terminal (InterPro:IPR011545), RNA helicase, ATP-dependent, DEAD-box, conserved site (InterPro:IPR000629), DEAD-like helicase, N-terminal (InterPro:IPR014001), DNA/RNA helicase, C-terminal (InterPro:IPR001650), Helicase, superfamily 1/2, ATP-binding domain (InterPro:IPR014021); BEST Arabidopsis thaliana protein match is: P-loop containing nucleoside triphosphate hydrolases superfamily protein (TAIR:AT5G63120.2)
AT1G55670	*0,735*	PHOTOSYSTEM I SUBUNIT G (PSAG)	Encodes subunit G of photosystem I, an 11-kDa membrane protein that plays an important role in electron transport between plastocyanin and PSI and is involved in the stability of the PSI complex. PSI-G subunit is bound to PSI-B and is in contact with Lhca1. The protein inserts into thylakoids by a direct or "spontaneous" pathway that does not involve the activities of any known chloroplast protein-targeting machinery. PSI-G appears to be directly or indirectly involved in the interaction between Photosystem I and plastocyanin.
AT2G30570	*0,733*	PHOTOSYSTEM II REACTION CENTER W (PSBW)	Encodes PsbW, a protein similar to photosystem II reaction center subunit W. Loss of PsbW destabilizes the supramolecular organization of PSII.
AT4G12130	*0,733*		Encodes a mitochondrial COG0354 protein that requires folate for its function in Fe/S cluster biogenesis.

AT5G52450	*0,733*		MATE efflux family protein; FUNCTIONS IN: antiporter activity, drug transmembrane transporter activity, transporter activity; INVOLVED IN: response to nematode; LOCATED IN: plasma membrane, membrane; EXPRESSED IN: 24 plant structures; EXPRESSED DURING: 15 growth stages; CONTAINS InterPro DOMAIN/s: MATE family transporter related protein (InterPro:IPR015521), Multi antimicrobial extrusion protein MatE (InterPro:IPR002528); BEST Arabidopsis thaliana protein match is: MATE efflux family protein (TAIR:AT2G34360.1)
AT1G23380	*0,732*	KNOTTED1-LIKE HOMEOBOX GENE 6 (KNAT6)	homeodomain transcription factor KNAT6, belonging to class I of KN transcription factor family (which also includes KNAT1 and KNAT2). Expression is increased in as and bop1 leaf mutants.
AT2G47550	*0,731*		Plant invertase/pectin methylesterase inhibitor superfamily; FUNCTIONS IN: enzyme inhibitor activity, pectinesterase activity; INVOLVED IN: cell wall modification; LOCATED IN: endomembrane system, cell wall, plant-type cell wall; EXPRESSED IN: 20 plant structures; EXPRESSED DURING: 9 growth stages; CONTAINS InterPro DOMAIN/s: Pectinesterase, active site (InterPro:IPR018040), Pectin lyase fold/virulence factor (InterPro:IPR011050), Pectinesterase, catalytic (InterPro:IPR000070), Pectinesterase inhibitor (InterPro:IPR006501), Pectin lyase fold (InterPro:IPR012334); BEST Arabidopsis thaliana protein match is: Plant invertase/pectin methylesterase inhibitor superfamily (TAIR:AT4G02330.1)
AT4G38710	*0,73*		glycine-rich protein; FUNCTIONS IN: translation initiation factor activity; INVOLVED IN: biological_process unknown; LOCATED IN: cellular_component unknown; EXPRESSED IN: 23 plant structures; EXPRESSED DURING: 14 growth stages; CONTAINS InterPro DOMAIN/s: Plant specific eukaryotic initiation factor 4B (InterPro:IPR010433); BEST Arabidopsis thaliana protein match is: eukaryotic translation initiation factor 4B1 (TAIR:AT3G26400.1).
AT5G45170	*0,73*		Haloacid dehalogenase-like hydrolase (HAD) superfamily protein; FUNCTIONS IN: molecular_function unknown; INVOLVED IN: biological_process unknown; LOCATED IN: chloroplast stroma, chloroplast; EXPRESSED IN: 23 plant structures; EXPRESSED DURING: 14 growth stages; BEST Arabidopsis thaliana protein match is: Haloacid dehalogenase-like hydrolase (HAD) superfamily protein (TAIR:AT3G48420.1)
AT2G33400	*0,729*		unknown protein; BEST Arabidopsis thaliana protein match is: unknown protein (TAIR:AT1G04030.1)
AT4G13110	*0,729*		BSD domain-containing protein; FUNCTIONS IN: molecular_function unknown; INVOLVED IN: biological_process unknown; LOCATED IN: chloroplast; CONTAINS InterPro DOMAIN/s: BSD (InterPro:IPR005607); BEST Arabidopsis thaliana protein match is: BSD domain-containing protein (TAIR:AT1G03350.1)
AT5G19090	*0,729*		Heavy metal transport/detoxification superfamily protein ; FUNCTIONS IN: metal ion binding; INVOLVED IN: metal ion transport; LOCATED IN: cellular_component unknown; EXPRESSED IN: 22 plant structures; EXPRESSED DURING: 13 growth stages; CONTAINS InterPro DOMAIN/s: Heavy metal transport/detoxification protein (InterPro:IPR006121); BEST Arabidopsis thaliana protein match is: Heavy metal transport/detoxification superfamily protein (TAIR:AT3G06130.2)
AT5G23570	*0,729*	SUPPRESSOR OF GENE SILENCING 3 (SGS3)	Required for posttranscriptional gene silencing and natural virus resistance.SGS3 is a member of an 'unknown' protein family. Members of this family have predicted coiled coiled domains suggesting oligomerization and a potential zinc finger domain. Involved in the production of trans-acting siRNAs, through direct or indirect stabilization of cleavage fragments of the primary ta-siRNA transcript. Acts before RDR6 in this pathway. The mRNA is cell-to-cell mobile.
AT1G27460	*0,728*	NO POLLEN GERMINATION RELATED 1 (NPGR1)	encodes a calmodulin-binding protein that is expressed in pollen, suspension culture cells, flowers, and fruits. The mRNA is cell-to-cell mobile.
AT1G10760	*0,727*	STARCH EXCESS 1 (SEX1)	Encodes an α-glucan, water dikinase required for starch degradation. Involved in cold-induced freezing tolerance. Mutations that eliminate the GWD protein or affect the dikinase domain of the enzyme dramatically reduce both the amount of phosphate in the amylopectin and the rate of starch degradation. Mature leaves of these mutants accumulate amounts of starch up to seven times greater than those in wild-type leaves. NMR analysis of the mutants, suggests that the gene is specifically involved in the phosphorylation of the glucosyl residues of starch at the C6 position.
AT5G14180	*0,727*	MYZUS PERSICAE-INDUCED LIPASE 1 (MPL1)	Myzus persicae-induced lipase 1 (MPL1); FUNCTIONS IN: catalytic activity; INVOLVED IN: glycerol biosynthetic process, lipid metabolic process; LOCATED IN: endomembrane system; EXPRESSED IN: 7 plant structures; EXPRESSED DURING: L mature pollen stage, M germinated pollen stage, 4 anthesis, petal differentiation and expansion stage; CONTAINS InterPro DOMAIN/s: AB-hydrolase-associated lipase region (InterPro:IPR006693), Alpha/beta hydrolase fold-1 (InterPro:IPR000073); BEST Arabidopsis thaliana protein match is: lipase 1 (TAIR:AT2G15230.1)

AT1G70250	*0,726*		Encodes a Protease inhibitor/seed storage/LTP family protein
AT3G21055	*0,724*	PHOTOSYSTEM II SUBUNIT T (PSBTN)	Encodes photosystem II 5 kD protein subunit PSII-T. This is a nuclear-encoded gene (PsbTn) which also has a plastid-encoded paralog (PsbTc).
AT4G02630	*0,724*		Protein kinase superfamily protein; FUNCTIONS IN: protein serine/threonine kinase activity, protein kinase activity, kinase activity, ATP binding; INVOLVED IN: protein amino acid phosphorylation; LOCATED IN: plasma membrane; EXPRESSED IN: 22 plant structures; EXPRESSED DURING: 13 growth stages; CONTAINS InterPro DOMAIN/s: Protein kinase, ATP binding site (InterPro:IPR017441), Protein kinase, catalytic domain (InterPro:IPR000719), Serine/threonine-protein kinase-like domain (InterPro:IPR017442), Protein kinase-like domain (InterPro:IPR011009), Serine/threonine-protein kinase, active site (InterPro:IPR008271); BEST Arabidopsis thaliana protein match is: Protein kinase superfamily protein (TAIR:AT1G01540.2)
AT1G80530	*0,723*		Major facilitator superfamily protein; FUNCTIONS IN: molecular_function unknown; INVOLVED IN: response to karrikin; LOCATED IN: nucleus; EXPRESSED IN: 23 plant structures; EXPRESSED DURING: 13 growth stages; CONTAINS InterPro DOMAIN/s: Nodulin-like (InterPro:IPR010658), Major facilitator superfamily, general substrate transporter (InterPro:IPR016196); BEST Arabidopsis thaliana protein match is: Major facilitator superfamily protein (TAIR:AT4G34950.1)
AT2G44450	*0,723*	BETA GLUCOSIDASE 15 (BGLU15)	beta glucosidase 15 (BGLU15); FUNCTIONS IN: cation binding, hydrolase activity, hydrolyzing O-glycosyl compounds, catalytic activity; INVOLVED IN: carbohydrate metabolic process; LOCATED IN: cell wall, plant-type cell wall; EXPRESSED IN: 23 plant structures; EXPRESSED DURING: 14 growth stages; CONTAINS InterPro DOMAIN/s: Glycoside hydrolase, family 1 (InterPro:IPR001360), Glycoside hydrolase, family 1, active site (InterPro:IPR018120), Glycoside hydrolase, catalytic core (InterPro:IPR017853), Glycoside hydrolase, subgroup, catalytic core (InterPro:IPR013781); BEST Arabidopsis thaliana protein match is: beta glucosidase 13 (TAIR:AT5G44640.1)
AT5G10080	*0,722*		Eukaryotic aspartyl protease family protein; FUNCTIONS IN: aspartic-type endopeptidase activity; INVOLVED IN: proteolysis; LOCATED IN: anchored to plasma membrane, anchored to membrane; EXPRESSED IN: cultured cell; CONTAINS InterPro DOMAIN/s: Peptidase aspartic (InterPro:IPR021109), Peptidase aspartic, catalytic (InterPro:IPR009007), Peptidase A1 (InterPro:IPR001461), Peptidase aspartic, active site (InterPro:IPR001969); BEST Arabidopsis thaliana protein match is: Eukaryotic aspartyl protease family protein (TAIR:AT4G35880.1)
AT1G64080	*0,721*	MEMBRANE-ASSOCIATED KINASE REGULATOR 2 (MAKR2)	Encodes a member of the MAKR (MEMBRANE-ASSOCIATED KINASE REGULATOR) gene family. MAKRs have putative kinase interacting motifs and membrane localization signals. Known members include: AT5G26230 (MAKR1), AT1G64080 (MAKR2), AT2G37380 (MAKR3), AT2G39370 (MAKR4), AT5G52870 (MAKR5) and AT5G52900 (MAKR6).
AT4G31110	*0,721*		Wall-associated kinase family protein; FUNCTIONS IN: kinase activity; INVOLVED IN: protein amino acid phosphorylation; LOCATED IN: plasma membrane; CONTAINS InterPro DOMAIN/s: Wall-associated kinase (InterPro:IPR013695), EGF-like calcium-binding, conserved site (InterPro:IPR018097), Protein kinase, catalytic domain (InterPro:IPR000719), EGF calcium-binding (InterPro:IPR013091), Serine-threonine/tyrosine-protein kinase (InterPro:IPR001245), Protein kinase-like domain (InterPro:IPR011009), Serine/threonine-protein kinase, active site (InterPro:IPR008271); BEST Arabidopsis thaliana protein match is: wall-associated kinase, putative (TAIR:AT4G31100.1)
AT1G06000	*0,72*	(UGT89C1)	encodes a flavonol-7-O-rhamnosyltransferase involved in the formation of rhamnosylated flavonols
AT1G44790	*0,72*		ChaC-like family protein; CONTAINS InterPro DOMAIN/s: ChaC-like protein (InterPro:IPR006840); BEST Arabidopsis thaliana protein match is: ChaC-like family protein (TAIR:AT4G31290.1)
AT2G25620	*0,72*	DNA-BINDING PROTEIN PHOSPHATASE 1 (DBP1)	Encodes DBP1, a member of the DBP factors (DNA-binding protein phosphatases) featuring sequence-specific DNA-binding and protein phosphatase activity. DBP1 is involved in plant-potyvirus interactions. Loss-of-function of DBP1 renders resistance to potyviruses.
AT5G57590	*0,72*	BIOTIN AUXOTROPH 1 (BIO1)	Encodes a bifunctional enzyme with both dethiobiotin synthetase and diaminopelargonic acid aminotransferase activities that is involved in biotin synthesis.
AT5G67300	*0,72*	MYB DOMAIN PROTEIN R1 (MYBR1)	Member of the R2R3 factor MYB gene family involved in mediating plant responses to a variety of abiotic stimiuli. The mRNA is cell-to-cell mobile.
AT1G09480	*0,719*		similar to Eucalyptus gunnii alcohol dehydrogenase of unknown physiological function (GI:1143445), Vigna unguiculata (gi:1854445), NOT a cinnamyl-alcohol dehydrogenase The mRNA is cell-to-cell mobile.

AT5G07920	*0,719*	DIACYLGLYCEROL KINASE1 (DGK1)	diacylglycerol kinase
AT2G44280	*0,718*		Major facilitator superfamily protein; CONTAINS InterPro DOMAIN/s: Major facilitator superfamily, general substrate transporter (InterPro:IPR016196); BEST Arabidopsis thaliana protein match is: Major facilitator superfamily protein (TAIR:AT3G60070.1)
AT2G36850	*0,717*	GLUCAN SYNTHASE-LIKE 8 (GSL8)	Encodes GSL8, a member of the Glucan Synthase-Like (GSL) family believed to be involved in the synthesis of the cell wall component callose. GSL8 is required for male gametophyte development and plant growth. Has a role in entry of microspores into mitosis. Also refer to GSL10 (At3g07160).
AT5G42720	*0,717*		Glycosyl hydrolase family 17 protein; FUNCTIONS IN: cation binding, hydrolase activity, hydrolyzing O-glycosyl compounds, catalytic activity; INVOLVED IN: carbohydrate metabolic process; LOCATED IN: anchored to membrane; EXPRESSED IN: 22 plant structures; EXPRESSED DURING: 13 growth stages; CONTAINS InterPro DOMAIN/s: Glycoside hydrolase, catalytic core (InterPro:IPR017853), Glycoside hydrolase, family 17 (InterPro:IPR000490), Glycoside hydrolase, subgroup, catalytic core (InterPro:IPR013781); BEST Arabidopsis thaliana protein match is: O-Glycosyl hydrolases family 17 protein (TAIR:AT2G16230.1)
AT2G34300	*0,716*		S-adenosyl-L-methionine-dependent methyltransferases superfamily protein; CONTAINS InterPro DOMAIN/s: Protein of unknown function DUF248, methyltransferase putative (InterPro:IPR004159); BEST Arabidopsis thaliana protein match is: S-adenosyl-L-methionine-dependent methyltransferases superfamily protein (TAIR:AT1G29470.2)
AT4G17240	*0,716*		unknown protein; FUNCTIONS IN: molecular_function unknown; INVOLVED IN: biological_process unknown; LOCATED IN: chloroplast; EXPRESSED IN: 16 plant structures; EXPRESSED DURING: 8 growth stages
AT4G28750	*0,716*	PSA E1 KNOCKOUT (PSAE-1)	mutant has Decreased effective quantum yield of photosystem II; Pale green plants; Reduced growth rate; Subunit E of Photosystem I
AT5G13840	*0,716*	FIZZY-RELATED 3 (FZR3)	FIZZY-related 3 (FZR3); FUNCTIONS IN: signal transducer activity; INVOLVED IN: signal transduction; LOCATED IN: chloroplast, heterotrimeric G-protein complex; EXPRESSED IN: 19 plant structures; EXPRESSED DURING: 11 growth stages; CONTAINS InterPro DOMAIN/s: WD40 repeat-like-containing domain (InterPro:IPR011046), WD40 repeat 2 (InterPro:IPR019782), WD40 repeat, conserved site (InterPro:IPR019775), WD40-repeat-containing domain (InterPro:IPR017986), WD40 repeat (InterPro:IPR001680), WD40/YVTN repeat-like-containing domain (InterPro:IPR015943), WD40 repeat, subgroup (InterPro:IPR019781); BEST Arabidopsis thaliana protein match is: FIZZY-related 2 (TAIR:AT4G22910.1)
AT5G13140	*0,713*		Pollen Ole e 1 allergen and extensin family protein; CONTAINS InterPro DOMAIN/s: Pollen Ole e 1 allergen/extensin (InterPro:IPR006041); BEST Arabidopsis thaliana protein match is: Pollen Ole e 1 allergen and extensin family protein (TAIR:AT3G26960.1)
AT1G55120	*0,712*	BETA-FRUCTOFURANOSIDASE 5 (FRUCT5)	Encodes a protein with fructan exohydrolase (FEH) activity acting on levan-type fructans (6-FEH, levanase). The enzyme does not have invertase activity.
AT2G46680	*0,712*	HOMEOBOX 7 (HB-7)	encodes a putative transcription factor that contains a homeodomain closely linked to a leucine zipper motif. Transcript is detected in all tissues examined. Is transcriptionally regulated in an ABA-dependent manner and may act in a signal transduction pathway which mediates a drought response.
AT3G23590	*0,711*	REF4-RELATED 1 (RFR1)	Encodes a protein shown to physically associate with the conserved transcriptional coregulatory complex, Mediator, and is involved in the regulation of phenylpropanoid homeostasis. Acts redundantly with REF4/MED5b (At2g48110). Required for expression of some dark-upregulated genes. RFR1 is the MED5a subunit of the mediator complex.
AT2G32240	*0,709*		FUNCTIONS IN: molecular_function unknown; INVOLVED IN: response to cadmium ion; LOCATED IN: plasma membrane; EXPRESSED IN: 25 plant structures; EXPRESSED DURING: 13 growth stages; CONTAINS InterPro DOMAIN/s: Prefoldin (InterPro:IPR009053); BEST Arabidopsis thaliana protein match is: unknown protein (TAIR:AT1G05320.3)
AT4G36900	*0,707*	RELATED TO AP2 10 (RAP2.10)	encodes a member of the DREB subfamily A-5 of ERF/AP2 transcription factor family (RAP2.10). The protein contains one AP2 domain. There are 16 members in this subfamily including RAP2.9 and RAP2.1.

AT4G38680	*0,707*	GLYCINE RICH PROTEIN 2 (GRP2)	Encodes a glycine-rich protein that binds nucleic acids and promotes DNA melting. Its transcript and protein levels are up-regulated in response to cold treatment with protein levels peaking earlier in shoots (~10-14 days) than in roots (~21 days). It is normally expressed in meristematic regions and developing tissues where cell division occurs. RNAi and antisense lines with lower levels of CSP2/GRP2 transcripts flower earlier than wild type plants and have some defects in anther and seed development.
AT1G31230	*0,705*	ASPARTATE KINASE-HOMOSERINE DEHYDROGENASE I (AK-HSDH	Encodes a bifunctional aspartate kinase/homoserine dehydrogenase. These two activities catalyze the first and the third steps toward the synthesis of the essential amino acids threonine, isoleucine and methionine.
AT2G05440	*0,705*	GLYCINE RICH PROTEIN 9 (GRP9)	GLYCINE RICH PROTEIN 9 (GRP9); FUNCTIONS IN: molecular_function unknown; INVOLVED IN: biological_process unknown; LOCATED IN: endomembrane system; CONTAINS InterPro DOMAIN/s: Glycine rich protein (InterPro:IPR010800); BEST Arabidopsis thaliana protein match is: Glycine-rich protein family (TAIR:AT2G05510.6)
AT4G26630	*0,705*	DEK-DOMAIN CONTAINING PROTEIN 3 (DEK3)	Encodes a chromatin-associated protein that specifically binds histones H3 and H4 and contributes to modulation of Arabidopsis chromatin structure and function.
AT5G13490	*0,705*	ADP/ATP CARRIER 2 (AAC2)	Encodes mitochondrial ADP/ATP carrier
AT5G66630	*0,704*	DA1-RELATED PROTEIN 5 (DAR5)	DA1-related protein 5 (DAR5); FUNCTIONS IN: zinc ion binding, ATP binding; INVOLVED IN: apoptosis; EXPRESSED IN: 12 plant structures; EXPRESSED DURING: LP.04 four leaves visible, 4 anthesis, petal differentiation and expansion stage; CONTAINS InterPro DOMAIN/s: Zinc finger, LIM-type (InterPro:IPR001781), NB-ARC (InterPro:IPR002182), Powdery mildew resistance protein, RPW8 domain (InterPro:IPR008808), Protein of unknown function DUF3633 (InterPro:IPR022087); BEST Arabidopsis thaliana protein match is: DA1-related protein 6 (TAIR:AT5G66620.1)
AT2G34070	*0,703*	TRICHOME BIREFRINGENCE-LIKE 37 (TBL37)	Encodes a member of the TBL (TRICHOME BIREFRINGENCE-LIKE) gene family containing a plant-specific DUF231 (domain of unknown function) domain. TBL gene family has 46 members, two of which (TBR/AT5G06700 and TBL3/AT5G01360) have been shown to be involved in the synthesis and deposition of secondary wall cellulose, presumably by influencing the esterification state of pectic polymers. A nomenclature for this gene family has been proposed (Volker Bischoff & Wolf Scheible, 2010, personal communication).
AT3G05690	*0,702*	NUCLEAR FACTOR Y, SUBUNIT A2 (NF-YA2)	Encodes a subunit of CCAAT-binding complex, binds to CCAAT box motif present in some plant promoter sequences. One of three members of this class (HAP2A, HAP2B, HAP2C), it is expressed in vegetative and reproductive tissues.
AT3G46010	*0,702*	ACTIN DEPOLYMERIZING FACTOR 1 (ADF1)	Actin-depolymerizing factor (ADF) and cofilin define a family of actin-binding proteins essential for the rapid turnover of filamentous actin in vivo.
AT1G77450	*0,701*	NAC DOMAIN CONTAINING PROTEIN 32 (NAC032)	NAC domain containing protein 32 (NAC032); FUNCTIONS IN: sequence-specific DNA binding transcription factor activity; INVOLVED IN: multicellular organismal development, regulation of transcription; LOCATED IN: cellular_component unknown; EXPRESSED IN: 16 plant structures; EXPRESSED DURING: LP.06 six leaves visible, LP.04 four leaves visible, 4 anthesis, petal differentiation and expansion stage, LP.08 eight leaves visible; CONTAINS InterPro DOMAIN/s: No apical meristem (NAM) protein (InterPro:IPR003441); BEST Arabidopsis thaliana protein match is: NAC (No Apical Meristem) domain transcriptional regulator superfamily protein (TAIR:AT1G01720.1)
AT4G23940	*0,701*	FTSH INACTIVE PROTEASE 1 (FTSHI1)	Encodes FtsHi1. Localizes to the chloroplast envelope membrane. Functions in chloroplast biogenesis and division.
AT1G17220	*0,7*	FU-GAERI1 (FUG1)	Encodes a chloroplast localized protein with similarity to translation initiation factor 2. Can complement loss of INFB in E.coli suggesting FUG1 does function as a translation initiation factor in vivo. Identified as a suppressor of the leaf variegation mutant var2-6. Suppression is only seen in hypomorphs as complete loss of function alleles are embryo lethal. The mRNA is cell-to-cell mobile.
AT2G30970	*0,7*	ASPARTATE AMINOTRANSFERASE 1 (ASP1)	ASPARTATE AMINOTRANSFERASE 1
AT4G09010	*0,7*	THYLAKOID LUMEN 29 (TL29)	Encodes a thylakoid lumen protein that was initially believed to act as a microsomal ascorbate peroxidase APX4 but to date, no evidence of enzymatic activity has been found.

AT4G30490	*0,7*		AFG1-like ATPase family protein; FUNCTIONS IN: ATPase activity, ATP binding; INVOLVED IN: biological_process unknown; EXPRESSED IN: 23 plant structures; EXPRESSED DURING: 15 growth stages; CONTAINS InterPro DOMAIN/s: ATPase, AFG1-like (InterPro:IPR005654); BEST Arabidopsis thaliana protein match is: AFG1-like ATPase family protein (TAIR:AT4G28070.2)
AT2G34860	*0,699*	EMBRYO SAC DEVELOPMENT ARREST 3 (EDA3)	embryo sac development arrest 3 (EDA3); FUNCTIONS IN: unfolded protein binding, heat shock protein binding; INVOLVED IN: megagametogenesis; LOCATED IN: chloroplast thylakoid membrane, chloroplast; EXPRESSED IN: 22 plant structures; EXPRESSED DURING: 13 growth stages; CONTAINS InterPro DOMAIN/s: Heat shock protein DnaJ, cysteine-rich domain (InterPro:IPR001305)
AT5G41600	*0,699*	VIRB2-INTERACTING PROTEIN 3 (BTI3)	VIRB2-interacting protein 3 (BTI3); INVOLVED IN: biological_process unknown; LOCATED IN: endoplasmic reticulum, plasma membrane, endoplasmic reticulum membrane; EXPRESSED IN: 23 plant structures; EXPRESSED DURING: 13 growth stages; CONTAINS InterPro DOMAIN/s: Reticulon (InterPro:IPR003388); BEST Arabidopsis thaliana protein match is: Reticulan like protein B3 (TAIR:AT1G64090.1)
AT3G03640	*0,698*	BETA GLUCOSIDASE 25 (BGLU25)	Encodes beta-glucosidase (GLUC).
AT1G35710	*0,697*		Protein kinase family protein with leucine-rich repeat domain; FUNCTIONS IN: protein serine/threonine kinase activity, protein tyrosine kinase activity, protein kinase activity, ATP binding; INVOLVED IN: transmembrane receptor protein tyrosine kinase signaling pathway, protein amino acid phosphorylation; LOCATED IN: endomembrane system; EXPRESSED IN: 22 plant structures; EXPRESSED DURING: 13 growth stages; CONTAINS InterPro DOMAIN/s: Protein kinase, ATP binding site (InterPro:IPR017441), Serine/threonine-protein kinase domain (InterPro:IPR002290), Leucine-rich repeat-containing N-terminal domain, type 2 (InterPro:IPR013210), Leucine-rich repeat (InterPro:IPR001611), Serine/threonine-protein kinase-like domain (InterPro:IPR017442), Protein kinase-like domain (InterPro:IPR011009), Protein kinase, catalytic domain (InterPro:IPR000719), Leucine-rich repeat, typical subtype (InterPro:IPR003591), Tyrosine-protein kinase, active site (InterPro:IPR008266), Tyrosine-protein kinase, catalytic domain (InterPro:IPR020635); BEST Arabidopsis thaliana protein match is: Leucine-rich repeat receptor-like protein kinase family protein (TAIR:AT4G08850.1)
AT1G71830	*0,697*	SOMATIC EMBRYOGENESIS RECEPTOR-LIKE KINASE 1 (SERK1)	Plasma membrane LRR receptor-like serine threonine kinase expressed during embryogenesis in locules until stage 6 anthers, with higher expression in the tapetal cell layer. SERK1 and SERK2 receptor kinases function redundantly as an important control point for sporophytic development controlling male gametophyte production. later
AT2G45290	*0,697*	TRANSKETOLASE 2 (TKL2)	Transketolase; FUNCTIONS IN: catalytic activity, transketolase activity; INVOLVED IN: response to cadmium ion; LOCATED IN: chloroplast stroma, chloroplast; EXPRESSED IN: 25 plant structures; EXPRESSED DURING: 16 growth stages; CONTAINS InterPro DOMAIN/s: Transketolase, bacterial-like (InterPro:IPR005478), Transketolase, N-terminal (InterPro:IPR005474), Transketolase, C-terminal (InterPro:IPR005476), Transketolase, C-terminal/Pyruvate-ferredoxin oxidoreductase, domain II (InterPro:IPR009014), Transketolase-like, C-terminal (InterPro:IPR015941), Transketolase-like, pyrimidine-binding domain (InterPro:IPR005475), Transketolase binding site (InterPro:IPR020826); BEST Arabidopsis thaliana protein match is: Transketolase (TAIR:AT3G60750.1)
AT1G77670	*0,696*		Pyridoxal phosphate (PLP)-dependent transferases superfamily protein; FUNCTIONS IN: 1-aminocyclopropane-1-carboxylate synthase activity, transferase activity, transferring nitrogenous groups, pyridoxal phosphate binding, transaminase activity, catalytic activity; INVOLVED IN: asparagine catabolic process, biosynthetic process, glutamate catabolic process to oxaloacetate, aspartate transamidation; EXPRESSED IN: 22 plant structures; EXPRESSED DURING: 13 growth stages; CONTAINS InterPro DOMAIN/s: 1-aminocyclopropane-1-carboxylate synthase (InterPro:IPR001176), Aminotransferase, class I/classII (InterPro:IPR004839), Pyridoxal phosphate-dependent transferase, major domain (InterPro:IPR015424), Pyridoxal phosphate-dependent transferase, major region, subdomain 1 (InterPro:IPR015421); BEST Arabidopsis thaliana protein match is: aspartate aminotransferase (TAIR:AT2G22250.3)
AT2G27810	*0,696*	NUCLEOBASE-ASCORBATE TRANSPORTER 12 (NAT12)	Encodes a plasma-membrane localized nucleobase transporter capable of transporting adenine, guanine, uracil and hypoxanthine. Likely to be a proton-nucleobase symporter.

AT4G26190	*0,695*		Haloacid dehalogenase-like hydrolase (HAD) superfamily protein; CONTAINS InterPro DOMAIN/s: NLI interacting factor (InterPro:IPR004274); BEST Arabidopsis thaliana protein match is: Haloacid dehalogenase-like hydrolase (HAD) superfamily protein (TAIR:AT2G36540.1)
AT1G59740	*0,694*	NRT1/ PTR FAMILY 4.3 (NPF4.3)	Major facilitator superfamily protein; FUNCTIONS IN: transporter activity; INVOLVED IN: oligopeptide transport, response to nematode; LOCATED IN: plasma membrane, membrane; EXPRESSED IN: 19 plant structures; EXPRESSED DURING: 10 growth stages; CONTAINS InterPro DOMAIN/s: PTR2 family proton/oligopeptide symporter, conserved site (InterPro:IPR018456), Oligopeptide transporter (InterPro:IPR000109), Major facilitator superfamily, general substrate transporter (InterPro:IPR016196); BEST Arabidopsis thaliana protein match is: Major facilitator superfamily protein (TAIR:AT1G33440.1)
AT2G45100	*0,694*		Cyclin/Brf1-like TBP-binding protein; FUNCTIONS IN: RNA polymerase II transcription factor activity, transcription regulator activity, transcription activator activity, zinc ion binding, translation initiation factor activity; INVOLVED IN: translational initiation, positive regulation of transcription, regulation of transcription, DNA-dependent, transcription initiation; LOCATED IN: nucleus; EXPRESSED IN: 21 plant structures; EXPRESSED DURING: 13 growth stages; CONTAINS InterPro DOMAIN/s: Transcription factor TFIIB related (InterPro:IPR000812), Cyclin-like (InterPro:IPR011028), Transcription factor TFIIB, cyclin-related (InterPro:IPR013150), Cyclin-related (InterPro:IPR013763), Cyclin (InterPro:IPR006670), Brf1-like TBP-binding (InterPro:IPR011665); BEST Arabidopsis thaliana protein match is: Cyclin/Brf1-like TBP-binding protein (TAIR:AT3G09360.1)
AT5G16030	*0,693*		unknown protein; FUNCTIONS IN: molecular_function unknown; INVOLVED IN: biological_process unknown; LOCATED IN: cellular_component unknown; EXPRESSED IN: 20 plant structures; EXPRESSED DURING: 13 growth stages; BEST Arabidopsis thaliana protein match is: unknown protein (TAIR:AT3G02500.1)
AT5G56980	*0,693*		unknown protein; FUNCTIONS IN: molecular_function unknown; INVOLVED IN: biological_process unknown; LOCATED IN: endomembrane system; EXPRESSED IN: 18 plant structures; EXPRESSED DURING: 12 growth stages; BEST Arabidopsis thaliana protein match is: unknown protein (TAIR:AT4G26130.1)
AT1G68760	*0,691*	NUDIX HYDROLASE 1 (NUDX1)	Encodes a cytosol-localized nudix hydrolase that hydrolyzes 8-oxo-(d)GTP to its monophosphate form. This protective mechanism prevents the misincorporation of these oxidized nucleotides into DNA and RNA. NUDX1 also has a low level of dihydroneopterin triphosphate pyrophosphatase activity in vitro and may participate in the folate synthesis pathway.
AT2G32410	*0,691*	AXR1-LIKE (AXL)	AXR1-like (AXL); FUNCTIONS IN: binding, catalytic activity; INVOLVED IN: auxin homeostasis, auxin mediated signaling pathway, post-embryonic development, protein ubiquitination; LOCATED IN: peroxisome; EXPRESSED IN: 23 plant structures; EXPRESSED DURING: 11 growth stages; CONTAINS InterPro DOMAIN/s: UBA/THIF-type NAD/FAD binding fold (InterPro:IPR000594), Molybdenum cofactor biosynthesis, MoeB (InterPro:IPR009036), NAD(P)-binding domain (InterPro:IPR016040); BEST Arabidopsis thaliana protein match is: NAD(P)-binding Rossmann-fold superfamily protein (TAIR:AT1G05180.1)
AT4G23500	*0,691*		Pectin lyase-like superfamily protein; FUNCTIONS IN: polygalacturonase activity; INVOLVED IN: carbohydrate metabolic process; LOCATED IN: cell wall; CONTAINS InterPro DOMAIN/s: Pectin lyase fold/virulence factor (InterPro:IPR011050), Glycoside hydrolase, family 28 (InterPro:IPR000743), Pectin lyase fold (InterPro:IPR012334), Parallel beta-helix repeat (InterPro:IPR006626); BEST Arabidopsis thaliana protein match is: Pectin lyase-like superfamily protein (TAIR:AT3G61490.3)
AT2G32800	*0,689*	L-TYPE LECTIN RECEPTOR KINASE S.2 (LECRK-S.2)	AP4.3A; FUNCTIONS IN: protein serine/threonine kinase activity, protein kinase activity, ATP binding; INVOLVED IN: protein amino acid phosphorylation; LOCATED IN: plasma membrane; EXPRESSED IN: 19 plant structures; EXPRESSED DURING: 10 growth stages; CONTAINS InterPro DOMAIN/s: Protein kinase, ATP binding site (InterPro:IPR017441), Serine/threonine-protein kinase domain (InterPro:IPR002290), Serine-threonine/tyrosine-protein kinase (InterPro:IPR001245), Serine/threonine-protein kinase-like domain (InterPro:IPR017442), Serine/threonine-protein kinase, active site (InterPro:IPR008271), Protein kinase-like domain (InterPro:IPR011009), Protein kinase, catalytic domain (InterPro:IPR000719), Tyrosine-protein kinase, catalytic domain (InterPro:IPR020635); BEST Arabidopsis thaliana protein match is: Concanavalin A-like lectin protein kinase family protein (TAIR:AT3G53810.1)
AT5G65630	*0,689*	GLOBAL TRANSCRIPTION FACTOR GROUP E7 (GTE7)	This gene is predicted to encode a bromodomain-containing protein. Plant lines expressing RNAi constructs targeted against GTE7 show some resistance to agrobacterium-mediated root transformation.

AT1G69295	*0,688*	PLASMODESMATA CALLOSE-BINDING PROTEIN 4 (PDCB4)	Encodes a member of the X8-GPI family of proteins. It localizes to the plasmodesmata and is predicted to bind callose.
AT3G49680	*0,688*	BRANCHED-CHAIN AMINOTRANSFERASE 3 (BCAT3)	Encodes a chloroplast branched-chain amino acid aminotransferase. Complements the yeast leu/iso-leu/val auxotrophy mutant.
AT2G30720	*0,687*		Thioesterase/thiol ester dehydrase-isomerase superfamily protein; BEST Arabidopsis thaliana protein match is: Thioesterase/thiol ester dehydrase-isomerase superfamily protein (TAIR:AT5G48370.1)
AT1G44575	*0,686*	NONPHOTOCHEMICAL QUENCHING 4 (NPQ4)	Encoding PSII-S (CP22), a ubiquitous pigment-binding protein associated with photosystem II (PSII) of higher plants. Involved in nonphotochemical quenching rather than in photosynthesis. Mutant has a normal violaxanthin cycle but has a limited capacity of quenching singlet excited chlorophylls and is tolerant to lipid peroxidation.
AT1G61520	*0,686*	PHOTOSYSTEM I LIGHT HARVESTING COMPLEX GENE 3 (LHCA3)	PSI type III chlorophyll a/b-binding protein (Lhca3*1) The mRNA is cell-to-cell mobile.
AT1G73010	*0,686*	PHOSPHATE STARVATION-INDUCED GENE 2 (PS2)	Encodes PPsPase1, a pyrophosphate-specific phosphatase catalyzing the specific cleavage of pyrophosphate (Km 38.8 uM) with an alkaline catalytic pH optimum. Expression is upregulated in the shoot of cax1/cax3 mutant.
AT3G04550	*0,686*	RUBISCO ACCUMULATION FACTOR 1 (RAF1)	Encodes an ancillary chaperone protein that functions in Rubisco biogenesis. RAF1 dimers function in the assembly of the large subunit of Rubisco. Co-expression of RAF1 and rbcL in tobacco cells results in increased photosynthesis and plant growth. The mRNA is cell-to-cell mobile.
AT4G00370	*0,686*	(ANTR2)	Encodes an inorganic phosphate transporter (PHT4;4) that can transport ascorbate and is located in the chloroplast envelope membrane. It has been shown to play a role in the xanthophyll cycle during photosynthesis and may be required for tolerance to strong light stress.
AT5G27600	*0,685*	LONG-CHAIN ACYL-COA SYNTHETASE 7 (LACS7)	Encode peroxisomal long-chain acyl-CoA synthetase. Activates fatty acids for further metabolism. Interacts with PEX5.
AT5G51600	*0,685*	PLEIADE (PLE)	Mutant has defective roots. Essential for giant cell ontogenesis. Role in organizing the mitotic microtubule array during both early and late mitosis in all plant organs.
AT1G63880	*0,684*		Encodes a TIR-NBS-LRR class of disease resistance protein effective against Leptosphaeria maculans. The mRNA is cell-to-cell mobile.
AT3G16000	*0,684*	MAR BINDING FILAMENT-LIKE PROTEIN 1 (MFP1)	encodes a DNA-binding protein that binds to plastid DNA non-specifically and is associated with nucleoids and thylakoid membranes. The expression of the gene is correlated with the development of thylakoid membranes.
AT1G20620	*0,683*	CATALASE 3 (CAT3)	Catalase, catalyzes the breakdown of hydrogen peroxide (H2O2) into water and oxygen. The mRNA is cell-to-cell mobile.
AT1G33110	*0,682*		MATE efflux family protein; FUNCTIONS IN: antiporter activity, drug transmembrane transporter activity, transporter activity; INVOLVED IN: drug transmembrane transport, transmembrane transport; LOCATED IN: membrane; EXPRESSED IN: 22 plant structures; EXPRESSED DURING: 13 growth stages; CONTAINS InterPro DOMAIN/s: Multi antimicrobial extrusion protein MatE (InterPro:IPR002528); BEST Arabidopsis thaliana protein match is: MATE efflux family protein (TAIR:AT1G33100.1)
AT3G54050	*0,681*	HIGH CYCLIC ELECTRON FLOW 1 (HCEF1)	Encodes a chloroplastic fructose 1,6-bisphosphate phosphatase. also known as HCEF1 (High Cyclic Electron Flow 1). hcef1 mutants have constitutively elevated electron flow (CEFI) and plants with antisense suppression of this enzyme have higher levels of net leaf photosynthesis and increased sucrose biosynthesis. The mRNA is cell-to-cell mobile.
AT1G14330	*0,68*		Galactose oxidase/kelch repeat superfamily protein; CONTAINS InterPro DOMAIN/s: Galactose oxidase/kelch, beta-propeller (InterPro:IPR011043), Kelch repeat type 1 (InterPro:IPR006652), Kelch related (InterPro:IPR013089), Kelch-type beta propeller (InterPro:IPR015915); BEST Arabidopsis thaliana protein match is: Galactose oxidase/kelch repeat superfamily protein (TAIR:AT2G02870.3)
AT1G35420	*0,68*		alpha/beta-Hydrolases superfamily protein; FUNCTIONS IN: hydrolase activity; INVOLVED IN: biological_process unknown; EXPRESSED IN: 22 plant structures; EXPRESSED DURING: 13 growth stages; CONTAINS InterPro DOMAIN/s: Dienelactone hydrolase (InterPro:IPR002925); BEST Arabidopsis thaliana protein match is: alpha/beta-Hydrolases superfamily protein (TAIR:AT3G23600.1).

AT1G74640	*0,68*		alpha/beta-Hydrolases superfamily protein; INVOLVED IN: biological_process unknown; LOCATED IN: chloroplast thylakoid membrane, chloroplast, chloroplast envelope; EXPRESSED IN: 24 plant structures; EXPRESSED DURING: 15 growth stages
AT1G80030	*0,68*	DNA J PROTEIN A7 (DJA7)	Molecular chaperone Hsp40/DnaJ family protein; FUNCTIONS IN: unfolded protein binding, heat shock protein binding, ATP binding; INVOLVED IN: protein folding, response to heat; LOCATED IN: chloroplast thylakoid membrane, chloroplast; EXPRESSED IN: 23 plant structures; EXPRESSED DURING: 14 growth stages; CONTAINS InterPro DOMAIN/s: Molecular chaperone, heat shock protein, Hsp40, DnaJ (InterPro:IPR015609), HSP40/DnaJ peptide-binding (InterPro:IPR008971), Chaperone DnaJ, C-terminal (InterPro:IPR002939), Heat shock protein DnaJ, N-terminal (InterPro:IPR001623), Heat shock protein DnaJ, conserved site (InterPro:IPR018253), Heat shock protein DnaJ, cysteine-rich domain (InterPro:IPR001305), Chaperone DnaJ (InterPro:IPR012724), Heat shock protein DnaJ (InterPro:IPR003095); BEST Arabidopsis thaliana protein match is: Molecular chaperone Hsp40/DnaJ family protein (TAIR:AT3G17830.1)
AT3G25610	*0,679*	AMINOPHOSPHOLIPID ATPASE10 (ALA10)	Encodes aminophospholipid ATPase10 (ALA10), a P4-type ATPase flippase that internalizes exogenous phospholipids across the plasma membrane.
AT4G30990	*0,679*		ARM repeat superfamily protein; FUNCTIONS IN: binding; INVOLVED IN: biological_process unknown; LOCATED IN: Golgi apparatus; CONTAINS InterPro DOMAIN/s: Down-regulated-in-metastasis protein (InterPro:IPR011430), Armadillo-type fold (InterPro:IPR016024); BEST Arabidopsis thaliana protein match is: unknown protein (TAIR:AT5G36722.1)
AT4G35630	*0,679*	PHOSPHOSERINE AMINOTRANSFERASE 1 (PSAT1)	Encodes a phosphoserine aminotransferase which is involved in serine biosynthesis in the chloroplast which operates via the phosphorylated pathway. The mRNA is cell-to-cell mobile.
AT5G01850	*0,679*		Protein kinase superfamily protein; FUNCTIONS IN: protein serine/threonine/tyrosine kinase activity, kinase activity; INVOLVED IN: protein amino acid phosphorylation; EXPRESSED IN: 22 plant structures; EXPRESSED DURING: 13 growth stages; CONTAINS InterPro DOMAIN/s: Protein kinase, ATP binding site (InterPro:IPR017441), Serine/threonine-protein kinase domain (InterPro:IPR002290), Serine-threonine/tyrosine-protein kinase (InterPro:IPR001245), Protein kinase-like domain (InterPro:IPR011009), Serine/threonine-protein kinase, active site (InterPro:IPR008271), Protein kinase, catalytic domain (InterPro:IPR000719), Serine/threonine-protein kinase, ATN1-like (InterPro:IPR015784); BEST Arabidopsis thaliana protein match is: Protein kinase superfamily protein (TAIR:AT3G27560.1)
AT1G49970	*0,678*	CLP PROTEASE PROTEOLYTIC SUBUNIT 1 (CLPR1)	Encodes a ClpP-related sequence. Though similar to ClpP proteins, this does not contains the highly conserved catalytic triad of Ser-type proteases (Ser-His-Asp). The name reflects nomenclature described in Adam et. al (2001).
AT1G22410	*0,677*		Class-II DAHP synthetase family protein; FUNCTIONS IN: 3-deoxy-7-phosphoheptulonate synthase activity; INVOLVED IN: aromatic amino acid family biosynthetic process; LOCATED IN: membrane; EXPRESSED IN: 23 plant structures; EXPRESSED DURING: 15 growth stages; CONTAINS InterPro DOMAIN/s: DAHP synthetase, class II (InterPro:IPR002480); BEST Arabidopsis thaliana protein match is: 3-deoxy-D-arabino-heptulosonate 7-phosphate synthase 1 (TAIR:AT4G39980.1)
AT1G64810	*0,677*	ACCUMULATION OF PHOTOSYSTEM ONE 1 (APO1)	Encodes a chloroplast localized RNA binding protein that is involved in group II intron splicing. Splicing defects can account for the loss of photosynthetic complexes in apo1 mutants.
AT2G05920	*0,677*	SUBTILASE 1.8 (SBT1.8)	Subtilase family protein; FUNCTIONS IN: identical protein binding, serine-type endopeptidase activity; INVOLVED IN: proteolysis, negative regulation of catalytic activity; LOCATED IN: cell wall, plant-type cell wall; EXPRESSED IN: 22 plant structures; EXPRESSED DURING: 13 growth stages; CONTAINS InterPro DOMAIN/s: Protease-associated PA (InterPro:IPR003137), Proteinase inhibitor, propeptide (InterPro:IPR009020), Peptidase S8/S53, subtilisin/kexin/sedolisin (InterPro:IPR000209), Peptidase S8, subtilisin-related (InterPro:IPR015500), Peptidase S8/S53, subtilisin, active site (InterPro:IPR022398), Proteinase inhibitor I9, subtilisin propeptide (InterPro:IPR010259); BEST Arabidopsis thaliana protein match is: Subtilase family protein (TAIR:AT5G67360.1)

AT2G44830	*0,677*		Protein kinase superfamily protein; FUNCTIONS IN: kinase activity; INVOLVED IN: protein amino acid phosphorylation; LOCATED IN: plasma membrane, nucleus; EXPRESSED IN: 19 plant structures; EXPRESSED DURING: 11 growth stages; CONTAINS InterPro DOMAIN/s: Protein kinase, catalytic domain (InterPro:IPR000719), Serine/threonine-protein kinase domain (InterPro:IPR002290), Tyrosine-protein kinase, catalytic domain (InterPro:IPR020635), Serine/threonine-protein kinase-like domain (InterPro:IPR017442), Serine/threonine-protein kinase, active site (InterPro:IPR008271), Protein kinase-like domain (InterPro:IPR011009); BEST Arabidopsis thaliana protein match is: D6 protein kinase like 2 (TAIR:AT5G47750.1)
AT5G66470	*0,677*	(ERA-1)	RNA binding;GTP binding; FUNCTIONS IN: RNA binding, GTP binding; LOCATED IN: chloroplast; EXPRESSED IN: 20 plant structures; EXPRESSED DURING: 13 growth stages; CONTAINS InterPro DOMAIN/s: K Homology, type 2 (InterPro:IPR004044), K Homology, prokaryotic type (InterPro:IPR009019), Small GTP-binding protein (InterPro:IPR005225), GTP1/OBG (InterPro:IPR006073), GTP-binding protein Era (InterPro:IPR005662), GTP-binding protein, HSR1-related (InterPro:IPR002917), K homology-like, alpha/beta (InterPro:IPR015946); BEST Arabidopsis thaliana protein match is: GTP-binding family protein (TAIR:AT1G30960.1)
AT4G14070	*0,676*	ACYL-ACTIVATING ENZYME 15 (AAE15)	Plastidic acyl activating enzyme involved in the elongation of exogenous medium-chain fatty acids to 16- and 18-carbon fatty acids.
AT1G31340	*0,675*	RELATED TO UBIQUITIN 1 (RUB1)	Encodes a ubiquitin-related protein that is conjugated to target proteins by neddylation. It has been shown to be conjugated to the cullin AtCUL1. The RUB-conjugation pathway has been implicated in in auxin response.
AT5G23240	*0,675*	DNA J PROTEIN C76 (DJC76)	DNAJ heat shock N-terminal domain-containing protein; FUNCTIONS IN: unfolded protein binding, heat shock protein binding; INVOLVED IN: protein folding; LOCATED IN: chloroplast; EXPRESSED IN: 21 plant structures; EXPRESSED DURING: 11 growth stages; CONTAINS InterPro DOMAIN/s: Molecular chaperone, heat shock protein, Hsp40, DnaJ (InterPro:IPR015609), Heat shock protein DnaJ, N-terminal (InterPro:IPR001623), Heat shock protein DnaJ (InterPro:IPR003095); BEST Arabidopsis thaliana protein match is: DNAJ heat shock N-terminal domain-containing protein (TAIR:AT2G42750.1)
AT4G34980	*0,674*	SUBTILISIN-LIKE SERINE PROTEASE 2 (SLP2)	Serine protease similar to subtilisin.
AT3G55040	*0,673*	GLUTATHIONE TRANSFERASE LAMBDA 2 (GSTL2)	Encodes a member of the lambda family of glutathione transferases. It has thiol transferase activity and self S-glutathionylation activity in vitro.
AT5G01730	*0,672*	SCAR FAMILY PROTEIN 4 (SCAR4)	Encodes a member of the SCAR family.These proteins are part of a complex (WAVE) complex.The SCAR subunit activates the ARP2/3 complex which in turn act as a nucleator for actin filaments.
AT5G47730	*0,672*		Sec14p-like phosphatidylinositol transfer family protein; CONTAINS InterPro DOMAIN/s: Cellular retinaldehyde-binding/triple function, C-terminal (InterPro:IPR001251), Cellular retinaldehyde-binding/triple function, N-terminal (InterPro:IPR008273), Phosphatidylinositol transfer protein-like, N-terminal (InterPro:IPR011074); BEST Arabidopsis thaliana protein match is: Sec14p-like phosphatidylinositol transfer family protein (TAIR:AT1G55840.1)
AT5G63550	*0,672*		DEK domain-containing chromatin associated protein; EXPRESSED IN: guard cell; CONTAINS InterPro DOMAIN/s: DEK, C-terminal (InterPro:IPR014876); BEST Arabidopsis thaliana protein match is: DEK domain-containing chromatin associated protein (TAIR:AT3G48710.1)
AT1G07910	*0,671*	RNALIGASE (RNL)	Encodes a tRNA ligase that resembles the yeast Trl1 RNA ligase in structure and function but very different in sequence. Like Trl1, AtRNL consists of two domains ? an N-terminal ligase component and a C-terminal 5'-kinase/2',3'-cyclic phosphodiesterase (CPD) component? that can function in tRNA splicing in vivo when expressed as separate polypeptides. Requires a 2'-PO4 end for tRNA splicing in vivo.
AT2G37710	*0,671*	L-TYPE LECTIN RECEPTOR KINASE IV.1 (LECRK-IV.1)	Induced in response to Salicylic acid. The mRNA is cell-to-cell mobile.
AT5G15450	*0,671*	CASEIN LYTIC PROTEINASE B3 (CLPB3)	Encodes a chloroplast-targeted Hsp101 homologue. Functions as a molecular chaperone involved in plastid differentiation mediating internal thylakoid membrane formation and conferring thermotolerance to chloroplasts during heat stress. APG6 is constitutively expressed in the root tips, the organ boundary region, the reproductive tissues of mature plants where plastids exist as proplastids, and slightly in the stems and leaves. APG6 expression is upregulated in response to heat shock in various organs, but not in response to other abiotic stresses. Apg6 mutants have a pale-green phenotype.

AT5G54380	*0,671*	THESEUS1 (THE1)	Encodes THESEUS1 (THE1), a receptor kinase regulated by Brassinosteroids and required for cell elongation during vegetative growth.
AT3G23670	*0,67*	(KINESIN-12B)	Microtubule motor kinesin PAKRP1L/Kinesin-12B. Together with PAKRP1/Kinesin-12A, serve as linkers of the plus ends of antiparallel microtubules in the phragmoplast.
AT5G03190	*0,669*	CONSERVED PEPTIDE UPSTREAM OPEN READING FRAME 47 (CPuORF47)	conserved peptide upstream open reading frame 47 (CPUORF47); FUNCTIONS IN: methyltransferase activity; INVOLVED IN: metabolic process; EXPRESSED IN: 22 plant structures; EXPRESSED DURING: 13 growth stages; CONTAINS InterPro DOMAIN/s: Methyltransferase type 11 (InterPro:IPR013216); BEST Arabidopsis thaliana protein match is: unknown protein (TAIR:AT3G53400.1)
AT1G08290	*0,667*	WIP DOMAIN PROTEIN 3 (WIP3)	WIP domain protein 3 (WIP3); CONTAINS InterPro DOMAIN/s: Zinc finger, C2H2-like (InterPro:IPR015880), Zinc finger, C2H2-type (InterPro:IPR007087); BEST Arabidopsis thaliana protein match is: WIP domain protein 5 (TAIR:AT1G51220.1)
AT2G22100	*0,666*		RNA-binding (RRM/RBD/RNP motifs) family protein; FUNCTIONS IN: RNA binding, nucleotide binding, nucleic acid binding; INVOLVED IN: biological_process unknown; LOCATED IN: cellular_component unknown; EXPRESSED IN: 21 plant structures; EXPRESSED DURING: 12 growth stages; CONTAINS InterPro DOMAIN/s: RNA recognition motif, RNP-1 (InterPro:IPR000504), Nucleotide-binding, alpha-beta plait (InterPro:IPR012677); BEST Arabidopsis thaliana protein match is: RNA recognition motif (RRM)-containing protein (TAIR:AT2G19380.1)
AT4G14680	*0,666*	(APS3)	ATP sulfurylase
AT1G74040	*0,665*	2-ISOPROPYLMALATE SYNTHASE 1 (IMS1)	Encodes an active Arabidopsis isopropylmalate synthase IPMS2. Involved in leucine biosynthesis. Do not participate in the chain elongation of glucosinolates. Expressed constitutively throughout the plant. Loss of IPMS2 can be compensated by a second isopropylmalate synthase gene IPMS1 (At1g18500).
AT3G46440	*0,665*	UDP-XYL SYNTHASE 5 (UXS5)	encodes a protein similar to UDP-glucuronic acid decarboxylase. UDP-glucuronic acid decarboxylase produces UDP-xylose, which is a substrate for many cell wall carbohydrates including hemicellulose and pectin. UDP-xylose is also known to feedback regulate several cell wall biosynthetic enzymes.
AT5G53800	*0,665*		unknown protein
AT5G54160	*0,665*	O-METHYLTRANSFERASE 1 (OMT1)	A caffeic acid/5-hydroxyferulic acid O-methyltransferase. Interacts with 14-4-3 proteins in yeast 2 hybrid assay. AtOMT1 (At5g54160) encodes a flavonol 3?-O-methyltransferase that is highly active towards quercetin and myricetin. The substrate specificity identifies the enzyme as flavonol 3?-methyltransferase which replaces the former annotation of the gene to encode a caffeic acid/5-hydroxyferulic acid O-methyltransferase The mRNA is cell-to-cell mobile.
AT5G17310	*0,664*	UDP-GLUCOSE PYROPHOSPHORYLASE 2 (UGP2)	UDP-glucose pyrophosphorylase 2 (UGP2); FUNCTIONS IN: UTP:glucose-1-phosphate uridylyltransferase activity, nucleotidyltransferase activity; INVOLVED IN: response to cadmium ion, callose deposition in cell wall, response to salt stress, metabolic process, pollen development; LOCATED IN: plasma membrane; EXPRESSED IN: 27 plant structures; EXPRESSED DURING: 17 growth stages; CONTAINS InterPro DOMAIN/s: UTP--glucose-1-phosphate uridylyltransferase, subgroup (InterPro:IPR016267), UTP--glucose-1-phosphate uridylyltransferase (InterPro:IPR002618); BEST Arabidopsis thaliana protein match is: UDP-GLUCOSE PYROPHOSPHORYLASE 1 (TAIR:AT3G03250.1)
AT3G46780	*0,663*	PLASTID TRANSCRIPTIONALLY ACTIVE 16 (PTAC16)	plastid transcriptionally active 16 (PTAC16); FUNCTIONS IN: binding, catalytic activity; INVOLVED IN: metabolic process; LOCATED IN: in 6 components; EXPRESSED IN: 21 plant structures; EXPRESSED DURING: 14 growth stages; CONTAINS InterPro DOMAIN/s: NAD(P)-binding domain (InterPro:IPR016040), NmrA-like (InterPro:IPR008030); BEST Arabidopsis thaliana protein match is: NAD(P)-binding Rossmann-fold superfamily protein (TAIR:AT3G18890.1)
AT5G46700	*0,663*	TORNADO 2 (TRN2)	Encodes a transmembrane protein of the tetraspanin (TET) family, one of 17 members found in Arabidopsis. Double mutant analysis showed that TRN1 and TRN2 act in the same pathway. Required for the maintenance of both the radial pattern of tissue differentiation in the root and for the subsequent circumferential pattern within the epidermis.
AT4G25100	*0,662*	FE SUPEROXIDE DISMUTASE 1 (FSD1)	Fe-superoxide dismutase
AT4G36680	*0,662*		Tetratricopeptide repeat (TPR)-like superfamily protein; CONTAINS InterPro DOMAIN/s: Pentatricopeptide repeat (InterPro:IPR002885); BEST Arabidopsis thaliana protein match is: Tetratricopeptide repeat (TPR)-like superfamily protein (TAIR:AT2G18520.1)

AT1G78815	*0,66*	LIGHT SENSITIVE HYPOCOTYLS 7 (LSH7)	LIGHT SENSITIVE HYPOCOTYLS 7 (LSH7); FUNCTIONS IN: molecular_function unknown; INVOLVED IN: biological_process unknown; LOCATED IN: chloroplast; EXPRESSED IN: inflorescence meristem, hypocotyl, root, flower; EXPRESSED DURING: petal differentiation and expansion stage; CONTAINS InterPro DOMAIN/s: Protein of unknown function DUF640 (InterPro:IPR006936); BEST Arabidopsis thaliana protein match is: Protein of unknown function (DUF640) (TAIR:AT1G16910.1)
AT5G09460	*0,66*	(SACL1)	sequence-specific DNA binding transcription factors;transcription regulators; CONTAINS InterPro DOMAIN/s: Helix-loop-helix DNA-binding domain (InterPro:IPR001092); BEST Arabidopsis thaliana protein match is: sequence-specific DNA binding transcription factors;transcription regulators (TAIR:AT5G64340.1)
AT1G55020	*0,658*	LIPOXYGENASE 1 (LOX1)	lipoxygenase, a defense gene conferring resistance Xanthomonas campestris The mRNA is cell-to-cell mobile.
AT5G24810	*0,658*		ABC1 family protein; CONTAINS InterPro DOMAIN/s: ABC-1 (InterPro:IPR004147), Beta-lactamase-type transpeptidase fold (InterPro:IPR012338), Beta-lactamase-related (InterPro:IPR001466), Protein kinase-like domain (InterPro:IPR011009); BEST Arabidopsis thaliana protein match is: ABC2 homolog 13 (TAIR:AT5G64940.2).
AT1G63420	*0,656*		CONTAINS InterPro DOMAIN/s: Lipopolysaccharide-modifying protein (InterPro:IPR006598), Protein of unknown function DUF821, CAP10-like (InterPro:IPR008539); BEST Arabidopsis thaliana protein match is: Arabidopsis thaliana protein of unknown function (DUF821) (TAIR:AT3G48980.1)
AT2G39350	*0,656*	ATP-BINDING CASSETTE G1 (ABCG1)	Belongs to a clade of five Arabidopsis thaliana ABCG half-transporters that are required for synthesis of an effective suberin barrier in roots and seed coats (ABCG2, ABCG6, and ABCG20) and for synthesis of an intact pollen wall (ABCG1 and ABCG16).
AT4G20880	*0,656*		ethylene-responsive nuclear protein / ethylene-regulated nuclear protein (ERT2); BEST Arabidopsis thaliana protein match is: ethylene-responsive nuclear protein -related (TAIR:AT5G44350.1)
AT2G39710	*0,655*		Encodes a Cysteine-rich peptide (CRP) family protein
AT3G05030	*0,655*	SODIUM HYDROGEN EXCHANGER 2 (NHX2)	Encodes a vacuolar K+/H+ exchanger essential for active K+ uptake at the tonoplast and involved in regulating stomatal closure.
AT5G39570	*0,655*		FUNCTIONS IN: molecular_function unknown; INVOLVED IN: biological_process unknown; LOCATED IN: cytosol, nucleus; EXPRESSED IN: 25 plant structures; EXPRESSED DURING: 14 growth stages; BEST Arabidopsis thaliana protein match is: glycine-rich protein (TAIR:AT3G29075.1)
AT1G25530	*0,654*		Transmembrane amino acid transporter family protein; CONTAINS InterPro DOMAIN/s: Amino acid transporter, transmembrane (InterPro:IPR013057); BEST Arabidopsis thaliana protein match is: lysine histidine transporter 1 (TAIR:AT5G40780.1)
AT5G10510	*0,654*	AINTEGUMENTA-LIKE 6 (AIL6)	Encodes an AP2-domain transcription factor involved in root stem cell identity and root development. It is also required to maintain high levels of PIN1 expression at the periphery of the meristem and modulate local auxin production in the central region of the SAM which underlies phyllotactic transitions. Intronic sequences are required for its expression in flowers.
AT5G23060	*0,653*	CALCIUM SENSING RECEPTOR (CaS)	Encodes a chloroplast-localized protein that modulates cytoplasmic Ca2+ concentration and is crucial for proper stomatal regulation in response to elevations of external Ca2+. Phosphorylation of this protein is dependent on calcium.
AT1G67720	*0,652*		Leucine-rich repeat protein kinase family protein; FUNCTIONS IN: protein kinase activity, kinase activity; INVOLVED IN: protein amino acid phosphorylation; LOCATED IN: endomembrane system; EXPRESSED IN: 21 plant structures; EXPRESSED DURING: 13 growth stages; CONTAINS InterPro DOMAIN/s: Protein kinase, ATP binding site (InterPro:IPR017441), Protein kinase, catalytic domain (InterPro:IPR000719), Serine-threonine/tyrosine-protein kinase (InterPro:IPR001245), Protein kinase-like domain (InterPro:IPR011009), Serine/threonine-protein kinase, active site (InterPro:IPR008271); BEST Arabidopsis thaliana protein match is: Leucine-rich repeat protein kinase family protein (TAIR:AT2G37050.3)
AT1G30620	*0,651*	MURUS 4 (MUR4)	encodes a type-II membrane protein that catalyzes 4-epimerization of UDP-D-Xylose to UDP-L-Arabinose in vitro, the nucleotide sugar used by glycosyltransferases in the arabinosylation of cell wall polysaccharides and wall-resident proteoglycans.

AT1G07220	*0,65*		FUNCTIONS IN: molecular_function unknown; INVOLVED IN: biological_process unknown; LOCATED IN: mitochondrion; EXPRESSED IN: 22 plant structures; EXPRESSED DURING: 13 growth stages; CONTAINS InterPro DOMAIN/s: Lipopolysaccharide-modifying protein (InterPro:IPR006598), Protein of unknown function DUF821, CAP10-like (InterPro:IPR008539); BEST Arabidopsis thaliana protein match is: Arabidopsis thaliana protein of unknown function (DUF821) (TAIR:AT5G23850.1)
AT1G68550	*0,65*	CYTOKININ RESPONSE FACTOR 10 (CRF10)	encodes a member of the ERF (ethylene response factor) subfamily B-6 of ERF/AP2 transcription factor family. The protein contains one AP2 domain. There are 12 members in this subfamily including RAP2.11.
AT5G59890	*0,65*	ACTIN DEPOLYMERIZING FACTOR 4 (ADF4)	actin depolymerizing factor 4 (ADF4) mRNA, complete cds
AT5G67210	*0,65*	IRX15-LIKE (IRX15-L)	Encode a DUF579 (domain of unknown function 579) containing protein essential for normal xylan synthesis and deposition in the secondary cell wall.
AT5G63420	*0,649*	EMBRYO DEFECTIVE 2746 (emb2746)	Encodes a member of the metallo-beta-lactamase protein family that plays a vital role in embryo morphogenesis and apical-basal pattern formation by regulating chloroplast development. In bacteria, RNase J plays an important role in rRNA maturation and in the 5′ stability of mRNA.
AT1G09340	*0,648*	CHLOROPLAST RNA BINDING (CRB)	Encodes CHLOROPLAST RNA BINDING (CRB), a putative RNA-binding protein. CRB is important for the proper functioning of the chloroplast. Mutations in CRB also affects the circadian system, altering the expression of both oscillator and output genes. The mRNA is cell-to-cell mobile.
AT5G60030	*0,648*		unknown protein; BEST Arabidopsis thaliana protein match is: unknown protein (TAIR:AT1G75335.1)
AT5G48610	*0,647*		unknown protein
AT1G78060	*0,646*		Glycosyl hydrolase family protein; FUNCTIONS IN: hydrolase activity, hydrolyzing O-glycosyl compounds; INVOLVED IN: carbohydrate metabolic process; LOCATED IN: apoplast, cell wall, chloroplast, plant-type cell wall; EXPRESSED IN: 22 plant structures; EXPRESSED DURING: 13 growth stages; CONTAINS InterPro DOMAIN/s: Glycoside hydrolase, family 3, N-terminal (InterPro:IPR001764), Glycoside hydrolase, family 3, C-terminal (InterPro:IPR002772), Glycoside hydrolase, catalytic core (InterPro:IPR017853); BEST Arabidopsis thaliana protein match is: Glycosyl hydrolase family protein (TAIR:AT5G10560.1)
AT1G50250	*0,643*	FTSH PROTEASE 1 (FTSH1)	encodes an FTSH protease that is localized to the chloroplast. Involved in the D1 repair cycle of Photosystem II. FtsH1 and FtsH5 are interchangeable in thylakoid membranes.
AT3G29200	*0,642*	CHORISMATE MUTASE 1 (CM1)	L-ascorbate peroxidase
AT4G17260	*0,642*		Lactate/malate dehydrogenase family protein; FUNCTIONS IN: in 6 functions; INVOLVED IN: response to salt stress, response to abscisic acid stimulus; LOCATED IN: plasma membrane; EXPRESSED IN: 24 plant structures; EXPRESSED DURING: 13 growth stages; CONTAINS InterPro DOMAIN/s: L-lactate dehydrogenase (InterPro:IPR011304), Lactate/malate dehydrogenase, C-terminal (InterPro:IPR022383), NAD(P)-binding domain (InterPro:IPR016040), L-lactate/malate dehydrogenase (InterPro:IPR001557), Lactate/malate dehydrogenase, N-terminal (InterPro:IPR001236), L-lactate dehydrogenase, active site (InterPro:IPR018177), Lactate dehydrogenase/glycoside hydrolase, family 4, C-terminal (InterPro:IPR015955); BEST Arabidopsis thaliana protein match is: peroxisomal NAD-malate dehydrogenase 1 (TAIR:AT2G22780.1)
AT1G72790	*0,641*		hydroxyproline-rich glycoprotein family protein; FUNCTIONS IN: molecular_function unknown; INVOLVED IN: biological_process unknown; LOCATED IN: cytosol, nucleus, plasma membrane; EXPRESSED IN: 14 plant structures; EXPRESSED DURING: 9 growth stages; BEST Arabidopsis thaliana protein match is: hydroxyproline-rich glycoprotein family protein (TAIR:AT5G57070.1)
AT1G30320	*0,64*		Remorin family protein; FUNCTIONS IN: DNA binding; LOCATED IN: plasma membrane; CONTAINS InterPro DOMAIN/s: Remorin, C-terminal (InterPro:IPR005516); BEST Arabidopsis thaliana protein match is: Remorin family protein (TAIR:AT2G02170.2)
AT1G72320	*0,639*	PUMILIO 23 (PUM23)	Encodes a member of the Arabidopsis Pumilio (APUM) proteins containing PUF domain (eight repeats of approximately 36 amino acids each). PUF proteins regulate both mRNA stability and translation through sequence-specific binding to the 3' UTR of target mRNA transcripts.

AT2G22910	*0,639*	N-ACETYL-L-GLUTAMATE SYNTHASE 1 (NAGS1)	N-acetyl-l-glutamate synthase 1 (NAGS1); FUNCTIONS IN: acetyl-CoA:L-glutamate N-acetyltransferase activity, N-acetyltransferase activity; INVOLVED IN: cellular amino acid biosynthetic process, arginine biosynthetic process, metabolic process; LOCATED IN: cytoplasm; EXPRESSED IN: 23 plant structures; EXPRESSED DURING: 13 growth stages; CONTAINS InterPro DOMAIN/s: GCN5-related N-acetyltransferase, C-terminal (InterPro:IPR022610), GCN5-related N-acetyltransferase (InterPro:IPR000182), Aspartate/glutamate/uridylate kinase (InterPro:IPR001048), Acyl-CoA N-acyltransferase (InterPro:IPR016181), Amino-acid N-acetyltransferase (ArgA) (InterPro:IPR010167); BEST Arabidopsis thaliana protein match is: N-acetyl-l-glutamate synthase 2 (TAIR:AT4G37670.2)
AT5G37500	*0,639*	GATED OUTWARDLY-RECTIFYING K+ CHANNEL (GORK)	Encodes a guard cell outward potassium channel. Belongs to the Shaker family K+ channel. This family includes five groups based on phylogenetic analysis (FEBS Letters (2007) 581: 2357): I (inward rectifying channel): AKT1 (AT2G26650), AKT5 (AT4G32500) and SPIK (also known as AKT6, AT2G25600); II (inward rectifying channel): KAT1 (AT5G46240) and KAT2 (AT4G18290); III (weakly inward rectifying channel): AKT2 (AT4G22200); IV (regulatory subunit involved in inwardly rectifying conductance formation): KAT3 (also known as AtKC1, AT4G32650); V (outward rectifying channel): SKOR (AT3G02850) and GORK (AT5G37500). Mutants have increased water consumption and limited stomatal closure in response to abscisic and jasmonic acids. It forms a heteromeric K(out) channels with SKOR. The gene is expressed ubiquitously in root and the vasculature and guard cells of leaves. Expression is suppressed during agrobacterium-induced tumor formation and increased in response to water deprivation and cold.
AT1G09830	*0,637*	PURINE BIOSYNTHESIS 2 (PUR2)	glycinamide ribonucleotide synthetase (GAR synthetase) that catalyzes the conversion of phosphoribosyl amine to phosphoribosyl glycineamide
AT2G29690	*0,637*	ANTHRANILATE SYNTHASE 2 (ASA2)	Encode a functional anthranilate synthase protein. Expressed at a constitutive basal level. Expression was not induced by wounding nor bacterial pathogen infiltration. Involved in aromatic amino acid biosynthesis.
AT1G54350	*0,636*	ATP-BINDING CASSETTE D2 (ABCD2)	ABC transporter family protein; FUNCTIONS IN: ATPase activity, coupled to transmembrane movement of substances; INVOLVED IN: transport, transmembrane transport; LOCATED IN: chloroplast; EXPRESSED IN: 23 plant structures; EXPRESSED DURING: 14 growth stages; CONTAINS InterPro DOMAIN/s: ATPase, AAA+ type, core (InterPro:IPR003593), ABC transporter, N-terminal (InterPro:IPR010509), ABC transporter-like (InterPro:IPR003439), ABC transporter, transmembrane domain, type 1 (InterPro:IPR011527), ABC transporter integral membrane type 1 (InterPro:IPR017940), ABC transporter, conserved site (InterPro:IPR017871); BEST Arabidopsis thaliana protein match is: peroxisomal ABC transporter 1 (TAIR:AT4G39850.3)
AT1G52230	*0,635*	PHOTOSYSTEM I SUBUNIT H2 (PSAH2)	Phosphorylation of this protein is dependent on calcium. The mRNA is cell-to-cell mobile.
AT5G05160	*0,635*	REDUCED IN LATERAL GROWTH1 (RUL1)	Encodes a receptor-like kinase that activates secondary growth, the production of secondary vascular tissues.
AT1G55490	*0,633*	CHAPERONIN 60 BETA (CPN60B)	encodes the beta subunit of the chloroplast chaperonin 60, a homologue of bacterial GroEL. Mutants in this gene develops lesions on its leaves, expresses systemic acquired resistance (SAR) and develops accelerated cell death to heat shock stress. The protein has molecular chaperone activity for suppressing protein aggregation in vitro.
AT2G47710	*0,632*		Adenine nucleotide alpha hydrolases-like superfamily protein; FUNCTIONS IN: molecular_function unknown; INVOLVED IN: response to stress; LOCATED IN: vacuole; EXPRESSED IN: 23 plant structures; EXPRESSED DURING: 13 growth stages; CONTAINS InterPro DOMAIN/s: UspA (InterPro:IPR006016), Rossmann-like alpha/beta/alpha sandwich fold (InterPro:IPR014729), Universal stress protein A (InterPro:IPR006015); BEST Arabidopsis thaliana protein match is: unknown protein (TAIR:AT5G49050.1)
AT5G51350	*0,632*	MORE LATERAL GROWTH1 (MOL1)	Encodes a receptor-like kinase that represses secondary growth, the production of secondary vascular tissues.
AT5G20540	*0,631*	BREVIS RADIX-LIKE 4 (BRXL4)	Belongs to five-member BRX gene family. Arabidopsis BRX genes share high levels of similarity among each others, with several conserved domains. The most distinct is BRX domain - highly conserved in all BRX genes among distantly related species. This protein-protein interaction domain is required and sufficient for BRX activity.
AT4G08685	*0,63*	(SAH7)	Encodes a protein, expressed in leaves, with similarity to pollen allergens. The mRNA is cell-to-cell mobile.

AT5G24760	*0,63*		GroES-like zinc-binding dehydrogenase family protein; FUNCTIONS IN: oxidoreductase activity, zinc ion binding; INVOLVED IN: oxidation reduction; LOCATED IN: chloroplast; EXPRESSED IN: 22 plant structures; EXPRESSED DURING: 13 growth stages; CONTAINS InterPro DOMAIN/s: GroES-like (InterPro:IPR011032), Polyketide synthase, enoylreductase (InterPro:IPR020843), Alcohol dehydrogenase GroES-like (InterPro:IPR013154), Alcohol dehydrogenase, zinc-containing, conserved site (InterPro:IPR002328), Alcohol dehydrogenase, C-terminal (InterPro:IPR013149), Alcohol dehydrogenase superfamily, zinc-containing (InterPro:IPR002085); BEST Arabidopsis thaliana protein match is: alcohol dehydrogenase 1 (TAIR:AT1G77120.1)
AT2G44210	*0,629*		Protein of Unknown Function (DUF239); INVOLVED IN: biological_process unknown; LOCATED IN: endomembrane system; EXPRESSED IN: 22 plant structures; EXPRESSED DURING: 14 growth stages; CONTAINS InterPro DOMAIN/s: Protein of unknown function DUF239, plant (InterPro:IPR004314); BEST Arabidopsis thaliana protein match is: Protein of Unknown Function (DUF239) (TAIR:AT1G55360.1)
AT5G57120	*0,629*		FUNCTIONS IN: molecular_function unknown; INVOLVED IN: biological_process unknown; LOCATED IN: nucleolus; EXPRESSED IN: 21 plant structures; EXPRESSED DURING: 13 growth stages; CONTAINS InterPro DOMAIN/s: LisH dimerisation motif (InterPro:IPR006594), SRP40, C-terminal (InterPro:IPR007718)
AT5G57610	*0,629*		Protein kinase superfamily protein with octicosapeptide/Phox/Bem1p domain; FUNCTIONS IN: protein serine/threonine/tyrosine kinase activity, protein kinase activity; INVOLVED IN: protein amino acid phosphorylation; LOCATED IN: cellular_component unknown; EXPRESSED IN: 24 plant structures; EXPRESSED DURING: 15 growth stages; CONTAINS InterPro DOMAIN/s: Octicosapeptide/Phox/Bem1p (InterPro:IPR000270), Protein kinase, ATP binding site (InterPro:IPR017441), Protein kinase, catalytic domain (InterPro:IPR000719), Serine-threonine/tyrosine-protein kinase (InterPro:IPR001245), Protein kinase-like domain (InterPro:IPR011009), Serine/threonine-protein kinase, active site (InterPro:IPR008271); BEST Arabidopsis thaliana protein match is: Protein kinase superfamily protein with octicosapeptide/Phox/Bem1p domain (TAIR:AT1G16270.2)
AT5G17910	*0,628*		unknown protein; FUNCTIONS IN: molecular_function unknown; INVOLVED IN: biological_process unknown; LOCATED IN: mitochondrion; EXPRESSED IN: 23 plant structures; EXPRESSED DURING: 15 growth stages; BEST Arabidopsis thaliana protein match is: unknown protein (TAIR:AT2G29620.1)
AT1G62710	*0,626*	BETA VACUOLAR PROCESSING ENZYME (BETA-VPE)	Encodes a vacuolar processing enzyme belonging to a novel group of cysteine proteases that is expressed specifically in seeds and is essential for the proper processing of storage proteins.
AT4G22890	*0,626*	(PGR5-LIKE A)	Encodes PGRL1A, a transmembrane protein present in thylakoids. PGRL1A has a highly homologous isoform PGRL1B encoded by At4g11960. Plants lacking PGRL1 show perturbation of cyclic electron flow, similar to PGR5-deficient plants. PGRL1 and PGR5 interact physically and associate with PSI (photosystem I). The mRNA is cell-to-cell mobile.
AT5G40910	*0,624*		Disease resistance protein (TIR-NBS-LRR class) family; FUNCTIONS IN: transmembrane receptor activity, nucleoside-triphosphatase activity, nucleotide binding, ATP binding; INVOLVED IN: signal transduction, apoptosis, defense response, innate immune response; LOCATED IN: intrinsic to membrane; EXPRESSED IN: 21 plant structures; EXPRESSED DURING: 13 growth stages; CONTAINS InterPro DOMAIN/s: ATPase, AAA+ type, core (InterPro:IPR003593), NB-ARC (InterPro:IPR002182), Leucine-rich repeat (InterPro:IPR001611), Toll-Interleukin receptor (InterPro:IPR000157), Disease resistance protein (InterPro:IPR000767); BEST Arabidopsis thaliana protein match is: Disease resistance protein (TIR-NBS-LRR class) family (TAIR:AT5G41550.1)
AT1G17620	*0,623*		Late embryogenesis abundant (LEA) hydroxyproline-rich glycoprotein family; FUNCTIONS IN: molecular_function unknown; INVOLVED IN: biological_process unknown; LOCATED IN: plasma membrane; EXPRESSED IN: 23 plant structures; EXPRESSED DURING: 13 growth stages; CONTAINS InterPro DOMAIN/s: Late embryogenesis abundant protein, group 2 (InterPro:IPR004864); BEST Arabidopsis thaliana protein match is: unknown protein (TAIR:AT5G11890.1)
AT1G18670	*0,623*	IMPAIRED IN BABA-INDUCED STERILITY 1 (IBS1)	Encodes a cyclin-dependent kinase-like protein with a ser/thr protein kinase domain and an N-terminal myristoylation sequence. Mutants in this gene are unable to express female sterility in response to beta-aminobutyric acid, as wild type plants do.
AT1G28100	*0,622*		unknown protein; FUNCTIONS IN: molecular_function unknown; INVOLVED IN: biological_process unknown; LOCATED IN: cellular_component unknown; EXPRESSED IN: 22 plant structures; EXPRESSED DURING: 13 growth stages

AT1G08380	*0,621*	PHOTOSYSTEM I SUBUNIT O (PSAO)	Encodes subunit O of photosystem I.
AT1G34430	*0,621*	EMBRYO DEFECTIVE 3003 (EMB3003)	embryo defective 3003 (EMB3003); FUNCTIONS IN: dihydrolipoyllysine-residue acetyltransferase activity, acyltransferase activity; INVOLVED IN: embryo development ending in seed dormancy; LOCATED IN: cytosolic ribosome, plasma membrane, chloroplast, chloroplast envelope; EXPRESSED IN: 23 plant structures; EXPRESSED DURING: 15 growth stages; CONTAINS InterPro DOMAIN/s: 2-oxo acid dehydrogenase, lipoyl-binding site (InterPro:IPR003016), E3 binding (InterPro:IPR004167), 2-oxoacid dehydrogenase acyltransferase, catalytic domain (InterPro:IPR001078), Single hybrid motif (InterPro:IPR011053), Biotin/lipoyl attachment (InterPro:IPR000089); BEST Arabidopsis thaliana protein match is: 2-oxoacid dehydrogenases acyltransferase family protein (TAIR:AT3G25860.1)
AT4G09760	*0,62*	CHOLINE/ETHANOLAMINE KINASE 3 (CEK3)	encodes a choline synthase whose gene expression is induced by high salt and mannitol.
AT5G44670	*0,62*	GALACTAN SYNTHASE 2 (GALS2)	Domain of unknown function (DUF23); CONTAINS InterPro DOMAIN/s: Protein of unknown function DUF23 (InterPro:IPR008166); BEST Arabidopsis thaliana protein match is: Domain of unknown function (DUF23) (TAIR:AT4G20170.1)
AT1G31800	*0,619*	CYTOCHROME P450, FAMILY 97, SUBFAMILY A, POLYPEPTIDE 3 (CYP97A3)	Encodes a protein with β-ring carotenoid hydroxylase activity. The mRNA is cell-to-cell mobile.
AT2G13790	*0,619*	SOMATIC EMBRYOGENESIS RECEPTOR-LIKE KINASE 4 (SERK4)	somatic embryogenesis receptor-like kinase 4 (SERK4); FUNCTIONS IN: protein kinase activity, transmembrane receptor protein serine/threonine kinase activity; INVOLVED IN: in 6 processes; LOCATED IN: endomembrane system; EXPRESSED IN: rosette leaf, cauline leaf, fruit, root, flower; CONTAINS InterPro DOMAIN/s: Protein kinase, ATP binding site (InterPro:IPR017441), Protein kinase, catalytic domain (InterPro:IPR000719), Leucine-rich repeat-containing N-terminal domain, type 2 (InterPro:IPR013210), Leucine-rich repeat (InterPro:IPR001611), Serine/threonine-protein kinase-like domain (InterPro:IPR017442), Protein kinase-like domain (InterPro:IPR011009), Serine/threonine-protein kinase, active site (InterPro:IPR008271); BEST Arabidopsis thaliana protein match is: somatic embryogenesis receptor-like kinase 5 (TAIR:AT2G13800.1)
AT3G21270	*0,619*	DOF ZINC FINGER PROTEIN 2 (DOF2)	Encodes Dof zinc finger protein adof2.
AT5G49910	*0,619*	CHLOROPLAST HEAT SHOCK PROTEIN 70-2 (cpHsc70-2)	Stromal heat shock protein involved in protein import into chloroplast. The mRNA is cell-to-cell mobile.
AT5G55040	*0,619*		DNA-binding bromodomain-containing protein; FUNCTIONS IN: DNA binding; INVOLVED IN: biological_process unknown; LOCATED IN: mitochondrion; EXPRESSED IN: 23 plant structures; EXPRESSED DURING: 13 growth stages; CONTAINS InterPro DOMAIN/s: Bromodomain, conserved site (InterPro:IPR018359), Bromodomain (InterPro:IPR001487); BEST Arabidopsis thaliana protein match is: DNA-binding bromodomain-containing protein (TAIR:AT1G20670.1)
AT4G24050	*0,618*		NAD(P)-binding Rossmann-fold superfamily protein; FUNCTIONS IN: oxidoreductase activity, binding, catalytic activity; INVOLVED IN: oxidation reduction, metabolic process; EXPRESSED IN: 15 plant structures; EXPRESSED DURING: 7 growth stages; CONTAINS InterPro DOMAIN/s: NAD(P)-binding domain (InterPro:IPR016040), Glucose/ribitol dehydrogenase (InterPro:IPR002347), Short-chain dehydrogenase/reductase SDR (InterPro:IPR002198); BEST Arabidopsis thaliana protein match is: NAD(P)-binding Rossmann-fold superfamily protein (TAIR:AT1G64590.1)
AT3G55560	*0,615*	AT-HOOK MOTIF NUCLEAR-LOCALIZED PROTEIN 15 (AHL15)	AT-hook protein of GA feedback 2 (AGF2); INVOLVED IN: biological_process unknown; LOCATED IN: chloroplast; EXPRESSED IN: 19 plant structures; EXPRESSED DURING: 6 growth stages; CONTAINS InterPro DOMAIN/s: Protein of unknown function DUF296 (InterPro:IPR005175), Predicted AT-hook DNA-binding (InterPro:IPR014476); BEST Arabidopsis thaliana protein match is: AT-hook motif nuclear-localized protein 20 (TAIR:AT4G14465.1)
AT3G63410	*0,615*	ALBINO OR PALE GREEN MUTANT 1 (APG1)	Encodes a MPBQ/MSBQ methyltransferase located in the chloroplast inner envelope membrane. Mutant plants lack plastoquinone (PQ), suggesting that the APG1 protein is involved in the methylation step of PQ biosynthesis. The gene product is also involved in tocopherol (vitamin E) biosynthesis.

AT4G09650	*0,615*	ATP SYNTHASE DELTA-SUBUNIT GENE (ATPD)	Encodes the chloroplast ATPase delta-subunit. The mRNA is cell-to-cell mobile.
AT4G32880	*0,615*	HOMEOBOX GENE 8 (HB-8)	member of homeodomain-leucine zipper family, acting as a differentiation-promoting transcription factor of the vascular meristems.
AT4G34160	*0,615*	CYCLIN D3;1 (CYCD3;1)	encodes a cyclin D-type protein involved in the switch from cell proliferation to the final stages of differentiation. The gene is transcriptionally regulated by cytokinin and brassinosteroid. Protein interacts with cyclin-dependent kinase inhibitor ICK1.
AT5G14920	*0,613*	A-STIMULATED IN ARABIDOPSIS 14 (GASA14)	Encodes a GASA domain containing protein that regulates increases in plant growth through GA-induced and DELLA-dependent signal transduction and that can increase abiotic stress resistance by reducing ROS accumulation.
AT5G16450	*0,613*		Ribonuclease E inhibitor RraA/Dimethylmenaquinone methyltransferase; FUNCTIONS IN: ribonuclease inhibitor activity; INVOLVED IN: regulation of RNA metabolic process; LOCATED IN: cellular_component unknown; EXPRESSED IN: 24 plant structures; EXPRESSED DURING: 13 growth stages; CONTAINS InterPro DOMAIN/s: Ribonuclease E inhibitor RraA/Dimethylmenaquinone methyltransferase (InterPro:IPR005493), Ribonuclease E inhibitor RraA (InterPro:IPR010203); BEST Arabidopsis thaliana protein match is: Ribonuclease E inhibitor RraA/Dimethylmenaquinone methyltransferase (TAIR:AT3G02770.1)
AT4G35550	*0,612*	WUSCHEL RELATED HOMEOBOX 13 (WOX13)	Encodes a WUSCHEL-related homeobox gene family member with 65 amino acids in its homeodomain. WOX13 is the only family member that does not contain a sequence of eight residues (TLPLFPMH) downstream of the homeodomain called the WUS box.
AT5G50150	*0,611*		Protein of Unknown Function (DUF239); CONTAINS InterPro DOMAIN/s: Protein of unknown function DUF239, plant (InterPro:IPR004314); BEST Arabidopsis thaliana protein match is: Protein of Unknown Function (DUF239) (TAIR:AT1G23340.2)
AT1G16530	*0,61*	ASYMMETRIC LEAVES 2-LIKE 9 (ASL9)	ASYMMETRIC LEAVES 2-like 9 (ASL9); CONTAINS InterPro DOMAIN/s: Lateral organ boundaries, LOB (InterPro:IPR004883); BEST Arabidopsis thaliana protein match is: Lateral organ boundaries (LOB) domain family protein (TAIR:AT2G30130.1)
AT1G74250	*0,61*		DNAJ heat shock N-terminal domain-containing protein; FUNCTIONS IN: heat shock protein binding, zinc ion binding, nucleic acid binding; INVOLVED IN: protein folding; LOCATED IN: intracellular; EXPRESSED IN: 22 plant structures; EXPRESSED DURING: 13 growth stages; CONTAINS InterPro DOMAIN/s: Zinc finger, C2H2-like (InterPro:IPR015880), Molecular chaperone, heat shock protein, Hsp40, DnaJ (InterPro:IPR015609), Zinc finger, U1-type (InterPro:IPR003604), Heat shock protein DnaJ, N-terminal (InterPro:IPR001623), Zinc finger, C2H2-type (InterPro:IPR007087), Zinc finger, double-stranded RNA binding (InterPro:IPR022755), Heat shock protein DnaJ, conserved site (InterPro:IPR018253); BEST Arabidopsis thaliana protein match is: DnaJ domain ;Myb-like DNA-binding domain (TAIR:AT3G11450.1)
AT2G46710	*0,61*	ROP GUANOSINE TRIPHOSPHATASE (GTPASE)-ACTIVATING PROTEIN 3 (ROPGAP3)	Rho GTPase activating protein with PAK-box/P21-Rho-binding domain; FUNCTIONS IN: Rac GTPase activator activity; INVOLVED IN: signal transduction; LOCATED IN: intracellular; EXPRESSED IN: 22 plant structures; EXPRESSED DURING: 13 growth stages; CONTAINS InterPro DOMAIN/s: PAK-box/P21-Rho-binding (InterPro:IPR000095), Rho GTPase activation protein (InterPro:IPR008936), RhoGAP (InterPro:IPR000198); BEST Arabidopsis thaliana protein match is: Rho GTPase activating protein with PAK-box/P21-Rho-binding domain (TAIR:AT4G03100.1)
AT2G47260	*0,608*	WRKY DNA-BINDING PROTEIN 23 (WRKY23)	Encodes a member of WRKY Transcription Factor; Group I. Involved in nematode feeding site establishment.
AT4G31350	*0,608*		Core-2/I-branching beta-1,6-N-acetylglucosaminyltransferase family protein; CONTAINS InterPro DOMAIN/s: Core-2/I-Branching enzyme (InterPro:IPR021141); BEST Arabidopsis thaliana protein match is: Core-2/I-branching beta-1,6-N-acetylglucosaminyltransferase family protein (TAIR:AT2G19160.1)
AT5G67470	*0,608*	FORMIN HOMOLOG 6 (FH6)	formin homolog 6 (FH6); FUNCTIONS IN: actin binding; INVOLVED IN: cellular component organization, actin cytoskeleton organization; LOCATED IN: in 7 components; EXPRESSED IN: 23 plant structures; EXPRESSED DURING: 13 growth stages; CONTAINS InterPro DOMAIN/s: Actin-binding FH2/DRF autoregulatory (InterPro:IPR003104), Actin-binding FH2 (InterPro:IPR015425); BEST Arabidopsis thaliana protein match is: formin homology 1 (TAIR:AT3G25500.1)

AT2G35490	*0,606*		Plastid-lipid associated protein PAP / fibrillin family protein; FUNCTIONS IN: structural molecule activity; INVOLVED IN: biological_process unknown; LOCATED IN: thylakoid, chloroplast thylakoid membrane, chloroplast, plastoglobule; EXPRESSED IN: 24 plant structures; EXPRESSED DURING: 13 growth stages; CONTAINS InterPro DOMAIN/s: Plastid lipid-associated protein/fibrillin (InterPro:IPR006843); BEST Arabidopsis thaliana protein match is: fibrillin (TAIR:AT4G04020.1)
AT4G30210	*0,604*	P450 REDUCTASE 2 (ATR2)	Encodes NADPH-cytochrome P450 reductase that catalyzes the first oxidative step of the phenylpropanoid general pathway. The mRNA is cell-to-cell mobile.
AT4G16890	*0,603*	SUPPRESSOR OF NPR1-1, CONSTITUTIVE 1 (SNC1)	Encodes a Toll Interleukin1 receptor-nucleotide binding-Leu- rich repeat-type resistance gene (TIR-NB-LRR-type) involved in the salicylic acid-dependent defense response pathway. Mutant plants constitutively express pathogenesis-related (PR) genes and are pathogen resistant. Resistance signaling in snc1 requires EDS1, MOS3 and PAD4.
AT2G13560	*0,602*	NAD-DEPENDENT MALIC ENZYME 1 (NAD-ME1)	Encodes an NAD-dependent malic enzyme (NAD-ME) that does not act on oxaloacetate, indicating that it belongs to EC 1.1.1.39. It is a member of the alpha family of NAD-MEs in plants. It appears to function as a homodimer or as a heterodimer with the beta-type NAD-ME2 (At4g00570). NAD-ME1 transcript and protein levels are higher during the night than during the day. The mRNA is cell-to-cell mobile.
AT2G38380	*0,602*		Peroxidase superfamily protein; FUNCTIONS IN: peroxidase activity, heme binding; INVOLVED IN: response to zinc ion, response to salt stress; LOCATED IN: plant-type cell wall; EXPRESSED IN: stem, root; CONTAINS InterPro DOMAIN/s: Haem peroxidase (InterPro:IPR010255), Plant peroxidase (InterPro:IPR000823), Peroxidases heam-ligand binding site (InterPro:IPR019793), Peroxidase, active site (InterPro:IPR019794), Haem peroxidase, plant/fungal/bacterial (InterPro:IPR002016); BEST Arabidopsis thaliana protein match is: Peroxidase superfamily protein (TAIR:AT2G38390.1)
AT5G08260	*0,601*	SERINE CARBOXYPEPTIDASE-LIKE 35 (scpl35)	serine carboxypeptidase-like 35 (scpl35); FUNCTIONS IN: serine-type carboxypeptidase activity; INVOLVED IN: proteolysis; LOCATED IN: endomembrane system; EXPRESSED IN: 22 plant structures; EXPRESSED DURING: 13 growth stages; CONTAINS InterPro DOMAIN/s: Peptidase S10, serine carboxypeptidase (InterPro:IPR001563), Peptidase S10, serine carboxypeptidase, active site (InterPro:IPR018202); BEST Arabidopsis thaliana protein match is: serine carboxypeptidase-like 34 (TAIR:AT5G23210.1)
AT5G26742	*0,601*	EMBRYO DEFECTIVE 1138 (emb1138)	embryo defective 1138 (emb1138); FUNCTIONS IN: in 6 functions; INVOLVED IN: embryo development ending in seed dormancy; LOCATED IN: chloroplast, membrane; EXPRESSED IN: guard cell, leaf; CONTAINS InterPro DOMAIN/s: DNA/RNA helicase, DEAD/DEAH box type, N-terminal (InterPro:IPR011545), GUCT (InterPro:IPR012562), RNA helicase, DEAD-box type, Q motif (InterPro:IPR014014), DEAD-like helicase, N-terminal (InterPro:IPR014001), DNA/RNA helicase, C-terminal (InterPro:IPR001650), Zinc finger, CCHC-type (InterPro:IPR001878), Helicase, superfamily 1/2, ATP-binding domain (InterPro:IPR014021); BEST Arabidopsis thaliana protein match is: putative mitochondrial RNA helicase 2 (TAIR:AT3G22330.1)
AT5G51550	*0,601*	EXORDIUM LIKE 3 (EXL3)	EXORDIUM like 3 (EXL3); CONTAINS InterPro DOMAIN/s: Phosphate-induced protein 1 (InterPro:IPR006766); BEST Arabidopsis thaliana protein match is: EXORDIUM like 5 (TAIR:AT2G17230.1)
AT3G48560	*0,6*	CHLORSULFURON/IMIDAZOLINONE RESISTANT 1 (CSR1)	Catalyzes the formation of acetolactate from pyruvate, the first step in valine and isoleucine biosynthesis. Requires FAD, thiamine pyrophosphate and Mg. Inhibited by the sulphonylurea herbicide, chlorsulphuron, and the imidazolinone herbicide, imazapyr. The obtained crystal structure of acetohydroxyacid synthase AHAS, EC 2.2.1.6)in complex with herbicides of the sulphonylurea and imidazolinone family reveals the molecular basis for substrate/inhibitor binding.
AT1G07200	*0,599*	SMAX1-LIKE 6 (SMXL6)	Encodes a member of an eight-gene family (SMAX1 and SMAX1-like) that has weak similarity to AtHSP101, a ClpB chaperonin required for thermotolerance.
AT5G65020	*0,599*	ANNEXIN 2 (ANNAT2)	Annexins are calcium binding proteins that are localized in the cytoplasm. When cytosolic Ca2+ increases, they relocate to the plasma membrane. They may be involved in the Golgi-mediated secretion of polysaccharides.
AT4G36020	*0,598*	COLD SHOCK DOMAIN PROTEIN 1 (CSDP1)	Encodes a cold shock domain protein. Involved in cold acclimation by blocking the secondary structure of mRNA which in turn facilitates translation at cold temperature.
AT1G58280	*0,596*		Phosphoglycerate mutase family protein; LOCATED IN: chloroplast; CONTAINS InterPro DOMAIN/s: Histidine phosphatase superfamily, clade-1 (InterPro:IPR013078); BEST Arabidopsis thaliana protein match is: Phosphoglycerate mutase family protein (TAIR:AT5G64460.8)

AT2G45300	*0,596*		encodes 3-phosphoshikimate 1-carboxyvinyltransferase / 5-enolpyruvylshikimate-3-phosphate / EPSP synthase involved in chorismate biosynthesis The mRNA is cell-to-cell mobile.
AT1G69830	*0,594*	ALPHA-AMYLASE-LIKE 3 (AMY3)	Encodes a plastid-localized α-amylase. Expression is reduced in the SEX4 mutant. Loss of function mutations show normal diurnal pattern of starch accumulation/degradation. Expression follows circadian rhythms.
AT2G42600	*0,594*	PHOSPHOENOLPYRUVATE CARBOXYLASE 2 (PPC2)	Encodes one of four Arabidopsis phosphoenolpyruvate carboxylase proteins.PPC1 and PPC2 are crucial for balancing carbon and nitrogen metabolism.
AT1G79820	*0,593*	SUPPRESSOR OF G PROTEIN BETA1 (SGB1)	SUPPRESSOR OF G PROTEIN BETA1 (SGB1); FUNCTIONS IN: carbohydrate transmembrane transporter activity, sugar:hydrogen symporter activity; INVOLVED IN: transport, transmembrane transport; LOCATED IN: integral to membrane, membrane; EXPRESSED IN: 20 plant structures; EXPRESSED DURING: 13 growth stages; CONTAINS InterPro DOMAIN/s: Sugar transporter, conserved site (InterPro:IPR005829), Major facilitator superfamily (InterPro:IPR020846), Sugar/inositol transporter (InterPro:IPR003663), General substrate transporter (InterPro:IPR005828), Major facilitator superfamily, general substrate transporter (InterPro:IPR016196); BEST Arabidopsis thaliana protein match is: Major facilitator superfamily protein (TAIR:AT1G67300.1)
AT5G53890	*0,593*	PHYTOSYLFOKINE-ALPHA RECEPTOR 2 (PSKR2)	Encodes a leucine-rich repeat receptor kinase (LRR-RK) involved in the perception of phytosulfokine (PSK), which is a 5-aa tyrosine-sulfated peptide that primarily promotes cellular proliferation.
AT5G60600	*0,593*	4-HYDROXY-3-METHYLBUT-2-ENYL DIPHOSPHATE SYNTHASE (HDS)	Encodes a chloroplast-localized hydroxy-2-methyl-2-(E)-butenyl 4-diphosphate (HMBPP) synthase (HDS), catalyzes the formation of HMBPP from 2-C-methyl-D-erythrytol 2,4-cyclodiphosphate (MEcPP). The HDS enzyme controls the penultimate steps of the biosynthesis of IPP and dimethylallyl diphosphate (DMAPP) via the MEP pathway and may serve as a metabolic control point for SA-mediated disease resistance. In the light, the electrons required for the reaction catalyzed by HDS are directly provided by the electron flow from photosynthesis via ferredoxin. In the dark however, the enzyme requires an electron shuttle: ferredoxin-NADP⁺ reductase. The mRNA is cell-to-cell mobile.
AT1G23800	*0,592*	ALDEHYDE DEHYDROGENASE 2B7 (ALDH2B7)	Encodes a mitochondrial aldehyde dehydrogenase; nuclear gene for mitochondrial product.
AT5G35700	*0,592*	FIMBRIN5 (FIM5)	Encodes FIMBRIN5, an actin bundling factor required for pollen germination and pollen tube growth. Different members of the fimbrin/plastin family have diverged biochemically during evolution to generate either tight actin bundles or loose networks with distinct biochemical and biophysical properties. FIM4 generates both actin bundles and branched actin filaments whereas FIM5 only generates actin bundles.
AT5G55920	*0,591*	OLIGOCELLULA 2 (OLI2)	Encodes a homolog of the S. cerevisiae Nop2 that is involved in ribosome biogenesis and plays a role on organ size control by promoting cell proliferation and preventing compensation in normal leaf development.
AT1G73370	*0,59*	SUCROSE SYNTHASE 6 (SUS6)	Encodes a protein with sucrose synthase activity (SUS6).
AT4G26900	*0,59*	HIS HF (AT-HF)	encodes a glutamine amidotransferase and cyclase, catalyzes the fifth and sixth steps of the histidine biosynthetic pathway
AT5G66190	*0,59*	FERREDOXIN-NADP(+)-OXIDOREDUCTASE 1 (FNR1)	Encodes a leaf-type ferredoxin:NADP(H) oxidoreductase. It is present in both chloroplast stroma and thylakoid membranes but is more abundant in the thylakoid. The affinity of this enzyme for ferredoxin is slightly, but significantly, higher than AtLFNR2, an isoform of the same enzyme. AtLFNR1 forms a heterodimer with AtFNR2 and is also a prerequisite to attach AtFNR2 to the thylakoid membrane.
AT1G08200	*0,588*	UDP-D-APIOSE/UDP-D-XYLOSE SYNTHASE 2 (AXS2)	Encodes a putative UDP-D-apiose/UPD-D-xylose synthetase.
AT2G30200	*0,588*	EMBRYO DEFECTIVE 3147 (EMB3147)	catalytics;transferases;[acyl-carrier-protein] S-malonyltransferases;binding; FUNCTIONS IN: binding, transferase activity, [acyl-carrier-protein] S-malonyltransferase activity, catalytic activity; INVOLVED IN: fatty acid biosynthetic process, metabolic process; LOCATED IN: chloroplast, chloroplast stroma; EXPRESSED IN: 23 plant structures; EXPRESSED DURING: 14 growth stages; CONTAINS InterPro DOMAIN/s: Acyl transferase/acyl hydrolase/lysophospholipase (InterPro:IPR016035), Malonyl CoA-acyl carrier protein transacylase (InterPro:IPR004410), Acyl transferase domain (InterPro:IPR001227), Acyl transferase (InterPro:IPR014043), Malonyl-CoA ACP transacylase, ACP-binding (InterPro:IPR016036)

AT2G39800	*0,588*	DELTA1-PYRROLINE-5-CARBOXYLATE SYNTHASE 1 (P5CS1)	encodes a delta1-pyrroline-5-carboxylate synthase that catalyzes the rate-limiting enzyme in the biosynthesis of proline. Gene is expressed in reproductive organs and tissues under non-stress conditions but in the whole plant under water-limiting condition. Expression is also induced by abscisic acid and salt stress in a light-dependent manner. encodes a delta1-pyrroline-5-carboxylate synthase that catalyzes the rate-limiting enzyme in the biosynthesis of proline. Gene is expressed in reproductive organs and tissues under non-stress conditions but in the whole plant under water-limiting condition. Expression is also induced by abscisic acid and salt stress in a light-dependent manner. P5CS1 appears to be involved in salt stress responses related to proline accumulation, including protection from reactive oxidative species. P5CS1 appears to be present in different cells and/or different subcellular locations from P5CS2 in a tissue-dependent manner.
AT1G69580	*0,587*		Homeodomain-like superfamily protein; CONTAINS InterPro DOMAIN/s: Homeodomain-like (InterPro:IPR009057), Myb, DNA-binding (InterPro:IPR014778), Myb-like DNA-binding domain, SHAQKYF class (InterPro:IPR006447), Homeodomain-related (InterPro:IPR012287); BEST Arabidopsis thaliana protein match is: Homeodomain-like superfamily protein (TAIR:AT3G04030.3)
AT3G53620	*0,587*	PYROPHOSPHORYLASE 4 (PPa4)	Encodes a soluble protein with inorganic pyrophosphatase activity that is highly specific for Mg-inorganic pyrophosphate. The mRNA is cell-to-cell mobile.
AT5G47810	*0,585*	PHOSPHOFRUCTOKINASE 2 (PFK2)	phosphofructokinase 2 (PFK2); CONTAINS InterPro DOMAIN/s: Pyrophosphate-dependent phosphofructokinase TP0108 (InterPro:IPR012004), Phosphofructokinase (InterPro:IPR000023); BEST Arabidopsis thaliana protein match is: phosphofructokinase 3 (TAIR:AT4G26270.1)
AT2G47940	*0,584*	DEGRADATION OF PERIPLASMIC PROTEINS 2	Encodes DegP2 protease (DEGP2); nuclear gene for chloroplast product.
AT1G55690	*0,583*		Sec14p-like phosphatidylinositol transfer family protein; CONTAINS InterPro DOMAIN/s: Cellular retinaldehyde-binding/triple function, C-terminal (InterPro:IPR001251), Cellular retinaldehyde-binding/triple function, N-terminal (InterPro:IPR008273), Phosphatidylinositol transfer protein-like, N-terminal (InterPro:IPR011074); BEST Arabidopsis thaliana protein match is: Sec14p-like phosphatidylinositol transfer family protein (TAIR:AT5G56160.1)
AT5G43810	*0,583*	ARGONAUTE 10 (AGO10)	Encodes Argonaute10, a member of the EIF2C (elongation initiation factor 2c)/ Argonaute class of proteins. Required to establish the central-peripheral organization of the embryo apex. Along with WUS and CLV genes, controls the relative organization of central zone and peripheral zone cells in meristems. Acts in embryonic provascular tissue potentiating WUSCHEL function during meristem development in the embryo. AGO10 specifically sequesters miR166/165 to regulate shoot apical meristem development.
AT3G57610	*0,582*	ADENYLOSUCCINATE SYNTHASE (ADSS)	encoding adenylosuccinate synthetase (AdSS), the enzyme involved in the first step of the formation of the purine nucleotide AMP (conversion of IMP to adenylo-succinate)
AT1G52150	*0,579*	(ATHB-15)	Member of the class III HD-ZIP protein family. Contains homeodomain and leucine zipper domain. Critical for vascular development and negatively regulates vascular cell differentiation.
AT4G38580	*0,579*	FARNESYLATED PROTEIN 6 (FP6)	putative farnesylated protein (At4g38580) mRNA, complete
AT1G74330	*0,577*		Protein kinase superfamily protein; FUNCTIONS IN: protein serine/threonine kinase activity, protein kinase activity, ATP binding; INVOLVED IN: protein amino acid phosphorylation, N-terminal protein myristoylation; LOCATED IN: cellular_component unknown; EXPRESSED IN: 23 plant structures; EXPRESSED DURING: 14 growth stages; CONTAINS InterPro DOMAIN/s: Protein kinase, ATP binding site (InterPro:IPR017441), Serine/threonine-protein kinase domain (InterPro:IPR002290), Serine/threonine-protein kinase-like domain (InterPro:IPR017442), Protein kinase-like domain (InterPro:IPR011009), Serine/threonine-protein kinase, active site (InterPro:IPR008271), Protein kinase, catalytic domain (InterPro:IPR000719), Tyrosine-protein kinase, catalytic domain (InterPro:IPR020635); BEST Arabidopsis thaliana protein match is: Protein kinase superfamily protein (TAIR:AT1G18670.1)
AT2G24050	*0,576*	EUKARYOTIC TRANSLATION INITIATION FACTOR ISOFORM 4G2 (eIFiso4G2)	Encodes a putative eukaryotic translation initiation factor.

AT5G19440	*0,575*		similar to Eucalyptus gunnii alcohol dehydrogenase of unknown physiological function (GI:1143445), apple tree, PIR:T16995; NOT a cinnamyl-alcohol dehydrogenase
AT3G06550	*0,573*	REDUCED WALL ACETYLATION 2 (RWA2)	Encodes a homolog of the protein Cas1p known to be involved in polysaccharide O-acetylation in Cryptococcus neoformans. Mutants show reduced cell wall polysaccharide acetylation and increased resistance to Botrytis cinerea. The protein is expressed in the Golgi and is involved in the acetylation of xylan during secondary wall biosynthesis.
AT4G01150	*0,573*	CURVATURE THYLAKOID 1A (CURT1A)	unknown protein; FUNCTIONS IN: molecular_function unknown; INVOLVED IN: biological_process unknown; LOCATED IN: thylakoid, chloroplast thylakoid membrane, chloroplast, plastoglobule, chloroplast envelope; EXPRESSED IN: 23 plant structures; EXPRESSED DURING: 14 growth stages; BEST Arabidopsis thaliana protein match is: unknown protein (TAIR:AT4G38100.1)
AT2G39020	*0,57*	(NATA2)	Although this locus shares considerable sequence similarity with the adjacent NATA1 gene (At2g39030), they appear to encode genes with different functions. NATA1 is involved in the production of N-delta-acetylornithine, but, overexpression of At2g39020 in tobacco does not lead to the formation of this defense compound. The mRNA is cell-to-cell mobile.
AT2G28760	*0,569*	UDP-XYL SYNTHASE 6 (UXS6)	UDP-XYL synthase 6 (UXS6); FUNCTIONS IN: coenzyme binding, binding, catalytic activity; INVOLVED IN: cellular metabolic process, nucleotide-sugar metabolic process, metabolic process; LOCATED IN: plasma membrane; EXPRESSED IN: 25 plant structures; EXPRESSED DURING: 13 growth stages; CONTAINS InterPro DOMAIN/s: NAD-dependent epimerase/dehydratase (InterPro:IPR001509), NAD(P)-binding domain (InterPro:IPR016040); BEST Arabidopsis thaliana protein match is: UDP-XYL synthase 5 (TAIR:AT3G46440.2)
AT3G06810	*0,568*	IBA-RESPONSE 3 (IBR3)	Encodes a protein with similarity to acyl-CoA dehydrogenases. Mutations in IBR3 render plants resistant to indole-3-butryic acid, a putative storage form of the biologically active auxin IAA (indole-3-acetic acid). IBR3 is hypothesized to carry out the second step in a β-oxidation-like process of IBA metabolism in Arabidopsis. Though its subcellular location has not been determined, IBR3 has a peroxisomal targeting sequence and two other putative IBA metabolic enzymes (IBR1 and IBR10) can be found in this organelle. No specific enzymatic activity has been documented for IBR3, but double mutant analyses with CHY1 argue against a role for IBR3 in general fatty acid β-oxidation. The mRNA is cell-to-cell mobile.
AT4G02680	*0,566*	ETO1-LIKE 1 (EOL1)	Encodes a paralog of ETO1, which is a negative regulator of ACS5 (a key enzyme in ethylene biosynthesis pathway). EOL1 also interacts with and inhibits the activity of ACS5.
AT5G65970	*0,563*	MILDEW RESISTANCE LOCUS O 10 (MLO10)	A member of a large family of seven-transmembrane domain proteins specific to plants, homologs of the barley mildew resistance locus o (MLO) protein. The Arabidopsis genome contains 15 genes encoding MLO proteins, with localization in plasma membrane. Phylogenetic analysis revealed four clades of closely-related AtMLO genes. ATMLO10 belongs to the clade III, with AtMLO5, AtMLO7, AtMLO8, and AtMLO9. The gene is expressed in root and cotyledon vascular system, in root-shoot junction and lateral root primordia and in developing siliques, as shown by GUS activity patterns. The expression of several phylogenetically closely-related AtMLO genes showed similar or overlapping tissue specificity and analogous responsiveness to external stimuli, suggesting functional redundancy, co-function, or antagonistic function(s
AT1G70730	*0,562*	PHOSPHOGLUCOMUTASE 2 (PGM2)	Encodes a cytosolic phosphoglucomutase (PGM). Two Arabidopsis PGM proteins (AT1G70730/PGM2 and AT1G23190/PGM3) have high sequence similarities and redundant functions. Mature plants possessing a single cPGM allele had a major reduction in cPGM activity. Whereas pgm2 and pgm3 single mutants are undistinguishable from the wild type, loss of both PGM2 and PGM3 severely impairs male and female gametophyte development.
AT5G53570	*0,561*	RAB GTPASE-ACTIVATING PROTEIN 22 (RABGAP22)	Ypt/Rab-GAP domain of gyp1p superfamily protein; FUNCTIONS IN: RAB GTPase activator activity; INVOLVED IN: regulation of Rab GTPase activity; LOCATED IN: intracellular; EXPRESSED IN: 22 plant structures; EXPRESSED DURING: 13 growth stages; CONTAINS InterPro DOMAIN/s: RabGAP/TBC (InterPro:IPR000195); BEST Arabidopsis thaliana protein match is: Ypt/Rab-GAP domain of gyp1p superfamily protein (TAIR:AT3G49350.1)
AT5G64300	*0,561*	GTP CYCLOHYDROLASE II (GCH)	encodes GTP cyclohydrolase II that can functionally complement E. coli mutant deficient in this gene. It also has 3,4-dihydroxy-2-butanone-4-phosphate synthase activity which makes it a bifunctional enzyme involved in the formation of the pyrimidine and of the carbohydrate from GTP and ribulose-5-phosphate, respectively The mRNA is cell-to-cell mobile.
AT1G67230	*0,56*	LITTLE NUCLEI1 (LINC1)	Encodes a nuclear coiled-coil protein related to the carrot peripheral nuclear protein NMCP1 that is involved in the determination of plant nuclear structure.

AT2G31360	*0,56*	16:0DELTA9 DESATURASE 2 (ADS2)	Encodes a protein homologous to delta 9 acyl-lipid desaturases of cyanobacteria and acyl-CoA desaturases of yeast and mammals. expression up-regulated by cold temperature. It is involved in the synthesis of the 24:1n-9 and 26:1n-9 components of seed lipids, sphingolipids and the membrane phospholipids phosphatidylserine (PS), and phosphatidylethanolamine (PE).
AT2G40890	*0,559*	CYTOCHROME P450, FAMILY 98, SUBFAMILY A, POLYPEPTIDE 3 (CYP98A3)	encodes coumarate 3-hydroxylase (C3H), a P450-dependent monooxygenase. Involved in lignin biosynthesis and flavonoid biosynthesis. Also affects the biosynthesis of coumarins such as scopoletin and scopolin as a branching-out-pathway from the phenylpropanoid acid level.
AT1G07890	*0,558*	ASCORBATE PEROXIDASE 1 (APX1)	Encodes a cytosolic ascorbate peroxidase APX1. Ascorbate peroxidases are enzymes that scavenge hydrogen peroxide in plant cells. Eight types of APX have been described for Arabidopsis: three cytosolic (APX1, APX2, APX6), two chloroplastic types (stromal sAPX, thylakoid tAPX), and three microsomal (APX3, APX4, APX5) isoforms. At least part of the induction of heat shock proteins during light stress in Arabidopsis is mediated by H2O2 that is scavenged by APX1. Expression of the gene is downregulated in the presence of paraquat, an inducer of photoxidative stress. The mRNA is cell-to-cell mobile.
AT2G30870	*0,558*	GLUTATHIONE S-TRANSFERASE PHI 10 (GSTF10)	early dehydration-induced gene ERD13 homologous to tobacco and maize glutathione S-transferases. Encodes glutathione transferase belonging to the phi class of GSTs. Naming convention according to Wagner et al. (2002)
AT5G07830	*0,558*	GLUCURONIDASE 2 (GUS2)	Belongs to the plant glycoside hydrolase family 79. Encodes a protein with several posttranslational modification sites including O-β-GlcNAc attachment sites and serine-, threonine- and tyrosine-phosphorylation sites, suggesting that this protein is extensively modified posttranslationally. The protein is predicted (WoLF PSORT program) to be membrane-associated. It is involved in cell elongation. The mRNA is cell-to-cell mobile.
AT5G55180	*0,558*		O-Glycosyl hydrolases family 17 protein; FUNCTIONS IN: cation binding, hydrolase activity, hydrolyzing O-glycosyl compounds, catalytic activity; INVOLVED IN: carbohydrate metabolic process; LOCATED IN: endomembrane system; EXPRESSED IN: 18 plant structures; EXPRESSED DURING: 11 growth stages; CONTAINS InterPro DOMAIN/s: X8 (InterPro:IPR012946), Glycoside hydrolase, catalytic core (InterPro:IPR017853), Glycoside hydrolase, family 17 (InterPro:IPR000490), Glycoside hydrolase, subgroup, catalytic core (InterPro:IPR013781); BEST Arabidopsis thaliana protein match is: O-Glycosyl hydrolases family 17 protein (TAIR:AT4G26830.1)
AT4G37930	*0,557*	SERINE TRANSHYDROXYMETHYLTRANSFERASE 1 (SHM1)	Encodes a protein with mitochondrial serine hydroxymethyltransferase activity, which functions in the photorespiratory pathway, catalyzes the conversion of serine and tetrahydrofolate to glycine and 5,10-methylene tetrahydrofolate. Involved in controlling cell damage caused by abiotic stress, such as high light and salt and the hypersensitive defense response of plants.
AT4G39280	*0,557*		phenylalanyl-tRNA synthetase, putative / phenylalanine--tRNA ligase, putative; FUNCTIONS IN: phenylalanine-tRNA ligase activity, tRNA binding, aminoacyl-tRNA ligase activity, nucleotide binding, ATP binding; INVOLVED IN: phenylalanyl-tRNA aminoacylation, tRNA aminoacylation, translation, tRNA aminoacylation for protein translation; LOCATED IN: cytoplasm; EXPRESSED IN: 23 plant structures; EXPRESSED DURING: 14 growth stages; CONTAINS InterPro DOMAIN/s: Phenylalanyl-tRNA synthetase, class IIc, alpha subunit (InterPro:IPR004529), Phenylalanyl-tRNA synthetase alpha chain (InterPro:IPR002319), Aminoacyl-tRNA synthetase, class II, conserved domain (InterPro:IPR006195); BEST Arabidopsis thaliana protein match is: phenylalanyl-tRNA synthetase class IIc family protein (TAIR:AT3G58140.1)
AT3G54890	*0,554*	PHOTOSYSTEM I LIGHT HARVESTING COMPLEX GENE 1 (LHCA1)	Encodes a component of the light harvesting complex associated with photosystem I.
AT3G18440	*0,552*	ALUMINUM-ACTIVATED MALATE TRANSPORTER 9 (ALMT9)	Belongs to the aluminum-activated malate transporter family. Encodes a vacuolar malate channel. Expressed in all parts of plants. Almost exclusively expressed in mesophyll cells of leaves. The mRNA is cell-to-cell mobile.
AT1G14040	*0,548*	PHO1 HOMOLOG 3 (PHO1;H3)	Encodes a PHO1 homologue that is upregulated in response to Zn deficiency and is involved in Pi homeostasis in response to Zn deficiency. The mRNA is cell-to-cell mobile.
AT3G44550	*0,547*	FATTY ACID REDUCTASE 5 (FAR5)	Encodes a member of the eight-member gene family encoding alcohol-forming fatty acyl-CoA reductases (FARs) identified in Arabidopsis thaliana. Three of the FARs, FAR1 (At5g22500), FAR4 (At3g44540) and FAR5 (At3g44550), are shown to generate the fatty alcohols found in root, seed coat, and wound-induced leaf tissue. The mRNA is cell-to-cell mobile.

AT4G30600	*0,545*		signal recognition particle receptor alpha subunit family protein; FUNCTIONS IN: nucleoside-triphosphatase activity, signal recognition particle binding, GTP binding, GTPase activity, nucleotide binding; INVOLVED IN: intracellular protein transport, protein targeting, SRP-dependent cotranslational protein targeting to membrane; LOCATED IN: endoplasmic reticulum, signal recognition particle, endoplasmic reticulum targeting; EXPRESSED IN: 25 plant structures; EXPRESSED DURING: 15 growth stages; CONTAINS InterPro DOMAIN/s: ATPase, AAA+ type, core (InterPro:IPR003593), Signal recognition particle receptor, alpha subunit, N-terminal (InterPro:IPR007222), Signal recognition particle, SRP54 subunit, helical bundle (InterPro:IPR013822), Signal recognition particle, SRP54 subunit, GTPase (InterPro:IPR000897); BEST Arabidopsis thaliana protein match is: signal recognition particle 54 kDa subunit (TAIR:AT1G15310.1)
AT4G33520	*0,543*	P-TYPE ATP-ASE 1 (PAA1)	Encodes a putative metal-transporting P-type ATPase PAA1. An alternative-splicing event of the PAA1 pre-mRNA produces a copper chaperon named PCH1. The mRNA is cell-to-cell mobile.
AT2G30110	*0,537*	UBIQUITIN-ACTIVATING ENZYME 1 (UBA1)	Encodes a ubiquitin-activating enzyme (E1), involved in the first step in conjugating multiple ubiquitins to proteins targeted for degradation. Gene is expressed in most tissues examined. Mutant is able to revert the constitutive defense responses phenotype of snc1, which indicates the gene is involved in defense response. It also indicates that ubiquitination plays a role in plant defense signalling.
AT3G47520	*0,537*	MALATE DEHYDROGENASE (MDH)	Encodes a protein with NAD-dependent malate dehydrogenase activity, located in chloroplasts. The mRNA is cell-to-cell mobile.
AT5G61410	*0,536*	D-RIBULOSE-5-PHOSPHATE-3-EPIMERASE (RPE)	Arabidopsis thaliana ribulose-5-phosphate-3-epimerase mRNA
AT3G12120	*0,535*	FATTY ACID DESATURASE 2 (FAD2)	Major enzyme responsible for the synthesis of 18:2 fatty acids in the endoplasmic reticulum. Contains His-rich motifs, which contribute to the interaction with the electron donor cytochrome b5. Mutations in this gene suppress the low temperature-induced phenotype of Arabidopsis tocopherol-deficient mutant vte2.
AT4G34640	*-0,531*	SQUALENE SYNTHASE 1 (SQS1)	Encodes squalene synthase, which converts two molecules of farnesyl diphosphate (FPP) into squalene via an intermediate: presqualene diphosphate (PSPP). It is generally thought to be one of the key enzymes of sterol biosynthesis, since it catalyzes the first pathway-specific reaction of the sterol branch of the isoprenoid pathway. The mRNA is cell-to-cell mobile.
AT5G10960	*-0,531*		Polynucleotidyl transferase, ribonuclease H-like superfamily protein; FUNCTIONS IN: ribonuclease activity, nucleic acid binding; INVOLVED IN: RNA modification; LOCATED IN: nucleus; EXPRESSED IN: 25 plant structures; EXPRESSED DURING: 15 growth stages; CONTAINS InterPro DOMAIN/s: Ribonuclease CAF1 (InterPro:IPR006941), Polynucleotidyl transferase, ribonuclease H fold (InterPro:IPR012337); BEST Arabidopsis thaliana protein match is: Polynucleotidyl transferase, ribonuclease H-like superfamily protein (TAIR:AT1G80780.2)
AT5G27150	*-0,534*	NA+/H+ EXCHANGER 1 (NHX1)	Encodes a vacuolar sodium/proton antiporter involved in salt tolerance, ion homeostasis, and leaf development. The mRNA is cell-to-cell mobile.
AT3G15950	*-0,535*	(NAI2)	Similar to TSK-associating protein 1 (TSA1), contains 10 EFE repeats, a novel repeat sequence unique to plants. Expressed preferentially in the roots.Protein is localized to ER bodies- an endoplasmic reticulum derived structure. Loss of function mutations lack ER bodies.
AT1G27090	*-0,536*		glycine-rich protein; BEST Arabidopsis thaliana protein match is: unknown protein (TAIR:AT3G24690.1)
AT2G38390	*-0,541*		Peroxidase superfamily protein; FUNCTIONS IN: peroxidase activity, heme binding; INVOLVED IN: response to salt stress; LOCATED IN: endomembrane system; EXPRESSED IN: root; CONTAINS InterPro DOMAIN/s: Haem peroxidase (InterPro:IPR010255), Plant peroxidase (InterPro:IPR000823), Peroxidases heam-ligand binding site (InterPro:IPR019793), Peroxidase, active site (InterPro:IPR019794), Haem peroxidase, plant/fungal/bacterial (InterPro:IPR002016); BEST Arabidopsis thaliana protein match is: Peroxidase superfamily protein (TAIR:AT2G38380.1)
AT4G35350	*-0,543*	XYLEM CYSTEINE PEPTIDASE 1 (XCP1)	tracheary element vacuolar protein

AT2G45670	*-0,545*		calcineurin B subunit-related; FUNCTIONS IN: calcium ion binding, acyltransferase activity; INVOLVED IN: metabolic process; EXPRESSED IN: 22 plant structures; EXPRESSED DURING: 14 growth stages; CONTAINS InterPro DOMAIN/s: EF-Hand 1, calcium-binding site (InterPro:IPR018247), Phospholipid/glycerol acyltransferase (InterPro:IPR002123), EF-HAND 2 (InterPro:IPR018249), EF-hand-like domain (InterPro:IPR011992), Calcium-binding EF-hand (InterPro:IPR002048), EF-hand (InterPro:IPR018248); BEST Arabidopsis thaliana protein match is: Phospholipid/glycerol acyltransferase family protein (TAIR:AT1G80950.1)
AT3G53090	*-0,548*	UBIQUITIN-PROTEIN LIGASE 7 (UPL7)	encodes a ubiquitin-protein ligase containing a HECT domain. There are six other HECT-domain UPLs in Arabidopsis.
AT1G31710	*-0,551*		Copper amine oxidase family protein; FUNCTIONS IN: primary amine oxidase activity, quinone binding, copper ion binding; INVOLVED IN: oxidation reduction, amine metabolic process; LOCATED IN: endomembrane system; EXPRESSED IN: 13 plant structures; EXPRESSED DURING: 8 growth stages; CONTAINS InterPro DOMAIN/s: Copper amine oxidase, N-terminal (InterPro:IPR016182), Copper amine oxidase, N2-terminal (InterPro:IPR015800), Copper amine oxidase, N2/N3-terminal (InterPro:IPR015801), Copper amine oxidase, N3-terminal (InterPro:IPR015802), Copper amine oxidase (InterPro:IPR000269), Copper amine oxidase, C-terminal (InterPro:IPR015798); BEST Arabidopsis thaliana protein match is: Copper amine oxidase family protein (TAIR:AT1G31690.1)
AT5G10030	*-0,553*	TGACG MOTIF-BINDING FACTOR 4 (TGA4)	Encodes a member of basic leucine zipper transcription gene family. Nomenclature according to Xiang, et al. (1997).
AT1G69960	*-0,554*	SERINE/THREONINE PROTEIN PHOSPHATASE 2A (PP2A)	type 2A serine/threonine protein phosphatase (PP2A) mRNA,
AT4G10380	*-0,554*	NOD26-LIKE INTRINSIC PROTEIN 5;1 (NIP5;1)	Boric acid channel. Essential for efficient boron uptake and plant development under boron limitation. Also functions in arsenite transport and tolerance. Localized preferentially in outer membrane domains of root cells.
AT4G31550	*-0,555*	WRKY DNA-BINDING PROTEIN 11 (WRKY11)	member of WRKY Transcription Factor; Group II-d; negative regulator of basal resistance to Pseudomonas syringae.
AT1G78600	*-0,557*	LIGHT-REGULATED ZINC FINGER PROTEIN 1 (LZF1)	light-regulated zinc finger protein 1 (LZF1); CONTAINS InterPro DOMAIN/s: Zinc finger, B-box (InterPro:IPR000315); BEST Arabidopsis thaliana protein match is: B-box zinc finger family protein (TAIR:AT1G06040.1).
AT3G55260	*-0,557*	BETA-HEXOSAMINIDASE 1 (HEXO1)	Encodes a protein with β-hexosaminidase activity (the enzyme is active with p-nitrophenyl-β-N-acetylglucosaminide as substrate but displayed only a minor activity toward p-nitrophenyl-β-N-acetylgalactosaminide). The enzyme displays no distinct preference for a specific terminal GlcNAc residue and indeed cleaved the asialoagalactodabsylglycopeptide GnGn to a mixture of products.
AT1G11910	*-0,558*	ASPARTIC PROTEINASE A1 (APA1)	Encodes an aspartic proteinase that forms a heterodimer and is stable over a broad pH range (ph 3-8).
AT1G30200	*-0,558*		F-box family protein; CONTAINS InterPro DOMAIN/s: F-box domain, Skp2-like (InterPro:IPR022364); BEST Arabidopsis thaliana protein match is: F-box family protein (TAIR:AT5G46170.1)
AT4G37410	*-0,558*	CYTOCHROME P450, FAMILY 81, SUBFAMILY F, POLYPEPTIDE 4 (CYP81F4)	member of CYP81F The mRNA is cell-to-cell mobile.
AT1G67730	*-0,559*	BETA-KETOACYL REDUCTASE 1 (KCR1)	Encodes one of the two Arabidopsis homologues to YBR159w encoding a S. cerevisiae beta-ketoacyl reductase (KCR), which catalyzes the first reduction during VLCFA (very long chain fatty acids, >18 carbon) elongation: KCR1 (At1g67730), KCR2 (At1g24470). Complementation of the yeast ybr159Delta mutant demonstrated that the two KCR proteins are divergent and that only AtKCR1 can restore heterologous elongase activity similar to the native yeast KCR gene. The mRNA is cell-to-cell mobile.

AT3G19860	*-0,559*	BASIC HELIX-LOOP-HELIX 121 (bHLH121)	basic Helix-Loop-Helix 121 (bHLH121); FUNCTIONS IN: DNA binding, sequence-specific DNA binding transcription factor activity; INVOLVED IN: regulation of transcription; LOCATED IN: nucleus; EXPRESSED IN: 22 plant structures; EXPRESSED DURING: 15 growth stages; CONTAINS InterPro DOMAIN/s: Helix-loop-helix DNA-binding domain (InterPro:IPR001092), Helix-loop-helix DNA-binding (InterPro:IPR011598); BEST Arabidopsis thaliana protein match is: basic helix-loop-helix (bHLH) DNA-binding superfamily protein (TAIR:AT4G36060.2)
AT5G42420	*-0,559*	UDP-RHA/UDP-GAL TRANSPORTER 3 (URGT3)	Nucleotide-sugar transporter family protein; CONTAINS InterPro DOMAIN/s: Protein of unknown function DUF250 (InterPro:IPR004853); BEST Arabidopsis thaliana protein match is: Nucleotide-sugar transporter family protein (TAIR:AT1G21070.1)
AT5G49460	*-0,56*	ATP CITRATE LYASE SUBUNIT B 2 (ACLB-2)	One of the two genes encoding subunit B of the cytosolic enzyme ATP Citrate Lyase (ACL)
AT1G54320	*-0,562*		LEM3 (ligand-effect modulator 3) family protein / CDC50 family protein; INVOLVED IN: biological_process unknown; LOCATED IN: chloroplast, membrane; EXPRESSED IN: 24 plant structures; EXPRESSED DURING: 15 growth stages; CONTAINS InterPro DOMAIN/s: Protein of unknown function DUF284, transmembrane eukaryotic (InterPro:IPR005045); BEST Arabidopsis thaliana protein match is: ALA-interacting subunit 1 (TAIR:AT3G12740.1)
AT2G47650	*-0,562*	UDP-XYLOSE SYNTHASE 4 (UXS4)	encodes a protein similar to UDP-glucuronic acid decarboxylase. UDP-glucuronic acid decarboxylase produces UDP-xylose, which is a substrate for many cell wall carbohydrates including hemicellulose and pectin. UDP-xylose is also known to feedback regulate several cell wall biosynthetic enzymes.
AT1G63000	*-0,563*	NUCLEOTIDE-RHAMNOSE SYNTHASE/EPIMERASE-REDUCTASE (NRS/ER)	nucleotide-rhamnose synthase/epimerase-reductase (NRS/ER); FUNCTIONS IN: UDP-4-keto-rhamnose-4-keto-reductase activity, dTDP-4-dehydrorhamnose reductase activity, UDP-4-keto-6-deoxy-glucose-3,5-epimerase activity, dTDP-4-dehydrorhamnose 3,5-epimerase activity; INVOLVED IN: dTDP-rhamnose biosynthetic process, UDP-rhamnose biosynthetic process; LOCATED IN: soluble fraction, plasma membrane; EXPRESSED IN: 26 plant structures; EXPRESSED DURING: 16 growth stages; CONTAINS InterPro DOMAIN/s: NAD(P)-binding domain (InterPro:IPR016040), dTDP-4-dehydrorhamnose reductase (InterPro:IPR005913); BEST Arabidopsis thaliana protein match is: rhamnose biosynthesis 1 (TAIR:AT1G78570.1)
AT2G01650	*-0,563*	PLANT UBX DOMAIN-CONTAINING PROTEIN 2 (PUX2)	encodes a peripheral membrane protein that contains UBX domain and interacts with AtCDC48 in vitro and co-fractionates with membrane-associated but not soluble AtCDC48 in vivo.
AT5G55960	*-0,563*		unknown protein; CONTAINS InterPro DOMAIN/s: Uncharacterised protein family UPF0118 (InterPro:IPR002549)
AT1G32230	*-0,564*	RADICAL-INDUCED CELL DEATH1 (RCD1)	Encodes a protein belonging to the (ADP-ribosyl)transferase domain-containing subfamily of WWE protein-protein interaction domain protein family. Superoxide radicals are necessary and sufficient to propagate cell death or lesion formation in rcd1 mutants. Without stress treatment, RCD1 is localized in the nucleus. Under high salt or oxidative stress, RCD1 is found not only in the nucleus but also in the cytoplasm. The mRNA is cell-to-cell mobile.
AT3G17810	*-0,564*	PYRIMIDINE 1 (PYD1)	Encodes a protein predicted to have dihydropyrimidine dehydrogenase activity. Its activity has not been demonstrated in vivo, but, it is required for efficient uracil catabolism in Arabidopsis. It localizes to the plastid.
AT5G17330	*-0,565*	GLUTAMATE DECARBOXYLASE (GAD)	Encodes one of two isoforms of glutamate decarboxylase. The mRNA is cell-to-cell mobile.
AT5G32440	*-0,568*		Ubiquitin system component Cue protein; LOCATED IN: chloroplast; EXPRESSED IN: 24 plant structures; EXPRESSED DURING: 15 growth stages; CONTAINS InterPro DOMAIN/s: Ubiquitin system component Cue (InterPro:IPR003892); BEST Arabidopsis thaliana protein match is: unknown protein (TAIR:AT1G80040.1).
AT2G24550	*-0,569*		unknown protein; BEST Arabidopsis thaliana protein match is: unknown protein (TAIR:AT4G31510.1)
AT5G64500	*-0,569*		Major facilitator superfamily protein; FUNCTIONS IN: molecular_function unknown; INVOLVED IN: transmembrane transport; LOCATED IN: membrane; EXPRESSED IN: 24 plant structures; EXPRESSED DURING: 15 growth stages; CONTAINS InterPro DOMAIN/s: Major facilitator superfamily (InterPro:IPR020846), Major facilitator superfamily MFS-1 (InterPro:IPR011701), Major facilitator superfamily, general substrate transporter (InterPro:IPR016196); BEST Arabidopsis thaliana protein match is: Major facilitator superfamily protein (TAIR:AT2G22730.1)
AT4G37590	*-0,573*	NAKED PINS IN YUC MUTANTS 5 (NPY5)	A member of the NPY gene family (NPY1/AT4G31820, NPY2/AT2G14820, NPY3/AT5G67440, NPY4/AT2G23050, NPY5/AT4G37590). Involved in auxin-mediated organogenesis.

AT5G35200	*-0,573*		ENTH/ANTH/VHS superfamily protein; FUNCTIONS IN: phospholipid binding, clathrin binding, binding, phosphatidylinositol binding; INVOLVED IN: clathrin coat assembly; LOCATED IN: plasma membrane; EXPRESSED IN: 24 plant structures; EXPRESSED DURING: 15 growth stages; CONTAINS InterPro DOMAIN/s: Epsin-like, N-terminal (InterPro:IPR013809), ANTH (InterPro:IPR011417), ENTH/VHS (InterPro:IPR008942), Clathrin adaptor, phosphoinositide-binding, GAT-like (InterPro:IPR014712); BEST Arabidopsis thaliana protein match is: ENTH/ANTH/VHS superfamily protein (TAIR:AT2G01600.1)
AT5G43780	*-0,573*	(APS4)	sulfate adenylyltransferase, ATP sulfurylase
AT1G80210	*-0,575*	BRCA1/BRCA2-CONTAINING COMPLEX 36 HOMOLOG A (BRCC36A)	Mov34/MPN/PAD-1 family protein; FUNCTIONS IN: molecular_function unknown; INVOLVED IN: biological_process unknown; LOCATED IN: endomembrane system; EXPRESSED IN: male gametophyte; EXPRESSED DURING: L mature pollen stage, M germinated pollen stage; CONTAINS InterPro DOMAIN/s: Mov34/MPN/PAD-1 (InterPro:IPR000555); BEST Arabidopsis thaliana protein match is: Mov34/MPN/PAD-1 family protein (TAIR:AT3G06820.2)
AT1G78100	*-0,576*	AUXIN UP-REGULATED F-BOX PROTEIN 1 (AUF1)	F-box family protein; CONTAINS InterPro DOMAIN/s: F-box domain, cyclin-like (InterPro:IPR001810), F-box domain, Skp2-like (InterPro:IPR022364); BEST Arabidopsis thaliana protein match is: F-box family protein (TAIR:AT1G22220.1)
AT5G09530	*-0,577*	PRO-GLU-LEU|ILE|VAL-PRO-LYS 1 (PELPK1)	The gene encodes a unique protein which contains 36 repeats of a unique pentapeptide (Pro-Glu-Leu|Ile|Val-Pro-Lys). It has been shown tobe involved in growth and development.
AT3G04560	*-0,578*		unknown protein; FUNCTIONS IN: molecular_function unknown; INVOLVED IN: biological_process unknown; EXPRESSED IN: 22 plant structures; EXPRESSED DURING: 16 growth stages
AT5G47100	*-0,578*	CALCINEURIN B-LIKE PROTEIN 9 (CBL9)	member of AtCBLs (Calcineurin B-like Calcium Sensor Proteins. CBL9 interacts with and targets CIPK23 to the plasma membrane in vivo.
AT3G62830	*-0,581*	(AUD1)	encodes an isoform of UDP-glucuronic acid decarboxylase, which is predicted to be membrane-bound by PSORT. This enzyme produces UDP-xylose, which is a substrate for many cell wall carbohydrates including hemicellulose and pectin. UDP-xylose is also known to feedback regulate several cell wall biosynthetic enzymes.
AT5G63620	*-0,581*		GroES-like zinc-binding alcohol dehydrogenase family protein; FUNCTIONS IN: cobalt ion binding, zinc ion binding; INVOLVED IN: oxidation reduction; EXPRESSED IN: 24 plant structures; EXPRESSED DURING: 13 growth stages; CONTAINS InterPro DOMAIN/s: GroES-like (InterPro:IPR011032), Alcohol dehydrogenase GroES-like (InterPro:IPR013154), Alcohol dehydrogenase, C-terminal (InterPro:IPR013149), Alcohol dehydrogenase superfamily, zinc-containing (InterPro:IPR002085); BEST Arabidopsis thaliana protein match is: GroES-like zinc-binding dehydrogenase family protein (TAIR:AT1G64710.1)
AT1G79530	*-0,582*	GLYCERALDEHYDE-3-PHOSPHATE DEHYDROGENASE OF PLASTID 1 (GAPCP-1)	Encodes one of the chloroplast/plastid localized GAPDH isoforms (GAPCp1/At1g79530 and GAPCp2/At1g16300). gapcp double mutants display a drastic phenotype of arrested root development, dwarfism and sterility. GAPCps are important for the synthesis of serine in roots.
AT4G13270	*-0,584*		Late embryogenesis abundant (LEA) hydroxyproline-rich glycoprotein family; FUNCTIONS IN: molecular_function unknown; INVOLVED IN: biological_process unknown; LOCATED IN: chloroplast; EXPRESSED IN: 23 plant structures; EXPRESSED DURING: 13 growth stages; CONTAINS InterPro DOMAIN/s: Late embryogenesis abundant protein, group 2 (InterPro:IPR004864); BEST Arabidopsis thaliana protein match is: Late embryogenesis abundant (LEA) hydroxyproline-rich glycoprotein family (TAIR:AT1G52330.1)
AT1G47640	*-0,585*		unknown protein; FUNCTIONS IN: molecular_function unknown; INVOLVED IN: biological_process unknown; LOCATED IN: mitochondrion; EXPRESSED IN: 23 plant structures; EXPRESSED DURING: 13 growth stages; CONTAINS InterPro DOMAIN/s: Protein of unknown function DUF2053, membrane (InterPro:IPR019164)
AT1G49300	*-0,585*	RAB GTPASE HOMOLOG G3E (RABG3E)	encodes a small GTPase involved in membrane trafficking. Gene expression is induced by hydrogen peroxide and lines. Lines overexpressing the gene are more tolerant to high salt and hyperosmotic conditions.
AT2G46550	*-0,585*		unknown protein; BEST Arabidopsis thaliana protein match is: unknown protein (TAIR:AT1G01240.3)
AT3G27190	*-0,585*	URIDINE KINASE-LIKE 2 (UKL2)	One of the homologous genes predicted to encode proteins with UPRT domains (Uracil phosphoribosyltransferase). Five of these genes (At5g40870, At3g27190, At1g55810, At4g26510 and At3g27440) show a high level of identity, and are annotated as also containing a N-terminal uracil kinase (UK) domain. These genes are referred to as UKL1 (UK-like 1), UKL2, UKL3, UKL4 and UKL5, respectively.

AT4G02620	*-0,585*		vacuolar ATPase subunit F family protein; FUNCTIONS IN: hydrogen ion transporting ATP synthase activity, rotational mechanism, proton-transporting ATPase activity, rotational mechanism; INVOLVED IN: ATP synthesis coupled proton transport; LOCATED IN: plasma membrane, vacuole; EXPRESSED IN: 25 plant structures; EXPRESSED DURING: 15 growth stages; CONTAINS InterPro DOMAIN/s: ATPase, V1 complex, subunit F, eukaryotic (InterPro:IPR005772), ATPase, V1/A1 complex, subunit F (InterPro:IPR008218)
AT1G68820	*-0,587*		Transmembrane Fragile-X-F-associated protein; FUNCTIONS IN: zinc ion binding; EXPRESSED IN: 22 plant structures; EXPRESSED DURING: 13 growth stages; CONTAINS InterPro DOMAIN/s: Transmembrane Fragile-X-F-associated protein (InterPro:IPR019396), Zinc finger, RING-type (InterPro:IPR001841); BEST Arabidopsis thaliana protein match is: Transmembrane Fragile-X-F-associated protein (TAIR:AT1G73950.1)
AT3G13520	*-0,589*	ARABINOGALACTAN PROTEIN 12 (AGP12)	Encodes a GPI-anchored arabinogalactan (AG) peptide with a short 'classical' backbone of 10 amino acids, seven of which are conserved among the 4 other Arabidopsis AG peptides. These peptides may be involved in cell signaling.
AT4G17100	*-0,589*		EXPRESSED IN: 23 plant structures; EXPRESSED DURING: 13 growth stages; CONTAINS InterPro DOMAIN/s: Endoribonuclease XendoU (InterPro:IPR018998).
AT1G20900	*-0,59*	AT-HOOK MOTIF NUCLEAR-LOCALIZED PROTEIN 27 (AHL27)	Encodes an AT hook domain containing protein that acts redundantly with SOB3 to modulate hypocotyl growth inhibition in response to light.
AT1G73670	*-0,59*	MAP KINASE 15 (MPK15)	member of MAP Kinase The mRNA is cell-to-cell mobile.
AT3G51840	*-0,59*	ACYL-COA OXIDASE 4 (ACX4)	Encodes a short-chain acyl-CoA oxidase, which catalyzes the first step of peroxisomal fatty acid beta-oxidation during early, post-germinative growth in oilseed species. Null mutants virtually lack short-chain acyl-CoA and are resistant to 2,4-dichlorophenoxybutyric acid, which is converted to the herbicide and auxin analogue 2,4-dichlorophenoxyacetic acid by beta-oxidation. Despite the almost complete loss of short-chain activity, lipid catabolism and seedling growth and establishment was unaltered in the acx4 mutant. However, double mutants in acx3acx4 (acx3 encodes medium chain acyl CoA oxidase) were not viable and arrested during embryogenesis.
AT5G19740	*-0,592*	LIKE AMP 1 (LAMP1)	LAMP is an AMP paralog that overlaps in expression within the vascular system. Along with LAMP it suppresses meristem activity within the peripheral zone of the shoot apical meristem. LAMP is localized to the endoplasmic reticulum.
AT5G59960	*-0,592*		unknown protein; FUNCTIONS IN: molecular_function unknown; INVOLVED IN: biological_process unknown; LOCATED IN: nucleus; EXPRESSED IN: 24 plant structures; EXPRESSED DURING: 15 growth stages
AT4G33580	*-0,593*	BETA CARBONIC ANHYDRASE 5 (BCA5)	beta carbonic anhydrase 5 (BCA5); FUNCTIONS IN: carbonate dehydratase activity, zinc ion binding; INVOLVED IN: carbon utilization; LOCATED IN: chloroplast; EXPRESSED IN: 25 plant structures; EXPRESSED DURING: 13 growth stages; CONTAINS InterPro DOMAIN/s: Carbonic anhydrase, prokaryotic-like, conserved site (InterPro:IPR015892), Carbonic anhydrase (InterPro:IPR001765); BEST Arabidopsis thaliana protein match is: beta carbonic anhydrase 6 (TAIR:AT1G58180.4)
AT1G76900	*-0,595*	TUBBY LIKE PROTEIN 1 (TLP1)	Member of TLP family
AT5G63190	*-0,595*		MA3 domain-containing protein; CONTAINS InterPro DOMAIN/s: Initiation factor eIF-4 gamma, MA3 (InterPro:IPR003891); BEST Arabidopsis thaliana protein match is: MA3 domain-containing protein (TAIR:AT3G48390.1)
AT3G14067	*-0,596*	SENESCENCE-ASSOCIATED SUBTILISIN PROTEASE (SASP)	Encodes a protein with similarity to serine protease, subtilisin, that is upregulated during senescence and expressed in the arial portions of the plant.Loss of function mutations have increased branch number but normal silique length and seed set and therefore have increased fertility.
AT2G39900	*-0,597*	WLIM2A (WLIM2a)	Encodes a member of the Arabidopsis LIM proteins: a family of actin bundlers with distinct expression patterns. WLIM1, WLIM2a, and WLIM2b are widely expressed, whereas PLIM2a, PLIM2b, and PLIM2c are predominantly expressed in pollen. Regulates actin cytoskeleton organization.
AT3G54960	*-0,598*	PDI-LIKE 1-3 (PDIL1-3)	Encodes a protein disulfide isomerase-like (PDIL) protein, a member of a multigene family within the thioredoxin (TRX) superfamily. Transcript levels for this gene are up-regulated in response to three different chemical inducers of ER stress (dithiothreitol, beta-mercaptoethanol, and tunicamycin). Neither AtIRE1-2 nor AtbZIP60 appear to be required for this response.
AT5G04860	*-0,598*		unknown protein; BEST Arabidopsis thaliana protein match is: unknown protein (TAIR:AT3G11760.1)

AT5G10480	*-0,599*	PASTICCINO 2 (PAS2)	Protein tyrosine phosphatase-like involved in cell division and differentiation. Interacts with CDKA;1 only in its phosphorylated form, preventing dephosphorylation. Overexpression slowed down cell division in suspension cell cultures at the G2-to-M transition and early mitosis and inhibited Arabidopsis seedling growth. Localized in the cytoplasm of dividing cells but moved into the nucleus upon cell differentiation. Based on complementation of yeast mutant PAS2 has acyl-CoA dehydratase activity. It interacts with CER10, a component of the microsomal fatty acid elongase complex, suggesting a role in synthesis of VLCFAs (very long chain fatty acids).
AT1G01070	*-0,601*	USUALLY MULTIPLE ACIDS MOVE IN AND OUT TRANSPORTERS 28 (UMAMIT28)	Encodes a plasma membrane-localized amino acid transporter likely involved in amino acid export in the developing seed.
AT1G13450	*-0,601*	GT-1 (GT-1)	Encodes GT-1, a plant transcription factor that binds to one of the cis-acting elements, BoxII, which resides within the upstream promoter region of light-responsive genes. GT-1 was assumed to act as a molecular switch modulated through Ca(2+)-dependent phosphorylation/dephosphorylation in response to light signals.
AT5G60360	*-0,601*	ALEURAIN-LIKE PROTEASE (ALP)	Encodes a senescence-associated thiol protease. The mRNA is cell-to-cell mobile.
AT4G24240	*-0,603*	WRKY DNA-BINDING PROTEIN 7 (WRKY7)	Encodes a Ca-dependent calmodulin binding protein. Sequence similarity to the WRKY transcription factor gene family.
AT4G10810	*-0,604*		unknown protein; FUNCTIONS IN: molecular_function unknown; INVOLVED IN: biological_process unknown; LOCATED IN: chloroplast; EXPRESSED IN: 23 plant structures; EXPRESSED DURING: 15 growth stages; BEST Arabidopsis thaliana protein match is: unknown protein (TAIR:AT4G24026.1)
AT1G62800	*-0,605*	ASPARTATE AMINOTRANSFERASE 4 (ASP4)	Encodes aspartate aminotransferase (Asp4).
AT4G14410	*-0,605*	BASIC HELIX-LOOP-HELIX 104 (bHLH104)	basic Helix-Loop-Helix 104 (bHLH104); FUNCTIONS IN: DNA binding, sequence-specific DNA binding transcription factor activity; INVOLVED IN: regulation of transcription; LOCATED IN: nucleus; EXPRESSED IN: 24 plant structures; EXPRESSED DURING: 15 growth stages; CONTAINS InterPro DOMAIN/s: Helix-loop-helix DNA-binding domain (InterPro:IPR001092), Helix-loop-helix DNA-binding (InterPro:IPR011598); BEST Arabidopsis thaliana protein match is: basic helix-loop-helix (bHLH) DNA-binding superfamily protein (TAIR:AT3G23210.1)
AT5G56340	*-0,605*	(ATCRT1)	ATCRT1; FUNCTIONS IN: zinc ion binding; EXPRESSED IN: 23 plant structures; EXPRESSED DURING: 15 growth stages; CONTAINS InterPro DOMAIN/s: Zinc finger, RING-type (InterPro:IPR001841), Zinc finger, C3HC4 RING-type (InterPro:IPR018957); BEST Arabidopsis thaliana protein match is: RING/U-box superfamily protein (TAIR:AT4G26400.1)
AT1G16180	*-0,606*		Serinc-domain containing serine and sphingolipid biosynthesis protein; FUNCTIONS IN: molecular_function unknown; LOCATED IN: endomembrane system, membrane; EXPRESSED IN: 23 plant structures; EXPRESSED DURING: 15 growth stages; CONTAINS InterPro DOMAIN/s: TMS membrane protein/tumour differentially expressed protein (InterPro:IPR005016); BEST Arabidopsis thaliana protein match is: Serinc-domain containing serine and sphingolipid biosynthesis protein (TAIR:AT3G06170.1)
AT4G15610	*-0,606*	CASP-LIKE PROTEIN 1D1 (CASPL1D1)	Uncharacterised protein family (UPF0497); CONTAINS InterPro DOMAIN/s: Uncharacterised protein family UPF0497, trans-membrane plant (InterPro:IPR006702), Uncharacterised protein family UPF0497, trans-membrane plant subgroup (InterPro:IPR006459); BEST Arabidopsis thaliana protein match is: Uncharacterised protein family (UPF0497) (TAIR:AT3G06390.1)
AT5G18640	*-0,606*		alpha/beta-Hydrolases superfamily protein; FUNCTIONS IN: triglyceride lipase activity; INVOLVED IN: lipid metabolic process; LOCATED IN: endomembrane system; EXPRESSED IN: 24 plant structures; EXPRESSED DURING: 14 growth stages; CONTAINS InterPro DOMAIN/s: Lipase, class 3 (InterPro:IPR002921); BEST Arabidopsis thaliana protein match is: alpha/beta-Hydrolases superfamily protein (TAIR:AT5G18630.2)
AT1G30840	*-0,607*	PURINE PERMEASE 4 (PUP4)	Member of a family of proteins related to PUP1, a purine transporter. May be involved in the transport of purine and purine derivatives such as cytokinins, across the plasma membrane.
AT1G49660	*-0,607*	CARBOXYESTERASE 5 (CXE5)	Encodes a protein with carboxylesterase whose activity was tested using pNA.

AT4G39080	*-0,607*	VACUOLAR PROTON ATPASE A3 (VHA-A3)	Vacuolar proton ATPase subunit VHA-a isoform 3. Localized in the tonoplast. The mRNA is cell-to-cell mobile.
AT1G04220	*-0,609*	3-KETOACYL-COA SYNTHASE 2 (KCS2)	Encodes KCS2, a member of the 3-ketoacyl-CoA synthase family involved in the biosynthesis of VLCFA (very long chain fatty acids).
AT5G47040	*-0,609*	LON PROTEASE 2 (LON2)	Encodes a member of the Lon protease-like proteins (Lon1/At5g26860, Lon2/At5g47040, Lon3/At3g05780, Lon4/At3g05790). Lon is a multifunctional ATP-dependent protease which exists in bacteria, archaea and within organelles in eukaryotic cells. Lon proteases are responsible for the degradation of abnormal, damaged and unstable proteins.
AT5G58730	*-0,609*	MYO-INOSITOL KINASE (MIK)	pfkB-like carbohydrate kinase family protein; CONTAINS InterPro DOMAIN/s: Carbohydrate/purine kinase (InterPro:IPR011611), Carbohydrate/puine kinase, PfkB, conserved site (InterPro:IPR002173); BEST Arabidopsis thaliana protein match is: pfkB-like carbohydrate kinase family protein (TAIR:AT4G10260.1)
AT1G06110	*-0,611*	SKP1/ASK-INTERACTING PROTEIN 16 (SKIP16)	SKP1/ASK-interacting protein 16 (SKIP16); CONTAINS InterPro DOMAIN/s: ApaG (InterPro:IPR007474)
AT4G34131	*-0,611*	UDP-GLUCOSYL TRANSFERASE 73B3 (UGT73B3)	UDP-glucosyl transferase 73B3 (UGT73B3); FUNCTIONS IN: transferase activity, transferring hexosyl groups, quercetin 3-O-glucosyltransferase activity, UDP-glycosyltransferase activity, UDP-glucosyltransferase activity; INVOLVED IN: response to cyclopentenone, response to other organism; LOCATED IN: endomembrane system; CONTAINS InterPro DOMAIN/s: UDP-glucuronosyl/UDP-glucosyltransferase (InterPro:IPR002213); BEST Arabidopsis thaliana protein match is: UDP-glucosyltransferase 73B2 (TAIR:AT4G34135.1)
AT2G36290	*-0,613*		alpha/beta-Hydrolases superfamily protein; FUNCTIONS IN: hydrolase activity; INVOLVED IN: biological_process unknown; LOCATED IN: endoplasmic reticulum; EXPRESSED IN: 24 plant structures; EXPRESSED DURING: 13 growth stages; CONTAINS InterPro DOMAIN/s: Alpha/beta hydrolase fold-1 (InterPro:IPR000073); BEST Arabidopsis thaliana protein match is: alpha/beta-Hydrolases superfamily protein (TAIR:AT1G74300.1)
AT2G40970	*-0,615*	(MYBC1)	MYBC1; CONTAINS InterPro DOMAIN/s: Homeodomain-like (InterPro:IPR009057), Myb, DNA-binding (InterPro:IPR014778), HTH transcriptional regulator, Myb-type, DNA-binding (InterPro:IPR017930), Myb-like DNA-binding domain, SHAQKYF class (InterPro:IPR006447), Homeodomain-related (InterPro:IPR012287); BEST Arabidopsis thaliana protein match is: Homeodomain-like superfamily protein (TAIR:AT3G10760.1)
AT5G56660	*-0,615*	IAA-LEUCINE RESISTANT (ILR)-LIKE 2 (ILL2)	encodes IAA-amino acid hydrolase that has a broad range of substrate specificity. It is most active on IAA-Ala among the IAA-conjugate hydrolases tested.
AT1G21760	*-0,617*	F-BOX PROTEIN 7 (FBP7)	This gene is predicted to encode an F-box protein that is evolutionarily conserved between Arabidopsis and other eukaryotes including S.cerevisiae and humans. It may play a role in regulating translation under conditions of temperature stress. FBP7 transcript levels are increased at high and low temperatures. The mRNA is cell-to-cell mobile.
AT1G01440	*-0,618*		Protein of unknown function (DUF3133); CONTAINS InterPro DOMAIN/s: Protein of unknown function DUF3133 (InterPro:IPR021480); BEST Arabidopsis thaliana protein match is: Protein of unknown function (DUF3133) (TAIR:AT4G01090.1)
AT2G44410	*-0,618*		RING/U-box superfamily protein; FUNCTIONS IN: zinc ion binding; EXPRESSED IN: cultured cell; CONTAINS InterPro DOMAIN/s: Zinc finger, RING-type (InterPro:IPR001841); BEST Arabidopsis thaliana protein match is: RING/U-box superfamily protein (TAIR:AT3G58030.4)
AT3G10230	*-0,619*	LYCOPENE CYCLASE (LYC)	Encodes a protein with lycopene β-cyclase activity. This enzyme uses the linear, symmetrical lycopene as substrate. However, unlike the ε-cyclase which adds only one ring, the β-cyclase introduces a ring at both ends of lycopene to form the bicyclic β-carotene.
AT4G02120	*-0,619*		CTP synthase family protein; FUNCTIONS IN: CTP synthase activity, catalytic activity; INVOLVED IN: pyrimidine ribonucleotide metabolic process, pyrimidine nucleotide biosynthetic process; LOCATED IN: endomembrane system; EXPRESSED IN: 24 plant structures; EXPRESSED DURING: 13 growth stages; CONTAINS InterPro DOMAIN/s: Glutamine amidotransferase class-I, C-terminal (InterPro:IPR000991), CTP synthase (InterPro:IPR004468), CTP synthase, N-terminal (InterPro:IPR017456), Glutamine amidotransferase type 1 (InterPro:IPR017926); BEST Arabidopsis thaliana protein match is: CTP synthase family protein (TAIR:AT3G12670.1)

AT1G73310	*-0,62*	SERINE CARBOXYPEPTIDASE-LIKE 4 (scpl4)	serine carboxypeptidase-like 4 (scpl4); FUNCTIONS IN: serine-type carboxypeptidase activity; INVOLVED IN: proteolysis; LOCATED IN: endomembrane system; EXPRESSED IN: 16 plant structures; EXPRESSED DURING: 8 growth stages; CONTAINS InterPro DOMAIN/s: Peptidase S10, serine carboxypeptidase (InterPro:IPR001563); BEST Arabidopsis thaliana protein match is: serine carboxypeptidase-like 3 (TAIR:AT1G73280.1)
AT2G28080	*-0,62*		UDP-Glycosyltransferase superfamily protein; FUNCTIONS IN: transferase activity, transferring glycosyl groups; INVOLVED IN: metabolic process; LOCATED IN: cellular_component unknown; EXPRESSED IN: 22 plant structures; EXPRESSED DURING: 15 growth stages; CONTAINS InterPro DOMAIN/s: UDP-glucuronosyl/UDP-glucosyltransferase (InterPro:IPR002213); BEST Arabidopsis thaliana protein match is: UDP-Glycosyltransferase superfamily protein (TAIR:AT2G36970.1)
AT2G45720	*-0,621*		ARM repeat superfamily protein; FUNCTIONS IN: binding; INVOLVED IN: biological_process unknown; EXPRESSED IN: 22 plant structures; EXPRESSED DURING: 13 growth stages; CONTAINS InterPro DOMAIN/s: Armadillo-like helical (InterPro:IPR011989), Armadillo (InterPro:IPR000225), Armadillo-type fold (InterPro:IPR016024); BEST Arabidopsis thaliana protein match is: ARM repeat superfamily protein (TAIR:AT1G01830.1)
AT1G30510	*-0,622*	ROOT FNR 2 (RFNR2)	Encodes a root-type ferredoxin:NADP(H) oxidoreductase.
AT1G61360	*-0,622*		S-locus lectin protein kinase family protein; FUNCTIONS IN: in 6 functions; INVOLVED IN: protein amino acid phosphorylation, recognition of pollen; LOCATED IN: endomembrane system; EXPRESSED IN: 21 plant structures; EXPRESSED DURING: 13 growth stages; CONTAINS InterPro DOMAIN/s: Curculin-like (mannose-binding) lectin (InterPro:IPR001480), Protein kinase, ATP binding site (InterPro:IPR017441), PAN-2 domain (InterPro:IPR013227), Apple-like (InterPro:IPR003609), Serine-threonine/tyrosine-protein kinase (InterPro:IPR001245), Protein kinase-like domain (InterPro:IPR011009), Serine/threonine-protein kinase, active site (InterPro:IPR008271), Protein kinase, catalytic domain (InterPro:IPR000719), S-locus glycoprotein (InterPro:IPR000858), EGF-like (InterPro:IPR006210); BEST Arabidopsis thaliana protein match is: S-domain-1 29 (TAIR:AT1G61380.1)
AT1G33990	*-0,625*	METHYL ESTERASE 14 (MES14)	Encodes a protein predicted to act as a carboxylesterase. It has similarity to the SABP2 methyl salicylate esterase from tobacco. This protein does not act on methyl IAA, methyl JA, MeSA, MeGA4, or MEGA9 in vitro.
AT4G38250	*-0,626*	(AT4G38250)	Transmembrane amino acid transporter family protein; CONTAINS InterPro DOMAIN/s: Amino acid transporter, transmembrane (InterPro:IPR013057); BEST Arabidopsis thaliana protein match is: Transmembrane amino acid transporter family protein (TAIR:AT2G42005.1)
AT5G04740	*-0,626*	ACT DOMAIN REPEATS 12 (ACR12)	Encodes a ACT domain-containing protein. The ACT domain, named after bacterial aspartate kinase, chorismate mutase and TyrA (prephenate dehydrogenase), is a regulatory domain that serves as an amino acid-binding site in feedback-regulated amino acid metabolic enzymes.
AT1G64040	*-0,627*	TYPE ONE SERINE/THREONINE PROTEIN PHOSPHATASE 3 (TOPP3)	Encodes the catalytic subunit of a Type 1 phosphoprotein Ser/Thr phosphatase, expressed in roots, shoots and flowers.
AT2G46490	*-0,627*		unknown protein; BEST Arabidopsis thaliana protein match is: unknown protein (TAIR:AT5G35110.1)
AT3G02580	*-0,629*	STEROL 1 (STE1)	Brassinosteroid biosynthetic enzyme, catalyzes delta7 sterol C-5 desaturation step. Mutant has dwarf phenotype.
AT4G00860	*-0,63*	(ATOZI1)	putative pathogenesis-related protein whose transcript level is induced in response to ozone and pathogenic Pseudomonas strains.
AT4G29210	*-0,631*	GAMMA-GLUTAMYL TRANSPEPTIDASE 4 (GGT4)	The gene encodes a gamma-glutamyltransferase (AKA gamma-glutamyl transpeptidase, EC 2.3.2.2) that is located in the vacuole and is most active in roots. The encoded enzyme is involved in the initial degradation of glutathione conjugates in this cell compartment. It is also induced by xenobiotics and contributes to xenobiotics metabolism. Note that conflicting nomenclature exists in the literature: At4g29210 is named as GGT3 in Plant J. 2007 Mar 49(5):878-88; At4g29210 is named as GGT4 and At1g69820 as GGT3 in Plant Physiol. 2007 Aug 144(4):1715-32.
AT1G05320	*-0,633*		FUNCTIONS IN: molecular_function unknown; INVOLVED IN: biological_process unknown; LOCATED IN: cellular_component unknown; EXPRESSED IN: fruit, egg cell; CONTAINS InterPro DOMAIN/s: Prefoldin (InterPro:IPR009053); BEST Arabidopsis thaliana protein match is: unknown protein (TAIR:AT2G32240.1)

AT1G12760	*-0,633*		Zinc finger, C3HC4 type (RING finger) family protein; FUNCTIONS IN: ubiquitin-protein ligase activity, zinc ion binding; LOCATED IN: chloroplast; CONTAINS InterPro DOMAIN/s: Zinc finger, RING-type (InterPro:IPR001841), Zinc finger, C3HC4 RING-type (InterPro:IPR018957); BEST Arabidopsis thaliana protein match is: Zinc finger, C3HC4 type (RING finger) family protein (TAIR:AT1G63170.1)
AT2G45980	*-0,633*	ATG8-INTERACTING PROTEIN 1 (ATI1)	Encodes an Atg8-interacting protein that is partially associated with the ER during favorable growth conditions and becomes mainly associated with a spherical compartment that dynamically moves along the ER network. In stress induced plants, ATI1 is localized to a novel plastid associated bodies that are transported to vesicles, in what appears to be an autophagy dependent process. ATI1 interacts with number of other plastid proteins such as NPQ4 and APE1.
AT4G29260	*-0,633*		HAD superfamily, subfamily IIIB acid phosphatase ; FUNCTIONS IN: acid phosphatase activity; INVOLVED IN: biological_process unknown; LOCATED IN: plasma membrane, vacuole; EXPRESSED IN: 26 plant structures; EXPRESSED DURING: 15 growth stages; CONTAINS InterPro DOMAIN/s: Acid phosphatase (Class B) (InterPro:IPR005519), Vegetative storage protein/acid phosphatase (InterPro:IPR014403), Acid phosphatase, plant (InterPro:IPR010028); BEST Arabidopsis thaliana protein match is: HAD superfamily, subfamily IIIB acid phosphatase (TAIR:AT4G29270.1)
AT1G75380	*-0,634*	BIFUNCTIONAL NUCLEASE IN BASAL DEFENSE RESPONSE 1 (BBD1)	Encodes a nuclease involved in ABA-mediated callose deposition. It has been shown to interact with JAZ proteins, binds to a jasmonic acid-responsive element (JARE) and repress AtJMT expression.
AT4G05060	*-0,634*		PapD-like superfamily protein; FUNCTIONS IN: structural molecule activity; INVOLVED IN: biological_process unknown; LOCATED IN: chloroplast; EXPRESSED IN: 22 plant structures; EXPRESSED DURING: 13 growth stages; CONTAINS InterPro DOMAIN/s: PapD-like (InterPro:IPR008962), Major sperm protein (InterPro:IPR000535); BEST Arabidopsis thaliana protein match is: PapD-like superfamily protein (TAIR:AT4G21450.1)
AT1G51420	*-0,635*	SUCROSE-PHOSPHATASE 1 (SPP1)	sucrose-phosphatase 1 (SPP1); FUNCTIONS IN: phosphatase activity, magnesium ion binding, sucrose-phosphatase activity, catalytic activity; INVOLVED IN: sucrose biosynthetic process, metabolic process; LOCATED IN: plasma membrane; EXPRESSED IN: petal, leaf whorl, root, flower, cultured cell; EXPRESSED DURING: 4 anthesis, petal differentiation and expansion stage; CONTAINS InterPro DOMAIN/s: Sucrose-phosphate synthase (InterPro:IPR006380), Sucrose-6-phosphate phosphohydrolase C-terminal (InterPro:IPR013679), HAD-superfamily hydrolase, subfamily IIB (InterPro:IPR006379), Sucrose phosphatase, plant/cyanobacteria (InterPro:IPR012847), Sucrose-phosphate phosphatase (InterPro:IPR006378); BEST Arabidopsis thaliana protein match is: Sucrose-6F-phosphate phosphohydrolase family protein (TAIR:AT2G35840.3)
AT4G38540	*-0,635*		FAD/NAD(P)-binding oxidoreductase family protein; FUNCTIONS IN: monooxygenase activity; INVOLVED IN: oxidation reduction; EXPRESSED IN: 15 plant structures; EXPRESSED DURING: 9 growth stages; CONTAINS InterPro DOMAIN/s: Monooxygenase, FAD-binding (InterPro:IPR002938); BEST Arabidopsis thaliana protein match is: FAD/NAD(P)-binding oxidoreductase family protein (TAIR:AT5G05320.1)
AT5G15220	*-0,635*		Ribosomal protein L27 family protein; FUNCTIONS IN: structural constituent of ribosome; INVOLVED IN: translation; LOCATED IN: ribosome, intracellular; CONTAINS InterPro DOMAIN/s: Ribosomal protein L27 (InterPro:IPR001684), Ribosomal protein L27, conserved site (InterPro:IPR018261); BEST Arabidopsis thaliana protein match is: Ribosomal protein L27 family protein (TAIR:AT2G16930.3)
AT5G54500	*-0,635*	FLAVODOXIN-LIKE QUINONE REDUCTASE 1 (FQR1)	Encodes a flavin mononucleotide-binding flavodoxin-like quinone reductase that is a primary auxin-response gene.
AT4G35100	*-0,636*	PLASMA MEMBRANE INTRINSIC PROTEIN 3 (PIP3)	a member of the plasma membrane intrinsic protein PIP. functions as aquaporin. Salt-stress-inducible MIP
AT1G55320	*-0,637*	ACYL-ACTIVATING ENZYME 18 (AAE18)	Encodes a protein with similarity to acyl activating enzymes. AAE18 is localized to the peroxisome where it may be involved in metabolism of auxin precursors to active auxins.

AT2G16790	*-0,637*		P-loop containing nucleoside triphosphate hydrolases superfamily protein; FUNCTIONS IN: shikimate kinase activity, kinase activity, ATP binding; INVOLVED IN: carbohydrate metabolic process; LOCATED IN: cellular_component unknown; EXPRESSED IN: 10 plant structures; EXPRESSED DURING: LP.04 four leaves visible, 4 anthesis, petal differentiation and expansion stage; CONTAINS InterPro DOMAIN/s: Shikimate kinase (InterPro:IPR000623), Carbohydrate kinase, thermoresistant glucokinase (InterPro:IPR006001)
AT2G47690	*-0,637*		NADH-ubiquinone oxidoreductase-related; CONTAINS InterPro DOMAIN/s: NADH:ubiquinone oxidoreductase, iron-sulphur subunit 5 (InterPro:IPR019342); BEST Arabidopsis thaliana protein match is: NADH-ubiquinone oxidoreductase-related (TAIR:AT3G62790.1)
AT1G03210	*-0,64*		Phenazine biosynthesis PhzC/PhzF protein; FUNCTIONS IN: catalytic activity; INVOLVED IN: biosynthetic process; LOCATED IN: endomembrane system; EXPRESSED IN: 24 plant structures; EXPRESSED DURING: 15 growth stages; CONTAINS InterPro DOMAIN/s: Phenazine biosynthesis PhzC/PhzF protein (InterPro:IPR003719); BEST Arabidopsis thaliana protein match is: Phenazine biosynthesis PhzC/PhzF protein (TAIR:AT4G02860.1)
AT4G13660	*-0,64*	PINORESINOL REDUCTASE 2 (PRR2)	Encodes a pinoresinol reductase involved in lignan biosynthesis. Expressed strongly in roots and less strongly in stems. Shows preference for pinoresinol and not lariciresinol. The mRNA is cell-to-cell mobile.
AT5G66250	*-0,64*		kinectin-related; INVOLVED IN: biological_process unknown; EXPRESSED IN: 24 plant structures; EXPRESSED DURING: 15 growth stages; BEST Arabidopsis thaliana protein match is: unknown protein (TAIR:AT2G17990.1).
AT5G56090	*-0,641*	CYTOCHROME C OXIDASE 15 (COX15)	Encodes a homolog of COX15. Microarray analysis show a 3.2 fold increase in transcription after treatment with rotenone, an electron transport chain inhibitor.
AT4G27410	*-0,643*	RESPONSIVE TO DESICCATION 26 (RD26)	Encodes a NAC transcription factor induced in response to desiccation. It is localized to the nucleus and acts as a transcriptional activator in ABA-mediated dehydration response.
AT5G37540	*-0,643*		Eukaryotic aspartyl protease family protein; FUNCTIONS IN: aspartic-type endopeptidase activity; INVOLVED IN: proteolysis; LOCATED IN: endomembrane system; EXPRESSED IN: 22 plant structures; EXPRESSED DURING: 13 growth stages; CONTAINS InterPro DOMAIN/s: Peptidase aspartic (InterPro:IPR021109), Peptidase aspartic, catalytic (InterPro:IPR009007), Peptidase A1 (InterPro:IPR001461), Peptidase aspartic, active site (InterPro:IPR001969); BEST Arabidopsis thaliana protein match is: Eukaryotic aspartyl protease family protein (TAIR:AT1G66180.1)
AT2G17130	*-0,644*	ISOCITRATE DEHYDROGENASE SUBUNIT 2 (IDH2)	Encodes a regulatory subunit of the mitochondrially-localized NAD+- dependent isocitrate dehydrogenase.
AT3G01720	*-0,645*	(ATSERGT1)	unknown protein; FUNCTIONS IN: molecular_function unknown; INVOLVED IN: biological_process unknown; LOCATED IN: endomembrane system; EXPRESSED IN: 24 plant structures; EXPRESSED DURING: 13 growth stages; BEST Arabidopsis thaliana protein match is: unknown protein (TAIR:AT5G25265.1)
AT1G12640	*-0,646*	LYSOPHOSPHOLIPID ACYLTRANSFERASE 1 (LPLAT1)	Encodes a lysophosphatidylcholine acyltransferase (LPCAT). Participates in the Lands cycle in developing seeds.
AT2G20230	*-0,647*		Tetraspanin family protein; LOCATED IN: plasma membrane, vacuole; EXPRESSED IN: 24 plant structures; EXPRESSED DURING: 16 growth stages; CONTAINS InterPro DOMAIN/s: Tetraspanin (InterPro:IPR018499), Tetraspanin, subgroup (InterPro:IPR000301); BEST Arabidopsis thaliana protein match is: Tetraspanin family protein (TAIR:AT4G28770.2)
AT1G75180	*-0,648*		Erythronate-4-phosphate dehydrogenase family protein; BEST Arabidopsis thaliana protein match is: Erythronate-4-phosphate dehydrogenase family protein (TAIR:AT1G19400.2)
AT5G23010	*-0,648*	METHYLTHIOALKYLMALATE SYNTHASE 1 (MAM1)	Encodes a methylthioalkylmalate synthase, catalyzes the condensation reactions of the first two rounds of methionine chain elongation in the biosynthesis of methionine-derived glucosinolates. The mRNA is cell-to-cell mobile.
AT1G24430	*-0,649*		HXXXD-type acyl-transferase family protein; FUNCTIONS IN: transferase activity, transferring acyl groups other than amino-acyl groups, transferase activity; INVOLVED IN: N-terminal protein myristoylation; LOCATED IN: cellular_component unknown; EXPRESSED IN: hypocotyl, root; CONTAINS InterPro DOMAIN/s: Transferase (InterPro:IPR003480); BEST Arabidopsis thaliana protein match is: HXXXD-type acyl-transferase family protein (TAIR:AT3G26040.1)

AT4G28060	*-0,649*		Cytochrome c oxidase, subunit Vib family protein; FUNCTIONS IN: cytochrome-c oxidase activity; LOCATED IN: mitochondrion; CONTAINS InterPro DOMAIN/s: Cytochrome c oxidase, subunit VIb (InterPro:IPR003213); BEST Arabidopsis thaliana protein match is: Cytochrome c oxidase, subunit Vib family protein (TAIR:AT5G57815.1)
AT4G36800	*-0,651*	RUB1 CONJUGATING ENZYME 1 (RCE1)	RUB1 conjugating enzyme that conjugates CUL1 and is involved in auxin response and embryogenesis. RCE1 protein physically interacts with RBX1, which may be the E3 for CUL1.
AT4G37940	*-0,651*	AGAMOUS-LIKE 21 (AGL21)	encodes a MADS box protein, highly expressed in the root.
AT5G45490	*-0,651*		P-loop containing nucleoside triphosphate hydrolases superfamily protein; FUNCTIONS IN: ATP binding; INVOLVED IN: apoptosis; LOCATED IN: cellular_component unknown; CONTAINS InterPro DOMAIN/s: NB-ARC (InterPro:IPR002182); BEST Arabidopsis thaliana protein match is: P-loop containing nucleoside triphosphate hydrolases superfamily protein (TAIR:AT5G45440.1)
AT5G02560	*-0,653*	HISTONE H2A 12 (HTA12)	Encodes HTA12, a histone H2A protein.
AT5G17400	*-0,653*	ENDOPLASMIC RETICULUM-ADENINE NUCLEOTIDE TRANSPORTER 1 (ER-ANT1)	This gene is predicted to encode an ER-localized adenine nucleotide transporter with six putative transmembrane helices. It appears to act as a ATP:ADP antiporter when expressed in E.coli plasma membranes. Transcript levels for several ER-localized chaperones (e.g. BIP1/2) and other ATP-requiring ER proteins (e.g. CPK2) are reduced in er-ant1 knock-out lines, suggesting a lack of adequate ATP transport into the ER in these mutants. They also have reduced seed oil and seed protein levels.
AT1G11380	*-0,654*		PLAC8 family protein; CONTAINS InterPro DOMAIN/s: Protein of unknown function Cys-rich (InterPro:IPR006461); BEST Arabidopsis thaliana protein match is: PLAC8 family protein (TAIR:AT5G05350.1)
AT5G56040	*-0,654*	STERILITY-REGULATING KINASE MEMBER 2 (SKM2)	Leucine-rich receptor-like protein kinase family protein; FUNCTIONS IN: protein serine/threonine kinase activity, kinase activity, ATP binding; INVOLVED IN: transmembrane receptor protein tyrosine kinase signaling pathway, protein amino acid phosphorylation; LOCATED IN: endomembrane system; EXPRESSED IN: 22 plant structures; EXPRESSED DURING: 13 growth stages; CONTAINS InterPro DOMAIN/s: Protein kinase, ATP binding site (InterPro:IPR017441), Protein kinase, catalytic domain (InterPro:IPR000719), Leucine-rich repeat-containing N-terminal domain, type 2 (InterPro:IPR013210), Leucine-rich repeat (InterPro:IPR001611), Serine/threonine-protein kinase-like domain (InterPro:IPR017442), Protein kinase-like domain (InterPro:IPR011009), Serine/threonine-protein kinase, active site (InterPro:IPR008271); BEST Arabidopsis thaliana protein match is: Leucine-rich repeat receptor-like protein kinase family protein (TAIR:AT4G26540.1)
AT1G63220	*-0,655*		Calcium-dependent lipid-binding (CaLB domain) family protein; CONTAINS InterPro DOMAIN/s: C2 membrane targeting protein (InterPro:IPR018029), C2 calcium/lipid-binding domain, CaLB (InterPro:IPR008973), C2 calcium-dependent membrane targeting (InterPro:IPR000008); BEST Arabidopsis thaliana protein match is: Calcium-dependent lipid-binding (CaLB domain) family protein (TAIR:AT3G55470.2)
AT1G72130	*-0,655*		Major facilitator superfamily protein; FUNCTIONS IN: transporter activity; INVOLVED IN: oligopeptide transport; LOCATED IN: membrane; CONTAINS InterPro DOMAIN/s: PTR2 family proton/oligopeptide symporter, conserved site (InterPro:IPR018456), Oligopeptide transporter (InterPro:IPR000109), Major facilitator superfamily, general substrate transporter (InterPro:IPR016196); BEST Arabidopsis thaliana protein match is: Major facilitator superfamily protein (TAIR:AT1G72140.1)
AT2G01490	*-0,655*	PHYTANOYL-COA 2-HYDROXYLASE (PAHX)	Encodes a phytanoyl-CoA 2-hydroxylase (PAHX). The mRNA is cell-to-cell mobile.
AT5G59090	*-0,655*	SUBTILASE 4.12 (SBT4.12)	subtilase 4.12 (SBT4.12); FUNCTIONS IN: identical protein binding, serine-type endopeptidase activity; INVOLVED IN: proteolysis, negative regulation of catalytic activity; LOCATED IN: apoplast, nucleus, cytoplasm; EXPRESSED IN: 6 plant structures; CONTAINS InterPro DOMAIN/s: Protease-associated PA (InterPro:IPR003137), Peptidase S8/S53, subtilisin/kexin/sedolisin (InterPro:IPR000209), Peptidase S8, subtilisin-related (InterPro:IPR015500), Peptidase S8/S53, subtilisin, active site (InterPro:IPR022398), Proteinase inhibitor I9, subtilisin propeptide (InterPro:IPR010259); BEST Arabidopsis thaliana protein match is: subtilase 4.13 (TAIR:AT5G59120.1)
AT1G67080	*-0,656*	ABSCISIC ACID (ABA)-DEFICIENT 4 (ABA4)	Encodes a protein involved in the photoprotection of PSII. An aba4-1 mutant completely lacks neoxanthin,a component of the chromophore of the peripheral antenna system in PSII. ABA4 is required for neoxanthin biosynthesis, an intermediary step in abscisic acid biosynthesis, but no catalytic activity has been detected for the ABA4 protein.

AT3G04640	-0,656		glycine-rich protein
AT3G43800	-0,656	GLUTATHIONE S-TRANSFERASE TAU 27	Encodes glutathione transferase belonging to the tau class of GSTs. Naming convention according to Wagner et al. (2002). The mRNA is cell-to-cell mobile.
AT3G60200	-0,656		unknown protein; BEST Arabidopsis thaliana protein match is: unknown protein (TAIR:AT2G44600.1)
AT1G12910	-0,657	ANTHOCYANIN11 (ATAN11)	Encodes LIGHT-REGULATED WD1 (LWD1), a clock proteins regulating circadian period length and photoperiodic flowering.
AT5G10930	-0,657	CBL-INTERACTING PROTEIN KINASE 5 (CIPK5)	Encodes CBL-interacting protein kinase 5 (CIPK5).
AT5G57480	-0,657		P-loop containing nucleoside triphosphate hydrolases superfamily protein; FUNCTIONS IN: nucleoside-triphosphatase activity, ATPase activity, nucleotide binding, ATP binding; LOCATED IN: endomembrane system; EXPRESSED IN: 9 plant structures; EXPRESSED DURING: 4 anthesis, petal differentiation and expansion stage; CONTAINS InterPro DOMAIN/s: ATPase, AAA-type, core (InterPro:IPR003959), ATPase, AAA+ type, core (InterPro:IPR003593); BEST Arabidopsis thaliana protein match is: P-loop containing nucleoside triphosphate hydrolases superfamily protein (TAIR:AT4G25835.1)
AT1G33080	-0,658		MATE efflux family protein; FUNCTIONS IN: antiporter activity, drug transmembrane transporter activity, transporter activity; INVOLVED IN: drug transmembrane transport, transmembrane transport; LOCATED IN: membrane; EXPRESSED IN: 22 plant structures; EXPRESSED DURING: 13 growth stages; CONTAINS InterPro DOMAIN/s: Multi antimicrobial extrusion protein MatE (InterPro:IPR002528); BEST Arabidopsis thaliana protein match is: MATE efflux family protein (TAIR:AT1G33110.1)
AT3G56930	-0,659		DHHC-type zinc finger family protein; FUNCTIONS IN: zinc ion binding; INVOLVED IN: biological_process unknown; LOCATED IN: plasma membrane; EXPRESSED IN: 22 plant structures; EXPRESSED DURING: 12 growth stages; CONTAINS InterPro DOMAIN/s: Zinc finger, DHHC-type (InterPro:IPR001594); BEST Arabidopsis thaliana protein match is: DHHC-type zinc finger family protein (TAIR:AT5G05070.1)
AT5G22440	-0,659		Ribosomal protein L1p/L10e family; FUNCTIONS IN: structural constituent of ribosome, RNA binding; INVOLVED IN: translation, RNA processing; LOCATED IN: cytosolic ribosome, ribosome, cytosolic large ribosomal subunit, nucleolus; EXPRESSED IN: 22 plant structures; EXPRESSED DURING: 13 growth stages; CONTAINS InterPro DOMAIN/s: Ribosomal protein L1 (InterPro:IPR002143), Ribosomal protein L1, 2-layer alpha/beta-sandwich (InterPro:IPR016094); BEST Arabidopsis thaliana protein match is: Ribosomal protein L1p/L10e family (TAIR:AT1G08360.1)
AT1G06290	-0,66	ACYL-COA OXIDASE 3 (ACX3)	Encodes an acyl-CoA oxidase with specificity for medium chain fatty acids. The mRNA is cell-to-cell mobile.
AT5G19530	-0,66	ACAULIS 5 (ACL5)	Encodes a spermine synthase. Required for internode elongation and vascular development, specifically in the mechanism that defines the boundaries between veins and nonvein regions. This mechanism may be mediated by polar auxin transport. Though ACL5 has been shown to function as a spermine synthase in E. coli, an ACL5 knockout has no effect on the endogenous levels of free and conjugated polyamines in Arabidopsis, suggesting that ACL5 may have a very specific or altogether different in vivo function.
AT1G04040	-0,661		HAD superfamily, subfamily IIIB acid phosphatase ; FUNCTIONS IN: acid phosphatase activity; INVOLVED IN: biological_process unknown; LOCATED IN: cell wall, plasma membrane, vacuole, plant-type cell wall; EXPRESSED IN: 19 plant structures; EXPRESSED DURING: 9 growth stages; CONTAINS InterPro DOMAIN/s: Acid phosphatase (Class B) (InterPro:IPR005519), Vegetative storage protein/acid phosphatase (InterPro:IPR014403), Acid phosphatase, plant (InterPro:IPR010028); BEST Arabidopsis thaliana protein match is: HAD superfamily, subfamily IIIB acid phosphatase (TAIR:AT5G44020.1)
AT1G32170	-0,661	XYLOGLUCAN ENDOTRANSGLUCOSYLASE/HYDROLASE 30 (XTH30)	xyloglucan endotransglycosylase-related protein (XTR4) The mRNA is cell-to-cell mobile.
AT2G32080	-0,661	PURIN-RICH ALPHA 1 (PUR ALPHA-1)	similar to the conserved animal nuclear protein PUR alpha which was implicated in the control of gene transcription and DNA replication

AT1G21440	*-0,662*		Phosphoenolpyruvate carboxylase family protein; FUNCTIONS IN: isocitrate lyase activity, catalytic activity; INVOLVED IN: metabolic process; LOCATED IN: chloroplast, chloroplast stroma; EXPRESSED IN: 22 plant structures; EXPRESSED DURING: 14 growth stages; CONTAINS InterPro DOMAIN/s: Pyruvate/Phosphoenolpyruvate kinase, catalytic core (InterPro:IPR015813), Isocitrate lyase/phosphorylmutase, conserved site (InterPro:IPR018523), Isocitrate lyase/phosphorylmutase (InterPro:IPR000918); BEST Arabidopsis thaliana protein match is: Phosphoenolpyruvate carboxylase family protein (TAIR:AT1G77060.1)
AT1G25230	*-0,662*		Calcineurin-like metallo-phosphoesterase superfamily protein; FUNCTIONS IN: hydrolase activity, protein serine/threonine phosphatase activity; INVOLVED IN: biological_process unknown; LOCATED IN: endomembrane system; EXPRESSED IN: 22 plant structures; EXPRESSED DURING: 13 growth stages; CONTAINS InterPro DOMAIN/s: Metallophosphoesterase (InterPro:IPR004843); BEST Arabidopsis thaliana protein match is: purple acid phosphatase 3 (TAIR:AT1G14700.1)
AT1G18590	*-0,663*	SULFOTRANSFERASE 17 (SOT17)	encodes a desulfoglucosinolate sulfotransferase, involved in the final step of glucosinolate core structure biosynthesis. Has a broad-substrate specificity with preference with methionine-derived desulfoglucosinolates.
AT2G17720	*-0,663*	PROLYL 4-HYDROXYLASE 5 (P4H5)	Encodes a prolyl 4-hydroxylase that modifies the extensin proteins in root hair cells.
AT1G19400	*-0,664*		Erythronate-4-phosphate dehydrogenase family protein; BEST Arabidopsis thaliana protein match is: Erythronate-4-phosphate dehydrogenase family protein (TAIR:AT1G75180.3)
AT3G01950	*-0,664*		Protein of unknown function (DUF 3339); CONTAINS InterPro DOMAIN/s: Protein of unknown function DUF3339 (InterPro:IPR021775); BEST Arabidopsis thaliana protein match is: Protein of unknown function (DUF 3339) (TAIR:AT5G14110.1)
AT1G20260	*-0,666*	V-ATPASE B SUBUNIT 3 (VAB3)	One of three genes encoding the vacuolar ATP synthase subunit B1. The protein binds to and co-localizes with F-actin, bundles F-actin to form higher-order structure, and stabilizes actin filaments in vitro. The mRNA is cell-to-cell mobile.
AT1G80310	*-0,666*	MOLYBDATE TRANSPORTER 2 (MOT2)	MOT2 encodes a molybdate transporter which locates to the vacuolar membrane. Loss-of-function (knock out) mutants show elevated molydate levels in rosette leaves and in fully senescent leaves, but decreased MoO4 levels in seeds. Under conditions of molybdate deficiency leaves from mot2::tDNA mutants show strongly reduced nitrate reductase activity. The mot2 gene is slightly expressed in young and mature leaves, but strongly in senescing leaves. This observation points to a function of MOT2 in molybdate transfer from leaves to seeds during plant senescence.
AT2G01170	*-0,666*	BIDIRECTIONAL AMINO ACID TRANSPORTER 1 (BAT1)	Encodes a bidirectional amino acid transporter that can transport ala, arg, glu and lys, GABA but not pro with both export and import activity. Its expression is localized in the vascular tissues suggesting a function in amino acids export from the phloem into sink tissue.
AT2G17420	*-0,666*	NADPH-DEPENDENT THIOREDOXIN REDUCTASE A (NTRA)	NADPH-dependent thioredoxin reductase, major cytosolic isoform The mRNA is cell-to-cell mobile.
AT2G33630	*-0,666*		NAD(P)-binding Rossmann-fold superfamily protein; FUNCTIONS IN: 3-beta-hydroxy-delta5-steroid dehydrogenase activity, binding, catalytic activity; INVOLVED IN: steroid biosynthetic process, metabolic process; LOCATED IN: endoplasmic reticulum; EXPRESSED IN: 23 plant structures; EXPRESSED DURING: 13 growth stages; CONTAINS InterPro DOMAIN/s: 3-beta hydroxysteroid dehydrogenase/isomerase (InterPro:IPR002225), NAD(P)-binding domain (InterPro:IPR016040); BEST Arabidopsis thaliana protein match is: 3beta-hydroxysteroid-dehydrogenase/decarboxylase isoform 1 (TAIR:AT1G47290.2)
AT2G43570	*-0,667*	CHITINASE, PUTATIVE (CHI)	chitinase, putative (CHI); FUNCTIONS IN: chitin binding, chitinase activity; INVOLVED IN: carbohydrate metabolic process, cell wall macromolecule catabolic process; LOCATED IN: apoplast, plant-type cell wall; EXPRESSED IN: 15 plant structures; EXPRESSED DURING: 10 growth stages; CONTAINS InterPro DOMAIN/s: Chitin-binding, type 1, conserved site (InterPro:IPR018371), Glycoside hydrolase, family 19 (InterPro:IPR016283), Chitin-binding, type 1 (InterPro:IPR001002), Glycoside hydrolase, family 19, catalytic (InterPro:IPR000726); BEST Arabidopsis thaliana protein match is: Chitinase family protein (TAIR:AT2G43590.1)

AT5G45280	*-0,668*	PECTIN ACETYLESTERASE 11 (PAE11)	Pectinacetylesterase family protein; FUNCTIONS IN: carboxylesterase activity; INVOLVED IN: biological_process unknown; LOCATED IN: cell wall; EXPRESSED IN: 22 plant structures; EXPRESSED DURING: 13 growth stages; CONTAINS InterPro DOMAIN/s: Pectinacetylesterase (InterPro:IPR004963); BEST Arabidopsis thaliana protein match is: Pectinacetylesterase family protein (TAIR:AT4G19410.1)
AT1G07520	*-0,669*		GRAS family transcription factor; CONTAINS InterPro DOMAIN/s: Transcription factor GRAS (InterPro:IPR005202); BEST Arabidopsis thaliana protein match is: GRAS family transcription factor (TAIR:AT2G29065.1)
AT4G26760	*-0,669*	MICROTUBULE-ASSOCIATED PROTEIN 65-2 (MAP65-2)	microtubule-associated protein 65-2 (MAP65-2); CONTAINS InterPro DOMAIN/s: Microtubule-associated protein, MAP65/ASE1-type (InterPro:IPR007145); BEST Arabidopsis thaliana protein match is: microtubule-associated proteins 65-1 (TAIR:AT5G55230.1)
AT5G18490	*-0,669*		Plant protein of unknown function (DUF946); CONTAINS InterPro DOMAIN/s: Protein of unknown function DUF946 (InterPro:IPR009291); BEST Arabidopsis thaliana protein match is: Plant protein of unknown function (DUF946) (TAIR:AT3G04350.1)
AT1G13730	*-0,67*		Nuclear transport factor 2 (NTF2) family protein with RNA binding (RRM-RBD-RNP motifs) domain; FUNCTIONS IN: RNA binding, nucleotide binding, nucleic acid binding; INVOLVED IN: transport, nucleocytoplasmic transport; LOCATED IN: intracellular; EXPRESSED IN: 17 plant structures; EXPRESSED DURING: 9 growth stages; CONTAINS InterPro DOMAIN/s: Nuclear transport factor 2 (InterPro:IPR002075), RNA recognition motif, RNP-1 (InterPro:IPR000504), Nuclear transport factor 2, Eukaryote (InterPro:IPR018222), Nucleotide-binding, alpha-beta plait (InterPro:IPR012677); BEST Arabidopsis thaliana protein match is: Nuclear transport factor 2 (NTF2) family protein with RNA binding (RRM-RBD-RNP motifs) domain (TAIR:AT2G03640.2)
AT1G32410	*-0,67*		Vacuolar protein sorting 55 (VPS55) family protein; FUNCTIONS IN: transporter activity; INVOLVED IN: transport; LOCATED IN: vacuole; EXPRESSED IN: 23 plant structures; EXPRESSED DURING: 15 growth stages; CONTAINS InterPro DOMAIN/s: Vacuolar protein sorting 55 (InterPro:IPR007262); BEST Arabidopsis thaliana protein match is: Vacuolar protein sorting 55 (VPS55) family protein (TAIR:AT3G11530.2)
AT5G03240	*-0,671*	POLYUBIQUITIN 3 (UBQ3)	encodes ubiquitin that is attached to proteins destined for degradation. UBQ3 is most homologous with UBQ4, and is expressed in higher levels in vegetative tissue but lower levels in flowers than UBQ4. UBQ3 encodes different number of ubiquitins in different ecotypes. UBQ3 transcript level is modulated by UV-B and light/dark treatments.
AT5G27320	*-0,671*	GA INSENSITIVE DWARF1C (GID1C)	Encodes a gibberellin (GA) receptor ortholog of the rice GA receptor gene (OsGID1). Has GA-binding activity, showing higher affinity to GA4. Interacts with DELLA proteins in vivo in the presence of GA4.
AT2G04305	*-0,673*		Magnesium transporter CorA-like family protein; FUNCTIONS IN: metal ion transmembrane transporter activity; INVOLVED IN: metal ion transport, transmembrane transport; LOCATED IN: membrane; EXPRESSED IN: 23 plant structures; EXPRESSED DURING: 13 growth stages; CONTAINS InterPro DOMAIN/s: Mg2+ transporter protein, CorA-like (InterPro:IPR002523)
AT4G17790	*-0,673*		SNARE associated Golgi protein family; CONTAINS InterPro DOMAIN/s: SNARE associated Golgi protein (InterPro:IPR015414); BEST Arabidopsis thaliana protein match is: SNARE associated Golgi protein family (TAIR:AT1G71940.1)
AT4G36880	*-0,673*	CYSTEINE PROTEINASE1 (CP1)	cysteine proteinase1 (CP1); FUNCTIONS IN: cysteine-type peptidase activity, cysteine-type endopeptidase activity; INVOLVED IN: proteolysis, response to gibberellin stimulus, response to red light; LOCATED IN: endomembrane system; EXPRESSED IN: 17 plant structures; EXPRESSED DURING: 8 growth stages; CONTAINS InterPro DOMAIN/s: Proteinase inhibitor I29, cathepsin propeptide (InterPro:IPR013201), Peptidase C1A, papain (InterPro:IPR013128), Peptidase C1A, papain C-terminal (InterPro:IPR000668), Peptidase, cysteine peptidase active site (InterPro:IPR000169); BEST Arabidopsis thaliana protein match is: Granulin repeat cysteine protease family protein (TAIR:AT5G43060.1)
AT1G07000	*-0,674*	EXOCYST SUBUNIT EXO70 FAMILY PROTEIN B2 (EXO70B2)	A member of EXO70 gene family, putative exocyst subunits, conserved in land plants. Arabidopsis thaliana contains 23 putative EXO70 genes, which can be classified into eight clusters on the phylogenetic tree.
AT2G39570	*-0,674*	ACT DOMAIN REPEATS 9 (ACR9)	Encodes a ACT domain-containing protein. The ACT domain, named after bacterial aspartate kinase, chorismate mutase and TyrA (prephenate dehydrogenase), is a regulatory domain that serves as an amino acid-binding site in feedback-regulated amino acid metabolic enzymes.

AT5G02600	*-0,674*	SODIUM POTASSIUM ROOT DEFECTIVE 1 (NAKR1)	Heavy metal transport/detoxification superfamily protein ; FUNCTIONS IN: metal ion binding; INVOLVED IN: metal ion transport; LOCATED IN: chloroplast; EXPRESSED IN: 22 plant structures; EXPRESSED DURING: 12 growth stages; CONTAINS InterPro DOMAIN/s: Heavy metal transport/detoxification protein (InterPro:IPR006121); BEST Arabidopsis thaliana protein match is: Chloroplast-targeted copper chaperone protein (TAIR:AT2G37390.2)
AT5G66530	*-0,674*		Galactose mutarotase-like superfamily protein; FUNCTIONS IN: carbohydrate binding, isomerase activity, aldose 1-epimerase activity, catalytic activity; INVOLVED IN: galactose metabolic process, carbohydrate metabolic process; LOCATED IN: apoplast, chloroplast, chloroplast stroma; EXPRESSED IN: 24 plant structures; EXPRESSED DURING: 13 growth stages; CONTAINS InterPro DOMAIN/s: Glycoside hydrolase-type carbohydrate-binding (InterPro:IPR011013), Aldose 1-epimerase (InterPro:IPR008183), Glycoside hydrolase-type carbohydrate-binding, subgroup (InterPro:IPR014718); BEST Arabidopsis thaliana protein match is: Galactose mutarotase-like superfamily protein (TAIR:AT5G57330.1)
AT1G02860	*-0,675*	NITROGEN LIMITATION ADAPTATION (NLA)	Encodes a ubiquitin E3 ligase with RING and SPX domains that is involved in mediating immune responses and mediates degradation of PHT1s at plasma membranes. Targeted by MIR827. Ubiquitinates PHT1;3, PHT1;2, PHT1;1/AtPT1 and PHT1;4/AtPT2.
AT1G72610	*-0,675*	GERMIN-LIKE PROTEIN 1	germin-like protein (GLP1)
AT1G20050	*-0,676*	HYDRA1 (HYD1)	C-8 sterol isomerase that also plays a role in miRNA function.
AT1G22780	*-0,677*	POINTED FIRST LEAVES (PFL)	S18 ribosomal protein involved in the binding of f-Met tRNA during initiation of mRNA translation. Expression restricted to meristems. Mutant phenotype-pointed first leaves,reduced fresh weight, growth retardation.
AT1G27210	*-0,677*		ARM repeat superfamily protein; FUNCTIONS IN: binding; LOCATED IN: chloroplast; EXPRESSED IN: 23 plant structures; EXPRESSED DURING: 13 growth stages; CONTAINS InterPro DOMAIN/s: HEAT (InterPro:IPR000357), Armadillo-like helical (InterPro:IPR011989), HEAT, type 2 (InterPro:IPR021133), Armadillo-type fold (InterPro:IPR016024); BEST Arabidopsis thaliana protein match is: ARM repeat superfamily protein (TAIR:AT1G59850.1)
AT2G29660	*-0,677*		zinc finger (C2H2 type) family protein; CONTAINS InterPro DOMAIN/s: Zinc finger, C2H2-like (InterPro:IPR015880), Zinc finger, C2H2-type (InterPro:IPR007087); BEST Arabidopsis thaliana protein match is: zinc finger protein-related (TAIR:AT5G54630.1)
AT1G74840	*-0,678*		Homeodomain-like superfamily protein; FUNCTIONS IN: DNA binding, sequence-specific DNA binding transcription factor activity; INVOLVED IN: in 9 processes; LOCATED IN: chloroplast; EXPRESSED IN: 22 plant structures; EXPRESSED DURING: 13 growth stages; CONTAINS InterPro DOMAIN/s: SANT, DNA-binding (InterPro:IPR001005), Homeodomain-like (InterPro:IPR009057), Myb, DNA-binding (InterPro:IPR014778), HTH transcriptional regulator, Myb-type, DNA-binding (InterPro:IPR017930), Myb-like DNA-binding domain, SHAQKYF class (InterPro:IPR006447); BEST Arabidopsis thaliana protein match is: Homeodomain-like superfamily protein (TAIR:AT1G19000.2)
AT3G16470	*-0,678*	JASMONATE RESPONSIVE 1 (JR1)	Encodes a JA-responsive gene that coordinates with GRP7 in shaping plant development through the regulation of RNA processing in Arabidopsis. AtJAC1 interacts with RNA binding protein GRP7 specifically in the cytoplasm to regulate its nucleocytoplasmic distribution.
AT3G58750	*-0,679*	CITRATE SYNTHASE 2 (CSY2)	Encodes a peroxisomal citrate synthase that is expressed throughout seedling and shoot development.
AT4G04695	*-0,679*	CALCIUM-DEPENDENT PROTEIN KINASE 31 (CPK31)	member of Calcium Dependent Protein Kinase. Involved in response to salicylic acid.
AT1G01610	*-0,681*	GLYCEROL-3-PHOSPHATE SN-2-ACYLTRANSFERASE 4 (GPAT4)	bifunctional sn-glycerol-3-phosphate 2-O-acyltransferase/phosphatase. Involved in cutin assembly and is functionally redundant with GPAT8.
AT1G03457	*-0,682*	BRUNO-LIKE 2 (BRN2)	RNA-binding (RRM/RBD/RNP motifs) family protein; FUNCTIONS IN: RNA binding, nucleotide binding, nucleic acid binding; INVOLVED IN: biological_process unknown; LOCATED IN: cellular_component unknown; EXPRESSED IN: 17 plant structures; EXPRESSED DURING: 14 growth stages; CONTAINS InterPro DOMAIN/s: RNA recognition motif, RNP-1 (InterPro:IPR000504), Ribonucleoprotein, BRUNO-like (InterPro:IPR015903), Paraneoplastic encephalomyelitis antigen (InterPro:IPR002343), Nucleotide-binding, alpha-beta plait (InterPro:IPR012677); BEST Arabidopsis thaliana protein match is: RNA-binding protein-defense related 1 (TAIR:AT4G03110.1)

AT5G42010	-0,682		Transducin/WD40 repeat-like superfamily protein; CONTAINS InterPro DOMAIN/s: WD40 repeat 2 (InterPro:IPR019782), WD40 repeat, conserved site (InterPro:IPR019775), WD40 repeat (InterPro:IPR001680), G-protein beta WD-40 repeat, region (InterPro:IPR020472), WD40 repeat-like-containing domain (InterPro:IPR011046), WD40-repeat-containing domain (InterPro:IPR017986), WD40/YVTN repeat-like-containing domain (InterPro:IPR015943), WD40 repeat, subgroup (InterPro:IPR019781); BEST Arabidopsis thaliana protein match is: Transducin/WD40 repeat-like superfamily protein (TAIR:AT1G64610.2)
AT2G19970	-0,683		CAP (Cysteine-rich secretory proteins, Antigen 5, and Pathogenesis-related 1 protein) superfamily protein; FUNCTIONS IN: molecular_function unknown; INVOLVED IN: biological_process unknown; LOCATED IN: extracellular region; EXPRESSED IN: hypocotyl, root, flower; EXPRESSED DURING: petal differentiation and expansion stage; CONTAINS InterPro DOMAIN/s: Allergen V5/Tpx-1 related (InterPro:IPR001283), SCP-like extracellular (InterPro:IPR014044); BEST Arabidopsis thaliana protein match is: CAP (Cysteine-rich secretory proteins, Antigen 5, and Pathogenesis-related 1 protein) superfamily protein (TAIR:AT2G19980.1)
AT5G20650	-0,683	COPPER TRANSPORTER 5 (COPT5)	Encodes COPT5, a member of copper transporter family and functionally complements a high affinity copper transporter mutant in yeast. Plays an important role in the plant response to environmental copper scarcity, probably by remobilizing copper from prevacuolar vesicles, which could act as internal stores or recycling vesicles to provide the metal cofactor to key copper-dependent processes such as photosynthesis.
AT5G52070	-0,683		Agenet domain-containing protein; FUNCTIONS IN: RNA binding; INVOLVED IN: biological_process unknown; LOCATED IN: cellular_component unknown; EXPRESSED IN: 22 plant structures; EXPRESSED DURING: 13 growth stages; CONTAINS InterPro DOMAIN/s: Tudor-like, plant (InterPro:IPR014002), Agenet (InterPro:IPR008395); BEST Arabidopsis thaliana protein match is: Agenet domain-containing protein (TAIR:AT5G42670.1)
AT1G47128	-0,684	RESPONSIVE TO DEHYDRATION 21A (RD21A)	cysteine proteinase precursor-like protein/ dehydration stress-responsive gene (RD21). Has been shown to have peptide ligase activity and protease activity in vitro. RD21 is involved in immunity to the necrotrophic fungal pathogen Botrytis cinerea.Activity detected in root, leaf, flower and cell culture.
AT2G28890	-0,684	POLTERGEIST LIKE 4 (PLL4)	Encodes a protein phosphatase 2C like gene, similar to POL. Involved in leaf development. Knockout mutants have abnormally shaped leaves.
AT5G23750	-0,684		Remorin family protein; CONTAINS InterPro DOMAIN/s: Remorin, C-terminal (InterPro:IPR005516), Remorin, N-terminal (InterPro:IPR005518); BEST Arabidopsis thaliana protein match is: Remorin family protein (TAIR:AT3G48940.1)
AT1G44820	-0,685		Peptidase M20/M25/M40 family protein; FUNCTIONS IN: hydrolase activity, aminoacylase activity; INVOLVED IN: metabolic process, cellular amino acid metabolic process; LOCATED IN: endomembrane system, cytoplasm; EXPRESSED IN: guard cell; CONTAINS InterPro DOMAIN/s: Peptidase M20 (InterPro:IPR002933), N-acyl-L-amino-acid amidohydrolase (InterPro:IPR010159); BEST Arabidopsis thaliana protein match is: Peptidase M20/M25/M40 family protein (TAIR:AT1G44180.1)
AT1G79590	-0,685	SYNTAXIN OF PLANTS 52 (SYP52)	Encodes one of 24 Arabidopsis syntaxins. Its mRNA has been shown to be expressed.
AT5G24890	-0,685		unknown protein; FUNCTIONS IN: molecular_function unknown; INVOLVED IN: biological_process unknown; LOCATED IN: cellular_component unknown; EXPRESSED IN: 24 plant structures; EXPRESSED DURING: 13 growth stages; BEST Arabidopsis thaliana protein match is: unknown protein (TAIR:AT2G24550.1)
AT2G40320	-0,686	TRICHOME BIREFRINGENCE-LIKE 33 (TBL33)	Encodes a member of the TBL (TRICHOME BIREFRINGENCE-LIKE) gene family containing a plant-specific DUF231 (domain of unknown function) domain. TBL gene family has 46 members, two of which (TBR/AT5G06700 and TBL3/AT5G01360) have been shown to be involved in the synthesis and deposition of secondary wall cellulose, presumably by influencing the esterification state of pectic polymers. A nomenclature for this gene family has been proposed (Volker Bischoff & Wolf Scheible, 2010, personal communication). Chemical evidence for function comes from xylan NMR analysis. Secondary wall thickening phenotype can be only observed in double or triple mutant with esk1.
AT1G03610	-0,687		Protein of unknown function (DUF789); CONTAINS InterPro DOMAIN/s: Protein of unknown function DUF789 (InterPro:IPR008507); BEST Arabidopsis thaliana protein match is: Protein of unknown function (DUF789) (TAIR:AT4G03420.1)

AT1G26930	*-0,687*		Galactose oxidase/kelch repeat superfamily protein; FUNCTIONS IN: molecular_function unknown; INVOLVED IN: biological_process unknown; LOCATED IN: peroxisome; EXPRESSED IN: 18 plant structures; EXPRESSED DURING: 8 growth stages; CONTAINS InterPro DOMAIN/s: Galactose oxidase/kelch, beta-propeller (InterPro:IPR011043), Kelch repeat type 1 (InterPro:IPR006652), Kelch related (InterPro:IPR013089), Kelch-type beta propeller (InterPro:IPR015915); BEST Arabidopsis thaliana protein match is: Galactose oxidase/kelch repeat superfamily protein (TAIR:AT1G14330.1)
AT2G43820	*-0,687*	UDP-GLUCOSYLTRANSFERASE 74F2 (UGT74F2)	Encodes a nicotinate-O-glycosyltransferase. Induced by Salicylic acid, virus, fungus and bacteria. Also involved in the tryptophan synthesis pathway. Independent of NPR1 for their induction by salicylic acid. UGT74F1 transfers UDP:glucose to salicylic acid (forming a glucoside (SAG) and a glucose ester (SGE)), benzoic acid, and anthranilate in vitro. UGT74F2 shows a weak ability to catalyze the formation of the p-aminobenzoate-glucose ester in vitro. But, UGT75B1 appears to be the dominant pABA acylglucosyltransferase in vivo based on assays in leaves, flowers, and siliques.
AT4G10170	*-0,688*		SNARE-like superfamily protein; FUNCTIONS IN: molecular_function unknown; INVOLVED IN: transport, vesicle-mediated transport; LOCATED IN: plasma membrane; EXPRESSED IN: 22 plant structures; EXPRESSED DURING: 13 growth stages; CONTAINS InterPro DOMAIN/s: Longin (InterPro:IPR010908), Longin-like (InterPro:IPR011012); BEST Arabidopsis thaliana protein match is: SNARE-like superfamily protein (TAIR:AT1G33475.1)
AT5G61820	*-0,688*		FUNCTIONS IN: molecular_function unknown; INVOLVED IN: biological_process unknown; LOCATED IN: vacuole; EXPRESSED IN: 24 plant structures; EXPRESSED DURING: 15 growth stages; CONTAINS InterPro DOMAIN/s: Stress up-regulated Nod 19 (InterPro:IPR011692)
AT2G42390	*-0,689*		protein kinase C substrate, heavy chain-related; FUNCTIONS IN: molecular_function unknown; INVOLVED IN: biological_process unknown; LOCATED IN: endomembrane system; EXPRESSED IN: 22 plant structures; EXPRESSED DURING: 13 growth stages; BEST Arabidopsis thaliana protein match is: calmodulin-binding protein (TAIR:AT5G56360.1)
AT4G35750	*-0,689*		SEC14 cytosolic factor family protein / phosphoglyceride transfer family protein; CONTAINS InterPro DOMAIN/s: Cellular retinaldehyde-binding/triple function, C-terminal (InterPro:IPR001251); BEST Arabidopsis thaliana protein match is: SEC14 cytosolic factor family protein / phosphoglyceride transfer family protein (TAIR:AT3G10210.1)
AT5G55290	*-0,689*		ATPase, V0 complex, subunit E; FUNCTIONS IN: hydrogen ion transmembrane transporter activity; INVOLVED IN: ATP hydrolysis coupled proton transport; LOCATED IN: vacuole; EXPRESSED IN: 24 plant structures; EXPRESSED DURING: 15 growth stages; CONTAINS InterPro DOMAIN/s: ATPase, V0 complex, subunit E (InterPro:IPR008389); BEST Arabidopsis thaliana protein match is: ATPase, V0 complex, subunit E (TAIR:AT4G26710.2)
AT1G78310	*-0,69*	VQ MOTIF-CONTAINING PROTEIN 9 (VQ9)	VQ motif-containing protein; LOCATED IN: chloroplast; EXPRESSED IN: 17 plant structures; EXPRESSED DURING: 10 growth stages; CONTAINS InterPro DOMAIN/s: VQ (InterPro:IPR008889); BEST Arabidopsis thaliana protein match is: VQ motif-containing protein (TAIR:AT2G35230.1)
AT1G58440	*-0,691*	(XF1)	Encodes a putative protein that has been speculated, based on sequence similarities, to have squalene monooxygenase activity.
AT2G44490	*-0,691*	PENETRATION 2 (PEN2)	Encodes a glycosyl hydrolase that localizes to peroxisomes and acts as a component of an inducible preinvasion resistance mechanism. Required for mlo resistance. The mRNA is cell-to-cell mobile.
AT4G29270	*-0,692*		HAD superfamily, subfamily IIIB acid phosphatase ; FUNCTIONS IN: acid phosphatase activity; INVOLVED IN: biological_process unknown; LOCATED IN: endomembrane system; EXPRESSED IN: 7 plant structures; EXPRESSED DURING: petal differentiation and expansion stage, D bilateral stage; CONTAINS InterPro DOMAIN/s: Acid phosphatase (Class B) (InterPro:IPR005519), Vegetative storage protein/acid phosphatase (InterPro:IPR014403), Acid phosphatase, plant (InterPro:IPR010028); BEST Arabidopsis thaliana protein match is: HAD superfamily, subfamily IIIB acid phosphatase (TAIR:AT4G29260.1)
AT5G54780	*-0,693*		Ypt/Rab-GAP domain of gyp1p superfamily protein; FUNCTIONS IN: RAB GTPase activator activity; INVOLVED IN: regulation of Rab GTPase activity; LOCATED IN: mitochondrion; EXPRESSED IN: 24 plant structures; EXPRESSED DURING: 15 growth stages; CONTAINS InterPro DOMAIN/s: RabGAP/TBC (InterPro:IPR000195); BEST Arabidopsis thaliana protein match is: Ypt/Rab-GAP domain of gyp1p superfamily protein (TAIR:AT4G27100.2)
AT1G27290	*-0,694*		unknown protein

AT4G11530	*-0,694*	CYSTEINE-RICH RLK (RECEPTOR-LIKE PROTEIN KINASE) 34 (CRK34)	Encodes a cysteine-rich receptor-like protein kinase. The mRNA is cell-to-cell mobile.
AT5G61260	*-0,694*		Plant calmodulin-binding protein-related; CONTAINS InterPro DOMAIN/s: Calmodulin-binding protein, plant (InterPro:IPR012417); BEST Arabidopsis thaliana protein match is: Plant calmodulin-binding protein-related (TAIR:AT5G07820.1)
AT1G80690	*-0,695*		PPPDE putative thiol peptidase family protein; CONTAINS InterPro DOMAIN/s: Protein of unknown function DUF862, eukaryotic (InterPro:IPR008580); BEST Arabidopsis thaliana protein match is: PPPDE putative thiol peptidase family protein (TAIR:AT5G25170.1)
AT4G14010	*-0,695*	RALF-LIKE 32 (RALFL32)	Member of a diversely expressed predicted peptide family showing sequence similarity to tobacco Rapid Alkalinization Factor (RALF), and is believed to play an essential role in the physiology of Arabidopsis. Consists of a single exon and is characterized by a conserved C-terminal motif and N-terminal signal peptide.
AT5G01620	*-0,695*	TRICHOME BIREFRINGENCE-LIKE 35 (TBL35)	Encodes a member of the TBL (TRICHOME BIREFRINGENCE-LIKE) gene family containing a plant-specific DUF231 (domain of unknown function) domain. TBL gene family has 46 members, two of which (TBR/AT5G06700 and TBL3/AT5G01360) have been shown to be involved in the synthesis and deposition of secondary wall cellulose, presumably by influencing the esterification state of pectic polymers. A nomenclature for this gene family has been proposed (Volker Bischoff & Wolf Scheible, 2010, personal communication).TBL35 are required only for xylan 3-O-monoacetylation and 2,3-di-O-acetylation. The biochemical phenotype can be observed in tbl35 esk1, double mutant and tbl34 tbl35 esk1 triple mutants.
AT1G22050	*-0,696*	MEMBRANE-ANCHORED UBIQUITIN-FOLD PROTEIN 6 PRECURSOR (MUB6)	membrane-anchored ubiquitin-fold protein 6 precursor (MUB6); CONTAINS InterPro DOMAIN/s: Membrane-anchored ubiquitin-fold protein, HCG-1 (InterPro:IPR017000), Ubiquitin supergroup (InterPro:IPR019955); BEST Arabidopsis thaliana protein match is: membrane-anchored ubiquitin-fold protein 5 precursor (TAIR:AT1G77870.1)
AT5G59780	*-0,696*	MYB DOMAIN PROTEIN 59 (MYB59)	Encodes a putative transcription factor (MYB59).
AT1G63460	*-0,697*	GLUTATHIONE PEROXIDASE 8 (GPX8)	Encodes GPX8 (glutathione peroxidase 8). Involved in the suppression of oxidative damages in nucleus and cytosol. The mRNA is cell-to-cell mobile.
AT1G78380	*-0,697*	GLUTATHIONE S-TRANSFERASE TAU 19 (GSTU19)	Encodes a glutathione transferase that is a member of Tau GST gene family. Expression is induced by drought stress, oxidative stress, and high doses of auxin and cytokinin. naming convention according to Wagner et al. (2002) The expression of this gene is upregulated by herbicide safeners such as benoxacor and fenclorim.
AT4G27270	*-0,697*		Quinone reductase family protein; FUNCTIONS IN: oxidoreductase activity, FMN binding; INVOLVED IN: negative regulation of transcription; LOCATED IN: plasma membrane; EXPRESSED IN: 21 plant structures; EXPRESSED DURING: 13 growth stages; CONTAINS InterPro DOMAIN/s: Flavoprotein WrbA (InterPro:IPR010089), NADPH-dependent FMN reductase (InterPro:IPR005025), Flavodoxin/nitric oxide synthase (InterPro:IPR008254); BEST Arabidopsis thaliana protein match is: flavodoxin-like quinone reductase 1 (TAIR:AT5G54500.1)
AT1G08650	*-0,698*	PHOSPHOENOLPYRUVATE CARBOXYLASE KINASE 1 (PPCK1)	Encodes a phosphoenolpyruvate carboxylase kinase that is expressed at highest levels in leaves. Expression is induced by light. The mRNA is cell-to-cell mobile.
AT5G13870	*-0,699*	XYLOGLUCAN ENDOTRANSGLUCOSYLASE/HYDROLASE 5 (XTH5)	EXGT-A4, endoxyloglucan transferase,
AT5G63990	*-0,699*		Inositol monophosphatase family protein; FUNCTIONS IN: 3'(2'),5'-bisphosphate nucleotidase activity, inositol or phosphatidylinositol phosphatase activity; INVOLVED IN: sulfur metabolic process; LOCATED IN: cellular_component unknown; EXPRESSED IN: 17 plant structures; EXPRESSED DURING: 6 growth stages; CONTAINS InterPro DOMAIN/s: Inositol monophosphatase (InterPro:IPR000760), 3(2),5 -bisphosphate nucleotidase HAL2 (InterPro:IPR006239); BEST Arabidopsis thaliana protein match is: Inositol monophosphatase family protein (TAIR:AT5G64000.1)

AT1G34670	*-0,7*	MYB DOMAIN PROTEIN 93 (MYB93)	Encodes a member of the R2R3 transcription factor gene family that is a negative regulator of lateral root (LR) development. It has been proposed that this transcription factor is part of a novel negative feedback loop stimulated specifically in the endodermis upon LR initiation to ensure that LRs are formed only in the correct place.
AT2G36420	*-0,7*	TON1 RECRUITING MOTIF 27 (TRM27)	unknown protein; BEST Arabidopsis thaliana protein match is: unknown protein (TAIR:AT5G03670.1)
AT2G41660	*-0,7*	MIZU-KUSSEI 1 (MIZ1)	Essential for hydrotropism in roots. Mutant roots are defective in hydrotropism, and have slightly reduced phototropism and modified wavy growth response. Has normal gravitropism and root elongation.
AT5G51060	*-0,7*	ROOT HAIR DEFECTIVE 2 (RHD2)	RHD2 (along with RHD3 and RHD4) is required for normal root hair elongation. Has NADPH oxidase activity. Gene is expressed in the elongation and differention zone in trichoblasts and elongating root hairs. RDH2 is localized to the growing tips of root hair cells. It is required for the production of reactive oxygen species in response to extracellular ATP stimulus. The increase in ROS production stimulates Ca2+ influx.
AT3G56170	*-0,701*	CA-2+ DEPENDENT NUCLEASE (CAN)	Encodes a calcium-dependent nuclease with similarity to staphylococcal nuclease.
AT4G13290	*-0,701*	CYTOCHROME P450, FAMILY 71, SUBFAMILY A, POLYPEPTIDE 19 (CYP71A19)	putative cytochrome P450
AT4G17170	*-0,701*	RAB GTPASE HOMOLOG B1C (RABB1C)	member of RAB gene family
AT1G76490	*-0,702*	HYDROXY METHYLGLUTARYL COA REDUCTASE 1 (HMG1)	Encodes a 3-hydroxy-3-methylglutaryl coenzyme A reductase, which is involved in melavonate biosynthesis and performs the first committed step in isoprenoid biosynthesis. Expression is activated in dark in leaf tissue but not controlled by light in the root (confine The mRNA is cell-to-cell mobile.
AT5G01830	*-0,702*		ARM repeat superfamily protein; FUNCTIONS IN: ubiquitin-protein ligase activity, binding; INVOLVED IN: response to chitin; LOCATED IN: ubiquitin ligase complex; EXPRESSED IN: root; CONTAINS InterPro DOMAIN/s: U box domain (InterPro:IPR003613), Armadillo-like helical (InterPro:IPR011989), Armadillo (InterPro:IPR000225), Armadillo-type fold (InterPro:IPR016024); BEST Arabidopsis thaliana protein match is: plant U-box 17 (TAIR:AT1G29340.1)
AT1G67480	*-0,703*		Galactose oxidase/kelch repeat superfamily protein; CONTAINS InterPro DOMAIN/s: F-box domain, cyclin-like (InterPro:IPR001810), Galactose oxidase/kelch, beta-propeller (InterPro:IPR011043), Kelch repeat type 1 (InterPro:IPR006652), Kelch related (InterPro:IPR013089), Kelch-type beta propeller (InterPro:IPR015915); BEST Arabidopsis thaliana protein match is: Galactose oxidase/kelch repeat superfamily protein (TAIR:AT1G27420.1)
AT3G07990	*-0,703*	SERINE CARBOXYPEPTIDASE-LIKE 27 (SCPL27)	serine carboxypeptidase-like 27 (SCPL27); FUNCTIONS IN: serine-type carboxypeptidase activity; INVOLVED IN: proteolysis; LOCATED IN: endomembrane system; EXPRESSED IN: 21 plant structures; EXPRESSED DURING: 13 growth stages; CONTAINS InterPro DOMAIN/s: Peptidase S10, serine carboxypeptidase (InterPro:IPR001563), Peptidase S10, serine carboxypeptidase, active site (InterPro:IPR018202); BEST Arabidopsis thaliana protein match is: serine carboxypeptidase-like 26 (TAIR:AT2G35780.1)
AT3G55370	*-0,703*	OBF-BINDING PROTEIN 3 (OBP3)	Encodes a nuclear localized Dof domain containing transcription factor expressed primarily in roots. Responsive to salicylic acid. Transgenic overexpressors have yellow leaves and short, defective roots.
AT4G21600	*-0,703*	ENDONUCLEASE 5 (ENDO5)	Encodes a protein with mismatch-specific endonuclease activity with a preference for T/G, A/G, and G/G of single base mismatches. It also has the ability to cleave indel types of mismatches (heteroduplexes with loops).
AT4G35600	*-0,703*	CAST AWAY (CST)	Encodes a receptor-like cytoplasmic kinase that acts as a spatial inhibitor of cell separation. Analysis of the cDNA previously described in Meiners et al., 1991 revealed mistakes in the predicted open reading frame. The mRNA is cell-to-cell mobile.

AT4G16920	*-0,704*		Disease resistance protein (TIR-NBS-LRR class) family; FUNCTIONS IN: transmembrane receptor activity, nucleoside-triphosphatase activity, nucleotide binding, ATP binding; INVOLVED IN: signal transduction, apoptosis, defense response, innate immune response; LOCATED IN: intrinsic to membrane; EXPRESSED IN: 18 plant structures; EXPRESSED DURING: 10 growth stages; CONTAINS InterPro DOMAIN/s: ATPase, AAA+ type, core (InterPro:IPR003593), NB-ARC (InterPro:IPR002182), Leucine-rich repeat (InterPro:IPR001611), Toll-Interleukin receptor (InterPro:IPR000157), Disease resistance protein (InterPro:IPR000767); BEST Arabidopsis thaliana protein match is: Disease resistance protein (TIR-NBS-LRR class) family (TAIR:AT4G16950.2)
AT5G03380	*-0,704*		Heavy metal transport/detoxification superfamily protein ; FUNCTIONS IN: metal ion binding; INVOLVED IN: metal ion transport; EXPRESSED IN: 24 plant structures; EXPRESSED DURING: 13 growth stages; CONTAINS InterPro DOMAIN/s: Heavy metal transport/detoxification protein (InterPro:IPR006121); BEST Arabidopsis thaliana protein match is: Heavy metal transport/detoxification superfamily protein (TAIR:AT2G36950.1)
AT5G11280	*-0,705*		unknown protein; BEST Arabidopsis thaliana protein match is: unknown protein (TAIR:AT1G80200.1)
AT5G54840	*-0,705*	(SGP1)	Monomeric G protein. Expressed in the root quiescent center, flowers, and leaf guard cells and hydathodes.
AT2G02390	*-0,706*	GLUTATHIONE S-TRANSFERASE ZETA 1 (GSTZ1)	Encodes glutathione transferase belonging to the zeta class of GSTs. Naming convention according to Wagner et al. (2002). The protein undergoes spontaneous thiolation following treatment with the oxidant tert-butylhydroperoxide. It functions in vitro as a maleylacetoacetate isomerase and is likely to be involved in tyrosine catabolism.
AT2G29980	*-0,706*	FATTY ACID DESATURASE 3 (FAD3)	Endoplasmic reticulum enzyme responsible for the synthesis of 18:3 fatty acids from phospholipids. Uses cytochrome b5 as electron donor.
AT5G22360	*-0,706*	VESICLE-ASSOCIATED MEMBRANE PROTEIN 714 (VAMP714)	Member of Synaptobrevin-like AtVAMP7C, v-SNARE protein family.
AT1G75540	*-0,707*	B-BOX DOMAIN PROTEIN 21 (BBX21)	Encodes a B-box zinc finger transcription factor BBX21 (also named STH2/salt tolerance homolog2 and LHUS/long hypocotyl under shade). Interacts with COP1 to control de-etiolation. Also genetically interacts with COP1 to regulate shade avoidance. The mRNA is cell-to-cell mobile.
AT2G19570	*-0,707*	CYTIDINE DEAMINASE 1 (CDA1)	Encodes a cytidine deaminase that deaminates cytidine and deoxycytidine and is competitively inhibited by cytosine-containing compounds.
AT4G25170	*-0,707*		Uncharacterised conserved protein (UCP012943); FUNCTIONS IN: molecular_function unknown; INVOLVED IN: biological_process unknown; EXPRESSED IN: 23 plant structures; EXPRESSED DURING: 13 growth stages; CONTAINS InterPro DOMAIN/s: Uncharacterised conserved protein UCP012943 (InterPro:IPR016606); BEST Arabidopsis thaliana protein match is: Uncharacterised conserved protein (UCP012943) (TAIR:AT5G61490.1)
AT1G19310	*-0,708*		RING/U-box superfamily protein; FUNCTIONS IN: zinc ion binding; EXPRESSED IN: 24 plant structures; EXPRESSED DURING: 15 growth stages; CONTAINS InterPro DOMAIN/s: Zinc finger, RING-type, conserved site (InterPro:IPR017907), Zinc finger, RING-type (InterPro:IPR001841), Zinc finger, C3HC4 RING-type (InterPro:IPR018957); BEST Arabidopsis thaliana protein match is: RING/U-box superfamily protein (TAIR:AT2G23780.1)
AT2G02360	*-0,708*	PHLOEM PROTEIN 2-B10 (PP2-B10)	Encodes an F-box protein containing a Nictaba-related lectin domain that can act as a carbohydrate-binding protein.Expression is induced by SA and pathogenic bacteria.
AT2G03450	*-0,708*	PURPLE ACID PHOSPHATASE 9 (PAP9)	purple acid phosphatase 9 (PAP9); FUNCTIONS IN: protein serine/threonine phosphatase activity, acid phosphatase activity; INVOLVED IN: biological_process unknown; LOCATED IN: endomembrane system; EXPRESSED IN: 24 plant structures; EXPRESSED DURING: 13 growth stages; CONTAINS InterPro DOMAIN/s: Purple acid phosphatase, N-terminal (InterPro:IPR015914), Metallophosphoesterase (InterPro:IPR004843), Purple acid phosphatase-like, N-terminal (InterPro:IPR008963); BEST Arabidopsis thaliana protein match is: Purple acid phosphatases superfamily protein (TAIR:AT1G13900.1)
AT2G26830	*-0,708*	EMBRYO DEFECTIVE 1187 (emb1187)	Encodes a member of a small family of choline/ethanolamine kinases that is localized to the plasma membrane. Homozygous loss of function alleles are embryo lethal. Overexpression results in altered phospholipid levels suggesting a critical role in phospholipid biosynthesis.

AT3G19450	*-0,709*	(ATCAD4)	Encodes a catalytically active cinnamyl alcohol dehydrogenase which uses p-coumaryl aldehyde as a preferred substrate. It can also use caffeyl, coniferyl and d-hydroxyconiferyl aldehydes as substrates. The mRNA is cell-to-cell mobile.
AT4G02850	*-0,71*	D-AMINO ACID RACEMASE1 (DAAR1)	DAAR1 encodes a PLP-independent racemase that catalyzes the conversion from L-Ile to D-allo-Ile.
AT4G16110	*-0,71*	RESPONSE REGULATOR 2 (RR2)	Encodes a pollen-specific transcription factor involved in the expression of nuclear genes for components of mitochondrial complex I in Arabidopsis. Acts in concert with other type-B ARRs in the cytokinin signaling pathway. AHK3 mediates cytokinin-induced phosphorylation of ARR2 on the Asp-80 residue. This phosphorylation plays a positive role of ARR2 in cytokinin-mediated control of leaf longevity. Also involved in cytokinin-dependent inhibition of hypocotyl elongation.
AT4G31450	*-0,711*		RING/U-box superfamily protein; FUNCTIONS IN: zinc ion binding; EXPRESSED IN: 22 plant structures; EXPRESSED DURING: 15 growth stages; CONTAINS InterPro DOMAIN/s: Zinc finger, RING-type (InterPro:IPR001841), Zinc finger, C3HC4 RING-type (InterPro:IPR018957); BEST Arabidopsis thaliana protein match is: RING/U-box superfamily protein (TAIR:AT5G24870.2)
AT5G20700	*-0,711*		Protein of unknown function (DUF581); CONTAINS InterPro DOMAIN/s: Protein of unknown function DUF581 (InterPro:IPR007650); BEST Arabidopsis thaliana protein match is: Protein of unknown function (DUF581) (TAIR:AT1G74940.1)
AT1G69160	*-0,712*		unknown protein; BEST Arabidopsis thaliana protein match is: unknown protein (TAIR:AT3G13980.1)
AT5G57070	*-0,712*		hydroxyproline-rich glycoprotein family protein; BEST Arabidopsis thaliana protein match is: hydroxyproline-rich glycoprotein family protein (TAIR:AT1G72790.1)
AT1G22750	*-0,713*		unknown protein; CONTAINS InterPro DOMAIN/s: Protein of unknown function DUF1475 (InterPro:IPR009943)
AT1G71090	*-0,713*	PIN-LIKES 2 (PILS2)	Auxin efflux carrier family protein; FUNCTIONS IN: auxin:hydrogen symporter activity; INVOLVED IN: auxin polar transport, transmembrane transport; LOCATED IN: integral to membrane; EXPRESSED IN: 22 plant structures; EXPRESSED DURING: 13 growth stages; CONTAINS InterPro DOMAIN/s: Auxin efflux carrier (InterPro:IPR004776); BEST Arabidopsis thaliana protein match is: Auxin efflux carrier family protein (TAIR:AT5G01990.1)
AT5G01990	*-0,713*	PIN-LIKES 6 (PILS6)	Auxin efflux carrier family protein; FUNCTIONS IN: auxin:hydrogen symporter activity; INVOLVED IN: auxin polar transport, transmembrane transport; LOCATED IN: integral to membrane; EXPRESSED IN: 23 plant structures; EXPRESSED DURING: 13 growth stages; CONTAINS InterPro DOMAIN/s: Auxin efflux carrier (InterPro:IPR004776); BEST Arabidopsis thaliana protein match is: Auxin efflux carrier family protein (TAIR:AT1G71090.1)
AT2G31090	*-0,715*	TAXIMIN 1 (TAX1)	Encodes a signalling peptide influencing lateral organ separation.
AT4G36780	*-0,715*	BES1/BZR1 HOMOLOG 2 (BEH2)	BES1/BZR1 homolog 2 (BEH2); CONTAINS InterPro DOMAIN/s: BZR1, transcriptional repressor (InterPro:IPR008540); BEST Arabidopsis thaliana protein match is: BES1/BZR1 homolog 1 (TAIR:AT3G50750.1)
AT5G18650	*-0,715*	MYB30-INTERACTING E3 LIGASE 1 (MIEL1)	Encodes a RING-type E3 ubiquitin ligase that interacts with and ubiquitinates MYB30, leads to MYB30 proteasomal degradation and downregulation of its transcriptional activity. Since MYB30 is a positive regulator of Arabidopsis HR and defence responses, MIEL1 is involved in the negative regulation of these processes. The mRNA is cell-to-cell mobile.
AT5G25050	*-0,715*		Major facilitator superfamily protein; CONTAINS InterPro DOMAIN/s: Major facilitator superfamily, general substrate transporter (InterPro:IPR016196), Biopterin transport-related protein BT1 (InterPro:IPR004324); BEST Arabidopsis thaliana protein match is: Major facilitator superfamily protein (TAIR:AT5G25040.2)
AT2G41150	*-0,716*		unknown protein; FUNCTIONS IN: molecular_function unknown; INVOLVED IN: biological_process unknown; LOCATED IN: endomembrane system; EXPRESSED IN: leaf; BEST Arabidopsis thaliana protein match is: unknown protein (TAIR:AT3G56750.1)
AT5G51630	*-0,716*		Disease resistance protein (TIR-NBS-LRR class) family; FUNCTIONS IN: transmembrane receptor activity, nucleoside-triphosphatase activity, nucleotide binding, ATP binding; INVOLVED IN: signal transduction, apoptosis, defense response, innate immune response; LOCATED IN: intrinsic to membrane; EXPRESSED IN: 9 plant structures; EXPRESSED DURING: 4 anthesis, petal differentiation and expansion stage, E expanded cotyledon stage, D bilateral stage; CONTAINS InterPro DOMAIN/s: ATPase, AAA+ type, core (InterPro:IPR003593), NB-ARC (InterPro:IPR002182), Leucine-rich repeat (InterPro:IPR001611), Toll-Interleukin receptor (InterPro:IPR000157), Disease resistance protein (InterPro:IPR000767); BEST Arabidopsis thaliana protein match is: disease resistance protein (TIR-NBS-LRR class), putative (TAIR:AT4G16890.1)

AT1G26380	*-0,717*	FAD-LINKED OXIDOREDUCTASE 1 (FOX1)	Functions in the biosynthesis of 4-hydroxy indole-3-carbonyl nitrile (4-OH-ICN), a cyanogenic phytoalexin in Arabidopsis. FOX1 acts as a dehydrogenase on indole cyanohydrin to form indole carbonyl nitrile.
AT1G64190	*-0,717*	6-PHOSPHOGLUCONATE DEHYDROGENASE 1 (PGD1)	6-phosphogluconate dehydrogenase family protein; FUNCTIONS IN: in 6 functions; INVOLVED IN: response to salt stress; LOCATED IN: chloroplast, membrane; EXPRESSED IN: 24 plant structures; EXPRESSED DURING: 14 growth stages; CONTAINS InterPro DOMAIN/s: 6-phosphogluconate dehydrogenase, NAD-binding (InterPro:IPR006115), 6-phosphogluconate dehydrogenase, C-terminal-like (InterPro:IPR008927), Dehydrogenase, multihelical (InterPro:IPR013328), 6-phosphogluconate dehydrogenase, decarboxylating (InterPro:IPR006113), 6-phosphogluconate dehydrogenase, C-terminal (InterPro:IPR006114), 6-phosphogluconate dehydrogenase (InterPro:IPR006183), NAD(P)-binding domain (InterPro:IPR016040), Fibritin/6-phosphogluconate dehydrogenase, C-terminal extension (InterPro:IPR012284); BEST Arabidopsis thaliana protein match is: 6-phosphogluconate dehydrogenase family protein (TAIR:AT5G41670.2)
AT1G64460	*-0,717*		Protein kinase superfamily protein; FUNCTIONS IN: inositol or phosphatidylinositol kinase activity, phosphotransferase activity, alcohol group as acceptor; INVOLVED IN: biological_process unknown; LOCATED IN: vacuole; EXPRESSED IN: 24 plant structures; EXPRESSED DURING: 15 growth stages; CONTAINS InterPro DOMAIN/s: Phosphatidylinositol 3-/4-kinase, catalytic (InterPro:IPR000403); BEST Arabidopsis thaliana protein match is: phosphoinositide 4-kinase gamma 4 (TAIR:AT2G46500.2)
AT5G35460	*-0,717*		unknown protein; CONTAINS InterPro DOMAIN/s: Protein of unknown function DUF2838 (InterPro:IPR021261)
AT5G10560	*-0,719*		Glycosyl hydrolase family protein; FUNCTIONS IN: xylan 1,4-beta-xylosidase activity, hydrolase activity, hydrolyzing O-glycosyl compounds; INVOLVED IN: carbohydrate metabolic process; LOCATED IN: plasma membrane, vacuole; EXPRESSED IN: 24 plant structures; EXPRESSED DURING: 13 growth stages; CONTAINS InterPro DOMAIN/s: Glycoside hydrolase, family 3, N-terminal (InterPro:IPR001764), Glycoside hydrolase, family 3, C-terminal (InterPro:IPR002772), Glycoside hydrolase, catalytic core (InterPro:IPR017853); BEST Arabidopsis thaliana protein match is: Glycosyl hydrolase family protein (TAIR:AT1G78060.1)
AT4G39660	*-0,721*	ALANINE:GLYOXYLATE AMINOTRANSFERASE 2 (AGT2)	alanine:glyoxylate aminotransferase 2 homolog (AGT2) mRNA, The mRNA is cell-to-cell mobile.
AT5G25830	*-0,721*	GATA TRANSCRIPTION FACTOR 12 (GATA12)	Encodes a member of the GATA factor family of zinc finger transcription factors.
AT5G53160	*-0,721*	REGULATORY COMPONENTS OF ABA RECEPTOR 3 (RCAR3)	Encodes RCAR3, a regulatory component of ABA receptor. Interacts with protein phosphatase 2Cs ABI1 and ABI2. Stimulates ABA signaling. The mRNA is cell-to-cell mobile.
AT2G17430	*-0,722*	MILDEW RESISTANCE LOCUS O 7 (MLO7)	A member of a large family of seven-transmembrane domain proteins specific to plants, homologs of the barley mildew resistance locus o (MLO) protein. Controls pollen tube reception in synergids. The Arabidopsis genome contains 15 genes encoding MLO proteins, with localization in plasma membrane. Phylogenetic analysis revealed four clades of closely-related AtMLO genes. ATMLO7 belongs to the clade III, with AtMLO5, AtMLO8, AtMLO9, and AtMLO10. The gene is expressed in vegetative organs (RT-PCR experiments)and in pollen grains, as shown by GUS activity patterns. The expression of several phylogenetically closely-related AtMLO genes showed similar or overlapping tissue specificity and analogous responsiveness to external stimuli, suggesting functional redundancy, co-function, or antagonistic function(s).
AT4G37900	*-0,722*	(ATGRDP2)	Protein of unknown function that contains DUF1399 domain and putative RNA binding motif. Expressed in many plant tissues and is involved in many aspects of plant growth and development as well as response to salt stress.
AT5G18630	*-0,722*		alpha/beta-Hydrolases superfamily protein; FUNCTIONS IN: triglyceride lipase activity; INVOLVED IN: lipid metabolic process; LOCATED IN: endomembrane system; EXPRESSED IN: 24 plant structures; EXPRESSED DURING: 15 growth stages; CONTAINS InterPro DOMAIN/s: Lipase, class 3 (InterPro:IPR002921); BEST Arabidopsis thaliana protein match is: alpha/beta-Hydrolases superfamily protein (TAIR:AT5G18640.1)
AT1G63720	*-0,723*		BEST Arabidopsis thaliana protein match is: hydroxyproline-rich glycoprotein family protein (TAIR:AT5G52430.1)
AT1G15350	*-0,724*		unknown protein; BEST Arabidopsis thaliana protein match is: unknown protein (TAIR:AT3G15770.2)
AT1G74280	*-0,724*		alpha/beta-Hydrolases superfamily protein; CONTAINS InterPro DOMAIN/s: Alpha/beta hydrolase fold-1 (InterPro:IPR000073); BEST Arabidopsis thaliana protein match is: alpha/beta-Hydrolases superfamily protein (TAIR:AT1G74290.1)

AT1G27420	*-0,725*		Galactose oxidase/kelch repeat superfamily protein; FUNCTIONS IN: molecular_function unknown; INVOLVED IN: biological_process unknown; LOCATED IN: chloroplast; CONTAINS InterPro DOMAIN/s: Kelch repeat type 1 (InterPro:IPR006652), Galactose oxidase/kelch, beta-propeller (InterPro:IPR011043), Kelch related (InterPro:IPR013089), Kelch-type beta propeller (InterPro:IPR015915); BEST Arabidopsis thaliana protein match is: Galactose oxidase/kelch repeat superfamily protein (TAIR:AT1G67480.2)
AT3G03610	*-0,725*		ELMO/CED-12 family protein; FUNCTIONS IN: molecular_function unknown; INVOLVED IN: phagocytosis; LOCATED IN: cytoskeleton; EXPRESSED IN: 24 plant structures; EXPRESSED DURING: 15 growth stages; CONTAINS InterPro DOMAIN/s: Engulfment/cell motility, ELMO (InterPro:IPR006816); BEST Arabidopsis thaliana protein match is: ELMO/CED-12 family protein (TAIR:AT1G03620.1)
AT1G08770	*-0,726*	PRENYLATED RAB ACCEPTOR 1.E (PRA1.E)	prenylated RAB acceptor 1.E (PRA1.E); CONTAINS InterPro DOMAIN/s: Prenylated rab acceptor PRA1 (InterPro:IPR004895); BEST Arabidopsis thaliana protein match is: PRA1 (Prenylated rab acceptor) family protein (TAIR:AT1G55190.1)
AT2G43670	*-0,726*		Carbohydrate-binding X8 domain superfamily protein; FUNCTIONS IN: molecular_function unknown; INVOLVED IN: biological_process unknown; LOCATED IN: endomembrane system; EXPRESSED IN: shoot apex, hypocotyl, root, pedicel; EXPRESSED DURING: 4 anthesis; CONTAINS InterPro DOMAIN/s: X8 (InterPro:IPR012946); BEST Arabidopsis thaliana protein match is: Carbohydrate-binding X8 domain superfamily protein (TAIR:AT1G78520.1)
AT1G51070	*-0,727*	BASIC HELIX-LOOP-HELIX 115 (bHLH115)	basic Helix-Loop-Helix 115 (bHLH115); FUNCTIONS IN: DNA binding, sequence-specific DNA binding transcription factor activity; INVOLVED IN: regulation of transcription; LOCATED IN: nucleus; EXPRESSED IN: 22 plant structures; EXPRESSED DURING: 13 growth stages; CONTAINS InterPro DOMAIN/s: Helix-loop-helix DNA-binding domain (InterPro:IPR001092), Helix-loop-helix DNA-binding (InterPro:IPR011598); BEST Arabidopsis thaliana protein match is: basic helix-loop-helix (bHLH) DNA-binding superfamily protein (TAIR:AT5G54680.1).
AT1G65590	*-0,727*	BETA-HEXOSAMINIDASE 3 (HEXO3)	Encodes a protein with beta-hexosaminidase activity. Located on the plasma membrane.
AT2G17380	*-0,727*	ASSOCIATED PROTEIN 19 (AP19)	Encodes clathrin assembly protein AP19. The mRNA is cell-to-cell mobile.
AT2G17710	*-0,727*		unknown protein
AT4G28050	*-0,727*	TETRASPANIN7 (TET7)	Member of TETRASPANIN family
AT5G34940	*-0,727*	GLUCURONIDASE 3 (GUS3)	The protein is predicted (WoLF PSORT program) to be membrane-associated.
AT4G33620	*-0,728*		Cysteine proteinases superfamily protein; FUNCTIONS IN: cysteine-type peptidase activity; INVOLVED IN: proteolysis; LOCATED IN: cellular_component unknown; CONTAINS InterPro DOMAIN/s: Peptidase C48, SUMO/Sentrin/Ubl1 (InterPro:IPR003653); BEST Arabidopsis thaliana protein match is: Cysteine proteinases superfamily protein (TAIR:AT1G09730.2)
AT5G07960	*-0,731*		unknown protein; CONTAINS InterPro DOMAIN/s: Uncharacterised protein family UPF0139 (InterPro:IPR005351)
AT5G48900	*-0,731*		Pectin lyase-like superfamily protein; FUNCTIONS IN: lyase activity, pectate lyase activity; LOCATED IN: endomembrane system; EXPRESSED IN: 22 plant structures; EXPRESSED DURING: 14 growth stages; CONTAINS InterPro DOMAIN/s: Pectin lyase fold/virulence factor (InterPro:IPR011050), AmbAllergen (InterPro:IPR018082), Pectate lyase/Amb allergen (InterPro:IPR002022), Pectin lyase fold (InterPro:IPR012334), Parallel beta-helix repeat (InterPro:IPR006626); BEST Arabidopsis thaliana protein match is: Pectin lyase-like superfamily protein (TAIR:AT3G07010.1)
AT1G54210	*-0,732*	AUTOPHAGY 12 A (ATG12A)	AUTOPHAGY 12 A (ATG12A); INVOLVED IN: autophagy, protein ubiquitination involved in ubiquitin-dependent protein catabolic process; LOCATED IN: cytoplasm; EXPRESSED IN: 20 plant structures; EXPRESSED DURING: 12 growth stages; CONTAINS InterPro DOMAIN/s: Autophagy-related protein 12 (InterPro:IPR007242); BEST Arabidopsis thaliana protein match is: Ubiquitin-like superfamily protein (TAIR:AT3G13970.1)
AT5G13580	*-0,732*	ATP-BINDING CASSETTE G6 (ABCG6)	Belongs to a clade of five Arabidopsis thaliana ABCG half-transporters that are required for synthesis of an effective suberin barrier in roots and seed coats (ABCG2, ABCG6, and ABCG20) and for synthesis of an intact pollen wall (ABCG1 and ABCG16).

AT5G40670	-0,732		PQ-loop repeat family protein / transmembrane family protein; FUNCTIONS IN: molecular_function unknown; INVOLVED IN: biological_process unknown; LOCATED IN: plasma membrane, membrane; EXPRESSED IN: 24 plant structures; EXPRESSED DURING: 15 growth stages; CONTAINS InterPro DOMAIN/s: Cystinosin/ERS1p repeat (InterPro:IPR006603), Lysosomal cystine transporter (InterPro:IPR005282)
AT5G45080	-0,733	PHLOEM PROTEIN 2-A6 (PP2-A6)	phloem protein 2-A6 (PP2-A6); FUNCTIONS IN: carbohydrate binding; INVOLVED IN: signal transduction, defense response, innate immune response; LOCATED IN: intrinsic to membrane; EXPRESSED IN: 9 plant structures; EXPRESSED DURING: 9 growth stages; CONTAINS InterPro DOMAIN/s: Toll-Interleukin receptor (InterPro:IPR000157); BEST Arabidopsis thaliana protein match is: phloem protein 2-A8 (TAIR:AT5G45070.1)
AT1G53180	-0,734		unknown protein; FUNCTIONS IN: molecular_function unknown; INVOLVED IN: biological_process unknown; LOCATED IN: cellular_component unknown; EXPRESSED IN: 13 plant structures; EXPRESSED DURING: 6 growth stages; BEST Arabidopsis thaliana protein match is: unknown protein (TAIR:AT3G15115.1)
AT1G73390	-0,734		Endosomal targeting BRO1-like domain-containing protein; FUNCTIONS IN: molecular_function unknown; INVOLVED IN: N-terminal protein myristoylation; LOCATED IN: cellular_component unknown; EXPRESSED IN: 24 plant structures; EXPRESSED DURING: 13 growth stages; CONTAINS InterPro DOMAIN/s: BRO1 (InterPro:IPR004328); BEST Arabidopsis thaliana protein match is: Endosomal targeting BRO1-like domain-containing protein (TAIR:AT1G17940.1)
AT1G80650	-0,735	RNASE THREE-LIKE PROTEIN 1 (RTL1)	RNAse THREE-like protein 1 (RTL1); FUNCTIONS IN: double-stranded RNA binding, RNA binding; INVOLVED IN: biological_process unknown; LOCATED IN: intracellular; CONTAINS InterPro DOMAIN/s: Double-stranded RNA-binding (InterPro:IPR001159), Double-stranded RNA-binding-like (InterPro:IPR014720); BEST Arabidopsis thaliana protein match is: Double-stranded RNA-binding domain (DsRBD)-containing protein (TAIR:AT4G00420.1)
AT3G15580	-0,735	AUTOPHAGY 8H (APG8H)	Encodes APG8, a component of autophagy conjugation pathway. Delivered to the lumens of vacuole under nitrogen-starvation condition.
AT4G04800	-0,735	METHIONINE SULFOXIDE REDUCTASE B3 (MSRB3)	methionine sulfoxide reductase B3 (MSRB3); FUNCTIONS IN: peptide-methionine-(S)-S-oxide reductase activity; INVOLVED IN: oxidation reduction; LOCATED IN: nucleus; EXPRESSED IN: 23 plant structures; EXPRESSED DURING: 15 growth stages; CONTAINS InterPro DOMAIN/s: Methionine sulphoxide reductase B (InterPro:IPR002579), Mss4-like (InterPro:IPR011057); BEST Arabidopsis thaliana protein match is: methionine sulfoxide reductase B 2 (TAIR:AT4G21860.3)
AT4G37540	-0,736	LOB DOMAIN-CONTAINING PROTEIN 39 (LBD39)	LOB domain-containing protein 39 (LBD39); CONTAINS InterPro DOMAIN/s: Lateral organ boundaries, LOB (InterPro:IPR004883); BEST Arabidopsis thaliana protein match is: LOB domain-containing protein 38 (TAIR:AT3G49940.1)
AT4G04960	-0,737	L-TYPE LECTIN RECEPTOR KINASE VII.1 (LECRK-VII.1)	Concanavalin A-like lectin protein kinase family protein; FUNCTIONS IN: kinase activity; INVOLVED IN: protein amino acid phosphorylation; LOCATED IN: endomembrane system; EXPRESSED IN: 21 plant structures; EXPRESSED DURING: 12 growth stages; CONTAINS InterPro DOMAIN/s: Legume lectin, beta chain (InterPro:IPR001220), Protein kinase, ATP binding site (InterPro:IPR017441), Serine/threonine-protein kinase-like domain (InterPro:IPR017442), Concanavalin A-like lectin/glucanase, subgroup (InterPro:IPR013320), Protein kinase-like domain (InterPro:IPR011009), Serine/threonine-protein kinase, active site (InterPro:IPR008271), Protein kinase, catalytic domain (InterPro:IPR000719), Concanavalin A-like lectin/glucanase (InterPro:IPR008985); BEST Arabidopsis thaliana protein match is: Concanavalin A-like lectin protein kinase family protein (TAIR:AT4G28350.1)
AT3G25070	-0,738	RPM1 INTERACTING PROTEIN 4 (RIN4)	Encodes a member of the R protein complex and may represent a virulence target of type III pili effector proteins (virulence factors) from bacterial pathogens, which is 'guarded' by R protein complex (RPM1 and RPS2 proteins). RIN4 physically interacts with RPS2 and RPM1 in vivo. Bacterial avirulence (Avr) effectors AvrB, AvrRpm1, and AvrRpt2 induce a mobility shift in RIN4 and expression of AvrRpt2 induces rapid degradation of RIN4. RIN4 contains 2 sites for AvrRpt2 autocleavage, called RCS1 and RCS2. Overexpression of RIN4 inhibits multiple phenotypes associated with AvrRpt2 function and also inhibits PAMP-induced defense signaling. Attached to the plasma membrane at its carboxyl terminus. Cleaved by AvrRpt2 at two PxFGxW motifs, one releasing a large portion of RIN4 from the plasma membrane and both exposing amino-terminal residues that destabilized the carboxyl-terminal cleavage products by targeting them for N-end ubiquitylation and proteasomal degradation. Major virulence target of the TTSE HopF2Pto. The mRNA is cell-to-cell mobile.

AT3G62020	*-0,738*	GERMIN-LIKE PROTEIN 10 (GLP10)	germin-like protein (GLP10)
AT4G29690	*-0,738*		Alkaline-phosphatase-like family protein; FUNCTIONS IN: hydrolase activity, catalytic activity; INVOLVED IN: metabolic process, nucleotide metabolic process; LOCATED IN: endomembrane system; EXPRESSED IN: 9 plant structures; EXPRESSED DURING: LP.06 six leaves visible, LP.04 four leaves visible, 4 anthesis, petal differentiation and expansion stage, E expanded cotyledon stage; CONTAINS InterPro DOMAIN/s: Alkaline phosphatase-like, alpha/beta/alpha (InterPro:IPR017849), Type I phosphodiesterase/nucleotide pyrophosphatase/phosphate transferase (InterPro:IPR002591), Alkaline-phosphatase-like, core domain (InterPro:IPR017850); BEST Arabidopsis thaliana protein match is: Alkaline-phosphatase-like family protein (TAIR:AT4G29700.1)
AT5G59530	*-0,739*		2-oxoglutarate (2OG) and Fe(II)-dependent oxygenase superfamily protein; CONTAINS InterPro DOMAIN/s: Oxoglutarate/iron-dependent oxygenase (InterPro:IPR005123); BEST Arabidopsis thaliana protein match is: 2-oxoglutarate (2OG) and Fe(II)-dependent oxygenase superfamily protein (TAIR:AT5G59540.1)
AT2G41880	*-0,74*	GUANYLATE KINASE 1 (GK-1)	Guanylate kinase. Involved in nucleotide metabolism.
AT5G42690	*-0,74*		Protein of unknown function, DUF547; LOCATED IN: chloroplast; EXPRESSED IN: 17 plant structures; EXPRESSED DURING: 9 growth stages; CONTAINS InterPro DOMAIN/s: Protein of unknown function DUF547 (InterPro:IPR006869); BEST Arabidopsis thaliana protein match is: Protein of unknown function, DUF547 (TAIR:AT4G37080.3)
AT3G06380	*-0,741*	TUBBY-LIKE PROTEIN 9 (TLP9)	Member of TLP family
AT3G32980	*-0,741*		Peroxidase superfamily protein; FUNCTIONS IN: peroxidase activity, heme binding; INVOLVED IN: response to oxidative stress, oxidation reduction; LOCATED IN: cell wall, vacuole, membrane; EXPRESSED IN: leaf; EXPRESSED DURING: seedling growth; CONTAINS InterPro DOMAIN/s: Haem peroxidase (InterPro:IPR010255), Plant peroxidase (InterPro:IPR000823), Peroxidases heam-ligand binding site (InterPro:IPR019793), Peroxidase, active site (InterPro:IPR019794), Haem peroxidase, plant/fungal/bacterial (InterPro:IPR002016); BEST Arabidopsis thaliana protein match is: peroxidase CB (TAIR:AT3G49120.1)
AT2G41250	*-0,742*		Haloacid dehalogenase-like hydrolase (HAD) superfamily protein; FUNCTIONS IN: hydrolase activity, catalytic activity, phosphoglycolate phosphatase activity; INVOLVED IN: metabolic process; LOCATED IN: mitochondrion; EXPRESSED IN: 24 plant structures; EXPRESSED DURING: 15 growth stages; CONTAINS InterPro DOMAIN/s: Haloacid dehalogenase-like hydrolase (InterPro:IPR005834), Haloacid dehydrogenase/epoxide hydrolase (InterPro:IPR005833), HAD-superfamily hydrolase, subfamily IA, REG-2-like (InterPro:IPR011949), HAD-superfamily hydrolase, subfamily IA, variant 1 (InterPro:IPR006439); BEST Arabidopsis thaliana protein match is: Haloacid dehalogenase-like hydrolase (HAD) superfamily protein (TAIR:AT1G14310.1)
AT4G23670	*-0,742*		Polyketide cyclase/dehydrase and lipid transport superfamily protein; FUNCTIONS IN: copper ion binding; INVOLVED IN: response to cadmium ion, response to salt stress, defense response to bacterium; LOCATED IN: vacuole; EXPRESSED IN: 24 plant structures; EXPRESSED DURING: 14 growth stages; CONTAINS InterPro DOMAIN/s: Bet v I allergen (InterPro:IPR000916); BEST Arabidopsis thaliana protein match is: Polyketide cyclase/dehydrase and lipid transport superfamily protein (TAIR:AT4G23680.1)
AT1G21780	*-0,743*		BTB/POZ domain-containing protein. Contains similarity to gb:AJ000644 SPOP (speckle-type POZ protein) from Homo sapiens and contains a PF:00651 BTB/POZ domain. ESTs gb:T75841, gb:R89974, gb:R30221, gb:N96386, gb:T76457, gb:AI100013 and gb:T76456 come from this gene;supported by full-length. Interacts with CUL3A and CUL3B.
AT5G40470	*-0,743*		RNI-like superfamily protein; FUNCTIONS IN: molecular_function unknown; INVOLVED IN: biological_process unknown; LOCATED IN: chloroplast; EXPRESSED IN: 22 plant structures; EXPRESSED DURING: 13 growth stages; BEST Arabidopsis thaliana protein match is: F-box/RNI-like superfamily protein (TAIR:AT4G15475.1)
AT5G54040	*-0,743*		Cysteine/Histidine-rich C1 domain family protein; INVOLVED IN: intracellular signaling pathway; LOCATED IN: cellular_component unknown; EXPRESSED IN: stem, hypocotyl, root; CONTAINS InterPro DOMAIN/s: Protein kinase C-like, phorbol ester/diacylglycerol binding (InterPro:IPR002219), DC1 (InterPro:IPR004146), C1-like (InterPro:IPR011424); BEST Arabidopsis thaliana protein match is: Cysteine/Histidine-rich C1 domain family protein (TAIR:AT5G54050.1)

AT1G49240	*-0,744*	ACTIN 8 (ACT8)	Member of a subclass of actins composed of ACT2 and ACT8. Its mRNA is strongly expressed in strongly expressed in leaves, roots, stems, flowers, pollen, and siliques. However, protein expression, assayed by a ACT8:GUS fusion reporter, is very low in pollen.
AT2G34470	*-0,744*	UREASE ACCESSORY PROTEIN G (UREG)	Encodes a urease accessory protein which is essential for the activation of plant urease.
AT5G63910	*-0,744*	FARNESYLCYSTEINE LYASE (FCLY)	Encodes a farnesylcysteine lyase (EC 1.8.3.5) involved in a salvage /detoxification pathway of farnesylcysteine (FC) residues that are liberated during the degradation of prenylated proteins. Because FC is a competitive inhibitor of prenylcysteine methyltransferases involved in the down-regulation of ABA signaling, fcly mutants with elevated FC levels are hypersensitive to ABA. The protein also appears to be glycosylated when translated in vitro in the presence of microsomal membranes and it likely requires FAD for enzymatic activity.
AT5G67520	*-0,744*	ADENOSINE-5'-PHOSPHOSULFATE (APS) KINASE 4 (APK4)	Provides activated sulfate for the sulfation of secondary metabolites, including the glucosinolates. Redundant with APK3.
AT1G56220	*-0,745*		Dormancy/auxin associated family protein; CONTAINS InterPro DOMAIN/s: Dormancyauxin associated (InterPro:IPR008406); BEST Arabidopsis thaliana protein match is: Dormancy/auxin associated family protein (TAIR:AT1G54070.1)
AT3G22942	*-0,745*	G-PROTEIN GAMMA SUBUNIT 2 (AGG2)	heterotrimeric G-protein gamma subunit 2(AGG2)
AT4G38220	*-0,745*	AQUAPORIN INTERACTOR (AQI)	Peptidase M20/M25/M40 family protein; FUNCTIONS IN: hydrolase activity, metallopeptidase activity, aminoacylase activity; INVOLVED IN: response to zinc ion; LOCATED IN: vacuole; EXPRESSED IN: 23 plant structures; EXPRESSED DURING: 15 growth stages; CONTAINS InterPro DOMAIN/s: ArgE/DapE/ACY1/CPG2/YscS, conserved site (InterPro:IPR001261), Peptidase M20 (InterPro:IPR002933), N-acyl-L-amino-acid amidohydrolase (InterPro:IPR010159), Peptidase M20, dimerisation (InterPro:IPR011650); BEST Arabidopsis thaliana protein match is: Peptidase M20/M25/M40 family protein (TAIR:AT1G44820.1)
AT5G38000	*-0,746*		Zinc-binding dehydrogenase family protein; FUNCTIONS IN: oxidoreductase activity, binding, zinc ion binding, catalytic activity; INVOLVED IN: response to oxidative stress; CONTAINS InterPro DOMAIN/s: GroES-like (InterPro:IPR011032), NAD(P)-binding domain (InterPro:IPR016040), Alcohol dehydrogenase, C-terminal (InterPro:IPR013149), Alcohol dehydrogenase superfamily, zinc-containing (InterPro:IPR002085); BEST Arabidopsis thaliana protein match is: Zinc-binding dehydrogenase family protein (TAIR:AT5G37940.1)
AT2G44360	*-0,747*		unknown protein
AT3G62660	*-0,747*	GALACTURONOSYLTRANSFERASE-LIKE 7 (GATL7)	Encodes a protein with putative galacturonosyltransferase activity.
AT5G57685	*-0,747*	GLUTAMINE DUMPER 3 (GDU3)	Encodes a member of the GDU (glutamine dumper) family proteins involved in amino acid export: At4g31730 (GDU1), At4g25760 (GDU2), At5g57685 (GDU3), At2g24762 (GDU4), At5g24920 (GDU5), At3g30725 (GDU6) and At5g38770 (GDU7).
AT5G28080	*-0,749*	(WNK9)	Encodes a member of the WNK family (9 members in all) of protein kinases, the structural design of which is clearly distinct from those of other known protein kinases, such as receptor-like kinases and mitogen-activated protein kinases.
AT2G25210	*-0,75*		Ribosomal protein L39 family protein; FUNCTIONS IN: structural constituent of ribosome; INVOLVED IN: translation; LOCATED IN: ribosome, cytosolic large ribosomal subunit; EXPRESSED IN: 23 plant structures; EXPRESSED DURING: 13 growth stages; CONTAINS InterPro DOMAIN/s: Ribosomal protein L39e, conserved site (InterPro:IPR020083), Ribosomal protein L39e (InterPro:IPR000077); BEST Arabidopsis thaliana protein match is: Ribosomal protein L39 family protein (TAIR:AT4G31985.1)
AT5G25160	*-0,75*	ZINC FINGER PROTEIN 3 (ZFP3)	Encodes a zinc finger protein containing only a single zinc finger.
AT5G04950	*-0,751*	NICOTIANAMINE SYNTHASE 1 (NAS1)	Encodes a nicotianamide synthase.
AT1G01340	*-0,752*	CYCLIC NUCLEOTIDE GATED CHANNEL 10 (CNGC10)	member of Cyclic nucleotide gated channel family

AT1G72430	*-0,752*	SMALL AUXIN UPREGULATED RNA 78 (SAUR78)	SAUR-like auxin-responsive protein family ; CONTAINS InterPro DOMAIN/s: Auxin responsive SAUR protein (InterPro:IPR003676); BEST Arabidopsis thaliana protein match is: SAUR-like auxin-responsive protein family (TAIR:AT1G17345.1)
AT5G43600	*-0,752*	UREIDOGLYCOLATE AMIDOHYDROLASE (UAH)	Encodes a protein with ureidoglycolate amidohydrolase activity in vitro. It is 27% identical and 43% similar to the E. coli allantoate amidohydrolase (AAH), but, in vitro assays with purified protein and allantoate as a substrate do not show any increase in ammonium concentration, indicating that there this enzyme has no AAH activity. The mRNA is cell-to-cell mobile.
AT1G02520	*-0,753*	ATP-BINDING CASSETTE B11 (ABCB11)	Encodes an ATP-binding cassette (ABC) transporter. Expressed in the vascular tissue of primary stem. The mRNA is cell-to-cell mobile.
AT2G30930	*-0,753*		unknown protein; FUNCTIONS IN: molecular_function unknown; INVOLVED IN: biological_process unknown; LOCATED IN: chloroplast, plasma membrane, membrane; EXPRESSED IN: 24 plant structures; EXPRESSED DURING: 14 growth stages; BEST Arabidopsis thaliana protein match is: unknown protein (TAIR:AT1G06540.1)
AT3G16430	*-0,753*	JACALIN-RELATED LECTIN 31 (JAL31)	Encodes a protein that increases the beta-glucosidase activities of three scopolin glucosidases in vitro.
AT4G24230	*-0,753*	ACYL-COA-BINDING DOMAIN 3 (ACBP3)	acyl-CoA-binding protein ACBP3. Localized extracellularly in transiently expressed tobacco BY-2 cells and onion epidermal cells. Binds arachidonyl-CoA with high affinity. Microarray data shows up-regulation of many biotic- and abiotic-stress-related genes in an ACBP3 OE-1 in comparison to wild type.
AT5G61590	*-0,753*	DECREASE WAX BIOSYNTHESIS (DEWAX)	Encodes an AP2/ERF-type transcription factor that is preferentially expressed in the epidermis and induced by darkness and negatively regulates cuticular wax biosynthesis.
AT1G75820	*-0,754*	CLAVATA 1 (CLV1)	Putative receptor kinase with an extracellular leucine-rich domain. Controls shoot and floral meristem size, and contributes to establish and maintain floral meristem identity. Negatively regulated by KAPP (kinase-associated protein phosphatase). CLV3 peptide binds directly CLV1 ectodomain.
AT4G40060	*-0,754*	HOMEOBOX PROTEIN 16 (HB16)	Encodes a homeodomain leucine zipper class I (HD-Zip I) protein.
AT4G27450	*-0,755*		Aluminium induced protein with YGL and LRDR motifs; FUNCTIONS IN: molecular_function unknown; INVOLVED IN: biological_process unknown; LOCATED IN: cytosol, nucleus, plasma membrane; EXPRESSED IN: 25 plant structures; EXPRESSED DURING: 14 growth stages; BEST Arabidopsis thaliana protein match is: Aluminium induced protein with YGL and LRDR motifs (TAIR:AT3G15450.1)
AT4G28880	*-0,755*	CASEIN KINASE I-LIKE 3 (ckl3)	casein kinase I-like 3 (ckl3); FUNCTIONS IN: protein serine/threonine kinase activity, protein kinase activity, kinase activity, ATP binding; INVOLVED IN: protein amino acid phosphorylation; LOCATED IN: cellular_component unknown; CONTAINS InterPro DOMAIN/s: Protein kinase, ATP binding site (InterPro:IPR017441), Protein kinase, catalytic domain (InterPro:IPR000719), Serine/threonine-protein kinase-like domain (InterPro:IPR017442), Protein kinase-like domain (InterPro:IPR011009), Serine/threonine-protein kinase, active site (InterPro:IPR008271); BEST Arabidopsis thaliana protein match is: casein kinase I-like 4 (TAIR:AT4G28860.1)
AT1G09920	*-0,756*		TRAF-type zinc finger-related; BEST Arabidopsis thaliana protein match is: Ubiquitin fusion degradation UFD1 family protein (TAIR:AT4G15420.1)
AT3G49940	*-0,756*	LOB DOMAIN-CONTAINING PROTEIN 38 (LBD38)	LOB domain-containing protein 38 (LBD38); CONTAINS InterPro DOMAIN/s: Lateral organ boundaries, LOB (InterPro:IPR004883); BEST Arabidopsis thaliana protein match is: LOB domain-containing protein 37 (TAIR:AT5G67420.1)
AT5G19120	*-0,756*		Eukaryotic aspartyl protease family protein; FUNCTIONS IN: aspartic-type endopeptidase activity; INVOLVED IN: proteolysis; LOCATED IN: endomembrane system; EXPRESSED IN: 24 plant structures; EXPRESSED DURING: 13 growth stages; CONTAINS InterPro DOMAIN/s: Peptidase aspartic (InterPro:IPR021109), Peptidase aspartic, catalytic (InterPro:IPR009007), Peptidase A1 (InterPro:IPR001461); BEST Arabidopsis thaliana protein match is: Eukaryotic aspartyl protease family protein (TAIR:AT1G03220.1)
AT4G11330	*-0,759*	MAP KINASE 5 (MPK5)	MAP kinase
AT1G48600	*-0,76*	PHOSPHOETHANOLAMINE N-METHYLTRANSFERASE (PMEAMT)	Encodes a phosphoethanolamine N-methyltransferase that catalyses the last two methylation steps of the three sequential methylations of phosphoethanolamine (PEA) that are required for the synthesis of phosphocholine (PCho) in plants.

AT2G03200	-0,76		Eukaryotic aspartyl protease family protein; FUNCTIONS IN: aspartic-type endopeptidase activity; INVOLVED IN: proteolysis; LOCATED IN: endomembrane system; EXPRESSED IN: 10 plant structures; EXPRESSED DURING: 4 anthesis, C globular stage, petal differentiation and expansion stage; CONTAINS InterPro DOMAIN/s: Peptidase aspartic (InterPro:IPR021109), Peptidase aspartic, catalytic (InterPro:IPR009007), Peptidase A1 (InterPro:IPR001461); BEST Arabidopsis thaliana protein match is: Eukaryotic aspartyl protease family protein (TAIR:AT1G01300.1)
AT5G28770	-0,76	(BZO2H3)	bZIP protein BZO2H3 mRNA, partial cds
AT1G79270	-0,761	EVOLUTIONARILY CONSERVED C-TERMINAL REGION 8 (ECT8)	evolutionarily conserved C-terminal region 8 (ECT8); CONTAINS InterPro DOMAIN/s: YTH domain (InterPro:IPR007275); BEST Arabidopsis thaliana protein match is: evolutionarily conserved C-terminal region 6 (TAIR:AT3G17330.1)
AT3G06300	-0,761	PROLYL 4-HYDROXYLASE 2 (P4H2)	Encodes a prolyl-4 hydroxylase that can hydroxylate poly(L-proline)and other proline rich peptides, including those with sequences corresponding to those in arabinogalactan proteins and extensins. The mRNA is cell-to-cell mobile.
AT2G16760	-0,762		Calcium-dependent phosphotriesterase superfamily protein; FUNCTIONS IN: molecular_function unknown; LOCATED IN: endoplasmic reticulum; EXPRESSED IN: 10 plant structures; EXPRESSED DURING: 6 growth stages; CONTAINS InterPro DOMAIN/s: SMP-30/Gluconolaconase/LRE-like region (InterPro:IPR013658), Six-bladed beta-propeller, TolB-like (InterPro:IPR011042); BEST Arabidopsis thaliana protein match is: Calcium-dependent phosphotriesterase superfamily protein (TAIR:AT2G47370.1)
AT3G55990	-0,762	ESKIMO 1 (ESK1)	Encodes ESK1 (Eskimo1). A member of a large gene family of DUF231 domain proteins whose members encode a total of 45 proteins of unknown function. ESK1 functions as a negative regulator of cold acclimation. Mutations in the ESK1 gene provides strong freezing tolerance. A member of the TBL (TRICHOME BIREFRINGENCE-LIKE) gene family containing a plant-specific DUF231 (domain of unknown function) domain. TBL gene family has 46 members, two of which (TBR/AT5G06700 and TBL3/AT5G01360) have been shown to be involved in the synthesis and deposition of secondary wall cellulose, presumably by influencing the esterification state of pectic polymers. A nomenclature for this gene family has been proposed (Volker Bischoff & Wolf Scheible, 2010, personal communication). The mRNA is cell-to-cell mobile.
AT3G50360	-0,763	CENTRIN2 (CEN2)	centrin2 (CEN2); FUNCTIONS IN: calcium ion binding; INVOLVED IN: biological_process unknown; LOCATED IN: plasma membrane; EXPRESSED IN: 24 plant structures; EXPRESSED DURING: 15 growth stages; CONTAINS InterPro DOMAIN/s: EF-Hand 1, calcium-binding site (InterPro:IPR018247), EF-HAND 2 (InterPro:IPR018249), EF-hand-like domain (InterPro:IPR011992), Calcium-binding EF-hand (InterPro:IPR002048), EF-hand (InterPro:IPR018248); BEST Arabidopsis thaliana protein match is: centrin 2 (TAIR:AT4G37010.2)
AT1G12160	-0,764	(FMOGS-OX7)	Encodes a flavin-containing monooxygenases involved in biosynthesis of aliphatic glucosinolates.
AT2G43850	-0,764	INTEGRIN-LINKED KINASE1 (ILK1)	Integrin-linked protein kinase family; FUNCTIONS IN: protein serine/threonine/tyrosine kinase activity, kinase activity; INVOLVED IN: regulation of signal transduction, protein amino acid phosphorylation; LOCATED IN: chloroplast; EXPRESSED IN: 24 plant structures; EXPRESSED DURING: 13 growth stages; CONTAINS InterPro DOMAIN/s: Integrin-linked protein kinase (InterPro:IPR016253), Serine/threonine-protein kinase domain (InterPro:IPR002290), Serine-threonine/tyrosine-protein kinase (InterPro:IPR001245), Protein kinase-like domain (InterPro:IPR011009), Serine/threonine-protein kinase, active site (InterPro:IPR008271), Protein kinase, catalytic domain (InterPro:IPR000719), Ankyrin repeat-containing domain (InterPro:IPR020683), Tyrosine-protein kinase, catalytic domain (InterPro:IPR020635), Ankyrin repeat (InterPro:IPR002110); BEST Arabidopsis thaliana protein match is: Integrin-linked protein kinase family (TAIR:AT3G59830.1)
AT1G11450	-0,766	USUALLY MULTIPLE ACIDS MOVE IN AND OUT TRANSPORTERS 27 (UMAMIT27)	nodulin MtN21-like transporter family protein
AT1G18470	-0,766		Transmembrane Fragile-X-F-associated protein; FUNCTIONS IN: zinc ion binding; LOCATED IN: endomembrane system; EXPRESSED IN: 24 plant structures; EXPRESSED DURING: 13 growth stages; CONTAINS InterPro DOMAIN/s: Transmembrane Fragile-X-F-associated protein (InterPro:IPR019396), Zinc finger, RING-type (InterPro:IPR001841); BEST Arabidopsis thaliana protein match is: Transmembrane Fragile-X-F-associated protein (TAIR:AT1G73950.1)

AT2G40160	*-0,766*	(TBL30)	Encodes a member of the TBL (TRICHOME BIREFRINGENCE-LIKE) gene family containing a plant-specific DUF231 (domain of unknown function) domain. TBL gene family has 46 members, two of which (TBR/AT5G06700 and TBL3/AT5G01360) have been shown to be involved in the synthesis and deposition of secondary wall cellulose, presumably by influencing the esterification state of pectic polymers. A nomenclature for this gene family has been proposed (Volker Bischoff & Wolf Scheible, 2010, personal communication).
AT5G43760	*-0,766*	3-KETOACYL-COA SYNTHASE 20 (KCS20)	Encodes KCS20, a member of the 3-ketoacyl-CoA synthase family involved in the biosynthesis of VLCFA (very long chain fatty acids). The mRNA is cell-to-cell mobile.
AT3G21550	*-0,768*	DUF679 DOMAIN MEMBRANE PROTEIN 2 (DMP2)	DUF679 domain membrane protein 2 (DMP2); CONTAINS InterPro DOMAIN/s: Protein of unknown function DUF679 (InterPro:IPR007770); BEST Arabidopsis thaliana protein match is: DUF679 domain membrane protein 1 (TAIR:AT3G21520.1)
AT4G25570	*-0,768*	(ACYB-2)	Encodes cytochrome b561.
AT4G27310	*-0,768*	B-BOX DOMAIN PROTEIN 28 (BBX28)	B-box type zinc finger family protein; FUNCTIONS IN: sequence-specific DNA binding transcription factor activity, zinc ion binding; INVOLVED IN: regulation of transcription; LOCATED IN: intracellular; CONTAINS InterPro DOMAIN/s: Zinc finger, B-box (InterPro:IPR000315); BEST Arabidopsis thaliana protein match is: B-box type zinc finger family protein (TAIR:AT5G54470.1)
AT4G37610	*-0,768*	BTB AND TAZ DOMAIN PROTEIN 5 (bt5)	BTB and TAZ domain protein. Located in cytoplasm and expressed in fruit, flower and leaves.
AT1G59580	*-0,769*	MITOGEN-ACTIVATED PROTEIN KINASE HOMOLOG 2 (MPK2)	encodes a mitogen-activated kinase involved in innate immunity The mRNA is cell-to-cell mobile.
AT1G70810	*-0,769*	C2-DOMAIN ABA-RELATED7 (CAR7)	Calcium-dependent lipid-binding (CaLB domain) family protein; CONTAINS InterPro DOMAIN/s: C2 membrane targeting protein (InterPro:IPR018029), C2 calcium/lipid-binding domain, CaLB (InterPro:IPR008973), C2 calcium-dependent membrane targeting (InterPro:IPR000008); BEST Arabidopsis thaliana protein match is: Calcium-dependent lipid-binding (CaLB domain) family protein (TAIR:AT1G70800.1)
AT3G27020	*-0,769*	YELLOW STRIPE LIKE 6 (YSL6)	Arabidopsis thaliana metal-nicotianamine transporter YSL6
AT4G19860	*-0,769*		Encodes a cytosolic calcium-independent phospholipase A.
AT4G20870	*-0,772*	FATTY ACID HYDROXYLASE 2 (FAH2)	encodes a fatty acid hydroxylase, required for the AtBI-1-mediated suppression of programmed cell death.
AT5G66480	*-0,772*		unknown protein; FUNCTIONS IN: molecular_function unknown; INVOLVED IN: biological_process unknown; LOCATED IN: chloroplast; BEST Arabidopsis thaliana protein match is: unknown protein (TAIR:AT3G50910.1)
AT1G70410	*-0,773*	BETA CARBONIC ANHYDRASE 4 (BCA4)	Encodes a putative beta-carbonic anhydrase betaCA4. Together with betaCA1 (At3g01500) regulates CO2-controlled stomatal movements in guard cells.
AT4G11820	*-0,773*	HYDROXYMETHYLGLUTARYL-COA SYNTHASE (HMGS)	Encodes a protein with hydroxymethylglutaryl-CoA synthase activity which was characterized by phenotypical complementation of the S. cerevisiae mutant.
AT1G70230	*-0,774*	TRICHOME BIREFRINGENCE-LIKE 27 (TBL27)	Encodes a member of the TBL (TRICHOME BIREFRINGENCE-LIKE) gene family containing a plant-specific DUF231 (domain of unknown function) domain. A putative xyloglucan O-acetyltransferase. TBL gene family has 46 members, two of which (TBR/AT5G06700 and TBL3/AT5G01360) have been shown to be involved in the synthesis and deposition of secondary wall cellulose, presumably by influencing the esterification state of pectic polymers. A nomenclature for this gene family has been proposed (Volker Bischoff & Wolf Scheible, 2010, personal communication).
AT2G04690	*-0,775*		Pyridoxamine 5'-phosphate oxidase family protein; FUNCTIONS IN: FMN binding; INVOLVED IN: biological_process unknown; LOCATED IN: vacuole; EXPRESSED IN: 22 plant structures; EXPRESSED DURING: 13 growth stages; CONTAINS InterPro DOMAIN/s: Cellular repressor of E1A-stimulated genes (CREG) (InterPro:IPR014631), FMN-binding split barrel (InterPro:IPR012349), FMN-binding split barrel, related (InterPro:IPR009002)
AT5G03260	*-0,775*	LACCASE 11 (LAC11)	LAC11 is a putative laccase, a member of laccase family of genes (17 members in Arabidopsis).
AT2G23030	*-0,776*	SNF1-RELATED PROTEIN KINASE 2.9 (SNRK2.9)	encodes a member of SNF1-related protein kinases (SnRK2)

AT1G05700	*-0,777*		Leucine-rich repeat transmembrane protein kinase protein; FUNCTIONS IN: kinase activity; INVOLVED IN: protein amino acid phosphorylation; LOCATED IN: endomembrane system; EXPRESSED IN: shoot, stem, hypocotyl, root; CONTAINS InterPro DOMAIN/s: Protein kinase, ATP binding site (InterPro:IPR017441), Protein kinase, catalytic domain (InterPro:IPR000719), Leucine-rich repeat (InterPro:IPR001611), Serine-threonine/tyrosine-protein kinase (InterPro:IPR001245), Protein kinase-like domain (InterPro:IPR011009), Serine/threonine-protein kinase, active site (InterPro:IPR008271); BEST Arabidopsis thaliana protein match is: Leucine-rich repeat transmembrane protein kinase protein (TAIR:AT4G29990.1)
AT1G69450	*-0,777*		Early-responsive to dehydration stress protein (ERD4); LOCATED IN: endomembrane system, membrane; EXPRESSED IN: 23 plant structures; EXPRESSED DURING: 13 growth stages; CONTAINS InterPro DOMAIN/s: Protein of unknown function DUF221 (InterPro:IPR003864); BEST Arabidopsis thaliana protein match is: hypothetical protein 1 (TAIR:AT3G01100.1)
AT2G20490	*-0,777*	(NOP10)	NOP10; FUNCTIONS IN: RNA binding; INVOLVED IN: polar nucleus fusion; LOCATED IN: nucleolus, Cajal body; EXPRESSED IN: 22 plant structures; EXPRESSED DURING: 13 growth stages; CONTAINS InterPro DOMAIN/s: H/ACA ribonucleoprotein complex, subunit Nop10 (InterPro:IPR007264)
AT2G36310	*-0,777*	URIDINE-RIBOHYDROLASE 1 (URH1)	Encodes a cytoplasmic nucleoside hydrolase. It has the highest levels of activity with uridine followed by xanthosine. It shows little activity with inosine and none with cytidine. Mutant analyses indicate that it plays a role in purine and pyrimidine catabolism.
AT1G21000	*-0,779*		PLATZ transcription factor family protein; CONTAINS InterPro DOMAIN/s: Protein of unknown function DUF597 (InterPro:IPR006734); BEST Arabidopsis thaliana protein match is: PLATZ transcription factor family protein (TAIR:AT1G76590.1)
AT1G50580	*-0,779*		UDP-Glycosyltransferase superfamily protein; FUNCTIONS IN: transferase activity, transferring glycosyl groups; INVOLVED IN: metabolic process; LOCATED IN: cellular_component unknown; EXPRESSED IN: root; CONTAINS InterPro DOMAIN/s: UDP-glucuronosyl/UDP-glucosyltransferase (InterPro:IPR002213); BEST Arabidopsis thaliana protein match is: UDP-Glycosyltransferase superfamily protein (TAIR:AT3G29630.1)
AT3G16920	*-0,779*	CHITINASE-LIKE PROTEIN 2 (CTL2)	Encodes a chitinase-like protein expressed predominantly in stems. Mutants accumulate ligning in etiolated hypocotyls.
AT2G28440	*-0,781*		proline-rich family protein; LOCATED IN: endomembrane system; EXPRESSED IN: male gametophyte, flower, pollen tube; EXPRESSED DURING: L mature pollen stage, M germinated pollen stage, 4 anthesis
AT2G39380	*-0,783*	EXOCYST SUBUNIT EXO70 FAMILY PROTEIN H2 (EXO70H2)	A member of EXO70 gene family, putative exocyst subunits, conserved in land plants. Arabidopsis thaliana contains 23 putative EXO70 genes, which can be classified into eight clusters on the phylogenetic tree.
AT3G13782	*-0,783*	NUCLEOSOME ASSEMBLY PROTEIN1;4 (NAP1;4)	Plants mutated in three ubiquitously expressed NAP1 genes (NAP1;1~NAP1;3) and organ-specifically expressed NAP1;4 gene show hypersensitivity to genotoxic stresses including UV and DSB-inducing agent Bleomycin. The NAP1 genes act synergistically with NRP genes in promoting somatic homologous recombination.
AT1G73270	*-0,784*	SERINE CARBOXYPEPTIDASE-LIKE 6 (scpl6)	serine carboxypeptidase-like 6 (scpl6); FUNCTIONS IN: serine-type carboxypeptidase activity; INVOLVED IN: proteolysis; LOCATED IN: endomembrane system; EXPRESSED IN: root; CONTAINS InterPro DOMAIN/s: Peptidase S10, serine carboxypeptidase (InterPro:IPR001563); BEST Arabidopsis thaliana protein match is: serine carboxypeptidase-like 3 (TAIR:AT1G73280.1)
AT2G01580	*-0,784*		unknown protein; BEST Arabidopsis thaliana protein match is: unknown protein (TAIR:AT3G07510.2)
AT3G16450	*-0,785*	JACALIN-RELATED LECTIN 33 (JAL33)	Mannose-binding lectin superfamily protein; FUNCTIONS IN: molecular_function unknown; INVOLVED IN: response to zinc ion, response to cold; LOCATED IN: nucleus; EXPRESSED IN: 6 plant structures; EXPRESSED DURING: LP.04 four leaves visible, seedling growth; CONTAINS InterPro DOMAIN/s: Mannose-binding lectin (InterPro:IPR001229); BEST Arabidopsis thaliana protein match is: myrosinase-binding protein-like protein-300B (TAIR:AT3G16440.1)

AT5G35370	*-0,785*		S-locus lectin protein kinase family protein; FUNCTIONS IN: in 6 functions; INVOLVED IN: protein amino acid phosphorylation; LOCATED IN: endomembrane system; EXPRESSED IN: 11 plant structures; EXPRESSED DURING: 6 growth stages; CONTAINS InterPro DOMAIN/s: Curculin-like (mannose-binding) lectin (InterPro:IPR001480), Protein kinase, ATP binding site (InterPro:IPR017441), Serine/threonine-protein kinase domain (InterPro:IPR002290), Serine/threonine-protein kinase-like domain (InterPro:IPR017442), Protein kinase-like domain (InterPro:IPR011009), Serine/threonine-protein kinase, active site (InterPro:IPR008271), Protein kinase, catalytic domain (InterPro:IPR000719), Tyrosine-protein kinase, catalytic domain (InterPro:IPR020635); BEST Arabidopsis thaliana protein match is: S-domain-2 5 (TAIR:AT4G32300.1)
AT5G42440	*-0,785*		Protein kinase superfamily protein; FUNCTIONS IN: protein serine/threonine kinase activity, protein kinase activity, kinase activity, ATP binding; INVOLVED IN: protein amino acid phosphorylation; LOCATED IN: endomembrane system; EXPRESSED IN: 17 plant structures; EXPRESSED DURING: 10 growth stages; CONTAINS InterPro DOMAIN/s: Protein kinase, ATP binding site (InterPro:IPR017441), Protein kinase, catalytic domain (InterPro:IPR000719), Serine-threonine/tyrosine-protein kinase (InterPro:IPR001245), Protein kinase-like domain (InterPro:IPR011009), Serine/threonine-protein kinase, active site (InterPro:IPR008271); BEST Arabidopsis thaliana protein match is: phytosulfokin receptor 1 (TAIR:AT2G02220.1)
AT2G26480	*-0,786*	UDP-GLUCOSYL TRANSFERASE 76D1 (UGT76D1)	UDP-glucosyl transferase 76D1 (UGT76D1); FUNCTIONS IN: UDP-glycosyltransferase activity, quercetin 7-O-glucosyltransferase activity, transferase activity, transferring glycosyl groups; INVOLVED IN: metabolic process; EXPRESSED IN: 10 plant structures; EXPRESSED DURING: 4 anthesis, LP.02 two leaves visible, petal differentiation and expansion stage, E expanded cotyledon stage, D bilateral stage; CONTAINS InterPro DOMAIN/s: UDP-glucuronosyl/UDP-glucosyltransferase (InterPro:IPR002213); BEST Arabidopsis thaliana protein match is: UDP-glucosyl transferase 76E1 (TAIR:AT5G59580.1)
AT3G21700	*-0,786*	(SGP2)	Monomeric G protein. Expressed in root epidermal cells that are destined to become atrichoblasts. Also expressed during pollen development and in the pollen tube tip.
AT3G23560	*-0,786*	ABERRANT LATERAL ROOT FORMATION 5 (ALF5)	Member of the multidrug and toxic compound extrusion (MATE) family, protects roots from inhibitory compounds.
AT4G30010	*-0,786*		unknown protein; FUNCTIONS IN: molecular_function unknown; INVOLVED IN: biological_process unknown; LOCATED IN: mitochondrion, plastid; EXPRESSED IN: 26 plant structures; EXPRESSED DURING: 15 growth stages
AT5G09290	*-0,786*		Inositol monophosphatase family protein; FUNCTIONS IN: 3'(2'),5'-bisphosphate nucleotidase activity, inositol or phosphatidylinositol phosphatase activity; INVOLVED IN: sulfur metabolic process; EXPRESSED IN: 6 plant structures; EXPRESSED DURING: LP.06 six leaves visible, LP.04 four leaves visible, 4 anthesis; CONTAINS InterPro DOMAIN/s: Inositol monophosphatase (InterPro:IPR000760), 3(2),5 -bisphosphate nucleotidase HAL2 (InterPro:IPR006239), Inositol monophosphatase, metal-binding site (InterPro:IPR020583); BEST Arabidopsis thaliana protein match is: Inositol monophosphatase family protein (TAIR:AT5G64000.1)
AT5G50740	*-0,786*		Heavy metal transport/detoxification superfamily protein ; FUNCTIONS IN: metal ion binding; INVOLVED IN: metal ion transport; LOCATED IN: cellular_component unknown; EXPRESSED IN: 20 plant structures; EXPRESSED DURING: 13 growth stages; CONTAINS InterPro DOMAIN/s: Heavy metal transport/detoxification protein (InterPro:IPR006121); BEST Arabidopsis thaliana protein match is: farnesylated protein 3 (TAIR:AT5G63530.2)
AT1G17170	*-0,787*	GLUTATHIONE S-TRANSFERASE TAU 24 (GSTU24)	Encodes glutathione transferase belonging to the tau class of GSTs. Naming convention according to Wagner et al. (2002). It is involved in the detoxification of the environmental pollutant 2,4,6-trinitrotoluene. Arabidopsis plants over-expressing At1g17170 were more resistant to TNT, removed more TNT from sterile and soil-based media, and had reduced levels of glutathione when grown in the presence of TNT.
AT1G26440	*-0,787*	UREIDE PERMEASE 5 (UPS5)	uptake assays in yeast mutants indicated this splice variant is a cellular importer for allantoin, uracil and xanthine
AT1G30550	*-0,787*	GLCNAC KINASE (GNK)	S-adenosyl-L-methionine-dependent methyltransferases superfamily protein; FUNCTIONS IN: methyltransferase activity; INVOLVED IN: RNA capping, RNA methylation; EXPRESSED IN: 21 plant structures; EXPRESSED DURING: 12 growth stages; CONTAINS InterPro DOMAIN/s: RNA cap guanine-N2 methyltransferase (InterPro:IPR019012); BEST Arabidopsis thaliana protein match is: S-adenosyl-L-methionine-dependent methyltransferases superfamily protein (TAIR:AT1G45231.2)
AT1G79670	*-0,788*	RESISTANCE TO FUSARIUM OXYSPORUM 1 (RFO1)	Encodes a receptor-like kinase that does not contain an extracellular leucine-rich repeat domain. A novel type of dominant disease-resistance protein that confers resistance to a broad spectrum of Fusarium races.

AT3G02780	*-0,789*	ISOPENTENYL PYROPHOSPHATE:DIMETHYLALLYL PYROPHOSPHATE ISOMERASE 2 (IPP2)	Encodes a protein with isopentenyl diphosphate:dimethylallyl diphosphate isomerase activity. There is genetic evidence that it functions in the mevalonate, but not the MEP biosynthetic pathway.
AT1G75680	*-0,79*	GLYCOSYL HYDROLASE 9B7 (GH9B7)	glycosyl hydrolase 9B7 (GH9B7); FUNCTIONS IN: hydrolase activity, hydrolyzing O-glycosyl compounds, catalytic activity; INVOLVED IN: carbohydrate metabolic process; LOCATED IN: plasma membrane; EXPRESSED IN: 23 plant structures; EXPRESSED DURING: 13 growth stages; CONTAINS InterPro DOMAIN/s: Six-hairpin glycosidase (InterPro:IPR012341), Glycoside hydrolase, family 9, active site (InterPro:IPR018221), Six-hairpin glycosidase-like (InterPro:IPR008928), Glycoside hydrolase, family 9 (InterPro:IPR001701); BEST Arabidopsis thaliana protein match is: glycosyl hydrolase 9B5 (TAIR:AT1G19940.1)
AT3G20935	*-0,79*	CYTOCHROME P450, FAMILY 705, SUBFAMILY A, POLYPEPTIDE 28 (CYP705A28)	cytochrome P450, family 705, subfamily A, polypeptide 28 (CYP705A28); FUNCTIONS IN: electron carrier activity, monooxygenase activity, iron ion binding, heme binding; INVOLVED IN: oxidation reduction; LOCATED IN: cellular_component unknown; CONTAINS InterPro DOMAIN/s: Cytochrome P450 (InterPro:IPR001128), Cytochrome P450, conserved site (InterPro:IPR017972), Cytochrome P450, E-class, group I (InterPro:IPR002401); BEST Arabidopsis thaliana protein match is: cytochrome P450, family 705, subfamily A, polypeptide 30 (TAIR:AT3G20940.1)
AT5G64880	*-0,79*		unknown protein
AT2G30520	*-0,791*	ROOT PHOTOTROPISM 2 (RPT2)	light inducible root phototropism 2 encoding a signal transducer of the phototropic response in Arabidopsis
AT5G05090	*-0,791*		Homeodomain-like superfamily protein; CONTAINS InterPro DOMAIN/s: Homeodomain-like (InterPro:IPR009057), Myb, DNA-binding (InterPro:IPR014778), HTH transcriptional regulator, Myb-type, DNA-binding (InterPro:IPR017930), Myb-like DNA-binding domain, SHAQKYF class (InterPro:IPR006447), Homeodomain-related (InterPro:IPR012287); BEST Arabidopsis thaliana protein match is: Homeodomain-like superfamily protein (TAIR:AT3G10760.1)
AT1G22740	*-0,792*	RAB GTPASE HOMOLOG G3B (RABG3B)	GTP-binding protein Rab7
AT4G39670	*-0,792*		Glycolipid transfer protein (GLTP) family protein; FUNCTIONS IN: glycolipid transporter activity, glycolipid binding; INVOLVED IN: glycolipid transport; LOCATED IN: cytoplasm; EXPRESSED IN: 16 plant structures; EXPRESSED DURING: 10 growth stages; CONTAINS InterPro DOMAIN/s: Glycolipid transfer protein, GLTP (InterPro:IPR014830); BEST Arabidopsis thaliana protein match is: Glycolipid transfer protein (GLTP) family protein (TAIR:AT2G34690.1)
AT5G62260	*-0,792*	AT-HOOK MOTIF NUCLEAR LOCALIZED PROTEIN 6 (AHL6)	AT hook motif DNA-binding family protein; FUNCTIONS IN: DNA binding; INVOLVED IN: regulation of transcription, DNA-dependent; LOCATED IN: nucleus; EXPRESSED IN: 11 plant structures; EXPRESSED DURING: 7 growth stages; CONTAINS InterPro DOMAIN/s: Protein of unknown function DUF296 (InterPro:IPR005175), AT hook, DNA-binding motif (InterPro:IPR017956); BEST Arabidopsis thaliana protein match is: AT hook motif DNA-binding family protein (TAIR:AT4G25320.1)
AT1G70290	*-0,793*	TREHALOSE-6-PHOSPHATASE SYNTHASE S8 (TPS8)	Encodes an enzyme putatively involved in trehalose biosynthesis. Though the protein has both trehalose-6-phosphate synthase (TPS)-like and trehalose-6-phosphate phosphatase (TPP)-like domains, neither activity has been detected in enzymatic assays nor has the protein been able to complement yeast TPS or TPP mutants.
AT2G02070	*-0,793*	INDETERMINATE(ID)-DOMAIN 5 (IDD5)	RAVEN is part of the network regulated by BLJUEJAY, JACKDAW, SACRECROW and SHORT-ROOT to regulate root tissue patterning through cell lineage specification and asymmetric cell division. RAVEN is directly activated by SHORT-ROOT and directly repressed by JACKDAW.
AT2G41310	*-0,793*	RESPONSE REGULATOR 3	Encodes an A- type response Regulator that is primarily expressed in the root and is involved in cytokinin-mediated signalling.
AT5G24870	*-0,794*		RING/U-box superfamily protein; FUNCTIONS IN: zinc ion binding; EXPRESSED IN: 20 plant structures; EXPRESSED DURING: 11 growth stages; CONTAINS InterPro DOMAIN/s: Zinc finger, RING-type (InterPro:IPR001841), Zinc finger, C3HC4 RING-type (InterPro:IPR018957); BEST Arabidopsis thaliana protein match is: RING/U-box superfamily protein (TAIR:AT5G10650.2)

AT5G59700	*-0,794*		Protein kinase superfamily protein; FUNCTIONS IN: kinase activity; INVOLVED IN: protein amino acid phosphorylation; LOCATED IN: plasma membrane; EXPRESSED IN: 8 plant structures; EXPRESSED DURING: 4 anthesis, petal differentiation and expansion stage; CONTAINS InterPro DOMAIN/s: Protein kinase, ATP binding site (InterPro:IPR017441), Malectin/receptor-like protein kinase (InterPro:IPR021720), Protein kinase, catalytic domain (InterPro:IPR000719), Serine-threonine/tyrosine-protein kinase (InterPro:IPR001245), Protein kinase-like domain (InterPro:IPR011009), Serine/threonine-protein kinase, active site (InterPro:IPR008271); BEST Arabidopsis thaliana protein match is: hercules receptor kinase 1 (TAIR:AT3G46290.1)
AT5G67260	*-0,794*	CYCLIN D3;2 (CYCD3;2)	Encode CYCD3;2, a CYCD3 D-type cyclin. Important for determining cell number in developing lateral organs. Mediating cytokinin effects in apical growth and development.
AT1G65520	*-0,795*	DELTA(3), DELTA(2)-ENOYL COA ISOMERASE 1 (ECI1)	encodes a peroxisomal delta3, delta2-enoyl CoA isomerase, involved in unsaturated fatty acid degradation
AT4G01330	*-0,795*		Protein kinase superfamily protein; FUNCTIONS IN: protein serine/threonine kinase activity, protein kinase activity, kinase activity, ATP binding; INVOLVED IN: protein amino acid phosphorylation; EXPRESSED IN: 22 plant structures; EXPRESSED DURING: 13 growth stages; CONTAINS InterPro DOMAIN/s: Protein kinase, ATP binding site (InterPro:IPR017441), Protein kinase, catalytic domain (InterPro:IPR000719), Serine/threonine-protein kinase domain (InterPro:IPR002290), Tyrosine-protein kinase, catalytic domain (InterPro:IPR020635), Serine/threonine-protein kinase-like domain (InterPro:IPR017442), Serine/threonine-protein kinase, active site (InterPro:IPR008271), Protein kinase-like domain (InterPro:IPR011009); BEST Arabidopsis thaliana protein match is: Protein kinase superfamily protein (TAIR:AT1G01540.2).
AT4G31130	*-0,795*		Protein of unknown function (DUF1218); FUNCTIONS IN: molecular_function unknown; INVOLVED IN: biological_process unknown; LOCATED IN: plasma membrane; EXPRESSED IN: 25 plant structures; EXPRESSED DURING: 15 growth stages; CONTAINS InterPro DOMAIN/s: Protein of unknown function DUF1218 (InterPro:IPR009606)
AT5G07220	*-0,795*	BCL-2-ASSOCIATED ATHANOGENE 3 (BAG3)	A member of Arabidopsis BAG (Bcl-2-associated athanogene) proteins, plant homologs of mammalian regulators of apoptosis. Plant BAG proteins are multi-functional and remarkably similar to their animal counterparts, as they regulate apoptotic-like processes ranging from pathogen attack, to abiotic stress, to plant development.
AT3G06370	*-0,796*	SODIUM HYDROGEN EXCHANGER 4 (NHX4)	member of Sodium proton exchanger family
AT4G38160	*-0,796*	PIGMENT DEFECTIVE 191 (pde191)	Encodes a member of the mitochondrial transcription termination factor family. MTERF6 is localized to both the mitochondria and chloroplast. It is required for ILE tRNA maturation during chloroplast development. Loss of function of MTERF6 results in plants with defective chloroplasts or reduced rate of photosynthesis when MTERF6 expression is low.
AT5G62900	*-0,796*		unknown protein; FUNCTIONS IN: molecular_function unknown; INVOLVED IN: N-terminal protein myristoylation; LOCATED IN: cellular_component unknown; EXPRESSED IN: 22 plant structures; EXPRESSED DURING: 12 growth stages; BEST Arabidopsis thaliana protein match is: unknown protein (TAIR:AT5G50090.1)
AT3G26690	*-0,797*	NUDIX HYDROLASE HOMOLOG 13 (NUDX13)	Encodes AtNUDT13, a mitochondrial Nudix hydrolase specific for long-chain diadenosine polyphosphates.
AT3G56950	*-0,798*	SMALL AND BASIC INTRINSIC PROTEIN 2;1 (SIP2;1)	One of the Major Intrinsic Proteins(MIPs) which facilitate the passive transport of small molecules across membranes.Belongs to a family of plant aquaporins.Similar to yeast and radish aquaporins. Located on ER.
AT2G46330	*-0,799*	ARABINOGALACTAN PROTEIN 16 (AGP16)	Encodes arabinogalactan protein (AGP16).
AT4G20140	*-0,8*	GASSHO1 (GSO1)	Encodes GASSHO1 (GSO1), a putative leucine-rich repeat transmembrane-type receptor kinase. GSO1 and a homolog GSO2 (At5g44700) are required for the formation of a normal epidermal surface during embryogenesis. Necessary for localizing CASPARIAN STRIP DOMAIN PROTEINS (CASPs) - major players of endodermal differentiation - into an uninterrupted, ring-like domain.
AT4G34750	*-0,8*	SMALL AUXIN UPREGULATED RNA 49 (SAUR49)	SAUR-like auxin-responsive protein family ; CONTAINS InterPro DOMAIN/s: Auxin responsive SAUR protein (InterPro:IPR003676); BEST Arabidopsis thaliana protein match is: SAUR-like auxin-responsive protein family (TAIR:AT5G10990.1)

AT2G40900	*-0,801*	USUALLY MULTIPLE ACIDS MOVE IN AND OUT TRANSPORTERS 11 (UMAMIT11)	Encodes a plasma membrane-localized amino acid transporter likely involved in amino acid export in the developing seed.
AT1G10030	*-0,802*	HOMOLOG OF YEAST ERGOSTEROL28 (ERG28)	homolog of yeast ergosterol28 (ERG28); INVOLVED IN: biological_process unknown; LOCATED IN: endomembrane system, integral to membrane; EXPRESSED IN: 22 plant structures; EXPRESSED DURING: 13 growth stages; CONTAINS InterPro DOMAIN/s: Erg28-like (InterPro:IPR005352)
AT5G58780	*-0,802*	CIS -PRENYLTRANSFERASE 5 (CPT5)	Encodes a novel Z,E-mixed heptaprenyl diphosphate (Z,E-HepPP) synthase, which may be responsible for short-chain betulaprenols. It catalyzes the formation of C 35 short-chain polyisoprenoids in which the optimal allylic substrate was E,E-FPP. It may have a role in response to cold stress in root.
AT1G10560	*-0,803*	PLANT U-BOX 18 (PUB18)	Encodes a protein containing a UND, a U-box, and an ARM domain. This protein has E3 ubiquitin ligase activity based on in vitro assays.
AT2G22970	*-0,804*	SERINE CARBOXYPEPTIDASE-LIKE 11 (SCPL11)	serine carboxypeptidase-like 11 (SCPL11); FUNCTIONS IN: serine-type carboxypeptidase activity; INVOLVED IN: proteolysis; LOCATED IN: plant-type cell wall; EXPRESSED IN: 22 plant structures; EXPRESSED DURING: 13 growth stages; CONTAINS InterPro DOMAIN/s: Peptidase S10, serine carboxypeptidase (InterPro:IPR001563); BEST Arabidopsis thaliana protein match is: serine carboxypeptidase-like 12 (TAIR:AT2G22920.2)
AT4G23410	*-0,804*	TETRASPANIN5 (TET5)	TET5 encodes a member of the TETRASPANIN gene family that is expressed in the embryo and vascular system and is involved in organ growth redundantly with TET6.
AT4G28240	*-0,804*		Wound-responsive family protein; CONTAINS InterPro DOMAIN/s: Protein of unknown function wound-induced (InterPro:IPR022251)
AT1G26680	*-0,805*		transcriptional factor B3 family protein; FUNCTIONS IN: DNA binding, sequence-specific DNA binding transcription factor activity; INVOLVED IN: regulation of transcription, DNA-dependent; LOCATED IN: chloroplast; EXPRESSED IN: ovule, embryo; EXPRESSED DURING: D bilateral stage; CONTAINS InterPro DOMAIN/s: Transcriptional factor B3 (InterPro:IPR003340); BEST Arabidopsis thaliana protein match is: Transcriptional factor B3 family protein (TAIR:AT2G24645.1)
AT2G30250	*-0,805*	WRKY DNA-BINDING PROTEIN 25 (WRKY25)	member of WRKY Transcription Factor; Group I. Located in nucleus. Involved in response to various abiotic stresses - especially salt stress.
AT5G28050	*-0,805*	GUANOSINE DEAMINASE (GSDA)	Cytidine/deoxycytidylate deaminase family protein; FUNCTIONS IN: hydrolase activity, zinc ion binding, catalytic activity; LOCATED IN: cellular_component unknown; EXPRESSED IN: 24 plant structures; EXPRESSED DURING: 15 growth stages; CONTAINS InterPro DOMAIN/s: APOBEC/CMP deaminase, zinc-binding (InterPro:IPR016192), CMP/dCMP deaminase, zinc-binding (InterPro:IPR002125), Cytidine deaminase-like (InterPro:IPR016193); BEST Arabidopsis thaliana protein match is: Cytidine/deoxycytidylate deaminase family protein (TAIR:AT3G05300.1)
AT1G19910	*-0,806*	(AVA-P2)	vacuolar H+-pumping ATPase 16 kDa proteolipid (ava-p2)
AT1G80460	*-0,806*	NONHOST RESISTANCE TO P. S. PHASEOLICOLA 1 (NHO1)	Encodes a protein similar to glycerol kinase, which converts glycerol to glycerol 3-phosphate and performs a rate-limiting step in glycerol metabolism. This gene is required for both general and specific resistance against bacteria and fungi. Arabidopsis thaliana glycerol kinase (GLR1) mRNA.Involved in flagellin-induced non-host resistance to Pseudomonas. Coronatine partially suppresses flagellin-induced expression of NHO1.
AT2G17450	*-0,806*	RING-H2 FINGER A3A (RHA3A)	Encodes a putative RING-H2 finger protein RHA3a.
AT2G39890	*-0,806*	PROLINE TRANSPORTER 1 (PROT1)	Encodes a proline transporter with affinity for gly betaine, proline and GABA. Protein is expressed in the vascular tissue, specifically the phloem.
AT3G28860	*-0,806*	ATP-BINDING CASSETTE B19 (ABCB19)	Encodes a member of the ATP-binding cassette (ABC) transporter family that is involved in auxin transport and is involved in postembryonic organ separation. Also known as AtMDR11 and PGP19. Possibly regulates auxin-dependent responses by influencing basipetal auxin transport in the root. Acts upstream of phyA in regulating hypocotyl elongation and gravitropic response. Exerts nonredundant, partially overlapping functions with the ABC transporter encoded by AtPGP1.
AT2G38700	*-0,807*	MEVALONATE DIPHOSPHATE DECARBOXYLASE 1 (MVD1)	Encodes mevalonate diphosphate decarboxylase, the enzyme that catalyzes the synthesis of isopentenyl diphosphate, used in sterol and isoprenoid biosynthesis. The protein appears to form a homodimeric complex. Incidentally, it was shown that the Arabidopsis MVD protein could also interact with its yeast homolog to form a heterodimer.

AT1G14200	-0,809		RING/U-box superfamily protein; FUNCTIONS IN: zinc ion binding; EXPRESSED IN: 20 plant structures; EXPRESSED DURING: 12 growth stages; CONTAINS InterPro DOMAIN/s: Zinc finger, RING-type (InterPro:IPR001841), Zinc finger, C3HC4 RING-type (InterPro:IPR018957); BEST Arabidopsis thaliana protein match is: RING/U-box superfamily protein (TAIR:AT1G26800.1)
AT4G27710	-0,809	CYTOCHROME P450, FAMILY 709, SUBFAMILY B, POLYPEPTIDE 3 (CYP709B3)	member of CYP709B The mRNA is cell-to-cell mobile.
AT5G10740	-0,809		Protein phosphatase 2C family protein; FUNCTIONS IN: protein serine/threonine phosphatase activity, catalytic activity; INVOLVED IN: protein amino acid dephosphorylation; LOCATED IN: protein serine/threonine phosphatase complex; EXPRESSED IN: 19 plant structures; EXPRESSED DURING: 11 growth stages; CONTAINS InterPro DOMAIN/s: Protein phosphatase 2C, manganese/magnesium aspartate binding site (InterPro:IPR000222), Protein phosphatase 2C-related (InterPro:IPR001932), Protein phosphatase 2C (InterPro:IPR015655), Protein phosphatase 2C, N-terminal (InterPro:IPR014045); BEST Arabidopsis thaliana protein match is: Protein phosphatase 2C family protein (TAIR:AT5G24940.1)
AT1G56020	-0,81		unknown protein; BEST Arabidopsis thaliana protein match is: unknown protein (TAIR:AT3G12970.1)
AT4G01850	-0,81	S-ADENOSYLMETHIONINE SYNTHETASE 2 (SAM-2)	S-adenosylmethionine synthetase 2 (SAM-2); FUNCTIONS IN: copper ion binding, methionine adenosyltransferase activity; INVOLVED IN: one-carbon metabolic process, S-adenosylmethionine biosynthetic process; LOCATED IN: nucleolus, cell wall; EXPRESSED IN: 27 plant structures; EXPRESSED DURING: 17 growth stages; CONTAINS InterPro DOMAIN/s: S-adenosylmethionine synthetase (InterPro:IPR002133), S-adenosylmethionine synthetase superfamily (InterPro:IPR022636), S-adenosylmethionine synthetase, N-terminal (InterPro:IPR022628), S-adenosylmethionine synthetase, C-terminal (InterPro:IPR022630), S-adenosylmethionine synthetase, conserved site (InterPro:IPR022631), S-adenosylmethionine synthetase, central domain (InterPro:IPR022629); BEST Arabidopsis thaliana protein match is: S-adenosylmethionine synthetase 1 (TAIR:AT1G02500.2)
AT4G34860	-0,81	ALKALINE/NEUTRAL INVERTASE B (A/N-InvB)	Plant neutral invertase family protein; FUNCTIONS IN: catalytic activity, beta-fructofuranosidase activity; INVOLVED IN: sucrose catabolic process, using beta-fructofuranosidase; LOCATED IN: cytosol; EXPRESSED IN: 20 plant structures; EXPRESSED DURING: 11 growth stages; CONTAINS InterPro DOMAIN/s: Plant neutral invertase (InterPro:IPR006937), Six-hairpin glycosidase-like (InterPro:IPR008928); BEST Arabidopsis thaliana protein match is: cytosolic invertase 2 (TAIR:AT4G09510.1)
AT4G16520	-0,811	AUTOPHAGY 8F (ATG8F)	autophagy 8f (ATG8F); CONTAINS InterPro DOMAIN/s: Light chain 3 (LC3) (InterPro:IPR004241); BEST Arabidopsis thaliana protein match is: Ubiquitin-like superfamily protein (TAIR:AT3G60640.1)
AT5G19930	-0,811	PLASMA MEMBRANE GLUCOSE-RESPONSIVE REGULATOR (PGR)	PGR is putative plasma membrane glucose- responsive regulator that is expressed in response to glucose stimulation.RNAi knockdown mutant seeds have enhanced sensitivity to glucose and 2-deoxyglucose.
AT5G48110	-0,811	TERPENE SYNTHASE 20	The Col variant has no enzyme activity due to various substitution and deletion mutations.
AT5G51910	-0,812	TCP DOMAIN PROTEIN 19 (TCP19)	TCP family transcription factor ; CONTAINS InterPro DOMAIN/s: Transcription factor, TCP (InterPro:IPR005333), Transcription factor TCP subgroup (InterPro:IPR017887); BEST Arabidopsis thaliana protein match is: TCP family transcription factor (TAIR:AT2G45680.1)
AT1G43790	-0,813	TRACHEARY ELEMENT DIFFERENTIATION-RELATED 6 (TED6)	tracheary element differentiation-related 6 (TED6); BEST Arabidopsis thaliana protein match is: tracheary element differentiation-related 7 (TAIR:AT5G48920.1)
AT4G24150	-0,813	GROWTH-REGULATING FACTOR 8 (GRF8)	Growth regulating factor encoding transcription activator. One of the nine members of a GRF gene family, containing nuclear targeting domain. Involved in leaf development and expressed in shoot and flower.
AT5G62560	-0,813		RING/U-box superfamily protein with ARM repeat domain; FUNCTIONS IN: ubiquitin-protein ligase activity, binding; INVOLVED IN: protein ubiquitination; LOCATED IN: ubiquitin ligase complex; CONTAINS InterPro DOMAIN/s: U box domain (InterPro:IPR003613), Armadillo-like helical (InterPro:IPR011989), Armadillo (InterPro:IPR000225), Armadillo-type fold (InterPro:IPR016024); BEST Arabidopsis thaliana protein match is: PLANT U-BOX 39 (TAIR:AT3G47820.1)

AT1G56700	-0,814		Peptidase C15, pyroglutamyl peptidase I-like; INVOLVED IN: proteolysis; LOCATED IN: cellular_component unknown; EXPRESSED IN: 24 plant structures; EXPRESSED DURING: 13 growth stages; CONTAINS InterPro DOMAIN/s: Peptidase C15, pyroglutamyl peptidase I (InterPro:IPR000816), Peptidase C15, pyroglutamyl peptidase I-like (InterPro:IPR016125); BEST Arabidopsis thaliana protein match is: Peptidase C15, pyroglutamyl peptidase I-like (TAIR:AT1G23440.1)
AT1G79330	-0,814	METACASPASE 5 (MC5)	Metacaspase AtMCPb2/AMC6. Caspase family protein. Arginine/lysine-specific cysteine protease activity. Induces apoptosis in yeast. Contains Pfam domain, PF00656: ICE-like protease (caspase) p20 domain. Arabidopsis contains three type I MCP genes (MCP1a-c) and six type II MCP genes (MCP2a?f): AtMCP1a/At5g64240, AtMCP1b/At1g02170, AtMCP1c/At4g25110, AtMCP2a/At1g79310, AtMCP2b/At1g79330, AtMCP2c/At1g79320, AtMCP2d/At1g79340, AtMCP2e/At1g16420, AtMCP2f/At5g04200.
AT1G49320	-0,815	UNKNOWN SEED PROTEIN LIKE 1 (USPL1)	Encodes USPL1, a BURP domain protein targeted to the protein storage vacuoles. Overexpression of USPL1 affects seed development, protein storage vacuoles and lipid vesicles morphology and function.
AT4G23750	-0,815	CYTOKININ RESPONSE FACTOR 2 (CRF2)	encodes a member of the ERF (ethylene response factor) subfamily B-5 of ERF/AP2 transcription factor family. The protein contains one AP2 domain. There are 7 members in this subfamily. Monopteros target gene.
AT1G51140	-0,816	FLOWERING BHLH 3 (FBH3)	Encodes a basic helix-loop-helix-type transcription factor involved in photoperiodism flowering. Binds to the E-box cis-element in the CONSTANS (CO) promoter to regulate flowering. Interacts with CFL1 and along with CFLAP2 negatively regulates cuticle development. Binds to the potassium channel gene KAT1 as a dimer. The DNA-binding capacity is inhibited in response to ABA through phosphorylation-dependent monomerization.
AT3G01690	-0,816		alpha/beta-Hydrolases superfamily protein; CONTAINS InterPro DOMAIN/s: Alpha/beta hydrolase fold-1 (InterPro:IPR000073); BEST Arabidopsis thaliana protein match is: alpha/beta-Hydrolases superfamily protein (TAIR:AT5G14390.1)
AT5G05890	-0,816	UDP-GLUCOSYL TRANSFERASE 76C5 (UGT76C5)	Encodes a nicotinate-N-glycosyltransferase.
AT5G66880	-0,817	SUCROSE NONFERMENTING 1(SNF1)-RELATED PROTEIN KINASE 2.3 (SNRK2.3)	encodes a member of SNF1-related protein kinases (SnRK2) whose activity is activated by ionic (salt) and non-ionic (mannitol) osmotic stress. Enzyme involved in the ABA signaling during seed germination, dormancy and seedling growth. The mRNA is cell-to-cell mobile.
AT1G18870	-0,818	ISOCHORISMATE SYNTHASE 2 (ICS2)	Encodes a protein with isochorismate synthase activity involved in phylloquinone biosynthesis. Mutant studies of this gene's function suggest that its function is redundant with that of ICS1 (AT1G7410).
AT1G34370	-0,818	SENSITIVE TO PROTON RHIZOTOXICITY 1 (STOP1)	Encodes a putative nuclear Cys(2)His(2)-type zinc finger protein involved in H+ and Al3+ rhizotoxicity. In mutants exposed to aluminum stress, there is no induction of AtALMT1, an malate transporter known to be involved in the mediation of aluminum toxicity. Cell wall of the mutant is unstable in low pH medium (pH 4.5) in low Ca solution. This would mediate Ca-alleviation of low pH stress through pectin-Ca interaction. In vitro binding and mutated-promoter-GUS assays identified that STOP1 directly activates AtALMT1 expression through the binding to the promoter by four zinc finger domains. Binding of STOP1 to promoter is an essential step of Al-inducible AtALMT1 expression. The mRNA is cell-to-cell mobile.
AT3G06330	-0,819		RING/U-box superfamily protein; CONTAINS InterPro DOMAIN/s: Zinc finger, C3HC4 RING-type (InterPro:IPR018957), Zinc finger, RING-CH-type (InterPro:IPR011016); BEST Arabidopsis thaliana protein match is: RING/U-box superfamily protein (TAIR:AT5G18760.1)
AT3G11280	-0,819		Putative transcription factors interacting with the gene product of VHA-B1 (vacuolar ATPase subunit B1; as shown through yeast two-hybrid assay).
AT4G40080	-0,821		ENTH/ANTH/VHS superfamily protein; FUNCTIONS IN: phospholipid binding, binding; INVOLVED IN: biological_process unknown; LOCATED IN: chloroplast; EXPRESSED IN: 9 plant structures; EXPRESSED DURING: 6 growth stages; CONTAINS InterPro DOMAIN/s: Epsin-like, N-terminal (InterPro:IPR013809), ANTH (InterPro:IPR011417), ENTH/VHS (InterPro:IPR008942); BEST Arabidopsis thaliana protein match is: ENTH/ANTH/VHS superfamily protein (TAIR:AT5G10410.1)
AT1G76760	-0,822	THIOREDOXIN Y1 (TY1)	Encodes a y-type thioredoxin (Trx-y1) localized in chloroplast stroma.

AT4G38470	-0,822	SERINE/THREONINE/TYROSINE KINASE 46 (STY46)	ACT-like protein tyrosine kinase family protein; FUNCTIONS IN: protein serine/threonine/tyrosine kinase activity, protein kinase activity; INVOLVED IN: protein amino acid phosphorylation, metabolic process; LOCATED IN: cytosol; EXPRESSED IN: 25 plant structures; EXPRESSED DURING: 15 growth stages; CONTAINS InterPro DOMAIN/s: Serine/threonine-protein kinase domain (InterPro:IPR002290), Amino acid-binding ACT (InterPro:IPR002912), Serine-threonine/tyrosine-protein kinase (InterPro:IPR001245), Serine/threonine protein kinase-like, ATMRK (InterPro:IPR015783), Protein kinase-like domain (InterPro:IPR011009), Serine/threonine-protein kinase, active site (InterPro:IPR008271), Protein kinase, catalytic domain (InterPro:IPR000719), Tyrosine-protein kinase, catalytic domain (InterPro:IPR020635); BEST Arabidopsis thaliana protein match is: ACT-like protein tyrosine kinase family protein (TAIR:AT4G35780.1)
AT1G77000	-0,823	(SKP2B)	AtSKP2;2 is a homolog of human SKP2, the human F-box protein that recruits E2F1. Contains an F-box motif at the N-terminal region and a C-terminal Leu-rich repeat domain. Forms part of an E3-ubiquitin-ligase SCF (Skp1, cullin, F-box) complex and recruits phosphorylated AtE2Fc, a transcriptional factor that might play a role in cell division and during the transition from skotomorphogenesis to photomorphogenesis. AtSKP2;1 (At1g21410) and AtSKP2;2 (At1g77000) may be duplicated genes. AtSKP2b may also be involved in the degradation of KRP1/ICK1, a CDK inhibitor.
AT3G04730	-0,823	INDOLEACETIC ACID-INDUCED PROTEIN 16 (IAA16)	early auxin-induced (IAA16)
AT4G08690	-0,823		Sec14p-like phosphatidylinositol transfer family protein; CONTAINS InterPro DOMAIN/s: Cellular retinaldehyde-binding/triple function, C-terminal (InterPro:IPR001251), Cellular retinaldehyde-binding/triple function, N-terminal (InterPro:IPR008273), Phosphatidylinositol transfer protein-like, N-terminal (InterPro:IPR011074); BEST Arabidopsis thaliana protein match is: Sec14p-like phosphatidylinositol transfer family protein (TAIR:AT1G22180.2)
AT4G30190	-0,823	H(+)-ATPASE 2 (HA2)	belongs to the P-type ATPase superfamily of cation-transporting ATPases, pumps protons out of the cell, generating a proton gradient that drives the active transport of nutrients by proton symport. has two autoinhibitory regions within the C-terminal dom
AT5G37600	-0,824	GLUTAMINE SYNTHASE CLONE R1 (GSR 1)	encodes a cytosolic glutamine synthetase, the enzyme has high affinity with substrate ammonium
AT1G68450	-0,826	PIGMENT DEFECTIVE 337 (PDE337)	VQ motif-containing protein; FUNCTIONS IN: molecular_function unknown; INVOLVED IN: biological_process unknown; LOCATED IN: chloroplast; EXPRESSED IN: stem; CONTAINS InterPro DOMAIN/s: VQ (InterPro:IPR008889); BEST Arabidopsis thaliana protein match is: VQ motif-containing protein (TAIR:AT3G18360.1)
AT1G72020	-0,827		unknown protein
AT4G29020	-0,827		glycine-rich protein; FUNCTIONS IN: molecular_function unknown; INVOLVED IN: biological_process unknown; LOCATED IN: endomembrane system; EXPRESSED IN: 22 plant structures; EXPRESSED DURING: 13 growth stages
AT1G44100	-0,829	AMINO ACID PERMEASE 5 (AAP5)	amino acid permease 5
AT5G52990	-0,829		SNARE-like superfamily protein; FUNCTIONS IN: molecular_function unknown; INVOLVED IN: transport; LOCATED IN: endomembrane system; CONTAINS InterPro DOMAIN/s: Longin-like (InterPro:IPR011012); BEST Arabidopsis thaliana protein match is: SNARE-like superfamily protein (TAIR:AT4G27840.1)
AT5G42880	-0,83		Plant protein of unknown function (DUF827); CONTAINS InterPro DOMAIN/s: Protein of unknown function DUF827, plant (InterPro:IPR008545); BEST Arabidopsis thaliana protein match is: Plant protein of unknown function (DUF827) (TAIR:AT1G45545.1)
AT3G07130	-0,831	PURPLE ACID PHOSPHATASE 15 (PAP15)	Encodes PAP15, a purple acid phosphatase with phytase activity. Expression of PAP15 is developmentally and temporally regulated, with strong expression at the early stages of seedling growth and pollen germination. The expression is also organ/tissue-specific, with strongest expression in the vasculature, pollen grains, and roots. Recombinant PAP protein exhibits broad substrate specificity with moderate phytase activity. PAP15 likely mobilizes phosphorus reserves in plants, particularly during seed and pollen germination.

AT4G30660	-0,831		Low temperature and salt responsive protein family; INVOLVED IN: response to cold, hyperosmotic salinity response; LOCATED IN: endomembrane system, integral to membrane; EXPRESSED IN: 22 plant structures; EXPRESSED DURING: 13 growth stages; CONTAINS InterPro DOMAIN/s: Uncharacterised protein family UPF0057 (InterPro:IPR000612); BEST Arabidopsis thaliana protein match is: Low temperature and salt responsive protein family (TAIR:AT2G24040.1)
AT3G57860	-0,832	UV-B-INSENSITIVE 4-LIKE (UVI4-LIKE)	Encodes a protein that controls entry into the second meiotic division. Negatively regulates the Anaphase-Promoting Complex/Cyclosome.
AT5G40090	-0,834	CHS1-LIKE 1 (CHL1)	Disease resistance protein (TIR-NBS class); FUNCTIONS IN: transmembrane receptor activity, nucleoside-triphosphatase activity, nucleotide binding, ATP binding; INVOLVED IN: signal transduction, apoptosis, defense response, innate immune response; LOCATED IN: intrinsic to membrane, endomembrane system; EXPRESSED IN: shoot apex, root, flower; EXPRESSED DURING: petal differentiation and expansion stage; CONTAINS InterPro DOMAIN/s: ATPase, AAA+ type, core (InterPro:IPR003593), NB-ARC (InterPro:IPR002182), Disease resistance protein (InterPro:IPR000767), Toll-Interleukin receptor (InterPro:IPR000157); BEST Arabidopsis thaliana protein match is: Disease resistance protein (TIR-NBS class) (TAIR:AT1G17610.1)
AT2G46660	-0,835	CYTOCHROME P450, FAMILY 78, SUBFAMILY A, POLYPEPTIDE 6 (CYP78A6)	Encodes a member of CYP78A cytochrome P450 monooxygenase protein family that is required in the sporophytic tissue of the mother plant to promote seed growth.
AT1G11260	-0,836	SUGAR TRANSPORTER 1 (STP1)	Encodes a H+/hexose cotransporter. The mRNA is cell-to-cell mobile.
AT2G02080	-0,836	INDETERMINATE(ID)-DOMAIN 4 (IDD4)	C2H2 BIRD transcription factor family.
AT2G24170	-0,836		Endomembrane protein 70 protein family; LOCATED IN: endomembrane system, integral to membrane; EXPRESSED IN: 21 plant structures; EXPRESSED DURING: 13 growth stages; CONTAINS InterPro DOMAIN/s: Nonaspanin (TM9SF) (InterPro:IPR004240); BEST Arabidopsis thaliana protein match is: Endomembrane protein 70 protein family (TAIR:AT5G10840.1)
AT2G43550	-0,836		Encodes a defensin-like (DEFL) family protein.
AT5G24530	-0,837	DOWNY MILDEW RESISTANT 6 (DMR6)	Encodes a putative 2OG-Fe(II) oxygenase that is defense-associated but required for susceptibility to downy mildew. The mRNA is cell-to-cell mobile.
AT1G18970	-0,838	GERMIN-LIKE PROTEIN 4 (GLP4)	Encodes a germin-like protein with possible oxalate oxidase activity (based on GenBank record).
AT1G52750	-0,838		alpha/beta-Hydrolases superfamily protein; CONTAINS InterPro DOMAIN/s: Epoxide hydrolase-like (InterPro:IPR000639), Alpha/beta hydrolase fold-1 (InterPro:IPR000073); BEST Arabidopsis thaliana protein match is: alpha/beta-Hydrolases superfamily protein (TAIR:AT1G80280.1)
AT4G12080	-0,838	AT-HOOK MOTIF NUCLEAR-LOCALIZED PROTEIN 1 (AHL1)	AT-hook motif nuclear-localized protein 1 (AHL1); FUNCTIONS IN: DNA binding; LOCATED IN: mitochondrion, nucleolus, nucleus, cytoplasm; EXPRESSED IN: 9 plant structures; EXPRESSED DURING: LP.04 four leaves visible, 4 anthesis, petal differentiation and expansion stage; CONTAINS InterPro DOMAIN/s: Protein of unknown function DUF296 (InterPro:IPR005175), AT hook, DNA-binding motif (InterPro:IPR017956); BEST Arabidopsis thaliana protein match is: AT hook motif DNA-binding family protein (TAIR:AT4G22770.1)
AT4G28770	-0,839		Tetraspanin family protein; CONTAINS InterPro DOMAIN/s: Tetraspanin (InterPro:IPR018499); BEST Arabidopsis thaliana protein match is: Tetraspanin family protein (TAIR:AT2G20230.1).
AT5G51890	-0,839		encodes peroxidase involved in the lignification of tracheary elements (TE) in roots
AT3G62030	-0,84	ROTAMASE CYP 4 (ROC4)	nuclear-encoded chloroplast stromal cyclophilin CYP20-3 (also known as ROC4). Protein is tyrosine-phosphorylated and its phosphorylation state is modulated in response to ABA in Arabidopsis thaliana seeds.
AT4G20240	-0,84	CYTOCHROME P450, FAMILY 71, SUBFAMILY A, POLYPEPTIDE 27 (CYP71A27)	putative cytochrome P450

AT1G09250	*-0,841*	ATBS1 INTERACTING FACTOR 4 (AIF4)	basic helix-loop-helix (bHLH) DNA-binding superfamily protein; FUNCTIONS IN: sequence-specific DNA binding transcription factor activity; INVOLVED IN: regulation of transcription; LOCATED IN: nucleus, chloroplast; EXPRESSED IN: 22 plant structures; EXPRESSED DURING: 13 growth stages; CONTAINS InterPro DOMAIN/s: Helix-loop-helix DNA-binding domain (InterPro:IPR001092), Helix-loop-helix DNA-binding (InterPro:IPR011598); BEST Arabidopsis thaliana protein match is: sequence-specific DNA binding transcription factors (TAIR:AT3G17100.2)
AT2G37060	*-0,841*	NUCLEAR FACTOR Y, SUBUNIT B8 (NF-YB8)	nuclear factor Y, subunit B8 (NF-YB8); FUNCTIONS IN: sequence-specific DNA binding transcription factor activity; INVOLVED IN: regulation of transcription, DNA-dependent; LOCATED IN: nucleus, intracellular; EXPRESSED IN: male gametophyte, pollen tube; EXPRESSED DURING: M germinated pollen stage; CONTAINS InterPro DOMAIN/s: Transcription factor, CBFA/NFYB, DNA topoisomerase (InterPro:IPR003957), Transcription factor CBF/NF-Y/archaeal histone (InterPro:IPR003958), Histone-fold (InterPro:IPR009072), Transcription factor, NFYB/HAP3, conserved site (InterPro:IPR003956); BEST Arabidopsis thaliana protein match is: nuclear factor Y, subunit B10 (TAIR:AT3G53340.1)
AT2G48130	*-0,841*		Bifunctional inhibitor/lipid-transfer protein/seed storage 2S albumin superfamily protein; FUNCTIONS IN: lipid binding; INVOLVED IN: lipid transport; LOCATED IN: anchored to membrane; EXPRESSED IN: hypocotyl, root, flower; EXPRESSED DURING: petal differentiation and expansion stage; CONTAINS InterPro DOMAIN/s: Bifunctional inhibitor/plant lipid transfer protein/seed storage (InterPro:IPR016140), Plant lipid transfer protein/seed storage/trypsin-alpha amylase inhibitor (InterPro:IPR003612), Plant lipid transfer protein/Par allergen (InterPro:IPR000528), Plant lipid transfer protein/hydrophobic protein, helical domain (InterPro:IPR013770); BEST Arabidopsis thaliana protein match is: Bifunctional inhibitor/lipid-transfer protein/seed storage 2S albumin superfamily protein (TAIR:AT3G22600.1)
AT1G48510	*-0,843*		Surfeit locus 1 cytochrome c oxidase biogenesis protein; CONTAINS InterPro DOMAIN/s: Surfeit locus 1 (InterPro:IPR002994); BEST Arabidopsis thaliana protein match is: Surfeit locus 1 cytochrome c oxidase biogenesis protein (TAIR:AT3G17910.1)
AT1G02900	*-0,844*	RAPID ALKALINIZATION FACTOR 1 (RALF1)	Member of a diversely expressed predicted peptide family showing sequence similarity to tobacco Rapid Alkalinization Factor (RALF), and is believed to play an essential role in the physiology of Arabidopsis. Consists of a single exon and is characterized by a conserved C-terminal motif and N-terminal signal peptide. Mediates Ca2+-dependent signaling.
AT1G47760	*-0,844*	AGAMOUS-LIKE 102 (AGL102)	AGAMOUS-like 102 (AGL102); FUNCTIONS IN: DNA binding, sequence-specific DNA binding transcription factor activity; INVOLVED IN: regulation of transcription, DNA-dependent; LOCATED IN: nucleus; EXPRESSED IN: endosperm; CONTAINS InterPro DOMAIN/s: Transcription factor, MADS-box (InterPro:IPR002100); BEST Arabidopsis thaliana protein match is: AGAMOUS-like 91 (TAIR:AT3G66656.1)
AT4G02370	*-0,844*		Protein of unknown function, DUF538; INVOLVED IN: biological_process unknown; LOCATED IN: plasma membrane, vacuole; EXPRESSED IN: 25 plant structures; EXPRESSED DURING: 13 growth stages; CONTAINS InterPro DOMAIN/s: Protein of unknown function DUF538 (InterPro:IPR007493); BEST Arabidopsis thaliana protein match is: Protein of unknown function, DUF538 (TAIR:AT1G02816.1)
AT1G64530	*-0,845*	NIN-LIKE PROTEIN 6 (NLP6)	Plant regulator RWP-RK family protein; CONTAINS InterPro DOMAIN/s: Octicosapeptide/Phox/Bem1p (InterPro:IPR000270), Plant regulator RWP-RK (InterPro:IPR003035); BEST Arabidopsis thaliana protein match is: NIN like protein 7 (TAIR:AT4G24020.1)
AT3G47780	*-0,845*	ATP-BINDING CASSETTE A7 (ABCA7)	member of ATH subfamily The mRNA is cell-to-cell mobile.
AT1G19600	*-0,846*		pfkB-like carbohydrate kinase family protein; FUNCTIONS IN: kinase activity; INVOLVED IN: D-ribose catabolic process; LOCATED IN: cellular_component unknown; EXPRESSED IN: 21 plant structures; EXPRESSED DURING: 13 growth stages; CONTAINS InterPro DOMAIN/s: Carbohydrate/purine kinase (InterPro:IPR011611); BEST Arabidopsis thaliana protein match is: pfkB-like carbohydrate kinase family protein (TAIR:AT4G27600.1)
AT1G62840	*-0,846*		Protein of unknown function (DUF1442); CONTAINS InterPro DOMAIN/s: Protein of unknown function DUF1442 (InterPro:IPR009902); BEST Arabidopsis thaliana protein match is: Protein of unknown function (DUF1442) (TAIR:AT1G12320.1)
AT1G77990	*-0,846*	SULPHATE TRANSPORTER 2;2 (SULTR2;2)	Encodes a low-affinity sulfate transporter.

AT2G26250	*-0,846*	3-KETOACYL-COA SYNTHASE 10 (KCS10)	epidermis-specific, encodes KCS10, a putative 3-ketoacyl-CoA synthase. probably involved in the synthesis of long-chain lipids found in the cuticle.
AT5G10860	*-0,846*	CBS DOMAIN CONTAINING PROTEIN 3 (CBSX3)	Encodes a single cystathionine beta-Synthase domain-containing protein. Modulates development by regulating the thioredoxin system.
AT1G02880	*-0,847*	THIAMIN PYROPHOSPHOKINASE1 (TPK1)	Encodes a thiamine pyrophosphokinase capable of producing thiamine pyrophosphate from free thiamine.
AT1G28330	*-0,847*	DORMANCY-ASSOCIATED PROTEIN-LIKE 1 (DYL1)	dormancy-associated protein (DRM1)
AT1G68000	*-0,847*	PHOSPHATIDYLINOSITOL SYNTHASE 1 (PIS1)	phosphatidylinositol synthase 1
AT5G64180	*-0,847*		unknown protein
AT2G02850	*-0,848*	PLANTACYANIN (ARPN)	Encodes plantacyanin one of blue copper proteins. Involved in anther development and pollination. Expressed in the transmitting tract of the pistil.
AT1G70420	*-0,85*		Protein of unknown function (DUF1645); CONTAINS InterPro DOMAIN/s: Protein of unknown function DUF1645 (InterPro:IPR012442); BEST Arabidopsis thaliana protein match is: Protein of unknown function (DUF1645) (TAIR:AT1G23710.1)
AT2G15970	*-0,85*	COLD REGULATED 413 PLASMA MEMBRANE 1 (COR413-PM1)	encodes an alpha form of a protein similar to the cold acclimation protein WCOR413 in wheat. Expression is induced by short-term cold-treatment, water deprivation, and abscisic acid treatment. The mRNA is cell-to-cell mobile.
AT5G48545	*-0,85*	HISTIDINE TRIAD NUCLEOTIDE-BINDING 3 (HINT3)	Encodes a protein that has adenylylsulfate sulfohydrolase activity (E.C. 3.6.2.1) in vitro.
AT2G16530	*-0,851*	POLYPRENOL REDUCTASE 2 (PPRD2)	Encodes polyprenol reductase involved in N-gylcosylation. Mutants are defective in pollen development. Knockouts are embryo lethal
AT3G63080	*-0,851*	GLUTATHIONE PEROXIDASE 5 (GPX5)	Encodes glutathione peroxidase.
AT4G34138	*-0,851*	UDP-GLUCOSYL TRANSFERASE 73B1 (UGT73B1)	UDP-glucosyl transferase 73B1 (UGT73B1); FUNCTIONS IN: quercetin 3-O-glucosyltransferase activity, UDP-glycosyltransferase activity, quercetin 7-O-glucosyltransferase activity, abscisic acid glucosyltransferase activity; INVOLVED IN: metabolic process; LOCATED IN: endomembrane system; EXPRESSED IN: 20 plant structures; EXPRESSED DURING: 10 growth stages; CONTAINS InterPro DOMAIN/s: UDP-glucuronosyl/UDP-glucosyltransferase (InterPro:IPR002213); BEST Arabidopsis thaliana protein match is: UDP-glucosyl transferase 73B3 (TAIR:AT4G34131.1)
AT4G19880	*-0,853*		Glutathione S-transferase family protein; FUNCTIONS IN: molecular_function unknown; INVOLVED IN: response to cadmium ion; LOCATED IN: chloroplast; EXPRESSED IN: 23 plant structures; EXPRESSED DURING: 13 growth stages; CONTAINS InterPro DOMAIN/s: Glutathione S-transferase, predicted (InterPro:IPR016639), Glutathione S-transferase, C-terminal-like (InterPro:IPR010987), Glutathione S-transferase/chloride channel, C-terminal (InterPro:IPR017933), Thioredoxin-like fold (InterPro:IPR012336); BEST Arabidopsis thaliana protein match is: Glutathione S-transferase family protein (TAIR:AT5G45020.2)
AT1G17455	*-0,855*	ELF4-LIKE 4 (ELF4-L4)	ELF4-like 4 (ELF4-L4); CONTAINS InterPro DOMAIN/s: Protein of unknown function DUF1313 (InterPro:IPR009741); BEST Arabidopsis thaliana protein match is: ELF4-like 2 (TAIR:AT1G72630.1)
AT2G01540	*-0,855*	C2-DOMAIN ABA-RELATED 10 (CAR10)	Calcium-dependent lipid-binding (CaLB domain) family protein; FUNCTIONS IN: molecular_function unknown; INVOLVED IN: biological_process unknown; LOCATED IN: plasma membrane, vacuole; EXPRESSED IN: 25 plant structures; EXPRESSED DURING: 15 growth stages; CONTAINS InterPro DOMAIN/s: C2 membrane targeting protein (InterPro:IPR018029), C2 calcium/lipid-binding domain, CaLB (InterPro:IPR008973), C2 calcium-dependent membrane targeting (InterPro:IPR000008); BEST Arabidopsis thaliana protein match is: Calcium-dependent lipid-binding (CaLB domain) family protein (TAIR:AT1G70790.1)

AT4G26710	*-0,855*		ATPase, V0 complex, subunit E; FUNCTIONS IN: hydrogen ion transmembrane transporter activity; INVOLVED IN: ATP hydrolysis coupled proton transport; LOCATED IN: vacuole; EXPRESSED IN: 24 plant structures; EXPRESSED DURING: 15 growth stages; CONTAINS InterPro DOMAIN/s: ATPase, V0 complex, subunit E (InterPro:IPR008389); BEST Arabidopsis thaliana protein match is: ATPase, V0 complex, subunit E (TAIR:AT5G55290.2)
AT1G67150	*-0,857*		Plant protein of unknown function (DUF247); CONTAINS InterPro DOMAIN/s: Protein of unknown function DUF247, plant (InterPro:IPR004158); BEST Arabidopsis thaliana protein match is: Plant protein of unknown function (DUF247) (TAIR:AT2G44930.1)
AT5G62180	*-0,857*	CARBOXYESTERASE 20 (CXE20)	carboxyesterase 20 (CXE20); INVOLVED IN: metabolic process; LOCATED IN: cellular_component unknown; EXPRESSED IN: root, flower; EXPRESSED DURING: petal differentiation and expansion stage; CONTAINS InterPro DOMAIN/s: Alpha/beta hydrolase fold-3 (InterPro:IPR013094); BEST Arabidopsis thaliana protein match is: alpha/beta-Hydrolases superfamily protein (TAIR:AT2G45600.1)
AT1G07150	*-0,859*	MITOGEN-ACTIVATED PROTEIN KINASE KINASE KINASE 13 (MAPKKK13)	member of MEKK subfamily
AT1G18910	*-0,859*		zinc ion binding;zinc ion binding; FUNCTIONS IN: zinc ion binding; EXPRESSED IN: 15 plant structures; EXPRESSED DURING: 7 growth stages; CONTAINS InterPro DOMAIN/s: Zinc finger, CTCHY-type (InterPro:IPR017921), Zinc finger, CHY-type (InterPro:IPR008913), Zinc finger, RING-type (InterPro:IPR001841), Haemerythrin/HHE cation-binding motif (InterPro:IPR012312); BEST Arabidopsis thaliana protein match is: zinc ion binding (TAIR:AT1G74770.1)
AT3G06590	*-0,859*	ATBS1 INTERACTING FACTOR 2 (AIF2)	Encodes RITF1, a bHLH transcription factor that regulates the transcription of several genes involved in the detoxification of reactive oxygen species generated by salt stress.
AT3G54870	*-0,859*	MORPHOGENESIS OF ROOT HAIR 2 (MRH2)	Armadillo-repeat containing kinesin-related protein. Plays a role during transition to root-hair tip growth.Mutants have short, branched root hairs and an excess of endoplasmic microtubles. Phenotype suggests ARK1 plays a role in modulating microtubule depolymerization during root hair tip growth.
AT4G26540	*-0,859*	(RGFR3)	Leucine-rich repeat receptor-like protein kinase family protein; FUNCTIONS IN: kinase activity; INVOLVED IN: protein amino acid phosphorylation; LOCATED IN: plasma membrane; EXPRESSED IN: inflorescence meristem; CONTAINS InterPro DOMAIN/s: Protein kinase, ATP binding site (InterPro:IPR017441), Protein kinase, catalytic domain (InterPro:IPR000719), Leucine-rich repeat-containing N-terminal domain, type 2 (InterPro:IPR013210), Leucine-rich repeat (InterPro:IPR001611), Serine/threonine-protein kinase-like domain (InterPro:IPR017442), Protein kinase-like domain (InterPro:IPR011009), Serine/threonine-protein kinase, active site (InterPro:IPR008271); BEST Arabidopsis thaliana protein match is: Leucine-rich receptor-like protein kinase family protein (TAIR:AT5G56040.2)
AT5G46050	*-0,859*	NRT1/ PTR FAMILY 5.2 (NPF5.2)	Encodes a di- and tri-peptide transporter involved in responses to wounding, virulent bacterial pathogens, and high NaCl concentrations. The protein is predicted to have 12 transmembrane helicies.
AT5G64240	*-0,859*	METACASPASE 3 (MC3)	Encodes a type I metacaspase. Two Arabidopsis metacaspases, AT1G02170 (MC1) and AT4G25110 (MC2) antagonistically control programmed cell death in Arabidopsis. MC1 is a positive regulator of cell death and requires conserved caspase-like putative catalytic residues for its function. MC2 negatively regulates cell death. This function is independent of the putative catalytic residues. A third type I Arabidopsis metacaspase is MC3 (AT5g64240).
AT4G33960	*-0,86*		unknown protein; FUNCTIONS IN: molecular_function unknown; INVOLVED IN: biological_process unknown; LOCATED IN: endomembrane system; EXPRESSED IN: 20 plant structures; EXPRESSED DURING: 10 growth stages; BEST Arabidopsis thaliana protein match is: unknown protein (TAIR:AT2G15830.1)
AT3G54650	*-0,861*	F BOX-LIKE17 (FBL17)	FBL17; FUNCTIONS IN: ubiquitin-protein ligase activity; INVOLVED IN: generative cell mitosis, seed development, embryo development, ubiquitin-dependent protein catabolic process, pollen development; LOCATED IN: chloroplast; EXPRESSED IN: 21 plant structures; EXPRESSED DURING: 12 growth stages; CONTAINS InterPro DOMAIN/s: F-box domain, cyclin-like (InterPro:IPR001810)

AT4G28650	*-0,861*	PXY-LIKE2 (PXL2)	Encodes one of the two putative eLRR kinase closely related to PXY (At1g08590/PXL1 and At4g28650/PXL2). Insertion mutants in either pxl1 or pxl2 do not exhibit an obvious phenotype in the stem; double-mutant combinations of a Col allele, of pxy (pxy-3) with pxl1 and pxl2, generate a more severe vascular phenotype than pxy-3 alone, suggesting that these genes act synergistically with PXY in regulating vascular-tissue development in the stem.
AT1G07620	*-0,862*	(ATOBGM)	ATOBGM; FUNCTIONS IN: magnesium ion binding, GTP binding, GTPase activity; INVOLVED IN: biological_process unknown; LOCATED IN: cellular_component unknown; CONTAINS InterPro DOMAIN/s: GTP-binding protein Obg/CgtA (InterPro:IPR014100); BEST Arabidopsis thaliana protein match is: Protein of unknown function (DUF3741) (TAIR:AT5G02390.1)
AT1G29640	*-0,862*		Protein of unknown function, DUF584; CONTAINS InterPro DOMAIN/s: Protein of unknown function DUF584 (InterPro:IPR007608); BEST Arabidopsis thaliana protein match is: Protein of unknown function, DUF584 (TAIR:AT2G34340.1)
AT2G47890	*-0,862*		B-box type zinc finger protein with CCT domain; FUNCTIONS IN: sequence-specific DNA binding transcription factor activity, zinc ion binding; INVOLVED IN: regulation of transcription; LOCATED IN: intracellular; EXPRESSED IN: stem, inflorescence meristem, root, flower, stamen; EXPRESSED DURING: 4 anthesis, petal differentiation and expansion stage; CONTAINS InterPro DOMAIN/s: CCT domain (InterPro:IPR010402), Zinc finger, B-box (InterPro:IPR000315); BEST Arabidopsis thaliana protein match is: B-box type zinc finger protein with CCT domain (TAIR:AT1G28050.1)
AT5G67430	*-0,862*		Acyl-CoA N-acyltransferases (NAT) superfamily protein; FUNCTIONS IN: N-acetyltransferase activity; INVOLVED IN: metabolic process; LOCATED IN: cellular_component unknown; CONTAINS InterPro DOMAIN/s: GCN5-related N-acetyltransferase, C-terminal (InterPro:IPR022610), GCN5-related N-acetyltransferase (InterPro:IPR000182), Acyl-CoA N-acyltransferase (InterPro:IPR016181); BEST Arabidopsis thaliana protein match is: Acyl-CoA N-acyltransferases (NAT) superfamily protein (TAIR:AT4G37580.1)
AT5G25210	*-0,863*		unknown protein; BEST Arabidopsis thaliana protein match is: unknown protein (TAIR:AT4G32030.1)
AT2G02020	*-0,864*	NRT1/ PTR FAMILY 8.4 (NPF8.4)	Major facilitator superfamily protein; FUNCTIONS IN: transporter activity; INVOLVED IN: oligopeptide transport; LOCATED IN: membrane; EXPRESSED IN: 6 plant structures; EXPRESSED DURING: LP.06 six leaves visible; CONTAINS InterPro DOMAIN/s: PTR2 family proton/oligopeptide symporter, conserved site (InterPro:IPR018456), Oligopeptide transporter (InterPro:IPR000109), Major facilitator superfamily, general substrate transporter (InterPro:IPR016196); BEST Arabidopsis thaliana protein match is: peptide transporter 2 (TAIR:AT2G02040.1)
AT3G20470	*-0,864*	GLYCINE-RICH PROTEIN 5 (GRP5)	encodes a glycine-rich protein that is expressed more abundantly in immature seed pods than in stems and leaves. Expression is not detected in roots or flowers.
AT5G05460	*-0,868*	ENDO-BETA-N-ACETYGLUCOSAMINIDASE 85A (ENGase85A)	Encodes a cytosolic beta-endo-N-acetyglucosaminidase (ENGase). ENGases N-glycans cleave the O-glycosidic linkage between the two GlcNAc residues of the N-glycan core structure and thus generate a protein with a single GlcNAc attached to asparagine.
AT5G61440	*-0,868*	ATYPICAL CYS HIS RICH THIOREDOXIN 5 (ACHT5)	Encodes a member of the thioredoxin family protein. Located in the chloroplast. The mRNA is cell-to-cell mobile.
AT5G02550	*-0,869*		unknown protein
AT1G79760	*-0,87*	DOWNSTREAM TARGET OF AGL15-4 (DTA4)	Identified as target of the AGL15 binding motif CArG.
AT1G28070	*-0,871*		unknown protein; BEST Arabidopsis thaliana protein match is: unknown protein (TAIR:AT2G33510.1)
AT3G02610	*-0,872*	ACYL-ACYL CARRIER PROTEIN DESATURASE2 (AAD2)	Encodes one of two ∆9 palmitoyl-ACP desaturases responsible for the biosynthesis of ω-7 fatty acids in the maturing endosperm.
AT3G16420	*-0,872*	PYK10-BINDING PROTEIN 1 (PBP1)	The PBP1(PYK10-binding protein 1) assists the PYK10 (beta-glucosidase complex) in its activity and may act like a molecular chaperone that facilitates the correct polymerization of PYK10, when tissues are damaged and subcellular structures are destroyed by pests. The mRNA is cell-to-cell mobile.
AT1G14080	*-0,873*	FUCOSYLTRANSFERASE 6 (FUT6)	Encodes an alpha-(1,2)-fucosyltransferase.

AT1G60140	*-0,873*	TREHALOSE PHOSPHATE SYNTHASE (TPS10)	Encodes an enzyme putatively involved in trehalose biosynthesis. The protein has a trehalose synthase (TPS)-like domain that may or may not be active as well as a trehalose phosphatase (TPP)-like domain.
AT4G23700	*-0,873*	CATION/H+ EXCHANGER 17 (CHX17)	member of Putative Na+/H+ antiporter family
AT5G18130	*-0,873*		unknown protein; BEST Arabidopsis thaliana protein match is: unknown protein (TAIR:AT3G03870.2)
AT1G27180	*-0,874*		disease resistance protein (TIR-NBS-LRR class), putative; FUNCTIONS IN: transmembrane receptor activity, ATP binding; INVOLVED IN: signal transduction, apoptosis, defense response, innate immune response; LOCATED IN: intrinsic to membrane; CONTAINS InterPro DOMAIN/s: NB-ARC (InterPro:IPR002182), Leucine-rich repeat, typical subtype (InterPro:IPR003591), Disease resistance protein (InterPro:IPR000767), Toll-Interleukin receptor (InterPro:IPR000157); BEST Arabidopsis thaliana protein match is: transmembrane receptors;ATP binding (TAIR:AT1G27170.1)
AT1G16120	*-0,876*	WALL ASSOCIATED KINASE-LIKE 1 (WAKL1)	Encodes a WAK-like receptor-like kinase with a cytoplasmic Ser/Thr protein kinase domain and an extracellular domain with EGF-like repeats.
AT1G56010	*-0,877*	NAC DOMAIN CONTAINING PROTEIN 1 (NAC1)	Encodes a transcription factor involved in shoot apical meristem formation and auxin-mediated lateral root formation. The gene is thought not to be involved in stress responses (NaCl, auxins, ethylene). NAC1 (NAC1)
AT5G06740	*-0,877*	L-TYPE LECTIN RECEPTOR KINASE S.5 (LECRK-S.5)	Concanavalin A-like lectin protein kinase family protein; FUNCTIONS IN: in 6 functions; INVOLVED IN: protein amino acid phosphorylation; LOCATED IN: endomembrane system; EXPRESSED IN: root; CONTAINS InterPro DOMAIN/s: Legume lectin, beta chain (InterPro:IPR001220), Protein kinase, ATP binding site (InterPro:IPR017441), Serine/threonine-protein kinase-like domain (InterPro:IPR017442), Concanavalin A-like lectin/glucanase, subgroup (InterPro:IPR013320), Protein kinase-like domain (InterPro:IPR011009), Serine/threonine-protein kinase, active site (InterPro:IPR008271), Protein kinase, catalytic domain (InterPro:IPR000719), Concanavalin A-like lectin/glucanase (InterPro:IPR008985), Legume lectin, beta chain, Mn/Ca-binding site (InterPro:IPR019825); BEST Arabidopsis thaliana protein match is: Concanavalin A-like lectin protein kinase family protein (TAIR:AT5G55830.1)
AT4G05150	*-0,878*		Octicosapeptide/Phox/Bem1p family protein; FUNCTIONS IN: molecular_function unknown; INVOLVED IN: biological_process unknown; LOCATED IN: cellular_component unknown; EXPRESSED IN: 24 plant structures; EXPRESSED DURING: 13 growth stages; CONTAINS InterPro DOMAIN/s: Octicosapeptide/Phox/Bem1p (InterPro:IPR000270); BEST Arabidopsis thaliana protein match is: Octicosapeptide/Phox/Bem1p family protein (TAIR:AT2G01190.1)
AT5G43020	*-0,878*		Leucine-rich repeat protein kinase family protein; FUNCTIONS IN: protein serine/threonine kinase activity, kinase activity, ATP binding; INVOLVED IN: transmembrane receptor protein tyrosine kinase signaling pathway, protein amino acid phosphorylation; LOCATED IN: endomembrane system; EXPRESSED IN: 21 plant structures; EXPRESSED DURING: 13 growth stages; CONTAINS InterPro DOMAIN/s: Protein kinase, catalytic domain (InterPro:IPR000719), Leucine-rich repeat (InterPro:IPR001611), Serine/threonine-protein kinase-like domain (InterPro:IPR017442), Protein kinase-like domain (InterPro:IPR011009); BEST Arabidopsis thaliana protein match is: Leucine-rich repeat protein kinase family protein (TAIR:AT5G67200.1)
AT1G53580	*-0,879*	GLYOXALASE II 3 (GLY3)	Mononuclear Fe(II)-containing member of the b-lactamase fold superfamily. ETHE1 is homodimeric in solution, exhibits low-level esterase activity, and specifically binds a single Fe(II) atom in the active site.
AT3G03190	*-0,88*	GLUTATHIONE S-TRANSFERASE F11 (GSTF11)	Encodes glutathione transferase belonging to the phi class of GSTs. Naming convention according to Wagner et al. (2002).
AT5G01360	*-0,88*	TRICHOME BIREFRINGENCE-LIKE 3 (TBL3)	Encodes a member of the TBL (TRICHOME BIREFRINGENCE-LIKE) gene family containing a plant-specific DUF231 (domain of unknown function) domain. TBL gene family has 46 members, two of which (TBR/AT5G06700 and TBL3/AT5G01360) have been shown to be involved in the synthesis and deposition of secondary wall cellulose, presumably by influencing the esterification state of pectic polymers. A nomenclature for this gene family has been proposed (Volker Bischoff & Wolf Scheible, 2010, personal communication).The dwarf phenotype can only be seen in tbl3 tbl31 esk1 triple mutant. tbl3 and tbl31 are specifically involved in 3-O-monoacetylation of xylan.
AT2G04800	*-0,881*		unknown protein; FUNCTIONS IN: molecular_function unknown; INVOLVED IN: N-terminal protein myristoylation; LOCATED IN: cellular_component unknown; EXPRESSED IN: hypocotyl, root

AT2G39110	-0,881		Protein kinase superfamily protein; FUNCTIONS IN: protein kinase activity, kinase activity, ATP binding; INVOLVED IN: protein amino acid phosphorylation; LOCATED IN: cellular_component unknown; EXPRESSED IN: 11 plant structures; EXPRESSED DURING: 4 anthesis, petal differentiation and expansion stage; CONTAINS InterPro DOMAIN/s: Protein kinase, ATP binding site (InterPro:IPR017441), Protein kinase, catalytic domain (InterPro:IPR000719), Serine/threonine-protein kinase-like domain (InterPro:IPR017442), Protein kinase-like domain (InterPro:IPR011009); BEST Arabidopsis thaliana protein match is: Protein kinase superfamily protein (TAIR:AT3G09830.2)
AT4G16980	-0,881		arabinogalactan-protein family; INVOLVED IN: biological_process unknown; LOCATED IN: endomembrane system; EXPRESSED IN: 22 plant structures; EXPRESSED DURING: 13 growth stages
AT2G48080	-0,882		oxidoreductase, 2OG-Fe(II) oxygenase family protein; BEST Arabidopsis thaliana protein match is: oxidoreductase, 2OG-Fe(II) oxygenase family protein (TAIR:AT4G02940.1)
AT4G09890	-0,882		Protein of unknown function (DUF3511); CONTAINS InterPro DOMAIN/s: Protein of unknown function DUF3511 (InterPro:IPR021899); BEST Arabidopsis thaliana protein match is: Protein of unknown function (DUF3511) (TAIR:AT5G11970.1)
AT5G65980	-0,882	PIN-LIKES 7 (PILS7)	Auxin efflux carrier family protein; FUNCTIONS IN: auxin:hydrogen symporter activity; INVOLVED IN: auxin polar transport, transmembrane transport; LOCATED IN: endomembrane system, integral to membrane; CONTAINS InterPro DOMAIN/s: Auxin efflux carrier (InterPro:IPR004776); BEST Arabidopsis thaliana protein match is: Auxin efflux carrier family protein (TAIR:AT2G17500.4)
AT1G47210	-0,883	CYCLIN-DEPENDENT PROTEIN KINASE 3;2 (CYCA3;2)	cyclin-dependent protein kinase 3;2 (CYCA3;2); CONTAINS InterPro DOMAIN/s: Cyclin, C-terminal (InterPro:IPR004367), Cyclin (InterPro:IPR006670), G2/mitotic-specific cyclin A (InterPro:IPR015453), Cyclin-like (InterPro:IPR011028), Cyclin-related (InterPro:IPR013763), Cyclin, N-terminal (InterPro:IPR006671), Cyclin, A/B/D/E (InterPro:IPR014400); BEST Arabidopsis thaliana protein match is: CYCLIN A3;4 (TAIR:AT1G47230.1)
AT4G20260	-0,884	PLASMA-MEMBRANE ASSOCIATED CATION-BINDING PROTEIN 1 (PCAP1)	Encodes a Ca2+ and Cu2+ binding protein. N-terminal myristylation on glycine 2 appears to enable it to associate tightly with the plasma membrane. Recombinant PCaP1 interacts strongly with phosphatidylinositol 3,5-bisphosphate (PtdIns(3,5)P2) and PtdIns (3,4,5)P3, and weakly with PtdIns(3,5)P2 and PtdIns(4,5). It also interacts with calmodulin (CaM) in a calcium-dependent manner. CaM does not interfere with PCaP1 membrane localization but does weaken interactions between it and the PtdInsPs. PCaP1 has an apparent Kd of 10 uM for Cu2+ and can bind six ions per protein. Transcript levels for PCaP1 first fall and then rise following exposure to CuCl2. Mannitol, sorbitol, and the flg22 oligopeptide also increase expression levels. The mRNA is cell-to-cell mobile.
AT5G05400	-0,885		LRR and NB-ARC domains-containing disease resistance protein; FUNCTIONS IN: ATP binding; INVOLVED IN: N-terminal protein myristoylation, defense response, apoptosis; LOCATED IN: chloroplast; EXPRESSED IN: stem, root; CONTAINS InterPro DOMAIN/s: NB-ARC (InterPro:IPR002182), Disease resistance protein (InterPro:IPR000767); BEST Arabidopsis thaliana protein match is: Disease resistance protein (CC-NBS-LRR class) family (TAIR:AT5G63020.1)
AT3G58710	-0,886	WRKY DNA-BINDING PROTEIN 69 (WRKY69)	member of WRKY Transcription Factor; Group II-e
AT4G04955	-0,886	ALLANTOINASE (ALN)	Encodes an allantoinase which is involved in allantoin degradation and assimilation. Gene expression was induced when allantoin was added to the medium. The insertion mutant, ataln m2-1, did not grow well on the MS medium where allantoin, instead of ammonium nitrate, was supplied.
AT5G63160	-0,887	BTB AND TAZ DOMAIN PROTEIN 1 (bt1)	BTB and TAZ domain protein. Short-lived nuclear-cytoplasmic protein targeted for degradation by the 26S proteosome pathway. Acts redundantly with BT2 and BT3 during female gametophyte development.
AT2G17440	-0,888	PLANT INTRACELLULAR RAS GROUP-RELATED LRR 5 (PIRL5)	Encodes PIRL5, a member of the Plant Intracellular Ras-group-related LRRs (Leucine rich repeat proteins). PIRLs are a distinct, plant-specific class of intracellular LRRs that likely mediate protein interactions, possibly in the context of signal transduction. The mRNA is cell-to-cell mobile.
AT5G06280	-0,888		unknown protein; BEST Arabidopsis thaliana protein match is: unknown protein (TAIR:AT3G52520.1)
AT3G18780	-0,89	ACTIN 2 (ACT2)	Encodes an actin that is constitutively expressed in vegetative structures but not pollen. ACT2 is involved in tip growth of root hairs.

AT3G58990	*-0,89*	ISOPROPYLMALATE ISOMERASE 1 (IPMI1)	isopropylmalate isomerase 1 (IPMI1); FUNCTIONS IN: hydro-lyase activity, 3-isopropylmalate dehydratase activity; INVOLVED IN: leucine biosynthetic process, metabolic process; LOCATED IN: plastid; EXPRESSED IN: 19 plant structures; EXPRESSED DURING: 9 growth stages; CONTAINS InterPro DOMAIN/s: 3-isopropylmalate dehydratase, small subunit-like (InterPro:IPR012305), Aconitase A/isopropylmalate dehydratase small subunit, swivel (InterPro:IPR000573), Aconitase/3-isopropylmalate dehydratase, swivel (InterPro:IPR015928), Aconitase-like core (InterPro:IPR015937); BEST Arabidopsis thaliana protein match is: Aconitase/3-isopropylmalate dehydratase protein (TAIR:AT2G43090.1)
AT5G49710	*-0,89*		unknown protein; BEST Arabidopsis thaliana protein match is: unknown protein (TAIR:AT4G24590.1)
AT1G26920	*-0,891*		unknown protein; BEST Arabidopsis thaliana protein match is: unknown protein (TAIR:AT1G69760.1)
AT1G44760	*-0,892*		Adenine nucleotide alpha hydrolases-like superfamily protein; FUNCTIONS IN: molecular_function unknown; INVOLVED IN: response to stress; EXPRESSED IN: 22 plant structures; EXPRESSED DURING: 13 growth stages; CONTAINS InterPro DOMAIN/s: UspA (InterPro:IPR006016), Rossmann-like alpha/beta/alpha sandwich fold (InterPro:IPR014729); BEST Arabidopsis thaliana protein match is: Adenine nucleotide alpha hydrolases-like superfamily protein (TAIR:AT1G69080.1)
AT3G05490	*-0,893*	RALF-LIKE 22 (RALFL22)	Member of a diversely expressed predicted peptide family showing sequence similarity to tobacco Rapid Alkalinization Factor (RALF), and is believed to play an essential role in the physiology of Arabidopsis. Consists of a single exon and is characterized by a conserved C-terminal motif and N-terminal signal peptide.
AT3G45600	*-0,893*	TETRASPANIN3 (TET3)	Member of TETRASPANIN family
AT5G08640	*-0,893*	FLAVONOL SYNTHASE 1 (FLS1)	Encodes a flavonol synthase that catalyzes formation of flavonols from dihydroflavonols. Co-expressed with CHI and CHS (qRT-PCR).
AT4G01480	*-0,894*	PYROPHOSPHORYLASE 5 (PPa5)	Encodes a protein that might have inorganic pyrophosphatase activity.
AT1G15740	*-0,895*		Leucine-rich repeat family protein; BEST Arabidopsis thaliana protein match is: Leucine-rich repeat (LRR) family protein (TAIR:AT4G23840.1)
AT1G21100	*-0,895*	INDOLE GLUCOSINOLATE O-METHYLTRANSFERASE 1 (IGMT1)	O-methyltransferase family protein; FUNCTIONS IN: methyltransferase activity, O-methyltransferase activity, protein dimerization activity; LOCATED IN: cytosol; EXPRESSED IN: stem, cotyledon, hypocotyl, root, leaf; EXPRESSED DURING: LP.04 four leaves visible; CONTAINS InterPro DOMAIN/s: Winged helix-turn-helix transcription repressor DNA-binding (InterPro:IPR011991), Plant methyltransferase dimerisation (InterPro:IPR012967), O-methyltransferase, family 2 (InterPro:IPR001077), O-methyltransferase, COMT, eukaryota (InterPro:IPR016461); BEST Arabidopsis thaliana protein match is: O-methyltransferase family protein (TAIR:AT1G21130.1)
AT3G54140	*-0,897*	NRT1/ PTR FAMILY 8.1 (NPF8.1)	Encodes a di- and tri-peptide transporter that recognizes a variety of different amino acid combinations. GFP-tagged PTR1 localizes to the plasma membrane and has 8 to 11 predicted transmembrane domains. PTR1 is expressed in a number of different vascular tissues throughout the plant based on promoter:GUS expression analysis. ptr1 mutants have a lower dry weight than wild type plants when both are grown with Pro-Ala or Ala-Ala dipeptides as their nitrogen source, suggesting that PTR1 plays a role in dipeptide uptake in the roots. Furthermore N content of ptr1 mutants is lower than that of wild type plants when grown with Pro-Ala or a mixture of dipeptides as nitrogen source
AT1G58030	*-0,898*	CATIONIC AMINO ACID TRANSPORTER 2 (CAT2)	Encodes a member of the cationic amino acid transporter (CAT) subfamily of amino acid polyamine choline transporters. Localized to the tonoplast.
AT1G67710	*-0,898*	RESPONSE REGULATOR 11 (ARR11)	Encodes an Arabidopsis response regulator (ARR) protein that acts in concert with other type-B ARRs in the cytokinin signaling pathway.
AT2G35730	*-0,898*		Heavy metal transport/detoxification superfamily protein ; FUNCTIONS IN: metal ion binding; INVOLVED IN: metal ion transport; LOCATED IN: endomembrane system; EXPRESSED IN: 6 plant structures; EXPRESSED DURING: 4 anthesis, petal differentiation and expansion stage; CONTAINS InterPro DOMAIN/s: Heavy metal transport/detoxification protein (InterPro:IPR006121), Heavy-metal-associated, conserved site (InterPro:IPR017969)

AT1G60160	*-0,899*		Potassium transporter family protein; FUNCTIONS IN: potassium ion transmembrane transporter activity; INVOLVED IN: potassium ion transport; LOCATED IN: chloroplast; EXPRESSED IN: 21 plant structures; EXPRESSED DURING: 13 growth stages; CONTAINS InterPro DOMAIN/s: Potassium uptake protein, kup (InterPro:IPR018519), K+ potassium transporter (InterPro:IPR003855); BEST Arabidopsis thaliana protein match is: K+ uptake permease 7 (TAIR:AT5G09400.1)
AT2G18280	*-0,899*	TUBBY LIKE PROTEIN 2 (TLP2)	Member of TLP family The mRNA is cell-to-cell mobile.
AT5G26310	*-0,899*	(UGT72E3)	UGT72E3 is an UDPG:coniferyl alcohol glucosyltransferase which glucosylates sinapyl- and coniferyl alcohol as well as sinapic acid. The enzyme is thought to be involved in lignin- and phenylpropanoid metabolism. A knockdown mutant line (72E3KD) was obtained using RNAi silencing. No reduction in coniferyl alcohol 4-<i>O</i>-glucoside and sinapyl alcohol 4-<i>O</i>-glucoside was detected in this line compared to wildtype, in contrast with the knockdown line constructed for UGT72E2 displayed a twofold reduction in the these phenylpropanoid 4-<i>O</i>-glucosides.
AT5G50200	*-0,899*	WOUND-RESPONSIVE 3 (WR3)	Wound-responsive gene 3 (WR3). Encodes a high-affinity nitrate transporter. Up-regulated by nitrate. Involved in jasmonic acid-independent wound signal transduction.
AT2G04780	*-0,9*	FASCICLIN-LIKE ARABINOOGALACTAN 7 (FLA7)	fasciclin-like arabinogalactan-protein 7 (Fla7)
AT2G24300	*-0,9*		Calmodulin-binding protein; CONTAINS InterPro DOMAIN/s: Calmodulin binding protein-like (InterPro:IPR012416); BEST Arabidopsis thaliana protein match is: Calmodulin-binding protein (TAIR:AT4G31000.1)
AT4G31290	*-0,9*	GAMMA-GLUTAMYL CYCLOTRANSFERASE 2;2 (GGCT2;2)	ChaC-like family protein; CONTAINS InterPro DOMAIN/s: ChaC-like protein (InterPro:IPR006840); BEST Arabidopsis thaliana protein match is: ChaC-like family protein (TAIR:AT5G26220.1)
AT3G16240	*-0,901*	DELTA TONOPLAST INTEGRAL PROTEIN (DELTA-TIP)	Delta tonoplast intrinsic protein, functions as a water channel and ammonium (NH3) transporter. Highly expressed in flower, shoot, and stem. Expression shows diurnal regulation and is induced by ammonium (NH3). Protein localized to vacuolar membrane. The mRNA is cell-to-cell mobile.
AT4G26080	*-0,902*	ABA INSENSITIVE 1 (ABI1)	Involved in abscisic acid (ABA) signal transduction. Negative regulator of ABA promotion of stomatal closure.
AT1G59590	*-0,903*	(ZCF37)	ZCF37 mRNA, complete cds The mRNA is cell-to-cell mobile.
AT2G19110	*-0,903*	HEAVY METAL ATPASE 4 (HMA4)	Encodes a protein with similarity to Zn ATPase. Can rescue Zn deficiency in yeast and Cd resistance, suggesting a role in Zn and Cd transport. The mRNA is cell-to-cell mobile.
AT5G46870	*-0,903*		RNA-binding (RRM/RBD/RNP motifs) family protein; FUNCTIONS IN: oxidoreductase activity, nucleotide binding, nucleic acid binding; INVOLVED IN: oxidation reduction; EXPRESSED IN: cultured cell; CONTAINS InterPro DOMAIN/s: Aldo/keto reductase (InterPro:IPR001395), RNA recognition motif, RNP-1 (InterPro:IPR000504), Nucleotide-binding, alpha-beta plait (InterPro:IPR012677); BEST Arabidopsis thaliana protein match is: RNA-binding (RRM/RBD/RNP motifs) family protein (TAIR:AT4G17720.1)
AT2G35270	*-0,904*	AT-HOOK MOTIF NUCLEAR LOCALIZED PROTEIN 21 (AHL21)	Direct target of AGAMOUS. Regulates patterning and differentiation of reproductive organs.
AT5G65830	*-0,904*	RECEPTOR LIKE PROTEIN 57 (RLP57)	receptor like protein 57 (RLP57); INVOLVED IN: signal transduction; LOCATED IN: endomembrane system; EXPRESSED IN: 21 plant structures; EXPRESSED DURING: 13 growth stages; CONTAINS InterPro DOMAIN/s: Leucine-rich repeat (InterPro:IPR001611); BEST Arabidopsis thaliana protein match is: receptor like protein 44 (TAIR:AT3G49750.1)
AT1G17240	*-0,906*	RECEPTOR LIKE PROTEIN 2 (RLP2)	Encodes a CLAVATA2 (CLV2)-related gene. Complements the clv2 mutant when expressed under the control of the CLV2 promoter.
AT1G67050	*-0,906*		unknown protein; BEST Arabidopsis thaliana protein match is: unknown protein (TAIR:AT5G38320.1)
AT1G67940	*-0,906*	ATP-BINDING CASSETTE I17 (ABCI17)	member of NAP subfamily The mRNA is cell-to-cell mobile.
AT5G67620	*-0,906*		unknown protein; FUNCTIONS IN: molecular_function unknown; INVOLVED IN: N-terminal protein myristoylation; LOCATED IN: microtubule; BEST Arabidopsis thaliana protein match is: unknown protein (TAIR:AT5G62900.1)

AT2G06200	-0,907	GROWTH-REGULATING FACTOR 6 (GRF6)	Growth regulating factor encoding transcription activator. One of the nine members of a GRF gene family, containing nuclear targeting domain. Involved in leaf development and expressed in root, shoot and flower
AT4G15070	-0,907		Cysteine/Histidine-rich C1 domain family protein; FUNCTIONS IN: molecular_function unknown; INVOLVED IN: intracellular signaling pathway; LOCATED IN: cellular_component unknown; CONTAINS InterPro DOMAIN/s: Protein kinase C-like, phorbol ester/diacylglycerol binding (InterPro:IPR002219), DC1 (InterPro:IPR004146), C1-like (InterPro:IPR011424); BEST Arabidopsis thaliana protein match is: Cysteine/Histidine-rich C1 domain family protein (TAIR:AT5G48320.1)
AT5G16170	-0,907		Core-2/I-branching beta-1,6-N-acetylglucosaminyltransferase family protein; CONTAINS InterPro DOMAIN/s: Core-2/I-Branching enzyme (InterPro:IPR021141); BEST Arabidopsis thaliana protein match is: Core-2/I-branching beta-1,6-N-acetylglucosaminyltransferase family protein (TAIR:AT5G11730.1)
AT4G36790	-0,908		Major facilitator superfamily protein; FUNCTIONS IN: carbohydrate transmembrane transporter activity, sugar:hydrogen symporter activity; INVOLVED IN: transmembrane transport; LOCATED IN: membrane; EXPRESSED IN: 18 plant structures; EXPRESSED DURING: 12 growth stages; CONTAINS InterPro DOMAIN/s: Major facilitator superfamily (InterPro:IPR020846), Major facilitator superfamily MFS-1 (InterPro:IPR011701), Major facilitator superfamily, general substrate transporter (InterPro:IPR016196); BEST Arabidopsis thaliana protein match is: Major facilitator superfamily protein (TAIR:AT2G18590.1)
AT2G38600	-0,909		HAD superfamily, subfamily IIIB acid phosphatase ; FUNCTIONS IN: acid phosphatase activity; INVOLVED IN: biological_process unknown; LOCATED IN: endomembrane system; EXPRESSED IN: 15 plant structures; EXPRESSED DURING: 6 growth stages; CONTAINS InterPro DOMAIN/s: Acid phosphatase (Class B) (InterPro:IPR005519), Vegetative storage protein/acid phosphatase (InterPro:IPR014403), Acid phosphatase, plant (InterPro:IPR010028); BEST Arabidopsis thaliana protein match is: HAD superfamily, subfamily IIIB acid phosphatase (TAIR:AT4G29260.1)
AT4G23050	-0,909		PAS domain-containing protein tyrosine kinase family protein; FUNCTIONS IN: protein serine/threonine/tyrosine kinase activity, protein kinase activity, signal transducer activity; INVOLVED IN: signal transduction, protein amino acid phosphorylation, regulation of transcription, DNA-dependent; EXPRESSED IN: 21 plant structures; EXPRESSED DURING: 11 growth stages; CONTAINS InterPro DOMAIN/s: PAC motif (InterPro:IPR001610), Protein kinase, ATP binding site (InterPro:IPR017441), PAS fold (InterPro:IPR013767), PAS (InterPro:IPR000014), Serine-threonine/tyrosine-protein kinase (InterPro:IPR001245), Protein kinase-like domain (InterPro:IPR011009), Serine/threonine-protein kinase, active site (InterPro:IPR008271), Protein kinase, catalytic domain (InterPro:IPR000719); BEST Arabidopsis thaliana protein match is: PAS domain-containing protein tyrosine kinase family protein (TAIR:AT5G49470.3)
AT1G72800	-0,91		RNA-binding (RRM/RBD/RNP motifs) family protein; FUNCTIONS IN: nucleotide binding, nucleic acid binding; INVOLVED IN: biological_process unknown; LOCATED IN: cellular_component unknown; EXPRESSED IN: 21 plant structures; EXPRESSED DURING: 10 growth stages; CONTAINS InterPro DOMAIN/s: RNA recognition motif, RNP-1 (InterPro:IPR000504), Nucleotide-binding, alpha-beta plait (InterPro:IPR012677); BEST Arabidopsis thaliana protein match is: nucleolin like 2 (TAIR:AT3G18610.1)
AT1G76990	-0,91	ACT DOMAIN REPEAT 3 (ACR3)	ACT domain repeat 3 (ACR3); FUNCTIONS IN: amino acid binding; INVOLVED IN: nitrogen compound metabolic process, metabolic process; LOCATED IN: cytosol; EXPRESSED IN: 22 plant structures; EXPRESSED DURING: 13 growth stages; CONTAINS InterPro DOMAIN/s: Protein-PII uridylyltransferase (InterPro:IPR010043), Amino acid-binding ACT (InterPro:IPR002912); BEST Arabidopsis thaliana protein match is: ACT domain repeat 4 (TAIR:AT1G69040.2)
AT5G58540	-0,91		Protein kinase superfamily protein; FUNCTIONS IN: protein kinase activity, kinase activity, ATP binding; INVOLVED IN: protein amino acid phosphorylation; EXPRESSED IN: 7 plant structures; EXPRESSED DURING: LP.04 four leaves visible, 4 anthesis, petal differentiation and expansion stage; CONTAINS InterPro DOMAIN/s: Protein kinase, catalytic domain (InterPro:IPR000719), Serine-threonine/tyrosine-protein kinase (InterPro:IPR001245), Protein kinase-like domain (InterPro:IPR011009); BEST Arabidopsis thaliana protein match is: Leucine-rich repeat protein kinase family protein (TAIR:AT5G07150.1)
AT4G31330	-0,911		Protein of unknown function, DUF599; FUNCTIONS IN: molecular_function unknown; INVOLVED IN: biological_process unknown; LOCATED IN: endomembrane system; EXPRESSED IN: 14 plant structures; EXPRESSED DURING: LP.06 six leaves visible, LP.04 four leaves visible, 4 anthesis, petal differentiation and expansion stage, LP.08 eight leaves visible; CONTAINS InterPro DOMAIN/s: Protein of unknown function DUF599 (InterPro:IPR006747); BEST Arabidopsis thaliana protein match is: Protein of unknown function, DUF599 (TAIR:AT5G10580.1)

AT1G04250	*-0,912*	AUXIN RESISTANT 3 (AXR3)	Transcription regulator acting as repressor of auxin-inducible gene expression. Auxin-inducible AUX/IAA gene. Short-lived nuclear protein with four conserved domains. Domain III has homology to beta alpha alpha dimerization and DNA binding domains. Involved in auxin signaling and is a positive modulator of natural leaf senescence. Auxin induces the degradation of the protein in a dosage-dependent manner in a process mediated by AtRac1. Auxin induced the relocalization of the protein within the nucleus from a diffused nucleoplasmic pattern to a discrete particulated pattern named nuclear protein bodies or NPB in a process also mediated by Rac1. Colocalizes with SCF, CSN and 26S proteasome components.
AT5G57630	*-0,912*	CBL-INTERACTING PROTEIN KINASE 21 (CIPK21)	CBL-interacting protein kinase.When mutated plants are hypersensitive to salt and osmotic stress.
AT4G34720	*-0,913*	(AVA-P1)	vacuolar H+-pumping ATPase 16 kDa proteolipid (ava-p1)
AT5G07870	*-0,913*		HXXXD-type acyl-transferase family protein; FUNCTIONS IN: transferase activity, transferring acyl groups other than amino-acyl groups, transferase activity; INVOLVED IN: biological_process unknown; LOCATED IN: cellular_component unknown; EXPRESSED IN: 20 plant structures; EXPRESSED DURING: 12 growth stages; CONTAINS InterPro DOMAIN/s: Transferase (InterPro:IPR003480); BEST Arabidopsis thaliana protein match is: HXXXD-type acyl-transferase family protein (TAIR:AT5G07860.1)
AT5G15330	*-0,913*	SPX DOMAIN GENE 4 (SPX4)	SPX domain gene 4 (SPX4); CONTAINS InterPro DOMAIN/s: SPX, N-terminal (InterPro:IPR004331); BEST Arabidopsis thaliana protein match is: SPX domain gene 1 (TAIR:AT5G20150.1)
AT3G09790	*-0,914*	UBIQUITIN 8 (UBQ8)	encodes a ubiquitin-like protein that contains tandem repeats of the ubiquitin coding region, but at least one repeat per gene encodes a protein with amino acid substitutions.
AT4G27260	*-0,914*	(WES1)	encodes an IAA-amido synthase that conjugates Asp and other amino acids to auxin in vitro. Lines carrying insertions in this gene are hypersensitive to auxin. It is involved in camalexin biosynthesis via conjugating indole-3-carboxylic acid (ICA) and cysteine (Cys). The mRNA is cell-to-cell mobile.
AT5G04080	*-0,914*		unknown protein; FUNCTIONS IN: molecular_function unknown; INVOLVED IN: biological_process unknown; LOCATED IN: plasma membrane; EXPRESSED IN: 16 plant structures; EXPRESSED DURING: 9 growth stages
AT1G49860	*-0,915*	GLUTATHIONE S-TRANSFERASE (CLASS PHI) 14	Encodes glutathione transferase belonging to the phi class of GSTs. Naming convention according to Wagner et al. (2002). The mRNA is cell-to-cell mobile.
AT5G37450	*-0,915*		Leucine-rich repeat protein kinase family protein; FUNCTIONS IN: protein serine/threonine kinase activity, kinase activity, ATP binding; INVOLVED IN: transmembrane receptor protein tyrosine kinase signaling pathway, protein amino acid phosphorylation; LOCATED IN: endomembrane system; EXPRESSED IN: root; CONTAINS InterPro DOMAIN/s: Protein kinase, ATP binding site (InterPro:IPR017441), Serine/threonine-protein kinase domain (InterPro:IPR002290), Leucine-rich repeat-containing N-terminal domain, type 2 (InterPro:IPR013210), Leucine-rich repeat (InterPro:IPR001611), Serine/threonine-protein kinase-like domain (InterPro:IPR017442), Serine/threonine-protein kinase, active site (InterPro:IPR008271), Protein kinase-like domain (InterPro:IPR011009), Protein kinase, catalytic domain (InterPro:IPR000719), Tyrosine-protein kinase, catalytic domain (InterPro:IPR020635); BEST Arabidopsis thaliana protein match is: Leucine-rich repeat protein kinase family protein (TAIR:AT1G06840.1)
AT1G63840	*-0,916*		RING/U-box superfamily protein; FUNCTIONS IN: zinc ion binding; INVOLVED IN: response to abscisic acid stimulus; LOCATED IN: endomembrane system; EXPRESSED IN: 22 plant structures; EXPRESSED DURING: 13 growth stages; CONTAINS InterPro DOMAIN/s: Zinc finger, RING-type (InterPro:IPR001841), Zinc finger, C3HC4 RING-type (InterPro:IPR018957); BEST Arabidopsis thaliana protein match is: RING/U-box superfamily protein (TAIR:AT5G41400.1)
AT5G01800	*-0,916*		saposin B domain-containing protein; FUNCTIONS IN: molecular_function unknown; INVOLVED IN: N-terminal protein myristoylation, lipid metabolic process; LOCATED IN: endomembrane system; EXPRESSED IN: 23 plant structures; EXPRESSED DURING: 15 growth stages; CONTAINS InterPro DOMAIN/s: Saposin B (InterPro:IPR008139), Saposin-like (InterPro:IPR011001), Saposin-like type B, 1 (InterPro:IPR007856), Saposin-like type B, 2 (InterPro:IPR008138); BEST Arabidopsis thaliana protein match is: saposin B domain-containing protein (TAIR:AT3G51730.1)

AT5G16590	-0,916	(LRR1)	Leucine-rich repeat protein kinase family protein; FUNCTIONS IN: protein serine/threonine kinase activity, kinase activity, ATP binding; INVOLVED IN: protein amino acid phosphorylation, transmembrane receptor protein tyrosine kinase signaling pathway, response to symbiotic fungus; LOCATED IN: plasma membrane, membrane, plant-type cell wall; EXPRESSED IN: 24 plant structures; EXPRESSED DURING: 13 growth stages; CONTAINS InterPro DOMAIN/s: Protein kinase, catalytic domain (InterPro:IPR000719), Serine/threonine-protein kinase domain (InterPro:IPR002290), Leucine-rich repeat-containing N-terminal domain, type 2 (InterPro:IPR013210), Tyrosine-protein kinase, catalytic domain (InterPro:IPR020635), Serine/threonine-protein kinase-like domain (InterPro:IPR017442), Protein kinase-like domain (InterPro:IPR011009); BEST Arabidopsis thaliana protein match is: Leucine-rich repeat protein kinase family protein (TAIR:AT3G02880.1)
AT1G21920	-0,917		Histone H3 K4-specific methyltransferase SET7/9 family protein; CONTAINS InterPro DOMAIN/s: MORN motif (InterPro:IPR003409); BEST Arabidopsis thaliana protein match is: Histone H3 K4-specific methyltransferase SET7/9 family protein (TAIR:AT1G77660.1)
AT2G20500	-0,918		unknown protein; FUNCTIONS IN: molecular_function unknown; INVOLVED IN: biological_process unknown; LOCATED IN: mitochondrion; EXPRESSED IN: 21 plant structures; EXPRESSED DURING: 13 growth stages
AT3G11660	-0,918	NDR1/HIN1-LIKE 1 (NHL1)	encodes a protein whose sequence is similar to tobacco hairpin-induced gene (HIN1) and Arabidopsis non-race specific disease resistance gene (NDR1). Expression of this gene is induced by cucumber mosaic virus. Localization of the gene product is similar to that of NHL3 (plasma membrane) but it is yet inconclusive.
AT4G35030	-0,919		Protein kinase superfamily protein; FUNCTIONS IN: protein serine/threonine kinase activity, protein kinase activity, kinase activity, ATP binding; INVOLVED IN: protein amino acid phosphorylation; LOCATED IN: cellular_component unknown; EXPRESSED IN: 20 plant structures; EXPRESSED DURING: 12 growth stages; CONTAINS InterPro DOMAIN/s: Protein kinase, catalytic domain (InterPro:IPR000719), Serine/threonine-protein kinase domain (InterPro:IPR002290), Tyrosine-protein kinase, catalytic domain (InterPro:IPR020635), Serine/threonine-protein kinase-like domain (InterPro:IPR017442), Serine/threonine-protein kinase, active site (InterPro:IPR008271), Protein kinase-like domain (InterPro:IPR011009); BEST Arabidopsis thaliana protein match is: Protein kinase protein with adenine nucleotide alpha hydrolases-like domain (TAIR:AT2G16750.1).
AT1G66180	-0,92		The gene encodes a putative aspartyl protease (ASP). Its expression is induced in response to light and ascorbate. The mRNA is cell-to-cell mobile.
AT3G19930	-0,92	SUGAR TRANSPORTER 4 (STP4)	Encodes a sucrose hydrogen symporter that is induced by wounding. The mRNA is cell-to-cell mobile.
AT3G21670	-0,92	NRT1/ PTR FAMILY 6.4 (NPF6.4)	Major facilitator superfamily protein; FUNCTIONS IN: transporter activity; INVOLVED IN: oligopeptide transport; LOCATED IN: membrane; EXPRESSED IN: 21 plant structures; EXPRESSED DURING: 13 growth stages; CONTAINS InterPro DOMAIN/s: Oligopeptide transporter (InterPro:IPR000109), Major facilitator superfamily, general substrate transporter (InterPro:IPR016196); BEST Arabidopsis thaliana protein match is: Major facilitator superfamily protein (TAIR:AT2G26690.1)
AT4G00360	-0,92	CYTOCHROME P450, FAMILY 86, SUBFAMILY A, POLYPEPTIDE 2 (CYP86A2)	Encodes a member of the CYP86A subfamily of cytochrome p450 genes. Expressed at moderate levels in flowers, leaves, roots and stems.
AT4G26060	-0,921		Ribosomal protein L18ae family; BEST Arabidopsis thaliana protein match is: unknown protein (TAIR:AT5G57060.1)
AT4G31730	-0,921	GLUTAMINE DUMPER 1 (GDU1)	Glutamine dumper1 is a putative transmembrane protein. It is involved in glutamine secretion The mRNA is cell-to-cell mobile.
AT5G44700	-0,921	GASSHO 2 (GSO2)	Encodes GASSHO2 (GSO2), a putative leucine-rich repeat transmembrane-type receptor kinase. GSO2 and a homolog GSO1 (At4g20140) are required for the formation of a normal epidermal surface during embryogenesis.
AT5G60760	-0,921		P-loop containing nucleoside triphosphate hydrolases superfamily protein; BEST Arabidopsis thaliana protein match is: P-loop containing nucleoside triphosphate hydrolases superfamily protein (TAIR:AT3G45090.1)
AT1G68440	-0,922		unknown protein; BEST Arabidopsis thaliana protein match is: unknown protein (TAIR:AT1G25400.2)
AT1G13750	-0,923	PURPLE ACID PHOSPHATASE 1 (PAP1)	Encodes a purple acid phosphatase whose expression is responsive to both phosphate (Pi) and phosphite (Phi) in roots.

AT2G38540	*-0,923*	LIPID TRANSFER PROTEIN 1 (LP1)	Non-specific lipid transfer protein. Binds calmodulin in a Ca2+-independent manner. Localized to the cell wall. Specifically expressed in L1 epidermal layer. Predicted to be a member of PR-14 pathogenesis-related protein family with the following members: At2g38540/LTP1, At2g38530/LTP2, At5g59320/LTP3, At5g59310/LTP4, At3g51600/LTP5, At3g08770/LTP6, At2g15050/LTP7, At2g18370/LTP8, At2g15325/LTP9, At5g01870/LTP10, At4g33355/LTP11, At3g51590/LTP12, At5g44265/LTP13, At5g62065/LTP14, At4g08530/LTP15. The mRNA is cell-to-cell mobile.
AT5G55970	*-0,923*		RING/U-box superfamily protein; FUNCTIONS IN: zinc ion binding; LOCATED IN: chloroplast; EXPRESSED IN: 15 plant structures; EXPRESSED DURING: LP.06 six leaves visible, LP.04 four leaves visible, 4 anthesis, 4 leaf senescence stage, petal differentiation and expansion stage; CONTAINS InterPro DOMAIN/s: Zinc finger, RING-type (InterPro:IPR001841), Zinc finger, C3HC4 RING-type (InterPro:IPR018957); BEST Arabidopsis thaliana protein match is: RING/U-box superfamily protein (TAIR:AT4G26580.2)
AT1G24320	*-0,925*		Six-hairpin glycosidases superfamily protein; FUNCTIONS IN: mannosyl-oligosaccharide glucosidase activity, alpha-glucosidase activity, catalytic activity; INVOLVED IN: oligosaccharide metabolic process; LOCATED IN: cellular_component unknown; EXPRESSED IN: 6 plant structures; EXPRESSED DURING: F mature embryo stage, petal differentiation and expansion stage, D bilateral stage; CONTAINS InterPro DOMAIN/s: Glycoside hydrolase, family 63 (InterPro:IPR004888), Six-hairpin glycosidase-like (InterPro:IPR008928); BEST Arabidopsis thaliana protein match is: glucosidase 1 (TAIR:AT1G67490.1)
AT1G53250	*-0,925*		unknown protein; BEST Arabidopsis thaliana protein match is: unknown protein (TAIR:AT1G53800.1)
AT5G43040	*-0,925*		Cysteine/Histidine-rich C1 domain family protein; FUNCTIONS IN: zinc ion binding; INVOLVED IN: intracellular signaling pathway; EXPRESSED IN: 26 plant structures; EXPRESSED DURING: 13 growth stages; CONTAINS InterPro DOMAIN/s: Protein kinase C-like, phorbol ester/diacylglycerol binding (InterPro:IPR002219), DC1 (InterPro:IPR004146), Zinc finger, PHD-type (InterPro:IPR001965), C1-like (InterPro:IPR011424); BEST Arabidopsis thaliana protein match is: Cysteine/Histidine-rich C1 domain family protein (TAIR:AT5G43030.1)
AT1G12010	*-0,926*		Encodes a protein that appears to have 1-amino-cyclopropane-1-carboxylic acid oxidase activity based on mutant analyses. The mRNA is cell-to-cell mobile.
AT1G26450	*-0,926*		Carbohydrate-binding X8 domain superfamily protein; CONTAINS InterPro DOMAIN/s: X8 (InterPro:IPR012946); BEST Arabidopsis thaliana protein match is: plasmodesmata callose-binding protein 3 (TAIR:AT1G18650.1)
AT2G33990	*-0,926*	IQ-DOMAIN 9 (iqd9)	IQ-domain 9 (iqd9); FUNCTIONS IN: calmodulin binding; INVOLVED IN: biological_process unknown; LOCATED IN: plasma membrane; EXPRESSED IN: 22 plant structures; EXPRESSED DURING: 13 growth stages; CONTAINS InterPro DOMAIN/s: IQ calmodulin-binding region (InterPro:IPR000048); BEST Arabidopsis thaliana protein match is: IQ-domain 10 (TAIR:AT3G15050.1)
AT5G47530	*-0,926*		Auxin-responsive family protein; CONTAINS InterPro DOMAIN/s: Cytochrome b561, eukaryote (InterPro:IPR004877), Uncharacterised conserved protein UCP037471 (InterPro:IPR017214), Protein of unknown function DUF568, DOMON-like (InterPro:IPR007613), DOMON related (InterPro:IPR005018), Cytochrome b561/ferric reductase transmembrane (InterPro:IPR006593); BEST Arabidopsis thaliana protein match is: Auxin-responsive family protein (TAIR:AT4G17280.1)
AT5G58350	*-0,926*	WITH NO LYSINE (K) KINASE 4 (WNK4)	Encodes a member of the WNK family (9 members in all) of protein kinases, the structural design of which is clearly distinct from those of other known protein kinases, such as receptor-like kinases and mitogen-activated protein kinases. Its transcription is under the control of circadian rhythms.
AT1G58190	*-0,927*	RECEPTOR LIKE PROTEIN 9 (RLP9)	receptor like protein 9 (RLP9); INVOLVED IN: signal transduction; LOCATED IN: endomembrane system; CONTAINS InterPro DOMAIN/s: Leucine-rich repeat, typical subtype (InterPro:IPR003591), Leucine-rich repeat-containing N-terminal domain, type 2 (InterPro:IPR013210), Leucine-rich repeat (InterPro:IPR001611); BEST Arabidopsis thaliana protein match is: receptor like protein 13 (TAIR:AT1G74170.1).
AT5G06930	*-0,927*		LOCATED IN: chloroplast; EXPRESSED IN: 15 plant structures; EXPRESSED DURING: 7 growth stages; BEST Arabidopsis thaliana protein match is: nucleolar protein gar2-related (TAIR:AT2G42320.2)
AT5G44110	*-0,927*	ATP-BINDING CASSETTE A21 (ABCI21)	Encodes a member of the NAP subfamily of ABC transporters.

AT2G18350	*-0,928*	HOMEOBOX PROTEIN 24 (HB24)	homeobox protein 24 (HB24); FUNCTIONS IN: DNA binding, sequence-specific DNA binding transcription factor activity; INVOLVED IN: regulation of transcription; LOCATED IN: cellular_component unknown; EXPRESSED IN: 19 plant structures; EXPRESSED DURING: 12 growth stages; CONTAINS InterPro DOMAIN/s: Homeobox domain, ZF-HD class (InterPro:IPR006455), ZF-HD homeobox protein, Cys/His-rich dimerisation domain (InterPro:IPR006456), Homeodomain-related (InterPro:IPR012287); BEST Arabidopsis thaliana protein match is: homeobox protein 28 (TAIR:AT3G50890.1)
AT5G06300	*-0,928*	LONELY GUY 7 (LOG7)	Putative lysine decarboxylase family protein; CONTAINS InterPro DOMAIN/s: Conserved hypothetical protein CHP00730 (InterPro:IPR005269); BEST Arabidopsis thaliana protein match is: lysine decarboxylase family protein (TAIR:AT2G37210.1)
AT4G02100	*-0,929*		Heat shock protein DnaJ with tetratricopeptide repeat; FUNCTIONS IN: binding, heat shock protein binding; INVOLVED IN: protein folding; LOCATED IN: cellular_component unknown; EXPRESSED IN: leaf; CONTAINS InterPro DOMAIN/s: Tetratricopeptide-like helical (InterPro:IPR011990), Heat shock protein DnaJ, N-terminal (InterPro:IPR001623); BEST Arabidopsis thaliana protein match is: Tetratricopeptide repeat (TPR)-like superfamily protein (TAIR:AT2G47440.1)
AT5G04840	*-0,929*		bZIP protein; FUNCTIONS IN: DNA binding, sequence-specific DNA binding transcription factor activity; INVOLVED IN: regulation of transcription, DNA-dependent; EXPRESSED IN: 12 plant structures; EXPRESSED DURING: 6 growth stages; CONTAINS InterPro DOMAIN/s: Basic-leucine zipper (bZIP) transcription factor (InterPro:IPR004827); BEST Arabidopsis thaliana protein match is: Basic-leucine zipper (bZIP) transcription factor family protein (TAIR:AT3G58120.1)
AT5G10970	*-0,929*		C2H2 and C2HC zinc fingers superfamily protein; FUNCTIONS IN: sequence-specific DNA binding transcription factor activity, zinc ion binding, nucleic acid binding; INVOLVED IN: regulation of transcription; LOCATED IN: intracellular; EXPRESSED IN: hypocotyl, root, leaf; EXPRESSED DURING: LP.04 four leaves visible; CONTAINS InterPro DOMAIN/s: Zinc finger, C2H2-like (InterPro:IPR015880), Zinc finger, C2H2-type (InterPro:IPR007087); BEST Arabidopsis thaliana protein match is: zinc finger protein 3 (TAIR:AT5G25160.1)
AT1G70950	*-0,93*		TPX2 (targeting protein for Xklp2) protein family; CONTAINS InterPro DOMAIN/s: Xklp2 targeting protein (InterPro:IPR009675); BEST Arabidopsis thaliana protein match is: unknown protein (TAIR:AT1G23060.1)
AT1G26250	*-0,931*	EXTENSIN18 (EXT18)	Proline-rich extensin-like family protein; FUNCTIONS IN: structural constituent of cell wall; INVOLVED IN: plant-type cell wall organization; LOCATED IN: endomembrane system; CONTAINS InterPro DOMAIN/s: Extensin-like repeat (InterPro:IPR006706); BEST Arabidopsis thaliana protein match is: Proline-rich extensin-like family protein (TAIR:AT4G08370.1)
AT1G54740	*-0,931*		Protein of unknown function (DUF3049); CONTAINS InterPro DOMAIN/s: Protein of unknown function DUF3049 (InterPro:IPR021410); BEST Arabidopsis thaliana protein match is: Protein of unknown function (DUF3049) (TAIR:AT5G22390.1)
AT1G55160	*-0,931*		unknown protein; FUNCTIONS IN: molecular_function unknown; INVOLVED IN: biological_process unknown; LOCATED IN: mitochondrion, plastid; EXPRESSED IN: 22 plant structures; EXPRESSED DURING: 13 growth stages; BEST Arabidopsis thaliana protein match is: unknown protein (TAIR:AT2G19530.1)
AT3G50740	*-0,931*	UDP-GLUCOSYL TRANSFERASE 72E1 (UGT72E1)	UGT72E1 is an UDPG:coniferyl alcohol glucosyltransferase which specifically glucosylates sinapyl- and coniferyl aldehydes. The enzyme is thought to be involved in lignin metabolism.
AT1G12110	*-0,932*	NRT1/ PTR FAMILY 6.3 (NPF6.3)	Encodes NRT1.1 (CHL1), a dual-affinity nitrate transporter. The protein is expressed in guard cells and function in stomatal opening. Mutants have less transpiration and are more tolerant to drought. Expressed in lateral roots. Involved in nitrate signaling which enables the plant root system to detect and exploit nitrate-rich soil patches. Comparing to the wild type, the mutant displays a strongly decreased lateral root proliferation phenotype in nitrate rich patches on growth medium.
AT1G32460	*-0,932*		unknown protein
AT5G19860	*-0,932*		Protein of unknown function, DUF538; INVOLVED IN: biological_process unknown; LOCATED IN: vacuole; EXPRESSED IN: 23 plant structures; EXPRESSED DURING: 15 growth stages; CONTAINS InterPro DOMAIN/s: Protein of unknown function DUF538 (InterPro:IPR007493); BEST Arabidopsis thaliana protein match is: Protein of unknown function, DUF538 (TAIR:AT1G55265.1)
AT5G43830	*-0,932*		Aluminium induced protein with YGL and LRDR motifs; FUNCTIONS IN: molecular_function unknown; INVOLVED IN: biological_process unknown; LOCATED IN: cytosol, nucleus; EXPRESSED IN: 26 plant structures; EXPRESSED DURING: 15 growth stages; BEST Arabidopsis thaliana protein match is: Aluminium induced protein with YGL and LRDR motifs (TAIR:AT3G22850.1)

AT1G29670	*-0,933*		GDSL-motif esterase/acyltransferase/lipase. Enzyme group with broad substrate specificity that may catalyze acyltransfer or hydrolase reactions with lipid and non-lipid substrates. The mRNA is cell-to-cell mobile.
AT1G55990	*-0,933*		glycine-rich protein; BEST Arabidopsis thaliana protein match is: glycine-rich protein (TAIR:AT4G33610.1)
AT3G11370	*-0,934*		Cysteine/Histidine-rich C1 domain family protein; CONTAINS InterPro DOMAIN/s: DC1 (InterPro:IPR004146), C1-like (InterPro:IPR011424); BEST Arabidopsis thaliana protein match is: Cysteine/Histidine-rich C1 domain family protein (TAIR:AT3G11390.1)
AT4G18550	*-0,934*	DAD1-LIKE SEEDING ESTABLISHMENT-RELATED LIPASE (DSEL)	DSEL is cytosolic acylhydrolase that shows prefential lipase activity against the sn-1 position of several classes of lipids, including 1,3-diacylglycerols and 1-monoacylglycerols. Overexpression of DSEL leads to increased peroxisome and oil body levels in cotyledons and reduced beta-oxidation activity in seedlings.
AT4G37520	*-0,934*		Peroxidase superfamily protein; FUNCTIONS IN: protein binding, peroxidase activity; INVOLVED IN: oxidation reduction, response to oxidative stress; LOCATED IN: cytoplasm; CONTAINS InterPro DOMAIN/s: Haem peroxidase (InterPro:IPR010255), Plant peroxidase (InterPro:IPR000823), Peroxidases heam-ligand binding site (InterPro:IPR019793), Haem peroxidase, plant/fungal/bacterial (InterPro:IPR002016); BEST Arabidopsis thaliana protein match is: Peroxidase superfamily protein (TAIR:AT4G37530.1)
AT1G19190	*-0,935*		alpha/beta-Hydrolases superfamily protein; FUNCTIONS IN: hydrolase activity; INVOLVED IN: metabolic process; LOCATED IN: cellular_component unknown; EXPRESSED IN: inflorescence meristem, root, flower; EXPRESSED DURING: petal differentiation and expansion stage; CONTAINS InterPro DOMAIN/s: Lipase, GDXG, active site (InterPro:IPR002168), Alpha/beta hydrolase fold-3 (InterPro:IPR013094); BEST Arabidopsis thaliana protein match is: alpha/beta-Hydrolases superfamily protein (TAIR:AT2G03550.1)
AT5G13180	*-0,936*	NAC DOMAIN CONTAINING PROTEIN 83 (NAC083)	Encodes a NAC domain transcription factor that interacts with VND7 and negatively regulates xylem vessel formation.
AT1G47290	*-0,937*	3BETA-HYDROXYSTEROID-DEHYDROGENASE/DECARBOXYLASE ISOFORM 1 (3BETAHSD/D1)	Encodes an enzyme with 3β-hydroxysteroid dehydrogenase/C4-decarboxylase activity <i>in vitro</i>. The activity of the enzyme was determined using microsomal extracts of yeast overexpressing the <i>Arabidopsis</i> gene. Cytosolic fractions failed to be associated to the activity, leading to the speculation that the enzyme is membrane-bound.
AT2G27830	*-0,937*		unknown protein; BEST Arabidopsis thaliana protein match is: unknown protein (TAIR:AT4G22758.1)
AT5G14200	*-0,937*	ISOPROPYLMALATE DEHYDROGENASE 1 (IMD1)	The AtIMD1 is one out of 3 genes encoding the enzyme 3-isopropylmalate dehydrogenase involved in leucine biosynthesis in Arabidopsis. Its subcellular location has been targeted to plastids. Encodes methylthioalkylmalate dehydrogenase. Involved in glucosinolate biosynthesis, in methionine chain elongation. The mRNA is cell-to-cell mobile.
AT5G19050	*-0,937*		alpha/beta-Hydrolases superfamily protein; INVOLVED IN: biological_process unknown; LOCATED IN: chloroplast; EXPRESSED IN: 18 plant structures; EXPRESSED DURING: 8 growth stages; CONTAINS InterPro DOMAIN/s: Protein of unknown function DUF1749 (InterPro:IPR013744)
AT4G10360	*-0,938*		TRAM, LAG1 and CLN8 (TLC) lipid-sensing domain containing protein; FUNCTIONS IN: molecular_function unknown; INVOLVED IN: biological_process unknown; LOCATED IN: integral to membrane; EXPRESSED IN: 24 plant structures; EXPRESSED DURING: 15 growth stages; CONTAINS InterPro DOMAIN/s: TRAM/LAG1/CLN8 homology domain (InterPro:IPR006634); BEST Arabidopsis thaliana protein match is: TRAM, LAG1 and CLN8 (TLC) lipid-sensing domain containing protein (TAIR:AT1G31300.2)
AT2G21560	*-0,939*		unknown protein; BEST Arabidopsis thaliana protein match is: unknown protein (TAIR:AT4G39190.1)
AT3G20960	*-0,941*	CYTOCHROME P450, FAMILY 705, SUBFAMILY A, POLYPEPTIDE 33 (CYP705A33)	member of CYP705A

AT3G61830	*-0,941*	AUXIN RESPONSE FACTOR 18 (ARF18)	auxin response factor 18 (ARF18); FUNCTIONS IN: sequence-specific DNA binding transcription factor activity; INVOLVED IN: response to hormone stimulus, regulation of transcription, DNA-dependent, regulation of transcription; LOCATED IN: nucleus; EXPRESSED IN: 22 plant structures; EXPRESSED DURING: 13 growth stages; CONTAINS InterPro DOMAIN/s: Aux/IAA-ARF-dimerisation (InterPro:IPR011525), Transcriptional factor B3 (InterPro:IPR003340), AUX/IAA protein (InterPro:IPR003311), Auxin response factor (InterPro:IPR010525); BEST Arabidopsis thaliana protein match is: auxin response factor 11 (TAIR:AT2G46530.1)
AT1G77580	*-0,942*	VESICLE TETHERING 3 (VETH3)	Plant protein of unknown function (DUF869); CONTAINS InterPro DOMAIN/s: Protein of unknown function DUF869, plant (InterPro:IPR008587); BEST Arabidopsis thaliana protein match is: Plant protein of unknown function (DUF869) (TAIR:AT1G21810.1)
AT4G21830	*-0,942*	METHIONINE SULFOXIDE REDUCTASE B7 (MSRB7)	methionine sulfoxide reductase B7 (MSRB7); CONTAINS InterPro DOMAIN/s: Methionine sulphoxide reductase B (InterPro:IPR002579), Mss4-like (InterPro:IPR011057); BEST Arabidopsis thaliana protein match is: methionine sulfoxide reductase B8 (TAIR:AT4G21840.1)
AT3G03000	*-0,944*		Calmodulin like protein localized in the plant vacuolar compartment with a function of binding and modifying the activity of a tonoplast transporter (AtNHX1) from within the vacuole in a Ca+2- and pH-dependent manner
AT4G11300	*-0,944*		CONTAINS InterPro DOMAIN/s: Protein BYPASS related (InterPro:IPR008511); BEST Arabidopsis thaliana protein match is: Protein of unknown function (DUF793) (TAIR:AT4G23530.1)
AT4G39090	*-0,944*	RESPONSIVE TO DEHYDRATION 19 (RD19)	Similar to cysteine proteinases, induced by desiccation but not abscisic acid. Required for RRS1-R mediated resistance against Ralstonia solanacearum. Interacts with the R. solanacearum type III effector PopP2. RD19 associates with PopP2 to form a nuclear complex that is required for activation of the RRS1-R?mediated resistance response.
AT5G11610	*-0,944*		Exostosin family protein; FUNCTIONS IN: catalytic activity; INVOLVED IN: biological_process unknown; LOCATED IN: endomembrane system, membrane; EXPRESSED IN: 6 plant structures; EXPRESSED DURING: LP.06 six leaves visible, LP.04 four leaves visible, LP.10 ten leaves visible, 4 leaf senescence stage; CONTAINS InterPro DOMAIN/s: Exostosin-like (InterPro:IPR004263); BEST Arabidopsis thaliana protein match is: Exostosin family protein (TAIR:AT5G25820.1)
AT5G52170	*-0,944*	HOMEODOMAIN GLABROUS 7 (HDG7)	Encodes a homeobox-leucine zipper family protein belonging to the HD-ZIP IV family.
AT2G15570	*-0,945*	(ATHM3)	chloroplast protein similar to prokaryotic thioredoxin.
AT5G41810	*-0,945*		unknown protein; BEST Arabidopsis thaliana protein match is: unknown protein (TAIR:AT1G64340.1)
AT1G70782	*-0,946*	CONSERVED PEPTIDE UPSTREAM OPEN READING FRAME 28 (CPuORF28)	Upstream open reading frames (uORFs) are small open reading frames found in the 5' UTR of a mature mRNA, and can potentially mediate translational regulation of the largest, or major, ORF (mORF). CPuORF28 represents a conserved upstream opening reading frame relative to major ORF AT1G70780.1
AT4G04990	*-0,946*		Protein of unknown function (DUF761); FUNCTIONS IN: molecular_function unknown; INVOLVED IN: biological_process unknown; LOCATED IN: endomembrane system; CONTAINS InterPro DOMAIN/s: Protein of unknown function DUF761, plant (InterPro:IPR008480); BEST Arabidopsis thaliana protein match is: Protein of unknown function (DUF761) (TAIR:AT1G61260.1)
AT5G02420	*-0,947*	(SMR3)	unknown protein
AT1G65180	*-0,948*		Cysteine/Histidine-rich C1 domain family protein; FUNCTIONS IN: zinc ion binding; INVOLVED IN: intracellular signaling pathway; LOCATED IN: intracellular; EXPRESSED IN: shoot apex, root, pedicel; EXPRESSED DURING: 4 anthesis; CONTAINS InterPro DOMAIN/s: Protein kinase C-like, phorbol ester/diacylglycerol binding (InterPro:IPR002219), DC1 (InterPro:IPR004146), Zinc finger, PHD-type (InterPro:IPR001965), Zinc finger, C2H2-type (InterPro:IPR007087), C1-like (InterPro:IPR011424); BEST Arabidopsis thaliana protein match is: Cysteine/Histidine-rich C1 domain family protein (TAIR:AT3G43890.1)
AT4G34500	*-0,948*		Protein kinase superfamily protein; FUNCTIONS IN: protein serine/threonine kinase activity, protein kinase activity, kinase activity, ATP binding; INVOLVED IN: protein amino acid phosphorylation; LOCATED IN: cellular_component unknown; EXPRESSED IN: 22 plant structures; EXPRESSED DURING: 13 growth stages; CONTAINS InterPro DOMAIN/s: Protein kinase, ATP binding site (InterPro:IPR017441), Protein kinase, catalytic domain (InterPro:IPR000719), Serine-threonine/tyrosine-protein kinase (InterPro:IPR001245), Protein kinase-like domain (InterPro:IPR011009), Serine/threonine-protein kinase, active site (InterPro:IPR008271); BEST Arabidopsis thaliana protein match is: Protein kinase superfamily protein (TAIR:AT1G01540.2)

AT5G02090	*-0,949*		unknown protein
AT5G49780	*-0,949*		Leucine-rich repeat protein kinase family protein; FUNCTIONS IN: protein serine/threonine kinase activity, kinase activity, ATP binding; INVOLVED IN: transmembrane receptor protein tyrosine kinase signaling pathway, protein amino acid phosphorylation; EXPRESSED IN: shoot, hypocotyl, root, flower; EXPRESSED DURING: petal differentiation and expansion stage; CONTAINS InterPro DOMAIN/s: Protein kinase, ATP binding site (InterPro:IPR017441), Serine/threonine-protein kinase domain (InterPro:IPR002290), Leucine-rich repeat (InterPro:IPR001611), Serine/threonine-protein kinase-like domain (InterPro:IPR017442), Protein kinase-like domain (InterPro:IPR011009), Serine/threonine-protein kinase, active site (InterPro:IPR008271), Protein kinase, catalytic domain (InterPro:IPR000719), Tyrosine-protein kinase, catalytic domain (InterPro:IPR020635); BEST Arabidopsis thaliana protein match is: Leucine-rich repeat protein kinase family protein (TAIR:AT5G49760.1)
AT4G30140	*-0,951*	CUTICLE DESTRUCTING FACTOR 1 (CDEF1)	Member of the GDSL lipase/esterase family of proteins that functions as cutinase. Expressed in pollen and at the zone of lateral root emergence.
AT4G35160	*-0,953*	N-ACETYLSEROTONIN O-METHYLTRANSFERASE (ASMT)	Encodes a cytosolic N-acetylserotonin O-methyltransferase that can convert N-acetylserotonin to melatonin and serotonin to 5-methoxytryptamine in the process of melatonin synthesis. It does not have caffeic acid O- methyltransferase activity.
AT4G11360	*-0,954*	RING-H2 FINGER A1B (RHA1B)	Encodes a putative RING-H2 finger protein RHA1b. The mRNA is cell-to-cell mobile.
AT1G55450	*-0,955*		S-adenosyl-L-methionine-dependent methyltransferases superfamily protein; FUNCTIONS IN: methyltransferase activity; EXPRESSED IN: 20 plant structures; EXPRESSED DURING: 13 growth stages; CONTAINS InterPro DOMAIN/s: Methyltransferase type 11 (InterPro:IPR013216); BEST Arabidopsis thaliana protein match is: S-adenosyl-L-methionine-dependent methyltransferases superfamily protein (TAIR:AT3G54150.1).
AT5G62720	*-0,955*		Integral membrane HPP family protein; FUNCTIONS IN: molecular_function unknown; INVOLVED IN: biological_process unknown; LOCATED IN: integral to membrane, chloroplast inner membrane, chloroplast envelope; EXPRESSED IN: 22 plant structures; EXPRESSED DURING: 13 growth stages; CONTAINS InterPro DOMAIN/s: HPP (InterPro:IPR007065); BEST Arabidopsis thaliana protein match is: Integral membrane HPP family protein (TAIR:AT3G47980.1)
AT5G24780	*-0,956*	VEGETATIVE STORAGE PROTEIN 1 (VSP1)	encodes an acid phosphatase similar to soybean vegetative storage proteins. Gene expression is induced by wounding and jasmonic acid.
AT5G66600	*-0,956*		Protein of unknown function, DUF547; FUNCTIONS IN: molecular_function unknown; INVOLVED IN: biological_process unknown; LOCATED IN: cellular_component unknown; CONTAINS InterPro DOMAIN/s: Protein of unknown function DUF547 (InterPro:IPR006869); BEST Arabidopsis thaliana protein match is: Protein of unknown function, DUF547 (TAIR:AT2G23700.1).
AT1G35250	*-0,957*	ACYL-LIPID THIOESTERASE 2 (ALT2)	Thioesterase superfamily protein; CONTAINS InterPro DOMAIN/s: Thioesterase superfamily (InterPro:IPR006683); BEST Arabidopsis thaliana protein match is: Thioesterase superfamily protein (TAIR:AT1G35290.1)
AT4G25160	*-0,957*	(PUB35)	Encodes a U-box domain-containing E3 ubiquitin ligase with central Ser/Thr protein kinase domain whose expression is responsive to both phosphate (Pi) and phosphite (Phi) in both roots and shoots.
AT1G13110	*-0,959*	CYTOCHROME P450, FAMILY 71 SUBFAMILY B, POLYPEPTIDE 7 (CYP71B7)	member of CYP71B The mRNA is cell-to-cell mobile.
AT1G77130	*-0,959*	PLANT GLYCOGENIN-LIKE STARCH INITIATION PROTEIN 2 (PGSIP2)	Encodes a glucuronyltransferase responsible for the addition of GlcA residues onto xylan and for secondary wall deposition.
AT2G03730	*-0,959*	ACT DOMAIN REPEAT 5 (ACR5)	Member of a small family of ACT domain containing proteins. ACT domains are thought to be involved in amino acid binding.
AT1G75620	*-0,96*		glyoxal oxidase-related protein; FUNCTIONS IN: molecular_function unknown; INVOLVED IN: biological_process unknown; LOCATED IN: endomembrane system; EXPRESSED IN: root, flower; EXPRESSED DURING: 4 anthesis; CONTAINS InterPro DOMAIN/s: Galactose oxidase/kelch, beta-propeller (InterPro:IPR011043), Galactose oxidase, beta-propeller (InterPro:IPR015916), Immunoglobulin E-set (InterPro:IPR014756), Glyoxal oxidase, N-terminal (InterPro:IPR009880), Domain of unknown function DUF1929 (InterPro:IPR015202); BEST Arabidopsis thaliana protein match is: glyoxal oxidase-related protein (TAIR:AT1G19900.1)

AT2G22330	-0,961	CYTOCHROME P450, FAMILY 79, SUBFAMILY B, POLYPEPTIDE 3 (CYP79B3)	Encodes a cytochrome P450. Involved in tryptophan metabolism. Converts Trp to indole-3-acetaldoxime (IAOx), a precursor to IAA and indole glucosinolates. The mRNA is cell-to-cell mobile.
AT5G04980	-0,961		DNAse I-like superfamily protein; FUNCTIONS IN: hydrolase activity, inositol or phosphatidylinositol phosphatase activity; INVOLVED IN: biological_process unknown; LOCATED IN: cellular_component unknown; EXPRESSED IN: 18 plant structures; EXPRESSED DURING: 8 growth stages; CONTAINS InterPro DOMAIN/s: Inositol polyphosphate related phosphatase (InterPro:IPR000300), Endonuclease/exonuclease/phosphatase (InterPro:IPR005135); BEST Arabidopsis thaliana protein match is: DNAse I-like superfamily protein (TAIR:AT5G65090.1)
AT4G13860	-0,962		RNA-binding (RRM/RBD/RNP motifs) family protein; FUNCTIONS IN: RNA binding, nucleotide binding, nucleic acid binding; INVOLVED IN: biological_process unknown; LOCATED IN: cellular_component unknown; CONTAINS InterPro DOMAIN/s: RNA recognition motif, glycine rich protein (InterPro:IPR015465), RNA recognition motif, RNP-1 (InterPro:IPR000504), Nucleotide-binding, alpha-beta plait (InterPro:IPR012677); BEST Arabidopsis thaliana protein match is: glycine-rich RNA-binding protein 2 (TAIR:AT4G13850.4)
AT5G23020	-0,963	2-ISOPROPYLMALATE SYNTHASE 2 (IMS2)	methylthioalkymalate synthase-like. Also known as 2-isopropylmalate synthase (IMS2). encodes a methylthioalkylmalate synthase involved in the biosynthesis of aliphatic glucosinolates which accepts all the omega-methylthio-2-oxoalkanoic acids needed to form the known C3 to C8 glucosinolates in Arabidopsis. The mRNA is cell-to-cell mobile.
AT1G70170	-0,964	MATRIX METALLOPROTEINASE (MMP)	mutant has Late flowering; Early senescence; Matrix Metalloproteinase
AT2G29680	-0,964	CELL DIVISION CONTROL 6 (CDC6)	Encodes cell division control protein 6 (CDC6).
AT1G09540	-0,966	MYB DOMAIN PROTEIN 61 (MYB61)	Encodes putative transcription factor. Mutants lack of mucilage extrusion from the seeds during imbibition. Reduced quantities of mucilage are deposited during the development of the seed coat epidermis in myb61 mutants. Expressed in guard cells,loss of function mutations show an increase in stomatal pore opening suggesting a role in ABA independent regulation of stomatal pore size.
AT1G61480	-0,966		S-locus lectin protein kinase family protein; FUNCTIONS IN: in 6 functions; INVOLVED IN: protein amino acid phosphorylation, recognition of pollen; LOCATED IN: endomembrane system; EXPRESSED IN: root; CONTAINS InterPro DOMAIN/s: Curculin-like (mannose-binding) lectin (InterPro:IPR001480), Apple-like (InterPro:IPR003609), Protein kinase, ATP binding site (InterPro:IPR017441), PAN-2 domain (InterPro:IPR013227), S-locus receptor kinase, C-terminal (InterPro:IPR021820), Serine/threonine-protein kinase domain (InterPro:IPR002290), Serine-threonine/tyrosine-protein kinase (InterPro:IPR001245), Protein kinase-like domain (InterPro:IPR011009), Serine/threonine-protein kinase, active site (InterPro:IPR008271), Protein kinase, catalytic domain (InterPro:IPR000719), S-locus glycoprotein (InterPro:IPR000858), Tyrosine-protein kinase, catalytic domain (InterPro:IPR020635); BEST Arabidopsis thaliana protein match is: S-locus lectin protein kinase family protein (TAIR:AT1G61490.1)
AT1G71870	-0,966	(BIGE1A)	MATE efflux family protein; FUNCTIONS IN: antiporter activity, drug transmembrane transporter activity, transporter activity; INVOLVED IN: drug transmembrane transport, transmembrane transport; LOCATED IN: membrane; EXPRESSED IN: 16 plant structures; EXPRESSED DURING: 6 growth stages; CONTAINS InterPro DOMAIN/s: Multi antimicrobial extrusion protein MatE (InterPro:IPR002528); BEST Arabidopsis thaliana protein match is: MATE efflux family protein (TAIR:AT4G29140.1)
AT1G01620	-0,968	PLASMA MEMBRANE INTRINSIC PROTEIN 1C (PIP1C)	a member of the plasma membrane intrinsic protein subfamily PIP1. localizes to the plasma membrane and exhibits water transport activity in Xenopus oocyte. expressed ubiquitously and protein level decreases slightly during leaf development.
AT2G39780	-0,968	RIBONUCLEASE 2 (RNS2)	Encodes the main endoribonuclease activity in plant cells and localizes to the endoplasmic reticulum (ER), ER-derived structures, and vacuoles. It is essential for normal ribosomal RNA recycling. The mRNA is cell-to-cell mobile.

AT3G09960	*-0,968*		Calcineurin-like metallo-phosphoesterase superfamily protein; FUNCTIONS IN: hydrolase activity, protein serine/threonine phosphatase activity; INVOLVED IN: biological_process unknown; LOCATED IN: cellular_component unknown; EXPRESSED IN: 8 plant structures; EXPRESSED DURING: 6 growth stages; CONTAINS InterPro DOMAIN/s: Metallophosphoesterase (InterPro:IPR004843), Serine/threonine-specific protein phosphatase/bis(5-nucleosyl)-tetraphosphatase (InterPro:IPR006186); BEST Arabidopsis thaliana protein match is: Calcineurin-like metallo-phosphoesterase superfamily protein (TAIR:AT3G09970.1)
AT5G21170	*-0,968*	(AKINBETA1)	Encodes AKINbeta1, a subunit of the SnRK1 kinase (Sucrose non-fermenting-1-related protein kinase). Involved in regulation of nitrogen and sugar metabolism.
AT3G23830	*-0,969*	RNA-BINDING GLYCINE-RICH PROTEIN A4 (RBGA4)	encodes a glycine-rich RNA binding protein. Gene expression is induced by cold and reduced by ionic (salt) and non-ionic (mannitol) osmotic stress. Lines overexpressing the gene are slightly more tolerant to osmotic stress during germination.
AT4G25070	*-0,969*		unknown protein; EXPRESSED IN: cultured cell; BEST Arabidopsis thaliana protein match is: unknown protein (TAIR:AT3G48860.2)
AT5G11260	*-0,969*	ELONGATED HYPOCOTYL 5 (HY5)	Basic leucine zipper (bZIP) transcription factor. Nuclear localization. Involved in light-regulated transcriptional activation of G-box-containing promoters. Negatively regulated by Cop1. Although cytokinins do not appear to affect the gene's promoter activity, they appear to stabilize the protein. HY5 plays a role in anthocyanin accumulation in far-red light and blue light, but not in red light or in the dark. Mutant studies showed that the gene product is involved in the positive regulation of the PHYA-mediated inhibition of hypocotyl elongation. Binds to G- and Z-boxes, and other ACEs, but not to E-box. Loss of function mutation shows ABA resistant seedling phenotypes suggesting involvement for HY5 in mediating ABA responses. Binds to the promoter of ABI5 and regulates its expression.
AT1G18860	*-0,97*	WRKY DNA-BINDING PROTEIN 61 (WRKY61)	member of WRKY Transcription Factor; Group II-b
AT1G09560	*-0,971*	GERMIN-LIKE PROTEIN 5 (GLP5)	Encodes a plasodesmata-located protein involved in regulating primary root growth by controlling phloem-mediated allocation of resources between the primary and lateral root meristems. The mRNA is cell-to-cell mobile.
AT2G29340	*-0,971*		NAD-dependent epimerase/dehydratase family protein; FUNCTIONS IN: oxidoreductase activity, binding, catalytic activity; INVOLVED IN: oxidation reduction, metabolic process; EXPRESSED IN: 22 plant structures; EXPRESSED DURING: 13 growth stages; CONTAINS InterPro DOMAIN/s: NAD(P)-binding domain (InterPro:IPR016040), Glucose/ribitol dehydrogenase (InterPro:IPR002347), Short-chain dehydrogenase/reductase SDR (InterPro:IPR002198); BEST Arabidopsis thaliana protein match is: NAD(P)-binding Rossmann-fold superfamily protein (TAIR:AT2G29320.1)
AT2G48140	*-0,971*	EMBRYO SAC DEVELOPMENT ARREST 4 (EDA4)	embryo sac development arrest 4 (EDA4); CONTAINS InterPro DOMAIN/s: Bifunctional inhibitor/plant lipid transfer protein/seed storage (InterPro:IPR016140), Plant lipid transfer protein/seed storage/trypsin-alpha amylase inhibitor (InterPro:IPR003612); BEST Arabidopsis thaliana protein match is: Bifunctional inhibitor/lipid-transfer protein/seed storage 2S albumin superfamily protein (TAIR:AT1G05450.2)
AT5G15530	*-0,971*	BIOTIN CARBOXYL CARRIER PROTEIN 2 (BCCP2)	biotin carboxyl carrier protein isoform 2 (BCCP2) mRNA,
AT3G09260	*-0,972*	(PYK10)	Encodes beta-glucosidase.The major constituent of ER bodies. One of the most abundant proteins in Arabidopsis seedlings. Exist in an soluble (inactive) and non-soluble (active) form, most probably formed in a polymerization process. Involved in the mutualistic interaction between Arabidopsis and the endophytic fungus Piriformospora indica.
AT3G24540	*-0,972*	PROLINE-RICH EXTENSIN-LIKE RECEPTOR KINASE 3 (PERK3)	Encodes a member of the proline-rich extensin-like receptor kinase (PERK) family. This family consists of 15 predicted receptor kinases (PMID: 15653807).
AT5G37790	*-0,972*		Protein kinase superfamily protein; FUNCTIONS IN: protein serine/threonine kinase activity, protein kinase activity, kinase activity, ATP binding; INVOLVED IN: protein amino acid phosphorylation; LOCATED IN: cellular_component unknown; EXPRESSED IN: 21 plant structures; EXPRESSED DURING: 7 growth stages; CONTAINS InterPro DOMAIN/s: Protein kinase, catalytic domain (InterPro:IPR000719), Serine/threonine-protein kinase-like domain (InterPro:IPR017442), Protein kinase-like domain (InterPro:IPR011009), Serine/threonine-protein kinase, active site (InterPro:IPR008271); BEST Arabidopsis thaliana protein match is: Protein kinase superfamily protein (TAIR:AT1G66460.1)

AT1G50520	*-0,973*	CYTOCHROME P450, FAMILY 705, SUBFAMILY A, POLYPEPTIDE 27 (CYP705A27)	member of CYP705A The mRNA is cell-to-cell mobile.
AT4G14980	*-0,974*		Cysteine/Histidine-rich C1 domain family protein; INVOLVED IN: intracellular signaling pathway; EXPRESSED IN: shoot apex, root, seed; EXPRESSED DURING: E expanded cotyledon stage; CONTAINS InterPro DOMAIN/s: Protein kinase C-like, phorbol ester/diacylglycerol binding (InterPro:IPR002219), DC1 (InterPro:IPR004146), C1-like (InterPro:IPR011424); BEST Arabidopsis thaliana protein match is: Cysteine/Histidine-rich C1 domain family protein (TAIR:AT5G54050.1)
AT5G39320	*-0,974*	UDP-GLUCOSE DEHYDROGENASE 4 (UDG4)	UDP-glucose 6-dehydrogenase family protein; FUNCTIONS IN: in 6 functions; INVOLVED IN: oxidation reduction, metabolic process; LOCATED IN: cytosol, nucleus; EXPRESSED IN: 24 plant structures; EXPRESSED DURING: 15 growth stages; CONTAINS InterPro DOMAIN/s: UDP-glucose/GDP-mannose dehydrogenase, N-terminal (InterPro:IPR001732), 6-phosphogluconate dehydrogenase, C-terminal-like (InterPro:IPR008927), UDP-glucose/GDP-mannose dehydrogenase, dimerisation and substrate-binding domain (InterPro:IPR014028), UDP-glucose/GDP-mannose dehydrogenase, C-terminal (InterPro:IPR014027), NAD(P)-binding domain (InterPro:IPR016040), UDP-glucose/GDP-mannose dehydrogenase, dimerisation (InterPro:IPR014026), Nucleotide sugar dehydrogenase (InterPro:IPR017476); BEST Arabidopsis thaliana protein match is: UDP-glucose 6-dehydrogenase family protein (TAIR:AT3G29360.2)
AT5G59940	*-0,974*		Cysteine/Histidine-rich C1 domain family protein; FUNCTIONS IN: zinc ion binding; INVOLVED IN: intracellular signaling pathway; LOCATED IN: cellular_component unknown; EXPRESSED IN: 13 plant structures; EXPRESSED DURING: LP.04 four leaves visible, 4 anthesis, petal differentiation and expansion stage, D bilateral stage; CONTAINS InterPro DOMAIN/s: Protein kinase C-like, phorbol ester/diacylglycerol binding (InterPro:IPR002219), DC1 (InterPro:IPR004146), Zinc finger, PHD-type (InterPro:IPR001965), C1-like (InterPro:IPR011424); BEST Arabidopsis thaliana protein match is: Cysteine/Histidine-rich C1 domain family protein (TAIR:AT5G59930.1)
AT2G45170	*-0,975*	AUTOPHAGY 8E (ATG8E)	Involved in autophagy. Under nutrient starvation the protein localizes to autophagosomes.
AT1G53470	*-0,976*	MECHANOSENSITIVE CHANNEL OF SMALL CONDUCTANCE-LIKE 4 (MSL4)	mechanosensitive channel of small conductance-like 4 (MSL4); INVOLVED IN: transmembrane transport; LOCATED IN: plasma membrane, membrane; CONTAINS InterPro DOMAIN/s: Membrane protein, At2g17000, predicted (InterPro:IPR016688), Mechanosensitive ion channel MscS (InterPro:IPR006685), Like-Sm ribonucleoprotein (LSM)-related domain (InterPro:IPR010920); BEST Arabidopsis thaliana protein match is: mechanosensitive channel of small conductance-like 5 (TAIR:AT3G14810.1)
AT3G13710	*-0,976*	PRENYLATED RAB ACCEPTOR 1.F4 (PRA1.F4)	prenylated RAB acceptor 1.F4 (PRA1.F4); CONTAINS InterPro DOMAIN/s: Prenylated rab acceptor PRA1 (InterPro:IPR004895); BEST Arabidopsis thaliana protein match is: PRA1 (Prenylated rab acceptor) family protein (TAIR:AT3G13720.1)
AT5G65390	*-0,977*	ARABINOGALACTAN PROTEIN 7 (AGP7)	arabinogalactan protein 7 (AGP7); BEST Arabidopsis thaliana protein match is: arabinogalactan protein 4 (TAIR:AT5G10430.1)
AT5G54020	*-0,978*		Cysteine/Histidine-rich C1 domain family protein; FUNCTIONS IN: zinc ion binding; INVOLVED IN: intracellular signaling pathway; LOCATED IN: intracellular; EXPRESSED IN: root; CONTAINS InterPro DOMAIN/s: Zinc finger, C2H2-like (InterPro:IPR015880), Protein kinase C-like, phorbol ester/diacylglycerol binding (InterPro:IPR002219), DC1 (InterPro:IPR004146), C1-like (InterPro:IPR011424); BEST Arabidopsis thaliana protein match is: Cysteine/Histidine-rich C1 domain family protein (TAIR:AT5G54050.1)
AT1G49000	*-0,979*		unknown protein; FUNCTIONS IN: molecular_function unknown; INVOLVED IN: biological_process unknown; LOCATED IN: chloroplast; EXPRESSED IN: stem; BEST Arabidopsis thaliana protein match is: unknown protein (TAIR:AT3G18560.1)
AT2G29750	*-0,979*	UDP-GLUCOSYL TRANSFERASE 71C1 (UGT71C1)	UDP-glucosyl transferase 71C1 (UGT71C1); FUNCTIONS IN: quercetin 3'-O-glucosyltransferase activity, UDP-glycosyltransferase activity, quercetin 7-O-glucosyltransferase activity, UDP-glucosyltransferase activity, transferase activity, transferring glycosyl groups; INVOLVED IN: metabolic process; LOCATED IN: cellular_component unknown; EXPRESSED IN: inflorescence meristem, hypocotyl, root; CONTAINS InterPro DOMAIN/s: UDP-glucuronosyl/UDP-glucosyltransferase (InterPro:IPR002213); BEST Arabidopsis thaliana protein match is: UDP-glucosyl transferase 71C2 (TAIR:AT2G29740.1)

AT2G37678	*-0,979*	FAR-RED ELONGATED HYPOCOTYL 1 (FHY1)	Positive regulator of photomorphogenesis in far-red light. Most abundant in young seedlings in the dark. Downregulated in the light and older as plants develop. Localized in the nucleus and the cytoplasm. Nuclear localization strongest in the dark. Degraded through the 26S proteasome. Regulated by PHYA. It is specifically required for the light-regulated nuclear accumulation of phyA but not phyB.
AT4G38080	*-0,979*		hydroxyproline-rich glycoprotein family protein; FUNCTIONS IN: molecular_function unknown; INVOLVED IN: biological_process unknown; LOCATED IN: endomembrane system; EXPRESSED IN: embryo, hypocotyl, root; EXPRESSED DURING: C globular stage; BEST Arabidopsis thaliana protein match is: hydroxyproline-rich glycoprotein family protein (TAIR:AT2G22510.1)
AT1G72300	*-0,98*	PSY1 RECEPTOR (PSY1R)	Encodes a leucine-rich repeat receptor kinase (LRR-RK) involved in the perception of PSY1. PSY1 is an 18-aa tyrosine-sulfated glycopeptide encoded by AT5G58650 that promotes cellular proliferation and expansion.
AT5G04730	*-0,98*		Ankyrin-repeat containing protein; BEST Arabidopsis thaliana protein match is: Ankyrin repeat family protein (TAIR:AT5G04700.1)
AT5G64000	*-0,98*	(SAL2)	3'(2'),5'-bisphosphate nucleotidase
AT4G16620	*-0,981*	USUALLY MULTIPLE ACIDS MOVE IN AND OUT TRANSPORTERS 8 (UMAMIT8)	nodulin MtN21-like transporter family protein
AT5G65530	*-0,982*	ARABIDOPSIS RECEPTOR-LIKE CYTOPLASMIC KINASE ATRLCK VI_A3 (ATRLCK VI_A3)	Encodes a protein kinase involved in mediating resistance to fungi and also trichome branch number. Kinase activity is increased by ROP6 which also affects its sub-cellular localization (becomes localized to the cell periphery_
AT1G23140	*-0,984*	C2-DOMAIN ABA-RELATED8 (CAR8)	Expression is upregulated in the shoot of cax1/cax3 mutant.
AT4G38550	*-0,984*		Arabidopsis phospholipase-like protein (PEARLI 4) family; LOCATED IN: chloroplast; EXPRESSED IN: 21 plant structures; EXPRESSED DURING: 13 growth stages; CONTAINS InterPro DOMAIN/s: Phospholipase-like, arabidopsis (InterPro:IPR007942); BEST Arabidopsis thaliana protein match is: Arabidopsis phospholipase-like protein (PEARLI 4) family (TAIR:AT2G20950.1)
AT3G16030	*-0,985*	CALLUS EXPRESSION OF RBCS 101 (CES101)	CALLUS EXPRESSION OF RBCS 101 (CES101); FUNCTIONS IN: in 6 functions; INVOLVED IN: protein amino acid phosphorylation; LOCATED IN: endomembrane system; EXPRESSED IN: 21 plant structures; EXPRESSED DURING: 13 growth stages; CONTAINS InterPro DOMAIN/s: Curculin-like (mannose-binding) lectin (InterPro:IPR001480), PAN-2 domain (InterPro:IPR013227), Apple-like (InterPro:IPR003609), Serine/threonine-protein kinase domain (InterPro:IPR002290), Serine/threonine-protein kinase-like domain (InterPro:IPR017442), Protein kinase-like domain (InterPro:IPR011009), Serine/threonine-protein kinase, active site (InterPro:IPR008271), Protein kinase, catalytic domain (InterPro:IPR000719), Tyrosine-protein kinase, catalytic domain (InterPro:IPR020635); BEST Arabidopsis thaliana protein match is: S-locus lectin protein kinase family protein (TAIR:AT4G21390.1)
AT5G05410	*-0,985*	DRE-BINDING PROTEIN 2A (DREB2A)	Encodes a transcription factor that specifically binds to DRE/CRT cis elements (responsive to drought and low-temperature stress). Belongs to the DREB subfamily A-2 of ERF/AP2 transcription factor family (DREB2A). There are eight members in this subfamily including DREB2B. The protein contains one AP2 domain. Overexpression of transcriptional activation domain of DREB2A resulted in significant drought stress tolerance but only slight freezing tolerance in transgenic Arabidopsis plants. Microarray and RNA gel blot analyses revealed that DREB2A regulates expression of many water stress?inducible genes. The mRNA is cell-to-cell mobile.
AT5G08240	*-0,985*		unknown protein; BEST Arabidopsis thaliana protein match is: unknown protein (TAIR:AT5G23160.1)
AT1G63530	*-0,986*		BEST Arabidopsis thaliana protein match is: hydroxyproline-rich glycoprotein family protein (TAIR:AT1G63540.1)
AT2G24240	*-0,986*		BTB/POZ domain with WD40/YVTN repeat-like protein; FUNCTIONS IN: voltage-gated potassium channel activity; INVOLVED IN: potassium ion transport; LOCATED IN: voltage-gated potassium channel complex, membrane; EXPRESSED IN: 17 plant structures; EXPRESSED DURING: 11 growth stages; CONTAINS InterPro DOMAIN/s: WD40 repeat-like-containing domain (InterPro:IPR011046), BTB/POZ fold (InterPro:IPR011333), Potassium channel, voltage dependent, Kv, tetramerisation (InterPro:IPR003131), BTB/POZ-like (InterPro:IPR000210); BEST Arabidopsis thaliana protein match is: BTB/POZ domain with WD40/YVTN repeat-like protein (TAIR:AT4G30940.1)

AT1G55240	*-0,987*		Family of unknown function (DUF716) ; FUNCTIONS IN: molecular_function unknown; INVOLVED IN: biological_process unknown; LOCATED IN: endomembrane system; EXPRESSED IN: hypocotyl, root, flower; EXPRESSED DURING: 4 anthesis; CONTAINS InterPro DOMAIN/s: Protein of unknown function DUF716 (InterPro:IPR006904); BEST Arabidopsis thaliana protein match is: Family of unknown function (DUF716) (TAIR:AT1G55230.1)
AT1G78320	*-0,987*	GLUTATHIONE S-TRANSFERASE TAU 23	Encodes glutathione transferase belonging to the tau class of GSTs. Naming convention according to Wagner et al. (2002).
AT3G10500	*-0,987*	NAC DOMAIN CONTAINING PROTEIN 53 (NAC053)	Encodes a transcriptional activator that is associated with the plasma membrane in a dormant form and is proteolytically cleaved to create a form that can enter the nucleus. It is thought to promote ROS production by binding directly to the promoters of genes encoding ROS biosynthetic enzymes during drought-induced leaf senescence. The mRNA is cell-to-cell mobile.
AT4G32870	*-0,987*		Polyketide cyclase/dehydrase and lipid transport superfamily protein; CONTAINS InterPro DOMAIN/s: Polyketide cyclase/dehydrase (InterPro:IPR019587); BEST Arabidopsis thaliana protein match is: Polyketide cyclase/dehydrase and lipid transport superfamily protein (TAIR:AT2G25770.2)
AT2G41800	*-0,988*	TEEBE, LONG IN THE MAYO-YOREME LANGUAGE (TEB)	Encodes a DUF642 cell wall protein that is highly induced during the M/G1 phases of the cell cycle and is involved in hypocotyl cell elongation.
AT4G20390	*-0,988*	CASP-LIKE PROTEIN 1B2 (CASPL1B2)	Uncharacterised protein family (UPF0497); CONTAINS InterPro DOMAIN/s: Uncharacterised protein family UPF0497, trans-membrane plant (InterPro:IPR006702), Uncharacterised protein family UPF0497, trans-membrane plant subgroup (InterPro:IPR006459); BEST Arabidopsis thaliana protein match is: Uncharacterised protein family (UPF0497) (TAIR:AT5G44550.1)
AT2G25000	*-0,989*	WRKY DNA-BINDING PROTEIN 60 (WRKY60)	Pathogen-induced transcription factor. Forms protein complexes with itself and with WRKY40. Coexpression with WRKY18 or WRKY40 made plants more susceptible to both P. syringae and B. cinerea. WRKY18, WRKY40, and WRKY60 have partially redundant roles in response to the hemibiotrophic bacterial pathogen Pseudomonas syringae and the necrotrophic fungal pathogen Botrytis cinerea, with WRKY18 playing a more important role than the other two.
AT4G18340	*-0,989*		Glycosyl hydrolase superfamily protein; FUNCTIONS IN: cation binding, hydrolase activity, hydrolyzing O-glycosyl compounds, catalytic activity; INVOLVED IN: carbohydrate metabolic process; LOCATED IN: endomembrane system; EXPRESSED IN: 21 plant structures; EXPRESSED DURING: 13 growth stages; CONTAINS InterPro DOMAIN/s: Glycoside hydrolase, catalytic core (InterPro:IPR017853), Glycoside hydrolase, family 17 (InterPro:IPR000490), Glycoside hydrolase, subgroup, catalytic core (InterPro:IPR013781); BEST Arabidopsis thaliana protein match is: Glycosyl hydrolase superfamily protein (TAIR:AT1G30080.1)
AT1G16930	*-0,99*		F-box/RNI-like/FBD-like domains-containing protein; CONTAINS InterPro DOMAIN/s: FBD (InterPro:IPR013596), F-box domain, cyclin-like (InterPro:IPR001810), F-box domain, Skp2-like (InterPro:IPR022364), FBD-like (InterPro:IPR006566), Leucine-rich repeat 2 (InterPro:IPR013101); BEST Arabidopsis thaliana protein match is: F-box/RNI-like/FBD-like domains-containing protein (TAIR:AT1G80470.1)
AT2G20960	*-0,99*	(pEARLI4)	pEARLI4; CONTAINS InterPro DOMAIN/s: Phospholipase-like, arabidopsis (InterPro:IPR007942); BEST Arabidopsis thaliana protein match is: Arabidopsis phospholipase-like protein (PEARLI 4) family (TAIR:AT4G38560.1)
AT3G44990	*-0,99*	XYLOGLUCAN ENDOTRANSGLUCOSYLASE/HYDROLASE 31 (XTH31)	Encodes a xyloglucan endotransglycosylase/hydrolase. Protein sequence and phylogenetic analysis indicates that this enzyme resides in Group III-A of the XTH family, with high similarity to Tropaeolum majus (nasturtium) xyloglucanase 1 (TmNXG1).Enzyme kinetic analysis indicates predominant xyloglucan endo-hydrolase activity (EC 3.2.1.151) with only limited potential to act as a xyloglucan endo-transglycosylase (EC 2.4.1.207).
AT4G11230	*-0,99*		Riboflavin synthase-like superfamily protein; FUNCTIONS IN: in 6 functions; INVOLVED IN: oxidation reduction; LOCATED IN: integral to membrane, membrane; EXPRESSED IN: root, flower; EXPRESSED DURING: petal differentiation and expansion stage; CONTAINS InterPro DOMAIN/s: Ferredoxin reductase-type FAD-binding domain (InterPro:IPR017927), NADPH oxidase Respiratory burst (InterPro:IPR013623), Ferric reductase, NAD binding (InterPro:IPR013121), Cytochrome b245, heavy chain (InterPro:IPR000778), FAD-binding 8 (InterPro:IPR013112), Riboflavin synthase-like beta-barrel (InterPro:IPR017938), Ferric reductase-like transmembrane component, N-terminal (InterPro:IPR013130); BEST Arabidopsis thaliana protein match is: respiratory burst oxidase protein F (TAIR:AT1G64060.1)

AT5G23210	*-0,99*	SERINE CARBOXYPEPTIDASE-LIKE 34 (SCPL34)	serine carboxypeptidase-like 34 (SCPL34); FUNCTIONS IN: serine-type carboxypeptidase activity; INVOLVED IN: proteolysis; LOCATED IN: vacuole, plant-type cell wall; EXPRESSED IN: 22 plant structures; EXPRESSED DURING: 13 growth stages; CONTAINS InterPro DOMAIN/s: Peptidase S10, serine carboxypeptidase (InterPro:IPR001563), Peptidase S10, serine carboxypeptidase, active site (InterPro:IPR018202); BEST Arabidopsis thaliana protein match is: serine carboxypeptidase-like 35 (TAIR:AT5G08260.1)
AT1G05210	*-0,991*		Transmembrane protein 97, predicted; FUNCTIONS IN: molecular_function unknown; INVOLVED IN: biological_process unknown; LOCATED IN: endomembrane system; EXPRESSED IN: 17 plant structures; EXPRESSED DURING: 11 growth stages; CONTAINS InterPro DOMAIN/s: Transmembrane protein 97, predicted (InterPro:IPR016964); BEST Arabidopsis thaliana protein match is: Transmembrane protein 97, predicted (TAIR:AT2G32380.1)
AT1G32100	*-0,991*	PINORESINOL REDUCTASE 1 (PRR1)	Encodes a pinoresinol reductase involved in lignan biosynthesis. Expressed strongly in roots and less strongly in stems. Shows specificity for pinoresinol and not lariciresinol.
AT1G80180	*-0,991*		Encodes a substrate of the MAPK kinases. Phenotypic analyses of Arabidopsis expressing phosphorylation site mutant forms of At1g80180.1 showed clustered stomata and higher stomatal index in cotyledons expressing the phosphomimetic form of At1g80180.1.
AT3G51910	*-0,991*	HEAT SHOCK TRANSCRIPTION FACTOR A7A (HSFA7A)	member of Heat Stress Transcription Factor (Hsf) family The mRNA is cell-to-cell mobile.
AT5G59520	*-0,991*	ZRT/IRT-LIKE PROTEIN 2 (ZIP2)	encodes a metal ion transporter whose expression is regulated by copper.
AT1G49580	*-0,992*		Calcium-dependent protein kinase (CDPK) family protein; FUNCTIONS IN: in 6 functions; INVOLVED IN: protein amino acid phosphorylation, N-terminal protein myristoylation; LOCATED IN: plasma membrane; EXPRESSED IN: 22 plant structures; EXPRESSED DURING: 13 growth stages; CONTAINS InterPro DOMAIN/s: Protein kinase, ATP binding site (InterPro:IPR017441), Serine/threonine-protein kinase domain (InterPro:IPR002290), EF-hand-like domain (InterPro:IPR011992), Serine/threonine-protein kinase-like domain (InterPro:IPR017442), Protein kinase-like domain (InterPro:IPR011009), Serine/threonine-protein kinase, active site (InterPro:IPR008271), Protein kinase, catalytic domain (InterPro:IPR000719), Calcium-dependent protein kinase (InterPro:IPR020642), Calcium/calmodulin-dependent protein kinase-like (InterPro:IPR020636); BEST Arabidopsis thaliana protein match is: Protein kinase superfamily protein (TAIR:AT3G19100.1)
AT5G16490	*-0,992*	ROP-INTERACTIVE CRIB MOTIF-CONTAINING PROTEIN 4 (RIC4)	encodes a member of a novel protein family that contains contain a CRIB (for Cdc42/Rac-interactive binding) motif required for their specific interaction with GTP-bound Rop1 (plant-specific Rho GTPase). It interacts with Rop1 and is involved in pollen tube growth and function, and exocytosis in the pollen tube tip. Protein most similar to RIC2 (family subgroup V). Gene is expressed in all tissues examined.Interacts with ROP2 during pavement cell morphogenesis and with ROP1 to promote apical F-actin assembly.
AT1G13430	*-0,993*	SULFOTRANSFERASE 4C (ST4C)	Encodes a sulfotransferase. Unlike the related ST4A protein (At2g14920), in vitro experiements show that this enzyme does not act brassinosteroids. ST4C is expressed in the roots and transcript levels rise in response to cytokinin treatment.
AT4G23450	*-0,993*	ABA INSENSITIVE RING PROTEIN 1 (AIRP1)	AtAIRP1 gene encodes a C3H2C3-type RING E3 Ub ligase. It has been shown to be a positive regulator in the Arabidopsis ABA-dependent drought response.
AT5G57800	*-0,993*	ECERIFERUM 3 (CER3)	encodes a transmembrane protein with similarity to the sterol desaturase family at the N-terminus and to the short-chain dehydrogenase/reductase family at the C-terminus. Mutant analyses indicate this protein is involved in cuticle membrane and wax biosynthesis. The mRNA is cell-to-cell mobile.
AT2G36890	*-0,994*	REGULATOR OF AXILLARY MERISTEMS 2 (RAX2)	Putative homolog of the Blind gene in tomato. Together with RAX1 and RAX3 belong to the class R2R3 MYB genes; encoded by the Myb-like transcription factor MYB38, regulates axillary meristem formation. The mRNA is cell-to-cell mobile.
AT5G44680	*-0,994*		DNA glycosylase superfamily protein; FUNCTIONS IN: DNA-3-methyladenine glycosylase I activity, catalytic activity; INVOLVED IN: DNA repair, base-excision repair; EXPRESSED IN: 21 plant structures; EXPRESSED DURING: 13 growth stages; CONTAINS InterPro DOMAIN/s: DNA glycosylase (InterPro:IPR011257), Methyladenine glycosylase (InterPro:IPR005019); BEST Arabidopsis thaliana protein match is: DNA glycosylase superfamily protein (TAIR:AT3G12710.1)

AT5G63500	*-0,994*		Protein of unknown function (DUF 3339); CONTAINS InterPro DOMAIN/s: Protein of unknown function DUF3339 (InterPro:IPR021775); BEST Arabidopsis thaliana protein match is: Protein of unknown function (DUF 3339) (TAIR:AT3G48660.1)
AT1G63650	*-0,995*	ENHANCER OF GLABRA 3 (EGL3)	Mutant has reduced trichomes, anthocyanin, and seed coat mucilage and abnormally patterned stomates. Mutants are defective in jasmonate-induced anthocyanin accumulation. Encodes a bHLH Transcription Factor 1. The protein is functionally redundant with GL3 and TT8 and interacts with TTG1, the myb proteins GL1, PAP1 and 2, CPC and TRY, and it will form heterodimers with GL3. Expression in N (non-hair cell forming) cell layers is negatively regulated by WER. Expression in H cells (hair cell forming) is promoted by CPC/TRY.
AT1G17430	*-0,996*		alpha/beta-Hydrolases superfamily protein; FUNCTIONS IN: hydrolase activity; INVOLVED IN: biological_process unknown; LOCATED IN: endomembrane system; EXPRESSED IN: 24 plant structures; EXPRESSED DURING: 13 growth stages; CONTAINS InterPro DOMAIN/s: Alpha/beta hydrolase fold-1 (InterPro:IPR000073); BEST Arabidopsis thaliana protein match is: alpha/beta-Hydrolases superfamily protein (TAIR:AT1G72620.1)
AT1G32700	*-0,996*		PLATZ transcription factor family protein; CONTAINS InterPro DOMAIN/s: Protein of unknown function DUF597 (InterPro:IPR006734); BEST Arabidopsis thaliana protein match is: PLATZ transcription factor family protein (TAIR:AT4G17900.1)
AT1G71695	*-0,996*		Peroxidase superfamily protein; FUNCTIONS IN: peroxidase activity, heme binding; INVOLVED IN: response to oxidative stress, oxidation reduction; LOCATED IN: cell wall, vacuole, membrane, plant-type cell wall; EXPRESSED IN: 23 plant structures; EXPRESSED DURING: 13 growth stages; CONTAINS InterPro DOMAIN/s: Haem peroxidase (InterPro:IPR010255), Plant peroxidase (InterPro:IPR000823), Peroxidases heam-ligand binding site (InterPro:IPR019793), Haem peroxidase, plant/fungal/bacterial (InterPro:IPR002016), Peroxidase, active site (InterPro:IPR019794); BEST Arabidopsis thaliana protein match is: Peroxidase superfamily protein (TAIR:AT2G18150.1)
AT5G23840	*-0,996*		MD-2-related lipid recognition domain-containing protein; FUNCTIONS IN: molecular_function unknown; INVOLVED IN: biological_process unknown; LOCATED IN: endomembrane system; EXPRESSED IN: root; CONTAINS InterPro DOMAIN/s: MD-2-related lipid-recognition (InterPro:IPR003172); BEST Arabidopsis thaliana protein match is: MD-2-related lipid recognition domain-containing protein (TAIR:AT5G23820.1)
AT5G47740	*-0,996*		Adenine nucleotide alpha hydrolases-like superfamily protein; FUNCTIONS IN: molecular_function unknown; INVOLVED IN: response to stress; LOCATED IN: cellular_component unknown; EXPRESSED IN: root; CONTAINS InterPro DOMAIN/s: UspA (InterPro:IPR006016); BEST Arabidopsis thaliana protein match is: U-box domain-containing protein kinase family protein (TAIR:AT2G45910.1).
AT5G50010	*-0,996*	(SACL2)	sequence-specific DNA binding transcription factors;transcription regulators; BEST Arabidopsis thaliana protein match is: sequence-specific DNA binding transcription factors;transcription regulators (TAIR:AT5G64340.1)
AT1G64000	*-0,998*	WRKY DNA-BINDING PROTEIN 56 (WRKY56)	member of WRKY Transcription Factor; Group II-c
AT2G20670	*-0,998*		Protein of unknown function (DUF506) ; LOCATED IN: chloroplast; EXPRESSED IN: 22 plant structures; EXPRESSED DURING: 14 growth stages; CONTAINS InterPro DOMAIN/s: Protein of unknown function DUF506, plant (InterPro:IPR006502); BEST Arabidopsis thaliana protein match is: Protein of unknown function (DUF506) (TAIR:AT4G32480.1)
AT2G37180	*-0,998*	RESPONSIVE TO DESICCATION 28 (RD28)	a member of the plasma membrane intrinsic protein PIP2. functions as aquaporin and is involved in desiccation.
AT3G47730	*-0,998*	ATP-BINDING CASSETTE A2 (ABCA2)	member of ATH subfamily
AT1G63180	*-1*	UDP-D-GLUCOSE/UDP-D-GALACTOSE 4-EPIMERASE 3 (UGE3)	Encodes a protein with UDP-D-glucose 4-epimerase activity. Involved in pollen development.
AT4G27320	*-1,001*	(PHOS34)	Contains a universal stress protein domain. Protein is phosphorylated in response to Phytophthora infestans zoospores and xylanase.

AT1G08930	*-1,002*	EARLY RESPONSE TO DEHYDRATION 6 (ERD6)	encodes a putative sucrose transporter whose gene expression is induced by dehydration and cold. The mRNA is cell-to-cell mobile.
AT2G02960	*-1,003*		RING/FYVE/PHD zinc finger superfamily protein; FUNCTIONS IN: zinc ion binding; EXPRESSED IN: 24 plant structures; EXPRESSED DURING: 15 growth stages; CONTAINS InterPro DOMAIN/s: Protein of unknown function DUF3675 (InterPro:IPR022143), Zinc finger, C3HC4 RING-type (InterPro:IPR018957), Zinc finger, RING-CH-type (InterPro:IPR011016); BEST Arabidopsis thaliana protein match is: RING/FYVE/PHD zinc finger superfamily protein (TAIR:AT1G14260.2)
AT4G37530	*-1,004*		Peroxidase superfamily protein; FUNCTIONS IN: protein binding, peroxidase activity; INVOLVED IN: response to salt stress; LOCATED IN: endomembrane system; EXPRESSED IN: root; CONTAINS InterPro DOMAIN/s: Haem peroxidase (InterPro:IPR010255), Plant peroxidase (InterPro:IPR000823), Peroxidases heam-ligand binding site (InterPro:IPR019793), Haem peroxidase, plant/fungal/bacterial (InterPro:IPR002016); BEST Arabidopsis thaliana protein match is: Peroxidase superfamily protein (TAIR:AT4G37520.1)
AT3G01350	*-1,007*		Major facilitator superfamily protein; FUNCTIONS IN: transporter activity; INVOLVED IN: oligopeptide transport; LOCATED IN: membrane; EXPRESSED IN: 12 plant structures; EXPRESSED DURING: 6 growth stages; CONTAINS InterPro DOMAIN/s: Oligopeptide transporter (InterPro:IPR000109), Major facilitator superfamily, general substrate transporter (InterPro:IPR016196); BEST Arabidopsis thaliana protein match is: Major facilitator superfamily protein (TAIR:AT5G14940.1)
AT3G02020	*-1,007*	ASPARTATE KINASE 3 (AK3)	encodes a monofunctional aspartate kinase
AT4G19720	*-1,008*		Glycosyl hydrolase family protein with chitinase insertion domain; FUNCTIONS IN: cation binding, chitinase activity, hydrolase activity, hydrolyzing O-glycosyl compounds, catalytic activity; INVOLVED IN: carbohydrate metabolic process; LOCATED IN: cellular_component unknown; EXPRESSED IN: root; CONTAINS InterPro DOMAIN/s: Glycoside hydrolase, family 18, catalytic domain (InterPro:IPR001223), Chitinase II (InterPro:IPR011583), Glycoside hydrolase, catalytic core (InterPro:IPR017853), Glycoside hydrolase, subgroup, catalytic core (InterPro:IPR013781); BEST Arabidopsis thaliana protein match is: Glycosyl hydrolase family protein with chitinase insertion domain (TAIR:AT4G19810.1)
AT2G02950	*-1,009*	PHYTOCHROME KINASE SUBSTRATE 1 (PKS1)	Encodes a basic soluble protein which can independently bind to either PHYA or PHYB, regardless of whether the phytochromes are in the Pr or Pfr state. PKS1 can be phosphorylated by oat phyA in vitro in a light regulated manner. It is postulated to be a negative regulator of phyB signalling.
AT3G10740	*-1,012*	ALPHA-L-ARABINOFURANOSIDASE 1 (ASD1)	Encodes a bifunctional alpha-l-arabinofuranosidase/beta-d-xylosidase that belongs to family 51 of glycoside hydrolases. It may be involved in cell wall modification.
AT2G26710	*-1,013*	PHYB ACTIVATION TAGGED SUPPRESSOR 1 (BAS1)	Encodes a member of the cytochrome p450 family that serves as a control point between multiple photoreceptor systems and brassinosteroid signal transduction. Involved in brassinolide metabolism. Mediates response to a variety of light signals including hypocotyl elongation and cotyledon expansion.
AT2G04430	*-1,014*	NUDIX HYDROLASE HOMOLOG 5 (NUDT5)	nudix hydrolase homolog 5 (NUDT5); FUNCTIONS IN: hydrolase activity; LOCATED IN: cytosol; EXPRESSED IN: 12 plant structures; EXPRESSED DURING: 11 growth stages; CONTAINS InterPro DOMAIN/s: NUDIX hydrolase domain-like (InterPro:IPR015797), NUDIX hydrolase, conserved site (InterPro:IPR020084), Nudix hydrolase 6-like (InterPro:IPR003293), NUDIX hydrolase domain (InterPro:IPR000086); BEST Arabidopsis thaliana protein match is: nudix hydrolase homolog 6 (TAIR:AT2G04450.1)
AT3G23000	*-1,014*	CBL-INTERACTING PROTEIN KINASE 7 (CIPK7)	Encodes a serine/threonine protein kinase with similarities to CBL-interacting protein kinases, SNF1 and SOS2. The mRNA is cell-to-cell mobile.
AT4G01750	*-1,015*	RHAMNOGALACTURONAN XYLOSYLTRANSFERASE 2 (RGXT2)	Encodes a protein with UDP-xylose-dependent xylosyltransferase activity, which transfers Xyl onto L-fucose and (albeit less efficiently) L-arabinose. The linkage to L-fucose was shown to be preferentially to the <i>O</i>-4 position. Analysis of mutant containing T-DNA insertion in this gene indicate that the RGXT2 protein might be involved in the synthesis of the α-D-Xyl-(1,3)-α-L-Fuc-(1,4)-L-Rha structure in pectic rhamnogalacturonan II. The mRNA is cell-to-cell mobile.
AT3G09270	*-1,017*	GLUTATHIONE S-TRANSFERASE TAU 8 (GSTU8)	Encodes glutathione transferase belonging to the tau class of GSTs. Naming convention according to Wagner et al. (2002).

AT1G16170	*-1,018*		unknown protein; FUNCTIONS IN: molecular_function unknown; INVOLVED IN: biological_process unknown; LOCATED IN: cellular_component unknown; EXPRESSED IN: 24 plant structures; EXPRESSED DURING: 15 growth stages; BEST Arabidopsis thaliana protein match is: unknown protein (TAIR:AT1G79660.1)
AT5G41000	*-1,019*	YELLOW STRIPE LIKE 4 (YSL4)	Arabidopsis thaliana metal-nicotianamine transporter YSL4
AT5G25010	*-1,02*		Protein of unknown function (DUF1336); CONTAINS InterPro DOMAIN/s: Protein of unknown function DUF1336 (InterPro:IPR009769); BEST Arabidopsis thaliana protein match is: Protein of unknown function (DUF1336) (TAIR:AT5G24990.1)
AT4G26580	*-1,021*		RING/U-box superfamily protein; FUNCTIONS IN: zinc ion binding; LOCATED IN: chloroplast; CONTAINS InterPro DOMAIN/s: Zinc finger, RING-type (InterPro:IPR001841), Zinc finger, C3HC4 RING-type (InterPro:IPR018957); BEST Arabidopsis thaliana protein match is: RING/U-box superfamily protein (TAIR:AT5G55970.2)
AT5G63410	*-1,021*		Leucine-rich repeat protein kinase family protein; FUNCTIONS IN: protein serine/threonine kinase activity, protein kinase activity, ATP binding; INVOLVED IN: transmembrane receptor protein tyrosine kinase signaling pathway, protein amino acid phosphorylation; LOCATED IN: endomembrane system; EXPRESSED IN: 22 plant structures; EXPRESSED DURING: 13 growth stages; CONTAINS InterPro DOMAIN/s: Protein kinase, catalytic domain (InterPro:IPR000719), Leucine-rich repeat (InterPro:IPR001611), Serine-threonine/tyrosine-protein kinase (InterPro:IPR001245), Protein kinase-like domain (InterPro:IPR011009); BEST Arabidopsis thaliana protein match is: Leucine-rich repeat protein kinase family protein (TAIR:AT5G14210.1)
AT3G14660	*-1,022*	CYTOCHROME P450, FAMILY 72, SUBFAMILY A, POLYPEPTIDE 13 (CYP72A13)	putative cytochrome P450 The mRNA is cell-to-cell mobile.
AT4G01540	*-1,023*	NAC WITH TRANSMEMBRANE MOTIF1 (NTM1)	Encodes a membrane-bound NAC (for NAM, ATAF1/2, CUC2) transcription factor, designated NTM1 (for NAC with transmembrane motif1). NTM1 regulates cell division in Arabidopsis.
AT4G03500	*-1,024*		Ankyrin repeat family protein; CONTAINS InterPro DOMAIN/s: Ankyrin repeat-containing domain (InterPro:IPR020683), Ankyrin repeat (InterPro:IPR002110); BEST Arabidopsis thaliana protein match is: Ankyrin repeat family protein (TAIR:AT4G03460.1)
AT4G17030	*-1,024*	EXPANSIN-LIKE B1 (EXLB1)	Encodes EXLB1 (expansin-like B1), a member of the expansin family.
AT1G07610	*-1,027*	METALLOTHIONEIN 1C (MT1C)	one of the five metallothioneins (MTs) genes identified in Arabidopsis. MTs are cysteine-rich proteins required for heavy metal tolerance. The mRNA is cell-to-cell mobile.
AT1G78000	*-1,027*	SULFATE TRANSPORTER 1;2 (SULTR1;2)	Encodes a sulfate transporter that can restore sulfate uptake capacity of a yeast mutant lacking sulfate transporter genes.
AT4G12690	*-1,027*		Plant protein of unknown function (DUF868); CONTAINS InterPro DOMAIN/s: Protein of unknown function DUF868, plant (InterPro:IPR008586); BEST Arabidopsis thaliana protein match is: Plant protein of unknown function (DUF868) (TAIR:AT2G04220.1)
AT1G35240	*-1,028*	AUXIN RESPONSE FACTOR 20 (ARF20)	auxin response factor 20 (ARF20); FUNCTIONS IN: sequence-specific DNA binding transcription factor activity; INVOLVED IN: regulation of transcription; LOCATED IN: nucleus; CONTAINS InterPro DOMAIN/s: Aux/IAA-ARF-dimerisation (InterPro:IPR011525), Transcriptional factor B3 (InterPro:IPR003340), AUX/IAA protein (InterPro:IPR003311), Auxin response factor (InterPro:IPR010525); BEST Arabidopsis thaliana protein match is: auxin response factor 21 (TAIR:AT1G34410.1)
AT3G23800	*-1,028*	SELENIUM-BINDING PROTEIN 3 (SBP3)	selenium-binding protein 3 (SBP3); FUNCTIONS IN: selenium binding; LOCATED IN: cellular_component unknown; EXPRESSED IN: root; CONTAINS InterPro DOMAIN/s: Cytochrome cd1-nitrite reductase-like, C-terminal haem d1 (InterPro:IPR011048), Selenium-binding protein (InterPro:IPR008826); BEST Arabidopsis thaliana protein match is: selenium-binding protein 1 (TAIR:AT4G14030.2)
AT5G45310	*-1,028*		unknown protein; LOCATED IN: endomembrane system; EXPRESSED IN: stem, inflorescence meristem, root, leaf; EXPRESSED DURING: LP.04 four leaves visible
AT4G37070	*-1,029*	(PLP1)	Patatin-related phospholipase A. Expressed strongly and exclusively in roots. AtplaIVA-null mutants have reduced lateral root development. Phosphorylation by calcium-dependent protein kinases in vitro enhances its activity.

AT5G65320	*-1,031*		basic helix-loop-helix (bHLH) DNA-binding superfamily protein; FUNCTIONS IN: DNA binding, sequence-specific DNA binding transcription factor activity; INVOLVED IN: regulation of transcription; LOCATED IN: nucleus; EXPRESSED IN: hypocotyl, root, leaf; EXPRESSED DURING: LP.04 four leaves visible, LP.10 ten leaves visible, 4 leaf senescence stage; CONTAINS InterPro DOMAIN/s: Helix-loop-helix DNA-binding domain (InterPro:IPR001092), Helix-loop-helix DNA-binding (InterPro:IPR011598); BEST Arabidopsis thaliana protein match is: basic helix-loop-helix (bHLH) DNA-binding superfamily protein (TAIR:AT1G72210.1)
AT1G44160	*-1,032*		HSP40/DnaJ peptide-binding protein; FUNCTIONS IN: unfolded protein binding, heat shock protein binding; INVOLVED IN: protein folding; EXPRESSED IN: 13 plant structures; EXPRESSED DURING: L mature pollen stage, M germinated pollen stage, 4 anthesis, C globular stage, petal differentiation and expansion stage; CONTAINS InterPro DOMAIN/s: Molecular chaperone, heat shock protein, Hsp40, DnaJ (InterPro:IPR015609), HSP40/DnaJ peptide-binding (InterPro:IPR008971), Chaperone DnaJ, C-terminal (InterPro:IPR002939), Heat shock protein DnaJ (InterPro:IPR003095); BEST Arabidopsis thaliana protein match is: HSP40/DnaJ peptide-binding protein (TAIR:AT1G11040.1)
AT4G08450	*-1,032*		Disease resistance protein (TIR-NBS-LRR class) family; FUNCTIONS IN: transmembrane receptor activity, nucleoside-triphosphatase activity, nucleotide binding, ATP binding; INVOLVED IN: signal transduction, apoptosis, defense response, innate immune response; LOCATED IN: intrinsic to membrane; CONTAINS InterPro DOMAIN/s: ATPase, AAA+ type, core (InterPro:IPR003593), NB-ARC (InterPro:IPR002182), Leucine-rich repeat (InterPro:IPR001611), Toll-Interleukin receptor (InterPro:IPR000157), Disease resistance protein (InterPro:IPR000767); BEST Arabidopsis thaliana protein match is: Disease resistance protein (TIR-NBS-LRR class) family (TAIR:AT5G46450.1)
AT1G76090	*-1,033*	STEROL METHYLTRANSFERASE 3	Encodes S-adenosyl-methionine-sterol-C-methyltransferase, an enzyme in the sterol biosynthetic pathway.
AT5G18170	*-1,034*	GLUTAMATE DEHYDROGENASE 1 (GDH1)	Encodes the 43 kDa alpha-subunit of the glutamate dehydrogenase with a putative mitochondrial transit polypeptide and NAD(H)- and alpha-ketoglutarate-binding domains. Mitochondrial localization confirmed by subcellular fractionation. Combines in several ratios with GDH2 protein (GDH-beta) to form seven isoenzymes. Catalyzes the cleavage of glycine residues. May be involved in ammonia assimilation under conditions of inorganic nitrogen excess. The enzyme is almost exclusively found in the mitochondria of stem and leaf companion cells.
AT3G03650	*-1,035*	EMBRYO SAC DEVELOPMENT ARREST 5 (EDA5)	embryo sac development arrest 5 (EDA5); FUNCTIONS IN: catalytic activity; INVOLVED IN: megagametogenesis, pollen tube development; LOCATED IN: membrane; CONTAINS InterPro DOMAIN/s: Exostosin-like (InterPro:IPR004263); BEST Arabidopsis thaliana protein match is: exostosin family protein (TAIR:AT3G45400.1)
AT3G15500	*-1,035*	NAC DOMAIN CONTAINING PROTEIN 3 (NAC3)	Encodes an ATAF-like NAC-domain transcription factor that doesn't contain C-terminal sequences shared by CUC1, CUC2 and NAM. Note: this protein (AtNAC3) is not to be confused with the protein encoded by locus AT3G29035, which, on occasion, has also been referred to as AtNAC3. The mRNA is cell-to-cell mobile.
AT3G46330	*-1,035*	MATERNAL EFFECT EMBRYO ARREST 39 (MEE39)	maternal effect embryo arrest 39 (MEE39); FUNCTIONS IN: kinase activity; INVOLVED IN: embryo development ending in seed dormancy; LOCATED IN: endomembrane system; CONTAINS InterPro DOMAIN/s: Protein kinase, ATP binding site (InterPro:IPR017441), Protein kinase, catalytic domain (InterPro:IPR000719), Serine-threonine/tyrosine-protein kinase (InterPro:IPR001245), Protein kinase-like domain (InterPro:IPR011009), Serine/threonine-protein kinase, active site (InterPro:IPR008271); BEST Arabidopsis thaliana protein match is: Leucine-rich repeat protein kinase family protein (TAIR:AT3G46400.1)
AT5G54140	*-1,035*	IAA-LEUCINE-RESISTANT (ILR1)-LIKE 3 (ILL3)	encodes a protein similar to IAA amino acid conjugate hydrolase
AT2G37330	*-1,036*	ALUMINUM SENSITIVE 3 (ALS3)	Encodes an ABC transporter-like protein, without an ATPase domain, required for aluminum (Al) resistance/tolerance and may function to redistribute accumulated Al away from sensitive tissues in order to protect the growing root from the toxic effects of Al.

AT5G60280	*-1,036*	L-TYPE LECTIN RECEPTOR KINASE I.8 (LECRK-I.8)	Concanavalin A-like lectin protein kinase family protein; FUNCTIONS IN: kinase activity; INVOLVED IN: protein amino acid phosphorylation; LOCATED IN: endomembrane system; EXPRESSED IN: root; CONTAINS InterPro DOMAIN/s: Legume lectin, beta chain (InterPro:IPR001220), Protein kinase, ATP binding site (InterPro:IPR017441), Serine/threonine-protein kinase-like domain (InterPro:IPR017442), Concanavalin A-like lectin/glucanase, subgroup (InterPro:IPR013320), Protein kinase-like domain (InterPro:IPR011009), Serine/threonine-protein kinase, active site (InterPro:IPR008271), Protein kinase, catalytic domain (InterPro:IPR000719), Concanavalin A-like lectin/glucanase (InterPro:IPR008985); BEST Arabidopsis thaliana protein match is: Concanavalin A-like lectin protein kinase family protein (TAIR:AT3G45440.1)
AT1G09155	*-1,038*	PHLOEM PROTEIN 2-B15 (PP2-B15)	phloem protein 2-B15 (PP2-B15); FUNCTIONS IN: carbohydrate binding; LOCATED IN: endomembrane system; EXPRESSED IN: 6 plant structures; EXPRESSED DURING: 4 anthesis, C globular stage, petal differentiation and expansion stage; BEST Arabidopsis thaliana protein match is: phloem protein 2-B13 (TAIR:AT1G56240.1)
AT2G45750	*-1,038*		S-adenosyl-L-methionine-dependent methyltransferases superfamily protein; CONTAINS InterPro DOMAIN/s: Protein of unknown function DUF248, methyltransferase putative (InterPro:IPR004159); BEST Arabidopsis thaliana protein match is: S-adenosyl-L-methionine-dependent methyltransferases superfamily protein (TAIR:AT4G00750.1)
AT4G24580	*-1,038*	ROP1 ENHANCER 1 (REN1)	Encodes a Rho GTPase-activating protein that interacts with ROP1 (a Rho GTPase) and regulates pollen tube development. This protein can be observed at the apical tip of growing pollen tubes and on endocytic vesicles traveling to this region of the pollen tube.
AT4G14780	*-1,039*		Protein kinase superfamily protein; FUNCTIONS IN: protein serine/threonine/tyrosine kinase activity, kinase activity; INVOLVED IN: protein amino acid phosphorylation; LOCATED IN: cellular_component unknown; EXPRESSED IN: 7 plant structures; EXPRESSED DURING: L mature pollen stage, M germinated pollen stage, 4 anthesis, petal differentiation and expansion stage; CONTAINS InterPro DOMAIN/s: Protein kinase, catalytic domain (InterPro:IPR000719), Serine-threonine/tyrosine-protein kinase (InterPro:IPR001245), Serine/threonine protein kinase-like, ATMRK (InterPro:IPR015783), Protein kinase-like domain (InterPro:IPR011009), Serine/threonine-protein kinase, active site (InterPro:IPR008271); BEST Arabidopsis thaliana protein match is: Protein kinase superfamily protein (TAIR:AT3G22750.1)
AT4G19160	*-1,039*		unknown protein
AT5G15230	*-1,039*	GAST1 PROTEIN HOMOLOG 4 (GASA4)	Encodes gibberellin-regulated protein GASA4. Promotes GA responses and exhibits redox activity.
AT3G26210	*-1,04*	CYTOCHROME P450, FAMILY 71, SUBFAMILY B, POLYPEPTIDE 23 (CYP71B23)	putative cytochrome P450 The mRNA is cell-to-cell mobile.
AT4G35900	*-1,04*	(FD)	bZIP protein required for positive regulation of flowering. Mutants are late flowering. FD interacts with FT to promote flowering.Expressed in the shoot apex in floral anlagen, then declines in floral primordia.
AT1G74770	*-1,041*		zinc ion binding; FUNCTIONS IN: zinc ion binding; INVOLVED IN: biological_process unknown; LOCATED IN: cellular_component unknown; EXPRESSED IN: hypocotyl, root; CONTAINS InterPro DOMAIN/s: Zinc finger, CTCHY-type (InterPro:IPR017921), Zinc finger, CHY-type (InterPro:IPR008913), Zinc finger, RING-type (InterPro:IPR001841), Haemerythrin/HHE cation-binding motif (InterPro:IPR012312); BEST Arabidopsis thaliana protein match is: zinc ion binding;zinc ion binding (TAIR:AT1G18910.1)
AT2G38320	*-1,042*	TRICHOME BIREFRINGENCE-LIKE 34 (TBL34)	Encodes a member of the TBL (TRICHOME BIREFRINGENCE-LIKE) gene family containing a plant-specific DUF231 (domain of unknown function) domain. TBL gene family has 46 members, two of which (TBR/AT5G06700 and TBL3/AT5G01360) have been shown to be involved in the synthesis and deposition of secondary wall cellulose, presumably by influencing the esterification state of pectic polymers. A nomenclature for this gene family has been proposed (Volker Bischoff & Wolf Scheible, 2010, personal communication).TBL34 are required only for xylan 3-O-monoacetylation and 2,3-di-O-acetylation. This biochemical phenotype can be observed in tbl34 esk1, double mutant and tbl34 tbl35 esk1 triple mutants.
AT3G56100	*-1,042*	MERISTEMATIC RECEPTOR-LIKE KINASE (MRLK)	Protein kinase expressed in meristematic cells. Phosphorylates AGL24.

AT4G23710	*-1,042*	VACUOLAR ATP SYNTHASE SUBUNIT G2 (VAG2)	vacuolar ATP synthase subunit G2 (VAG2); FUNCTIONS IN: hydrolase activity, acting on acid anhydrides, catalyzing transmembrane movement of substances; INVOLVED IN: proton transport; LOCATED IN: plasma membrane, vacuole; EXPRESSED IN: 25 plant structures; EXPRESSED DURING: 15 growth stages; CONTAINS InterPro DOMAIN/s: Vacuolar (H+)-ATPase G subunit (InterPro:IPR005124); BEST Arabidopsis thaliana protein match is: vacuolar membrane ATPase 10 (TAIR:AT3G01390.2)
AT1G19790	*-1,043*	SHI-RELATED SEQUENCE 7 (SRS7)	A member of SHI gene family. Arabidopsis thaliana has ten members that encode proteins with a RING finger-like zinc finger motif. Despite being highly divergent in sequence, many of the SHI-related genes are partially redundant in function and synergistically promote gynoecium, stamen and leaf development in Arabidopsis.
AT1G19660	*-1,045*	BIFUNCTIONAL NUCLEASE IN BASAL DEFENSE RESPONSE 2 (BBD2)	Wound-responsive family protein; FUNCTIONS IN: DNA binding, nuclease activity; INVOLVED IN: response to wounding, nucleotide-excision repair; EXPRESSED IN: ovule; CONTAINS InterPro DOMAIN/s: Protein of unknown function DUF151 (InterPro:IPR003729), UvrB/UvrC protein (InterPro:IPR001943); BEST Arabidopsis thaliana protein match is: bifunctional nuclease in basal defense response 1 (TAIR:AT1G75380.3)
AT2G37440	*-1,045*		DNAse I-like superfamily protein; FUNCTIONS IN: hydrolase activity, inositol or phosphatidylinositol phosphatase activity; INVOLVED IN: biological_process unknown; EXPRESSED IN: sperm cell, root; CONTAINS InterPro DOMAIN/s: Inositol polyphosphate related phosphatase (InterPro:IPR000300), Endonuclease/exonuclease/phosphatase (InterPro:IPR005135); BEST Arabidopsis thaliana protein match is: DNAse I-like superfamily protein (TAIR:AT2G01900.1)
AT4G30120	*-1,045*	HEAVY METAL ATPASE 3 (HMA3)	encodes a protein similar to Zn-ATPase, a P1B-type ATPases transport zinc
AT1G44740	*-1,046*		unknown protein; FUNCTIONS IN: molecular_function unknown; INVOLVED IN: biological_process unknown; LOCATED IN: mitochondrion; EXPRESSED IN: embryo, leaf whorl, flower, seed; EXPRESSED DURING: F mature embryo stage, petal differentiation and expansion stage, E expanded cotyledon stage, D bilateral stage
AT2G45550	*-1,046*	CYTOCHROME P450, FAMILY 76, SUBFAMILY C, POLYPEPTIDE 4 (CYP76C4)	member of CYP76C
AT1G52190	*-1,047*	NRT1/ PTR FAMILY 1.2 (NPF1.2)	Encodes a low affinity nitrate transporter that is expressed in the plasma membrane and found in the phloem of the major veins of leaves. It is responsible for nitrate redistribution to young leaves.
AT3G03470	*-1,047*	CYTOCHROME P450, FAMILY 87, SUBFAMILY A, POLYPEPTIDE 9 (CYP89A9)	member of CYP89A
AT1G07740	*-1,048*		Tetratricopeptide repeat (TPR)-like superfamily protein; FUNCTIONS IN: molecular_function unknown; INVOLVED IN: biological_process unknown; LOCATED IN: mitochondrion; EXPRESSED IN: 10 plant structures; EXPRESSED DURING: 6 growth stages; CONTAINS InterPro DOMAIN/s: Pentatricopeptide repeat (InterPro:IPR002885); BEST Arabidopsis thaliana protein match is: Pentatricopeptide repeat (PPR-like) superfamily protein (TAIR:AT5G16420.1)
AT4G21810	*-1,048*	DERLIN-2.1 (DER2.1)	DERLIN-2.1 (DER2.1); CONTAINS InterPro DOMAIN/s: Der1-like (InterPro:IPR007599); BEST Arabidopsis thaliana protein match is: DERLIN-2.2 (TAIR:AT4G04860.1)
AT4G00780	*-1,049*		TRAF-like family protein; CONTAINS InterPro DOMAIN/s: TRAF-like (InterPro:IPR008974), MATH (InterPro:IPR002083); BEST Arabidopsis thaliana protein match is: TRAF-like family protein (TAIR:AT4G01390.1)
AT5G07770	*-1,049*	FORMIN HOMOLOG 16 (FH16)	Actin-binding FH2 protein; FUNCTIONS IN: actin binding; INVOLVED IN: cellular component organization, actin cytoskeleton organization; LOCATED IN: chloroplast; EXPRESSED IN: 20 plant structures; EXPRESSED DURING: 8 growth stages; CONTAINS InterPro DOMAIN/s: Actin-binding FH2/DRF autoregulatory (InterPro:IPR003104), Actin-binding FH2 (InterPro:IPR015425); BEST Arabidopsis thaliana protein match is: actin binding (TAIR:AT5G07740.1)
AT3G56000	*-1,051*	CELLULOSE SYNTHASE LIKE A14 (CSLA14)	encodes a gene similar to cellulose synthase

AT5G24490	*-1,051*		30S ribosomal protein, putative; FUNCTIONS IN: structural constituent of ribosome, binding; INVOLVED IN: translation, primary metabolic process; LOCATED IN: ribosome, chloroplast; EXPRESSED IN: 22 plant structures; EXPRESSED DURING: 13 growth stages; CONTAINS InterPro DOMAIN/s: Ribosomal protein S30Ae/sigma 54 modulation protein (InterPro:IPR003489)
AT1G75130	*-1,053*	CYTOCHROME P450, FAMILY 721, SUBFAMILY A, POLYPEPTIDE 1 (CYP721A1)	member of CYP721A
AT3G09400	*-1,053*	POL-LIKE 3 (PLL3)	Similar to POLTERGEIST (POL) protein phosphatase 2C. No phenotype observed in plants homozygous for a null allele. Ubiquitously expressed.
AT4G16400	*-1,053*		unknown protein; BEST Arabidopsis thaliana protein match is: unknown protein (TAIR:AT3G13175.1)
AT2G13360	*-1,054*	ALANINE:GLYOXYLATE AMINOTRANSFERASE (AGT)	Encodes a peroxisomal photorespiratory enzyme that catalyzes transamination reactions with multiple substrates. It is involved in photorespiration.
AT1G07590	*-1,056*		Tetratricopeptide repeat (TPR)-like superfamily protein; INVOLVED IN: response to cadmium ion; LOCATED IN: mitochondrion; EXPRESSED IN: 12 plant structures; EXPRESSED DURING: 4 anthesis, F mature embryo stage, petal differentiation and expansion stage, E expanded cotyledon stage, D bilateral stage; CONTAINS InterPro DOMAIN/s: Pentatricopeptide repeat (InterPro:IPR002885); BEST Arabidopsis thaliana protein match is: PENTATRICOPEPTIDE REPEAT 596 (TAIR:AT1G80270.3)
AT4G39770	*-1,056*	TREHALOSE-6-PHOSPHATE PHOSPHATASE H (TPPH)	Haloacid dehalogenase-like hydrolase (HAD) superfamily protein; FUNCTIONS IN: catalytic activity, trehalose-phosphatase activity; INVOLVED IN: trehalose biosynthetic process, metabolic process; LOCATED IN: cellular_component unknown; EXPRESSED IN: 10 plant structures; EXPRESSED DURING: 8 growth stages; CONTAINS InterPro DOMAIN/s: HAD-superfamily hydrolase, subfamily IIB (InterPro:IPR006379), Trehalose-phosphatase (InterPro:IPR003337); BEST Arabidopsis thaliana protein match is: Haloacid dehalogenase-like hydrolase (HAD) superfamily protein (TAIR:AT2G22190.1)
AT5G10280	*-1,056*	MYB DOMAIN PROTEIN 92 (MYB92)	Encodes a putative transcription factor (MYB92).
AT2G24762	*-1,057*	GLUTAMINE DUMPER 4 (GDU4)	Encodes a member of the GDU (glutamine dumper) family proteins involved in amino acid export: At4g31730 (GDU1), At4g25760 (GDU2), At5g57685 (GDU3), At2g24762 (GDU4), At5g24920 (GDU5), At3g30725 (GDU6) and At5g38770 (GDU7).
AT1G80320	*-1,058*		2-oxoglutarate (2OG) and Fe(II)-dependent oxygenase superfamily protein; FUNCTIONS IN: oxidoreductase activity; INVOLVED IN: biological_process unknown; LOCATED IN: cellular_component unknown; EXPRESSED IN: root; CONTAINS InterPro DOMAIN/s: Oxoglutarate/iron-dependent oxygenase (InterPro:IPR005123); BEST Arabidopsis thaliana protein match is: 2-oxoglutarate (2OG) and Fe(II)-dependent oxygenase superfamily protein (TAIR:AT1G15540.1)
AT1G18390	*-1,059*	LEAF RUST 10 DISEASE-RESISTANCE LOCUS RECEPTOR- LIKE PROTEIN KINASE-LIKE 1.2 (LRK10L1.2)	Protein kinase superfamily protein; FUNCTIONS IN: protein serine/threonine kinase activity, protein kinase activity, kinase activity, ATP binding; INVOLVED IN: protein amino acid phosphorylation; EXPRESSED IN: 22 plant structures; EXPRESSED DURING: 13 growth stages; CONTAINS InterPro DOMAIN/s: Protein kinase, ATP binding site (InterPro:IPR017441), Protein kinase, catalytic domain (InterPro:IPR000719), Serine/threonine-protein kinase domain (InterPro:IPR002290), Tyrosine-protein kinase, catalytic domain (InterPro:IPR020635), Serine/threonine-protein kinase-like domain (InterPro:IPR017442), Serine/threonine-protein kinase, active site (InterPro:IPR008271), Protein kinase-like domain (InterPro:IPR011009); BEST Arabidopsis thaliana protein match is: Protein kinase superfamily protein (TAIR:AT1G66880.1)
AT2G43100	*-1,059*	ISOPROPYLMALATE ISOMERASE 2 (IPMI2)	isopropylmalate isomerase 2 (IPMI2); FUNCTIONS IN: hydro-lyase activity, 3-isopropylmalate dehydratase activity; INVOLVED IN: leucine biosynthetic process, metabolic process; LOCATED IN: plastid; EXPRESSED IN: 20 plant structures; EXPRESSED DURING: 10 growth stages; CONTAINS InterPro DOMAIN/s: 3-isopropylmalate dehydratase, small subunit-like (InterPro:IPR012305), Aconitase A/isopropylmalate dehydratase small subunit, swivel (InterPro:IPR000573), Aconitase/3-isopropylmalate dehydratase, swivel (InterPro:IPR015928), Aconitase-like core (InterPro:IPR015937); BEST Arabidopsis thaliana protein match is: Aconitase/3-isopropylmalate dehydratase protein (TAIR:AT2G43090.1)
AT3G04060	*-1,059*	NAC DOMAIN CONTAINING PROTEIN 46 (NAC046)	NAC046 is a member of the NAC domain containing family of transcription factors. It was identified in a screen for regulators of chlorophyll protein gene expression. Mutants in NAC046 have delayed senescence and increased CHL content suggesting a role in regulation of senescence and chlorophyll degradation.

AT3G63200	*-1,059*	PATATIN-LIKE PROTEIN 9 (PLP9)	PATATIN-like protein 9 (PLP9); FUNCTIONS IN: nutrient reservoir activity; INVOLVED IN: metabolic process, lipid metabolic process; LOCATED IN: cellular_component unknown; EXPRESSED IN: 18 plant structures; EXPRESSED DURING: 13 growth stages; CONTAINS InterPro DOMAIN/s: Acyl transferase/acyl hydrolase/lysophospholipase (InterPro:IPR016035), Patatin (InterPro:IPR002641); BEST Arabidopsis thaliana protein match is: PATATIN-like protein 6 (TAIR:AT2G39220.1)
AT1G72160	*-1,06*		Sec14p-like phosphatidylinositol transfer family protein; FUNCTIONS IN: transporter activity; INVOLVED IN: transport; LOCATED IN: plasma membrane; EXPRESSED IN: 24 plant structures; EXPRESSED DURING: 13 growth stages; CONTAINS InterPro DOMAIN/s: Cellular retinaldehyde-binding/triple function, C-terminal (InterPro:IPR001251), Cellular retinaldehyde-binding/triple function, N-terminal (InterPro:IPR008273), GOLD (InterPro:IPR009038), Phosphatidylinositol transfer protein-like, N-terminal (InterPro:IPR011074); BEST Arabidopsis thaliana protein match is: SEC14 cytosolic factor family protein / phosphoglyceride transfer family protein (TAIR:AT4G09160.1)
AT4G04810	*-1,06*	METHIONINE SULFOXIDE REDUCTASE B4 (MSRB4)	methionine sulfoxide reductase B4 (MSRB4); FUNCTIONS IN: peptide-methionine-(S)-S-oxide reductase activity; INVOLVED IN: oxidation reduction; LOCATED IN: cytosol; EXPRESSED IN: root; CONTAINS InterPro DOMAIN/s: Methionine sulphoxide reductase B (InterPro:IPR002579), Mss4-like (InterPro:IPR011057); BEST Arabidopsis thaliana protein match is: methionine sulfoxide reductase B5 (TAIR:AT4G04830.1)
AT4G08930	*-1,06*	APR-LIKE 6 (APRL6)	Encodes a protein disulfide isomerase-like (PDIL) protein, a member of a multigene family within the thioredoxin (TRX) superfamily. This protein also belongs to the adenosine 5'-phosphosulfate reductase-like (APRL) group. The mRNA is cell-to-cell mobile.
AT5G42750	*-1,06*	BRI1 KINASE INHIBITOR 1 (BKI1)	Encodes a plasma-membrane associated phosphoprotein that interacts directly with the kinase domain of BRI1 through the evolutionarily conserved C-terminal BIM motif binding to the C-lobe of the BRI1 kinase domain. It interferes with the interaction between BRI1 with its signalling partner, the plasma membrane localised LRR-receptor kinase BAK1 by inhibiting the transphosphorylation to keep BRI1 at a basal level of activity. It is phosphorylated by BRI1 at Ser270 & Ser274 and at tyrosine site Tyr211 and dissociates from plasma membrane to end up in the cytosol after phosphorylation. Its loss-of-function mutant shows higher sensitivity to BR treatment.
AT2G40260	*-1,061*		Homeodomain-like superfamily protein; CONTAINS InterPro DOMAIN/s: Homeodomain-like (InterPro:IPR009057), Myb, DNA-binding (InterPro:IPR014778), HTH transcriptional regulator, Myb-type, DNA-binding (InterPro:IPR017930), Myb-like DNA-binding domain, SHAQKYF class (InterPro:IPR006447), Homeodomain-related (InterPro:IPR012287); BEST Arabidopsis thaliana protein match is: myb-like HTH transcriptional regulator family protein (TAIR:AT2G38300.1)
AT5G66790	*-1,061*		Protein kinase superfamily protein; FUNCTIONS IN: kinase activity; INVOLVED IN: protein amino acid phosphorylation; LOCATED IN: endomembrane system; CONTAINS InterPro DOMAIN/s: Protein kinase, ATP binding site (InterPro:IPR017441), Serine/threonine-protein kinase domain (InterPro:IPR002290), Serine/threonine-protein kinase-like domain (InterPro:IPR017442), Serine/threonine-protein kinase, active site (InterPro:IPR008271), Protein kinase-like domain (InterPro:IPR011009), Protein kinase, catalytic domain (InterPro:IPR000719), Tyrosine-protein kinase, catalytic domain (InterPro:IPR020635); BEST Arabidopsis thaliana protein match is: Protein kinase superfamily protein (TAIR:AT2G23450.1)
AT1G17090	*-1,062*		unknown protein; INVOLVED IN: biological_process unknown; LOCATED IN: endomembrane system; EXPRESSED IN: 17 plant structures; EXPRESSED DURING: 7 growth stages
AT2G45330	*-1,062*	EMBRYO DEFECTIVE 1067 (emb1067)	embryo defective 1067 (emb1067); FUNCTIONS IN: tRNA 2'-phosphotransferase activity, transferase activity, transferring phosphorus-containing groups; INVOLVED IN: tRNA splicing, via endonucleolytic cleavage and ligation, embryo development ending in seed dormancy; EXPRESSED IN: male gametophyte, pollen tube; EXPRESSED DURING: L mature pollen stage, M germinated pollen stage; CONTAINS InterPro DOMAIN/s: Phosphotransferase KptA/Tpt1 (InterPro:IPR002745); BEST Arabidopsis thaliana protein match is: RNA 2'-phosphotransferase, Tpt1 / KptA family (TAIR:AT5G23600.1)
AT4G03510	*-1,064*	RING MEMBRANE-ANCHOR 1 (RMA1)	RMA1 encodes a novel 28 kDa protein with a RING finger motif and a C-terminal membrane-anchoring domain that is involved in the secretory pathway. Has E3 ubiquitin ligase activity.

AT1G77760	*-1,066*	NITRATE REDUCTASE 1 (NIA1)	Encodes the cytosolic minor isoform of nitrate reductase (NR). Involved in the first step of nitrate assimilation, it contributes about 15% of the nitrate reductase activity in shoots. Similar to molybdopterin oxidoreductases at the N-terminus, and to FAD/NAD-binding cytochrome reductases at the C-terminus. Cofactors: FAD, heme iron (cytochrome B-557), and molybdenum-pterin.
AT1G02310	*-1,067*	ENDO-BETA-MANNANASE 1 (MAN1)	Glycosyl hydrolase superfamily protein; FUNCTIONS IN: cation binding, hydrolase activity, hydrolyzing O-glycosyl compounds, catalytic activity; INVOLVED IN: carbohydrate metabolic process; LOCATED IN: endomembrane system; EXPRESSED IN: 7 plant structures; EXPRESSED DURING: 4 anthesis, petal differentiation and expansion stage, D bilateral stage; CONTAINS InterPro DOMAIN/s: Glycoside hydrolase, catalytic core (InterPro:IPR017853), Glycoside hydrolase, family 5 (InterPro:IPR001547), Glycoside hydrolase, subgroup, catalytic core (InterPro:IPR013781); BEST Arabidopsis thaliana protein match is: Glycosyl hydrolase superfamily protein (TAIR:AT5G66460.1)
AT2G21840	*-1,067*		Cysteine/Histidine-rich C1 domain family protein; FUNCTIONS IN: zinc ion binding; INVOLVED IN: intracellular signaling pathway; LOCATED IN: cellular_component unknown; CONTAINS InterPro DOMAIN/s: Protein kinase C-like, phorbol ester/diacylglycerol binding (InterPro:IPR002219), Zinc finger, PHD-type, conserved site (InterPro:IPR019786), Zinc finger, RING-type (InterPro:IPR001841), Zinc finger, PHD-type (InterPro:IPR001965), C1-like (InterPro:IPR011424); BEST Arabidopsis thaliana protein match is: Cysteine/Histidine-rich C1 domain family protein (TAIR:AT2G21850.1)
AT4G02410	*-1,067*	L-TYPE LECTIN RECEPTOR KINASE IV.3 (LECRK-IV.3)	Concanavalin A-like lectin protein kinase family protein; FUNCTIONS IN: kinase activity; INVOLVED IN: protein amino acid phosphorylation; LOCATED IN: endomembrane system; EXPRESSED IN: 19 plant structures; EXPRESSED DURING: 10 growth stages; CONTAINS InterPro DOMAIN/s: Legume lectin, beta chain (InterPro:IPR001220), Protein kinase, ATP binding site (InterPro:IPR017441), Serine/threonine-protein kinase-like domain (InterPro:IPR017442), Concanavalin A-like lectin/glucanase, subgroup (InterPro:IPR013320), Protein kinase-like domain (InterPro:IPR011009), Serine/threonine-protein kinase, active site (InterPro:IPR008271), Protein kinase, catalytic domain (InterPro:IPR000719), Concanavalin A-like lectin/glucanase (InterPro:IPR008985), Legume lectin, beta chain, Mn/Ca-binding site (InterPro:IPR019825); BEST Arabidopsis thaliana protein match is: Concanavalin A-like lectin protein kinase family protein (TAIR:AT4G02420.1)
AT4G21850	*-1,067*	METHIONINE SULFOXIDE REDUCTASE B9 (MSRB9)	methionine sulfoxide reductase B9 (MSRB9); FUNCTIONS IN: peptide-methionine-(S)-S-oxide reductase activity; INVOLVED IN: oxidation reduction; LOCATED IN: cytosol; EXPRESSED IN: 18 plant structures; EXPRESSED DURING: 12 growth stages; CONTAINS InterPro DOMAIN/s: Methionine sulphoxide reductase B (InterPro:IPR002579), Mss4-like (InterPro:IPR011057); BEST Arabidopsis thaliana protein match is: methionine sulfoxide reductase B7 (TAIR:AT4G21830.2)
AT1G26820	*-1,068*	RIBONUCLEASE 3 (RNS3)	Encodes ribonuclease RNS3.
AT1G75640	*-1,07*	MUSTACHES (MUS)	Encodes a Leucine-Rich Repeat Receptor-Like Kinase MUSTACHES (MUS). Regulates stomatal bilateral symmetry.
AT4G34135	*-1,07*	UDP-GLUCOSYLTRANSFERASE 73B2 (UGT73B2)	The At4g34135 gene encodes a flavonol 7-O-glucosyltransferase (EC 2.4.1.237) that glucosylates also with a 20 fold lower activity flavonols (kaempferol and quercetin) at the 3-O-position.
AT5G47560	*-1,07*	TONOPLAST DICARBOXYLATE TRANSPORTER (TDT)	Encodes a tonoplast malate/fumarate transporter.
AT4G10550	*-1,071*		Subtilase family protein; FUNCTIONS IN: identical protein binding, serine-type endopeptidase activity; INVOLVED IN: proteolysis, negative regulation of catalytic activity; EXPRESSED IN: 8 plant structures; EXPRESSED DURING: F mature embryo stage, petal differentiation and expansion stage, E expanded cotyledon stage, D bilateral stage; CONTAINS InterPro DOMAIN/s: Protease-associated PA (InterPro:IPR003137), Proteinase inhibitor, propeptide (InterPro:IPR009020), Peptidase S8/S53, subtilisin/kexin/sedolisin (InterPro:IPR000209), Proteinase inhibitor I9, subtilisin propeptide (InterPro:IPR010259), Peptidase S8/S53, subtilisin, active site (InterPro:IPR022398), Peptidase S8, subtilisin-related (InterPro:IPR015500); BEST Arabidopsis thaliana protein match is: Subtilase family protein (TAIR:AT4G10540.1).
AT4G34760	*-1,071*	SMALL AUXIN UPREGULATED RNA 50 (SAUR50)	SAUR-like auxin-responsive protein family ; CONTAINS InterPro DOMAIN/s: Auxin responsive SAUR protein (InterPro:IPR003676); BEST Arabidopsis thaliana protein match is: SAUR-like auxin-responsive protein family (TAIR:AT2G16580.1)
AT3G07310	*-1,073*		Protein of unknown function (DUF760); CONTAINS InterPro DOMAIN/s: Protein of unknown function DUF760 (InterPro:IPR008479); BEST Arabidopsis thaliana protein match is: Protein of unknown function (DUF760) (TAIR:AT5G48590.1)

AT3G20130	*-1,073*	CYTOCHROME P450, FAMILY 705, SUBFAMILY A, POLYPEPTIDE 22 (CYP705A22)	Encodes a member of the CYP705A family of cytochrome P450 enzymes. Mutants show altered gravitropic responses.
AT1G62770	*-1,074*	(PMEI9)	Plant invertase/pectin methylesterase inhibitor superfamily protein; FUNCTIONS IN: enzyme inhibitor activity, pectinesterase inhibitor activity, pectinesterase activity; INVOLVED IN: biological_process unknown; LOCATED IN: endomembrane system; EXPRESSED IN: 16 plant structures; EXPRESSED DURING: 8 growth stages; CONTAINS InterPro DOMAIN/s: Pectinesterase inhibitor (InterPro:IPR006501); BEST Arabidopsis thaliana protein match is: pectin methylesterase inhibitor 1 (TAIR:AT4G12390.1)
AT5G40590	*-1,074*		Cysteine/Histidine-rich C1 domain family protein; CONTAINS InterPro DOMAIN/s: DC1 (InterPro:IPR004146), C1-like (InterPro:IPR011424); BEST Arabidopsis thaliana protein match is: Cysteine/Histidine-rich C1 domain family protein (TAIR:AT2G44370.1)
AT1G50630	*-1,076*		Protein of unknown function (DUF3537); CONTAINS InterPro DOMAIN/s: Protein of unknown function DUF3537 (InterPro:IPR021924); BEST Arabidopsis thaliana protein match is: Protein of unknown function (DUF3537) (TAIR:AT3G20300.1)
AT1G67980	*-1,076*	CAFFEOYL-COA 3-O-METHYLTRANSFERASE (CCOAMT)	Encodes S-adenosyl-L-methionine: transcaffeoyl Coenzyme A 3-O-methyltransferase.
AT5G47770	*-1,076*	FARNESYL DIPHOSPHATE SYNTHASE 1 (FPS1)	Encodes a protein with farnesyl diphosphate synthase activity.
AT1G78090	*-1,077*	TREHALOSE-6-PHOSPHATE PHOSPHATASE B (TPPB)	homologous to the C-terminal part of microbial trehalose-6-phosphate phosphatases
AT4G13130	*-1,077*		Cysteine/Histidine-rich C1 domain family protein; FUNCTIONS IN: zinc ion binding; INVOLVED IN: biological_process unknown; LOCATED IN: chloroplast envelope; EXPRESSED IN: leaf whorl, hypocotyl, root, pollen tube; CONTAINS InterPro DOMAIN/s: DC1 (InterPro:IPR004146), Zinc finger, PHD-type (InterPro:IPR001965), C1-like (InterPro:IPR011424); BEST Arabidopsis thaliana protein match is: Cysteine/Histidine-rich C1 domain family protein (TAIR:AT4G02540.1)
AT5G13910	*-1,077*	LEAFY PETIOLE (LEP)	Encodes a member of the ERF (ethylene response factor) subfamily B-1 of ERF/AP2 transcription factor family (LEAFY PETIOLE). The protein contains one AP2 domain. There are 15 members in this subfamily including ATERF-3, ATERF-4, ATERF-7, and LEAFY PETIOLE. Acts as a positive regulator of gibberellic acid-induced germination.
AT3G23430	*-1,079*	PHOSPHATE 1 (PHO1)	Encodes a protein with the mainly hydrophilic N-terminal and the C-terminal containing 6 potential membrane-spanning domains. The mutant is deficient in the transfer of phosphate from root epidermal and cortical cells to the xylem. Its expression is repressed by phosphate (Pi) in shoots, and transiently induced by phosphite (Phi) in roots and shoots.
AT5G49100	*-1,079*		unknown protein; BEST Arabidopsis thaliana protein match is: unknown protein (TAIR:AT3G06868.1)
AT4G20460	*-1,081*		NAD(P)-binding Rossmann-fold superfamily protein; FUNCTIONS IN: coenzyme binding, UDP-glucose 4-epimerase activity, binding, catalytic activity; INVOLVED IN: galactose metabolic process, cellular metabolic process, nucleotide-sugar metabolic process, metabolic process; EXPRESSED IN: 18 plant structures; EXPRESSED DURING: 9 growth stages; CONTAINS InterPro DOMAIN/s: NAD-dependent epimerase/dehydratase (InterPro:IPR001509), NAD(P)-binding domain (InterPro:IPR016040), UDP-glucose 4-epimerase (InterPro:IPR005886); BEST Arabidopsis thaliana protein match is: NAD(P)-binding Rossmann-fold superfamily protein (TAIR:AT1G30620.2)
AT4G28040	*-1,081*	USUALLY MULTIPLE ACIDS MOVE IN AND OUT TRANSPORTERS 33 (UMAMIT33)	nodulin MtN21-like transporter family protein
AT3G09220	*-1,082*	LACCASE 7 (LAC7)	putative laccase, a member of laccase family of genes (17 members in Arabidopsis).
AT4G05590	*-1,082*	NEGATIVE REGULATOR OF GUARD CELL ABA SIGNALING 1 (NRGA1)	Encodes NRGA1, a putative mitochondrial pyruvate carrier that mediates ABA regulation of guard cell ion channels and drought stress responses.

AT4G31250	*-1,083*	(PRK7)	Leucine-rich repeat protein kinase family protein; FUNCTIONS IN: protein serine/threonine kinase activity, kinase activity, ATP binding; INVOLVED IN: transmembrane receptor protein tyrosine kinase signaling pathway, protein amino acid phosphorylation; LOCATED IN: endomembrane system; EXPRESSED IN: shoot apex, embryo, leaf whorl, sepal, flower; EXPRESSED DURING: petal differentiation and expansion stage, D bilateral stage; CONTAINS InterPro DOMAIN/s: Protein kinase, catalytic domain (InterPro:IPR000719), Serine/threonine-protein kinase domain (InterPro:IPR002290), Leucine-rich repeat-containing N-terminal domain, type 2 (InterPro:IPR013210), Tyrosine-protein kinase, catalytic domain (InterPro:IPR020635), Serine/threonine-protein kinase-like domain (InterPro:IPR017442), Protein kinase-like domain (InterPro:IPR011009); BEST Arabidopsis thaliana protein match is: Leucine-rich repeat protein kinase family protein (TAIR:AT5G35390.1)
AT4G35380	*-1,083*	BIG4 (BIG4)	Encodes one of the functionally redundant ARF guanine-nucleotide exchange factors (ARF-GEFs). Functions as regulators of post-Golgi trafficking.
AT5G15130	*-1,083*	WRKY DNA-BINDING PROTEIN 72 (WRKY72)	member of WRKY Transcription Factor; Group II-b; contribute to basal immunity. The mRNA is cell-to-cell mobile.
AT1G64200	*-1,084*	VACUOLAR H+-ATPASE SUBUNIT E ISOFORM 3 (VHA-E3)	vacuolar H+-ATPase subunit E isoform 3 (VHA-E3); FUNCTIONS IN: proton-transporting ATPase activity, rotational mechanism; INVOLVED IN: ATP synthesis coupled proton transport; LOCATED IN: plasma membrane, vacuole, mitochondrial proton-transporting ATP synthase complex; EXPRESSED IN: 27 plant structures; EXPRESSED DURING: 17 growth stages; CONTAINS InterPro DOMAIN/s: ATPase, V1/A1 complex, subunit E (InterPro:IPR002842); BEST Arabidopsis thaliana protein match is: vacuolar ATP synthase subunit E1 (TAIR:AT4G11150.1)
AT2G24720	*-1,084*	GLUTAMATE RECEPTOR 2.2 (GLR2.2)	member of Putative ligand-gated ion channel subunit family
AT5G24270	*-1,084*	SALT OVERLY SENSITIVE 3 (SOS3)	encodes a calcium sensor that is essential for K+ nutrition, K+/Na+ selectivity, and salt tolerance. The protein is similar to calcineurin B. Lines carrying recessive mutations are hypersensitive to Na+ and Li+ stresses and is unable to grow in low K+. The growth defect is rescued by extracellular calcium.
AT1G30130	*-1,085*		unknown protein; FUNCTIONS IN: molecular_function unknown; INVOLVED IN: biological_process unknown; LOCATED IN: endomembrane system; EXPRESSED IN: 23 plant structures; EXPRESSED DURING: 13 growth stages; CONTAINS InterPro DOMAIN/s: Protein of unknown function DUF1365 (InterPro:IPR010775)
AT2G25900	*-1,085*	(ATCTH)	Encodes a protein with two tandem-arrayed CCCH-type zinc fingers that binds RNA and is involved in RNA turnover. The mRNA is cell-to-cell mobile.
AT5G24800	*-1,085*	BASIC LEUCINE ZIPPER 9	Encodes bZIP protein BZO2H2.
AT1G23750	*-1,086*		Nucleic acid-binding, OB-fold-like protein; CONTAINS InterPro DOMAIN/s: Nucleic acid-binding, OB-fold-like (InterPro:IPR016027), Nucleic acid-binding, OB-fold (InterPro:IPR012340); BEST Arabidopsis thaliana protein match is: Nucleic acid-binding, OB-fold-like protein (TAIR:AT1G10590.2)
AT5G03960	*-1,086*	IQ-DOMAIN 12 (IQD12)	IQ-domain 12 (IQD12); CONTAINS InterPro DOMAIN/s: IQ calmodulin-binding region (InterPro:IPR000048); BEST Arabidopsis thaliana protein match is: IQ-domain 11 (TAIR:AT5G13460.1)
AT3G13720	*-1,088*	(PRA8)	PRA8; CONTAINS InterPro DOMAIN/s: Prenylated rab acceptor PRA1 (InterPro:IPR004895); BEST Arabidopsis thaliana protein match is: prenylated RAB acceptor 1.F4 (TAIR:AT3G13710.1)
AT2G02710	*-1,091*	PAS/LOV PROTEIN B (PLPB)	Encodes a putative blue light receptor protein.
AT3G24503	*-1,093*	ALDEHYDE DEHYDROGENASE 2C4 (ALDH2C4)	Arabidopsis thaliana aldehyde dehydrogenase AtALDH1a mRNA. a sinapaldehyde dehydrogenase catalyzes both the oxidation of coniferylaldehyde and sinapaldehyde forming ferulic acid and sinapic acid, respectively
AT1G34040	*-1,094*		Pyridoxal phosphate (PLP)-dependent transferases superfamily protein; FUNCTIONS IN: pyridoxal phosphate binding, carbon-sulfur lyase activity, catalytic activity; INVOLVED IN: biological_process unknown; LOCATED IN: endomembrane system; CONTAINS InterPro DOMAIN/s: Pyridoxal phosphate-dependent transferase, major domain (InterPro:IPR015424), EGF-like, alliinase (InterPro:IPR006947), Pyridoxal phosphate-dependent transferase, major region, subdomain 1 (InterPro:IPR015421), Allinase, C-terminal (InterPro:IPR006948), Pyridoxal phosphate-dependent transferase, major region, subdomain 2 (InterPro:IPR015422); BEST Arabidopsis thaliana protein match is: Pyridoxal phosphate (PLP)-dependent transferases superfamily protein (TAIR:AT1G34060.1)

AT5G07680	*-1,094*	NAC DOMAIN CONTAINING PROTEIN 80 (NAC080)	NAC domain containing protein 80 (NAC080); FUNCTIONS IN: sequence-specific DNA binding transcription factor activity; INVOLVED IN: multicellular organismal development, regulation of transcription; LOCATED IN: cellular_component unknown; EXPRESSED IN: 12 plant structures; EXPRESSED DURING: LP.04 four leaves visible, 4 anthesis, C globular stage, petal differentiation and expansion stage; CONTAINS InterPro DOMAIN/s: No apical meristem (NAM) protein (InterPro:IPR003441); BEST Arabidopsis thaliana protein match is: NAC domain containing protein 100 (TAIR:AT5G61430.1)
AT5G18350	*-1,094*		Disease resistance protein (TIR-NBS-LRR class) family; FUNCTIONS IN: transmembrane receptor activity, nucleoside-triphosphatase activity, nucleotide binding, ATP binding; INVOLVED IN: signal transduction, defense response, apoptosis, innate immune response; LOCATED IN: intrinsic to membrane; CONTAINS InterPro DOMAIN/s: ATPase, AAA+ type, core (InterPro:IPR003593), NB-ARC (InterPro:IPR002182), Leucine-rich repeat (InterPro:IPR001611), Disease resistance protein (InterPro:IPR000767), Toll-Interleukin receptor (InterPro:IPR000157); BEST Arabidopsis thaliana protein match is: Disease resistance protein (TIR-NBS-LRR class) family (TAIR:AT5G18370.1)
AT1G62320	*-1,095*		ERD (early-responsive to dehydration stress) family protein; FUNCTIONS IN: molecular_function unknown; INVOLVED IN: biological_process unknown; LOCATED IN: endomembrane system, membrane; EXPRESSED IN: pollen tube; CONTAINS InterPro DOMAIN/s: Protein of unknown function DUF221 (InterPro:IPR003864); BEST Arabidopsis thaliana protein match is: ERD (early-responsive to dehydration stress) family protein (TAIR:AT1G11960.1)
AT1G78680	*-1,095*	GAMMA-GLUTAMYL HYDROLASE 2 (GGH2)	The Arabidopsis protein AtGGH2 is a gamma-glutamyl hydrolase acting specifically on monoglutamates. The enzyme is involved in the tetrahydrofolate metabolism and located to the vacuole.
AT3G16460	*-1,095*	JACALIN-RELATED LECTIN 34 (JAL34)	Mannose-binding lectin superfamily protein; FUNCTIONS IN: copper ion binding; INVOLVED IN: response to cold; LOCATED IN: cytosol, nucleus, membrane; EXPRESSED IN: 23 plant structures; EXPRESSED DURING: 13 growth stages; CONTAINS InterPro DOMAIN/s: Mannose-binding lectin (InterPro:IPR001229); BEST Arabidopsis thaliana protein match is: myrosinase-binding protein 1 (TAIR:AT1G52040.1)
AT1G13245	*-1,096*	ROTUNDIFOLIA LIKE 17 (RTFL17)	ROTUNDIFOLIA like 17 (RTFL17); CONTAINS InterPro DOMAIN/s: DVL (InterPro:IPR012552); BEST Arabidopsis thaliana protein match is: ROTUNDIFOLIA like 15 (TAIR:AT1G68825.2)
AT2G30080	*-1,096*	(ZIP6)	member of Fe(II) transporter isolog family. Gene expression is not regulated by iron, copper, or zinc deficiency or excess.
AT5G10210	*-1,097*		CONTAINS InterPro DOMAIN/s: C2 calcium-dependent membrane targeting (InterPro:IPR000008); BEST Arabidopsis thaliana protein match is: unknown protein (TAIR:AT5G65030.1)
AT2G43910	*-1,099*	HARMLESS TO OZONE LAYER 1 (HOL1)	HARMLESS TO OZONE LAYER 1 (HOL1); FUNCTIONS IN: methyltransferase activity, thiopurine S-methyltransferase activity; INVOLVED IN: metabolic process; LOCATED IN: plasma membrane; EXPRESSED IN: guard cell, cultured cell, leaf; CONTAINS InterPro DOMAIN/s: Thiopurine S-methyltransferase (InterPro:IPR008854); BEST Arabidopsis thaliana protein match is: S-adenosyl-L-methionine-dependent methyltransferases superfamily protein (TAIR:AT2G43920.1)
AT3G47820	*-1,099*	PLANT U-BOX 39 (PUB39)	PLANT U-BOX 39 (PUB39); FUNCTIONS IN: ubiquitin-protein ligase activity, binding; INVOLVED IN: protein ubiquitination; LOCATED IN: ubiquitin ligase complex; CONTAINS InterPro DOMAIN/s: U box domain (InterPro:IPR003613), Armadillo-like helical (InterPro:IPR011989), Armadillo (InterPro:IPR000225), Armadillo-type fold (InterPro:IPR016024); BEST Arabidopsis thaliana protein match is: RING/U-box superfamily protein with ARM repeat domain (TAIR:AT5G62560.1)
AT2G21550	*-1,101*		Bifunctional dihydrofolate reductase/thymidylate synthase; FUNCTIONS IN: thymidylate synthase activity, dihydrofolate reductase activity; INVOLVED IN: oxidation reduction, glycine biosynthetic process, one-carbon metabolic process, nucleotide biosynthetic process, dTMP biosynthetic process; EXPRESSED IN: stem, hypocotyl, root; CONTAINS InterPro DOMAIN/s: Dihydrofolate reductase domain (InterPro:IPR001796), Dihydrofolate reductase conserved site (InterPro:IPR017925), Bifunctional dihydrofolate reductase/thymidylate synthase (InterPro:IPR012262), Thymidylate synthase (InterPro:IPR000398); BEST Arabidopsis thaliana protein match is: thymidylate synthase 1 (TAIR:AT2G16370.1)
AT2G22170	*-1,101*	PLAT DOMAIN PROTEIN 2 (PLAT2)	Lipase/lipooxygenase, PLAT/LH2 family protein; FUNCTIONS IN: molecular_function unknown; INVOLVED IN: biological_process unknown; LOCATED IN: thylakoid, chloroplast, membrane; EXPRESSED IN: 19 plant structures; EXPRESSED DURING: 13 growth stages; CONTAINS InterPro DOMAIN/s: Lipoxygenase, LH2 (InterPro:IPR001024), Lipase/lipooxygenase, PLAT/LH2 (InterPro:IPR008976); BEST Arabidopsis thaliana protein match is: Lipase/lipooxygenase, PLAT/LH2 family protein (TAIR:AT4G39730.1)

AT2G32270	*-1,101*	ZINC TRANSPORTER 3 PRECURSOR (ZIP3)	A member of Zrt- and Irt-related protein (ZIP) family. transcript is induced in response to zinc deficiency in the root. also response to iron deficiency.
AT2G44370	*-1,101*		Cysteine/Histidine-rich C1 domain family protein; CONTAINS InterPro DOMAIN/s: DC1 (InterPro:IPR004146), C1-like (InterPro:IPR011424); BEST Arabidopsis thaliana protein match is: Cysteine/Histidine-rich C1 domain family protein (TAIR:AT2G17740.1)
AT3G16490	*-1,101*	IQ-DOMAIN 26 (IQD26)	IQ-domain 26 (IQD26); CONTAINS InterPro DOMAIN/s: IQ calmodulin-binding region (InterPro:IPR000048); BEST Arabidopsis thaliana protein match is: IQ-domain 27 (TAIR:AT1G51960.1)
AT1G80760	*-1,103*	NOD26-LIKE INTRINSIC PROTEIN 6;1 (NIP6;1)	Encodes a protein with boron transporter activity. It helps to preferentially direct boron to young developing tissues in the shoot, such as immature leaves, under low boron conditions. This boron channel appears to be impermeable to water, unlike the closely related NIP5;1 boron transporter. This protein also allows the transport of glycerol, urea, and formimide but not larger uncharged solutes such as arabitol and sucrose when it is expressed heterologously.
AT4G15960	*-1,103*		alpha/beta-Hydrolases superfamily protein; FUNCTIONS IN: epoxide hydrolase activity, catalytic activity; INVOLVED IN: biological_process unknown; LOCATED IN: mitochondrion; CONTAINS InterPro DOMAIN/s: Epoxide hydrolase-like (InterPro:IPR000639), Alpha/beta hydrolase fold-1 (InterPro:IPR000073); BEST Arabidopsis thaliana protein match is: alpha/beta-Hydrolases superfamily protein (TAIR:AT4G15955.3)
AT4G30060	*-1,103*		Core-2/I-branching beta-1,6-N-acetylglucosaminyltransferase family protein; CONTAINS InterPro DOMAIN/s: Core-2/I-Branching enzyme (InterPro:IPR021141); BEST Arabidopsis thaliana protein match is: Core-2/I-branching beta-1,6-N-acetylglucosaminyltransferase family protein (TAIR:AT2G19160.1)
AT4G02075	*-1,104*	PITCHOUN 1 (PIT1)	pitchoun 1 (PIT1); FUNCTIONS IN: zinc ion binding; LOCATED IN: plasma membrane; EXPRESSED IN: 18 plant structures; EXPRESSED DURING: 10 growth stages; CONTAINS InterPro DOMAIN/s: Protein of unknown function DUF3675 (InterPro:IPR022143), Zinc finger, C3HC4 RING-type (InterPro:IPR018957), Zinc finger, RING-CH-type (InterPro:IPR011016); BEST Arabidopsis thaliana protein match is: RING/FYVE/PHD zinc finger superfamily protein (TAIR:AT1G02610.1)
AT1G44770	*-1,105*		unknown protein; BEST Arabidopsis thaliana protein match is: unknown protein (TAIR:AT5G49710.3)
AT1G10530	*-1,106*		unknown protein; FUNCTIONS IN: molecular_function unknown; INVOLVED IN: N-terminal protein myristoylation; BEST Arabidopsis thaliana protein match is: unknown protein (TAIR:AT1G60010.1)
AT2G17060	*-1,106*		Disease resistance protein (TIR-NBS-LRR class) family; FUNCTIONS IN: transmembrane receptor activity, ATP binding; INVOLVED IN: signal transduction, apoptosis, defense response, innate immune response; LOCATED IN: intrinsic to membrane; EXPRESSED IN: root; CONTAINS InterPro DOMAIN/s: NB-ARC (InterPro:IPR002182), Leucine-rich repeat (InterPro:IPR001611), Disease resistance protein (InterPro:IPR000767), Toll-Interleukin receptor (InterPro:IPR000157); BEST Arabidopsis thaliana protein match is: Disease resistance protein (TIR-NBS-LRR class) family (TAIR:AT4G36150.1)
AT2G21850	*-1,106*		Cysteine/Histidine-rich C1 domain family protein; FUNCTIONS IN: zinc ion binding; INVOLVED IN: intracellular signaling pathway; LOCATED IN: cellular_component unknown; EXPRESSED IN: embryo, hypocotyl, root; EXPRESSED DURING: D bilateral stage; CONTAINS InterPro DOMAIN/s: Protein kinase C-like, phorbol ester/diacylglycerol binding (InterPro:IPR002219), Zinc finger, PHD-type, conserved site (InterPro:IPR019786), DC1 (InterPro:IPR004146), Zinc finger, PHD-type (InterPro:IPR001965), C1-like (InterPro:IPR011424); BEST Arabidopsis thaliana protein match is: Cysteine/Histidine-rich C1 domain family protein (TAIR:AT2G21840.1)
AT5G19730	*-1,107*		Pectin lyase-like superfamily protein; FUNCTIONS IN: pectinesterase activity; INVOLVED IN: cell wall modification; LOCATED IN: cell wall, plant-type cell wall, cytoplasm; EXPRESSED IN: 17 plant structures; EXPRESSED DURING: 8 growth stages; CONTAINS InterPro DOMAIN/s: Pectinesterase, active site (InterPro:IPR018040), Pectin lyase fold/virulence factor (InterPro:IPR011050), Pectinesterase, catalytic (InterPro:IPR000070), Pectin lyase fold (InterPro:IPR012334); BEST Arabidopsis thaliana protein match is: Pectin lyase-like superfamily protein (TAIR:AT2G36710.1)

AT4G20230	-1,11	TERPENE SYNTHASE 30 (TPS30)	Terpenoid cyclases/Protein prenyltransferases superfamily protein; FUNCTIONS IN: lyase activity, magnesium ion binding; INVOLVED IN: metabolic process; EXPRESSED IN: hypocotyl, root; CONTAINS InterPro DOMAIN/s: Terpene synthase, metal-binding domain (InterPro:IPR005630), Terpenoid synthase (InterPro:IPR008949), Terpenoid cylases/protein prenyltransferase alpha-alpha toroid (InterPro:IPR008930), Terpene synthase-like (InterPro:IPR001906); BEST Arabidopsis thaliana protein match is: Terpenoid cyclases/Protein prenyltransferases superfamily protein (TAIR:AT4G20200.1)
AT4G37150	-1,11	METHYL ESTERASE 9 (MES9)	Encodes a protein shown to have carboxylesterase activity, methyl salicylate esterase activity, methyl jasmonate esterase activity, and methyl IAA esterase activity in vitro. MES9 appears to be involved in MeSA hydrolysis in planta. Expression of MES9 can restore systemic acquired resistance in SAR-deficient tobacco plants. This protein does not act on MeGA4, or MEGA9 in vitro.
AT5G04820	-1,11	OVATE FAMILY PROTEIN 13 (OFP13)	ovate family protein 13 (OFP13); INVOLVED IN: biological_process unknown; LOCATED IN: endomembrane system; EXPRESSED IN: 23 plant structures; EXPRESSED DURING: 14 growth stages; CONTAINS InterPro DOMAIN/s: Protein of unknown function DUF623 (InterPro:IPR006458); BEST Arabidopsis thaliana protein match is: ovate family protein 15 (TAIR:AT2G36050.1)
AT5G45100	-1,11	BOI-RELATED GENE 1 (BRG1)	Encodes one of the BRGs (BOI-related gene) involved in resistance to Botrytis cinerea.
AT1G76560	-1,111	CP12 DOMAIN-CONTAINING PROTEIN 3 (CP12-3)	CP12 domain-containing protein 3 (CP12-3); FUNCTIONS IN: molecular_function unknown; INVOLVED IN: negative regulation of reductive pentose-phosphate cycle; LOCATED IN: chloroplast; EXPRESSED IN: 20 plant structures; EXPRESSED DURING: 13 growth stages; CONTAINS InterPro DOMAIN/s: Protein of unknown function CP12 (InterPro:IPR003823); BEST Arabidopsis thaliana protein match is: CP12 domain-containing protein 1 (TAIR:AT2G47400.1)
AT2G46030	-1,111	UBIQUITIN-CONJUGATING ENZYME 6 (UBC6)	Ubiquitin conjugating enzyme E2
AT3G58810	-1,111	METAL TOLERANCE PROTEIN A2 (MTPA2)	Member of Zinc transporter (ZAT) family. Contributes to basic cellular Zn tolerance and controls Zn partitioning, particularly under conditions of high rates of Zn influx into the root symplasm. Localizes to the vacuolar membrane.
AT1G61050	-1,112		alpha 1,4-glycosyltransferase family protein; FUNCTIONS IN: transferase activity, transferring glycosyl groups, transferase activity; INVOLVED IN: biological_process unknown; LOCATED IN: Golgi stack; CONTAINS InterPro DOMAIN/s: Alpha 1,4-glycosyltransferase conserved region (InterPro:IPR007652), Glycosyltransferase, DXD sugar-binding region (InterPro:IPR007577); BEST Arabidopsis thaliana protein match is: alpha 1,4-glycosyltransferase family protein (TAIR:AT5G01250.1)
AT2G02130	-1,113	LOW-MOLECULAR-WEIGHT CYSTEINE-RICH 68 (LCR68)	Predicted to encode a PR (pathogenesis-related) protein. Belongs to the plant defensin (PDF) family with the following members: At1g75830/PDF1.1, At5g44420/PDF1.2a, At2g26020/PDF1.2b, At5g44430/PDF1.2c, At2g26010/PDF1.3, At1g19610/PDF1.4, At1g55010/PDF1.5, At2g02120/PDF2.1, At2g02100/PDF2.2, At2g02130/PDF2.3, At1g61070/PDF2.4, At5g63660/PDF2.5, At2g02140/PDF2.6, At5g38330/PDF3.1 and At4g30070/PDF3.2. The mRNA is cell-to-cell mobile.
AT2G34150	-1,113	(WAVE1)	Encodes a member of the SCAR family.These proteins are part of a complex (WAVE) complex.The SCAR subunit activates the ARP2/3 complex which in turn act as a nucleator for actin filaments.
AT2G45400	-1,114	BRI1-5 ENHANCED 1 (BEN1)	involved in the regulation of brassinosteroid metabolic pathway
AT1G08090	-1,115	NITRATE TRANSPORTER 2:1 (NRT2:1)	High-affinity nitrate transporter. Up-regulated by nitrate. Functions as a repressor of lateral root initiation independently of nitrate uptake.
AT1G72870	-1,116		Disease resistance protein (TIR-NBS class); FUNCTIONS IN: transmembrane receptor activity, ATP binding; INVOLVED IN: signal transduction, defense response, apoptosis, innate immune response; LOCATED IN: intrinsic to membrane; EXPRESSED IN: stem, sperm cell, root; CONTAINS InterPro DOMAIN/s: NB-ARC (InterPro:IPR002182), Disease resistance protein (InterPro:IPR000767), Toll-Interleukin receptor (InterPro:IPR000157); BEST Arabidopsis thaliana protein match is: disease resistance protein (TIR-NBS class) (TAIR:AT5G48780.2)
AT1G13830	-1,118		Carbohydrate-binding X8 domain superfamily protein; FUNCTIONS IN: molecular_function unknown; INVOLVED IN: biological_process unknown; LOCATED IN: endomembrane system; EXPRESSED IN: 8 plant structures; EXPRESSED DURING: 4 anthesis, petal differentiation and expansion stage; CONTAINS InterPro DOMAIN/s: X8 (InterPro:IPR012946); BEST Arabidopsis thaliana protein match is: Carbohydrate-binding X8 domain superfamily protein (TAIR:AT2G03505.1)

AT4G27250	*-1,119*		NAD(P)-binding Rossmann-fold superfamily protein; FUNCTIONS IN: cinnamyl-alcohol dehydrogenase activity; INVOLVED IN: cellular metabolic process, metabolic process; LOCATED IN: cellular_component unknown; EXPRESSED IN: 13 plant structures; EXPRESSED DURING: 6 growth stages; CONTAINS InterPro DOMAIN/s: NAD-dependent epimerase/dehydratase (InterPro:IPR001509), NAD(P)-binding domain (InterPro:IPR016040); BEST Arabidopsis thaliana protein match is: dihydroflavonol 4-reductase (TAIR:AT5G42800.1)
AT2G46750	*-1,12*	L -GULONO-1,4-LACTONE (L -GULL) OXIDASE 2 (GULLO2)	Encodes a homolog of rat L-gulono-1,4-lactone (L-GulL) oxidase that is involved in the biosynthesis of L-ascorbic acid.
AT1G12080	*-1,121*		Vacuolar calcium-binding protein-related; BEST Arabidopsis thaliana protein match is: Vacuolar calcium-binding protein-related (TAIR:AT1G62480.1)
AT5G42590	*-1,121*	CYTOCHROME P450, FAMILY 71, SUBFAMILY A, POLYPEPTIDE 16 (CYP71A16)	putative cytochrome P450
AT2G04680	*-1,122*		Cysteine/Histidine-rich C1 domain family protein; FUNCTIONS IN: zinc ion binding; EXPRESSED IN: root; CONTAINS InterPro DOMAIN/s: Zinc finger, RING-type (InterPro:IPR001841), DC1 (InterPro:IPR004146), Zinc finger, PHD-type (InterPro:IPR001965), C1-like (InterPro:IPR011424); BEST Arabidopsis thaliana protein match is: Cysteine/Histidine-rich C1 domain family protein (TAIR:AT5G37620.1)
AT5G23340	*-1,122*		RNI-like superfamily protein; CONTAINS InterPro DOMAIN/s: Leucine-rich repeat, cysteine-containing subtype (InterPro:IPR006553); BEST Arabidopsis thaliana protein match is: F-box/RNI-like superfamily protein (TAIR:AT4G15475.1)
AT1G23040	*-1,123*		hydroxyproline-rich glycoprotein family protein; BEST Arabidopsis thaliana protein match is: proline-rich family protein (TAIR:AT1G70990.1)
AT2G28710	*-1,123*		C2H2-type zinc finger family protein; FUNCTIONS IN: sequence-specific DNA binding transcription factor activity, zinc ion binding, nucleic acid binding; INVOLVED IN: regulation of transcription; LOCATED IN: intracellular; EXPRESSED IN: 8 plant structures; EXPRESSED DURING: 4 anthesis; CONTAINS InterPro DOMAIN/s: Zinc finger, C2H2-like (InterPro:IPR015880), Zinc finger, C2H2-type (InterPro:IPR007087); BEST Arabidopsis thaliana protein match is: C2H2-type zinc finger family protein (TAIR:AT5G59820.1)
AT1G04240	*-1,124*	SHORT HYPOCOTYL 2 (SHY2)	SHY2/IAA3 regulates multiple auxin responses in roots. It is induced rapidly by IAA, and has been shown to be phosphorylated by oat phytochrome A in vitro.
AT4G16190	*-1,125*		Papain family cysteine protease; FUNCTIONS IN: cysteine-type peptidase activity, cysteine-type endopeptidase activity; INVOLVED IN: proteolysis; LOCATED IN: vacuole; EXPRESSED IN: 23 plant structures; EXPRESSED DURING: 13 growth stages; CONTAINS InterPro DOMAIN/s: Peptidase C1A, papain (InterPro:IPR013128), Proteinase inhibitor I29, cathepsin propeptide (InterPro:IPR013201), Peptidase C1A, papain C-terminal (InterPro:IPR000668), Peptidase, cysteine peptidase active site (InterPro:IPR000169); BEST Arabidopsis thaliana protein match is: Papain family cysteine protease (TAIR:AT4G39090.1)
AT3G04810	*-1,126*	NIMA-RELATED KINASE 2 (NEK2)	Encodes AtNek2, a member of the NIMA-related serine/threonine kinases (Neks) that have been linked to cell-cycle regulation in fungi and mammals. Plant Neks might be involved in plant development processes.
AT3G23510	*-1,128*		Cyclopropane-fatty-acyl-phospholipid synthase; FUNCTIONS IN: cyclopropane-fatty-acyl-phospholipid synthase activity; INVOLVED IN: lipid biosynthetic process; LOCATED IN: endomembrane system; CONTAINS InterPro DOMAIN/s: Amine oxidase (InterPro:IPR002937), Cyclopropane-fatty-acyl-phospholipid/mycolic acid synthase (InterPro:IPR003333), Adrenodoxin reductase (InterPro:IPR000759); BEST Arabidopsis thaliana protein match is: Cyclopropane-fatty-acyl-phospholipid synthase (TAIR:AT3G23530.1)
AT4G26140	*-1,128*	BETA-GALACTOSIDASE 12 (BGAL12)	putative beta-galactosidase

AT4G32650	*-1,129*	POTASSIUM CHANNEL IN ARABIDOPSIS THALIANA 3 (KAT3)	Encodes KAT3, a member of the Shaker family of voltage-gated potassium channel subunits. Does not form functional potassium channel on its own. Involved in down-regulating AKT1 and KAT1 channel activity by forming heteromers with AKT1 or KAT1. The Shaker family K+ ion channels include five groups based on phylogenetic analysis (FEBS Letters (2007) 581: 2357): I (inwardly rectifying conductance): AKT1 (AT2G26650), AKT5 (AT4G32500) and SPIK (also known as AKT6, AT2G25600); II (inward rectifying channel): KAT1 (AT5G46240) and KAT2 (AT4G18290); III (weakly inward rectifying channel): AKT2 (AT4G22200); IV (regulatory subunit involved in inwardly rectifying conductance formation): KAT3 (also known as AtKC1, AT4G32650); V (outward rectifying channel): SKOR (AT3G02850) and GORK (AT5G37500).
AT4G29800	*-1,13*	PATATIN-LIKE PROTEIN 8 (PLP8)	PATATIN-like protein 8 (PLP8); INVOLVED IN: metabolic process, lipid metabolic process; LOCATED IN: cellular_component unknown; EXPRESSED IN: sperm cell, hypocotyl, root; CONTAINS InterPro DOMAIN/s: Acyl transferase/acyl hydrolase/lysophospholipase (InterPro:IPR016035), Patatin (InterPro:IPR002641); BEST Arabidopsis thaliana protein match is: patatin-like protein 6 (TAIR:AT3G54950.1).
AT2G34770	*-1,131*	FATTY ACID HYDROXYLASE 1 (FAH1)	encodes a fatty acid hydroxylase, required for the AtBI-1-mediated suppression of programmed cell death.
AT5G25820	*-1,131*		Exostosin family protein; FUNCTIONS IN: catalytic activity; LOCATED IN: membrane; EXPRESSED IN: stem, root, stamen; EXPRESSED DURING: 4 anthesis; CONTAINS InterPro DOMAIN/s: Exostosin-like (InterPro:IPR004263); BEST Arabidopsis thaliana protein match is: Exostosin family protein (TAIR:AT4G32790.1)
AT3G52820	*-1,132*	PURPLE ACID PHOSPHATASE 22 (PAP22)	purple acid phosphatase 22 (PAP22); FUNCTIONS IN: protein serine/threonine phosphatase activity, acid phosphatase activity; INVOLVED IN: biological_process unknown; LOCATED IN: endomembrane system; EXPRESSED IN: 12 plant structures; EXPRESSED DURING: 6 growth stages; CONTAINS InterPro DOMAIN/s: Metallophosphoesterase (InterPro:IPR004843), Purple acid phosphatase-like, N-terminal (InterPro:IPR008963); BEST Arabidopsis thaliana protein match is: purple acid phosphatase 21 (TAIR:AT3G52810.1)
AT1G29740	*-1,133*		Leucine-rich repeat transmembrane protein kinase; FUNCTIONS IN: kinase activity; INVOLVED IN: protein amino acid phosphorylation; LOCATED IN: endomembrane system; CONTAINS InterPro DOMAIN/s: Protein kinase, ATP binding site (InterPro:IPR017441), Serine/threonine-protein kinase domain (InterPro:IPR002290), Serine/threonine-protein kinase-like domain (InterPro:IPR017442), Serine/threonine-protein kinase, active site (InterPro:IPR008271), Protein kinase-like domain (InterPro:IPR011009), Malectin/receptor-like protein kinase (InterPro:IPR021720), Protein kinase, catalytic domain (InterPro:IPR000719), Tyrosine-protein kinase, catalytic domain (InterPro:IPR020635); BEST Arabidopsis thaliana protein match is: Leucine-rich repeat transmembrane protein kinase (TAIR:AT1G29730.1)
AT1G76800	*-1,133*	VACUOLAR IRON TRANSPORTER-LIKE 2 (VTL2)	The gene encodes nodulin-like2 whose transcript abundance was repressed under conditions of Fe-deficient growth.
AT2G45430	*-1,133*	AT-HOOK MOTIF NUCLEAR-LOCALIZED PROTEIN 22 (AHL22)	Encodes a nuclear localized AT hook domain containing protein that can bind AT rich DNA in vitro. Overexpression of the gene results in delayed flowering. Is likely to act redundantly with AHL18, AHL27 and AHL29 in the regulation of flowering. It is also involved in both photo- and skotomorphogenesis.
AT3G16560	*-1,133*		Protein phosphatase 2C family protein; FUNCTIONS IN: protein serine/threonine phosphatase activity, catalytic activity; EXPRESSED IN: 24 plant structures; EXPRESSED DURING: 15 growth stages; CONTAINS InterPro DOMAIN/s: Protein phosphatase 2C-related (InterPro:IPR001932), Protein phosphatase 2C (InterPro:IPR015655), Protein phosphatase 2C, N-terminal (InterPro:IPR014045); BEST Arabidopsis thaliana protein match is: pol-like 5 (TAIR:AT1G07630.1)
AT1G33240	*-1,134*	GT-2-LIKE 1 (GTL1)	Encodes a plant transcriptional activator that contains two separate, but similar, trihelix DNA-binding domains, similar to GT-2. Gene is expressed in all aerial parts of the plant, with higher level of expression in siliques. At-GTL2 was thought to be a duplicated copy of this gene but is likely to be a cloning artefact, the result of a chimeric clone. Regulates ploidy-dependent cell growth in trichome.
AT1G14860	*-1,136*	NUDIX HYDROLASE HOMOLOG 18 (NUDT18)	nudix hydrolase homolog 18 (NUDT18); FUNCTIONS IN: hydrolase activity; INVOLVED IN: biological_process unknown; CONTAINS InterPro DOMAIN/s: NUDIX hydrolase domain-like (InterPro:IPR015797), NUDIX hydrolase, conserved site (InterPro:IPR020084), NUDIX hydrolase domain (InterPro:IPR000086); BEST Arabidopsis thaliana protein match is: nudix hydrolase homolog 17 (TAIR:AT2G01670.1)

AT2G39050	*-1,136*	EUONYMUS LECTIN S3 (EULS3)	Encodes a nucleocytoplasmic lectin that is capable of binding carbohydrates. It is involved in ABA mediated stomatal movement and increased expression is correlated with increased resistance to Pseudomonas syringae.
AT1G23440	*-1,137*		Peptidase C15, pyroglutamyl peptidase I-like; FUNCTIONS IN: peptidase activity; INVOLVED IN: proteolysis; LOCATED IN: cellular_component unknown; EXPRESSED IN: 22 plant structures; EXPRESSED DURING: 13 growth stages; CONTAINS InterPro DOMAIN/s: Peptidase C15, pyroglutamyl peptidase I (InterPro:IPR000816), Peptidase C15, pyroglutamyl peptidase I-like (InterPro:IPR016125); BEST Arabidopsis thaliana protein match is: Peptidase C15, pyroglutamyl peptidase I-like (TAIR:AT1G56700.2)
AT1G47480	*-1,137*		alpha/beta-Hydrolases superfamily protein; FUNCTIONS IN: hydrolase activity; INVOLVED IN: metabolic process; LOCATED IN: cellular_component unknown; EXPRESSED IN: stem, hypocotyl, root, flower; EXPRESSED DURING: petal differentiation and expansion stage; CONTAINS InterPro DOMAIN/s: Lipase, GDXG, active site (InterPro:IPR002168), Alpha/beta hydrolase fold-3 (InterPro:IPR013094); BEST Arabidopsis thaliana protein match is: carboxyesterase 13 (TAIR:AT3G48700.1)
AT1G51270	*-1,137*		structural molecules;transmembrane receptors;structural molecules; FUNCTIONS IN: transmembrane receptor activity, structural molecule activity; INVOLVED IN: signal transduction, innate immune response; LOCATED IN: intrinsic to membrane; EXPRESSED IN: 22 plant structures; EXPRESSED DURING: 12 growth stages; CONTAINS InterPro DOMAIN/s: PapD-like (InterPro:IPR008962), Major sperm protein (InterPro:IPR000535), Toll-Interleukin receptor (InterPro:IPR000157); BEST Arabidopsis thaliana protein match is: Disease resistance protein (TIR-NBS-LRR class) family (TAIR:AT5G45000.1)
AT5G15900	*-1,137*	TRICHOME BIREFRINGENCE-LIKE 19 (TBL19)	Encodes a member of the TBL (TRICHOME BIREFRINGENCE-LIKE) gene family containing a plant-specific DUF231 (domain of unknown function) domain. TBL gene family has 46 members, two of which (TBR/AT5G06700 and TBL3/AT5G01360) have been shown to be involved in the synthesis and deposition of secondary wall cellulose, presumably by influencing the esterification state of pectic polymers. A nomenclature for this gene family has been proposed (Volker Bischoff & Wolf Scheible, 2010, personal communication).
AT5G58940	*-1,137*	CALMODULIN-BINDING RECEPTOR-LIKE CYTOPLASMIC KINASE 1 (CRCK1)	Arabidopsis thaliana calmodulin-binding receptor-like kinase mRNA The mRNA is cell-to-cell mobile.
AT5G14000	*-1,138*	NAC DOMAIN CONTAINING PROTEIN 84 (NAC084)	NAC domain containing protein 84 (NAC084); FUNCTIONS IN: sequence-specific DNA binding transcription factor activity; INVOLVED IN: multicellular organismal development, regulation of transcription; LOCATED IN: cellular_component unknown; EXPRESSED IN: 7 plant structures; EXPRESSED DURING: 4 anthesis, C globular stage, petal differentiation and expansion stage; CONTAINS InterPro DOMAIN/s: No apical meristem (NAM) protein (InterPro:IPR003441); BEST Arabidopsis thaliana protein match is: NAC domain containing protein 83 (TAIR:AT5G13180.1)
AT3G48530	*-1,141*	SNF1-RELATED PROTEIN KINASE REGULATORY SUBUNIT GAMMA 1 (KING1)	SNF1-related protein kinase regulatory subunit gamma 1 (KING1); FUNCTIONS IN: molecular_function unknown; INVOLVED IN: biological_process unknown; LOCATED IN: plant-type cell wall; EXPRESSED IN: 23 plant structures; EXPRESSED DURING: 13 growth stages; CONTAINS InterPro DOMAIN/s: Cystathionine beta-synthase, core (InterPro:IPR000644); BEST Arabidopsis thaliana protein match is: Cystathionine beta-synthase (CBS) protein (TAIR:AT1G69800.1)
AT4G02050	*-1,142*	SUGAR TRANSPORTER PROTEIN 7 (STP7)	sugar transporter protein 7 (STP7); FUNCTIONS IN: carbohydrate transmembrane transporter activity, sugar:hydrogen symporter activity; INVOLVED IN: transport, transmembrane transport; LOCATED IN: plasma membrane, membrane; EXPRESSED IN: 22 plant structures; EXPRESSED DURING: 13 growth stages; CONTAINS InterPro DOMAIN/s: Sugar transporter, conserved site (InterPro:IPR005829), Major facilitator superfamily (InterPro:IPR020846), General substrate transporter (InterPro:IPR005828), Sugar/inositol transporter (InterPro:IPR003663), Major facilitator superfamily, general substrate transporter (InterPro:IPR016196); BEST Arabidopsis thaliana protein match is: Major facilitator superfamily protein (TAIR:AT5G26340.1)
AT4G03480	*-1,142*		Ankyrin repeat family protein; INVOLVED IN: biological_process unknown; EXPRESSED IN: cultured cell; CONTAINS InterPro DOMAIN/s: Ankyrin repeat-containing domain (InterPro:IPR020683), Ankyrin repeat (InterPro:IPR002110); BEST Arabidopsis thaliana protein match is: Ankyrin repeat family protein (TAIR:AT4G03440.1)

AT3G50770	*-1,143*	CALMODULIN-LIKE 41 (CML41)	calmodulin-like 41 (CML41); FUNCTIONS IN: calcium ion binding; INVOLVED IN: biological_process unknown; LOCATED IN: chloroplast; EXPRESSED IN: 17 plant structures; EXPRESSED DURING: 11 growth stages; CONTAINS InterPro DOMAIN/s: EF-Hand 1, calcium-binding site (InterPro:IPR018247), EF-HAND 2 (InterPro:IPR018249), EF-hand-like domain (InterPro:IPR011992), Calcium-binding EF-hand (InterPro:IPR002048), EF-hand (InterPro:IPR018248); BEST Arabidopsis thaliana protein match is: calmodulin-like 38 (TAIR:AT1G76650.3)
AT5G59070	*-1,143*		UDP-Glycosyltransferase superfamily protein; FUNCTIONS IN: transferase activity, transferring glycosyl groups; INVOLVED IN: biosynthetic process; LOCATED IN: endomembrane system; CONTAINS InterPro DOMAIN/s: Glycosyl transferase, group 1 (InterPro:IPR001296); BEST Arabidopsis thaliana protein match is: UDP-Glycosyltransferase superfamily protein (TAIR:AT4G19460.1)
AT1G10400	*-1,144*		UDP-Glycosyltransferase superfamily protein; FUNCTIONS IN: UDP-glycosyltransferase activity, transferase activity, transferring glycosyl groups; INVOLVED IN: metabolic process; LOCATED IN: endomembrane system; EXPRESSED IN: root; CONTAINS InterPro DOMAIN/s: UDP-glucuronosyl/UDP-glucosyltransferase (InterPro:IPR002213), Tudor subgroup (InterPro:IPR018351); BEST Arabidopsis thaliana protein match is: UDP-Glycosyltransferase superfamily protein (TAIR:AT5G14860.1)
AT5G52830	*-1,144*	WRKY DNA-BINDING PROTEIN 27 (WRKY27)	Encodes a WRKY transcription factor WRKY27. Mutation in Arabidopsis WRKY27 results in delayed symptom development in response to the bacterial wilt pathogen Ralstonia solanacearum.
AT1G16110	*-1,146*	WALL ASSOCIATED KINASE-LIKE 6 (WAKL6)	Encodes a WAK-like receptor-like kinase with a cytoplasmic Ser/Thr protein kinase domain and an extracellular domain with EGF-like repeats. It has been shown to be localized to the cell wall.
AT2G27010	*-1,147*	CYTOCHROME P450, FAMILY 705, SUBFAMILY A, POLYPEPTIDE 9 (CYP705A9)	member of CYP705A
AT2G36320	*-1,148*		A20/AN1-like zinc finger family protein; FUNCTIONS IN: DNA binding, zinc ion binding; INVOLVED IN: biological_process unknown; LOCATED IN: cellular_component unknown; EXPRESSED IN: 25 plant structures; EXPRESSED DURING: 15 growth stages; CONTAINS InterPro DOMAIN/s: Zinc finger, AN1-type (InterPro:IPR000058), Zinc finger, A20-type (InterPro:IPR002653); BEST Arabidopsis thaliana protein match is: A20/AN1-like zinc finger family protein (TAIR:AT3G52800.1)
AT5G25240	*-1,148*		unknown protein; FUNCTIONS IN: molecular_function unknown; INVOLVED IN: biological_process unknown; LOCATED IN: cellular_component unknown; EXPRESSED IN: 23 plant structures; EXPRESSED DURING: 13 growth stages
AT5G48090	*-1,148*	EDM2-LIKE PROTEIN1 (ELP1)	EDM2-like protein1 (ELP1); BEST Arabidopsis thaliana protein match is: ENHANCED DOWNY MILDEW 2 (TAIR:AT5G55390.2)
AT1G03220	*-1,149*		Eukaryotic aspartyl protease family protein; FUNCTIONS IN: aspartic-type endopeptidase activity; INVOLVED IN: response to salt stress; LOCATED IN: cell wall, plasma membrane, membrane, plant-type cell wall; EXPRESSED IN: 6 plant structures; CONTAINS InterPro DOMAIN/s: Peptidase aspartic (InterPro:IPR021109), Peptidase aspartic, catalytic (InterPro:IPR009007), Peptidase A1 (InterPro:IPR001461); BEST Arabidopsis thaliana protein match is: Eukaryotic aspartyl protease family protein (TAIR:AT1G03230.1)
AT1G08340	*-1,15*		Rho GTPase activating protein with PAK-box/P21-Rho-binding domain; FUNCTIONS IN: Rac GTPase activator activity; INVOLVED IN: signal transduction; LOCATED IN: intracellular; EXPRESSED IN: 23 plant structures; EXPRESSED DURING: 13 growth stages; CONTAINS InterPro DOMAIN/s: PAK-box/P21-Rho-binding (InterPro:IPR000095), Rho GTPase activation protein (InterPro:IPR008936), RhoGAP (InterPro:IPR000198); BEST Arabidopsis thaliana protein match is: Rho GTPase activating protein with PAK-box/P21-Rho-binding domain (TAIR:AT5G22400.1)
AT2G35000	*-1,15*	ARABIDOPSIS TOXICOS EN LEVADURA 9 (ATL9)	E3 ligase-like protein induced by chitin oligomers.
AT2G36380	*-1,152*	ATP-BINDING CASSETTE G34 (ABCG34)	pleiotropic drug resistance 6 (PDR6); FUNCTIONS IN: ATPase activity, coupled to transmembrane movement of substances; LOCATED IN: plasma membrane; EXPRESSED IN: 24 plant structures; EXPRESSED DURING: 13 growth stages; CONTAINS InterPro DOMAIN/s: ATPase, AAA+ type, core (InterPro:IPR003593), ABC transporter-like (InterPro:IPR003439), Plant PDR ABC transporter associated (InterPro:IPR013581), ABC-2 type transporter (InterPro:IPR013525); BEST Arabidopsis thaliana protein match is: pleiotropic drug resistance 11 (TAIR:AT1G66950.1)

AT5G01520	*-1,152*	ABA INSENSITIVE RING PROTEIN 2 (AIRP2)	RING/U-box superfamily protein; FUNCTIONS IN: zinc ion binding; EXPRESSED IN: 17 plant structures; EXPRESSED DURING: 8 growth stages; CONTAINS InterPro DOMAIN/s: Zinc finger, RING-type, conserved site (InterPro:IPR017907), Zinc finger, RING-type (InterPro:IPR001841), Zinc finger, C3HC4 RING-type (InterPro:IPR018957); BEST Arabidopsis thaliana protein match is: RING/U-box superfamily protein (TAIR:AT3G47160.1)
AT5G05840	*-1,152*		Protein of unknown function (DUF620); CONTAINS InterPro DOMAIN/s: Protein of unknown function DUF620 (InterPro:IPR006873); BEST Arabidopsis thaliana protein match is: Protein of unknown function (DUF620) (TAIR:AT3G55720.1)
AT2G27430	*-1,153*		ARM repeat superfamily protein; FUNCTIONS IN: binding; INVOLVED IN: biological_process unknown; LOCATED IN: cellular_component unknown; CONTAINS InterPro DOMAIN/s: Armadillo-like helical (InterPro:IPR011989), Armadillo-type fold (InterPro:IPR016024); BEST Arabidopsis thaliana protein match is: ARM repeat superfamily protein (TAIR:AT4G31890.2)
AT5G45070	*-1,153*	PHLOEM PROTEIN 2-A8 (PP2-A8)	phloem protein 2-A8 (PP2-A8); FUNCTIONS IN: carbohydrate binding; INVOLVED IN: signal transduction, defense response, innate immune response; LOCATED IN: intrinsic to membrane; EXPRESSED IN: stem, root; CONTAINS InterPro DOMAIN/s: Toll-Interleukin receptor (InterPro:IPR000157); BEST Arabidopsis thaliana protein match is: phloem protein 2-A6 (TAIR:AT5G45080.1)
AT1G75520	*-1,154*	SHI-RELATED SEQUENCE 5 (SRS5)	A member of SHI gene family. Arabidopsis thaliana has ten members that encode proteins with a RING finger-like zinc finger motif. Despite being highly divergent in sequence, many of the SHI-related genes are partially redundant in function and synergistically promote gynoecium, stamen and leaf development in Arabidopsis.
AT4G11050	*-1,154*	GLYCOSYL HYDROLASE 9C3 (GH9C3)	glycosyl hydrolase 9C3 (GH9C3); FUNCTIONS IN: carbohydrate binding, hydrolase activity, hydrolyzing O-glycosyl compounds, catalytic activity; INVOLVED IN: carbohydrate metabolic process; LOCATED IN: endomembrane system, extracellular region; EXPRESSED IN: 7 plant structures; EXPRESSED DURING: 4 anthesis, E expanded cotyledon stage, D bilateral stage; CONTAINS InterPro DOMAIN/s: Six-hairpin glycosidase (InterPro:IPR012341), Carbohydrate-binding (InterPro:IPR008965), Glycoside hydrolase, family 9, active site (InterPro:IPR018221), Six-hairpin glycosidase-like (InterPro:IPR008928), Glycoside hydrolase, family 9 (InterPro:IPR001701), Carbohydrate binding domain CBM49 (InterPro:IPR019028); BEST Arabidopsis thaliana protein match is: glycosyl hydrolase 9C2 (TAIR:AT1G64390.1)
AT5G05880	*-1,154*	UDP-GLUCOSYL TRANSFERASE 76C4 (UGT76C4)	Encodes a nicotinate-N-glycosyltransferase.
AT5G38200	*-1,155*		Class I glutamine amidotransferase-like superfamily protein; FUNCTIONS IN: hydrolase activity; INVOLVED IN: glutamine metabolic process; LOCATED IN: cellular_component unknown; CONTAINS InterPro DOMAIN/s: Peptidase C26 (InterPro:IPR011697), Glutamine amidotransferase type 1 (InterPro:IPR017926); BEST Arabidopsis thaliana protein match is: Class I glutamine amidotransferase-like superfamily protein (TAIR:AT1G66860.1)
AT1G67910	*-1,156*		unknown protein; FUNCTIONS IN: molecular_function unknown; INVOLVED IN: biological_process unknown; LOCATED IN: chloroplast; EXPRESSED IN: 21 plant structures; EXPRESSED DURING: 12 growth stages; BEST Arabidopsis thaliana protein match is: unknown protein (TAIR:AT1G24577.1)
AT3G06420	*-1,156*	AUTOPHAGY 8H (ATG8H)	autophagy 8h (ATG8H); CONTAINS InterPro DOMAIN/s: Light chain 3 (LC3) (InterPro:IPR004241); BEST Arabidopsis thaliana protein match is: Ubiquitin-like superfamily protein (TAIR:AT3G15580.1)
AT5G48920	*-1,156*	TRACHEARY ELEMENT DIFFERENTIATION-RELATED 7 (TED7)	tracheary element differentiation-related 7 (TED7); BEST Arabidopsis thaliana protein match is: tracheary element differentiation-related 6 (TAIR:AT1G43790.1)
AT5G65380	*-1,156*		MATE efflux family protein; FUNCTIONS IN: antiporter activity, drug transmembrane transporter activity, transporter activity; INVOLVED IN: drug transmembrane transport, ripening, transmembrane transport; LOCATED IN: membrane; EXPRESSED IN: 22 plant structures; EXPRESSED DURING: 13 growth stages; CONTAINS InterPro DOMAIN/s: Multi antimicrobial extrusion protein MatE (InterPro:IPR002528); BEST Arabidopsis thaliana protein match is: MATE efflux family protein (TAIR:AT5G44050.1)

AT4G25090	*-1,157*		Riboflavin synthase-like superfamily protein; FUNCTIONS IN: in 7 functions; INVOLVED IN: oxidation reduction; LOCATED IN: cytosolic ribosome, vacuole; EXPRESSED IN: root; CONTAINS InterPro DOMAIN/s: Ferredoxin reductase-type FAD-binding domain (InterPro:IPR017927), Cytochrome b245, heavy chain (InterPro:IPR000778), EF-Hand 1, calcium-binding site (InterPro:IPR018247), EF-hand-like domain (InterPro:IPR011992), Ferric reductase-like transmembrane component, N-terminal (InterPro:IPR013130), Ferric reductase, NAD binding (InterPro:IPR013121), NADPH oxidase Respiratory burst (InterPro:IPR013623), EF-HAND 2 (InterPro:IPR018249), FAD-binding 8 (InterPro:IPR013112), Riboflavin synthase-like beta-barrel (InterPro:IPR017938); BEST Arabidopsis thaliana protein match is: NADPH/respiratory burst oxidase protein D (TAIR:AT5G51060.1)
AT5G41050	*-1,157*		Pollen Ole e 1 allergen and extensin family protein; FUNCTIONS IN: molecular_function unknown; INVOLVED IN: biological_process unknown; LOCATED IN: endomembrane system; EXPRESSED IN: 22 plant structures; EXPRESSED DURING: 13 growth stages; CONTAINS InterPro DOMAIN/s: Pollen Ole e 1 allergen/extensin (InterPro:IPR006041); BEST Arabidopsis thaliana protein match is: Pollen Ole e 1 allergen and extensin family protein (TAIR:AT3G26960.1)
AT1G13600	*-1,158*	BASIC LEUCINE-ZIPPER 58 (bZIP58)	basic leucine-zipper 58 (bZIP58); FUNCTIONS IN: DNA binding, protein homodimerization activity, protein heterodimerization activity, sequence-specific DNA binding transcription factor activity; INVOLVED IN: regulation of transcription, DNA-dependent; EXPRESSED IN: 16 plant structures; EXPRESSED DURING: 7 growth stages; CONTAINS InterPro DOMAIN/s: Basic-leucine zipper (bZIP) transcription factor (InterPro:IPR004827), bZIP transcription factor, bZIP-1 (InterPro:IPR011616); BEST Arabidopsis thaliana protein match is: basic leucine-zipper 48 (TAIR:AT2G04038.1)
AT1G27110	*-1,158*		Tetratricopeptide repeat (TPR)-like superfamily protein; FUNCTIONS IN: binding; INVOLVED IN: biological_process unknown; LOCATED IN: cellular_component unknown; CONTAINS InterPro DOMAIN/s: Tetratricopeptide-like helical (InterPro:IPR011990); BEST Arabidopsis thaliana protein match is: Tetratricopeptide repeat (TPR)-like superfamily protein (TAIR:AT1G27150.1)
AT5G63800	*-1,158*	MUCILAGE-MODIFIED 2 (MUM2)	Involved in mucilage formation. Mutants form columella and outer cell wall architecture of the mucilage cells resembles wild-type. However, mum2 seeds completely lack seed coat mucilage. This mutation appears to represent a later step in the development of this cell-type. Encodes a beta-galactosidase involved in seed coat mucilage biosynthesis. Member of Glycoside Hydrolase Family 35
AT5G15580	*-1,159*	LONGIFOLIA1 (LNG1)	Encodes LONGIFOLIA1 (LNG1). Regulates leaf morphology by promoting cell expansion in the leaf-length direction. The LNG1 homologue LNG2 (At3g02170) has similar function.
AT5G02400	*-1,161*	POL-LIKE 2 (PLL2)	Encodes a protein with similarity to the POL locus which is a novel protein phosphatase 2C. Ubiquitously expressed. No phenotype observed in homozygous null mutant background.
AT4G22230	*-1,165*		Encodes a defensin-like (DEFL) family protein.
AT1G76890	*-1,166*	(GT2)	encodes a plant trihelix DNA-binding protein
AT1G02610	*-1,167*		RING/FYVE/PHD zinc finger superfamily protein; FUNCTIONS IN: zinc ion binding; CONTAINS InterPro DOMAIN/s: Protein of unknown function DUF3675 (InterPro:IPR022143), Zinc finger, C3HC4 RING-type (InterPro:IPR018957), Zinc finger, RING-CH-type (InterPro:IPR011016); BEST Arabidopsis thaliana protein match is: RING/FYVE/PHD zinc finger superfamily protein (TAIR:AT4G02075.1)
AT2G24070	*-1,167*	QWRF DOMAIN CONTAINING 4 (QWRF4)	Family of unknown function (DUF566) ; LOCATED IN: chloroplast; EXPRESSED IN: shoot apex, inflorescence meristem, root, flower, carpel; EXPRESSED DURING: petal differentiation and expansion stage; CONTAINS InterPro DOMAIN/s: Protein of unknown function DUF566 (InterPro:IPR007573); BEST Arabidopsis thaliana protein match is: Family of unknown function (DUF566) (TAIR:AT4G30710.1)
AT3G26500	*-1,167*	PLANT INTRACELLULAR RAS GROUP-RELATED LRR 2 (PIRL2)	Encodes PIRL2, a member of the Plant Intracellular Ras-group-related LRRs (Leucine rich repeat proteins). PIRLs are a distinct, plant-specific class of intracellular LRRs that likely mediate protein interactions, possibly in the context of signal transduction.
AT4G39780	*-1,169*		encodes a member of the DREB subfamily A-6 of ERF/AP2 transcription factor family. The protein contains one AP2 domain. There are 8 members in this subfamily including RAP2.4.
AT3G52450	*-1,17*	PLANT U-BOX 22 (PUB22)	Encodes a cytoplasmically localized U-box domain E3 ubiquitin ligase protein that is involved in the response to water stress and acts as a negative regulator of PAMP-triggered immunity.

AT1G69810	*-1,173*	WRKY DNA-BINDING PROTEIN 36 (WRKY36)	member of WRKY Transcription Factor; Group II-b
AT1G78955	*-1,173*	CAMELLIOL C SYNTHASE 1 (CAMS1)	Encodes a cyclase that generates predominantly a monocyclic triterpene alcohol. The product is 97% camelliol, 2% achilleol A and 0.2% beta-amyrin. Achilleol is an isomer of camelliol C with a 4-methylenecyclohexanol ring system.
AT2G33810	*-1,173*	SQUAMOSA PROMOTER BINDING PROTEIN-LIKE 3 (SPL3)	Encodes a member of the SPL (squamosa-promoter binding protein-like)gene family, a novel gene family encoding DNA binding proteins and putative transcription factors. Contains the SBP-box, which encodes the SBP-domain, required and sufficient for interaction with DNA. It binds DNA, may directly regulate AP1, and is involved in regulation of flowering and vegetative phase change. Its temporal expression is regulated by the microRNA miR156. The target site for the microRNA is in the 3'UTR.
AT2G31570	*-1,174*	GLUTATHIONE PEROXIDASE 2 (GPX2)	glutathione peroxidase GPx
AT4G14060	*-1,174*		Polyketide cyclase/dehydrase and lipid transport superfamily protein; FUNCTIONS IN: molecular_function unknown; INVOLVED IN: response to biotic stimulus, defense response; LOCATED IN: cellular_component unknown; EXPRESSED IN: cotyledon, root; CONTAINS InterPro DOMAIN/s: Bet v I allergen (InterPro:IPR000916); BEST Arabidopsis thaliana protein match is: MLP-like protein 328 (TAIR:AT2G01520.1)
AT1G24620	*-1,175*	CALMODULIN LIKE 25 (CML25)	Encodes a EF-hand calcium-binding protein family member. Mutants exhibit longer root hairs under phosphate-deficient conditions.
AT5G60020	*-1,175*	LACCASE 17 (LAC17)	LAC17 appears to have laccase activity based on enzyme assays performed using lac17 mutants. Notably, these mutants appear to have a reduced deposition of G lignin units. LAC17 is expressed in interfascicular fibers and likely contributes to lignin biosynthesis, and hence, cell wall biosynthesis, there.
AT5G06570	*-1,176*		alpha/beta-Hydrolases superfamily protein; FUNCTIONS IN: hydrolase activity; INVOLVED IN: metabolic process; LOCATED IN: cellular_component unknown; EXPRESSED IN: 6 plant structures; EXPRESSED DURING: petal differentiation and expansion stage; CONTAINS InterPro DOMAIN/s: Alpha/beta hydrolase fold-3 (InterPro:IPR013094); BEST Arabidopsis thaliana protein match is: carboxyesterase 17 (TAIR:AT5G16080.1)
AT1G34050	*-1,178*		Ankyrin repeat family protein; CONTAINS InterPro DOMAIN/s: Ankyrin repeat-containing domain (InterPro:IPR020683), Ankyrin repeat (InterPro:IPR002110); BEST Arabidopsis thaliana protein match is: Ankyrin repeat family protein (TAIR:AT5G54710.1)
AT5G44910	*-1,178*		Toll-Interleukin-Resistance (TIR) domain family protein; FUNCTIONS IN: transmembrane receptor activity; INVOLVED IN: signal transduction, defense response, innate immune response; LOCATED IN: intrinsic to membrane; EXPRESSED IN: hypocotyl, root; CONTAINS InterPro DOMAIN/s: Toll-Interleukin receptor (InterPro:IPR000157); BEST Arabidopsis thaliana protein match is: Disease resistance protein (TIR-NBS-LRR class) family (TAIR:AT5G45000.1)
AT3G04110	*-1,179*	GLUTAMATE RECEPTOR 1.1 (GLR1.1)	putative glutamate receptor (GLR1.1). Contains a functional cation - permeable pore domain. Involved in cellular cation homeostasis.
AT1G75390	*-1,18*	BASIC LEUCINE-ZIPPER 44 (bZIP44)	basic leucine-zipper 44 (bZIP44); FUNCTIONS IN: DNA binding, protein heterodimerization activity, sequence-specific DNA binding transcription factor activity; INVOLVED IN: regulation of transcription, DNA-dependent; LOCATED IN: chloroplast; CONTAINS InterPro DOMAIN/s: Basic-leucine zipper (bZIP) transcription factor (InterPro:IPR004827), bZIP transcription factor, bZIP-1 (InterPro:IPR011616); BEST Arabidopsis thaliana protein match is: G-box binding factor 6 (TAIR:AT4G34590.1)
AT2G14210	*-1,181*	AGAMOUS-LIKE 44 (AGL44)	MADS box gene, transcription factor
AT2G41070	*-1,181*	ENHANCED EM LEVEL (EEL)	Transcription factor homologous to ABI5. Regulates AtEm1 expression by binding directly at the AtEm1 promoter. Located in the nucleus and expressed during seed maturation in the cotyledons and later in the whole embryo.
AT3G05800	*-1,182*	ATBS1(ACTIVATION-TAGGED BRI1 SUPPRESSOR 1)-INTERACTING FACTOR 1 (AIF1)	AtBS1(activation-tagged BRI1 suppressor 1)-interacting factor 1 (AIF1); BEST Arabidopsis thaliana protein match is: basic helix-loop-helix (bHLH) DNA-binding superfamily protein (TAIR:AT1G09250.1)

AT1G52560	*-1,183*		HSP20-like chaperones superfamily protein; FUNCTIONS IN: molecular_function unknown; INVOLVED IN: response to high light intensity, response to hydrogen peroxide, response to heat; LOCATED IN: mitochondrion; EXPRESSED IN: 6 plant structures; EXPRESSED DURING: 4 anthesis, petal differentiation and expansion stage; CONTAINS InterPro DOMAIN/s: Heat shock protein Hsp20 (InterPro:IPR002068), HSP20-like chaperone (InterPro:IPR008978); BEST Arabidopsis thaliana protein match is: heat shock protein 21 (TAIR:AT4G27670.1)
AT2G14900	*-1,183*		Gibberellin-regulated family protein; FUNCTIONS IN: molecular_function unknown; INVOLVED IN: response to gibberellin stimulus; LOCATED IN: endomembrane system; EXPRESSED IN: 18 plant structures; EXPRESSED DURING: 12 growth stages; CONTAINS InterPro DOMAIN/s: Gibberellin regulated protein (InterPro:IPR003854); BEST Arabidopsis thaliana protein match is: Gibberellin-regulated family protein (TAIR:AT5G59845.1)
AT4G33420	*-1,183*		Peroxidase superfamily protein; FUNCTIONS IN: peroxidase activity, heme binding; INVOLVED IN: response to oxidative stress, oxidation reduction; EXPRESSED IN: 20 plant structures; EXPRESSED DURING: 10 growth stages; CONTAINS InterPro DOMAIN/s: Haem peroxidase (InterPro:IPR010255), Plant peroxidase (InterPro:IPR000823), Peroxidases heam-ligand binding site (InterPro:IPR019793), Haem peroxidase, plant/fungal/bacterial (InterPro:IPR002016), Peroxidase, active site (InterPro:IPR019794); BEST Arabidopsis thaliana protein match is: Peroxidase superfamily protein (TAIR:AT5G51890.1)
AT2G02610	*-1,185*		Cysteine/Histidine-rich C1 domain family protein; CONTAINS InterPro DOMAIN/s: DC1 (InterPro:IPR004146), C1-like (InterPro:IPR011424); BEST Arabidopsis thaliana protein match is: Cysteine/Histidine-rich C1 domain family protein (TAIR:AT2G02640.1)
AT1G24440	*-1,186*		RING/U-box superfamily protein; FUNCTIONS IN: zinc ion binding; EXPRESSED IN: 24 plant structures; EXPRESSED DURING: 15 growth stages; CONTAINS InterPro DOMAIN/s: Zinc finger, RING-type, conserved site (InterPro:IPR017907), Zinc finger, RING-type (InterPro:IPR001841); BEST Arabidopsis thaliana protein match is: RING/U-box superfamily protein (TAIR:AT1G13195.1)
AT1G29280	*-1,186*	WRKY DNA-BINDING PROTEIN 65 (WRKY65)	member of WRKY Transcription Factor; Group II-e The mRNA is cell-to-cell mobile.
AT1G66200	*-1,186*	GLUTAMINE SYNTHASE CLONE F11 (GSR2)	encodes a cytosolic glutamate synthetase, this enzyme has low affinity with substrate ammonium
AT1G04330	*-1,187*		unknown protein; FUNCTIONS IN: molecular_function unknown; INVOLVED IN: biological_process unknown; LOCATED IN: chloroplast; BEST Arabidopsis thaliana protein match is: unknown protein (TAIR:AT3G23170.1)
AT1G27380	*-1,187*	ROP-INTERACTIVE CRIB MOTIF-CONTAINING PROTEIN 2 (RIC2)	encodes a member of a novel protein family that contains contain a CRIB (for Cdc42/Rac-interactive binding) motif required for their specific interaction with GTP-bound Rop1 (plant-specific Rho GTPase). Interacts with Rop1 and is involved in pollen tube growth and function. Protein most similar to RIC4 (family subgroup V). Gene is expressed in all tissues examined.
AT1G60050	*-1,187*	USUALLY MULTIPLE ACIDS MOVE IN AND OUT TRANSPORTERS 35 (UMAMIT35)	nodulin MtN21-like transporter family protein
AT2G27360	*-1,188*		GDSL-motif esterase/acyltransferase/lipase. Enzyme group with broad substrate specificity that may catalyze acyltransfer or hydrolase reactions with lipid and non-lipid substrates.
AT1G61750	*-1,189*		Receptor-like protein kinase-related family protein; FUNCTIONS IN: molecular_function unknown; INVOLVED IN: biological_process unknown; LOCATED IN: endomembrane system; EXPRESSED IN: root; CONTAINS InterPro DOMAIN/s: Protein of unknown function DUF26 (InterPro:IPR002902); BEST Arabidopsis thaliana protein match is: Receptor-like protein kinase-related family protein (TAIR:AT1G63590.1)
AT2G44790	*-1,189*	UCLACYANIN 2 (UCC2)	Encodes a uclacyanin, a protein precursor that is closely related to precursors of stellacyanins and a blue copper protein from pea pods.
AT1G06160	*-1,191*	OCTADECANOID-RESPONSIVE ARABIDOPSIS AP2/ERF 59 (ORA59)	encodes a member of the ERF (ethylene response factor) subfamily B-3 of ERF/AP2 transcription factor family. The protein contains one AP2 domain. There are 18 members in this subfamily including ATERF-1, ATERF-2, AND ATERF-5.
AT4G23550	*-1,191*	(WRKY29)	Encodes WRKY DNA-binding protein 29 (WRKY29). The mRNA is cell-to-cell mobile.

AT5G40890	-1,193	CHLORIDE CHANNEL A (CLC-A)	Encodes a member of the voltage-dependent chloride channel. Also functions as a NO3-/H+ exchanger that serves to accumulate nitrate nutrient in vacuoles. Mutants homozygous for the T-DNA insertion mutation have reduced nitrate uptake capacity in high nitrate environment and exhibit hypersensitivity to chlorate.
AT1G35350	-1,194		EXS (ERD1/XPR1/SYG1) family protein; LOCATED IN: integral to membrane; EXPRESSED IN: 18 plant structures; EXPRESSED DURING: 9 growth stages; CONTAINS InterPro DOMAIN/s: EXS, C-terminal (InterPro:IPR004342), SPX, N-terminal (InterPro:IPR004331); BEST Arabidopsis thaliana protein match is: EXS (ERD1/XPR1/SYG1) family protein (TAIR:AT1G26730.1)
AT3G05390	-1,194		FUNCTIONS IN: molecular_function unknown; INVOLVED IN: biological_process unknown; LOCATED IN: mitochondrion; EXPRESSED IN: 15 plant structures; EXPRESSED DURING: 7 growth stages; CONTAINS InterPro DOMAIN/s: Protein of unknown function DUF248, methyltransferase putative (InterPro:IPR004159); BEST Arabidopsis thaliana protein match is: S-adenosyl-L-methionine-dependent methyltransferases superfamily protein (TAIR:AT4G01240.1)
AT4G12330	-1,194	CYTOCHROME P450, FAMILY 706, SUBFAMILY A, POLYPEPTIDE 7 (CYP706A7)	member of CYP706A
AT5G46790	-1,194	PYR1-LIKE 1 (PYL1)	Encodes a member of the PYR (pyrabactin resistance)/PYL(PYR1-like)/RCAR (regulatory components of ABA receptor) family proteins with 14 members. PYR/PYL/RCAR family proteins function as abscisic acid sensors. Mediate ABA-dependent regulation of protein phosphatase 2Cs ABI1 and ABI2.
AT4G15340	-1,196	PENTACYCLIC TRITERPENE SYNTHASE 1 (PEN1)	Encodes a protein that catalyzes the production of the tricyclic triterpene arabidiol when expressed in yeast.
AT4G30290	-1,196	XYLOGLUCAN ENDOTRANSGLUCOSYLASE/HYDROLASE 19 (XTH19)	Encodes a xyloglucan endotransglucosylase/hydrolase with only only the endotransglucosylase (XET; EC 2.4.1.207) activity towards xyloglucan and non-detectable endohydrolytic (XEH; EC 3.2.1.151) activity. Expressed throughout both the main and the lateral root, with intensive expression at the dividing and elongating regions. Is expressed in lateral root primordia but expression ceases after lateral root begins to grow. Involved in cell proliferation in incised inflorescence stems.
AT1G31050	-1,197		basic helix-loop-helix (bHLH) DNA-binding superfamily protein; FUNCTIONS IN: sequence-specific DNA binding transcription factor activity; INVOLVED IN: regulation of transcription; LOCATED IN: nucleus; EXPRESSED IN: sperm cell, hypocotyl, root; CONTAINS InterPro DOMAIN/s: Helix-loop-helix DNA-binding (InterPro:IPR011598); BEST Arabidopsis thaliana protein match is: basic helix-loop-helix (bHLH) DNA-binding superfamily protein (TAIR:AT1G61660.3)
AT1G49310	-1,197		unknown protein; FUNCTIONS IN: molecular_function unknown; INVOLVED IN: biological_process unknown; LOCATED IN: endomembrane system; EXPRESSED IN: 9 plant structures; EXPRESSED DURING: LP.04 four leaves visible, petal differentiation and expansion stage
AT3G10525	-1,197	LOSS OF GIANT CELLS FROM ORGANS (LGO)	Encodes LGO (loss of giant cells from organs) required for endoreduplication in sepal giant cell formation. Giant cells in both leaves and sepals are absent in lgo mutants. LGO is a member of a plant specific cell cycle inhibitor family SIAMESE and was originally named as SMR1(SIAMESE RELATED 1).
AT4G36740	-1,197	HOMEOBOX PROTEIN 40 (HB40)	Encodes a homeodomain leucine zipper class I (HD-Zip I) protein.
AT3G51920	-1,199	CALMODULIN 9 (CAM9)	encodes a divergent member of calmodulin, which is an EF-hand family of Ca2+-binding proteins. This gene is expressed in leaves, flowers and siliques. The gene functionally complements yeast calmodulin 1 (CAM1) but only when selected against the plasmid harboring wild-type yeast sequences. Also the protein does not form formed a complex with a basic amphiphilic helical peptide in the presence of Ca2+ in vitro. Authors suggest that this gene may represent a Ca2+-binding sensor protein that interacts with a more limited set of target proteins than do more conventional CaM isoforms. Mutations in this gene alter plant responses to abiotic stress and abscisic acid.
AT5G45670	-1,199		GDSL-motif esterase/acyltransferase/lipase. Enzyme group with broad substrate specificity that may catalyze acyltransfer or hydrolase reactions with lipid and non-lipid substrates.
AT3G47740	-1,2	ATP-BINDING CASSETTE A3 (ABCA3)	member of ATH subfamily

AT1G16400	*-1,202*	CYTOCHROME P450, FAMILY 79, SUBFAMILY F, POLYPEPTIDE 2 (CYP79F2)	Encodes cytochrome P450 CYP79F2.
AT3G30350	*-1,203*	ROOT MERISTEM GROWTH FACTOR 4 (RGF4)	Encodes a root meristem growth factor (RGF). Belongs to a family of functionally redundant homologous peptides that are secreted, tyrosine-sulfated, and expressed mainly in the stem cell area and the innermost layer of central columella cells. RGFs are required for maintenance of the root stem cell niche and transit amplifying cell proliferation. Members of this family include: At5g60810 (RGF1), At1g13620 (RGF2), At2g04025 (RGF3), At3g30350 (RGF4), At5g51451 (RGF5), At4g16515 (RGF6), At3g02240 (RGF7), At2g03830 (RGF8) and At5g64770 (RGF9).
AT4G00880	*-1,203*	SMALL AUXIN UPREGULATED RNA 31 (SAUR31)	SAUR-like auxin-responsive protein family ; CONTAINS InterPro DOMAIN/s: Auxin responsive SAUR protein (InterPro:IPR003676); BEST Arabidopsis thaliana protein match is: SAUR-like auxin-responsive protein family (TAIR:AT2G46690.1)
AT4G09770	*-1,204*		TRAF-like family protein; CONTAINS InterPro DOMAIN/s: TRAF-like (InterPro:IPR008974), MATH (InterPro:IPR002083); BEST Arabidopsis thaliana protein match is: TRAF-like family protein (TAIR:AT4G09780.1)
AT1G30900	*-1,205*	VACUOLAR SORTING RECEPTOR 6 (VSR6)	VACUOLAR SORTING RECEPTOR 6 (VSR6); FUNCTIONS IN: calcium ion binding; INVOLVED IN: protein targeting to vacuole; LOCATED IN: integral to plasma membrane, Golgi transport complex; EXPRESSED IN: 20 plant structures; EXPRESSED DURING: 10 growth stages; CONTAINS InterPro DOMAIN/s: Protease-associated PA (InterPro:IPR003137), EGF-like calcium-binding, conserved site (InterPro:IPR018097), EGF-like calcium-binding (InterPro:IPR001881), Growth factor, receptor (InterPro:IPR009030); BEST Arabidopsis thaliana protein match is: VACUOLAR SORTING RECEPTOR 5 (TAIR:AT2G34940.1)
AT5G57980	*-1,205*	RNA POLYMERASE II FIFTH LARGEST SUBUNIT, C (RPB5C)	NRPB5-like protein of unknown function; homologous to budding yeast RPB5
AT1G30370	*-1,207*	DAD1-LIKE ACYLHYDROLASE (DLAH)	Encodes a mitochondria-localized class III phospholipase A1 that plays a role in seed viability.
AT5G11570	*-1,208*		Major facilitator superfamily protein; FUNCTIONS IN: transporter activity; INVOLVED IN: oligopeptide transport; LOCATED IN: membrane; EXPRESSED IN: 9 plant structures; EXPRESSED DURING: 6 growth stages; CONTAINS InterPro DOMAIN/s: Oligopeptide transporter (InterPro:IPR000109), Major facilitator superfamily, general substrate transporter (InterPro:IPR016196); BEST Arabidopsis thaliana protein match is: Major facilitator superfamily protein (TAIR:AT1G52190.1)
AT2G03260	*-1,209*		EXS (ERD1/XPR1/SYG1) family protein; LOCATED IN: integral to membrane; EXPRESSED IN: shoot apex, root; CONTAINS InterPro DOMAIN/s: SPX, N-terminal (InterPro:IPR004331), EXS, C-terminal (InterPro:IPR004342); BEST Arabidopsis thaliana protein match is: EXS (ERD1/XPR1/SYG1) family protein (TAIR:AT1G14040.1)
AT3G02000	*-1,209*	(ROXY1)	Roxy1 encodes a glutaredoxin belonging to a subgroup specific to higher plants. It is required for proper petal initiation and organogenesis. It is likely to function in the temporal and spatial expression regulation of AGAMOUS in the first and second whorl. It's function is dependent on the Cysteine 49 residue and its nuclear localization. ROXY1 interacts in vitro and in vivo with members of the TGA family of transcription factors (e.g. TGA2, TGA3, TGA7 and PAN).
AT5G59510	*-1,209*	ROTUNDIFOLIA LIKE 5 (RTFL5)	ROTUNDIFOLIA like 5 (RTFL5); CONTAINS InterPro DOMAIN/s: DVL (InterPro:IPR012552); BEST Arabidopsis thaliana protein match is: ROTUNDIFOLIA like 4 (TAIR:AT3G46613.1)
AT4G25260	*-1,21*		Plant invertase/pectin methylesterase inhibitor superfamily protein; FUNCTIONS IN: enzyme inhibitor activity, pectinesterase inhibitor activity, pectinesterase activity; INVOLVED IN: shade avoidance; LOCATED IN: endomembrane system; EXPRESSED IN: 21 plant structures; EXPRESSED DURING: 13 growth stages; CONTAINS InterPro DOMAIN/s: Pectinesterase inhibitor (InterPro:IPR006501); BEST Arabidopsis thaliana protein match is: Plant invertase/pectin methylesterase inhibitor superfamily protein (TAIR:AT5G62350.1)

AT4G27290	*-1,21*		S-locus lectin protein kinase family protein; FUNCTIONS IN: sugar binding, protein serine/threonine kinase activity, protein kinase activity, ATP binding; INVOLVED IN: protein amino acid phosphorylation, recognition of pollen; LOCATED IN: endomembrane system; CONTAINS InterPro DOMAIN/s: Curculin-like (mannose-binding) lectin (InterPro:IPR001480), Apple-like (InterPro:IPR003609), PAN-2 domain (InterPro:IPR013227), Serine/threonine-protein kinase domain (InterPro:IPR002290), EGF-like, type 3 (InterPro:IPR000742), Serine-threonine/tyrosine-protein kinase (InterPro:IPR001245), Protein kinase-like domain (InterPro:IPR011009), Serine/threonine-protein kinase, active site (InterPro:IPR008271), Protein kinase, catalytic domain (InterPro:IPR000719), S-locus glycoprotein (InterPro:IPR000858), Tyrosine-protein kinase, catalytic domain (InterPro:IPR020635); BEST Arabidopsis thaliana protein match is: receptor kinase 3 (TAIR:AT4G21380.1)
AT2G02820	*-1,211*	MYB DOMAIN PROTEIN 88 (MYB88)	Encodes a putative transcription factor (MYB88), involved in stomata development, double loss of MYB88 and FLP (MYB124) activity results in a failure of guard mother cells (GMCs) to adopt the guard cell fate, thus they continue to divide resulting in abnormal stomata consisting of clusters of numerous guard cell-like cells. This phenotype is enhanced in double mutants over the single mutant flp phenotype. Also regulates female reproductive development.
AT1G14280	*-1,213*	PHYTOCHROME KINASE SUBSTRATE 2 (PKS2)	Encodes phytochrome kinase substrate 2. PKS proteins are critical for hypocotyl phototropism. Forms a complex with Phot1, Phot2 and NPH3.
AT4G13510	*-1,213*	AMMONIUM TRANSPORTER 1;1 (AMT1;1)	Encodes a plasma membrane localized ammonium transporter. Contains a cytosolic trans-activation domain essential for ammonium uptake. The mRNA is cell-to-cell mobile.
AT1G51890	*-1,214*		Leucine-rich repeat protein kinase family protein; FUNCTIONS IN: kinase activity; INVOLVED IN: protein amino acid phosphorylation; LOCATED IN: endomembrane system; EXPRESSED IN: 9 plant structures; EXPRESSED DURING: 8 growth stages; CONTAINS InterPro DOMAIN/s: Protein kinase, ATP binding site (InterPro:IPR017441), Serine/threonine-protein kinase domain (InterPro:IPR002290), Leucine-rich repeat (InterPro:IPR001611), Serine/threonine-protein kinase-like domain (InterPro:IPR017442), Serine/threonine-protein kinase, active site (InterPro:IPR008271), Protein kinase-like domain (InterPro:IPR011009), Protein kinase, catalytic domain (InterPro:IPR000719), Tyrosine-protein kinase, catalytic domain (InterPro:IPR020635); BEST Arabidopsis thaliana protein match is: Leucine-rich repeat protein kinase family protein (TAIR:AT1G51860.1)
AT4G15990	*-1,215*		unknown protein; BEST Arabidopsis thaliana protein match is: unknown protein (TAIR:AT4G16024.1)
AT5G19600	*-1,215*	SULFATE TRANSPORTER 3;5 (SULTR3;5)	Encodes sulfate transporter Sultr3;5.
AT1G23870	*-1,216*	TREHALOSE-PHOSPHATASE/SYNTHASE 9 (TPS9)	Encodes an enzyme putatively involved in trehalose biosynthesis. The protein has a trehalose synthase (TPS)-like domain that may or may not be active as well as a trehalose phosphatase (TPP)-like domain. The mRNA is cell-to-cell mobile.
AT1G25450	*-1,218*	3-KETOACYL-COA SYNTHASE 5 (KCS5)	Encodes KCS5, a member of the 3-ketoacyl-CoA synthase family involved in the biosynthesis of VLCFA (very long chain fatty acids).
AT2G30130	*-1,218*	(ASL5)	Overexpression/activation tagged allele has epinastic leaves, reduced apical dominance and is sterile. Gene is similar to asymmetric leaves (AS)/lateral organ boundary (LOB) genes which repress KNOX gene expression.
AT2G05180	*-1,219*	CYTOCHROME P450, FAMILY 705, SUBFAMILY A, POLYPEPTIDE 6 (CYP705A6)	member of CYP705A
AT4G21980	*-1,221*	AUTOPHAGY 8A (APG8A)	Encodes APG8, a component of autophagy conjugation pathway. Delivered to the lumens of vacuole under nitrogen-starvation condition. Highest expression in flowers. mRNA abundance increased during dark-induced carbon starvation. Predominantly cytoplasmic with or without N starvation. Upon concanamycin A the protein accumulates in the central vacuole as punctuate structures that resemble autophagic bodies. This localization is more abundant upon N starvation. The mRNA is cell-to-cell mobile.
AT5G22920	*-1,221*	RING ZINC-FINGER PROTEIN 34 (RZPF34)	Encodes a protein with sequence similarity to RING, zinc finger proteins. Loss of function mutations show reduced (15%) stomatal aperture under non stress conditions.

AT5G43150	*-1,221*		unknown protein; FUNCTIONS IN: molecular_function unknown; INVOLVED IN: biological_process unknown; LOCATED IN: mitochondrion; EXPRESSED IN: 22 plant structures; EXPRESSED DURING: 13 growth stages
AT1G35580	*-1,222*	CYTOSOLIC INVERTASE 1 (CINV1)	CINV1 / A/N-InvG is an alkaline/neutral invertase that breaks sucrose down into fructose and glucose (GH100). The exact localization of CINV1 remains under investigation but there is evidence that fluorescently-tagged CINV1 localizes to the cytoplasm. atinvg mutants have reduced root growth, reduced invertase activity, and increased expression of antioxidant genes under basal conditions. The levels of CINV1 / A/N-InvG transcripts rise in response to a hydrogen peroxide treatment. The protein has been shown to interact with PIP5K9.
AT1G72220	*-1,223*		RING/U-box superfamily protein; FUNCTIONS IN: zinc ion binding; EXPRESSED IN: inflorescence meristem, root, flower; EXPRESSED DURING: 4 anthesis, petal differentiation and expansion stage; CONTAINS InterPro DOMAIN/s: Zinc finger, RING-type (InterPro:IPR001841), Zinc finger, C3HC4 RING-type (InterPro:IPR018957); BEST Arabidopsis thaliana protein match is: RING/U-box superfamily protein (TAIR:AT4G33565.1)
AT1G10380	*-1,225*		Putative membrane lipoprotein; FUNCTIONS IN: molecular_function unknown; INVOLVED IN: biological_process unknown; LOCATED IN: endomembrane system; EXPRESSED IN: 19 plant structures; EXPRESSED DURING: 10 growth stages; BEST Arabidopsis thaliana protein match is: unknown protein (TAIR:AT3G17350.1)
AT2G21400	*-1,226*	SHI-RELATED SEQUENCE3 (SRS3)	A member of SHI gene family. Arabidopsis thaliana has ten members that encode proteins with a RING finger-like zinc finger motif. Despite being highly divergent in sequence, many of the SHI-related genes are partially redundant in function and synergistically promote gynoecium, stamen and leaf development in Arabidopsis.
AT2G47130	*-1,226*	SHORT-CHAIN DEHYDROGENASE/REDUCTASE 2 (SDR3)	Encodes a short-chain dehydrogenase/reductase that is not involved in ABA biosynthesis but plays an important role in plant defense response to bacteria.
AT5G18840	*-1,226*		Major facilitator superfamily protein; FUNCTIONS IN: carbohydrate transmembrane transporter activity, sugar:hydrogen symporter activity; INVOLVED IN: transport, transmembrane transport; LOCATED IN: integral to membrane, membrane; EXPRESSED IN: 17 plant structures; EXPRESSED DURING: 7 growth stages; CONTAINS InterPro DOMAIN/s: Major facilitator superfamily (InterPro:IPR020846), General substrate transporter (InterPro:IPR005828), Sugar/inositol transporter (InterPro:IPR003663), Major facilitator superfamily, general substrate transporter (InterPro:IPR016196); BEST Arabidopsis thaliana protein match is: Major facilitator superfamily protein (TAIR:AT2G48020.1)
AT3G50660	*-1,227*	DWARF 4 (DWF4)	Encodes a 22α hydroxylase whose reaction is a rate-limiting step in brassinosteroid biosynthetic pathway. The protein is a member of CYP90B gene family. CLM is an epi-allele with small, compressed rosette, reduced internode length, and reduced fertility, appears in selfed ddm mutant plants possibly due to loss of cytosine methylation. Transcripts accumulate in actively growing tissues, and GUS expression is negatively regulated by brassinosteroids. Localized in the endoplasmic reticulum. The in vitro expressed protein can perform the C-22 hydroxylation of a variety of C27-, C28- and C29-sterols. Cholesterol was the best substrate, followed by campesterol. Sitosterol was a poor substrate.
AT5G07650	*-1,228*		Actin-binding FH2 protein; FUNCTIONS IN: actin binding; INVOLVED IN: cellular component organization, actin cytoskeleton organization; LOCATED IN: chloroplast; CONTAINS InterPro DOMAIN/s: Actin-binding FH2/DRF autoregulatory (InterPro:IPR003104), Actin-binding FH2 (InterPro:IPR015425); BEST Arabidopsis thaliana protein match is: formin homology 2 domain-containing protein / FH2 domain-containing protein (TAIR:AT5G07760.1)
AT5G27920	*-1,228*		F-box family protein; CONTAINS InterPro DOMAIN/s: Leucine-rich repeat, cysteine-containing subtype (InterPro:IPR006553); BEST Arabidopsis thaliana protein match is: RNI-like superfamily protein (TAIR:AT5G01720.1)
AT5G54030	*-1,228*		Cysteine/Histidine-rich C1 domain family protein; FUNCTIONS IN: molecular_function unknown; INVOLVED IN: intracellular signaling pathway; LOCATED IN: cellular_component unknown; EXPRESSED IN: root; CONTAINS InterPro DOMAIN/s: Protein kinase C-like, phorbol ester/diacylglycerol binding (InterPro:IPR002219), DC1 (InterPro:IPR004146), C1-like (InterPro:IPR011424); BEST Arabidopsis thaliana protein match is: Cysteine/Histidine-rich C1 domain family protein (TAIR:AT5G29624.1)

AT1G20380	-1,229		Prolyl oligopeptidase family protein; FUNCTIONS IN: serine-type peptidase activity, serine-type endopeptidase activity; INVOLVED IN: proteolysis; LOCATED IN: cellular_component unknown; EXPRESSED IN: 19 plant structures; EXPRESSED DURING: 9 growth stages; CONTAINS InterPro DOMAIN/s: Peptidase S9, prolyl oligopeptidase, catalytic domain (InterPro:IPR001375), Peptidase S9A, oligopeptidase, N-terminal beta-propeller (InterPro:IPR004106), Peptidase S9A, prolyl oligopeptidase (InterPro:IPR002470); BEST Arabidopsis thaliana protein match is: Prolyl oligopeptidase family protein (TAIR:AT1G76140.1)
AT4G14630	-1,229	GERMIN-LIKE PROTEIN 9 (GLP9)	germin-like protein with N-terminal signal sequence that may target it to the vacuole, plasma membrane and/or outside the cell. The mRNA is cell-to-cell mobile.
AT5G18860	-1,23	NUCLEOSIDE HYDROLASE 3 (NSH3)	Encodes a purine nucleoside hydrolase active in the apoplast. It might play a role in salvaging extracellular ATP. NSH3 transcript levels rise in response to jasmonic acid and wounding.
AT1G60110	-1,232		Mannose-binding lectin superfamily protein; EXPRESSED IN: cultured cell; CONTAINS InterPro DOMAIN/s: Mannose-binding lectin (InterPro:IPR001229); BEST Arabidopsis thaliana protein match is: Mannose-binding lectin superfamily protein (TAIR:AT1G60130.1)
AT3G13790	-1,233	(ATBFRUCT1)	Encodes a protein with invertase activity.
AT4G09130	-1,233		RING/U-box superfamily protein; FUNCTIONS IN: zinc ion binding; LOCATED IN: endomembrane system; CONTAINS InterPro DOMAIN/s: Zinc finger, RING-type (InterPro:IPR001841), Zinc finger, C3HC4 RING-type (InterPro:IPR018957); BEST Arabidopsis thaliana protein match is: RING/U-box superfamily protein (TAIR:AT4G09120.1)
AT3G26300	-1,234	CYTOCHROME P450, FAMILY 71, SUBFAMILY B, POLYPEPTIDE 34 (CYP71B34)	putative cytochrome P450
AT2G03830	-1,235	ROOT MERISTEM GROWTH FACTOR 8 (RGF8)	Encodes a root meristem growth factor (RGF). Belongs to a family of functionally redundant homologous peptides that are secreted, tyrosine-sulfated, and expressed mainly in the stem cell area and the innermost layer of central columella cells. RGFs are required for maintenance of the root stem cell niche and transit amplifying cell proliferation. Members of this family include: At5g60810 (RGF1), At1g13620 (RGF2), At2g04025 (RGF3), At3g30350 (RGF4), At5g51451 (RGF5), At4g16515 (RGF6), At3g02240 (RGF7), At2g03830 (RGF8) and At5g64770 (RGF9).
AT2G25240	-1,235	CONSERVED IN CILIATED SPECIES AND IN THE LAND PLANTS 3 (CCP3)	Serine protease inhibitor (SERPIN) family protein; FUNCTIONS IN: serine-type endopeptidase inhibitor activity; INVOLVED IN: biological_process unknown; LOCATED IN: cellular_component unknown; CONTAINS InterPro DOMAIN/s: Protease inhibitor I4, serpin, plant (InterPro:IPR015554), Protease inhibitor I4, serpin (InterPro:IPR000215); BEST Arabidopsis thaliana protein match is: Serine protease inhibitor (SERPIN) family protein (TAIR:AT2G26390.1)
AT4G34600	-1,235		unknown protein; FUNCTIONS IN: molecular_function unknown; INVOLVED IN: pollen tube growth; LOCATED IN: endomembrane system; EXPRESSED IN: 11 plant structures; EXPRESSED DURING: M germinated pollen stage, LP.04 four leaves visible, 4 anthesis, petal differentiation and expansion stage; BEST Arabidopsis thaliana protein match is: unknown protein (TAIR:AT2G16385.1)
AT3G04030	-1,236	(MYR2)	Homeodomain-like superfamily protein; FUNCTIONS IN: sequence-specific DNA binding transcription factor activity; INVOLVED IN: regulation of transcription; EXPRESSED IN: 9 plant structures; EXPRESSED DURING: 4 anthesis, 4 leaf senescence stage, petal differentiation and expansion stage; CONTAINS InterPro DOMAIN/s: Homeodomain-like (InterPro:IPR009057), Myb, DNA-binding (InterPro:IPR014778), Myb-like DNA-binding domain, SHAQKYF class (InterPro:IPR006447), HTH transcriptional regulator, Myb-type, DNA-binding (InterPro:IPR017930), Homeodomain-related (InterPro:IPR012287); BEST Arabidopsis thaliana protein match is: myb-related protein 1 (TAIR:AT5G18240.4)
AT2G47730	-1,238	GLUTATHIONE S-TRANSFERASE PHI 8 (GSTF8)	Encodes glutathione transferase belonging to the phi class of GSTs. Naming convention according to Wagner et al. (2002).
AT3G03870	-1,238		unknown protein; BEST Arabidopsis thaliana protein match is: unknown protein (TAIR:AT5G18130.1)
AT1G58070	-1,239		unknown protein

AT3G12040	*-1,239*		Encodes a 3-methyladenine-DNA glycosylase. Arabdiopsis cDNA complements the methyl methanesulfonate-sensitive phenotype of an Escherichia coli double mutant deficient in 3-methyladenine glycosylases (DNA-3-methyladenine glycosidases I and II, EC 3.2.2.20 and 3.2.2.21, respectively, encoded by tag and alkA).
AT3G26610	*-1,239*	POLYGALACTURONASE INVOLVED IN EXPANSION 1 (PGX1)	Encodes an apoplast-localized polygalacturonase involved in cell elongation and flower development.
AT4G12270	*-1,239*		Copper amine oxidase family protein; FUNCTIONS IN: quinone binding, primary amine oxidase activity, copper ion binding; INVOLVED IN: oxidation reduction, amine metabolic process; LOCATED IN: endomembrane system; CONTAINS InterPro DOMAIN/s: Copper amine oxidase, N-terminal (InterPro:IPR016182), Copper amine oxidase, N2-terminal (InterPro:IPR015800), Copper amine oxidase, N2/N3-terminal (InterPro:IPR015801), Copper amine oxidase, N3-terminal (InterPro:IPR015802), Copper amine oxidase (InterPro:IPR000269), Copper amine oxidase, C-terminal (InterPro:IPR015798); BEST Arabidopsis thaliana protein match is: Copper amine oxidase family protein (TAIR:AT4G12290.1)
AT4G09420	*-1,241*		Disease resistance protein (TIR-NBS class); FUNCTIONS IN: transmembrane receptor activity, ATP binding; INVOLVED IN: signal transduction, apoptosis, defense response, innate immune response; LOCATED IN: intrinsic to membrane; EXPRESSED IN: shoot apex, cotyledon, leaf whorl, leaf; EXPRESSED DURING: LP.06 six leaves visible, LP.04 four leaves visible, LP.10 ten leaves visible, 4 leaf senescence stage, LP.08 eight leaves visible; CONTAINS InterPro DOMAIN/s: NB-ARC (InterPro:IPR002182), Disease resistance protein (InterPro:IPR000767), Toll-Interleukin receptor (InterPro:IPR000157); BEST Arabidopsis thaliana protein match is: Disease resistance protein (TIR-NBS class) (TAIR:AT1G72850.1)
AT5G45210	*-1,241*		Disease resistance protein (TIR-NBS-LRR class) family; FUNCTIONS IN: transmembrane receptor activity, ATP binding; INVOLVED IN: signal transduction, apoptosis, defense response, innate immune response; LOCATED IN: intrinsic to membrane; EXPRESSED IN: root; CONTAINS InterPro DOMAIN/s: NB-ARC (InterPro:IPR002182), Leucine-rich repeat (InterPro:IPR001611), Toll-Interleukin receptor (InterPro:IPR000157), Disease resistance protein (InterPro:IPR000767); BEST Arabidopsis thaliana protein match is: Disease resistance protein (TIR-NBS-LRR class) family (TAIR:AT3G51560.1)
AT3G49380	*-1,242*	IQ-DOMAIN 15 (iqd15)	IQ-domain 15 (iqd15); CONTAINS InterPro DOMAIN/s: IQ calmodulin-binding region (InterPro:IPR000048); BEST Arabidopsis thaliana protein match is: IQ-domain 16 (TAIR:AT4G10640.1)
AT1G55550	*-1,244*		P-loop containing nucleoside triphosphate hydrolases superfamily protein; FUNCTIONS IN: microtubule motor activity, ATP binding; INVOLVED IN: microtubule-based movement; LOCATED IN: mitochondrion; EXPRESSED IN: seed; EXPRESSED DURING: E expanded cotyledon stage; CONTAINS InterPro DOMAIN/s: Kinesin, motor domain (InterPro:IPR001752); BEST Arabidopsis thaliana protein match is: P-loop containing nucleoside triphosphate hydrolases superfamily protein (TAIR:AT5G27950.1)
AT4G05070	*-1,244*		Wound-responsive family protein; FUNCTIONS IN: molecular_function unknown; INVOLVED IN: biological_process unknown; LOCATED IN: chloroplast; EXPRESSED IN: 22 plant structures; EXPRESSED DURING: 13 growth stages; CONTAINS InterPro DOMAIN/s: Protein of unknown function wound-induced (InterPro:IPR022251)
AT1G49390	*-1,245*		2-oxoglutarate (2OG) and Fe(II)-dependent oxygenase superfamily protein; FUNCTIONS IN: oxidoreductase activity, acting on paired donors, with incorporation or reduction of molecular oxygen, 2-oxoglutarate as one donor, and incorporation of one atom each of oxygen into both donors, oxidoreductase activity; INVOLVED IN: flavonoid biosynthetic process; LOCATED IN: cellular_component unknown; EXPRESSED IN: hypocotyl, root, flower; EXPRESSED DURING: petal differentiation and expansion stage; CONTAINS InterPro DOMAIN/s: Oxoglutarate/iron-dependent oxygenase (InterPro:IPR005123); BEST Arabidopsis thaliana protein match is: 2-oxoglutarate (2OG) and Fe(II)-dependent oxygenase superfamily protein (TAIR:AT5G20400.1)
AT1G50560	*-1,245*	CYTOCHROME P450, FAMILY 705, SUBFAMILY A, POLYPEPTIDE 25 (CYP705A25)	member of CYP705A

AT5G19240	-1,245		Glycoprotein membrane precursor GPI-anchored; FUNCTIONS IN: molecular_function unknown; INVOLVED IN: biological_process unknown; LOCATED IN: endomembrane system; EXPRESSED IN: 24 plant structures; EXPRESSED DURING: 14 growth stages; BEST Arabidopsis thaliana protein match is: Glycoprotein membrane precursor GPI-anchored (TAIR:AT5G19230.1)
AT2G02310	-1,246	PHLOEM PROTEIN 2-B6 (PP2-B6)	phloem protein 2-B6 (PP2-B6); FUNCTIONS IN: carbohydrate binding; INVOLVED IN: N-terminal protein myristoylation; LOCATED IN: cellular_component unknown; EXPRESSED IN: leaf whorl, sperm cell, root, leaf; EXPRESSED DURING: LP.04 four leaves visible; CONTAINS InterPro DOMAIN/s: F-box domain, cyclin-like (InterPro:IPR001810), F-box domain, Skp2-like (InterPro:IPR022364); BEST Arabidopsis thaliana protein match is: phloem protein 2-B2 (TAIR:AT2G02250.1)
AT2G47160	-1,246	REQUIRES HIGH BORON 1 (BOR1)	Boron transporter. Protein accumulates in shoots and roots under conditions of boron deficiency and is degraded within several hours of restoring boron supply. Localized to the plasma membrane under B limitation, and to the cytoplasm after B application before degradation. Protein is transferred via the endosomes to the vacuole for degradation. Localized to the inner plasma membrane domain in the columella, lateral root cap, epidermis, and endodermis in the root tip region, and in the epidermis and endodermis in the elongation zone. Under high-boron is transported to the vacuole for degradation.
AT4G21230	-1,246	CYSTEINE-RICH RLK (RECEPTOR-LIKE PROTEIN KINASE) 27 (CRK27)	Encodes a cysteine-rich receptor-like protein kinase.
AT5G16900	-1,246		Leucine-rich repeat protein kinase family protein; FUNCTIONS IN: kinase activity; INVOLVED IN: protein amino acid phosphorylation; LOCATED IN: endomembrane system; EXPRESSED IN: embryo, leaf whorl, sperm cell, sepal, root; EXPRESSED DURING: petal differentiation and expansion stage, D bilateral stage; CONTAINS InterPro DOMAIN/s: Protein kinase, ATP binding site (InterPro:IPR017441), Protein kinase, catalytic domain (InterPro:IPR000719), Leucine-rich repeat (InterPro:IPR001611), Serine/threonine-protein kinase-like domain (InterPro:IPR017442), Protein kinase-like domain (InterPro:IPR011009), Serine/threonine-protein kinase, active site (InterPro:IPR008271); BEST Arabidopsis thaliana protein match is: Leucine-rich repeat protein kinase family protein (TAIR:AT1G07560.1)
AT2G23770	-1,247	LYSM-CONTAINING RECEPTOR-LIKE KINASE 4 (LYK4)	Encodes a putative LysM-containing receptor-like kinase LYK4. Shares overlapping function with LYK5 in mediating chitin-triggered immune responses. Based on protein sequence alignment analysis, it was determined as a pseudo kinase due to a lack of the ATP-binding P-loop in the kinase domain.
AT2G32150	-1,247		Haloacid dehalogenase-like hydrolase (HAD) superfamily protein; FUNCTIONS IN: hydrolase activity, catalytic activity; INVOLVED IN: metabolic process; LOCATED IN: cellular_component unknown; EXPRESSED IN: 24 plant structures; EXPRESSED DURING: 13 growth stages; CONTAINS InterPro DOMAIN/s: Haloacid dehalogenase-like hydrolase (InterPro:IPR005834), Pyrimidine 5-nucleotidase (InterPro:IPR010237), HAD-superfamily hydrolase, subfamily IA, variant 3 (InterPro:IPR006402); BEST Arabidopsis thaliana protein match is: Haloacid dehalogenase-like hydrolase (HAD) superfamily protein (TAIR:AT5G02230.2)
AT5G55170	-1,247	SMALL UBIQUITIN-LIKE MODIFIER 3 (SUMO3)	Encodes a small ubiquitin-like modifier (SUMO) polypeptide that becomes covalently attached to various intracellular protein targets, much like ubiquitination, leading to post-translational modification of those targets.
AT1G10340	-1,248		Ankyrin repeat family protein; CONTAINS InterPro DOMAIN/s: Ankyrin repeat-containing domain (InterPro:IPR020683), Ankyrin repeat (InterPro:IPR002110); BEST Arabidopsis thaliana protein match is: Ankyrin repeat family protein (TAIR:AT2G24600.4)
AT1G26800	-1,25		RING/U-box superfamily protein; FUNCTIONS IN: zinc ion binding; EXPRESSED IN: 20 plant structures; EXPRESSED DURING: 11 growth stages; CONTAINS InterPro DOMAIN/s: Zinc finger, RING-type (InterPro:IPR001841), Zinc finger, C3HC4 RING-type (InterPro:IPR018957); BEST Arabidopsis thaliana protein match is: RING/U-box superfamily protein (TAIR:AT1G14200.1)
AT1G30650	-1,25	WRKY DNA-BINDING PROTEIN 14 (WRKY14)	member of WRKY Transcription Factor; Group II-e
AT1G15670	-1,252	KISS ME DEADLY 2 (KMD2)	Encodes a member of a family of F-box proteins, called the KISS ME DEADLY (KMD) family, that targets type-B ARR proteins for degradation and is involved in the negative regulation of the cytokinin response. Also named as KFB1, a member of a group of Kelch repeat F-box proteins that negatively regulate phenylpropanoid biosynthesis by targeting the phenypropanoid biosynthesis enzyme phenylalanine ammonia-lyase.

AT1G46768	*-1,252*	RELATED TO AP2 1 (RAP2.1)	encodes a member of the DREB subfamily A-5 of ERF/AP2 transcription factor family (RAP2.1). The protein contains one AP2 domain. There are 16 members in this subfamily including RAP2.9 and RAP2.10.
AT5G01610	*-1,252*		Protein of unknown function, DUF538; CONTAINS InterPro DOMAIN/s: Protein of unknown function DUF538 (InterPro:IPR007493); BEST Arabidopsis thaliana protein match is: Protein of unknown function, DUF538 (TAIR:AT3G08890.2)
AT5G14340	*-1,252*	MYB DOMAIN PROTEIN 40 (MYB40)	Member of the R2R3 factor gene family.
AT1G08990	*-1,254*	PLANT GLYCOGENIN-LIKE STARCH INITIATION PROTEIN 5 (PGSIP5)	plant glycogenin-like starch initiation protein 5 (PGSIP5); FUNCTIONS IN: transferase activity, transferring glycosyl groups; INVOLVED IN: biosynthetic process; LOCATED IN: endomembrane system; EXPRESSED IN: root; CONTAINS InterPro DOMAIN/s: Glycosyl transferase, family 8 (InterPro:IPR002495); BEST Arabidopsis thaliana protein match is: plant glycogenin-like starch initiation protein 4 (TAIR:AT1G54940.1)
AT1G21360	*-1,254*	GLYCOLIPID TRANSFER PROTEIN 2 (GLTP2)	glycolipid transfer protein 2 (GLTP2); FUNCTIONS IN: glycolipid transporter activity, glycolipid binding; INVOLVED IN: glycolipid transport; LOCATED IN: cytoplasm; CONTAINS InterPro DOMAIN/s: Glycolipid transfer protein, GLTP (InterPro:IPR014830); BEST Arabidopsis thaliana protein match is: Glycolipid transfer protein (GLTP) family protein (TAIR:AT3G21260.3)
AT1G68240	*-1,255*		basic helix-loop-helix (bHLH) DNA-binding superfamily protein; FUNCTIONS IN: sequence-specific DNA binding transcription factor activity; INVOLVED IN: regulation of transcription; LOCATED IN: nucleus; CONTAINS InterPro DOMAIN/s: Helix-loop-helix DNA-binding domain (InterPro:IPR001092), Helix-loop-helix DNA-binding (InterPro:IPR011598); BEST Arabidopsis thaliana protein match is: phytochrome interacting factor 3-like 1 (TAIR:AT2G46970.1)
AT1G02260	*-1,256*		Divalent ion symporter; FUNCTIONS IN: citrate transmembrane transporter activity, transporter activity; INVOLVED IN: citrate transport, transmembrane transport; LOCATED IN: endomembrane system, integral to membrane; EXPRESSED IN: 24 plant structures; EXPRESSED DURING: 13 growth stages; CONTAINS InterPro DOMAIN/s: Divalent ion symporter (InterPro:IPR004680)
AT1G08590	*-1,256*	PXY-LIKE1 (PXL1)	Encodes one of the two putative eLRR kinase closely related to PXY (At1g08590/PXL1 and At4g28650/PXL2). Insertion mutants in either pxl1 or pxl2 do not exhibit an obvious phenotype in the stem; double-mutant combinations of a Col allele, of pxy (pxy-3) with pxl1 and pxl2, generate a more severe vascular phenotype than pxy-3 alone, suggesting that these genes act synergistically with PXY in regulating vascular-tissue development in the stem.
AT3G63210	*-1,256*	(MARD1)	encodes a novel zinc-finger protein with a proline-rich N-terminus, identical to senescence-associated protein SAG102
AT4G13770	*-1,257*	CYTOCHROME P450, FAMILY 83, SUBFAMILY A, POLYPEPTIDE 1 (CYP83A1)	Encodes a cytochrome p450 enzyme that catalyzes the initial conversion of aldoximes to thiohydroximates in the synthesis of glucosinolates not derived from tryptophan. Also has a role in auxin homeostasis.
AT4G00480	*-1,259*	(ATMYC1)	MYC-related protein with a basic helix-loop-helix motif at the C-terminus and a region similar to the maize B/R family at the N-terminus
AT4G33770	*-1,259*	INOSITOL 1,3,4-TRISPHOSPHATE 5/6 KINASE 2 (ITPK2)	Inositol 1,3,4-trisphosphate 5/6-kinase family protein; FUNCTIONS IN: inositol or phosphatidylinositol kinase activity, magnesium ion binding, inositol-1,3,4-trisphosphate 5/6-kinase activity, inositol tetrakisphosphate 1-kinase activity, ATP binding; INVOLVED IN: inositol trisphosphate metabolic process; LOCATED IN: intracellular; EXPRESSED IN: 21 plant structures; EXPRESSED DURING: 9 growth stages; CONTAINS InterPro DOMAIN/s: Inositol-tetrakisphosphate 1-kinase (InterPro:IPR017427), Inositol 1, 3, 4-trisphosphate 56-kinase (InterPro:IPR008656); BEST Arabidopsis thaliana protein match is: Inositol 1,3,4-trisphosphate 5/6-kinase family protein (TAIR:AT4G08170.2)
AT5G27000	*-1,259*	KINESIN 4 (ATK4)	Encodes a kinesin-like protein that binds microtubules in an ATP-dependent manner.
AT4G11320	*-1,26*	CYSTEINE PROTEASE 2 (CP2)	Papain family cysteine protease; FUNCTIONS IN: cysteine-type endopeptidase activity, cysteine-type peptidase activity; INVOLVED IN: proteolysis; LOCATED IN: endomembrane system; CONTAINS InterPro DOMAIN/s: Proteinase inhibitor I29, cathepsin propeptide (InterPro:IPR013201), Peptidase C1A, papain (InterPro:IPR013128), Peptidase C1A, papain C-terminal (InterPro:IPR000668), Peptidase, cysteine peptidase active site (InterPro:IPR000169); BEST Arabidopsis thaliana protein match is: Papain family cysteine protease (TAIR:AT4G11310.1)

AT1G16150	-1,262	WALL ASSOCIATED KINASE-LIKE 4 (WAKL4)	Encodes a WAK-like receptor-like kinase with a cytoplasmic Ser/Thr protein kinase domain and an extracellular domain with EGF-like repeats. Likely involved in Arabidopsis root mineral responses to Zn2+, Cu2+, K+, Na+ and Ni+. The mRNA is cell-to-cell mobile.
AT1G18330	-1,262	EARLY-PHYTOCHROME-RESPONSIVE1 (EPR1)	EARLY-PHYTOCHROME-RESPONSIVE1
AT1G79060	-1,262		unknown protein; BEST Arabidopsis thaliana protein match is: unknown protein (TAIR:AT1G56020.1)
AT4G00700	-1,262		C2 calcium/lipid-binding plant phosphoribosyltransferase family protein; CONTAINS InterPro DOMAIN/s: C2 membrane targeting protein (InterPro:IPR018029), C2 calcium/lipid-binding domain, CaLB (InterPro:IPR008973), Phosphoribosyltransferase C-terminal (InterPro:IPR013583), C2 calcium-dependent membrane targeting (InterPro:IPR000008); BEST Arabidopsis thaliana protein match is: C2 calcium/lipid-binding plant phosphoribosyltransferase family protein (TAIR:AT4G11610.1)
AT2G24710	-1,263	GLUTAMATE RECEPTOR 2.3 (GLR2.3)	member of Putative ligand-gated ion channel subunit family
AT5G64120	-1,263	PEROXIDASE 71 (PRX71)	Encodes a cell wall bound peroxidase that is induced by hypo-osmolarity and is involved in the lignification of cell walls.
AT1G55430	-1,264		Cysteine/Histidine-rich C1 domain family protein; FUNCTIONS IN: zinc ion binding; LOCATED IN: cellular_component unknown; EXPRESSED IN: root; CONTAINS InterPro DOMAIN/s: DC1 (InterPro:IPR004146), Zinc finger, PHD-type (InterPro:IPR001965), C1-like (InterPro:IPR011424); BEST Arabidopsis thaliana protein match is: Cysteine/Histidine-rich C1 domain family protein (TAIR:AT1G55420.1)
AT1G69900	-1,265		Actin cross-linking protein; CONTAINS InterPro DOMAIN/s: Protein of unknown function DUF569 (InterPro:IPR007679), Actin cross-linking (InterPro:IPR008999); BEST Arabidopsis thaliana protein match is: Actin cross-linking protein (TAIR:AT1G27100.1)
AT3G27025	-1,266		unknown protein
AT1G33800	-1,267	GLUCURONOXYLAN METHYLTRANSFERASE 1 (GXMT1)	Encodes a glucuronoxylan(GX)-speciﬁc 4-O-methyltransferase responsible for methylating GlcA residues in GX. Reduced methylation of GX ingxmt1-1 plants is correlated with altered lignin composition. The mRNA is cell-to-cell mobile.
AT5G61340	-1,268		unknown protein; BEST Arabidopsis thaliana protein match is: unknown protein (TAIR:AT1G26650.1)
AT1G03700	-1,269	CASP-LIKE PROTEIN 1C2 (CASPL1C2)	Uncharacterised protein family (UPF0497); FUNCTIONS IN: molecular_function unknown; INVOLVED IN: biological_process unknown; LOCATED IN: endomembrane system; EXPRESSED IN: hypocotyl, flower; EXPRESSED DURING: petal differentiation and expansion stage; CONTAINS InterPro DOMAIN/s: Uncharacterised protein family UPF0497, trans-membrane plant (InterPro:IPR006702), Uncharacterised protein family UPF0497, trans-membrane plant subgroup (InterPro:IPR006459); BEST Arabidopsis thaliana protein match is: Uncharacterised protein family (UPF0497) (TAIR:AT4G03540.1)
AT5G22660	-1,269		FBD, F-box, Skp2-like and Leucine Rich Repeat domains containing protein; FUNCTIONS IN: molecular_function unknown; INVOLVED IN: biological_process unknown; LOCATED IN: cellular_component unknown; CONTAINS InterPro DOMAIN/s: FBD (InterPro:IPR013596), F-box domain, cyclin-like (InterPro:IPR001810), F-box domain, Skp2-like (InterPro:IPR022364), FBD-like (InterPro:IPR006566), Leucine-rich repeat 2 (InterPro:IPR013101); BEST Arabidopsis thaliana protein match is: F-box/RNI-like/FBD-like domains-containing protein (TAIR:AT5G22700.1)
AT5G57770	-1,269		CONTAINS InterPro DOMAIN/s: Pleckstrin-like, plant (InterPro:IPR013666), Protein of unknown function DUF828 (InterPro:IPR008546); BEST Arabidopsis thaliana protein match is: Plant protein of unknown function (DUF828) with plant pleckstrin homology-like region (TAIR:AT3G22810.1)
AT2G40110	-1,27		Yippee family putative zinc-binding protein; CONTAINS InterPro DOMAIN/s: Yippee-like protein (InterPro:IPR004910); BEST Arabidopsis thaliana protein match is: Yippee family putative zinc-binding protein (TAIR:AT3G11230.2)
AT4G26470	-1,27		Calcium-binding EF-hand family protein; FUNCTIONS IN: calcium ion binding; INVOLVED IN: N-terminal protein myristoylation; LOCATED IN: cytoplasm; EXPRESSED IN: 8 plant structures; EXPRESSED DURING: L mature pollen stage, M germinated pollen stage, 4 anthesis, petal differentiation and expansion stage; CONTAINS InterPro DOMAIN/s: EF-Hand 1, calcium-binding site (InterPro:IPR018247), EF-HAND 2 (InterPro:IPR018249), Calcium-binding EF-hand (InterPro:IPR002048), EF-hand-like domain (InterPro:IPR011992); BEST Arabidopsis thaliana protein match is: Calcium-binding EF-hand family protein (TAIR:AT3G24110.1)

AT1G50930	-1,272	VASCULAR-RELATED UNKNOWN PROTEIN 2 (VUP2)	unknown protein; BEST Arabidopsis thaliana protein match is: unknown protein (TAIR:AT3G20557.1)
AT3G10520	-1,274	HAEMOGLOBIN 2 (HB2)	Encodes a class 2 non-symbiotic hemoglobin. Over-expression of AHb2 in seeds led to a 40% increase in the total fatty acid content of developing and mature seeds in three subsequent generations. This was mainly due to an increase in the poly-unsaturated C18:2 (omega-6) linoleic and C18:3 (omega-3) alpha-linolenic acids.
AT3G14680	-1,275	CYTOCHROME P450, FAMILY 72, SUBFAMILY A, POLYPEPTIDE 14 (CYP72A14)	putative cytochrome P450
AT5G27450	-1,277	MEVALONATE KINASE (MK)	Encodes a protein with mevalonate kinase activity involved in the mevalonate pathway.
AT1G13970	-1,278		Protein of unknown function (DUF1336); FUNCTIONS IN: molecular_function unknown; INVOLVED IN: N-terminal protein myristoylation; EXPRESSED IN: 20 plant structures; EXPRESSED DURING: 10 growth stages; CONTAINS InterPro DOMAIN/s: Protein of unknown function DUF1336 (InterPro:IPR009769); BEST Arabidopsis thaliana protein match is: Protein of unknown function (DUF1336) (TAIR:AT3G29180.2)
AT1G62660	-1,278		Glycosyl hydrolases family 32 protein; FUNCTIONS IN: hydrolase activity, hydrolyzing O-glycosyl compounds; INVOLVED IN: sucrose catabolic process, using beta-fructofuranosidase, carbohydrate metabolic process; LOCATED IN: vacuole; EXPRESSED IN: 23 plant structures; EXPRESSED DURING: 12 growth stages; CONTAINS InterPro DOMAIN/s: Protein of unknown function DUF3357 (InterPro:IPR021792), Glycoside hydrolase, family 32 (InterPro:IPR001362), Glycosyl hydrolases family 32, N-terminal (InterPro:IPR013148), Glycoside hydrolase, family 32, active site (InterPro:IPR018053), Glycosyl hydrolase family 32, C-terminal (InterPro:IPR013189), Concanavalin A-like lectin/glucanase (InterPro:IPR008985); BEST Arabidopsis thaliana protein match is: Glycosyl hydrolases family 32 protein (TAIR:AT1G12240.1)
AT2G04240	-1,278	(XERICO)	Encodes a small protein with an N-terminal trans-membrane domain and a RING-H2 zinc finger motif located at the C-terminus. Gene expression is induced by salt and osmotic stress. Transcrips levels are induced by DELLA proteins and repressed by gibberellic acid. Involved in ABA metabolism.
AT2G37820	-1,278		Cysteine/Histidine-rich C1 domain family protein; FUNCTIONS IN: zinc ion binding; INVOLVED IN: biological_process unknown; LOCATED IN: cellular_component unknown; EXPRESSED IN: root; CONTAINS InterPro DOMAIN/s: Zinc finger, RING-type (InterPro:IPR001841), C1-like (InterPro:IPR011424); BEST Arabidopsis thaliana protein match is: Cysteine/Histidine-rich C1 domain family protein (TAIR:AT2G37800.1)
AT4G01630	-1,278	EXPANSIN A17 (EXPA17)	member of Alpha-Expansin Gene Family. Naming convention from the Expansin Working Group (Kende et al, 2004. Plant Mol Bio)
AT5G11290	-1,279		Plant protein of unknown function (DUF247); CONTAINS InterPro DOMAIN/s: Protein of unknown function DUF247, plant (InterPro:IPR004158); BEST Arabidopsis thaliana protein match is: unknown protein (TAIR:AT4G31980.1)
AT3G50470	-1,28	HOMOLOG OF RPW8 3 (HR3)	Homolog of RPW8
AT5G45840	-1,28	MALE DISCOVERER1 (MDIS1)	Encodes a leucine-rich-repeat RLK that is localized to the plasma membrane of pollen tubes and functions with MIK1/2 as the male receptor of the pollen tube chemo-attractant LURE1.MDIS1 forms a complex with MIK1/2 and binds LURE1.
AT4G03110	-1,281	RNA-BINDING PROTEIN-DEFENSE RELATED 1 (RBP-	Encodes a putative RNA-binding protein that is located in the cytoplasm and is involved in the hypersensitive response and positively regulates salicylic acid-mediated immunity.
AT4G11880	-1,284	AGAMOUS-LIKE 14 (AGL14)	AGL12, AGL14, and AGL17 are all preferentially expressed in root tissues and therefore represent the only characterized MADS box genes expressed in roots. The mRNA is cell-to-cell mobile.
AT4G21590	-1,286	ENDONUCLEASE 3 (ENDO3)	Encodes a putative endonuclease but no demonstrable endonuclease activity, either towards single stranded DNA or mismatches, has been seen in vitro. Activated by AGAMOUS in a cal-1, ap1-1 background. Expressed in the floral meristem and during stamen development.
AT5G03550	-1,286		BEST Arabidopsis thaliana protein match is: TRAF-like family protein (TAIR:AT2G42460.1)

AT2G47460	*-1,288*	MYB DOMAIN PROTEIN 12 (MYB12)	MYB12 belongs to subgroup 7 of the R2R3-MYB family. It strongly activates the promoters of chalcone synthase (CHS), flavanone 3-hydroxylase (F3H), flavonol synthase (FLS) and - to a lesser extent - chalcone flavanone isomerase (CHI), but cannot activate the promoters of flavonoid-3'hydroxylase (F3'H) and dihydroflavonol 4-reductase (DF). The activation requires a functional MYB recognition element (MRE). Results from the myb12-1f allele indicate that an activation domain might be present in the C-terminus. Overexpression or knock-out plants do not show any obvious phenotype under greenhouse conditions. Young myb12-ko seedlings contain reduced amounts of flavonoids (quercetin and kaempferol), while seedlings as well as leaves of MYB12-OX plants displayed an increased flavonoid content. They did not show any significant difference in anthocyanin content. Expression of CHS and FLS shows a clear correlation to MYB12 expression levels. CHI and F3H show increased transcript levels in the MYB12-OX lines, but no differences in the knock-out. Even in the absence of functional MYB12, flavonol biosynthesis is not completely absent, suggesting functional redundancy. The redundant factors are MYB11 and MYB111 although MYB12 is primarily required for flavonol biosynthesis in roots. Mutations in MYB12 block both auxin and ethylene stimulation of flavonoid synthesis.
AT5G44410	*-1,29*	(ATBBE27)	FAD-binding Berberine family protein; FUNCTIONS IN: electron carrier activity, oxidoreductase activity, FAD binding, catalytic activity; INVOLVED IN: oxidation reduction; LOCATED IN: endomembrane system; EXPRESSED IN: 22 plant structures; EXPRESSED DURING: 13 growth stages; CONTAINS InterPro DOMAIN/s: FAD-binding, type 2 (InterPro:IPR016166), Oxygen oxidoreductase covalent FAD-binding site (InterPro:IPR006093), Berberine/berberine-like (InterPro:IPR012951), FAD linked oxidase, N-terminal (InterPro:IPR006094); BEST Arabidopsis thaliana protein match is: FAD-binding Berberine family protein (TAIR:AT5G44440.1)
AT5G66280	*-1,29*	GDP-D-MANNOSE 4,6-DEHYDRATASE 1 (GMD1)	GDP-D-mannose 4,6-dehydratase
AT1G48700	*-1,291*		2-oxoglutarate (2OG) and Fe(II)-dependent oxygenase superfamily protein; FUNCTIONS IN: oxidoreductase activity, acting on paired donors, with incorporation or reduction of molecular oxygen, L-ascorbic acid binding, iron ion binding; INVOLVED IN: oxidation reduction; LOCATED IN: cellular_component unknown; CONTAINS InterPro DOMAIN/s: Prolyl 4-hydroxylase, alpha subunit (InterPro:IPR006620); BEST Arabidopsis thaliana protein match is: 2-oxoglutarate (2OG) and Fe(II)-dependent oxygenase superfamily protein (TAIR:AT5G43660.1)
AT2G15890	*-1,292*	MATERNAL EFFECT EMBRYO ARREST 14 (MEE14)	Encodes CBP1, a regulator of transcription initiation in central cell-mediated pollen tube guidance.
AT5G12880	*-1,293*		proline-rich family protein; FUNCTIONS IN: molecular_function unknown; INVOLVED IN: biological_process unknown; LOCATED IN: endomembrane system; EXPRESSED IN: 10 plant structures; EXPRESSED DURING: LP.06 six leaves visible, LP.04 four leaves visible, LP.10 ten leaves visible, petal differentiation and expansion stage, LP.08 eight leaves visible
AT1G13300	*-1,294*	HYPERSENSITIVITY TO LOW PI-ELICITED PRIMARY ROOT SHORTENING 1 (HRS1)	Encodes a nuclear localized member of the GARP family of transcription factors. Involved in nitrate/phosphate signaling in roots. It is transcriptionally regulated by nitrate and post transcriptionally by phospate and functions to integrate these two nutrient signaling pathways in the root.
AT1G74660	*-1,294*	MINI ZINC FINGER 1 (MIF1)	Encodes MINI ZINC FINGER 1 (MIF1) which has a zinc finger domain but lacks other protein motifs normally present in transcription factors. MIF1 physically interact with a group of zinc finger-homeodomain (ZHD) transcription factors, such as ZHD5 (AT1G75240), that regulate floral architecture and leaf development. Gel mobility shift assays revealed that MIF1 blocks the DNA binding activity of ZHD5 homodimers by competitively forming MIF1-ZHD5 heterodimers. Constitutive overexpression of MIF1 caused dramatic developmental defects, seedlings were non-responsive to gibberellin (GA) for cell elongation, hypersensitive to the GA synthesis inhibitor paclobutrazol (PAC) and abscisic acid (ABA), and hyposensitive to auxin, brassinosteroid and cytokinin, but normally responsive to ethylene.
AT5G24330	*-1,294*	ARABIDOPSIS TRITHORAX-RELATED PROTEIN 6 (ATXR6)	Encodes a SET-domain protein, a H3K27 monomethyltransferases required for chromatin structure and gene silencing. Regulates heterochromatic DNA replication. Contains a PCNA-binding domain. ATXR6 accumulates preferentially during the late G1 or S phase, suggesting that it plays a role in cell-cycle regulation or progression.
AT4G05200	*-1,295*	CYSTEINE-RICH RLK (RECEPTOR-LIKE PROTEIN KINASE) 25 (CRK25)	Encodes a cysteine-rich receptor-like protein kinase.

AT2G30230	-1,297		unknown protein; FUNCTIONS IN: molecular_function unknown; INVOLVED IN: biological_process unknown; LOCATED IN: endomembrane system; BEST Arabidopsis thaliana protein match is: unknown protein (TAIR:AT1G06980.1)
AT5G10720	-1,297	HISTIDINE KINASE 5 (HK5)	member of Histidine Kinase
AT3G02170	-1,299	LONGIFOLIA2 (LNG2)	Encodes LONGIFOLIA2 (LNG2). Regulates leaf morphology by promoting cell expansion in the leaf-length direction. The LNG2 homologue LNG1 (At5g15580) has similar function.
AT1G08320	-1,3	TGACG (TGA) MOTIF-BINDING PROTEIN 9 (TGA9)	bZIP transcription factor family protein; FUNCTIONS IN: DNA binding, sequence-specific DNA binding transcription factor activity; INVOLVED IN: regulation of transcription, DNA-dependent; EXPRESSED IN: 9 plant structures; EXPRESSED DURING: 6 growth stages; CONTAINS InterPro DOMAIN/s: Basic-leucine zipper (bZIP) transcription factor (InterPro:IPR004827), bZIP transcription factor, bZIP-1 (InterPro:IPR011616); BEST Arabidopsis thaliana protein match is: bZIP transcription factor family protein (TAIR:AT5G06839.3)
AT2G28960	-1,302		Leucine-rich repeat protein kinase family protein; FUNCTIONS IN: protein serine/threonine kinase activity, protein kinase activity, kinase activity, ATP binding; INVOLVED IN: protein amino acid phosphorylation; LOCATED IN: endomembrane system; EXPRESSED IN: 6 plant structures; EXPRESSED DURING: 4 anthesis, C globular stage; CONTAINS InterPro DOMAIN/s: Protein kinase, ATP binding site (InterPro:IPR017441), Protein kinase, catalytic domain (InterPro:IPR000719), Serine/threonine-protein kinase-like domain (InterPro:IPR017442), Protein kinase-like domain (InterPro:IPR011009), Serine/threonine-protein kinase, active site (InterPro:IPR008271); BEST Arabidopsis thaliana protein match is: Leucine-rich repeat protein kinase family protein (TAIR:AT2G29000.1)
AT2G45360	-1,302		Protein of unknown function (DUF1442); CONTAINS InterPro DOMAIN/s: Protein of unknown function DUF1442 (InterPro:IPR009902); BEST Arabidopsis thaliana protein match is: Protein of unknown function (DUF1442) (TAIR:AT3G60780.1)
AT4G23010	-1,302	UDP-GALACTOSE TRANSPORTER 2 (UTR2)	UDP-galactose transporter 2 (UTR2); FUNCTIONS IN: molecular_function unknown; INVOLVED IN: transmembrane transport; LOCATED IN: cellular_component unknown; EXPRESSED IN: 24 plant structures; EXPRESSED DURING: 14 growth stages; CONTAINS InterPro DOMAIN/s: UAA transporter (InterPro:IPR013657); BEST Arabidopsis thaliana protein match is: UDP-N-acetylglucosamine (UAA) transporter family (TAIR:AT1G12600.1).
AT5G20250	-1,302	DARK INDUCIBLE 10 (DIN10)	encodes a member of glycosyl hydrolase family 36. Expression is induced within 3 hours of dark treatment, in senescing leaves and treatment with exogenous photosynthesis inhibitor. Induction of gene expression was suppressed in excised leaves supplied with sugar. The authors suggest that the gene's expression pattern is responding to the level of sugar in the cell. The mRNA is cell-to-cell mobile.
AT5G44900	-1,303		Toll-Interleukin-Resistance (TIR) domain family protein; FUNCTIONS IN: transmembrane receptor activity; INVOLVED IN: signal transduction, N-terminal protein myristoylation, defense response, innate immune response; LOCATED IN: intrinsic to membrane; CONTAINS InterPro DOMAIN/s: Toll-Interleukin receptor (InterPro:IPR000157); BEST Arabidopsis thaliana protein match is: Disease resistance protein (TIR-NBS-LRR class) family (TAIR:AT5G45000.1)
AT1G75910	-1,305	EXTRACELLULAR LIPASE 4 (EXL4)	member of Lipase proteins
AT5G09430	-1,306		alpha/beta-Hydrolases superfamily protein; CONTAINS InterPro DOMAIN/s: Alpha/beta hydrolase fold-1 (InterPro:IPR000073); BEST Arabidopsis thaliana protein match is: alpha/beta-Hydrolases superfamily protein (TAIR:AT4G39955.1)
AT1G10220	-1,307		BEST Arabidopsis thaliana protein match is: ZCF37 (TAIR:AT1G59590.1)
AT2G26670	-1,307	REVERSAL OF THE DET PHENOTYPE 4 (TED4)	Encodes a plastid heme oxygenase necessary for phytochrome chromophore biosynthesis and for coupling the expression of some nuclear genes to the functional state of the chloroplast.
AT3G32047	-1,308		Cytochrome P450 superfamily protein; FUNCTIONS IN: electron carrier activity, monooxygenase activity, iron ion binding, heme binding; INVOLVED IN: oxidation reduction; LOCATED IN: endomembrane system; CONTAINS InterPro DOMAIN/s: Cytochrome P450 (InterPro:IPR001128), Cytochrome P450, conserved site (InterPro:IPR017972), Cytochrome P450, E-class, group I (InterPro:IPR002401); BEST Arabidopsis thaliana protein match is: cytochrome P450, family 705, subfamily A, polypeptide 13 (TAIR:AT2G14100.1)
AT5G01050	-1,308		putative laccase, a member of laccase family of genes (17 members in Arabidopsis).

AT3G26120	*-1,311*	TERMINAL EAR1-LIKE 1 (TEL1)	Similar to terminal ear1 in Zea mays. A member of mei2-like gene family; phylogenetic analysis revealed that TEL1 belongs to the third clade of mei2-like proteins (TEL clade), with conserved two N-terminal RNA recognition motifs (RRM), in addition to the C-terminal RRM, shared among all mei2-like proteins.
AT3G14370	*-1,312*	(WAG2)	The WAG2 and its homolog, WAG1 each encodes protein-serine/threonine kinase that are nearly 70% identical to PsPK3 protein. All three together with CsPK3 belong to PsPK3-type kinases. At the N-terminus, all four possess a serine/threonine-rich domain. They are closely related to Arabidopsis kinases PINOID. wag1/wag2 double mutants exhibit a pronounced wavy root phenotype when grown vertically on agar plates (while wild-type plants develop wavy roots only on plates inclined to angles less than 90 degrees), indicating an overlapping role for WAG1 and WAG2 as suppressors of root waving. Simultaneous disruption of PID(AT2G34650) and its 3 closest homologs (PID2/AT2G26700, WAG1/AT1G53700, and WAG2/AT3G14370) abolishes the formation of cotyledons.
AT5G48290	*-1,312*		Heavy metal transport/detoxification superfamily protein ; FUNCTIONS IN: metal ion binding; INVOLVED IN: metal ion transport; LOCATED IN: cellular_component unknown; EXPRESSED IN: 6 plant structures; EXPRESSED DURING: 4 anthesis, petal differentiation and expansion stage; CONTAINS InterPro DOMAIN/s: Heavy metal transport/detoxification protein (InterPro:IPR006121); BEST Arabidopsis thaliana protein match is: Heavy metal transport/detoxification superfamily protein (TAIR:AT3G07600.1)
AT4G00820	*-1,313*	IQ-DOMAIN 17 (iqd17)	IQ-domain 17 (iqd17); CONTAINS InterPro DOMAIN/s: IQ calmodulin-binding region (InterPro:IPR000048); BEST Arabidopsis thaliana protein match is: IQ-domain 18 (TAIR:AT1G01110.2)
AT5G48320	*-1,313*		Cysteine/Histidine-rich C1 domain family protein; CONTAINS InterPro DOMAIN/s: DC1 (InterPro:IPR004146), C1-like (InterPro:IPR011424); BEST Arabidopsis thaliana protein match is: Cysteine/Histidine-rich C1 domain family protein (TAIR:AT4G15070.1)
AT2G43150	*-1,315*		Proline-rich extensin-like family protein; FUNCTIONS IN: structural constituent of cell wall; INVOLVED IN: plant-type cell wall organization; LOCATED IN: endomembrane system; EXPRESSED IN: 16 plant structures; EXPRESSED DURING: 12 growth stages; CONTAINS InterPro DOMAIN/s: Extensin-like repeat (InterPro:IPR006706)
AT5G55780	*-1,315*		Cysteine/Histidine-rich C1 domain family protein; FUNCTIONS IN: zinc ion binding; INVOLVED IN: intracellular signaling pathway; CONTAINS InterPro DOMAIN/s: Protein kinase C-like, phorbol ester/diacylglycerol binding (InterPro:IPR002219), Zinc finger, PHD-type, conserved site (InterPro:IPR019786), DC1 (InterPro:IPR004146), Zinc finger, PHD-type (InterPro:IPR001965), C1-like (InterPro:IPR011424); BEST Arabidopsis thaliana protein match is: Cysteine/Histidine-rich C1 domain family protein (TAIR:AT3G26550.1)
AT1G34520	*-1,316*		MBOAT (membrane bound O-acyl transferase) family protein; CONTAINS InterPro DOMAIN/s: Wax synthase (InterPro:IPR017088); BEST Arabidopsis thaliana protein match is: MBOAT (membrane bound O-acyl transferase) family protein (TAIR:AT1G34490.1)
AT2G04025	*-1,317*	ROOT MERISTEM GROWTH FACTOR 3 (RGF3)	Encodes a root meristem growth factor (RGF). Belongs to a family of functionally redundant homologous peptides that are secreted, tyrosine-sulfated, and expressed mainly in the stem cell area and the innermost layer of central columella cells. RGFs are required for maintenance of the root stem cell niche and transit amplifying cell proliferation. Members of this family include: At5g60810 (RGF1), At1g13620 (RGF2), At2g04025 (RGF3), At3g30350 (RGF4), At5g51451 (RGF5), At4g16515 (RGF6), At3g02240 (RGF7), At2g03830 (RGF8) and At5g64770 (RGF9).
AT5G44380	*-1,317*	(ATBBE28)	FAD-binding Berberine family protein; FUNCTIONS IN: electron carrier activity, oxidoreductase activity, FAD binding, catalytic activity; INVOLVED IN: response to oxidative stress; LOCATED IN: cell wall; EXPRESSED IN: shoot apex, hypocotyl, sepal, root, stamen; EXPRESSED DURING: 4 anthesis; CONTAINS InterPro DOMAIN/s: FAD-linked oxidase, FAD-binding, subdomain 2 (InterPro:IPR016168), FAD-binding, type 2 (InterPro:IPR016166), Berberine/berberine-like (InterPro:IPR012951), FAD linked oxidase, N-terminal (InterPro:IPR006094); BEST Arabidopsis thaliana protein match is: FAD-binding Berberine family protein (TAIR:AT5G44390.1)

AT5G02360	*-1,319*		DC1 domain-containing protein; FUNCTIONS IN: zinc ion binding; INVOLVED IN: biological_process unknown; LOCATED IN: intracellular; CONTAINS InterPro DOMAIN/s: DC1 (InterPro:IPR004146), Zinc finger, RanBP2-type (InterPro:IPR001876), C1-like (InterPro:IPR011424); BEST Arabidopsis thaliana protein match is: Cysteine/Histidine-rich C1 domain family protein (TAIR:AT1G53340.1)
AT5G07450	*-1,319*	CYCLIN P4;3 (CYCP4;3)	cyclin p4;3 (CYCP4;3); CONTAINS InterPro DOMAIN/s: Negative regulatory factor PREG (InterPro:IPR012389), Cyclin-like (InterPro:IPR011028), Cyclin-related 2 (InterPro:IPR013922), Cyclin (InterPro:IPR006670); BEST Arabidopsis thaliana protein match is: CYCLIN P4;2 (TAIR:AT5G61650.1)
AT5G62165	*-1,319*	AGAMOUS-LIKE 42 (AGL42)	Encodes a MADS box transcription factor. Expressed in quiescent center. Involved in floral transition.
AT2G28755	*-1,32*		UDP-D-glucuronate carboxy-lyase-related; BEST Arabidopsis thaliana protein match is: UDP-XYL synthase 6 (TAIR:AT2G28760.3)
AT4G12470	*-1,32*	AZELAIC ACID INDUCED 1 (AZI1)	Encodes AZI1 (AZELAIC ACID INDUCED 1). Involved in the priming of salicylic acid induction and systemic immunity triggered by pathogen or azelaic acid. Targeting if AZI1 to chloroplasts is increased during SAR induction and that localization requires the PRR domain.It is involved in the uptake and movement of the azelaic acid signal.
AT2G23690	*-1,324*		unknown protein; FUNCTIONS IN: molecular_function unknown; INVOLVED IN: N-terminal protein myristoylation; LOCATED IN: cellular_component unknown; EXPRESSED IN: 7 plant structures; EXPRESSED DURING: petal differentiation and expansion stage; BEST Arabidopsis thaliana protein match is: unknown protein (TAIR:AT4G37240.1)
AT4G10510	*-1,324*		Subtilase family protein; FUNCTIONS IN: identical protein binding, serine-type endopeptidase activity; INVOLVED IN: proteolysis, negative regulation of catalytic activity; EXPRESSED IN: male gametophyte, root; CONTAINS InterPro DOMAIN/s: Protease-associated PA (InterPro:IPR003137), Proteinase inhibitor, propeptide (InterPro:IPR009020), Peptidase S8/S53, subtilisin/kexin/sedolisin (InterPro:IPR000209), Peptidase S8, subtilisin-related (InterPro:IPR015500), Peptidase S8/S53, subtilisin, active site (InterPro:IPR022398), Proteinase inhibitor I9, subtilisin propeptide (InterPro:IPR010259); BEST Arabidopsis thaliana protein match is: Subtilase family protein (TAIR:AT4G10540.1)
AT4G36380	*-1,324*	ROTUNDIFOLIA 3 (ROT3)	Encodes a cytochrome P-450 gene that is involved in leaf blade expansion by controlling polar cell expansion in the leaf length direction. Member of the CYP90C CYP450 family. ROT3 was shown to be involved in brassinosteroid biosynthesis, most likely in the conversion step of typhasterol (TY) to castasterone (CS). As 6-deoxo-CS was unable to restore the phenotype of rot3-1, it has been postulated that ROT3 might be specifically involved in the conversion of TY to CS in the C6-oxidation pathway of brassinolide. Recently, CYP90C1 was shown to catalyse the C-23 hydroxylation of several brassinosteroids (the enzyme has a broad specificity for 22-hydroxylated substrates).
AT5G38940	*-1,324*		RmlC-like cupins superfamily protein; FUNCTIONS IN: manganese ion binding, nutrient reservoir activity; INVOLVED IN: response to salt stress; LOCATED IN: cell wall; EXPRESSED IN: fruit, root; CONTAINS InterPro DOMAIN/s: Cupin, RmlC-type (InterPro:IPR011051), Cupin 1 (InterPro:IPR006045), RmlC-like jelly roll fold (InterPro:IPR014710), Germin (InterPro:IPR001929), Germin, manganese binding site (InterPro:IPR019780); BEST Arabidopsis thaliana protein match is: RmlC-like cupins superfamily protein (TAIR:AT5G38930.1)
AT4G01680	*-1,325*	MYB DOMAIN PROTEIN 55 (MYB55)	Encodes a putative transcription factor (MYB55).
AT1G02390	*-1,326*	GLYCEROL-3-PHOSPHATE SN-2-ACYLTRANSFERASE 2 (GPAT2)	putative sn-glycerol-3-phosphate 2-O-acyltransferase
AT3G50460	*-1,326*	HOMOLOG OF RPW8 2 (HR2)	Homolog of RPW8
AT1G68740	*-1,327*	(PHO1;H1)	Encodes PHO1;H1, a member of the PHO1 family. Involved in inorganic phosphate (Pi) transport and homeostasis. Complements pho1 mutation. Its expression is responsive to both phosphate (Pi) and phosphite (Phi) in shoots.
AT3G26330	*-1,328*	CYTOCHROME P450, FAMILY 71, SUBFAMILY B, POLYPEPTIDE 37 (CYP71B37)	putative cytochrome P450
AT5G24240	*-1,328*	(ATPI4Kγ3)	Encodes PI4Kc3, localizes to the nucleus and has autophosphorylation activity, but no lipid kinase activity. Overexpression mutants display late-flowering phenotype.

AT2G38460	*-1,329*	IRON REGULATED 1 (IREG1)	Encodes IRON REGULATED1 (IREG1/FPN1), one of the Arabidopsis orthologs (AT2G38460/IREG1/FPN1 and AT5G03570/IREG2/FPN2) the iron efflux transporter ferroportin (FPN) identified in animals.
AT5G04200	*-1,329*	METACASPASE 9 (MC9)	Encodes a putative metacaspase. Arabidopsis contains three type I MCP genes (MCP1a-c) and six type II MCP genes (MCP2a?f): AtMCP1a/At5g64240, AtMCP1b/At1g02170, AtMCP1c/At4g25110, AtMCP2a/At1g79310, AtMCP2b/At1g79330, AtMCP2c/At1g79320, AtMCP2d/At1g79340, AtMCP2e/At1g16420, AtMCP2f/At5g04200.
AT2G44390	*-1,33*		Cysteine/Histidine-rich C1 domain family protein; CONTAINS InterPro DOMAIN/s: DC1 (InterPro:IPR004146), C1-like (InterPro:IPR011424); BEST Arabidopsis thaliana protein match is: Cysteine/Histidine-rich C1 domain family protein (TAIR:AT2G44380.1)
AT4G03380	*-1,33*		BEST Arabidopsis thaliana protein match is: nuclear assembly factor 1 (TAIR:AT1G03530.1)
AT5G26280	*-1,33*		TRAF-like family protein; FUNCTIONS IN: molecular_function unknown; INVOLVED IN: biological_process unknown; LOCATED IN: membrane; EXPRESSED IN: 8 plant structures; EXPRESSED DURING: seedling growth; CONTAINS InterPro DOMAIN/s: TRAF-like (InterPro:IPR008974), MATH (InterPro:IPR002083); BEST Arabidopsis thaliana protein match is: TRAF-like family protein (TAIR:AT5G26300.1)
AT5G53110	*-1,33*		RING/U-box superfamily protein; FUNCTIONS IN: zinc ion binding; INVOLVED IN: biological_process unknown; LOCATED IN: endomembrane system; EXPRESSED IN: 8 plant structures; EXPRESSED DURING: 4 anthesis, C globular stage, petal differentiation and expansion stage; CONTAINS InterPro DOMAIN/s: Zinc finger, RING-type (InterPro:IPR001841), Zinc finger, C3HC4 RING-type (InterPro:IPR018957); BEST Arabidopsis thaliana protein match is: RING/U-box superfamily protein (TAIR:AT2G46495.1)
AT1G14870	*-1,332*	PLANT CADMIUM RESISTANCE 2 (PCR2)	PCR2 encodes a membrane protein involved in zinc transport and detoxification.
AT1G49500	*-1,333*		unknown protein; FUNCTIONS IN: molecular_function unknown; INVOLVED IN: biological_process unknown; LOCATED IN: endomembrane system; EXPRESSED IN: 19 plant structures; EXPRESSED DURING: 10 growth stages; BEST Arabidopsis thaliana protein match is: unknown protein (TAIR:AT3G19030.1)
AT3G27170	*-1,333*	CHLORIDE CHANNEL B (CLC-B)	member of Anion channel protein family The mRNA is cell-to-cell mobile.
AT1G66270	*-1,334*	(BGLU21)	BGLU21 encodes a beta-glucosidase that has a high level of activity against the naturally occuring secondary metabolite scopolin. Recombinant BGLU21 produced in insect cells shows lower levels of activity using the structurally similar substrates esculin (33%) and 4-MU-glucoside (9%). BGLU21 is glycosylated in planta and has a C-terminal ER retention signal. Transcript analysis as well as protein extraction and western blotting from various plant organs suggest that BGLU21 is only present in roots and seedlings. Transcript levels increase in response to cold, auxin (2,4-D), and methyl jasmonate treatments.
AT1G77520	*-1,335*		O-methyltransferase family protein; CONTAINS InterPro DOMAIN/s: Winged helix-turn-helix transcription repressor DNA-binding (InterPro:IPR011991), Plant methyltransferase dimerisation (InterPro:IPR012967), O-methyltransferase, family 2 (InterPro:IPR001077), O-methyltransferase, COMT, eukaryota (InterPro:IPR016461); BEST Arabidopsis thaliana protein match is: O-methyltransferase family protein (TAIR:AT1G77530.1)
AT3G47770	*-1,335*	ATP-BINDING CASSETTE A6 (ABCA6)	member of ATH subfamily
AT5G66590	*-1,335*		CAP (Cysteine-rich secretory proteins, Antigen 5, and Pathogenesis-related 1 protein) superfamily protein; FUNCTIONS IN: molecular_function unknown; INVOLVED IN: biological_process unknown; LOCATED IN: endomembrane system, extracellular region; EXPRESSED IN: 22 plant structures; EXPRESSED DURING: 13 growth stages; CONTAINS InterPro DOMAIN/s: Allergen V5/Tpx-1 related (InterPro:IPR001283), SCP-like extracellular (InterPro:IPR014044); BEST Arabidopsis thaliana protein match is: CAP (Cysteine-rich secretory proteins, Antigen 5, and Pathogenesis-related 1 protein) superfamily protein (TAIR:AT5G57625.1)
AT4G37340	*-1,336*	CYTOCHROME P450, FAMILY 81, SUBFAMILY D, POLYPEPTIDE 3 (CYP81D3)	member of CYP81D

AT1G28310	*-1,338*		Dof-type zinc finger DNA-binding family protein; CONTAINS InterPro DOMAIN/s: Zinc finger, Dof-type (InterPro:IPR003851); BEST Arabidopsis thaliana protein match is: OBF-binding protein 3 (TAIR:AT3G55370.3)
AT1G11540	*-1,339*		Sulfite exporter TauE/SafE family protein; FUNCTIONS IN: molecular_function unknown; INVOLVED IN: biological_process unknown; LOCATED IN: endomembrane system, integral to membrane; EXPRESSED IN: 13 plant structures; EXPRESSED DURING: M germinated pollen stage, 4 anthesis, C globular stage, 4 leaf senescence stage, petal differentiation and expansion stage; CONTAINS InterPro DOMAIN/s: Protein of unknown function DUF81 (InterPro:IPR002781); BEST Arabidopsis thaliana protein match is: Sulfite exporter TauE/SafE family protein (TAIR:AT1G61740.1)
AT5G50820	*-1,339*	NAC DOMAIN CONTAINING PROTEIN 97 (NAC097)	NAC domain containing protein 97 (NAC097); FUNCTIONS IN: sequence-specific DNA binding transcription factor activity; INVOLVED IN: multicellular organismal development, regulation of transcription; LOCATED IN: cellular_component unknown; EXPRESSED IN: 9 plant structures; EXPRESSED DURING: 6 growth stages; CONTAINS InterPro DOMAIN/s: No apical meristem (NAM) protein (InterPro:IPR003441); BEST Arabidopsis thaliana protein match is: NAC domain containing protein 83 (TAIR:AT5G13180.1)
AT1G13100	*-1,34*	CYTOCHROME P450, FAMILY 71, SUBFAMILY B, POLYPEPTIDE 29 (CYP71B29)	putative cytochrome P450
AT4G17800	*-1,34*	AT-HOOK MOTIF NUCLEAR LOCALIZED PROTEIN 23 (AHL23)	Predicted AT-hook DNA-binding family protein; CONTAINS InterPro DOMAIN/s: Protein of unknown function DUF296 (InterPro:IPR005175), Predicted AT-hook DNA-binding (InterPro:IPR014476); BEST Arabidopsis thaliana protein match is: Predicted AT-hook DNA-binding family protein (TAIR:AT2G35270.1)
AT5G09220	*-1,34*	AMINO ACID PERMEASE 2 (AAP2)	member of AAAP family The mRNA is cell-to-cell mobile.
AT3G60870	*-1,342*	AT-HOOK MOTIF NUCLEAR-LOCALIZED PROTEIN 18 (AHL18)	Encodes an AT hook domain containing protein that, when overexpressed, delays flowering.
AT4G20210	*-1,343*	TERPENE SYNTHASE 8 (TPS08)	Terpenoid cyclases/Protein prenyltransferases superfamily protein; FUNCTIONS IN: lyase activity, magnesium ion binding; INVOLVED IN: metabolic process; LOCATED IN: chloroplast; EXPRESSED IN: root; CONTAINS InterPro DOMAIN/s: Terpene synthase, metal-binding domain (InterPro:IPR005630), Terpenoid synthase (InterPro:IPR008949), Terpenoid cylases/protein prenyltransferase alpha-alpha toroid (InterPro:IPR008930), Terpene synthase-like (InterPro:IPR001906); BEST Arabidopsis thaliana protein match is: Terpenoid cyclases/Protein prenyltransferases superfamily protein (TAIR:AT4G20200.1)
AT5G44160	*-1,345*	NUTCRACKER (NUC)	nutcracker (NUC); FUNCTIONS IN: sequence-specific DNA binding transcription factor activity, zinc ion binding, nucleic acid binding; INVOLVED IN: regulation of transcription; LOCATED IN: intracellular; EXPRESSED IN: primary root apical meristem, stem vascular system, embryo, root, embryonic root; CONTAINS InterPro DOMAIN/s: Zinc finger, C2H2-like (InterPro:IPR015880), Zinc finger, C2H2-type (InterPro:IPR007087); BEST Arabidopsis thaliana protein match is: C2H2 and C2HC zinc fingers superfamily protein (TAIR:AT1G03840.1)
AT5G19560	*-1,348*	ROP (RHO OF PLANTS) GUANINE NUCLEOTIDE EXCHANGE FACTOR 10 (ROPGEF10)	Encodes a member of KPP-like gene family, homolog of KPP (kinase partner protein) gene in tomato. Also a member of the RopGEF (guanine nucleotide exchange factor) family, containing the novel PRONE domain (plant-specific Rop nucleotide exchanger), which is exclusively active towards members of the Rop subfamily.
AT1G56550	*-1,351*	RHAMNOGALACTURONAN SPECIﬁC XYLOSYLTRANSFERASE 3 (RGXT3)	Encodes a rhamnogalacturonan II specific xylosyltransferase.
AT1G60095	*-1,351*		Mannose-binding lectin superfamily protein; CONTAINS InterPro DOMAIN/s: Mannose-binding lectin (InterPro:IPR001229); BEST Arabidopsis thaliana protein match is: Mannose-binding lectin superfamily protein (TAIR:AT1G60110.1)
AT3G55150	*-1,351*	EXOCYST SUBUNIT EXO70 FAMILY PROTEIN H1 (EXO70H1)	A member of EXO70 gene family, putative exocyst subunits, conserved in land plants. Arabidopsis thaliana contains 23 putative EXO70 genes, which can be classified into eight clusters on the phylogenetic tree.

AT3G60530	-1,351	GATA TRANSCRIPTION FACTOR 4 (GATA4)	Encodes a member of the GATA factor family of zinc finger transcription factors.
AT1G22160	-1,352		Protein of unknown function (DUF581); CONTAINS InterPro DOMAIN/s: Protein of unknown function DUF581 (InterPro:IPR007650); BEST Arabidopsis thaliana protein match is: Protein of unknown function (DUF581) (TAIR:AT1G78020.1)
AT2G36500	-1,352		CBS / octicosapeptide/Phox/Bemp1 (PB1) domains-containing protein; CONTAINS InterPro DOMAIN/s: Octicosapeptide/Phox/Bem1p (InterPro:IPR000270), Cystathionine beta-synthase, core (InterPro:IPR000644); BEST Arabidopsis thaliana protein match is: CBS / octicosapeptide/Phox/Bemp1 (PB1) domains-containing protein (TAIR:AT3G52950.2)
AT3G47220	-1,353	PHOSPHATIDYLINOSITOL-SPECIFIC PHOSPHOLIPASE C9 (PLC9)	Encodes a plasma membrane-localized phosphoinositide-specific phospholipase C with a role in thermotolerance.
AT5G04120	-1,353		Encodes a cofactor-dependent phosphoglycerate mutase (dPGM) - like protein with phosphoserine phosphatase activity that may be responsible for serine anabolism.
AT2G39370	-1,354	MEMBRANE-ASSOCIATED KINASE REGULATOR 4 (MAKR4)	Encodes a member of the MAKR (MEMBRANE-ASSOCIATED KINASE REGULATOR) gene family. MAKRs have putative kinase interacting motifs and membrane localization signals. Known members include: AT5G26230 (MAKR1), AT1G64080 (MAKR2), AT2G37380 (MAKR3), AT2G39370 (MAKR4), AT5G52870 (MAKR5) and AT5G52900 (MAKR6).
AT3G23050	-1,354	INDOLE-3-ACETIC ACID 7 (IAA7)	Transcription regulator acting as repressor of auxin-inducible gene expression. Plays role in the control of gravitropic growth and development in light-grown seedlings. Auxin induces the degradation of the protein in a dosage-dependent manner in a process mediated by AtRac1. Auxin induced the relocalization of the protein within the nucleus from a diffused nucleoplasmic pattern to a discrete particulated pattern named nuclear protein bodies or NPB in a process also mediated by Rac1. Colocalizes with SCF, CSN and 26S proteasome components. Pseudomonas syringae type III effector AvrRpt2 stimulates AXR2 protein turnover.
AT2G26980	-1,356	CBL-INTERACTING PROTEIN KINASE 3 (CIPK3)	encodes a serine-threonine protein kinase whose expression increases in response to abscisic acid, cold, drought, high salt, and wounding conditions. The gene is expressed in developing seeds and seedlings. Lines carrying a T-DNA insertions have reduced germination efficiency and expression of cold, high-salt, and abscisic acid marker genes are altered, but not drought-response markers.
AT2G44740	-1,357	CYCLIN P4;1 (CYCP4;1)	cyclin p4;1 (CYCP4;1); CONTAINS InterPro DOMAIN/s: Negative regulatory factor PREG (InterPro:IPR012389), Cyclin-like (InterPro:IPR011028), Cyclin, N-terminal (InterPro:IPR006671), Cyclin (InterPro:IPR006670); BEST Arabidopsis thaliana protein match is: CYCLIN P4;2 (TAIR:AT5G61650.1)
AT4G02380	-1,357	SENESCENCE-ASSOCIATED GENE 21 (SAG21)	Encodes AtLEA5 (late embryogenesis abundant like protein). Also known as SENESCENCE-ASSOCIATED GENE 21 (SAG21). Has a role on oxidative stress tolerance. mRNA levels are elevated in response to various stresses.
AT5G51310	-1,359		Mutants exhibit longer root hairs under phosphate-deficient conditions.
AT4G31910	-1,361	BR-RELATED ACYLTRANSFERASE1 (BAT1)	Encodes an acyltransferase that can modify brassinosteroids (BRs) by acylation and may modulate endogenous BR levels.
AT5G37550	-1,361		unknown protein; BEST Arabidopsis thaliana protein match is: unknown protein (TAIR:AT1G66190.1)
AT1G69040	-1,363	ACT DOMAIN REPEAT 4 (ACR4)	ACT-domain containing protein involved in feedback regulation of amino acid metabolism
AT1G12090	-1,366	EXTENSIN-LIKE PROTEIN (ELP)	extensin-like protein (ELP)
AT1G62370	-1,367		RING/U-box superfamily protein; FUNCTIONS IN: zinc ion binding; CONTAINS InterPro DOMAIN/s: Zinc finger, RING-type (InterPro:IPR001841), Zinc finger, C3HC4 RING-type (InterPro:IPR018957); BEST Arabidopsis thaliana protein match is: RING/U-box superfamily protein (TAIR:AT4G03965.1)
AT1G60460	-1,368	MEIOTIC TOPOISOMERASE VIB-LIKE (MTOPVIB)	Encodes a structural homolog of the archaeal topo VIB subunit that forms a complex with the two Arabidopsis thaliana SPO11 orthologs required for meiotic DSB formation (SPO11-1 and SPO11-2) and is essential for meiotic DSB formation.
AT4G30640	-1,369		RNI-like superfamily protein; CONTAINS InterPro DOMAIN/s: Leucine-rich repeat (InterPro:IPR001611); BEST Arabidopsis thaliana protein match is: SKP1 interacting partner 1 (TAIR:AT5G57900.1)

AT1G20840	*-1,37*	TONOPLAST MONOSACCHARIDE TRANSPORTER1 (TMT1)	The protein encoded by this gene is found in the tonoplast (vacuole membrane) of Arabidopsis cells. The gene is expressed at highest levels in juvenile (sink) and adult (source) leaves, followed by flower tissues.
AT1G27670	*-1,37*		unknown protein; BEST Arabidopsis thaliana protein match is: unknown protein (TAIR:AT1G75360.1)
AT1G01180	*-1,371*		S-adenosyl-L-methionine-dependent methyltransferases superfamily protein; FUNCTIONS IN: methyltransferase activity; INVOLVED IN: lipid biosynthetic process; EXPRESSED IN: sperm cell, hypocotyl; CONTAINS InterPro DOMAIN/s: Rhamnosyl O-methyltransferase/Cephalosporin hydroxylase (InterPro:IPR007072)
AT5G43030	*-1,373*		Cysteine/Histidine-rich C1 domain family protein; INVOLVED IN: intracellular signaling pathway; LOCATED IN: cellular_component unknown; EXPRESSED IN: male gametophyte, root, flower, pollen tube; EXPRESSED DURING: L mature pollen stage, M germinated pollen stage, 4 anthesis; CONTAINS InterPro DOMAIN/s: Protein kinase C-like, phorbol ester/diacylglycerol binding (InterPro:IPR002219), DC1 (InterPro:IPR004146), C1-like (InterPro:IPR011424); BEST Arabidopsis thaliana protein match is: Cysteine/Histidine-rich C1 domain family protein (TAIR:AT5G43040.1)
AT1G48330	*-1,374*		unknown protein; BEST Arabidopsis thaliana protein match is: unknown protein (TAIR:AT3G17580.1)
AT5G67420	*-1,374*	LOB DOMAIN-CONTAINING PROTEIN 37 (LBD37)	Encodes a LOB-domain protein involved in nitrogen metabolism and affecting leaf morphogenesis.
AT1G64900	*-1,375*	CYTOCHROME P450, FAMILY 89, SUBFAMILY A, POLYPEPTIDE 2 (CYP89A2)	Encodes cytochrome P450 (CYP89A2). The mRNA is cell-to-cell mobile.
AT5G43350	*-1,376*	PHOSPHATE TRANSPORTER 1;1 (PHT1;1)	Encodes an inorganic phosphate transporter Pht1;1. Mutants display enhanced arsenic accumulation. Members of the Pht1 family of phosphate transporters include: Pht1;1/At5g43350, Pht1;2/At5g43370, Pht1;3/At5g43360, Pht1;4/At2g38940, Pht1;5/At2g32830, Pht1;6/At5g43340, Pht1;7/At3g54700, Pht1;8/At1g20860, Pht1;9/At1g76430 (Plant Journal 2002, 31:341).
AT5G53380	*-1,376*		O-acyltransferase (WSD1-like) family protein; CONTAINS InterPro DOMAIN/s: O-acyltransferase, WSD1, C-terminal (InterPro:IPR009721), O-acyltransferase, WSD1, N-terminal (InterPro:IPR004255); BEST Arabidopsis thaliana protein match is: O-acyltransferase (WSD1-like) family protein (TAIR:AT5G12420.1)
AT1G23540	*-1,378*	PROLINE-RICH EXTENSIN-LIKE RECEPTOR KINASE 12 (PERK12)	Encodes a member of the PERK family of putative receptor kinases. Overexpression leads to morphological defects and reduced fertility and increased expression of MAX genes.
AT2G46950	*-1,378*	CYTOCHROME P450, FAMILY 709, SUBFAMILY B, POLYPEPTIDE 2 (CYP709B2)	member of CYP709B
AT1G51480	*-1,383*	RESISTANCE SILENCED GENE 1 (RSG1)	Disease resistance protein (CC-NBS-LRR class) family; FUNCTIONS IN: ATP binding; INVOLVED IN: apoptosis, defense response; LOCATED IN: cellular_component unknown; CONTAINS InterPro DOMAIN/s: Leucine-rich repeat-containing protein (InterPro:IPR015766), NB-ARC (InterPro:IPR002182), Disease resistance protein (InterPro:IPR000767); BEST Arabidopsis thaliana protein match is: Disease resistance protein (CC-NBS-LRR class) family (TAIR:AT5G43730.1)
AT1G62440	*-1,385*	LEUCINE-RICH REPEAT/EXTENSIN 2 (LRX2)	encodes a paralog of LRX1 (LEUCINE-RICH REPEAT/EXTENSIN 1) which acts synergistically with LRX1 in root hair cell morphogenesis.
AT5G54230	*-1,385*	MYB DOMAIN PROTEIN 49 (MYB49)	Encodes a putative transcription factor (MYB49).
AT2G15880	*-1,386*		Leucine-rich repeat (LRR) family protein; FUNCTIONS IN: structural constituent of cell wall; LOCATED IN: endomembrane system; EXPRESSED IN: 15 plant structures; EXPRESSED DURING: 8 growth stages; CONTAINS InterPro DOMAIN/s: Leucine-rich repeat (InterPro:IPR001611); BEST Arabidopsis thaliana protein match is: Leucine-rich repeat (LRR) family protein (TAIR:AT4G33970.1)
AT1G08100	*-1,387*	NITRATE TRANSPORTER 2.2 (NRT2.2)	Encodes a high-affinity nitrate transporter.

AT1G66930	*-1,387*	LEAF RUST 10 DISEASE-RESISTANCE LOCUS RECEPTOR- LIKE PROTEIN KINASE-LIKE2 (LRK10L2)	Protein kinase superfamily protein; FUNCTIONS IN: kinase activity; INVOLVED IN: protein amino acid phosphorylation; LOCATED IN: cellular_component unknown; EXPRESSED IN: root; CONTAINS InterPro DOMAIN/s: Protein kinase, ATP binding site (InterPro:IPR017441), Serine/threonine-protein kinase domain (InterPro:IPR002290), Serine-threonine/tyrosine-protein kinase (InterPro:IPR001245), Protein kinase-like domain (InterPro:IPR011009), Serine/threonine-protein kinase, active site (InterPro:IPR008271), Protein kinase, catalytic domain (InterPro:IPR000719), Tyrosine-protein kinase, catalytic domain (InterPro:IPR020635); BEST Arabidopsis thaliana protein match is: Protein kinase superfamily protein (TAIR:AT1G67000.1)
AT5G50140	*-1,387*		Ankyrin repeat family protein; CONTAINS InterPro DOMAIN/s: Ankyrin repeat-containing domain (InterPro:IPR020683), Ankyrin repeat (InterPro:IPR002110); BEST Arabidopsis thaliana protein match is: Ankyrin repeat family protein (TAIR:AT1G34050.1)
AT5G50760	*-1,387*	SMALL AUXIN UPREGULATED RNA 55 (SAUR55)	SAUR-like auxin-responsive protein family ; CONTAINS InterPro DOMAIN/s: Auxin responsive SAUR protein (InterPro:IPR003676); BEST Arabidopsis thaliana protein match is: SAUR-like auxin-responsive protein family (TAIR:AT3G43120.1)
AT3G23730	*-1,388*	XYLOGLUCAN ENDOTRANSGLUCOSYLASE/HYDROLASE 16 (XTH16)	xyloglucan endotransglucosylase/hydrolase 16 (XTH16); FUNCTIONS IN: hydrolase activity, acting on glycosyl bonds, hydrolase activity, hydrolyzing O-glycosyl compounds, xyloglucan:xyloglucosyl transferase activity; INVOLVED IN: carbohydrate metabolic process, cellular glucan metabolic process; LOCATED IN: endomembrane system, cell wall, apoplast; EXPRESSED IN: 22 plant structures; EXPRESSED DURING: 13 growth stages; CONTAINS InterPro DOMAIN/s: Xyloglucan endotransglucosylase/hydrolase (InterPro:IPR016455), Xyloglucan endo-transglycosylase, C-terminal (InterPro:IPR010713), Concanavalin A-like lectin/glucanase, subgroup (InterPro:IPR013320), Concanavalin A-like lectin/glucanase (InterPro:IPR008985), Glycoside hydrolase, family 16, active site (InterPro:IPR008263), Glycoside hydrolase, family 16 (InterPro:IPR000757); BEST Arabidopsis thaliana protein match is: xyloglucan endotransglucosylase/hydrolase 15 (TAIR:AT4G14130.1)
AT1G73860	*-1,389*		P-loop containing nucleoside triphosphate hydrolases superfamily protein; FUNCTIONS IN: microtubule motor activity, ATP binding; INVOLVED IN: microtubule-based movement; LOCATED IN: chloroplast; EXPRESSED IN: petal, sepal, male gametophyte, flower, pollen tube; EXPRESSED DURING: L mature pollen stage, M germinated pollen stage, 4 anthesis, petal differentiation and expansion stage; CONTAINS InterPro DOMAIN/s: Kinesin, motor domain (InterPro:IPR001752); BEST Arabidopsis thaliana protein match is: P-loop containing nucleoside triphosphate hydrolases superfamily protein (TAIR:AT1G18410.1)
AT2G23050	*-1,391*	NAKED PINS IN YUC MUTANTS 4 (NPY4)	A member of the NPY gene family (NPY1/AT4G31820, NPY2/AT2G14820, NPY3/AT5G67440, NPY4/AT2G23050, NPY5/AT4G37590). Involved in auxin-mediated organogenesis.
AT1G70990	*-1,393*		proline-rich family protein; BEST Arabidopsis thaliana protein match is: hydroxyproline-rich glycoprotein family protein (TAIR:AT1G23040.1)
AT4G23680	*-1,394*		Polyketide cyclase/dehydrase and lipid transport superfamily protein; FUNCTIONS IN: molecular_function unknown; INVOLVED IN: response to biotic stimulus, defense response; LOCATED IN: cellular_component unknown; EXPRESSED IN: 19 plant structures; EXPRESSED DURING: 9 growth stages; CONTAINS InterPro DOMAIN/s: Bet v I allergen (InterPro:IPR000916); BEST Arabidopsis thaliana protein match is: Polyketide cyclase/dehydrase and lipid transport superfamily protein (TAIR:AT4G23670.1)
AT2G37950	*-1,395*		RING/FYVE/PHD zinc finger superfamily protein; FUNCTIONS IN: zinc ion binding; EXPRESSED IN: 10 plant structures; EXPRESSED DURING: 6 growth stages; CONTAINS InterPro DOMAIN/s: Zinc finger, C3HC4 RING-type (InterPro:IPR018957), Zinc finger, RING-CH-type (InterPro:IPR011016); BEST Arabidopsis thaliana protein match is: RING/FYVE/PHD zinc finger superfamily protein (TAIR:AT5G05830.1)
AT1G17180	*-1,398*	GLUTATHIONE S-TRANSFERASE TAU 25 (GSTU25)	Encodes glutathione transferase belonging to the tau class of GSTs. Naming convention according to Wagner et al. (2002). Detoxification of the environmental pollutant 2,4,6-trinitrotoluene. Arabidopsis plant over-expressing At1g17180 were more resistant to TNT, removed more TNT from sterile and soil-based media, and had reduced levels of glutathione when grown in the presence of TNT.

AT1G52910	*-1,399*		Protein of unknown function (DUF1218); FUNCTIONS IN: molecular_function unknown; INVOLVED IN: biological_process unknown; LOCATED IN: endomembrane system; EXPRESSED IN: 20 plant structures; EXPRESSED DURING: 13 growth stages; CONTAINS InterPro DOMAIN/s: Protein of unknown function DUF1218 (InterPro:IPR009606); BEST Arabidopsis thaliana protein match is: Protein of unknown function (DUF1218) (TAIR:AT3G15480.1)
AT5G25110	*-1,399*	CBL-INTERACTING PROTEIN KINASE 25 (CIPK25)	member of AtCIPKs
AT5G62150	*-1,399*		peptidoglycan-binding LysM domain-containing protein; FUNCTIONS IN: molecular_function unknown; INVOLVED IN: cell wall macromolecule catabolic process; LOCATED IN: endomembrane system; EXPRESSED IN: 15 plant structures; EXPRESSED DURING: 6 growth stages; BEST Arabidopsis thaliana protein match is: peptidoglycan-binding LysM domain-containing protein (TAIR:AT4G25433.1)
AT3G21260	*-1,4*	GLYCOLIPID TRANSFER PROTEIN 3 (GLTP3)	Glycolipid transfer protein (GLTP) family protein; FUNCTIONS IN: glycolipid transporter activity, glycolipid binding; INVOLVED IN: glycolipid transport; LOCATED IN: cytoplasm; EXPRESSED IN: 20 plant structures; EXPRESSED DURING: 13 growth stages; CONTAINS InterPro DOMAIN/s: Glycolipid transfer protein, GLTP (InterPro:IPR014830); BEST Arabidopsis thaliana protein match is: glycolipid transfer protein 2 (TAIR:AT1G21360.1)
AT5G48100	*-1,4*	TRANSPARENT TESTA 10 (TT10)	Encodes a protein that is similar to laccase-like polyphenol oxidases. Involved in lignin and flavonoids biosynthesis. It has four conserved copper binding domains. Expressed in developing testa, where it colocalizes with the flavonoid end products proanthocyanidins and flavonols. Mutant plants exhibited a delay in developmentally determined browning of the testa, characterized by the pale brown color of seed coat. The tt10 mutant seeds accumulate more epicatechin monomers and more soluble proanthocyanidins than wild-type seeds. Flavonol composition was also affected in tt10 seeds, which exhibited a higher ratio of quercetin rhamnoside monomers versus dimers than wild-type seeds.
AT1G33870	*-1,401*		P-loop containing nucleoside triphosphate hydrolases superfamily protein; FUNCTIONS IN: GTP binding; INVOLVED IN: response to bacterium; LOCATED IN: cellular_component unknown; EXPRESSED IN: stem, root, seed; EXPRESSED DURING: F mature embryo stage; CONTAINS InterPro DOMAIN/s: AIG1 (InterPro:IPR006703); BEST Arabidopsis thaliana protein match is: P-loop containing nucleoside triphosphate hydrolases superfamily protein (TAIR:AT1G33900.1)
AT1G47603	*-1,401*	PURINE PERMEASE 19 (PUP19)	Member of a family of proteins related to PUP1, a purine transporter. May be involved in the transport of purine and purine derivatives such as cytokinins, across the plasma membrane.
AT4G18430	*-1,401*	RAB GTPASE HOMOLOG A1E (RABA1e)	RAB GTPase homolog A1E (RABA1e); FUNCTIONS IN: GTP binding; INVOLVED IN: protein transport, small GTPase mediated signal transduction; LOCATED IN: plasma membrane; EXPRESSED IN: 23 plant structures; EXPRESSED DURING: 12 growth stages; CONTAINS InterPro DOMAIN/s: Ras GTPase (InterPro:IPR001806), Small GTP-binding protein (InterPro:IPR005225), Small GTPase (InterPro:IPR020851), Ras (InterPro:IPR013753), Ras small GTPase, Rab type (InterPro:IPR003579), Rab11-related (InterPro:IPR015595); BEST Arabidopsis thaliana protein match is: RAB GTPase homolog A1F (TAIR:AT5G60860.1)
AT2G01505	*-1,402*	CLAVATA3/ESR-RELATED 16 (CLE16)	Member of a large family of putative ligands homologous to the Clavata3 gene. Consists of a single exon.
AT2G22190	*-1,402*	TREHALOSE-6-PHOSPHATE PHOSPHATASE E (TPPE)	Haloacid dehalogenase-like hydrolase (HAD) superfamily protein; FUNCTIONS IN: catalytic activity, trehalose-phosphatase activity; INVOLVED IN: trehalose biosynthetic process, metabolic process; LOCATED IN: cellular_component unknown; EXPRESSED IN: 23 plant structures; EXPRESSED DURING: 12 growth stages; CONTAINS InterPro DOMAIN/s: HAD-superfamily hydrolase, subfamily IIB (InterPro:IPR006379), Trehalose-phosphatase (InterPro:IPR003337); BEST Arabidopsis thaliana protein match is: Haloacid dehalogenase-like hydrolase (HAD) superfamily protein (TAIR:AT4G39770.1)
AT5G04470	*-1,402*	SIAMESE (SIM)	Encodes a novel nuclear 14-kD protein containing a cyclin binding motif and a motif found in ICK/KRP cell cycle inhibitor proteins. It is required for coordinating cell division and cell differentiation during the development of Arabidopsis trichomes, playing a key role in the mitosis-to-endoreduplication transition. It interacts with D-type cyclins in vivo.

AT5G44770	*-1,404*		Cysteine/Histidine-rich C1 domain family protein; FUNCTIONS IN: zinc ion binding; INVOLVED IN: intracellular signaling pathway; CONTAINS InterPro DOMAIN/s: Protein kinase C-like, phorbol ester/diacylglycerol binding (InterPro:IPR002219), Zinc finger, PHD-type, conserved site (InterPro:IPR019786), DC1 (InterPro:IPR004146), Zinc finger, PHD-type (InterPro:IPR001965), C1-like (InterPro:IPR011424); BEST Arabidopsis thaliana protein match is: Cysteine/Histidine-rich C1 domain family protein (TAIR:AT4G14980.1)
AT1G73160	*-1,405*		UDP-Glycosyltransferase superfamily protein; FUNCTIONS IN: transferase activity, transferring glycosyl groups; INVOLVED IN: biosynthetic process; EXPRESSED IN: root; CONTAINS InterPro DOMAIN/s: Glycosyl transferase, group 1 (InterPro:IPR001296); BEST Arabidopsis thaliana protein match is: UDP-Glycosyltransferase superfamily protein (TAIR:AT4G19460.1)
AT3G46130	*-1,406*	MYB DOMAIN PROTEIN 48 (MYB48)	Encodes a putative transcription factor (MYB48) that functions to regulate flavonol biosynthesis primarily in cotyledons.
AT4G01600	*-1,406*		GRAM domain family protein; CONTAINS InterPro DOMAIN/s: GRAM (InterPro:IPR004182); BEST Arabidopsis thaliana protein match is: FH interacting protein 1 (TAIR:AT1G28200.1)
AT5G40330	*-1,406*	MYB DOMAIN PROTEIN 23 (MYB23)	Encodes a MYB gene that, when overexpressed ectopically, can induce ectopic trichome formation. It is a member of subgroup 15, together with WER and GL1. Members of this subgroup share a conserved motif of 19 amino acids in the putative transcription activation domain at the C-terminal end. The gene is expressed in leaves, stems, flowers, seeds and roots and quite strongly in trichomes. There is partial functional redundancy between ATMYB23 and GL1. The two proteins are functionally equivalent with respect to the regulation of trichome initiation but not with respect to trichome branching - which is controlled by MYB23 and not GL1.
AT3G52290	*-1,407*	IQ-DOMAIN 3 (IQD3)	IQ-domain 3 (IQD3); FUNCTIONS IN: calmodulin binding; INVOLVED IN: biological_process unknown; LOCATED IN: plasma membrane; EXPRESSED IN: 23 plant structures; EXPRESSED DURING: 13 growth stages; CONTAINS InterPro DOMAIN/s: IQ calmodulin-binding region (InterPro:IPR000048); BEST Arabidopsis thaliana protein match is: IQ-domain 2 (TAIR:AT5G03040.3)
AT1G14550	*-1,408*		Peroxidase superfamily protein; FUNCTIONS IN: peroxidase activity, heme binding; INVOLVED IN: oxidation reduction, response to oxidative stress; LOCATED IN: endomembrane system; EXPRESSED IN: root; CONTAINS InterPro DOMAIN/s: Haem peroxidase (InterPro:IPR010255), Plant peroxidase (InterPro:IPR000823), Peroxidases heam-ligand binding site (InterPro:IPR019793), Haem peroxidase, plant/fungal/bacterial (InterPro:IPR002016), Peroxidase, active site (InterPro:IPR019794); BEST Arabidopsis thaliana protein match is: Peroxidase superfamily protein (TAIR:AT1G14540.1)
AT1G73290	*-1,408*	SERINE CARBOXYPEPTIDASE-LIKE 5 (scpl5)	serine carboxypeptidase-like 5 (scpl5); FUNCTIONS IN: serine-type carboxypeptidase activity; INVOLVED IN: proteolysis; LOCATED IN: endomembrane system; EXPRESSED IN: hypocotyl, root; CONTAINS InterPro DOMAIN/s: Peptidase S10, serine carboxypeptidase (InterPro:IPR001563); BEST Arabidopsis thaliana protein match is: serine carboxypeptidase-like 3 (TAIR:AT1G73280.1)
AT2G19210	*-1,408*		Leucine-rich repeat transmembrane protein kinase protein; FUNCTIONS IN: kinase activity; INVOLVED IN: protein amino acid phosphorylation; LOCATED IN: endomembrane system; CONTAINS InterPro DOMAIN/s: Protein kinase, ATP binding site (InterPro:IPR017441), Protein kinase, catalytic domain (InterPro:IPR000719), Leucine-rich repeat (InterPro:IPR001611), Serine/threonine-protein kinase-like domain (InterPro:IPR017442), Protein kinase-like domain (InterPro:IPR011009); BEST Arabidopsis thaliana protein match is: Leucine-rich repeat transmembrane protein kinase protein (TAIR:AT2G19230.1)
AT4G12050	*-1,408*	AT-HOOK MOTIF NUCLEAR LOCALIZED PROTEIN 26 (AHL26)	Predicted AT-hook DNA-binding family protein; CONTAINS InterPro DOMAIN/s: Protein of unknown function DUF296 (InterPro:IPR005175), Predicted AT-hook DNA-binding (InterPro:IPR014476); BEST Arabidopsis thaliana protein match is: Predicted AT-hook DNA-binding family protein (TAIR:AT4G22810.1)
AT4G15330	*-1,408*	CYTOCHROME P450, FAMILY 705, SUBFAMILY A, POLYPEPTIDE 1 (CYP705A1)	a member of the cytochrome P450 family
AT1G52800	*-1,409*		2-oxoglutarate (2OG) and Fe(II)-dependent oxygenase superfamily protein; FUNCTIONS IN: oxidoreductase activity; LOCATED IN: cellular_component unknown; EXPRESSED IN: stem, root; CONTAINS InterPro DOMAIN/s: Oxoglutarate/iron-dependent oxygenase (InterPro:IPR005123); BEST Arabidopsis thaliana protein match is: 2-oxoglutarate (2OG) and Fe(II)-dependent oxygenase superfamily protein (TAIR:AT1G52790.1)

AT4G22950	*-1,409*	AGAMOUS-LIKE 19 (AGL19)	MADS-box protein AGL19
AT1G26970	*-1,411*		Protein kinase superfamily protein; FUNCTIONS IN: protein serine/threonine kinase activity, protein kinase activity, kinase activity, ATP binding; INVOLVED IN: protein amino acid phosphorylation, N-terminal protein myristoylation; LOCATED IN: chloroplast; CONTAINS InterPro DOMAIN/s: Protein kinase, ATP binding site (InterPro:IPR017441), Protein kinase, catalytic domain (InterPro:IPR000719), Serine-threonine/tyrosine-protein kinase (InterPro:IPR001245), Protein kinase-like domain (InterPro:IPR011009), Serine/threonine-protein kinase, active site (InterPro:IPR008271); BEST Arabidopsis thaliana protein match is: Protein kinase superfamily protein (TAIR:AT1G69790.1)
AT1G33090	*-1,411*		MATE efflux family protein; FUNCTIONS IN: antiporter activity, drug transmembrane transporter activity, transporter activity; INVOLVED IN: drug transmembrane transport, transmembrane transport; LOCATED IN: membrane; CONTAINS InterPro DOMAIN/s: Multi antimicrobial extrusion protein MatE (InterPro:IPR002528); BEST Arabidopsis thaliana protein match is: MATE efflux family protein (TAIR:AT1G33110.1)
AT5G24860	*-1,412*	FLOWERING PROMOTING FACTOR 1 (FPF1)	encodes a small protein of 12.6 kDa that regulates flowering and is involved in gibberellin signalling pathway. It is expressed in apical meristems immediately after the photoperiodic induction of flowering. Genetic interactions with flowering time and floral organ identity genes suggest that this gene may be involved in modulating the competence to flower. There are two other genes similar to FPF1, FLP1 (At4g31380) and FLP2 (no locus name yet, on BAC F8F16 on chr 4). This is so far a plant-specific gene and is only found in long-day mustard, arabidopsis, and rice.
AT1G22440	*-1,413*		Zinc-binding alcohol dehydrogenase family protein; FUNCTIONS IN: oxidoreductase activity, zinc ion binding; INVOLVED IN: oxidation reduction; LOCATED IN: cellular_component unknown; EXPRESSED IN: hypocotyl, root; CONTAINS InterPro DOMAIN/s: GroES-like (InterPro:IPR011032), Alcohol dehydrogenase GroES-like (InterPro:IPR013154), Alcohol dehydrogenase, zinc-containing, conserved site (InterPro:IPR002328), Alcohol dehydrogenase, C-terminal (InterPro:IPR013149), Alcohol dehydrogenase superfamily, zinc-containing (InterPro:IPR002085); BEST Arabidopsis thaliana protein match is: GroES-like zinc-binding dehydrogenase family protein (TAIR:AT1G22430.2)
AT5G13150	*-1,414*	EXOCYST SUBUNIT EXO70 FAMILY PROTEIN C1 (EXO70C1)	A member of EXO70 gene family, putative exocyst subunits, conserved in land plants. Arabidopsis thaliana contains 23 putative EXO70 genes, which can be classified into eight clusters on the phylogenetic tree.
AT1G80440	*-1,415*	KISS ME DEADLY 1 (KMD1)	Encodes a member of a family of F-box proteins, called the KISS ME DEADLY (KMD) family, that targets type-B ARR proteins for degradation and is involved in the negative regulation of the cytokinin response. Also named as KFB20, a member of a group of Kelch repeat F-box proteins that negatively regulate phenylpropanoid biosynthesis by targeting the phenypropanoid biosynthesis enzyme phenylalanine ammonia-lyase. The mRNA is cell-to-cell mobile.
AT5G67390	*-1,415*		unknown protein; BEST Arabidopsis thaliana protein match is: Plant protein of unknown function (DUF863) (TAIR:AT1G69360.1)
AT5G47050	*-1,419*		SBP (S-ribonuclease binding protein) family protein; FUNCTIONS IN: zinc ion binding; EXPRESSED IN: 22 plant structures; EXPRESSED DURING: 13 growth stages; CONTAINS InterPro DOMAIN/s: Zinc finger, RING-type (InterPro:IPR001841), S-ribonuclease binding protein, SBP1, pollen (InterPro:IPR017066); BEST Arabidopsis thaliana protein match is: SBP (S-ribonuclease binding protein) family protein (TAIR:AT4G17680.1)
AT5G56220	*-1,42*		P-loop containing nucleoside triphosphate hydrolases superfamily protein; FUNCTIONS IN: nucleoside-triphosphatase activity, nucleotide binding, ATP binding; INVOLVED IN: apoptosis; LOCATED IN: chloroplast; EXPRESSED IN: 12 plant structures; EXPRESSED DURING: 7 growth stages; CONTAINS InterPro DOMAIN/s: ATPase, AAA+ type, core (InterPro:IPR003593), NB-ARC (InterPro:IPR002182); BEST Arabidopsis thaliana protein match is: Disease resistance protein (TIR-NBS class) (TAIR:AT4G23440.1)
AT5G63600	*-1,42*	FLAVONOL SYNTHASE 5 (FLS5)	encodes a protein whose sequence is similar to flavonol synthase
AT1G79910	*-1,424*	IST1-LIKE 8 (ISTL8)	Regulator of Vps4 activity in the MVB pathway protein; CONTAINS InterPro DOMAIN/s: Protein of unknown function DUF292, eukaryotic (InterPro:IPR005061); BEST Arabidopsis thaliana protein match is: Regulator of Vps4 activity in the MVB pathway protein (TAIR:AT4G32350.1)
AT2G44010	*-1,424*		unknown protein; BEST Arabidopsis thaliana protein match is: unknown protein (TAIR:AT3G59880.1)

AT1G09740	*-1,427*		Adenine nucleotide alpha hydrolases-like superfamily protein; FUNCTIONS IN: molecular_function unknown; INVOLVED IN: response to stress; LOCATED IN: cellular_component unknown; EXPRESSED IN: 13 plant structures; EXPRESSED DURING: 7 growth stages; CONTAINS InterPro DOMAIN/s: UspA (InterPro:IPR006016), Rossmann-like alpha/beta/alpha sandwich fold (InterPro:IPR014729), Universal stress protein A (InterPro:IPR006015); BEST Arabidopsis thaliana protein match is: Adenine nucleotide alpha hydrolases-like superfamily protein (TAIR:AT3G11930.1)
AT1G18940	*-1,427*		Nodulin-like / Major Facilitator Superfamily protein; INVOLVED IN: transmembrane transport; LOCATED IN: endomembrane system; EXPRESSED IN: sperm cell, male gametophyte; EXPRESSED DURING: L mature pollen stage, M germinated pollen stage; CONTAINS InterPro DOMAIN/s: Nodulin-like (InterPro:IPR010658), Major facilitator superfamily MFS-1 (InterPro:IPR011701), Major facilitator superfamily, general substrate transporter (InterPro:IPR016196); BEST Arabidopsis thaliana protein match is: Nodulin-like / Major Facilitator Superfamily protein (TAIR:AT1G74780.1)
AT3G01080	*-1,427*	WRKY DNA-BINDING PROTEIN 58 (WRKY58)	member of WRKY Transcription Factor; Group I
AT1G13590	*-1,428*	PHYTOSULFOKINE 1 PRECURSOR (PSK1)	Encodes a phytosulfokine-alpha (PSK) precursor, a unique plant peptide growth factor first described in Asparagus.
AT1G16160	*-1,429*	WALL ASSOCIATED KINASE-LIKE 5 (WAKL5)	WAK-like kinase The mRNA is cell-to-cell mobile.
AT4G01350	*-1,429*		Cysteine/Histidine-rich C1 domain family protein; FUNCTIONS IN: zinc ion binding; INVOLVED IN: response to chitin; CONTAINS InterPro DOMAIN/s: Protein kinase C-like, phorbol ester/diacylglycerol binding (InterPro:IPR002219), DC1 (InterPro:IPR004146), Zinc finger, PHD-type (InterPro:IPR001965), C1-like (InterPro:IPR011424); BEST Arabidopsis thaliana protein match is: Cysteine/Histidine-rich C1 domain family protein (TAIR:AT5G59920.1)
AT4G12090	*-1,429*		Cornichon family protein; FUNCTIONS IN: molecular_function unknown; INVOLVED IN: intracellular signaling pathway; LOCATED IN: endomembrane system, membrane; CONTAINS InterPro DOMAIN/s: Cornichon (InterPro:IPR003377); BEST Arabidopsis thaliana protein match is: Cornichon family protein (TAIR:AT1G12390.1)
AT2G14510	*-1,43*		Leucine-rich repeat protein kinase family protein; FUNCTIONS IN: kinase activity; INVOLVED IN: protein amino acid phosphorylation; LOCATED IN: endomembrane system; CONTAINS InterPro DOMAIN/s: Protein kinase, ATP binding site (InterPro:IPR017441), Protein kinase, catalytic domain (InterPro:IPR000719), Serine-threonine/tyrosine-protein kinase (InterPro:IPR001245), Protein kinase-like domain (InterPro:IPR011009), Serine/threonine-protein kinase, active site (InterPro:IPR008271); BEST Arabidopsis thaliana protein match is: Leucine-rich repeat protein kinase family protein (TAIR:AT2G14440.1)
AT3G11600	*-1,431*		unknown protein; FUNCTIONS IN: molecular_function unknown; INVOLVED IN: response to karrikin; EXPRESSED IN: 21 plant structures; EXPRESSED DURING: 12 growth stages; BEST Arabidopsis thaliana protein match is: unknown protein (TAIR:AT5G06270.1)
AT5G25020	*-1,431*		Protein of unknown function (DUF1336); CONTAINS InterPro DOMAIN/s: Protein of unknown function DUF1336 (InterPro:IPR009769); BEST Arabidopsis thaliana protein match is: Protein of unknown function (DUF1336) (TAIR:AT5G24990.1)
AT5G62420	*-1,435*		NAD(P)-linked oxidoreductase superfamily protein; FUNCTIONS IN: oxidoreductase activity; INVOLVED IN: oxidation reduction; EXPRESSED IN: root; CONTAINS InterPro DOMAIN/s: Aldo/keto reductase (InterPro:IPR001395), Aldo/keto reductase subgroup (InterPro:IPR020471), Aldo/keto reductase, conserved site (InterPro:IPR018170); BEST Arabidopsis thaliana protein match is: NAD(P)-linked oxidoreductase superfamily protein (TAIR:AT2G37770.2)
AT3G02240	*-1,436*	ROOT MERISTEM GROWTH FACTOR 7 (RGF7)	Encodes a root meristem growth factor (RGF). Belongs to a family of functionally redundant homologous peptides that are secreted, tyrosine-sulfated, and expressed mainly in the stem cell area and the innermost layer of central columella cells. RGFs are required for maintenance of the root stem cell niche and transit amplifying cell proliferation. Members of this family include: At5g60810 (RGF1), At1g13620 (RGF2), At2g04025 (RGF3), At3g30350 (RGF4), At5g51451 (RGF5), At4g16515 (RGF6), At3g02240 (RGF7), At2g03830 (RGF8) and At5g64770 (RGF9).

AT1G59725	-1,437		DNAJ heat shock family protein; FUNCTIONS IN: unfolded protein binding, heat shock protein binding; INVOLVED IN: protein folding; LOCATED IN: cellular_component unknown; EXPRESSED IN: root; CONTAINS InterPro DOMAIN/s: Molecular chaperone, heat shock protein, Hsp40, DnaJ (InterPro:IPR015609), HSP40/DnaJ peptide-binding (InterPro:IPR008971), Chaperone DnaJ, C-terminal (InterPro:IPR002939), Heat shock protein DnaJ, N-terminal (InterPro:IPR001623), Heat shock protein DnaJ (InterPro:IPR003095), Heat shock protein DnaJ, conserved site (InterPro:IPR018253); BEST Arabidopsis thaliana protein match is: DNAJ heat shock family protein (TAIR:AT1G10350.1)
AT1G65510	-1,437		unknown protein; FUNCTIONS IN: molecular_function unknown; INVOLVED IN: N-terminal protein myristoylation; LOCATED IN: endomembrane system; EXPRESSED IN: 9 plant structures; EXPRESSED DURING: LP.06 six leaves visible, LP.04 four leaves visible, 4 anthesis, petal differentiation and expansion stage, LP.08 eight leaves visible; BEST Arabidopsis thaliana protein match is: unknown protein (TAIR:AT1G65486.1)
AT4G33610	-1,437		glycine-rich protein; BEST Arabidopsis thaliana protein match is: glycine-rich protein (TAIR:AT1G55990.1)
AT1G02360	-1,439		Chitinase family protein; FUNCTIONS IN: chitinase activity; INVOLVED IN: carbohydrate metabolic process, cell wall macromolecule catabolic process; LOCATED IN: endomembrane system; EXPRESSED IN: stem, cotyledon, leaf whorl, root; CONTAINS InterPro DOMAIN/s: Glycoside hydrolase, family 19 (InterPro:IPR016283), Glycoside hydrolase, family 19, catalytic (InterPro:IPR000726); BEST Arabidopsis thaliana protein match is: Chitinase family protein (TAIR:AT4G01700.1)
AT1G79410	-1,44	ORGANIC CATION/CARNITINE TRANSPORTER5 (OCT5)	organic cation/carnitine transporter5 (5-Oct); FUNCTIONS IN: carbohydrate transmembrane transporter activity, sugar:hydrogen symporter activity; INVOLVED IN: transport, transmembrane transport; LOCATED IN: integral to membrane, membrane; EXPRESSED IN: 19 plant structures; EXPRESSED DURING: 13 growth stages; CONTAINS InterPro DOMAIN/s: Major facilitator superfamily (InterPro:IPR020846), General substrate transporter (InterPro:IPR005828), Major facilitator superfamily, general substrate transporter (InterPro:IPR016196); BEST Arabidopsis thaliana protein match is: organic cation/carnitine transporter 6 (TAIR:AT1G16370.1)
AT3G04300	-1,44		RmlC-like cupins superfamily protein; CONTAINS InterPro DOMAIN/s: Cupin, RmlC-type (InterPro:IPR011051), Protein of unknown function DUF861, cupin-3 (InterPro:IPR008579), RmlC-like jelly roll fold (InterPro:IPR014710); BEST Arabidopsis thaliana protein match is: RmlC-like cupins superfamily protein (TAIR:AT4G10300.1)
AT4G08620	-1,44	SULPHATE TRANSPORTER 1;1 (SULTR1;1)	Encodes a high-af?nity sulfate transporter. Contains STAS domain. Expressed in roots and guard cells. Up-regulated by sulfur deficiency.
AT5G06800	-1,44		myb-like HTH transcriptional regulator family protein; CONTAINS InterPro DOMAIN/s: Homeodomain-like (InterPro:IPR009057), Myb, DNA-binding (InterPro:IPR014778), HTH transcriptional regulator, Myb-type, DNA-binding (InterPro:IPR017930), Myb-like DNA-binding domain, SHAQKYF class (InterPro:IPR006447), Homeodomain-related (InterPro:IPR012287); BEST Arabidopsis thaliana protein match is: Homeodomain-like superfamily protein (TAIR:AT3G04450.1)
AT5G19230	-1,44		Glycoprotein membrane precursor GPI-anchored; FUNCTIONS IN: molecular_function unknown; INVOLVED IN: biological_process unknown; LOCATED IN: anchored to plasma membrane, plasma membrane, anchored to membrane; EXPRESSED IN: callus; BEST Arabidopsis thaliana protein match is: Glycoprotein membrane precursor GPI-anchored (TAIR:AT5G19240.1)
AT2G32610	-1,441	CELLULOSE SYNTHASE-LIKE B1 (CSLB01)	encodes a gene similar to cellulose synthase
AT2G25410	-1,442		RING/U-box superfamily protein; FUNCTIONS IN: zinc ion binding; INVOLVED IN: biological_process unknown; LOCATED IN: endomembrane system; CONTAINS InterPro DOMAIN/s: Zinc finger, RING-type (InterPro:IPR001841); BEST Arabidopsis thaliana protein match is: RING/U-box superfamily protein (TAIR:AT2G46495.1)
AT5G65210	-1,442	TGACG SEQUENCE-SPECIFIC BINDING PROTEIN 1 (TGA1)	Encodes TGA1, a redox-controlled regulator of systemic acquired resistance. TGA1 targets the activation sequence-1 (as-1) element of the promoter region of defense proteins. TGA1 are S-nitrosylated.

AT3G54220	*-1,444*	SCARECROW (SCR)	Encodes a member of a novel family having similarity to DNA binding proteins containing basic-leucine zipper regions; scr is expressed in cortex/endodermal initial cells and in the endodermal cell lineage. Regulates the radial organization of the root. Is required cell-autonomously for distal specification of the quiescent center, which in turn regulates stem cell fate of immediately surrounding cells. SCR appears to be a direct target of SHR. SCR and SCR-LIKE 23 act redundantly in bundle sheath cell fate specification.
AT4G37390	*-1,445*	(BRU6)	Encodes an IAA-amido synthase that conjugates Asp and other amino acids to auxin in vitro. Lines carrying insertions in this gene are hypersensitive to auxin. May function as a negative component in auxin signaling by regulating auxin activity.
AT5G40510	*-1,445*		Sucrase/ferredoxin-like family protein; CONTAINS InterPro DOMAIN/s: Thioredoxin fold (InterPro:IPR012335), Sucraseferredoxin-like (InterPro:IPR009737), Thioredoxin-like fold (InterPro:IPR012336); BEST Arabidopsis thaliana protein match is: Sucrase/ferredoxin-like family protein (TAIR:AT3G27570.1)
AT1G21210	*-1,446*	WALL ASSOCIATED KINASE 4 (WAK4)	cell wall-associated ser/thr kinase involved in cell elongation and lateral root development
AT1G13710	*-1,448*	CYTOCHROME P450, FAMILY 78, SUBFAMILY A, POLYPEPTIDE 5 (CYP78A5)	Encodes the cytochrome P450 CYP78A5 monooxygenase. Contributes to the generation of a growth-stimulating signal distinct from the classical phytohormones that prevents proliferation arrest, promoting organ growth.
AT4G01110	*-1,448*		unknown protein; BEST Arabidopsis thaliana protein match is: unknown protein (TAIR:AT1G01453.1)
AT4G03460	*-1,449*		Ankyrin repeat family protein; CONTAINS InterPro DOMAIN/s: Ankyrin repeat-containing domain (InterPro:IPR020683), Ankyrin repeat (InterPro:IPR002110); BEST Arabidopsis thaliana protein match is: Ankyrin repeat family protein (TAIR:AT4G03500.1)
AT4G25420	*-1,449*	(GA20OX1)	Encodes gibberellin 20-oxidase that is involved in the later steps of the gibberellin biosynthetic pathway. Regulated by a circadian clock. Weak expression response to far red light.
AT1G71692	*-1,45*	AGAMOUS-LIKE 12 (AGL12)	Encodes a member of the MADS box family of transcription factors. Involved in root cell differentiation and flowering time. Loss of function mutations have abnormal cellular differentiation in the roots and are late flowering. AGL12 along with AGL14, and AGL17 is preferentially expressed in root tissues and represent the only characterized MADS box genes expressed in roots.
AT4G10530	*-1,45*		Subtilase family protein; FUNCTIONS IN: identical protein binding, serine-type endopeptidase activity; INVOLVED IN: proteolysis, negative regulation of catalytic activity; LOCATED IN: endomembrane system; CONTAINS InterPro DOMAIN/s: Protease-associated PA (InterPro:IPR003137), Proteinase inhibitor, propeptide (InterPro:IPR009020), Peptidase S8/S53, subtilisin/kexin/sedolisin (InterPro:IPR000209), Peptidase S8, subtilisin-related (InterPro:IPR015500), Proteinase inhibitor I9, subtilisin propeptide (InterPro:IPR010259), Peptidase S8/S53, subtilisin, active site (InterPro:IPR022398); BEST Arabidopsis thaliana protein match is: Subtilase family protein (TAIR:AT4G10520.1)
AT4G15380	*-1,45*	CYTOCHROME P450, FAMILY 705, SUBFAMILY A, POLYPEPTIDE 4 (CYP705A4)	member of CYP705A
AT1G17860	*-1,451*		Kunitz family trypsin and protease inhibitor protein; FUNCTIONS IN: endopeptidase inhibitor activity; INVOLVED IN: biological_process unknown; LOCATED IN: apoplast, cell wall; EXPRESSED IN: 17 plant structures; EXPRESSED DURING: 13 growth stages; CONTAINS InterPro DOMAIN/s: Proteinase inhibitor I3, Kunitz legume (InterPro:IPR002160), Kunitz inhibitor ST1-like (InterPro:IPR011065); BEST Arabidopsis thaliana protein match is: kunitz trypsin inhibitor 1 (TAIR:AT1G73260.1)
AT5G57920	*-1,452*	EARLY NODULIN-LIKE PROTEIN 10 (ENODL10)	early nodulin-like protein 10 (ENODL10); FUNCTIONS IN: electron carrier activity, copper ion binding; LOCATED IN: endomembrane system; EXPRESSED IN: 14 plant structures; EXPRESSED DURING: 10 growth stages; CONTAINS InterPro DOMAIN/s: Plastocyanin-like (InterPro:IPR003245), Cupredoxin (InterPro:IPR008972); BEST Arabidopsis thaliana protein match is: early nodulin-like protein 15 (TAIR:AT4G31840.1)
AT2G26420	*-1,453*	1-PHOSPHATIDYLINOSITOL-4-PHOSPHATE 5-KINASE 3 (PIP5K3)	Encodes a phosphatidylinositol-4-phosphate 5-kinase. Exclusively expressed in roots. Essential for root hair growth.

AT1G04150	*-1,455*		C2 calcium/lipid-binding plant phosphoribosyltransferase family protein; CONTAINS InterPro DOMAIN/s: C2 membrane targeting protein (InterPro:IPR018029), C2 calcium/lipid-binding domain, CaLB (InterPro:IPR008973), Phosphoribosyltransferase C-terminal (InterPro:IPR013583), C2 calcium-dependent membrane targeting (InterPro:IPR000008); BEST Arabidopsis thaliana protein match is: C2 calcium/lipid-binding plant phosphoribosyltransferase family protein (TAIR:AT4G11610.1)
AT2G43610	*-1,455*		Chitinase family protein; FUNCTIONS IN: chitin binding, chitinase activity; INVOLVED IN: carbohydrate metabolic process, cell wall macromolecule catabolic process; LOCATED IN: plasma membrane; EXPRESSED IN: 15 plant structures; EXPRESSED DURING: 7 growth stages; CONTAINS InterPro DOMAIN/s: Chitin-binding, type 1, conserved site (InterPro:IPR018371), Glycoside hydrolase, family 19 (InterPro:IPR016283), Chitin-binding, type 1 (InterPro:IPR001002), Glycoside hydrolase, family 19, catalytic (InterPro:IPR000726); BEST Arabidopsis thaliana protein match is: Chitinase family protein (TAIR:AT2G43620.1)
AT1G53700	*-1,456*	WAG 1 (WAG1)	The WAG1 and its homolog, WAG2 each encodes a protein-serine/threonine kinase that are nearly 70% identical to PsPK3 protein. All three together with CsPK3 belong to PsPK3-type kinases. At the N-terminus, all four possess a serine/threonine-rich domain. They are closely related to Arabidopsis kinases PINOID. wag1/wag2 double mutants exhibit a pronounced wavy root phenotype when grown vertically on agar plates (while wild-type plants develop wavy roots only on plates inclined to angles less than 90 degrees), indicating an overlapping role for WAG1 and WAG2 as suppressors of root waving. Simultaneous disruption of PID(AT2G34650) and its 3 closest homologs (PID2/AT2G26700, WAG1/AT1G53700, and WAG2/AT3G14370) abolishes the formation of cotyledons.
AT4G19920	*-1,457*		Toll-Interleukin-Resistance (TIR) domain family protein; FUNCTIONS IN: transmembrane receptor activity; INVOLVED IN: signal transduction, defense response, innate immune response; LOCATED IN: intrinsic to membrane, chloroplast; EXPRESSED IN: cotyledon; CONTAINS InterPro DOMAIN/s: Toll-Interleukin receptor (InterPro:IPR000157); BEST Arabidopsis thaliana protein match is: Toll-Interleukin-Resistance (TIR) domain family protein (TAIR:AT4G19925.1)
AT1G64590	*-1,458*		NAD(P)-binding Rossmann-fold superfamily protein; FUNCTIONS IN: oxidoreductase activity, binding, catalytic activity; INVOLVED IN: oxidation reduction, metabolic process; EXPRESSED IN: 20 plant structures; EXPRESSED DURING: 12 growth stages; CONTAINS InterPro DOMAIN/s: NAD(P)-binding domain (InterPro:IPR016040), Glucose/ribitol dehydrogenase (InterPro:IPR002347), Short-chain dehydrogenase/reductase SDR (InterPro:IPR002198); BEST Arabidopsis thaliana protein match is: NAD(P)-binding Rossmann-fold superfamily protein (TAIR:AT4G24050.1)
AT4G01140	*-1,458*		Protein of unknown function (DUF1191); FUNCTIONS IN: molecular_function unknown; INVOLVED IN: biological_process unknown; LOCATED IN: endomembrane system; EXPRESSED IN: inflorescence meristem, leaf whorl, root, flower; EXPRESSED DURING: 4 anthesis; CONTAINS InterPro DOMAIN/s: Protein of unknown function DUF1191 (InterPro:IPR010605); BEST Arabidopsis thaliana protein match is: Protein of unknown function (DUF1191) (TAIR:AT4G23720.1)
AT1G33840	*-1,459*		Protein of unknown function (DUF567); CONTAINS InterPro DOMAIN/s: Protein of unknown function DUF567 (InterPro:IPR007612); BEST Arabidopsis thaliana protein match is: Protein of unknown function (DUF567) (TAIR:AT5G01750.2)
AT5G13930	*-1,46*	TRANSPARENT TESTA 4 (TT4)	Encodes chalcone synthase (CHS), a key enzyme involved in the biosynthesis of flavonoids. Required for the accumulation of purple anthocyanins in leaves and stems. Also involved in the regulation of auxin transport and the modulation of root gravitropism. The mRNA is cell-to-cell mobile.
AT4G19980	*-1,461*		unknown protein; FUNCTIONS IN: molecular_function unknown; INVOLVED IN: biological_process unknown; LOCATED IN: chloroplast; EXPRESSED IN: root, pedicel; EXPRESSED DURING: 4 anthesis
AT5G28640	*-1,461*	ANGUSTIFOLIA 3 (AN3)	Encodes a protein with similarity to mammalian transcriptional coactivator that is involved in cell proliferation during leaf and flower development. Loss of function mutations have narrow, pointed leaves and narrow floral organs. AN3 interacts with members of the growth regulating factor (GRF) family of transcription factors.
AT5G56100	*-1,461*		glycine-rich protein / oleosin; FUNCTIONS IN: molecular_function unknown; INVOLVED IN: lipid storage; LOCATED IN: monolayer-surrounded lipid storage body, integral to membrane, membrane; EXPRESSED IN: 21 plant structures; EXPRESSED DURING: 12 growth stages; CONTAINS InterPro DOMAIN/s: Oleosin (InterPro:IPR000136)
AT4G37030	*-1,462*		unknown protein; FUNCTIONS IN: molecular_function unknown; INVOLVED IN: biological_process unknown; LOCATED IN: endomembrane system; BEST Arabidopsis thaliana protein match is: unknown protein (TAIR:AT4G12680.1)

AT5G24910	*-1,462*	CYTOCHROME P450, FAMILY 714, SUBFAMILY A, POLYPEPTIDE 1 (CYP714A1)	Member of CYP714A. Encodes one of the two tandemly duplicated gene pair ELA1 (CYP714A1) and ELA2 (CYP714A2), homologs of the rice cytochrome P450 monooxygenase gene EUI1. Double mutation of ELA1 and ELA2 results in increased biomass and enlarged organs.
AT5G65340	*-1,462*		Protein of unknown function, DUF617; CONTAINS InterPro DOMAIN/s: Protein of unknown function DUF617, plant (InterPro:IPR006460); BEST Arabidopsis thaliana protein match is: Protein of unknown function, DUF617 (TAIR:AT2G22460.1)
AT2G01890	*-1,465*	PURPLE ACID PHOSPHATASE 8 (PAP8)	Encodes a purple acid phosphatase (PAP) belonging to the low molecular weight plant PAP group.
AT3G05980	*-1,465*		unknown protein; BEST Arabidopsis thaliana protein match is: unknown protein (TAIR:AT5G19340.1)
AT1G14390	*-1,466*		Leucine-rich repeat protein kinase family protein; FUNCTIONS IN: protein serine/threonine kinase activity, protein kinase activity, ATP binding; INVOLVED IN: transmembrane receptor protein tyrosine kinase signaling pathway, protein amino acid phosphorylation; LOCATED IN: endomembrane system; EXPRESSED IN: inflorescence meristem, root, flower; EXPRESSED DURING: petal differentiation and expansion stage; CONTAINS InterPro DOMAIN/s: Protein kinase, catalytic domain (InterPro:IPR000719), Serine/threonine-protein kinase domain (InterPro:IPR002290), Leucine-rich repeat (InterPro:IPR001611), Tyrosine-protein kinase, catalytic domain (InterPro:IPR020635), Serine-threonine/tyrosine-protein kinase (InterPro:IPR001245), Protein kinase-like domain (InterPro:IPR011009); BEST Arabidopsis thaliana protein match is: Leucine-rich repeat protein kinase family protein (TAIR:AT2G02780.1)
AT3G10985	*-1,466*	SENESCENCE ASSOCIATED GENE 20 (SAG20)	A senescence-associated gene whose expression is induced in response to treatment with Nep1, a fungal protein that causes necrosis. The mRNA is cell-to-cell mobile.
AT1G51790	*-1,467*		Leucine-rich repeat protein kinase family protein; FUNCTIONS IN: kinase activity; INVOLVED IN: protein amino acid phosphorylation; LOCATED IN: endomembrane system; EXPRESSED IN: 9 plant structures; EXPRESSED DURING: 6 growth stages; CONTAINS InterPro DOMAIN/s: Protein kinase, catalytic domain (InterPro:IPR000719), Serine/threonine-protein kinase domain (InterPro:IPR002290), Leucine-rich repeat (InterPro:IPR001611), Serine-threonine/tyrosine-protein kinase (InterPro:IPR001245), Tyrosine-protein kinase, catalytic domain (InterPro:IPR020635), Protein kinase-like domain (InterPro:IPR011009); BEST Arabidopsis thaliana protein match is: Leucine-rich repeat protein kinase family protein (TAIR:AT1G51800.1)
AT4G20820	*-1,467*	(ATBBE18)	FAD-binding Berberine family protein; FUNCTIONS IN: electron carrier activity, oxidoreductase activity, FAD binding, catalytic activity; INVOLVED IN: oxidation reduction; EXPRESSED IN: 6 plant structures; EXPRESSED DURING: LP.06 six leaves visible, LP.04 four leaves visible, LP.08 eight leaves visible; CONTAINS InterPro DOMAIN/s: FAD-binding, type 2 (InterPro:IPR016166), Oxygen oxidoreductase covalent FAD-binding site (InterPro:IPR006093), Berberine/berberine-like (InterPro:IPR012951), FAD linked oxidase, N-terminal (InterPro:IPR006094); BEST Arabidopsis thaliana protein match is: FAD-binding Berberine family protein (TAIR:AT5G44440.1)
AT4G27740	*-1,467*		Yippee family putative zinc-binding protein; CONTAINS InterPro DOMAIN/s: Yippee-like protein (InterPro:IPR004910); BEST Arabidopsis thaliana protein match is: Yippee family putative zinc-binding protein (TAIR:AT4G27745.1)
AT4G14550	*-1,468*	INDOLE-3-ACETIC ACID INDUCIBLE 14 (IAA14)	IAA14 is a member of the Aux/IAA protein family. Involved in lateral root development. Gain of function mutation decreases auxin-inducible gene expression. Protein is localized to the nucleus. Expressed in stele and root tip epidermis. Functions as a negative regulator of ARF7/19.
AT1G02810	*-1,469*		Plant invertase/pectin methylesterase inhibitor superfamily; FUNCTIONS IN: enzyme inhibitor activity, pectinesterase activity; INVOLVED IN: cell wall modification; LOCATED IN: endomembrane system, cell wall, plant-type cell wall; EXPRESSED IN: 14 plant structures; EXPRESSED DURING: 6 growth stages; CONTAINS InterPro DOMAIN/s: Pectinesterase, active site (InterPro:IPR018040), Pectin lyase fold/virulence factor (InterPro:IPR011050), Pectinesterase, catalytic (InterPro:IPR000070), Pectinesterase inhibitor (InterPro:IPR006501), Pectin lyase fold (InterPro:IPR012334); BEST Arabidopsis thaliana protein match is: Plant invertase/pectin methylesterase inhibitor superfamily (TAIR:AT4G02330.1)

AT2G02780	*-1,47*		Leucine-rich repeat protein kinase family protein; FUNCTIONS IN: protein serine/threonine kinase activity, protein kinase activity, ATP binding; INVOLVED IN: transmembrane receptor protein tyrosine kinase signaling pathway, protein amino acid phosphorylation; LOCATED IN: endomembrane system; EXPRESSED IN: 22 plant structures; EXPRESSED DURING: 13 growth stages; CONTAINS InterPro DOMAIN/s: Protein kinase, catalytic domain (InterPro:IPR000719), Leucine-rich repeat (InterPro:IPR001611), Serine-threonine/tyrosine-protein kinase (InterPro:IPR001245), Protein kinase-like domain (InterPro:IPR011009); BEST Arabidopsis thaliana protein match is: Leucine-rich repeat protein kinase family protein (TAIR:AT1G14390.1)
AT4G28410	*-1,47*	ROOT SYSTEM ARCHITECTURE 1 (RSA1)	Tyrosine transaminase family protein; FUNCTIONS IN: 1-aminocyclopropane-1-carboxylate synthase activity, pyridoxal phosphate binding, transferase activity, transferring nitrogenous groups, transaminase activity, catalytic activity; INVOLVED IN: cellular amino acid and derivative metabolic process, biosynthetic process; LOCATED IN: cellular_component unknown; EXPRESSED IN: root; CONTAINS InterPro DOMAIN/s: 1-aminocyclopropane-1-carboxylate synthase (InterPro:IPR001176), Aminotransferase, class I/classII (InterPro:IPR004839), Pyridoxal phosphate-dependent transferase, major domain (InterPro:IPR015424), Tyrosine transaminase (InterPro:IPR021178), Tyrosine/nicotianamine aminotransferase (InterPro:IPR005958), Aminotransferases, class-I, pyridoxal-phosphate-binding site (InterPro:IPR004838), Pyridoxal phosphate-dependent transferase, major region, subdomain 1 (InterPro:IPR015421), Pyridoxal phosphate-dependent transferase, major region, subdomain 2 (InterPro:IPR015422); BEST Arabidopsis thaliana protein match is: Tyrosine transaminase family protein (TAIR:AT2G20610.1)
AT5G24960	*-1,47*	CYTOCHROME P450, FAMILY 71, SUBFAMILY A, POLYPEPTIDE 14 (CYP71A14)	putative cytochrome P450
AT2G34940	*-1,471*	VACUOLAR SORTING RECEPTOR 5 (VSR5)	VACUOLAR SORTING RECEPTOR 5 (VSR5); FUNCTIONS IN: calcium ion binding; INVOLVED IN: protein targeting to vacuole; LOCATED IN: integral to plasma membrane, Golgi transport complex; EXPRESSED IN: 14 plant structures; EXPRESSED DURING: 11 growth stages; CONTAINS InterPro DOMAIN/s: Protease-associated PA (InterPro:IPR003137), EGF-like calcium-binding, conserved site (InterPro:IPR018097), EGF-like calcium-binding (InterPro:IPR001881), Growth factor, receptor (InterPro:IPR009030); BEST Arabidopsis thaliana protein match is: VACUOLAR SORTING RECEPTOR 6 (TAIR:AT1G30900.1)
AT4G08160	*-1,471*		Encodes a putative glycosyl hydrolase family 10 protein (xylanase).
AT5G50120	*-1,472*		Transducin/WD40 repeat-like superfamily protein; CONTAINS InterPro DOMAIN/s: WD40 repeat 2 (InterPro:IPR019782), WD40 repeat, conserved site (InterPro:IPR019775), WD40 repeat (InterPro:IPR001680), G-protein beta WD-40 repeat, region (InterPro:IPR020472), WD40 repeat-like-containing domain (InterPro:IPR011046), WD40-repeat-containing domain (InterPro:IPR017986), WD40/YVTN repeat-like-containing domain (InterPro:IPR015943), WD40 repeat, subgroup (InterPro:IPR019781); BEST Arabidopsis thaliana protein match is: Transducin/WD40 repeat-like superfamily protein (TAIR:AT1G24130.1)
AT1G30720	*-1,473*	(ATBBE10)	FAD-binding Berberine family protein; FUNCTIONS IN: electron carrier activity, oxidoreductase activity, FAD binding, catalytic activity; LOCATED IN: endomembrane system; CONTAINS InterPro DOMAIN/s: FAD-binding, type 2 (InterPro:IPR016166), Berberine/berberine-like (InterPro:IPR012951), FAD linked oxidase, N-terminal (InterPro:IPR006094); BEST Arabidopsis thaliana protein match is: FAD-binding Berberine family protein (TAIR:AT1G30730.1)
AT2G46850	*-1,473*		Protein kinase superfamily protein; FUNCTIONS IN: protein kinase activity, ATP binding; INVOLVED IN: protein amino acid phosphorylation; LOCATED IN: endomembrane system; CONTAINS InterPro DOMAIN/s: Protein kinase, catalytic domain (InterPro:IPR000719), Serine-threonine/tyrosine-protein kinase (InterPro:IPR001245), Protein kinase-like domain (InterPro:IPR011009); BEST Arabidopsis thaliana protein match is: Protein kinase superfamily protein (TAIR:AT2G23450.1)
AT2G14100	*-1,474*	CYTOCHROME P450, FAMILY 705, SUBFAMILY A, POLYPEPTIDE 13 (CYP705A13)	a member of the cytochrome P450 family

AT2G25160	*-1,474*	CYTOCHROME P450, FAMILY 82, SUBFAMILY F, POLYPEPTIDE 1 (CYP82F1)	member of CYP82F
AT5G19890	*-1,478*		Peroxidase superfamily protein; FUNCTIONS IN: peroxidase activity, heme binding; INVOLVED IN: oxidation reduction, response to oxidative stress; LOCATED IN: nucleus, cytoplasm; EXPRESSED IN: 17 plant structures; EXPRESSED DURING: 10 growth stages; CONTAINS InterPro DOMAIN/s: Haem peroxidase (InterPro:IPR010255), Plant peroxidase (InterPro:IPR000823), Peroxidases heam-ligand binding site (InterPro:IPR019793), Haem peroxidase, plant/fungal/bacterial (InterPro:IPR002016), Peroxidase, active site (InterPro:IPR019794); BEST Arabidopsis thaliana protein match is: Peroxidase superfamily protein (TAIR:AT5G06730.1)
AT5G38550	*-1,478*		Mannose-binding lectin superfamily protein; CONTAINS InterPro DOMAIN/s: Mannose-binding lectin (InterPro:IPR001229); BEST Arabidopsis thaliana protein match is: Mannose-binding lectin superfamily protein (TAIR:AT5G38540.1)
AT3G26520	*-1,479*	TONOPLAST INTRINSIC PROTEIN 2 (TIP2)	gamma tonoplast intrinsic protein 2 (TIP2). expressed throughout the plant and transcript level is increased upon NaCl or ABA treatments. NaCl stress-sensitive yeast mutant strains exhibit more resistance to salt when expressing this protein.
AT4G00730	*-1,48*	ANTHOCYANINLESS 2 (ANL2)	Encodes a homeodomain protein of the HD-GLABRA2 group. Involved in the accumulation of anthocyanin and in root development. Loss of function mutants have increased cell wall polysaccharide content.
AT5G38910	*-1,481*		RmlC-like cupins superfamily protein; FUNCTIONS IN: manganese ion binding, nutrient reservoir activity; INVOLVED IN: biological_process unknown; LOCATED IN: endomembrane system, apoplast; CONTAINS InterPro DOMAIN/s: Cupin, RmlC-type (InterPro:IPR011051), Cupin 1 (InterPro:IPR006045), RmlC-like jelly roll fold (InterPro:IPR014710), Germin (InterPro:IPR001929), Germin, manganese binding site (InterPro:IPR019780); BEST Arabidopsis thaliana protein match is: RmlC-like cupins superfamily protein (TAIR:AT5G38940.1)
AT1G64370	*-1,483*		unknown protein
AT1G14580	*-1,485*	BLUEJAY (BLJ)	C2H2-like zinc finger protein; FUNCTIONS IN: sequence-specific DNA binding transcription factor activity, zinc ion binding, nucleic acid binding; LOCATED IN: intracellular; EXPRESSED IN: 7 plant structures; EXPRESSED DURING: LP.04 four leaves visible, LP.10 ten leaves visible, LP.02 two leaves visible, 4 leaf senescence stage; CONTAINS InterPro DOMAIN/s: Zinc finger, C2H2-like (InterPro:IPR015880), Zinc finger, C2H2-type (InterPro:IPR007087); BEST Arabidopsis thaliana protein match is: indeterminate(ID)-domain 4 (TAIR:AT2G02080.1)
AT1G30730	*-1,485*	(ATBBE11)	FAD-binding Berberine family protein; FUNCTIONS IN: electron carrier activity, oxidoreductase activity, FAD binding, catalytic activity; LOCATED IN: endomembrane system; CONTAINS InterPro DOMAIN/s: FAD-binding, type 2 (InterPro:IPR016166), Berberine/berberine-like (InterPro:IPR012951), FAD linked oxidase, N-terminal (InterPro:IPR006094); BEST Arabidopsis thaliana protein match is: FAD-binding Berberine family protein (TAIR:AT1G30720.1)
AT1G11460	*-1,486*	USUALLY MULTIPLE ACIDS MOVE IN AND OUT TRANSPORTERS 26 (UMAMIT26)	nodulin MtN21-like transporter family protein
AT3G21510	*-1,486*	HISTIDINE-CONTAINING PHOSPHOTRANSMITTER 1 (AHP1)	Encodes AHP1, one of the six Arabidopsis thaliana histidine phosphotransfer proteins (AHPs). AHPs function as redundant positive regulators of cytokinin signaling. Members of the AHP gene family include: AT3G21510 (AHP1), AT3G29350 (AHP2), AT5G39340 (AHP3), AT3G16360 (AHP4), AT1G03430 (AHP5) and AT1G80100 (AHP6).
AT5G22940	*-1,486*	FRA8 HOMOLOG (F8H)	Homolog of FRA8 (AT2G28110), a member of a member of glycosyltransferase family 47; exhibits high sequence similarity to tobacco (Nicotiana plumbaginifolia) pectin glucuronyltransferase.
AT5G45380	*-1,488*	DEGRADATION OF UREA 3 (DUR3)	DEGRADATION OF UREA 3 (DUR3); CONTAINS InterPro DOMAIN/s: Sodium/solute symporter, subgroup (InterPro:IPR019900), Sodium/solute symporter (InterPro:IPR001734)
AT3G01220	*-1,489*	HOMEOBOX PROTEIN 20 (HB20)	Encodes a homeodomain leucine zipper class I (HD-Zip I) protein. Expressed during seed germination in the micropylar endosperm and in the root cap, and increases ABA sensitivity and seed dormancy when mutated. The mRNA is cell-to-cell mobile.

AT4G01440	*-1,489*	USUALLY MULTIPLE ACIDS MOVE IN AND OUT TRANSPORTERS 31 (UMAMIT31)	nodulin MtN21-like transporter family protein
AT2G14920	*-1,49*	SULFOTRANSFERASE 4A (ST4A)	Encodes a brassinosteroid sulfotransferase that may be involved in brassinosteroid inactivation. In vitro experiements show that this enzyme can act on a broad group of naturally occurring brassinosteroids, including the 24-epimers and (22R,23R)-28 homobrassinosteroids, that have an array of different side chains, though it shows a preference for (22R,23R)-28 homobrassinosteroids. ST4A is expressed in the roots and transcript levels fall in response to cytokinin treatment.
AT5G19040	*-1,49*	ISOPENTENYLTRANSFERASE 5 (IPT5)	Encodes cytokinin synthase.
AT1G07560	*-1,492*		Leucine-rich repeat protein kinase family protein; FUNCTIONS IN: kinase activity; INVOLVED IN: protein amino acid phosphorylation; LOCATED IN: endomembrane system; EXPRESSED IN: root; CONTAINS InterPro DOMAIN/s: Protein kinase, ATP binding site (InterPro:IPR017441), Protein kinase, catalytic domain (InterPro:IPR000719), Serine/threonine-protein kinase-like domain (InterPro:IPR017442), Protein kinase-like domain (InterPro:IPR011009), Serine/threonine-protein kinase, active site (InterPro:IPR008271); BEST Arabidopsis thaliana protein match is: Leucine-rich repeat protein kinase family protein (TAIR:AT1G51820.1)
AT1G65850	*-1,497*		Disease resistance protein (TIR-NBS-LRR class) family; FUNCTIONS IN: transmembrane receptor activity, ATP binding; INVOLVED IN: signal transduction, apoptosis, defense response, innate immune response; LOCATED IN: intrinsic to membrane; EXPRESSED IN: 8 plant structures; EXPRESSED DURING: LP.04 four leaves visible, 4 anthesis, LP.02 two leaves visible, petal differentiation and expansion stage, D bilateral stage; CONTAINS InterPro DOMAIN/s: NB-ARC (InterPro:IPR002182), Toll-Interleukin receptor (InterPro:IPR000157), Disease resistance protein (InterPro:IPR000767); BEST Arabidopsis thaliana protein match is: Disease resistance protein (TIR-NBS-LRR class) family (TAIR:AT5G38340.1).
AT1G70690	*-1,497*	HOPW1-1-INDUCED GENE1 (HWI1)	Encodes a plasmodesmal protein that may be involved in the intercellular movement of molecules through the plasmodesmata. The protein has two DUF26 domains and a single transmembrane domain.
AT5G26230	*-1,497*	MEMBRANE-ASSOCIATED KINASE REGULATOR 1 (MAKR1)	Encodes a member of the MAKR (MEMBRANE-ASSOCIATED KINASE REGULATOR) gene family. MAKRs have putative kinase interacting motifs and membrane localization signals. Known members include: AT5G26230 (MAKR1), AT1G64080 (MAKR2), AT2G37380 (MAKR3), AT2G39370 (MAKR4), AT5G52870 (MAKR5) and AT5G52900 (MAKR6).
AT3G51570	*-1,498*		Disease resistance protein (TIR-NBS-LRR class) family; FUNCTIONS IN: transmembrane receptor activity, ATP binding; INVOLVED IN: signal transduction, defense response, apoptosis, innate immune response; LOCATED IN: membrane; EXPRESSED IN: leaf; CONTAINS InterPro DOMAIN/s: NB-ARC (InterPro:IPR002182), Toll-Interleukin receptor (InterPro:IPR000157), Disease resistance protein (InterPro:IPR000767); BEST Arabidopsis thaliana protein match is: Disease resistance protein (TIR-NBS-LRR class) family (TAIR:AT5G45250.1)
AT4G39700	*-1,498*		Heavy metal transport/detoxification superfamily protein ; FUNCTIONS IN: metal ion binding; INVOLVED IN: metal ion transport; LOCATED IN: cellular_component unknown; CONTAINS InterPro DOMAIN/s: Heavy metal transport/detoxification protein (InterPro:IPR006121); BEST Arabidopsis thaliana protein match is: Heavy metal transport/detoxification superfamily protein (TAIR:AT1G22990.1)
AT5G50300	*-1,498*	AZA-GUANINE RESISTANT2 (AZG2)	Encodes a homolog of the adenine-guanine-hypoxanthine transporter AzgA of Aspergillus nidulans. Function as a plant adenine-guanine transporter. Two closely related genes exist in Arabidopsis: AT3G10960 (Azg1) and AT5G50300 (Azg2).
AT1G65610	*-1,502*	KORRIGAN 2 (KOR2)	KORRIGAN 2 (KOR2); FUNCTIONS IN: hydrolase activity, hydrolyzing O-glycosyl compounds, catalytic activity; INVOLVED IN: carbohydrate metabolic process; LOCATED IN: cellular_component unknown; EXPRESSED IN: stem, sepal, root, leaf, stamen; EXPRESSED DURING: LP.06 six leaves visible, 4 anthesis, petal differentiation and expansion stage, LP.08 eight leaves visible; CONTAINS InterPro DOMAIN/s: Six-hairpin glycosidase (InterPro:IPR012341), Glycoside hydrolase, family 9, active site (InterPro:IPR018221), Six-hairpin glycosidase-like (InterPro:IPR008928), Glycoside hydrolase, family 9 (InterPro:IPR001701); BEST Arabidopsis thaliana protein match is: glycosyl hydrolase 9A1 (TAIR:AT5G49720.1)

AT2G34020	-1,502		Calcium-binding EF-hand family protein; FUNCTIONS IN: calcium ion binding; INVOLVED IN: biological_process unknown; EXPRESSED IN: root; CONTAINS InterPro DOMAIN/s: EF-Hand 1, calcium-binding site (InterPro:IPR018247), EF-HAND 2 (InterPro:IPR018249), EF-hand-like domain (InterPro:IPR011992), Calcium-binding EF-hand (InterPro:IPR002048); BEST Arabidopsis thaliana protein match is: Calcium-binding EF-hand family protein (TAIR:AT2G34030.1)
AT2G46430	-1,502	CYCLIC NUCLEOTIDE GATED CHANNEL 3 (CNGC3)	Encodes a cyclic nucleotide gated channel, downstream component of the signaling pathways leading to hypersensitive response (HR) resistance.
AT2G46150	-1,503		Late embryogenesis abundant (LEA) hydroxyproline-rich glycoprotein family; FUNCTIONS IN: molecular_function unknown; INVOLVED IN: biological_process unknown; LOCATED IN: plasma membrane; EXPRESSED IN: 15 plant structures; EXPRESSED DURING: 7 growth stages; CONTAINS InterPro DOMAIN/s: Late embryogenesis abundant protein, group 2 (InterPro:IPR004864); BEST Arabidopsis thaliana protein match is: Late embryogenesis abundant (LEA) hydroxyproline-rich glycoprotein family (TAIR:AT1G64065.1)
AT2G25150	-1,504		HXXXD-type acyl-transferase family protein; FUNCTIONS IN: transferase activity, transferring acyl groups other than amino-acyl groups, transferase activity; INVOLVED IN: biological_process unknown; LOCATED IN: cellular_component unknown; EXPRESSED IN: 9 plant structures; EXPRESSED DURING: petal differentiation and expansion stage, E expanded cotyledon stage, D bilateral stage; CONTAINS InterPro DOMAIN/s: Transferase (InterPro:IPR003480); BEST Arabidopsis thaliana protein match is: spermidine disinapoyl acyltransferase (TAIR:AT2G23510.1)
AT5G26290	-1,505		TRAF-like family protein; CONTAINS InterPro DOMAIN/s: TRAF-like (InterPro:IPR008974), MATH (InterPro:IPR002083); BEST Arabidopsis thaliana protein match is: TRAF-like family protein (TAIR:AT5G26280.1)
AT2G01900	-1,507	INOSITOL POLYPHOSPHATE PHOSPHATIDYLINOSITOL 5-PHOSPHATASE9 (T5PTASE9)	Encodes an inositol polyphosphate phosphatidylinositol 5-phosphatase that is expressed in roots and is involved in mediating salt tolerance through endocytosis.
AT1G23000	-1,508		Heavy metal transport/detoxification superfamily protein ; FUNCTIONS IN: metal ion binding; INVOLVED IN: metal ion transport; LOCATED IN: cellular_component unknown; EXPRESSED IN: 12 plant structures; EXPRESSED DURING: 6 growth stages; CONTAINS InterPro DOMAIN/s: Heavy metal transport/detoxification protein (InterPro:IPR006121); BEST Arabidopsis thaliana protein match is: Heavy metal transport/detoxification superfamily protein (TAIR:AT3G06130.1)
AT5G24880	-1,508		BEST Arabidopsis thaliana protein match is: calmodulin-binding protein-related (TAIR:AT5G10660.1)
AT1G15550	-1,51	GIBBERELLIN 3-OXIDASE 1 (GA3OX1)	Involved in later steps of the gibberellic acid biosynthetic pathway. Activated by AGAMOUS in a cal-1, ap1-1 background. Deletion of 208 bp from -1016 to -809 (Δ-808) resulted in loss of GA-negative feedback (this sequence, which contains a 43-bp sequence GNFEI, was shown to be sufficient for GA-negative feedback).
AT3G54420	-1,51	HOMOLOG OF CARROT EP3-3 CHITINASE (EP3)	encodes an EP3 chitinase that is expressed during somatic embryogenesis in 'nursing' cells surrounding the embryos but not in embryos themselves. The gene is also expressed in mature pollen and growing pollen tubes until they enter the receptive synergid, but not in endosperm and integuments as in carrot. Post-embryonically, expression is found in hydathodes, stipules, root epidermis and emerging root hairs.
AT3G10912	-1,511	CONSERVED PEPTIDE UPSTREAM OPEN READING FRAME 63 (CPuORF63)	Upstream open reading frames (uORFs) are small open reading frames found in the 5' UTR of a mature mRNA, and can potentially mediate translational regulation of the largest, or major, ORF (mORF). CPuORF63 represents a conserved upstream opening reading frame relative to major ORF AT3G10910.1
AT4G18510	-1,511	CLAVATA3/ESR-RELATED 2 (CLE2)	CLE2, putative ligand, member of large gene family homologous to Clavata3

AT5G49770	*-1,511*		Leucine-rich repeat protein kinase family protein; FUNCTIONS IN: protein serine/threonine kinase activity, protein kinase activity, ATP binding; INVOLVED IN: transmembrane receptor protein tyrosine kinase signaling pathway, protein amino acid phosphorylation; LOCATED IN: endomembrane system; EXPRESSED IN: sperm cell, root; CONTAINS InterPro DOMAIN/s: Protein kinase, ATP binding site (InterPro:IPR017441), Protein kinase, catalytic domain (InterPro:IPR000719), Leucine-rich repeat-containing N-terminal domain, type 2 (InterPro:IPR013210), Leucine-rich repeat (InterPro:IPR001611), Serine-threonine/tyrosine-protein kinase (InterPro:IPR001245), Protein kinase-like domain (InterPro:IPR011009), Serine/threonine-protein kinase, active site (InterPro:IPR008271); BEST Arabidopsis thaliana protein match is: Leucine-rich repeat protein kinase family protein (TAIR:AT5G49760.1)
AT1G26960	*-1,512*	HOMEOBOX PROTEIN 23 (AtHB23)	Encodes a homeodomain leucine zipper class I (HD-Zip I) protein.
AT4G01430	*-1,512*	USUALLY MULTIPLE ACIDS MOVE IN AND OUT TRANSPORTERS 29 (UMAMIT29)	Encodes a plasma membrane-localized amino acid transporter likely involved in amino acid export in the developing seed.
AT1G64400	*-1,513*	LONG-CHAIN ACYL-COA SYNTHETASE 3 (LACS3)	long-chain acyl-CoA synthetase 3 (LACS3); FUNCTIONS IN: catalytic activity; INVOLVED IN: fatty acid biosynthetic process; EXPRESSED IN: 22 plant structures; EXPRESSED DURING: 13 growth stages; CONTAINS InterPro DOMAIN/s: AMP-binding, conserved site (InterPro:IPR020845), AMP-dependent synthetase/ligase (InterPro:IPR000873); BEST Arabidopsis thaliana protein match is: AMP-dependent synthetase and ligase family protein (TAIR:AT4G23850.1)
AT4G25110	*-1,514*	METACASPASE 2 (MC2)	Encodes a type I metacaspase. Two Arabidopsis metacaspases, AT1G02170 (MC1) and AT4G25110 (MC2) antagonistically control programmed cell death in Arabidopsis. MC1 is a positive regulator of cell death and requires conserved caspase-like putative catalytic residues for its function. MC2 negatively regulates cell death. This function is independent of the putative catalytic residues. A third type I Arabidopsis metacaspase is MC3 (AT5g64240).
AT2G28990	*-1,515*		Leucine-rich repeat protein kinase family protein; FUNCTIONS IN: kinase activity; INVOLVED IN: protein amino acid phosphorylation; LOCATED IN: endomembrane system; EXPRESSED IN: root; CONTAINS InterPro DOMAIN/s: Protein kinase, ATP binding site (InterPro:IPR017441), Protein kinase, catalytic domain (InterPro:IPR000719), Serine/threonine-protein kinase-like domain (InterPro:IPR017442), Protein kinase-like domain (InterPro:IPR011009), Serine/threonine-protein kinase, active site (InterPro:IPR008271); BEST Arabidopsis thaliana protein match is: Leucine-rich repeat protein kinase family protein (TAIR:AT2G28970.1)
AT1G79580	*-1,516*	SOMBRERO (SMB)	NAC-domain protein. Involved in root cap development. Involved in a regulatory feedback loop with FEZ. FEZ activates SMB in hte root cap daughter cells soon after division, and SMB in turn represses FEZ expression in these cells, thereby preventing further stem cell divisions.
AT2G34500	*-1,516*	CYTOCHROME P450, FAMILY 710, SUBFAMILY A, POLYPEPTIDE 1 (CYP710A1)	Encodes a protein with C22-sterol desaturase activity. The enzyme was shown to catalyze in the presence of NADPH the conversion of β-sitosterol to stigmasterol, but not that of 24-<i>epi</i>-campesterol to brassicasterol (unlike CYP710A2).
AT1G33890	*-1,519*		Avirulence induced gene (AIG1) family protein; FUNCTIONS IN: GTP binding; INVOLVED IN: response to bacterium; LOCATED IN: cellular_component unknown; EXPRESSED IN: pedicel; EXPRESSED DURING: 4 anthesis; CONTAINS InterPro DOMAIN/s: AIG1 (InterPro:IPR006703); BEST Arabidopsis thaliana protein match is: P-loop containing nucleoside triphosphate hydrolases superfamily protein (TAIR:AT1G33900.1)
AT1G78260	*-1,521*		RNA-binding (RRM/RBD/RNP motifs) family protein; FUNCTIONS IN: RNA binding, nucleotide binding, nucleic acid binding; INVOLVED IN: biological_process unknown; LOCATED IN: cellular_component unknown; EXPRESSED IN: 21 plant structures; EXPRESSED DURING: 11 growth stages; CONTAINS InterPro DOMAIN/s: RNA recognition motif, RNP-1 (InterPro:IPR000504), Nucleotide-binding, alpha-beta plait (InterPro:IPR012677); BEST Arabidopsis thaliana protein match is: RNA-binding (RRM/RBD/RNP motifs) family protein (TAIR:AT1G22330.1)
AT2G26820	*-1,521*	PHLOEM PROTEIN 2-A3 (PP2-A3)	phloem protein 2-A3 (PP2-A3); CONTAINS InterPro DOMAIN/s: AIG1 (InterPro:IPR006703); BEST Arabidopsis thaliana protein match is: P-loop containing nucleoside triphosphate hydrolases superfamily protein (TAIR:AT1G33900.1)

AT1G29020	-1,522		Calcium-binding EF-hand family protein; FUNCTIONS IN: calcium ion binding; LOCATED IN: endomembrane system; EXPRESSED IN: root, callus; CONTAINS InterPro DOMAIN/s: EF-Hand 1, calcium-binding site (InterPro:IPR018247), EF-HAND 2 (InterPro:IPR018249), EF-hand-like domain (InterPro:IPR011992), Calcium-binding EF-hand (InterPro:IPR002048); BEST Arabidopsis thaliana protein match is: Calcium-binding EF-hand family protein (TAIR:AT1G29025.1)
AT1G10155	-1,523	PHLOEM PROTEIN 2-A10 (PP2-A10)	phloem protein 2-A10 (PP2-A10); BEST Arabidopsis thaliana protein match is: phloem protein 2-A9 (TAIR:AT1G31200.1)
AT3G18660	-1,523	PLANT GLYCOGENIN-LIKE STARCH INITIATION PROTEIN 1 (PGSIP1)	Plants expressing an RNAi construct specifically targeting PGSIP1 was shown to have a dramatically reduced amount of starch. Encodes a glucuronyltransferase responsible for the addition of GlcA residues onto xylan and for secondary wall deposition.
AT4G01670	-1,523		unknown protein; BEST Arabidopsis thaliana protein match is: unknown protein (TAIR:AT3G62070.1)
AT5G24230	-1,524		Lipase class 3-related protein; FUNCTIONS IN: triglyceride lipase activity; INVOLVED IN: lipid metabolic process; LOCATED IN: cellular_component unknown; EXPRESSED IN: root; CONTAINS InterPro DOMAIN/s: Lipase, class 3 (InterPro:IPR002921); BEST Arabidopsis thaliana protein match is: Lipase class 3-related protein (TAIR:AT5G24220.1)
AT5G62480	-1,525	GLUTATHIONE S-TRANSFERASE TAU 9 (GSTU9)	Encodes glutathione transferase belonging to the tau class of GSTs. Naming convention according to Wagner et al. (2002).
AT1G05810	-1,528	RAB GTPASE HOMOLOG A5E (RABA5E)	RAB GTPase homolog A5E (RABA5E); FUNCTIONS IN: GTP binding; INVOLVED IN: protein transport, small GTPase mediated signal transduction; LOCATED IN: chloroplast; EXPRESSED IN: 23 plant structures; EXPRESSED DURING: 13 growth stages; CONTAINS InterPro DOMAIN/s: Ras GTPase (InterPro:IPR001806), Small GTP-binding protein (InterPro:IPR005225), Small GTPase (InterPro:IPR020851), Ras (InterPro:IPR013753), Ras small GTPase, Rab type (InterPro:IPR003579), Rab11-related (InterPro:IPR015595); BEST Arabidopsis thaliana protein match is: RAB GTPase homolog A5D (TAIR:AT2G31680.1)
AT4G18940	-1,529		RNA ligase/cyclic nucleotide phosphodiesterase family protein; FUNCTIONS IN: cyclic-nucleotide phosphodiesterase activity, catalytic activity; INVOLVED IN: RNA metabolic process; LOCATED IN: intracellular; CONTAINS InterPro DOMAIN/s: RNA ligase/cyclic nucleotide phosphodiesterase (InterPro:IPR009097), 2',3'-cyclic-nucleotide 3'-phosphodiesterase (InterPro:IPR012386); BEST Arabidopsis thaliana protein match is: RNA ligase/cyclic nucleotide phosphodiesterase family protein (TAIR:AT4G18930.1)
AT5G14880	-1,531	POTASSIUM UPTAKE 8 (KUP8)	Potassium transporter family protein; FUNCTIONS IN: potassium ion transmembrane transporter activity; INVOLVED IN: potassium ion transport; LOCATED IN: membrane; EXPRESSED IN: 22 plant structures; EXPRESSED DURING: 13 growth stages; CONTAINS InterPro DOMAIN/s: Potassium uptake protein, kup (InterPro:IPR018519), K+ potassium transporter (InterPro:IPR003855); BEST Arabidopsis thaliana protein match is: K+ uptake permease 6 (TAIR:AT1G70300.1)
AT1G64210	-1,533		Leucine-rich repeat protein kinase family protein; FUNCTIONS IN: protein serine/threonine kinase activity, kinase activity, ATP binding; INVOLVED IN: transmembrane receptor protein tyrosine kinase signaling pathway, protein amino acid phosphorylation; LOCATED IN: mitochondrion; EXPRESSED IN: flower; EXPRESSED DURING: petal differentiation and expansion stage; CONTAINS InterPro DOMAIN/s: Protein kinase, ATP binding site (InterPro:IPR017441), Protein kinase, catalytic domain (InterPro:IPR000719), Leucine-rich repeat-containing N-terminal domain, type 2 (InterPro:IPR013210), Leucine-rich repeat (InterPro:IPR001611), Serine/threonine-protein kinase-like domain (InterPro:IPR017442), Protein kinase-like domain (InterPro:IPR011009); BEST Arabidopsis thaliana protein match is: Leucine-rich repeat protein kinase family protein (TAIR:AT4G23740.1)
AT1G66690	-1,533		S-adenosyl-L-methionine-dependent methyltransferases superfamily protein; FUNCTIONS IN: S-adenosylmethionine-dependent methyltransferase activity, methyltransferase activity; INVOLVED IN: biological_process unknown; LOCATED IN: cellular_component unknown; CONTAINS InterPro DOMAIN/s: SAM dependent carboxyl methyltransferase (InterPro:IPR005299); BEST Arabidopsis thaliana protein match is: S-adenosyl-L-methionine-dependent methyltransferases superfamily protein (TAIR:AT1G66700.1)
AT1G44608	-1,534		unknown protein; CONTAINS InterPro DOMAIN/s: Protein of unknown function DUF740 (InterPro:IPR008004); BEST Arabidopsis thaliana protein match is: unknown protein (TAIR:AT1G21830.1)

AT1G80100	-1,535	HISTIDINE PHOSPHOTRANSFER PROTEIN 6 (HP6)	AHP6 lacks the conserved histidine residue (Asn83 in AHP6b), which is required for phosphotransfer, present in the other AHPs. AHP6 does not appear to have phosphotransfer activity. Acts as an inhibitor of cytokinin signaling by interacting with the phosphorelay machinery. Expressed in developing protoxylem and associated pericycle cell files. Negative regulator of cytokinin signaling. Expression is down-regulated by cytokinins. There are two alternatively spliced genes for this locus, AHP6a and AHP6b, differing in the length of the first exon. In ahp6-2 seedlings, only the AHP6a transcript is present. Members of the AHP gene family include: AT3G21510 (AHP1), AT3G29350 (AHP2), AT5G39340 (AHP3), AT3G16360 (AHP4), AT1G03430 (AHP5) and AT1G80100 (AHP6).
AT2G24610	-1,535	CYCLIC NUCLEOTIDE-GATED CHANNEL 14 (CNGC14)	member of Cyclic nucleotide gated channel family
AT2G14820	-1,536	NAKED PINS IN YUC MUTANTS 2 (NPY2)	A member of the NPY gene family (NPY1/AT4G31820, NPY2/AT2G14820, NPY3/AT5G67440, NPY4/AT2G23050, NPY5/AT4G37590). Involved in auxin-mediated organogenesis.
AT2G29320	-1,536		NAD(P)-binding Rossmann-fold superfamily protein; FUNCTIONS IN: oxidoreductase activity, binding, catalytic activity; INVOLVED IN: oxidation reduction, metabolic process; LOCATED IN: cellular_component unknown; EXPRESSED IN: 21 plant structures; EXPRESSED DURING: 13 growth stages; CONTAINS InterPro DOMAIN/s: NAD(P)-binding domain (InterPro:IPR016040), Glucose/ribitol dehydrogenase (InterPro:IPR002347), Short-chain dehydrogenase/reductase SDR (InterPro:IPR002198); BEST Arabidopsis thaliana protein match is: NAD(P)-binding Rossmann-fold superfamily protein (TAIR:AT2G29300.1)
AT3G04010	-1,537		O-Glycosyl hydrolases family 17 protein; FUNCTIONS IN: cation binding, hydrolase activity, hydrolyzing O-glycosyl compounds, catalytic activity; INVOLVED IN: carbohydrate metabolic process; LOCATED IN: anchored to membrane; EXPRESSED IN: 11 plant structures; EXPRESSED DURING: L mature pollen stage, LP.06 six leaves visible, M germinated pollen stage, LP.04 four leaves visible, 4 anthesis; CONTAINS InterPro DOMAIN/s: X8 (InterPro:IPR012946), Glycoside hydrolase, catalytic core (InterPro:IPR017853), Glycoside hydrolase, family 17 (InterPro:IPR000490), Glycoside hydrolase, subgroup, catalytic core (InterPro:IPR013781); BEST Arabidopsis thaliana protein match is: O-Glycosyl hydrolases family 17 protein (TAIR:AT5G18220.1)
AT3G06390	-1,537	CASP-LIKE PROTEIN 1D2 (CASPL1D2)	Uncharacterised protein family (UPF0497); CONTAINS InterPro DOMAIN/s: Uncharacterised protein family UPF0497, trans-membrane plant (InterPro:IPR006702), Uncharacterised protein family UPF0497, trans-membrane plant subgroup (InterPro:IPR006459); BEST Arabidopsis thaliana protein match is: Uncharacterised protein family (UPF0497) (TAIR:AT4G15610.1)
AT5G35770	-1,538	STERILE APETALA (SAP)	A recessive mutation in the Arabidopsis STERILE APETALA (SAP) causes severe aberrations in inflorescence and flower and ovule development.
AT5G14120	-1,539		Major facilitator superfamily protein; LOCATED IN: plasma membrane, vacuole; EXPRESSED IN: 26 plant structures; EXPRESSED DURING: 15 growth stages; CONTAINS InterPro DOMAIN/s: Nodulin-like (InterPro:IPR010658), Major facilitator superfamily, general substrate transporter (InterPro:IPR016196); BEST Arabidopsis thaliana protein match is: Major facilitator superfamily protein (TAIR:AT3G01930.2)
AT2G19150	-1,541		Pectin lyase-like superfamily protein; FUNCTIONS IN: pectinesterase activity; INVOLVED IN: cell wall modification; LOCATED IN: endomembrane system, cell wall, plant-type cell wall; EXPRESSED IN: 6 plant structures; EXPRESSED DURING: LP.04 four leaves visible; CONTAINS InterPro DOMAIN/s: Pectin lyase fold/virulence factor (InterPro:IPR011050), Pectinesterase, catalytic (InterPro:IPR000070), Pectin lyase fold (InterPro:IPR012334); BEST Arabidopsis thaliana protein match is: Pectin lyase-like superfamily protein (TAIR:AT2G47280.1)
AT2G42350	-1,543		RING/U-box superfamily protein; FUNCTIONS IN: zinc ion binding; EXPRESSED IN: stem, root, stamen; EXPRESSED DURING: 4 anthesis; CONTAINS InterPro DOMAIN/s: Zinc finger, RING-type (InterPro:IPR001841), Zinc finger, C3HC4 RING-type (InterPro:IPR018957); BEST Arabidopsis thaliana protein match is: RING/U-box superfamily protein (TAIR:AT2G42360.1)
AT3G58190	-1,544	LATERAL ORGAN BOUNDARIES-DOMAIN 29 (LBD29)	This gene contains two auxin-responsive element (AuxRE).
AT1G67870	-1,545		glycine-rich protein

AT2G03370	*-1,546*		Glycosyltransferase family 61 protein; FUNCTIONS IN: transferase activity, transferring glycosyl groups; INVOLVED IN: biological_process unknown; LOCATED IN: endomembrane system; CONTAINS InterPro DOMAIN/s: Glycosyltransferase AER61, uncharacterised (InterPro:IPR007657); BEST Arabidopsis thaliana protein match is: Glycosyltransferase family 61 protein (TAIR:AT2G03360.1)
AT1G15890	*-1,55*		Disease resistance protein (CC-NBS-LRR class) family; FUNCTIONS IN: ATP binding; INVOLVED IN: N-terminal protein myristoylation, apoptosis, defense response; LOCATED IN: cellular_component unknown; EXPRESSED IN: 8 plant structures; EXPRESSED DURING: 6 growth stages; CONTAINS InterPro DOMAIN/s: NB-ARC (InterPro:IPR002182), Leucine-rich repeat (InterPro:IPR001611), Disease resistance protein (InterPro:IPR000767); BEST Arabidopsis thaliana protein match is: Disease resistance protein (CC-NBS-LRR class) family (TAIR:AT5G43730.1)
AT2G42430	*-1,55*	LATERAL ORGAN BOUNDARIES-DOMAIN 16 (LBD16)	LOB-domain protein gene LBD16. This gene contains one auxin-responsive element (AuxRE).
AT1G07550	*-1,551*		Leucine-rich repeat protein kinase family protein; FUNCTIONS IN: kinase activity; INVOLVED IN: protein amino acid phosphorylation; LOCATED IN: endomembrane system; EXPRESSED IN: root, leaf; EXPRESSED DURING: LP.08 eight leaves visible; CONTAINS InterPro DOMAIN/s: Protein kinase, ATP binding site (InterPro:IPR017441), Protein kinase, catalytic domain (InterPro:IPR000719), Leucine-rich repeat (InterPro:IPR001611), Serine-threonine/tyrosine-protein kinase (InterPro:IPR001245), Protein kinase-like domain (InterPro:IPR011009), Serine/threonine-protein kinase, active site (InterPro:IPR008271); BEST Arabidopsis thaliana protein match is: Leucine-rich repeat protein kinase family protein (TAIR:AT2G14510.1)
AT5G43770	*-1,551*		proline-rich family protein; FUNCTIONS IN: molecular_function unknown; INVOLVED IN: biological_process unknown; LOCATED IN: chloroplast
AT4G34800	*-1,552*	SMALL AUXIN UPREGULATED RNA 4 (SAUR4)	SAUR-like auxin-responsive protein family ; CONTAINS InterPro DOMAIN/s: Auxin responsive SAUR protein (InterPro:IPR003676); BEST Arabidopsis thaliana protein match is: SAUR-like auxin-responsive protein family (TAIR:AT4G34810.1)
AT1G51800	*-1,553*	IMPAIRED OOMYCETE SUSCEPTIBILITY 1 (IOS1)	The gene encodes a putative member of the LRR-RLK protein family. Expressin and mutant analysis revealed that it contributes to the interaction between Arabidopsis and Hyaloperonospora arabidopsidis. and The mRNA is cell-to-cell mobile.
AT4G05170	*-1,553*		basic helix-loop-helix (bHLH) DNA-binding superfamily protein; FUNCTIONS IN: DNA binding, sequence-specific DNA binding transcription factor activity; INVOLVED IN: regulation of transcription; LOCATED IN: nucleus; EXPRESSED IN: 9 plant structures; EXPRESSED DURING: 6 growth stages; CONTAINS InterPro DOMAIN/s: Helix-loop-helix DNA-binding domain (InterPro:IPR001092), Helix-loop-helix DNA-binding (InterPro:IPR011598); BEST Arabidopsis thaliana protein match is: basic helix-loop-helix (bHLH) DNA-binding superfamily protein (TAIR:AT4G21340.1)
AT3G24300	*-1,556*	AMMONIUM TRANSPORTER 1;3 (AMT1;3)	Encodes a plasma membrane localized ammonium transporter.
AT1G80340	*-1,557*	GIBBERELLIN 3-OXIDASE 2 (GA3OX2)	Encodes a protein with gibberellin 3 β-hydroxylase activity. The protein was heterologously expressed in E. coli and shown to catalyze the hydroxylation of both GA9 and GA20.
AT5G14070	*-1,557*	(ROXY2)	Encodes glutaredoxin ROXY2. ROXY2, together with ROXY1 (AT3G02000), controls anther development. roxy1 roxy2 double mutants are sterile and do not produce pollen.
AT5G07190	*-1,558*	SEED GENE 3 (ATS3)	Gene is expressed preferentially in the embryo and encodes a unique protein of unknown function.
AT5G51160	*-1,558*		Ankyrin repeat family protein; CONTAINS InterPro DOMAIN/s: Ankyrin repeat-containing domain (InterPro:IPR020683), Ankyrin repeat (InterPro:IPR002110); BEST Arabidopsis thaliana protein match is: Ankyrin repeat family protein (TAIR:AT4G10720.1)
AT5G66580	*-1,559*		unknown protein; FUNCTIONS IN: molecular_function unknown; INVOLVED IN: N-terminal protein myristoylation; LOCATED IN: cellular_component unknown; BEST Arabidopsis thaliana protein match is: unknown protein (TAIR:AT3G50800.1)
AT2G17890	*-1,56*	CALCIUM-DEPENDENT PROTEIN KINASE 16 (CPK16)	Encodes a member of Calcium Dependent Protein Kinase. Protein is N-myristoylated. Localizes to the plasma membrane. Localizes to the chloroplast when the myristoylation motif is mutated.
AT5G22390	*-1,56*		Protein of unknown function (DUF3049); CONTAINS InterPro DOMAIN/s: Protein of unknown function DUF3049 (InterPro:IPR021410); BEST Arabidopsis thaliana protein match is: Protein of unknown function (DUF3049) (TAIR:AT1G54740.1)

AT2G45960	-1,562	PLASMA MEMBRANE INTRINSIC PROTEIN 1B (PIP1B)	a member of the plasma membrane intrinsic protein subfamily PIP1. localizes to the plasma membrane and exhibits water transport activity in Xenopus oocyte. expressed ubiquitously and protein level decreases slightly during leaf development.
AT1G58270	-1,563	(ZW9)	ZW9 mRNA, complete cds The mRNA is cell-to-cell mobile.
AT1G55290	-1,564	(F6'H2)	encodes a protein whose sequence is similar to oxidoreductase, 2OG-Fe(II) oxygenase
AT2G04170	-1,565		TRAF-like family protein; CONTAINS InterPro DOMAIN/s: TRAF-like (InterPro:IPR008974), MATH (InterPro:IPR002083); BEST Arabidopsis thaliana protein match is: TRAF-like family protein (TAIR:AT5G26280.1)
AT2G38300	-1,567		myb-like HTH transcriptional regulator family protein; CONTAINS InterPro DOMAIN/s: Homeodomain-like (InterPro:IPR009057), Myb, DNA-binding (InterPro:IPR014778), Myb-like DNA-binding domain, SHAQKYF class (InterPro:IPR006447), HTH transcriptional regulator, Myb-type, DNA-binding (InterPro:IPR017930), Homeodomain-related (InterPro:IPR012287); BEST Arabidopsis thaliana protein match is: Homeodomain-like superfamily protein (TAIR:AT2G40260.1)
AT2G47150	-1,569		NAD(P)-binding Rossmann-fold superfamily protein; FUNCTIONS IN: oxidoreductase activity, binding, catalytic activity; INVOLVED IN: oxidation reduction, metabolic process; LOCATED IN: cellular_component unknown; CONTAINS InterPro DOMAIN/s: NAD(P)-binding domain (InterPro:IPR016040), Glucose/ribitol dehydrogenase (InterPro:IPR002347), Short-chain dehydrogenase/reductase SDR (InterPro:IPR002198); BEST Arabidopsis thaliana protein match is: NAD(P)-binding Rossmann-fold superfamily protein (TAIR:AT2G47130.1)
AT5G01490	-1,569	CATION EXCHANGER 4 (CAX4)	Encodes a cation/proton antiporter, a member of low affinity calcium antiporter CAX2 family. Involved in root development under metal stress.
AT1G68150	-1,57	WRKY DNA-BINDING PROTEIN 9 (WRKY9)	member of WRKY Transcription Factor; Group II-b The mRNA is cell-to-cell mobile.
AT2G42380	-1,571	(BZIP34)	Encodes a member of the BZIP family of transcription factors. Forms heterodimers with the related protein AtbZIP61. Binds to G-boxes in vitro and is localized to the nucleus in onion epidermal cells.
AT1G43140	-1,573		Cullin family protein; FUNCTIONS IN: ubiquitin protein ligase binding; INVOLVED IN: ubiquitin-dependent protein catabolic process; LOCATED IN: endomembrane system, cullin-RING ubiquitin ligase complex; EXPRESSED IN: root, flower; EXPRESSED DURING: petal differentiation and expansion stage; CONTAINS InterPro DOMAIN/s: Winged helix-turn-helix transcription repressor DNA-binding (InterPro:IPR011991), Cullin homology (InterPro:IPR016158), Cullin protein, neddylation domain (InterPro:IPR019559), Cullin, N-terminal (InterPro:IPR001373), Cullin repeat-like-containing domain (InterPro:IPR016159); BEST Arabidopsis thaliana protein match is: cullin 2 (TAIR:AT1G02980.1)
AT2G43870	-1,576		Pectin lyase-like superfamily protein; FUNCTIONS IN: polygalacturonase activity; INVOLVED IN: carbohydrate metabolic process; LOCATED IN: endomembrane system; EXPRESSED IN: sepal, male gametophyte, stamen; EXPRESSED DURING: 4 anthesis; CONTAINS InterPro DOMAIN/s: Pectin lyase fold/virulence factor (InterPro:IPR011050), Pectin lyase fold (InterPro:IPR012334), Glycoside hydrolase, family 28 (InterPro:IPR000743), Parallel beta-helix repeat (InterPro:IPR006626); BEST Arabidopsis thaliana protein match is: Pectin lyase-like superfamily protein (TAIR:AT3G59850.1)
AT4G19030	-1,576	NOD26-LIKE MAJOR INTRINSIC PROTEIN 1 (NLM1)	an aquaporin whose expression level is reduced by ABA, NaCl, dark, and desiccation. is expressed at relatively low levels under normal conditions. Also functions in arsenite transport and tolerance.
AT2G02700	-1,577		Cysteine/Histidine-rich C1 domain family protein; FUNCTIONS IN: zinc ion binding; LOCATED IN: cellular_component unknown; CONTAINS InterPro DOMAIN/s: DC1 (InterPro:IPR004146), Zinc finger, PHD-type (InterPro:IPR001965), C1-like (InterPro:IPR011424); BEST Arabidopsis thaliana protein match is: Cysteine/Histidine-rich C1 domain family protein (TAIR:AT2G02680.1)
AT2G14960	-1,578	(GH3.1)	encodes a protein similar to IAA-amido synthases. Lines carrying an insertion in this gene are hypersensitive to auxin.
AT2G42060	-1,578		Cysteine/Histidine-rich C1 domain family protein; INVOLVED IN: intracellular signaling pathway; LOCATED IN: cellular_component unknown; EXPRESSED IN: root; CONTAINS InterPro DOMAIN/s: Protein kinase C-like, phorbol ester/diacylglycerol binding (InterPro:IPR002219), C1-like (InterPro:IPR011424); BEST Arabidopsis thaliana protein match is: Cysteine/Histidine-rich C1 domain family protein (TAIR:AT2G27660.1)
AT5G17430	-1,579	BABY BOOM (BBM)	Encodes an AP2-domain containing protein similar to ANT. Expressed in embryos and lateral root primordium.

AT1G02640	*-1,58*	BETA-XYLOSIDASE 2 (BXL2)	encodes a protein similar to a beta-xylosidase located in the extracellular matrix. This is a member of glycosyl hydrolase family 3 and has six other closely related members.
AT1G49030	*-1,58*		PLAC8 family protein; CONTAINS InterPro DOMAIN/s: Protein of unknown function Cys-rich (InterPro:IPR006461); BEST Arabidopsis thaliana protein match is: PLAC8 family protein (TAIR:AT3G18460.1)
AT3G63470	*-1,581*	SERINE CARBOXYPEPTIDASE-LIKE 40 (scpl40)	serine carboxypeptidase-like 40 (scpl40); FUNCTIONS IN: serine-type carboxypeptidase activity; INVOLVED IN: proteolysis; LOCATED IN: endomembrane system; EXPRESSED IN: 8 plant structures; EXPRESSED DURING: 4 anthesis, C globular stage, petal differentiation and expansion stage; CONTAINS InterPro DOMAIN/s: Peptidase S10, serine carboxypeptidase (InterPro:IPR001563), Peptidase S10, serine carboxypeptidase, active site (InterPro:IPR018202); BEST Arabidopsis thaliana protein match is: serine carboxypeptidase-like 39 (TAIR:AT3G52020.1)
AT5G20110	*-1,582*		Dynein light chain type 1 family protein; FUNCTIONS IN: microtubule motor activity; INVOLVED IN: microtubule-based process; LOCATED IN: microtubule associated complex; EXPRESSED IN: 19 plant structures; EXPRESSED DURING: 11 growth stages; CONTAINS InterPro DOMAIN/s: Dynein light chain, type 1/2 (InterPro:IPR001372); BEST Arabidopsis thaliana protein match is: Dynein light chain type 1 family protein (TAIR:AT1G23220.1)
AT5G24220	*-1,582*		Lipase class 3-related protein; FUNCTIONS IN: triglyceride lipase activity; INVOLVED IN: lipid metabolic process; LOCATED IN: cellular_component unknown; CONTAINS InterPro DOMAIN/s: Lipase, class 3 (InterPro:IPR002921); BEST Arabidopsis thaliana protein match is: Lipase class 3-related protein (TAIR:AT5G24180.1)
AT4G32460	*-1,583*		FUNCTIONS IN: molecular_function unknown; INVOLVED IN: biological_process unknown; LOCATED IN: plant-type cell wall; EXPRESSED IN: 24 plant structures; EXPRESSED DURING: 15 growth stages; CONTAINS InterPro DOMAIN/s: Protein of unknown function DUF642 (InterPro:IPR006946), Galactose-binding domain-like (InterPro:IPR008979); BEST Arabidopsis thaliana protein match is: Protein of unknown function, DUF642 (TAIR:AT5G11420.1)
AT5G26010	*-1,585*		Protein phosphatase 2C family protein; FUNCTIONS IN: protein serine/threonine phosphatase activity, catalytic activity; INVOLVED IN: N-terminal protein myristoylation; LOCATED IN: cellular_component unknown; EXPRESSED IN: hypocotyl, root, leaf; EXPRESSED DURING: LP.04 four leaves visible; CONTAINS InterPro DOMAIN/s: Protein phosphatase 2C-related (InterPro:IPR001932), Protein phosphatase 2C, N-terminal (InterPro:IPR014045); BEST Arabidopsis thaliana protein match is: Protein phosphatase 2C family protein (TAIR:AT4G32950.1)
AT1G05220	*-1,588*		Transmembrane protein 97, predicted; FUNCTIONS IN: molecular_function unknown; INVOLVED IN: biological_process unknown; LOCATED IN: endomembrane system; CONTAINS InterPro DOMAIN/s: Transmembrane protein 97, predicted (InterPro:IPR016964); BEST Arabidopsis thaliana protein match is: Transmembrane protein 97, predicted (TAIR:AT1G05210.1)
AT2G34910	*-1,588*		BEST Arabidopsis thaliana protein match is: root hair specific 4 (TAIR:AT1G30850.1)
AT3G12970	*-1,588*		unknown protein; BEST Arabidopsis thaliana protein match is: unknown protein (TAIR:AT1G56020.1)
AT5G58770	*-1,589*	CIS-PRENYLTRANSFERASE 4 (CPT4)	Undecaprenyl pyrophosphate synthetase family protein; FUNCTIONS IN: dehydrodolichyl diphosphate synthase activity; INVOLVED IN: dolichol biosynthetic process; LOCATED IN: chloroplast; EXPRESSED IN: 15 plant structures; EXPRESSED DURING: 9 growth stages; CONTAINS InterPro DOMAIN/s: Di-trans-poly-cis-decaprenylcistransferase-like, conserved site (InterPro:IPR018520), Di-trans-poly-cis-decaprenylcistransferase-like (InterPro:IPR001441); BEST Arabidopsis thaliana protein match is: Undecaprenyl pyrophosphate synthetase family protein (TAIR:AT5G58782.1)
AT5G02260	*-1,59*	EXPANSIN A9 (EXPA9)	member of Alpha-Expansin Gene Family. Naming convention from the Expansin Working Group (Kende et al, 2004. Plant Mol Bio)
AT5G37800	*-1,59*	RHD SIX-LIKE 1 (RSL1)	RHD SIX-LIKE 1 (RSL1); FUNCTIONS IN: DNA binding, sequence-specific DNA binding transcription factor activity; INVOLVED IN: regulation of transcription; LOCATED IN: nucleus; CONTAINS InterPro DOMAIN/s: Helix-loop-helix DNA-binding domain (InterPro:IPR001092), Helix-loop-helix DNA-binding (InterPro:IPR011598); BEST Arabidopsis thaliana protein match is: ROOT HAIR DEFECTIVE6 (TAIR:AT1G66470.1)
AT5G52260	*-1,59*	MYB DOMAIN PROTEIN 19 (MYB19)	Member of the R2R3 factor gene family.
AT4G16350	*-1,591*	CALCINEURIN B-LIKE PROTEIN 6 (CBL6)	Calcium sensor protein. Binds CIPK14.

AT4G33800	*-1,591*		unknown protein
AT2G18650	*-1,592*	MATERNAL EFFECT EMBRYO ARREST 16 (MEE16)	maternal effect embryo arrest 16 (MEE16); FUNCTIONS IN: zinc ion binding; INVOLVED IN: embryo development ending in seed dormancy; LOCATED IN: chloroplast; CONTAINS InterPro DOMAIN/s: Zinc finger, RING-type (InterPro:IPR001841), Zinc finger, C3HC4 RING-type (InterPro:IPR018957); BEST Arabidopsis thaliana protein match is: RING/U-box superfamily protein (TAIR:AT4G30400.1)
AT3G08770	*-1,592*	LIPID TRANSFER PROTEIN 6 (LTP6)	Predicted to encode a PR (pathogenesis-related) protein. Belongs to the lipid transfer protein (PR-14) family with the following members: At2g38540/LTP1, At2g38530/LTP2, At5g59320/LTP3, At5g59310/LTP4, At3g51600/LTP5, At3g08770/LTP6, At2g15050/LTP7, At2g18370/LTP8, At2g15325/LTP9, At5g01870/LTP10, At4g33355/LTP11, At3g51590/LTP12, At5g44265/LTP13, At5g62065/LTP14, At4g08530/LTP15.
AT1G13080	*-1,593*	CYTOCHROME P450, FAMILY 71, SUBFAMILY B, POLYPEPTIDE 2 (CYP71B2)	cytochrome P450 monooxygenase
AT2G31081	*-1,594*	CLAVATA3/ESR-RELATED 4 (CLE4)	Member of a large family of putative ligands homologous to the Clavata3 gene. Consists of a single exon.
AT2G40620	*-1,594*	BASIC LEUCINE-ZIPPER 18 (BZIP18)	Basic-leucine zipper (bZIP) transcription factor family protein; FUNCTIONS IN: DNA binding, sequence-specific DNA binding transcription factor activity; INVOLVED IN: regulation of transcription, DNA-dependent; EXPRESSED IN: 24 plant structures; EXPRESSED DURING: 15 growth stages; CONTAINS InterPro DOMAIN/s: Basic-leucine zipper (bZIP) transcription factor (InterPro:IPR004827), bZIP transcription factor, bZIP-1 (InterPro:IPR011616); BEST Arabidopsis thaliana protein match is: Basic-leucine zipper (bZIP) transcription factor family protein (TAIR:AT2G31370.5)
AT4G15760	*-1,594*	MONOOXYGENASE 1 (MO1)	Encodes a protein with similarity to monooxygenases that are known to degrade salicylic acid (SA).
AT1G11000	*-1,595*	MILDEW RESISTANCE LOCUS O 4 (MLO4)	A member of a large family of seven-transmembrane domain proteins specific to plants, homologs of the barley mildew resistance locus o (MLO) protein. The Arabidopsis genome contains 15 genes encoding MLO proteins, with localization in plasma membrane. Phylogenetic analysis revealed four clades of closely-related AtMLO genes. ATMLO4 belongs to the clade I, with AtMLO11 and AtMLO14. The gene is expressed during early seedling growth, in roots and lateral root primordia, in flower and fruit abscission zone, in vascular system of root, cotyledons and young leaves, it was not expressed in mature rosette leaves, as shown by GUS activity patterns. The expression of several phylogenetically closely-related AtMLO genes showed similar or overlapping tissue specificity and analogous responsiveness to external stimuli, suggesting functional redundancy, co-function, or antagonistic function(s).
AT1G66470	*-1,598*	ROOT HAIR DEFECTIVE6 (RHD6)	ROOT HAIR DEFECTIVE6 (RHD6); CONTAINS InterPro DOMAIN/s: Helix-loop-helix DNA-binding domain (InterPro:IPR001092), Helix-loop-helix DNA-binding (InterPro:IPR011598); BEST Arabidopsis thaliana protein match is: RHD SIX-LIKE 1 (TAIR:AT5G37800.1)
AT5G44260	*-1,598*	TANDEM CCCH ZINC FINGER PROTEIN 5 (TZF5)	Encodes a Tandem CCCH Zinc Finger protein. Interacts and co-localizes with MARD1 and RD21A in processing bodies (PBs) and stress granules (SGs).
AT5G53550	*-1,598*	YELLOW STRIPE LIKE 3 (YSL3)	YELLOW STRIPE like 3 (YSL3); CONTAINS InterPro DOMAIN/s: Oligopeptide transporter OPT superfamily (InterPro:IPR004813); BEST Arabidopsis thaliana protein match is: YELLOW STRIPE like 2 (TAIR:AT5G24380.1)
AT2G15390	*-1,599*	FUCOSYLTRANSFERASE 4 (FUT4)	Encodes an alpha-(1,2)-fucosyltransferase.
AT4G00070	*-1,6*		RING/U-box superfamily protein; BEST Arabidopsis thaliana protein match is: RING/U-box superfamily protein (TAIR:AT1G36950.1)
AT2G42660	*-1,601*		Homeodomain-like superfamily protein; CONTAINS InterPro DOMAIN/s: Homeodomain-like (InterPro:IPR009057), Myb, DNA-binding (InterPro:IPR014778), Myb-like DNA-binding domain, SHAQKYF class (InterPro:IPR006447), HTH transcriptional regulator, Myb-type, DNA-binding (InterPro:IPR017930), Homeodomain-related (InterPro:IPR012287); BEST Arabidopsis thaliana protein match is: myb-like HTH transcriptional regulator family protein (TAIR:AT2G38300.1)

AT2G25260	-1,603	HYDROXYPROLINE O-ARABINOSYLATRANSFERASE 2 (HPAT2)	unknown protein; FUNCTIONS IN: molecular_function unknown; INVOLVED IN: biological_process unknown; LOCATED IN: endomembrane system; BEST Arabidopsis thaliana protein match is: unknown protein (TAIR:AT5G25265.1)
AT1G06980	-1,604		unknown protein; FUNCTIONS IN: molecular_function unknown; INVOLVED IN: biological_process unknown; LOCATED IN: endomembrane system; EXPRESSED IN: sepal, male gametophyte, carpel; EXPRESSED DURING: 4 anthesis; BEST Arabidopsis thaliana protein match is: unknown protein (TAIR:AT2G30230.1)
AT4G28890	-1,606		RING/U-box superfamily protein; FUNCTIONS IN: ubiquitin-protein ligase activity, zinc ion binding; LOCATED IN: endomembrane system; EXPRESSED IN: root stele, root endodermis, hypocotyl, root, primary root differentiation zone; CONTAINS InterPro DOMAIN/s: Zinc finger, RING-type (InterPro:IPR001841), Zinc finger, C3HC4 RING-type (InterPro:IPR018957); BEST Arabidopsis thaliana protein match is: RING/U-box superfamily protein (TAIR:AT2G20030.1)
AT3G01290	-1,607	HYPERSENSITIVE INDUCED REACTION 2 (HIR2)	SPFH/Band 7/PHB domain-containing membrane-associated protein family; INVOLVED IN: N-terminal protein myristoylation; LOCATED IN: mitochondrion, plasma membrane, vacuole, membrane; EXPRESSED IN: 21 plant structures; EXPRESSED DURING: 9 growth stages; CONTAINS InterPro DOMAIN/s: Band 7 protein (InterPro:IPR001107); BEST Arabidopsis thaliana protein match is: SPFH/Band 7/PHB domain-containing membrane-associated protein family (TAIR:AT5G62740.1)
AT2G35210	-1,61	ROOT AND POLLEN ARFGAP (RPA)	A member of ARF GAP domain (AGD), A thaliana has 15 members, grouped into four classes.
AT3G30180	-1,61	BRASSINOSTEROID-6-OXIDASE 2 (BR6OX2)	Encodes a cytochrome p450 enzyme that catalyzes the last reaction in the production of brassinolide. It is capable of converting 6-deoxocastasterone into castasterone, a C-6 oxidation, as well as the further conversion of castasterone into brassinolide by a Baeyer-Villinger oxidation reaction at C-6, resulting in the formation of an unusual seven-membered lactone ring. The enzyme possesses high affinity for both C28- and C27-Brassinosteroids. The expression of the gene using a CYP85A2 promoter:LUC fusion construct was shown to be under circadian and light control.
AT1G80920	-1,611	(J8)	A nuclear encoded soluble protein found in the chloroplast stroma. Negatively regulated by light and has rapid turnover in darkness.
AT5G01330	-1,611	PYRUVATE DECARBOXYLASE-3 (PDC3)	pyruvate decarboxylase
AT4G26220	-1,613	CAFFEOYL COENZYME A ESTER O-METHYLTRANSFERASE 7 (CCOAOMT7)	Encodes a caffeoyl-coenzyme A O-methyltransferase (CCoAOMT)-like protein with a strong preference for methylating the para position of flavanones and dihydroflavonols, whereas flavones and flavonols are methylated in the meta-position.
AT5G61250	-1,614	GLUCURONIDASE 1 (GUS1)	Belongs to the plant glycoside hydrolase family 79. Encodes a protein with several posttranslational modification sites including O-β-GlcNAc attachment sites and serine-, threonine- and tyrosine-phosphorylation sites, suggesting that this protein is extensively modified posttranslationally. The protein is predicted (WoLF PSORT program) to be secreted.
AT3G58120	-1,617	(BZIP61)	Encodes a member of the BZIP family of transcription factors. Forms heterodimers with the related protein AtbZIP34. Binds to G-boxes in vitro and is localized to the nucleus in onion epidermal cells.
AT5G23950	-1,617		Calcium-dependent lipid-binding (CaLB domain) family protein; CONTAINS InterPro DOMAIN/s: C2 calcium/lipid-binding domain, CaLB (InterPro:IPR008973), C2 calcium-dependent membrane targeting (InterPro:IPR000008); BEST Arabidopsis thaliana protein match is: Calcium-dependent lipid-binding (CaLB domain) family protein (TAIR:AT1G07310.1)
AT5G12940	-1,618		Leucine-rich repeat (LRR) family protein; INVOLVED IN: signal transduction; LOCATED IN: endomembrane system; EXPRESSED IN: 18 plant structures; EXPRESSED DURING: 10 growth stages; CONTAINS InterPro DOMAIN/s: Leucine-rich repeat-containing N-terminal domain, type 2 (InterPro:IPR013210), Leucine-rich repeat (InterPro:IPR001611); BEST Arabidopsis thaliana protein match is: Leucine-rich repeat (LRR) family protein (TAIR:AT3G20820.1)
AT4G14650	-1,619		unknown protein; FUNCTIONS IN: molecular_function unknown; INVOLVED IN: N-terminal protein myristoylation; EXPRESSED IN: 6 plant structures; EXPRESSED DURING: petal differentiation and expansion stage
AT4G23870	-1,619		unknown protein; BEST Arabidopsis thaliana protein match is: unknown protein (TAIR:AT4G11020.1)

AT5G58750	-1,62		NAD(P)-binding Rossmann-fold superfamily protein; BEST Arabidopsis thaliana protein match is: NAD(P)-binding Rossmann-fold superfamily protein (TAIR:AT4G24220.2)
AT1G78340	-1,621	GLUTATHIONE S-TRANSFERASE TAU 22	Encodes glutathione transferase belonging to the tau class of GSTs. Naming convention according to Wagner et al. (2002).
AT5G51930	-1,621		Glucose-methanol-choline (GMC) oxidoreductase family protein; FUNCTIONS IN: aldehyde-lyase activity, oxidoreductase activity, acting on CH-OH group of donors, FAD binding; INVOLVED IN: alcohol metabolic process; LOCATED IN: endomembrane system; CONTAINS InterPro DOMAIN/s: Glucose-methanol-choline oxidoreductase, N-terminal (InterPro:IPR000172), Glucose-methanol-choline oxidoreductase (InterPro:IPR012132), Glucose-methanol-choline oxidoreductase, C-terminal (InterPro:IPR007867); BEST Arabidopsis thaliana protein match is: Glucose-methanol-choline (GMC) oxidoreductase family protein (TAIR:AT3G56060.1)
AT1G28160	-1,622		encodes a member of the ERF (ethylene response factor) subfamily B-1 of ERF/AP2 transcription factor family. The protein contains one AP2 domain. There are 15 members in this subfamily including ATERF-3, ATERF-4, ATERF-7, and leafy petiole.
AT1G33540	-1,623	SERINE CARBOXYPEPTIDASE-LIKE 18 (scpl18)	serine carboxypeptidase-like 18 (scpl18); FUNCTIONS IN: serine-type carboxypeptidase activity; INVOLVED IN: proteolysis; LOCATED IN: endomembrane system; EXPRESSED IN: root; CONTAINS InterPro DOMAIN/s: Peptidase S10, serine carboxypeptidase (InterPro:IPR001563); BEST Arabidopsis thaliana protein match is: serine carboxypeptidase-like 7 (TAIR:AT3G10450.1)
AT4G36410	-1,623	UBIQUITIN-CONJUGATING ENZYME 17 (UBC17)	ubiquitin-conjugating enzyme
AT1G51850	-1,626		Leucine-rich repeat protein kinase family protein; FUNCTIONS IN: kinase activity; INVOLVED IN: protein amino acid phosphorylation; LOCATED IN: plasma membrane; EXPRESSED IN: root; CONTAINS InterPro DOMAIN/s: Protein kinase, ATP binding site (InterPro:IPR017441), Serine/threonine-protein kinase domain (InterPro:IPR002290), Leucine-rich repeat (InterPro:IPR001611), Serine/threonine-protein kinase-like domain (InterPro:IPR017442), Protein kinase-like domain (InterPro:IPR011009), Serine/threonine-protein kinase, active site (InterPro:IPR008271), Protein kinase, catalytic domain (InterPro:IPR000719), Tyrosine-protein kinase, catalytic domain (InterPro:IPR020635); BEST Arabidopsis thaliana protein match is: Leucine-rich repeat protein kinase family protein (TAIR:AT1G51820.1)
AT2G28110	-1,627	FRAGILE FIBER 8 (FRA8)	Homolog to AT5G22940, a member of glycosyltransferase family 47 that is involved in secondary cell wall biosynthesis. It exhibits high sequence similarity to tobacco (Nicotiana plumbaginifolia) pectin glucuronyltransferase. Protein has a domain that shares significant similarity with the pfam03016 domain. It is expressed specifically in developing vessels and fiber cells, and FRA8 is targeted to Golgi. Mutants have irregular xylem formation, reduced cellulose levels and plants are smaller than normal siblings.
AT2G39520	-1,627		unknown protein
AT4G02830	-1,627		unknown protein
AT5G66490	-1,627		unknown protein; FUNCTIONS IN: molecular_function unknown; INVOLVED IN: biological_process unknown; LOCATED IN: plasma membrane; EXPRESSED IN: 17 plant structures; EXPRESSED DURING: 9 growth stages; BEST Arabidopsis thaliana protein match is: unknown protein (TAIR:AT3G50900.1)
AT1G21110	-1,632	INDOLE GLUCOSINOLATE O-METHYLTRANSFERASE 3 (IGMT3)	O-methyltransferase family protein; FUNCTIONS IN: methyltransferase activity, O-methyltransferase activity, protein dimerization activity; LOCATED IN: cytosol; CONTAINS InterPro DOMAIN/s: Winged helix-turn-helix transcription repressor DNA-binding (InterPro:IPR011991), Plant methyltransferase dimerisation (InterPro:IPR012967), O-methyltransferase, family 2 (InterPro:IPR001077), O-methyltransferase, COMT, eukaryota (InterPro:IPR016461); BEST Arabidopsis thaliana protein match is: O-methyltransferase family protein (TAIR:AT1G21120.1)
AT4G12450	-1,632		unknown protein; BEST Arabidopsis thaliana protein match is: unknown protein (TAIR:AT4G22560.1)
AT3G10660	-1,633	CALMODULIN-DOMAIN PROTEIN KINASE CDPK ISOFORM 2 (CPK2)	predicted to encode calcium-dependent protein kinase and is localized to the ER. Protein is myristoylated in a cell-free extract. Changing the proposed myristoylated site, G residue in the amino terminal, to A prevented the meristoylation . The G to A mutation decreased AtCPK2 membrane association by approximately 50%.

AT2G38400	*-1,635*	ALANINE:GLYOXYLATE AMINOTRANSFERASE 3 (AGT3)	alanine:glyoxylate aminotransferase 2 homolog (AGT3) mRNA,
AT4G17460	*-1,635*	(HAT1)	Encodes a class II HD-ZIP protein that regulates meristematic activity in different tissues, and that it is necessary for the correct formation of the gynoecium.
AT2G17080	*-1,636*		Arabidopsis protein of unknown function (DUF241); FUNCTIONS IN: molecular_function unknown; INVOLVED IN: biological_process unknown; LOCATED IN: chloroplast; EXPRESSED IN: root; CONTAINS InterPro DOMAIN/s: Protein of unknown function DUF241, plant (InterPro:IPR004320); BEST Arabidopsis thaliana protein match is: Arabidopsis protein of unknown function (DUF241) (TAIR:AT2G17070.1)
AT1G44020	*-1,637*		Cysteine/Histidine-rich C1 domain family protein; CONTAINS InterPro DOMAIN/s: DC1 (InterPro:IPR004146), C1-like (InterPro:IPR011424); BEST Arabidopsis thaliana protein match is: Cysteine/Histidine-rich C1 domain family protein (TAIR:AT1G44030.1)
AT5G01280	*-1,637*	BASIC PROLINE-RICH PROTEIN3 (BPP3)	Encodes a microtubule-associated protein.
AT5G02350	*-1,64*		Cysteine/Histidine-rich C1 domain family protein; FUNCTIONS IN: zinc ion binding; EXPRESSED IN: root; CONTAINS InterPro DOMAIN/s: Zinc finger, RING-type (InterPro:IPR001841), DC1 (InterPro:IPR004146), Zinc finger, PHD-type (InterPro:IPR001965), C1-like (InterPro:IPR011424); BEST Arabidopsis thaliana protein match is: Cysteine/Histidine-rich C1 domain family protein (TAIR:AT2G13900.1)
AT4G11390	*-1,641*		Cysteine/Histidine-rich C1 domain family protein; INVOLVED IN: intracellular signaling pathway; CONTAINS InterPro DOMAIN/s: Protein kinase C-like, phorbol ester/diacylglycerol binding (InterPro:IPR002219), DC1 (InterPro:IPR004146), C1-like (InterPro:IPR011424); BEST Arabidopsis thaliana protein match is: Cysteine/Histidine-rich C1 domain family protein (TAIR:AT4G11550.1)
AT4G12350	*-1,642*	MYB DOMAIN PROTEIN 42 (MYB42)	Encodes a putative transcription factor (MYB42).
AT5G48940	*-1,642*	(RGFR2)	RGFR2 is a leucine--rich repeat receptor kinase that, together with RGFR1 and RGFR3, binds ROOT GROWTH FACTORS and is required for establishing the gradient of PLETHORA1 and PLETHORA2 essential for proper root growth and development.
AT1G29270	*-1,643*		unknown protein; BEST Arabidopsis thaliana protein match is: unknown protein (TAIR:AT2G40435.1)
AT4G34810	*-1,643*	SMALL AUXIN UPREGULATED RNA 5 (SAUR5)	SAUR-like auxin-responsive protein family ; CONTAINS InterPro DOMAIN/s: Auxin responsive SAUR protein (InterPro:IPR003676); BEST Arabidopsis thaliana protein match is: SAUR-like auxin-responsive protein family (TAIR:AT4G34790.1)
AT1G72590	*-1,644*	POLYPRENOL REDUCTASE 1 (PPRD1)	Encodes a polyphenol reductase.
AT4G20110	*-1,646*	VACUOLAR SORTING RECEPTOR 7 (VSR7)	VACUOLAR SORTING RECEPTOR 7 (VSR7); FUNCTIONS IN: calcium ion binding; INVOLVED IN: intracellular protein transport, protein targeting to vacuole; LOCATED IN: trans-Golgi network, integral to plasma membrane, Golgi transport complex; EXPRESSED IN: 17 plant structures; EXPRESSED DURING: 8 growth stages; CONTAINS InterPro DOMAIN/s: Protease-associated PA (InterPro:IPR003137), EGF-like calcium-binding, conserved site (InterPro:IPR018097), EGF-like calcium-binding (InterPro:IPR001881), Growth factor, receptor (InterPro:IPR009030); BEST Arabidopsis thaliana protein match is: VACUOLAR SORTING RECEPTOR 6 (TAIR:AT1G30900.1).
AT4G15400	*-1,649*	BRASSINOSTEROID INACTIVATOR1 (BIA1)	Encodes BIA1, a member of the BAHD acyltransferase family. Plays a role in controlling brassinosteroids levels, particularly in the root and hypocotyl in darkness.
AT1G52240	*-1,65*	ROP (RHO OF PLANTS) GUANINE NUCLEOTIDE EXCHANGE FACTOR 11 (ROPGEF11)	Encodes a member of KPP-like gene family, homolog of KPP (kinase partner protein) gene in tomato. Also a member of the RopGEF (guanine nucleotide exchange factor) family, containing the novel PRONE domain (plant-specific Rop nucleotide exchanger), which is exclusively active towards members of the Rop subfamily .
AT2G21040	*-1,65*		C2 domain-containing protein. Possible pseudogene of AT2G20990.

AT3G20840	*-1,65*	PLETHORA 1 (PLT1)	Encodes a member of the AINTEGUMENTA-like (AIL) subclass of the AP2/EREBP family of transcription factors and is essential for quiescent center (QC) specification and stem cell activity. It is a key effector for establishment of the stem cell niche during embryonic pattern formation. It is transcribed in response to auxin accumulation and is dependent on auxin response transcription factors.
AT2G23960	*-1,651*		Class I glutamine amidotransferase-like superfamily protein; FUNCTIONS IN: catalytic activity; INVOLVED IN: defense response; LOCATED IN: cellular_component unknown; EXPRESSED IN: 7 plant structures; EXPRESSED DURING: LP.04 four leaves visible, F mature embryo stage; CONTAINS InterPro DOMAIN/s: Glutamine amidotransferase class-I, C-terminal (InterPro:IPR000991), Glutamine amidotransferase type 1 (InterPro:IPR017926); BEST Arabidopsis thaliana protein match is: Class I glutamine amidotransferase-like superfamily protein (TAIR:AT4G30530.1)
AT4G28840	*-1,652*	TCP INTERACTOR CONTAINING EAR MOTIF PROTEIN 1 (TIE1)	unknown protein; FUNCTIONS IN: molecular_function unknown; INVOLVED IN: biological_process unknown; LOCATED IN: chloroplast; BEST Arabidopsis thaliana protein match is: unknown protein (TAIR:AT2G20080.1)
AT1G09170	*-1,654*		P-loop nucleoside triphosphate hydrolases superfamily protein with CH (Calponin Homology) domain; FUNCTIONS IN: microtubule motor activity, ATP binding; INVOLVED IN: microtubule-based movement; EXPRESSED IN: shoot, male gametophyte, root, flower, pollen tube; EXPRESSED DURING: L mature pollen stage, M germinated pollen stage, 4 anthesis; CONTAINS InterPro DOMAIN/s: Kinesin, motor region, conserved site (InterPro:IPR019821), Calponin-homology (InterPro:IPR016146), Calponin-like actin-binding (InterPro:IPR001715), Kinesin, motor domain (InterPro:IPR001752); BEST Arabidopsis thaliana protein match is: kinesin 4 (TAIR:AT5G27000.1)
AT4G35200	*-1,658*		Arabidopsis protein of unknown function (DUF241); CONTAINS InterPro DOMAIN/s: Protein of unknown function DUF241, plant (InterPro:IPR004320); BEST Arabidopsis thaliana protein match is: Arabidopsis protein of unknown function (DUF241) (TAIR:AT4G35210.1)
AT5G22310	*-1,658*		unknown protein; BEST Arabidopsis thaliana protein match is: unknown protein (TAIR:AT3G11590.1)
AT5G24040	*-1,658*		Protein of unknown function (DUF295); CONTAINS InterPro DOMAIN/s: Protein of unknown function DUF295 (InterPro:IPR005174); BEST Arabidopsis thaliana protein match is: F-box family protein with a domain of unknown function (DUF295) (TAIR:AT2G16290.1)
AT5G36260	*-1,658*	(A36)	Eukaryotic aspartyl protease family protein; FUNCTIONS IN: aspartic-type endopeptidase activity; INVOLVED IN: proteolysis; LOCATED IN: anchored to membrane; EXPRESSED IN: 7 plant structures; EXPRESSED DURING: L mature pollen stage, M germinated pollen stage, 4 anthesis, petal differentiation and expansion stage; CONTAINS InterPro DOMAIN/s: Peptidase aspartic (InterPro:IPR021109), Peptidase aspartic, catalytic (InterPro:IPR009007), Peptidase A1 (InterPro:IPR001461); BEST Arabidopsis thaliana protein match is: Eukaryotic aspartyl protease family protein (TAIR:AT1G65240.1)
AT5G44690	*-1,658*		unknown protein; BEST Arabidopsis thaliana protein match is: unknown protein (TAIR:AT4G20160.1)
AT1G73620	*-1,659*		Pathogenesis-related thaumatin superfamily protein; INVOLVED IN: response to other organism; LOCATED IN: membrane; EXPRESSED IN: 17 plant structures; EXPRESSED DURING: 9 growth stages; CONTAINS InterPro DOMAIN/s: Thaumatin, conserved site (InterPro:IPR017949), Thaumatin, pathogenesis-related (InterPro:IPR001938); BEST Arabidopsis thaliana protein match is: Pathogenesis-related thaumatin superfamily protein (TAIR:AT1G18250.1)
AT2G35910	*-1,661*		RING/U-box superfamily protein; FUNCTIONS IN: zinc ion binding; CONTAINS InterPro DOMAIN/s: Zinc finger, RING-type (InterPro:IPR001841), Zinc finger, C3HC4 RING-type (InterPro:IPR018957); BEST Arabidopsis thaliana protein match is: RING/U-box superfamily protein (TAIR:AT5G06490.1)
AT3G16440	*-1,662*	MYROSINASE-BINDING PROTEIN-LIKE PROTEIN-300B	myrosinase-binding protein-like protein (AtMLP-300B) mRNA,
AT1G33280	*-1,666*	NAC DOMAIN CONTAINING PROTEIN 15 (NAC015)	NAC domain protein. SMB, BRN1, and BRN2 act to regulate root cap maturation, in a partially redundant fashion.BRN1 and BRN2, control the cell wall maturation processes that are required to detach root cap layers from the root.
AT5G15290	*-1,666*	CASPARIAN STRIP MEMBRANE DOMAIN PROTEIN 5 (CASP5)	Uncharacterised protein family (UPF0497); CONTAINS InterPro DOMAIN/s: Uncharacterised protein family UPF0497, trans-membrane plant (InterPro:IPR006702), Uncharacterised protein family UPF0497, trans-membrane plant subgroup (InterPro:IPR006459); BEST Arabidopsis thaliana protein match is: Uncharacterised protein family (UPF0497) (TAIR:AT1G14160.1)

AT1G21050	*-1,667*		Protein of unknown function, DUF617; CONTAINS InterPro DOMAIN/s: Protein of unknown function DUF617, plant (InterPro:IPR006460); BEST Arabidopsis thaliana protein match is: Protein of unknown function, DUF617 (TAIR:AT1G76610.1)
AT5G41300	*-1,667*		Receptor-like protein kinase-related family protein; CONTAINS InterPro DOMAIN/s: Protein of unknown function DUF26 (InterPro:IPR002902); BEST Arabidopsis thaliana protein match is: Receptor-like protein kinase-related family protein (TAIR:AT5G41290.1)
AT2G38500	*-1,668*		2-oxoglutarate (2OG) and Fe(II)-dependent oxygenase superfamily protein
AT3G48360	*-1,67*	BTB AND TAZ DOMAIN PROTEIN 2 (bt2)	encodes a protein (BT2) that is an essential component of the TAC1-mediated telomerase activation pathway. Acts redundantly with BT3 and BT1 during female gametophyte development and with BT3 during male gametophyte development. BT2 also mediates multiple responses to nutrients, stresses, and hormones.
AT4G01820	*-1,67*	ATP-BINDING CASSETTE B3 (ABCB3)	member of MDR subfamily
AT1G34760	*-1,671*	GENERAL REGULATORY FACTOR 11 (GRF11)	Encodes a 14-3-3 protein. Binds H+-ATPase in response to blue light.
AT5G62310	*-1,671*	INCOMPLETE ROOT HAIR ELONGATION (IRE)	Encodes a protein with a serine/threonine kinase domain. There are two other closely related members in Arabidopsis. Knock-out mutation results in incomplete root hair elongation. Expression is found all organs examined but is especially strong in elongating root hairs.
AT2G41480	*-1,672*	PEROXIDASE 25 (PRX25)	Encodes a cationic cell-wall-bound peroxidase homolog that is involved in the lignification of cell walls.
AT2G34830	*-1,673*	WRKY DNA-BINDING PROTEIN 35 (WRKY35)	member of WRKY Transcription Factor; Group II-e
AT3G09290	*-1,673*	TELOMERASE ACTIVATOR1 (TAC1)	encodes activation factor TAC1 which mediates telomerase activity
AT4G21970	*-1,673*		Protein of unknown function, DUF584; CONTAINS InterPro DOMAIN/s: Protein of unknown function DUF584 (InterPro:IPR007608); BEST Arabidopsis thaliana protein match is: Protein of unknown function, DUF584 (TAIR:AT4G04630.1)
AT1G58340	*-1,675*	(ZF14)	Encodes a plant MATE (multidrug and toxic compound extrusion) transporter that is localized to the Golgi complex and small organelles and is involved in determining the rate of organ initiation. It is also involved in iron homeostasis when plants are under osmotic stress.
AT3G12240	*-1,676*	SERINE CARBOXYPEPTIDASE-LIKE 15 (SCPL15)	serine carboxypeptidase-like 15 (SCPL15); FUNCTIONS IN: serine-type carboxypeptidase activity; INVOLVED IN: proteolysis; LOCATED IN: endomembrane system; CONTAINS InterPro DOMAIN/s: Peptidase S10, serine carboxypeptidase (InterPro:IPR001563); BEST Arabidopsis thaliana protein match is: serine carboxypeptidase-like 14 (TAIR:AT3G12230.1)
AT1G22460	*-1,679*		O-fucosyltransferase family protein; CONTAINS InterPro DOMAIN/s: GDP-fucose protein O-fucosyltransferase (InterPro:IPR019378); BEST Arabidopsis thaliana protein match is: O-fucosyltransferase family protein (TAIR:AT5G64600.1)
AT1G78950	*-1,679*	BETA-AMYRIN SYNTHASE (BAS)	Terpenoid cyclases family protein; CONTAINS InterPro DOMAIN/s: Terpene synthase, conserved site (InterPro:IPR002365), Squalene cyclase (InterPro:IPR018333), Terpenoid cylases/protein prenyltransferase alpha-alpha toroid (InterPro:IPR008930), Prenyltransferase/squalene oxidase (InterPro:IPR001330); BEST Arabidopsis thaliana protein match is: camelliol C synthase 1 (TAIR:AT1G78955.1)
AT2G16230	*-1,679*		O-Glycosyl hydrolases family 17 protein; FUNCTIONS IN: cation binding, hydrolase activity, hydrolyzing O-glycosyl compounds, catalytic activity; INVOLVED IN: carbohydrate metabolic process; LOCATED IN: endomembrane system; EXPRESSED IN: root; CONTAINS InterPro DOMAIN/s: X8 (InterPro:IPR012946), Glycoside hydrolase, catalytic core (InterPro:IPR017853), Glycoside hydrolase, family 17 (InterPro:IPR000490), Glycoside hydrolase, subgroup, catalytic core (InterPro:IPR013781); BEST Arabidopsis thaliana protein match is: O-Glycosyl hydrolases family 17 protein (TAIR:AT4G34480.1)
AT1G58320	*-1,68*		PLAC8 family protein; CONTAINS InterPro DOMAIN/s: Protein of unknown function Cys-rich (InterPro:IPR006461); BEST Arabidopsis thaliana protein match is: PLAC8 family protein (TAIR:AT1G68630.1)
AT2G38640	*-1,68*		Protein of unknown function (DUF567); CONTAINS InterPro DOMAIN/s: Protein of unknown function DUF567 (InterPro:IPR007612); BEST Arabidopsis thaliana protein match is: Protein of unknown function (DUF567) (TAIR:AT5G41590.1)

AT1G63930	*-1,681*	FROM THE CZECH 'ROH' MEANING 'CORNER' (ROH1)	from the Czech 'roh' meaning 'corner' (ROH1); CONTAINS InterPro DOMAIN/s: Protein BYPASS related (InterPro:IPR008511); BEST Arabidopsis thaliana protein match is: Protein of unknown function (DUF793) (TAIR:AT4G23530.1)
AT1G29180	*-1,682*		Cysteine/Histidine-rich C1 domain family protein; CONTAINS InterPro DOMAIN/s: DC1 (InterPro:IPR004146), C1-like (InterPro:IPR011424); BEST Arabidopsis thaliana protein match is: Cysteine/Histidine-rich C1 domain family protein (TAIR:AT5G48320.1)
AT5G01550	*-1,682*	L-TYPE LECTIN RECEPTOR KINASE VI.3 (LECRK-VI.3)	Encodes LecRKA4.2, a member of the lectin receptor kinase subfamily A4 (LecRKA4.1 At5g01540; LecRKA4.2 At5g01550; LecRKA4.3 At5g01560). Together with other members of the subfamily, functions redundantly in the negative regulation of ABA response in seed germination.
AT1G06330	*-1,683*		Heavy metal transport/detoxification superfamily protein ; FUNCTIONS IN: copper ion binding, metal ion binding; INVOLVED IN: copper ion transport, metal ion transport; LOCATED IN: cellular_component unknown; CONTAINS InterPro DOMAIN/s: Heavy metal transport/detoxification protein (InterPro:IPR006121); BEST Arabidopsis thaliana protein match is: Heavy metal transport/detoxification superfamily protein (TAIR:AT2G18196.1)
AT2G16750	*-1,683*		Protein kinase protein with adenine nucleotide alpha hydrolases-like domain; FUNCTIONS IN: protein serine/threonine kinase activity, protein kinase activity, kinase activity, ATP binding; INVOLVED IN: protein amino acid phosphorylation; LOCATED IN: endomembrane system; EXPRESSED IN: 18 plant structures; EXPRESSED DURING: 11 growth stages; CONTAINS InterPro DOMAIN/s: Protein kinase, catalytic domain (InterPro:IPR000719), Serine/threonine-protein kinase-like domain (InterPro:IPR017442), Protein kinase-like domain (InterPro:IPR011009), Serine/threonine-protein kinase, active site (InterPro:IPR008271); BEST Arabidopsis thaliana protein match is: Protein kinase superfamily protein (TAIR:AT4G35030.3)
AT1G29600	*-1,684*		Zinc finger C-x8-C-x5-C-x3-H type family protein; FUNCTIONS IN: zinc ion binding, nucleic acid binding; INVOLVED IN: biological_process unknown; LOCATED IN: cellular_component unknown; EXPRESSED IN: cotyledon, leaf; EXPRESSED DURING: LP.04 four leaves visible; CONTAINS InterPro DOMAIN/s: Zinc finger, CCCH-type (InterPro:IPR000571); BEST Arabidopsis thaliana protein match is: Zinc finger C-x8-C-x5-C-x3-H type family protein (TAIR:AT1G29570.1)
AT5G28960	*-1,686*		unknown protein; FUNCTIONS IN: molecular_function unknown; INVOLVED IN: biological_process unknown; LOCATED IN: endomembrane system; BEST Arabidopsis thaliana protein match is: unknown protein (TAIR:AT5G28910.2)
AT2G14160	*-1,687*		RNA-binding (RRM/RBD/RNP motifs) family protein; FUNCTIONS IN: nucleotide binding, nucleic acid binding; INVOLVED IN: biological_process unknown; LOCATED IN: cellular_component unknown; EXPRESSED IN: root, synergid; CONTAINS InterPro DOMAIN/s: RNA recognition motif, RNP-1 (InterPro:IPR000504), Nucleotide-binding, alpha-beta plait (InterPro:IPR012677); BEST Arabidopsis thaliana protein match is: cold, circadian rhythm, and rna binding 2 (TAIR:AT2G21660.2)
AT5G24100	*-1,691*		Leucine-rich repeat protein kinase family protein; FUNCTIONS IN: protein serine/threonine kinase activity, protein kinase activity, ATP binding; INVOLVED IN: transmembrane receptor protein tyrosine kinase signaling pathway, protein amino acid phosphorylation; LOCATED IN: endomembrane system; EXPRESSED IN: root; CONTAINS InterPro DOMAIN/s: Protein kinase, ATP binding site (InterPro:IPR017441), Protein kinase, catalytic domain (InterPro:IPR000719), Leucine-rich repeat-containing N-terminal domain, type 2 (InterPro:IPR013210), Leucine-rich repeat (InterPro:IPR001611), Serine-threonine/tyrosine-protein kinase (InterPro:IPR001245), Protein kinase-like domain (InterPro:IPR011009); BEST Arabidopsis thaliana protein match is: Leucine-rich repeat protein kinase family protein (TAIR:AT5G53320.1)
AT5G57400	*-1,692*		unknown protein
AT1G54120	*-1,694*		unknown protein; BEST Arabidopsis thaliana protein match is: unknown protein (TAIR:AT3G14060.1)
AT3G51060	*-1,694*	STYLISH 1 (STY1)	A member of SHI gene family. Arabidopsis thaliana has ten members that encode proteins with a RING finger-like zinc finger motif. Despite being highly divergent in sequence, many of the SHI-related genes are partially redundant in function and synergistically promote gynoecium, stamen and leaf development in Arabidopsis. STY1/STY2 double mutants showed defective style, stigma as well as serrated leaves. Binds to the promoter of YUC4 and YUC8 (binding site ACTCTAC)
AT5G67060	*-1,695*	HECATE 1 (HEC1)	Encodes a bHLH transcription factor that is involved in transmitting tract and stigma development and acts as a local modulator of auxin and cytokinin responses to control gynoecium development. HEC1 affects auxin transport by acting as a transcriptional regulator of PIN1 and PIN3.

AT4G25400	*-1,697*		basic helix-loop-helix (bHLH) DNA-binding superfamily protein; FUNCTIONS IN: DNA binding, sequence-specific DNA binding transcription factor activity; INVOLVED IN: regulation of transcription; LOCATED IN: nucleus; CONTAINS InterPro DOMAIN/s: Helix-loop-helix DNA-binding domain (InterPro:IPR001092), Helix-loop-helix DNA-binding (InterPro:IPR011598); BEST Arabidopsis thaliana protein match is: basic helix-loop-helix (bHLH) DNA-binding superfamily protein (TAIR:AT5G51780.1)
AT2G17500	*-1,698*	PIN-LIKES 5 (PILS5)	Auxin efflux carrier family protein; FUNCTIONS IN: auxin:hydrogen symporter activity; INVOLVED IN: auxin polar transport, transmembrane transport; LOCATED IN: endomembrane system, integral to membrane; EXPRESSED IN: 19 plant structures; EXPRESSED DURING: 13 growth stages; CONTAINS InterPro DOMAIN/s: Auxin efflux carrier (InterPro:IPR004776); BEST Arabidopsis thaliana protein match is: Auxin efflux carrier family protein (TAIR:AT5G65980.1)
AT5G03570	*-1,699*	IRON REGULATED 2 (IREG2)	Encodes FPN2, a tonoplast localized nickel transport protein. FPN2 is one of the Arabidopsis orthologs (AT2G38460/IREG1/FPN1 and AT5G03570/IREG2/FPN2) the iron efflux transporter ferroportin (FPN) identified in animals.
AT4G14750	*-1,7*	IQ-DOMAIN 19 (IQD19)	IQ-domain 19 (IQD19); CONTAINS InterPro DOMAIN/s: IQ calmodulin-binding region (InterPro:IPR000048); BEST Arabidopsis thaliana protein match is: IQ-domain 26 (TAIR:AT3G16490.1)
AT2G31540	*-1,701*		GDSL-motif esterase/acyltransferase/lipase. Enzyme group with broad substrate specificity that may catalyze acyltransfer or hydrolase reactions with lipid and non-lipid substrates.
AT1G51840	*-1,702*		protein kinase-related; FUNCTIONS IN: molecular_function unknown; INVOLVED IN: biological_process unknown; LOCATED IN: endomembrane system; BEST Arabidopsis thaliana protein match is: Leucine-rich repeat protein kinase family protein (TAIR:AT1G51850.1)
AT2G28670	*-1,704*	ENHANCED SUBERIN 1 (ESB1)	Encodes a protein of unknown function that is involved in formation of the casparian strip. ESB1 is localized to the casparian strip and this localization is depended on CASP1 and 2. esb1 mutants have increased levels of suberin, disordered casparian strip and altered levels of several ions in their leaves.
AT4G15740	*-1,704*		Calcium-dependent lipid-binding (CaLB domain) family protein; CONTAINS InterPro DOMAIN/s: C2 membrane targeting protein (InterPro:IPR018029), C2 calcium/lipid-binding domain, CaLB (InterPro:IPR008973), C2 calcium-dependent membrane targeting (InterPro:IPR000008); BEST Arabidopsis thaliana protein match is: Calcium-dependent lipid-binding (CaLB domain) family protein (TAIR:AT3G16510.1)
AT1G02440	*-1,706*	ADP-RIBOSYLATION FACTOR D1A (ARFD1A)	A member of ARF GTPase family. A thaliana has 21 members of this family, known to be essential for vesicle coating and uncoating and functions in GTP-binding. Gene encoding ADP-ribosylation factor and similar to other ARFs and ARF-like proteins.
AT1G06080	*-1,706*	DELTA 9 DESATURASE 1 (ADS1)	Encodes a protein homologous to delta 9 acyl-lipid desaturases of cyanobacteria and acyl-CoA desaturases of yeast and mammals. expression down-regulated by cold temperature. It is involved in the desaturation of VLCFAs to make monounsaturated VLCFAs.
AT2G30395	*-1,706*	OVATE FAMILY PROTEIN 17 (OFP17)	Member of the plant specific ovate protein family of unknown function.
AT1G55640	*-1,709*	PRENYLATED RAB ACCEPTOR 1.G1 (PRA1.G1)	prenylated RAB acceptor 1.G1 (PRA1.G1); CONTAINS InterPro DOMAIN/s: Prenylated rab acceptor PRA1 (InterPro:IPR004895); BEST Arabidopsis thaliana protein match is: prenylated RAB acceptor 1.G2 (TAIR:AT5G56230.1)
AT4G24310	*-1,709*		Protein of unknown function (DUF679); CONTAINS InterPro DOMAIN/s: Protein of unknown function DUF679 (InterPro:IPR007770); BEST Arabidopsis thaliana protein match is: Protein of unknown function (DUF679) (TAIR:AT3G02430.1)
AT1G04180	*-1,711*	YUCCA 9 (YUC9)	YUCCA 9 (YUC9); FUNCTIONS IN: NADP or NADPH binding, oxidoreductase activity, monooxygenase activity, FAD binding, flavin-containing monooxygenase activity; INVOLVED IN: oxidation reduction; EXPRESSED IN: root; CONTAINS InterPro DOMAIN/s: Pyridine nucleotide-disulphide oxidoreductase, class-II (InterPro:IPR000103), FAD-dependent pyridine nucleotide-disulphide oxidoreductase (InterPro:IPR013027), Flavin-containing monooxygenase-like (InterPro:IPR020946); BEST Arabidopsis thaliana protein match is: Flavin-binding monooxygenase family protein (TAIR:AT5G43890.1)

AT1G62510	*-1,711*		Bifunctional inhibitor/lipid-transfer protein/seed storage 2S albumin superfamily protein; FUNCTIONS IN: lipid binding; INVOLVED IN: lipid transport; LOCATED IN: endomembrane system; EXPRESSED IN: shoot, cotyledon, leaf whorl, leaf; EXPRESSED DURING: LP.04 four leaves visible, LP.02 two leaves visible; CONTAINS InterPro DOMAIN/s: Bifunctional inhibitor/plant lipid transfer protein/seed storage (InterPro:IPR016140), Plant lipid transfer protein/seed storage/trypsin-alpha amylase inhibitor (InterPro:IPR003612), Plant lipid transfer protein/hydrophobic protein, helical domain (InterPro:IPR013770); BEST Arabidopsis thaliana protein match is: Bifunctional inhibitor/lipid-transfer protein/seed storage 2S albumin superfamily protein (TAIR:AT4G12490.1)
AT1G64920	*-1,712*		UDP-Glycosyltransferase superfamily protein; FUNCTIONS IN: transferase activity, transferring glycosyl groups; INVOLVED IN: metabolic process; LOCATED IN: cellular_component unknown; EXPRESSED IN: root; CONTAINS InterPro DOMAIN/s: UDP-glucuronosyl/UDP-glucosyltransferase (InterPro:IPR002213); BEST Arabidopsis thaliana protein match is: UDP-Glycosyltransferase superfamily protein (TAIR:AT1G64910.1)
AT4G38830	*-1,714*	CYSTEINE-RICH RLK (RECEPTOR-LIKE PROTEIN KINASE) 26 (CRK26)	Encodes a cysteine-rich receptor-like protein kinase.
AT5G03150	*-1,714*	JACKDAW (JKD)	JKD is a nuclear-localized putative transcription factor with three zinc finger domains. jkd mutants show a number of root patterning defects including ectopic periclinal divisions in the cortex, increased cell numbers in the cortical and epidermal layers, a disrupted QC marker expression pattern, and disorganized QC and columella cells. jkd mutants also have a reduced number of meristematic cells in their roots. JKD can interact with the SCR and SHR proteins implicated in root patterning, as well as another zinc finger transcription factor, MAGPIE. All of these interactions require the first zinc finger in JKD according to a Y2H assay. There are also transcriptional interactions among these proteins. The initiation of JKD transcription does not appear to depend on SCR and SHR, but later expression in the post-embryonic QC cells and ground tissue initials is reduced in scr and shr mutants. JKD also appears to be required for SCR transcription beginning in the embryo. There is also some evidence that JKD plays a role in promoting the movement of SHR into the nucleus, particularly in QC cells, but this may be indirect. The mRNA is cell-to-cell mobile.
AT2G17070	*-1,715*		Arabidopsis protein of unknown function (DUF241); CONTAINS InterPro DOMAIN/s: Protein of unknown function DUF241, plant (InterPro:IPR004320); BEST Arabidopsis thaliana protein match is: Arabidopsis protein of unknown function (DUF241) (TAIR:AT2G17080.1)
AT4G00680	*-1,715*	ACTIN DEPOLYMERIZING FACTOR 8 (ADF8)	actin depolymerizing factor 8 (ADF8); FUNCTIONS IN: actin binding; INVOLVED IN: biological_process unknown; LOCATED IN: intracellular; EXPRESSED IN: root, flower; EXPRESSED DURING: petal differentiation and expansion stage; CONTAINS InterPro DOMAIN/s: Actin-binding, cofilin/tropomyosin type (InterPro:IPR002108); BEST Arabidopsis thaliana protein match is: actin depolymerizing factor 11 (TAIR:AT1G01750.1)
AT1G67270	*-1,716*		Zinc-finger domain of monoamine-oxidase A repressor R1 protein; CONTAINS InterPro DOMAIN/s: DDT domain superfamily (InterPro:IPR018501), DDT domain, subgroup (InterPro:IPR018500), Cell division cycle-associated protein (InterPro:IPR018866); BEST Arabidopsis thaliana protein match is: Zinc-finger domain of monoamine-oxidase A repressor R1 protein (TAIR:AT1G67780.1)
AT2G25090	*-1,717*	CBL-INTERACTING PROTEIN KINASE 16 (CIPK16)	Encodes a member of the SNF1-related kinase (SnRK) gene family (SnRK3.18), which has also been reported as a member of the CBL-interacting protein kinases (CIPK16) and is involved in salinity tolerance.
AT2G33460	*-1,718*	ROP-INTERACTIVE CRIB MOTIF-CONTAINING PROTEIN 1 (RIC1)	encodes a member of a novel protein family that contains contain a CRIB (for Cdc42/Rac-interactive binding) motif required for their specific interaction with GTP-bound Rop1 (plant-specific Rho GTPase). Interacts with Rop1 and is involved in pollen tube growth and function. Protein most similar to RIC3(family subgroup III). RIC1 is localized to the apical region of the plasma membrane in pollen tube and mutation analyses indicate that this localization is dependent on ROP1 binding. Gene is expressed in all tissues examined.Analysis of overexpression and loss of function mutants indicates a role in cortical microtubule organization during pavement cell morphogenesis.

AT1G11080	*-1,721*	SERINE CARBOXYPEPTIDASE-LIKE 31 (scpl31)	serine carboxypeptidase-like 31 (scpl31); FUNCTIONS IN: serine-type carboxypeptidase activity; INVOLVED IN: proteolysis; LOCATED IN: endomembrane system; EXPRESSED IN: 17 plant structures; EXPRESSED DURING: 4 anthesis, C globular stage, petal differentiation and expansion stage, E expanded cotyledon stage, D bilateral stage; CONTAINS InterPro DOMAIN/s: Peptidase S10, serine carboxypeptidase (InterPro:IPR001563), Peptidase S10, serine carboxypeptidase, active site (InterPro:IPR018202); BEST Arabidopsis thaliana protein match is: serine carboxypeptidase-like 30 (TAIR:AT4G15100.1).
AT1G75750	*-1,722*	GAST1 PROTEIN HOMOLOG 1 (GASA1)	GA-responsive GAST1 protein homolog regulated by BR and GA antagonistically. Possibly involved in cell elongation based on expression data The mRNA is cell-to-cell mobile.
AT2G27000	*-1,722*	CYTOCHROME P450, FAMILY 705, SUBFAMILY A, POLYPEPTIDE 8 (CYP705A8)	member of CYP705A
AT5G24410	*-1,724*	6-PHOSPHOGLUCONOLACTONASE 4 (PGL4)	Encodes a cytosolic 6-phosphogluconolactonase (PGL) thought to be involved in the oxidative pentose-phosphate pathway (OPPP).
AT1G14540	*-1,725*	PEROXIDASE 4 (PER4)	Peroxidase superfamily protein; FUNCTIONS IN: peroxidase activity, heme binding; INVOLVED IN: response to oxidative stress, oxidation reduction; LOCATED IN: endomembrane system; EXPRESSED IN: leaf apex, sepal, root, stamen; EXPRESSED DURING: 4 anthesis; CONTAINS InterPro DOMAIN/s: Haem peroxidase (InterPro:IPR010255), Plant peroxidase (InterPro:IPR000823), Peroxidases heam-ligand binding site (InterPro:IPR019793), Haem peroxidase, plant/fungal/bacterial (InterPro:IPR002016), Peroxidase, active site (InterPro:IPR019794); BEST Arabidopsis thaliana protein match is: Peroxidase superfamily protein (TAIR:AT1G14550.1)
AT4G03330	*-1,725*	SYNTAXIN OF PLANTS 123 (SYP123)	member of SYP12 Gene Family
AT5G66700	*-1,725*	HOMEOBOX 53 (HB53)	Encodes a homeodomain protein. Member of HD-ZIP 1 family, most closely related to HB5. AtHB53 is auxin-inducible and its induction is inhibited by cytokinin, especially in roots therefore may be involved in root development.
AT5G67450	*-1,729*	ZINC-FINGER PROTEIN 1 (ZF1)	Encodes zinc-finger protein. mRNA levels are elevated in response to low temperature, cold temperatures and high salt. The protein is localized to the nucleus and acts as a transcriptional repressor.
AT1G73340	*-1,731*	ABIETANE DITERPENE OXIDASE 1 (ADTO1)	ADTO1 is required for the activation of systemic acquired resistance.
AT2G27660	*-1,732*		Cysteine/Histidine-rich C1 domain family protein; FUNCTIONS IN: zinc ion binding; LOCATED IN: chloroplast; EXPRESSED IN: 12 plant structures; EXPRESSED DURING: 9 growth stages; CONTAINS InterPro DOMAIN/s: DC1 (InterPro:IPR004146), Zinc finger, ZZ-type (InterPro:IPR000433), C1-like (InterPro:IPR011424); BEST Arabidopsis thaliana protein match is: Cysteine/Histidine-rich C1 domain family protein (TAIR:AT2G44400.1)
AT4G25350	*-1,736*	SHORT HYPOCOTYL UNDER BLUE1 (SHB1)	SHB1 encodes a nuclear and cytosolic protein that has motifs homologous with SYG1 protein family members. Acts in cryptochrome signaling. Overexpression of SHB1 enhanced the expression of PHYTOCHROME-INTERACTING FACTOR4 (PIF4) under red light and promoted proteasome-mediated degradation of phytochrome A and hypocotyl elongation under far-red light. A knockout allele suppressed LONG HYPOCOTYL IN FAR-RED LIGHT1 (HFR1) expression and showed several deetiolation phenotypes. Acts upstream of HFR1. Regulates seed development.
AT1G68825	*-1,738*	ROTUNDIFOLIA LIKE 15 (RTFL15)	ROTUNDIFOLIA like 15 (RTFL15); CONTAINS InterPro DOMAIN/s: DVL (InterPro:IPR012552); BEST Arabidopsis thaliana protein match is: ROTUNDIFOLIA like 17 (TAIR:AT1G13245.1)
AT4G07820	*-1,739*		CAP (Cysteine-rich secretory proteins, Antigen 5, and Pathogenesis-related 1 protein) superfamily protein; FUNCTIONS IN: molecular_function unknown; LOCATED IN: endomembrane system, extracellular region; EXPRESSED IN: stem, hypocotyl, root; CONTAINS InterPro DOMAIN/s: Allergen V5/Tpx-1 related (InterPro:IPR001283), SCP-like extracellular (InterPro:IPR014044); BEST Arabidopsis thaliana protein match is: pathogenesis-related gene 1 (TAIR:AT2G14610.1)

AT5G46040	*-1,74*		Major facilitator superfamily protein; FUNCTIONS IN: transporter activity; INVOLVED IN: oligopeptide transport, response to nematode; LOCATED IN: membrane; EXPRESSED IN: root; CONTAINS InterPro DOMAIN/s: Oligopeptide transporter (InterPro:IPR000109), Major facilitator superfamily, general substrate transporter (InterPro:IPR016196); BEST Arabidopsis thaliana protein match is: peptide transporter 3 (TAIR:AT5G46050.1)
AT1G56680	*-1,745*		Chitinase family protein; FUNCTIONS IN: chitin binding, chitinase activity; INVOLVED IN: carbohydrate metabolic process, cell wall macromolecule catabolic process; LOCATED IN: endomembrane system; EXPRESSED IN: shoot apex, sepal, root, flower; EXPRESSED DURING: petal differentiation and expansion stage; CONTAINS InterPro DOMAIN/s: Glycoside hydrolase, family 19 (InterPro:IPR016283), Chitin-binding, type 1 (InterPro:IPR001002), Glycoside hydrolase, family 19, catalytic (InterPro:IPR000726); BEST Arabidopsis thaliana protein match is: Chitinase family protein (TAIR:AT2G43600.1)
AT3G01760	*-1,745*	LYSINE-HISTIDINE-LIKE TRANSPORTER 6 (LHT6)	Encodes an amino acid transporter expressed in the root that is involved in the uptake of acidic amino acids, glutamine and alanine, and probably phenylalanine.
AT5G46060	*-1,745*		Protein of unknown function, DUF599; FUNCTIONS IN: molecular_function unknown; INVOLVED IN: biological_process unknown; LOCATED IN: endomembrane system; CONTAINS InterPro DOMAIN/s: Protein of unknown function DUF599 (InterPro:IPR006747); BEST Arabidopsis thaliana protein match is: Protein of unknown function, DUF599 (TAIR:AT5G24600.2)
AT1G61080	*-1,748*		Hydroxyproline-rich glycoprotein family protein; FUNCTIONS IN: molecular_function unknown; INVOLVED IN: biological_process unknown; LOCATED IN: chloroplast; EXPRESSED IN: male gametophyte, flower, pollen tube; EXPRESSED DURING: L mature pollen stage, M germinated pollen stage, 4 anthesis; BEST Arabidopsis thaliana protein match is: unknown protein (TAIR:AT1G11070.1)
AT3G29370	*-1,748*	P1R3 (P1R3)	Encodes a atypical member of the bHLH (basic helix-loop-helix) family transcriptional factors.
AT5G44480	*-1,748*	DEFECTIVE UGE IN ROOT (DUR)	mutant has Altered lateral root; UDP Glucose Epimerase The mRNA is cell-to-cell mobile.
AT5G13990	*-1,749*	EXOCYST SUBUNIT EXO70 FAMILY PROTEIN C2 (EXO70C2)	A member of EXO70 gene family, putative exocyst subunits, conserved in land plants. Arabidopsis thaliana contains 23 putative EXO70 genes, which can be classified into eight clusters on the phylogenetic tree.
AT2G29130	*-1,75*	LACCASE 2 (LAC2)	putative laccase, knockout mutant had reduced root elongation under PEG-induced dehydration
AT5G09978	*-1,75*	ELICITOR PEPTIDE 7 PRECURSOR (PEP7)	elicitor peptide 7 precursor (PEP7)
AT4G22810	*-1,751*	AT-HOOK MOTIF NUCLEAR LOCALIZED PROTEIN 24 (AHL24)	Predicted AT-hook DNA-binding family protein; CONTAINS InterPro DOMAIN/s: Protein of unknown function DUF296 (InterPro:IPR005175), Predicted AT-hook DNA-binding (InterPro:IPR014476); BEST Arabidopsis thaliana protein match is: Predicted AT-hook DNA-binding family protein (TAIR:AT4G12050.1)
AT5G11440	*-1,751*	CTC-INTERACTING DOMAIN 5 (CID5)	Interacts with PAB (poly A binding protein) in yeast two hybrid experiments. Contains PAM2 motif, a PABC interacting domain.
AT5G25460	*-1,751*	DUF642 L-GALL RESPONSIVE GENE 2 (DGR2)	Protein of unknown function, DUF642; FUNCTIONS IN: molecular_function unknown; INVOLVED IN: response to karrikin; LOCATED IN: plant-type cell wall; EXPRESSED IN: 22 plant structures; EXPRESSED DURING: 13 growth stages; CONTAINS InterPro DOMAIN/s: Protein of unknown function DUF642 (InterPro:IPR006946); BEST Arabidopsis thaliana protein match is: Protein of unknown function, DUF642 (TAIR:AT5G11420.1)
AT3G51360	*-1,755*		Eukaryotic aspartyl protease family protein; FUNCTIONS IN: aspartic-type endopeptidase activity; INVOLVED IN: proteolysis; LOCATED IN: endomembrane system; EXPRESSED IN: root; CONTAINS InterPro DOMAIN/s: Peptidase aspartic (InterPro:IPR021109), Peptidase aspartic, catalytic (InterPro:IPR009007), Peptidase A1 (InterPro:IPR001461), Peptidase aspartic, active site (InterPro:IPR001969); BEST Arabidopsis thaliana protein match is: Eukaryotic aspartyl protease family protein (TAIR:AT2G17760.1)
AT2G29060	*-1,757*		GRAS family transcription factor; CONTAINS InterPro DOMAIN/s: Transcription factor GRAS (InterPro:IPR005202); BEST Arabidopsis thaliana protein match is: SCARECROW-like 14 (TAIR:AT1G07530.1)
AT4G13620	*-1,76*		encodes a member of the DREB subfamily A-6 of ERF/AP2 transcription factor family. The protein contains one AP2 domain. There are 8 members in this subfamily including RAP2.4. The mRNA is cell-to-cell mobile.
AT5G20470	*-1,76*	HEADLESS DERIVATIVE OF MYOSIN XI-K (HDK)	Encodes a headless derivative of myosin XI-K, which likely arose from a partial duplication of the XI-K gene and is developmentally regulated.

AT1G64910	*-1,762*		UDP-Glycosyltransferase superfamily protein; FUNCTIONS IN: transferase activity, transferring glycosyl groups; INVOLVED IN: metabolic process; EXPRESSED IN: 10 plant structures; EXPRESSED DURING: 7 growth stages; CONTAINS InterPro DOMAIN/s: UDP-glucuronosyl/UDP-glucosyltransferase (InterPro:IPR002213); BEST Arabidopsis thaliana protein match is: UDP-Glycosyltransferase superfamily protein (TAIR:AT5G53990.1)
AT4G02090	*-1,762*		unknown protein; FUNCTIONS IN: molecular_function unknown; INVOLVED IN: N-terminal protein myristoylation; LOCATED IN: cellular_component unknown; EXPRESSED IN: root
AT1G48640	*-1,763*		Transmembrane amino acid transporter family protein; CONTAINS InterPro DOMAIN/s: Amino acid transporter, transmembrane (InterPro:IPR013057); BEST Arabidopsis thaliana protein match is: lysine histidine transporter 1 (TAIR:AT5G40780.1)
AT2G45660	*-1,763*	AGAMOUS-LIKE 20 (AGL20)	Controls flowering and is required for CO to promote flowering. It acts downstream of FT. Overexpression of (SOC1) AGL20 suppresses not only the late flowering of plants that have functional FRI and FLC alleles but also the delayed phase transitions during the vegetative stages of development. AGL20/SOC1 acts with AGL24 to promote flowering and inflorescence meristem identity.AGL20 upregulates expression of AGL24 in response to GA.
AT4G24670	*-1,764*	TRYPTOPHAN AMINOTRANSFERASE RELATED 2 (TAR2)	Encodes a protein with similarity to the TAA1 trytophan aminotransferase involved in IAA biosynthesis. Double mutant analyses suggest that this protein is involved in regulating many aspects of plant growth and development from embryogenesis to flower formation and plays a role in ethylene-mediated signaling.TAR2 is required for reprogramming root architecture in response to low nitrogen conditions.
AT4G15393	*-1,768*	CYTOCHROME P450, FAMILY 702, SUBFAMILY A, POLYPEPTIDE 5 (CYP702A5)	a member of the cytochrome P450 gene family. molecular function unknown.
AT4G26050	*-1,768*	PLANT INTRACELLULAR RAS GROUP-RELATED LRR 8 (PIRL8)	Encodes PIRL8, a member of the Plant Intracellular Ras-group-related LRRs (Leucine rich repeat proteins). PIRLs are a distinct, plant-specific class of intracellular LRRs that likely mediate protein interactions, possibly in the context of signal transduction. The mRNA is cell-to-cell mobile.
AT4G33120	*-1,768*		S-adenosyl-L-methionine-dependent methyltransferases superfamily protein; FUNCTIONS IN: (S)-coclaurine-N-methyltransferase activity; INVOLVED IN: lipid biosynthetic process; CONTAINS InterPro DOMAIN/s: Cyclopropane-fatty-acyl-phospholipid/mycolic acid synthase (InterPro:IPR003333); BEST Arabidopsis thaliana protein match is: S-adenosyl-L-methionine-dependent methyltransferases superfamily protein (TAIR:AT4G33110.2)
AT5G14150	*-1,768*		Protein of unknown function, DUF642; CONTAINS InterPro DOMAIN/s: Protein of unknown function DUF642 (InterPro:IPR006946); BEST Arabidopsis thaliana protein match is: Protein of unknown function, DUF642 (TAIR:AT5G11420.1)
AT1G05260	*-1,769*	RARE COLD INDUCIBLE GENE 3 (RCI3)	Encodes a cold-inducible cationic peroxidase that is involved in the stress response. In response to low temperature, RCI3 transcripts accumulate in the aerial part and in roots of etiolated seedlings but only in roots of light-grown seedlings. The mRNA is cell-to-cell mobile.
AT5G40690	*-1,769*		CONTAINS InterPro DOMAIN/s: EF-Hand 1, calcium-binding site (InterPro:IPR018247); BEST Arabidopsis thaliana protein match is: unknown protein (TAIR:AT2G41730.1)
AT5G59680	*-1,769*		Leucine-rich repeat protein kinase family protein; FUNCTIONS IN: kinase activity; INVOLVED IN: protein amino acid phosphorylation; LOCATED IN: endomembrane system; EXPRESSED IN: 7 plant structures; EXPRESSED DURING: 6 growth stages; CONTAINS InterPro DOMAIN/s: Protein kinase, ATP binding site (InterPro:IPR017441), Protein kinase, catalytic domain (InterPro:IPR000719), Leucine-rich repeat (InterPro:IPR001611), Serine/threonine-protein kinase-like domain (InterPro:IPR017442), Protein kinase-like domain (InterPro:IPR011009), Serine/threonine-protein kinase, active site (InterPro:IPR008271); BEST Arabidopsis thaliana protein match is: Leucine-rich repeat protein kinase family protein (TAIR:AT5G59650.1)
AT1G79860	*-1,77*	ROP (RHO OF PLANTS) GUANINE NUCLEOTIDE EXCHANGE FACTOR 12 (ROPGEF12)	Encodes a member of KPP-like gene family, homolog of KPP (kinase partner protein) gene in tomato. Also a member of the RopGEF (guanine nucleotide exchange factor) family, containing the novel PRONE domain (plant-specific Rop nucleotide exchanger), which is exclusively active towards members of the Rop subfamily. Coexpression of AtPRK2a with AtRopGEF12 resulted in isotropic pollen tube growth Growth.

AT4G40070	-1,771		RING/U-box superfamily protein; FUNCTIONS IN: zinc ion binding; EXPRESSED IN: 9 plant structures; EXPRESSED DURING: 4 anthesis, petal differentiation and expansion stage; CONTAINS InterPro DOMAIN/s: Zinc finger, RING-type (InterPro:IPR001841), Zinc finger, C3HC4 RING-type (InterPro:IPR018957); BEST Arabidopsis thaliana protein match is: RING/U-box superfamily protein (TAIR:AT3G05200.1)
AT5G37210	-1,771		Cysteine/Histidine-rich C1 domain family protein; CONTAINS InterPro DOMAIN/s: DC1 (InterPro:IPR004146), C1-like (InterPro:IPR011424); BEST Arabidopsis thaliana protein match is: Cysteine/Histidine-rich C1 domain family protein (TAIR:AT4G02540.1)
AT5G44460	-1,771	CALMODULIN LIKE 43 (CML43)	calmodulin like 43 (CML43); FUNCTIONS IN: calcium ion binding; INVOLVED IN: response to cadmium ion; EXPRESSED IN: root, cultured cell; CONTAINS InterPro DOMAIN/s: EF-Hand 1, calcium-binding site (InterPro:IPR018247), EF-HAND 2 (InterPro:IPR018249), EF-hand-like domain (InterPro:IPR011992), Calcium-binding EF-hand (InterPro:IPR002048), EF-hand (InterPro:IPR018248); BEST Arabidopsis thaliana protein match is: calmodulin like 42 (TAIR:AT4G20780.1)
AT2G34330	-1,773		unknown protein; BEST Arabidopsis thaliana protein match is: unknown protein (TAIR:AT1G29540.1)
AT4G26560	-1,774	CALCINEURIN B-LIKE PROTEIN 7 (CBL7)	Encodes calcineurin B-like protein 7 (CBL7).
AT5G02580	-1,774		Plant protein 1589 of unknown function; CONTAINS InterPro DOMAIN/s: Conserved hypothetical protein CHP01589, plant (InterPro:IPR006476); BEST Arabidopsis thaliana protein match is: Plant protein 1589 of unknown function (TAIR:AT3G55240.1)
AT3G02430	-1,775		Protein of unknown function (DUF679); CONTAINS InterPro DOMAIN/s: Protein of unknown function DUF679 (InterPro:IPR007770); BEST Arabidopsis thaliana protein match is: Protein of unknown function (DUF679) (TAIR:AT4G24310.1)
AT1G77530	-1,778		O-methyltransferase family protein; CONTAINS InterPro DOMAIN/s: Winged helix-turn-helix transcription repressor DNA-binding (InterPro:IPR011991), Plant methyltransferase dimerisation (InterPro:IPR012967), O-methyltransferase, family 2 (InterPro:IPR001077), O-methyltransferase, COMT, eukaryota (InterPro:IPR016461); BEST Arabidopsis thaliana protein match is: O-methyltransferase family protein (TAIR:AT1G77520.1)
AT1G75710	-1,782		C2H2-like zinc finger protein; FUNCTIONS IN: sequence-specific DNA binding transcription factor activity, zinc ion binding, nucleic acid binding; LOCATED IN: intracellular; EXPRESSED IN: 22 plant structures; EXPRESSED DURING: 13 growth stages; CONTAINS InterPro DOMAIN/s: Zinc finger, C2H2-like (InterPro:IPR015880), Zinc finger, C2H2-type (InterPro:IPR007087); BEST Arabidopsis thaliana protein match is: zinc finger protein-related (TAIR:AT5G54630.1)
AT3G51340	-1,782		Eukaryotic aspartyl protease family protein; FUNCTIONS IN: aspartic-type endopeptidase activity; INVOLVED IN: proteolysis; LOCATED IN: endomembrane system; CONTAINS InterPro DOMAIN/s: Peptidase aspartic, catalytic (InterPro:IPR009007), Peptidase aspartic (InterPro:IPR021109), Peptidase A1 (InterPro:IPR001461), Peptidase aspartic, active site (InterPro:IPR001969); BEST Arabidopsis thaliana protein match is: Eukaryotic aspartyl protease family protein (TAIR:AT3G51350.1)
AT5G45720	-1,782		AAA-type ATPase family protein; FUNCTIONS IN: nucleoside-triphosphatase activity, DNA binding, DNA-directed DNA polymerase activity, nucleotide binding, ATP binding; INVOLVED IN: DNA replication; LOCATED IN: DNA polymerase III complex; CONTAINS InterPro DOMAIN/s: ATPase, AAA+ type, core (InterPro:IPR003593), ATPase, AAA-type, core (InterPro:IPR003959), DNA polymerase III, clamp loader complex, gamma/delta/delta subunit, C-terminal (InterPro:IPR008921), DNA polymerase III, subunit gamma/ tau (InterPro:IPR012763); BEST Arabidopsis thaliana protein match is: AAA-type ATPase family protein (TAIR:AT4G18820.1)
AT1G64480	-1,784	CALCINEURIN B-LIKE PROTEIN 8 (CBL8)	calcineurin B-like protein 8, member of plant-specific family of calcium sensor proteins containing 3 EF-hand motifs
AT4G38410	-1,784		Dehydrin family protein; FUNCTIONS IN: molecular_function unknown; INVOLVED IN: response to water, response to stress; LOCATED IN: cellular_component unknown; EXPRESSED IN: stem, hypocotyl, root; CONTAINS InterPro DOMAIN/s: Dehydrin (InterPro:IPR000167)

AT5G10430	*-1,785*	ARABINOGALACTAN PROTEIN 4 (AGP4)	Encodes arabinogalactan-protein (AGP4) that is expressed in female reproductive tissues. It is involved in promoting degeneration of the persistent synergid after fertilization. In mutant ovules, the persistent synergid does not degrade resulting in polytuby.
AT2G05430	*-1,789*		Ubiquitin-specific protease family C19-related protein; BEST Arabidopsis thaliana protein match is: Ubiquitin-specific protease family C19-related protein (TAIR:AT3G58230.1)
AT1G67330	*-1,79*		Protein of unknown function (DUF579); FUNCTIONS IN: molecular_function unknown; INVOLVED IN: biological_process unknown; LOCATED IN: endomembrane system; EXPRESSED IN: 8 plant structures; EXPRESSED DURING: LP.06 six leaves visible, LP.04 four leaves visible, LP.10 ten leaves visible, 4 leaf senescence stage; CONTAINS InterPro DOMAIN/s: Protein of unknown function DUF579 (InterPro:IPR021148), Conserved hypothetical protein CHP01627 (InterPro:IPR006514); BEST Arabidopsis thaliana protein match is: Protein of unknown function (DUF579) (TAIR:AT1G27930.1)
AT4G23590	*-1,791*		Tyrosine transaminase family protein; FUNCTIONS IN: 1-aminocyclopropane-1-carboxylate synthase activity, pyridoxal phosphate binding, transferase activity, transferring nitrogenous groups, transaminase activity, catalytic activity; INVOLVED IN: cellular amino acid and derivative metabolic process, biosynthetic process; LOCATED IN: cellular_component unknown; EXPRESSED IN: root; CONTAINS InterPro DOMAIN/s: 1-aminocyclopropane-1-carboxylate synthase (InterPro:IPR001176), Pyridoxal phosphate-dependent transferase, major domain (InterPro:IPR015424), Aminotransferase, class I/classII (InterPro:IPR004839), Tyrosine transaminase (InterPro:IPR021178), Tyrosine/nicotianamine aminotransferase (InterPro:IPR005958), Pyridoxal phosphate-dependent transferase, major region, subdomain 1 (InterPro:IPR015421); BEST Arabidopsis thaliana protein match is: Tyrosine transaminase family protein (TAIR:AT4G23600.1)
AT1G27740	*-1,793*	ROOT HAIR DEFECTIVE 6-LIKE 4 (RSL4)	Basic helix-loop-helix (bHLH) transcription factor that is sufficient to promote postmitotic cell growth in root-hair cells. RSL4 is a direct transcriptional target of RHD6
AT1G63560	*-1,793*		Receptor-like protein kinase-related family protein; CONTAINS InterPro DOMAIN/s: Protein of unknown function DUF26 (InterPro:IPR002902); BEST Arabidopsis thaliana protein match is: Receptor-like protein kinase-related family protein (TAIR:AT1G63600.1)
AT5G15830	*-1,794*	BASIC LEUCINE-ZIPPER 3 (bZIP3)	basic leucine-zipper 3 (bZIP3); FUNCTIONS IN: DNA binding, sequence-specific DNA binding transcription factor activity; INVOLVED IN: regulation of transcription, DNA-dependent; EXPRESSED IN: 9 plant structures; EXPRESSED DURING: 6 growth stages; CONTAINS InterPro DOMAIN/s: Basic-leucine zipper (bZIP) transcription factor (InterPro:IPR004827), bZIP transcription factor, bZIP-1 (InterPro:IPR011616); BEST Arabidopsis thaliana protein match is: basic leucine-zipper 42 (TAIR:AT3G30530.1)
AT1G80380	*-1,795*		encodes a glycerate kinase which catalyzes the last step of photorespiration C2 cycle.
AT2G26410	*-1,795*	IQ-DOMAIN 4 (Iqd4)	IQ-domain 4 (Iqd4); CONTAINS InterPro DOMAIN/s: IQ calmodulin-binding region (InterPro:IPR000048); BEST Arabidopsis thaliana protein match is: IQ-domain 2 (TAIR:AT5G03040.3)
AT5G19520	*-1,796*	MECHANOSENSITIVE CHANNEL OF SMALL CONDUCTANCE-LIKE 9 (MSL9)	mechanosensitive channel of small conductance-like 9 (MSL9); CONTAINS InterPro DOMAIN/s: Membrane protein, At2g17000, predicted (InterPro:IPR016688), Mechanosensitive ion channel MscS (InterPro:IPR006685), Like-Sm ribonucleoprotein (LSM)-related domain (InterPro:IPR010920); BEST Arabidopsis thaliana protein match is: mechanosensitive channel of small conductance-like 10 (TAIR:AT5G12080.3)
AT5G60520	*-1,796*		Late embryogenesis abundant (LEA) protein-related; FUNCTIONS IN: molecular_function unknown; LOCATED IN: endomembrane system; EXPRESSED IN: root; CONTAINS InterPro DOMAIN/s: Root cap (InterPro:IPR009646); BEST Arabidopsis thaliana protein match is: late embryogenesis abundant protein-related / LEA protein-related (TAIR:AT5G60530.1)
AT1G57570	*-1,802*		Mannose-binding lectin superfamily protein; CONTAINS InterPro DOMAIN/s: Mannose-binding lectin (InterPro:IPR001229); BEST Arabidopsis thaliana protein match is: Mannose-binding lectin superfamily protein (TAIR:AT1G61230.1)
AT4G10310	*-1,803*	HIGH-AFFINITY K+ TRANSPORTER 1 (HKT1)	encodes a sodium transporter (HKT1) expressed in xylem parenchyma cells. Mutants over-accumulate sodium in shoot tissue and have increased sodium in the xylem sap and reduced sodium in phloem sap and roots.
AT1G74830	*-1,805*		Protein of unknown function, DUF593; FUNCTIONS IN: molecular_function unknown; INVOLVED IN: biological_process unknown; LOCATED IN: endomembrane system; CONTAINS InterPro DOMAIN/s: Protein of unknown function DUF593 (InterPro:IPR007656); BEST Arabidopsis thaliana protein match is: Protein of unknown function, DUF593 (TAIR:AT1G18990.1)

AT3G16360	*-1,807*	HPT PHOSPHOTRANSMITTER 4 (AHP4)	Encodes AHP4, a histidine-containing phosphotransmitter involved in Histidine (His)-to-Aspartate (Asp) phosphorelay signal transduction. AHP4 is one of the six Arabidopsis thaliana histidine phosphotransfer proteins (AHPs). AHPs function as redundant positive regulators of cytokinin signaling. Members of the AHP gene family include: AT3G21510 (AHP1), AT3G29350 (AHP2), AT5G39340 (AHP3), AT3G16360 (AHP4), AT1G03430 (AHP5) and AT1G80100 (AHP6).
AT4G11290	*-1,807*		Peroxidase superfamily protein; FUNCTIONS IN: peroxidase activity, heme binding; INVOLVED IN: response to oxidative stress, oxidation reduction; LOCATED IN: endomembrane system; EXPRESSED IN: 11 plant structures; EXPRESSED DURING: 7 growth stages; CONTAINS InterPro DOMAIN/s: Haem peroxidase (InterPro:IPR010255), Plant peroxidase (InterPro:IPR000823), Peroxidases heam-ligand binding site (InterPro:IPR019793), Peroxidase, active site (InterPro:IPR019794), Haem peroxidase, plant/fungal/bacterial (InterPro:IPR002016); BEST Arabidopsis thaliana protein match is: Peroxidase superfamily protein (TAIR:AT1G05260.1)
AT5G48430	*-1,807*		Eukaryotic aspartyl protease family protein; FUNCTIONS IN: aspartic-type endopeptidase activity; INVOLVED IN: proteolysis; LOCATED IN: endomembrane system; EXPRESSED IN: shoot apex, hypocotyl, root, leaf; CONTAINS InterPro DOMAIN/s: Peptidase aspartic (InterPro:IPR021109), Peptidase aspartic, catalytic (InterPro:IPR009007), Peptidase A1 (InterPro:IPR001461); BEST Arabidopsis thaliana protein match is: Eukaryotic aspartyl protease family protein (TAIR:AT1G03220.1)
AT5G51270	*-1,807*		U-box domain-containing protein kinase family protein; FUNCTIONS IN: ubiquitin-protein ligase activity, protein serine/threonine kinase activity, protein kinase activity, kinase activity, ATP binding; INVOLVED IN: protein amino acid phosphorylation, protein ubiquitination; LOCATED IN: ubiquitin ligase complex; EXPRESSED IN: embryo, flower; EXPRESSED DURING: C globular stage, petal differentiation and expansion stage; CONTAINS InterPro DOMAIN/s: Protein kinase, ATP binding site (InterPro:IPR017441), Protein kinase, catalytic domain (InterPro:IPR000719), U box domain (InterPro:IPR003613), Serine/threonine-protein kinase-like domain (InterPro:IPR017442), Protein kinase-like domain (InterPro:IPR011009), Serine/threonine-protein kinase, active site (InterPro:IPR008271); BEST Arabidopsis thaliana protein match is: U-box domain-containing protein kinase family protein (TAIR:AT4G25160.1)
AT4G39330	*-1,811*	CINNAMYL ALCOHOL DEHYDROGENASE 9 (CAD9)	cinnamyl alcohol dehydrogenase 9 (CAD9); FUNCTIONS IN: oxidoreductase activity, zinc ion binding; INVOLVED IN: oxidation reduction; LOCATED IN: apoplast; EXPRESSED IN: 25 plant structures; EXPRESSED DURING: 15 growth stages; CONTAINS InterPro DOMAIN/s: GroES-like (InterPro:IPR011032), Polyketide synthase, enoylreductase (InterPro:IPR020843), Alcohol dehydrogenase, zinc-containing, conserved site (InterPro:IPR002328), Alcohol dehydrogenase GroES-like (InterPro:IPR013154), Alcohol dehydrogenase, C-terminal (InterPro:IPR013149), Alcohol dehydrogenase superfamily, zinc-containing (InterPro:IPR002085); BEST Arabidopsis thaliana protein match is: cinnamyl alcohol dehydrogenase homolog 2 (TAIR:AT2G21730.1)
AT1G23060	*-1,816*	MICROTUBULE DESTABILIZING PROTEIN 40 (MDP40)	BEST Arabidopsis thaliana protein match is: TPX2 (targeting protein for Xklp2) protein family (TAIR:AT1G70950.1)
AT5G64110	*-1,816*		Peroxidase superfamily protein; FUNCTIONS IN: peroxidase activity, heme binding; INVOLVED IN: response to oxidative stress, oxidation reduction; LOCATED IN: membrane; EXPRESSED IN: shoot apex, shoot, stamen, leaf; EXPRESSED DURING: 4 anthesis; CONTAINS InterPro DOMAIN/s: Haem peroxidase (InterPro:IPR010255), Plant peroxidase (InterPro:IPR000823), Peroxidases heam-ligand binding site (InterPro:IPR019793), Haem peroxidase, plant/fungal/bacterial (InterPro:IPR002016), Peroxidase, active site (InterPro:IPR019794); BEST Arabidopsis thaliana protein match is: Peroxidase superfamily protein (TAIR:AT5G64100.1)
AT3G52910	*-1,817*	GROWTH-REGULATING FACTOR 4 (GRF4)	Growth regulating factor encoding transcription activator. One of the nine members of a GRF gene family, containing nuclear targeting domain. Involved in leaf development and expressed in root, shoot and flower.
AT5G26660	*-1,817*	MYB DOMAIN PROTEIN 86 (MYB86)	myb domain protein 86 (MYB86); CONTAINS InterPro DOMAIN/s: SANT, DNA-binding (InterPro:IPR001005), Homeodomain-like (InterPro:IPR009057), Myb, DNA-binding (InterPro:IPR014778), HTH transcriptional regulator, Myb-type, DNA-binding (InterPro:IPR017930), Homeodomain-related (InterPro:IPR012287), Myb transcription factor (InterPro:IPR015495); BEST Arabidopsis thaliana protein match is: myb domain protein 50 (TAIR:AT1G57560.1)
AT4G38390	*-1,818*	ROOT HAIR SPECIFIC 17 (RHS17)	root hair specific 17 (RHS17); CONTAINS InterPro DOMAIN/s: GDP-fucose protein O-fucosyltransferase (InterPro:IPR019378); BEST Arabidopsis thaliana protein match is: O-fucosyltransferase family protein (TAIR:AT1G20550.1)

AT1G08500	*-1,82*	EARLY NODULIN-LIKE PROTEIN 18 (ENODL18)	early nodulin-like protein 18 (ENODL18); FUNCTIONS IN: electron carrier activity, copper ion binding; LOCATED IN: anchored to membrane; EXPRESSED IN: 14 plant structures; EXPRESSED DURING: LP.06 six leaves visible, 4 anthesis, petal differentiation and expansion stage, LP.08 eight leaves visible, E expanded cotyledon stage; CONTAINS InterPro DOMAIN/s: Plastocyanin-like (InterPro:IPR003245), Cupredoxin (InterPro:IPR008972); BEST Arabidopsis thaliana protein match is: Cupredoxin superfamily protein (TAIR:AT2G31050.1)
AT5G24140	*-1,822*	SQUALENE MONOOXYGENASE 2 (SQP2)	Encodes a protein with similarity to squalene monoxygenases.
AT1G16390	*-1,824*	ORGANIC CATION/CARNITINE TRANSPORTER 3 (OCT3)	organic cation/carnitine transporter 3 (3-Oct); FUNCTIONS IN: carbohydrate transmembrane transporter activity, sugar:hydrogen symporter activity; INVOLVED IN: transport, transmembrane transport; LOCATED IN: integral to membrane, membrane; EXPRESSED IN: 9 plant structures; EXPRESSED DURING: 6 growth stages; CONTAINS InterPro DOMAIN/s: Major facilitator superfamily (InterPro:IPR020846), General substrate transporter (InterPro:IPR005828), Major facilitator superfamily, general substrate transporter (InterPro:IPR016196); BEST Arabidopsis thaliana protein match is: organic cation/carnitine transporter 2 (TAIR:AT1G79360.1)
AT2G19410	*-1,825*		U-box domain-containing protein kinase family protein; FUNCTIONS IN: ubiquitin-protein ligase activity, protein serine/threonine kinase activity, protein kinase activity, kinase activity, ATP binding; INVOLVED IN: protein amino acid phosphorylation, protein ubiquitination; LOCATED IN: ubiquitin ligase complex; CONTAINS InterPro DOMAIN/s: Protein kinase, ATP binding site (InterPro:IPR017441), Protein kinase, catalytic domain (InterPro:IPR000719), U box domain (InterPro:IPR003613), Serine/threonine-protein kinase-like domain (InterPro:IPR017442), Protein kinase-like domain (InterPro:IPR011009), Serine/threonine-protein kinase, active site (InterPro:IPR008271); BEST Arabidopsis thaliana protein match is: U-box domain-containing protein kinase family protein (TAIR:AT5G57035.1)
AT4G01970	*-1,825*	STACHYOSE SYNTHASE (STS)	Encodes a a raffinose and high affinity stachyose synthase as well as a stachyose and Gol specific galactosylhydrolase enzyme activity.AtRS4 is a sequential multifunctional RafS and StaS as well as a high affinity StaS, accepting only Raf and Gol for Sta product formation. AtRS4 possesses a Sta and Gol specific galactosylhydrolase enzyme activity.
AT5G57090	*-1,828*	ETHYLENE INSENSITIVE ROOT 1 (EIR1)	Encodes an auxin efflux carrier that is similar to bacterial membrane transporters. Root-specific role in the transport of auxin. Acts downstream of CTR1 and ethylene biosynthesis, in the same pathway as EIN2 and AUX1, and independent from EIN3 and EIN5/AIN1 pathway. In the root, the protein localizes apically in epidermal and lateral root cap cells and predominantly basally in cortical cells. Functions may be regulated by phosphorylation status. EIR1 expression is induced by brassinolide treatment in the brassinosteroid-insensitive br1 mutant. Gravistimulation resulted in asymmetric PIN2 distribution, with more protein degraded at the upper side of the gravistimulated root. Protein turnover is affected by the proteasome and by endosomal cycling. Plasma membrane-localized PIN proteins mediate a saturable efflux of auxin. PINs mediate auxin efflux from mammalian and yeast cells without needing additional plant-specific factors. The action of PINs in auxin efflux is distinct from PGPs, rate-limiting, specific to auxins and sensitive to auxin transport inhibitors. Membrane sterol composition is essential for the acquisition of PIN2 polarity.
AT1G05990	*-1,83*		EF hand calcium-binding protein family; FUNCTIONS IN: calcium ion binding; LOCATED IN: cellular_component unknown; EXPRESSED IN: root hair; CONTAINS InterPro DOMAIN/s: Parvalbumin (InterPro:IPR008080), EF-Hand 1, calcium-binding site (InterPro:IPR018247), EF-HAND 2 (InterPro:IPR018249), EF-hand-like domain (InterPro:IPR011992), Calcium-binding EF-hand (InterPro:IPR002048), EF-hand (InterPro:IPR018248); BEST Arabidopsis thaliana protein match is: Calcium-binding EF-hand family protein (TAIR:AT2G43290.1)
AT4G20070	*-1,83*	ALLANTOATE AMIDOHYDROLASE (AAH)	The gene encoding Arabidopsis thaliana Allantoate Amidohydrolase (AtAAH)which catalyzes the allantoate deiminase reaction (EC 3.5.3.9)is expressed in all parts of the plant being consistent with a function in purine turnover in Arabidopsis. The mRNA is cell-to-cell mobile.
AT5G24105	*-1,831*	ARABINOGALACTAN PROTEIN 41 (AGP41)	Encodes a putative arabinogalactan-protein (AGP41).

AT2G37700	-1,832		Fatty acid hydroxylase superfamily; FUNCTIONS IN: oxidoreductase activity, iron ion binding, catalytic activity; INVOLVED IN: oxidation reduction, fatty acid biosynthetic process; LOCATED IN: endoplasmic reticulum; CONTAINS InterPro DOMAIN/s: Fatty acid hydroxylase (InterPro:IPR006694), Uncharacterised protein, Wax2 C-terminal (InterPro:IPR021940); BEST Arabidopsis thaliana protein match is: Fatty acid hydroxylase superfamily (TAIR:AT1G02190.1)
AT1G64780	-1,833	AMMONIUM TRANSPORTER 1;2 (AMT1;2)	encodes an ammonium transporter protein believed to act as a high affinity transporter. It is expressed in the root, primarily in endodermal and cortical cells, and contributes to ammonium uptake in the root.
AT2G28470	-1,834	BETA-GALACTOSIDASE 8 (BGAL8)	putative beta-galactosidase (BGAL8 gene)
AT4G09990	-1,834	GLUCURONOXYLAN METHYLTRANSFERASE 2 (GXM2)	Protein of unknown function (DUF579); FUNCTIONS IN: molecular_function unknown; INVOLVED IN: biological_process unknown; LOCATED IN: endomembrane system; EXPRESSED IN: 9 plant structures; EXPRESSED DURING: 4 anthesis, LP.10 ten leaves visible, petal differentiation and expansion stage; CONTAINS InterPro DOMAIN/s: Protein of unknown function DUF579 (InterPro:IPR021148), Conserved hypothetical protein CHP01627 (InterPro:IPR006514); BEST Arabidopsis thaliana protein match is: Protein of unknown function (DUF579) (TAIR:AT1G33800.1)
AT1G24420	-1,835		HXXXD-type acyl-transferase family protein; FUNCTIONS IN: transferase activity, transferring acyl groups other than amino-acyl groups, transferase activity; INVOLVED IN: biological_process unknown; LOCATED IN: cellular_component unknown; EXPRESSED IN: hypocotyl, root; CONTAINS InterPro DOMAIN/s: Transferase (InterPro:IPR003480); BEST Arabidopsis thaliana protein match is: HXXXD-type acyl-transferase family protein (TAIR:AT4G15390.1)
AT5G21080	-1,835		Uncharacterized protein; BEST Arabidopsis thaliana protein match is: Uncharacterized protein (TAIR:AT2G41830.1)
AT2G24400	-1,836	SMALL AUXIN UPREGULATED RNA 38 (SAUR38)	SAUR-like auxin-responsive protein family ; CONTAINS InterPro DOMAIN/s: Auxin responsive SAUR protein (InterPro:IPR003676); BEST Arabidopsis thaliana protein match is: SAUR-like auxin-responsive protein family (TAIR:AT4G31320.1)
AT3G48920	-1,836	MYB DOMAIN PROTEIN 45 (MYB45)	Member of the R2R3 factor gene family.
AT5G17960	-1,836		Encodes a member of a Cys-rich protein family known as C1-clan proteins, that contains C1_2, C1_3 and ZZ/PHD type C1 domains. Its expression is responsive to phytohormones and is affected by biotic (chitin) and different abiotic (salinity, drought, cold and UV) treatments.
AT1G03870	-1,838	FASCICLIN-LIKE ARABINOOGALACTAN 9 (FLA9)	fasciclin-like arabinogalactan-protein 9 (Fla9)
AT2G32300	-1,845	UCLACYANIN 1 (UCC1)	Encodes a uclacyanin, a protein precursor that is closely related to precursors of stellacyanins and a blue copper protein from pea pods.
AT5G51470	-1,845		Auxin-responsive GH3 family protein; CONTAINS InterPro DOMAIN/s: GH3 auxin-responsive promoter (InterPro:IPR004993); BEST Arabidopsis thaliana protein match is: Auxin-responsive GH3 family protein (TAIR:AT1G23160.1)
AT5G26070	-1,846		Encodes a protein with 23.5% proline residues and proline-rich extensin domains, INTERPRO:IPR002965; similar to root nodule extensin (Pisum sativum) gi:15021750/gb:AAK77902; Common family members: At5g19800, At5g57070, At1g72790 (Arabidopsis thaliana)
AT1G17300	-1,847		unknown protein; BEST Arabidopsis thaliana protein match is: unknown protein (TAIR:AT1G17285.1)
AT1G22330	-1,847		RNA-binding (RRM/RBD/RNP motifs) family protein; FUNCTIONS IN: RNA binding, nucleotide binding, nucleic acid binding; INVOLVED IN: biological_process unknown; LOCATED IN: cellular_component unknown; EXPRESSED IN: 20 plant structures; EXPRESSED DURING: 13 growth stages; CONTAINS InterPro DOMAIN/s: RNA recognition motif, RNP-1 (InterPro:IPR000504), Nucleotide-binding, alpha-beta plait (InterPro:IPR012677); BEST Arabidopsis thaliana protein match is: RNA-binding (RRM/RBD/RNP motifs) family protein (TAIR:AT1G78260.1)
AT4G30450	-1,848		glycine-rich protein
AT1G06310	-1,85	ACYL-COA OXIDASE 6 (ACX6)	Encodes a putative acyl-CoA oxidase. However, no transcripts have been detected for this gene and no altered phenotypes have been detected in plants mutant for this gene. This suggests that ACX6 does not significantly contribute to seedling beta-oxidation of fatty acids or indole-3-butyric acid in vivo.

AT1G79250	*-1,85*	AGC KINASE 1.7 (AGC1.7)	AGC kinase 1.7 (AGC1.7); CONTAINS InterPro DOMAIN/s: Protein kinase, catalytic domain (InterPro:IPR000719), Serine/threonine-protein kinase domain (InterPro:IPR002290), Serine/threonine-protein kinase-like domain (InterPro:IPR017442), Protein kinase-like domain (InterPro:IPR011009), Serine/threonine-protein kinase, active site (InterPro:IPR008271); BEST Arabidopsis thaliana protein match is: root hair specific 3 (TAIR:AT1G16440.1)
AT3G51350	*-1,85*		Eukaryotic aspartyl protease family protein; FUNCTIONS IN: aspartic-type endopeptidase activity; INVOLVED IN: proteolysis; LOCATED IN: anchored to membrane; CONTAINS InterPro DOMAIN/s: Peptidase aspartic, catalytic (InterPro:IPR009007), Peptidase aspartic (InterPro:IPR021109), Peptidase A1 (InterPro:IPR001461), Peptidase aspartic, active site (InterPro:IPR001969); BEST Arabidopsis thaliana protein match is: Eukaryotic aspartyl protease family protein (TAIR:AT3G51330.1)
AT1G50720	*-1,851*		Stigma-specific Stig1 family protein; FUNCTIONS IN: molecular_function unknown; INVOLVED IN: biological_process unknown; LOCATED IN: endomembrane system; CONTAINS InterPro DOMAIN/s: Stigma-specific protein Stig1 (InterPro:IPR006969); BEST Arabidopsis thaliana protein match is: Stigma-specific Stig1 family protein (TAIR:AT5G55110.1)
AT2G32620	*-1,851*	CELLULOSE SYNTHASE-LIKE B (CSLB02)	encodes a gene similar to cellulose synthase
AT3G03280	*-1,851*		unknown protein; FUNCTIONS IN: molecular_function unknown; INVOLVED IN: N-terminal protein myristoylation; LOCATED IN: cellular_component unknown; BEST Arabidopsis thaliana protein match is: unknown protein (TAIR:AT5G17350.1)
AT4G09110	*-1,851*		RING/U-box superfamily protein; FUNCTIONS IN: zinc ion binding; LOCATED IN: endomembrane system; CONTAINS InterPro DOMAIN/s: Zinc finger, RING-type (InterPro:IPR001841), Zinc finger, C3HC4 RING-type (InterPro:IPR018957); BEST Arabidopsis thaliana protein match is: RING/U-box superfamily protein (TAIR:AT4G09120.1)
AT1G79320	*-1,852*	METACASPASE 6 (MC6)	Encodes a putative metacaspase. Arabidopsis contains three type I MCP genes (MCP1a-c) and six type II MCP genes (MCP2a?f): AtMCP1a/At5g64240, AtMCP1b/At1g02170, AtMCP1c/At4g25110, AtMCP2a/At1g79310, AtMCP2b/At1g79330, AtMCP2c/At1g79320, AtMCP2d/At1g79340, AtMCP2e/At1g16420, AtMCP2f/At5g04200.
AT1G31885	*-1,853*	NOD26-LIKE INTRINSIC PROTEIN 3;1 (NIP3;1)	NOD26-like intrinsic protein 3;1 (NIP3;1); FUNCTIONS IN: transporter activity; INVOLVED IN: transport, transmembrane transport; LOCATED IN: integral to membrane, membrane; CONTAINS InterPro DOMAIN/s: Major intrinsic protein, conserved site (InterPro:IPR022357), Aquaporin (InterPro:IPR012269), Major intrinsic protein (InterPro:IPR000425); BEST Arabidopsis thaliana protein match is: NOD26-like intrinsic protein 1;2 (TAIR:AT4G18910.1)
AT2G25980	*-1,854*		Mannose-binding lectin superfamily protein; CONTAINS InterPro DOMAIN/s: Mannose-binding lectin (InterPro:IPR001229); BEST Arabidopsis thaliana protein match is: jacalin lectin family protein (TAIR:AT1G33790.1)
AT2G31085	*-1,857*	CLAVATA3/ESR-RELATED 6 (CLE6)	Member of a large family of putative ligands homologous to the Clavata3 gene. Consists of a single exon. Can replace CLV3 function in vivo.
AT2G47360	*-1,858*		unknown protein; BEST Arabidopsis thaliana protein match is: unknown protein (TAIR:AT1G02570.1)
AT1G44050	*-1,859*		Cysteine/Histidine-rich C1 domain family protein; FUNCTIONS IN: zinc ion binding; INVOLVED IN: intracellular signaling pathway; LOCATED IN: cellular_component unknown; EXPRESSED IN: root; CONTAINS InterPro DOMAIN/s: Protein kinase C-like, phorbol ester/diacylglycerol binding (InterPro:IPR002219), DC1 (InterPro:IPR004146), Zinc finger, PHD-type (InterPro:IPR001965), C1-like (InterPro:IPR011424); BEST Arabidopsis thaliana protein match is: Cysteine/Histidine-rich C1 domain family protein (TAIR:AT5G55780.1)
AT4G25190	*-1,863*	QWRF DOMAIN CONTAINING 7 (QWRF7)	Family of unknown function (DUF566) ; INVOLVED IN: biological_process unknown; LOCATED IN: chloroplast; CONTAINS InterPro DOMAIN/s: Protein of unknown function DUF566 (InterPro:IPR007573); BEST Arabidopsis thaliana protein match is: Family of unknown function (DUF566) (TAIR:AT4G30710.1)
AT2G47140	*-1,864*	SHORT-CHAIN DEHYDROGENASE REDUCTASE 5 (SDR5)	NAD(P)-binding Rossmann-fold superfamily protein; FUNCTIONS IN: oxidoreductase activity, binding, catalytic activity; INVOLVED IN: oxidation reduction, metabolic process; LOCATED IN: cellular_component unknown; EXPRESSED IN: 6 plant structures; EXPRESSED DURING: 4 anthesis, LP.10 ten leaves visible, petal differentiation and expansion stage, LP.08 eight leaves visible, LP.12 twelve leaves visible; CONTAINS InterPro DOMAIN/s: NAD(P)-binding domain (InterPro:IPR016040), Glucose/ribitol dehydrogenase (InterPro:IPR002347), Short-chain dehydrogenase/reductase SDR (InterPro:IPR002198); BEST Arabidopsis thaliana protein match is: NAD(P)-binding Rossmann-fold superfamily protein (TAIR:AT2G47130.1)

AT5G39000	-1,873		Malectin/receptor-like protein kinase family protein; FUNCTIONS IN: kinase activity; INVOLVED IN: protein amino acid phosphorylation; LOCATED IN: endomembrane system; CONTAINS InterPro DOMAIN/s: Protein kinase, ATP binding site (InterPro:IPR017441), Malectin/receptor-like protein kinase (InterPro:IPR021720), Protein kinase, catalytic domain (InterPro:IPR000719), Serine-threonine/tyrosine-protein kinase (InterPro:IPR001245), Protein kinase-like domain (InterPro:IPR011009), Serine/threonine-protein kinase, active site (InterPro:IPR008271); BEST Arabidopsis thaliana protein match is: Malectin/receptor-like protein kinase family protein (TAIR:AT5G38990.1)
AT3G50870	-1,874	MONOPOLE (MNP)	Encodes a GATA transcriptional regulator required to position the proembryo boundary in the early embryo. Regulates shoot apical meristem and flower development.
AT1G30850	-1,875	ROOT HAIR SPECIFIC 4 (RSH4)	root hair specific 4 (RSH4); BEST Arabidopsis thaliana protein match is: unknown protein (TAIR:AT2G34910.1)
AT2G35890	-1,875	CALCIUM-DEPENDENT PROTEIN KINASE 25 (CPK25)	member of Calcium Dependent Protein Kinase
AT2G42250	-1,875	CYTOCHROME P450, FAMILY 712, SUBFAMILY A, POLYPEPTIDE 1 (CYP712A1)	member of CYP712A
AT1G30220	-1,876	INOSITOL TRANSPORTER 2 (INT2)	Inositol transporter presenting conserved extracellular loop domains homologs of plexins/semaphorin/integrin (PSI) domains from animal type I receptors.
AT3G47720	-1,879	SIMILAR TO RCD ONE 4 (SRO4)	Encodes a protein with similarity to RCD1 but without the WWE domain. The protein does have a PARP signature upstream of the C-terminal protein interaction domain. The PARP signature may bind NAD+ and attach the ADP-ribose-moiety from NAD+ to the target molecule. Its presence suggests a role for the protein in ADP ribosylation.
AT1G13310	-1,882		Endosomal targeting BRO1-like domain-containing protein; CONTAINS InterPro DOMAIN/s: BRO1 (InterPro:IPR004328)
AT4G04450	-1,882	(WRKY42)	member of WRKY Transcription Factor; Group II-b
AT3G05140	-1,883	ROP BINDING PROTEIN KINASES 2 (RBK2)	ROP binding protein kinases 2 (RBK2); FUNCTIONS IN: protein serine/threonine kinase activity, protein kinase activity, kinase activity, ATP binding; INVOLVED IN: protein amino acid phosphorylation; LOCATED IN: nucleus, cytoplasm; EXPRESSED IN: 8 plant structures; EXPRESSED DURING: L mature pollen stage, M germinated pollen stage, 4 anthesis; CONTAINS InterPro DOMAIN/s: Protein kinase, ATP binding site (InterPro:IPR017441), Protein kinase, catalytic domain (InterPro:IPR000719), Serine-threonine/tyrosine-protein kinase (InterPro:IPR001245), Protein kinase-like domain (InterPro:IPR011009), Serine/threonine-protein kinase, active site (InterPro:IPR008271); BEST Arabidopsis thaliana protein match is: Protein kinase superfamily protein (TAIR:AT5G18910.1)
AT1G63410	-1,885		Protein of unknown function (DUF567); CONTAINS InterPro DOMAIN/s: Protein of unknown function DUF567 (InterPro:IPR007612); BEST Arabidopsis thaliana protein match is: Protein of unknown function (DUF567) (TAIR:AT3G14260.1)
AT1G56630	-1,887		alpha/beta-Hydrolases superfamily protein; FUNCTIONS IN: triglyceride lipase activity; INVOLVED IN: lipid metabolic process; LOCATED IN: cellular_component unknown; CONTAINS InterPro DOMAIN/s: Lipase, class 3 (InterPro:IPR002921); BEST Arabidopsis thaliana protein match is: alpha/beta-Hydrolases superfamily protein (TAIR:AT5G42930.1)
AT1G73300	-1,89	SERINE CARBOXYPEPTIDASE-LIKE 2 (scpl2)	serine carboxypeptidase-like 2 (scpl2); FUNCTIONS IN: serine-type carboxypeptidase activity; INVOLVED IN: proteolysis; LOCATED IN: endomembrane system; CONTAINS InterPro DOMAIN/s: Peptidase S10, serine carboxypeptidase (InterPro:IPR001563); BEST Arabidopsis thaliana protein match is: serine carboxypeptidase-like 1 (TAIR:AT5G36180.1)
AT5G26320	-1,89		TRAF-like family protein; FUNCTIONS IN: molecular_function unknown; INVOLVED IN: biological_process unknown; LOCATED IN: endomembrane system; CONTAINS InterPro DOMAIN/s: TRAF-like (InterPro:IPR008974), MATH (InterPro:IPR002083); BEST Arabidopsis thaliana protein match is: TRAF-like family protein (TAIR:AT5G26280.1)
AT1G33750	-1,891	TERPENE SYNTHASE 22 (TPS22)	Terpenoid cyclases/Protein prenyltransferases superfamily protein; FUNCTIONS IN: lyase activity, magnesium ion binding; INVOLVED IN: metabolic process; EXPRESSED IN: root; CONTAINS InterPro DOMAIN/s: Terpene synthase, metal-binding domain (InterPro:IPR005630), Terpenoid synthase (InterPro:IPR008949), Terpenoid cylases/protein prenyltransferase alpha-alpha toroid (InterPro:IPR008930), Terpene synthase-like (InterPro:IPR001906); BEST Arabidopsis thaliana protein match is: Terpenoid cyclases/Protein prenyltransferases superfamily protein (TAIR:AT3G14490.1)

AT2G35380	*-1,891*		Peroxidase superfamily protein; FUNCTIONS IN: peroxidase activity, heme binding; INVOLVED IN: oxidation reduction, response to oxidative stress; LOCATED IN: endomembrane system; EXPRESSED IN: hypocotyl, root; CONTAINS InterPro DOMAIN/s: Haem peroxidase (InterPro:IPR010255), Plant peroxidase (InterPro:IPR000823), Peroxidases heam-ligand binding site (InterPro:IPR019793), Haem peroxidase, plant/fungal/bacterial (InterPro:IPR002016), Peroxidase, active site (InterPro:IPR019794); BEST Arabidopsis thaliana protein match is: Peroxidase superfamily protein (TAIR:AT2G18150.1)
AT2G28970	*-1,893*		Leucine-rich repeat protein kinase family protein; FUNCTIONS IN: protein serine/threonine kinase activity, kinase activity, ATP binding; INVOLVED IN: transmembrane receptor protein tyrosine kinase signaling pathway, protein amino acid phosphorylation; LOCATED IN: endomembrane system; EXPRESSED IN: root; CONTAINS InterPro DOMAIN/s: Protein kinase, ATP binding site (InterPro:IPR017441), Protein kinase, catalytic domain (InterPro:IPR000719), Leucine-rich repeat (InterPro:IPR001611), Serine/threonine-protein kinase-like domain (InterPro:IPR017442), Protein kinase-like domain (InterPro:IPR011009), Serine/threonine-protein kinase, active site (InterPro:IPR008271); BEST Arabidopsis thaliana protein match is: Leucine-rich repeat protein kinase family protein (TAIR:AT2G28990.1)
AT1G51860	*-1,895*		Leucine-rich repeat protein kinase family protein; FUNCTIONS IN: kinase activity; INVOLVED IN: protein amino acid phosphorylation; LOCATED IN: endomembrane system; EXPRESSED IN: stem, root, stamen; EXPRESSED DURING: 4 anthesis; CONTAINS InterPro DOMAIN/s: Protein kinase, ATP binding site (InterPro:IPR017441), Protein kinase, catalytic domain (InterPro:IPR000719), Leucine-rich repeat (InterPro:IPR001611), Serine-threonine/tyrosine-protein kinase (InterPro:IPR001245), Protein kinase-like domain (InterPro:IPR011009), Serine/threonine-protein kinase, active site (InterPro:IPR008271); BEST Arabidopsis thaliana protein match is: root hair specific 6 (TAIR:AT1G51880.1)
AT4G04900	*-1,896*	ROP-INTERACTIVE CRIB MOTIF-CONTAINING PROTEIN 10 (RIC10)	encodes a member of a novel protein family that contains contain a CRIB (for Cdc42/Rac-interactive binding) motif required for their specific interaction with GTP-bound Rop1 (plant-specific Rho GTPase). Most similar to RIC9 and RIC11 (subfamily group I). Gene is expressed predominantly in roots, leaves, and seedlings.
AT4G25560	*-1,897*	LONG AFTER FAR-RED LIGHT 1 (LAF1)	LAF1 is a R2R3-MYB transcription factor and positive regulator of the phyA photoresponse. Interaction of LAF1 with HFR1 stabilize the proteins against ubiquitination by COP1(AT2G32950) and subsequent degrations. Mutants have an elongated hypocotyl specifically under far-red light but retain wild-type responses to other light wavelengths.
AT1G08670	*-1,9*		ENTH/VHS family protein; CONTAINS InterPro DOMAIN/s: Epsin, N-terminal (InterPro:IPR001026), Epsin-like, N-terminal (InterPro:IPR013809), ENTH/VHS (InterPro:IPR008942); BEST Arabidopsis thaliana protein match is: ENTH/VHS family protein (TAIR:AT3G46540.1)
AT1G60130	*-1,9*		Mannose-binding lectin superfamily protein; CONTAINS InterPro DOMAIN/s: Mannose-binding lectin (InterPro:IPR001229); BEST Arabidopsis thaliana protein match is: Mannose-binding lectin superfamily protein (TAIR:AT1G60110.1)
AT2G37100	*-1,903*		protamine P1 family protein; FUNCTIONS IN: DNA binding; INVOLVED IN: chromosome organization; LOCATED IN: chloroplast; BEST Arabidopsis thaliana protein match is: unknown protein (TAIR:AT5G03110.1)
AT4G15300	*-1,903*	CYTOCHROME P450, FAMILY 702, SUBFAMILY A, POLYPEPTIDE 2 (CYP702A2)	a member of the cytochrome P450 gene family. molecular function unknown.
AT1G52790	*-1,906*		encodes a putative oxidoreductase, 2OG-Fe(II) oxygenase family protein, similar to GS-AOP loci (GI:16118889, GI:16118887, GI:16118891, GI:16118893); contains PF03171 2OG-Fe(II) oxygenase superfamily domain
AT1G26945	*-1,909*	KIDARI (KDR)	Encodes a basic helix-loop-helix (bHLH) protein involved in blue/far-red light signaling. Physically interacts with HFR1 and negatively regulates its activity.
AT1G53680	*-1,909*	GLUTATHIONE S-TRANSFERASE TAU 28	Encodes glutathione transferase belonging to the tau class of GSTs. Naming convention according to Wagner et al. (2002).

AT3G60330	*-1,909*	H(+)-ATPASE 7 (HA7)	H(+)-ATPase 7 (HA7); FUNCTIONS IN: hydrogen-exporting ATPase activity, phosphorylative mechanism; INVOLVED IN: cation transport, metabolic process, ATP biosynthetic process; LOCATED IN: plasma membrane; EXPRESSED IN: 7 plant structures; EXPRESSED DURING: L mature pollen stage, M germinated pollen stage, 4 anthesis, petal differentiation and expansion stage; CONTAINS InterPro DOMAIN/s: ATPase, P-type, ATPase-associated domain (InterPro:IPR008250), ATPase, P-type cation-transporter, N-terminal (InterPro:IPR004014), Haloacid dehalogenase-like hydrolase (InterPro:IPR005834), ATPase, P-type, H+ transporting proton pump (InterPro:IPR000695), ATPase, P-type, K/Mg/Cd/Cu/Zn/Na/Ca/Na/H-transporter (InterPro:IPR001757), ATPase, P-type, plasma-membrane proton-efflux (InterPro:IPR006534), ATPase, P-type phosphorylation site (InterPro:IPR018303); BEST Arabidopsis thaliana protein match is: H(+)-ATPase 11 (TAIR:AT5G62670.1)
AT1G12740	*-1,91*	CYTOCHROME P450, FAMILY 87, SUBFAMILY A, POLYPEPTIDE 2 (CYP87A2)	encodes a protein with cytochrome P450 domain
AT1G53940	*-1,91*	GDSL-MOTIF LIPASE 2 (GLIP2)	Encodes a lipase, has in vitro lipase activity with p-nitrophenyl acetate and p-nitrophenyl butyrate, gene expression induced by hormones, negatively regulates auxin signaling, involved in disease resistance
AT2G43480	*-1,91*		Peroxidase superfamily protein; FUNCTIONS IN: peroxidase activity, heme binding; INVOLVED IN: oxidation reduction, response to oxidative stress; LOCATED IN: endomembrane system; EXPRESSED IN: root, flower; EXPRESSED DURING: petal differentiation and expansion stage; CONTAINS InterPro DOMAIN/s: Haem peroxidase (InterPro:IPR010255), Plant peroxidase (InterPro:IPR000823), Peroxidases heam-ligand binding site (InterPro:IPR019793), Haem peroxidase, plant/fungal/bacterial (InterPro:IPR002016); BEST Arabidopsis thaliana protein match is: Peroxidase superfamily protein (TAIR:AT5G24070.1)
AT4G11780	*-1,911*	TON1 RECRUITING MOTIF 10 (TRM10)	unknown protein; BEST Arabidopsis thaliana protein match is: unknown protein (TAIR:AT4G23020.2)
AT1G75030	*-1,912*	THAUMATIN-LIKE PROTEIN 3 (TLP-3)	encodes a PR5-like protein
AT1G03010	*-1,913*		Phototropic-responsive NPH3 family protein; FUNCTIONS IN: signal transducer activity; INVOLVED IN: response to light stimulus, pollen tube growth; EXPRESSED IN: leaf apex, inflorescence meristem, cauline leaf, root, flower; EXPRESSED DURING: M germinated pollen stage, petal differentiation and expansion stage; CONTAINS InterPro DOMAIN/s: NPH3 (InterPro:IPR004249), BTB/POZ fold (InterPro:IPR011333), BTB/POZ-like (InterPro:IPR000210); BEST Arabidopsis thaliana protein match is: Phototropic-responsive NPH3 family protein (TAIR:AT2G47860.1)
AT1G61590	*-1,913*		Protein kinase superfamily protein; FUNCTIONS IN: protein serine/threonine kinase activity, protein kinase activity, kinase activity, ATP binding; INVOLVED IN: protein amino acid phosphorylation; LOCATED IN: chloroplast; EXPRESSED IN: 6 plant structures; EXPRESSED DURING: 4 anthesis, petal differentiation and expansion stage; CONTAINS InterPro DOMAIN/s: Protein kinase, ATP binding site (InterPro:IPR017441), Protein kinase, catalytic domain (InterPro:IPR000719), Serine-threonine/tyrosine-protein kinase (InterPro:IPR001245), Protein kinase-like domain (InterPro:IPR011009), Serine/threonine-protein kinase, active site (InterPro:IPR008271); BEST Arabidopsis thaliana protein match is: Protein kinase superfamily protein (TAIR:AT2G05940.1)
AT5G60950	*-1,913*	COBRA-LIKE PROTEIN 5 PRECURSOR (COBL5)	COBRA-like protein 5 precursor (COBL5); INVOLVED IN: biological_process unknown; LOCATED IN: endomembrane system; EXPRESSED IN: 15 plant structures; EXPRESSED DURING: 12 growth stages; CONTAINS InterPro DOMAIN/s: Glycosyl-phosphatidyl inositol-anchored, plant (InterPro:IPR006918); BEST Arabidopsis thaliana protein match is: COBRA-like extracellular glycosyl-phosphatidyl inositol-anchored protein family (TAIR:AT5G60920.1)
AT3G06460	*-1,917*		GNS1/SUR4 membrane protein family; FUNCTIONS IN: molecular_function unknown; INVOLVED IN: biological_process unknown; LOCATED IN: integral to membrane; EXPRESSED IN: root; CONTAINS InterPro DOMAIN/s: GNS1/SUR4 membrane protein (InterPro:IPR002076); BEST Arabidopsis thaliana protein match is: GNS1/SUR4 membrane protein family (TAIR:AT3G06470.1)
AT2G36430	*-1,918*		Plant protein of unknown function (DUF247); CONTAINS InterPro DOMAIN/s: Protein of unknown function DUF247, plant (InterPro:IPR004158); BEST Arabidopsis thaliana protein match is: Plant protein of unknown function (DUF247) (TAIR:AT5G22550.2)

AT4G01770	*-1,918*	RHAMNOGALACTURONAN XYLOSYLTRANSFERASE 1 (RGXT1)	Encodes a protein with UDP-xylose-dependent xylosyltransferase activity, which transfers Xyl onto L-fucose and (albeit less efficiently) L-arabinose. The linkage to L-fucose was shown to be preferentially to the <i>O</i>-4 position. Analysis of mutant containing T-DNA insertion in this gene indicate that the RGXT1 protein might be involved in the synthesis of the α-D-Xyl-(1,3)-α-L-Fuc-(1,4)-L-Rha structure in pectic rhamnogalacturonan II.
AT5G15890	*-1,918*	TRICHOME BIREFRINGENCE-LIKE 21 (TBL21)	Encodes a member of the TBL (TRICHOME BIREFRINGENCE-LIKE) gene family containing a plant-specific DUF231 (domain of unknown function) domain. TBL gene family has 46 members, two of which (TBR/AT5G06700 and TBL3/AT5G01360) have been shown to be involved in the synthesis and deposition of secondary wall cellulose, presumably by influencing the esterification state of pectic polymers. A nomenclature for this gene family has been proposed (Volker Bischoff & Wolf Scheible, 2010, personal communication).
AT4G10370	*-1,92*		Cysteine/Histidine-rich C1 domain family protein; FUNCTIONS IN: zinc ion binding; INVOLVED IN: intracellular signaling pathway; CONTAINS InterPro DOMAIN/s: Protein kinase C-like, phorbol ester/diacylglycerol binding (InterPro:IPR002219), DC1 (InterPro:IPR004146), Zinc finger, PHD-type (InterPro:IPR001965), C1-like (InterPro:IPR011424); BEST Arabidopsis thaliana protein match is: Cysteine/Histidine-rich C1 domain family protein (TAIR:AT3G26550.1)
AT4G23400	*-1,926*	PLASMA MEMBRANE INTRINSIC PROTEIN 1;5 (PIP1;5)	plasma membrane intrinsic protein 1;5 (PIP1;5); FUNCTIONS IN: water channel activity; INVOLVED IN: response to salt stress, transport; LOCATED IN: plasma membrane, membrane; EXPRESSED IN: 22 plant structures; EXPRESSED DURING: 14 growth stages; CONTAINS InterPro DOMAIN/s: Major intrinsic protein, conserved site (InterPro:IPR022357), Aquaporin (InterPro:IPR012269), Major intrinsic protein (InterPro:IPR000425); BEST Arabidopsis thaliana protein match is: plasma membrane intrinsic protein 1;4 (TAIR:AT4G00430.1)
AT4G20190	*-1,929*		unknown protein; BEST Arabidopsis thaliana protein match is: unknown protein (TAIR:AT5G44660.1)
AT5G19970	*-1,929*		unknown protein
AT5G28020	*-1,931*	CYSTEINE SYNTHASE D2 (CYSD2)	Encodes cysteine synthase CysD2.
AT1G70860	*-1,934*		Polyketide cyclase/dehydrase and lipid transport superfamily protein; FUNCTIONS IN: molecular_function unknown; INVOLVED IN: response to biotic stimulus, defense response; LOCATED IN: cellular_component unknown; CONTAINS InterPro DOMAIN/s: Bet v I allergen (InterPro:IPR000916); BEST Arabidopsis thaliana protein match is: Polyketide cyclase/dehydrase and lipid transport superfamily protein (TAIR:AT1G70880.1).
AT5G23700	*-1,935*		unknown protein; BEST Arabidopsis thaliana protein match is: unknown protein (TAIR:AT3G48860.2)
AT4G04830	*-1,938*	METHIONINE SULFOXIDE REDUCTASE B5 (MSRB5)	methionine sulfoxide reductase B5 (MSRB5); FUNCTIONS IN: peptide-methionine-(S)-S-oxide reductase activity; INVOLVED IN: oxidation reduction; LOCATED IN: cytosol; EXPRESSED IN: 24 plant structures; EXPRESSED DURING: 14 growth stages; CONTAINS InterPro DOMAIN/s: Methionine sulphoxide reductase B (InterPro:IPR002579), Mss4-like (InterPro:IPR011057); BEST Arabidopsis thaliana protein match is: methionine sulfoxide reductase B4 (TAIR:AT4G04810.1)
AT5G10580	*-1,94*		Protein of unknown function, DUF599; FUNCTIONS IN: molecular_function unknown; INVOLVED IN: biological_process unknown; LOCATED IN: endomembrane system; EXPRESSED IN: hypocotyl, root, flower; EXPRESSED DURING: petal differentiation and expansion stage; CONTAINS InterPro DOMAIN/s: Protein of unknown function DUF599 (InterPro:IPR006747); BEST Arabidopsis thaliana protein match is: Protein of unknown function, DUF599 (TAIR:AT4G31330.1)
AT2G23630	*-1,942*	SKU5 SIMILAR 16 (sks16)	SKU5 similar 16 (sks16); FUNCTIONS IN: pectinesterase activity, copper ion binding; INVOLVED IN: oxidation reduction; LOCATED IN: endomembrane system; EXPRESSED IN: shoot apex, hypocotyl, root; CONTAINS InterPro DOMAIN/s: Multicopper oxidase, type 3 (InterPro:IPR011707), Cupredoxin (InterPro:IPR008972), Multicopper oxidase, type 2 (InterPro:IPR011706), Multicopper oxidase, type 1 (InterPro:IPR001117); BEST Arabidopsis thaliana protein match is: SKU5 similar 15 (TAIR:AT4G37160.1)
AT2G39430	*-1,942*		Disease resistance-responsive (dirigent-like protein) family protein; FUNCTIONS IN: molecular_function unknown; INVOLVED IN: lignan biosynthetic process; LOCATED IN: endomembrane system; EXPRESSED IN: leaf apex, hypocotyl, root, petiole, leaf; EXPRESSED DURING: LP.04 four leaves visible, LP.08 eight leaves visible; CONTAINS InterPro DOMAIN/s: Plant disease resistance response protein (InterPro:IPR004265); BEST Arabidopsis thaliana protein match is: Disease resistance-responsive (dirigent-like protein) family protein (TAIR:AT3G55230.1)

AT1G61130	*-1,943*	SERINE CARBOXYPEPTIDASE-LIKE 32 (SCPL32)	serine carboxypeptidase-like 32 (SCPL32); FUNCTIONS IN: serine-type carboxypeptidase activity; INVOLVED IN: proteolysis; LOCATED IN: endomembrane system; EXPRESSED IN: male gametophyte, root, flower; EXPRESSED DURING: 4 anthesis; CONTAINS InterPro DOMAIN/s: Peptidase S10, serine carboxypeptidase (InterPro:IPR001563), Peptidase S10, serine carboxypeptidase, active site (InterPro:IPR018202); BEST Arabidopsis thaliana protein match is: serine carboxypeptidase-like 31 (TAIR:AT1G11080.2)
AT4G36060	*-1,943*	BASIC HELIX-LOOP-HELIX 11 (bHLH11)	basic Helix-Loop-Helix 11 (bHLH11); FUNCTIONS IN: DNA binding, sequence-specific DNA binding transcription factor activity; INVOLVED IN: regulation of transcription; LOCATED IN: nucleus, chloroplast, cytoplasm; CONTAINS InterPro DOMAIN/s: Helix-loop-helix DNA-binding domain (InterPro:IPR001092), Helix-loop-helix DNA-binding (InterPro:IPR011598); BEST Arabidopsis thaliana protein match is: basic helix-loop-helix (bHLH) DNA-binding superfamily protein (TAIR:AT3G19860.1)
AT5G54790	*-1,943*	VASCULAR-RELATED UNKNOWN PROTEIN 4 (VUP4)	unknown protein; BEST Arabidopsis thaliana protein match is: unknown protein (TAIR:AT1G50930.1)
AT3G03500	*-1,945*		TatD related DNase; FUNCTIONS IN: endodeoxyribonuclease activity, producing 5'-phosphomonoesters, deoxyribonuclease activity; INVOLVED IN: biological_process unknown; LOCATED IN: endomembrane system; CONTAINS InterPro DOMAIN/s: Deoxyribonuclease, TatD Mg-dependent (InterPro:IPR012278), Deoxyribonuclease, TatD (InterPro:IPR015992), Deoxyribonuclease, TatD-related, conserved site (InterPro:IPR018228), Deoxyribonuclease, TatD-related (InterPro:IPR001130); BEST Arabidopsis thaliana protein match is: TatD related DNase (TAIR:AT5G17570.1)
AT5G06270	*-1,945*		unknown protein; FUNCTIONS IN: molecular_function unknown; INVOLVED IN: biological_process unknown; LOCATED IN: chloroplast; EXPRESSED IN: 21 plant structures; EXPRESSED DURING: 11 growth stages; BEST Arabidopsis thaliana protein match is: unknown protein (TAIR:AT3G11600.1)
AT1G75920	*-1,948*		GDSL-motif esterase/acyltransferase/lipase. Enzyme group with broad substrate specificity that may catalyze acyltransfer or hydrolase reactions with lipid and non-lipid substrates. The mRNA is cell-to-cell mobile.
AT5G46090	*-1,949*		Protein of unknown function (DUF679); FUNCTIONS IN: molecular_function unknown; INVOLVED IN: biological_process unknown; LOCATED IN: chloroplast; CONTAINS InterPro DOMAIN/s: Protein of unknown function DUF679 (InterPro:IPR007770); BEST Arabidopsis thaliana protein match is: Protein of unknown function (DUF679) (TAIR:AT4G18425.1)
AT3G17600	*-1,95*	INDOLE-3-ACETIC ACID INDUCIBLE 31 (IAA31)	Encodes a member of the Aux/IAA family of proteins implicated in auxin signaling. IAA31 shares several residues with the conserved domain II region, believed to act as a degron in many of the rapidly degraded Aux/IAA family members. An IAA31 fusion protein is quite long-lived, but can be degraded more rapidly in the presence of auxin. Unlike many other family members, IAA31 transcript levels do not rise in response to auxin. Nevertheless, overexpression of IAA31 leads to defects in auxin-related processes such as gravitropism, root development, shoot development, and cotyledon vascular development.
AT1G52200	*-1,951*		PLAC8 family protein; FUNCTIONS IN: molecular_function unknown; INVOLVED IN: response to oxidative stress; LOCATED IN: plasma membrane; EXPRESSED IN: 10 plant structures; EXPRESSED DURING: 6 growth stages; CONTAINS InterPro DOMAIN/s: Protein of unknown function Cys-rich (InterPro:IPR006461); BEST Arabidopsis thaliana protein match is: PLAC8 family protein (TAIR:AT3G18450.1)
AT1G70850	*-1,951*	MLP-LIKE PROTEIN 34 (MLP34)	MLP-like protein 34 (MLP34); FUNCTIONS IN: molecular_function unknown; INVOLVED IN: response to biotic stimulus, defense response; LOCATED IN: cellular_component unknown; EXPRESSED IN: 6 plant structures; EXPRESSED DURING: 4 anthesis, petal differentiation and expansion stage; CONTAINS InterPro DOMAIN/s: Bet v I allergen (InterPro:IPR000916); BEST Arabidopsis thaliana protein match is: MLP-like protein 28 (TAIR:AT1G70830.1)
AT3G01730	*-1,954*		Mutants exhibit shorter root hairs under phosphate-deficient conditions.
AT2G05160	*-1,955*		CCCH-type zinc fingerfamily protein with RNA-binding domain; FUNCTIONS IN: RNA binding, nucleotide binding, zinc ion binding, nucleic acid binding; INVOLVED IN: biological_process unknown; LOCATED IN: cellular_component unknown; EXPRESSED IN: 6 plant structures; EXPRESSED DURING: 4 anthesis, petal differentiation and expansion stage; CONTAINS InterPro DOMAIN/s: Zinc finger, CCCH-type (InterPro:IPR000571), RNA recognition motif, RNP-1 (InterPro:IPR000504), Nucleotide-binding, alpha-beta plait (InterPro:IPR012677); BEST Arabidopsis thaliana protein match is: Zinc finger (CCCH-type) family protein / RNA recognition motif (RRM)-containing protein (TAIR:AT3G52980.1)

AT3G60550	*-1,955*	CYCLIN P3;2 (CYCP3;2)	cyclin p3;2 (CYCP3;2); CONTAINS InterPro DOMAIN/s: Negative regulatory factor PREG (InterPro:IPR012389), Cyclin-like (InterPro:IPR011028), Cyclin-related 2 (InterPro:IPR013922); BEST Arabidopsis thaliana protein match is: cyclin p3;1 (TAIR:AT2G45080.1)
AT1G22500	*-1,957*	ARABIDOPSIS TOXICOS EN LEVADURA 15 (ATL15)	Gene encodes a putative C3HC4-type RING zinc finger factor. it is induced in response to light and ascorbate stimulus.
AT2G01880	*-1,96*	PURPLE ACID PHOSPHATASE 7 (PAP7)	purple acid phosphatase 7 (PAP7); FUNCTIONS IN: protein serine/threonine phosphatase activity, acid phosphatase activity; LOCATED IN: endomembrane system; EXPRESSED IN: 7 plant structures; CONTAINS InterPro DOMAIN/s: Metallophosphoesterase (InterPro:IPR004843); BEST Arabidopsis thaliana protein match is: purple acid phosphatase 17 (TAIR:AT3G17790.1)
AT2G20750	*-1,961*	EXPANSIN B1 (EXPB1)	member of BETA-EXPANSINS. Naming convention from the Expansin Working Group (Kende et al, 2004. Plant Mol Bio)
AT4G29450	*-1,961*		Leucine-rich repeat protein kinase family protein; FUNCTIONS IN: kinase activity; INVOLVED IN: protein amino acid phosphorylation; LOCATED IN: endomembrane system; EXPRESSED IN: male gametophyte, pollen tube; EXPRESSED DURING: L mature pollen stage, M germinated pollen stage; CONTAINS InterPro DOMAIN/s: Protein kinase, ATP binding site (InterPro:IPR017441), Protein kinase, catalytic domain (InterPro:IPR000719), Leucine-rich repeat (InterPro:IPR001611), Serine-threonine/tyrosine-protein kinase (InterPro:IPR001245), Protein kinase-like domain (InterPro:IPR011009), Serine/threonine-protein kinase, active site (InterPro:IPR008271); BEST Arabidopsis thaliana protein match is: root hair specific 16 (TAIR:AT4G29180.2)
AT5G07780	*-1,963*	FORMIN HOMOLOG 19 (FH19)	Encodes a class II formin that nucleates actin assembly, binds to the barbed-end of actin filaments and antagonizes the effect of FH1 on actin dynamics. The mRNA is cell-to-cell mobile.
AT5G15600	*-1,965*	SPIRAL1-LIKE4 (SP1L4)	SPIRAL1-LIKE4 belongs to a six-member gene family in Arabidopsis; all members share high sequence similarity in amino- and carboxy-terminal regions. Regulates cortical microtubule organization. Mutant plants exhibit altered patterns of root, leaf and petal growth as a result of defective anisotropic cell expansion.
AT4G13440	*-1,966*		Calcium-binding EF-hand family protein; FUNCTIONS IN: calcium ion binding; INVOLVED IN: biological_process unknown; LOCATED IN: cellular_component unknown; EXPRESSED IN: root; CONTAINS InterPro DOMAIN/s: EF-Hand 1, calcium-binding site (InterPro:IPR018247), EF-HAND 2 (InterPro:IPR018249), EF-hand-like domain (InterPro:IPR011992), Calcium-binding EF-hand (InterPro:IPR002048)
AT5G43180	*-1,968*		Protein of unknown function, DUF599; FUNCTIONS IN: molecular_function unknown; INVOLVED IN: biological_process unknown; LOCATED IN: endomembrane system; EXPRESSED IN: 10 plant structures; EXPRESSED DURING: LP.04 four leaves visible, 4 anthesis, petal differentiation and expansion stage; CONTAINS InterPro DOMAIN/s: Protein of unknown function DUF599 (InterPro:IPR006747); BEST Arabidopsis thaliana protein match is: Protein of unknown function, DUF599 (TAIR:AT4G31330.1)
AT2G27550	*-1,969*	CENTRORADIALIS (ATC)	encodes a protein similar to TFL1. overexpression leads to similar phenotype as TFL1 overexpression. expressed specifically in the hypocotyl and null mutation does not result in phenotypes exhibited by TFL1 null mutations. It acts non-cell autonomously to inhibit floral initiation.
AT5G62280	*-1,97*		Protein of unknown function (DUF1442); CONTAINS InterPro DOMAIN/s: Protein of unknown function DUF1442 (InterPro:IPR009902); BEST Arabidopsis thaliana protein match is: Protein of unknown function (DUF1442) (TAIR:AT2G45360.1)
AT5G49870	*-1,971*		Mannose-binding lectin superfamily protein; CONTAINS InterPro DOMAIN/s: Mannose-binding lectin (InterPro:IPR001229); BEST Arabidopsis thaliana protein match is: Mannose-binding lectin superfamily protein (TAIR:AT5G49850.1)
AT1G05250	*-1,975*	PEROXIDASE 2 (PRX2)	Encodes a cationic cell-wall-bound peroxidase homolog that is involved in the lignification of cell walls.

AT1G49570	*-1,978*		Peroxidase superfamily protein; FUNCTIONS IN: peroxidase activity, heme binding; INVOLVED IN: oxidation reduction, response to oxidative stress; LOCATED IN: endomembrane system; EXPRESSED IN: leaf lamina base, leaf whorl, hypocotyl, sepal, root; EXPRESSED DURING: 4 anthesis; CONTAINS InterPro DOMAIN/s: Haem peroxidase (InterPro:IPR010255), Plant peroxidase (InterPro:IPR000823), Peroxidases heam-ligand binding site (InterPro:IPR019793), Haem peroxidase, plant/fungal/bacterial (InterPro:IPR002016), Peroxidase, active site (InterPro:IPR019794); BEST Arabidopsis thaliana protein match is: Peroxidase superfamily protein (TAIR:AT5G06730.1)
AT1G68040	*-1,978*		S-adenosyl-L-methionine-dependent methyltransferases superfamily protein; FUNCTIONS IN: S-adenosylmethionine-dependent methyltransferase activity, methyltransferase activity; INVOLVED IN: biological_process unknown; LOCATED IN: cellular_component unknown; EXPRESSED IN: root; CONTAINS InterPro DOMAIN/s: SAM dependent carboxyl methyltransferase (InterPro:IPR005299); BEST Arabidopsis thaliana protein match is: S-adenosyl-L-methionine-dependent methyltransferases superfamily protein (TAIR:AT5G38100.1)
AT5G59260	*-1,979*	L-TYPE LECTIN RECEPTOR KINASE II.1 (LECRK-II.1)	Concanavalin A-like lectin protein kinase family protein; FUNCTIONS IN: protein serine/threonine kinase activity, binding, protein kinase activity, kinase activity, ATP binding; INVOLVED IN: protein amino acid phosphorylation; LOCATED IN: endomembrane system; EXPRESSED IN: hypocotyl, root, stamen; EXPRESSED DURING: 4 anthesis; CONTAINS InterPro DOMAIN/s: Legume lectin, beta chain (InterPro:IPR001220), Protein kinase, ATP binding site (InterPro:IPR017441), Serine/threonine-protein kinase-like domain (InterPro:IPR017442), Concanavalin A-like lectin/glucanase, subgroup (InterPro:IPR013320), Protein kinase-like domain (InterPro:IPR011009), Serine/threonine-protein kinase, active site (InterPro:IPR008271), Protein kinase, catalytic domain (InterPro:IPR000719), Concanavalin A-like lectin/glucanase (InterPro:IPR008985); BEST Arabidopsis thaliana protein match is: Concanavalin A-like lectin protein kinase family protein (TAIR:AT5G59270.1)
AT1G67590	*-1,98*		Remorin family protein; FUNCTIONS IN: DNA binding; INVOLVED IN: biological_process unknown; LOCATED IN: nucleus; EXPRESSED IN: 23 plant structures; EXPRESSED DURING: 12 growth stages; CONTAINS InterPro DOMAIN/s: Remorin, C-terminal (InterPro:IPR005516); BEST Arabidopsis thaliana protein match is: Remorin family protein (TAIR:AT2G02170.2)
AT5G51490	*-1,984*		Plant invertase/pectin methylesterase inhibitor superfamily; FUNCTIONS IN: enzyme inhibitor activity, pectinesterase activity; INVOLVED IN: cell wall modification; LOCATED IN: endomembrane system, cell wall, plant-type cell wall; EXPRESSED IN: flower; CONTAINS InterPro DOMAIN/s: Pectinesterase, active site (InterPro:IPR018040), Pectin lyase fold/virulence factor (InterPro:IPR011050), Pectinesterase, catalytic (InterPro:IPR000070), Pectinesterase inhibitor (InterPro:IPR006501), Pectin lyase fold (InterPro:IPR012334); BEST Arabidopsis thaliana protein match is: Plant invertase/pectin methylesterase inhibitor superfamily (TAIR:AT5G51500.1)
AT5G42250	*-1,989*		Zinc-binding alcohol dehydrogenase family protein; FUNCTIONS IN: oxidoreductase activity, zinc ion binding; INVOLVED IN: oxidation reduction; EXPRESSED IN: 11 plant structures; EXPRESSED DURING: 6 growth stages; CONTAINS InterPro DOMAIN/s: GroES-like (InterPro:IPR011032), Alcohol dehydrogenase GroES-like (InterPro:IPR013154), Alcohol dehydrogenase, zinc-containing, conserved site (InterPro:IPR002328), Alcohol dehydrogenase, C-terminal (InterPro:IPR013149), Alcohol dehydrogenase superfamily, zinc-containing (InterPro:IPR002085); BEST Arabidopsis thaliana protein match is: GroES-like zinc-binding dehydrogenase family protein (TAIR:AT4G22110.2)
AT2G28160	*-1,99*	FER-LIKE REGULATOR OF IRON UPTAKE (FRU)	Encodes a putative transcription factor that regulates iron uptake responses. mRNA is detected in the outer cell layers of the root and accumulates in response to iron deficiency. The expression of many iron-regulated genes is dependent on FIT1. It specifically regulates FRO2 at the level of mRNA accumulation and IRT1 at the level of protein accumulation.Similar to FER in tomato and is a regulator of iron uptake. It is post-transcriptionally controlled.
AT2G23410	*-1,992*	CIS-PRENYLTRANSFERASE (CPT)	encodes cis-prenyltransferase
AT4G10350	*-1,992*	NAC DOMAIN CONTAINING PROTEIN 70 (NAC070)	NAC domain protein. SMB, BRN1, and BRN2 act to regulate root cap maturation, in a partially redundant fashion.BRN1 and BRN2, control the cell wall maturation processes that are required to detach root cap layers from the root.
AT2G37750	*-1,996*		unknown protein
AT2G46740	*-2,001*	L -GULONO-1,4-LACTONE (L -GULL) OXIDASE 5 (GULLO5)	Encodes a homolog of rat L-gulono-1,4-lactone (L-GulL) oxidase that is involved in the biosynthesis of L-ascorbic acid.

AT5G46650	*-2,002*		RING/U-box superfamily protein; FUNCTIONS IN: zinc ion binding; CONTAINS InterPro DOMAIN/s: Zinc finger, RING-type (InterPro:IPR001841), Zinc finger, C3HC4 RING-type (InterPro:IPR018957); BEST Arabidopsis thaliana protein match is: RING/U-box superfamily protein (TAIR:AT4G17920.1)
AT1G75780	*-2,005*	TUBULIN BETA-1 CHAIN (TUB1)	beta tubulin gene downregulated by phytochrome A (phyA)-mediated far-red light high-irradiance and the phytochrome B (phyB)-mediated red light high-irradiance responses
AT1G48690	*-2,009*		Auxin-responsive GH3 family protein; CONTAINS InterPro DOMAIN/s: GH3 auxin-responsive promoter (InterPro:IPR004993); BEST Arabidopsis thaliana protein match is: Auxin-responsive GH3 family protein (TAIR:AT5G51470.1)
AT5G59270	*-2,01*	L-TYPE LECTIN RECEPTOR KINASE II.2 (LECRK-II.2)	Concanavalin A-like lectin protein kinase family protein; FUNCTIONS IN: kinase activity; INVOLVED IN: protein amino acid phosphorylation; LOCATED IN: endomembrane system; CONTAINS InterPro DOMAIN/s: Legume lectin, beta chain (InterPro:IPR001220), Protein kinase, ATP binding site (InterPro:IPR017441), Serine/threonine-protein kinase domain (InterPro:IPR002290), Serine/threonine-protein kinase-like domain (InterPro:IPR017442), Concanavalin A-like lectin/glucanase, subgroup (InterPro:IPR013320), Serine/threonine-protein kinase, active site (InterPro:IPR008271), Protein kinase-like domain (InterPro:IPR011009), Protein kinase, catalytic domain (InterPro:IPR000719), Tyrosine-protein kinase, catalytic domain (InterPro:IPR020635), Concanavalin A-like lectin/glucanase (InterPro:IPR008985), Legume lectin, beta chain, Mn/Ca-binding site (InterPro:IPR019825); BEST Arabidopsis thaliana protein match is: Concanavalin A-like lectin protein kinase family protein (TAIR:AT5G59260.1)
AT5G61350	*-2,012*	[CA2+]CYT-ASSOCIATED PROTEIN KINASE 1 (CAP1)	Encodes a tonoplast-localized receptor-like kinase that regulates root hair tip growth by maintaining cytoplasmic Ca2+ gradients.CAP1, a tonoplast-localized receptor-like kinase, regulates root hair tip growth by maintaining cytoplasmic Ca2+ gradients. Knockouts of CAP1 produced more cytoplasmic NH4+ and ceased growth of root hairs on MS medium except when NH4+ was depleted; NH4+ depletion reestablished the Ca2+ gradient necessary for normal growth. The lower net NH4+ influx across the vacuolar membrane and relatively alkaline cytosolic pH of root hairs in cap1-1 relative to wild type implied that mutation of CAP1 results in more NH4+ accumulation in the cytoplasm. Furthermore, CAP1 functionally complemented npr1 kinase yeast mutant defective in high-affinity NH4+ uptake via MEP2, distinguishing CAP1 as a cytosolic modulator of NH4+ level that participates in NH4+ homeostasis-regulated root hair growth by modulating tip-focused cytoplasmic Ca2+ gradients.
AT2G20080	*-2,015*	TCP INTERACTOR CONTAINING EAR MOTIF PROTEIN 2 (TIE2)	unknown protein; FUNCTIONS IN: molecular_function unknown; INVOLVED IN: biological_process unknown; LOCATED IN: chloroplast; BEST Arabidopsis thaliana protein match is: unknown protein (TAIR:AT4G28840.1)
AT2G29620	*-2,015*		unknown protein; BEST Arabidopsis thaliana protein match is: unknown protein (TAIR:AT1G07330.1)
AT5G06990	*-2,015*		Protein of unknown function, DUF617; FUNCTIONS IN: molecular_function unknown; INVOLVED IN: biological_process unknown; LOCATED IN: chloroplast; CONTAINS InterPro DOMAIN/s: Protein of unknown function DUF617, plant (InterPro:IPR006460); BEST Arabidopsis thaliana protein match is: Protein of unknown function, DUF617 (TAIR:AT2G21990.1)
AT2G28780	*-2,022*		unknown protein; INVOLVED IN: biological_process unknown; LOCATED IN: mitochondrion; EXPRESSED IN: inflorescence meristem, root, flower; EXPRESSED DURING: petal differentiation and expansion stage; CONTAINS InterPro DOMAIN/s: Protein of unknown function DUF939, bacterial (InterPro:IPR010343); BEST Arabidopsis thaliana protein match is: unknown protein (TAIR:AT3G09450.1)
AT1G49100	*-2,023*		Leucine-rich repeat protein kinase family protein; FUNCTIONS IN: kinase activity; INVOLVED IN: protein amino acid phosphorylation; LOCATED IN: endomembrane system; CONTAINS InterPro DOMAIN/s: Protein kinase, ATP binding site (InterPro:IPR017441), Serine/threonine-protein kinase domain (InterPro:IPR002290), Leucine-rich repeat (InterPro:IPR001611), Serine-threonine/tyrosine-protein kinase (InterPro:IPR001245), Serine/threonine-protein kinase, active site (InterPro:IPR008271), Protein kinase-like domain (InterPro:IPR011009), Protein kinase, catalytic domain (InterPro:IPR000719), Tyrosine-protein kinase, catalytic domain (InterPro:IPR020635); BEST Arabidopsis thaliana protein match is: Leucine-rich repeat protein kinase family protein (TAIR:AT3G21340.1)
AT1G63450	*-2,023*	ROOT HAIR SPECIFIC 8 (RHS8)	Encodes a xyloglucan-specific galacturonosyltransferase (XUT1) that forms the beta-d-galactosyluronic acid-(1->2)-alpha-d-xylosyl linkage.
AT1G21310	*-2,024*	EXTENSIN 3 (EXT3)	Encodes extensin 3.

AT3G61430	*-2,024*	PLASMA MEMBRANE INTRINSIC PROTEIN 1A (PIP1A)	a member of the plasma membrane intrinsic protein subfamily PIP1. localizes to the plasma membrane and exhibits water transport activity in Xenopus oocyte. expressed ubiquitously and protein level decreases slightly during leaf development. The mRNA is cell-to-cell mobile.
AT5G26200	*-2,024*		Mitochondrial substrate carrier family protein; FUNCTIONS IN: binding; INVOLVED IN: transport, mitochondrial transport, transmembrane transport; LOCATED IN: mitochondrial inner membrane, membrane; EXPRESSED IN: 7 plant structures; EXPRESSED DURING: 7 growth stages; CONTAINS InterPro DOMAIN/s: Mitochondrial carrier protein (InterPro:IPR002067), Mitochondrial substrate carrier (InterPro:IPR001993), Mitochondrial substrate/solute carrier (InterPro:IPR018108); BEST Arabidopsis thaliana protein match is: Mitochondrial substrate carrier family protein (TAIR:AT1G72820.1)
AT1G73780	*-2,026*		Bifunctional inhibitor/lipid-transfer protein/seed storage 2S albumin superfamily protein; FUNCTIONS IN: lipid binding; INVOLVED IN: lipid transport; LOCATED IN: endomembrane system; EXPRESSED IN: root; CONTAINS InterPro DOMAIN/s: Bifunctional inhibitor/plant lipid transfer protein/seed storage (InterPro:IPR016140), Plant lipid transfer protein/seed storage/trypsin-alpha amylase inhibitor (InterPro:IPR003612), Plant lipid transfer protein/hydrophobic protein, helical domain (InterPro:IPR013770); BEST Arabidopsis thaliana protein match is: Bifunctional inhibitor/lipid-transfer protein/seed storage 2S albumin superfamily protein (TAIR:AT3G18280.1)
AT1G11740	*-2,029*		ankyrin repeat family protein; CONTAINS InterPro DOMAIN/s: Ankyrin repeat-containing domain (InterPro:IPR020683), Protein of unknown function DUF3424 (InterPro:IPR021832), Ankyrin repeat (InterPro:IPR002110); BEST Arabidopsis thaliana protein match is: Ankyrin repeat family protein (TAIR:AT1G62050.1)
AT4G20450	*-2,029*		Leucine-rich repeat protein kinase family protein; FUNCTIONS IN: kinase activity; INVOLVED IN: protein amino acid phosphorylation; LOCATED IN: endomembrane system; EXPRESSED IN: 6 plant structures; EXPRESSED DURING: 4 anthesis, 4 leaf senescence stage, E expanded cotyledon stage; CONTAINS InterPro DOMAIN/s: Protein kinase, ATP binding site (InterPro:IPR017441), Protein kinase, catalytic domain (InterPro:IPR000719), Leucine-rich repeat (InterPro:IPR001611), Serine/threonine-protein kinase-like domain (InterPro:IPR017442), Protein kinase-like domain (InterPro:IPR011009), Serine/threonine-protein kinase, active site (InterPro:IPR008271); BEST Arabidopsis thaliana protein match is: Leucine-rich repeat protein kinase family protein (TAIR:AT2G29000.1)
AT5G36870	*-2,029*	GLUCAN SYNTHASE-LIKE 9 (GSL09)	encodes a gene similar to callose synthase
AT1G53640	*-2,03*		unknown protein; FUNCTIONS IN: molecular_function unknown; INVOLVED IN: biological_process unknown; LOCATED IN: endomembrane system; EXPRESSED IN: 20 plant structures; EXPRESSED DURING: 11 growth stages
AT2G30340	*-2,03*	LOB DOMAIN-CONTAINING PROTEIN 13 (LBD13)	LOB domain-containing protein 13 (LBD13); FUNCTIONS IN: molecular_function unknown; INVOLVED IN: biological_process unknown; LOCATED IN: membrane; EXPRESSED IN: root, leaf; CONTAINS InterPro DOMAIN/s: Lateral organ boundaries, LOB (InterPro:IPR004883); BEST Arabidopsis thaliana protein match is: LOB domain-containing protein 15 (TAIR:AT2G40470.1)
AT2G36690	*-2,031*		2-oxoglutarate (2OG) and Fe(II)-dependent oxygenase superfamily protein; FUNCTIONS IN: oxidoreductase activity, acting on paired donors, with incorporation or reduction of molecular oxygen, 2-oxoglutarate as one donor, and incorporation of one atom each of oxygen into both donors, oxidoreductase activity; INVOLVED IN: biosynthetic process; LOCATED IN: cellular_component unknown; EXPRESSED IN: hypocotyl, root; CONTAINS InterPro DOMAIN/s: Oxoglutarate/iron-dependent oxygenase (InterPro:IPR005123); BEST Arabidopsis thaliana protein match is: 2-oxoglutarate (2OG) and Fe(II)-dependent oxygenase superfamily protein (TAIR:AT3G60290.1)
AT4G21340	*-2,031*	(B70)	B70; FUNCTIONS IN: sequence-specific DNA binding transcription factor activity; INVOLVED IN: regulation of transcription; LOCATED IN: nucleus; EXPRESSED IN: root endodermis, primary root differentiation zone; CONTAINS InterPro DOMAIN/s: Helix-loop-helix DNA-binding domain (InterPro:IPR001092), Helix-loop-helix DNA-binding (InterPro:IPR011598); BEST Arabidopsis thaliana protein match is: basic helix-loop-helix (bHLH) DNA-binding superfamily protein (TAIR:AT4G05170.1)
AT2G25680	*-2,034*	MOLYBDATE TRANSPORTER 1 (MOT1)	Encodes a high-affinity molybdate transporter. Mutant has reduced concentrations of molybdate in roots and shoots, and reduced shoot and root length when growing on Mo-limited medium.

AT1G51870	*-2,035*		protein kinase family protein; FUNCTIONS IN: protein serine/threonine kinase activity, protein kinase activity, kinase activity, ATP binding; INVOLVED IN: protein amino acid phosphorylation; LOCATED IN: endomembrane system; CONTAINS InterPro DOMAIN/s: Protein kinase, ATP binding site (InterPro:IPR017441), Serine/threonine-protein kinase domain (InterPro:IPR002290), Serine-threonine/tyrosine-protein kinase (InterPro:IPR001245), Protein kinase-like domain (InterPro:IPR011009), Serine/threonine-protein kinase, active site (InterPro:IPR008271), Protein kinase, catalytic domain (InterPro:IPR000719), Tyrosine-protein kinase, catalytic domain (InterPro:IPR020635); BEST Arabidopsis thaliana protein match is: Leucine-rich repeat protein kinase family protein (TAIR:AT1G51860.1)
AT3G25905	*-2,036*	CLAVATA3/ESR-RELATED 27 (CLE27)	Member of a large family of putative ligands homologous to the Clavata3 gene. Consists of a single exon.
AT5G06200	*-2,036*	CASPARIAN STRIP MEMBRANE DOMAIN PROTEIN 4 (CASP4)	Uncharacterised protein family (UPF0497); CONTAINS InterPro DOMAIN/s: Uncharacterised protein family UPF0497, trans-membrane plant (InterPro:IPR006702), Uncharacterised protein family UPF0497, trans-membrane plant subgroup (InterPro:IPR006459); BEST Arabidopsis thaliana protein match is: Uncharacterised protein family (UPF0497) (TAIR:AT3G11550.1)
AT4G15390	*-2,037*		HXXXD-type acyl-transferase family protein; FUNCTIONS IN: transferase activity, transferring acyl groups other than amino-acyl groups, transferase activity; INVOLVED IN: biological_process unknown; LOCATED IN: cellular_component unknown; EXPRESSED IN: hypocotyl, root; CONTAINS InterPro DOMAIN/s: Transferase (InterPro:IPR003480); BEST Arabidopsis thaliana protein match is: HXXXD-type acyl-transferase family protein (TAIR:AT3G30280.1)
AT2G42870	*-2,038*	PHY RAPIDLY REGULATED 1 (PAR1)	Encodes PHYTOCHROME RAPIDLY REGULATED1 (PAR1), an atypical basic helix-loop-helix (bHLP) protein. Closely related to PAR2 (At3g58850). Up regulated after simulated shade perception. Acts in the nucleus to control plant development and as a negative regulator of shade avoidance response. Functions as transcriptional repressor of auxin-responsive genes SAUR15 (AT4G38850) and SAUR68 (AT1G29510).
AT1G26360	*-2,04*	METHYL ESTERASE 13 (MES13)	Encodes a protein predicted to act as a carboxylesterase. It has similarity to the SABP2 methyl salicylate esterase from tobacco but no enzymatic activity has been identified for this protein.
AT1G79840	*-2,041*	GLABRA 2 (GL2)	Glabra 2, a homeodomain protein affects epidermal cell identity including trichomes, root hairs, and seed coat. It also down-regulates seed oil content. Expressed in atrichoblasts and required to suppress root hair development. Also expressed abundantly during early seed development. Directly regulated by WER.
AT1G15540	*-2,042*		2-oxoglutarate (2OG) and Fe(II)-dependent oxygenase superfamily protein; FUNCTIONS IN: oxidoreductase activity; INVOLVED IN: N-terminal protein myristoylation; CONTAINS InterPro DOMAIN/s: Oxoglutarate/iron-dependent oxygenase (InterPro:IPR005123); BEST Arabidopsis thaliana protein match is: 2-oxoglutarate (2OG) and Fe(II)-dependent oxygenase superfamily protein (TAIR:AT1G80320.1)
AT1G71740	*-2,043*		unknown protein
AT2G18800	*-2,047*	XYLOGLUCAN ENDOTRANSGLUCOSYLASE/HYDROLASE 21 (XTH21)	xyloglucan endotransglucosylase/hydrolase 21 (XTH21); FUNCTIONS IN: hydrolase activity, acting on glycosyl bonds, xyloglucan endotransglucosylase activity; INVOLVED IN: primary root development, cell wall modification; LOCATED IN: endomembrane system, apoplast, cell wall; EXPRESSED IN: stem, root, flower, leaf; CONTAINS InterPro DOMAIN/s: Xyloglucan endotransglucosylase/hydrolase (InterPro:IPR016455), Xyloglucan endo-transglycosylase, C-terminal (InterPro:IPR010713), Concanavalin A-like lectin/glucanase, subgroup (InterPro:IPR013320), Concanavalin A-like lectin/glucanase (InterPro:IPR008985), Glycoside hydrolase, family 16 (InterPro:IPR000757), Glycoside hydrolase, family 16, active site (InterPro:IPR008263); BEST Arabidopsis thaliana protein match is: Xyloglucan endotransglucosylase/hydrolase family protein (TAIR:AT5G57560.1)
AT1G52660	*-2,055*		P-loop containing nucleoside triphosphate hydrolases superfamily protein; FUNCTIONS IN: ATP binding; INVOLVED IN: defense response, apoptosis; EXPRESSED IN: root; CONTAINS InterPro DOMAIN/s: NB-ARC (InterPro:IPR002182), Disease resistance protein (InterPro:IPR000767); BEST Arabidopsis thaliana protein match is: P-loop containing nucleoside triphosphate hydrolases superfamily protein (TAIR:AT3G15700.1)

AT2G21030	*-2,059*		CONTAINS InterPro DOMAIN/s: Disease resistance/zinc finger/chromosome condensation-like region (InterPro:IPR013591); BEST Arabidopsis thaliana protein match is: DZC (Disease resistance/zinc finger/chromosome condensation-like region) domain containing protein (TAIR:AT1G31880.1)
AT1G14220	*-2,06*		Ribonuclease T2 family protein; FUNCTIONS IN: ribonuclease T2 activity, endoribonuclease activity, RNA binding; INVOLVED IN: N-terminal protein myristoylation; LOCATED IN: endomembrane system; EXPRESSED IN: sperm cell, root, egg cell; CONTAINS InterPro DOMAIN/s: Ribonuclease T2 (InterPro:IPR001568), Ribonuclease T2, active site (InterPro:IPR018188); BEST Arabidopsis thaliana protein match is: ribonuclease 3 (TAIR:AT1G26820.1)
AT2G29330	*-2,061*	TROPINONE REDUCTASE (TRI)	tropinone reductase (TRI); FUNCTIONS IN: oxidoreductase activity, binding, catalytic activity; INVOLVED IN: oxidation reduction, metabolic process; EXPRESSED IN: embryo, hypocotyl, root; EXPRESSED DURING: C globular stage; CONTAINS InterPro DOMAIN/s: NAD(P)-binding domain (InterPro:IPR016040), Glucose/ribitol dehydrogenase (InterPro:IPR002347), Short-chain dehydrogenase/reductase SDR (InterPro:IPR002198); BEST Arabidopsis thaliana protein match is: NAD(P)-binding Rossmann-fold superfamily protein (TAIR:AT2G30670.1)
AT2G22000	*-2,062*	ELICITOR PEPTIDE 6 PRECURSOR (PROPEP6)	elicitor peptide 6 precursor (PROPEP6)
AT2G22800	*-2,062*	(HAT9)	Encodes homeobox protein HAT9.
AT1G77330	*-2,065*	ACC OXIDASE 5 (ACO5)	similar to 1-aminocyclopropane-1-carboxylate oxidase GI:3386565 from (Sorghum bicolor)
AT4G30170	*-2,065*		Peroxidase family protein; FUNCTIONS IN: peroxidase activity, heme binding; INVOLVED IN: oxidation reduction, response to oxidative stress; LOCATED IN: endomembrane system; EXPRESSED IN: hypocotyl, root; CONTAINS InterPro DOMAIN/s: Haem peroxidase (InterPro:IPR010255), Plant peroxidase (InterPro:IPR000823), Peroxidases heam-ligand binding site (InterPro:IPR019793), Haem peroxidase, plant/fungal/bacterial (InterPro:IPR002016), Peroxidase, active site (InterPro:IPR019794); BEST Arabidopsis thaliana protein match is: Peroxidase superfamily protein (TAIR:AT2G18980.1)
AT5G06900	*-2,066*	CYTOCHROME P450, FAMILY 93, SUBFAMILY D, POLYPEPTIDE 1 (CYP93D1)	member of CYP93D
AT1G70880	*-2,067*		Polyketide cyclase/dehydrase and lipid transport superfamily protein; FUNCTIONS IN: molecular_function unknown; INVOLVED IN: response to biotic stimulus, defense response; LOCATED IN: cellular_component unknown; EXPRESSED IN: root; CONTAINS InterPro DOMAIN/s: Bet v I allergen (InterPro:IPR000916); BEST Arabidopsis thaliana protein match is: MLP-like protein 28 (TAIR:AT1G70830.5)
AT2G18980	*-2,068*		Peroxidase superfamily protein; FUNCTIONS IN: peroxidase activity, heme binding; INVOLVED IN: oxidation reduction, response to oxidative stress; LOCATED IN: endomembrane system; EXPRESSED IN: 6 plant structures; EXPRESSED DURING: LP.04 four leaves visible, petal differentiation and expansion stage; CONTAINS InterPro DOMAIN/s: Haem peroxidase (InterPro:IPR010255), Plant peroxidase (InterPro:IPR000823), Peroxidases heam-ligand binding site (InterPro:IPR019793), Peroxidase, active site (InterPro:IPR019794), Haem peroxidase, plant/fungal/bacterial (InterPro:IPR002016); BEST Arabidopsis thaliana protein match is: Peroxidase family protein (TAIR:AT4G30170.1)
AT4G00080	*-2,069*	UNFERTILIZED EMBRYO SAC 11 (UNE11)	unfertilized embryo sac 11 (UNE11); FUNCTIONS IN: enzyme inhibitor activity, pectinesterase inhibitor activity, pectinesterase activity; INVOLVED IN: double fertilization forming a zygote and endosperm; LOCATED IN: endomembrane system; EXPRESSED IN: root; CONTAINS InterPro DOMAIN/s: Pectinesterase inhibitor (InterPro:IPR006501); BEST Arabidopsis thaliana protein match is: Plant invertase/pectin methylesterase inhibitor superfamily protein (TAIR:AT1G62770.1)
AT5G40320	*-2,07*		Cysteine/Histidine-rich C1 domain family protein; FUNCTIONS IN: zinc ion binding; INVOLVED IN: intracellular signaling pathway; LOCATED IN: cellular_component unknown; CONTAINS InterPro DOMAIN/s: Protein kinase C-like, phorbol ester/diacylglycerol binding (InterPro:IPR002219), DC1 (InterPro:IPR004146), Zinc finger, PHD-type (InterPro:IPR001965), C1-like (InterPro:IPR011424); BEST Arabidopsis thaliana protein match is: Cysteine/Histidine-rich C1 domain family protein (TAIR:AT5G45730.1)

AT2G28270	*-2,071*		Cysteine/Histidine-rich C1 domain family protein; CONTAINS InterPro DOMAIN/s: DC1 (InterPro:IPR004146), C1-like (InterPro:IPR011424); BEST Arabidopsis thaliana protein match is: Cysteine/Histidine-rich C1 domain family protein (TAIR:AT2G44380.1)
AT4G28530	*-2,071*	NAC DOMAIN CONTAINING PROTEIN 74 (NAC074)	NAC domain containing protein 74 (NAC074); FUNCTIONS IN: sequence-specific DNA binding transcription factor activity; INVOLVED IN: multicellular organismal development, regulation of transcription; LOCATED IN: cellular_component unknown; EXPRESSED IN: 18 plant structures; EXPRESSED DURING: 13 growth stages; CONTAINS InterPro DOMAIN/s: No apical meristem (NAM) protein (InterPro:IPR003441); BEST Arabidopsis thaliana protein match is: NAC (No Apical Meristem) domain transcriptional regulator superfamily protein (TAIR:AT1G76420.1)
AT5G59930	*-2,072*		Cysteine/Histidine-rich C1 domain family protein; FUNCTIONS IN: zinc ion binding; INVOLVED IN: intracellular signaling pathway; EXPRESSED IN: root; CONTAINS InterPro DOMAIN/s: Protein kinase C-like, phorbol ester/diacylglycerol binding (InterPro:IPR002219), DC1 (InterPro:IPR004146), Zinc finger, PHD-type (InterPro:IPR001965), Zinc finger, ZZ-type (InterPro:IPR000433), C1-like (InterPro:IPR011424); BEST Arabidopsis thaliana protein match is: Cysteine/Histidine-rich C1 domain family protein (TAIR:AT5G59940.1)
AT1G23720	*-2,078*		Proline-rich extensin-like family protein; FUNCTIONS IN: structural constituent of cell wall; INVOLVED IN: plant-type cell wall organization; EXPRESSED IN: hypocotyl, root, carpel; EXPRESSED DURING: petal differentiation and expansion stage; CONTAINS InterPro DOMAIN/s: Extensin-like repeat (InterPro:IPR006706); BEST Arabidopsis thaliana protein match is: Proline-rich extensin-like family protein (TAIR:AT4G13390.1)
AT1G63580	*-2,078*		Encodes a plasma membrane-localized protein with two DUF26 domains and a GPI anchor domain.
AT1G14960	*-2,079*		Polyketide cyclase/dehydrase and lipid transport superfamily protein; FUNCTIONS IN: molecular_function unknown; INVOLVED IN: response to biotic stimulus, defense response; LOCATED IN: cellular_component unknown; EXPRESSED IN: root; CONTAINS InterPro DOMAIN/s: Bet v I allergen (InterPro:IPR000916); BEST Arabidopsis thaliana protein match is: Polyketide cyclase/dehydrase and lipid transport superfamily protein (TAIR:AT1G14950.1)
AT1G31950	*-2,08*		Terpenoid cyclases/Protein prenyltransferases superfamily protein; FUNCTIONS IN: lyase activity, magnesium ion binding; INVOLVED IN: metabolic process; LOCATED IN: membrane; EXPRESSED IN: 7 plant structures; EXPRESSED DURING: LP.04 four leaves visible, LP.02 two leaves visible, petal differentiation and expansion stage; CONTAINS InterPro DOMAIN/s: Terpene synthase, metal-binding domain (InterPro:IPR005630), Terpenoid synthase (InterPro:IPR008949), Terpenoid cylases/protein prenyltransferase alpha-alpha toroid (InterPro:IPR008930), Terpene synthase-like (InterPro:IPR001906); BEST Arabidopsis thaliana protein match is: Terpenoid cyclases/Protein prenyltransferases superfamily protein (TAIR:AT3G14520.1)
AT5G18560	*-2,08*	(PUCHI)	Encodes PUCHI, a member of the ERF (ethylene response factor) subfamily B-1 of ERF/AP2 transcription factor family. The protein contains one AP2 domain. There are 15 members in this subfamily including ATERF-3, ATERF-4, ATERF-7, and leafy petiole. PUCHI is required for morphogenesis in the early lateral root primordium of Arabidopsis. Expressed in early floral meristem (stage 1 to 2). Required for early floral meristem growth and for bract suppression. Triple mutant with bop1 and bop2 displays a strong defect in the determination of floral meristem identity with reduced LFY expression and the lack of AP1 expression.
AT1G27140	*-2,084*	GLUTATHIONE S-TRANSFERASE TAU 14	Encodes glutathione transferase belonging to the tau class of GSTs. Naming convention according to Wagner et al. (2002).
AT2G45890	*-2,091*	ROP (RHO OF PLANTS) GUANINE NUCLEOTIDE EXCHANGE FACTOR 4 (ROPGEF4)	Encodes a member of KPP-like gene family, homolog of KPP (kinase partner protein) gene in tomato. Also a member of the RopGEF (guanine nucleotide exchange factor) family, containing the novel PRONE domain (plant-specific Rop nucleotide exchanger), which is exclusively active towards members of the Rop subfamily. Mutants exhibit longer root hairs under phosphate-deficient conditions.
AT4G11210	*-2,093*		Disease resistance-responsive (dirigent-like protein) family protein; FUNCTIONS IN: molecular_function unknown; INVOLVED IN: lignan biosynthetic process, defense response; LOCATED IN: endomembrane system; EXPRESSED IN: leaf lamina base, petal, hypocotyl, cauline leaf, root; EXPRESSED DURING: 4 anthesis; CONTAINS InterPro DOMAIN/s: Plant disease resistance response protein (InterPro:IPR004265); BEST Arabidopsis thaliana protein match is: Disease resistance-responsive (dirigent-like protein) family protein (TAIR:AT4G11190.1)

AT5G42610	-2,096		Protein of unknown function (DUF607); CONTAINS InterPro DOMAIN/s: Protein of unknown function DUF607 (InterPro:IPR006769); BEST Arabidopsis thaliana protein match is: Protein of unknown function (DUF607) (TAIR:AT2G23790.1)
AT5G50560	-2,097		Protein of unknown function (DUF 3339); FUNCTIONS IN: molecular_function unknown; INVOLVED IN: biological_process unknown; LOCATED IN: endomembrane system; CONTAINS InterPro DOMAIN/s: Protein of unknown function DUF3339 (InterPro:IPR021775); BEST Arabidopsis thaliana protein match is: Protein of unknown function (DUF 3339) (TAIR:AT5G50660.1)
AT2G44380	-2,101		Cysteine/Histidine-rich C1 domain family protein; CONTAINS InterPro DOMAIN/s: DC1 (InterPro:IPR004146), C1-like (InterPro:IPR011424); BEST Arabidopsis thaliana protein match is: Cysteine/Histidine-rich C1 domain family protein (TAIR:AT5G43520.1)
AT4G22640	-2,103		Bifunctional inhibitor/lipid-transfer protein/seed storage 2S albumin superfamily protein; CONTAINS InterPro DOMAIN/s: Bifunctional inhibitor/plant lipid transfer protein/seed storage (InterPro:IPR016140); BEST Arabidopsis thaliana protein match is: Bifunctional inhibitor/lipid-transfer protein/seed storage 2S albumin superfamily protein (TAIR:AT4G22666.2)
AT5G10230	-2,104	ANNEXIN 7 (ANNAT7)	Encodes a calcium-binding protein annexin (AnnAt7).
AT2G01520	-2,106	MLP-LIKE PROTEIN 328 (MLP328)	Encodes a cis-cinnamic acid responsive gene that is a member of the major latex protein-like gene family and plays a role in promoting vegetative growth and delaying ﬂowering. The mRNA is cell-to-cell mobile.
AT3G26290	-2,109	CYTOCHROME P450, FAMILY 71, SUBFAMILY B, POLYPEPTIDE 26 (CYP71B26)	putative cytochrome P450
AT5G65800	-2,11	ACC SYNTHASE 5 (ACS5)	1-aminocyclopropane-1-carboxylate synthase (ACS) is encoded by a multigene family consisting of at least five members whose expression is induced by hormones, developmental signals, and protein synthesis inhibition.
AT2G15370	-2,111	FUCOSYLTRANSFERASE 5 (FUT5)	Predicted fucosyltransferase, based on similarity to FUT1, but not functionally redundant with FUT1.
AT5G63270	-2,112		RPM1-interacting protein 4 (RIN4) family protein; CONTAINS InterPro DOMAIN/s: RPM1-interacting protein 4, defence response (InterPro:IPR008700); BEST Arabidopsis thaliana protein match is: RPM1-interacting protein 4 (RIN4) family protein (TAIR:AT3G48450.1)
AT4G25830	-2,113	CASP-LIKE PROTEIN 2C1 (CASPL2C1)	Uncharacterised protein family (UPF0497); FUNCTIONS IN: molecular_function unknown; INVOLVED IN: biological_process unknown; LOCATED IN: endomembrane system; EXPRESSED IN: 22 plant structures; EXPRESSED DURING: 13 growth stages; CONTAINS InterPro DOMAIN/s: Uncharacterised protein family UPF0497, trans-membrane plant (InterPro:IPR006702), Uncharacterised protein family UPF0497, trans-membrane plant subgroup (InterPro:IPR006459); BEST Arabidopsis thaliana protein match is: Uncharacterised protein family (UPF0497) (TAIR:AT2G35760.1)
AT4G40010	-2,113	SNF1-RELATED PROTEIN KINASE 2.7 (SNRK2.7)	encodes a member of SNF1-related protein kinases (SnRK2) whose activity is activated by ionic (salt) and non-ionic (mannitol) osmotic stress.
AT5G44610	-2,113	MICROTUBULE-ASSOCIATED PROTEIN 18 (MAP18)	Encodes a protein with seven repeated VEEKK motifs. RNAi and overexpression experiments suggest that the gene is not involved in cell division but might be consequential for cell shape of epidermal and cortical cells. The protein encoded by this gene binds to cortical microtubules and inhibits tubulin polymerization. Associates to the plasma membrane and interacts with calmodulin and phosphatidylinositol phosphates, indicating an involvement in cellular signal transduction. Expression is enhanced by abiotic and hormonal factors. Induced during senescence.Interacts with Ca2+/calmodulin complex, phosphatidylinositol phosphates, and free Ca2+.
AT5G66390	-2,113	PEROXIDASE 72 (PRX72)	Encodes a peroxidase that is involved in lignin biosynthesis.
AT5G05280	-2,118	DEFECTIVE IN ANTHER DEHISCENCE1- (DAD1-) ACTIVATING FACTOR (DAF)	Encodes a RING-finger E3 ligase protein that controls anther dehiscence by positively regulating the expression of DAD1 in the jasmonic acid biosynthesis pathway.
AT2G44220	-2,12		Protein of Unknown Function (DUF239); INVOLVED IN: biological_process unknown; LOCATED IN: endomembrane system; CONTAINS InterPro DOMAIN/s: Protein of unknown function DUF239, plant (InterPro:IPR004314); BEST Arabidopsis thaliana protein match is: Protein of Unknown Function (DUF239) (TAIR:AT2G44240.1)

AT4G30420	-2,12	USUALLY MULTIPLE ACIDS MOVE IN AND OUT TRANSPORTERS 34 (UMAMIT34)	nodulin MtN21-like transporter family protein
AT1G05280	-2,125		Protein of unknown function (DUF604); CONTAINS InterPro DOMAIN/s: Protein of unknown function DUF604 (InterPro:IPR006740); BEST Arabidopsis thaliana protein match is: Protein of unknown function (DUF604) (TAIR:AT4G15240.1)
AT2G15830	-2,125		unknown protein; FUNCTIONS IN: molecular_function unknown; INVOLVED IN: biological_process unknown; LOCATED IN: endomembrane system; EXPRESSED IN: 16 plant structures; EXPRESSED DURING: 10 growth stages; BEST Arabidopsis thaliana protein match is: unknown protein (TAIR:AT4G33960.1)
AT2G37460	-2,126	USUALLY MULTIPLE ACIDS MOVE IN AND OUT TRANSPORTERS 12 (UMAMIT12)	nodulin MtN21-like transporter family protein
AT1G17190	-2,136	GLUTATHIONE S-TRANSFERASE TAU 26	Encodes glutathione transferase belonging to the tau class of GSTs. Naming convention according to Wagner et al. (2002).
AT1G72200	-2,136		RING/U-box superfamily protein; FUNCTIONS IN: zinc ion binding; EXPRESSED IN: 13 plant structures; EXPRESSED DURING: LP.04 four leaves visible, 4 anthesis, petal differentiation and expansion stage, LP.08 eight leaves visible; CONTAINS InterPro DOMAIN/s: Zinc finger, RING-type (InterPro:IPR001841), Zinc finger, C3HC4 RING-type (InterPro:IPR018957); BEST Arabidopsis thaliana protein match is: RING/U-box superfamily protein (TAIR:AT1G22500.1)
AT2G17590	-2,137		Cysteine/Histidine-rich C1 domain family protein; CONTAINS InterPro DOMAIN/s: DC1 (InterPro:IPR004146), C1-like (InterPro:IPR011424); BEST Arabidopsis thaliana protein match is: Cysteine/Histidine-rich C1 domain family protein (TAIR:AT2G17600.1)
AT1G07460	-2,139		Concanavalin A-like lectin family protein; FUNCTIONS IN: carbohydrate binding, binding; INVOLVED IN: biological_process unknown; LOCATED IN: anchored to membrane; CONTAINS InterPro DOMAIN/s: Legume lectin, beta chain (InterPro:IPR001220), Concanavalin A-like lectin/glucanase, subgroup (InterPro:IPR013320), Concanavalin A-like lectin/glucanase (InterPro:IPR008985), Legume lectin, beta chain, Mn/Ca-binding site (InterPro:IPR019825); BEST Arabidopsis thaliana protein match is: Concanavalin A-like lectin protein kinase family protein (TAIR:AT2G29220.1)
AT1G43020	-2,143		Protein of unknown function, DUF547; CONTAINS InterPro DOMAIN/s: Protein of unknown function DUF547 (InterPro:IPR006869); BEST Arabidopsis thaliana protein match is: Protein of unknown function, DUF547 (TAIR:AT1G21060.1)
AT5G02890	-2,143		HXXXD-type acyl-transferase family protein; FUNCTIONS IN: transferase activity, transferring acyl groups other than amino-acyl groups, transferase activity; INVOLVED IN: biological_process unknown; LOCATED IN: cellular_component unknown; EXPRESSED IN: 21 plant structures; EXPRESSED DURING: 12 growth stages; CONTAINS InterPro DOMAIN/s: Transferase (InterPro:IPR003480); BEST Arabidopsis thaliana protein match is: HXXXD-type acyl-transferase family protein (TAIR:AT4G29250.1)
AT1G59940	-2,15	RESPONSE REGULATOR 3 (ARR3)	Type A response regulator highly similar to bacterial two-component response regulators. Rapidly induced by cytokinin. Involved in red-light signaling. Acts redundantly with ARR3 in the control of circadian period in a cytokinin-independent manner.
AT1G06090	-2,16		Fatty acid desaturase family protein; FUNCTIONS IN: oxidoreductase activity, oxidoreductase activity, acting on paired donors, with oxidation of a pair of donors resulting in the reduction of molecular oxygen to two molecules of water; INVOLVED IN: oxidation reduction, lipid metabolic process; LOCATED IN: cellular_component unknown; CONTAINS InterPro DOMAIN/s: Fatty acid desaturase, type 1, core (InterPro:IPR015876), Fatty acid desaturase, type 1 (InterPro:IPR005804); BEST Arabidopsis thaliana protein match is: Fatty acid desaturase family protein (TAIR:AT1G06120.1)
AT3G54590	-2,163	HYDROXYPROLINE-RICH GLYCOPROTEIN (HRGP1)	Encodes a hydroxyproline-rich glycoprotein. The mRNA is cell-to-cell mobile.

AT5G42180	*-2,163*	PEROXIDASE 64 (PER64)	Peroxidase superfamily protein; FUNCTIONS IN: peroxidase activity, heme binding; INVOLVED IN: oxidation reduction, response to oxidative stress; LOCATED IN: plant-type cell wall; EXPRESSED IN: 10 plant structures; EXPRESSED DURING: 4 anthesis, C globular stage, petal differentiation and expansion stage; CONTAINS InterPro DOMAIN/s: Haem peroxidase (InterPro:IPR010255), Plant peroxidase (InterPro:IPR000823), Peroxidases heam-ligand binding site (InterPro:IPR019793), Haem peroxidase, plant/fungal/bacterial (InterPro:IPR002016); BEST Arabidopsis thaliana protein match is: Peroxidase superfamily protein (TAIR:AT5G51890.1)
AT1G30990	*-2,168*		Polyketide cyclase/dehydrase and lipid transport superfamily protein; FUNCTIONS IN: molecular_function unknown; INVOLVED IN: response to biotic stimulus, defense response; LOCATED IN: cellular_component unknown; EXPRESSED IN: sperm cell, root; CONTAINS InterPro DOMAIN/s: Bet v I allergen (InterPro:IPR000916); BEST Arabidopsis thaliana protein match is: Polyketide cyclase/dehydrase and lipid transport superfamily protein (TAIR:AT1G14960.1)
AT3G05890	*-2,17*	RARE-COLD-INDUCIBLE 2B (RCI2B)	RARE-COLD-INDUCIBLE 2B (RCI2B); CONTAINS InterPro DOMAIN/s: Uncharacterised protein family UPF0057 (InterPro:IPR000612); BEST Arabidopsis thaliana protein match is: Low temperature and salt responsive protein family (TAIR:AT3G05880.1)
AT1G05650	*-2,171*		Pectin lyase-like superfamily protein; FUNCTIONS IN: polygalacturonase activity; INVOLVED IN: carbohydrate metabolic process; LOCATED IN: endomembrane system; CONTAINS InterPro DOMAIN/s: Pectin lyase fold/virulence factor (InterPro:IPR011050), Pectin lyase fold (InterPro:IPR012334), Glycoside hydrolase, family 28 (InterPro:IPR000743), Parallel beta-helix repeat (InterPro:IPR006626); BEST Arabidopsis thaliana protein match is: Pectin lyase-like superfamily protein (TAIR:AT1G05660.1)
AT5G14690	*-2,171*		unknown protein; BEST Arabidopsis thaliana protein match is: unknown protein (TAIR:AT3G01516.1)
AT4G10560	*-2,172*	MATERNAL EFFECT EMBRYO ARREST 53 (MEE53)	maternal effect embryo arrest 53 (MEE53); FUNCTIONS IN: zinc ion binding; INVOLVED IN: embryo development ending in seed dormancy; LOCATED IN: cellular_component unknown; CONTAINS InterPro DOMAIN/s: Zinc finger, RING-type (InterPro:IPR001841), DC1 (InterPro:IPR004146), C1-like (InterPro:IPR011424); BEST Arabidopsis thaliana protein match is: Cysteine/Histidine-rich C1 domain family protein (TAIR:AT4G02540.1)
AT5G01250	*-2,174*		alpha 1,4-glycosyltransferase family protein; FUNCTIONS IN: transferase activity, transferring glycosyl groups, transferase activity; INVOLVED IN: biological_process unknown; LOCATED IN: Golgi stack; CONTAINS InterPro DOMAIN/s: Alpha 1,4-glycosyltransferase conserved region (InterPro:IPR007652), Glycosyltransferase, DXD sugar-binding region (InterPro:IPR007577); BEST Arabidopsis thaliana protein match is: alpha 1,4-glycosyltransferase family protein (TAIR:AT3G09020.1)
AT1G03660	*-2,175*		Ankyrin-repeat containing protein; BEST Arabidopsis thaliana protein match is: ankyrin repeat family protein (TAIR:AT1G03670.1)
AT4G09100	*-2,177*		RING/U-box superfamily protein; FUNCTIONS IN: zinc ion binding; LOCATED IN: endomembrane system; EXPRESSED IN: stem, root; CONTAINS InterPro DOMAIN/s: Zinc finger, RING-type (InterPro:IPR001841), Zinc finger, C3HC4 RING-type (InterPro:IPR018957); BEST Arabidopsis thaliana protein match is: RING/U-box superfamily protein (TAIR:AT2G35000.1)
AT4G08570	*-2,178*		Heavy metal transport/detoxification superfamily protein ; FUNCTIONS IN: metal ion binding; INVOLVED IN: metal ion transport; LOCATED IN: cellular_component unknown; CONTAINS InterPro DOMAIN/s: Heavy metal transport/detoxification protein (InterPro:IPR006121), Heavy-metal-associated, conserved site (InterPro:IPR017969); BEST Arabidopsis thaliana protein match is: Heavy metal transport/detoxification superfamily protein (TAIR:AT1G22990.1)
AT2G44340	*-2,179*		VQ motif-containing protein; CONTAINS InterPro DOMAIN/s: VQ (InterPro:IPR008889); BEST Arabidopsis thaliana protein match is: VQ motif-containing protein (TAIR:AT3G60090.1)
AT2G43590	*-2,186*		Chitinase family protein; FUNCTIONS IN: chitin binding, chitinase activity; INVOLVED IN: carbohydrate metabolic process, cell wall macromolecule catabolic process; LOCATED IN: endomembrane system; EXPRESSED IN: 7 plant structures; EXPRESSED DURING: LP.04 four leaves visible, 4 anthesis, C globular stage, LP.02 two leaves visible; CONTAINS InterPro DOMAIN/s: Chitin-binding, type 1, conserved site (InterPro:IPR018371), Glycoside hydrolase, family 19 (InterPro:IPR016283), Chitin-binding, type 1 (InterPro:IPR001002), Glycoside hydrolase, family 19, catalytic (InterPro:IPR000726); BEST Arabidopsis thaliana protein match is: Chitinase family protein (TAIR:AT2G43580.1)

AT5G36180	*-2,186*	SERINE CARBOXYPEPTIDASE-LIKE 1 (scpl1)	serine carboxypeptidase-like 1 (scpl1); FUNCTIONS IN: serine-type carboxypeptidase activity; INVOLVED IN: proteolysis; LOCATED IN: endomembrane system; CONTAINS InterPro DOMAIN/s: Peptidase S10, serine carboxypeptidase (InterPro:IPR001563); BEST Arabidopsis thaliana protein match is: serine carboxypeptidase-like 2 (TAIR:AT1G73300.1)
AT2G02300	*-2,193*	PHLOEM PROTEIN 2-B5 (PP2-B5)	phloem protein 2-B5 (PP2-B5); FUNCTIONS IN: carbohydrate binding; INVOLVED IN: N-terminal protein myristoylation; LOCATED IN: cellular_component unknown; EXPRESSED IN: carpel; EXPRESSED DURING: 4 anthesis; CONTAINS InterPro DOMAIN/s: F-box domain, Skp2-like (InterPro:IPR022364); BEST Arabidopsis thaliana protein match is: phloem protein 2-B6 (TAIR:AT2G02310.1)
AT5G43230	*-2,193*		unknown protein; BEST Arabidopsis thaliana protein match is: unknown protein (TAIR:AT3G01810.3)
AT5G61550	*-2,198*		U-box domain-containing protein kinase family protein; FUNCTIONS IN: ubiquitin-protein ligase activity, protein serine/threonine kinase activity, protein kinase activity, kinase activity, ATP binding; INVOLVED IN: protein amino acid phosphorylation, protein ubiquitination; LOCATED IN: ubiquitin ligase complex; CONTAINS InterPro DOMAIN/s: U box domain (InterPro:IPR003613), Serine/threonine-protein kinase domain (InterPro:IPR002290), Serine/threonine-protein kinase-like domain (InterPro:IPR017442), Serine/threonine-protein kinase, active site (InterPro:IPR008271), Protein kinase-like domain (InterPro:IPR011009), Protein kinase, catalytic domain (InterPro:IPR000719), Tyrosine-protein kinase, catalytic domain (InterPro:IPR020635); BEST Arabidopsis thaliana protein match is: U-box domain-containing protein kinase family protein (TAIR:AT4G25160.1).
AT5G37990	*-2,199*	(CIMT1)	S-adenosyl-L-methionine-dependent methyltransferases superfamily protein; FUNCTIONS IN: S-adenosylmethionine-dependent methyltransferase activity, methyltransferase activity; INVOLVED IN: biological_process unknown; LOCATED IN: endomembrane system; EXPRESSED IN: hypocotyl, root; CONTAINS InterPro DOMAIN/s: SAM dependent carboxyl methyltransferase (InterPro:IPR005299); BEST Arabidopsis thaliana protein match is: S-adenosyl-L-methionine-dependent methyltransferases superfamily protein (TAIR:AT5G37970.1)
AT1G01750	*-2,2*	ACTIN DEPOLYMERIZING FACTOR 11 (ADF11)	actin depolymerizing factor 11 (ADF11); FUNCTIONS IN: actin binding; INVOLVED IN: biological_process unknown; LOCATED IN: intracellular; EXPRESSED IN: 6 plant structures; EXPRESSED DURING: 4 anthesis, petal differentiation and expansion stage; CONTAINS InterPro DOMAIN/s: Actin-binding, cofilin/tropomyosin type (InterPro:IPR002108); BEST Arabidopsis thaliana protein match is: actin depolymerizing factor 8 (TAIR:AT4G00680.1)
AT5G14750	*-2,204*	MYB DOMAIN PROTEIN 66 (MYB66)	Encodes a MyB-related protein containing R2 and R3 repeats, involved in root and hypocotyl epidermal cell fate determination. Loss of function mutations make extra root hairs. Nuclear localized protein is a positive regulator for expression of CAPRICE (CPC).
AT5G14020	*-2,207*		Endosomal targeting BRO1-like domain-containing protein; INVOLVED IN: N-terminal protein myristoylation; LOCATED IN: vacuole; EXPRESSED IN: root; CONTAINS InterPro DOMAIN/s: BRO1 (InterPro:IPR004328); BEST Arabidopsis thaliana protein match is: Endosomal targeting BRO1-like domain-containing protein (TAIR:AT1G73390.3)
AT2G21210	*-2,208*	SMALL AUXIN UPREGULATED RNA 6 (SAUR6)	Putative auxin-regulated protein whose expression is downregulated in response to chitin oligomers.
AT1G11600	*-2,209*	CYTOCHROME P450, FAMILY 77, SUBFAMILY B, POLYPEPTIDE 1 (CYP77B1)	member of CYP77B
AT2G43890	*-2,209*		Pectin lyase-like superfamily protein; FUNCTIONS IN: polygalacturonase activity; INVOLVED IN: carbohydrate metabolic process; LOCATED IN: endomembrane system; EXPRESSED IN: root; CONTAINS InterPro DOMAIN/s: Pectin lyase fold/virulence factor (InterPro:IPR011050), Pectin lyase fold (InterPro:IPR012334), Glycoside hydrolase, family 28 (InterPro:IPR000743), Parallel beta-helix repeat (InterPro:IPR006626); BEST Arabidopsis thaliana protein match is: Pectin lyase-like superfamily protein (TAIR:AT1G05650.1)
AT1G71050	*-2,21*	HEAVY METAL ASSOCIATED ISOPRENYLATED PLANT PROTEIN 20 (HIPP20)	Heavy metal transport/detoxification superfamily protein ; FUNCTIONS IN: metal ion binding; INVOLVED IN: metal ion transport; LOCATED IN: cellular_component unknown; EXPRESSED IN: 19 plant structures; EXPRESSED DURING: 13 growth stages; CONTAINS InterPro DOMAIN/s: Heavy metal transport/detoxification protein (InterPro:IPR006121); BEST Arabidopsis thaliana protein match is: Heavy metal transport/detoxification superfamily protein (TAIR:AT1G22990.1)

AT5G40730	*-2,211*	ARABINOGALACTAN PROTEIN 24 (AGP24)	Encodes an arabinogalactan-protein (AGP24).
AT1G09090	*-2,216*	RESPIRATORY BURST OXIDASE HOMOLOG B (RBOHB)	NADPH-oxidase AtrbohB plays a role in seed after-ripening. Major producer of superoxide in germinating seeds. AtrbohB pre-mRNA is alternatively spliced in seeds in a hormonally and developmentally regulated manner. ABA caused accumulation of AtrbohB-? mRNA and prevented prevented AtrbohB-a mRNA expression in fresh seeds.
AT4G17785	*-2,218*	MYB DOMAIN PROTEIN 39 (MYB39)	Encodes a putative transcription factor (MYB39).
AT5G60060	*-2,22*		CONTAINS InterPro DOMAIN/s: F-box domain, cyclin-like (InterPro:IPR001810), Protein of unknown function DUF295 (InterPro:IPR005174); BEST Arabidopsis thaliana protein match is: F-box family protein with a domain of unknown function (DUF295) (TAIR:AT2G17030.1)
AT5G42630	*-2,221*	ABERRANT TESTA SHAPE (ATS)	Encodes a member of the KANADI family of putative transcription factors. Involved in integument formation during ovule development and expressed at the boundary between the inner and outer integuments. It is essential for directing laminar growth of the inner integument.Along with KAN1 and KAN2, KAN4 is involved in proper localization of PIN1 in early embryogenesis.
AT5G54700	*-2,223*		Ankyrin repeat family protein; CONTAINS InterPro DOMAIN/s: Ankyrin repeat-containing domain (InterPro:IPR020683), Ankyrin repeat (InterPro:IPR002110); BEST Arabidopsis thaliana protein match is: Ankyrin repeat family protein (TAIR:AT5G54710.1)
AT5G55110	*-2,224*		Stigma-specific Stig1 family protein; FUNCTIONS IN: molecular_function unknown; INVOLVED IN: biological_process unknown; LOCATED IN: endomembrane system; CONTAINS InterPro DOMAIN/s: Stigma-specific protein Stig1 (InterPro:IPR006969); BEST Arabidopsis thaliana protein match is: Stigma-specific Stig1 family protein (TAIR:AT4G26880.1)
AT2G39530	*-2,227*	CASP-LIKE PROTEIN 4D1 (CASPL4D1)	Uncharacterised protein family (UPF0497); CONTAINS InterPro DOMAIN/s: Uncharacterised protein family UPF0497, trans-membrane plant (InterPro:IPR006702), Uncharacterised protein family UPF0497, trans-membrane plant subgroup (InterPro:IPR006459); BEST Arabidopsis thaliana protein match is: Uncharacterised protein family (UPF0497) (TAIR:AT2G39518.1)
AT3G20940	*-2,229*	CYTOCHROME P450, FAMILY 705, SUBFAMILY A, POLYPEPTIDE 30 (CYP705A30)	a member of A-type cytochrome P450
AT2G22630	*-2,231*	AGAMOUS-LIKE 17 (AGL17)	Encodes a MADs domain containing protein involved in promoting flowering. Loss of function mutations show delayed flowering in long days and reduced levels of LFY and AP1 expression.
AT2G19060	*-2,234*		SGNH hydrolase-type esterase superfamily protein; FUNCTIONS IN: lipase activity, hydrolase activity, acting on ester bonds; INVOLVED IN: lipid metabolic process; LOCATED IN: endomembrane system; EXPRESSED IN: root; CONTAINS InterPro DOMAIN/s: Lipase, GDSL (InterPro:IPR001087), Esterase, SGNH hydrolase-type (InterPro:IPR013830); BEST Arabidopsis thaliana protein match is: GDSL-like Lipase/Acylhydrolase superfamily protein (TAIR:AT2G19050.1)
AT3G49760	*-2,236*	BASIC LEUCINE-ZIPPER 5 (bZIP5)	basic leucine-zipper 5 (bZIP5); FUNCTIONS IN: DNA binding, sequence-specific DNA binding transcription factor activity; INVOLVED IN: regulation of transcription, DNA-dependent; LOCATED IN: nucleus; EXPRESSED IN: root stele, pericycle, root, primary root elongation zone, primary root differentiation zone; CONTAINS InterPro DOMAIN/s: Basic-leucine zipper (bZIP) transcription factor (InterPro:IPR004827), Basic leucine zipper (InterPro:IPR011700); BEST Arabidopsis thaliana protein match is: basic leucine-zipper 6 (TAIR:AT2G22850.2)
AT1G02570	*-2,239*		unknown protein; BEST Arabidopsis thaliana protein match is: unknown protein (TAIR:AT1G02575.1)
AT4G25250	*-2,239*	PECTINMETHYLESTERASE INHIBITOR 4 (PMEI4)	Plant invertase/pectin methylesterase inhibitor superfamily protein; FUNCTIONS IN: enzyme inhibitor activity, pectinesterase inhibitor activity, pectinesterase activity; INVOLVED IN: biological_process unknown; EXPRESSED IN: root; CONTAINS InterPro DOMAIN/s: Pectinesterase inhibitor (InterPro:IPR006501); BEST Arabidopsis thaliana protein match is: Plant invertase/pectin methylesterase inhibitor superfamily protein (TAIR:AT5G51520.1)
AT5G40860	*-2,24*		unknown protein; BEST Arabidopsis thaliana protein match is: unknown protein (TAIR:AT3G27210.1)

AT4G26770	*-2,242*		Phosphatidate cytidylyltransferase family protein; FUNCTIONS IN: phosphatidate cytidylyltransferase activity; INVOLVED IN: phospholipid biosynthetic process; LOCATED IN: membrane; EXPRESSED IN: petal, leaf whorl, flower, pollen tube; EXPRESSED DURING: 4 anthesis, petal differentiation and expansion stage; CONTAINS InterPro DOMAIN/s: Phosphatidate cytidylyltransferase (InterPro:IPR000374), Phosphatidate cytidylyltransferase, eukaryota (InterPro:IPR016720); BEST Arabidopsis thaliana protein match is: CDP-diacylglycerol synthase 1 (TAIR:AT1G62430.1)
AT5G03640	*-2,242*		Protein kinase superfamily protein; FUNCTIONS IN: kinase activity; INVOLVED IN: protein amino acid phosphorylation, N-terminal protein myristoylation; LOCATED IN: cellular_component unknown; EXPRESSED IN: stem, inflorescence meristem, male gametophyte; CONTAINS InterPro DOMAIN/s: Protein kinase, catalytic domain (InterPro:IPR000719), Serine/threonine-protein kinase domain (InterPro:IPR002290), Serine/threonine-protein kinase-like domain (InterPro:IPR017442), Protein kinase-like domain (InterPro:IPR011009), Serine/threonine-protein kinase, active site (InterPro:IPR008271); BEST Arabidopsis thaliana protein match is: KCBP-interacting protein kinase (TAIR:AT3G52890.2)
AT1G30750	*-2,246*		unknown protein; FUNCTIONS IN: molecular_function unknown; INVOLVED IN: biological_process unknown; LOCATED IN: endomembrane system; EXPRESSED IN: hypocotyl, root, pollen tube
AT5G19340	*-2,248*		unknown protein; BEST Arabidopsis thaliana protein match is: unknown protein (TAIR:AT3G05980.1)
AT4G14130	*-2,26*	XYLOGLUCAN ENDOTRANSGLUCOSYLASE/HYDROLASE 15 (XTH15)	xyloglucan endotransglycosylase-related protein (XTR7)
AT2G16970	*-2,262*	MATERNAL EFFECT EMBRYO ARREST 15 (MEE15)	maternal effect embryo arrest 15 (MEE15); FUNCTIONS IN: tetracycline transporter activity; INVOLVED IN: embryo development ending in seed dormancy; LOCATED IN: integral to membrane; EXPRESSED IN: 22 plant structures; EXPRESSED DURING: 13 growth stages; CONTAINS InterPro DOMAIN/s: Tetracycline resistance protein, TetA (InterPro:IPR001958), Major facilitator superfamily MFS-1 (InterPro:IPR011701), Major facilitator superfamily, general substrate transporter (InterPro:IPR016196); BEST Arabidopsis thaliana protein match is: Major facilitator superfamily protein (TAIR:AT2G16980.2)
AT1G54890	*-2,263*		Late embryogenesis abundant (LEA) protein-related; FUNCTIONS IN: molecular_function unknown; INVOLVED IN: biological_process unknown; LOCATED IN: endomembrane system; EXPRESSED IN: 7 plant structures; EXPRESSED DURING: 4 anthesis, petal differentiation and expansion stage; CONTAINS InterPro DOMAIN/s: Root cap (InterPro:IPR009646); BEST Arabidopsis thaliana protein match is: Late embryogenesis abundant (LEA) protein-related (TAIR:AT5G54370.1)
AT1G62280	*-2,263*	SLAC1 HOMOLOGUE 1 (SLAH1)	Encodes a protein with ten predicted transmembrane helices. The SLAH1 protein has similarity to the SLAC1 protein involved in ion homeostasis in guard cells. Although it is not expressed in guard cells, it can complement a slac1-2 mutant suggesting that it performs a similar function. SLAH1:GFP localizes to the plasma membrane.
AT4G29180	*-2,263*	ROOT HAIR SPECIFIC 16 (RHS16)	root hair specific 16 (RHS16); FUNCTIONS IN: kinase activity; INVOLVED IN: protein amino acid phosphorylation; LOCATED IN: endomembrane system; EXPRESSED IN: root hair; CONTAINS InterPro DOMAIN/s: Protein kinase, ATP binding site (InterPro:IPR017441), Protein kinase, catalytic domain (InterPro:IPR000719), Leucine-rich repeat (InterPro:IPR001611), Serine-threonine/tyrosine-protein kinase (InterPro:IPR001245), Protein kinase-like domain (InterPro:IPR011009), Serine/threonine-protein kinase, active site (InterPro:IPR008271); BEST Arabidopsis thaliana protein match is: Leucine-rich repeat protein kinase family protein (TAIR:AT4G29450.1).
AT2G41810	*-2,267*		FUNCTIONS IN: molecular_function unknown; INVOLVED IN: biological_process unknown; LOCATED IN: endomembrane system; EXPRESSED IN: root; CONTAINS InterPro DOMAIN/s: Protein of unknown function DUF642 (InterPro:IPR006946), Galactose-binding domain-like (InterPro:IPR008979); BEST Arabidopsis thaliana protein match is: Protein of unknown function, DUF642 (TAIR:AT2G41800.1)
AT5G60530	*-2,267*		late embryogenesis abundant protein-related / LEA protein-related; FUNCTIONS IN: molecular_function unknown; LOCATED IN: endomembrane system; EXPRESSED IN: root; CONTAINS InterPro DOMAIN/s: Root cap (InterPro:IPR009646); BEST Arabidopsis thaliana protein match is: Late embryogenesis abundant (LEA) protein-related (TAIR:AT5G60520.1)
AT1G70460	*-2,269*	PROLINE-RICH EXTENSIN-LIKE RECEPTOR KINASE 13 (PERK13)	Encodes a member of the proline-rich extensin-like receptor kinase (PERK) family. This family consists of 15 predicted receptor kinases (PMID: 15653807).

AT1G53610	*-2,272*		unknown protein; FUNCTIONS IN: molecular_function unknown; INVOLVED IN: biological_process unknown; LOCATED IN: endomembrane system; BEST Arabidopsis thaliana protein match is: unknown protein (TAIR:AT1G53620.1)
AT2G21540	*-2,273*	SEC14-LIKE 3 (SFH3)	SEC14-like 3 (SFH3); FUNCTIONS IN: phosphatidylinositol transporter activity; INVOLVED IN: flower development, transport; LOCATED IN: intracellular; EXPRESSED IN: 25 plant structures; EXPRESSED DURING: 17 growth stages; CONTAINS InterPro DOMAIN/s: Cellular retinaldehyde-binding/triple function, C-terminal (InterPro:IPR001251), Cellular retinaldehyde-binding/triple function, N-terminal (InterPro:IPR008273), Cellular retinaldehyde binding/alpha-tocopherol transport (InterPro:IPR001071), Phosphatidylinositol transfer protein-like, N-terminal (InterPro:IPR011074); BEST Arabidopsis thaliana protein match is: Sec14p-like phosphatidylinositol transfer family protein (TAIR:AT4G39180.2)
AT1G70890	*-2,275*	MLP-LIKE PROTEIN 43 (MLP43)	MLP-like protein 43 (MLP43); FUNCTIONS IN: molecular_function unknown; INVOLVED IN: response to biotic stimulus, defense response; LOCATED IN: chloroplast; EXPRESSED IN: 17 plant structures; EXPRESSED DURING: 10 growth stages; CONTAINS InterPro DOMAIN/s: Bet v I allergen (InterPro:IPR000916); BEST Arabidopsis thaliana protein match is: MLP-like protein 28 (TAIR:AT1G70830.5)
AT2G35990	*-2,275*	LONELY GUY 2 (LOG2)	Putative lysine decarboxylase family protein; CONTAINS InterPro DOMAIN/s: Conserved hypothetical protein CHP00730 (InterPro:IPR005269); BEST Arabidopsis thaliana protein match is: Putative lysine decarboxylase family protein (TAIR:AT5G06300.1)
AT1G70450	*-2,277*		Its expression is enriched in root hair cells (compared to non-root hair cells) and this enrichment is associated with increase in the transcription-associated mark trimethylation of H3 lysine 4 (H3K4me3) and decrease in the Polycomb silencing-associated mark trimethylation of H3 lysine 27 (H3K27me3) in root hair cells relative to non-root hair cells.
AT1G49470	*-2,279*		Family of unknown function (DUF716) ; FUNCTIONS IN: molecular_function unknown; INVOLVED IN: biological_process unknown; LOCATED IN: endomembrane system; EXPRESSED IN: 20 plant structures; EXPRESSED DURING: 10 growth stages; CONTAINS InterPro DOMAIN/s: Protein of unknown function DUF716 (InterPro:IPR006904); BEST Arabidopsis thaliana protein match is: Family of unknown function (DUF716) (TAIR:AT1G55230.1)
AT4G31470	*-2,279*		CAP (Cysteine-rich secretory proteins, Antigen 5, and Pathogenesis-related 1 protein) superfamily protein; FUNCTIONS IN: molecular_function unknown; INVOLVED IN: biological_process unknown; LOCATED IN: endomembrane system, extracellular region; EXPRESSED IN: root; CONTAINS InterPro DOMAIN/s: Allergen V5/Tpx-1 related, conserved site (InterPro:IPR018244), Allergen V5/Tpx-1 related (InterPro:IPR001283), Ves allergen (InterPro:IPR002413), SCP-like extracellular (InterPro:IPR014044); BEST Arabidopsis thaliana protein match is: CAP (Cysteine-rich secretory proteins, Antigen 5, and Pathogenesis-related 1 protein) superfamily protein (TAIR:AT5G57625.1)
AT2G36100	*-2,282*	CASPARIAN STRIP MEMBRANE DOMAIN PROTEIN 1 (CASP1)	Encodes a membrane bound protein involved in formation of the casparian strip. Along with CASP 2 it is required for the localization of ESB1.
AT2G47540	*-2,287*		Pollen Ole e 1 allergen and extensin family protein; FUNCTIONS IN: molecular_function unknown; INVOLVED IN: biological_process unknown; LOCATED IN: endomembrane system; EXPRESSED IN: 6 plant structures; EXPRESSED DURING: petal differentiation and expansion stage; CONTAINS InterPro DOMAIN/s: Pollen Ole e 1 allergen/extensin (InterPro:IPR006041); BEST Arabidopsis thaliana protein match is: root hair specific 13 (TAIR:AT4G02270.1)
AT3G53420	*-2,287*	PLASMA MEMBRANE INTRINSIC PROTEIN 2A (PIP2A)	a member of the plasma membrane intrinsic protein subfamily PIP2. localizes to the plasma membrane and exhibits water transport activity in Xenopus oocyte. expressed specifically in the vascular bundles and protein level increases slightly during leaf dev. When expressed in yeast cells can conduct hydrogen peroxide into those cells.
AT4G30670	*-2,287*		Putative membrane lipoprotein; FUNCTIONS IN: molecular_function unknown; INVOLVED IN: biological_process unknown; LOCATED IN: endomembrane system; EXPRESSED IN: 11 plant structures; EXPRESSED DURING: 4 anthesis, petal differentiation and expansion stage
AT5G02000	*-2,287*		unknown protein

AT2G16980	-2,288		Major facilitator superfamily protein; FUNCTIONS IN: tetracycline transporter activity; INVOLVED IN: response to antibiotic, tetracycline transport, transmembrane transport; LOCATED IN: endomembrane system, integral to membrane; EXPRESSED IN: 6 plant structures; EXPRESSED DURING: L mature pollen stage, 4 anthesis; CONTAINS InterPro DOMAIN/s: Tetracycline resistance protein, TetA (InterPro:IPR001958), Major facilitator superfamily MFS-1 (InterPro:IPR011701), Major facilitator superfamily, general substrate transporter (InterPro:IPR016196); BEST Arabidopsis thaliana protein match is: Major facilitator superfamily protein (TAIR:AT2G16970.1)
AT5G61650	-2,288	CYCLIN P4;2 (CYCP4;2)	The P-type cyclins (CYCPs) share a conserved central region of 100 amino acids ('cyclin box') displaying homology to the corresponding region of the PHO80 cyclin from Saccharomyces cerevisiae and the related G1 cyclins from Trypanosoma cruzi and T. brucei.
AT1G15460	-2,292	REQUIRES HIGH BORON 4 (BOR4)	Encodes a efflux-type boron transporter. Over-expression improved plant growth under B toxic conditions.
AT2G39200	-2,293	MILDEW RESISTANCE LOCUS O 12 (MLO12)	A member of a large family of seven-transmembrane domain proteins specific to plants, homologs of the barley mildew resistance locus o (MLO) protein. The Arabidopsis genome contains 15 genes encoding MLO proteins, with localization in plasma membrane. Phylogenetic analysis revealed four clades of closely-related AtMLO genes. ATMLO6 belongs to the clade IV, with AtMLO2, AtMLO3 and AtMLO12. The gene is expressed during early seedling growth, in root tips and cotyledon vascular system, in floral organs (anthers and stigma), and in fruit abscission zone, as shown by GUS activity patterns. The expression of several phylogenetically closely-related AtMLO genes showed similar or overlapping tissue specificity and analogous responsiveness to external stimuli, suggesting functional redundancy, co-function, or antagonistic function(s).
AT1G51880	-2,296	ROOT HAIR SPECIFIC 6 (RHS6)	root hair specific 6 (RHS6); FUNCTIONS IN: kinase activity; INVOLVED IN: protein amino acid phosphorylation; LOCATED IN: endomembrane system; EXPRESSED IN: root hair; CONTAINS InterPro DOMAIN/s: Protein kinase, ATP binding site (InterPro:IPR017441), Serine/threonine-protein kinase domain (InterPro:IPR002290), Leucine-rich repeat (InterPro:IPR001611), Serine-threonine/tyrosine-protein kinase (InterPro:IPR001245), Protein kinase-like domain (InterPro:IPR011009), Serine/threonine-protein kinase, active site (InterPro:IPR008271), Protein kinase, catalytic domain (InterPro:IPR000719), Tyrosine-protein kinase, catalytic domain (InterPro:IPR020635); BEST Arabidopsis thaliana protein match is: Leucine-rich repeat protein kinase family protein (TAIR:AT1G51860.1)
AT4G01240	-2,297		S-adenosyl-L-methionine-dependent methyltransferases superfamily protein; CONTAINS InterPro DOMAIN/s: Protein of unknown function DUF248, methyltransferase putative (InterPro:IPR004159); BEST Arabidopsis thaliana protein match is: unknown protein (TAIR:AT3G05390.1)
AT4G26320	-2,297	ARABINOGALACTAN PROTEIN 13 (AGP13)	arabinogalactan protein 13 (AGP13); BEST Arabidopsis thaliana protein match is: arabinogalactan protein 14 (TAIR:AT5G56540.1)
AT4G35060	-2,297	HEAVY METAL ASSOCIATED ISOPRENYLATED PLANT PROTEIN 25 (HIPP25)	Heavy metal transport/detoxification superfamily protein ; FUNCTIONS IN: metal ion binding; INVOLVED IN: metal ion transport; LOCATED IN: cellular_component unknown; EXPRESSED IN: 14 plant structures; EXPRESSED DURING: LP.06 six leaves visible, LP.04 four leaves visible, 4 anthesis, C globular stage, petal differentiation and expansion stage; CONTAINS InterPro DOMAIN/s: Heavy metal transport/detoxification protein (InterPro:IPR006121); BEST Arabidopsis thaliana protein match is: farnesylated protein 6 (TAIR:AT4G38580.1)
AT5G56870	-2,304	BETA-GALACTOSIDASE 4 (BGAL4)	beta-galactosidase 4 (BGAL4); FUNCTIONS IN: beta-galactosidase activity; INVOLVED IN: lactose catabolic process, using glucoside 3-dehydrogenase, carbohydrate metabolic process, lactose catabolic process via UDP-galactose, lactose catabolic process; LOCATED IN: endomembrane system; EXPRESSED IN: 25 plant structures; EXPRESSED DURING: 12 growth stages; CONTAINS InterPro DOMAIN/s: Glycoside hydrolase, family 35, conserved site (InterPro:IPR019801), Glycoside hydrolase, family 35 (InterPro:IPR001944), Glycoside hydrolase, catalytic core (InterPro:IPR017853), Glycoside hydrolase, subgroup, catalytic core (InterPro:IPR013781), Galactose-binding domain-like (InterPro:IPR008979); BEST Arabidopsis thaliana protein match is: beta-galactosidase 12 (TAIR:AT4G26140.1)

AT2G33880	*-2,311*	HOMEOBOX-3 (HB-3)	Encodes a protein with similarity to WUS type homeodomain protein. Required for meristem growth and development and acts through positive regulation of WUS. Loss of function phenotypes include embryo lethality, hyponastic cotyledons, reduced root development and smaller meristems. Phenotypes can be rescued by addition of sucrose in the growth media. Overexpression can partially rescue the triple mutant cytokinin receptor phenotype suggesting HB-3 is a downstream effector of cytokinin signaling.
AT4G21250	*-2,311*		Sulfite exporter TauE/SafE family protein; FUNCTIONS IN: molecular_function unknown; INVOLVED IN: biological_process unknown; LOCATED IN: endomembrane system, integral to membrane; CONTAINS InterPro DOMAIN/s: Protein of unknown function DUF81 (InterPro:IPR002781); BEST Arabidopsis thaliana protein match is: Sulfite exporter TauE/SafE family protein (TAIR:AT4G21260.1)
AT1G54940	*-2,317*	PLANT GLYCOGENIN-LIKE STARCH INITIATION PROTEIN 4 (PGSIP4)	Encodes a xylan glucuronosyltransferase.
AT3G21180	*-2,323*	AUTOINHIBITED CA(2+)-ATPASE 9 (ACA9)	one of the type IIB calcium pump isoforms. encodes an autoinhibited Ca(2+)-ATPase that contains an N-terminal calmodulin binding autoinhibitory domain.
AT3G62680	*-2,327*	PROLINE-RICH PROTEIN 3	Proline-rich protein The mRNA is cell-to-cell mobile.
AT5G19790	*-2,333*	RELATED TO AP2 11 (RAP2.11)	encodes a member of the ERF (ethylene response factor) subfamily B-6 of ERF/AP2 transcription factor family (RAP2.11). The protein contains one AP2 domain. There are 12 members in this subfamily including RAP2.11.
AT1G26700	*-2,342*	MILDEW RESISTANCE LOCUS O 14 (MLO14)	A member of a large family of seven-transmembrane domain proteins specific to plants, homologs of the barley mildew resistance locus o (MLO) protein. The Arabidopsis genome contains 15 genes encoding MLO proteins, with localization in plasma membrane. Phylogenetic analysis revealed four clades of closely-related AtMLO genes. ATMLO14 belongs to the clade I, with AtMLO4 and AtMLO11. The gene is expressed during early seedling growth, in developing primary root, and particularly in root tips of 10-day old seedlings; it was not expressed in leaves or flowers, as shown by GUS activity patterns. The expression of several phylogenetically closely-related AtMLO genes showed similar or overlapping tissue specificity and analogous responsiveness to external stimuli, suggesting functional redundancy, co-function, or antagonistic function(s).
AT2G47270	*-2,343*	UPBEAT1 (UPB1)	Encodes UPBEAT1 (UPB1), a transcription factor with a bHLH domain. Regulates the expression of a set of peroxidases that modulate the balance of reactive oxygen species (ROS) between the zones of cell proliferation and the zone of cell elongation where differentiation begins. Disruption of UPB1 activity alters this ROS balance, leading to a delay in the onset of differentiation.
AT1G06930	*-2,346*		unknown protein
AT2G34000	*-2,346*		RING/U-box superfamily protein; FUNCTIONS IN: zinc ion binding; LOCATED IN: endomembrane system; CONTAINS InterPro DOMAIN/s: Zinc finger, RING-type (InterPro:IPR001841), Zinc finger, C3HC4 RING-type (InterPro:IPR018957); BEST Arabidopsis thaliana protein match is: RING/U-box superfamily protein (TAIR:AT3G05200.1)
AT4G31320	*-2,347*	SMALL AUXIN UPREGULATED RNA 37 (SAUR37)	SAUR-like auxin-responsive protein family ; CONTAINS InterPro DOMAIN/s: Auxin responsive SAUR protein (InterPro:IPR003676); BEST Arabidopsis thaliana protein match is: SAUR-like auxin-responsive protein family (TAIR:AT2G24400.1)
AT5G07080	*-2,352*		HXXXD-type acyl-transferase family protein; FUNCTIONS IN: transferase activity, transferring acyl groups other than amino-acyl groups, transferase activity; INVOLVED IN: biological_process unknown; LOCATED IN: cellular_component unknown; EXPRESSED IN: 18 plant structures; EXPRESSED DURING: 9 growth stages; CONTAINS InterPro DOMAIN/s: Transferase (InterPro:IPR003480); BEST Arabidopsis thaliana protein match is: HXXXD-type acyl-transferase family protein (TAIR:AT3G47170.1)
AT1G51470	*-2,353*	BETA GLUCOSIDASE 35 (BGLU35)	Encodes a myrosinase.
AT1G12040	*-2,358*	LEUCINE-RICH REPEAT/EXTENSIN 1 (LRX1)	encodes a a chimeric leucine-rich repeat/extensin protein that regulates root hair morphogenesis and elongation. Null mutants develop root hairs that frequently abort, swell, or branch. Gene is expressed in root hair cells and protein is specifically localized in the wall of the hair proper. The mRNA is cell-to-cell mobile.

AT1G30870	*-2,358*	PEROXIDASE7 (PER7)	Peroxidase superfamily protein; FUNCTIONS IN: peroxidase activity, heme binding; INVOLVED IN: oxidation reduction, response to oxidative stress; LOCATED IN: endomembrane system; EXPRESSED IN: root; CONTAINS InterPro DOMAIN/s: Haem peroxidase (InterPro:IPR010255), Plant peroxidase (InterPro:IPR000823), Peroxidases heam-ligand binding site (InterPro:IPR019793), Haem peroxidase, plant/fungal/bacterial (InterPro:IPR002016), Peroxidase, active site (InterPro:IPR019794); BEST Arabidopsis thaliana protein match is: Peroxidase superfamily protein (TAIR:AT1G49570.1)
AT3G05150	*-2,364*		Major facilitator superfamily protein; FUNCTIONS IN: carbohydrate transmembrane transporter activity, sugar:hydrogen symporter activity; INVOLVED IN: transport, transmembrane transport; LOCATED IN: nucleus, membrane; EXPRESSED IN: 12 plant structures; EXPRESSED DURING: L mature pollen stage, M germinated pollen stage, 4 anthesis, petal differentiation and expansion stage; CONTAINS InterPro DOMAIN/s: Sugar transporter, conserved site (InterPro:IPR005829), Major facilitator superfamily (InterPro:IPR020846), General substrate transporter (InterPro:IPR005828), Sugar/inositol transporter (InterPro:IPR003663), Major facilitator superfamily, general substrate transporter (InterPro:IPR016196); BEST Arabidopsis thaliana protein match is: Major facilitator superfamily protein (TAIR:AT5G18840.1)
AT4G25220	*-2,368*	GLYCEROL-3-PHOSPHATE PERMEASE 2 (G3Pp2)	Encodes a member of the phosphate starvation-induced glycerol-3-phosphate permease gene family: AT3G47420(G3Pp1), AT4G25220(G3Pp2), AT1G30560(G3Pp3), AT4G17550(G3Pp4) and AT2G13100(G3Pp5).
AT1G69240	*-2,373*	METHYL ESTERASE 15 (MES15)	Encodes a protein predicted to act as a carboxylesterase. It has similarity to the SABP2 methyl salicylate esterase from tobacco but no enzymatic activity has been identified for this protein.
AT1G48930	*-2,374*	GLYCOSYL HYDROLASE 9C1 (GH9C1)	glycosyl hydrolase 9C1 (GH9C1); FUNCTIONS IN: carbohydrate binding, hydrolase activity, hydrolyzing O-glycosyl compounds, catalytic activity; INVOLVED IN: carbohydrate metabolic process; LOCATED IN: endomembrane system, extracellular region; EXPRESSED IN: root; CONTAINS InterPro DOMAIN/s: Six-hairpin glycosidase (InterPro:IPR012341), Glycoside hydrolase, family 9, active site (InterPro:IPR018221), Six-hairpin glycosidase-like (InterPro:IPR008928), Glycoside hydrolase, family 9 (InterPro:IPR001701), Carbohydrate binding domain CBM49 (InterPro:IPR019028); BEST Arabidopsis thaliana protein match is: glycosyl hydrolase 9C2 (TAIR:AT1G64390.1)
AT3G02850	*-2,384*	STELAR K+ OUTWARD RECTIFIER (SKOR)	Encodes SKOR, a member of Shaker family potassium ion (K+) channel. This family includes five groups based on phylogenetic analysis (FEBS Letters (2007) 581: 2357): I (inward rectifying channel): AKT1 (AT2G26650), AKT5 (AT4G32500) and SPIK (also known as AKT6, AT2G25600); II (inward rectifying channel): KAT1 (AT5G46240) and KAT2 (AT4G18290); III (weakly inward rectifying channel): AKT2 (AT4G22200); IV (regulatory subunit involved in inwardly rectifying conductance formation): KAT3 (also known as AtKC1, AT4G32650); V (outward rectifying channel): SKOR (AT3G02850) and GORK (AT5G37500). Mediates the delivery of K+ from stelar cells to the xylem in the roots towards the shoot. mRNA accumulation is modulated by abscisic acid. K+ gating activity is modulated by external and internal K+. Involved in response to low potassium.
AT1G70210	*-2,385*	CYCLIN D1;1 (CYCD1;1)	Encodes a D-type cyclin that physically interacts with CDC2A. Its expression is upregulated early during germination.
AT1G51830	*-2,386*		Leucine-rich repeat protein kinase family protein; FUNCTIONS IN: protein serine/threonine kinase activity, kinase activity, ATP binding; INVOLVED IN: transmembrane receptor protein tyrosine kinase signaling pathway, protein amino acid phosphorylation; LOCATED IN: cellular_component unknown; EXPRESSED IN: root; CONTAINS InterPro DOMAIN/s: Protein kinase, ATP binding site (InterPro:IPR017441), Protein kinase, catalytic domain (InterPro:IPR000719), Leucine-rich repeat (InterPro:IPR001611), Serine/threonine-protein kinase-like domain (InterPro:IPR017442), Protein kinase-like domain (InterPro:IPR011009), Serine/threonine-protein kinase, active site (InterPro:IPR008271); BEST Arabidopsis thaliana protein match is: Leucine-rich repeat protein kinase family protein (TAIR:AT1G51850.1)
AT5G14330	*-2,386*		unknown protein
AT1G20190	*-2,387*	EXPANSIN 11 (EXPA11)	member of Alpha-Expansin Gene Family. Naming convention from the Expansin Working Group (Kende et al, 2004. Plant Mol Bio)
AT1G05530	*-2,392*	UDP-GLUCOSYL TRANSFERASE 75B2 (UGT75B2)	Encodes a protein with glucosyltransferase activity with high sequence homology to UGT1 (AT1G05560). It belongs to an UGT subfamily that binds UDP-glucose but not UDP-glucuronate, UDP-galactose, or UDP-rhamnose as the glycosyl donor. UGT2 was shown to be able to use abscisic acid as glycosylation substrate in the presence of UDP-glucose.

AT3G61390	*-2,395*		RING/U-box superfamily protein; FUNCTIONS IN: ubiquitin-protein ligase activity; INVOLVED IN: protein ubiquitination; LOCATED IN: ubiquitin ligase complex; EXPRESSED IN: 17 plant structures; EXPRESSED DURING: 11 growth stages; CONTAINS InterPro DOMAIN/s: U box domain (InterPro:IPR003613); BEST Arabidopsis thaliana protein match is: U-box domain-containing protein (TAIR:AT2G45920.1)
AT1G48070	*-2,402*		Thioredoxin superfamily protein; CONTAINS InterPro DOMAIN/s: Thioredoxin fold (InterPro:IPR012335), Thioredoxin, core (InterPro:IPR015467), Thioredoxin-like fold (InterPro:IPR012336)
AT2G27370	*-2,405*	CASPARIAN STRIP MEMBRANE DOMAIN PROTEIN 3 (CASP3)	Uncharacterised protein family (UPF0497); CONTAINS InterPro DOMAIN/s: Uncharacterised protein family UPF0497, trans-membrane plant (InterPro:IPR006702), Uncharacterised protein family UPF0497, trans-membrane plant subgroup (InterPro:IPR006459); BEST Arabidopsis thaliana protein match is: Uncharacterised protein family (UPF0497) (TAIR:AT2G36100.1)
AT1G60750	*-2,407*		NAD(P)-linked oxidoreductase superfamily protein; FUNCTIONS IN: oxidoreductase activity; INVOLVED IN: oxidation reduction; CONTAINS InterPro DOMAIN/s: Aldo/keto reductase (InterPro:IPR001395), Aldo/keto reductase subgroup (InterPro:IPR020471); BEST Arabidopsis thaliana protein match is: NAD(P)-linked oxidoreductase superfamily protein (TAIR:AT1G60730.1)
AT1G71030	*-2,409*	MYB-LIKE 2 (MYBL2)	Encodes a putative myb family transcription factor. In contrast to most other myb-like proteins its myb domain consists of a single repeat. A proline-rich region potentially involved in transactivation is found in the C-terminal part of the protein. Its transcript accumulates mainly in leaves.
AT3G14225	*-2,409*	GDSL-MOTIF LIPASE 4 (GLIP4)	Contains lipase signature motif and GDSL domain.
AT1G43010	*-2,41*		Pentatricopeptide repeat (PPR) superfamily protein; CONTAINS InterPro DOMAIN/s: Pentatricopeptide repeat (InterPro:IPR002885); BEST Arabidopsis thaliana protein match is: Tetratricopeptide repeat (TPR)-like superfamily protein (TAIR:AT1G28020.1)
AT2G22290	*-2,412*	RAB GTPASE HOMOLOG H1D (RABH1d)	RAB GTPase homolog H1D (RABH1d); FUNCTIONS IN: GTP binding; INVOLVED IN: protein transport, small GTPase mediated signal transduction; LOCATED IN: mitochondrion; EXPRESSED IN: male gametophyte, flower, pollen tube; EXPRESSED DURING: L mature pollen stage, M germinated pollen stage, 4 anthesis; CONTAINS InterPro DOMAIN/s: Ras GTPase (InterPro:IPR001806), Small GTP-binding protein (InterPro:IPR005225), Small GTPase (InterPro:IPR020851), Ras (InterPro:IPR013753), Ras small GTPase, Rab type (InterPro:IPR003579), Rab6-related (InterPro:IPR015600); BEST Arabidopsis thaliana protein match is: RAB GTPase homolog H1E (TAIR:AT5G10260.1)
AT4G33790	*-2,412*	ECERIFERUM 4 (CER4)	Encodes an alcohol-forming fatty acyl-CoA reductase, involved in cuticular wax biosynthesis. Lines carrying recessive mutations are deficient in primary alcohol and have glossy stem surfaces.
AT2G41970	*-2,414*	MARIS (MRI)	Encodes MRI, a plasma membrane-localized member of the RLCK-VIII subfamily. Preferentially expressed in both pollen tubes and root hairs. mri-knockout mutants display spontaneous pollen tube and root-hair bursting.
AT5G56795	*-2,417*	METALLOTHIONEIN 1B (MT1B)	one of the five metallothioneins (MTs) genes identified in Arabidopsis. MTs are cysteine-rich proteins required for heavy metal tolerance. The MT1b gene, however, is indicated to be inactive.
AT1G47600	*-2,418*	BETA GLUCOSIDASE 34 (BGLU34)	Encodes a myrosinase. Over-expression led to a glucosinolate profile change.
AT3G25820	*-2,42*	TERPENE SYNTHASE-LIKE SEQUENCE-1,8-CINEOLE (TPS-CIN)	Encodes the monoterpene 1,8-cineole synthase, atTPS-Cin. This polypeptide was also shown to synthesize other monoterpenes albeit in minor quantities. The same polypeptide is encoded at two different loci, the result of gene duplication: at3g25820 and at3g25830.
AT5G43540	*-2,424*		C2H2 and C2HC zinc fingers superfamily protein; FUNCTIONS IN: sequence-specific DNA binding transcription factor activity, zinc ion binding, nucleic acid binding; INVOLVED IN: regulation of transcription; LOCATED IN: intracellular, chloroplast; CONTAINS InterPro DOMAIN/s: Zinc finger, C2H2-like (InterPro:IPR015880), Zinc finger, C2H2-type (InterPro:IPR007087); BEST Arabidopsis thaliana protein match is: C2H2 and C2HC zinc fingers superfamily protein (TAIR:AT3G53820.1)

AT4G01830	-2,426	ATP-BINDING CASSETTE B5 (ABCB5)	P-glycoprotein 5 (PGP5); FUNCTIONS IN: ATPase activity, coupled to transmembrane movement of substances; INVOLVED IN: transport, transmembrane transport; LOCATED IN: integral to membrane; CONTAINS InterPro DOMAIN/s: ATPase, AAA+ type, core (InterPro:IPR003593), ABC transporter-like (InterPro:IPR003439), ABC transporter, transmembrane domain, type 1 (InterPro:IPR011527), ABC transporter integral membrane type 1 (InterPro:IPR017940), ABC transporter, transmembrane domain (InterPro:IPR001140), ABC transporter, conserved site (InterPro:IPR017871); BEST Arabidopsis thaliana protein match is: P-glycoprotein 3 (TAIR:AT4G01820.1)
AT2G04160	-2,428	AUXIN-INDUCED IN ROOT CULTURES 3 (AIR3)	isolated from differential screening of a cDNA library from auxin-treated root culture. encodes a protein similar to subtilisin-like serine protease which is believed to be active outside the plant cell.
AT1G44800	-2,433	SILIQUES ARE RED 1 (SIAR1)	Encodes Siliques Are Red 1 (SIAR1). Functions as a bidirectional amino acid transporter that is crucial for the amino acid homeostasis of siliques. Member of nodulin MtN21-like transporter family.
AT1G03840	-2,434	MAGPIE (MGP)	MGP is a nuclear-localized putative transcription factor with three zinc finger domains. MGP can interact with three proteins implicated in root patterning: SCR, SHR, and JKD in Y2H assays, and these interactions depend on the first zinc finger in MGP. MGP appears to be a direct transcriptional target of SHR and SCR, based on promoter binding assays, though it is not expressed in the QC, based on in situ hybridizations.
AT1G62980	-2,434	EXPANSIN A18 (EXPA18)	Alpha-expansin 18. Naming convention from the Expansin Working Group (Kende et al, 2004. Plant Mol Bio)
AT1G19390	-2,435		Wall-associated kinase family protein; FUNCTIONS IN: kinase activity; INVOLVED IN: protein amino acid phosphorylation; LOCATED IN: endomembrane system, integral to membrane; CONTAINS InterPro DOMAIN/s: Wall-associated kinase (InterPro:IPR013695), EGF-like calcium-binding, conserved site (InterPro:IPR018097), Protein kinase, catalytic domain (InterPro:IPR000719), Serine/threonine-protein kinase-like domain (InterPro:IPR017442), Protein kinase-like domain (InterPro:IPR011009), Serine/threonine-protein kinase, active site (InterPro:IPR008271); BEST Arabidopsis thaliana protein match is: wall-associated kinase, putative (TAIR:AT4G31100.1)
AT5G53980	-2,438	HOMEOBOX PROTEIN 52 (HB52)	Encodes a homeodomain leucine zipper class I (HD-Zip I) protein.
AT2G31310	-2,439	LOB DOMAIN-CONTAINING PROTEIN 14 (LBD14)	LOB domain-containing protein 14 (LBD14); CONTAINS InterPro DOMAIN/s: Lateral organ boundaries, LOB (InterPro:IPR004883); BEST Arabidopsis thaliana protein match is: lateral organ boundaries-domain 29 (TAIR:AT3G58190.1)
AT1G57590	-2,441		Pectinacetylesterase family protein; CONTAINS InterPro DOMAIN/s: Pectinacetylesterase (InterPro:IPR004963); BEST Arabidopsis thaliana protein match is: Pectinacetylesterase family protein (TAIR:AT5G26670.1)
AT1G32450	-2,446	NRT1/ PTR FAMILY 7.3 (NPF7.3)	Transmembrane nitrate transporter. Involved in xylem transport of nitrate from root to shoot. Induced in response to high and low concentrations of nitrate. Not involved in nitrate uptake. Expressed in root pericycle cells.
AT5G19800	-2,446	HYDROXYPROLINE-RICH GLYCOPROTEIN 2 (HRGP2)	hydroxyproline-rich glycoprotein family protein; INVOLVED IN: biological_process unknown; LOCATED IN: endomembrane system; BEST Arabidopsis thaliana protein match is: hydroxyproline-rich glycoprotein family protein (TAIR:AT5G26070.1)
AT5G05500	-2,449	(MOP10)	Encodes Proline-rich protein-like PRPL1, controls elongation of root hairs.
AT3G25190	-2,455	VACUOLAR IRON TRANSPORTER-LIKE 5 (VTL5)	The gene encodes nodulin-like21 whose transcript abundance was repressed under conditions of Fe-deficient growth.
AT1G64930	-2,457	CYTOCHROME P450, FAMILY 87, SUBFAMILY A, POLYPEPTIDE 7 (CYP89A7)	member of CYP89A
AT5G62340	-2,457		Plant invertase/pectin methylesterase inhibitor superfamily protein; FUNCTIONS IN: enzyme inhibitor activity, pectinesterase inhibitor activity, pectinesterase activity; INVOLVED IN: biological_process unknown; LOCATED IN: endomembrane system; EXPRESSED IN: hypocotyl, root; CONTAINS InterPro DOMAIN/s: Pectinesterase inhibitor (InterPro:IPR006501); BEST Arabidopsis thaliana protein match is: Plant invertase/pectin methylesterase inhibitor superfamily protein (TAIR:AT5G62350.1)
AT2G32530	-2,459	CELLULOSE SYNTHASE-LIKE B3 (CSLB03)	encodes a gene similar to cellulose synthase

AT4G11040	*-2,461*	REDUCED DORMANCY 5 (RDO5)	Encodes a nuclear localized protein with sequence similarity to PP2C phosphatases that is involved in seed dormancy. Loss of function mutations have reduced seed dormancy but does not act through ABA or DOG1 pathways. Lacks several conserved key residues and does not possess any appreciable phosphatase activity in in vitro assays. QTL allele with a nonsynonymous amino acid change confers seed dormancy phenotype.
AT3G07490	*-2,463*	ARF-GAP DOMAIN 11 (AGD11)	A member of ARF GAP domain (AGD), A thaliana has 15 members, grouped into four classes.
AT5G51500	*-2,468*		Plant invertase/pectin methylesterase inhibitor superfamily; FUNCTIONS IN: enzyme inhibitor activity, pectinesterase activity; INVOLVED IN: cell wall modification; LOCATED IN: endomembrane system, cell wall, plant-type cell wall; EXPRESSED IN: sperm cell, flower, root; EXPRESSED DURING: 4 anthesis; CONTAINS InterPro DOMAIN/s: Pectinesterase, active site (InterPro:IPR018040), Pectin lyase fold/virulence factor (InterPro:IPR011050), Pectinesterase, catalytic (InterPro:IPR000070), Pectinesterase inhibitor (InterPro:IPR006501), Pectin lyase fold (InterPro:IPR012334); BEST Arabidopsis thaliana protein match is: Plant invertase/pectin methylesterase inhibitor superfamily (TAIR:AT5G51490.1)
AT4G31710	*-2,469*	GLUTAMATE RECEPTOR 2.4 (GLR2.4)	member of Putative ligand-gated ion channel subunit family
AT4G00670	*-2,471*		Remorin family protein; CONTAINS InterPro DOMAIN/s: Remorin, C-terminal (InterPro:IPR005516); BEST Arabidopsis thaliana protein match is: Remorin family protein (TAIR:AT3G61260.1)
AT5G44020	*-2,471*		HAD superfamily, subfamily IIIB acid phosphatase ; FUNCTIONS IN: acid phosphatase activity; INVOLVED IN: biological_process unknown; LOCATED IN: chloroplast, vacuole, membrane; EXPRESSED IN: 23 plant structures; EXPRESSED DURING: 13 growth stages; CONTAINS InterPro DOMAIN/s: Acid phosphatase (Class B) (InterPro:IPR005519), Vegetative storage protein/acid phosphatase (InterPro:IPR014403), Acid phosphatase, plant (InterPro:IPR010028); BEST Arabidopsis thaliana protein match is: HAD superfamily, subfamily IIIB acid phosphatase (TAIR:AT1G04040.1)
AT3G60280	*-2,473*	UCLACYANIN 3 (UCC3)	Encodes blue copper-binding protein III.
AT5G49270	*-2,475*	SHAVEN 2 (SHV2)	Involved in successfully establishing tip growth in root hairs.
AT5G43520	*-2,478*		Cysteine/Histidine-rich C1 domain family protein; CONTAINS InterPro DOMAIN/s: DC1 (InterPro:IPR004146), C1-like (InterPro:IPR011424); BEST Arabidopsis thaliana protein match is: Cysteine/Histidine-rich C1 domain family protein (TAIR:AT2G44380.1)
AT2G36830	*-2,48*	GAMMA TONOPLAST INTRINSIC PROTEIN (GAMMA-TIP)	Encodes a tonoplast intrinsic protein, which functions as water channel. It has also been shown to be able to facilitate the transport of urea and hydrogen peroxide. Highly expressed in vascular tissues of the root, stem, cauline leaves and flowers but not in the apical meristems. The mRNA is cell-to-cell mobile.
AT5G38970	*-2,482*	BRASSINOSTEROID-6-OXIDASE 1 (BR6OX1)	Encodes a polypeptide involved in the C-6 oxidation of brassinosteroids. Heterologous expression of the protein in yeast conferred the ability to catalyze multiple reactions in which the C-6 position of 6-deoxocastasterone, 6-deoxotyphasterol, 3-dehydro-6-deoxoteasterone and 6-deoxoteasterone are oxidized.
AT4G13580	*-2,486*		Disease resistance-responsive (dirigent-like protein) family protein; FUNCTIONS IN: molecular_function unknown; LOCATED IN: endomembrane system; EXPRESSED IN: root; CONTAINS InterPro DOMAIN/s: Plant disease resistance response protein (InterPro:IPR004265); BEST Arabidopsis thaliana protein match is: Disease resistance-responsive (dirigent-like protein) family protein (TAIR:AT3G24020.1)
AT5G42500	*-2,488*		Disease resistance-responsive (dirigent-like protein) family protein; FUNCTIONS IN: molecular_function unknown; INVOLVED IN: defense response; LOCATED IN: endomembrane system; CONTAINS InterPro DOMAIN/s: Plant disease resistance response protein (InterPro:IPR004265); BEST Arabidopsis thaliana protein match is: Disease resistance-responsive (dirigent-like protein) family protein (TAIR:AT5G42510.1)
AT3G10710	*-2,493*	ROOT HAIR SPECIFIC 12 (RHS12)	root hair specific 12 (RHS12); FUNCTIONS IN: enzyme inhibitor activity, pectinesterase activity; INVOLVED IN: cell wall modification; LOCATED IN: cell wall, plant-type cell wall; EXPRESSED IN: root hair, flower; CONTAINS InterPro DOMAIN/s: Pectinesterase, active site (InterPro:IPR018040), Pectin lyase fold/virulence factor (InterPro:IPR011050), Pectinesterase, catalytic (InterPro:IPR000070), Pectinesterase inhibitor (InterPro:IPR006501), Pectin lyase fold (InterPro:IPR012334); BEST Arabidopsis thaliana protein match is: Plant invertase/pectin methylesterase inhibitor superfamily (TAIR:AT5G04960.1)

AT1G63600	*-2,495*		Receptor-like protein kinase-related family protein; FUNCTIONS IN: molecular_function unknown; INVOLVED IN: biological_process unknown; LOCATED IN: endomembrane system; CONTAINS InterPro DOMAIN/s: Protein of unknown function DUF26 (InterPro:IPR002902); BEST Arabidopsis thaliana protein match is: Receptor-like protein kinase-related family protein (TAIR:AT1G63560.1)
AT2G14760	*-2,496*		basic helix-loop-helix (bHLH) DNA-binding superfamily protein; FUNCTIONS IN: sequence-specific DNA binding transcription factor activity; INVOLVED IN: regulation of transcription; LOCATED IN: nucleus; CONTAINS InterPro DOMAIN/s: Helix-loop-helix DNA-binding domain (InterPro:IPR001092), Helix-loop-helix DNA-binding (InterPro:IPR011598); BEST Arabidopsis thaliana protein match is: ROOT HAIR DEFECTIVE 6-LIKE 2 (TAIR:AT4G33880.1).
AT5G18910	*-2,502*		Protein kinase superfamily protein; FUNCTIONS IN: protein serine/threonine kinase activity, protein kinase activity, kinase activity, ATP binding; INVOLVED IN: protein amino acid phosphorylation; LOCATED IN: peroxisome; EXPRESSED IN: 9 plant structures; EXPRESSED DURING: L mature pollen stage, M germinated pollen stage, 4 anthesis, C globular stage, petal differentiation and expansion stage; CONTAINS InterPro DOMAIN/s: Protein kinase, ATP binding site (InterPro:IPR017441), Protein kinase, catalytic domain (InterPro:IPR000719), Serine-threonine/tyrosine-protein kinase (InterPro:IPR001245), Protein kinase-like domain (InterPro:IPR011009), Serine/threonine-protein kinase, active site (InterPro:IPR008271); BEST Arabidopsis thaliana protein match is: ROP binding protein kinases 2 (TAIR:AT3G05140.1)
AT1G15380	*-2,511*	GLYOXYLASE I 4 (GLYI4)	Lactoylglutathione lyase / glyoxalase I family protein; CONTAINS InterPro DOMAIN/s: Glyoxalase/bleomycin resistance protein/dioxygenase (InterPro:IPR004360); BEST Arabidopsis thaliana protein match is: Lactoylglutathione lyase / glyoxalase I family protein (TAIR:AT1G80160.1)
AT5G17820	*-2,519*	PEROXIDASE 57 (PER57)	Peroxidase superfamily protein; FUNCTIONS IN: peroxidase activity, heme binding; INVOLVED IN: oxidation reduction, response to oxidative stress; LOCATED IN: cell wall, membrane, plant-type cell wall; EXPRESSED IN: root, leaf; CONTAINS InterPro DOMAIN/s: Haem peroxidase (InterPro:IPR010255), Plant peroxidase (InterPro:IPR000823), Peroxidases heam-ligand binding site (InterPro:IPR019793), Peroxidase, active site (InterPro:IPR019794), Haem peroxidase, plant/fungal/bacterial (InterPro:IPR002016); BEST Arabidopsis thaliana protein match is: Peroxidase superfamily protein (TAIR:AT3G03670.1)
AT5G58010	*-2,523*	LJRHL1-LIKE 3 (LRL3)	Encodes a basic helix-loop-helix (bHLH) protein that regulates root hair development. One of the three Arabidopsis homologs of the Lotus japonicus ROOTHAIRLESS1 (LjRHL1) gene: At2g24260 (AtLRL1), At4g30980 (AtLRL2), and At5g58010 (AtLRL3).
AT4G34580	*-2,532*	CAN OF WORMS1 (COW1)	Encodes COW1 (can of worms1), a phosphatidylinositol transfer protein essential for root hair tip growth. The N-terminus of the COW1 protein is 32% identical to an essential phosphatidylinositol transfer protein (PITP), the yeast Sec14 protein (sec14p) while the C-terminus is 34.5% identical to a late nodulin of Lotus japonicus, Nlj16. Expression of COW1 complements the growth defect associated with Sec14p dysfunction in yeast. GFP fused to the COW1 protein specifically accumulates at the site of root hair outgrowth.
AT5G47450	*-2,538*	TONOPLAST INTRINSIC PROTEIN 2;3 (TIP2;3)	Tonoplast intrinsic protein, transports ammonium (NH3) and methylammonium across the tonoplast membrane, gene expression shows diurnal regulation and is upregulated by ammonium (NH3).
AT2G43880	*-2,543*		Pectin lyase-like superfamily protein; FUNCTIONS IN: polygalacturonase activity; INVOLVED IN: carbohydrate metabolic process; LOCATED IN: endomembrane system; EXPRESSED IN: root, carpel; EXPRESSED DURING: 4 anthesis; CONTAINS InterPro DOMAIN/s: Pectin lyase fold/virulence factor (InterPro:IPR011050), Pectin lyase fold (InterPro:IPR012334), Glycoside hydrolase, family 28 (InterPro:IPR000743), Parallel beta-helix repeat (InterPro:IPR006626); BEST Arabidopsis thaliana protein match is: Pectin lyase-like superfamily protein (TAIR:AT2G43890.1)
AT3G01260	*-2,543*		Galactose mutarotase-like superfamily protein; FUNCTIONS IN: carbohydrate binding, isomerase activity, aldose 1-epimerase activity, catalytic activity; INVOLVED IN: galactose metabolic process, hexose metabolic process, carbohydrate metabolic process; LOCATED IN: endomembrane system; EXPRESSED IN: root; CONTAINS InterPro DOMAIN/s: Aldose 1-epimerase (InterPro:IPR008183), Glycoside hydrolase-type carbohydrate-binding (InterPro:IPR011013), Aldose 1-epimerase, subgroup (InterPro:IPR015443), Glycoside hydrolase-type carbohydrate-binding, subgroup (InterPro:IPR014718); BEST Arabidopsis thaliana protein match is: Galactose mutarotase-like superfamily protein (TAIR:AT5G15140.1)

AT5G04970	*-2,545*		Plant invertase/pectin methylesterase inhibitor superfamily; FUNCTIONS IN: enzyme inhibitor activity, pectinesterase activity; INVOLVED IN: cell wall modification; LOCATED IN: cell wall; EXPRESSED IN: flower, root; CONTAINS InterPro DOMAIN/s: Pectinesterase, active site (InterPro:IPR018040), Pectin lyase fold/virulence factor (InterPro:IPR011050), Pectinesterase, catalytic (InterPro:IPR000070), Pectinesterase inhibitor (InterPro:IPR006501), Pectin lyase fold (InterPro:IPR012334); BEST Arabidopsis thaliana protein match is: Plant invertase/pectin methylesterase inhibitor superfamily (TAIR:AT3G10720.2)
AT2G47530	*-2,549*		Pollen Ole e 1 allergen and extensin family protein; FUNCTIONS IN: molecular_function unknown; INVOLVED IN: biological_process unknown; LOCATED IN: endomembrane system; CONTAINS InterPro DOMAIN/s: Pollen Ole e 1 allergen/extensin (InterPro:IPR006041); BEST Arabidopsis thaliana protein match is: proline-rich protein 1 (TAIR:AT1G54970.1)
AT2G25810	*-2,563*	TONOPLAST INTRINSIC PROTEIN 4;1 (TIP4;1)	tonoplast intrinsic protein 4;1 (TIP4;1); FUNCTIONS IN: water channel activity; INVOLVED IN: transport, transmembrane transport; LOCATED IN: plant-type vacuole membrane, membrane, central vacuole; EXPRESSED IN: 14 plant structures; EXPRESSED DURING: 4 anthesis, F mature embryo stage, petal differentiation and expansion stage, E expanded cotyledon stage, D bilateral stage; CONTAINS InterPro DOMAIN/s: Major intrinsic protein, conserved site (InterPro:IPR022357), Aquaporin (InterPro:IPR012269), Major intrinsic protein (InterPro:IPR000425); BEST Arabidopsis thaliana protein match is: gamma tonoplast intrinsic protein (TAIR:AT2G36830.1)
AT1G80240	*-2,567*	DUF642 L-GALL RESPONSIVE GENE 1 (DGR1)	Protein of unknown function, DUF642; CONTAINS InterPro DOMAIN/s: Protein of unknown function DUF642 (InterPro:IPR006946); BEST Arabidopsis thaliana protein match is: Protein of unknown function, DUF642 (TAIR:AT5G25460.1)
AT3G48260	*-2,57*	WITH NO LYSINE (K) KINASE 3 (WNK3)	Encodes a member of the WNK family (9 members in all) of protein kinases, the structural design of which is clearly distinct from those of other known protein kinases, such as receptor-like kinases and mitogen-activated protein kinases.
AT1G76240	*-2,574*		Arabidopsis protein of unknown function (DUF241); FUNCTIONS IN: molecular_function unknown; LOCATED IN: chloroplast; EXPRESSED IN: 19 plant structures; EXPRESSED DURING: 9 growth stages; CONTAINS InterPro DOMAIN/s: Protein of unknown function DUF241, plant (InterPro:IPR004320); BEST Arabidopsis thaliana protein match is: Arabidopsis protein of unknown function (DUF241) (TAIR:AT2G17080.1)
AT3G05920	*-2,585*		Heavy metal transport/detoxification superfamily protein ; FUNCTIONS IN: metal ion binding; INVOLVED IN: metal ion transport; LOCATED IN: cellular_component unknown; EXPRESSED IN: root; CONTAINS InterPro DOMAIN/s: Heavy metal transport/detoxification protein (InterPro:IPR006121); BEST Arabidopsis thaliana protein match is: Heavy metal transport/detoxification superfamily protein (TAIR:AT5G26690.1)
AT5G56320	*-2,587*	EXPANSIN A14 (EXPA14)	member of Alpha-Expansin Gene Family. Naming convention from the Expansin Working Group (Kende et al, 2004. Plant Mol Bio)
AT4G37700	*-2,591*		unknown protein
AT2G01530	*-2,593*	MLP-LIKE PROTEIN 329 (MLP329)	MLP-like protein 329 (MLP329); FUNCTIONS IN: copper ion binding; INVOLVED IN: response to biotic stimulus, defense response; LOCATED IN: cellular_component unknown; EXPRESSED IN: hypocotyl, root; CONTAINS InterPro DOMAIN/s: Bet v I allergen (InterPro:IPR000916); BEST Arabidopsis thaliana protein match is: MLP-like protein 328 (TAIR:AT2G01520.1)
AT2G21610	*-2,597*	PECTINESTERASE 11 (PE11)	pectinesterase 11 (PE11); FUNCTIONS IN: pectinesterase activity; INVOLVED IN: N-terminal protein myristoylation, cell wall modification; LOCATED IN: cell wall, plant-type cell wall; EXPRESSED IN: flower; CONTAINS InterPro DOMAIN/s: Pectin lyase fold/virulence factor (InterPro:IPR011050), Pectinesterase, catalytic (InterPro:IPR000070), Pectin lyase fold (InterPro:IPR012334); BEST Arabidopsis thaliana protein match is: Pectin lyase-like superfamily protein (TAIR:AT5G19730.1)
AT5G55250	*-2,602*	IAA CARBOXYLMETHYLTRANSFERASE 1 (IAMT1)	Encodes an enzyme which specifically converts IAA to its methyl ester form MeIIAA. This gene belongs to the family of carboxyl methyltransferases whose members catalyze the transfer of the methyl group from S-adenosyl-L-methionine to carboxylic acid-containing substrates to form small molecule methyl esters. Expression of TCP genes is downregulated in mutant iamt1-D.

AT4G12520	*-2,603*		Bifunctional inhibitor/lipid-transfer protein/seed storage 2S albumin superfamily protein; FUNCTIONS IN: lipid binding; INVOLVED IN: lipid transport; LOCATED IN: endomembrane system; CONTAINS InterPro DOMAIN/s: Bifunctional inhibitor/plant lipid transfer protein/seed storage (InterPro:IPR016140), Plant lipid transfer protein/seed storage/trypsin-alpha amylase inhibitor (InterPro:IPR003612), Plant lipid transfer protein/hydrophobic protein, helical domain (InterPro:IPR013770); BEST Arabidopsis thaliana protein match is: Bifunctional inhibitor/lipid-transfer protein/seed storage 2S albumin superfamily protein (TAIR:AT4G12510.1)
AT5G64100	*-2,604*		Peroxidase superfamily protein; FUNCTIONS IN: peroxidase activity, heme binding; INVOLVED IN: oxidation reduction, response to oxidative stress; LOCATED IN: cell wall; EXPRESSED IN: hypocotyl, root, callus; EXPRESSED DURING: seedling growth; CONTAINS InterPro DOMAIN/s: Haem peroxidase (InterPro:IPR010255), Plant peroxidase (InterPro:IPR000823), Peroxidases heam-ligand binding site (InterPro:IPR019793), Haem peroxidase, plant/fungal/bacterial (InterPro:IPR002016), Peroxidase, active site (InterPro:IPR019794); BEST Arabidopsis thaliana protein match is: Peroxidase superfamily protein (TAIR:AT5G64110.1)
AT4G01380	*-2,615*		plastocyanin-like domain-containing protein; FUNCTIONS IN: electron carrier activity, copper ion binding; INVOLVED IN: biological_process unknown; LOCATED IN: cellular_component unknown; CONTAINS InterPro DOMAIN/s: Plastocyanin-like (InterPro:IPR003245), Cupredoxin (InterPro:IPR008972); BEST Arabidopsis thaliana protein match is: copper ion binding;electron carriers (TAIR:AT1G45063.2)
AT1G53830	*-2,619*	PECTIN METHYLESTERASE 2 (PME2)	encodes a pectin methylesterase
AT1G79130	*-2,62*	SMALL AUXIN UPREGULATED 40 (SAUR40)	SAUR-like auxin-responsive protein family ; CONTAINS InterPro DOMAIN/s: Auxin responsive SAUR protein (InterPro:IPR003676); BEST Arabidopsis thaliana protein match is: SAUR-like auxin-responsive protein family (TAIR:AT1G16510.1)
AT2G46860	*-2,625*	PYROPHOSPHORYLASE 3 (PPa3)	Encodes a protein that might have inorganic pyrophosphatase activity.
AT5G39240	*-2,626*		Encodes a atypical member of the bHLH (basic helix-loop-helix) family transcriptional factors.
AT2G37170	*-2,628*	PLASMA MEMBRANE INTRINSIC PROTEIN 2 (PIP2B)	a member of the plasma membrane intrinsic protein subfamily PIP2. localizes to the plasma membrane and exhibits water transport activity in Xenopus oocyte. expressed specifically in the vascular bundles and protein level increases slightly during leaf dev
AT5G67400	*-2,629*	ROOT HAIR SPECIFIC 19 (RHS19)	root hair specific 19 (RHS19); FUNCTIONS IN: peroxidase activity, heme binding; INVOLVED IN: oxidation reduction, response to oxidative stress; LOCATED IN: endomembrane system; EXPRESSED IN: root hair, root; CONTAINS InterPro DOMAIN/s: Haem peroxidase (InterPro:IPR010255), Plant peroxidase (InterPro:IPR000823), Peroxidases heam-ligand binding site (InterPro:IPR019793), Haem peroxidase, plant/fungal/bacterial (InterPro:IPR002016); BEST Arabidopsis thaliana protein match is: Peroxidase superfamily protein (TAIR:AT3G49960.1)
AT5G10990	*-2,632*	SMALL AUXIN UPREGULATED RNA 69 (SAUR69)	SAUR-like auxin-responsive protein family ; CONTAINS InterPro DOMAIN/s: Auxin responsive SAUR protein (InterPro:IPR003676); BEST Arabidopsis thaliana protein match is: SAUR-like auxin-responsive protein family (TAIR:AT1G75590.1)
AT2G37130	*-2,635*		Peroxidase superfamily protein; FUNCTIONS IN: peroxidase activity, heme binding; INVOLVED IN: defense response to fungus; LOCATED IN: endomembrane system; EXPRESSED IN: 15 plant structures; EXPRESSED DURING: 7 growth stages; CONTAINS InterPro DOMAIN/s: Haem peroxidase (InterPro:IPR010255), Plant peroxidase (InterPro:IPR000823), Haem peroxidase, plant/fungal/bacterial (InterPro:IPR002016); BEST Arabidopsis thaliana protein match is: Peroxidase superfamily protein (TAIR:AT4G21960.1)
AT1G74500	*-2,647*	ACTIVATION-TAGGED BRI1(BRASSINOSTEROID-INSENSITIVE 1)-SUPPRESSOR 1 (BS1)	Encodes a basic helix?loop?helix transcription factor that acts downstream of MP in root initiation. TMO7 protein moves to the hypophysis and to vascular cells, contributing to MP-dependent root formation. Promotes the correct definition of the hypophysis cell division plane.

AT2G39040	*-2,657*		Peroxidase superfamily protein; FUNCTIONS IN: peroxidase activity, heme binding; INVOLVED IN: response to oxidative stress, oxidation reduction; LOCATED IN: endomembrane system; EXPRESSED IN: root; CONTAINS InterPro DOMAIN/s: Haem peroxidase (InterPro:IPR010255), Plant peroxidase (InterPro:IPR000823), Peroxidases heam-ligand binding site (InterPro:IPR019793), Haem peroxidase, plant/fungal/bacterial (InterPro:IPR002016); BEST Arabidopsis thaliana protein match is: Peroxidase superfamily protein (TAIR:AT1G05260.1)
AT5G56540	*-2,663*	ARABINOGALACTAN PROTEIN 14 (AGP14)	Encodes arabinogalactan protein (AGP14). Mutants exhibit longer root hairs. The mRNA is cell-to-cell mobile.
AT4G02270	*-2,672*	ROOT HAIR SPECIFIC 13 (RHS13)	root hair specific 13 (RHS13); FUNCTIONS IN: molecular_function unknown; INVOLVED IN: biological_process unknown; LOCATED IN: endomembrane system; EXPRESSED IN: hypocotyl, root hair, root; CONTAINS InterPro DOMAIN/s: Pollen Ole e 1 allergen/extensin (InterPro:IPR006041); BEST Arabidopsis thaliana protein match is: Pollen Ole e 1 allergen and extensin family protein (TAIR:AT2G47540.1)
AT3G01190	*-2,687*		Peroxidase superfamily protein; FUNCTIONS IN: peroxidase activity, heme binding; INVOLVED IN: response to oxidative stress, oxidation reduction; LOCATED IN: endomembrane system; EXPRESSED IN: hypocotyl, root; CONTAINS InterPro DOMAIN/s: Haem peroxidase (InterPro:IPR010255), Plant peroxidase (InterPro:IPR000823), Peroxidases heam-ligand binding site (InterPro:IPR019793), Haem peroxidase, plant/fungal/bacterial (InterPro:IPR002016), Peroxidase, active site (InterPro:IPR019794); BEST Arabidopsis thaliana protein match is: Peroxidase superfamily protein (TAIR:AT5G15180.1)
AT4G22080	*-2,688*	ROOT HAIR SPECIFIC 14 (RHS14)	root hair specific 14 (RHS14); FUNCTIONS IN: lyase activity, pectate lyase activity; INVOLVED IN: biological_process unknown; LOCATED IN: endomembrane system; EXPRESSED IN: root hair; CONTAINS InterPro DOMAIN/s: Pectin lyase fold/virulence factor (InterPro:IPR011050), AmbAllergen (InterPro:IPR018082), Pectate lyase/Amb allergen (InterPro:IPR002022), Pectin lyase fold (InterPro:IPR012334), Parallel beta-helix repeat (InterPro:IPR006626); BEST Arabidopsis thaliana protein match is: Pectin lyase-like superfamily protein (TAIR:AT4G22090.1)
AT1G50050	*-2,69*		CAP (Cysteine-rich secretory proteins, Antigen 5, and Pathogenesis-related 1 protein) superfamily protein; FUNCTIONS IN: molecular_function unknown; INVOLVED IN: biological_process unknown; LOCATED IN: endomembrane system, extracellular region; EXPRESSED IN: root; CONTAINS InterPro DOMAIN/s: Ves allergen (InterPro:IPR002413), Allergen V5/Tpx-1 related (InterPro:IPR001283), SCP-like extracellular (InterPro:IPR014044); BEST Arabidopsis thaliana protein match is: CAP (Cysteine-rich secretory proteins, Antigen 5, and Pathogenesis-related 1 protein) superfamily protein (TAIR:AT1G50060.1)
AT1G61950	*-2,69*	CALCIUM-DEPENDENT PROTEIN KINASE 19 (CPK19)	member of Calcium Dependent Protein Kinase
AT4G26010	*-2,693*		Peroxidase superfamily protein; FUNCTIONS IN: peroxidase activity, heme binding; INVOLVED IN: oxidation reduction, response to oxidative stress; LOCATED IN: endomembrane system; EXPRESSED IN: root; CONTAINS InterPro DOMAIN/s: Haem peroxidase (InterPro:IPR010255), Plant peroxidase (InterPro:IPR000823), Peroxidase, active site (InterPro:IPR019794), Haem peroxidase, plant/fungal/bacterial (InterPro:IPR002016); BEST Arabidopsis thaliana protein match is: Peroxidase superfamily protein (TAIR:AT1G34510.1)
AT1G44030	*-2,702*		Cysteine/Histidine-rich C1 domain family protein; LOCATED IN: chloroplast; EXPRESSED IN: root; CONTAINS InterPro DOMAIN/s: DC1 (InterPro:IPR004146), C1-like (InterPro:IPR011424); BEST Arabidopsis thaliana protein match is: Cysteine/Histidine-rich C1 domain family protein (TAIR:AT1G44020.1)
AT2G19050	*-2,702*		GDSL-motif esterase/acyltransferase/lipase. Enzyme group with broad substrate specificity that may catalyze acyltransfer or hydrolase reactions with lipid and non-lipid substrates.
AT4G11610	*-2,705*		C2 calcium/lipid-binding plant phosphoribosyltransferase family protein; CONTAINS InterPro DOMAIN/s: C2 membrane targeting protein (InterPro:IPR018029), C2 calcium/lipid-binding domain, CaLB (InterPro:IPR008973), Phosphoribosyltransferase C-terminal (InterPro:IPR013583), C2 calcium-dependent membrane targeting (InterPro:IPR000008); BEST Arabidopsis thaliana protein match is: Calcium-dependent lipid-binding (CaLB domain) plant phosphoribosyltransferase family protein (TAIR:AT3G57880.1)
AT2G30370	*-2,713*	CHALLAH (CHAL)	Encodes a small, potentially secreted protein that acts as an inhibitor of stomatal production though likely not through direct interaction with the TMM receptor. It is homologous to known stomatal regulators EPF1 and EPF2.

AT4G15290	-2,715	(ATCSLB05)	Encodes a gene similar to cellulose synthase. Mutants exhibit shorter root hairs under phosphate-deficient conditions.
AT5G07040	-2,748		RING/U-box superfamily protein; FUNCTIONS IN: zinc ion binding; LOCATED IN: endomembrane system; EXPRESSED IN: 12 plant structures; EXPRESSED DURING: 6 growth stages; CONTAINS InterPro DOMAIN/s: Zinc finger, RING-type (InterPro:IPR001841), Zinc finger, C3HC4 RING-type (InterPro:IPR018957); BEST Arabidopsis thaliana protein match is: RING/U-box superfamily protein (TAIR:AT5G53110.1)
AT5G46890	-2,751		Bifunctional inhibitor/lipid-transfer protein/seed storage 2S albumin superfamily protein; FUNCTIONS IN: lipid binding; INVOLVED IN: lipid transport; LOCATED IN: endomembrane system; CONTAINS InterPro DOMAIN/s: Bifunctional inhibitor/plant lipid transfer protein/seed storage (InterPro:IPR016140), Plant lipid transfer protein/seed storage/trypsin-alpha amylase inhibitor (InterPro:IPR003612), Plant lipid transfer protein/hydrophobic protein, helical domain (InterPro:IPR013770); BEST Arabidopsis thaliana protein match is: Bifunctional inhibitor/lipid-transfer protein/seed storage 2S albumin superfamily protein (TAIR:AT5G46900.1)
AT5G52790	-2,752		FUNCTIONS IN: molecular_function unknown; INVOLVED IN: biological_process unknown; LOCATED IN: endomembrane system; CONTAINS InterPro DOMAIN/s: Protein of unknown function DUF21 (InterPro:IPR002550), Cystathionine beta-synthase, core (InterPro:IPR000644); BEST Arabidopsis thaliana protein match is: CBS domain-containing protein with a domain of unknown function (DUF21) (TAIR:AT2G14520.1)
AT2G03720	-2,755	MORPHOGENESIS OF ROOT HAIR 6 (MRH6)	Involved in root hair development
AT4G01390	-2,756		TRAF-like family protein; CONTAINS InterPro DOMAIN/s: TRAF-like (InterPro:IPR008974), MATH (InterPro:IPR002083); BEST Arabidopsis thaliana protein match is: TRAF-like family protein (TAIR:AT4G00780.1)
AT5G53250	-2,763	ARABINOGALACTAN PROTEIN 22 (AGP22)	arabinogalactan protein 22 (AGP22); CONTAINS InterPro DOMAIN/s: Protein of unknown function DUF1070 (InterPro:IPR009424); BEST Arabidopsis thaliana protein match is: arabinogalactan protein 20 (TAIR:AT3G61640.1)
AT5G63660	-2,764	(PDF2.5)	Predicted to encode a PR (pathogenesis-related) protein. Belongs to the plant defensin (PDF) family with the following members: At1g75830/PDF1.1, At5g44420/PDF1.2a, At2g26020/PDF1.2b, At5g44430/PDF1.2c, At2g26010/PDF1.3, At1g19610/PDF1.4, At1g55010/PDF1.5, At2g02120/PDF2.1, At2g02100/PDF2.2, At2g02130/PDF2.3, At1g61070/PDF2.4, At5g63660/PDF2.5, At2g02140/PDF2.6, At5g38330/PDF3.1 and At4g30070/PDF3.2.
AT5G15180	-2,788	PEROXIDASE 56 (PRX56)	Peroxidase superfamily protein; FUNCTIONS IN: peroxidase activity, heme binding; INVOLVED IN: response to oxidative stress, oxidation reduction; LOCATED IN: endomembrane system; EXPRESSED IN: root, flower, carpel; EXPRESSED DURING: 4 anthesis, petal differentiation and expansion stage; CONTAINS InterPro DOMAIN/s: Haem peroxidase (InterPro:IPR010255), Plant peroxidase (InterPro:IPR000823), Peroxidases heam-ligand binding site (InterPro:IPR019793), Peroxidase, active site (InterPro:IPR019794), Haem peroxidase, plant/fungal/bacterial (InterPro:IPR002016); BEST Arabidopsis thaliana protein match is: Peroxidase superfamily protein (TAIR:AT3G01190.1)
AT5G06640	-2,789	EXTENSIN 10 (EXT10)	Proline-rich extensin-like family protein; FUNCTIONS IN: structural constituent of cell wall; INVOLVED IN: plant-type cell wall organization; EXPRESSED IN: shoot apex, hypocotyl, sepal, root, leaf; EXPRESSED DURING: 4 anthesis, LP.12 twelve leaves visible; CONTAINS InterPro DOMAIN/s: Extensin-like repeat (InterPro:IPR006706); BEST Arabidopsis thaliana protein match is: Proline-rich extensin-like family protein (TAIR:AT4G08410.1)
AT5G23030	-2,789	TETRASPANIN12 (TET12)	Member of TETRASPANIN family
AT5G10130	-2,8		Pollen Ole e 1 allergen and extensin family protein; FUNCTIONS IN: molecular_function unknown; INVOLVED IN: biological_process unknown; LOCATED IN: extracellular space, endomembrane system; EXPRESSED IN: root; CONTAINS InterPro DOMAIN/s: Pollen Ole e 1 allergen/extensin (InterPro:IPR006041), Allergen Ole e 1, conserved site (InterPro:IPR006040); BEST Arabidopsis thaliana protein match is: Pollen Ole e 1 allergen and extensin family protein (TAIR:AT4G08685.1)
AT1G52820	-2,804		2-oxoglutarate (2OG) and Fe(II)-dependent oxygenase superfamily protein; FUNCTIONS IN: oxidoreductase activity; EXPRESSED IN: root; CONTAINS InterPro DOMAIN/s: Oxoglutarate/iron-dependent oxygenase (InterPro:IPR005123); BEST Arabidopsis thaliana protein match is: 2-oxoglutarate (2OG) and Fe(II)-dependent oxygenase superfamily protein (TAIR:AT4G03070.1)

AT3G62760	*-2,813*	(ATGSTF13)	Encodes glutathione transferase belonging to the phi class of GSTs. Naming convention according to Wagner et al. (2002).
AT4G30320	*-2,816*	(ATCAPE6)	CAP (Cysteine-rich secretory proteins, Antigen 5, and Pathogenesis-related 1 protein) superfamily protein; FUNCTIONS IN: molecular_function unknown; INVOLVED IN: biological_process unknown; LOCATED IN: endomembrane system, extracellular region; EXPRESSED IN: root; CONTAINS InterPro DOMAIN/s: Allergen V5/Tpx-1 related, conserved site (InterPro:IPR018244), Allergen V5/Tpx-1 related (InterPro:IPR001283), Ves allergen (InterPro:IPR002413), SCP-like extracellular (InterPro:IPR014044); BEST Arabidopsis thaliana protein match is: CAP (Cysteine-rich secretory proteins, Antigen 5, and Pathogenesis-related 1 protein) superfamily protein (TAIR:AT5G57625.1)
AT4G40090	*-2,816*	ARABINOGALACTAN PROTEIN 3 (AGP3)	arabinogalactan protein 3 (AGP3); INVOLVED IN: multicellular organismal development; LOCATED IN: endomembrane system; EXPRESSED IN: shoot apex, hypocotyl, root, egg cell; BEST Arabidopsis thaliana protein match is: arabinogalactan protein 2 (TAIR:AT2G22470.1)
AT2G29000	*-2,817*		Leucine-rich repeat protein kinase family protein; FUNCTIONS IN: protein serine/threonine kinase activity, protein kinase activity, ATP binding; INVOLVED IN: protein amino acid phosphorylation; LOCATED IN: endomembrane system; CONTAINS InterPro DOMAIN/s: Protein kinase, ATP binding site (InterPro:IPR017441), Protein kinase, catalytic domain (InterPro:IPR000719), Serine/threonine-protein kinase domain (InterPro:IPR002290), Tyrosine-protein kinase, catalytic domain (InterPro:IPR020635), Serine/threonine-protein kinase-like domain (InterPro:IPR017442), Serine/threonine-protein kinase, active site (InterPro:IPR008271), Protein kinase-like domain (InterPro:IPR011009); BEST Arabidopsis thaliana protein match is: Leucine-rich repeat protein kinase family protein (TAIR:AT2G28960.1)
AT5G51520	*-2,819*		Plant invertase/pectin methylesterase inhibitor superfamily protein; FUNCTIONS IN: enzyme inhibitor activity, pectinesterase inhibitor activity, pectinesterase activity; INVOLVED IN: biological_process unknown; EXPRESSED IN: root; CONTAINS InterPro DOMAIN/s: Pectinesterase inhibitor (InterPro:IPR006501); BEST Arabidopsis thaliana protein match is: Plant invertase/pectin methylesterase inhibitor superfamily protein (TAIR:AT4G25250.1)
AT5G46900	*-2,826*		Bifunctional inhibitor/lipid-transfer protein/seed storage 2S albumin superfamily protein; FUNCTIONS IN: lipid binding; INVOLVED IN: lipid transport; LOCATED IN: endomembrane system; CONTAINS InterPro DOMAIN/s: Bifunctional inhibitor/plant lipid transfer protein/seed storage (InterPro:IPR016140), Plant lipid transfer protein/seed storage/trypsin-alpha amylase inhibitor (InterPro:IPR003612), Plant lipid transfer protein/hydrophobic protein, helical domain (InterPro:IPR013770); BEST Arabidopsis thaliana protein match is: Bifunctional inhibitor/lipid-transfer protein/seed storage 2S albumin superfamily protein (TAIR:AT5G46890.1)
AT3G14850	*-2,829*	TRICHOME BIREFRINGENCE-LIKE 41 (TBL41)	Encodes a member of the TBL (TRICHOME BIREFRINGENCE-LIKE) gene family containing a plant-specific DUF231 (domain of unknown function) domain. TBL gene family has 46 members, two of which (TBR/AT5G06700 and TBL3/AT5G01360) have been shown to be involved in the synthesis and deposition of secondary wall cellulose, presumably by influencing the esterification state of pectic polymers. A nomenclature for this gene family has been proposed (Volker Bischoff & Wolf Scheible, 2010, personal communication).
AT3G52970	*-2,835*	CYTOCHROME P450, FAMILY 76, SUBFAMILY G, POLYPEPTIDE 1 (CYP76G1)	member of CYP76G
AT1G12560	*-2,841*	EXPANSIN A7 (EXPA7)	Member of Alpha-Expansin Gene Family. Naming convention from the Expansin Working Group (Kende et al, 2004. Plant Mol Bio). Containing a conserved root hair-specific cis-element RHE. Expressed specifically in root hair cell and involved in root hair elongation.
AT5G57540	*-2,861*	XYLOGLUCAN ENDOTRANSGLUCOSYLASE/HYDROLASE 13 (XTH13)	Encodes a xyloglucan endotransglucosylase/hydrolase with only only the endotransglucosylase (XET; EC 2.4.1.207) activity towards xyloglucan and non-detectable endohydrolytic (XEH; EC 3.2.1.151) activity.
AT5G60660	*-2,869*	PLASMA MEMBRANE INTRINSIC PROTEIN 2;4 (PIP2;4)	A member of the plasma membrane intrinsic protein subfamily PIP2.When expressed in yeast cells can conduct hydrogen peroxide into those cells. Mutants exhibit longer root hairs.

AT4G15350	*-2,871*	CYTOCHROME P450, FAMILY 705, SUBFAMILY A, POLYPEPTIDE 2 (CYP705A2)	member of CYP705A
AT4G33880	*-2,871*	ROOT HAIR DEFECTIVE 6-LIKE 2 (RSL2)	RSL2 was expressed concurrently with RSL4 and its expression was controlled by RHD6 and RSL1. Required for root-hair growth.
AT3G29780	*-2,877*	RALF-LIKE 27 (RALFL27)	Member of a diversely expressed predicted peptide family showing sequence similarity to tobacco Rapid Alkalinization Factor (RALF), and is believed to play an essential role in the physiology of Arabidopsis. Consists of a single exon and is characterized by a conserved C-terminal motif and N-terminal signal peptide.
AT4G33730	*-2,881*	CAP-DERIVED PEPTIDE 1 (ATCAPE1)	Member of CAP protein superfamily. Encoding a small 11 AA peptide (PAGNYIGARPY) involved in negative regulation of salt tolerance.
AT4G22460	*-2,907*		Bifunctional inhibitor/lipid-transfer protein/seed storage 2S albumin superfamily protein; FUNCTIONS IN: lipid binding; INVOLVED IN: lipid transport; LOCATED IN: endomembrane system; EXPRESSED IN: root; CONTAINS InterPro DOMAIN/s: Bifunctional inhibitor/plant lipid transfer protein/seed storage (InterPro:IPR016140), Plant lipid transfer protein/seed storage/trypsin-alpha amylase inhibitor (InterPro:IPR003612), Plant lipid transfer protein/hydrophobic protein, helical domain (InterPro:IPR013770); BEST Arabidopsis thaliana protein match is: Bifunctional inhibitor/lipid-transfer protein/seed storage 2S albumin superfamily protein (TAIR:AT4G12510.1)
AT5G58360	*-2,908*	OVATE FAMILY PROTEIN 3 (OFP3)	ovate family protein 3 (OFP3); CONTAINS InterPro DOMAIN/s: Protein of unknown function DUF623 (InterPro:IPR006458); BEST Arabidopsis thaliana protein match is: ovate family protein 4 (TAIR:AT1G06920.1)
AT2G44110	*-2,911*	MILDEW RESISTANCE LOCUS O 15 (MLO15)	A member of a large family of seven-transmembrane domain proteins specific to plants, homologs of the barley mildew resistance locus o (MLO) protein. The Arabidopsis genome contains 15 genes encoding MLO proteins, with localization in plasma membrane. Phylogenetic analysis revealed four clades of closely-related AtMLO genes. ATMLO15 belongs to the clade II, with ATMLO13 and ATMLO15. The gene is expressed during early seedling growth, in root tips and flower (papillae, anthers and pollen grains), as shown by GUS activity patterns. The expression of several phylogenetically closely-related AtMLO genes showed similar or overlapping tissue specificity and analogous responsiveness to external stimuli, suggesting functional redundancy, co-function, or antagonistic function(s).
AT5G26080	*-2,916*		proline-rich family protein; LOCATED IN: endomembrane system; BEST Arabidopsis thaliana protein match is: hydroxyproline-rich glycoprotein family protein (TAIR:AT5G26070.1)
AT2G21880	*-2,92*	RAB GTPASE HOMOLOG 7A (RAB7A)	RAB GTPase homolog 7A (RAB7A); FUNCTIONS IN: GTP binding; INVOLVED IN: protein transport, small GTPase mediated signal transduction; EXPRESSED IN: 24 plant structures; EXPRESSED DURING: 14 growth stages; CONTAINS InterPro DOMAIN/s: Ras GTPase (InterPro:IPR001806), Small GTP-binding protein (InterPro:IPR005225), Small GTPase (InterPro:IPR020851), Ras (InterPro:IPR013753), Ras small GTPase, Rab type (InterPro:IPR003579); BEST Arabidopsis thaliana protein match is: RAB GTPase homolog G3A (TAIR:AT4G09720.1)
AT2G24980	*-2,928*	EXTENSIN 6 (EXT6)	Proline-rich extensin-like family protein; FUNCTIONS IN: structural constituent of cell wall; INVOLVED IN: plant-type cell wall organization; LOCATED IN: endomembrane system; EXPRESSED IN: root; CONTAINS InterPro DOMAIN/s: Extensin-like repeat (InterPro:IPR006706); BEST Arabidopsis thaliana protein match is: proline-rich extensin-like family protein (TAIR:AT5G35190.1)
AT4G23496	*-2,937*	SPIRAL1-LIKE5 (SP1L5)	Belongs to a six-member gene family in Arabidopsis; all members share high sequence similarity in amino- and carboxy-terminal regions. Regulates cortical microtubule organization. Mutant plants exhibit altered patterns of root, leaf and petal growth as a result of defective anisotropic cell expansion.
AT4G14280	*-2,941*		ARM repeat superfamily protein; FUNCTIONS IN: binding; INVOLVED IN: biological_process unknown; LOCATED IN: cellular_component unknown; CONTAINS InterPro DOMAIN/s: Armadillo-like helical (InterPro:IPR011989), Armadillo-type fold (InterPro:IPR016024); BEST Arabidopsis thaliana protein match is: ARM repeat superfamily protein (TAIR:AT5G18980.1)

AT3G07070	*-2,951*		Protein kinase superfamily protein; FUNCTIONS IN: protein serine/threonine kinase activity, protein kinase activity, kinase activity, ATP binding; INVOLVED IN: protein amino acid phosphorylation; LOCATED IN: cellular_component unknown; EXPRESSED IN: male gametophyte, flower, pollen tube; EXPRESSED DURING: L mature pollen stage, M germinated pollen stage, 4 anthesis; CONTAINS InterPro DOMAIN/s: Protein kinase, ATP binding site (InterPro:IPR017441), Protein kinase, catalytic domain (InterPro:IPR000719), Serine/threonine-protein kinase-like domain (InterPro:IPR017442), Protein kinase-like domain (InterPro:IPR011009), Serine/threonine-protein kinase, active site (InterPro:IPR008271); BEST Arabidopsis thaliana protein match is: Protein kinase superfamily protein (TAIR:AT3G24790.1)
AT3G15370	*-2,957*	EXPANSIN 12 (EXPA12)	member of Alpha-Expansin Gene Family. Naming convention from the Expansin Working Group (Kende et al, 2004. Plant Mol Bio)
AT4G12510	*-2,963*	(AZI5)	Encodes a member of the AZI family of lipid transfer proteins.
AT4G08410	*-2,974*		Proline-rich extensin-like family protein; FUNCTIONS IN: structural constituent of cell wall; INVOLVED IN: plant-type cell wall organization; EXPRESSED IN: shoot apex, embryo, hypocotyl, sepal, root; EXPRESSED DURING: 4 anthesis, E expanded cotyledon stage; CONTAINS InterPro DOMAIN/s: Extensin-like repeat (InterPro:IPR006706); BEST Arabidopsis thaliana protein match is: Proline-rich extensin-like family protein (TAIR:AT4G08400.1)
AT2G42850	*-2,98*	CYTOCHROME P450, FAMILY 718 (CYP718)	member of CYP718
AT1G05660	*-2,993*		Pectin lyase-like superfamily protein; FUNCTIONS IN: polygalacturonase activity; INVOLVED IN: carbohydrate metabolic process; LOCATED IN: endomembrane system; CONTAINS InterPro DOMAIN/s: Pectin lyase fold/virulence factor (InterPro:IPR011050), Pectin lyase fold (InterPro:IPR012334), Glycoside hydrolase, family 28 (InterPro:IPR000743), Parallel beta-helix repeat (InterPro:IPR006626); BEST Arabidopsis thaliana protein match is: Pectin lyase-like superfamily protein (TAIR:AT1G05650.1)
AT4G01890	*-3,007*		Pectin lyase-like superfamily protein; FUNCTIONS IN: polygalacturonase activity; INVOLVED IN: carbohydrate metabolic process; LOCATED IN: endomembrane system; EXPRESSED IN: root; CONTAINS InterPro DOMAIN/s: Pectin lyase fold/virulence factor (InterPro:IPR011050), Parallel beta-helix repeat (InterPro:IPR006626), Pectin lyase fold (InterPro:IPR012334), Glycoside hydrolase, family 28 (InterPro:IPR000743); BEST Arabidopsis thaliana protein match is: Pectin lyase-like superfamily protein (TAIR:AT1G02460.1)
AT4G25820	*-3,017*	XYLOGLUCAN ENDOTRANSGLUCOSYLASE/HYDROLASE 14 (XTH14)	Encodes a xyloglucan endotransglycosylase with a clear preference for non-fucosylated xyloglucan polymer. The mRNA is cell-to-cell mobile.
AT1G74490	*-3,024*		Protein kinase superfamily protein; FUNCTIONS IN: protein serine/threonine kinase activity, protein kinase activity, kinase activity, ATP binding; INVOLVED IN: protein amino acid phosphorylation; LOCATED IN: cellular_component unknown; EXPRESSED IN: 13 plant structures; EXPRESSED DURING: L mature pollen stage, M germinated pollen stage, 4 anthesis, C globular stage, petal differentiation and expansion stage; CONTAINS InterPro DOMAIN/s: Protein kinase, ATP binding site (InterPro:IPR017441), Protein kinase, catalytic domain (InterPro:IPR000719), Serine/threonine-protein kinase-like domain (InterPro:IPR017442), Protein kinase-like domain (InterPro:IPR011009), Serine/threonine-protein kinase, active site (InterPro:IPR008271); BEST Arabidopsis thaliana protein match is: protein kinase 2B (TAIR:AT2G02800.2)
AT1G48670	*-3,03*		auxin-responsive GH3 family protein; CONTAINS InterPro DOMAIN/s: GH3 auxin-responsive promoter (InterPro:IPR004993); BEST Arabidopsis thaliana protein match is: Auxin-responsive GH3 family protein (TAIR:AT1G48660.1)
AT4G17340	*-3,032*	TONOPLAST INTRINSIC PROTEIN 2;2 (TIP2;2)	tonoplast intrinsic protein 2;2 (TIP2;2); FUNCTIONS IN: water channel activity; INVOLVED IN: response to salt stress, transport; LOCATED IN: in 6 components; EXPRESSED IN: 25 plant structures; EXPRESSED DURING: 10 growth stages; CONTAINS InterPro DOMAIN/s: Major intrinsic protein, conserved site (InterPro:IPR022357), Aquaporin (InterPro:IPR012269), Major intrinsic protein (InterPro:IPR000425); BEST Arabidopsis thaliana protein match is: tonoplast intrinsic protein 2;3 (TAIR:AT5G47450.1)
AT5G60770	*-3,05*	NITRATE TRANSPORTER 2.4 (NRT2.4)	member of High affinity nitrate transporter family

AT3G29430	-3,057		Terpenoid synthases superfamily protein; INVOLVED IN: isoprenoid biosynthetic process; EXPRESSED IN: root; CONTAINS InterPro DOMAIN/s: Polyprenyl synthetase-related (InterPro:IPR017446), Terpenoid synthase (InterPro:IPR008949), Polyprenyl synthetase (InterPro:IPR000092); BEST Arabidopsis thaliana protein match is: Terpenoid synthases superfamily protein (TAIR:AT3G32040.1)
AT5G06630	-3,065		proline-rich extensin-like family protein; FUNCTIONS IN: structural constituent of cell wall; INVOLVED IN: plant-type cell wall organization; LOCATED IN: endomembrane system; EXPRESSED IN: leaf whorl, hypocotyl, male gametophyte, root, leaf; EXPRESSED DURING: 6 growth stages; CONTAINS InterPro DOMAIN/s: Extensin-like repeat (InterPro:IPR006706); BEST Arabidopsis thaliana protein match is: Proline-rich extensin-like family protein (TAIR:AT2G24980.1)
AT1G14160	-3,084	CASP-LIKE PROTEIN 1A1 (CASPL1A1)	Uncharacterised protein family (UPF0497); CONTAINS InterPro DOMAIN/s: Uncharacterised protein family UPF0497, trans-membrane plant (InterPro:IPR006702), Uncharacterised protein family UPF0497, trans-membrane plant subgroup (InterPro:IPR006459); BEST Arabidopsis thaliana protein match is: Uncharacterised protein family (UPF0497) (TAIR:AT5G15290.1)
AT1G73580	-3,096	C2-DOMAIN ABA-RELATED3 (CAR3)	Calcium-dependent lipid-binding (CaLB domain) family protein; CONTAINS InterPro DOMAIN/s: C2 membrane targeting protein (InterPro:IPR018029), C2 calcium/lipid-binding domain, CaLB (InterPro:IPR008973), C2 calcium-dependent membrane targeting (InterPro:IPR000008); BEST Arabidopsis thaliana protein match is: Calcium-dependent lipid-binding (CaLB domain) family protein (TAIR:AT3G17980.1)
AT3G02100	-3,13		UDP-Glycosyltransferase superfamily protein; FUNCTIONS IN: UDP-glycosyltransferase activity, transferase activity, transferring glycosyl groups; INVOLVED IN: metabolic process; LOCATED IN: cellular_component unknown; EXPRESSED IN: root; CONTAINS InterPro DOMAIN/s: UDP-glucuronosyl/UDP-glucosyltransferase (InterPro:IPR002213); BEST Arabidopsis thaliana protein match is: UDP-Glycosyltransferase superfamily protein (TAIR:AT1G05675.1)
AT5G59320	-3,134	LIPID TRANSFER PROTEIN 3 (LTP3)	Predicted to encode a PR (pathogenesis-related) protein. Belongs to the lipid transfer protein (PR-14) family with the following members: At2g38540/LTP1, At2g38530/LTP2, At5g59320/LTP3, At5g59310/LTP4, At3g51600/LTP5, At3g08770/LTP6, At2g15050/LTP7, At2g18370/LTP8, At2g15325/LTP9, At5g01870/LTP10, At4g33355/LTP11, At3g51590/LTP12, At5g44265/LTP13, At5g62065/LTP14, At4g08530/LTP15. The mRNA is cell-to-cell mobile.
AT4G13390	-3,146	EXTENSIN 12 (EXT12)	Proline-rich extensin-like family protein; FUNCTIONS IN: structural constituent of cell wall; INVOLVED IN: plant-type cell wall organization; LOCATED IN: endomembrane system; CONTAINS InterPro DOMAIN/s: Extensin-like repeat (InterPro:IPR006706); BEST Arabidopsis thaliana protein match is: Proline-rich extensin-like family protein (TAIR:AT5G06640.1)
AT4G37160	-3,167	SKU5 SIMILAR 15 (sks15)	SKU5 similar 15 (sks15); FUNCTIONS IN: oxidoreductase activity, copper ion binding; INVOLVED IN: oxidation reduction; LOCATED IN: endomembrane system; EXPRESSED IN: root; CONTAINS InterPro DOMAIN/s: Multicopper oxidase, type 3 (InterPro:IPR011707), Cupredoxin (InterPro:IPR008972), Multicopper oxidase, type 2 (InterPro:IPR011706), Multicopper oxidase, type 1 (InterPro:IPR001117); BEST Arabidopsis thaliana protein match is: SKU5 similar 16 (TAIR:AT2G23630.1)
AT2G45050	-3,193	GATA TRANSCRIPTION FACTOR 2 (GATA2)	Encodes a member of the GATA factor family of zinc finger transcription factors. A positive regulator of photomorphogenesis.
AT1G34540	-3,205	CYTOCHROME P450, FAMILY 94, SUBFAMILY D, POLYPEPTIDE 1 (CYP94D1)	member of CYP94D
AT3G04320	-3,211		Kunitz family trypsin and protease inhibitor protein; FUNCTIONS IN: endopeptidase inhibitor activity; INVOLVED IN: biological_process unknown; LOCATED IN: endomembrane system; CONTAINS InterPro DOMAIN/s: Proteinase inhibitor I3, Kunitz legume (InterPro:IPR002160), Kunitz inhibitor ST1-like (InterPro:IPR011065); BEST Arabidopsis thaliana protein match is: Kunitz family trypsin and protease inhibitor protein (TAIR:AT3G04330.1)
AT1G06120	-3,231		Fatty acid desaturase family protein; FUNCTIONS IN: oxidoreductase activity, oxidoreductase activity, acting on paired donors, with oxidation of a pair of donors resulting in the reduction of molecular oxygen to two molecules of water; INVOLVED IN: oxidation reduction, lipid metabolic process; CONTAINS InterPro DOMAIN/s: Fatty acid desaturase, type 1, core (InterPro:IPR015876), Fatty acid desaturase, type 1 (InterPro:IPR005804); BEST Arabidopsis thaliana protein match is: Fatty acid desaturase family protein (TAIR:AT1G06090.1)

AT5G04960	*-3,231*		Plant invertase/pectin methylesterase inhibitor superfamily; FUNCTIONS IN: enzyme inhibitor activity, pectinesterase activity; INVOLVED IN: cell wall modification; LOCATED IN: cell wall, plant-type cell wall; EXPRESSED IN: flower, root; CONTAINS InterPro DOMAIN/s: Pectinesterase, active site (InterPro:IPR018040), Pectin lyase fold/virulence factor (InterPro:IPR011050), Pectinesterase, catalytic (InterPro:IPR000070), Pectinesterase inhibitor (InterPro:IPR006501), Pectin lyase fold (InterPro:IPR012334); BEST Arabidopsis thaliana protein match is: root hair specific 12 (TAIR:AT3G10710.1)
AT5G01740	*-3,251*		Nuclear transport factor 2 (NTF2) family protein; CONTAINS InterPro DOMAIN/s: Wound-induced protein, Wun1 (InterPro:IPR009798); BEST Arabidopsis thaliana protein match is: senescence associated gene 20 (TAIR:AT3G10985.1)
AT4G08400	*-3,258*		Proline-rich extensin-like family protein; FUNCTIONS IN: structural constituent of cell wall; INVOLVED IN: plant-type cell wall organization; LOCATED IN: cellular_component unknown; EXPRESSED IN: male gametophyte; EXPRESSED DURING: M germinated pollen stage; CONTAINS InterPro DOMAIN/s: Extensin-like repeat (InterPro:IPR006706); BEST Arabidopsis thaliana protein match is: Proline-rich extensin-like family protein (TAIR:AT4G08410.1)
AT5G57530	*-3,27*	XYLOGLUCAN ENDOTRANSGLUCOSYLASE/HYDROLASE 12 (XTH12)	xyloglucan endotransglucosylase/hydrolase 12 (XTH12); CONTAINS InterPro DOMAIN/s: Xyloglucan endotransglucosylase/hydrolase (InterPro:IPR016455), Xyloglucan endo-transglycosylase, C-terminal (InterPro:IPR010713), Concanavalin A-like lectin/glucanase, subgroup (InterPro:IPR013320), Concanavalin A-like lectin/glucanase (InterPro:IPR008985), Glycoside hydrolase, family 16 (InterPro:IPR000757), Glycoside hydrolase, family 16, active site (InterPro:IPR008263); BEST Arabidopsis thaliana protein match is: xyloglucan endotransglucosylase/hydrolase 13 (TAIR:AT5G57540.1)
AT1G34510	*-3,289*		Peroxidase superfamily protein; FUNCTIONS IN: peroxidase activity, heme binding; INVOLVED IN: oxidation reduction, response to oxidative stress; LOCATED IN: endomembrane system; EXPRESSED IN: root; CONTAINS InterPro DOMAIN/s: Haem peroxidase (InterPro:IPR010255), Plant peroxidase (InterPro:IPR000823), Peroxidase, active site (InterPro:IPR019794), Haem peroxidase, plant/fungal/bacterial (InterPro:IPR002016); BEST Arabidopsis thaliana protein match is: Peroxidase superfamily protein (TAIR:AT4G26010.1)
AT3G47710	*-3,294*	BANQUO 3 (BNQ3)	BNQ3 belongs to a family of atypical non-DNA binding basic helix-loop-helix (bHLH) proteins that heterodimerize with and negatively regulate bHLH transcription factors. Directly and negatively regulated by AP3 and PI in petals. Required for appropriate regulation of flowering time.May also have a role in regulating light responses.
AT5G42510	*-3,311*		Disease resistance-responsive (dirigent-like protein) family protein; FUNCTIONS IN: molecular_function unknown; INVOLVED IN: defense response; LOCATED IN: endomembrane system; CONTAINS InterPro DOMAIN/s: Plant disease resistance response protein (InterPro:IPR004265); BEST Arabidopsis thaliana protein match is: Disease resistance-responsive (dirigent-like protein) family protein (TAIR:AT5G42500.1)
AT4G08290	*-3,322*	USUALLY MULTIPLE ACIDS MOVE IN AND OUT TRANSPORTERS 20 (UMAMIT20)	nodulin MtN21-like transporter family protein
AT4G28850	*-3,328*	XYLOGLUCAN ENDOTRANSGLUCOSYLASE/HYDROLASE 26 (XTH26)	xyloglucan endotransglucosylase/hydrolase 26 (XTH26); FUNCTIONS IN: hydrolase activity, acting on glycosyl bonds, hydrolase activity, hydrolyzing O-glycosyl compounds, xyloglucan:xyloglucosyl transferase activity; INVOLVED IN: carbohydrate metabolic process, cellular glucan metabolic process; LOCATED IN: endomembrane system, apoplast, cell wall; EXPRESSED IN: root; CONTAINS InterPro DOMAIN/s: Xyloglucan endotransglucosylase/hydrolase (InterPro:IPR016455), Xyloglucan endo-transglycosylase, C-terminal (InterPro:IPR010713), Concanavalin A-like lectin/glucanase, subgroup (InterPro:IPR013320), Concanavalin A-like lectin/glucanase (InterPro:IPR008985), Glycoside hydrolase, family 16 (InterPro:IPR000757), Glycoside hydrolase, family 16, active site (InterPro:IPR008263); BEST Arabidopsis thaliana protein match is: xyloglucan endotransglucosylase/hydrolase 16 (TAIR:AT3G23730.1)

AT2G45220	*-3,343*	PECTIN METHYLESTERASE 17 (PME17)	Plant invertase/pectin methylesterase inhibitor superfamily; FUNCTIONS IN: enzyme inhibitor activity, pectinesterase activity; INVOLVED IN: cell wall modification; LOCATED IN: membrane, plant-type cell wall; EXPRESSED IN: 9 plant structures; EXPRESSED DURING: 4 anthesis, C globular stage, petal differentiation and expansion stage; CONTAINS InterPro DOMAIN/s: Carbohydrate-binding/sugar hydrolysis domain (InterPro:IPR006633), Pectinesterase, active site (InterPro:IPR018040), Pectin lyase fold/virulence factor (InterPro:IPR011050), Pectinesterase, catalytic (InterPro:IPR000070), Pectinesterase inhibitor (InterPro:IPR006501), Pectin lyase fold (InterPro:IPR012334); BEST Arabidopsis thaliana protein match is: Plant invertase/pectin methylesterase inhibitor superfamily (TAIR:AT5G51490.1)
AT1G78230	*-3,349*		Outer arm dynein light chain 1 protein; INVOLVED IN: biological_process unknown; LOCATED IN: vacuole; CONTAINS InterPro DOMAIN/s: Leucine-rich repeat, typical subtype (InterPro:IPR003591), Leucine-rich repeat (InterPro:IPR001611); BEST Arabidopsis thaliana protein match is: Outer arm dynein light chain 1 protein (TAIR:AT4G03260.2)
AT3G04330	*-3,349*		Kunitz family trypsin and protease inhibitor protein; FUNCTIONS IN: endopeptidase inhibitor activity; INVOLVED IN: biological_process unknown; LOCATED IN: endomembrane system; CONTAINS InterPro DOMAIN/s: Proteinase inhibitor I3, Kunitz legume (InterPro:IPR002160), Kunitz inhibitor ST1-like (InterPro:IPR011065); BEST Arabidopsis thaliana protein match is: Kunitz family trypsin and protease inhibitor protein (TAIR:AT3G04320.1)
AT4G08300	*-3,419*	USUALLY MULTIPLE ACIDS MOVE IN AND OUT TRANSPORTERS 17 (UMAMIT17)	nodulin MtN21-like transporter family protein
AT2G43600	*-3,439*		Chitinase family protein; FUNCTIONS IN: chitin binding, chitinase activity; INVOLVED IN: carbohydrate metabolic process, cell wall macromolecule catabolic process; LOCATED IN: endomembrane system; EXPRESSED IN: shoot apex, sperm cell, root; CONTAINS InterPro DOMAIN/s: Chitin-binding, type 1, conserved site (InterPro:IPR018371), Glycoside hydrolase, family 19 (InterPro:IPR016283), Chitin-binding, type 1 (InterPro:IPR001002), Glycoside hydrolase, family 19, catalytic (InterPro:IPR000726); BEST Arabidopsis thaliana protein match is: Chitinase family protein (TAIR:AT1G56680.1)
AT2G43050	*-3,44*	(ATPMEPCRD)	ATPMEPCRD; FUNCTIONS IN: enzyme inhibitor activity, pectinesterase activity; INVOLVED IN: cell wall modification; LOCATED IN: cell wall, plant-type cell wall; EXPRESSED IN: 9 plant structures; EXPRESSED DURING: 4 anthesis, petal differentiation and expansion stage; CONTAINS InterPro DOMAIN/s: Pectinesterase, active site (InterPro:IPR018040), Pectin lyase fold/virulence factor (InterPro:IPR011050), Pectinesterase, catalytic (InterPro:IPR000070), Pectinesterase inhibitor (InterPro:IPR006501), Pectin lyase fold (InterPro:IPR012334); BEST Arabidopsis thaliana protein match is: pectin methylesterase 61 (TAIR:AT3G59010.1)
AT4G15160	*-3,466*		Bifunctional inhibitor/lipid-transfer protein/seed storage 2S albumin superfamily protein; FUNCTIONS IN: lipid binding; INVOLVED IN: lipid transport; LOCATED IN: endomembrane system; EXPRESSED IN: 18 plant structures; EXPRESSED DURING: 9 growth stages; CONTAINS InterPro DOMAIN/s: Bifunctional inhibitor/plant lipid transfer protein/seed storage (InterPro:IPR016140), Plant lipid transfer protein/hydrophobic protein, helical domain (InterPro:IPR013770); BEST Arabidopsis thaliana protein match is: cell wall-plasma membrane linker protein (TAIR:AT3G22120.1)
AT4G37950	*-3,48*		Rhamnogalacturonate lyase family protein; CONTAINS InterPro DOMAIN/s: Rhamnogalacturonate lyase (InterPro:IPR010325), Carbohydrate-binding-like fold (InterPro:IPR013784), Galactose-binding domain-like (InterPro:IPR008979); BEST Arabidopsis thaliana protein match is: Rhamnogalacturonate lyase family protein (TAIR:AT2G22620.1)
AT4G27400	*-3,582*		Late embryogenesis abundant (LEA) protein-related; INVOLVED IN: response to salt stress; LOCATED IN: endomembrane system; EXPRESSED IN: sepal, root; EXPRESSED DURING: petal differentiation and expansion stage; CONTAINS InterPro DOMAIN/s: Root cap (InterPro:IPR009646); BEST Arabidopsis thaliana protein match is: Late embryogenesis abundant (LEA) protein-related (TAIR:AT5G54370.1)
AT5G22410	*-3,609*	ROOT HAIR SPECIFIC 18 (RHS18)	root hair specific 18 (RHS18); FUNCTIONS IN: peroxidase activity, heme binding; INVOLVED IN: oxidation reduction, response to oxidative stress; LOCATED IN: endomembrane system; EXPRESSED IN: root hair, root, synergid; CONTAINS InterPro DOMAIN/s: Haem peroxidase (InterPro:IPR010255), Plant peroxidase (InterPro:IPR000823), Peroxidases heam-ligand binding site (InterPro:IPR019793), Peroxidase, active site (InterPro:IPR019794), Haem peroxidase, plant/fungal/bacterial (InterPro:IPR002016); BEST Arabidopsis thaliana protein match is: Peroxidase superfamily protein (TAIR:AT5G17820.1)

AT2G31083	*-3,616*	CLAVATA3/ESR-RELATED 5 (CLE5)	Member of a large family of putative ligands homologous to the Clavata3 gene. Consists of a single exon.
AT5G06905	*-3,725*	CYTOCHROME P450, FAMILY 712, SUBFAMILY A, POLYPEPTIDE 2 (CYP712A2)	member of CYP712A
AT1G65570	*-3,773*	ROOT CAP POLYGALACTURONASE (RCPG)	Encodes a glycosyl hydrolase 28 (GH28) family polygalacturonase (PG) protein. Involved in root cap development.
AT1G54970	*-3,79*	PROLINE-RICH PROTEIN 1	encodes a proline-rich protein that is specifically expressed in the root. The mRNA is cell-to-cell mobile.
AT1G65310	*-3,825*	XYLOGLUCAN ENDOTRANSGLUCOSYLASE/HYDROLASE 17 (XTH17)	Encodes a xyloglucan endotransglucosylase/hydrolase with only only the endotransglucosylase (XET; EC 2.4.1.207) activity towards xyloglucan and non-detectable endohydrolytic (XEH; EC 3.2.1.151) activity. Expressed in the mature or basal regions of both the main and lateral roots, but not in the tip of these roots where cell division occurs.
AT5G24070	*-3,835*		Peroxidase superfamily protein; FUNCTIONS IN: peroxidase activity, heme binding; INVOLVED IN: response to oxidative stress, oxidation reduction; LOCATED IN: endomembrane system; EXPRESSED IN: root; CONTAINS InterPro DOMAIN/s: Haem peroxidase (InterPro:IPR010255), Plant peroxidase (InterPro:IPR000823), Peroxidases heam-ligand binding site (InterPro:IPR019793), Haem peroxidase, plant/fungal/bacterial (InterPro:IPR002016); BEST Arabidopsis thaliana protein match is: Peroxidase superfamily protein (TAIR:AT2G43480.1)
AT1G50060	*-3,936*		CAP (Cysteine-rich secretory proteins, Antigen 5, and Pathogenesis-related 1 protein) superfamily protein; FUNCTIONS IN: molecular_function unknown; INVOLVED IN: biological_process unknown; LOCATED IN: endomembrane system, extracellular region; EXPRESSED IN: root; CONTAINS InterPro DOMAIN/s: Allergen V5/Tpx-1 related (InterPro:IPR001283), Ves allergen (InterPro:IPR002413), SCP-like extracellular (InterPro:IPR014044); BEST Arabidopsis thaliana protein match is: CAP (Cysteine-rich secretory proteins, Antigen 5, and Pathogenesis-related 1 protein) superfamily protein (TAIR:AT1G50050.1)
AT4G15360	*-3,956*	CYTOCHROME P450, FAMILY 705, SUBFAMILY A, POLYPEPTIDE 3 (CYP705A3)	member of CYP705A
AT2G20520	*-3,989*	FASCICLIN-LIKE ARABINOGALACTAN 6 (FLA6)	fasciclin-like arabinogalactan-protein 6 (Fla6)
AT1G33320	*-3,998*		Pyridoxal phosphate (PLP)-dependent transferases superfamily protein; FUNCTIONS IN: pyridoxal phosphate binding, cystathionine gamma-synthase activity, catalytic activity; INVOLVED IN: methionine biosynthetic process, cellular amino acid metabolic process; EXPRESSED IN: root; CONTAINS InterPro DOMAIN/s: Pyridoxal phosphate-dependent transferase, major domain (InterPro:IPR015424), Cys/Met metabolism, pyridoxal phosphate-dependent enzyme (InterPro:IPR000277), Pyridoxal phosphate-dependent transferase, major region, subdomain 1 (InterPro:IPR015421), Pyridoxal phosphate-dependent transferase, major region, subdomain 2 (InterPro:IPR015422); BEST Arabidopsis thaliana protein match is: Pyridoxal phosphate (PLP)-dependent transferases superfamily protein (TAIR:AT3G01120.1)
AT4G39000	*-4,108*	GLYCOSYL HYDROLASE 9B17 (GH9B17)	glycosyl hydrolase 9B17 (GH9B17); FUNCTIONS IN: hydrolase activity, hydrolyzing O-glycosyl compounds, catalytic activity; INVOLVED IN: carbohydrate metabolic process; LOCATED IN: endomembrane system; EXPRESSED IN: petal, flower; EXPRESSED DURING: 4 anthesis, petal differentiation and expansion stage; CONTAINS InterPro DOMAIN/s: Six-hairpin glycosidase (InterPro:IPR012341), Six-hairpin glycosidase-like (InterPro:IPR008928), Glycoside hydrolase, family 9 (InterPro:IPR001701); BEST Arabidopsis thaliana protein match is: glycosyl hydrolase 9B18 (TAIR:AT4G39010.1)
AT5G35190	*-4,145*	EXTENSIN 13 (EXT13)	proline-rich extensin-like family protein; FUNCTIONS IN: structural constituent of cell wall; INVOLVED IN: plant-type cell wall organization; LOCATED IN: endomembrane system; EXPRESSED IN: root; CONTAINS InterPro DOMAIN/s: Extensin-like repeat (InterPro:IPR006706); BEST Arabidopsis thaliana protein match is: Proline-rich extensin-like family protein (TAIR:AT4G13390.1)

AT1G19900	*-4,156*		glyoxal oxidase-related protein; FUNCTIONS IN: molecular_function unknown; INVOLVED IN: biological_process unknown; LOCATED IN: endomembrane system; EXPRESSED IN: root; CONTAINS InterPro DOMAIN/s: Galactose oxidase/kelch, beta-propeller (InterPro:IPR011043), Galactose oxidase, beta-propeller (InterPro:IPR015916), Immunoglobulin E-set (InterPro:IPR014756), Glyoxal oxidase, N-terminal (InterPro:IPR009880), Domain of unknown function DUF1929 (InterPro:IPR015202); BEST Arabidopsis thaliana protein match is: glyoxal oxidase-related protein (TAIR:AT1G75620.1)
AT4G15370	*-4,172*	BARUOL SYNTHASE 1 (BARS1)	Encodes an oxidosqualene cyclase that primarily produces the tetracyclic triterpene baruol in vitro and when expressed in yeast. It can also make 22 other minor triterpenoid products with varying numbers of rings.
AT1G52050	*-4,454*		Mannose-binding lectin superfamily protein; FUNCTIONS IN: molecular_function unknown; INVOLVED IN: biological_process unknown; LOCATED IN: endomembrane system; EXPRESSED IN: 7 plant structures; EXPRESSED DURING: 4 anthesis, E expanded cotyledon stage; CONTAINS InterPro DOMAIN/s: Mannose-binding lectin (InterPro:IPR001229); BEST Arabidopsis thaliana protein match is: Mannose-binding lectin superfamily protein (TAIR:AT1G52070.1)
AT5G54370	*-4,543*		Late embryogenesis abundant (LEA) protein-related; FUNCTIONS IN: molecular_function unknown; INVOLVED IN: biological_process unknown; LOCATED IN: endomembrane system; EXPRESSED IN: root; CONTAINS InterPro DOMAIN/s: Root cap (InterPro:IPR009646); BEST Arabidopsis thaliana protein match is: Late embryogenesis abundant (LEA) protein-related (TAIR:AT4G27400.1)
AT1G52060	*-4,588*		Mannose-binding lectin superfamily protein; FUNCTIONS IN: molecular_function unknown; INVOLVED IN: biological_process unknown; LOCATED IN: endomembrane system; EXPRESSED IN: root; CONTAINS InterPro DOMAIN/s: Mannose-binding lectin (InterPro:IPR001229); BEST Arabidopsis thaliana protein match is: Mannose-binding lectin superfamily protein (TAIR:AT1G52070.1)
AT2G33790	*-5,103*	ARABINOGALACTAN PROTEIN 30 (AGP30)	pollen Ole e 1 allergen protein containing 14.6% proline residues, similar to arabinogalactan protein (Daucus carota) GI:11322245, SP:Q03211 Pistil-specific extensin-like protein precursor (PELP) {Nicotiana tabacum}; contains Pfam profile PF01190: Pollen proteins Ole e I family
AT1G52070	*-5,528*		Mannose-binding lectin superfamily protein; FUNCTIONS IN: molecular_function unknown; INVOLVED IN: biological_process unknown; LOCATED IN: endomembrane system; EXPRESSED IN: root; CONTAINS InterPro DOMAIN/s: Mannose-binding lectin (InterPro:IPR001229); BEST Arabidopsis thaliana protein match is: Mannose-binding lectin superfamily protein (TAIR:AT1G52060.1)
