# Supplementary material for: The Role of Programmed Cell Death Regulator LSD1 in Nematode-Induced Syncytium Formation
Source: Front Plant Sci. 2018 Mar 19;9:314. doi: 10.3389/fpls.2018.00314 (PMC5868158; doi:10.3389/fpls.2018.00314)
Supplement: Supplementary file 2 [file Table_2.PDF]

Supplementary Table 2 - RNA-seq analysis of syncytia induced in *lsd1* (Col0) mutant

lsd1 infected vs lsd1 uninfected			
**Locus ID**	**log2FC**	**Primary Gene Symbol**	**Gene Model Description**
AT2G41240	*4,006*	BASIC HELIX-LOOP-HELIX PROTEIN 100 (BHLH100)	Encodes a member of the basic helix-loop-helix transcription factor family protein. Functions as a key regulator of iron-deficiency responses independent of the master regulator FIT. Likely regulates genes involved in the distribution of iron within the plant.
AT5G52300	*3,956*	LOW-TEMPERATURE-INDUCED 65 (LTI65)	Encodes a protein that is induced in expression in response to water deprivation such as cold, high-salt, and desiccation. The response appears to be via abscisic acid. The promoter region contains two ABA-responsive elements (ABREs) that are required for the dehydration-responsive expression of rd29B as cis-acting elements. Protein is a member of a gene family with other members found plants, animals and fungi.
AT3G60120	*3,804*	BETA GLUCOSIDASE 27 (BGLU27)	beta glucosidase 27 (BGLU27); FUNCTIONS IN: cation binding, hydrolase activity, hydrolyzing O-glycosyl compounds, catalytic activity; INVOLVED IN: carbohydrate metabolic process; LOCATED IN: cellular_component unknown; EXPRESSED IN: stem, hypocotyl, sepal, stamen; EXPRESSED DURING: 4 anthesis; CONTAINS InterPro DOMAIN/s: Glycoside hydrolase, family 1 (InterPro:IPR001360), Glycoside hydrolase, family 1, active site (InterPro:IPR018120), Glycoside hydrolase, catalytic core (InterPro:IPR017853), Glycoside hydrolase, subgroup, catalytic core (InterPro:IPR013781); BEST Arabidopsis thaliana protein match is: Glycosyl hydrolase superfamily protein (TAIR:AT2G44490.1)
AT1G69930	*3,71*	GLUTATHIONE S-TRANSFERASE TAU 11	Encodes glutathione transferase belonging to the tau class of GSTs. Naming convention according to Wagner et al. (2002).
AT1G68460	*3,626*	ISOPENTENYLTRANSFERASE 1 (IPT1)	Encodes a putative adenylate isopentenyltransferase. It catalyzes the formation of isopentenyladenosine 5'-monophosphate (iPMP) from AMP and dimethylallylpyrophosphate (DMAPP), but it has a lower Km for ADP and likely works using ADP or ATP in plants. It is involved in cytokinin biosynthesis.
AT1G70130	*3,468*	L-TYPE LECTIN RECEPTOR KINASE V.2 (LECRK-V.2)	Concanavalin A-like lectin protein kinase family protein; FUNCTIONS IN: kinase activity; INVOLVED IN: protein amino acid phosphorylation; LOCATED IN: endomembrane system; EXPRESSED IN: hypocotyl, male gametophyte, root; CONTAINS InterPro DOMAIN/s: Legume lectin, beta chain (InterPro:IPR001220), Protein kinase, ATP binding site (InterPro:IPR017441), Serine/threonine-protein kinase-like domain (InterPro:IPR017442), Concanavalin A-like lectin/glucanase, subgroup (InterPro:IPR013320), Protein kinase-like domain (InterPro:IPR011009), Serine/threonine-protein kinase, active site (InterPro:IPR008271), Protein kinase, catalytic domain (InterPro:IPR000719), Concanavalin A-like lectin/glucanase (InterPro:IPR008985); BEST Arabidopsis thaliana protein match is: Concanavalin A-like lectin protein kinase family protein (TAIR:AT4G29050.1)
AT5G50360	*3,337*		unknown protein; BEST Arabidopsis thaliana protein match is: unknown protein (TAIR:AT3G48510.1)
AT4G17660	*3,334*		Protein kinase superfamily protein; FUNCTIONS IN: protein serine/threonine kinase activity, protein kinase activity, kinase activity, ATP binding; INVOLVED IN: protein amino acid phosphorylation; LOCATED IN: cellular_component unknown; EXPRESSED IN: 6 plant structures; EXPRESSED DURING: LP.06 six leaves visible, 4 anthesis, C globular stage; CONTAINS InterPro DOMAIN/s: Protein kinase, ATP binding site (InterPro:IPR017441), Protein kinase, catalytic domain (InterPro:IPR000719), Serine/threonine-protein kinase domain (InterPro:IPR002290), Tyrosine-protein kinase, catalytic domain (InterPro:IPR020635), Serine/threonine-protein kinase-like domain (InterPro:IPR017442), Protein kinase-like domain (InterPro:IPR011009), Serine/threonine-protein kinase, active site (InterPro:IPR008271); BEST Arabidopsis thaliana protein match is: Protein kinase superfamily protein (TAIR:AT5G47070.1)
AT4G25010	*3,286*	(SWEET14)	Encodes a member of the SWEET sucrose efflux transporter family proteins.

AT4G02670	*3,278*	INDETERMINATE(ID)-DOMAIN 12 (IDD12)	indeterminate(ID)-domain 12 (IDD12); FUNCTIONS IN: sequence-specific DNA binding transcription factor activity, zinc ion binding, nucleic acid binding; INVOLVED IN: regulation of transcription; LOCATED IN: intracellular, chloroplast; EXPRESSED IN: shoot apex, embryo, flower, pedicel, seed; EXPRESSED DURING: 4 anthesis, C globular stage, petal differentiation and expansion stage, E expanded cotyledon stage, D bilateral stage; CONTAINS InterPro DOMAIN/s: Zinc finger, C2H2-like (InterPro:IPR015880), Zinc finger, C2H2-type (InterPro:IPR007087), Zinc finger, double-stranded RNA binding (InterPro:IPR022755); BEST Arabidopsis thaliana protein match is: C2H2-like zinc finger protein (TAIR:AT5G66730.1)
AT5G06730	*3,11*		Peroxidase superfamily protein; FUNCTIONS IN: peroxidase activity, heme binding; INVOLVED IN: response to oxidative stress, oxidation reduction; LOCATED IN: plasma membrane; EXPRESSED IN: root, cultured cell; CONTAINS InterPro DOMAIN/s: Haem peroxidase (InterPro:IPR010255), Plant peroxidase (InterPro:IPR000823), Peroxidases heam-ligand binding site (InterPro:IPR019793), Haem peroxidase, plant/fungal/bacterial (InterPro:IPR002016), Peroxidase, active site (InterPro:IPR019794); BEST Arabidopsis thaliana protein match is: peroxidase 2 (TAIR:AT5G06720.1)
AT5G24540	*3,103*	BETA GLUCOSIDASE 31 (BGLU31)	beta glucosidase 31 (BGLU31); FUNCTIONS IN: cation binding, hydrolase activity, hydrolyzing O-glycosyl compounds, catalytic activity; INVOLVED IN: response to other organism; LOCATED IN: endomembrane system; EXPRESSED IN: stem, sepal; EXPRESSED DURING: 4 anthesis; CONTAINS InterPro DOMAIN/s: Glycoside hydrolase, family 1 (InterPro:IPR001360), Glycoside hydrolase, family 1, active site (InterPro:IPR018120), Glycoside hydrolase, catalytic core (InterPro:IPR017853), Glycoside hydrolase, subgroup, catalytic core (InterPro:IPR013781); BEST Arabidopsis thaliana protein match is: beta glucosidase 32 (TAIR:AT5G24550.1)
AT5G61890	*3,103*		encodes a member of the ERF (ethylene response factor) subfamily B-4 of ERF/AP2 transcription factor family. The protein contains one AP2 domain. There are 7 members in this subfamily.
AT2G30760	*3,063*		unknown protein
AT2G27690	*3,001*	CYTOCHROME P450, FAMILY 94, SUBFAMILY C, POLYPEPTIDE 1 (CYP94C1)	Encodes a CYP94C1. Has highest omega-hydroxylase activity with 9,10-epoxystearic acid, while also metabolized lauric acid (C12:0) and C18 unsaturated fatty acids. Gene expression is induced in response to wounding and jasmonic acid treatment.
AT1G69920	*2,946*	GLUTATHIONE S-TRANSFERASE TAU 12	Encodes glutathione transferase belonging to the tau class of GSTs. Naming convention according to Wagner et al. (2002).
AT5G01900	*2,918*	WRKY DNA-BINDING PROTEIN 62 (WRKY62)	member of WRKY Transcription Factor; Group III
AT1G12030	*2,771*		Protein of unknown function (DUF506) ; CONTAINS InterPro DOMAIN/s: Protein of unknown function DUF506, plant (InterPro:IPR006502); BEST Arabidopsis thaliana protein match is: Protein of unknown function (DUF506) (TAIR:AT1G62420.1)
AT3G55515	*2,762*	ROTUNDIFOLIA LIKE 7 (RTFL7)	ROTUNDIFOLIA like 7 (RTFL7); CONTAINS InterPro DOMAIN/s: DVL (InterPro:IPR012552); BEST Arabidopsis thaliana protein match is: ROTUNDIFOLIA like 8 (TAIR:AT2G39705.1)
AT3G55970	*2,747*	JASMONATE-REGULATED GENE 21 (JRG21)	jasmonate-regulated gene 21 (JRG21); FUNCTIONS IN: oxidoreductase activity, iron ion binding; INVOLVED IN: oxidation reduction; LOCATED IN: cellular_component unknown; EXPRESSED IN: 10 plant structures; EXPRESSED DURING: LP.06 six leaves visible, LP.04 four leaves visible, 4 anthesis, C globular stage, petal differentiation and expansion stage; CONTAINS InterPro DOMAIN/s: Isopenicillin N synthase (InterPro:IPR002283), Oxoglutarate/iron-dependent oxygenase (InterPro:IPR005123); BEST Arabidopsis thaliana protein match is: 2-oxoglutarate (2OG) and Fe(II)-dependent oxygenase superfamily protein (TAIR:AT5G05600.1)
AT1G17420	*2,738*	LIPOXYGENASE 3 (LOX3)	LOX3 encode a Lipoxygenase. Lipoxygenases (LOXs) catalyze the oxygenation of fatty acids (FAs).
AT2G43520	*2,717*	TRYPSIN INHIBITOR PROTEIN 2 (TI2)	Encodes putative trypsin inhibitor protein which may function in defense against herbivory. Member of the defensin-like (DEFL) family.
AT4G25580	*2,709*		CAP160 protein; CONTAINS InterPro DOMAIN/s: CAP160 (InterPro:IPR012418); BEST Arabidopsis thaliana protein match is: CAP160 protein (TAIR:AT5G52300.1)
AT1G10010	*2,662*	AMINO ACID PERMEASE 8 (AAP8)	Encodes a high affinity amino acid transporter that is probably responsible for import of organic nitrogen into developing seeds. One of eight gene family members that encode amino acid permeases. Most closely related to AAP1 (75%) identity.

AT2G47950	*2,636*		unknown protein; FUNCTIONS IN: molecular_function unknown; INVOLVED IN: biological_process unknown; LOCATED IN: endomembrane system; EXPRESSED IN: root, flower; EXPRESSED DURING: petal differentiation and expansion stage; BEST Arabidopsis thaliana protein match is: unknown protein (TAIR:AT3G62990.1)
AT5G01380	*2,627*		Homeodomain-like superfamily protein; CONTAINS InterPro DOMAIN/s: SANT, DNA-binding (InterPro:IPR001005), MYB-like (InterPro:IPR017877); BEST Arabidopsis thaliana protein match is: Homeodomain-like superfamily protein (TAIR:AT2G38250.1)
AT4G13480	*2,587*	MYB DOMAIN PROTEIN 79 (MYB79)	Member of the R2R3 factor gene family.
AT4G37710	*2,584*	VQ MOTIF-CONTAINING PROTEIN 29 (VQ29)	VQ motif-containing protein; FUNCTIONS IN: molecular_function unknown; INVOLVED IN: biological_process unknown; LOCATED IN: chloroplast; EXPRESSED IN: pollen tube; CONTAINS InterPro DOMAIN/s: VQ (InterPro:IPR008889); BEST Arabidopsis thaliana protein match is: VQ motif-containing protein (TAIR:AT2G22880.1)
AT1G62420	*2,573*		Protein of unknown function (DUF506) ; CONTAINS InterPro DOMAIN/s: Protein of unknown function DUF506, plant (InterPro:IPR006502); BEST Arabidopsis thaliana protein match is: Protein of unknown function (DUF506) (TAIR:AT1G12030.1)
AT1G06030	*2,567*		pfkB-like carbohydrate kinase family protein; FUNCTIONS IN: kinase activity, ribokinase activity; INVOLVED IN: D-ribose metabolic process, acetate fermentation, sucrose biosynthetic process, sucrose catabolic process, using beta-fructofuranosidase; EXPRESSED IN: guard cell; CONTAINS InterPro DOMAIN/s: Carbohydrate/purine kinase (InterPro:IPR011611), Ribokinase (InterPro:IPR002139), Carbohydrate/puine kinase, PfkB, conserved site (InterPro:IPR002173); BEST Arabidopsis thaliana protein match is: pfkB-like carbohydrate kinase family protein (TAIR:AT1G06020.1)
AT4G12290	*2,561*		Copper amine oxidase family protein; FUNCTIONS IN: quinone binding, primary amine oxidase activity, copper ion binding; INVOLVED IN: oxidation reduction, amine metabolic process; LOCATED IN: vacuole; CONTAINS InterPro DOMAIN/s: Copper amine oxidase, N-terminal (InterPro:IPR016182), Copper amine oxidase, N2-terminal (InterPro:IPR015800), Copper amine oxidase, N2/N3-terminal (InterPro:IPR015801), Copper amine oxidase, N3-terminal (InterPro:IPR015802), Copper amine oxidase (InterPro:IPR000269), Copper amine oxidase, C-terminal (InterPro:IPR015798); BEST Arabidopsis thaliana protein match is: Copper amine oxidase family protein (TAIR:AT1G62810.1)
AT1G14520	*2,536*	MYO-INOSITOL OXYGENASE 1 (MIOX1)	Encodes MIOX1. Belongs to myo-inositol oxygenase gene family.
AT5G39180	*2,534*		RmlC-like cupins superfamily protein; FUNCTIONS IN: manganese ion binding, nutrient reservoir activity; INVOLVED IN: biological_process unknown; LOCATED IN: endomembrane system, apoplast; CONTAINS InterPro DOMAIN/s: Cupin, RmlC-type (InterPro:IPR011051), Cupin 1 (InterPro:IPR006045), RmlC-like jelly roll fold (InterPro:IPR014710), Germin (InterPro:IPR001929), Germin, manganese binding site (InterPro:IPR019780); BEST Arabidopsis thaliana protein match is: RmlC-like cupins superfamily protein (TAIR:AT5G39150.1)
AT3G23120	*2,509*	RECEPTOR LIKE PROTEIN 38 (RLP38)	receptor like protein 38 (RLP38); FUNCTIONS IN: kinase activity; INVOLVED IN: signal transduction, defense response; LOCATED IN: endomembrane system; CONTAINS InterPro DOMAIN/s: Leucine-rich repeat-containing N-terminal domain, type 2 (InterPro:IPR013210), Leucine-rich repeat (InterPro:IPR001611); BEST Arabidopsis thaliana protein match is: receptor like protein 37 (TAIR:AT3G23110.1)
AT5G52050	*2,478*	DETOXIFICATION EFFLUX CARRIER 50 (DTX50)	MATE efflux family protein; FUNCTIONS IN: antiporter activity, drug transmembrane transporter activity; INVOLVED IN: drug transmembrane transport, transmembrane transport; LOCATED IN: membrane; EXPRESSED IN: 7 plant structures; EXPRESSED DURING: 7 growth stages; CONTAINS InterPro DOMAIN/s: Multi antimicrobial extrusion protein MatE (InterPro:IPR002528); BEST Arabidopsis thaliana protein match is: MATE efflux family protein (TAIR:AT4G23030.1)
AT5G13220	*2,455*	JASMONATE-ZIM-DOMAIN PROTEIN 10 (JAZ10)	Plants overexpressing At5g13220.3, but not At5g13220.1 showed enhanced insensitivity to MeJa.
AT5G64870	*2,444*		SPFH/Band 7/PHB domain-containing membrane-associated protein family; FUNCTIONS IN: molecular_function unknown; INVOLVED IN: biological_process unknown; LOCATED IN: plasma membrane, vacuole; EXPRESSED IN: 18 plant structures; EXPRESSED DURING: 11 growth stages; CONTAINS InterPro DOMAIN/s: Band 7 protein (InterPro:IPR001107); BEST Arabidopsis thaliana protein match is: SPFH/Band 7/PHB domain-containing membrane-associated protein family (TAIR:AT5G25250.1)

AT4G17600	*2,435*	LIGHT-HARVESTING-LIKE 3:1 (LIL3:1)	Encodes Lil3:1 (light-harvesting-like) protein. Belongs to the Lhc super-gene family encodes the light-harvesting chlorophyll a/b-binding (LHC) proteins that constitute the antenna system of the photosynthetic apparatus. A generic LHC motif is present in Lil3:1. The mRNA is cell-to-cell mobile.
AT5G05340	*2,433*	PEROXIDASE 52 (PRX52)	Encodes a protein with sequence similarity to peroxidases that is involved in lignin biosynthesis. Loss of function mutations show abnormal development of xylem fibers and reduced levels of lignin biosynthetic enxymes.
AT2G37770	*2,422*	CHLOROPLASTIC ALDO-KETO REDUCTASE (ChlAKR)	Encodes an NADPH-dependent aldo-keto reductase that can act on a wide variety of substrates in vitro including saturated and unsaturated aldehydes, steroids, and sugars. GFP-tagged AKR4C9 localizes to the chloroplast where it may play a role in detoxifying reactive carbonyl compounds that threaten to impair the photosynthetic process. Transcript levels for this gene are up-regulated in response to cold, salt, and drought stress.
AT3G56970	*2,42*	BASIC HELIX-LOOP-HELIX 38 (BHLH38)	Encodes a member of the basic helix-loop-helix transcription factor family protein.
AT5G59390	*2,408*		XH/XS domain-containing protein; CONTAINS InterPro DOMAIN/s: Domain of unknown function XS (InterPro:IPR005380), Domain of unknown function XH (InterPro:IPR005379); BEST Arabidopsis thaliana protein match is: XH/XS domain-containing protein (TAIR:AT4G01180.1)
AT5G20230	*2,404*	BLUE-COPPER-BINDING PROTEIN (BCB)	Encodes a Al-stress-induced gene. Along with TCF, it promotes lignin biosynthesis in response to cold stress. The mRNA is cell-to-cell mobile.
AT5G57550	*2,399*	XYLOGLUCAN ENDOTRANSGLUCOSYLASE/HYDROLASE 25 (XTH25)	xyloglucan endotransglycosylase-related protein (XTR3)
AT1G75040	*2,394*	PATHOGENESIS-RELATED GENE 5 (PR5)	Thaumatin-like protein involved in response to pathogens. mRNA level of the PR-5 gene (At1g75040)is significantly changed after cutting the inflorescence stem indicating the existence of a network of signal transducing pathways as other stress-regulated genes (At5g01410, At3g17800, At1g29930)do not response to the treatment. The mRNA is cell-to-cell mobile.
AT2G43530	*2,387*		Encodes a defensin-like (DEFL) family protein. The mRNA is cell-to-cell mobile.
AT2G36970	*2,383*		UDP-Glycosyltransferase superfamily protein; FUNCTIONS IN: UDP-glycosyltransferase activity, transferase activity, transferring glycosyl groups; INVOLVED IN: metabolic process; LOCATED IN: endomembrane system; EXPRESSED IN: 20 plant structures; EXPRESSED DURING: 13 growth stages; CONTAINS InterPro DOMAIN/s: UDP-glucuronosyl/UDP-glucosyltransferase (InterPro:IPR002213); BEST Arabidopsis thaliana protein match is: UDP-Glycosyltransferase superfamily protein (TAIR:AT2G28080.1)
AT4G35480	*2,377*	RING-H2 FINGER A3B (RHA3B)	Encodes a putative RING-H2 finger protein RHA3b.
AT4G18170	*2,373*	WRKY DNA-BINDING PROTEIN 28 (WRKY28)	member of WRKY Transcription Factor; Group II-c. Involved in the activation of salicylic acid biosynthesis genes ICS1 and PBS3.
AT5G52720	*2,37*		Copper transport protein family; BEST Arabidopsis thaliana protein match is: Copper transport protein family (TAIR:AT5G52670.1)
AT1G56250	*2,365*	PHLOEM PROTEIN 2-B14 (PP2-B14)	Encodes an F-box protein that can functionally replace VirF, regulating levels of the VirE2 and VIP1 proteins via a VBF-containing SCF complex. It is thought to be involved in DNA integration and T-DNA degradation.
AT2G45570	*2,36*	CYTOCHROME P450, FAMILY 76, SUBFAMILY C, POLYPEPTIDE 2 (CYP76C2)	member of CYP76C
AT5G24600	*2,353*		Protein of unknown function, DUF599; INVOLVED IN: biological_process unknown; LOCATED IN: plasma membrane; EXPRESSED IN: 15 plant structures; EXPRESSED DURING: 6 growth stages; CONTAINS InterPro DOMAIN/s: Protein of unknown function DUF599 (InterPro:IPR006747); BEST Arabidopsis thaliana protein match is: Protein of unknown function, DUF599 (TAIR:AT3G18215.1)

AT2G21510	*2,352*		DNAJ heat shock N-terminal domain-containing protein; FUNCTIONS IN: unfolded protein binding, heat shock protein binding; INVOLVED IN: protein folding; LOCATED IN: cellular_component unknown; EXPRESSED IN: 8 plant structures; EXPRESSED DURING: 4 anthesis, petal differentiation and expansion stage; CONTAINS InterPro DOMAIN/s: Molecular chaperone, heat shock protein, Hsp40, DnaJ (InterPro:IPR015609), Heat shock protein DnaJ, N-terminal (InterPro:IPR001623), Heat shock protein DnaJ (InterPro:IPR003095), Heat shock protein DnaJ, conserved site (InterPro:IPR018253); BEST Arabidopsis thaliana protein match is: DNAJ heat shock N-terminal domain-containing protein (TAIR:AT4G39150.2)
AT1G12940	*2,351*	NITRATE TRANSPORTER2.5 (NRT2.5)	member of High affinity nitrate transporter family
AT1G33770	*2,343*		Protein kinase superfamily protein; FUNCTIONS IN: protein serine/threonine kinase activity, protein kinase activity, kinase activity, ATP binding; INVOLVED IN: protein amino acid phosphorylation, N-terminal protein myristoylation; LOCATED IN: cellular_component unknown; EXPRESSED IN: 7 plant structures; EXPRESSED DURING: 6 growth stages; CONTAINS InterPro DOMAIN/s: Protein kinase, ATP binding site (InterPro:IPR017441), Protein kinase, catalytic domain (InterPro:IPR000719), Serine/threonine-protein kinase domain (InterPro:IPR002290), Serine/threonine-protein kinase-like domain (InterPro:IPR017442), Protein kinase-like domain (InterPro:IPR011009), Serine/threonine-protein kinase, active site (InterPro:IPR008271); BEST Arabidopsis thaliana protein match is: Protein kinase superfamily protein (TAIR:AT4G10010.1)
AT4G23220	*2,34*	CYSTEINE-RICH RLK (RECEPTOR-LIKE PROTEIN KINASE) 14 (CRK14)	Encodes a cysteine-rich receptor-like protein kinase.
AT2G44460	*2,333*	BETA GLUCOSIDASE 28 (BGLU28)	beta glucosidase 28 (BGLU28); FUNCTIONS IN: cation binding, hydrolase activity, hydrolyzing O-glycosyl compounds, catalytic activity; INVOLVED IN: response to karrikin; LOCATED IN: membrane; EXPRESSED IN: 10 plant structures; EXPRESSED DURING: 7 growth stages; CONTAINS InterPro DOMAIN/s: Glycoside hydrolase, family 1 (InterPro:IPR001360), Glycoside hydrolase, family 1, active site (InterPro:IPR018120), Glycoside hydrolase, catalytic core (InterPro:IPR017853), Glycoside hydrolase, subgroup, catalytic core (InterPro:IPR013781); BEST Arabidopsis thaliana protein match is: beta glucosidase 29 (TAIR:AT2G44470.3)
AT4G38400	*2,33*	EXPANSIN-LIKE A2 (EXLA2)	member of EXPANSIN-LIKE. Naming convention from the Expansin Working Group (Kende et al, 2004. Plant Mol Bio)
AT4G18280	*2,322*		glycine-rich cell wall protein-related
AT3G04000	*2,32*		ChlADR is an aldehyde reductase that catalyzes the reduction of the aldehyde carbonyl groups on saturated and alpha,beta-unsaturated aldehydes with more than 5 carbons in vitro. The N-terminal region of this protein directs GFP to the chloroplast where where ChlADR likely helps to maintain the photosynthetic process by detoxifying reactive carbonyls formed during lipid peroxidation. In addition, this enzyme can also reduce cis-3-hexenal, a major plant volatile compound that contributes to green leaf odor, as well as methylglyoxal in vitro.
AT5G06720	*2,319*	PEROXIDASE 2 (PA2)	Encodes a peroxidase with diverse roles in the wound response, flower development, and syncytium formation.
AT1G31290	*2,317*	ARGONAUTE 3 (AGO3)	ARGONAUTE 3 (AGO3); FUNCTIONS IN: nucleic acid binding; INVOLVED IN: biological_process unknown; LOCATED IN: cellular_component unknown; EXPRESSED IN: 11 plant structures; EXPRESSED DURING: LP.06 six leaves visible, LP.04 four leaves visible, 4 anthesis, C globular stage, petal differentiation and expansion stage; CONTAINS InterPro DOMAIN/s: Domain of unknown function DUF1785 (InterPro:IPR014811), Stem cell self-renewal protein Piwi (InterPro:IPR003165), Argonaute/Dicer protein, PAZ (InterPro:IPR003100), Polynucleotidyl transferase, ribonuclease H fold (InterPro:IPR012337); BEST Arabidopsis thaliana protein match is: Argonaute family protein (TAIR:AT1G31280.1)
AT1G56510	*2,307*	WHITE RUST RESISTANCE 4 (WRR4)	TIR-NB-LRR protein that confers resistance to four races of Albugo candida. The mRNA is cell-to-cell mobile.

AT2G34650	*2,302*	PINOID (PID)	Encodes a protein serine/threonine kinase that may act as a positive regulator of cellular auxin efflux, as a a binary switch for PIN polarity, and as a negative regulator of auxin signaling. Recessive mutants exhibit similar phenotypes as pin-formed mutants in flowers and inflorescence but distinct phenotypes in cotyledons and leaves. Expressed in the vascular tissue proximal to root and shoot meristems, shoot apex, and embryos. Expression is induced by auxin. Overexpression of the gene results in phenotypes in the root and shoot similar to those found in auxin-insensitive mutants. The protein physically interacts with TCH3 (TOUCH3) and PID-BINDING PROTEIN 1 (PBP1), a previously uncharacterized protein containing putative EF-hand calcium-binding motifs. Acts together with ENP (ENHANCER OF PINOID) to instruct precursor cells to elaborate cotyledons in the transition stage embryo. Interacts with PDK1. PID autophosphorylation is required for the ability of PID to phosphorylate an exogenous substrate. PID activation loop is required for PDK1-dependent PID phosphorylation and requires the PIF domain. Negative regulator of root hair growth. PID kinase activity is critical for the inhibition of root hair growth and for maintaining the proper subcellular localization of PID.
AT2G18660	*2,298*	PLANT NATRIURETIC PEPTIDE A (PNP-A)	Encodes PNP-A (Plant Natriuretic Peptide A). PNPs are a class of systemically mobile molecules distantly related to expansins; their biological role has remained elusive. PNP-A contains a signal peptide domain and is secreted into the extracellular space. Co-expression analyses using microarray data suggest that PNP-A may function as a component of plant defence response and SAR in particular, and could be classified as a newly identified PR protein. It is stress responsive and can enhance its own expression.
AT2G35820	*2,295*		ureidoglycolate hydrolases; FUNCTIONS IN: ureidoglycolate hydrolase activity; INVOLVED IN: allantoin catabolic process; LOCATED IN: cellular_component unknown; EXPRESSED IN: 17 plant structures; EXPRESSED DURING: 13 growth stages; CONTAINS InterPro DOMAIN/s: Ureidoglycolate hydrolase (InterPro:IPR007247); BEST Arabidopsis thaliana protein match is: unknown protein (TAIR:AT2G35810.1)
AT1G03445	*2,285*	BRI1 SUPPRESSOR 1 (BSU1)	encodes a serine?threonine protein phosphatase with an N-terminal Kelch-repeat domain, which is nuclear localized and expressed preferentially in elongating cells. Genetic evidence suggest that this gene plays a redundant role (along with other members of the same gene family) in modulating growth in response to brassinosteroid.
AT3G19270	*2,285*	CYTOCHROME P450, FAMILY 707, SUBFAMILY A, POLYPEPTIDE 4 (CYP707A4)	Encodes a protein with ABA 8'-hydroxylase activity, involved in ABA catabolism. Member of the CYP707A gene family.
AT2G21130	*2,283*		Cyclophilin-like peptidyl-prolyl cis-trans isomerase family protein; FUNCTIONS IN: peptidyl-prolyl cis-trans isomerase activity; INVOLVED IN: protein folding; LOCATED IN: plasma membrane; EXPRESSED IN: 26 plant structures; EXPRESSED DURING: 15 growth stages; CONTAINS InterPro DOMAIN/s: Cyclophilin-like (InterPro:IPR015891), Peptidyl-prolyl cis-trans isomerase, cyclophilin-type (InterPro:IPR002130), Peptidyl-prolyl cis-trans isomerase, cyclophilin-type, conserved site (InterPro:IPR020892); BEST Arabidopsis thaliana protein match is: rotamase CYP 1 (TAIR:AT4G38740.1)
AT1G32960	*2,27*	(SBT3.3)	SBT3.3; FUNCTIONS IN: identical protein binding, serine-type endopeptidase activity; INVOLVED IN: proteolysis, negative regulation of catalytic activity; LOCATED IN: apoplast, plant-type cell wall; EXPRESSED IN: 7 plant structures; EXPRESSED DURING: 4 anthesis; CONTAINS InterPro DOMAIN/s: Protease-associated PA (InterPro:IPR003137), Proteinase inhibitor, propeptide (InterPro:IPR009020), Peptidase S8/S53, subtilisin/kexin/sedolisin (InterPro:IPR000209), Peptidase S8, subtilisin-related (InterPro:IPR015500), Proteinase inhibitor I9, subtilisin propeptide (InterPro:IPR010259), Peptidase S8/S53, subtilisin, active site (InterPro:IPR022398); BEST Arabidopsis thaliana protein match is: Subtilase family protein (TAIR:AT1G32950.1)
AT5G20740	*2,24*		Plant invertase/pectin methylesterase inhibitor superfamily protein; FUNCTIONS IN: enzyme inhibitor activity, pectinesterase inhibitor activity, pectinesterase activity; INVOLVED IN: biological_process unknown; LOCATED IN: endomembrane system; EXPRESSED IN: 13 plant structures; EXPRESSED DURING: 7 growth stages; CONTAINS InterPro DOMAIN/s: Pectinesterase inhibitor (InterPro:IPR006501); BEST Arabidopsis thaliana protein match is: Plant invertase/pectin methylesterase inhibitor superfamily protein (TAIR:AT1G62770.1)
AT2G44070	*2,236*		NagB/RpiA/CoA transferase-like superfamily protein; FUNCTIONS IN: GTP binding, translation initiation factor activity; INVOLVED IN: translational initiation, cellular metabolic process; LOCATED IN: eukaryotic translation initiation factor 2B complex; CONTAINS InterPro DOMAIN/s: Initiation factor 2B related (InterPro:IPR000649); BEST Arabidopsis thaliana protein match is: NagB/RpiA/CoA transferase-like superfamily protein (TAIR:AT5G38640.1)

AT4G37770	*2,219*	1-AMINO-CYCLOPROPANE-1-CARBOXYLATE SYNTHASE 8 (ACS8)	Encodes an auxin inducible ACC synthase.
AT4G22753	*2,216*	STEROL 4-ALPHA METHYL OXIDASE 1-3 (SMO1-3)	Encodes a member of the SMO1 family of sterol 4alpha-methyl oxidases.
AT4G25020	*2,205*		D111/G-patch domain-containing protein; FUNCTIONS IN: nucleic acid binding; INVOLVED IN: biological_process unknown; LOCATED IN: intracellular; EXPRESSED IN: 16 plant structures; EXPRESSED DURING: 8 growth stages; CONTAINS InterPro DOMAIN/s: D111/G-patch (InterPro:IPR000467), KOW (InterPro:IPR005824); BEST Arabidopsis thaliana protein match is: D111/G-patch domain-containing protein (TAIR:AT1G33520.1)
AT2G30770	*2,193*	CYTOCHROME P450, FAMILY 71, SUBFAMILY A, POLYPEPTIDE 13 (CYP71A13)	putative cytochrome P450
AT5G66110	*2,19*	HEAVY METAL ASSOCIATED ISOPRENYLATED PLANT PROTEIN 27 (HIPP27)	Heavy metal transport/detoxification superfamily protein ; FUNCTIONS IN: metal ion binding; INVOLVED IN: metal ion transport; LOCATED IN: plasma membrane; EXPRESSED IN: cultured cell, leaf; EXPRESSED DURING: LP.04 four leaves visible; CONTAINS InterPro DOMAIN/s: Heavy metal transport/detoxification protein (InterPro:IPR006121); BEST Arabidopsis thaliana protein match is: farnesylated protein 6 (TAIR:AT4G38580.1)
AT1G71770	*2,174*	POLY(A)-BINDING PROTEIN 5 (PAB5)	Encodes a Class I polyA-binding protein. Expressed in floral organs. Binds polyA sepharose in vitro.
AT2G17780	*2,172*	MID1-COMPLEMENTING ACTIVITY 2 (MCA2)	Encodes a mechanosensitive channel candidate MCA2. The three-dimensional structure of MCA2 was reconstructed and appears to comprise a small transmembrane region and large cytoplasmic region.
AT3G24650	*2,163*	ABA INSENSITIVE 3 (ABI3)	Homologous to the maize transcription factor Viviparous-1. Full length ABI3 protein binds to the highly conserved RY motif [DNA motif CATGCA(TG)], present in many seed-specific promoters, and the B3 domains of this transcription factor is necessary for the specific interaction with the RY element. Transcriptional activity of ABI3 requires the B3 DNA-binding domain and an activation domain. In addition to the known N-terminal-located activation domain, a second transcription activation domain was found in the B1 region of ABI3. ABI3 is essential for seed maturation. Regulator of the transition between embryo maturation and early seedling development. Putative seed-specific transcriptional activator. ABI3 is a central regulator in ABA signaling and is unstable in vivo. It interacts with and can by polyubiquitinated by AIP2 in vivo. Based on double mutant analyses, ABI3 interacts genetically with both FUS3 and LEC1 and is involved in controlling accumulation of chlorophyll and anthocyanins, sensitivity to abscisic acid, and expression of the members of the 12S storage protein gene family. In addition, both FUS3 and LEC1 regulate positively the abundance of the ABI3 protein in the seed. Alternative splicing of ABI3 is developmentally regulated by SUA (AT3G54230).
AT2G42540	*2,16*	COLD-REGULATED 15A (COR15A)	A cold-regulated gene whose product is targeted to the chloroplast. Cor15am protects stromal proteins from aggregation under various stress conditions. Constitutive expression increases freezing tolerance in protoplasts in vitro and chloroplasts in vivo. NMR and x-ray diffraction studies suggest that COR15a alters the intrinsic curvature of the inner membrane of chloroplast envelope. Late Embryogenesis abundant protein (LEA). Protects chloroplast membranes during freezing.
AT5G39110	*2,16*		RmlC-like cupins superfamily protein; FUNCTIONS IN: manganese ion binding, nutrient reservoir activity; INVOLVED IN: biological_process unknown; LOCATED IN: endomembrane system, apoplast; CONTAINS InterPro DOMAIN/s: Cupin, RmlC-type (InterPro:IPR011051), Cupin 1 (InterPro:IPR006045), RmlC-like jelly roll fold (InterPro:IPR014710), Germin (InterPro:IPR001929), Germin, manganese binding site (InterPro:IPR019780); BEST Arabidopsis thaliana protein match is: RmlC-like cupins superfamily protein (TAIR:AT5G39150.1)
AT1G66490	*2,158*		F-box and associated interaction domains-containing protein; CONTAINS InterPro DOMAIN/s: F-box domain, cyclin-like (InterPro:IPR001810), F-box domain, Skp2-like (InterPro:IPR022364), F-box associated domain, type 1 (InterPro:IPR006527), F-box associated interaction domain (InterPro:IPR017451); BEST Arabidopsis thaliana protein match is: F-box and associated interaction domains-containing protein (TAIR:AT3G13680.1)
AT1G47400	*2,147*		unknown protein; BEST Arabidopsis thaliana protein match is: unknown protein (TAIR:AT1G47395.1)

AT4G22710	*2,147*	CYTOCHROME P450, FAMILY 706, SUBFAMILY A, POLYPEPTIDE 2 (CYP706A2)	member of CYP706A
AT5G40000	*2,139*		P-loop containing nucleoside triphosphate hydrolases superfamily protein; FUNCTIONS IN: nucleoside-triphosphatase activity, ATPase activity, nucleotide binding, ATP binding; INVOLVED IN: biological_process unknown; LOCATED IN: endomembrane system; CONTAINS InterPro DOMAIN/s: ATPase, AAA+ type, core (InterPro:IPR003593), ATPase, AAA-type, core (InterPro:IPR003959), ATPase, AAA-type, conserved site (InterPro:IPR003960); BEST Arabidopsis thaliana protein match is: P-loop containing nucleoside triphosphate hydrolases superfamily protein (TAIR:AT3G28600.1)
AT1G56240	*2,135*	PHLOEM PROTEIN 2-B13 (PP2-B13)	phloem protein 2-B13 (PP2-B13); FUNCTIONS IN: carbohydrate binding; LOCATED IN: endomembrane system; CONTAINS InterPro DOMAIN/s: F-box domain, cyclin-like (InterPro:IPR001810), F-box domain, Skp2-like (InterPro:IPR022364); BEST Arabidopsis thaliana protein match is: phloem protein 2-B14 (TAIR:AT1G56250.1)
AT5G56510	*2,131*	PUMILIO 12 (PUM12)	Encodes a member of the Arabidopsis Pumilio (APUM) proteins containing PUF domain (eight repeats of approximately 36 amino acids each). PUF proteins regulate both mRNA stability and translation through sequence-specific binding to the 3' UTR of target mRNA transcripts.
AT2G05520	*2,128*	GLYCINE-RICH PROTEIN 3 (GRP-3)	Encodes a glycine-rich protein that is expressed mainly in stems and leaves. AtGRP3 functions in root size determination during development and in Al stress. mRNA levels are upregulated in response to ABA, salicylic acid and ethylene but downregulated in response to desiccation. The mRNA is cell-to-cell mobile.
AT2G43360	*2,128*	BIOTIN AUXOTROPH 2 (BIO2)	Catalyzes the conversion of dethiobiotin to biotin.
AT2G40610	*2,119*	EXPANSIN A8 (EXPA8)	member of Alpha-Expansin Gene Family. Naming convention from the Expansin Working Group (Kende et al, 2004. Plant Mol Bio). Involved in the formation of nematode-induced syncytia in roots of Arabidopsis thaliana.
AT1G29395	*2,108*	COLD REGULATED 314 INNER MEMBRANE 1 (COR413IM1)	Integral membrane protein in the inner envelope of chloroplasts. Provide freezing tolerance. Expression is induced by short-term cold-treatment, water deprivation, and abscisic acid treatment.
AT1G62290	*2,105*	PUTATIVE ASPARTIC PROTEINASE A2 (PASPA2)	Saposin-like aspartyl protease family protein; FUNCTIONS IN: aspartic-type endopeptidase activity; INVOLVED IN: proteolysis, lipid metabolic process; LOCATED IN: vacuole; EXPRESSED IN: 10 plant structures; EXPRESSED DURING: LP.06 six leaves visible, LP.04 four leaves visible, seedling growth, petal differentiation and expansion stage, LP.08 eight leaves visible; CONTAINS InterPro DOMAIN/s: Saposin-like (InterPro:IPR011001), Peptidase aspartic (InterPro:IPR021109), Peptidase aspartic, catalytic (InterPro:IPR009007), Saposin-like type B, 1 (InterPro:IPR007856), Saposin-like type B, 2 (InterPro:IPR008138), Saposin B (InterPro:IPR008139), Peptidase A1 (InterPro:IPR001461), Peptidase aspartic, active site (InterPro:IPR001969); BEST Arabidopsis thaliana protein match is: aspartic proteinase A1 (TAIR:AT1G11910.1)
AT2G32830	*2,103*	PHOSPHATE TRANSPORTER 1;5 (PHT1;5)	Encodes Pht1;5, a member of the Pht1 family of phosphate transporters which include: Pht1;1/At5g43350, Pht1;2/At5g43370, Pht1;3/At5g43360, Pht1;4/At2g38940, Pht1;5/At2g32830, Pht1;6/At5g43340, Pht1;7/At3g54700, Pht1;8/At1g20860, Pht1;9/At1g76430 (Plant Journal 2002, 31:341).
AT5G06870	*2,1*	POLYGALACTURONASE INHIBITING PROTEIN 2 (PGIP2)	Encodes a polygalacturonase inhibiting protein involved in plant defense response. PGIPs inhibit the activity of pectin degrading enzymes such as those produced by fungal pathogens. PGIP2 is induced by fungal infection and methyl jasmonate.Suppressed in the proton sensitive stop1-mutant, but the transcription level was recovered by transformation of STOP2. Knockout mutant showed severe damage in the root tip in low Ca and low pH medium.
AT5G39150	*2,098*		RmlC-like cupins superfamily protein; FUNCTIONS IN: manganese ion binding, nutrient reservoir activity; INVOLVED IN: biological_process unknown; LOCATED IN: endomembrane system, apoplast; CONTAINS InterPro DOMAIN/s: Cupin, RmlC-type (InterPro:IPR011051), Cupin 1 (InterPro:IPR006045), RmlC-like jelly roll fold (InterPro:IPR014710), Germin (InterPro:IPR001929), Germin, manganese binding site (InterPro:IPR019780); BEST Arabidopsis thaliana protein match is: RmlC-like cupins superfamily protein (TAIR:AT5G39180.1)
AT1G30040	*2,086*	GIBBERELLIN 2-OXIDASE (GA2OX2)	Encodes a gibberellin 2-oxidase that acts on C-19 gibberellins. AtGA2OX2 expression is responsive to cytokinin and KNOX activities.

AT5G62520	*2,08*	SIMILAR TO RCD ONE 5 (SRO5)	Encodes a protein with similarity to RCD1 but without the WWE domain. The protein does have a PARP signature upstream of the C-terminal protein interaction domain. The PARP signature may bind NAD+ and attach the ADP-ribose-moiety from NAD+ to the target molecule. Its presence suggests a role for the protein in ADP ribosylation. Up-regulated by NaCl. SRO5 and P5CDH (an overlapping gene in the antisense orientation) generate 24-nt and 21-nt siRNAs, which together are components of a regulatory loop controlling reactive oxygen species (ROS) production and stress response.
AT5G43870	*2,078*		FUNCTIONS IN: phosphoinositide binding; INVOLVED IN: signal transduction; LOCATED IN: plasma membrane; EXPRESSED IN: 21 plant structures; EXPRESSED DURING: 13 growth stages; CONTAINS InterPro DOMAIN/s: Pleckstrin-like, plant (InterPro:IPR013666), Protein of unknown function DUF828 (InterPro:IPR008546); BEST Arabidopsis thaliana protein match is: Plant protein of unknown function (DUF828) with plant pleckstrin homology-like region (TAIR:AT3G22810.1)
AT1G60970	*2,065*		SNARE-like superfamily protein; INVOLVED IN: intracellular protein transport, transport; LOCATED IN: clathrin vesicle coat; EXPRESSED IN: leaf whorl, sperm cell, pedicel; EXPRESSED DURING: 4 anthesis; CONTAINS InterPro DOMAIN/s: Longin-like (InterPro:IPR011012); BEST Arabidopsis thaliana protein match is: SNARE-like superfamily protein (TAIR:AT3G09800.1)
AT4G39650	*2,062*	GAMMA-GLUTAMYL TRANSPEPTIDASE 2 (GGT2)	The gene encodes a gamma-glutamyltransferase (AKA gamma-glutamyl transpeptidase, EC 2.3.2.2) that is located in the apoplast of young siliques (within the ovules of the carpel) and is involved in the degradation of glutathione. The encoded enzyme also acts as part of a GSH pumping gamma-glutamyl cycle in this tissue and may also be involved in gamma-glutamyl amino acid formation.
AT1G07900	*2,06*	LOB DOMAIN-CONTAINING PROTEIN 1 (LBD1)	LOB domain-containing protein 1 (LBD1); CONTAINS InterPro DOMAIN/s: Lateral organ boundaries, LOB (InterPro:IPR004883); BEST Arabidopsis thaliana protein match is: LOB domain-containing protein 11 (TAIR:AT2G28500.1)
AT2G43510	*2,056*	TRYPSIN INHIBITOR PROTEIN 1 (TI1)	Member of the defensin-like (DEFL) family. Encodes putative trypsin inhibitor protein which may function in defense against herbivory.
AT1G61550	*2,054*		S-locus lectin protein kinase family protein; FUNCTIONS IN: in 6 functions; INVOLVED IN: protein amino acid phosphorylation, recognition of pollen; LOCATED IN: endomembrane system; EXPRESSED IN: sperm cell, root; CONTAINS InterPro DOMAIN/s: Curculin-like (mannose-binding) lectin (InterPro:IPR001480), PAN-2 domain (InterPro:IPR013227), Apple-like (InterPro:IPR003609), S-locus receptor kinase, C-terminal (InterPro:IPR021820), EGF-like, type 3 (InterPro:IPR000742), Serine-threonine/tyrosine-protein kinase (InterPro:IPR001245), Protein kinase-like domain (InterPro:IPR011009), Serine/threonine-protein kinase, active site (InterPro:IPR008271), Protein kinase, catalytic domain (InterPro:IPR000719), S-locus glycoprotein (InterPro:IPR000858); BEST Arabidopsis thaliana protein match is: S-locus lectin protein kinase family protein (TAIR:AT1G61490.1)
AT5G25970	*2,045*		Core-2/I-branching beta-1,6-N-acetylglucosaminyltransferase family protein; CONTAINS InterPro DOMAIN/s: Core-2/I-Branching enzyme (InterPro:IPR021141); BEST Arabidopsis thaliana protein match is: Core-2/I-branching beta-1,6-N-acetylglucosaminyltransferase family protein (TAIR:AT5G11730.1)
AT5G42680	*2,039*		Protein of unknown function, DUF617; CONTAINS InterPro DOMAIN/s: Protein of unknown function DUF617, plant (InterPro:IPR006460); BEST Arabidopsis thaliana protein match is: Protein of unknown function, DUF617 (TAIR:AT4G39610.1)
AT5G66780	*2,032*		unknown protein
AT2G40100	*2,031*	LIGHT HARVESTING COMPLEX PHOTOSYSTEM II (LHCB4.3)	Lhcb4:3 protein (Lhcb4.3, light harvesting complex of photosystem II The mRNA is cell-to-cell mobile.
AT1G71380	*2,029*	CELLULASE 3 (CEL3)	cellulase 3 (CEL3); FUNCTIONS IN: hydrolase activity, hydrolyzing O-glycosyl compounds, catalytic activity; INVOLVED IN: carbohydrate metabolic process; LOCATED IN: cell wall, plasma membrane, plant-type cell wall; EXPRESSED IN: 7 plant structures; EXPRESSED DURING: L mature pollen stage, M germinated pollen stage, 4 anthesis, petal differentiation and expansion stage; CONTAINS InterPro DOMAIN/s: Six-hairpin glycosidase (InterPro:IPR012341), Six-hairpin glycosidase-like (InterPro:IPR008928), Glycoside hydrolase, family 9, active site (InterPro:IPR018221), Glycoside hydrolase, family 9 (InterPro:IPR001701); BEST Arabidopsis thaliana protein match is: cellulase 5 (TAIR:AT1G22880.1)
AT1G79180	*2,023*	MYB DOMAIN PROTEIN 63 (MYB63)	Member of the R2R3 factor gene family.

AT1G71890	*2,022*	(SUC5)	Encodes a sucrose transporter that is expressed in the endosperm. Mutants have delayed accumulation of fatty acids and embryo maturation.
AT2G17280	*2,022*		Phosphoglycerate mutase family protein; FUNCTIONS IN: catalytic activity; INVOLVED IN: metabolic process; LOCATED IN: cellular_component unknown; EXPRESSED IN: 24 plant structures; EXPRESSED DURING: 13 growth stages; CONTAINS InterPro DOMAIN/s: Histidine phosphatase superfamily, clade-1 (InterPro:IPR013078); BEST Arabidopsis thaliana protein match is: Phosphoglycerate mutase family protein (TAIR:AT5G64460.8)
AT4G36430	*2,018*		Peroxidase superfamily protein; FUNCTIONS IN: peroxidase activity, heme binding; INVOLVED IN: response to other organism; LOCATED IN: cell wall; EXPRESSED IN: 8 plant structures; EXPRESSED DURING: 4 anthesis, C globular stage, 4 leaf senescence stage; CONTAINS InterPro DOMAIN/s: Haem peroxidase (InterPro:IPR010255), Plant peroxidase (InterPro:IPR000823), Peroxidases heam-ligand binding site (InterPro:IPR019793), Haem peroxidase, plant/fungal/bacterial (InterPro:IPR002016), Peroxidase, active site (InterPro:IPR019794); BEST Arabidopsis thaliana protein match is: Peroxidase superfamily protein (TAIR:AT2G18150.1)
AT5G01300	*2,018*		PEBP (phosphatidylethanolamine-binding protein) family protein; FUNCTIONS IN: phosphatidylethanolamine binding; INVOLVED IN: biological_process unknown; LOCATED IN: cellular_component unknown; EXPRESSED IN: 7 plant structures; EXPRESSED DURING: LP.04 four leaves visible, 4 anthesis, 4 leaf senescence stage, petal differentiation and expansion stage, LP.08 eight leaves visible; CONTAINS InterPro DOMAIN/s: YbhB/YbcL (InterPro:IPR005247), Phosphatidylethanolamine-binding protein PEBP (InterPro:IPR008914)
AT4G37720	*2,017*	PHYTOSULFOKINE 6 PRECURSOR (PSK6)	Probable phytosulfokines 6 precursor, coding for a unique plant peptide growth factor.
AT4G38420	*2,017*	SKU5 SIMILAR 9 (sks9)	SKU5 similar 9 (sks9); FUNCTIONS IN: oxidoreductase activity, copper ion binding; INVOLVED IN: oxidation reduction; LOCATED IN: plant-type cell wall; EXPRESSED IN: 12 plant structures; EXPRESSED DURING: 9 growth stages; CONTAINS InterPro DOMAIN/s: Multicopper oxidase, type 3 (InterPro:IPR011707), Cupredoxin (InterPro:IPR008972), Multicopper oxidase, type 2 (InterPro:IPR011706), Multicopper oxidase, type 1 (InterPro:IPR001117); BEST Arabidopsis thaliana protein match is: SKU5 similar 10 (TAIR:AT4G28090.1)
AT2G30830	*2,015*		encodes a protein whose sequence is similar to 2-oxoglutarate-dependent dioxygenase
AT5G26340	*2,014*	(MSS1)	Encodes a protein with high affinity, hexose-specific/H+ symporter activity. The activity of the transporter appears to be negatively regulated by phosphorylation. Importantly, microarray analysis, as well as the study of the expression of this gene in mutants involved in programmed cell death (PCD) demonstrated a tight correlation between this gene's expression and PCD.
AT4G35180	*2,009*	LYS/HIS TRANSPORTER 7 (LHT7)	LYS/HIS transporter 7 (LHT7); CONTAINS InterPro DOMAIN/s: Amino acid transporter, transmembrane (InterPro:IPR013057); BEST Arabidopsis thaliana protein match is: Transmembrane amino acid transporter family protein (TAIR:AT1G47670.1)
AT4G30430	*2,007*	TETRASPANIN9 (TET9)	Member of TETRASPANIN family
AT5G40990	*2,006*	GDSL LIPASE 1 (GLIP1)	Component of plant resistance. Contains lipase signature motif and GDSL domain. Directly interferes with the fungal infection process by acting on fungal cell walls through its action as a antimicrobial compound. Critical component for both local and systemic resistance responses in the incompatible interaction with Alternaria brassicicola in the ethylene-dependent pathway.
AT5G50160	*2*	FERRIC REDUCTION OXIDASE 8 (FRO8)	Encodes a ferric chelate reductase that is expressed in shoots and flowers.
AT5G38240	*1,985*		Protein kinase family protein; FUNCTIONS IN: protein serine/threonine kinase activity, protein kinase activity, kinase activity, ATP binding; INVOLVED IN: protein amino acid phosphorylation; LOCATED IN: endomembrane system; CONTAINS InterPro DOMAIN/s: Protein kinase, ATP binding site (InterPro:IPR017441), Serine/threonine-protein kinase domain (InterPro:IPR002290), Serine-threonine/tyrosine-protein kinase (InterPro:IPR001245), Protein kinase-like domain (InterPro:IPR011009), Serine/threonine-protein kinase, active site (InterPro:IPR008271), Protein kinase, catalytic domain (InterPro:IPR000719), Tyrosine-protein kinase, catalytic domain (InterPro:IPR020635); BEST Arabidopsis thaliana protein match is: Protein kinase family protein (TAIR:AT5G38250.1)
AT5G52710	*1,979*		Copper transport protein family; BEST Arabidopsis thaliana protein match is: Copper transport protein family (TAIR:AT5G52680.1)

AT2G05510	*1,972*		Glycine-rich protein family; FUNCTIONS IN: molecular_function unknown; INVOLVED IN: biological_process unknown; LOCATED IN: endomembrane system; CONTAINS InterPro DOMAIN/s: Glycine rich protein (InterPro:IPR010800); BEST Arabidopsis thaliana protein match is: GLYCINE RICH PROTEIN 9 (TAIR:AT2G05440.1)
AT3G57260	*1,969*	BETA-1,3-GLUCANASE 2 (BGL2)	beta 1,3-glucanase
AT5G19880	*1,968*		Peroxidase superfamily protein; FUNCTIONS IN: peroxidase activity, heme binding; INVOLVED IN: response to ethylene stimulus, oxidation reduction, response to oxidative stress, response to virus, N-terminal protein myristoylation; LOCATED IN: endomembrane system; EXPRESSED IN: 6 plant structures; EXPRESSED DURING: 4 anthesis, C globular stage; CONTAINS InterPro DOMAIN/s: Haem peroxidase (InterPro:IPR010255), Plant peroxidase (InterPro:IPR000823), Peroxidases heam-ligand binding site (InterPro:IPR019793), Peroxidase, active site (InterPro:IPR019794), Haem peroxidase, plant/fungal/bacterial (InterPro:IPR002016); BEST Arabidopsis thaliana protein match is: peroxidase 2 (TAIR:AT5G06720.1)
AT4G26950	*1,967*		Protein of unknown function, DUF584; CONTAINS InterPro DOMAIN/s: Protein of unknown function DUF584 (InterPro:IPR007608); BEST Arabidopsis thaliana protein match is: Protein of unknown function, DUF584 (TAIR:AT4G04630.1)
AT1G27480	*1,965*		alpha/beta-Hydrolases superfamily protein; FUNCTIONS IN: phosphatidylcholine-sterol O-acyltransferase activity; INVOLVED IN: lipid metabolic process; LOCATED IN: plasma membrane, vacuole; EXPRESSED IN: 21 plant structures; EXPRESSED DURING: 13 growth stages; CONTAINS InterPro DOMAIN/s: Lecithin:cholesterol acyltransferase (InterPro:IPR003386)
AT2G29460	*1,96*	GLUTATHIONE S-TRANSFERASE TAU 4 (GSTU4)	Encodes glutathione transferase belonging to the tau class of GSTs. Naming convention according to Wagner et al. (2002).
AT3G05650	*1,954*	RECEPTOR LIKE PROTEIN 32 (RLP32)	receptor like protein 32 (RLP32); FUNCTIONS IN: kinase activity; INVOLVED IN: signal transduction, defense response; LOCATED IN: endomembrane system; EXPRESSED IN: 22 plant structures; EXPRESSED DURING: 13 growth stages; CONTAINS InterPro DOMAIN/s: Leucine-rich repeat-containing N-terminal domain, type 2 (InterPro:IPR013210), Leucine-rich repeat (InterPro:IPR001611); BEST Arabidopsis thaliana protein match is: receptor like protein 35 (TAIR:AT3G11080.1)
AT5G59570	*1,95*	BROTHER OF LUX ARRHYTHMO (BOA)	Encodes BOA (BROTHER OF LUX ARRHYTHMO), a component of the circadian clock. The mRNA is cell-to-cell mobile.
AT1G65500	*1,946*		unknown protein; FUNCTIONS IN: molecular_function unknown; INVOLVED IN: biological_process unknown; LOCATED IN: endomembrane system; EXPRESSED IN: 14 plant structures; EXPRESSED DURING: 9 growth stages; BEST Arabidopsis thaliana protein match is: unknown protein (TAIR:AT1G65486.1)
AT4G33040	*1,944*		Thioredoxin superfamily protein; FUNCTIONS IN: electron carrier activity, protein disulfide oxidoreductase activity; INVOLVED IN: cell redox homeostasis; LOCATED IN: cellular_component unknown; EXPRESSED IN: 23 plant structures; EXPRESSED DURING: 13 growth stages; CONTAINS InterPro DOMAIN/s: Glutaredoxin-like, plant II (InterPro:IPR011905), Thioredoxin fold (InterPro:IPR012335), Glutaredoxin (InterPro:IPR002109), Thioredoxin-like fold (InterPro:IPR012336); BEST Arabidopsis thaliana protein match is: Thioredoxin superfamily protein (TAIR:AT5G11930.1)
AT1G64110	*1,94*	DUO1-ACTIVATED ATPASE 1 (DAA1)	Target promoter of the male germline-specific transcription factor DUO1.
AT4G24860	*1,939*		P-loop containing nucleoside triphosphate hydrolases superfamily protein; FUNCTIONS IN: nucleoside-triphosphatase activity, nucleotide binding, ATP binding; LOCATED IN: chloroplast; EXPRESSED IN: shoot apex, leaf whorl, sperm cell, sepal, flower; EXPRESSED DURING: petal differentiation and expansion stage; CONTAINS InterPro DOMAIN/s: ATPase, AAA-type, core (InterPro:IPR003959), ATPase, AAA+ type, core (InterPro:IPR003593), ATPase, AAA-type, conserved site (InterPro:IPR003960); BEST Arabidopsis thaliana protein match is: AAA-type ATPase family protein (TAIR:AT1G62130.1)
AT2G40520	*1,938*		Nucleotidyltransferase family protein; FUNCTIONS IN: nucleotidyltransferase activity; INVOLVED IN: biological_process unknown; LOCATED IN: cellular_component unknown; CONTAINS InterPro DOMAIN/s: Nucleotidyl transferase domain (InterPro:IPR002934); BEST Arabidopsis thaliana protein match is: PAP/OAS1 substrate-binding domain superfamily (TAIR:AT3G56320.1)

AT1G51090	*1,936*	(ATHMAD1)	Heavy metal associated domain containing protein involved in plant immunity.Mutants show an increase in root length under NO stress and reduction in root length under H2O2 stress conditions. Mutants show increases in defense responses to pathogens including hypersensitive lesions, increased resistance and induction of SAR genes.
AT1G21890	*1,932*	USUALLY MULTIPLE ACIDS MOVE IN AND OUT TRANSPORTERS 19 (UMAMIT19)	nodulin MtN21-like transporter family protein
AT1G66090	*1,932*		Disease resistance protein (TIR-NBS class); FUNCTIONS IN: transmembrane receptor activity, nucleoside-triphosphatase activity, nucleotide binding, ATP binding; INVOLVED IN: signal transduction, defense response, apoptosis, innate immune response; LOCATED IN: intrinsic to membrane, chloroplast; EXPRESSED IN: 22 plant structures; EXPRESSED DURING: 10 growth stages; CONTAINS InterPro DOMAIN/s: ATPase, AAA+ type, core (InterPro:IPR003593), NB-ARC (InterPro:IPR002182), Disease resistance protein (InterPro:IPR000767), Toll-Interleukin receptor (InterPro:IPR000157); BEST Arabidopsis thaliana protein match is: Disease resistance protein (TIR-NBS-LRR class) family (TAIR:AT1G56540.1)
AT5G19700	*1,93*	EARLY LEAF SENESCENCE 1 (ELS1)	Encodes a MATE transporter involved in leaf senescence and iron homeostasis.
AT3G17820	*1,927*	GLUTAMINE SYNTHETASE 1.3 (GLN1.3)	encodes a cytosolic glutamine synthetase, the enzyme has low affinity with substrate ammonium The mRNA is cell-to-cell mobile.
AT5G50750	*1,927*	REVERSIBLY GLYCOSYLATED POLYPEPTIDE 4 (RGP4)	RGP4 is a reversibly glycosylated polypeptide. Analyses using tagged RGP4 suggest that it is present in the cytosol and in association with the Golgi apparatus. Recombinant RGP4 does not have UDP-arabinose mutase activity based on an in vitro assay even though the related RGP1, RGP2, and RGP3 proteins do have activity in the same assay. RGP4 can form complexes with RGP1 and RGP2. RGP4 is expressed during seed development.
AT2G03020	*1,926*		Heat shock protein HSP20/alpha crystallin family; FUNCTIONS IN: aspartic-type endopeptidase activity; INVOLVED IN: proteolysis; LOCATED IN: cellular_component unknown; CONTAINS InterPro DOMAIN/s: Heat shock protein Hsp20 (InterPro:IPR002068), Small heat shock protein, predicted, plant (InterPro:IPR016952), Peptidase A1 (InterPro:IPR001461), HSP20-like chaperone (InterPro:IPR008978); BEST Arabidopsis thaliana protein match is: Heat shock protein HSP20/alpha crystallin family (TAIR:AT4G16540.1)
AT1G33030	*1,913*		O-methyltransferase family protein; CONTAINS InterPro DOMAIN/s: Winged helix-turn-helix transcription repressor DNA-binding (InterPro:IPR011991), Plant methyltransferase dimerisation (InterPro:IPR012967), O-methyltransferase, family 2 (InterPro:IPR001077), O-methyltransferase, COMT, eukaryota (InterPro:IPR016461); BEST Arabidopsis thaliana protein match is: O-methyltransferase 1 (TAIR:AT5G54160.1)
AT3G46090	*1,896*	(ZAT7)	ZAT7; FUNCTIONS IN: sequence-specific DNA binding transcription factor activity, zinc ion binding, nucleic acid binding; INVOLVED IN: response to chitin, regulation of transcription; LOCATED IN: intracellular; CONTAINS InterPro DOMAIN/s: Zinc finger, C2H2-like (InterPro:IPR015880), Zinc finger, C2H2-type (InterPro:IPR007087); BEST Arabidopsis thaliana protein match is: C2H2-type zinc finger family protein (TAIR:AT3G46080.1)
AT1G26730	*1,894*		EXS (ERD1/XPR1/SYG1) family protein; LOCATED IN: integral to membrane, plasma membrane; EXPRESSED IN: 22 plant structures; EXPRESSED DURING: 13 growth stages; CONTAINS InterPro DOMAIN/s: EXS, C-terminal (InterPro:IPR004342), SPX, N-terminal (InterPro:IPR004331); BEST Arabidopsis thaliana protein match is: EXS (ERD1/XPR1/SYG1) family protein (TAIR:AT1G35350.1)
AT4G19530	*1,892*		Encodes a TIR-NB-LRR resistance protein. Transient expression in tobacco induces cell death.
AT2G47520	*1,89*	ETHYLENE RESPONSE FACTOR 71 (ERF71)	encodes a member of the ERF (ethylene response factor) subfamily B-2 of ERF/AP2 transcription factor family. The protein contains one AP2 domain. There are 5 members in this subfamily including RAP2.2 AND RAP2.12.
AT1G18280	*1,888*	GLYCOSYLPHOSPHATIDYLINOSITOL-ANCHORED LIPID PROTEIN TRANSFER 3 (LTPG3)	Bifunctional inhibitor/lipid-transfer protein/seed storage 2S albumin superfamily protein; CONTAINS InterPro DOMAIN/s: Bifunctional inhibitor/plant lipid transfer protein/seed storage (InterPro:IPR016140), Plant lipid transfer protein/seed storage/trypsin-alpha amylase inhibitor (InterPro:IPR003612); BEST Arabidopsis thaliana protein match is: Bifunctional inhibitor/lipid-transfer protein/seed storage 2S albumin superfamily protein (TAIR:AT1G73560.1)

AT1G56150	*1,884*	SMALL AUXIN UPREGULATED 71 (SAUR71)	SAUR-like auxin-responsive protein family ; CONTAINS InterPro DOMAIN/s: Auxin responsive SAUR protein (InterPro:IPR003676); BEST Arabidopsis thaliana protein match is: SAUR-like auxin-responsive protein family (TAIR:AT3G12830.1)
AT1G15010	*1,876*		unknown protein; BEST Arabidopsis thaliana protein match is: unknown protein (TAIR:AT2G01300.1)
AT1G08440	*1,858*		Aluminium activated malate transporter family protein; INVOLVED IN: response to aluminum ion; CONTAINS InterPro DOMAIN/s: Malate transporter, aliminium toerance (InterPro:IPR020966); BEST Arabidopsis thaliana protein match is: aluminum-activated malate transporter 1 (TAIR:AT1G08430.1)
AT5G62800	*1,854*		Protein with RING/U-box and TRAF-like domains; FUNCTIONS IN: ubiquitin-protein ligase activity, zinc ion binding; INVOLVED IN: multicellular organismal development, protein ubiquitination, ubiquitin-dependent protein catabolic process; LOCATED IN: nucleus; EXPRESSED IN: stem; CONTAINS InterPro DOMAIN/s: TRAF-like (InterPro:IPR008974), Seven-in-absentia protein, TRAF-like domain (InterPro:IPR018121), Zinc finger, SIAH-type (InterPro:IPR013010), Seven In Absentia Homolog-type (InterPro:IPR013323), Seven-in-absentia protein, sina (InterPro:IPR004162); BEST Arabidopsis thaliana protein match is: Protein with RING/U-box and TRAF-like domains (TAIR:AT5G37890.1)
AT3G25620	*1,85*	ATP-BINDING CASSETTE G21 (ABCG21)	ABC-2 type transporter family protein; FUNCTIONS IN: ATPase activity, coupled to transmembrane movement of substances; LOCATED IN: chloroplast, membrane; EXPRESSED IN: 17 plant structures; EXPRESSED DURING: 9 growth stages; CONTAINS InterPro DOMAIN/s: ATPase, AAA+ type, core (InterPro:IPR003593), ABC transporter-like (InterPro:IPR003439), ABC transporter, conserved site (InterPro:IPR017871), ABC-2 type transporter (InterPro:IPR013525); BEST Arabidopsis thaliana protein match is: ATP-binding cassette 14 (TAIR:AT1G31770.1).
AT5G21150	*1,848*	ARGONAUTE 9 (AGO9)	AGO9-dependent sRNA silencing is crucial to specify cell fate in the Arabidopsis ovule. AGO9 is expressed in reproductive companion cells but not in the associated male or female gametes or their precursors. Therefore, AGO9 acts non-cell autonomously to silencing the activity of TEs activity in the female gametophyte.Loss of function mutants produce ectopic megaspore mother cell and supernumary female gametophytes.
AT2G38250	*1,846*		Homeodomain-like superfamily protein; FUNCTIONS IN: sequence-specific DNA binding transcription factor activity; INVOLVED IN: regulation of transcription; LOCATED IN: nucleolus; EXPRESSED IN: stamen; EXPRESSED DURING: 4 anthesis, petal differentiation and expansion stage; CONTAINS InterPro DOMAIN/s: SANT, DNA-binding (InterPro:IPR001005), MYB-like (InterPro:IPR017877); BEST Arabidopsis thaliana protein match is: Homeodomain-like superfamily protein (TAIR:AT5G01380.1)
AT5G17780	*1,842*		alpha/beta-Hydrolases superfamily protein; FUNCTIONS IN: hydrolase activity; LOCATED IN: endomembrane system; EXPRESSED IN: 13 plant structures; EXPRESSED DURING: 8 growth stages; CONTAINS InterPro DOMAIN/s: Alpha/beta hydrolase fold-1 (InterPro:IPR000073); BEST Arabidopsis thaliana protein match is: alpha/beta-Hydrolases superfamily protein (TAIR:AT5G17720.1)
AT4G36950	*1,84*	MITOGEN-ACTIVATED PROTEIN KINASE KINASE KINASE 21 (MAPKKK21)	member of MEKK subfamily
AT5G46330	*1,84*	FLAGELLIN-SENSITIVE 2 (FLS2)	Encodes a leucine-rich repeat serine/threonine protein kinase that is expressed ubiquitously. FLS2 is involved in MAP kinase signalling relay involved in innate immunity. Essential in the perception of flagellin, a potent elicitor of the defense response. FLS2 is directed for degradation by the bacterial ubiquitin ligase AvrPtoB. The mRNA is cell-to-cell mobile.
AT4G00970	*1,834*	CYSTEINE-RICH RLK (RECEPTOR-LIKE PROTEIN KINASE) 41 (CRK41)	Encodes a cysteine-rich receptor-like protein kinase.

AT3G05630	*1,832*	PHOSPHOLIPASE D P2 (PLDP2)	Encodes a member of the PXPH-PLD subfamily of phospholipase D proteins. Regulates vesicle trafficking. Required for auxin transport and distribution and hence auxin responses. This subfamily is novel structurally different from the majority of plant PLDs by having phox homology (PX) and pleckstrin homology (PH) domains. Involved regulating root development in response to nutrient limitation. Plays a major role in phosphatidic acid production during phosphate deprivation. Induced upon Pi starvation in both shoots and roots. Involved in hydrolyzing phosphatidylcholine and phosphatidylethanolamine to produce diacylglycerol for digalactosyldiacylglycerol synthesis and free Pi to sustain other Pi-requiring processes. Does not appear to be involved in root hair patterning. Expression is upregulated in the shoot of cax1/cax3 mutant and is responsive to phosphate (Pi) and not phosphite (Phi) in roots and shoots.
AT1G32350	*1,827*	ALTERNATIVE OXIDASE 1D (AOX1D)	alternative oxidase 1D (AOX1D); FUNCTIONS IN: alternative oxidase activity; INVOLVED IN: oxidation reduction, response to cyclopentenone; LOCATED IN: mitochondrial envelope, mitochondrion; EXPRESSED IN: stem, sepal, leaf, stamen; EXPRESSED DURING: LP.04 four leaves visible, 4 anthesis, petal differentiation and expansion stage; CONTAINS InterPro DOMAIN/s: Alternative oxidase (InterPro:IPR002680); BEST Arabidopsis thaliana protein match is: alternative oxidase 1A (TAIR:AT3G22370.1)
AT5G03790	*1,826*	HOMEOBOX 51 (HB51)	Encodes a homeodomain leucine zipper class I (HD-Zip I) meristem identity regulator that acts together with LFY to induce CAL expression. It binds to the CAL promoter proximal CAATNATTG element. LMI1 acts primarily downstream of LFY in meristem identity regulation. The interaction between LFY, LMI1 and CAL resembles a feed-forward loop transcriptional network motif. The gene also had additional LFY-independent roles in leaf morphogenesis and bract formation.
AT2G18150	*1,824*		Peroxidase superfamily protein; FUNCTIONS IN: peroxidase activity, heme binding; INVOLVED IN: response to oxidative stress, oxidation reduction, response to nematode; LOCATED IN: cell wall; CONTAINS InterPro DOMAIN/s: Haem peroxidase (InterPro:IPR010255), Plant peroxidase (InterPro:IPR000823), Peroxidases heam-ligand binding site (InterPro:IPR019793), Haem peroxidase, plant/fungal/bacterial (InterPro:IPR002016), Peroxidase, active site (InterPro:IPR019794); BEST Arabidopsis thaliana protein match is: Peroxidase superfamily protein (TAIR:AT2G18140.1)
AT4G11480	*1,824*	CYSTEINE-RICH RLK (RECEPTOR-LIKE PROTEIN KINASE) 32 (CRK32)	Encodes a cysteine-rich receptor-like protein kinase.
AT1G80580	*1,821*		encodes a member of the ERF (ethylene response factor) subfamily B-1 of ERF/AP2 transcription factor family. The protein contains one AP2 domain. There are 15 members in this subfamily including ATERF-3, ATERF-4, ATERF-7, and leafy petiole.
AT5G04310	*1,819*		Pectin lyase-like superfamily protein; CONTAINS InterPro DOMAIN/s: Pectin lyase fold/virulence factor (InterPro:IPR011050), AmbAllergen (InterPro:IPR018082), Pectate lyase/Amb allergen (InterPro:IPR002022), Pectin lyase fold (InterPro:IPR012334); BEST Arabidopsis thaliana protein match is: Pectin lyase-like superfamily protein (TAIR:AT3G54920.1)
AT1G72520	*1,818*	LIPOXYGENASE 4 (LOX4)	PLAT/LH2 domain-containing lipoxygenase family protein; FUNCTIONS IN: oxidoreductase activity, acting on single donors with incorporation of molecular oxygen, incorporation of two atoms of oxygen, lipoxygenase activity, iron ion binding, metal ion binding; INVOLVED IN: growth, jasmonic acid biosynthetic process, response to wounding, defense response; LOCATED IN: chloroplast; EXPRESSED IN: 17 plant structures; EXPRESSED DURING: 8 growth stages; CONTAINS InterPro DOMAIN/s: Lipoxygenase, iron binding site (InterPro:IPR020833), Lipoxygenase, C-terminal (InterPro:IPR013819), Lipoxygenase, LH2 (InterPro:IPR001024), Lipase/lipooxygenase, PLAT/LH2 (InterPro:IPR008976), Lipoxygenase, conserved site (InterPro:IPR020834), Lipoxygenase (InterPro:IPR000907), Lipoxygenase, plant (InterPro:IPR001246); BEST Arabidopsis thaliana protein match is: lipoxygenase 3 (TAIR:AT1G17420.1)
AT3G48520	*1,816*	CYTOCHROME P450, FAMILY 94, SUBFAMILY B, POLYPEPTIDE 3 (CYP94B3)	CYP94B3 is a jasmonoyl-isoleucine-12-hydroxylase that catalyzes the formation of 12-OH-JA-Ile from JA-Ile. By reducing the levels of this the biologically active phytohormone, CYP94B3 attenuates the jasmonic acid signaling cascade. CYP94B3 transcript levels rise in response to wounding.
AT3G26830	*1,812*	PHYTOALEXIN DEFICIENT 3 (PAD3)	Mutations in pad3 are defective in biosynthesis of the indole derived phytoalexin camalexin. Encodes a cytochrome P450 enzyme that catalyzes the conversion of dihydrocamalexic acid to camalexin. The mRNA is cell-to-cell mobile.
AT5G20150	*1,799*	SPX DOMAIN GENE 1 (SPX1)	Expression is upregulated in the shoot of cax1/cax3 mutant. Additionally, its expression is responsive to both phosphate (Pi) and phosphite (Phi) in both roots and shoots. The mRNA is cell-to-cell mobile.

AT5G02030	*1,795*	REPLUMLESS (RPL)	Mutant has additional lateral organs and phyllotaxy defect. Encodes a homeodomain transcription factor. Has sequence similarity to the Arabidopsis ovule development regulator Bell1. Binds directly to the AGAMOUS cis-regulatory element. Its localization to the nucleus is dependent on the coexpression of either STM or BP.
AT1G59860	*1,79*		HSP20-like chaperones superfamily protein; CONTAINS InterPro DOMAIN/s: Heat shock protein Hsp20 (InterPro:IPR002068), HSP20-like chaperone (InterPro:IPR008978); BEST Arabidopsis thaliana protein match is: HSP20-like chaperones superfamily protein (TAIR:AT1G07400.1)
AT5G22540	*1,79*		Plant protein of unknown function (DUF247); CONTAINS InterPro DOMAIN/s: Protein of unknown function DUF247, plant (InterPro:IPR004158); BEST Arabidopsis thaliana protein match is: Plant protein of unknown function (DUF247) (TAIR:AT5G22550.2)
AT5G44310	*1,789*		Late embryogenesis abundant protein (LEA) family protein; INVOLVED IN: embryo development ending in seed dormancy; EXPRESSED IN: cotyledon; BEST Arabidopsis thaliana protein match is: Late embryogenesis abundant protein (LEA) family protein (TAIR:AT4G21020.1)
AT5G58120	*1,777*		Disease resistance protein (TIR-NBS-LRR class) family; FUNCTIONS IN: transmembrane receptor activity, nucleoside-triphosphatase activity, nucleotide binding, ATP binding; INVOLVED IN: signal transduction, apoptosis, defense response, innate immune response; LOCATED IN: intrinsic to membrane; EXPRESSED IN: 16 plant structures; EXPRESSED DURING: 9 growth stages; CONTAINS InterPro DOMAIN/s: ATPase, AAA+ type, core (InterPro:IPR003593), NB-ARC (InterPro:IPR002182), Leucine-rich repeat (InterPro:IPR001611), Toll-Interleukin receptor (InterPro:IPR000157), Disease resistance protein (InterPro:IPR000767); BEST Arabidopsis thaliana protein match is: Disease resistance protein (TIR-NBS-LRR class) family (TAIR:AT1G63750.3)
AT5G65510	*1,768*	AINTEGUMENTA-LIKE 7 (AIL7)	Encodes one of three PLETHORA transcription factors required to maintain high levels of PIN1 expression at the periphery of the meristem and modulate local auxin production in the central region of the SAM which underlies phyllotactic transitions.
AT1G71140	*1,765*		MATE efflux family protein; FUNCTIONS IN: antiporter activity, drug transmembrane transporter activity, transporter activity; INVOLVED IN: drug transmembrane transport, transmembrane transport; LOCATED IN: plasma membrane, membrane; EXPRESSED IN: 13 plant structures; EXPRESSED DURING: 8 growth stages; CONTAINS InterPro DOMAIN/s: MATE family transporter related protein (InterPro:IPR015521), Multi antimicrobial extrusion protein MatE (InterPro:IPR002528); BEST Arabidopsis thaliana protein match is: MATE efflux family protein (TAIR:AT1G15150.1)
AT1G43910	*1,758*		P-loop containing nucleoside triphosphate hydrolases superfamily protein; FUNCTIONS IN: nucleoside-triphosphatase activity, ATPase activity, nucleotide binding, ATP binding; LOCATED IN: endomembrane system; EXPRESSED IN: 23 plant structures; EXPRESSED DURING: 13 growth stages; CONTAINS InterPro DOMAIN/s: ATPase, AAA-type, core (InterPro:IPR003959), ATPase, AAA+ type, core (InterPro:IPR003593); BEST Arabidopsis thaliana protein match is: P-loop containing nucleoside triphosphate hydrolases superfamily protein (TAIR:AT4G05380.1)
AT2G16890	*1,758*		UDP-Glycosyltransferase superfamily protein; FUNCTIONS IN: transferase activity, transferring hexosyl groups, UDP-glycosyltransferase activity, transferase activity, transferring glycosyl groups; INVOLVED IN: metabolic process; LOCATED IN: endomembrane system; EXPRESSED IN: 17 plant structures; EXPRESSED DURING: 7 growth stages; CONTAINS InterPro DOMAIN/s: UDP-glucuronosyl/UDP-glucosyltransferase (InterPro:IPR002213), Tudor subgroup (InterPro:IPR018351); BEST Arabidopsis thaliana protein match is: UDP-Glycosyltransferase superfamily protein (TAIR:AT5G14860.1)
AT5G43860	*1,758*	CHLOROPHYLLASE 2 (CLH2)	Encodes a chlorophyllase, the first enzyme in chlorophyll degradation. It catalyzes the hydrolysis of the ester bond to chlorophyllide and phytol. AtCLH2 has a typical signal sequence for the chloroplast. Gene expression does not respond to methyljasmonate, a known promoter of senescence and chlorophyll degradation.
AT2G26380	*1,752*		Leucine-rich repeat (LRR) family protein; INVOLVED IN: signal transduction, defense response; LOCATED IN: endomembrane system; EXPRESSED IN: hypocotyl, root; CONTAINS InterPro DOMAIN/s: Leucine-rich repeat-containing N-terminal domain, type 2 (InterPro:IPR013210), Leucine-rich repeat (InterPro:IPR001611); BEST Arabidopsis thaliana protein match is: Leucine-rich repeat (LRR) family protein (TAIR:AT1G33590.1)

AT5G06760	*1,751*	LATE EMBRYOGENESIS ABUNDANT 4-5 (LEA4-5)	Encodes LEA4-5, a member of the Late Embryogenesis Abundant (LEA) proteins which typically accumulate in response to low water availability conditions imposed during development or by the environment. Most of the diverse set of LEA proteins can be grouped according to properties such as high hydrophilicity and high content of glycine or other small amino acids in what has been termed hydrophilins. LEA4-5 protects enzyme activities from the adverse effects induced by freeze-thaw cycles in vitro.
AT3G47290	*1,749*	PHOSPHATIDYLINOSITOL-SPECIWC PHOSPHOLIPASE C8 (PLC8)	phosphatidylinositol-speciwc phospholipase C8 (PLC8); FUNCTIONS IN: phospholipase C activity, phosphoinositide phospholipase C activity, phosphoric diester hydrolase activity; INVOLVED IN: signal transduction, intracellular signaling pathway, lipid metabolic process; CONTAINS InterPro DOMAIN/s: Phospholipase C, phosphatidylinositol-specific , X domain (InterPro:IPR000909), PLC-like phosphodiesterase, TIM beta/alpha-barrel domain (InterPro:IPR017946), C2 membrane targeting protein (InterPro:IPR018029), C2 calcium/lipid-binding domain, CaLB (InterPro:IPR008973), Phospholipase C, phosphoinositol-specific (InterPro:IPR001192), C2 calcium-dependent membrane targeting (InterPro:IPR000008), Phospholipase C, phosphatidylinositol-specific, Y domain (InterPro:IPR001711); BEST Arabidopsis thaliana protein match is: phosphatidylinositol-speciwc phospholipase C9 (TAIR:AT3G47220.1)
AT1G13370	*1,747*		Histone superfamily protein; FUNCTIONS IN: DNA binding; INVOLVED IN: nucleosome assembly; LOCATED IN: chloroplast, nucleosome; EXPRESSED IN: root, inflorescence, flower, leaf; EXPRESSED DURING: seedling growth; CONTAINS InterPro DOMAIN/s: Histone H3 (InterPro:IPR000164), Histone-fold (InterPro:IPR009072), Histone core (InterPro:IPR007125); BEST Arabidopsis thaliana protein match is: Histone superfamily protein (TAIR:AT4G40030.2)
AT2G46420	*1,743*		Plant protein 1589 of unknown function; CONTAINS InterPro DOMAIN/s: Conserved hypothetical protein CHP01589, plant (InterPro:IPR006476); BEST Arabidopsis thaliana protein match is: Plant protein 1589 of unknown function (TAIR:AT3G61700.1)
AT4G23250	*1,741*	EMBRYO DEFECTIVE 1290 (emb1290)	EMBRYO DEFECTIVE 1290 (EMB1290); FUNCTIONS IN: protein kinase activity, kinase activity; INVOLVED IN: protein amino acid autophosphorylation, embryo development ending in seed dormancy; LOCATED IN: plasma membrane; EXPRESSED IN: stem; CONTAINS InterPro DOMAIN/s: Protein kinase, ATP binding site (InterPro:IPR017441), Serine/threonine-protein kinase domain (InterPro:IPR002290), Protein of unknown function DUF26 (InterPro:IPR002902), Serine-threonine/tyrosine-protein kinase (InterPro:IPR001245), Protein kinase-like domain (InterPro:IPR011009), Serine/threonine-protein kinase, active site (InterPro:IPR008271), Protein kinase, catalytic domain (InterPro:IPR000719), Tyrosine-protein kinase, catalytic domain (InterPro:IPR020635), Concanavalin A-like lectin/glucanase (InterPro:IPR008985); BEST Arabidopsis thaliana protein match is: cysteine-rich RLK (RECEPTOR-like protein kinase) 18 (TAIR:AT4G23260.1)
AT2G22590	*1,738*		UDP-Glycosyltransferase superfamily protein; FUNCTIONS IN: transferase activity, transferring glycosyl groups; INVOLVED IN: metabolic process; LOCATED IN: cellular_component unknown; CONTAINS InterPro DOMAIN/s: UDP-glucuronosyl/UDP-glucosyltransferase (InterPro:IPR002213); BEST Arabidopsis thaliana protein match is: UDP-Glycosyltransferase superfamily protein (TAIR:AT5G65550.1)
AT4G37780	*1,738*	MYB DOMAIN PROTEIN 87 (MYB87)	encoded by the Myb-like transcription factor MYB87, regulates axillary meristem formation, expressed throughout the plant. Member of the R2R3 factor gene family.
AT1G03800	*1,737*	ERF DOMAIN PROTEIN 10 (ERF10)	encodes a member of the ERF (ethylene response factor) subfamily B-1 of ERF/AP2 transcription factor family (ATERF-10). The protein contains one AP2 domain. There are 15 members in this subfamily including ATERF-3, ATERF-4, ATERF-7, and leafy petiole.
AT3G09390	*1,733*	METALLOTHIONEIN 2A (MT2A)	metallothionein, binds to and detoxifies excess copper and other metals, limiting oxidative damage
AT5G49690	*1,733*		UDP-Glycosyltransferase superfamily protein; FUNCTIONS IN: transferase activity, transferring glycosyl groups; INVOLVED IN: metabolic process; CONTAINS InterPro DOMAIN/s: UDP-glucuronosyl/UDP-glucosyltransferase (InterPro:IPR002213); BEST Arabidopsis thaliana protein match is: UDP-Glycosyltransferase superfamily protein (TAIR:AT5G65550.1)

AT2G21330	*1,729*	FRUCTOSE-BISPHOSPHATE ALDOLASE 1 (FBA1)	fructose-bisphosphate aldolase 1 (FBA1); FUNCTIONS IN: fructose-bisphosphate aldolase activity, catalytic activity; INVOLVED IN: response to cadmium ion, pentose-phosphate shunt; LOCATED IN: in 8 components; EXPRESSED IN: 25 plant structures; EXPRESSED DURING: 15 growth stages; CONTAINS InterPro DOMAIN/s: Aldolase-type TIM barrel (InterPro:IPR013785), Fructose-bisphosphate aldolase, class-I (InterPro:IPR000741); BEST Arabidopsis thaliana protein match is: fructose-bisphosphate aldolase 2 (TAIR:AT4G38970.1)
AT4G33070	*1,728*	PYRUVATE DECARBOXYLASE 1 (PDC1)	Thiamine pyrophosphate dependent pyruvate decarboxylase family protein; FUNCTIONS IN: in 6 functions; LOCATED IN: membrane; EXPRESSED IN: 11 plant structures; EXPRESSED DURING: 4 anthesis, C globular stage, F mature embryo stage, petal differentiation and expansion stage, E expanded cotyledon stage; CONTAINS InterPro DOMAIN/s: TPP-binding enzyme, conserved site (InterPro:IPR000399), Thiamine pyrophosphate enzyme, central domain (InterPro:IPR012000), Pyruvate decarboxylase/indolepyruvate decarboxylase (InterPro:IPR012110), Thiamine pyrophosphate enzyme, N-terminal TPP-binding domain (InterPro:IPR012001), Thiamine pyrophosphate enzyme, C-terminal TPP-binding (InterPro:IPR011766); BEST Arabidopsis thaliana protein match is: Thiamine pyrophosphate dependent pyruvate decarboxylase family protein (TAIR:AT5G01320.1)
AT5G11410	*1,728*		Protein kinase superfamily protein; FUNCTIONS IN: protein kinase activity, kinase activity, ATP binding; INVOLVED IN: protein amino acid phosphorylation; EXPRESSED IN: 12 plant structures; EXPRESSED DURING: 8 growth stages; CONTAINS InterPro DOMAIN/s: Protein kinase, catalytic domain (InterPro:IPR000719), Serine/threonine-protein kinase-like domain (InterPro:IPR017442), Protein kinase-like domain (InterPro:IPR011009); BEST Arabidopsis thaliana protein match is: Protein kinase superfamily protein (TAIR:AT5G11400.2)
AT4G39210	*1,726*	(APL3)	Encodes the large subunit of ADP-Glucose Pyrophosphorylase which catalyzes the first, rate limiting step in starch biosynthesis. The large subunit plays a regulatory role whereas the small subunit (ApS) is the catalytic isoform. Four isoforms (ApL1-4) have been identified. ApL3 is the major large subunit isoform present in inflorescences, fruits and roots.
AT2G24270	*1,725*	ALDEHYDE DEHYDROGENASE 11A3 (ALDH11A3)	Encodes a protein with non-phosphorylating NADP-dependent glyceraldehyde-3-phosphate dehydrogenase activity. The activity of the enzyme was determined from leaf extracts; the enzyme has not been purified to confirm activity.
AT3G04220	*1,721*		Disease resistance protein (TIR-NBS-LRR class) family; FUNCTIONS IN: transmembrane receptor activity, nucleoside-triphosphatase activity, nucleotide binding, ATP binding; INVOLVED IN: signal transduction, defense response, apoptosis, innate immune response; LOCATED IN: intrinsic to membrane; EXPRESSED IN: 21 plant structures; EXPRESSED DURING: 13 growth stages; CONTAINS InterPro DOMAIN/s: ATPase, AAA+ type, core (InterPro:IPR003593), NB-ARC (InterPro:IPR002182), Leucine-rich repeat (InterPro:IPR001611), Disease resistance protein (InterPro:IPR000767), Toll-Interleukin receptor (InterPro:IPR000157); BEST Arabidopsis thaliana protein match is: Disease resistance protein (TIR-NBS-LRR class) (TAIR:AT5G11250.1)
AT5G22530	*1,719*		unknown protein; BEST Arabidopsis thaliana protein match is: unknown protein (TAIR:AT5G22520.1)
AT1G12200	*1,713*	FLAVIN MONOOXYGENASE (FMO)	Putative flavin monooxygenase.
AT5G07800	*1,713*		Flavin-binding monooxygenase family protein; FUNCTIONS IN: NADP or NADPH binding, monooxygenase activity, FAD binding, flavin-containing monooxygenase activity; INVOLVED IN: oxidation reduction; LOCATED IN: cellular_component unknown; EXPRESSED IN: 16 plant structures; EXPRESSED DURING: 6 growth stages; CONTAINS InterPro DOMAIN/s: Flavin-containing monooxygenase FMO (InterPro:IPR000960), Flavin-containing monooxygenase-like (InterPro:IPR020946); BEST Arabidopsis thaliana protein match is: Flavin-binding monooxygenase family protein (TAIR:AT5G61290.1)
AT5G43360	*1,711*	PHOSPHATE TRANSPORTER 1;3 (PHT1;3)	Encodes Pht1;3, a member of the Pht1 family of phosphate transporters which include: Pht1;1/At5g43350, Pht1;2/At5g43370, Pht1;3/At5g43360, Pht1;4/At2g38940, Pht1;5/At2g32830, Pht1;6/At5g43340, Pht1;7/At3g54700, Pht1;8/At1g20860, Pht1;9/At1g76430 (Plant Journal 2002, 31:341).
AT5G46590	*1,709*	NAC DOMAIN CONTAINING PROTEIN 96 (NAC096)	NAC domain containing protein 96 (NAC096); FUNCTIONS IN: sequence-specific DNA binding transcription factor activity; INVOLVED IN: multicellular organismal development, regulation of transcription; LOCATED IN: cellular_component unknown; EXPRESSED IN: 9 plant structures; EXPRESSED DURING: 6 growth stages; CONTAINS InterPro DOMAIN/s: No apical meristem (NAM) protein (InterPro:IPR003441); BEST Arabidopsis thaliana protein match is: NAC domain containing protein 71 (TAIR:AT4G17980.1)

AT3G03240	*1,708*		alpha/beta-Hydrolases superfamily protein; FUNCTIONS IN: catalytic activity; INVOLVED IN: biological_process unknown; LOCATED IN: endomembrane system; EXPRESSED IN: embryo, sepal, carpel; EXPRESSED DURING: 4 anthesis, C globular stage; BEST Arabidopsis thaliana protein match is: alpha/beta-Hydrolases superfamily protein (TAIR:AT3G03230.1)
AT5G53870	*1,706*	EARLY NODULIN-LIKE PROTEIN 1 (ENODL1)	early nodulin-like protein 1 (ENODL1); FUNCTIONS IN: electron carrier activity, copper ion binding; LOCATED IN: plasma membrane, anchored to membrane; EXPRESSED IN: 22 plant structures; EXPRESSED DURING: 9 growth stages; CONTAINS InterPro DOMAIN/s: Plastocyanin-like (InterPro:IPR003245), Cupredoxin (InterPro:IPR008972); BEST Arabidopsis thaliana protein match is: early nodulin-like protein 2 (TAIR:AT4G27520.1)
AT4G26200	*1,704*	1-AMINO-CYCLOPROPANE-1-CARBOXYLATE SYNTHASE 7 (ACS7)	Member of a family of proteins in Arabidopsis that encode 1-Amino-cyclopropane-1-carboxylate synthase, an enzyme involved in ethylene biosynthesis. Not expressed in response to IAA.
AT1G10960	*1,703*	FERREDOXIN 1 (FD1)	ferredoxin 1 (FD1); FUNCTIONS IN: electron carrier activity, iron-sulfur cluster binding, 2 iron, 2 sulfur cluster binding; INVOLVED IN: response to karrikin; LOCATED IN: chloroplast stroma, chloroplast; EXPRESSED IN: 22 plant structures; EXPRESSED DURING: 13 growth stages; CONTAINS InterPro DOMAIN/s: 2Fe-2S ferredoxin, iron-sulphur binding site (InterPro:IPR006058), Ferredoxin (InterPro:IPR001041), Ferredoxin [2Fe-2S], plant (InterPro:IPR010241), Beta-grasp fold, ferredoxin-type (InterPro:IPR012675); BEST Arabidopsis thaliana protein match is: 2Fe-2S ferredoxin-like superfamily protein (TAIR:AT1G60950.1)
AT3G61150	*1,7*	HOMEODOMAIN GLABROUS 1 (HDG1)	Encodes a homeobox-leucine zipper family protein belonging to the HD-ZIP IV family.
AT2G39030	*1,699*	N-ACETYLTRANSFERASE ACTIVITY 1 (NATA1)	Encodes a protein that acts as an ornithine N-delta-acetyltransferase, leading to the formation of N-delta-actetylornithine. This compound is likely used in plant defense and levels of it are increased in Arabidopsis plants in response to MeJA and ABA. The mRNA is cell-to-cell mobile.
AT2G35830	*1,697*		unknown protein; FUNCTIONS IN: molecular_function unknown; INVOLVED IN: biological_process unknown; LOCATED IN: cellular_component unknown; BEST Arabidopsis thaliana protein match is: unknown protein (TAIR:AT2G35810.1).
AT4G39070	*1,696*	BZS1 (BZS1)	Encodes BZS1, a brassinosteroids-regulated BZR1 target (BRBT) gene. BZS1 is a putative zinc finger transcription factor. Expression of BZS1 was increased under BR-deficient condition and repressed by BR. Transgenic Arabidopsis plants overexpressing BZS1 showed a hypersensitivity to the BR biosynthetic inhibitor brassinazole (BRZ). In contrast, transgenic plants expressing reduced level of BZS1 had longer hypocotyls than wild type when grown on BRZ.
AT5G07330	*1,689*		unknown protein; FUNCTIONS IN: molecular_function unknown; INVOLVED IN: biological_process unknown; LOCATED IN: mitochondrion; BEST Arabidopsis thaliana protein match is: unknown protein (TAIR:AT1G63060.1)
AT1G49330	*1,687*		hydroxyproline-rich glycoprotein family protein; BEST Arabidopsis thaliana protein match is: unknown protein (TAIR:AT2G16190.1)
AT1G54280	*1,687*	AMINOPHOSPHOLIPID ATPASE 6 (ALA6)	Encodes a member of the P4 subfamily of P-type ATPases expressed in the pollen plasma membrane. Double mutants with ALA7 display pollen and pollen tube defects.
AT1G06520	*1,681*	GLYCEROL-3-PHOSPHATE SN-2-ACYLTRANSFERASE 1 (GPAT1)	sn-glycerol-3-phosphate 2-O-acyltransferase. Expressed in flower buds and siliques. Homozygous mutant plants are male sterile.
AT1G32900	*1,681*	GRANULE BOUND STARCH SYNTHASE 1 (GBSS1)	UDP-Glycosyltransferase superfamily protein; FUNCTIONS IN: protein binding, transferase activity, transferring glycosyl groups; INVOLVED IN: biosynthetic process, glucan biosynthetic process; LOCATED IN: chloroplast; EXPRESSED IN: 21 plant structures; EXPRESSED DURING: 13 growth stages; CONTAINS InterPro DOMAIN/s: Glycogen/starch synthases, ADP-glucose type (InterPro:IPR011835), Starch synthase, catalytic domain (InterPro:IPR013534), Glycosyl transferase, group 1 (InterPro:IPR001296); BEST Arabidopsis thaliana protein match is: starch synthase 2 (TAIR:AT3G01180.1)
AT4G25810	*1,679*	XYLOGLUCAN ENDOTRANSGLYCOSYLASE 6 (XTR6)	xyloglucan endotransglycosylase-related protein (XTR6)

AT5G61520	*1,678*		Major facilitator superfamily protein; FUNCTIONS IN: carbohydrate transmembrane transporter activity, sugar:hydrogen symporter activity; INVOLVED IN: transport, transmembrane transport; LOCATED IN: integral to membrane, membrane; EXPRESSED IN: 15 plant structures; EXPRESSED DURING: 8 growth stages; CONTAINS InterPro DOMAIN/s: Sugar transporter, conserved site (InterPro:IPR005829), Major facilitator superfamily (InterPro:IPR020846), General substrate transporter (InterPro:IPR005828), Sugar/inositol transporter (InterPro:IPR003663), Major facilitator superfamily, general substrate transporter (InterPro:IPR016196); BEST Arabidopsis thaliana protein match is: Major facilitator superfamily protein (TAIR:AT1G34580.1)
AT5G52760	*1,675*		Copper transport protein family; BEST Arabidopsis thaliana protein match is: Heavy metal transport/detoxification superfamily protein (TAIR:AT5G52750.1)
AT4G16820	*1,674*	PHOSPHOLIPASE A I BETA 2 (PLA-I{beta]2)	Encodes a lipase that hydrolyzes phosphatidylcholine, glycolipids as well as triacylglycerols.
AT1G18710	*1,672*	MYB DOMAIN PROTEIN 47 (MYB47)	Member of the R2R3 factor gene family.
AT2G41470	*1,672*		unknown protein; FUNCTIONS IN: molecular_function unknown; INVOLVED IN: biological_process unknown; LOCATED IN: vacuole; BEST Arabidopsis thaliana protein match is: unknown protein (TAIR:AT2G41440.1)
AT5G40380	*1,672*	CYSTEINE-RICH RLK (RECEPTOR-LIKE PROTEIN KINASE) 42 (CRK42)	Encodes a cysteine-rich receptor-like protein kinase.
AT2G33420	*1,671*		CONTAINS InterPro DOMAIN/s: Munc13 homology 1 (InterPro:IPR014770), Protein of unknown function DUF810 (InterPro:IPR008528), Mammalian uncoordinated homology 13, domain 2 (InterPro:IPR014772); BEST Arabidopsis thaliana protein match is: Protein of unknown function (DUF810) (TAIR:AT1G04470.1)
AT5G13370	*1,669*		Auxin-responsive GH3 family protein; CONTAINS InterPro DOMAIN/s: GH3 auxin-responsive promoter (InterPro:IPR004993); BEST Arabidopsis thaliana protein match is: Auxin-responsive GH3 family protein (TAIR:AT5G13360.3)
AT5G52400	*1,668*	CYTOCHROME P450, FAMILY 715, SUBFAMILY A, POLYPEPTIDE 1 (CYP715A1)	member of CYP715A
AT4G11720	*1,666*	HAPLESS 2 (HAP2)	Encodes HAP2 with the following predicted motifs: an N-terminal secretion signal, a single transmembrane domain and a C-terminal histidine-rich domain. HAP2 is expressed only in the haploid sperm and is required for pollen tube guidance and fertilization. Predominantly localized to sperm endoplasmic reticulum membranes. May also reside in other endomembranes, including the plasma membrane. Target promoter of the male germline-specific transcription factor DUO1.
AT4G22690	*1,666*	CYTOCHROME P450, FAMILY 706, SUBFAMILY A, POLYPEPTIDE 1 (CYP706A1)	member of CYP706A The mRNA is cell-to-cell mobile.
AT2G37640	*1,665*	(EXP3)	member of Alpha-Expansin Gene Family. Naming convention from the Expansin Working Group (Kende et al, 2004. Plant Mol Bio). Involved in the formation of nematode-induced syncytia in roots of Arabidopsis thaliana.
AT5G51920	*1,665*		Pyridoxal phosphate (PLP)-dependent transferases superfamily protein; FUNCTIONS IN: pyridoxal phosphate binding, catalytic activity; INVOLVED IN: metabolic process; LOCATED IN: chloroplast; CONTAINS InterPro DOMAIN/s: Pyridoxal phosphate-dependent transferase, major domain (InterPro:IPR015424), Aminotransferase, class V/Cysteine desulfurase (InterPro:IPR000192), Pyridoxal phosphate-dependent transferase, major region, subdomain 1 (InterPro:IPR015421); BEST Arabidopsis thaliana protein match is: unknown protein (TAIR:AT4G22980.1)
AT2G22750	*1,661*		basic helix-loop-helix (bHLH) DNA-binding superfamily protein; FUNCTIONS IN: DNA binding, sequence-specific DNA binding transcription factor activity; INVOLVED IN: regulation of transcription; LOCATED IN: nucleus; EXPRESSED IN: sepal; EXPRESSED DURING: petal differentiation and expansion stage; CONTAINS InterPro DOMAIN/s: Helix-loop-helix DNA-binding domain (InterPro:IPR001092), Helix-loop-helix DNA-binding (InterPro:IPR011598); BEST Arabidopsis thaliana protein match is: basic helix-loop-helix (bHLH) DNA-binding superfamily protein (TAIR:AT4G37850.1)

AT4G15280	*1,659*	UDP-GLUCOSYL TRANSFERASE 71B5 (UGT71B5)	UDP-glucosyl transferase 71B5 (UGT71B5); FUNCTIONS IN: quercetin 3-O-glucosyltransferase activity, UDP-glycosyltransferase activity, transferase activity, transferring glycosyl groups; INVOLVED IN: metabolic process; LOCATED IN: cellular_component unknown; CONTAINS InterPro DOMAIN/s: UDP-glucuronosyl/UDP-glucosyltransferase (InterPro:IPR002213); BEST Arabidopsis thaliana protein match is: UDP-Glycosyltransferase superfamily protein (TAIR:AT4G15260.1)
AT2G36660	*1,658*	POLY(A) BINDING PROTEIN 7 (PAB7)	polyadenylate-binding protein, putative / PABP, putative. Member of the class III family of PABP proteins.
AT1G06540	*1,657*		unknown protein; BEST Arabidopsis thaliana protein match is: unknown protein (TAIR:AT2G30930.1)
AT1G09380	*1,657*	USUALLY MULTIPLE ACIDS MOVE IN AND OUT TRANSPORTERS 25 (UMAMIT25)	nodulin MtN21-like transporter family protein
AT3G53130	*1,656*	LUTEIN DEFICIENT 1 (LUT1)	Lutein-deficient 1 (LUT1) required for lutein biosynthesis, member of the xanthophyll class of carotenoids. Involved in epsilon ring hydroxylation. Maps at 67.3 cM on chromosome 3.
AT5G49520	*1,656*	WRKY DNA-BINDING PROTEIN 48 (WRKY48)	Encodes WRKY48, a member of the WRKY Transcription Factor. WRKY48 is a stress- and pathogen-induced transcriptional activator that represses plant basal defense. The mRNA is cell-to-cell mobile.
AT1G09950	*1,655*	RESPONSE TO ABA AND SALT 1 (RAS1)	RESPONSE TO ABA AND SALT 1 (RAS1); FUNCTIONS IN: molecular_function unknown; INVOLVED IN: biological_process unknown; LOCATED IN: endomembrane system; EXPRESSED IN: leaf apex, root, flower, leaf; EXPRESSED DURING: petal differentiation and expansion stage; BEST Arabidopsis thaliana protein match is: transcription factor-related (TAIR:AT1G58330.1)
AT2G15760	*1,653*		Protein of unknown function (DUF1645); FUNCTIONS IN: calmodulin binding; INVOLVED IN: biological_process unknown; LOCATED IN: plasma membrane; EXPRESSED IN: 19 plant structures; EXPRESSED DURING: 10 growth stages; CONTAINS InterPro DOMAIN/s: Protein of unknown function DUF1645 (InterPro:IPR012442); BEST Arabidopsis thaliana protein match is: Protein of unknown function (DUF1645) (TAIR:AT2G26530.2)
AT2G41850	*1,651*	POLYGALACTURONASE ABSCISSION ZONE A. THALIANA (PGAZAT)	ADPG2.
AT5G49630	*1,65*	AMINO ACID PERMEASE 6 (AAP6)	Is a high affinity amino acid transporter capable of transporting aspartate and tryptophan. May be involved in the amino acid uptake from xylem.
AT2G41100	*1,647*	TOUCH 3 (TCH3)	encodes a calmodulin-like protein, with six potential calcium binding domains. Calcium binding shown by Ca(2+)-specific shift in electrophoretic mobility. Expression induced by touch and darkness. Expression may also be developmentally controlled. Expression in growing regions of roots, vascular tissue, root/shoot junctions, trichomes, branch points of the shoot, and regions of siliques and flowers. The mRNA is cell-to-cell mobile.
AT5G02490	*1,647*	(Hsp70-2)	Heat shock protein 70 (Hsp 70) family protein; FUNCTIONS IN: protein binding; INVOLVED IN: protein folding, response to cadmium ion, response to heat, response to bacterium; LOCATED IN: cytosol, cell wall, plasma membrane; EXPRESSED IN: 19 plant structures; EXPRESSED DURING: 10 growth stages; CONTAINS InterPro DOMAIN/s: Heat shock protein 70, conserved site (InterPro:IPR018181), Heat shock protein Hsp70 (InterPro:IPR001023), Heat shock protein 70 (InterPro:IPR013126); BEST Arabidopsis thaliana protein match is: heat shock cognate protein 70-1 (TAIR:AT5G02500.1)
AT5G22520	*1,642*		unknown protein; BEST Arabidopsis thaliana protein match is: unknown protein (TAIR:AT5G22530.1)
AT1G18210	*1,641*		Calcium-binding EF-hand family protein; FUNCTIONS IN: calcium ion binding; INVOLVED IN: biological_process unknown; LOCATED IN: plasma membrane, vacuole; EXPRESSED IN: 25 plant structures; EXPRESSED DURING: 13 growth stages; CONTAINS InterPro DOMAIN/s: EF-Hand 1, calcium-binding site (InterPro:IPR018247), EF-HAND 2 (InterPro:IPR018249), EF-hand-like domain (InterPro:IPR011992), Calcium-binding EF-hand (InterPro:IPR002048), EF-hand (InterPro:IPR018248); BEST Arabidopsis thaliana protein match is: EF hand calcium-binding protein family (TAIR:AT1G73630.1)

AT2G02010	*1,639*	GLUTAMATE DECARBOXYLASE 4 (GAD4)	glutamate decarboxylase 4 (GAD4); FUNCTIONS IN: calmodulin binding; INVOLVED IN: carboxylic acid metabolic process, glutamate metabolic process, glutamate decarboxylation to succinate; LOCATED IN: cytosol, nucleus; CONTAINS InterPro DOMAIN/s: Pyridoxal phosphate-dependent transferase, major domain (InterPro:IPR015424), Pyridoxal phosphate-dependent decarboxylase (InterPro:IPR002129), Glutamate decarboxylase (InterPro:IPR010107), Pyridoxal phosphate-dependent transferase, major region, subdomain 1 (InterPro:IPR015421); BEST Arabidopsis thaliana protein match is: glutamate decarboxylase 3 (TAIR:AT2G02000.1)
AT5G46130	*1,638*		Protein of unknown function (DUF295); CONTAINS InterPro DOMAIN/s: Protein of unknown function DUF295 (InterPro:IPR005174); BEST Arabidopsis thaliana protein match is: Protein of unknown function (DUF295) (TAIR:AT5G46140.1)
AT2G20825	*1,633*	ULTRAPETALA 2 (ULT2)	ULTRAPETALA 2 (ULT2); FUNCTIONS IN: DNA binding; INVOLVED IN: biological_process unknown; LOCATED IN: cytosol, nucleus; EXPRESSED IN: 7 plant structures; EXPRESSED DURING: 6 growth stages; CONTAINS InterPro DOMAIN/s: Developmental regulator, ULTRAPETALA (InterPro:IPR020533), SAND domain (InterPro:IPR000770); BEST Arabidopsis thaliana protein match is: Developmental regulator, ULTRAPETALA (TAIR:AT4G28190.1)
AT2G19800	*1,631*	MYO-INOSITOL OXYGENASE 2 (MIOX2)	Encodes a myo-inositol oxygenase family gene.
AT1G79680	*1,629*	WALL ASSOCIATED KINASE (WAK)-LIKE 10 (WAKL10)	Encodes a twin-domain, kinase-GC signaling molecule that may function in biotic stress responses that is critically dependent on the second messenger cGMP.
AT2G14610	*1,629*	PATHOGENESIS-RELATED GENE 1 (PR1)	PR1 gene expression is induced in response to a variety of pathogens. It is a useful molecular marker for the SAR response. Though the Genbank record for the cDNA associated to this gene is called 'PR-1-like', the sequence actually corresponds to PR1. Expression of this gene is salicylic-acid responsive.
AT4G19720	*1,627*		Glycosyl hydrolase family protein with chitinase insertion domain; FUNCTIONS IN: cation binding, chitinase activity, hydrolase activity, hydrolyzing O-glycosyl compounds, catalytic activity; INVOLVED IN: carbohydrate metabolic process; LOCATED IN: cellular_component unknown; EXPRESSED IN: root; CONTAINS InterPro DOMAIN/s: Glycoside hydrolase, family 18, catalytic domain (InterPro:IPR001223), Chitinase II (InterPro:IPR011583), Glycoside hydrolase, catalytic core (InterPro:IPR017853), Glycoside hydrolase, subgroup, catalytic core (InterPro:IPR013781); BEST Arabidopsis thaliana protein match is: Glycosyl hydrolase family protein with chitinase insertion domain (TAIR:AT4G19810.1)
AT2G45450	*1,626*	LITTLE ZIPPER 1 (ZPR1)	ZPR1, a small leucine zipper-containing protein that interacts with REV HD-ZIPIII and is involved in the establishment of leaf polarity.
AT1G21520	*1,619*		unknown protein
AT5G17170	*1,618*	ENHANCER OF SOS3-1 (ENH1)	enhancer of sos3-1 (ENH1); FUNCTIONS IN: electron carrier activity, metal ion binding; LOCATED IN: chloroplast thylakoid membrane, chloroplast, chloroplast envelope; EXPRESSED IN: 22 plant structures; EXPRESSED DURING: 14 growth stages; CONTAINS InterPro DOMAIN/s: Rubredoxin-type Fe(Cys)4 protein (InterPro:IPR004039), PDZ/DHR/GLGF (InterPro:IPR001478); BEST Arabidopsis thaliana protein match is: Rubredoxin-like superfamily protein (TAIR:AT5G51010.1)
AT5G56970	*1,618*	CYTOKININ OXIDASE 3 (CKX3)	It encodes a protein whose sequence is similar to cytokinin oxidase/dehydrogenase, which catalyzes the degradation of cytokinins.
AT3G02480	*1,616*	ABA-RESPONSE PROTEIN (ABR)	Late embryogenesis abundant protein (LEA) family protein; BEST Arabidopsis thaliana protein match is: Late embryogenesis abundant protein (LEA) family protein (TAIR:AT5G38760.1)
AT3G13540	*1,612*	MYB DOMAIN PROTEIN 5 (MYB5)	Encodes a member of the MYB family of transcriptional regulators. MYB5 act as a negative regulator of trichome branching and play a role in the correct formation of the seed coat and possibly the formation the underlying endosperm layers. Loss of function mutations have defects in seed coat mucilage and columella cells as well as trichome defects (smaller and reduced number of branches).
AT1G66300	*1,61*		F-box/RNI-like/FBD-like domains-containing protein; CONTAINS InterPro DOMAIN/s: F-box domain, cyclin-like (InterPro:IPR001810), FBD (InterPro:IPR013596), F-box domain, Skp2-like (InterPro:IPR022364), FBD-like (InterPro:IPR006566), Leucine-rich repeat 2 (InterPro:IPR013101); BEST Arabidopsis thaliana protein match is: F-box/RNI-like/FBD-like domains-containing protein (TAIR:AT1G66310.1)

AT4G27730	*1,608*	OLIGOPEPTIDE TRANSPORTER 1 (OPT6)	oligopeptide transporter
AT4G28290	*1,606*		unknown protein; FUNCTIONS IN: molecular_function unknown; INVOLVED IN: biological_process unknown; LOCATED IN: chloroplast; EXPRESSED IN: 23 plant structures; EXPRESSED DURING: 15 growth stages
AT4G17090	*1,604*	CHLOROPLAST BETA-AMYLASE (CT-BMY)	Encodes a beta-amylase targeted to the chloroplast. Transgenic BMY8 RNAi lines fail to accumulate maltose during cold shock suggesting that maltose accumulation coincides with BMY8 expression. Apart from maltose, the sugar content of the RNAi lines were similar to wildtype (glucose and sucrose unaffected).
AT3G16530	*1,602*		Lectin like protein whose expression is induced upon treatment with chitin oligomers.
AT1G67000	*1,599*		Protein kinase superfamily protein; FUNCTIONS IN: protein serine/threonine kinase activity, protein kinase activity, kinase activity, ATP binding; INVOLVED IN: protein amino acid phosphorylation; LOCATED IN: endomembrane system; EXPRESSED IN: stem; CONTAINS InterPro DOMAIN/s: Protein kinase, ATP binding site (InterPro:IPR017441), Protein kinase, catalytic domain (InterPro:IPR000719), Serine-threonine/tyrosine-protein kinase (InterPro:IPR001245), Protein kinase-like domain (InterPro:IPR011009), Serine/threonine-protein kinase, active site (InterPro:IPR008271); BEST Arabidopsis thaliana protein match is: Protein kinase superfamily protein (TAIR:AT1G66930.1)
AT5G52750	*1,598*		Heavy metal transport/detoxification superfamily protein ; FUNCTIONS IN: metal ion binding; INVOLVED IN: metal ion transport; LOCATED IN: cellular_component unknown; EXPRESSED IN: 20 plant structures; EXPRESSED DURING: 12 growth stages; CONTAINS InterPro DOMAIN/s: Heavy metal transport/detoxification protein (InterPro:IPR006121); BEST Arabidopsis thaliana protein match is: Copper transport protein family (TAIR:AT5G52760.1)
AT5G61270	*1,598*	PHYTOCHROME-INTERACTING FACTOR7 (PIF7)	Basic helix-loop-helix (bHLH) phytochrome interacting factor. Interacts specifically with the far-red light?absorbing Pfr form of phyB through a conserved domain called the active phyB binding motif. Upon light exposure, PIF7 rapidly migrates to intranuclear speckles, where it colocalizes with phyB. Role as negative regulator of phyB-mediated seedling deetiolation.
AT4G16370	*1,597*	OLIGOPEPTIDE TRANSPORTER (OPT3)	Encodes a phloem-specific iron transporter that is essential for systemic iron signaling and redistribution of iron and cadmium. It loads iron into the phloem, facilitates iron recirculation from the xylem to the phloem, and regulates both shoot-to-root iron signaling and iron redistribution from mature to developing tissues.
AT4G15210	*1,592*	BETA-AMYLASE 5 (BAM5)	cytosolic beta-amylase expressed in rosette leaves and inducible by sugar. RAM1 mutants have reduced beta amylase in leaves and stems.
AT4G29770	*1,59*	HEAT-INDUCED TAS1 TARGET 1 (HTT1)	Target of trans acting-siR480/255. Testing.
AT3G21750	*1,589*	UDP-GLUCOSYL TRANSFERASE 71B1 (UGT71B1)	Encodes a glucosyltransferase that can attach glucose to a number of hydroxylated phenolic compounds as well as quercetins in vitro
AT5G39050	*1,587*	PHENOLIC GLUCOSIDE MALONYLTRANSFERASE 1 (PMAT1)	Encodes a malonyltransferase that may play a role in phenolic xenobiotic detoxification.
AT1G68320	*1,586*	MYB DOMAIN PROTEIN 62 (MYB62)	putative transcription factor: R2R3-MYB transcription family. Involved in regulation of phosphate starvation responses and gibberellic acid biosynthesis.
AT1G77380	*1,583*	AMINO ACID PERMEASE 3 (AAP3)	Amino acid permease which transports basic amino acids.
AT3G21720	*1,581*	ISOCITRATE LYASE (ICL)	Encodes a glyoxylate cycle enzyme isocitrate lyase (ICL).
AT2G47770	*1,58*	TSPO(OUTER MEMBRANE TRYPTOPHAN-RICH SENSORY PROTEIN)-RELATED (TSPO)	Encodes a membrane-bound protein designated AtTSPO (Arabidopsis thaliana TSPO-related). AtTSPO is related to the bacterial outer membrane tryptophan-rich sensory protein (TspO) and the mammalian mitochondrial 18 kDa Translocator Protein (18 kDa TSPO), members of the TspO/MBR domain-containing membrane proteins. Mainly detected in dry seeds, but can be induced in vegetative tissues by osmotic or salt stress or abscisic acid treatment. Located in endoplasmic reticulum and the Golgi stacks. It is degraded through the autophagy pathway.

AT1G32910	*1,579*		HXXXD-type acyl-transferase family protein; FUNCTIONS IN: transferase activity, transferring acyl groups other than amino-acyl groups, transferase activity; INVOLVED IN: biological_process unknown; LOCATED IN: cellular_component unknown; CONTAINS InterPro DOMAIN/s: Transferase (InterPro:IPR003480); BEST Arabidopsis thaliana protein match is: HXXXD-type acyl-transferase family protein (TAIR:AT1G78990.1)
AT2G17840	*1,576*	EARLY-RESPONSIVE TO DEHYDRATION 7 (ERD7)	Identified as drought-inducible gene by differential hybridization. Upregulated by high light, drought, cold and salt stress determined by microarray analysis.
AT3G05260	*1,574*		NAD(P)-binding Rossmann-fold superfamily protein; FUNCTIONS IN: oxidoreductase activity, binding, catalytic activity; INVOLVED IN: oxidation reduction, metabolic process; LOCATED IN: cellular_component unknown; EXPRESSED IN: seed; CONTAINS InterPro DOMAIN/s: Short-chain dehydrogenase/reductase, conserved site (InterPro:IPR020904), NAD(P)-binding domain (InterPro:IPR016040), Glucose/ribitol dehydrogenase (InterPro:IPR002347), Short-chain dehydrogenase/reductase SDR (InterPro:IPR002198); BEST Arabidopsis thaliana protein match is: NAD(P)-binding Rossmann-fold superfamily protein (TAIR:AT1G54870.1)
AT2G12190	*1,573*		Cytochrome P450 superfamily protein; FUNCTIONS IN: electron carrier activity, monooxygenase activity, iron ion binding, oxygen binding, heme binding; INVOLVED IN: oxidation reduction; EXPRESSED IN: stem, leaf whorl, male gametophyte, flower, stamen; EXPRESSED DURING: 4 anthesis; CONTAINS InterPro DOMAIN/s: Cytochrome P450 (InterPro:IPR001128), Cytochrome P450, conserved site (InterPro:IPR017972), Cytochrome P450, E-class, group I (InterPro:IPR002401); BEST Arabidopsis thaliana protein match is: cytochrome P450, family 87, subfamily A, polypeptide 6 (TAIR:AT1G64940.1)
AT1G26770	*1,571*	EXPANSIN A10 (EXPA10)	Encodes an expansin. Naming convention from the Expansin Working Group (Kende et al, Plant Mol Bio). Involved in the formation of nematode-induced syncytia in roots of Arabidopsis thaliana.
AT4G18250	*1,57*		receptor serine/threonine kinase, putative; FUNCTIONS IN: transmembrane receptor protein serine/threonine kinase activity, kinase activity; INVOLVED IN: protein amino acid phosphorylation; EXPRESSED IN: 8 plant structures; EXPRESSED DURING: LP.06 six leaves visible, LP.04 four leaves visible, LP.10 ten leaves visible, LP.02 two leaves visible, LP.08 eight leaves visible; CONTAINS InterPro DOMAIN/s: Protein kinase, ATP binding site (InterPro:IPR017441), Serine/threonine-protein kinase domain (InterPro:IPR002290), Serine-threonine/tyrosine-protein kinase (InterPro:IPR001245), Protein kinase-like domain (InterPro:IPR011009), Serine/threonine-protein kinase, active site (InterPro:IPR008271), Protein kinase, catalytic domain (InterPro:IPR000719), Tyrosine-protein kinase, catalytic domain (InterPro:IPR020635), Thaumatin, conserved site (InterPro:IPR017949), Thaumatin, pathogenesis-related (InterPro:IPR001938); BEST Arabidopsis thaliana protein match is: receptor serine/threonine kinase, putative (TAIR:AT1G70250.1)
AT5G14760	*1,57*	L-ASPARTATE OXIDASE (AO)	At5g14760 encodes for L-aspartate oxidase involved in the early steps of NAD biosynthesis. In contrary to the EC 1.4.3.16 (l-aspartate oxidase - deaminating) the enzyme catalyzes the reaction L-aspartate + O2 = iminoaspartate (alpha-iminosuccinate) + H2O2
AT3G63140	*1,565*	CHLOROPLAST STEM-LOOP BINDING PROTEIN OF 41 KDA (CSP41A)	Encodes a protein with ribonuclease activity that is involved in plastid rRNA maturation.
AT3G20520	*1,562*	SHV3-LIKE 3 (SVL3)	Encodes a member of the glycerophosphodiester phosphodiesterase like (GDPD-like) family.
AT4G14730	*1,562*	LIFEGUARD 1 (LFG1)	Bax inhibitor-1 family protein; CONTAINS InterPro DOMAIN/s: Inhibitor of apoptosis-promoting Bax1 related (InterPro:IPR006214); BEST Arabidopsis thaliana protein match is: Bax inhibitor-1 family protein (TAIR:AT1G03070.2)
AT1G77920	*1,561*	TGACG SEQUENCE-SPECIFIC BINDING PROTEIN 7 (TGA7)	bZIP transcription factor family protein; CONTAINS InterPro DOMAIN/s: Basic-leucine zipper (bZIP) transcription factor (InterPro:IPR004827), bZIP transcription factor, bZIP-1 (InterPro:IPR011616); BEST Arabidopsis thaliana protein match is: TGA1A-related gene 3 (TAIR:AT1G22070.1)
AT5G10760	*1,558*	APOPLASTIC, EDS1-DEPENDENT 1 (AED1)	Eukaryotic aspartyl protease family protein; FUNCTIONS IN: aspartic-type endopeptidase activity; INVOLVED IN: proteolysis; LOCATED IN: apoplast; EXPRESSED IN: 18 plant structures; EXPRESSED DURING: 12 growth stages; CONTAINS InterPro DOMAIN/s: Peptidase aspartic (InterPro:IPR021109), Peptidase aspartic, catalytic (InterPro:IPR009007), Peptidase A1 (InterPro:IPR001461); BEST Arabidopsis thaliana protein match is: Eukaryotic aspartyl protease family protein (TAIR:AT5G10770.1)

AT1G67855	*1,553*		unknown protein
AT2G42530	*1,553*	COLD REGULATED 15B (COR15B)	Encodes COR15B, a protein that protects chloroplast membranes during freezing.
AT4G22470	*1,553*		protease inhibitor/seed storage/lipid transfer protein (LTP) family protein; FUNCTIONS IN: lipid binding; INVOLVED IN: lipid transport; LOCATED IN: endomembrane system; EXPRESSED IN: embryo, hypocotyl, sepal, stamen; EXPRESSED DURING: 4 anthesis, C globular stage, petal differentiation and expansion stage; CONTAINS InterPro DOMAIN/s: Bifunctional inhibitor/plant lipid transfer protein/seed storage (InterPro:IPR016140), Plant lipid transfer protein/hydrophobic protein, helical domain (InterPro:IPR013770); BEST Arabidopsis thaliana protein match is: Bifunctional inhibitor/lipid-transfer protein/seed storage 2S albumin superfamily protein (TAIR:AT4G22485.1)
AT1G70140	*1,548*	FORMIN 8 (FH8)	Encodes a group I formin. Binds to F-actin barbed ends. Has severing actin filaments activity. Binds profilin. Involved in the initiation and tip growth of root hairs through regulation of actin cytoskeleton.
AT4G33930	*1,545*		Encodes a protein with 14.6% glycine residues, similar to hyphally regulated protein from Candida albicans, PIR2:S58135
AT1G44890	*1,542*		BEST Arabidopsis thaliana protein match is: homolog of yeast oxidase assembly 1 (OXA1) (TAIR:AT5G62050.1)
AT4G15530	*1,541*	PYRUVATE ORTHOPHOSPHATE DIKINASE (PPDK)	Encodes a dual-targeted protein believed to act as a pyruvate, orthophosphate dikinase. These enzymes are normally associated with C4 photosynthesis which does not occur in Arabidopsis. However, PPDK may play a role in remobilizing nitrogen during leaf senescence in Arabidopsis. The product of the long transcript (.1 gene model) was shown to be targeted to the chloroplast, whereas the shorter transcript (no targeting sequence) accumulates in the cytosol. The two proteins were also found to be expressed in slightly different tissues.
AT4G16540	*1,541*		Heat shock protein HSP20/alpha crystallin family; FUNCTIONS IN: aspartic-type endopeptidase activity; INVOLVED IN: proteolysis; LOCATED IN: cellular_component unknown; CONTAINS InterPro DOMAIN/s: Heat shock protein Hsp20 (InterPro:IPR002068), Small heat shock protein, predicted, plant (InterPro:IPR016952), Peptidase A1 (InterPro:IPR001461), HSP20-like chaperone (InterPro:IPR008978); BEST Arabidopsis thaliana protein match is: Heat shock protein HSP20/alpha crystallin family (TAIR:AT2G03020.2)
AT4G34210	*1,541*	SKP1-LIKE 11 (SK11)	one of 20 SKP1 homologs in Arabidopsis. Protein is most similar to ASK12 and RNAi lines show defects in stamen development.
AT3G28150	*1,54*	TRICHOME BIREFRINGENCE-LIKE 22 (TBL22)	Encodes a member of the TBL (TRICHOME BIREFRINGENCE-LIKE) gene family containing a plant-specific DUF231 (domain of unknown function) domain. A putative xyloglucan O-acetyltransferase. TBL gene family has 46 members, two of which (TBR/AT5G06700 and TBL3/AT5G01360) have been shown to be involved in the synthesis and deposition of secondary wall cellulose, presumably by influencing the esterification state of pectic polymers. A nomenclature for this gene family has been proposed (Volker Bischoff & Wolf Scheible, 2010, personal communication).
AT5G49740	*1,533*	FERRIC REDUCTION OXIDASE 7 (FRO7)	Encodes a chloroplast ferric chelate reductase. Shows differential splicing and has three different mRNA products. Expressed in the shoot, flower and cotyledon.
AT1G51700	*1,532*	DOF ZINC FINGER PROTEIN 1 (DOF1)	Encodes dof zinc finger protein (adof1). The mRNA is cell-to-cell mobile.
AT5G10380	*1,528*	(RING1)	Encodes a RING finger domain protein with E3 ligase activity that is localized to the lipid rafts of the plasma membrane. Expression is increased in response to fungal pathogen. May be involved in regulation of programmed cell death by facilitating degredation of regulation of PDC activators. The mRNA is cell-to-cell mobile.
AT4G08770	*1,527*	PEROXIDASE 37 (Prx37)	Encodes a putative apoplastic peroxidase Prx37. Primarily expressed in the vascular bundles. Overexpression renders a dwarf phenotype with smaller plants and delayed development. Plants overexpressing Prx37 also shows an increase in the amount of esterified phenolic material associated with their walls.
AT2G41300	*1,522*	STRICTOSIDINE SYNTHASE-LIKE 1 (SSL1)	Although this enzyme is predicted to encode a strictosidine synthase (SS), it lacks a conserved catalytic glutamate residue found in active SS enzymes and it is not expected to have SS activity.
AT2G22860	*1,521*	PHYTOSULFOKINE 2 PRECURSOR (PSK2)	Phytosulfokine 2 precursor, coding for a unique plant peptide growth factor. The mRNA is cell-to-cell mobile.

AT4G31800	*1,519*	WRKY DNA-BINDING PROTEIN 18 (WRKY18)	Pathogen-induced transcription factor. Binds W-box sequences in vitro. Forms protein complexes with itself and with WRKY40 and WRKY60. Constitutive expression of WRKY18 enhanced resistance to P. syringae, but its coexpression with WRKY40 or WRKY60 made plants more susceptible to both P. syringae and B. cinerea. WRKY18, WRKY40, and WRKY60 have partially redundant roles in response to the hemibiotrophic bacterial pathogen Pseudomonas syringae and the necrotrophic fungal pathogen Botrytis cinerea, with WRKY18 playing a more important role than the other two. The mRNA is cell-to-cell mobile.
AT4G13820	*1,518*		Leucine-rich repeat (LRR) family protein; FUNCTIONS IN: kinase activity; INVOLVED IN: signal transduction; LOCATED IN: endomembrane system; EXPRESSED IN: stem, leaf whorl, hypocotyl, root, leaf; EXPRESSED DURING: LP.06 six leaves visible; CONTAINS InterPro DOMAIN/s: Leucine-rich repeat-containing N-terminal domain, type 2 (InterPro:IPR013210), Leucine-rich repeat (InterPro:IPR001611); BEST Arabidopsis thaliana protein match is: receptor like protein 47 (TAIR:AT4G13810.1)
AT5G53750	*1,514*		CBS domain-containing protein; CONTAINS InterPro DOMAIN/s: Cystathionine beta-synthase, core (InterPro:IPR000644); BEST Arabidopsis thaliana protein match is: Cystathionine beta-synthase (CBS) family protein (TAIR:AT4G27460.1)
AT2G41190	*1,513*		Transmembrane amino acid transporter family protein; FUNCTIONS IN: amino acid transmembrane transporter activity; INVOLVED IN: amino acid transport; LOCATED IN: plasma membrane, membrane; EXPRESSED IN: 24 plant structures; EXPRESSED DURING: 13 growth stages; CONTAINS InterPro DOMAIN/s: Amino acid transporter, transmembrane (InterPro:IPR013057); BEST Arabidopsis thaliana protein match is: Transmembrane amino acid transporter family protein (TAIR:AT2G39130.1)
AT5G53190	*1,51*	(SWEET3)	Nodulin MtN3 family protein; INVOLVED IN: biological_process unknown; LOCATED IN: integral to membrane, membrane; EXPRESSED IN: 8 plant structures; EXPRESSED DURING: 4 anthesis, C globular stage, petal differentiation and expansion stage, E expanded cotyledon stage; CONTAINS InterPro DOMAIN/s: MtN3/saliva-related transmembrane protein, conserved region (InterPro:IPR018169), RAG1-activating protein 1 homologue (InterPro:IPR018179), RAG1-activating protein-1-related (InterPro:IPR004316); BEST Arabidopsis thaliana protein match is: Nodulin MtN3 family protein (TAIR:AT1G21460.1)
AT3G11170	*1,508*	FATTY ACID DESATURASE 7 (FAD7)	Chloroplastic enzyme responsible for the synthesis of 16:3 and 18:3 fatty acids from galactolipids, sulpholipids and phosphatidylglycerol. Uses ferredoxin as electron donor. Gene expression is induced by wounding in shoot and root. The wound-response in shoot is independent of jasmonic acid mediated pathway whereas the root response is mediated by jasmonic acid. The mRNA is cell-to-cell mobile.
AT1G19380	*1,507*		Protein of unknown function (DUF1195); CONTAINS InterPro DOMAIN/s: Protein of unknown function DUF1195 (InterPro:IPR010608); BEST Arabidopsis thaliana protein match is: Protein of unknown function (DUF1195) (TAIR:AT4G36660.1)
AT2G26740	*1,505*	SOLUBLE EPOXIDE HYDROLASE (SEH)	Encodes a soluble epoxide hydrolase whose expression is induced by auxin and water stress.
AT2G33020	*1,504*	RECEPTOR LIKE PROTEIN 24 (RLP24)	receptor like protein 24 (RLP24); FUNCTIONS IN: kinase activity; INVOLVED IN: signal transduction; LOCATED IN: endomembrane system; EXPRESSED IN: root, cultured cell; CONTAINS InterPro DOMAIN/s: Leucine-rich repeat (InterPro:IPR001611); BEST Arabidopsis thaliana protein match is: receptor like protein 23 (TAIR:AT2G32680.1)
AT5G11740	*1,502*	ARABINOGALACTAN PROTEIN 15 (AGP15)	Encodes arabinogalactan protein (AGP15). The mRNA is cell-to-cell mobile.
AT1G10370	*1,5*	EARLY-RESPONSIVE TO DEHYDRATION 9 (ERD9)	Encodes GSTU17 (Glutathione S-Transferase U17). Functions as a negative component of stress-mediated signal transduction pathways in drought and salt stress responses.
AT1G12320	*1,491*		Protein of unknown function (DUF1442); CONTAINS InterPro DOMAIN/s: Protein of unknown function DUF1442 (InterPro:IPR009902); BEST Arabidopsis thaliana protein match is: Protein of unknown function (DUF1442) (TAIR:AT1G62840.1)

AT1G74010	*1,491*		Calcium-dependent phosphotriesterase superfamily protein; FUNCTIONS IN: strictosidine synthase activity; INVOLVED IN: biosynthetic process; LOCATED IN: plant-type cell wall; EXPRESSED IN: 11 plant structures; EXPRESSED DURING: L mature pollen stage, M germinated pollen stage, 4 anthesis, C globular stage, petal differentiation and expansion stage; CONTAINS InterPro DOMAIN/s: Strictosidine synthase, conserved region (InterPro:IPR018119), Strictosidine synthase (InterPro:IPR004141), Six-bladed beta-propeller, TolB-like (InterPro:IPR011042); BEST Arabidopsis thaliana protein match is: strictosidine synthase 2 (TAIR:AT1G74020.1)
AT2G39710	*1,491*		Encodes a Cysteine-rich peptide (CRP) family protein
AT4G39410	*1,491*	WRKY DNA-BINDING PROTEIN 13 (WRKY13)	Encodes a member of the Group II-c WRKY Transcription Factor family that is involved in stem development and has been shown to directly bind to the promoter of NST2. Mutants show a weak stem phenotype and show decreased expression of lignin-synthesis-related genes.
AT3G25780	*1,487*	ALLENE OXIDE CYCLASE 3 (AOC3)	Encodes allene oxide cyclase, one of the enzymes involved in jasmonic acid biosynthesis. One of four genes in Arabidopsis that encode this enzyme. mRNA expression is upregulated in senescing leaves. Note: Nomenclature for Arabidopsis allene oxide cyclase 3 (AOC3, AT3G25780) gene is based on Stenzel et al. 2003 Plant Molecular Biology 51:895-911. AOC3 (AT3G25780) is also referred to as AOC2 in He et al. 2002 Plant Physiology, 128:876-884. The mRNA is cell-to-cell mobile.
AT3G03990	*1,485*	DWARF 14 (D14)	Encodes an alpha/beta hydrolase essential for strigolactone signaling. Degradation of the protein is promoted by strigolactone. The mRNA is cell-to-cell mobile.
AT4G06746	*1,483*	RELATED TO AP2 9 (RAP2.9)	encodes a member of the DREB subfamily A-5 of ERF/AP2 transcription factor family (RAP2.9). The protein contains one AP2 domain. There are 16 members in this subfamily including RAP2.1 and RAP2.10.
AT1G72930	*1,48*	TOLL/INTERLEUKIN-1 RECEPTOR-LIKE (TIR)	Toll/interleukin-1 receptor-like protein (TIR) mRNA,
AT2G01340	*1,48*	(At17.1)	Encodes a protein whose expression is responsive to nematode infection.
AT1G01480	*1,475*	1-AMINO-CYCLOPROPANE-1-CARBOXYLATE SYNTHASE 2 (ACS2)	a member of the 1-aminocyclopropane-1-carboxylate (ACC) synthase (S-adenosyl-L-methionine methylthioadenosine-lyase, EC 4.4.1.14) gene family, isolated from a flower-specific cDNA library.
AT2G32210	*1,474*		unknown protein; BEST Arabidopsis thaliana protein match is: unknown protein (TAIR:AT2G32190.1)
AT5G42900	*1,474*	COLD REGULATED GENE 27 (COR27)	cold regulated gene 27 (COR27); BEST Arabidopsis thaliana protein match is: unknown protein (TAIR:AT4G33980.1)
AT2G34600	*1,472*	JASMONATE-ZIM-DOMAIN PROTEIN 7 (JAZ7)	jasmonate-zim-domain protein 7 (JAZ7); CONTAINS InterPro DOMAIN/s: Tify (InterPro:IPR010399); BEST Arabidopsis thaliana protein match is: jasmonate-zim-domain protein 8 (TAIR:AT1G30135.1)
AT1G23840	*1,468*		unknown protein; LOCATED IN: endomembrane system; EXPRESSED IN: 18 plant structures; EXPRESSED DURING: 12 growth stages; BEST Arabidopsis thaliana protein match is: unknown protein (TAIR:AT1G23850.1)
AT5G16080	*1,466*	CARBOXYESTERASE 17 (CXE17)	carboxyesterase 17 (CXE17); FUNCTIONS IN: hydrolase activity; INVOLVED IN: metabolic process; LOCATED IN: cellular_component unknown; EXPRESSED IN: 11 plant structures; EXPRESSED DURING: 4 anthesis, petal differentiation and expansion stage; CONTAINS InterPro DOMAIN/s: Lipase, GDXG, active site (InterPro:IPR002168), Alpha/beta hydrolase fold-3 (InterPro:IPR013094); BEST Arabidopsis thaliana protein match is: alpha/beta-Hydrolases superfamily protein (TAIR:AT1G68620.1)
AT1G68290	*1,464*	ENDONUCLEASE 2 (ENDO2)	Encodes an endonuclease ENDO2. ENDO2 purified from transgenic Arabidopsis digests RNA, ssDNA, and dsDNA, with a substrate preference for ssDNA and RNA. ENDO2 produced and purified from Nicotiana benthamiana expression showed no demonstrable endonuclease activity, either towards single stranded DNA or mismatches, in vitro.
AT5G52020	*1,462*		encodes a member of the DREB subfamily A-4 of ERF/AP2 transcription factor family. The protein contains one AP2 domain. There are 17 members in this subfamily including TINY.
AT1G76930	*1,461*	EXTENSIN 4 (EXT4)	Encodes an Arabidopsis extensin gene that belongs to cell-wall hydroxyproline-rich glycoproteins. The cross-link of extensins enforces cell wall strength. Transgenic plants overexpressing this gene show an increase in stem thickness.

AT4G10500	*1,459*	DMR6-LIKE OXYGENASE 1 (DLO1)	2-oxoglutarate (2OG) and Fe(II)-dependent oxygenase superfamily protein; FUNCTIONS IN: oxidoreductase activity, acting on paired donors, with incorporation or reduction of molecular oxygen, 2-oxoglutarate as one donor, and incorporation of one atom each of oxygen into both donors, oxidoreductase activity; INVOLVED IN: secondary metabolic process; EXPRESSED IN: 12 plant structures; EXPRESSED DURING: 9 growth stages; CONTAINS InterPro DOMAIN/s: Oxoglutarate/iron-dependent oxygenase (InterPro:IPR005123); BEST Arabidopsis thaliana protein match is: 2-oxoglutarate (2OG) and Fe(II)-dependent oxygenase superfamily protein (TAIR:AT4G10490.1)
AT5G39760	*1,459*	HOMEOBOX PROTEIN 23 (HB23)	homeobox protein 23 (HB23); FUNCTIONS IN: DNA binding, sequence-specific DNA binding transcription factor activity; INVOLVED IN: regulation of transcription; EXPRESSED IN: 21 plant structures; EXPRESSED DURING: 12 growth stages; CONTAINS InterPro DOMAIN/s: Homeobox domain, ZF-HD class (InterPro:IPR006455), ZF-HD homeobox protein, Cys/His-rich dimerisation domain (InterPro:IPR006456), Homeodomain-related (InterPro:IPR012287); BEST Arabidopsis thaliana protein match is: homeobox protein 34 (TAIR:AT3G28920.1)
AT3G12910	*1,456*		NAC (No Apical Meristem) domain transcriptional regulator superfamily protein; CONTAINS InterPro DOMAIN/s: No apical meristem (NAM) protein (InterPro:IPR003441); BEST Arabidopsis thaliana protein match is: NAC domain containing protein 42 (TAIR:AT2G43000.1)
AT2G36630	*1,448*		Sulfite exporter TauE/SafE family protein; INVOLVED IN: N-terminal protein myristoylation; LOCATED IN: endomembrane system, integral to membrane; EXPRESSED IN: 22 plant structures; EXPRESSED DURING: 15 growth stages; CONTAINS InterPro DOMAIN/s: Protein of unknown function DUF81 (InterPro:IPR002781); BEST Arabidopsis thaliana protein match is: Sulfite exporter TauE/SafE family protein (TAIR:AT2G25737.1)
AT4G23180	*1,448*	CYSTEINE-RICH RLK (RECEPTOR-LIKE PROTEIN KINASE) 10 (CRK10)	Encodes a receptor-like protein kinase. Naming convention from Chen et al 2003 (PMID 14756307) The mRNA is cell-to-cell mobile.
AT4G34230	*1,446*	CINNAMYL ALCOHOL DEHYDROGENASE 5 (CAD5)	Encodes a catalytically active cinnamyl alcohol dehydrogenase which uses p-coumaryl aldehyde as a preferred substrate. It can also use sinapyl, caffeyl, coniferyl and d-hydroxyconiferyl aldehydes as substrates.
AT4G15150	*1,445*		glycine-rich protein
AT4G21840	*1,445*	METHIONINE SULFOXIDE REDUCTASE B8 (MSRB8)	methionine sulfoxide reductase B8 (MSRB8); FUNCTIONS IN: peptide-methionine-(S)-S-oxide reductase activity; INVOLVED IN: oxidation reduction; LOCATED IN: cytosol; EXPRESSED IN: root; CONTAINS InterPro DOMAIN/s: Methionine sulphoxide reductase B (InterPro:IPR002579), Mss4-like (InterPro:IPR011057); BEST Arabidopsis thaliana protein match is: methionine sulfoxide reductase B7 (TAIR:AT4G21830.2)
AT5G41610	*1,444*	CATION/H+ EXCHANGER 18 (CHX18)	member of Putative Na+/H+ antiporter family
AT1G27020	*1,443*		unknown protein; BEST Arabidopsis thaliana protein match is: unknown protein (TAIR:AT1G27030.1)
AT4G02280	*1,443*	SUCROSE SYNTHASE 3 (SUS3)	Encodes a protein with sucrose synthase activity (SUS3). It appears to be important for sucrose metabolism in developing seeds, especially during the late maturation phase, about 18 days after flowering.
AT2G36270	*1,441*	ABA INSENSITIVE 5 (ABI5)	Encodes a member of the basic leucine zipper transcription factor family, involved in ABA signalling during seed maturation and germination. The Arabidopsis abscisic acid (ABA)-insensitive abi5 mutants have pleiotropic defects in ABA response, including decreased sensitivity to ABA inhibition of germination and altered expression of some ABA-regulated genes. Comparison of seed and ABA-inducible vegetative gene expression in wild-type and abi5-1 plants indicates that ABI5 regulates a subset of late embryogenesis-abundant genes during both developmental stages.
AT5G14700	*1,44*		NAD(P)-binding Rossmann-fold superfamily protein; FUNCTIONS IN: coenzyme binding, binding, cinnamoyl-CoA reductase activity, catalytic activity; INVOLVED IN: lignin biosynthetic process, cellular metabolic process, metabolic process; LOCATED IN: cellular_component unknown; EXPRESSED IN: 22 plant structures; EXPRESSED DURING: 13 growth stages; CONTAINS InterPro DOMAIN/s: NAD-dependent epimerase/dehydratase (InterPro:IPR001509), NAD(P)-binding domain (InterPro:IPR016040); BEST Arabidopsis thaliana protein match is: NAD(P)-binding Rossmann-fold superfamily protein (TAIR:AT2G23910.1)

AT2G41230	*1,439*	ORGAN SIZE RELATED 1 (OSR1)	Encodes an ER-localized plant hormone-responsive gene and appears to act redundantly with ARGOS and ARL during organ growth. Over-expression modifies plant sensitivity to ethylene, leading to improved drought tolerance.
AT4G11470	*1,439*	CYSTEINE-RICH RLK (RECEPTOR-LIKE PROTEIN KINASE) 31 (CRK31)	Encodes a cysteine-rich receptor-like protein kinase.
AT4G02520	*1,437*	GLUTATHIONE S-TRANSFERASE PHI 2 (GSTF2)	Encodes glutathione transferase belonging to the phi class of GSTs. Naming convention according to Wagner et al. (2002). The expression of this gene is upregulated by herbicide safeners such as benoxacor and fenclorim.
AT5G43330	*1,435*	CYTOSOLIC-NAD-DEPENDENT MALATE DEHYDROGENASE 2 (c-NAD-MDH2)	predicted to encode a cytosolic malate dehydrogenase. The mRNA is cell-to-cell mobile.
AT2G39490	*1,432*		F-box family protein; FUNCTIONS IN: molecular_function unknown; INVOLVED IN: biological_process unknown; LOCATED IN: endomembrane system; CONTAINS InterPro DOMAIN/s: F-box domain, cyclin-like (InterPro:IPR001810), F-box domain, Skp2-like (InterPro:IPR022364)
AT5G01040	*1,432*	LACCASE 8 (LAC8)	putative laccase, knockout mutant showed early flowering
AT4G18810	*1,429*		NAD(P)-binding Rossmann-fold superfamily protein; FUNCTIONS IN: binding, catalytic activity; INVOLVED IN: metabolic process; EXPRESSED IN: 22 plant structures; EXPRESSED DURING: 13 growth stages; CONTAINS InterPro DOMAIN/s: NAD(P)-binding domain (InterPro:IPR016040), NADH:ubiquinone oxidoreductase intermediate-associated protein 30 (InterPro:IPR013857), NmrA-like (InterPro:IPR008030); BEST Arabidopsis thaliana protein match is: high chlorophyll fluorescence phenotype 173 (TAIR:AT1G16720.1).
AT3G54820	*1,424*	PLASMA MEMBRANE INTRINSIC PROTEIN 2;5 (PIP2;5)	plasma membrane intrinsic protein 2;5 (PIP2;5); FUNCTIONS IN: water channel activity; INVOLVED IN: transport, transmembrane transport; LOCATED IN: integral to membrane, membrane; EXPRESSED IN: 17 plant structures; EXPRESSED DURING: 10 growth stages; CONTAINS InterPro DOMAIN/s: Major intrinsic protein, conserved site (InterPro:IPR022357), Aquaporin (InterPro:IPR012269), Major intrinsic protein (InterPro:IPR000425); BEST Arabidopsis thaliana protein match is: plasma membrane intrinsic protein 2 (TAIR:AT2G37170.1)
AT2G40200	*1,422*		basic helix-loop-helix (bHLH) DNA-binding superfamily protein; FUNCTIONS IN: DNA binding, sequence-specific DNA binding transcription factor activity; INVOLVED IN: regulation of transcription; LOCATED IN: nucleus; EXPRESSED IN: 21 plant structures; EXPRESSED DURING: 13 growth stages; CONTAINS InterPro DOMAIN/s: Helix-loop-helix DNA-binding domain (InterPro:IPR001092), Helix-loop-helix DNA-binding (InterPro:IPR011598); BEST Arabidopsis thaliana protein match is: basic helix-loop-helix (bHLH) DNA-binding superfamily protein (TAIR:AT3G56770.1)
AT2G21590	*1,421*	(APL4)	Encodes the large subunit of ADP-glucose pyrophosphorylase, the enzyme which catalyzes the first and limiting step in starch biosynthesis. The large subunit plays a regulatory role whereas the small subunit (ApS) is the catalytic isoform. Four isoforms of the large subunit (ApL1-4) have been described.
AT5G26220	*1,42*	GAMMA-GLUTAMYL CYCLOTRANSFERASE 2;1 (GGCT2;1)	ChaC-like family protein; CONTAINS InterPro DOMAIN/s: ChaC-like protein (InterPro:IPR006840); BEST Arabidopsis thaliana protein match is: ChaC-like family protein (TAIR:AT4G31290.1)
AT2G30140	*1,416*	UDP-GLUCOSYL TRANSFERASE 87A2 (UGT87A2)	Encodes a putative glycosyltransferase. Regulates flowering time via FLOWERING LOCUS C.
AT5G53830	*1,416*	MPK3/6-TARGETED VQP 3 (MVQ3)	VQ motif-containing protein; FUNCTIONS IN: molecular_function unknown; INVOLVED IN: biological_process unknown; LOCATED IN: cellular_component unknown; EXPRESSED IN: 19 plant structures; EXPRESSED DURING: 11 growth stages; CONTAINS InterPro DOMAIN/s: VQ (InterPro:IPR008889); BEST Arabidopsis thaliana protein match is: VQ motif-containing protein (TAIR:AT3G15300.1)
AT1G14890	*1,415*		Plant invertase/pectin methylesterase inhibitor superfamily protein; FUNCTIONS IN: enzyme inhibitor activity, pectinesterase inhibitor activity, pectinesterase activity; INVOLVED IN: biological_process unknown; EXPRESSED IN: 22 plant structures; EXPRESSED DURING: 13 growth stages; CONTAINS InterPro DOMAIN/s: Pectinesterase inhibitor (InterPro:IPR006501); BEST Arabidopsis thaliana protein match is: Plant invertase/pectin methylesterase inhibitor superfamily protein (TAIR:AT2G01610.1)

AT3G45970	*1,414*	EXPANSIN-LIKE A1 (EXLA1)	member of EXPANSIN-LIKE. Naming convention from the Expansin Working Group (Kende et al, 2004. Plant Mol Bio) The mRNA is cell-to-cell mobile.
AT5G23850	*1,414*		FUNCTIONS IN: molecular_function unknown; INVOLVED IN: biological_process unknown; LOCATED IN: cellular_component unknown; EXPRESSED IN: 23 plant structures; EXPRESSED DURING: 13 growth stages; CONTAINS InterPro DOMAIN/s: Lipopolysaccharide-modifying protein (InterPro:IPR006598), Protein of unknown function DUF821, CAP10-like (InterPro:IPR008539); BEST Arabidopsis thaliana protein match is: Arabidopsis thaliana protein of unknown function (DUF821) (TAIR:AT3G48980.1)
AT4G39230	*1,411*	PHENYLCOUMARAN BENZYLIC ETHER REDUCTASE 1 (PCBER1)	encodes a protein whose sequence is similar to phenylcoumaran benzylic ether reductase (PCBER), which catalyzes NADPH-dependent reduction of 8-5' linked lignans such as dehydrodiconiferyl alcohol to give isodihydrodehydrodiconiferyl alcohol.
AT5G37260	*1,411*	REVEILLE 2 (RVE2)	Encodes a MYB family transcription factor Circadian 1 (CIR1). Involved in circadian regulation in Arabidopsis.
AT5G53820	*1,411*		Late embryogenesis abundant protein (LEA) family protein; BEST Arabidopsis thaliana protein match is: Late embryogenesis abundant protein (LEA) family protein (TAIR:AT5G38760.1)
AT5G19110	*1,408*		Eukaryotic aspartyl protease family protein; FUNCTIONS IN: aspartic-type endopeptidase activity; INVOLVED IN: proteolysis, N-terminal protein myristoylation; LOCATED IN: endomembrane system; EXPRESSED IN: shoot apex, leaf apex, hypocotyl, root, leaf; CONTAINS InterPro DOMAIN/s: Peptidase aspartic (InterPro:IPR021109), Peptidase aspartic, catalytic (InterPro:IPR009007), Peptidase A1 (InterPro:IPR001461); BEST Arabidopsis thaliana protein match is: Eukaryotic aspartyl protease family protein (TAIR:AT1G03220.1)
AT2G45600	*1,405*		alpha/beta-Hydrolases superfamily protein; FUNCTIONS IN: hydrolase activity; INVOLVED IN: metabolic process; LOCATED IN: cellular_component unknown; EXPRESSED IN: 20 plant structures; EXPRESSED DURING: 8 growth stages; CONTAINS InterPro DOMAIN/s: Lipase, GDXG, active site (InterPro:IPR002168), Alpha/beta hydrolase fold-3 (InterPro:IPR013094); BEST Arabidopsis thaliana protein match is: alpha/beta-Hydrolases superfamily protein (TAIR:AT2G45610.1)
AT5G61560	*1,401*		U-box domain-containing protein kinase family protein; FUNCTIONS IN: ubiquitin-protein ligase activity, protein serine/threonine kinase activity, protein kinase activity, kinase activity, ATP binding; INVOLVED IN: protein amino acid phosphorylation, protein ubiquitination, response to stress; LOCATED IN: chloroplast, plasma membrane; EXPRESSED IN: 21 plant structures; EXPRESSED DURING: 13 growth stages; CONTAINS InterPro DOMAIN/s: UspA (InterPro:IPR006016), Protein kinase, ATP binding site (InterPro:IPR017441), U box domain (InterPro:IPR003613), Serine/threonine-protein kinase domain (InterPro:IPR002290), Serine-threonine/tyrosine-protein kinase (InterPro:IPR001245), Serine/threonine-protein kinase, active site (InterPro:IPR008271), Protein kinase-like domain (InterPro:IPR011009), Protein kinase, catalytic domain (InterPro:IPR000719), Tyrosine-protein kinase, catalytic domain (InterPro:IPR020635); BEST Arabidopsis thaliana protein match is: U-box domain-containing protein kinase family protein (TAIR:AT4G25160.1)
AT1G61840	*1,4*		Cysteine/Histidine-rich C1 domain family protein; FUNCTIONS IN: zinc ion binding; INVOLVED IN: intracellular signaling pathway; LOCATED IN: cellular_component unknown; CONTAINS InterPro DOMAIN/s: Protein kinase C-like, phorbol ester/diacylglycerol binding (InterPro:IPR002219), DC1 (InterPro:IPR004146), Zinc finger, PHD-type (InterPro:IPR001965), C1-like (InterPro:IPR011424); BEST Arabidopsis thaliana protein match is: Cysteine/Histidine-rich C1 domain family protein (TAIR:AT2G40050.1)
AT5G24660	*1,396*	RESPONSE TO LOW SULFUR 2 (LSU2)	RESPONSE TO LOW SULFUR 2 (LSU2); BEST Arabidopsis thaliana protein match is: response to low sulfur 4 (TAIR:AT5G24655.1)
AT2G18500	*1,391*	OVATE FAMILY PROTEIN 7 (OFP7)	ovate family protein 7 (OFP7); INVOLVED IN: biological_process unknown; LOCATED IN: plasma membrane; EXPRESSED IN: shoot apex, embryo, hypocotyl, flower, seed; EXPRESSED DURING: F mature embryo stage, petal differentiation and expansion stage, E expanded cotyledon stage, D bilateral stage; CONTAINS InterPro DOMAIN/s: Protein of unknown function DUF623 (InterPro:IPR006458); BEST Arabidopsis thaliana protein match is: ovate family protein 8 (TAIR:AT5G19650.1)
AT5G11110	*1,391*	SUCROSE PHOSPHATE SYNTHASE 2F (SPS2F)	Encodes a protein with putative sucrose-phosphate synthase activity.Involved in pollen exine formation.
AT3G23410	*1,39*	FATTY ALCOHOL OXIDASE 3 (FAO3)	Encodes a fatty alcohol oxidase.

AT3G03980	*1,388*		NAD(P)-binding Rossmann-fold superfamily protein; FUNCTIONS IN: oxidoreductase activity, binding, catalytic activity; INVOLVED IN: oxidation reduction, metabolic process; EXPRESSED IN: 25 plant structures; EXPRESSED DURING: 15 growth stages; CONTAINS InterPro DOMAIN/s: Short-chain dehydrogenase/reductase, conserved site (InterPro:IPR020904), NAD(P)-binding domain (InterPro:IPR016040), Glucose/ribitol dehydrogenase (InterPro:IPR002347), Short-chain dehydrogenase/reductase SDR (InterPro:IPR002198); BEST Arabidopsis thaliana protein match is: NAD(P)-binding Rossmann-fold superfamily protein (TAIR:AT3G04000.1)
AT5G23000	*1,386*	MYB DOMAIN PROTEIN 37 (MYB37)	Putative homolog of the Blind gene in tomato. Together with RAX2 and RAX3 belong to the class R2R3 MYB genes; encoded by the Myb-like transcription factor MYB37, regulates axillary meristem formation. RAX1 is expressed in a small central domain within the boundary zone separating SAM and leaf primordia during early leaf primordium development and is currently the earliest spatial marker for future axillary meristems. Member of the R2R3 factor gene family.
AT5G38710	*1,386*		Methylenetetrahydrofolate reductase family protein; FUNCTIONS IN: proline dehydrogenase activity; INVOLVED IN: oxidation reduction, proline catabolic process, glutamate biosynthetic process; EXPRESSED IN: 21 plant structures; EXPRESSED DURING: 12 growth stages; CONTAINS InterPro DOMAIN/s: Proline dehydrogenase (InterPro:IPR002872), Proline oxidase (InterPro:IPR015659); BEST Arabidopsis thaliana protein match is: Methylenetetrahydrofolate reductase family protein (TAIR:AT3G30775.1)
AT3G18610	*1,383*	NUCLEOLIN LIKE 2 (NUC-L2)	Encodes ATNUC-L2 (NUCLEOLIN LIKE 2).
AT1G22570	*1,378*		Major facilitator superfamily protein; FUNCTIONS IN: transporter activity; INVOLVED IN: oligopeptide transport; LOCATED IN: membrane; EXPRESSED IN: 17 plant structures; EXPRESSED DURING: 10 growth stages; CONTAINS InterPro DOMAIN/s: PTR2 family proton/oligopeptide symporter, conserved site (InterPro:IPR018456), Oligopeptide transporter (InterPro:IPR000109), Major facilitator superfamily, general substrate transporter (InterPro:IPR016196); BEST Arabidopsis thaliana protein match is: Major facilitator superfamily protein (TAIR:AT1G22550.1)
AT2G42170	*1,378*		Actin family protein; FUNCTIONS IN: structural constituent of cytoskeleton, ATP binding; INVOLVED IN: biological_process unknown; LOCATED IN: actin cytoskeleton; EXPRESSED IN: vascular bundle; CONTAINS InterPro DOMAIN/s: Actin, conserved site (InterPro:IPR004001), Actin/actin-like (InterPro:IPR004000); BEST Arabidopsis thaliana protein match is: Actin-like ATPase superfamily protein (TAIR:AT2G42100.1)
AT5G14780	*1,376*	FORMATE DEHYDROGENASE (FDH)	Encodes a NAD-dependent formate dehydrogenase.
AT4G16690	*1,371*	METHYL ESTERASE 16 (MES16)	Encodes a protein shown to have carboxylesterase activity, methyl IAA esterase activity, and methyl jasmonate esterase activity in vitro. This protein does not act on MeSA, MeGA4, or MEGA9 in vitro. Although MES16 is similar to MES17, a MeIAA hydrolase, two mes16 mutant lines (SALK_151578) and (SALK_139756) do not show altered sensitivity to MeIAA in root growth assays. MES16 transcripts appear to be more than 10-fold less abundant than those of MES17 in roots.
AT5G11160	*1,371*	ADENINE PHOSPHORIBOSYLTRANSFERASE 5 (APT5)	adenine phosphoribosyltransferase 5 (APT5); FUNCTIONS IN: adenine phosphoribosyltransferase activity; INVOLVED IN: nucleoside metabolic process, adenine salvage, anaerobic respiration, nucleotide metabolic process; LOCATED IN: cytoplasm; EXPRESSED IN: 23 plant structures; EXPRESSED DURING: 13 growth stages; CONTAINS InterPro DOMAIN/s: Adenine phosphoribosyl transferase (InterPro:IPR005764), Phosphoribosyltransferase (InterPro:IPR000836); BEST Arabidopsis thaliana protein match is: adenine phosphoribosyl transferase 2 (TAIR:AT1G80050.1)
AT4G27480	*1,369*		Core-2/I-branching beta-1,6-N-acetylglucosaminyltransferase family protein; FUNCTIONS IN: transferase activity, transferring glycosyl groups, acetylglucosaminyltransferase activity; INVOLVED IN: carbohydrate biosynthetic process, N-terminal protein myristoylation; LOCATED IN: endomembrane system, membrane; EXPRESSED IN: 12 plant structures; EXPRESSED DURING: 8 growth stages; CONTAINS InterPro DOMAIN/s: Glycosyl transferase, family 14 (InterPro:IPR003406), Core-2/I-Branching enzyme (InterPro:IPR021141); BEST Arabidopsis thaliana protein match is: Core-2/I-branching beta-1,6-N-acetylglucosaminyltransferase family protein (TAIR:AT3G15350.2)
AT4G29670	*1,36*	ATYPICAL CYS HIS RICH THIOREDOXIN 2 (ACHT2)	Encodes a member of the thioredoxin family protein. Located in the chloroplast. Shows high activity towards the chloroplast 2-Cys peroxiredoxin A, and poor activity towards the chloroplast NADP-malate dehydrogenase.

AT1G16640	*1,358*		AP2/B3-like transcriptional factor family protein; FUNCTIONS IN: DNA binding, sequence-specific DNA binding transcription factor activity; INVOLVED IN: regulation of transcription, DNA-dependent; LOCATED IN: cellular_component unknown; EXPRESSED IN: 16 plant structures; EXPRESSED DURING: 11 growth stages; CONTAINS InterPro DOMAIN/s: Transcriptional factor B3 (InterPro:IPR003340); BEST Arabidopsis thaliana protein match is: AP2/B3-like transcriptional factor family protein (TAIR:AT3G06220.1)
AT1G48000	*1,353*	MYB DOMAIN PROTEIN 112 (MYB112)	Encodes a putative transcription factor (MYB112).
AT1G17380	*1,351*	JASMONATE-ZIM-DOMAIN PROTEIN 5 (JAZ5)	jasmonate-zim-domain protein 5 (JAZ5); CONTAINS InterPro DOMAIN/s: Tify (InterPro:IPR010399), CCT domain-like (InterPro:IPR018467); BEST Arabidopsis thaliana protein match is: jasmonate-zim-domain protein 6 (TAIR:AT1G72450.1)
AT5G13730	*1,348*	SIGMA FACTOR 4 (SIG4)	Encodes sigma 4 factor, involved in regulating the activity of the plastid-encoded RNA polymerase PEP. Regulates the overall quantity of NDH complexes and thus influences NDH activity.
AT1G71110	*1,347*		unknown protein; FUNCTIONS IN: molecular_function unknown; INVOLVED IN: biological_process unknown; LOCATED IN: endomembrane system; BEST Arabidopsis thaliana protein match is: unknown protein (TAIR:AT2G12400.1)
AT3G51820	*1,347*	(G4)	Encodes a protein with chlorophyll synthase activity. This enzyme has been shown to perform the esterification of chlorophyllide (a and b), the last step of chlorophyll biosynthesis. Although it can use either geranylgeranyl pyrophosphate (GGPP) or phytyl pyrophosphate (PhyPP) as substrates, the esterification reaction was faster with GGPP than with PhyPP.
AT4G26890	*1,347*	MITOGEN-ACTIVATED PROTEIN KINASE KINASE KINASE 16 (MAPKKK16)	member of MEKK subfamily
AT5G55090	*1,343*	MITOGEN-ACTIVATED PROTEIN KINASE KINASE KINASE 15 (MAPKKK15)	member of MEKK subfamily
AT1G19850	*1,329*	MONOPTEROS (MP)	Encodes a transcription factor (IAA24) mediating embryo axis formation and vascular development. Similar to AUXIN RESPONSIVE FACTOR 1 (ARF1) shown to bind to auxin responsive elements (AREs), and to the maize transcriptional activator VIVIPAROUS 1(VP1). In situ hybridization shows expression in provascular tissue of embryos, the emerging shoot primordia, then is restricted to provascular tissue, and in the root central vascular cylinder.
AT2G26440	*1,328*	PECTIN METHYLESTERASE 12 (PME12)	Plant invertase/pectin methylesterase inhibitor superfamily; FUNCTIONS IN: enzyme inhibitor activity, pectinesterase activity; INVOLVED IN: cell wall modification; LOCATED IN: endomembrane system, cell wall, plant-type cell wall; EXPRESSED IN: 20 plant structures; EXPRESSED DURING: 13 growth stages; CONTAINS InterPro DOMAIN/s: Pectinesterase, active site (InterPro:IPR018040), Pectin lyase fold/virulence factor (InterPro:IPR011050), Pectinesterase, catalytic (InterPro:IPR000070), Pectinesterase inhibitor (InterPro:IPR006501), Pectin lyase fold (InterPro:IPR012334); BEST Arabidopsis thaliana protein match is: Plant invertase/pectin methylesterase inhibitor superfamily (TAIR:AT4G02330.1)
AT3G56090	*1,323*	FERRITIN 3 (FER3)	Encodes FERRITIN 3, AtFER3. Ferritins are a class of 24-mer multi-meric proteins found in all kingdoms of life. Function as the main iron store in mammals. Evidence suggests that Arabidopsis ferritins are essential to protect cells against oxidative damage, but they do not constitute the major iron pool.
AT2G37040	*1,32*	PHE AMMONIA LYASE 1 (pal1)	Encodes PAL1, a phenylalanine ammonia-lyase. Arabidopsis has four PALs: AT2G37040 (PAL1), AT3G53260 (PAL2), AT5G04230 (PAL3) and AT3G10340 (PAL4).
AT2G39705	*1,311*	ROTUNDIFOLIA LIKE 8 (RTFL8)	ROTUNDIFOLIA like 8 (RTFL8); FUNCTIONS IN: molecular_function unknown; INVOLVED IN: shoot development; LOCATED IN: chloroplast; EXPRESSED IN: 23 plant structures; EXPRESSED DURING: 13 growth stages; CONTAINS InterPro DOMAIN/s: DVL (InterPro:IPR012552); BEST Arabidopsis thaliana protein match is: ROTUNDIFOLIA like 7 (TAIR:AT3G55515.1)
AT5G67370	*1,311*	CONSERVED IN THE GREEN LINEAGE AND DIATOMS 27 (CGLD27)	Protein of unknown function (DUF1230); CONTAINS InterPro DOMAIN/s: Protein of unknown function DUF1230 (InterPro:IPR009631); BEST Arabidopsis thaliana protein match is: Protein of unknown function (DUF1230) (TAIR:AT5G11840.1)
AT3G06490	*1,31*	MYB DOMAIN PROTEIN 108 (MYB108)	putative transcription factor MYB108 (MYB108) mRNA,

AT5G18470	*1,309*		Curculin-like (mannose-binding) lectin family protein; FUNCTIONS IN: sugar binding; INVOLVED IN: response to karrikin; LOCATED IN: plant-type cell wall; EXPRESSED IN: 22 plant structures; EXPRESSED DURING: 12 growth stages; CONTAINS InterPro DOMAIN/s: Curculin-like (mannose-binding) lectin (InterPro:IPR001480); BEST Arabidopsis thaliana protein match is: lectin protein kinase family protein (TAIR:AT1G67520.1)
AT5G38310	*1,301*		unknown protein
AT1G72030	*1,3*		Acyl-CoA N-acyltransferases (NAT) superfamily protein; FUNCTIONS IN: N-acetyltransferase activity; INVOLVED IN: metabolic process; EXPRESSED IN: 22 plant structures; EXPRESSED DURING: 13 growth stages; CONTAINS InterPro DOMAIN/s: GCN5-related N-acetyltransferase, C-terminal (InterPro:IPR022610), GCN5-related N-acetyltransferase (InterPro:IPR000182), Acyl-CoA N-acyltransferase (InterPro:IPR016181)
AT1G69310	*1,299*	WRKY DNA-BINDING PROTEIN 57 (WRKY57)	Encodes WRKY57, a member of the WRKY Transcription Factor. Activation of WRKY57 confers drought tolerance.
AT4G15120	*1,299*		VQ motif-containing protein; CONTAINS InterPro DOMAIN/s: VQ (InterPro:IPR008889); BEST Arabidopsis thaliana protein match is: VQ motif-containing protein (TAIR:AT3G22160.1)
AT1G16850	*1,296*		unknown protein; FUNCTIONS IN: molecular_function unknown; INVOLVED IN: response to salt stress; LOCATED IN: endomembrane system; EXPRESSED IN: leaf apex, leaf whorl, male gametophyte, flower, leaf; EXPRESSED DURING: LP.06 six leaves visible, LP.04 four leaves visible, LP.10 ten leaves visible, petal differentiation and expansion stage, LP.08 eight leaves visible; BEST Arabidopsis thaliana protein match is: unknown protein (TAIR:AT5G64820.1)
AT4G24040	*1,296*	TREHALASE 1 (TRE1)	Encodes a trehalase, member of Glycoside Hydrolase Family 37.
AT1G67470	*1,295*		Protein kinase superfamily protein; FUNCTIONS IN: protein kinase activity, kinase activity, ATP binding; INVOLVED IN: protein amino acid phosphorylation; LOCATED IN: cellular_component unknown; EXPRESSED IN: 20 plant structures; EXPRESSED DURING: 13 growth stages; CONTAINS InterPro DOMAIN/s: Protein kinase, catalytic domain (InterPro:IPR000719), Serine/threonine-protein kinase-like domain (InterPro:IPR017442), Protein kinase-like domain (InterPro:IPR011009); BEST Arabidopsis thaliana protein match is: Protein kinase superfamily protein (TAIR:AT1G65250.1)
AT1G69840	*1,293*	HYPERSENSITIVE INDUCED REACTION 1 (ATHIR1)	SPFH/Band 7/PHB domain-containing membrane-associated protein family; INVOLVED IN: biological_process unknown; LOCATED IN: plasma membrane, chloroplast, vacuole; EXPRESSED IN: 26 plant structures; EXPRESSED DURING: 15 growth stages; CONTAINS InterPro DOMAIN/s: Band 7 protein (InterPro:IPR001107); BEST Arabidopsis thaliana protein match is: SPFH/Band 7/PHB domain-containing membrane-associated protein family (TAIR:AT5G62740.1)
AT3G22930	*1,288*	CALMODULIN-LIKE 11 (CML11)	Encodes a calmodulin-like protein.
AT4G32300	*1,288*	S-DOMAIN-2 5 (SD2-5)	S-domain-2 5 (SD2-5); FUNCTIONS IN: carbohydrate binding, protein kinase activity, kinase activity; INVOLVED IN: protein amino acid autophosphorylation; LOCATED IN: plasma membrane; EXPRESSED IN: 25 plant structures; EXPRESSED DURING: 15 growth stages; CONTAINS InterPro DOMAIN/s: Curculin-like (mannose-binding) lectin (InterPro:IPR001480), Protein kinase, ATP binding site (InterPro:IPR017441), Protein kinase, catalytic domain (InterPro:IPR000719), Serine/threonine-protein kinase-like domain (InterPro:IPR017442), Protein kinase-like domain (InterPro:IPR011009), Serine/threonine-protein kinase, active site (InterPro:IPR008271); BEST Arabidopsis thaliana protein match is: S-locus lectin protein kinase family protein (TAIR:AT5G35370.1)
AT4G37370	*1,288*	CYTOCHROME P450, FAMILY 81, SUBFAMILY D, POLYPEPTIDE 8 (CYP81D8)	member of CYP81D
AT4G27970	*1,287*	SLAC1 HOMOLOGUE 2 (SLAH2)	Encodes a protein with ten predicted transmembrane helices. The SLAH2 protein has similarity to the SLAC1 protein involved in ion homeostasis in guard cells. But, it is not expressed in guard cells and cannot complement a slac1-2 mutant suggesting that it performs a different function. SLAH2:GFP localizes to the plasma membrane.
AT5G65870	*1,286*	PHYTOSULFOKINE 5 PRECURSOR (PSK5)	Probable phytosulfokines 5 precursor, coding for a unique plant peptide growth factor.
AT1G52570	*1,285*	PHOSPHOLIPASE D ALPHA 2 (PLDALPHA2)	member of C2-PLD subfamily

AT1G11330	*1,283*		S-locus lectin protein kinase family protein; FUNCTIONS IN: in 6 functions; INVOLVED IN: protein amino acid phosphorylation, recognition of pollen; LOCATED IN: plasma membrane; EXPRESSED IN: 21 plant structures; EXPRESSED DURING: 13 growth stages; CONTAINS InterPro DOMAIN/s: Curculin-like (mannose-binding) lectin (InterPro:IPR001480), Apple-like (InterPro:IPR003609), PAN-2 domain (InterPro:IPR013227), Serine/threonine-protein kinase domain (InterPro:IPR002290), Serine/threonine-protein kinase-like domain (InterPro:IPR017442), Protein kinase-like domain (InterPro:IPR011009), Serine/threonine-protein kinase, active site (InterPro:IPR008271), Protein kinase, catalytic domain (InterPro:IPR000719), S-locus glycoprotein (InterPro:IPR000858), Tyrosine-protein kinase, catalytic domain (InterPro:IPR020635); BEST Arabidopsis thaliana protein match is: S-domain-1 13 (TAIR:AT1G11350.1).
AT3G21780	*1,282*	UDP-GLUCOSYL TRANSFERASE 71B6 (UGT71B6)	Encodes a protein with UDP-glucosyl transferase activity that was shown to preferentially glucosylates abscisic acid (ABA), and not its catabolites. Moreover, UGT71B6 was shown to have a strict preference for the naturally-occurring ABA enantiomer, (+)-ABA, and not its 'unnatural' relative, (-)-ABA. This is in contrast to the other identified UGT genes catalyzing the glucosylation of ABA which were shown to accept both stereoisomers as substrates.
AT5G24760	*1,281*		GroES-like zinc-binding dehydrogenase family protein; FUNCTIONS IN: oxidoreductase activity, zinc ion binding; INVOLVED IN: oxidation reduction; LOCATED IN: chloroplast; EXPRESSED IN: 22 plant structures; EXPRESSED DURING: 13 growth stages; CONTAINS InterPro DOMAIN/s: GroES-like (InterPro:IPR011032), Polyketide synthase, enoylreductase (InterPro:IPR020843), Alcohol dehydrogenase GroES-like (InterPro:IPR013154), Alcohol dehydrogenase, zinc-containing, conserved site (InterPro:IPR002328), Alcohol dehydrogenase, C-terminal (InterPro:IPR013149), Alcohol dehydrogenase superfamily, zinc-containing (InterPro:IPR002085); BEST Arabidopsis thaliana protein match is: alcohol dehydrogenase 1 (TAIR:AT1G77120.1)
AT1G11960	*1,278*		ERD (early-responsive to dehydration stress) family protein; FUNCTIONS IN: molecular_function unknown; INVOLVED IN: biological_process unknown; LOCATED IN: plasma membrane; EXPRESSED IN: 22 plant structures; EXPRESSED DURING: 13 growth stages; CONTAINS InterPro DOMAIN/s: Protein of unknown function DUF221 (InterPro:IPR003864); BEST Arabidopsis thaliana protein match is: ERD (early-responsive to dehydration stress) family protein (TAIR:AT1G62320.1)
AT3G26180	*1,274*	CYTOCHROME P450, FAMILY 71, SUBFAMILY B, POLYPEPTIDE 20 (CYP71B20)	putative cytochrome P450
AT2G32670	*1,269*	VESICLE-ASSOCIATED MEMBRANE PROTEIN 725 (VAMP725)	member of Synaptobrevin -like protein family
AT5G65600	*1,269*	L-TYPE LECTIN RECEPTOR KINASE IX.2 (LECRK-IX.2)	Concanavalin A-like lectin protein kinase family protein; FUNCTIONS IN: kinase activity; INVOLVED IN: protein amino acid phosphorylation; LOCATED IN: endomembrane system; EXPRESSED IN: stem; CONTAINS InterPro DOMAIN/s: Legume lectin, beta chain (InterPro:IPR001220), Protein kinase, ATP binding site (InterPro:IPR017441), Serine/threonine-protein kinase-like domain (InterPro:IPR017442), Concanavalin A-like lectin/glucanase, subgroup (InterPro:IPR013320), Protein kinase-like domain (InterPro:IPR011009), Serine/threonine-protein kinase, active site (InterPro:IPR008271), Protein kinase, catalytic domain (InterPro:IPR000719), Concanavalin A-like lectin/glucanase (InterPro:IPR008985); BEST Arabidopsis thaliana protein match is: Concanavalin A-like lectin protein kinase family protein (TAIR:AT5G10530.1)
AT5G17490	*1,268*	RGA-LIKE PROTEIN 3 (RGL3)	DELLA subfamily member involved in GA signal transduction
AT2G23120	*1,258*		Late embryogenesis abundant protein, group 6; FUNCTIONS IN: molecular_function unknown; INVOLVED IN: biological_process unknown; LOCATED IN: plasma membrane; EXPRESSED IN: 23 plant structures; EXPRESSED DURING: 14 growth stages; CONTAINS InterPro DOMAIN/s: Late embryogenesis abundant protein, group 6 (InterPro:IPR018930); BEST Arabidopsis thaliana protein match is: Late embryogenesis abundant protein, group 6 (TAIR:AT2G23110.2)

AT2G29360	*1,252*		NAD(P)-binding Rossmann-fold superfamily protein; FUNCTIONS IN: oxidoreductase activity, binding, catalytic activity; INVOLVED IN: oxidation reduction, metabolic process; LOCATED IN: cellular_component unknown; EXPRESSED IN: 22 plant structures; EXPRESSED DURING: 14 growth stages; CONTAINS InterPro DOMAIN/s: Short-chain dehydrogenase/reductase, conserved site (InterPro:IPR020904), NAD(P)-binding domain (InterPro:IPR016040), Glucose/ribitol dehydrogenase (InterPro:IPR002347), Short-chain dehydrogenase/reductase SDR (InterPro:IPR002198); BEST Arabidopsis thaliana protein match is: NAD(P)-binding Rossmann-fold superfamily protein (TAIR:AT2G29150.1)
AT2G28660	*1,25*		Chloroplast-targeted copper chaperone protein; FUNCTIONS IN: copper ion binding, metal ion binding; INVOLVED IN: copper ion transport, metal ion transport; LOCATED IN: chloroplast; EXPRESSED IN: 21 plant structures; EXPRESSED DURING: 13 growth stages; CONTAINS InterPro DOMAIN/s: Heavy metal transport/detoxification protein (InterPro:IPR006121), Copper chaperone, chloroplast-targeted, predicted (InterPro:IPR016578); BEST Arabidopsis thaliana protein match is: Chloroplast-targeted copper chaperone protein (TAIR:AT3G53530.1)
AT3G53260	*1,248*	PHENYLALANINE AMMONIA-LYASE 2 (PAL2)	Encodes phenylalanine lyase. Arabidopsis has four PALs: AT2G37040 (PAL1), AT3G53260 (PAL2), AT5G04230 (PAL3) and AT3G10340 (PAL4).
AT5G38520	*1,247*	CHLOROPHYLL DEPHYTYLASE1 (CLD1)	CLD1 is involved in steady-state chlorophyll turnover; CLD1 dephytylates chlorophyll a, chlorophyll b, and pheophytin a in vitro; CLD1 and CHLG form a salvage cycle in recycling chlorophyll. Suppression of CLD1 expression results in reduced tolerance to moderately high temperature.
AT1G02850	*1,245*	BETA GLUCOSIDASE 11 (BGLU11)	beta glucosidase 11 (BGLU11); FUNCTIONS IN: cation binding, hydrolase activity, hydrolyzing O-glycosyl compounds, catalytic activity; INVOLVED IN: carbohydrate metabolic process; LOCATED IN: endomembrane system; EXPRESSED IN: 24 plant structures; EXPRESSED DURING: 13 growth stages; CONTAINS InterPro DOMAIN/s: Glycoside hydrolase, family 1 (InterPro:IPR001360), Glycoside hydrolase, family 1, active site (InterPro:IPR018120), Glycoside hydrolase, catalytic core (InterPro:IPR017853), Glycoside hydrolase, subgroup, catalytic core (InterPro:IPR013781); BEST Arabidopsis thaliana protein match is: beta glucosidase 10 (TAIR:AT4G27830.1)
AT2G29440	*1,24*	GLUTATHIONE S-TRANSFERASE TAU 6 (GSTU6)	Encodes glutathione transferase belonging to the tau class of GSTs. Naming convention according to Wagner et al. (2002).
AT1G14600	*1,238*		Homeodomain-like superfamily protein; CONTAINS InterPro DOMAIN/s: Myb, DNA-binding (InterPro:IPR014778), Homeodomain-like (InterPro:IPR009057), Homeodomain-related (InterPro:IPR012287), HTH transcriptional regulator, Myb-type, DNA-binding (InterPro:IPR017930), Myb-like DNA-binding domain, SHAQKYF class (InterPro:IPR006447); BEST Arabidopsis thaliana protein match is: Homeodomain-like superfamily protein (TAIR:AT2G02060.1)
AT3G13090	*1,238*	ATP-BINDING CASSETTE C6 (ABCC6)	member of MRP subfamily
AT3G27650	*1,237*	LOB DOMAIN-CONTAINING PROTEIN 25 (LBD25)	LOB domain-containing protein 25 (LBD25); CONTAINS InterPro DOMAIN/s: Lateral organ boundaries, LOB (InterPro:IPR004883); BEST Arabidopsis thaliana protein match is: Lateral organ boundaries (LOB) domain family protein (TAIR:AT5G63090.4)
AT5G49280	*1,237*		hydroxyproline-rich glycoprotein family protein
AT1G68520	*1,234*	B-BOX DOMAIN PROTEIN 14 (BBX14)	B-box type zinc finger protein with CCT domain; FUNCTIONS IN: sequence-specific DNA binding transcription factor activity, zinc ion binding; INVOLVED IN: regulation of transcription; LOCATED IN: intracellular; EXPRESSED IN: 21 plant structures; EXPRESSED DURING: 13 growth stages; CONTAINS InterPro DOMAIN/s: CCT domain (InterPro:IPR010402), Zinc finger, B-box (InterPro:IPR000315); BEST Arabidopsis thaliana protein match is: B-box type zinc finger protein with CCT domain (TAIR:AT1G25440.1)
AT3G05320	*1,233*		O-fucosyltransferase family protein; CONTAINS InterPro DOMAIN/s: GDP-fucose protein O-fucosyltransferase (InterPro:IPR019378)
AT5G46730	*1,233*		glycine-rich protein; FUNCTIONS IN: molecular_function unknown; INVOLVED IN: biological_process unknown; LOCATED IN: endomembrane system; EXPRESSED IN: 21 plant structures; EXPRESSED DURING: 13 growth stages

AT2G02120	*1,232*	(PDF2.1)	Predicted to encode a PR (pathogenesis-related) protein. Belongs to the plant defensin (PDF) family with the following members: At1g75830/PDF1.1, At5g44420/PDF1.2a, At2g26020/PDF1.2b, At5g44430/PDF1.2c, At2g26010/PDF1.3, At1g19610/PDF1.4, At1g55010/PDF1.5, At2g02120/PDF2.1, At2g02100/PDF2.2, At2g02130/PDF2.3, At1g61070/PDF2.4, At5g63660/PDF2.5, At2g02140/PDF2.6, At5g38330/PDF3.1 and At4g30070/PDF3.2.
AT3G60140	*1,232*	DARK INDUCIBLE 2 (DIN2)	Encodes a protein similar to beta-glucosidase and is a member of glycoside hydrolase family 1. Expression is induced after 24 hours of dark treatment, in senescing leaves and treatment with exogenous photosynthesis inhibitor. Induction of gene expression was suppressed in excised leaves supplied with sugar. The authors suggest that the gene's expression pattern is responding to the level of sugar in the cell. The mRNA is cell-to-cell mobile.
AT5G01370	*1,232*	ALC-INTERACTING PROTEIN 1 (ACI1)	Nuclear protein with a lysine-rich domain and a C-terminal serine-rich domain. Interacts with Alcatraz (ALC). ACI1 is mainly expressed in the vascular system. Involved in cell separation during fruit dehiscence.
AT1G02930	*1,229*	GLUTATHIONE S-TRANSFERASE 6 (GSTF6)	Encodes glutathione transferase belonging to the phi class of GSTs. Naming convention according to Wagner et al. (2002).
AT3G51030	*1,228*	THIOREDOXIN H-TYPE 1 (TRX1)	encodes a cytosolic thioredoxin that reduces disulfide bridges of target proteins by the reversible formation of a disulfide bridge between two neighboring Cys residues present in the active site. Thioredoxins have been found to regulate a variety of biological reactions in prokaryotic and eukaryotic cells.
AT3G53160	*1,228*	UDP-GLUCOSYL TRANSFERASE 73C7 (UGT73C7)	UDP-glucosyl transferase 73C7 (UGT73C7); CONTAINS InterPro DOMAIN/s: UDP-glucuronosyl/UDP-glucosyltransferase (InterPro:IPR002213); BEST Arabidopsis thaliana protein match is: UDP-Glycosyltransferase superfamily protein (TAIR:AT2G36770.1)
AT3G46530	*1,226*	RECOGNITION OF PERONOSPORA PARASITICA 13 (RPP13)	Confers resistance to the biotrophic oomycete, Peronospora parasitica. Encodes an NBS-LRR type R protein with a putative amino-terminal leucine zipper. Fungal protein ATR13 induces RPP13 gene expression and disease resistance. The mRNA is cell-to-cell mobile.
AT3G20500	*1,223*	PURPLE ACID PHOSPHATASE 18 (PAP18)	purple acid phosphatase 18 (PAP18); FUNCTIONS IN: protein serine/threonine phosphatase activity, acid phosphatase activity; INVOLVED IN: biological_process unknown; LOCATED IN: endomembrane system; EXPRESSED IN: 24 plant structures; EXPRESSED DURING: 13 growth stages; CONTAINS InterPro DOMAIN/s: Metallophosphoesterase (InterPro:IPR004843), Purple acid phosphatase-like, N-terminal (InterPro:IPR008963); BEST Arabidopsis thaliana protein match is: purple acid phosphatase 22 (TAIR:AT3G52820.1)
AT5G53880	*1,217*		unknown protein; FUNCTIONS IN: molecular_function unknown; INVOLVED IN: biological_process unknown; LOCATED IN: mitochondrion, plasma membrane; EXPRESSED IN: 23 plant structures; EXPRESSED DURING: 13 growth stages
AT1G47960	*1,216*	CELL WALL / VACUOLAR INHIBITOR OF FRUCTOSIDASE 1 (C/VIF1)	Plant cell wall (CWI) and vacuolar invertases (VI) play important roles in carbohydrate metabolism, stress responses and sugar signaling. This protein may inhibit their activity.
AT1G63750	*1,215*		Disease resistance protein (TIR-NBS-LRR class) family; FUNCTIONS IN: transmembrane receptor activity, nucleoside-triphosphatase activity, nucleotide binding, ATP binding; INVOLVED IN: signal transduction, apoptosis, defense response, innate immune response; LOCATED IN: intrinsic to membrane; EXPRESSED IN: 7 plant structures; EXPRESSED DURING: 7 growth stages; CONTAINS InterPro DOMAIN/s: ATPase, AAA+ type, core (InterPro:IPR003593), NB-ARC (InterPro:IPR002182), Leucine-rich repeat (InterPro:IPR001611), Toll-Interleukin receptor (InterPro:IPR000157), Disease resistance protein (InterPro:IPR000767); BEST Arabidopsis thaliana protein match is: Disease resistance protein (TIR-NBS-LRR class) family (TAIR:AT5G58120.1)
AT4G19700	*1,215*	(RING)	Encodes BOI (Botrytis Susceptible 1 Interactor). Has E3 ubiquitin ligase activity. Interacts with and ubiquitinates BOS1 (Botrytis Susceptible 1). It prevents caspase activation and attenuates cell death.
AT3G49780	*1,213*	PHYTOSULFOKINE 4 PRECURSOR (PSK4)	Phytosulfokine 3 precursor, coding for a unique plant peptide growth factor. Plants overexpressing this gene (under a 35S promoter), develop normal cotyledons and hypocotyls but their growth, in particular that of their roots, was faster than that of wildtype.

AT1G77120	*1,212*	ALCOHOL DEHYDROGENASE 1 (ADH1)	Catalyzes the reduction of acetaldehyde using NADH as reductant. Requires zinc for activity. Dimer. Anaerobic response polypeptide (ANP). Fermentation. The protein undergoes thiolation following treatment with the oxidant tert-butylhydroperoxide. The mRNA is cell-to-cell mobile.
AT5G02750	*1,209*	SHOOT GRAVITROPISM 9 (SGR9)	Encodes an E3 ligase, SHOOT GRAVITROPISM9. Modulates the interaction between statoliths and F-Actin in gravity sensing.
AT5G16980	*1,208*		Zinc-binding dehydrogenase family protein; FUNCTIONS IN: oxidoreductase activity, binding, zinc ion binding, catalytic activity; INVOLVED IN: response to oxidative stress, response to cyclopentenone; EXPRESSED IN: 11 plant structures; EXPRESSED DURING: LP.06 six leaves visible, LP.04 four leaves visible, 4 anthesis, petal differentiation and expansion stage; CONTAINS InterPro DOMAIN/s: GroES-like (InterPro:IPR011032), Polyketide synthase, enoylreductase (InterPro:IPR020843), NAD(P)-binding domain (InterPro:IPR016040), Alcohol dehydrogenase, C-terminal (InterPro:IPR013149), Alcohol dehydrogenase superfamily, zinc-containing (InterPro:IPR002085); BEST Arabidopsis thaliana protein match is: alkenal reductase (TAIR:AT5G16970.1).
AT1G15960	*1,204*	NRAMP METAL ION TRANSPORTER 6 (NRAMP6)	member of Nramp2 family
AT4G39830	*1,204*		Cupredoxin superfamily protein; FUNCTIONS IN: oxidoreductase activity, L-ascorbate oxidase activity, copper ion binding; INVOLVED IN: oxidation reduction; LOCATED IN: endomembrane system, extracellular region; EXPRESSED IN: 19 plant structures; EXPRESSED DURING: 12 growth stages; CONTAINS InterPro DOMAIN/s: Multicopper oxidase, type 3 (InterPro:IPR011707), Multicopper oxidase, type 2 (InterPro:IPR011706), Cupredoxin (InterPro:IPR008972), Multicopper oxidase, copper-binding site (InterPro:IPR002355), Multicopper oxidase, type 1 (InterPro:IPR001117), L-ascorbate oxidase, plants (InterPro:IPR017760); BEST Arabidopsis thaliana protein match is: Plant L-ascorbate oxidase (TAIR:AT5G21100.1)
AT1G12900	*1,202*	GLYCERALDEHYDE 3-PHOSPHATE DEHYDROGENASE A SUBUNIT 2 (GAPA-2)	glyceraldehyde 3-phosphate dehydrogenase A subunit 2 (GAPA-2); FUNCTIONS IN: NAD or NADH binding, glyceraldehyde-3-phosphate dehydrogenase activity; INVOLVED IN: oxidation reduction, glycolysis, glucose metabolic process; LOCATED IN: apoplast, chloroplast stroma, chloroplast, membrane, chloroplast envelope; EXPRESSED IN: 24 plant structures; EXPRESSED DURING: 14 growth stages; CONTAINS InterPro DOMAIN/s: Glyceraldehyde 3-phosphate dehydrogenase subfamily (InterPro:IPR000173), Glyceraldehyde 3-phosphate dehydrogenase family (InterPro:IPR020831), Glyceraldehyde-3-phosphate dehydrogenase, type I (InterPro:IPR006424), Glyceraldehyde 3-phosphate dehydrogenase, active site (InterPro:IPR020830), Glyceraldehyde 3-phosphate dehydrogenase, catalytic domain (InterPro:IPR020829), Glyceraldehyde 3-phosphate dehydrogenase, catalytic domain, subgroup (InterPro:IPR020832), Glyceraldehyde 3-phosphate dehydrogenase, NAD(P) binding domain (InterPro:IPR020828); BEST Arabidopsis thaliana protein match is: glyceraldehyde 3-phosphate dehydrogenase A subunit (TAIR:AT3G26650.1)
AT5G13330	*1,202*	RELATED TO AP2 6L (Rap2.6L)	encodes a member of the ERF (ethylene response factor) subfamily B-4 of ERF/AP2 transcription factor family. The protein contains one AP2 domain. There are 7 members in this subfamily.
AT1G15980	*1,201*	PHOTOSYNTHETIC NDH SUBCOMPLEX B 1 (PnsB1)	encodes a novel subunit of the chloroplast NAD(P)H dehydrogenase complex, involved in cyclic electron flow around photosystem I to produce ATP.
AT5G16970	*1,197*	ALKENAL REDUCTASE (AER)	encodes a 2-alkenal reductase (EC 1.3.1.74), plays a key role in the detoxification of reactive carbonyls
AT1G71360	*1,195*	SUN-DOMAIN PROTEIN 4 (SUN4)	Encodes a member of the mid-SUN subfamily of SUN-domain proteins that is localized to both the nuclear envelope and the ER. It is involved in early seed development and nuclear morphology.
AT1G58360	*1,194*	AMINO ACID PERMEASE 1 (AAP1)	Encodes AAP1 (amino acid permease 1), a neutral amino acid transporter expressed in seeds. Functions in amino acid uptake into embryos. The transporter also functions in acquisition of glutamate and neutral amino acids by the root.
AT3G55950	*1,193*	CRINKLY4 RELATED 3 (CCR3)	CRINKLY4 related 3 (CCR3); FUNCTIONS IN: kinase activity; INVOLVED IN: protein amino acid phosphorylation; LOCATED IN: endomembrane system; EXPRESSED IN: 23 plant structures; EXPRESSED DURING: 10 growth stages; CONTAINS InterPro DOMAIN/s: Protein kinase, ATP binding site (InterPro:IPR017441), Regulator of chromosome condensation/beta-lactamase-inhibitor protein II (InterPro:IPR009091), Protein kinase, catalytic domain (InterPro:IPR000719), Serine/threonine-protein kinase-like domain (InterPro:IPR017442), Protein kinase-like domain (InterPro:IPR011009), Serine/threonine-protein kinase, active site (InterPro:IPR008271); BEST Arabidopsis thaliana protein match is: CRINKLY4 related 4 (TAIR:AT5G47850.1)

AT4G22590	*1,193*	TREHALOSE-6-PHOSPHATE PHOSPHATASE G (TPPG)	Haloacid dehalogenase-like hydrolase (HAD) superfamily protein; FUNCTIONS IN: catalytic activity, trehalose-phosphatase activity; INVOLVED IN: trehalose biosynthetic process, metabolic process; LOCATED IN: chloroplast; EXPRESSED IN: sperm cell, male gametophyte, pollen tube; EXPRESSED DURING: L mature pollen stage, M germinated pollen stage; CONTAINS InterPro DOMAIN/s: HAD-superfamily hydrolase, subfamily IIB (InterPro:IPR006379), Trehalose-phosphatase (InterPro:IPR003337); BEST Arabidopsis thaliana protein match is: Haloacid dehalogenase-like hydrolase (HAD) superfamily protein (TAIR:AT4G12430.1)
AT3G12145	*1,191*	FLOR1 (FLR1)	A novel leucine-rich repeat protein. Interacts directly with MADS domain transcription factor.
AT3G21240	*1,19*	4-COUMARATE:COA LIGASE 2 (4CL2)	encodes an isoform of 4-coumarate:CoA ligase (4CL), which is involved in the last step of the general phenylpropanoid pathway. The catalytic efficiency was in the following (descending) order: p-coumaric acid, caffeic acid, ferulic acid, 5-OH-ferulic acid and cinnamic acid. At4CL2 was unable to use sinapic acid as substrate.
AT3G22420	*1,189*	WITH NO LYSINE (K) KINASE 2 (WNK2)	Encodes a member of the WNK family (9 members in all) of protein kinases, the structural design of which is clearly distinct from those of other known protein kinases, such as receptor-like kinases and mitogen-activated protein kinases. Its transcription is under the control of circadian rhythms.
AT5G17850	*1,186*		Sodium/calcium exchanger family protein; FUNCTIONS IN: cation:cation antiporter activity, calcium:sodium antiporter activity; INVOLVED IN: cation transport, transmembrane transport; LOCATED IN: endomembrane system, integral to membrane; EXPRESSED IN: 19 plant structures; EXPRESSED DURING: 12 growth stages; CONTAINS InterPro DOMAIN/s: Sodium/calcium exchanger membrane region (InterPro:IPR004837); BEST Arabidopsis thaliana protein match is: calcium exchanger 7 (TAIR:AT5G17860.1)
AT5G20420	*1,186*	CHROMATIN REMODELING 42 (CHR42)	chromatin remodeling 42 (CHR42); FUNCTIONS IN: helicase activity, DNA binding, ATP binding, nucleic acid binding; EXPRESSED IN: shoot apex, embryo, flower, seed; EXPRESSED DURING: F mature embryo stage, petal differentiation and expansion stage, E expanded cotyledon stage, D bilateral stage; CONTAINS InterPro DOMAIN/s: DEAD-like helicase, N-terminal (InterPro:IPR014001), DNA/RNA helicase, C-terminal (InterPro:IPR001650), Helicase, superfamily 1/2, ATP-binding domain (InterPro:IPR014021), SNF2-related (InterPro:IPR000330); BEST Arabidopsis thaliana protein match is: chromatin remodeling 38 (TAIR:AT3G42670.1)
AT2G02060	*1,184*		Homeodomain-like superfamily protein; CONTAINS InterPro DOMAIN/s: Homeodomain-like (InterPro:IPR009057), Myb, DNA-binding (InterPro:IPR014778), HTH transcriptional regulator, Myb-type, DNA-binding (InterPro:IPR017930), Myb-like DNA-binding domain, SHAQKYF class (InterPro:IPR006447), Homeodomain-related (InterPro:IPR012287); BEST Arabidopsis thaliana protein match is: Homeodomain-like superfamily protein (TAIR:AT1G14600.1)
AT1G69910	*1,179*		Protein kinase superfamily protein; FUNCTIONS IN: kinase activity; INVOLVED IN: protein amino acid phosphorylation; EXPRESSED IN: 20 plant structures; EXPRESSED DURING: 13 growth stages; CONTAINS InterPro DOMAIN/s: Protein kinase, ATP binding site (InterPro:IPR017441), Protein kinase, catalytic domain (InterPro:IPR000719), Serine/threonine-protein kinase domain (InterPro:IPR002290), Tyrosine-protein kinase, active site (InterPro:IPR008266), Tyrosine-protein kinase, catalytic domain (InterPro:IPR020635), Serine/threonine-protein kinase-like domain (InterPro:IPR017442), Protein kinase-like domain (InterPro:IPR011009); BEST Arabidopsis thaliana protein match is: Protein kinase superfamily protein (TAIR:AT1G18390.2)
AT1G17100	*1,177*	HAEM-BINDING PROTEIN 1 (HBP1)	SOUL heme-binding family protein; FUNCTIONS IN: binding; INVOLVED IN: biological_process unknown; LOCATED IN: plasma membrane, vacuole; EXPRESSED IN: 24 plant structures; EXPRESSED DURING: 14 growth stages; CONTAINS InterPro DOMAIN/s: SOUL haem-binding protein (InterPro:IPR006917); BEST Arabidopsis thaliana protein match is: SOUL heme-binding family protein (TAIR:AT1G78450.1)
AT3G61890	*1,177*	HOMEOBOX 12 (HB-12)	Encodes a homeodomain leucine zipper class I (HD-Zip I) protein. Loss of function mutant has abnormally shaped leaves and stems.
AT5G48930	*1,176*	HYDROXYCINNAMOYL-COA SHIKIMATE/QUINATE HYDROXYCINNAMOYL TRANSFERASE (HCT)	At5g48930 has been shown to encode for the hydroxycinnamoyl-Coenzyme A shikimate/quinate hydroxycinnamoyltransferase (HCT) both synthesizing and catabolizing the hydroxycinnamoylesters (coumaroyl/caffeoyl shikimate and quinate) involved in the phenylpropanoid pathway. Influence on the accumulation of flavonoids which in turn inhibit auxin transport and reduce plant growth. The mRNA is cell-to-cell mobile.

AT5G20190	*1,175*		Tetratricopeptide repeat (TPR)-like superfamily protein; FUNCTIONS IN: binding; INVOLVED IN: biological_process unknown; LOCATED IN: chloroplast; CONTAINS InterPro DOMAIN/s: Tetratricopeptide-like helical (InterPro:IPR011990), Tetratricopeptide repeat-containing (InterPro:IPR013026); BEST Arabidopsis thaliana protein match is: Tetratricopeptide repeat (TPR)-like superfamily protein (TAIR:AT1G80130.1)
AT3G44720	*1,174*	AROGENATE DEHYDRATASE 4 (ADT4)	Encodes a plastid-localized arogenate dehydratase involved in phenylalanine biosynthesis. Not less than six genes encoding ADT were identiﬁed in the Arabidopsis genome: ADT1 [At1g11790]; ADT2 [At3g07630]; ADT3 [At2g27820]; ADT4 [At3g44720]; ADT5 [At5g22630]; and ADT6 [At1g08250]. The mRNA is cell-to-cell mobile.
AT4G21680	*1,174*	NRT1/ PTR FAMILY 7.2 (NPF7.2)	Encodes a nitrate transporter (NRT1.8). Functions in nitrate removal from the xylem sap. Mediates cadmium tolerance.
AT2G05440	*1,169*	GLYCINE RICH PROTEIN 9 (GRP9)	GLYCINE RICH PROTEIN 9 (GRP9); FUNCTIONS IN: molecular_function unknown; INVOLVED IN: biological_process unknown; LOCATED IN: endomembrane system; CONTAINS InterPro DOMAIN/s: Glycine rich protein (InterPro:IPR010800); BEST Arabidopsis thaliana protein match is: Glycine-rich protein family (TAIR:AT2G05510.6)
AT1G44350	*1,161*	IAA-LEUCINE RESISTANT (ILR)-LIKE GENE 6 (ILL6)	encodes a protein similar to IAA amino acid conjugate hydrolase.
AT4G24830	*1,159*		arginosuccinate synthase family; FUNCTIONS IN: argininosuccinate synthase activity, ATP binding; INVOLVED IN: arginine biosynthetic process; LOCATED IN: chloroplast stroma, chloroplast; EXPRESSED IN: 25 plant structures; EXPRESSED DURING: 15 growth stages; CONTAINS InterPro DOMAIN/s: Rossmann-like alpha/beta/alpha sandwich fold (InterPro:IPR014729), Argininosuccinate synthase, conserved site (InterPro:IPR018223), Argininosuccinate synthase (InterPro:IPR001518)
AT2G39210	*1,157*		Major facilitator superfamily protein; FUNCTIONS IN: molecular_function unknown; INVOLVED IN: transmembrane transport; LOCATED IN: cellular_component unknown; EXPRESSED IN: 22 plant structures; EXPRESSED DURING: 13 growth stages; CONTAINS InterPro DOMAIN/s: Nodulin-like (InterPro:IPR010658), Major facilitator superfamily MFS-1 (InterPro:IPR011701), Major facilitator superfamily, general substrate transporter (InterPro:IPR016196); BEST Arabidopsis thaliana protein match is: Major facilitator superfamily protein (TAIR:AT2G28120.1)
AT1G75060	*1,154*		unknown protein; BEST Arabidopsis thaliana protein match is: unknown protein (TAIR:AT1G19330.2)
AT5G65990	*1,153*	(ATAVT3)	Transmembrane amino acid transporter family protein; CONTAINS InterPro DOMAIN/s: Amino acid transporter, transmembrane (InterPro:IPR013057); BEST Arabidopsis thaliana protein match is: Transmembrane amino acid transporter family protein (TAIR:AT2G42005.1)
AT5G12010	*1,148*		unknown protein; INVOLVED IN: response to salt stress; LOCATED IN: chloroplast, plasma membrane, membrane; EXPRESSED IN: 23 plant structures; EXPRESSED DURING: 13 growth stages; BEST Arabidopsis thaliana protein match is: unknown protein (TAIR:AT4G29780.1)
AT4G04570	*1,145*	CYSTEINE-RICH RLK (RECEPTOR-LIKE PROTEIN KINASE) 40 (CRK40)	Encodes a cysteine-rich receptor-like protein kinase. The mRNA is cell-to-cell mobile.
AT2G34510	*1,143*		FUNCTIONS IN: molecular_function unknown; INVOLVED IN: biological_process unknown; LOCATED IN: anchored to membrane; EXPRESSED IN: 20 plant structures; EXPRESSED DURING: 14 growth stages; CONTAINS InterPro DOMAIN/s: Protein of unknown function DUF642 (InterPro:IPR006946), Galactose-binding domain-like (InterPro:IPR008979); BEST Arabidopsis thaliana protein match is: Protein of unknown function, DUF642 (TAIR:AT1G29980.1)
AT2G46110	*1,137*	KETOPANTOATE HYDROXYMETHYLTRANSFERAS E 1 (KPHMT1)	Encodes a ketopentoate hydroxymethyltransferase that appears to localize to the mitochondria. This protein is expected to play a role in pantothenate (vitamin B5) biosynthesis.
AT3G07390	*1,137*	AUXIN-INDUCED IN ROOT CULTURES 12 (AIR12)	isolated from differential screening of a cDNA library from auxin-treated root culture. sequence does not show homology to any known proteins and is predicted to be extracellular. The mRNA is cell-to-cell mobile.
AT3G26980	*1,137*	MEMBRANE-ANCHORED UBIQUITIN-FOLD PROTEIN 4 PRECURSOR (MUB4)	membrane-anchored ubiquitin-fold protein 4 precursor (MUB4); CONTAINS InterPro DOMAIN/s: Membrane-anchored ubiquitin-fold protein, HCG-1 (InterPro:IPR017000), Ubiquitin (InterPro:IPR000626), Ubiquitin supergroup (InterPro:IPR019955); BEST Arabidopsis thaliana protein match is: Ubiquitin family protein (TAIR:AT4G24990.1)

AT1G01140	*1,136*	CBL-INTERACTING PROTEIN KINASE 9 (CIPK9)	Encodes a CBL-interacting protein kinase with similarity to SOS2
AT5G49620	*1,136*	MYB DOMAIN PROTEIN 78 (MYB78)	Member of the R2R3 factor gene family.
AT4G01700	*1,133*		Chitinase family protein; FUNCTIONS IN: chitinase activity; INVOLVED IN: carbohydrate metabolic process, cell wall macromolecule catabolic process; LOCATED IN: cell wall; EXPRESSED IN: 18 plant structures; EXPRESSED DURING: 13 growth stages; CONTAINS InterPro DOMAIN/s: Glycoside hydrolase, family 19 (InterPro:IPR016283), Glycoside hydrolase, family 19, catalytic (InterPro:IPR000726); BEST Arabidopsis thaliana protein match is: Chitinase family protein (TAIR:AT1G02360.1)
AT1G79900	*1,132*	(BAC2)	encodes a mitochondrial ornithine transporter that exports ornithine from the mitochondria to the cytosol
AT1G09420	*1,131*	GLUCOSE-6-PHOSPHATE DEHYDROGENASE 4 (G6PD4)	Encodes a protein similar to glucose-6-phosphate dehydrogenase but, based on amino acid differences in the active site and lack of activity, does not encode a functional G6PDH. The amino acid sequence for the consensus sequence of the G6PDH active site (DHYLGKE) differs in three places in this protein. gc exon splice site at 20574 is based on protein alignment, and is not confirmed experimentally.
AT1G67090	*1,129*	RIBULOSE BISPHOSPHATE CARBOXYLASE SMALL CHAIN 1A (RBCS1A)	Encodes a member of the Rubisco small subunit (RBCS) multigene family: RBCS1A (At1g67090), RBCS1B (At5g38430), RBCS2B (At5g38420), and RBCS3B (At5g38410). Functions to yield sufficient Rubisco content for leaf photosynthetic capacity.
AT1G68470	*1,128*		Exostosin family protein; FUNCTIONS IN: catalytic activity; INVOLVED IN: biological_process unknown; LOCATED IN: membrane; EXPRESSED IN: 18 plant structures; EXPRESSED DURING: 13 growth stages; CONTAINS InterPro DOMAIN/s: Exostosin-like (InterPro:IPR004263); BEST Arabidopsis thaliana protein match is: Exostosin family protein (TAIR:AT2G20370.1)
AT1G69520	*1,128*		S-adenosyl-L-methionine-dependent methyltransferases superfamily protein; FUNCTIONS IN: methyltransferase activity; INVOLVED IN: metabolic process; LOCATED IN: mitochondrion; CONTAINS InterPro DOMAIN/s: Methyltransferase type 11 (InterPro:IPR013216); BEST Arabidopsis thaliana protein match is: S-adenosyl-L-methionine-dependent methyltransferases superfamily protein (TAIR:AT1G69526.2)
AT3G29810	*1,128*	COBRA-LIKE PROTEIN 2 PRECURSOR (COBL2)	During the course of seed coat epidermal cell differentiation, COBRA-LIKE 2 plays a role in cellulose deposition into mucilage secretory cells of Arabidopsis seeds. COBRA-LIKE 2 affects mucilage solubility and cellulosic ray formation.
AT4G18910	*1,128*	NOD26-LIKE INTRINSIC PROTEIN 1;2 (NIP1;2)	Encodes an aquaporin homolog. Functions in arsenite transport and tolerance.When expressed in yeast cells can conduct hydrogen peroxide into those cells.
AT4G34710	*1,126*	ARGININE DECARBOXYLASE 2 (ADC2)	Encodes a arginine decarboxylase (ADC), a rate-limiting enzyme that catalyzes the first step of polyamine (PA) biosynthesis via ADC pathway in Arabidopsis thaliana. Arabidopsis genome has two ADC paralogs, ADC1 and ADC2. ADC2 is stress-inducible (osmotic stress). Double mutant analysis showed that ADC genes are essential for the production of PA, and are required for normal seed development. Overexpression causes phenotypes similar to GA-deficient plants and these plants show reduced levels of GA due to lower expression levels of AtGA20ox1, AtGA3ox3 and AtGA3ox1.
AT1G60870	*1,125*	MATERNAL EFFECT EMBRYO ARREST 9 (MEE9)	maternal effect embryo arrest 9 (MEE9); FUNCTIONS IN: molecular_function unknown; INVOLVED IN: embryo development ending in seed dormancy, pollen development; LOCATED IN: plasma membrane; EXPRESSED IN: 22 plant structures; EXPRESSED DURING: 13 growth stages
AT2G30550	*1,124*	DAD1-LIKE LIPASE 3 (DALL3)	Encodes a lipase that hydrolyzes phosphatidylcholine, glycolipids as well as triacylglycerols.
AT1G67560	*1,123*	LIPOXYGENASE 6 (LOX6)	PLAT/LH2 domain-containing lipoxygenase family protein; FUNCTIONS IN: oxidoreductase activity, acting on single donors with incorporation of molecular oxygen, incorporation of two atoms of oxygen, lipoxygenase activity, iron ion binding, metal ion binding; INVOLVED IN: oxidation reduction, growth; LOCATED IN: chloroplast; EXPRESSED IN: 22 plant structures; EXPRESSED DURING: 13 growth stages; CONTAINS InterPro DOMAIN/s: Lipoxygenase, iron binding site (InterPro:IPR020833), Lipoxygenase, C-terminal (InterPro:IPR013819), Lipoxygenase, LH2 (InterPro:IPR001024), Lipase/lipooxygenase, PLAT/LH2 (InterPro:IPR008976), Lipoxygenase, conserved site (InterPro:IPR020834), Lipoxygenase (InterPro:IPR000907), Lipoxygenase, plant (InterPro:IPR001246); BEST Arabidopsis thaliana protein match is: PLAT/LH2 domain-containing lipoxygenase family protein (TAIR:AT1G72520.1)
AT5G16990	*1,123*		molecular function has not been defined, was shown involved in oxidative stress tolerance. The mRNA is cell-to-cell mobile.

AT1G73110	*1,12*		P-loop containing nucleoside triphosphate hydrolases superfamily protein; FUNCTIONS IN: ATPase activity, ATP binding; LOCATED IN: chloroplast thylakoid membrane, chloroplast; EXPRESSED IN: 21 plant structures; EXPRESSED DURING: 13 growth stages; CONTAINS InterPro DOMAIN/s: ATPase, AAA-type, core (InterPro:IPR003959); BEST Arabidopsis thaliana protein match is: rubisco activase (TAIR:AT2G39730.1)
AT5G54170	*1,119*		Polyketide cyclase/dehydrase and lipid transport superfamily protein; CONTAINS InterPro DOMAIN/s: Lipid-binding START (InterPro:IPR002913); BEST Arabidopsis thaliana protein match is: Polyketide cyclase/dehydrase and lipid transport superfamily protein (TAIR:AT1G64720.1)
AT5G65470	*1,112*		O-fucosyltransferase family protein; CONTAINS InterPro DOMAIN/s: GDP-fucose protein O-fucosyltransferase (InterPro:IPR019378); BEST Arabidopsis thaliana protein match is: O-fucosyltransferase family protein (TAIR:AT4G24530.1)
AT1G68360	*1,111*	GLABROUS INFLORESCENCE STEMS 3 (GIS3)	Encodes a nuclear localized member of the C2H2 family of TFIIIA transcription factors.GIS3 is involved in trichome initiation and development downstream of GA and cytokinin signaling. GIS regulates the expression GIS and GIS2.
AT4G30980	*1,11*	LJRHL1-LIKE 2 (LRL2)	Encodes a basic helix-loop-helix (bHLH) protein that regulates root hair development. One of the three Arabidopsis homologs of the Lotus japonicus ROOTHAIRLESS1 (LjRHL1) gene: At2g24260 (AtLRL1), At4g30980 (AtLRL2), and At5g58010 (AtLRL3).
AT2G36080	*1,109*	ABNORMAL SHOOT 2 (ABS2)	Encodes a plant-specific B3 DNA-binding domain transcription factor. Has transcription repressor activity.
AT4G34220	*1,106*	RECEPTOR DEAD KINASE1 (RDK1)	Encodes a receptor like kinase involved in ABA-mediated seedling development and drought tolerance.RDK1 is an atypical or pseudokinase and has no phosphorylation activity. Its expression is upregulated in response to ABA.interacts with ABI1 and other PP2C phosphatases.
AT2G44480	*1,104*	BETA GLUCOSIDASE 17 (BGLU17)	beta glucosidase 17 (BGLU17); FUNCTIONS IN: cation binding, hydrolase activity, hydrolyzing O-glycosyl compounds, catalytic activity; INVOLVED IN: carbohydrate metabolic process; LOCATED IN: endomembrane system; EXPRESSED IN: stem, hypocotyl, sepal, male gametophyte, root; EXPRESSED DURING: 4 anthesis; CONTAINS InterPro DOMAIN/s: Glycoside hydrolase, family 1 (InterPro:IPR001360), Glycoside hydrolase, family 1, active site (InterPro:IPR018120), Glycoside hydrolase, catalytic core (InterPro:IPR017853), Glycoside hydrolase, subgroup, catalytic core (InterPro:IPR013781); BEST Arabidopsis thaliana protein match is: beta glucosidase 15 (TAIR:AT2G44450.1)
AT5G09660	*1,104*	PEROXISOMAL NAD-MALATE DEHYDROGENASE 2 (PMDH2)	encodes a microbody NAD-dependent malate dehydrogenase encodes an peroxisomal NAD-malate dehydrogenase that is involved in fatty acid beta-oxidation through providing NAD to the process of converting fatty acyl CoA to acetyl CoA.
AT4G21326	*1,102*	SUBTILASE 3.12 (SBT3.12)	subtilase 3.12 (SBT3.12); FUNCTIONS IN: identical protein binding, serine-type endopeptidase activity; INVOLVED IN: proteolysis, negative regulation of catalytic activity; LOCATED IN: endomembrane system; EXPRESSED IN: 17 plant structures; EXPRESSED DURING: 8 growth stages; CONTAINS InterPro DOMAIN/s: Peptidase S8/S53, subtilisin/kexin/sedolisin (InterPro:IPR000209), Peptidase S8, subtilisin-related (InterPro:IPR015500), Peptidase S8/S53, subtilisin, active site (InterPro:IPR022398), Proteinase inhibitor I9, subtilisin propeptide (InterPro:IPR010259); BEST Arabidopsis thaliana protein match is: Subtilase family protein (TAIR:AT4G10550.1)
AT2G28900	*1,096*	OUTER PLASTID ENVELOPE PROTEIN 16-1 (OEP16-1)	Encodes AtOEP16, a 16-KDa plastid outer membrane protein involved in plastid import of protochlorophyllide oxidoreductase A. Predominantly expressed in leaves and is also inducible by cold treatment.
AT5G08380	*1,094*	ALPHA-GALACTOSIDASE 1 (AGAL1)	alpha-galactosidase 1 (AGAL1); FUNCTIONS IN: alpha-galactosidase activity, hydrolase activity, hydrolyzing O-glycosyl compounds, catalytic activity; INVOLVED IN: carbohydrate metabolic process, metabolic process, lactose catabolic process; LOCATED IN: apoplast, cell wall, plant-type cell wall; EXPRESSED IN: 21 plant structures; EXPRESSED DURING: 13 growth stages; CONTAINS InterPro DOMAIN/s: Glycoside hydrolase, family 27 (InterPro:IPR002241), Aldolase-type TIM barrel (InterPro:IPR013785), Glycoside hydrolase, clan GH-D (InterPro:IPR000111), Glycoside hydrolase, catalytic core (InterPro:IPR017853); BEST Arabidopsis thaliana protein match is: alpha-galactosidase 2 (TAIR:AT5G08370.1)

AT2G19400	*1,092*		AGC (cAMP-dependent, cGMP-dependent and protein kinase C) kinase family protein; FUNCTIONS IN: kinase activity; INVOLVED IN: protein amino acid phosphorylation; LOCATED IN: cellular_component unknown; EXPRESSED IN: 9 plant structures; EXPRESSED DURING: 6 growth stages; CONTAINS InterPro DOMAIN/s: Protein kinase, ATP binding site (InterPro:IPR017441), Serine/threonine-protein kinase domain (InterPro:IPR002290), Serine/threonine-protein kinase-like domain (InterPro:IPR017442), Protein kinase, C-terminal (InterPro:IPR017892), Protein kinase-like domain (InterPro:IPR011009), Serine/threonine-protein kinase, active site (InterPro:IPR008271), AGC-kinase, C-terminal (InterPro:IPR000961), Protein kinase, catalytic domain (InterPro:IPR000719); BEST Arabidopsis thaliana protein match is: AGC (cAMP-dependent, cGMP-dependent and protein kinase C) kinase family protein (TAIR:AT4G33080.1)
AT3G52340	*1,092*	SUCROSE-6F-PHOSPHATE PHOSPHOHYDROLASE 2 (SPP2)	sucrose-phosphatase (SPP2)
AT4G33270	*1,092*	CELL DIVISION CYCLE 20.1 (CDC20.1)	Encodes a CDC20 protein that interacts with APC subunits, components of the mitochondrial checkpoint complex and mitotic cyclin substrates and is indispensable for normal plant development and fertility.
AT2G40890	*1,091*	CYTOCHROME P450, FAMILY 98, SUBFAMILY A, POLYPEPTIDE 3 (CYP98A3)	encodes coumarate 3-hydroxylase (C3H), a P450-dependent monooxygenase. Involved in lignin biosynthesis and flavonoid biosynthesis. Also affects the biosynthesis of coumarins such as scopoletin and scopolin as a branching-out-pathway from the phenylpropanoid acid level.
AT3G28930	*1,091*	AVRRPT2-INDUCED GENE 2 (AIG2)	avrRpt2-induced gene that exhibits RPS2- and avrRpt2-dependent induction early after infection with Pseudomonas syringae pv maculicola strain ES4326 carrying avrRpt2
AT3G29200	*1,091*	CHORISMATE MUTASE 1 (CM1)	L-ascorbate peroxidase
AT1G78070	*1,09*		Transducin/WD40 repeat-like superfamily protein; CONTAINS InterPro DOMAIN/s: WD40 repeat 2 (InterPro:IPR019782), WD40 repeat-like-containing domain (InterPro:IPR011046), WD40 repeat, conserved site (InterPro:IPR019775), WD40-repeat-containing domain (InterPro:IPR017986), WD40/YVTN repeat-like-containing domain (InterPro:IPR015943), WD40 repeat (InterPro:IPR001680), WD40 repeat, subgroup (InterPro:IPR019781); BEST Arabidopsis thaliana protein match is: Transducin/WD40 repeat-like superfamily protein (TAIR:AT1G36070.1)
AT5G62620	*1,082*	(GALT6)	Encodes a Golgi-localized hydroxyproline-O-galactosyltransferase. Mutants display multiple phenotypes including reduced seed coat mucilage and accelerated leaf senescence.
AT1G54200	*1,081*		unknown protein; BEST Arabidopsis thaliana protein match is: unknown protein (TAIR:AT3G13980.1)
AT4G34480	*1,078*		O-Glycosyl hydrolases family 17 protein; FUNCTIONS IN: cation binding, hydrolase activity, hydrolyzing O-glycosyl compounds, catalytic activity; INVOLVED IN: carbohydrate metabolic process; LOCATED IN: endomembrane system; EXPRESSED IN: 22 plant structures; EXPRESSED DURING: 13 growth stages; CONTAINS InterPro DOMAIN/s: X8 (InterPro:IPR012946), Glycoside hydrolase, catalytic core (InterPro:IPR017853), Glycoside hydrolase, family 17 (InterPro:IPR000490), Glycoside hydrolase, subgroup, catalytic core (InterPro:IPR013781); BEST Arabidopsis thaliana protein match is: O-Glycosyl hydrolases family 17 protein (TAIR:AT2G16230.1)
AT2G47800	*1,071*	ATP-BINDING CASSETTE C4 (ABCC4)	Encodes a plasma membrane localized ATPase transporter involved in multidrug transport. The expression of this gene is upregulated by herbicide safeners such as benoxacor, fluxofenim and fenclorim. The mRNA is cell-to-cell mobile.
AT5G48450	*1,069*	SKU5 SIMILAR 3 (sks3)	Encodes a protein with two DUF26 domains and a signal peptide for secretion. The protein is transported to the apoplast when it is expressed as a GFP fusion protein.
AT5G15850	*1,066*	CONSTANS-LIKE 1 (COL1)	Homologous to the flowering-time gene CONSTANS.
AT5G22630	*1,063*	AROGENATE DEHYDRATASE 5 (ADT5)	Encodes a plastid-localized arogenate dehydratase involved in phenylalanine biosynthesis. Not less than six genes encoding ADT were identiﬁed in the Arabidopsis genome: ADT1 [At1g11790]; ADT2 [At3g07630]; ADT3 [At2g27820]; ADT4 [At3g44720]; ADT5 [At5g22630]; and ADT6 [At1g08250]. The mRNA is cell-to-cell mobile.
AT1G66480	*1,06*		Involved in chloroplast avoidance movement under intermediate and high light intensities
AT5G12890	*1,06*		UDP-Glycosyltransferase superfamily protein; FUNCTIONS IN: UDP-glycosyltransferase activity, transferase activity, transferring glycosyl groups; INVOLVED IN: metabolic process; EXPRESSED IN: 22 plant structures; EXPRESSED DURING: 12 growth stages; CONTAINS InterPro DOMAIN/s: UDP-glucuronosyl/UDP-glucosyltransferase (InterPro:IPR002213); BEST Arabidopsis thaliana protein match is: UDP-Glycosyltransferase superfamily protein (TAIR:AT2G36780.1)

AT4G38780	*1,055*		Pre-mRNA-processing-splicing factor; FUNCTIONS IN: molecular_function unknown; INVOLVED IN: nuclear mRNA splicing, via spliceosome; LOCATED IN: spliceosomal complex; EXPRESSED IN: 19 plant structures; EXPRESSED DURING: 8 growth stages; CONTAINS InterPro DOMAIN/s: Mov34/MPN/PAD-1 (InterPro:IPR000555), Pre-mRNA-processing-splicing factor 8, U5-snRNA-binding (InterPro:IPR019581), Pre-mRNA-processing-splicing factor 8 (InterPro:IPR012591), Pre-mRNA-processing-splicing factor 8, U6-snRNA-binding (InterPro:IPR019580), PROCN (InterPro:IPR012592), PRP8 domain IV core (InterPro:IPR021983), PRO, C-terminal (InterPro:IPR012984), RNA recognition motif, spliceosomal PrP8 (InterPro:IPR019582); BEST Arabidopsis thaliana protein match is: Pre-mRNA-processing-splicing factor (TAIR:AT1G80070.1)
AT5G24010	*1,055*		Protein kinase superfamily protein; FUNCTIONS IN: protein serine/threonine kinase activity, protein kinase activity, kinase activity, ATP binding; INVOLVED IN: protein amino acid phosphorylation; LOCATED IN: plasma membrane; EXPRESSED IN: 23 plant structures; EXPRESSED DURING: 13 growth stages; CONTAINS InterPro DOMAIN/s: Protein kinase, ATP binding site (InterPro:IPR017441), Protein kinase, catalytic domain (InterPro:IPR000719), Serine-threonine/tyrosine-protein kinase (InterPro:IPR001245), Protein kinase-like domain (InterPro:IPR011009), Serine/threonine-protein kinase, active site (InterPro:IPR008271); BEST Arabidopsis thaliana protein match is: hercules receptor kinase 1 (TAIR:AT3G46290.1)
AT3G26650	*1,053*	GLYCERALDEHYDE 3-PHOSPHATE DEHYDROGENASE A SUBUNIT (GAPA)	Encodes one of the two subunits forming the photosynthetic glyceraldehyde-3-phosphate dehydrogenase (GAPDH) and as such a constituent of the supramolecular complex with phosphoribulokinase (PRK) thought to be linked by a small peptide encoded by CP12-2. GapA-1 is coordinately expressed by light with PRK and CP12-2. The enzyme activity, tested in leaf protein extracts dropped significantly after external sucrose treatment for the photosynthetic GAPDH (NADPH-dependent) but not for the cytosolic GAPDH (NADH-dependent).
AT5G06860	*1,049*	POLYGALACTURONASE INHIBITING PROTEIN 1 (PGIP1)	Encodes a polygalacturonase inhibiting protein involved in defense response. PGIPs inhibit the function of cell wall pectin degrading enzymes such as those produced by fungal pathogens. PGIP1 is induced by fungal infection. Suppressed in the proton sensitive stop1-mutant, but the transcription level was recovered by transformation of STOP2. Knockout mutant showed severe damage in the root tip in low Ca and low pH medium.
AT1G73020	*1,046*		unknown protein; CONTAINS InterPro DOMAIN/s: Protein of unknown function DUF590 (InterPro:IPR007632)
AT5G62940	*1,045*	HIGH CAMBIAL ACTIVITY2 (HCA2)	HCA2 induces the formation of interfascicular cambium and regulates vascular tissue development in the aerial parts of the plant. Evidence from both gain of function and dominant negative alleles.
AT1G68010	*1,042*	HYDROXYPYRUVATE REDUCTASE (HPR)	Encodes hydroxypyruvate reductase.
AT2G38230	*1,041*	PYRIDOXINE BIOSYNTHESIS 1.1 (PDX1.1)	Encodes a protein predicted to function in tandem with PDX2 to form glutamine amidotransferase complex with involved in vitamin B6 biosynthesis. The mRNA is cell-to-cell mobile.
AT3G50410	*1,031*	OBF BINDING PROTEIN 1 (OBP1)	Arabidopsis Dof protein containing a single 51-amino acid zinc finger DNA-binding domain, which may play an important roles in plant growth and development.
AT4G29050	*1,031*	L-TYPE LECTIN RECEPTOR KINASE V.9 (LECRK-V.9)	Concanavalin A-like lectin protein kinase family protein; FUNCTIONS IN: carbohydrate binding, kinase activity; INVOLVED IN: protein amino acid phosphorylation; LOCATED IN: endomembrane system; EXPRESSED IN: 7 plant structures; EXPRESSED DURING: 8 growth stages; CONTAINS InterPro DOMAIN/s: Legume lectin, beta chain (InterPro:IPR001220), Protein kinase, ATP binding site (InterPro:IPR017441), Serine/threonine-protein kinase-like domain (InterPro:IPR017442), Concanavalin A-like lectin/glucanase, subgroup (InterPro:IPR013320), Protein kinase-like domain (InterPro:IPR011009), Serine/threonine-protein kinase, active site (InterPro:IPR008271), Protein kinase, catalytic domain (InterPro:IPR000719), Concanavalin A-like lectin/glucanase (InterPro:IPR008985), Legume lectin, beta chain, Mn/Ca-binding site (InterPro:IPR019825); BEST Arabidopsis thaliana protein match is: Concanavalin A-like lectin protein kinase family protein (TAIR:AT1G70110.1)
AT4G36990	*1,031*	HEAT SHOCK FACTOR 4 (HSF4)	Encodes a protein whose sequence is similar to heat shock factors that regulate the expression of heat shock proteins. Transcript level is increased in response to heat shock. However, overexpression of this gene did not result in the increase of decrease of heat shock proteins.

AT5G38510	*1,031*		Rhomboid-related intramembrane serine protease family protein; FUNCTIONS IN: serine-type endopeptidase activity; INVOLVED IN: biological_process unknown; LOCATED IN: integral to membrane; EXPRESSED IN: 22 plant structures; EXPRESSED DURING: 13 growth stages; CONTAINS InterPro DOMAIN/s: Peptidase S54, rhomboid (InterPro:IPR002610); BEST Arabidopsis thaliana protein match is: RHOMBOID-like protein 10 (TAIR:AT1G25290.2)
AT3G13080	*1,022*	ATP-BINDING CASSETTE C3 (ABCC3)	encodes an ATP-dependent MRP-like ABC transporter able to transport glutathione-conjugates as well as chlorophyll catabolites. The expression of this gene is upregulated by herbicide safeners such as benoxacor and fenclorim.
AT3G44735	*1,021*	PHYTOSULFOKINE 3 PRECURSOR (PSK3)	Phytosulfokine 3 precursor, coding for a unique plant peptide growth factor.
AT1G29560	*1,02*		Zinc finger C-x8-C-x5-C-x3-H type family protein; FUNCTIONS IN: zinc ion binding, nucleic acid binding; LOCATED IN: cellular_component unknown; CONTAINS InterPro DOMAIN/s: Zinc finger, CCCH-type (InterPro:IPR000571); BEST Arabidopsis thaliana protein match is: Zinc finger C-x8-C-x5-C-x3-H type family protein (TAIR:AT1G29570.1)
AT3G08860	*1,02*	PYRIMIDINE 4 (PYD4)	Encodes a protein that is predicted to have beta-alanine aminotransferase activity.
AT5G63770	*1,02*	DIACYLGLYCEROL KINASE 2 (DGK2)	a member of the diacylglycerol kinase gene family. Encodes a functional diacylglycerol kinase. Involved in root elongation and plant development. Gene expression is induced by wounding or cold.
AT3G47690	*1,016*	MICROTUBULE END BINDING PROTEIN EB1A (EB1A)	encodes a homolog of animal microtubule-end-binding protein. There are two other members of this family. EB1 forms foci at regions where the minus ends of microtubules are gathered during mitosis and early cytokinesis.
AT1G72900	*1,014*		Toll-Interleukin-Resistance (TIR) domain-containing protein; FUNCTIONS IN: transmembrane receptor activity, ATP binding; INVOLVED IN: signal transduction, defense response, apoptosis, innate immune response; LOCATED IN: intrinsic to membrane; EXPRESSED IN: 20 plant structures; EXPRESSED DURING: 10 growth stages; CONTAINS InterPro DOMAIN/s: NB-ARC (InterPro:IPR002182), Toll-Interleukin receptor (InterPro:IPR000157); BEST Arabidopsis thaliana protein match is: Toll-Interleukin-Resistance (TIR) domain-containing protein (TAIR:AT1G72940.1)
AT5G02240	*1,012*		Protein is tyrosine-phosphorylated and its phosphorylation state is modulated in response to ABA in Arabidopsis thaliana seeds. The mRNA is cell-to-cell mobile.
AT4G21620	*1,009*		glycine-rich protein; FUNCTIONS IN: molecular_function unknown; INVOLVED IN: biological_process unknown; LOCATED IN: endomembrane system; EXPRESSED IN: 22 plant structures; EXPRESSED DURING: 13 growth stages; BEST Arabidopsis thaliana protein match is: unknown protein (TAIR:AT2G19000.1)
AT5G10510	*1,006*	AINTEGUMENTA-LIKE 6 (AIL6)	Encodes an AP2-domain transcription factor involved in root stem cell identity and root development. It is also required to maintain high levels of PIN1 expression at the periphery of the meristem and modulate local auxin production in the central region of the SAM which underlies phyllotactic transitions. Intronic sequences are required for its expression in flowers.
AT5G42370	*1,005*		Calcineurin-like metallo-phosphoesterase superfamily protein; FUNCTIONS IN: molecular_function unknown; INVOLVED IN: biological_process unknown; LOCATED IN: endomembrane system; EXPRESSED IN: 22 plant structures; EXPRESSED DURING: 13 growth stages; CONTAINS InterPro DOMAIN/s: Alkaline phosphatase D-related (InterPro:IPR018946).
AT2G45290	*1,004*	TRANSKETOLASE 2 (TKL2)	Transketolase; FUNCTIONS IN: catalytic activity, transketolase activity; INVOLVED IN: response to cadmium ion; LOCATED IN: chloroplast stroma, chloroplast; EXPRESSED IN: 25 plant structures; EXPRESSED DURING: 16 growth stages; CONTAINS InterPro DOMAIN/s: Transketolase, bacterial-like (InterPro:IPR005478), Transketolase, N-terminal (InterPro:IPR005474), Transketolase, C-terminal (InterPro:IPR005476), Transketolase, C-terminal/Pyruvate-ferredoxin oxidoreductase, domain II (InterPro:IPR009014), Transketolase-like, C-terminal (InterPro:IPR015941), Transketolase-like, pyrimidine-binding domain (InterPro:IPR005475), Transketolase binding site (InterPro:IPR020826); BEST Arabidopsis thaliana protein match is: Transketolase (TAIR:AT3G60750.1)
AT3G07970	*1*	QUARTET 2 (QRT2)	Required for pollen separation during normal development. In qrt mutants, the outer walls of the four meiotic products of the pollen mother cell are fused, and pollen grains are released in tetrads.May be required for cell type-specific pectin degradation.

AT5G08370	*0,999*	ALPHA-GALACTOSIDASE 2 (AGAL2)	alpha-galactosidase 2 (AGAL2); FUNCTIONS IN: alpha-galactosidase activity, hydrolase activity, hydrolyzing O-glycosyl compounds, catalytic activity; INVOLVED IN: positive regulation of flower development, leaf morphogenesis; LOCATED IN: plant-type cell wall; EXPRESSED IN: 22 plant structures; EXPRESSED DURING: 13 growth stages; CONTAINS InterPro DOMAIN/s: Aldolase-type TIM barrel (InterPro:IPR013785), Glycoside hydrolase, family 27 (InterPro:IPR002241), Glycoside hydrolase, clan GH-D (InterPro:IPR000111), Glycoside hydrolase, catalytic core (InterPro:IPR017853); BEST Arabidopsis thaliana protein match is: alpha-galactosidase 1 (TAIR:AT5G08380.1)
AT1G62780	*0,997*		unknown protein; FUNCTIONS IN: molecular_function unknown; INVOLVED IN: biological_process unknown; LOCATED IN: chloroplast, chloroplast stroma; EXPRESSED IN: 22 plant structures; EXPRESSED DURING: 14 growth stages
AT1G69295	*0,997*	PLASMODESMATA CALLOSE-BINDING PROTEIN 4 (PDCB4)	Encodes a member of the X8-GPI family of proteins. It localizes to the plasmodesmata and is predicted to bind callose.
AT3G25740	*0,993*	METHIONINE AMINOPEPTIDASE 1C (MAP1B)	Encodes a plastid localized methionine aminopeptidase. Formerly called MAP1C, now called MAP1B.
AT3G22840	*0,992*	EARLY LIGHT-INDUCABLE PROTEIN (ELIP1)	Encodes an early light-inducible protein.
AT4G11350	*0,992*		Protein of unknown function (DUF604); CONTAINS InterPro DOMAIN/s: Protein of unknown function DUF604 (InterPro:IPR006740); BEST Arabidopsis thaliana protein match is: Protein of unknown function (DUF604) (TAIR:AT4G23490.1)
AT5G64850	*0,992*		FUNCTIONS IN: molecular_function unknown; INVOLVED IN: biological_process unknown; LOCATED IN: plasma membrane; EXPRESSED IN: 22 plant structures; EXPRESSED DURING: 13 growth stages; CONTAINS InterPro DOMAIN/s: RPM1-interacting protein 4, defence response (InterPro:IPR008700); BEST Arabidopsis thaliana protein match is: unknown protein (TAIR:AT5G09960.1)
AT1G09480	*0,988*		similar to Eucalyptus gunnii alcohol dehydrogenase of unknown physiological function (GI:1143445), Vigna unguiculata (gi:1854445), NOT a cinnamyl-alcohol dehydrogenase The mRNA is cell-to-cell mobile.
AT5G42570	*0,987*		B-cell receptor-associated 31-like; FUNCTIONS IN: molecular_function unknown; INVOLVED IN: intracellular protein transport; LOCATED IN: endoplasmic reticulum, plasma membrane; EXPRESSED IN: 26 plant structures; EXPRESSED DURING: 13 growth stages; CONTAINS InterPro DOMAIN/s: B-cell receptor-associated 31-like (InterPro:IPR008417); BEST Arabidopsis thaliana protein match is: B-cell receptor-associated protein 31-like (TAIR:AT1G11905.1)
AT1G06550	*0,986*		ATP-dependent caseinolytic (Clp) protease/crotonase family protein; FUNCTIONS IN: 3-hydroxyisobutyryl-CoA hydrolase activity, catalytic activity; INVOLVED IN: response to karrikin, fatty acid beta-oxidation; LOCATED IN: cellular_component unknown; EXPRESSED IN: 23 plant structures; EXPRESSED DURING: 13 growth stages; CONTAINS InterPro DOMAIN/s: Crotonase, core (InterPro:IPR001753); BEST Arabidopsis thaliana protein match is: beta-hydroxyisobutyryl-CoA hydrolase 1 (TAIR:AT5G65940.1)
AT1G63020	*0,984*	NUCLEAR RNA POLYMERASE D1A (NRPD1A)	Encodes one of two alternative largest subunits of a putative plant-specific RNA polymerase IV (aka RNA polymerase D). Required for posttranscriptional gene silencing.
AT5G17980	*0,98*		C2 calcium/lipid-binding plant phosphoribosyltransferase family protein; LOCATED IN: endoplasmic reticulum, plasma membrane; EXPRESSED IN: 18 plant structures; EXPRESSED DURING: 7 growth stages; CONTAINS InterPro DOMAIN/s: C2 membrane targeting protein (InterPro:IPR018029), C2 calcium/lipid-binding domain, CaLB (InterPro:IPR008973), Phosphoribosyltransferase C-terminal (InterPro:IPR013583), C2 calcium-dependent membrane targeting (InterPro:IPR000008); BEST Arabidopsis thaliana protein match is: C2 calcium/lipid-binding plant phosphoribosyltransferase family protein (TAIR:AT1G74720.1)
AT5G20280	*0,98*	SUCROSE PHOSPHATE SYNTHASE 1F (SPS1F)	Encodes a protein with putative sucrose-phosphate synthase activity. When the gene was expressed in transgenic tobacco plants, a clear trend for increased SPS activity was noted.
AT5G05180	*0,978*		unknown protein; BEST Arabidopsis thaliana protein match is: unknown protein (TAIR:AT3G10880.1)
AT5G17230	*0,978*	PHYTOENE SYNTHASE (PSY)	Encodes phytoene synthase that is the rate-limiting enzyme in the carotenoid biosynthetic pathway and that interacts with the ORANGE (OR) protein. PSY's expression is posttransciptionally regulated by OR.

AT1G76100	*0,977*	PLASTOCYANIN 1 (PETE1)	One of two Arabidopsis plastocyanin genes. Expressed at 1/10th level of PETE2. Does not respond to increased copper levels and is thought to be the isoform that participates in electron transport under copper-limiting conditions. Mutation of this gene does not have obvious effect on photosynthesis.
AT2G31865	*0,976*	POLY(ADP-RIBOSE) GLYCOHYDROLASE 2 (PARG2)	poly(ADP-ribose) glycohydrolase 2 (PARG2); FUNCTIONS IN: poly(ADP-ribose) glycohydrolase activity; INVOLVED IN: defense response to fungus; LOCATED IN: cellular_component unknown; CONTAINS InterPro DOMAIN/s: Poly(ADP-ribose) glycohydrolase (InterPro:IPR007724); BEST Arabidopsis thaliana protein match is: Poly (ADP-ribose) glycohydrolase (PARG) (TAIR:AT2G31870.2)
AT2G39730	*0,975*	RUBISCO ACTIVASE (RCA)	Rubisco activase, a nuclear-encoded chloroplast protein that consists of two isoforms arising from alternative splicing in most plants. Required for the light activation of rubisco. Involved in jasmonate-induced leaf senescence.
AT2G18050	*0,973*	HISTONE H1-3 (HIS1-3)	encodes a structurally divergent linker histone whose gene expression is induced by dehydration and ABA. The mRNA is cell-to-cell mobile.
AT5G25440	*0,973*		Protein kinase superfamily protein; FUNCTIONS IN: protein kinase activity, kinase activity, ATP binding; INVOLVED IN: protein amino acid phosphorylation, N-terminal protein myristoylation; LOCATED IN: nucleus, cytoplasm; EXPRESSED IN: 15 plant structures; EXPRESSED DURING: 9 growth stages; CONTAINS InterPro DOMAIN/s: Protein kinase, catalytic domain (InterPro:IPR000719), Serine-threonine/tyrosine-protein kinase (InterPro:IPR001245), Protein kinase-like domain (InterPro:IPR011009); BEST Arabidopsis thaliana protein match is: Protein kinase superfamily protein (TAIR:AT5G11410.1)
AT2G06050	*0,972*	OXOPHYTODIENOATE-REDUCTASE 3 (OPR3)	Encodes a 12-oxophytodienoate reductase that is required for jasmonate biosynthesis. Mutants are male sterile and defective in pollen dehiscence. Shows activity towards 2,4,6-trinitrotoluene. CFA-Ile, CFA-Leu, CFA-Val, CFA-Met and CFA-Ala can restore the fertility of opr3 plants by inducing filament elongation and anther dehiscence.
AT3G25900	*0,97*	(HMT-1)	HMT-1; CONTAINS InterPro DOMAIN/s: Homocysteine S-methyltransferase (InterPro:IPR003726); BEST Arabidopsis thaliana protein match is: homocysteine S-methyltransferase 3 (TAIR:AT3G22740.1)
AT1G80450	*0,969*		VQ motif-containing protein; FUNCTIONS IN: molecular_function unknown; INVOLVED IN: biological_process unknown; LOCATED IN: chloroplast; EXPRESSED IN: 14 plant structures; EXPRESSED DURING: 7 growth stages; CONTAINS InterPro DOMAIN/s: VQ (InterPro:IPR008889); BEST Arabidopsis thaliana protein match is: VQ motif-containing protein (TAIR:AT1G28280.2)
AT3G03020	*0,967*		unknown protein; FUNCTIONS IN: molecular_function unknown; INVOLVED IN: biological_process unknown; LOCATED IN: chloroplast; EXPRESSED IN: 21 plant structures; EXPRESSED DURING: 13 growth stages
AT5G60100	*0,966*	PSEUDO-RESPONSE REGULATOR 3 (PRR3)	Encodes pseudo-response regulator 3 (APRR3/PRR3). PRR3 transcript levels vary in a circadian pattern with peak expression at dusk under long and short day conditions. PRR3 affects the period of the circadian clock and seedlings with reduced levels of PRR3 have shorter periods, based on transcriptional assays of clock-regulated genes. PRR3 is expressed in the vasculature of cotyledons and leaves where it may help stabilize the TOC1 protein by preventing interactions between TOC1 and the F-box protein ZTL.
AT5G66170	*0,966*	SULFURTRANSFERASE 18 (STR18)	Encodes a thiosulfate sulfurtransferase/rhodanese.
AT4G34050	*0,965*	CAFFEOYL COENZYME A O-METHYLTRANSFERASE 1 (CCoAOMT1)	caffeoyl coenzyme A O-methyltransferase 1 (CCoAOMT1); FUNCTIONS IN: caffeoyl-CoA O-methyltransferase activity; INVOLVED IN: coumarin biosynthetic process, response to cadmium ion; LOCATED IN: cytosol; EXPRESSED IN: 24 plant structures; EXPRESSED DURING: 14 growth stages; CONTAINS InterPro DOMAIN/s: O-methyltransferase, family 3 (InterPro:IPR002935); BEST Arabidopsis thaliana protein match is: S-adenosyl-L-methionine-dependent methyltransferases superfamily protein (TAIR:AT4G26220.1)
AT4G21570	*0,959*		Protein of unknown function (DUF300); INVOLVED IN: biological_process unknown; LOCATED IN: endomembrane system; CONTAINS InterPro DOMAIN/s: Protein of unknown function DUF300 (InterPro:IPR005178); BEST Arabidopsis thaliana protein match is: Protein of unknown function (DUF300) (TAIR:AT1G11200.1)

AT5G04620	*0,956*	BIOTIN F (BIOF)	The cDNA encoding 7-keto-8-aminopelargonic acid (KAPA) synthase, the first committed enzyme of the biotin synthesis pathway has been cloned and its molecular function confirmed (functional complementation of an E. coli mutant). The subcellular localization of the enzyme (cytosol) proves that the biotin biosynthesis in plants takes place in different compartments which differs from the biosynthetic route found in microorganisms.
AT1G64700	*0,954*		unknown protein; FUNCTIONS IN: molecular_function unknown; INVOLVED IN: N-terminal protein myristoylation; LOCATED IN: cellular_component unknown; EXPRESSED IN: 17 plant structures; EXPRESSED DURING: 11 growth stages; BEST Arabidopsis thaliana protein match is: unknown protein (TAIR:AT3G61920.1)
AT2G23670	*0,954*	HOMOLOG OF SYNECHOCYSTIS YCF37 (YCF37)	homolog of Synechocystis YCF37 (YCF37); FUNCTIONS IN: molecular_function unknown; INVOLVED IN: biological_process unknown; LOCATED IN: thylakoid, chloroplast thylakoid membrane, chloroplast thylakoid lumen, chloroplast; EXPRESSED IN: 20 plant structures; EXPRESSED DURING: 13 growth stages
AT1G05000	*0,953*	PLANT AND FUNGI ATYPICAL DUAL-SPECIﬁCITY PHOSPHATASE 1 (PFA-DSP1)	Encodes an atypical dual-speciﬁcity phosphatase.
AT2G29630	*0,951*	THIAMINC (THIC)	Encodes a protein involved in thiamin biosynthesis. The protein is an iron-sulfur cluster protein predicted to catalyze the conversion of 5-aminoimidazole ribonucleotide (AIR) to hydroxymethylpyrimidine (HMP) or hydroxymethylpyrimidine phosphate (HMP-P). A severe reduction of THIC levels in plants decreases vitamin B1 (thiamin diphosphate (TPP)) levels and also leads to changes in the levels of numerous other metabolites since so many primary metabolic enzymes require a TPP co-factor. thiC mutants are chlorotic and arrest in their development at the cotyledon stage. A N-terminal targeting sequence directs the THIC protein to the chloroplast stroma. A conserved TPP-binding site is located in the 3' UTR of the At2g29630.2 gene model, and is predicted to function as a riboswitch. The riboswitch controls the formation of transcripts with alternative 3' UTR lengths, which affect mRNA accumulation and protein production. THIC transcripts are observed in seedlings 5 or more days after germination, and light promotes the expression of this gene. Recessive mutant isolated by Redei. Leaves but not cotyledons white, lethal; restored to normal by thiamine or 2,5-dimethyl-4-aminopyrimidine.
AT4G00950	*0,95*	MATERNAL EFFECT EMBRYO ARREST 47 (MEE47)	maternal effect embryo arrest 47 (MEE47); CONTAINS InterPro DOMAIN/s: Protein of unknown function DUF688 (InterPro:IPR007789)
AT2G17230	*0,949*	EXORDIUM LIKE 5 (EXL5)	EXORDIUM like 5 (EXL5); FUNCTIONS IN: molecular_function unknown; INVOLVED IN: biological_process unknown; LOCATED IN: endomembrane system; EXPRESSED IN: 22 plant structures; EXPRESSED DURING: 13 growth stages; CONTAINS InterPro DOMAIN/s: Phosphate-induced protein 1 (InterPro:IPR006766); BEST Arabidopsis thaliana protein match is: EXORDIUM like 1 (TAIR:AT2G35150.1)
AT5G47910	*0,942*	RESPIRATORY BURST OXIDASE HOMOLOGUE D (RBOHD)	NADPH/respiratory burst oxidase protein D (RbohD).Interacts with AtrbohF gene to fine tune the spatial control of ROI production and hypersensitive response to cell in and around infection site. The mRNA is cell-to-cell mobile.
AT5G11060	*0,941*	KNOTTED1-LIKE HOMEOBOX GENE 4 (KNAT4)	A member of Class II KN1-like homeodomain transcription factors (together with KNAT3 and KNAT5), with greatest homology to the maize knox1 homeobox protein. Expression regulated by light. Detected in all tissues examined, but most prominent in leaves and young siliques. Transient expression of GFP translational fusion protein suggests bipartite localization in nucleus and cytoplasm. KNAT4 promoter activity showed cell-type specific pattern along longitudinal root axis; GUS expression pattern started at the elongation zone, predominantly in the phloem and pericycle cells, extending to endodermis toward the base of the root.
AT3G27010	*0,938*	TEOSINTE BRANCHED 1, CYCLOIDEA, PCF (TCP)-DOMAIN FAMILY PROTEIN 20 (TCP20)	Belongs to a TCP protein transcription factor family. Members of this family contain a predicted basic-helix-loop-helix domain involved in DNA binding. Related to rice PCF1 and PCF2 genes. Binds to the GCCCR element of CYCB1;1. Involved in regulation of expression of cell cycle control and ribosomal protein genes.
AT5G43420	*0,938*		RING/U-box superfamily protein; FUNCTIONS IN: zinc ion binding; CONTAINS InterPro DOMAIN/s: Zinc finger, RING-type (InterPro:IPR001841), Zinc finger, C3HC4 RING-type (InterPro:IPR018957); BEST Arabidopsis thaliana protein match is: RING/U-box superfamily protein (TAIR:AT1G04360.1)

AT1G50450	*0,937*		Saccharopine dehydrogenase ; FUNCTIONS IN: binding, catalytic activity; INVOLVED IN: metabolic process; LOCATED IN: chloroplast; EXPRESSED IN: 21 plant structures; EXPRESSED DURING: 13 growth stages; CONTAINS InterPro DOMAIN/s: Saccharopine dehydrogenase / Homospermidine synthase (InterPro:IPR005097), NAD(P)-binding domain (InterPro:IPR016040)
AT1G50950	*0,936*		INVOLVED IN: cell redox homeostasis; EXPRESSED IN: 20 plant structures; EXPRESSED DURING: 11 growth stages; CONTAINS InterPro DOMAIN/s: Thioredoxin fold (InterPro:IPR012335), Thioredoxin-like (InterPro:IPR017936), Thioredoxin-like fold (InterPro:IPR012336), Thioredoxin domain (InterPro:IPR013766), Protein of unknown function DUF1692 (InterPro:IPR012936); BEST Arabidopsis thaliana protein match is: PDI-like 5-3 (TAIR:AT3G20560.1)
AT4G22770	*0,935*	AT-HOOK MOTIF NUCLEAR LOCALIZED PROTEIN 2 (AHL2)	AT hook motif DNA-binding family protein; FUNCTIONS IN: DNA binding; EXPRESSED IN: 14 plant structures; EXPRESSED DURING: 4 anthesis, F mature embryo stage, petal differentiation and expansion stage, E expanded cotyledon stage, D bilateral stage; CONTAINS InterPro DOMAIN/s: Protein of unknown function DUF296 (InterPro:IPR005175), AT hook, DNA-binding motif (InterPro:IPR017956); BEST Arabidopsis thaliana protein match is: AT-hook motif nuclear-localized protein 1 (TAIR:AT4G12080.1)
AT2G44080	*0,934*	ARGOS-LIKE (ARL)	Encodes ARL, a gene similar to ARGOS involved in cell expansion-dependent organ growth. Upregulated by brassinosteroid. Acts downstream of BRI1. The mRNA is cell-to-cell mobile.
AT1G09795	*0,933*	ATP PHOSPHORIBOSYL TRANSFERASE 2 (ATP-PRT2)	ATP phosphoribosyl transferase, catalyses first step of histidine biosynthesis
AT2G32280	*0,929*	VASCULATURE COMPLEXITY AND CONNECTIVITY (VCC)	Encodes a member of a plant-specific gene family that is required for embryo provasculature development. The gene product regulates vascular network complexity and connectivity in cotyledons.
AT3G63180	*0,929*	TIC-LIKE (TKL)	TIC-like (TKL); BEST Arabidopsis thaliana protein match is: time for coffee (TAIR:AT3G22380.2)
AT2G03530	*0,928*	UREIDE PERMEASE 2 (UPS2)	Mediate high-affinity uracil and 5-FU (a toxic uracil analogue) transport when expressed in yeast and Xenopus oocytes.Involved in allantoin transport.
AT5G43440	*0,927*		encodes a protein whose sequence is similar to ACC oxidase
AT2G25200	*0,925*		Plant protein of unknown function (DUF868); CONTAINS InterPro DOMAIN/s: Protein of unknown function DUF868, plant (InterPro:IPR008586); BEST Arabidopsis thaliana protein match is: Plant protein of unknown function (DUF868) (TAIR:AT5G11000.1)
AT4G23690	*0,925*	DIRIGENT PROTEIN 6 (DIR6)	Encodes a homodimeric all-beta dirigent protein in the superfamily of calycins. Dirigent proteins impart stereoselectivity on the phenoxy radical coupling reaction yielding optically active lignans from two molecules of coniferyl alcohol.
AT1G70800	*0,923*	ENHANCED BENDING 1 (EHB1)	Encodes a novel NPH3/phototropin binding factor with a calcium binding domain that negatively affects hypocotyl bending under blue light conditions in Arabidopsis thaliana and may regulate phototropism.
AT1G21670	*0,922*		LOCATED IN: cell wall, plant-type cell wall; EXPRESSED IN: 23 plant structures; EXPRESSED DURING: 14 growth stages; CONTAINS InterPro DOMAIN/s: WD40-like Beta Propeller (InterPro:IPR011659), Six-bladed beta-propeller, TolB-like (InterPro:IPR011042); BEST Arabidopsis thaliana protein match is: DPP6 N-terminal domain-like protein (TAIR:AT1G21680.1)
AT1G55120	*0,922*	BETA-FRUCTOFURANOSIDASE 5 (FRUCT5)	Encodes a protein with fructan exohydrolase (FEH) activity acting on levan-type fructans (6-FEH, levanase). The enzyme does not have invertase activity.
AT5G13280	*0,917*	ASPARTATE KINASE 1 (AK-LYS1)	Asp kinase inhibited by Lys and S-adenosylmethionine. Contains regulatory domains that belong to the ACT domain family, which allow binding to a extreme variety of ligands. Can function as a monomer or as a dimer with acetohydroxyacid synthase (HSDH).
AT1G42970	*0,915*	GLYCERALDEHYDE-3-PHOSPHATE DEHYDROGENASE B SUBUNIT (GAPB)	Encodes chloroplast localized glyceraldehyde-3-phosphate dehydrogenase that can use both NADH and NADPH to reduce 1,3-diphosphate glycerate. It forms A2B2 heterotetramers with GapA forms of the GADPH enzyme. These complexes are active in the light under reducing conditions, but show reduced NADPH-dependent activity in response to oxidized thioredoxins and increased NAD(H)/NADP(H) ratios due to the formation of inactive A8B8 hexadecamers. The mRNA is cell-to-cell mobile.

AT2G35340	*0,914*	MATERNAL EFFECT EMBRYO ARREST 29 (MEE29)	maternal effect embryo arrest 29 (MEE29); FUNCTIONS IN: helicase activity, ATP-dependent RNA helicase activity, ATP binding, ATP-dependent helicase activity, nucleic acid binding; INVOLVED IN: embryo development ending in seed dormancy; LOCATED IN: endomembrane system; EXPRESSED IN: 22 plant structures; EXPRESSED DURING: 13 growth stages; CONTAINS InterPro DOMAIN/s: Helicase-associated domain (InterPro:IPR007502), DNA/RNA helicase, DEAD/DEAH box type, N-terminal (InterPro:IPR011545), Domain of unknown function DUF1605 (InterPro:IPR011709), DNA/RNA helicase, ATP-dependent, DEAH-box type, conserved site (InterPro:IPR002464), DEAD-like helicase, N-terminal (InterPro:IPR014001), DNA/RNA helicase, C-terminal (InterPro:IPR001650), Helicase, superfamily 1/2, ATP-binding domain (InterPro:IPR014021); BEST Arabidopsis thaliana protein match is: RNA helicase family protein (TAIR:AT1G32490.1)
AT5G11880	*0,913*		Pyridoxal-dependent decarboxylase family protein; FUNCTIONS IN: diaminopimelate decarboxylase activity; INVOLVED IN: lysine biosynthetic process via diaminopimelate; LOCATED IN: chloroplast; EXPRESSED IN: guard cell, cultured cell; CONTAINS InterPro DOMAIN/s: Alanine racemase/group IV decarboxylase, C-terminal (InterPro:IPR009006), Ornithine/DAP/Arg decarboxylase (InterPro:IPR000183), Orn/DAP/Arg decarboxylase 2, N-terminal (InterPro:IPR022644), Orn/DAP/Arg decarboxylase 2, C-terminal (InterPro:IPR022643), Diaminopimelate decarboxylase (InterPro:IPR002986), Orn/DAP/Arg decarboxylase 2, conserved site (InterPro:IPR022657), Orn/DAP/Arg decarboxylase 2, pyridoxal-phosphate binding site (InterPro:IPR022653); BEST Arabidopsis thaliana protein match is: Pyridoxal-dependent decarboxylase family protein (TAIR:AT3G14390.1)
AT2G35370	*0,912*	GLYCINE DECARBOXYLASE COMPLEX H (GDCH)	Encodes glycine decarboxylase complex H protein. Involved in photorespiration. The mRNA is cell-to-cell mobile.
AT1G20620	*0,91*	CATALASE 3 (CAT3)	Catalase, catalyzes the breakdown of hydrogen peroxide (H2O2) into water and oxygen. The mRNA is cell-to-cell mobile.
AT1G77020	*0,91*		DNAJ heat shock N-terminal domain-containing protein; FUNCTIONS IN: unfolded protein binding, heat shock protein binding; INVOLVED IN: protein folding; LOCATED IN: cellular_component unknown; EXPRESSED IN: 22 plant structures; EXPRESSED DURING: 12 growth stages; CONTAINS InterPro DOMAIN/s: Molecular chaperone, heat shock protein, Hsp40, DnaJ (InterPro:IPR015609), Heat shock protein DnaJ, N-terminal (InterPro:IPR001623), Heat shock protein DnaJ (InterPro:IPR003095), Heat shock protein DnaJ, conserved site (InterPro:IPR018253); BEST Arabidopsis thaliana protein match is: DNAJ heat shock N-terminal domain-containing protein (TAIR:AT1G76700.1)
AT1G16220	*0,908*		Protein phosphatase 2C family protein; FUNCTIONS IN: protein serine/threonine phosphatase activity, catalytic activity; INVOLVED IN: N-terminal protein myristoylation; LOCATED IN: plasma membrane; EXPRESSED IN: 10 plant structures; EXPRESSED DURING: 6 growth stages; CONTAINS InterPro DOMAIN/s: Protein phosphatase 2C-related (InterPro:IPR001932), Protein phosphatase 2C (InterPro:IPR015655), Protein phosphatase 2C, N-terminal (InterPro:IPR014045); BEST Arabidopsis thaliana protein match is: Protein phosphatase 2C family protein (TAIR:AT1G79630.1)
AT2G33110	*0,905*	VESICLE-ASSOCIATED MEMBRANE PROTEIN 723 (VAMP723)	member of VAMP72 Gene Family
AT2G28510	*0,899*		Dof-type zinc finger DNA-binding family protein; FUNCTIONS IN: DNA binding, sequence-specific DNA binding transcription factor activity; INVOLVED IN: regulation of transcription; LOCATED IN: nucleolus; EXPRESSED IN: 22 plant structures; EXPRESSED DURING: 13 growth stages; CONTAINS InterPro DOMAIN/s: Zinc finger, Dof-type (InterPro:IPR003851); BEST Arabidopsis thaliana protein match is: TARGET OF MONOPTEROS 6 (TAIR:AT5G60200.1)
AT3G17790	*0,899*	PURPLE ACID PHOSPHATASE 17 (PAP17)	Expression is upregulated in the shoot of cax1/cax3 mutant and is responsive to phosphate (Pi) and not phosphite (Phi) in roots and shoots.
AT4G36910	*0,899*	LOSS OF THE TIMING OF ET AND JA BIOSYNTHESIS 2 (LEJ2)	Encodes a single cystathionine beta-synthase domain-containing protein. Modulates development by regulating the thioredoxin system.

AT1G74390	*0,898*	NAC45/86-DEPENDENT EXONUCLEASE-DOMAIN PROTEIN 3 (NEN3)	Polynucleotidyl transferase, ribonuclease H-like superfamily protein; FUNCTIONS IN: exonuclease activity, nucleic acid binding; LOCATED IN: intracellular; EXPRESSED IN: 12 plant structures; EXPRESSED DURING: 6 growth stages; CONTAINS InterPro DOMAIN/s: Exonuclease (InterPro:IPR006055), Polynucleotidyl transferase, ribonuclease H fold (InterPro:IPR012337), Exonuclease, RNase T/DNA polymerase III (InterPro:IPR013520); BEST Arabidopsis thaliana protein match is: Polynucleotidyl transferase, ribonuclease H-like superfamily protein (TAIR:AT5G61390.1)
AT5G39580	*0,897*		Peroxidase superfamily protein; FUNCTIONS IN: peroxidase activity, heme binding; INVOLVED IN: defense response to fungus, N-terminal protein myristoylation; LOCATED IN: endomembrane system; EXPRESSED IN: 17 plant structures; EXPRESSED DURING: 12 growth stages; CONTAINS InterPro DOMAIN/s: Haem peroxidase (InterPro:IPR010255), Plant peroxidase (InterPro:IPR000823), Haem peroxidase, plant/fungal/bacterial (InterPro:IPR002016); BEST Arabidopsis thaliana protein match is: Peroxidase superfamily protein (TAIR:AT5G64120.1)
AT5G52450	*0,896*		MATE efflux family protein; FUNCTIONS IN: antiporter activity, drug transmembrane transporter activity, transporter activity; INVOLVED IN: response to nematode; LOCATED IN: plasma membrane, membrane; EXPRESSED IN: 24 plant structures; EXPRESSED DURING: 15 growth stages; CONTAINS InterPro DOMAIN/s: MATE family transporter related protein (InterPro:IPR015521), Multi antimicrobial extrusion protein MatE (InterPro:IPR002528); BEST Arabidopsis thaliana protein match is: MATE efflux family protein (TAIR:AT2G34360.1)
AT3G28920	*0,895*	HOMEOBOX PROTEIN 34 (HB34)	homeobox protein 34 (HB34); FUNCTIONS IN: DNA binding, sequence-specific DNA binding transcription factor activity; INVOLVED IN: regulation of transcription; EXPRESSED IN: 22 plant structures; EXPRESSED DURING: 13 growth stages; CONTAINS InterPro DOMAIN/s: Homeobox domain, ZF-HD class (InterPro:IPR006455), ZF-HD homeobox protein, Cys/His-rich dimerisation domain (InterPro:IPR006456), Homeodomain-related (InterPro:IPR012287); BEST Arabidopsis thaliana protein match is: homeobox protein 23 (TAIR:AT5G39760.1)
AT4G31000	*0,895*		Calmodulin-binding protein; CONTAINS InterPro DOMAIN/s: Calmodulin binding protein-like (InterPro:IPR012416); BEST Arabidopsis thaliana protein match is: Calmodulin-binding protein (TAIR:AT2G24300.2)
AT3G49690	*0,894*	MYB DOMAIN PROTEIN 84 (MYB84)	Putative homolog of the Blind gene in tomato. Together with RAX1 and RAX3 belong to the class R2R3 MYB genes; encoded by the Myb-like transcription factor MYB84, regulates axillary meristem formation.
AT5G65020	*0,892*	ANNEXIN 2 (ANNAT2)	Annexins are calcium binding proteins that are localized in the cytoplasm. When cytosolic Ca2+ increases, they relocate to the plasma membrane. They may be involved in the Golgi-mediated secretion of polysaccharides.
AT4G13250	*0,891*	NON-YELLOW COLORING 1 (NYC1)	Encodes a chlorophyll b reductase involved in the degradation of chlorophyll b and LHCII (light harvesting complex II).
AT1G78610	*0,89*	MECHANOSENSITIVE CHANNEL OF SMALL CONDUCTANCE-LIKE 6 (MSL6)	mechanosensitive channel of small conductance-like 6 (MSL6); INVOLVED IN: transmembrane transport; LOCATED IN: plasma membrane, membrane; EXPRESSED IN: 23 plant structures; EXPRESSED DURING: 13 growth stages; CONTAINS InterPro DOMAIN/s: Membrane protein, At2g17000, predicted (InterPro:IPR016688), Mechanosensitive ion channel MscS (InterPro:IPR006685), Like-Sm ribonucleoprotein (LSM)-related domain (InterPro:IPR010920); BEST Arabidopsis thaliana protein match is: mechanosensitive channel of small conductance-like 4 (TAIR:AT1G53470.1)
AT5G48300	*0,89*	ADP GLUCOSE PYROPHOSPHORYLASE 1 (ADG1)	Encodes the small subunit of ADP-glucose pyrophosphorylase. The small subunit is the catalytic isoform responsible for ADP-glucose pyrophosphorylase activity. The presence of the small subunit is required for large subunit stability. Two isoforms of the small subunit (ApS1 and ApS2) have been described. ApS1 is the major small subunit isoform present in all plant tissues tested. The mRNA is cell-to-cell mobile.
AT1G04770	*0,885*	SULFUR DEFICIENCY INDUCED 2 (SD!2)	Tetratricopeptide repeat (TPR)-like superfamily protein; FUNCTIONS IN: binding; INVOLVED IN: biological_process unknown; EXPRESSED IN: 23 plant structures; EXPRESSED DURING: 13 growth stages; CONTAINS InterPro DOMAIN/s: Tetratricopeptide TPR-1 (InterPro:IPR001440), Tetratricopeptide-like helical (InterPro:IPR011990), Tetratricopeptide repeat-containing (InterPro:IPR013026), Tetratricopeptide repeat (InterPro:IPR019734); BEST Arabidopsis thaliana protein match is: Tetratricopeptide repeat (TPR)-like superfamily protein (TAIR:AT5G48850.1)

AT2G32220	*0,882*		Ribosomal L27e protein family; FUNCTIONS IN: structural constituent of ribosome; INVOLVED IN: translation; LOCATED IN: ribosome, cytosolic large ribosomal subunit, intracellular, membrane; EXPRESSED IN: 20 plant structures; EXPRESSED DURING: 13 growth stages; CONTAINS InterPro DOMAIN/s: Ribosomal protein L27e, conserved site (InterPro:IPR018262), Ribosomal protein L27e (InterPro:IPR001141); BEST Arabidopsis thaliana protein match is: Ribosomal L27e protein family (TAIR:AT3G22230.1)
AT5G43380	*0,88*	TYPE ONE SERINE/THREONINE PROTEIN PHOSPHATASE 6 (TOPP6)	encodes a type I serine/threonine protein phosphatase expressed in expressed in roots, rosettes and flowers.
AT5G56790	*0,88*		Protein kinase superfamily protein; FUNCTIONS IN: protein tyrosine kinase activity, protein kinase activity, kinase activity, ATP binding; INVOLVED IN: protein amino acid phosphorylation; EXPRESSED IN: 22 plant structures; EXPRESSED DURING: 13 growth stages; CONTAINS InterPro DOMAIN/s: Protein kinase, catalytic domain (InterPro:IPR000719), Tyrosine-protein kinase, active site (InterPro:IPR008266), Serine-threonine/tyrosine-protein kinase (InterPro:IPR001245), Protein kinase-like domain (InterPro:IPR011009); BEST Arabidopsis thaliana protein match is: Protein kinase protein with adenine nucleotide alpha hydrolases-like domain (TAIR:AT3G13690.1)
AT1G69870	*0,877*	NRT1/ PTR FAMILY 2.13 (NPF2.13)	Encodes a low affinity nitrate transporter NRT1.7. Expressed in phloem. Responsible for source-to-sink remobilization of nitrate. The mRNA is cell-to-cell mobile.
AT5G14370	*0,877*		CCT motif family protein; LOCATED IN: chloroplast; EXPRESSED IN: 21 plant structures; EXPRESSED DURING: 13 growth stages; CONTAINS InterPro DOMAIN/s: CCT domain (InterPro:IPR010402); BEST Arabidopsis thaliana protein match is: CCT motif family protein (TAIR:AT4G25990.1)
AT2G01320	*0,873*	ATP-BINDING CASSETTE G7 (ABCG7)	ABC-2 type transporter family protein; FUNCTIONS IN: ATPase activity, coupled to transmembrane movement of substances; LOCATED IN: chloroplast; EXPRESSED IN: 22 plant structures; EXPRESSED DURING: 14 growth stages; CONTAINS InterPro DOMAIN/s: ATPase, AAA+ type, core (InterPro:IPR003593), ABC transporter-like (InterPro:IPR003439), ABC-2 type transporter (InterPro:IPR013525), ABC transporter, conserved site (InterPro:IPR017871); BEST Arabidopsis thaliana protein match is: ABC-2 type transporter family protein (TAIR:AT3G25620.2)
AT4G05180	*0,872*	PHOTOSYSTEM II SUBUNIT Q-2 (PSBQ-2)	Encodes the PsbQ subunit of the oxygen evolving complex of photosystem II.
AT3G44880	*0,871*	ACCELERATED CELL DEATH 1 (ACD1)	Encodes a pheide a oxygenase (PAO). Accelerated cell death (acd1) mutants show rapid, spreading necrotic responses to both virulent and avirulent Pseudomonas syringae pv. maculicola or pv. tomato pathogens and to ethylene.
AT5G01030	*0,869*		Protein of unknown function (DUF3527); FUNCTIONS IN: molecular_function unknown; INVOLVED IN: biological_process unknown; LOCATED IN: cellular_component unknown; EXPRESSED IN: 24 plant structures; EXPRESSED DURING: 13 growth stages; CONTAINS InterPro DOMAIN/s: Protein of unknown function DUF3527 (InterPro:IPR021916); BEST Arabidopsis thaliana protein match is: Protein of unknown function (DUF3527) (TAIR:AT2G37930.1)
AT5G66630	*0,869*	DA1-RELATED PROTEIN 5 (DAR5)	DA1-related protein 5 (DAR5); FUNCTIONS IN: zinc ion binding, ATP binding; INVOLVED IN: apoptosis; EXPRESSED IN: 12 plant structures; EXPRESSED DURING: LP.04 four leaves visible, 4 anthesis, petal differentiation and expansion stage; CONTAINS InterPro DOMAIN/s: Zinc finger, LIM-type (InterPro:IPR001781), NB-ARC (InterPro:IPR002182), Powdery mildew resistance protein, RPW8 domain (InterPro:IPR008808), Protein of unknown function DUF3633 (InterPro:IPR022087); BEST Arabidopsis thaliana protein match is: DA1-related protein 6 (TAIR:AT5G66620.1)
AT5G53420	*0,866*		CCT motif family protein; FUNCTIONS IN: molecular_function unknown; INVOLVED IN: biological_process unknown; LOCATED IN: cellular_component unknown; EXPRESSED IN: 23 plant structures; EXPRESSED DURING: 13 growth stages; CONTAINS InterPro DOMAIN/s: CCT domain (InterPro:IPR010402); BEST Arabidopsis thaliana protein match is: CCT motif family protein (TAIR:AT4G27900.2)

AT4G20430	*0,864*		Subtilase family protein; FUNCTIONS IN: identical protein binding, serine-type endopeptidase activity; INVOLVED IN: proteolysis, negative regulation of catalytic activity; LOCATED IN: endomembrane system; EXPRESSED IN: 19 plant structures; EXPRESSED DURING: 13 growth stages; CONTAINS InterPro DOMAIN/s: Peptidase S8/S53, subtilisin/kexin/sedolisin (InterPro:IPR000209), Peptidase S8, subtilisin-related (InterPro:IPR015500), Peptidase S8/S53, subtilisin, active site (InterPro:IPR022398), Proteinase inhibitor I9, subtilisin propeptide (InterPro:IPR010259); BEST Arabidopsis thaliana protein match is: Subtilase family protein (TAIR:AT5G44530.1)
AT5G27760	*0,863*		Hypoxia-responsive family protein; CONTAINS InterPro DOMAIN/s: Hypoxia induced protein, conserved region (InterPro:IPR007667); BEST Arabidopsis thaliana protein match is: Hypoxia-responsive family protein (TAIR:AT3G05550.1)
AT4G19200	*0,861*		proline-rich family protein
AT4G34290	*0,86*		SWIB/MDM2 domain superfamily protein; FUNCTIONS IN: molecular_function unknown; INVOLVED IN: biological_process unknown; LOCATED IN: chloroplast; EXPRESSED IN: 21 plant structures; EXPRESSED DURING: 13 growth stages; CONTAINS InterPro DOMAIN/s: SWIB/MDM2 domain (InterPro:IPR003121), SWIB domain (InterPro:IPR019835); BEST Arabidopsis thaliana protein match is: SWIB/MDM2 domain superfamily protein (TAIR:AT2G14880.1)
AT3G44300	*0,859*	NITRILASE 2 (NIT2)	Encodes an enzyme that catalyzes the hydrolysis of indole-3-acetonitrile (IAN) to indole-3-acetic acid (IAA) (nitrile aminohydrolase, EC 3.5.5.1) and IAN to indole-3-acetamide (IAM) at lower levels. Mutants have reduced sensitivity to IAN and are sensitive to IAA. This enzyme likely participates in other non-auxin-related metabolic pathways. The mRNA is cell-to-cell mobile.
AT2G05910	*0,853*		Protein of unknown function (DUF567); CONTAINS InterPro DOMAIN/s: Protein of unknown function DUF567 (InterPro:IPR007612); BEST Arabidopsis thaliana protein match is: Protein of unknown function (DUF567) (TAIR:AT5G20640.1)
AT2G26500	*0,851*		cytochrome b6f complex subunit (petM), putative; FUNCTIONS IN: plastoquinol-plastocyanin reductase activity; LOCATED IN: chloroplast thylakoid membrane; EXPRESSED IN: 22 plant structures; EXPRESSED DURING: 13 growth stages; CONTAINS InterPro DOMAIN/s: PetM of cytochrome b6/f complex subunit 7 (InterPro:IPR012595)
AT5G10770	*0,848*		Eukaryotic aspartyl protease family protein; FUNCTIONS IN: DNA binding, aspartic-type endopeptidase activity; INVOLVED IN: proteolysis; EXPRESSED IN: 12 plant structures; EXPRESSED DURING: 4 anthesis, C globular stage, petal differentiation and expansion stage, E expanded cotyledon stage; CONTAINS InterPro DOMAIN/s: Peptidase aspartic (InterPro:IPR021109), Peptidase aspartic, catalytic (InterPro:IPR009007), Peptidase A1 (InterPro:IPR001461); BEST Arabidopsis thaliana protein match is: Eukaryotic aspartyl protease family protein (TAIR:AT5G10760.1)
AT3G02790	*0,842*	METHYLENE BLUE SENSITIVITY 1 (MBS1)	zinc finger (C2H2 type) family protein; FUNCTIONS IN: sequence-specific DNA binding transcription factor activity; INVOLVED IN: regulation of transcription; LOCATED IN: intracellular; EXPRESSED IN: 22 plant structures; EXPRESSED DURING: 14 growth stages; CONTAINS InterPro DOMAIN/s: Zinc finger, C2H2-like (InterPro:IPR015880); BEST Arabidopsis thaliana protein match is: zinc finger (C2H2 type) family protein (TAIR:AT5G16470.1)
AT2G43500	*0,837*	NIN-LIKE PROTEIN 8 (NLP8)	Plant regulator RWP-RK family protein; CONTAINS InterPro DOMAIN/s: Octicosapeptide/Phox/Bem1p (InterPro:IPR000270), Plant regulator RWP-RK (InterPro:IPR003035); BEST Arabidopsis thaliana protein match is: Plant regulator RWP-RK family protein (TAIR:AT3G59580.2)
AT5G46700	*0,837*	TORNADO 2 (TRN2)	Encodes a transmembrane protein of the tetraspanin (TET) family, one of 17 members found in Arabidopsis. Double mutant analysis showed that TRN1 and TRN2 act in the same pathway. Required for the maintenance of both the radial pattern of tissue differentiation in the root and for the subsequent circumferential pattern within the epidermis.
AT3G23790	*0,833*	ACYL ACTIVATING ENZYME 16 (AAE16)	acyl activating enzyme 16 (AAE16); FUNCTIONS IN: catalytic activity; INVOLVED IN: metabolic process; LOCATED IN: chloroplast envelope; EXPRESSED IN: 21 plant structures; EXPRESSED DURING: 9 growth stages; CONTAINS InterPro DOMAIN/s: AMP-binding (InterPro:IPR020459), AMP-binding, conserved site (InterPro:IPR020845), AMP-dependent synthetase/ligase (InterPro:IPR000873); BEST Arabidopsis thaliana protein match is: acyl-activating enzyme 15 (TAIR:AT4G14070.1)
AT5G04360	*0,831*	LIMIT DEXTRINASE (LDA)	Encodes an enzyme thought to be involved in the hydrolysis of the α-1,6 linkages during starch degradation in seed endosperm. However, a knockout mutant of Arabidopsis lacking limit dextrinase has normal rates of starch degradation in the leaf at night, indicating that more than one isoamylases might be involved in this process.

AT5G08500	*0,828*		Transmembrane CLPTM1 family protein; CONTAINS InterPro DOMAIN/s: Cleft lip and palate transmembrane 1 (InterPro:IPR008429); BEST Arabidopsis thaliana protein match is: Transmembrane CLPTM1 family protein (TAIR:AT5G23575.1)
AT5G19090	*0,826*		Heavy metal transport/detoxification superfamily protein ; FUNCTIONS IN: metal ion binding; INVOLVED IN: metal ion transport; LOCATED IN: cellular_component unknown; EXPRESSED IN: 22 plant structures; EXPRESSED DURING: 13 growth stages; CONTAINS InterPro DOMAIN/s: Heavy metal transport/detoxification protein (InterPro:IPR006121); BEST Arabidopsis thaliana protein match is: Heavy metal transport/detoxification superfamily protein (TAIR:AT3G06130.2)
AT2G44280	*0,822*		Major facilitator superfamily protein; CONTAINS InterPro DOMAIN/s: Major facilitator superfamily, general substrate transporter (InterPro:IPR016196); BEST Arabidopsis thaliana protein match is: Major facilitator superfamily protein (TAIR:AT3G60070.1)
AT4G09760	*0,82*	CHOLINE/ETHANOLAMINE KINASE 3 (CEK3)	encodes a choline synthase whose gene expression is induced by high salt and mannitol.
AT1G78280	*0,811*		transferases, transferring glycosyl groups; CONTAINS InterPro DOMAIN/s: Transcription factor jumonji/aspartyl beta-hydroxylase (InterPro:IPR003347), F-box domain, cyclin-like (InterPro:IPR001810), F-box domain, Skp2-like (InterPro:IPR022364), Transcription factor jumonji (InterPro:IPR013129), Aminoglycoside phosphotransferase (InterPro:IPR002575), Protein kinase-like domain (InterPro:IPR011009); BEST Arabidopsis thaliana protein match is: unknown protein (TAIR:AT5G06550.1)
AT2G24260	*0,811*	LJRHL1-LIKE 1 (LRL1)	Encodes a basic helix-loop-helix (bHLH) protein that regulates root hair development. One of the three Arabidopsis homologs of the Lotus japonicus ROOTHAIRLESS1 (LjRHL1) gene: At2g24260 (AtLRL1), At4g30980 (AtLRL2), and At5g58010 (AtLRL3).
AT2G21280	*0,795*	(SULA)	A nuclear-encoded, plastid-targeted protein (AtSulA) whose overexpression causes severe yet stochastic plastid (shown in chloroplasts and leucoplasts) division defects. The protein does not appear to interact with either AtFtsZ proteins when studied in a yeast two-hybrid system.
AT1G12420	*0,794*	ACT DOMAIN REPEAT 8 (ACR8)	ACT domain repeat 8 (ACR8); CONTAINS InterPro DOMAIN/s: Amino acid-binding ACT (InterPro:IPR002912); BEST Arabidopsis thaliana protein match is: ACT domain repeat 7 (TAIR:AT4G22780.1)
AT1G73080	*0,789*	PEP1 RECEPTOR 1 (PEPR1)	Encodes a leucine-rich repeat receptor kinase. Functions as a receptor for AtPep1 to amplify innate immunity response to pathogen attacks. The mRNA is cell-to-cell mobile.
AT5G54770	*0,788*	(THI1)	Encodes a thiamine biosynthetic gene that has a dual function in thiamine biosynthesis and mitochondrial DNA damage tolerance. It appears to be involved in producing the thiazole portion of thiamine (vitamin B1). A crystal structure of the protein reveals that it forms a 2-ring homo-octamer. The mRNA is cell-to-cell mobile.
AT5G58160	*0,783*		actin binding; FUNCTIONS IN: actin binding; INVOLVED IN: cellular component organization, actin cytoskeleton organization; EXPRESSED IN: 8 plant structures; EXPRESSED DURING: 4 anthesis, petal differentiation and expansion stage; CONTAINS InterPro DOMAIN/s: Actin-binding FH2/DRF autoregulatory (InterPro:IPR003104), C2 calcium/lipid-binding domain, CaLB (InterPro:IPR008973), Actin-binding FH2 (InterPro:IPR015425), Tensin phosphatase, C2 domain (InterPro:IPR014020); BEST Arabidopsis thaliana protein match is: Actin-binding FH2 (Formin Homology) protein (TAIR:AT2G25050.1)
AT1G03040	*0,781*		basic helix-loop-helix (bHLH) DNA-binding superfamily protein; FUNCTIONS IN: DNA binding, sequence-specific DNA binding transcription factor activity; INVOLVED IN: regulation of transcription, acetyl-CoA biosynthetic process from pyruvate; LOCATED IN: nucleus; EXPRESSED IN: 22 plant structures; EXPRESSED DURING: 13 growth stages; CONTAINS InterPro DOMAIN/s: Helix-loop-helix DNA-binding domain (InterPro:IPR001092), Helix-loop-helix DNA-binding (InterPro:IPR011598); BEST Arabidopsis thaliana protein match is: basic helix-loop-helix (bHLH) DNA-binding superfamily protein (TAIR:AT4G02590.2)
AT5G47070	*0,775*		Protein kinase superfamily protein; FUNCTIONS IN: protein serine/threonine kinase activity, protein kinase activity, kinase activity, ATP binding; INVOLVED IN: protein amino acid phosphorylation; LOCATED IN: plasma membrane; EXPRESSED IN: 22 plant structures; EXPRESSED DURING: 13 growth stages; CONTAINS InterPro DOMAIN/s: Protein kinase, ATP binding site (InterPro:IPR017441), Protein kinase, catalytic domain (InterPro:IPR000719), Serine/threonine-protein kinase-like domain (InterPro:IPR017442), Protein kinase-like domain (InterPro:IPR011009), Serine/threonine-protein kinase, active site (InterPro:IPR008271); BEST Arabidopsis thaliana protein match is: Protein kinase superfamily protein (TAIR:AT4G17660.1)

AT1G09470	*0,769*	(ATNEAP3)	NEAP3 is a member of a small family containing coiled-coil domains, a nuclear localization signal and a C-terminal predicted transmembrane domain. It localizes to the nuclear periphery. Mutants have altered nuclear morphology and chromatin structure.
AT1G73880	*0,769*	UDP-GLUCOSYL TRANSFERASE 89B1 (UGT89B1)	UDP-glucosyl transferase 89B1 (UGT89B1); FUNCTIONS IN: in 7 functions; INVOLVED IN: metabolic process; LOCATED IN: cellular_component unknown; CONTAINS InterPro DOMAIN/s: UDP-glucuronosyl/UDP-glucosyltransferase (InterPro:IPR002213); BEST Arabidopsis thaliana protein match is: UDP-Glycosyltransferase superfamily protein (TAIR:AT1G51210.1)
AT5G16290	*0,761*	VALINE-TOLERANT 1 (VAT1)	Encodes a regulatory subunit of acetohydroxy acid synthase (AHAS), the ﬁrst committed enzyme in the branched chain amino acid biosynthesis pathway.
AT4G02590	*0,758*	UNFERTILIZED EMBRYO SAC 12 (UNE12)	unfertilized embryo sac 12 (UNE12); FUNCTIONS IN: DNA binding, sequence-specific DNA binding transcription factor activity; INVOLVED IN: double fertilization forming a zygote and endosperm, regulation of transcription; LOCATED IN: nucleus; EXPRESSED IN: 22 plant structures; EXPRESSED DURING: 13 growth stages; CONTAINS InterPro DOMAIN/s: Helix-loop-helix DNA-binding domain (InterPro:IPR001092), Helix-loop-helix DNA-binding (InterPro:IPR011598); BEST Arabidopsis thaliana protein match is: basic helix-loop-helix (bHLH) DNA-binding superfamily protein (TAIR:AT1G03040.1)
AT4G21280	*0,758*	PHOTOSYSTEM II SUBUNIT QA (PSBQA)	Encodes the PsbQ subunit of the oxygen evolving complex of photosystem II.
AT2G37620	*0,753*	ACTIN 1 (ACT1)	Member of the actin gene family. Expressed in mature pollen.
AT5G25930	*0,753*		Protein kinase family protein with leucine-rich repeat domain; FUNCTIONS IN: kinase activity; INVOLVED IN: protein amino acid phosphorylation; LOCATED IN: plasma membrane, membrane; EXPRESSED IN: 23 plant structures; EXPRESSED DURING: 14 growth stages; CONTAINS InterPro DOMAIN/s: Protein kinase, ATP binding site (InterPro:IPR017441), Protein kinase, catalytic domain (InterPro:IPR000719), Leucine-rich repeat-containing N-terminal domain, type 2 (InterPro:IPR013210), Leucine-rich repeat (InterPro:IPR001611), Serine/threonine-protein kinase-like domain (InterPro:IPR017442), Protein kinase-like domain (InterPro:IPR011009), Serine/threonine-protein kinase, active site (InterPro:IPR008271); BEST Arabidopsis thaliana protein match is: receptor like protein 52 (TAIR:AT5G25910.1)
AT1G04710	*0,746*	PEROXISOMAL 3-KETOACYL-COA THIOLASE 4 (PKT4)	EC2.3.1.16 thiolase. Its transcript levels change after inducing MUTE expression in a mute background.
AT5G16570	*0,744*	GLUTAMINE SYNTHETASE 1;4 (GLN1;4)	Encodes a cytosolic glutamine synthetase, the enzyme has high affinity with substrate ammonium
AT4G03280	*0,741*	PHOTOSYNTHETIC ELECTRON TRANSFER C (PETC)	Encodes the Rieske FeS center of cytochrome b6f complex. Gene is expressed in shoot but not in root. Mutant has reduced electron transport at saturating light intensities and Q-cycle activity is hypersensitive to acidification of the thylakoid lumen. The mRNA is cell-to-cell mobile.
AT2G19940	*0,735*		oxidoreductases, acting on the aldehyde or oxo group of donors, NAD or NADP as acceptor;copper ion binding; FUNCTIONS IN: oxidoreductase activity, acting on the aldehyde or oxo group of donors, NAD or NADP as acceptor, copper ion binding; INVOLVED IN: response to cadmium ion, cellular amino acid metabolic process; LOCATED IN: nucleolus, chloroplast stroma, chloroplast, membrane; EXPRESSED IN: 25 plant structures; EXPRESSED DURING: 14 growth stages; CONTAINS InterPro DOMAIN/s: Semialdehyde dehydrogenase, dimerisation domain (InterPro:IPR012280), Semialdehyde dehydrogenase, NAD-binding (InterPro:IPR000534), N-acetyl-gamma-glutamyl-phosphate reductase (InterPro:IPR000706)
AT5G25900	*0,732*	GA REQUIRING 3 (GA3)	Encodes a member of the CYP701A cytochrome p450 family that is involved in later steps of the gibberellin biosynthetic pathway.
AT2G33770	*0,731*	PHOSPHATE 2 (PHO2)	Encodes a ubiquitin-conjugating E2 enzyme. UBC24 mRNA accumulation is suppressed by miR399f, miR399b and miR399c. Involved in phosphate starvation response and mediates degradation of PHO1 and PHT1s at endomembrane. Its expression is responsive to phosphate (Pi) and not phosphite (Phi) in roots and shoots. The mRNA is cell-to-cell mobile.

AT2G44450	*0,728*	BETA GLUCOSIDASE 15 (BGLU15)	beta glucosidase 15 (BGLU15); FUNCTIONS IN: cation binding, hydrolase activity, hydrolyzing O-glycosyl compounds, catalytic activity; INVOLVED IN: carbohydrate metabolic process; LOCATED IN: cell wall, plant-type cell wall; EXPRESSED IN: 23 plant structures; EXPRESSED DURING: 14 growth stages; CONTAINS InterPro DOMAIN/s: Glycoside hydrolase, family 1 (InterPro:IPR001360), Glycoside hydrolase, family 1, active site (InterPro:IPR018120), Glycoside hydrolase, catalytic core (InterPro:IPR017853), Glycoside hydrolase, subgroup, catalytic core (InterPro:IPR013781); BEST Arabidopsis thaliana protein match is: beta glucosidase 13 (TAIR:AT5G44640.1)
AT1G74020	*0,727*	STRICTOSIDINE SYNTHASE 2 (SS2)	Encodes AtSS-2 strictosidine synthase.
AT5G57340	*0,721*		unknown protein
AT2G26170	*0,718*	CYTOCHROME P450, FAMILY 711, SUBFAMILY A, POLYPEPTIDE 1 (CYP711A1)	Encodes a protein with similarity to thromboxane-A synthase, member of the CYP711A cytochrome P450 family. MAX1 is a specific repressor of vegetative axillary buds generated by the axillary meristem. Expressed in vascular traces in the rosette stem and axillary buds throughout plant development. Mutants have increased axillary branches. Along with MAX3,4 thought to mediate control of shoot branching via synthesis of a signal molecule which is transported over long distance mediated by MAX2. cDNA supports the existence of the longer transcript predicted for this locus, no cDNA isolated for shorter transcript. MAX1 downregulates 11 genes involved in flavonoid pathway (CHS, CHI, F3H, F3'H, FLS, DFR, ANS, UFGT, RT, AAC and GST).
AT5G13490	*0,718*	ADP/ATP CARRIER 2 (AAC2)	Encodes mitochondrial ADP/ATP carrier
AT2G30570	*0,713*	PHOTOSYSTEM II REACTION CENTER W (PSBW)	Encodes PsbW, a protein similar to photosystem II reaction center subunit W. Loss of PsbW destabilizes the supramolecular organization of PSII.
AT4G19710	*0,711*	ASPARTATE KINASE-HOMOSERINE DEHYDROGENASE II (AK-HSDH II)	Encodes a bifunctional aspartate kinase/homoserine dehydrogenase. These two activities catalyze the first and the third steps toward the synthesis of the essential amino acids threonine, isoleucine and methionine.
AT5G12900	*0,709*		unknown protein; FUNCTIONS IN: molecular_function unknown; INVOLVED IN: biological_process unknown; LOCATED IN: chloroplast; EXPRESSED IN: 21 plant structures; EXPRESSED DURING: 13 growth stages; BEST Arabidopsis thaliana protein match is: unknown protein (TAIR:AT1G12330.1)
AT2G20850	*0,707*	STRUBBELIG-RECEPTOR FAMILY 1 (SRF1)	STRUBBELIG-receptor family 1 (SRF1); FUNCTIONS IN: kinase activity; INVOLVED IN: protein amino acid phosphorylation; LOCATED IN: plasma membrane; EXPRESSED IN: 22 plant structures; EXPRESSED DURING: 13 growth stages; CONTAINS InterPro DOMAIN/s: Serine/threonine-protein kinase domain (InterPro:IPR002290), Leucine-rich repeat-containing N-terminal domain, type 2 (InterPro:IPR013210), Leucine-rich repeat (InterPro:IPR001611), Serine-threonine/tyrosine-protein kinase (InterPro:IPR001245), Protein kinase-like domain (InterPro:IPR011009), Protein kinase, catalytic domain (InterPro:IPR000719), Tyrosine-protein kinase, catalytic domain (InterPro:IPR020635); BEST Arabidopsis thaliana protein match is: STRUBBELIG-receptor family 3 (TAIR:AT4G03390.1)
AT5G20040	*0,704*	ISOPENTENYLTRANSFERASE 9 (IPT9)	Encodes tRNA isopentenyltransferase AtIPT9.
AT3G12500	*0,699*	BASIC CHITINASE (HCHIB)	encodes a basic chitinase involved in ethylene/jasmonic acid mediated signalling pathway during systemic acquired resistance based on expression analyses.
AT2G30590	*0,691*	WRKY DNA-BINDING PROTEIN 21 (WRKY21)	Encodes WRKY DNA-binding protein 21 (WRKY21).

AT3G48730	*0,69*	GLUTAMATE-1-SEMIALDEHYDE 2,1-AMINOMUTASE 2 (GSA2)	glutamate-1-semialdehyde 2,1-aminomutase 2 (GSA2); FUNCTIONS IN: glutamate-1-semialdehyde 2,1-aminomutase activity, pyridoxal phosphate binding, transaminase activity, catalytic activity; INVOLVED IN: porphyrin biosynthetic process; LOCATED IN: chloroplast stroma, chloroplast, chloroplast envelope; EXPRESSED IN: 23 plant structures; EXPRESSED DURING: 15 growth stages; CONTAINS InterPro DOMAIN/s: Pyridoxal phosphate-dependent transferase, major domain (InterPro:IPR015424), Aminotransferase class-III (InterPro:IPR005814), Tetrapyrrole biosynthesis, glutamate-1-semialdehyde aminotransferase (InterPro:IPR004639), Pyridoxal phosphate-dependent transferase, major region, subdomain 1 (InterPro:IPR015421); BEST Arabidopsis thaliana protein match is: glutamate-1-semialdehyde-2,1-aminomutase (TAIR:AT5G63570.1)
AT1G60730	*0,681*		NAD(P)-linked oxidoreductase superfamily protein; FUNCTIONS IN: oxidoreductase activity, aldo-keto reductase activity; INVOLVED IN: oxidation reduction; EXPRESSED IN: 7 plant structures; EXPRESSED DURING: LP.06 six leaves visible, LP.04 four leaves visible, 4 anthesis, petal differentiation and expansion stage; CONTAINS InterPro DOMAIN/s: Aldo/keto reductase (InterPro:IPR001395); BEST Arabidopsis thaliana protein match is: NAD(P)-linked oxidoreductase superfamily protein (TAIR:AT1G60710.1).
AT3G63250	*0,681*	HOMOCYSTEINE METHYLTRANSFERASE 2 (HMT2)	Encodes a homocysteine methyltransferase (HMT). Among the three HMT coding genes in the genome, HMT2 is responsible for a significant proportion of HMT activity in the flower stalks and silique hulls. However, HMT2 does not significantly contribute to the total HMT activity in seeds.
AT5G01410	*0,673*	REDUCED SUGAR RESPONSE 4 (RSR4)	Encodes a protein predicted to function in tandem with PDX2 to form glutamine amidotransferase complex with involved in vitamin B6 biosynthesis.
AT4G27900	*0,672*		CCT motif family protein; FUNCTIONS IN: molecular_function unknown; INVOLVED IN: biological_process unknown; LOCATED IN: cellular_component unknown; EXPRESSED IN: 23 plant structures; EXPRESSED DURING: 13 growth stages; CONTAINS InterPro DOMAIN/s: CCT domain (InterPro:IPR010402); BEST Arabidopsis thaliana protein match is: CCT motif family protein (TAIR:AT5G53420.1)
AT5G43580	*0,671*	UNUSUAL SERINE PROTEASE INHIBITOR (UPI)	Predicted to encode a PR (pathogenesis-related) peptide that belongs to the PR-6 proteinase inhibitor family. Functions in resistance to necrotrophic fungi and insect herbivory. Six putative PR-6-type protein encoding genes are found in Arabidopsis: At2g38900, At2g38870, At5g43570, At5g43580, At3g50020 and At3g46860.
AT1G08290	*0,667*	WIP DOMAIN PROTEIN 3 (WIP3)	WIP domain protein 3 (WIP3); CONTAINS InterPro DOMAIN/s: Zinc finger, C2H2-like (InterPro:IPR015880), Zinc finger, C2H2-type (InterPro:IPR007087); BEST Arabidopsis thaliana protein match is: WIP domain protein 5 (TAIR:AT1G51220.1)
AT3G13730	*0,659*	CYTOCHROME P450, FAMILY 90, SUBFAMILY D, POLYPEPTIDE 1 (CYP90D1)	Encodes a cytochrome P-450 gene that is involved in brassinosteroid biosynthesis, most likely in the conversion step of teasterone (TE) to 3-dehydroteasterone (3DT), and/or 6-deoxoteasterone (6-deoxoTE) to 6-deoxo-3-dehydroteasterone (6-deoxo3DT); or the conversion of cathasterone (CT) to TE, and/or 6-deoxocathasterone (6-deoxoCT) to 6-deoxoTE. Recently, CYP90D1 was shown to catalyse the C-23 hydroxylation of several brassinosteroids (the enzyme has a broad specificity for 22-hydroxylated substrates). Member of the CYP90C CYP450 family. Similar to Cytochrome P450 90C1 (ROT3).
AT5G55530	*0,659*		Calcium-dependent lipid-binding (CaLB domain) family protein; FUNCTIONS IN: molecular_function unknown; INVOLVED IN: biological_process unknown; LOCATED IN: chloroplast; EXPRESSED IN: 22 plant structures; EXPRESSED DURING: 13 growth stages; CONTAINS InterPro DOMAIN/s: C2 calcium/lipid-binding domain, CaLB (InterPro:IPR008973), C2 calcium-dependent membrane targeting (InterPro:IPR000008); BEST Arabidopsis thaliana protein match is: Calcium-dependent lipid-binding (CaLB domain) family protein (TAIR:AT1G50570.2)
AT5G42050	*0,64*		DCD (Development and Cell Death) domain protein; CONTAINS InterPro DOMAIN/s: Development/cell death domain (InterPro:IPR013989), Kelch related (InterPro:IPR013089); BEST Arabidopsis thaliana protein match is: DCD (Development and Cell Death) domain protein (TAIR:AT3G27090.1)
AT1G62750	*0,636*	SNOWY COTYLEDON 1 (SCO1)	Nuclear encoded protein consists of the five domains conserved in EF-G proteins, with two GTP-binding sites in the first domain, and an additional transit peptide at the N-terminus. Localized in chloroplasts. Point mutation results in a delay in the onset of germination. At early developmental stage embryos still contain undifferentiated proplastids. The greening of cotyledons is severely impaired in light-grown mutant sco1 seedlings, whereas the following true leaves develop normally as in wild-type plants.

AT5G09620	*0,626*		Octicosapeptide/Phox/Bem1p family protein; FUNCTIONS IN: molecular_function unknown; INVOLVED IN: biological_process unknown; LOCATED IN: cellular_component unknown; EXPRESSED IN: 23 plant structures; EXPRESSED DURING: 13 growth stages; CONTAINS InterPro DOMAIN/s: Octicosapeptide/Phox/Bem1p (InterPro:IPR000270); BEST Arabidopsis thaliana protein match is: Octicosapeptide/Phox/Bem1p family protein (TAIR:AT5G64430.1)
AT3G61470	*0,623*	PHOTOSYSTEM I LIGHT HARVESTING COMPLEX GENE 2 (LHCA2)	Encodes a component of the light harvesting antenna complex of photosystem I. The mRNA is cell-to-cell mobile.
AT2G03240	*0,611*		EXS (ERD1/XPR1/SYG1) family protein; LOCATED IN: integral to membrane; EXPRESSED IN: 18 plant structures; EXPRESSED DURING: 10 growth stages; CONTAINS InterPro DOMAIN/s: SPX, N-terminal (InterPro:IPR004331), EXS, C-terminal (InterPro:IPR004342); BEST Arabidopsis thaliana protein match is: EXS (ERD1/XPR1/SYG1) family protein (TAIR:AT1G14040.1)
AT2G35940	*0,591*	BEL1-LIKE HOMEODOMAIN 1 (BLH1)	Encodes a member of the BEL-like homeodomain protein family. Ecotopic expression in the embryo sac leads to defects in nuclear migration and cellularization and embryo sacs with multiple egg cells. Loss of function alleles have no female gametophyte defects. The ecotopic expression phenotype requires KNAT3 because it can be suppressed by loss of KNAT3 function alleles. Localized to the nucleus but interaction with OFP1 relocates it to the cytoplasm.
AT1G31812	*-0,562*	ACYL-COA-BINDING PROTEIN 6 (ACBP6)	Acyl-CoA-binding protein. Bind acyl-CoA esters and protect acyl-CoAs from degradation by microsomal acyl-hydrolases.
AT1G63000	*-0,584*	NUCLEOTIDE-RHAMNOSE SYNTHASE/EPIMERASE-REDUCTASE (NRS/ER)	nucleotide-rhamnose synthase/epimerase-reductase (NRS/ER); FUNCTIONS IN: UDP-4-keto-rhamnose-4-keto-reductase activity, dTDP-4-dehydrorhamnose reductase activity, UDP-4-keto-6-deoxy-glucose-3,5-epimerase activity, dTDP-4-dehydrorhamnose 3,5-epimerase activity; INVOLVED IN: dTDP-rhamnose biosynthetic process, UDP-rhamnose biosynthetic process; LOCATED IN: soluble fraction, plasma membrane; EXPRESSED IN: 26 plant structures; EXPRESSED DURING: 16 growth stages; CONTAINS InterPro DOMAIN/s: NAD(P)-binding domain (InterPro:IPR016040), dTDP-4-dehydrorhamnose reductase (InterPro:IPR005913); BEST Arabidopsis thaliana protein match is: rhamnose biosynthesis 1 (TAIR:AT1G78570.1)
AT5G13110	*-0,588*	GLUCOSE-6-PHOSPHATE DEHYDROGENASE 2 (G6PD2)	Encodes a plastidic glucose-6-phosphate dehydrogenase that is sensitive to reduction by DTT and whose mRNA is most highly expressed in root.
AT1G19910	*-0,599*	(AVA-P2)	vacuolar H+-pumping ATPase 16 kDa proteolipid (ava-p2)
AT5G27150	*-0,611*	NA+/H+ EXCHANGER 1 (NHX1)	Encodes a vacuolar sodium/proton antiporter involved in salt tolerance, ion homeostasis, and leaf development. The mRNA is cell-to-cell mobile.
AT2G48130	*-0,616*		Bifunctional inhibitor/lipid-transfer protein/seed storage 2S albumin superfamily protein; FUNCTIONS IN: lipid binding; INVOLVED IN: lipid transport; LOCATED IN: anchored to membrane; EXPRESSED IN: hypocotyl, root, flower; EXPRESSED DURING: petal differentiation and expansion stage; CONTAINS InterPro DOMAIN/s: Bifunctional inhibitor/plant lipid transfer protein/seed storage (InterPro:IPR016140), Plant lipid transfer protein/seed storage/trypsin-alpha amylase inhibitor (InterPro:IPR003612), Plant lipid transfer protein/Par allergen (InterPro:IPR000528), Plant lipid transfer protein/hydrophobic protein, helical domain (InterPro:IPR013770); BEST Arabidopsis thaliana protein match is: Bifunctional inhibitor/lipid-transfer protein/seed storage 2S albumin superfamily protein (TAIR:AT3G22600.1)
AT1G26440	*-0,629*	UREIDE PERMEASE 5 (UPS5)	uptake assays in yeast mutants indicated this splice variant is a cellular importer for allantoin, uracil and xanthine
AT4G38220	*-0,634*	AQUAPORIN INTERACTOR (AQI)	Peptidase M20/M25/M40 family protein; FUNCTIONS IN: hydrolase activity, metallopeptidase activity, aminoacylase activity; INVOLVED IN: response to zinc ion; LOCATED IN: vacuole; EXPRESSED IN: 23 plant structures; EXPRESSED DURING: 15 growth stages; CONTAINS InterPro DOMAIN/s: ArgE/DapE/ACY1/CPG2/YscS, conserved site (InterPro:IPR001261), Peptidase M20 (InterPro:IPR002933), N-acyl-L-amino-acid amidohydrolase (InterPro:IPR010159), Peptidase M20, dimerisation (InterPro:IPR011650); BEST Arabidopsis thaliana protein match is: Peptidase M20/M25/M40 family protein (TAIR:AT1G44820.1)
AT1G01240	*-0,635*		unknown protein; INVOLVED IN: N-terminal protein myristoylation; EXPRESSED IN: 17 plant structures; EXPRESSED DURING: 11 growth stages; BEST Arabidopsis thaliana protein match is: unknown protein (TAIR:AT2G46550.1)
AT5G40850	*-0,635*	UROPHORPHYRIN METHYLASE 1 (UPM1)	Encodes a urophorphyrin III methylase that catalyzes S-adenosyl-L-methionine-dependent transmethylation in a multistep process involving the formation of a covalently linked complex with S-adenosyl-L-methionine. The mRNA is cell-to-cell mobile.

AT4G38550	*-0,639*		Arabidopsis phospholipase-like protein (PEARLI 4) family; LOCATED IN: chloroplast; EXPRESSED IN: 21 plant structures; EXPRESSED DURING: 13 growth stages; CONTAINS InterPro DOMAIN/s: Phospholipase-like, arabidopsis (InterPro:IPR007942); BEST Arabidopsis thaliana protein match is: Arabidopsis phospholipase-like protein (PEARLI 4) family (TAIR:AT2G20950.1)
AT1G79670	*-0,644*	RESISTANCE TO FUSARIUM OXYSPORUM 1 (RFO1)	Encodes a receptor-like kinase that does not contain an extracellular leucine-rich repeat domain. A novel type of dominant disease-resistance protein that confers resistance to a broad spectrum of Fusarium races.
AT1G43710	*-0,645*	EMBRYO DEFECTIVE 1075 (emb1075)	Encodes a serine decarboxylase that is involved in ethanolamine metabolism and is crucial for plant growth.
AT2G32080	*-0,653*	PURIN-RICH ALPHA 1 (PUR ALPHA-1)	similar to the conserved animal nuclear protein PUR alpha which was implicated in the control of gene transcription and DNA replication
AT2G16990	*-0,659*		Major facilitator superfamily protein; FUNCTIONS IN: tetracycline transporter activity; INVOLVED IN: transmembrane transport; LOCATED IN: endomembrane system; EXPRESSED IN: 22 plant structures; EXPRESSED DURING: 13 growth stages; CONTAINS InterPro DOMAIN/s: Major facilitator superfamily MFS-1 (InterPro:IPR011701), Major facilitator superfamily, general substrate transporter (InterPro:IPR016196); BEST Arabidopsis thaliana protein match is: Major facilitator superfamily protein (TAIR:AT2G16980.2)
AT4G21810	*-0,66*	DERLIN-2.1 (DER2.1)	DERLIN-2.1 (DER2.1); CONTAINS InterPro DOMAIN/s: Der1-like (InterPro:IPR007599); BEST Arabidopsis thaliana protein match is: DERLIN-2.2 (TAIR:AT4G04860.1)
AT5G45100	*-0,663*	BOI-RELATED GENE 1 (BRG1)	Encodes one of the BRGs (BOI-related gene) involved in resistance to Botrytis cinerea.
AT1G73390	*-0,664*		Endosomal targeting BRO1-like domain-containing protein; FUNCTIONS IN: molecular_function unknown; INVOLVED IN: N-terminal protein myristoylation; LOCATED IN: cellular_component unknown; EXPRESSED IN: 24 plant structures; EXPRESSED DURING: 13 growth stages; CONTAINS InterPro DOMAIN/s: BRO1 (InterPro:IPR004328); BEST Arabidopsis thaliana protein match is: Endosomal targeting BRO1-like domain-containing protein (TAIR:AT1G17940.1)
AT2G36290	*-0,664*		alpha/beta-Hydrolases superfamily protein; FUNCTIONS IN: hydrolase activity; INVOLVED IN: biological_process unknown; LOCATED IN: endoplasmic reticulum; EXPRESSED IN: 24 plant structures; EXPRESSED DURING: 13 growth stages; CONTAINS InterPro DOMAIN/s: Alpha/beta hydrolase fold-1 (InterPro:IPR000073); BEST Arabidopsis thaliana protein match is: alpha/beta-Hydrolases superfamily protein (TAIR:AT1G74300.1)
AT3G56950	*-0,667*	SMALL AND BASIC INTRINSIC PROTEIN 2;1 (SIP2;1)	One of the Major Intrinsic Proteins(MIPs) which facilitate the passive transport of small molecules across membranes.Belongs to a family of plant aquaporins.Similar to yeast and radish aquaporins. Located on ER.
AT1G19660	*-0,673*	BIFUNCTIONAL NUCLEASE IN BASAL DEFENSE RESPONSE 2 (BBD2)	Wound-responsive family protein; FUNCTIONS IN: DNA binding, nuclease activity; INVOLVED IN: response to wounding, nucleotide-excision repair; EXPRESSED IN: ovule; CONTAINS InterPro DOMAIN/s: Protein of unknown function DUF151 (InterPro:IPR003729), UvrB/UvrC protein (InterPro:IPR001943); BEST Arabidopsis thaliana protein match is: bifunctional nuclease in basal defense response 1 (TAIR:AT1G75380.3)
AT4G38080	*-0,674*		hydroxyproline-rich glycoprotein family protein; FUNCTIONS IN: molecular_function unknown; INVOLVED IN: biological_process unknown; LOCATED IN: endomembrane system; EXPRESSED IN: embryo, hypocotyl, root; EXPRESSED DURING: C globular stage; BEST Arabidopsis thaliana protein match is: hydroxyproline-rich glycoprotein family protein (TAIR:AT2G22510.1)
AT4G27430	*-0,678*	COP1-INTERACTING PROTEIN 7 (CIP7)	Positive regulator of light-regulated genes. Novel nuclear protein which requires light for its high level expression. The mRNA is cell-to-cell mobile.
AT1G30110	*-0,679*	NUDIX HYDROLASE HOMOLOG 25 (NUDX25)	Encodes a ppGpp pyrophosphohydrolase.
AT3G10230	*-0,68*	LYCOPENE CYCLASE (LYC)	Encodes a protein with lycopene β-cyclase activity. This enzyme uses the linear, symmetrical lycopene as substrate. However, unlike the ε-cyclase which adds only one ring, the β-cyclase introduces a ring at both ends of lycopene to form the bicyclic β-carotene.
AT5G53160	*-0,684*	REGULATORY COMPONENTS OF ABA RECEPTOR 3 (RCAR3)	Encodes RCAR3, a regulatory component of ABA receptor. Interacts with protein phosphatase 2Cs ABI1 and ABI2. Stimulates ABA signaling. The mRNA is cell-to-cell mobile.

AT3G55610	*-0,685*	DELTA 1-PYRROLINE-5-CARBOXYLATE SYNTHASE 2 (P5CS2)	encodes delta 1-pyrroline-5-carboxylate synthetase B. Gene expression is induced by dehydration, high salt and ABA. Knock-out mutations in P5CS2 are embryo-lethal. P5CS2 appears to be present in different cells and/or different subcellular locations from P5CS1 in a tissue-dependent manner. Mutants are defective in pollen development.
AT3G14350	*-0,688*	STRUBBELIG-RECEPTOR FAMILY 7 (SRF7)	STRUBBELIG-receptor family 7 (SRF7); FUNCTIONS IN: protein serine/threonine kinase activity, protein kinase activity, ATP binding; INVOLVED IN: transmembrane receptor protein tyrosine kinase signaling pathway, protein amino acid phosphorylation; LOCATED IN: cytosol, nucleus, plasma membrane; EXPRESSED IN: 23 plant structures; EXPRESSED DURING: 13 growth stages; CONTAINS InterPro DOMAIN/s: Protein kinase, ATP binding site (InterPro:IPR017441), Protein kinase, catalytic domain (InterPro:IPR000719), Serine/threonine-protein kinase domain (InterPro:IPR002290), Leucine-rich repeat-containing N-terminal domain, type 2 (InterPro:IPR013210), Leucine-rich repeat (InterPro:IPR001611), Tyrosine-protein kinase, catalytic domain (InterPro:IPR020635), Serine/threonine-protein kinase-like domain (InterPro:IPR017442), Protein kinase-like domain (InterPro:IPR011009); BEST Arabidopsis thaliana protein match is: STRUBBELIG-receptor family 6 (TAIR:AT1G53730.1)
AT1G58030	*-0,689*	CATIONIC AMINO ACID TRANSPORTER 2 (CAT2)	Encodes a member of the cationic amino acid transporter (CAT) subfamily of amino acid polyamine choline transporters. Localized to the tonoplast.
AT4G31450	*-0,697*		RING/U-box superfamily protein; FUNCTIONS IN: zinc ion binding; EXPRESSED IN: 22 plant structures; EXPRESSED DURING: 15 growth stages; CONTAINS InterPro DOMAIN/s: Zinc finger, RING-type (InterPro:IPR001841), Zinc finger, C3HC4 RING-type (InterPro:IPR018957); BEST Arabidopsis thaliana protein match is: RING/U-box superfamily protein (TAIR:AT5G24870.2)
AT3G20080	*-0,699*	CYTOCHROME P450, FAMILY 705, SUBFAMILY A, POLYPEPTIDE 15 (CYP705A15)	member of CYP705A
AT3G07130	*-0,708*	PURPLE ACID PHOSPHATASE 15 (PAP15)	Encodes PAP15, a purple acid phosphatase with phytase activity. Expression of PAP15 is developmentally and temporally regulated, with strong expression at the early stages of seedling growth and pollen germination. The expression is also organ/tissue-specific, with strongest expression in the vasculature, pollen grains, and roots. Recombinant PAP protein exhibits broad substrate specificity with moderate phytase activity. PAP15 likely mobilizes phosphorus reserves in plants, particularly during seed and pollen germination.
AT5G43780	*-0,709*	(APS4)	sulfate adenylyltransferase, ATP sulfurylase
AT3G16470	*-0,718*	JASMONATE RESPONSIVE 1 (JR1)	Encodes a JA-responsive gene that coordinates with GRP7 in shaping plant development through the regulation of RNA processing in Arabidopsis. AtJAC1 interacts with RNA binding protein GRP7 specifically in the cytoplasm to regulate its nucleocytoplasmic distribution.
AT5G09530	*-0,722*	PRO-GLU-LEU|ILE|VAL-PRO-LYS 1 (PELPK1)	The gene encodes a unique protein which contains 36 repeats of a unique pentapeptide (Pro-Glu-Leu|Ile|Val-Pro-Lys). It has been shown tobe involved in growth and development.
AT5G66530	*-0,725*		Galactose mutarotase-like superfamily protein; FUNCTIONS IN: carbohydrate binding, isomerase activity, aldose 1-epimerase activity, catalytic activity; INVOLVED IN: galactose metabolic process, carbohydrate metabolic process; LOCATED IN: apoplast, chloroplast, chloroplast stroma; EXPRESSED IN: 24 plant structures; EXPRESSED DURING: 13 growth stages; CONTAINS InterPro DOMAIN/s: Glycoside hydrolase-type carbohydrate-binding (InterPro:IPR011013), Aldose 1-epimerase (InterPro:IPR008183), Glycoside hydrolase-type carbohydrate-binding, subgroup (InterPro:IPR014718); BEST Arabidopsis thaliana protein match is: Galactose mutarotase-like superfamily protein (TAIR:AT5G57330.1)
AT1G27150	*-0,73*		Tetratricopeptide repeat (TPR)-like superfamily protein; FUNCTIONS IN: binding; INVOLVED IN: biological_process unknown; LOCATED IN: cellular_component unknown; EXPRESSED IN: 23 plant structures; EXPRESSED DURING: 13 growth stages; CONTAINS InterPro DOMAIN/s: Tetratricopeptide-like helical (InterPro:IPR011990); BEST Arabidopsis thaliana protein match is: Tetratricopeptide repeat (TPR)-like superfamily protein (TAIR:AT1G27110.1)
AT3G02290	*-0,731*		RING/U-box superfamily protein; FUNCTIONS IN: zinc ion binding; INVOLVED IN: N-terminal protein myristoylation; EXPRESSED IN: 22 plant structures; EXPRESSED DURING: 13 growth stages; CONTAINS InterPro DOMAIN/s: Zinc finger, RING-type (InterPro:IPR001841), Zinc finger, C3HC4 RING-type (InterPro:IPR018957); BEST Arabidopsis thaliana protein match is: RING/U-box superfamily protein (TAIR:AT5G15790.2)
AT1G65680	*-0,735*	EXPANSIN B2 (EXPB2)	member of BETA-EXPANSINS. Naming convention from the Expansin Working Group (Kende et al, 2004. Plant Mol Bio)

AT3G13040	*-0,735*		myb-like HTH transcriptional regulator family protein; CONTAINS InterPro DOMAIN/s: Homeodomain-like (InterPro:IPR009057), Myb, DNA-binding (InterPro:IPR014778), HTH transcriptional regulator, Myb-type, DNA-binding (InterPro:IPR017930), Myb-like DNA-binding domain, SHAQKYF class (InterPro:IPR006447), Homeodomain-related (InterPro:IPR012287); BEST Arabidopsis thaliana protein match is: phosphate starvation response 1 (TAIR:AT4G28610.1)
AT3G01670	*-0,738*	SIEVE ELEMENT OCCLUSION A (SEOA)	Encodes a protein localized to phloem filaments that is required for phloem filament formation. The mRNA is cell-to-cell mobile.
AT1G15740	*-0,739*		Leucine-rich repeat family protein; BEST Arabidopsis thaliana protein match is: Leucine-rich repeat (LRR) family protein (TAIR:AT4G23840.1)
AT3G04810	*-0,746*	NIMA-RELATED KINASE 2 (NEK2)	Encodes AtNek2, a member of the NIMA-related serine/threonine kinases (Neks) that have been linked to cell-cycle regulation in fungi and mammals. Plant Neks might be involved in plant development processes.
AT2G40110	*-0,747*		Yippee family putative zinc-binding protein; CONTAINS InterPro DOMAIN/s: Yippee-like protein (InterPro:IPR004910); BEST Arabidopsis thaliana protein match is: Yippee family putative zinc-binding protein (TAIR:AT3G11230.2)
AT5G10030	*-0,751*	TGACG MOTIF-BINDING FACTOR 4 (TGA4)	Encodes a member of basic leucine zipper transcription gene family. Nomenclature according to Xiang, et al. (1997).
AT2G39890	*-0,754*	PROLINE TRANSPORTER 1 (PROT1)	Encodes a proline transporter with affinity for gly betaine, proline and GABA. Protein is expressed in the vascular tissue, specifically the phloem.
AT3G54720	*-0,754*	ALTERED MERISTEM PROGRAM 1 (AMP1)	Encodes glutamate carboxypeptidase. Various alleles show-increased cotyledon number and rate of leaf initiation, show transformation of leaves to cotyledons, altered flowering time and photomorphogenesis and an increased level of cytokinin biosynthesis. Involved in ethylene enhanced hypocotyl elongation in the light. Strong genetic interaction between TGH and AMP1.
AT2G16530	*-0,755*	POLYPRENOL REDUCTASE 2 (PPRD2)	Encodes polyprenol reductase involved in N-gylcosylation. Mutants are defective in pollen development. Knockouts are embryo lethal
AT5G06280	*-0,755*		unknown protein; BEST Arabidopsis thaliana protein match is: unknown protein (TAIR:AT3G52520.1)
AT4G21350	*-0,758*	PLANT U-BOX 8 (PUB8)	Encodes a U-box/ARM repeat protein required fore self-incompatibility.
AT1G68710	*-0,762*		ATPase E1-E2 type family protein / haloacid dehalogenase-like hydrolase family protein; FUNCTIONS IN: ATPase activity, coupled to transmembrane movement of ions, phosphorylative mechanism; INVOLVED IN: metabolic process, phospholipid transport, ATP biosynthetic process; LOCATED IN: mitochondrion, integral to membrane, membrane; EXPRESSED IN: 23 plant structures; EXPRESSED DURING: 15 growth stages; CONTAINS InterPro DOMAIN/s: Haloacid dehalogenase-like hydrolase (InterPro:IPR005834), ATPase, P-type, phospholipid-translocating, flippase (InterPro:IPR006539), ATPase, P-type, ATPase-associated domain (InterPro:IPR008250), ATPase, P-type, K/Mg/Cd/Cu/Zn/Na/Ca/Na/H-transporter (InterPro:IPR001757), ATPase, P-type phosphorylation site (InterPro:IPR018303); BEST Arabidopsis thaliana protein match is: ATPase E1-E2 type family protein / haloacid dehalogenase-like hydrolase family protein (TAIR:AT1G26130.2)
AT1G78680	*-0,762*	GAMMA-GLUTAMYL HYDROLASE 2 (GGH2)	The Arabidopsis protein AtGGH2 is a gamma-glutamyl hydrolase acting specifically on monoglutamates. The enzyme is involved in the tetrahydrofolate metabolism and located to the vacuole.
AT4G26130	*-0,762*		unknown protein; FUNCTIONS IN: molecular_function unknown; INVOLVED IN: biological_process unknown; LOCATED IN: endomembrane system; EXPRESSED IN: 23 plant structures; EXPRESSED DURING: 13 growth stages; BEST Arabidopsis thaliana protein match is: unknown protein (TAIR:AT5G56980.1)
AT2G35860	*-0,765*	FASCICLIN-LIKE ARABINOGALACTAN PROTEIN 16 PRECURSOR (FLA16)	FASCICLIN-like arabinogalactan protein 16 precursor (FLA16); INVOLVED IN: cell adhesion; LOCATED IN: endomembrane system; EXPRESSED IN: 22 plant structures; EXPRESSED DURING: 13 growth stages; CONTAINS InterPro DOMAIN/s: FAS1 domain (InterPro:IPR000782); BEST Arabidopsis thaliana protein match is: FASCICLIN-like arabinogalactan protein 15 precursor (TAIR:AT3G52370.1)
AT4G15920	*-0,765*	(SWEET17)	Encodes a vacuolar fructose transporter expressed in parenchyma and xylem that controls leaf fructose content. When its expression is reduced, fructose accumulates in leaves.

AT1G22790	*-0,767*		unknown protein; FUNCTIONS IN: molecular_function unknown; INVOLVED IN: biological_process unknown; LOCATED IN: plasma membrane; EXPRESSED IN: 22 plant structures; EXPRESSED DURING: 13 growth stages; BEST Arabidopsis thaliana protein match is: unknown protein (TAIR:AT1G34010.1)
AT2G46430	*-0,769*	CYCLIC NUCLEOTIDE GATED CHANNEL 3 (CNGC3)	Encodes a cyclic nucleotide gated channel, downstream component of the signaling pathways leading to hypersensitive response (HR) resistance.
AT4G30190	*-0,769*	H(+)-ATPASE 2 (HA2)	belongs to the P-type ATPase superfamily of cation-transporting ATPases, pumps protons out of the cell, generating a proton gradient that drives the active transport of nutrients by proton symport. has two autoinhibitory regions within the C-terminal dom
AT2G30100	*-0,774*		pentatricopeptide (PPR) repeat-containing protein; LOCATED IN: chloroplast; EXPRESSED IN: 22 plant structures; EXPRESSED DURING: 13 growth stages; CONTAINS InterPro DOMAIN/s: Pentatricopeptide repeat (InterPro:IPR002885); BEST Arabidopsis thaliana protein match is: pentatricopeptide (PPR) repeat-containing protein (TAIR:AT5G04810.1)
AT2G48140	*-0,774*	EMBRYO SAC DEVELOPMENT ARREST 4 (EDA4)	embryo sac development arrest 4 (EDA4); CONTAINS InterPro DOMAIN/s: Bifunctional inhibitor/plant lipid transfer protein/seed storage (InterPro:IPR016140), Plant lipid transfer protein/seed storage/trypsin-alpha amylase inhibitor (InterPro:IPR003612); BEST Arabidopsis thaliana protein match is: Bifunctional inhibitor/lipid-transfer protein/seed storage 2S albumin superfamily protein (TAIR:AT1G05450.2)
AT3G53090	*-0,775*	UBIQUITIN-PROTEIN LIGASE 7 (UPL7)	encodes a ubiquitin-protein ligase containing a HECT domain. There are six other HECT-domain UPLs in Arabidopsis.
AT1G05850	*-0,777*	POM-POM1 (POM1)	Encodes an endo chitinase-like protein AtCTL1. Essential for tolerance to heat, salt and drought stresses. Also involved in root hair development, cell expansion and response to cytokinin. Allelic to erh2. 11 alleles described in Hauser (1995). Mutant is defective in acquired thermotolerance, appears semidwarf throughout its life cycle and has extra lateral branches. There are two EMS alleles. Expression of AtHSP101 is not affected in the mutants.
AT3G10740	*-0,779*	ALPHA-L-ARABINOFURANOSIDASE 1 (ASD1)	Encodes a bifunctional alpha-l-arabinofuranosidase/beta-d-xylosidase that belongs to family 51 of glycoside hydrolases. It may be involved in cell wall modification.
AT5G20700	*-0,779*		Protein of unknown function (DUF581); CONTAINS InterPro DOMAIN/s: Protein of unknown function DUF581 (InterPro:IPR007650); BEST Arabidopsis thaliana protein match is: Protein of unknown function (DUF581) (TAIR:AT1G74940.1)
AT3G45600	*-0,781*	TETRASPANIN3 (TET3)	Member of TETRASPANIN family
AT4G26540	*-0,786*	(RGFR3)	Leucine-rich repeat receptor-like protein kinase family protein; FUNCTIONS IN: kinase activity; INVOLVED IN: protein amino acid phosphorylation; LOCATED IN: plasma membrane; EXPRESSED IN: inflorescence meristem; CONTAINS InterPro DOMAIN/s: Protein kinase, ATP binding site (InterPro:IPR017441), Protein kinase, catalytic domain (InterPro:IPR000719), Leucine-rich repeat-containing N-terminal domain, type 2 (InterPro:IPR013210), Leucine-rich repeat (InterPro:IPR001611), Serine/threonine-protein kinase-like domain (InterPro:IPR017442), Protein kinase-like domain (InterPro:IPR011009), Serine/threonine-protein kinase, active site (InterPro:IPR008271); BEST Arabidopsis thaliana protein match is: Leucine-rich receptor-like protein kinase family protein (TAIR:AT5G56040.2)
AT4G35560	*-0,788*	DUO1-ACTIVATED WD40 1 (DAW1)	Target promoter of the male germline-specific transcription factor DUO1. The mRNA is cell-to-cell mobile.
AT4G02410	*-0,792*	L-TYPE LECTIN RECEPTOR KINASE IV.3 (LECRK-IV.3)	Concanavalin A-like lectin protein kinase family protein; FUNCTIONS IN: kinase activity; INVOLVED IN: protein amino acid phosphorylation; LOCATED IN: endomembrane system; EXPRESSED IN: 19 plant structures; EXPRESSED DURING: 10 growth stages; CONTAINS InterPro DOMAIN/s: Legume lectin, beta chain (InterPro:IPR001220), Protein kinase, ATP binding site (InterPro:IPR017441), Serine/threonine-protein kinase-like domain (InterPro:IPR017442), Concanavalin A-like lectin/glucanase, subgroup (InterPro:IPR013320), Protein kinase-like domain (InterPro:IPR011009), Serine/threonine-protein kinase, active site (InterPro:IPR008271), Protein kinase, catalytic domain (InterPro:IPR000719), Concanavalin A-like lectin/glucanase (InterPro:IPR008985), Legume lectin, beta chain, Mn/Ca-binding site (InterPro:IPR019825); BEST Arabidopsis thaliana protein match is: Concanavalin A-like lectin protein kinase family protein (TAIR:AT4G02420.1)

AT5G63990	*-0,793*		Inositol monophosphatase family protein; FUNCTIONS IN: 3'(2'),5'-bisphosphate nucleotidase activity, inositol or phosphatidylinositol phosphatase activity; INVOLVED IN: sulfur metabolic process; LOCATED IN: cellular_component unknown; EXPRESSED IN: 17 plant structures; EXPRESSED DURING: 6 growth stages; CONTAINS InterPro DOMAIN/s: Inositol monophosphatase (InterPro:IPR000760), 3(2),5 -bisphosphate nucleotidase HAL2 (InterPro:IPR006239); BEST Arabidopsis thaliana protein match is: Inositol monophosphatase family protein (TAIR:AT5G64000.1)
AT1G17860	*-0,794*		Kunitz family trypsin and protease inhibitor protein; FUNCTIONS IN: endopeptidase inhibitor activity; INVOLVED IN: biological_process unknown; LOCATED IN: apoplast, cell wall; EXPRESSED IN: 17 plant structures; EXPRESSED DURING: 13 growth stages; CONTAINS InterPro DOMAIN/s: Proteinase inhibitor I3, Kunitz legume (InterPro:IPR002160), Kunitz inhibitor ST1-like (InterPro:IPR011065); BEST Arabidopsis thaliana protein match is: kunitz trypsin inhibitor 1 (TAIR:AT1G73260.1)
AT4G15340	*-0,794*	PENTACYCLIC TRITERPENE SYNTHASE 1 (PEN1)	Encodes a protein that catalyzes the production of the tricyclic triterpene arabidiol when expressed in yeast.
AT1G02950	*-0,795*	GLUTATHIONE S-TRANSFERASE F4 (GSTF4)	Encodes glutathione transferase belonging to the phi class of GSTs. Naming convention according to Wagner et al. (2002).
AT1G27180	*-0,798*		disease resistance protein (TIR-NBS-LRR class), putative; FUNCTIONS IN: transmembrane receptor activity, ATP binding; INVOLVED IN: signal transduction, apoptosis, defense response, innate immune response; LOCATED IN: intrinsic to membrane; CONTAINS InterPro DOMAIN/s: NB-ARC (InterPro:IPR002182), Leucine-rich repeat, typical subtype (InterPro:IPR003591), Disease resistance protein (InterPro:IPR000767), Toll-Interleukin receptor (InterPro:IPR000157); BEST Arabidopsis thaliana protein match is: transmembrane receptors;ATP binding (TAIR:AT1G27170.1)
AT5G46790	*-0,798*	PYR1-LIKE 1 (PYL1)	Encodes a member of the PYR (pyrabactin resistance)/PYL(PYR1-like)/RCAR (regulatory components of ABA receptor) family proteins with 14 members. PYR/PYL/RCAR family proteins function as abscisic acid sensors. Mediate ABA-dependent regulation of protein phosphatase 2Cs ABI1 and ABI2.
AT1G24440	*-0,811*		RING/U-box superfamily protein; FUNCTIONS IN: zinc ion binding; EXPRESSED IN: 24 plant structures; EXPRESSED DURING: 15 growth stages; CONTAINS InterPro DOMAIN/s: Zinc finger, RING-type, conserved site (InterPro:IPR017907), Zinc finger, RING-type (InterPro:IPR001841); BEST Arabidopsis thaliana protein match is: RING/U-box superfamily protein (TAIR:AT1G13195.1)
AT1G26920	*-0,811*		unknown protein; BEST Arabidopsis thaliana protein match is: unknown protein (TAIR:AT1G69760.1)
AT1G80180	*-0,812*		Encodes a substrate of the MAPK kinases. Phenotypic analyses of Arabidopsis expressing phosphorylation site mutant forms of At1g80180.1 showed clustered stomata and higher stomatal index in cotyledons expressing the phosphomimetic form of At1g80180.1.
AT1G30130	*-0,814*		unknown protein; FUNCTIONS IN: molecular_function unknown; INVOLVED IN: biological_process unknown; LOCATED IN: endomembrane system; EXPRESSED IN: 23 plant structures; EXPRESSED DURING: 13 growth stages; CONTAINS InterPro DOMAIN/s: Protein of unknown function DUF1365 (InterPro:IPR010775)
AT4G37540	*-0,815*	LOB DOMAIN-CONTAINING PROTEIN 39 (LBD39)	LOB domain-containing protein 39 (LBD39); CONTAINS InterPro DOMAIN/s: Lateral organ boundaries, LOB (InterPro:IPR004883); BEST Arabidopsis thaliana protein match is: LOB domain-containing protein 38 (TAIR:AT3G49940.1)
AT1G77130	*-0,816*	PLANT GLYCOGENIN-LIKE STARCH INITIATION PROTEIN 2 (PGSIP2)	Encodes a glucuronyltransferase responsible for the addition of GlcA residues onto xylan and for secondary wall deposition.
AT2G23030	*-0,816*	SNF1-RELATED PROTEIN KINASE 2.9 (SNRK2.9)	encodes a member of SNF1-related protein kinases (SnRK2)
AT5G50010	*-0,816*	(SACL2)	sequence-specific DNA binding transcription factors;transcription regulators; BEST Arabidopsis thaliana protein match is: sequence-specific DNA binding transcription factors;transcription regulators (TAIR:AT5G64340.1)
AT5G55960	*-0,816*		unknown protein; CONTAINS InterPro DOMAIN/s: Uncharacterised protein family UPF0118 (InterPro:IPR002549)
AT2G42060	*-0,824*		Cysteine/Histidine-rich C1 domain family protein; INVOLVED IN: intracellular signaling pathway; LOCATED IN: cellular_component unknown; EXPRESSED IN: root; CONTAINS InterPro DOMAIN/s: Protein kinase C-like, phorbol ester/diacylglycerol binding (InterPro:IPR002219), C1-like (InterPro:IPR011424); BEST Arabidopsis thaliana protein match is: Cysteine/Histidine-rich C1 domain family protein (TAIR:AT2G27660.1)

AT3G53240	*-0,824*	RECEPTOR LIKE PROTEIN 45 (RLP45)	receptor like protein 45 (RLP45); CONTAINS InterPro DOMAIN/s: Leucine-rich repeat, typical subtype (InterPro:IPR003591), Leucine-rich repeat (InterPro:IPR001611); BEST Arabidopsis thaliana protein match is: receptor like protein 15 (TAIR:AT1G74190.1)
AT5G12250	*-0,825*	BETA-6 TUBULIN (TUB6)	Encodes a beta-tubulin. Expression of TUB6 has been shown to decrease in response to cold treatment.
AT2G03730	*-0,826*	ACT DOMAIN REPEAT 5 (ACR5)	Member of a small family of ACT domain containing proteins. ACT domains are thought to be involved in amino acid binding.
AT3G47820	*-0,827*	PLANT U-BOX 39 (PUB39)	PLANT U-BOX 39 (PUB39); FUNCTIONS IN: ubiquitin-protein ligase activity, binding; INVOLVED IN: protein ubiquitination; LOCATED IN: ubiquitin ligase complex; CONTAINS InterPro DOMAIN/s: U box domain (InterPro:IPR003613), Armadillo-like helical (InterPro:IPR011989), Armadillo (InterPro:IPR000225), Armadillo-type fold (InterPro:IPR016024); BEST Arabidopsis thaliana protein match is: RING/U-box superfamily protein with ARM repeat domain (TAIR:AT5G62560.1)
AT4G02075	*-0,827*	PITCHOUN 1 (PIT1)	pitchoun 1 (PIT1); FUNCTIONS IN: zinc ion binding; LOCATED IN: plasma membrane; EXPRESSED IN: 18 plant structures; EXPRESSED DURING: 10 growth stages; CONTAINS InterPro DOMAIN/s: Protein of unknown function DUF3675 (InterPro:IPR022143), Zinc finger, C3HC4 RING-type (InterPro:IPR018957), Zinc finger, RING-CH-type (InterPro:IPR011016); BEST Arabidopsis thaliana protein match is: RING/FYVE/PHD zinc finger superfamily protein (TAIR:AT1G02610.1)
AT4G35920	*-0,828*	MID1-COMPLEMENTING ACTIVITY 1 (MCA1)	Encodes an integral plasma membrane protein. Functionally complements the yeast mid1 mutant, a deficiency of Ca2+ influx. Involved in Ca2+ influx and mechanical sensing in roots. An over-expression line showed increased Ca2+ uptake than the wild type plant. The primary root of a knock-out mutant failed to penetrate a harder agar medium from a softer medium.
AT4G37730	*-0,83*	BASIC LEUCINE-ZIPPER 7 (bZIP7)	basic leucine-zipper 7 (bZIP7); FUNCTIONS IN: DNA binding, sequence-specific DNA binding transcription factor activity; INVOLVED IN: regulation of transcription, DNA-dependent; EXPRESSED IN: 19 plant structures; EXPRESSED DURING: 7 growth stages; CONTAINS InterPro DOMAIN/s: Basic-leucine zipper (bZIP) transcription factor (InterPro:IPR004827), bZIP transcription factor, bZIP-1 (InterPro:IPR011616); BEST Arabidopsis thaliana protein match is: basic leucine-zipper 6 (TAIR:AT2G22850.2)
AT1G76990	*-0,834*	ACT DOMAIN REPEAT 3 (ACR3)	ACT domain repeat 3 (ACR3); FUNCTIONS IN: amino acid binding; INVOLVED IN: nitrogen compound metabolic process, metabolic process; LOCATED IN: cytosol; EXPRESSED IN: 22 plant structures; EXPRESSED DURING: 13 growth stages; CONTAINS InterPro DOMAIN/s: Protein-PII uridylyltransferase (InterPro:IPR010043), Amino acid-binding ACT (InterPro:IPR002912); BEST Arabidopsis thaliana protein match is: ACT domain repeat 4 (TAIR:AT1G69040.2)
AT3G06590	*-0,837*	ATBS1 INTERACTING FACTOR 2 (AIF2)	Encodes RITF1, a bHLH transcription factor that regulates the transcription of several genes involved in the detoxification of reactive oxygen species generated by salt stress.
AT1G22750	*-0,84*		unknown protein; CONTAINS InterPro DOMAIN/s: Protein of unknown function DUF1475 (InterPro:IPR009943)
AT4G17350	*-0,84*		CONTAINS InterPro DOMAIN/s: Pleckstrin-like, plant (InterPro:IPR013666), Protein of unknown function DUF828 (InterPro:IPR008546), Pleckstrin homology (InterPro:IPR001849); BEST Arabidopsis thaliana protein match is: Plant protein of unknown function (DUF828) with plant pleckstrin homology-like region (TAIR:AT5G47440.1)
AT2G20340	*-0,842*	AROMATIC ALDEHYDE SYNTHASE (AAS)	Encodes an aromatic aldehyde synthase (AtAAS), which catalyzes the in vitro conversion of phenylalanine and 3,4-dihydroxy-L-phenylalanine to phenylacetaldehyde and dopaldehyde, respectively. The mRNA is cell-to-cell mobile.
AT5G04200	*-0,842*	METACASPASE 9 (MC9)	Encodes a putative metacaspase. Arabidopsis contains three type I MCP genes (MCP1a-c) and six type II MCP genes (MCP2a?f): AtMCP1a/At5g64240, AtMCP1b/At1g02170, AtMCP1c/At4g25110, AtMCP2a/At1g79310, AtMCP2b/At1g79330, AtMCP2c/At1g79320, AtMCP2d/At1g79340, AtMCP2e/At1g16420, AtMCP2f/At5g04200.
AT1G32700	*-0,844*		PLATZ transcription factor family protein; CONTAINS InterPro DOMAIN/s: Protein of unknown function DUF597 (InterPro:IPR006734); BEST Arabidopsis thaliana protein match is: PLATZ transcription factor family protein (TAIR:AT4G17900.1)
AT3G01860	*-0,845*		unknown protein; FUNCTIONS IN: molecular_function unknown; INVOLVED IN: response to cadmium ion; LOCATED IN: chloroplast; EXPRESSED IN: 24 plant structures; EXPRESSED DURING: 13 growth stages; BEST Arabidopsis thaliana protein match is: unknown protein (TAIR:AT3G27210.1)
AT3G52370	*-0,845*	FASCICLIN-LIKE ARABINOGALACTAN PROTEIN 15 PRECURSOR (FLA15)	FASCICLIN-like arabinogalactan protein 15 precursor (FLA15); INVOLVED IN: cell adhesion; LOCATED IN: endomembrane system; EXPRESSED IN: 21 plant structures; EXPRESSED DURING: 13 growth stages; CONTAINS InterPro DOMAIN/s: FAS1 domain (InterPro:IPR000782); BEST Arabidopsis thaliana protein match is: FASCICLIN-like arabinogalactan protein 16 precursor (TAIR:AT2G35860.1)

AT1G09740	*-0,847*		Adenine nucleotide alpha hydrolases-like superfamily protein; FUNCTIONS IN: molecular_function unknown; INVOLVED IN: response to stress; LOCATED IN: cellular_component unknown; EXPRESSED IN: 13 plant structures; EXPRESSED DURING: 7 growth stages; CONTAINS InterPro DOMAIN/s: UspA (InterPro:IPR006016), Rossmann-like alpha/beta/alpha sandwich fold (InterPro:IPR014729), Universal stress protein A (InterPro:IPR006015); BEST Arabidopsis thaliana protein match is: Adenine nucleotide alpha hydrolases-like superfamily protein (TAIR:AT3G11930.1)
AT1G16170	*-0,847*		unknown protein; FUNCTIONS IN: molecular_function unknown; INVOLVED IN: biological_process unknown; LOCATED IN: cellular_component unknown; EXPRESSED IN: 24 plant structures; EXPRESSED DURING: 15 growth stages; BEST Arabidopsis thaliana protein match is: unknown protein (TAIR:AT1G79660.1)
AT2G04800	*-0,847*		unknown protein; FUNCTIONS IN: molecular_function unknown; INVOLVED IN: N-terminal protein myristoylation; LOCATED IN: cellular_component unknown; EXPRESSED IN: hypocotyl, root
AT1G64190	*-0,85*	6-PHOSPHOGLUCONATE DEHYDROGENASE 1 (PGD1)	6-phosphogluconate dehydrogenase family protein; FUNCTIONS IN: in 6 functions; INVOLVED IN: response to salt stress; LOCATED IN: chloroplast, membrane; EXPRESSED IN: 24 plant structures; EXPRESSED DURING: 14 growth stages; CONTAINS InterPro DOMAIN/s: 6-phosphogluconate dehydrogenase, NAD-binding (InterPro:IPR006115), 6-phosphogluconate dehydrogenase, C-terminal-like (InterPro:IPR008927), Dehydrogenase, multihelical (InterPro:IPR013328), 6-phosphogluconate dehydrogenase, decarboxylating (InterPro:IPR006113), 6-phosphogluconate dehydrogenase, C-terminal (InterPro:IPR006114), 6-phosphogluconate dehydrogenase (InterPro:IPR006183), NAD(P)-binding domain (InterPro:IPR016040), Fibritin/6-phosphogluconate dehydrogenase, C-terminal extension (InterPro:IPR012284); BEST Arabidopsis thaliana protein match is: 6-phosphogluconate dehydrogenase family protein (TAIR:AT5G41670.2)
AT1G75130	*-0,85*	CYTOCHROME P450, FAMILY 721, SUBFAMILY A, POLYPEPTIDE 1 (CYP721A1)	member of CYP721A
AT2G03200	*-0,85*		Eukaryotic aspartyl protease family protein; FUNCTIONS IN: aspartic-type endopeptidase activity; INVOLVED IN: proteolysis; LOCATED IN: endomembrane system; EXPRESSED IN: 10 plant structures; EXPRESSED DURING: 4 anthesis, C globular stage, petal differentiation and expansion stage; CONTAINS InterPro DOMAIN/s: Peptidase aspartic (InterPro:IPR021109), Peptidase aspartic, catalytic (InterPro:IPR009007), Peptidase A1 (InterPro:IPR001461); BEST Arabidopsis thaliana protein match is: Eukaryotic aspartyl protease family protein (TAIR:AT1G01300.1)
AT3G26300	*-0,85*	CYTOCHROME P450, FAMILY 71, SUBFAMILY B, POLYPEPTIDE 34 (CYP71B34)	putative cytochrome P450
AT3G21510	*-0,852*	HISTIDINE-CONTAINING PHOSPHOTRANSMITTER 1 (AHP1)	Encodes AHP1, one of the six Arabidopsis thaliana histidine phosphotransfer proteins (AHPs). AHPs function as redundant positive regulators of cytokinin signaling. Members of the AHP gene family include: AT3G21510 (AHP1), AT3G29350 (AHP2), AT5G39340 (AHP3), AT3G16360 (AHP4), AT1G03430 (AHP5) and AT1G80100 (AHP6).
AT5G47050	*-0,853*		SBP (S-ribonuclease binding protein) family protein; FUNCTIONS IN: zinc ion binding; EXPRESSED IN: 22 plant structures; EXPRESSED DURING: 13 growth stages; CONTAINS InterPro DOMAIN/s: Zinc finger, RING-type (InterPro:IPR001841), S-ribonuclease binding protein, SBP1, pollen (InterPro:IPR017066); BEST Arabidopsis thaliana protein match is: SBP (S-ribonuclease binding protein) family protein (TAIR:AT4G17680.1)
AT5G64000	*-0,853*	(SAL2)	3'(2'),5'-bisphosphate nucleotidase
AT1G24100	*-0,865*	UDP-GLUCOSYL TRANSFERASE 74B1 (UGT74B1)	Encodes a UDP-glucose:thiohydroximate S-glucosyltransferase, involved in glucosinolate biosynthesis
AT5G06850	*-0,867*	FT-INTERACTING PROTEIN 1 (FTIP1)	Encodes an endoplasmic reticulum protein that is involved in the transport of the florigen FT from companion cells to sieve elements, thus affecting FT transport through the phloem to the SAM.
AT5G65480	*-0,868*	CLAVATA COMPLEX INTERACTOR 1 (CCI1)	unknown protein; FUNCTIONS IN: molecular_function unknown; INVOLVED IN: biological_process unknown; LOCATED IN: chloroplast; EXPRESSED IN: 24 plant structures; EXPRESSED DURING: 15 growth stages; BEST Arabidopsis thaliana protein match is: unknown protein (TAIR:AT4G38060.2)

AT1G78380	*-0,876*	GLUTATHIONE S-TRANSFERASE TAU 19 (GSTU19)	Encodes a glutathione transferase that is a member of Tau GST gene family. Expression is induced by drought stress, oxidative stress, and high doses of auxin and cytokinin. naming convention according to Wagner et al. (2002) The expression of this gene is upregulated by herbicide safeners such as benoxacor and fenclorim.
AT1G62770	*-0,878*	(PMEI9)	Plant invertase/pectin methylesterase inhibitor superfamily protein; FUNCTIONS IN: enzyme inhibitor activity, pectinesterase inhibitor activity, pectinesterase activity; INVOLVED IN: biological_process unknown; LOCATED IN: endomembrane system; EXPRESSED IN: 16 plant structures; EXPRESSED DURING: 8 growth stages; CONTAINS InterPro DOMAIN/s: Pectinesterase inhibitor (InterPro:IPR006501); BEST Arabidopsis thaliana protein match is: pectin methylesterase inhibitor 1 (TAIR:AT4G12390.1)
AT2G02710	*-0,879*	PAS/LOV PROTEIN B (PLPB)	Encodes a putative blue light receptor protein.
AT1G79470	*-0,881*		Aldolase-type TIM barrel family protein; FUNCTIONS IN: IMP dehydrogenase activity, oxidoreductase activity, catalytic activity; INVOLVED IN: oxidation reduction, GMP biosynthetic process, metabolic process; EXPRESSED IN: 17 plant structures; EXPRESSED DURING: 8 growth stages; CONTAINS InterPro DOMAIN/s: IMP dehydrogenase (InterPro:IPR005990), IMP dehydrogenase related (InterPro:IPR018529), Aldolase-type TIM barrel (InterPro:IPR013785), IMP dehydrogenase/GMP reductase (InterPro:IPR001093), IMP dehydrogenase / GMP reductase, conserved site (InterPro:IPR015875); BEST Arabidopsis thaliana protein match is: Aldolase-type TIM barrel family protein (TAIR:AT1G16350.1)
AT5G65830	*-0,881*	RECEPTOR LIKE PROTEIN 57 (RLP57)	receptor like protein 57 (RLP57); INVOLVED IN: signal transduction; LOCATED IN: endomembrane system; EXPRESSED IN: 21 plant structures; EXPRESSED DURING: 13 growth stages; CONTAINS InterPro DOMAIN/s: Leucine-rich repeat (InterPro:IPR001611); BEST Arabidopsis thaliana protein match is: receptor like protein 44 (TAIR:AT3G49750.1)
AT1G18910	*-0,882*		zinc ion binding;zinc ion binding; FUNCTIONS IN: zinc ion binding; EXPRESSED IN: 15 plant structures; EXPRESSED DURING: 7 growth stages; CONTAINS InterPro DOMAIN/s: Zinc finger, CTCHY-type (InterPro:IPR017921), Zinc finger, CHY-type (InterPro:IPR008913), Zinc finger, RING-type (InterPro:IPR001841), Haemerythrin/HHE cation-binding motif (InterPro:IPR012312); BEST Arabidopsis thaliana protein match is: zinc ion binding (TAIR:AT1G74770.1)
AT4G05070	*-0,883*		Wound-responsive family protein; FUNCTIONS IN: molecular_function unknown; INVOLVED IN: biological_process unknown; LOCATED IN: chloroplast; EXPRESSED IN: 22 plant structures; EXPRESSED DURING: 13 growth stages; CONTAINS InterPro DOMAIN/s: Protein of unknown function wound-induced (InterPro:IPR022251)
AT4G27740	*-0,883*		Yippee family putative zinc-binding protein; CONTAINS InterPro DOMAIN/s: Yippee-like protein (InterPro:IPR004910); BEST Arabidopsis thaliana protein match is: Yippee family putative zinc-binding protein (TAIR:AT4G27745.1)
AT5G42860	*-0,883*	COMPANION OF CELLULOSE SYNTHASE 2 (CC2)	CC2 is a plant specific gene that interacts with with the cellulose synthase complex and microtubules. It appears to play a role in localizing CESA to the membrane, microtuble dynamics , particularly during salt stress.
AT1G59590	*-0,887*	(ZCF37)	ZCF37 mRNA, complete cds The mRNA is cell-to-cell mobile.
AT5G57630	*-0,887*	CBL-INTERACTING PROTEIN KINASE 21 (CIPK21)	CBL-interacting protein kinase.When mutated plants are hypersensitive to salt and osmotic stress.
AT1G74090	*-0,888*	DESULFO-GLUCOSINOLATE SULFOTRANSFERASE 18 (SOT18)	encodes a desulfoglucosinolate sulfotransferase, involved in the final step of glucosinolate core structure biosynthesis. Has a broad-substrate specificity with preference with methionine-derived desulfoglucosinolates.
AT5G57685	*-0,891*	GLUTAMINE DUMPER 3 (GDU3)	Encodes a member of the GDU (glutamine dumper) family proteins involved in amino acid export: At4g31730 (GDU1), At4g25760 (GDU2), At5g57685 (GDU3), At2g24762 (GDU4), At5g24920 (GDU5), At3g30725 (GDU6) and At5g38770 (GDU7).
AT2G38700	*-0,892*	MEVALONATE DIPHOSPHATE DECARBOXYLASE 1 (MVD1)	Encodes mevalonate diphosphate decarboxylase, the enzyme that catalyzes the synthesis of isopentenyl diphosphate, used in sterol and isoprenoid biosynthesis. The protein appears to form a homodimeric complex. Incidentally, it was shown that the Arabidopsis MVD protein could also interact with its yeast homolog to form a heterodimer.
AT2G37440	*-0,896*		DNAse I-like superfamily protein; FUNCTIONS IN: hydrolase activity, inositol or phosphatidylinositol phosphatase activity; INVOLVED IN: biological_process unknown; EXPRESSED IN: sperm cell, root; CONTAINS InterPro DOMAIN/s: Inositol polyphosphate related phosphatase (InterPro:IPR000300), Endonuclease/exonuclease/phosphatase (InterPro:IPR005135); BEST Arabidopsis thaliana protein match is: DNAse I-like superfamily protein (TAIR:AT2G01900.1)

AT2G15890	*-0,9*	MATERNAL EFFECT EMBRYO ARREST 14 (MEE14)	Encodes CBP1, a regulator of transcription initiation in central cell-mediated pollen tube guidance.
AT4G13860	*-0,9*		RNA-binding (RRM/RBD/RNP motifs) family protein; FUNCTIONS IN: RNA binding, nucleotide binding, nucleic acid binding; INVOLVED IN: biological_process unknown; LOCATED IN: cellular_component unknown; CONTAINS InterPro DOMAIN/s: RNA recognition motif, glycine rich protein (InterPro:IPR015465), RNA recognition motif, RNP-1 (InterPro:IPR000504), Nucleotide-binding, alpha-beta plait (InterPro:IPR012677); BEST Arabidopsis thaliana protein match is: glycine-rich RNA-binding protein 2 (TAIR:AT4G13850.4)
AT1G76560	*-0,901*	CP12 DOMAIN-CONTAINING PROTEIN 3 (CP12-3)	CP12 domain-containing protein 3 (CP12-3); FUNCTIONS IN: molecular_function unknown; INVOLVED IN: negative regulation of reductive pentose-phosphate cycle; LOCATED IN: chloroplast; EXPRESSED IN: 20 plant structures; EXPRESSED DURING: 13 growth stages; CONTAINS InterPro DOMAIN/s: Protein of unknown function CP12 (InterPro:IPR003823); BEST Arabidopsis thaliana protein match is: CP12 domain-containing protein 1 (TAIR:AT2G47400.1)
AT3G32980	*-0,901*		Peroxidase superfamily protein; FUNCTIONS IN: peroxidase activity, heme binding; INVOLVED IN: response to oxidative stress, oxidation reduction; LOCATED IN: cell wall, vacuole, membrane; EXPRESSED IN: leaf; EXPRESSED DURING: seedling growth; CONTAINS InterPro DOMAIN/s: Haem peroxidase (InterPro:IPR010255), Plant peroxidase (InterPro:IPR000823), Peroxidases heam-ligand binding site (InterPro:IPR019793), Peroxidase, active site (InterPro:IPR019794), Haem peroxidase, plant/fungal/bacterial (InterPro:IPR002016); BEST Arabidopsis thaliana protein match is: peroxidase CB (TAIR:AT3G49120.1)
AT5G22920	*-0,901*	RING ZINC-FINGER PROTEIN 34 (RZPF34)	Encodes a protein with sequence similarity to RING, zinc finger proteins. Loss of function mutations show reduced (15%) stomatal aperture under non stress conditions.
AT3G61750	*-0,902*		Cytochrome b561/ferric reductase transmembrane with DOMON related domain; FUNCTIONS IN: dopamine beta-monooxygenase activity; INVOLVED IN: histidine catabolic process; LOCATED IN: membrane; CONTAINS InterPro DOMAIN/s: Cytochrome b561, eukaryote (InterPro:IPR004877), DOMON related (InterPro:IPR005018), Cytochrome b561/ferric reductase transmembrane (InterPro:IPR006593), DOMON (InterPro:IPR013050); BEST Arabidopsis thaliana protein match is: Cytochrome b561/ferric reductase transmembrane with DOMON related domain (TAIR:AT3G07570.1)
AT5G61260	*-0,903*		Plant calmodulin-binding protein-related; CONTAINS InterPro DOMAIN/s: Calmodulin-binding protein, plant (InterPro:IPR012417); BEST Arabidopsis thaliana protein match is: Plant calmodulin-binding protein-related (TAIR:AT5G07820.1)
AT1G67710	*-0,908*	RESPONSE REGULATOR 11 (ARR11)	Encodes an Arabidopsis response regulator (ARR) protein that acts in concert with other type-B ARRs in the cytokinin signaling pathway.
AT2G18450	*-0,908*	SUCCINATE DEHYDROGENASE 1-2 (SDH1-2)	Nuclear encoded mitochondrial flavoprotein subunit of succinate dehydrogenase complex .
AT4G01450	*-0,91*	USUALLY MULTIPLE ACIDS MOVE IN AND OUT TRANSPORTERS 30 (UMAMIT30)	nodulin MtN21-like transporter family protein
AT3G26210	*-0,912*	CYTOCHROME P450, FAMILY 71, SUBFAMILY B, POLYPEPTIDE 23 (CYP71B23)	putative cytochrome P450 The mRNA is cell-to-cell mobile.
AT1G34670	*-0,914*	MYB DOMAIN PROTEIN 93 (MYB93)	Encodes a member of the R2R3 transcription factor gene family that is a negative regulator of lateral root (LR) development. It has been proposed that this transcription factor is part of a novel negative feedback loop stimulated specifically in the endodermis upon LR initiation to ensure that LRs are formed only in the correct place.
AT1G06640	*-0,915*		encodes a protein whose sequence is similar to a 2-oxoglutarate-dependent dioxygenase The mRNA is cell-to-cell mobile.
AT1G32100	*-0,915*	PINORESINOL REDUCTASE 1 (PRR1)	Encodes a pinoresinol reductase involved in lignan biosynthesis. Expressed strongly in roots and less strongly in stems. Shows specificity for pinoresinol and not lariciresinol.
AT1G70690	*-0,915*	HOPW1-1-INDUCED GENE1 (HWI1)	Encodes a plasmodesmal protein that may be involved in the intercellular movement of molecules through the plasmodesmata. The protein has two DUF26 domains and a single transmembrane domain.

AT2G15790	*-0,92*	SQUINT (SQN)	SQN encodes the Arabidopsis homolog of cyclophilin 40 (CyP40). It is specifically required for the vegetative but not the reproductive maturation of the shoot. Belongs to one of the 36 carboxylate clamp (CC)-tetratricopeptide repeat (TPR) proteins (Prasad 2010, Pubmed ID: 20856808) with potential to interact with Hsp90/Hsp70 as co-chaperones.
AT1G71010	*-0,921*	FORMS APLOID AND BINUCLEATE CELLS 1C (FAB1C)	Encodes a protein that is predicted to act as a phosphatidylinositol-3P 5-kinase, but, because it lacks a FYVE domain, it is unlikely to be efficiently targeted to membranes containing the proposed phosphatidylinositol-3P substrate. Therefore, its molecular function remains unknown. The mRNA is cell-to-cell mobile.
AT4G04810	*-0,925*	METHIONINE SULFOXIDE REDUCTASE B4 (MSRB4)	methionine sulfoxide reductase B4 (MSRB4); FUNCTIONS IN: peptide-methionine-(S)-S-oxide reductase activity; INVOLVED IN: oxidation reduction; LOCATED IN: cytosol; EXPRESSED IN: root; CONTAINS InterPro DOMAIN/s: Methionine sulphoxide reductase B (InterPro:IPR002579), Mss4-like (InterPro:IPR011057); BEST Arabidopsis thaliana protein match is: methionine sulfoxide reductase B5 (TAIR:AT4G04830.1)
AT1G62800	*-0,926*	ASPARTATE AMINOTRANSFERASE 4 (ASP4)	Encodes aspartate aminotransferase (Asp4).
AT4G27260	*-0,926*	(WES1)	encodes an IAA-amido synthase that conjugates Asp and other amino acids to auxin in vitro. Lines carrying insertions in this gene are hypersensitive to auxin. It is involved in camalexin biosynthesis via conjugating indole-3-carboxylic acid (ICA) and cysteine (Cys). The mRNA is cell-to-cell mobile.
AT2G19110	*-0,927*	HEAVY METAL ATPASE 4 (HMA4)	Encodes a protein with similarity to Zn ATPase. Can rescue Zn deficiency in yeast and Cd resistance, suggesting a role in Zn and Cd transport. The mRNA is cell-to-cell mobile.
AT1G60140	*-0,929*	TREHALOSE PHOSPHATE SYNTHASE (TPS10)	Encodes an enzyme putatively involved in trehalose biosynthesis. The protein has a trehalose synthase (TPS)-like domain that may or may not be active as well as a trehalose phosphatase (TPP)-like domain.
AT2G26670	*-0,929*	REVERSAL OF THE DET PHENOTYPE 4 (TED4)	Encodes a plastid heme oxygenase necessary for phytochrome chromophore biosynthesis and for coupling the expression of some nuclear genes to the functional state of the chloroplast.
AT4G34500	*-0,93*		Protein kinase superfamily protein; FUNCTIONS IN: protein serine/threonine kinase activity, protein kinase activity, kinase activity, ATP binding; INVOLVED IN: protein amino acid phosphorylation; LOCATED IN: cellular_component unknown; EXPRESSED IN: 22 plant structures; EXPRESSED DURING: 13 growth stages; CONTAINS InterPro DOMAIN/s: Protein kinase, ATP binding site (InterPro:IPR017441), Protein kinase, catalytic domain (InterPro:IPR000719), Serine-threonine/tyrosine-protein kinase (InterPro:IPR001245), Protein kinase-like domain (InterPro:IPR011009), Serine/threonine-protein kinase, active site (InterPro:IPR008271); BEST Arabidopsis thaliana protein match is: Protein kinase superfamily protein (TAIR:AT1G01540.2)
AT1G72160	*-0,931*		Sec14p-like phosphatidylinositol transfer family protein; FUNCTIONS IN: transporter activity; INVOLVED IN: transport; LOCATED IN: plasma membrane; EXPRESSED IN: 24 plant structures; EXPRESSED DURING: 13 growth stages; CONTAINS InterPro DOMAIN/s: Cellular retinaldehyde-binding/triple function, C-terminal (InterPro:IPR001251), Cellular retinaldehyde-binding/triple function, N-terminal (InterPro:IPR008273), GOLD (InterPro:IPR009038), Phosphatidylinositol transfer protein-like, N-terminal (InterPro:IPR011074); BEST Arabidopsis thaliana protein match is: SEC14 cytosolic factor family protein / phosphoglyceride transfer family protein (TAIR:AT4G09160.1)
AT1G50560	*-0,932*	CYTOCHROME P450, FAMILY 705, SUBFAMILY A, POLYPEPTIDE 25 (CYP705A25)	member of CYP705A
AT1G72230	*-0,932*		Cupredoxin superfamily protein; FUNCTIONS IN: electron carrier activity, copper ion binding; LOCATED IN: anchored to membrane; EXPRESSED IN: 17 plant structures; EXPRESSED DURING: 9 growth stages; CONTAINS InterPro DOMAIN/s: Plastocyanin-like (InterPro:IPR003245), Cupredoxin (InterPro:IPR008972), Blue (type 1) copper domain (InterPro:IPR000923); BEST Arabidopsis thaliana protein match is: Cupredoxin superfamily protein (TAIR:AT1G22480.1)
AT5G04980	*-0,932*		DNAse I-like superfamily protein; FUNCTIONS IN: hydrolase activity, inositol or phosphatidylinositol phosphatase activity; INVOLVED IN: biological_process unknown; LOCATED IN: cellular_component unknown; EXPRESSED IN: 18 plant structures; EXPRESSED DURING: 8 growth stages; CONTAINS InterPro DOMAIN/s: Inositol polyphosphate related phosphatase (InterPro:IPR000300), Endonuclease/exonuclease/phosphatase (InterPro:IPR005135); BEST Arabidopsis thaliana protein match is: DNAse I-like superfamily protein (TAIR:AT5G65090.1)

AT5G48290	*-0,932*		Heavy metal transport/detoxification superfamily protein ; FUNCTIONS IN: metal ion binding; INVOLVED IN: metal ion transport; LOCATED IN: cellular_component unknown; EXPRESSED IN: 6 plant structures; EXPRESSED DURING: 4 anthesis, petal differentiation and expansion stage; CONTAINS InterPro DOMAIN/s: Heavy metal transport/detoxification protein (InterPro:IPR006121); BEST Arabidopsis thaliana protein match is: Heavy metal transport/detoxification superfamily protein (TAIR:AT3G07600.1)
AT4G19160	*-0,933*		unknown protein
AT4G11300	*-0,934*		CONTAINS InterPro DOMAIN/s: Protein BYPASS related (InterPro:IPR008511); BEST Arabidopsis thaliana protein match is: Protein of unknown function (DUF793) (TAIR:AT4G23530.1)
AT1G51850	*-0,937*		Leucine-rich repeat protein kinase family protein; FUNCTIONS IN: kinase activity; INVOLVED IN: protein amino acid phosphorylation; LOCATED IN: plasma membrane; EXPRESSED IN: root; CONTAINS InterPro DOMAIN/s: Protein kinase, ATP binding site (InterPro:IPR017441), Serine/threonine-protein kinase domain (InterPro:IPR002290), Leucine-rich repeat (InterPro:IPR001611), Serine/threonine-protein kinase-like domain (InterPro:IPR017442), Protein kinase-like domain (InterPro:IPR011009), Serine/threonine-protein kinase, active site (InterPro:IPR008271), Protein kinase, catalytic domain (InterPro:IPR000719), Tyrosine-protein kinase, catalytic domain (InterPro:IPR020635); BEST Arabidopsis thaliana protein match is: Leucine-rich repeat protein kinase family protein (TAIR:AT1G51820.1)
AT4G20110	*-0,937*	VACUOLAR SORTING RECEPTOR 7 (VSR7)	VACUOLAR SORTING RECEPTOR 7 (VSR7); FUNCTIONS IN: calcium ion binding; INVOLVED IN: intracellular protein transport, protein targeting to vacuole; LOCATED IN: trans-Golgi network, integral to plasma membrane, Golgi transport complex; EXPRESSED IN: 17 plant structures; EXPRESSED DURING: 8 growth stages; CONTAINS InterPro DOMAIN/s: Protease-associated PA (InterPro:IPR003137), EGF-like calcium-binding, conserved site (InterPro:IPR018097), EGF-like calcium-binding (InterPro:IPR001881), Growth factor, receptor (InterPro:IPR009030); BEST Arabidopsis thaliana protein match is: VACUOLAR SORTING RECEPTOR 6 (TAIR:AT1G30900.1).
AT4G20820	*-0,937*	(ATBBE18)	FAD-binding Berberine family protein; FUNCTIONS IN: electron carrier activity, oxidoreductase activity, FAD binding, catalytic activity; INVOLVED IN: oxidation reduction; EXPRESSED IN: 6 plant structures; EXPRESSED DURING: LP.06 six leaves visible, LP.04 four leaves visible, LP.08 eight leaves visible; CONTAINS InterPro DOMAIN/s: FAD-binding, type 2 (InterPro:IPR016166), Oxygen oxidoreductase covalent FAD-binding site (InterPro:IPR006093), Berberine/berberine-like (InterPro:IPR012951), FAD linked oxidase, N-terminal (InterPro:IPR006094); BEST Arabidopsis thaliana protein match is: FAD-binding Berberine family protein (TAIR:AT5G44440.1)
AT4G01850	*-0,938*	S-ADENOSYLMETHIONINE SYNTHETASE 2 (SAM-2)	S-adenosylmethionine synthetase 2 (SAM-2); FUNCTIONS IN: copper ion binding, methionine adenosyltransferase activity; INVOLVED IN: one-carbon metabolic process, S-adenosylmethionine biosynthetic process; LOCATED IN: nucleolus, cell wall; EXPRESSED IN: 27 plant structures; EXPRESSED DURING: 17 growth stages; CONTAINS InterPro DOMAIN/s: S-adenosylmethionine synthetase (InterPro:IPR002133), S-adenosylmethionine synthetase superfamily (InterPro:IPR022636), S-adenosylmethionine synthetase, N-terminal (InterPro:IPR022628), S-adenosylmethionine synthetase, C-terminal (InterPro:IPR022630), S-adenosylmethionine synthetase, conserved site (InterPro:IPR022631), S-adenosylmethionine synthetase, central domain (InterPro:IPR022629); BEST Arabidopsis thaliana protein match is: S-adenosylmethionine synthetase 1 (TAIR:AT1G02500.2)
AT1G13830	*-0,942*		Carbohydrate-binding X8 domain superfamily protein; FUNCTIONS IN: molecular_function unknown; INVOLVED IN: biological_process unknown; LOCATED IN: endomembrane system; EXPRESSED IN: 8 plant structures; EXPRESSED DURING: 4 anthesis, petal differentiation and expansion stage; CONTAINS InterPro DOMAIN/s: X8 (InterPro:IPR012946); BEST Arabidopsis thaliana protein match is: Carbohydrate-binding X8 domain superfamily protein (TAIR:AT2G03505.1)
AT3G06370	*-0,943*	SODIUM HYDROGEN EXCHANGER 4 (NHX4)	member of Sodium proton exchanger family
AT5G03380	*-0,943*		Heavy metal transport/detoxification superfamily protein ; FUNCTIONS IN: metal ion binding; INVOLVED IN: metal ion transport; EXPRESSED IN: 24 plant structures; EXPRESSED DURING: 13 growth stages; CONTAINS InterPro DOMAIN/s: Heavy metal transport/detoxification protein (InterPro:IPR006121); BEST Arabidopsis thaliana protein match is: Heavy metal transport/detoxification superfamily protein (TAIR:AT2G36950.1)

AT1G45130	*-0,944*	BETA-GALACTOSIDASE 5 (BGAL5)	beta-galactosidase 5 (BGAL5); FUNCTIONS IN: cation binding, beta-galactosidase activity, hydrolase activity, hydrolyzing O-glycosyl compounds, catalytic activity; INVOLVED IN: lactose catabolic process, using glucoside 3-dehydrogenase, carbohydrate metabolic process, lactose catabolic process via UDP-galactose, lactose catabolic process; LOCATED IN: endomembrane system; EXPRESSED IN: 24 plant structures; EXPRESSED DURING: 13 growth stages; CONTAINS InterPro DOMAIN/s: Glycoside hydrolase, family 35, conserved site (InterPro:IPR019801), Glycoside hydrolase family 2, carbohydrate-binding (InterPro:IPR006104), Glycoside hydrolase, family 35 (InterPro:IPR001944), Glycoside hydrolase, catalytic core (InterPro:IPR017853), Glycoside hydrolase, subgroup, catalytic core (InterPro:IPR013781), Galactose-binding domain-like (InterPro:IPR008979); BEST Arabidopsis thaliana protein match is: beta-galactosidase 3 (TAIR:AT4G36360.1)
AT1G30900	*-0,945*	VACUOLAR SORTING RECEPTOR 6 (VSR6)	VACUOLAR SORTING RECEPTOR 6 (VSR6); FUNCTIONS IN: calcium ion binding; INVOLVED IN: protein targeting to vacuole; LOCATED IN: integral to plasma membrane, Golgi transport complex; EXPRESSED IN: 20 plant structures; EXPRESSED DURING: 10 growth stages; CONTAINS InterPro DOMAIN/s: Protease-associated PA (InterPro:IPR003137), EGF-like calcium-binding, conserved site (InterPro:IPR018097), EGF-like calcium-binding (InterPro:IPR001881), Growth factor, receptor (InterPro:IPR009030); BEST Arabidopsis thaliana protein match is: VACUOLAR SORTING RECEPTOR 5 (TAIR:AT2G34940.1)
AT1G51800	*-0,947*	IMPAIRED OOMYCETE SUSCEPTIBILITY 1 (IOS1)	The gene encodes a putative member of the LRR-RLK protein family. Expressin and mutant analysis revealed that it contributes to the interaction between Arabidopsis and Hyaloperonospora arabidopsidis. and The mRNA is cell-to-cell mobile.
AT1G72180	*-0,947*	C-TERMINALLY ENCODED PEPTIDE RECEPTOR 2 (CEPR2)	Encodes a leucine-rich repeat receptor kinase that functions as a receptor for CEP1 peptide. Mediates nitrate uptake signaling.
AT4G00360	*-0,947*	CYTOCHROME P450, FAMILY 86, SUBFAMILY A, POLYPEPTIDE 2 (CYP86A2)	Encodes a member of the CYP86A subfamily of cytochrome p450 genes. Expressed at moderate levels in flowers, leaves, roots and stems.
AT4G11190	*-0,95*		Disease resistance-responsive (dirigent-like protein) family protein; FUNCTIONS IN: molecular_function unknown; INVOLVED IN: lignan biosynthetic process, defense response; LOCATED IN: endomembrane system; EXPRESSED IN: 10 plant structures; EXPRESSED DURING: F mature embryo stage, petal differentiation and expansion stage, E expanded cotyledon stage, D bilateral stage; CONTAINS InterPro DOMAIN/s: Plant disease resistance response protein (InterPro:IPR004265); BEST Arabidopsis thaliana protein match is: Disease resistance-responsive (dirigent-like protein) family protein (TAIR:AT4G11210.1)
AT1G68810	*-0,953*	ABNORMAL SHOOT 5 (ABS5)	basic helix-loop-helix (bHLH) DNA-binding superfamily protein; FUNCTIONS IN: DNA binding, sequence-specific DNA binding transcription factor activity; INVOLVED IN: regulation of transcription; LOCATED IN: nucleus; EXPRESSED IN: 20 plant structures; EXPRESSED DURING: 12 growth stages; CONTAINS InterPro DOMAIN/s: Helix-loop-helix DNA-binding domain (InterPro:IPR001092), Helix-loop-helix DNA-binding (InterPro:IPR011598); BEST Arabidopsis thaliana protein match is: basic helix-loop-helix 32 (TAIR:AT3G25710.1)
AT5G10280	*-0,955*	MYB DOMAIN PROTEIN 92 (MYB92)	Encodes a putative transcription factor (MYB92).
AT5G66490	*-0,955*		unknown protein; FUNCTIONS IN: molecular_function unknown; INVOLVED IN: biological_process unknown; LOCATED IN: plasma membrane; EXPRESSED IN: 17 plant structures; EXPRESSED DURING: 9 growth stages; BEST Arabidopsis thaliana protein match is: unknown protein (TAIR:AT3G50900.1)
AT4G34560	*-0,963*		unknown protein; BEST Arabidopsis thaliana protein match is: unknown protein (TAIR:AT5G66440.1)
AT5G01360	*-0,967*	TRICHOME BIREFRINGENCE-LIKE 3 (TBL3)	Encodes a member of the TBL (TRICHOME BIREFRINGENCE-LIKE) gene family containing a plant-specific DUF231 (domain of unknown function) domain. TBL gene family has 46 members, two of which (TBR/AT5G06700 and TBL3/AT5G01360) have been shown to be involved in the synthesis and deposition of secondary wall cellulose, presumably by influencing the esterification state of pectic polymers. A nomenclature for this gene family has been proposed (Volker Bischoff & Wolf Scheible, 2010, personal communication).The dwarf phenotype can only be seen in tbl3 tbl31 esk1 triple mutant. tbl3 and tbl31 are specifically involved in 3-O-monoacetylation of xylan.

AT5G01520	*-0,969*	ABA INSENSITIVE RING PROTEIN 2 (AIRP2)	RING/U-box superfamily protein; FUNCTIONS IN: zinc ion binding; EXPRESSED IN: 17 plant structures; EXPRESSED DURING: 8 growth stages; CONTAINS InterPro DOMAIN/s: Zinc finger, RING-type, conserved site (InterPro:IPR017907), Zinc finger, RING-type (InterPro:IPR001841), Zinc finger, C3HC4 RING-type (InterPro:IPR018957); BEST Arabidopsis thaliana protein match is: RING/U-box superfamily protein (TAIR:AT3G47160.1)
AT1G70782	*-0,971*	CONSERVED PEPTIDE UPSTREAM OPEN READING FRAME 28 (CPuORF28)	Upstream open reading frames (uORFs) are small open reading frames found in the 5' UTR of a mature mRNA, and can potentially mediate translational regulation of the largest, or major, ORF (mORF). CPuORF28 represents a conserved upstream opening reading frame relative to major ORF AT1G70780.1
AT4G15760	*-0,974*	MONOOXYGENASE 1 (MO1)	Encodes a protein with similarity to monooxygenases that are known to degrade salicylic acid (SA).
AT4G15960	*-0,974*		alpha/beta-Hydrolases superfamily protein; FUNCTIONS IN: epoxide hydrolase activity, catalytic activity; INVOLVED IN: biological_process unknown; LOCATED IN: mitochondrion; CONTAINS InterPro DOMAIN/s: Epoxide hydrolase-like (InterPro:IPR000639), Alpha/beta hydrolase fold-1 (InterPro:IPR000073); BEST Arabidopsis thaliana protein match is: alpha/beta-Hydrolases superfamily protein (TAIR:AT4G15955.3)
AT2G27830	*-0,977*		unknown protein; BEST Arabidopsis thaliana protein match is: unknown protein (TAIR:AT4G22758.1)
AT4G24130	*-0,977*		Protein of unknown function, DUF538; INVOLVED IN: biological_process unknown; LOCATED IN: nucleus; EXPRESSED IN: 21 plant structures; EXPRESSED DURING: 12 growth stages; CONTAINS InterPro DOMAIN/s: Protein of unknown function DUF538 (InterPro:IPR007493); BEST Arabidopsis thaliana protein match is: Protein of unknown function, DUF538 (TAIR:AT5G46230.1)
AT4G25760	*-0,983*	GLUTAMINE DUMPER 2 (GDU2)	Encodes a member of the GDU (glutamine dumper) family proteins involved in amino acid export: At4g31730 (GDU1), At4g25760 (GDU2), At5g57685 (GDU3), At2g24762 (GDU4), At5g24920 (GDU5), At3g30725 (GDU6) and At5g38770 (GDU7).
AT5G59700	*-0,984*		Protein kinase superfamily protein; FUNCTIONS IN: kinase activity; INVOLVED IN: protein amino acid phosphorylation; LOCATED IN: plasma membrane; EXPRESSED IN: 8 plant structures; EXPRESSED DURING: 4 anthesis, petal differentiation and expansion stage; CONTAINS InterPro DOMAIN/s: Protein kinase, ATP binding site (InterPro:IPR017441), Malectin/receptor-like protein kinase (InterPro:IPR021720), Protein kinase, catalytic domain (InterPro:IPR000719), Serine-threonine/tyrosine-protein kinase (InterPro:IPR001245), Protein kinase-like domain (InterPro:IPR011009), Serine/threonine-protein kinase, active site (InterPro:IPR008271); BEST Arabidopsis thaliana protein match is: hercules receptor kinase 1 (TAIR:AT3G46290.1)
AT2G25000	*-0,986*	WRKY DNA-BINDING PROTEIN 60 (WRKY60)	Pathogen-induced transcription factor. Forms protein complexes with itself and with WRKY40. Coexpression with WRKY18 or WRKY40 made plants more susceptible to both P. syringae and B. cinerea. WRKY18, WRKY40, and WRKY60 have partially redundant roles in response to the hemibiotrophic bacterial pathogen Pseudomonas syringae and the necrotrophic fungal pathogen Botrytis cinerea, with WRKY18 playing a more important role than the other two.
AT5G10970	*-0,986*		C2H2 and C2HC zinc fingers superfamily protein; FUNCTIONS IN: sequence-specific DNA binding transcription factor activity, zinc ion binding, nucleic acid binding; INVOLVED IN: regulation of transcription; LOCATED IN: intracellular; EXPRESSED IN: hypocotyl, root, leaf; EXPRESSED DURING: LP.04 four leaves visible; CONTAINS InterPro DOMAIN/s: Zinc finger, C2H2-like (InterPro:IPR015880), Zinc finger, C2H2-type (InterPro:IPR007087); BEST Arabidopsis thaliana protein match is: zinc finger protein 3 (TAIR:AT5G25160.1)
AT1G13930	*-0,987*		Involved in response to salt stress. Knockout mutants are hypersensitive to salt stress. The mRNA is cell-to-cell mobile.
AT4G38470	*-0,987*	SERINE/THREONINE/TYROSINE KINASE 46 (STY46)	ACT-like protein tyrosine kinase family protein; FUNCTIONS IN: protein serine/threonine/tyrosine kinase activity, protein kinase activity; INVOLVED IN: protein amino acid phosphorylation, metabolic process; LOCATED IN: cytosol; EXPRESSED IN: 25 plant structures; EXPRESSED DURING: 15 growth stages; CONTAINS InterPro DOMAIN/s: Serine/threonine-protein kinase domain (InterPro:IPR002290), Amino acid-binding ACT (InterPro:IPR002912), Serine-threonine/tyrosine-protein kinase (InterPro:IPR001245), Serine/threonine protein kinase-like, ATMRK (InterPro:IPR015783), Protein kinase-like domain (InterPro:IPR011009), Serine/threonine-protein kinase, active site (InterPro:IPR008271), Protein kinase, catalytic domain (InterPro:IPR000719), Tyrosine-protein kinase, catalytic domain (InterPro:IPR020635); BEST Arabidopsis thaliana protein match is: ACT-like protein tyrosine kinase family protein (TAIR:AT4G35780.1)

AT1G79410	*-0,99*	ORGANIC CATION/CARNITINE TRANSPORTER5 (OCT5)	organic cation/carnitine transporter5 (5-Oct); FUNCTIONS IN: carbohydrate transmembrane transporter activity, sugar:hydrogen symporter activity; INVOLVED IN: transport, transmembrane transport; LOCATED IN: integral to membrane, membrane; EXPRESSED IN: 19 plant structures; EXPRESSED DURING: 13 growth stages; CONTAINS InterPro DOMAIN/s: Major facilitator superfamily (InterPro:IPR020846), General substrate transporter (InterPro:IPR005828), Major facilitator superfamily, general substrate transporter (InterPro:IPR016196); BEST Arabidopsis thaliana protein match is: organic cation/carnitine transporter 6 (TAIR:AT1G16370.1)
AT1G70420	*-0,991*		Protein of unknown function (DUF1645); CONTAINS InterPro DOMAIN/s: Protein of unknown function DUF1645 (InterPro:IPR012442); BEST Arabidopsis thaliana protein match is: Protein of unknown function (DUF1645) (TAIR:AT1G23710.1)
AT3G60900	*-0,992*	FASCICLIN-LIKE ARABINOGALACTAN-PROTEIN 10 (FLA10)	FASCICLIN-like arabinogalactan-protein 10 (FLA10); LOCATED IN: anchored to plasma membrane, plasma membrane, anchored to membrane; EXPRESSED IN: 17 plant structures; EXPRESSED DURING: 8 growth stages; CONTAINS InterPro DOMAIN/s: FAS1 domain (InterPro:IPR000782); BEST Arabidopsis thaliana protein match is: FASCICLIN-like arabinogalactan protein 8 (TAIR:AT2G45470.1)
AT1G69160	*-0,996*		unknown protein; BEST Arabidopsis thaliana protein match is: unknown protein (TAIR:AT3G13980.1)
AT2G38540	*-0,996*	LIPID TRANSFER PROTEIN 1 (LP1)	Non-specific lipid transfer protein. Binds calmodulin in a Ca2+-independent manner. Localized to the cell wall. Specifically expressed in L1 epidermal layer. Predicted to be a member of PR-14 pathogenesis-related protein family with the following members: At2g38540/LTP1, At2g38530/LTP2, At5g59320/LTP3, At5g59310/LTP4, At3g51600/LTP5, At3g08770/LTP6, At2g15050/LTP7, At2g18370/LTP8, At2g15325/LTP9, At5g01870/LTP10, At4g33355/LTP11, At3g51590/LTP12, At5g44265/LTP13, At5g62065/LTP14, At4g08530/LTP15. The mRNA is cell-to-cell mobile.
AT5G40890	*-0,999*	CHLORIDE CHANNEL A (CLC-A)	Encodes a member of the voltage-dependent chloride channel. Also functions as a NO3-/H+ exchanger that serves to accumulate nitrate nutrient in vacuoles. Mutants homozygous for the T-DNA insertion mutation have reduced nitrate uptake capacity in high nitrate environment and exhibit hypersensitivity to chlorate.
AT1G08320	*-1,002*	TGACG (TGA) MOTIF-BINDING PROTEIN 9 (TGA9)	bZIP transcription factor family protein; FUNCTIONS IN: DNA binding, sequence-specific DNA binding transcription factor activity; INVOLVED IN: regulation of transcription, DNA-dependent; EXPRESSED IN: 9 plant structures; EXPRESSED DURING: 6 growth stages; CONTAINS InterPro DOMAIN/s: Basic-leucine zipper (bZIP) transcription factor (InterPro:IPR004827), bZIP transcription factor, bZIP-1 (InterPro:IPR011616); BEST Arabidopsis thaliana protein match is: bZIP transcription factor family protein (TAIR:AT5G06839.3)
AT2G25900	*-1,002*	(ATCTH)	Encodes a protein with two tandem-arrayed CCCH-type zinc fingers that binds RNA and is involved in RNA turnover. The mRNA is cell-to-cell mobile.
AT3G21260	*-1,002*	GLYCOLIPID TRANSFER PROTEIN 3 (GLTP3)	Glycolipid transfer protein (GLTP) family protein; FUNCTIONS IN: glycolipid transporter activity, glycolipid binding; INVOLVED IN: glycolipid transport; LOCATED IN: cytoplasm; EXPRESSED IN: 20 plant structures; EXPRESSED DURING: 13 growth stages; CONTAINS InterPro DOMAIN/s: Glycolipid transfer protein, GLTP (InterPro:IPR014830); BEST Arabidopsis thaliana protein match is: glycolipid transfer protein 2 (TAIR:AT1G21360.1)
AT5G52830	*-1,002*	WRKY DNA-BINDING PROTEIN 27 (WRKY27)	Encodes a WRKY transcription factor WRKY27. Mutation in Arabidopsis WRKY27 results in delayed symptom development in response to the bacterial wilt pathogen Ralstonia solanacearum.
AT1G51790	*-1,003*		Leucine-rich repeat protein kinase family protein; FUNCTIONS IN: kinase activity; INVOLVED IN: protein amino acid phosphorylation; LOCATED IN: endomembrane system; EXPRESSED IN: 9 plant structures; EXPRESSED DURING: 6 growth stages; CONTAINS InterPro DOMAIN/s: Protein kinase, catalytic domain (InterPro:IPR000719), Serine/threonine-protein kinase domain (InterPro:IPR002290), Leucine-rich repeat (InterPro:IPR001611), Serine-threonine/tyrosine-protein kinase (InterPro:IPR001245), Tyrosine-protein kinase, catalytic domain (InterPro:IPR020635), Protein kinase-like domain (InterPro:IPR011009); BEST Arabidopsis thaliana protein match is: Leucine-rich repeat protein kinase family protein (TAIR:AT1G51800.1)
AT2G15390	*-1,003*	FUCOSYLTRANSFERASE 4 (FUT4)	Encodes an alpha-(1,2)-fucosyltransferase.

AT5G44910	*-1,006*		Toll-Interleukin-Resistance (TIR) domain family protein; FUNCTIONS IN: transmembrane receptor activity; INVOLVED IN: signal transduction, defense response, innate immune response; LOCATED IN: intrinsic to membrane; EXPRESSED IN: hypocotyl, root; CONTAINS InterPro DOMAIN/s: Toll-Interleukin receptor (InterPro:IPR000157); BEST Arabidopsis thaliana protein match is: Disease resistance protein (TIR-NBS-LRR class) family (TAIR:AT5G45000.1)
AT5G67420	*-1,007*	LOB DOMAIN-CONTAINING PROTEIN 37 (LBD37)	Encodes a LOB-domain protein involved in nitrogen metabolism and affecting leaf morphogenesis.
AT1G33080	*-1,009*		MATE efflux family protein; FUNCTIONS IN: antiporter activity, drug transmembrane transporter activity, transporter activity; INVOLVED IN: drug transmembrane transport, transmembrane transport; LOCATED IN: membrane; EXPRESSED IN: 22 plant structures; EXPRESSED DURING: 13 growth stages; CONTAINS InterPro DOMAIN/s: Multi antimicrobial extrusion protein MatE (InterPro:IPR002528); BEST Arabidopsis thaliana protein match is: MATE efflux family protein (TAIR:AT1G33110.1)
AT5G67030	*-1,009*	ABA DEFICIENT 1 (ABA1)	Encodes a single copy zeaxanthin epoxidase gene that functions in first step of the biosynthesis of the abiotic stress hormone abscisic acid (ABA). Mutants in this gene are unable to express female sterility in response to beta-aminobutyric acid, as wild type plants do.
AT1G23870	*-1,01*	TREHALOSE-PHOSPHATASE/SYNTHASE 9 (TPS9)	Encodes an enzyme putatively involved in trehalose biosynthesis. The protein has a trehalose synthase (TPS)-like domain that may or may not be active as well as a trehalose phosphatase (TPP)-like domain. The mRNA is cell-to-cell mobile.
AT1G72220	*-1,01*		RING/U-box superfamily protein; FUNCTIONS IN: zinc ion binding; EXPRESSED IN: inflorescence meristem, root, flower; EXPRESSED DURING: 4 anthesis, petal differentiation and expansion stage; CONTAINS InterPro DOMAIN/s: Zinc finger, RING-type (InterPro:IPR001841), Zinc finger, C3HC4 RING-type (InterPro:IPR018957); BEST Arabidopsis thaliana protein match is: RING/U-box superfamily protein (TAIR:AT4G33565.1)
AT4G12050	*-1,01*	AT-HOOK MOTIF NUCLEAR LOCALIZED PROTEIN 26 (AHL26)	Predicted AT-hook DNA-binding family protein; CONTAINS InterPro DOMAIN/s: Protein of unknown function DUF296 (InterPro:IPR005175), Predicted AT-hook DNA-binding (InterPro:IPR014476); BEST Arabidopsis thaliana protein match is: Predicted AT-hook DNA-binding family protein (TAIR:AT4G22810.1)
AT2G43850	*-1,013*	INTEGRIN-LINKED KINASE1 (ILK1)	Integrin-linked protein kinase family; FUNCTIONS IN: protein serine/threonine/tyrosine kinase activity, kinase activity; INVOLVED IN: regulation of signal transduction, protein amino acid phosphorylation; LOCATED IN: chloroplast; EXPRESSED IN: 24 plant structures; EXPRESSED DURING: 13 growth stages; CONTAINS InterPro DOMAIN/s: Integrin-linked protein kinase (InterPro:IPR016253), Serine/threonine-protein kinase domain (InterPro:IPR002290), Serine-threonine/tyrosine-protein kinase (InterPro:IPR001245), Protein kinase-like domain (InterPro:IPR011009), Serine/threonine-protein kinase, active site (InterPro:IPR008271), Protein kinase, catalytic domain (InterPro:IPR000719), Ankyrin repeat-containing domain (InterPro:IPR020683), Tyrosine-protein kinase, catalytic domain (InterPro:IPR020635), Ankyrin repeat (InterPro:IPR002110); BEST Arabidopsis thaliana protein match is: Integrin-linked protein kinase family (TAIR:AT3G59830.1)
AT3G25572	*-1,015*	CONSERVED PEPTIDE UPSTREAM OPEN READING FRAME 11 (CPuORF11)	Upstream open reading frames (uORFs) are small open reading frames found in the 5' UTR of a mature mRNA, and can potentially mediate translational regulation of the largest, or major, ORF (mORF). CPuORF11 represents a conserved upstream opening reading frame relative to major ORF AT3G25570.1
AT4G11230	*-1,017*		Riboflavin synthase-like superfamily protein; FUNCTIONS IN: in 6 functions; INVOLVED IN: oxidation reduction; LOCATED IN: integral to membrane, membrane; EXPRESSED IN: root, flower; EXPRESSED DURING: petal differentiation and expansion stage; CONTAINS InterPro DOMAIN/s: Ferredoxin reductase-type FAD-binding domain (InterPro:IPR017927), NADPH oxidase Respiratory burst (InterPro:IPR013623), Ferric reductase, NAD binding (InterPro:IPR013121), Cytochrome b245, heavy chain (InterPro:IPR000778), FAD-binding 8 (InterPro:IPR013112), Riboflavin synthase-like beta-barrel (InterPro:IPR017938), Ferric reductase-like transmembrane component, N-terminal (InterPro:IPR013130); BEST Arabidopsis thaliana protein match is: respiratory burst oxidase protein F (TAIR:AT1G64060.1)

AT3G18080	*-1,018*	B-S GLUCOSIDASE 44 (BGLU44)	B-S glucosidase 44 (BGLU44); FUNCTIONS IN: in 6 functions; INVOLVED IN: carbohydrate metabolic process; LOCATED IN: cytosolic ribosome, cell wall, plant-type cell wall; EXPRESSED IN: 22 plant structures; EXPRESSED DURING: 13 growth stages; CONTAINS InterPro DOMAIN/s: Glycoside hydrolase, family 1 (InterPro:IPR001360), Glycoside hydrolase, catalytic core (InterPro:IPR017853), Glycoside hydrolase, subgroup, catalytic core (InterPro:IPR013781); BEST Arabidopsis thaliana protein match is: beta glucosidase 43 (TAIR:AT3G18070.1)
AT5G04470	*-1,019*	SIAMESE (SIM)	Encodes a novel nuclear 14-kD protein containing a cyclin binding motif and a motif found in ICK/KRP cell cycle inhibitor proteins. It is required for coordinating cell division and cell differentiation during the development of Arabidopsis trichomes, playing a key role in the mitosis-to-endoreduplication transition. It interacts with D-type cyclins in vivo.
AT2G46650	*-1,022*	CYTOCHROME B5 ISOFORM C (CB5-C)	member of Cytochromes b5 The mRNA is cell-to-cell mobile.
AT1G78340	*-1,026*	GLUTATHIONE S-TRANSFERASE TAU 22	Encodes glutathione transferase belonging to the tau class of GSTs. Naming convention according to Wagner et al. (2002).
AT5G61600	*-1,03*	ETHYLENE RESPONSE FACTOR 104 (ERF104)	encodes a member of the ERF (ethylene response factor) subfamily B-3 of ERF/AP2 transcription factor family. The protein contains one AP2 domain. There are 18 members in this subfamily including ATERF-1, ATERF-2, AND ATERF-5.
AT2G38640	*-1,031*		Protein of unknown function (DUF567); CONTAINS InterPro DOMAIN/s: Protein of unknown function DUF567 (InterPro:IPR007612); BEST Arabidopsis thaliana protein match is: Protein of unknown function (DUF567) (TAIR:AT5G41590.1)
AT5G57070	*-1,032*		hydroxyproline-rich glycoprotein family protein; BEST Arabidopsis thaliana protein match is: hydroxyproline-rich glycoprotein family protein (TAIR:AT1G72790.1)
AT3G63210	*-1,034*	(MARD1)	encodes a novel zinc-finger protein with a proline-rich N-terminus, identical to senescence-associated protein SAG102
AT4G28890	*-1,035*		RING/U-box superfamily protein; FUNCTIONS IN: ubiquitin-protein ligase activity, zinc ion binding; LOCATED IN: endomembrane system; EXPRESSED IN: root stele, root endodermis, hypocotyl, root, primary root differentiation zone; CONTAINS InterPro DOMAIN/s: Zinc finger, RING-type (InterPro:IPR001841), Zinc finger, C3HC4 RING-type (InterPro:IPR018957); BEST Arabidopsis thaliana protein match is: RING/U-box superfamily protein (TAIR:AT2G20030.1)
AT1G07590	*-1,036*		Tetratricopeptide repeat (TPR)-like superfamily protein; INVOLVED IN: response to cadmium ion; LOCATED IN: mitochondrion; EXPRESSED IN: 12 plant structures; EXPRESSED DURING: 4 anthesis, F mature embryo stage, petal differentiation and expansion stage, E expanded cotyledon stage, D bilateral stage; CONTAINS InterPro DOMAIN/s: Pentatricopeptide repeat (InterPro:IPR002885); BEST Arabidopsis thaliana protein match is: PENTATRICOPEPTIDE REPEAT 596 (TAIR:AT1G80270.3)
AT1G49580	*-1,036*		Calcium-dependent protein kinase (CDPK) family protein; FUNCTIONS IN: in 6 functions; INVOLVED IN: protein amino acid phosphorylation, N-terminal protein myristoylation; LOCATED IN: plasma membrane; EXPRESSED IN: 22 plant structures; EXPRESSED DURING: 13 growth stages; CONTAINS InterPro DOMAIN/s: Protein kinase, ATP binding site (InterPro:IPR017441), Serine/threonine-protein kinase domain (InterPro:IPR002290), EF-hand-like domain (InterPro:IPR011992), Serine/threonine-protein kinase-like domain (InterPro:IPR017442), Protein kinase-like domain (InterPro:IPR011009), Serine/threonine-protein kinase, active site (InterPro:IPR008271), Protein kinase, catalytic domain (InterPro:IPR000719), Calcium-dependent protein kinase (InterPro:IPR020642), Calcium/calmodulin-dependent protein kinase-like (InterPro:IPR020636); BEST Arabidopsis thaliana protein match is: Protein kinase superfamily protein (TAIR:AT3G19100.1)
AT1G67330	*-1,04*		Protein of unknown function (DUF579); FUNCTIONS IN: molecular_function unknown; INVOLVED IN: biological_process unknown; LOCATED IN: endomembrane system; EXPRESSED IN: 8 plant structures; EXPRESSED DURING: LP.06 six leaves visible, LP.04 four leaves visible, LP.10 ten leaves visible, 4 leaf senescence stage; CONTAINS InterPro DOMAIN/s: Protein of unknown function DUF579 (InterPro:IPR021148), Conserved hypothetical protein CHP01627 (InterPro:IPR006514); BEST Arabidopsis thaliana protein match is: Protein of unknown function (DUF579) (TAIR:AT1G27930.1)
AT5G44480	*-1,04*	DEFECTIVE UGE IN ROOT (DUR)	mutant has Altered lateral root; UDP Glucose Epimerase The mRNA is cell-to-cell mobile.
AT5G18860	*-1,042*	NUCLEOSIDE HYDROLASE 3 (NSH3)	Encodes a purine nucleoside hydrolase active in the apoplast. It might play a role in salvaging extracellular ATP. NSH3 transcript levels rise in response to jasmonic acid and wounding.

AT5G61570	*-1,042*		Protein kinase superfamily protein; FUNCTIONS IN: protein serine/threonine kinase activity, protein kinase activity, kinase activity, ATP binding; INVOLVED IN: protein amino acid phosphorylation; LOCATED IN: endomembrane system; EXPRESSED IN: 16 plant structures; EXPRESSED DURING: 12 growth stages; CONTAINS InterPro DOMAIN/s: Protein kinase, catalytic domain (InterPro:IPR000719), Serine/threonine-protein kinase domain (InterPro:IPR002290), Serine-threonine/tyrosine-protein kinase (InterPro:IPR001245), Tyrosine-protein kinase, catalytic domain (InterPro:IPR020635), Protein kinase-like domain (InterPro:IPR011009); BEST Arabidopsis thaliana protein match is: Protein kinase superfamily protein (TAIR:AT5G07620.1)
AT1G26820	*-1,044*	RIBONUCLEASE 3 (RNS3)	Encodes ribonuclease RNS3.
AT1G72300	*-1,046*	PSY1 RECEPTOR (PSY1R)	Encodes a leucine-rich repeat receptor kinase (LRR-RK) involved in the perception of PSY1. PSY1 is an 18-aa tyrosine-sulfated glycopeptide encoded by AT5G58650 that promotes cellular proliferation and expansion.
AT1G19600	*-1,047*		pfkB-like carbohydrate kinase family protein; FUNCTIONS IN: kinase activity; INVOLVED IN: D-ribose catabolic process; LOCATED IN: cellular_component unknown; EXPRESSED IN: 21 plant structures; EXPRESSED DURING: 13 growth stages; CONTAINS InterPro DOMAIN/s: Carbohydrate/purine kinase (InterPro:IPR011611); BEST Arabidopsis thaliana protein match is: pfkB-like carbohydrate kinase family protein (TAIR:AT4G27600.1)
AT4G22950	*-1,047*	AGAMOUS-LIKE 19 (AGL19)	MADS-box protein AGL19
AT1G68560	*-1,049*	ALPHA-XYLOSIDASE 1 (XYL1)	Encodes a bifunctional alpha-l-arabinofuranosidase/beta-d-xylosidase that belongs to family 3 of glycoside hydrolases.
AT1G44050	*-1,05*		Cysteine/Histidine-rich C1 domain family protein; FUNCTIONS IN: zinc ion binding; INVOLVED IN: intracellular signaling pathway; LOCATED IN: cellular_component unknown; EXPRESSED IN: root; CONTAINS InterPro DOMAIN/s: Protein kinase C-like, phorbol ester/diacylglycerol binding (InterPro:IPR002219), DC1 (InterPro:IPR004146), Zinc finger, PHD-type (InterPro:IPR001965), C1-like (InterPro:IPR011424); BEST Arabidopsis thaliana protein match is: Cysteine/Histidine-rich C1 domain family protein (TAIR:AT5G55780.1)
AT4G36410	*-1,052*	UBIQUITIN-CONJUGATING ENZYME 17 (UBC17)	ubiquitin-conjugating enzyme
AT5G19230	*-1,052*		Glycoprotein membrane precursor GPI-anchored; FUNCTIONS IN: molecular_function unknown; INVOLVED IN: biological_process unknown; LOCATED IN: anchored to plasma membrane, plasma membrane, anchored to membrane; EXPRESSED IN: callus; BEST Arabidopsis thaliana protein match is: Glycoprotein membrane precursor GPI-anchored (TAIR:AT5G19240.1)
AT3G07310	*-1,056*		Protein of unknown function (DUF760); CONTAINS InterPro DOMAIN/s: Protein of unknown function DUF760 (InterPro:IPR008479); BEST Arabidopsis thaliana protein match is: Protein of unknown function (DUF760) (TAIR:AT5G48590.1)
AT1G11540	*-1,057*		Sulfite exporter TauE/SafE family protein; FUNCTIONS IN: molecular_function unknown; INVOLVED IN: biological_process unknown; LOCATED IN: endomembrane system, integral to membrane; EXPRESSED IN: 13 plant structures; EXPRESSED DURING: M germinated pollen stage, 4 anthesis, C globular stage, 4 leaf senescence stage, petal differentiation and expansion stage; CONTAINS InterPro DOMAIN/s: Protein of unknown function DUF81 (InterPro:IPR002781); BEST Arabidopsis thaliana protein match is: Sulfite exporter TauE/SafE family protein (TAIR:AT1G61740.1)
AT2G43910	*-1,061*	HARMLESS TO OZONE LAYER 1 (HOL1)	HARMLESS TO OZONE LAYER 1 (HOL1); FUNCTIONS IN: methyltransferase activity, thiopurine S-methyltransferase activity; INVOLVED IN: metabolic process; LOCATED IN: plasma membrane; EXPRESSED IN: guard cell, cultured cell, leaf; CONTAINS InterPro DOMAIN/s: Thiopurine S-methyltransferase (InterPro:IPR008854); BEST Arabidopsis thaliana protein match is: S-adenosyl-L-methionine-dependent methyltransferases superfamily protein (TAIR:AT2G43920.1)
AT2G27360	*-1,062*		GDSL-motif esterase/acyltransferase/lipase. Enzyme group with broad substrate specificity that may catalyze acyltransfer or hydrolase reactions with lipid and non-lipid substrates.
AT5G47560	*-1,062*	TONOPLAST DICARBOXYLATE TRANSPORTER (TDT)	Encodes a tonoplast malate/fumarate transporter.
AT5G01490	*-1,064*	CATION EXCHANGER 4 (CAX4)	Encodes a cation/proton antiporter, a member of low affinity calcium antiporter CAX2 family. Involved in root development under metal stress.

AT4G36380	*-1,068*	ROTUNDIFOLIA 3 (ROT3)	Encodes a cytochrome P-450 gene that is involved in leaf blade expansion by controlling polar cell expansion in the leaf length direction. Member of the CYP90C CYP450 family. ROT3 was shown to be involved in brassinosteroid biosynthesis, most likely in the conversion step of typhasterol (TY) to castasterone (CS). As 6-deoxo-CS was unable to restore the phenotype of rot3-1, it has been postulated that ROT3 might be specifically involved in the conversion of TY to CS in the C6-oxidation pathway of brassinolide. Recently, CYP90C1 was shown to catalyse the C-23 hydroxylation of several brassinosteroids (the enzyme has a broad specificity for 22-hydroxylated substrates).
AT1G49500	*-1,072*		unknown protein; FUNCTIONS IN: molecular_function unknown; INVOLVED IN: biological_process unknown; LOCATED IN: endomembrane system; EXPRESSED IN: 19 plant structures; EXPRESSED DURING: 10 growth stages; BEST Arabidopsis thaliana protein match is: unknown protein (TAIR:AT3G19030.1)
AT4G33960	*-1,072*		unknown protein; FUNCTIONS IN: molecular_function unknown; INVOLVED IN: biological_process unknown; LOCATED IN: endomembrane system; EXPRESSED IN: 20 plant structures; EXPRESSED DURING: 10 growth stages; BEST Arabidopsis thaliana protein match is: unknown protein (TAIR:AT2G15830.1)
AT5G06800	*-1,08*		myb-like HTH transcriptional regulator family protein; CONTAINS InterPro DOMAIN/s: Homeodomain-like (InterPro:IPR009057), Myb, DNA-binding (InterPro:IPR014778), HTH transcriptional regulator, Myb-type, DNA-binding (InterPro:IPR017930), Myb-like DNA-binding domain, SHAQKYF class (InterPro:IPR006447), Homeodomain-related (InterPro:IPR012287); BEST Arabidopsis thaliana protein match is: Homeodomain-like superfamily protein (TAIR:AT3G04450.1)
AT1G77990	*-1,081*	SULPHATE TRANSPORTER 2;2 (SULTR2;2)	Encodes a low-affinity sulfate transporter.
AT2G41560	*-1,083*	AUTOINHIBITED CA(2+)-ATPASE, ISOFORM 4 (ACA4)	Encodes a calmodulin-regulated Ca(2+)-ATPase that improves salt tolerance in yeast. Localized to the vacuole. Lesion mimic phenotype when mutation in the gene is combined with a mutation in ACA11. Lesion mimic phenotype of double knockout can be suppressed by nutritional supplements that increase anion levels (e.g. 15 mM Nitrate, Chloride, or Phosphate).
AT5G18840	*-1,083*		Major facilitator superfamily protein; FUNCTIONS IN: carbohydrate transmembrane transporter activity, sugar:hydrogen symporter activity; INVOLVED IN: transport, transmembrane transport; LOCATED IN: integral to membrane, membrane; EXPRESSED IN: 17 plant structures; EXPRESSED DURING: 7 growth stages; CONTAINS InterPro DOMAIN/s: Major facilitator superfamily (InterPro:IPR020846), General substrate transporter (InterPro:IPR005828), Sugar/inositol transporter (InterPro:IPR003663), Major facilitator superfamily, general substrate transporter (InterPro:IPR016196); BEST Arabidopsis thaliana protein match is: Major facilitator superfamily protein (TAIR:AT2G48020.1)
AT1G02260	*-1,084*		Divalent ion symporter; FUNCTIONS IN: citrate transmembrane transporter activity, transporter activity; INVOLVED IN: citrate transport, transmembrane transport; LOCATED IN: endomembrane system, integral to membrane; EXPRESSED IN: 24 plant structures; EXPRESSED DURING: 13 growth stages; CONTAINS InterPro DOMAIN/s: Divalent ion symporter (InterPro:IPR004680)
AT1G15890	*-1,088*		Disease resistance protein (CC-NBS-LRR class) family; FUNCTIONS IN: ATP binding; INVOLVED IN: N-terminal protein myristoylation, apoptosis, defense response; LOCATED IN: cellular_component unknown; EXPRESSED IN: 8 plant structures; EXPRESSED DURING: 6 growth stages; CONTAINS InterPro DOMAIN/s: NB-ARC (InterPro:IPR002182), Leucine-rich repeat (InterPro:IPR001611), Disease resistance protein (InterPro:IPR000767); BEST Arabidopsis thaliana protein match is: Disease resistance protein (CC-NBS-LRR class) family (TAIR:AT5G43730.1)
AT1G41830	*-1,089*	SKU5-SIMILAR 6 (SKS6)	SKU5-similar 6 (SKS6); FUNCTIONS IN: pectinesterase activity; INVOLVED IN: oxidation reduction; LOCATED IN: apoplast, cell wall, membrane, plant-type cell wall; EXPRESSED IN: 23 plant structures; EXPRESSED DURING: 13 growth stages; CONTAINS InterPro DOMAIN/s: Cupredoxin (InterPro:IPR008972), Multicopper oxidase, type 2 (InterPro:IPR011706), Multicopper oxidase, type 3 (InterPro:IPR011707), Multicopper oxidase, type 1 (InterPro:IPR001117); BEST Arabidopsis thaliana protein match is: SKU5 similar 5 (TAIR:AT1G76160.1)

AT4G23680	*-1,091*		Polyketide cyclase/dehydrase and lipid transport superfamily protein; FUNCTIONS IN: molecular_function unknown; INVOLVED IN: response to biotic stimulus, defense response; LOCATED IN: cellular_component unknown; EXPRESSED IN: 19 plant structures; EXPRESSED DURING: 9 growth stages; CONTAINS InterPro DOMAIN/s: Bet v I allergen (InterPro:IPR000916); BEST Arabidopsis thaliana protein match is: Polyketide cyclase/dehydrase and lipid transport superfamily protein (TAIR:AT4G23670.1)
AT4G23670	*-1,093*		Polyketide cyclase/dehydrase and lipid transport superfamily protein; FUNCTIONS IN: copper ion binding; INVOLVED IN: response to cadmium ion, response to salt stress, defense response to bacterium; LOCATED IN: vacuole; EXPRESSED IN: 24 plant structures; EXPRESSED DURING: 14 growth stages; CONTAINS InterPro DOMAIN/s: Bet v I allergen (InterPro:IPR000916); BEST Arabidopsis thaliana protein match is: Polyketide cyclase/dehydrase and lipid transport superfamily protein (TAIR:AT4G23680.1)
AT1G35350	*-1,097*		EXS (ERD1/XPR1/SYG1) family protein; LOCATED IN: integral to membrane; EXPRESSED IN: 18 plant structures; EXPRESSED DURING: 9 growth stages; CONTAINS InterPro DOMAIN/s: EXS, C-terminal (InterPro:IPR004342), SPX, N-terminal (InterPro:IPR004331); BEST Arabidopsis thaliana protein match is: EXS (ERD1/XPR1/SYG1) family protein (TAIR:AT1G26730.1)
AT1G61050	*-1,098*		alpha 1,4-glycosyltransferase family protein; FUNCTIONS IN: transferase activity, transferring glycosyl groups, transferase activity; INVOLVED IN: biological_process unknown; LOCATED IN: Golgi stack; CONTAINS InterPro DOMAIN/s: Alpha 1,4-glycosyltransferase conserved region (InterPro:IPR007652), Glycosyltransferase, DXD sugar-binding region (InterPro:IPR007577); BEST Arabidopsis thaliana protein match is: alpha 1,4-glycosyltransferase family protein (TAIR:AT5G01250.1)
AT4G29690	*-1,1*		Alkaline-phosphatase-like family protein; FUNCTIONS IN: hydrolase activity, catalytic activity; INVOLVED IN: metabolic process, nucleotide metabolic process; LOCATED IN: endomembrane system; EXPRESSED IN: 9 plant structures; EXPRESSED DURING: LP.06 six leaves visible, LP.04 four leaves visible, 4 anthesis, petal differentiation and expansion stage, E expanded cotyledon stage; CONTAINS InterPro DOMAIN/s: Alkaline phosphatase-like, alpha/beta/alpha (InterPro:IPR017849), Type I phosphodiesterase/nucleotide pyrophosphatase/phosphate transferase (InterPro:IPR002591), Alkaline-phosphatase-like, core domain (InterPro:IPR017850); BEST Arabidopsis thaliana protein match is: Alkaline-phosphatase-like family protein (TAIR:AT4G29700.1)
AT3G06390	*-1,103*	CASP-LIKE PROTEIN 1D2 (CASPL1D2)	Uncharacterised protein family (UPF0497); CONTAINS InterPro DOMAIN/s: Uncharacterised protein family UPF0497, trans-membrane plant (InterPro:IPR006702), Uncharacterised protein family UPF0497, trans-membrane plant subgroup (InterPro:IPR006459); BEST Arabidopsis thaliana protein match is: Uncharacterised protein family (UPF0497) (TAIR:AT4G15610.1)
AT1G08590	*-1,107*	PXY-LIKE1 (PXL1)	Encodes one of the two putative eLRR kinase closely related to PXY (At1g08590/PXL1 and At4g28650/PXL2). Insertion mutants in either pxl1 or pxl2 do not exhibit an obvious phenotype in the stem; double-mutant combinations of a Col allele, of pxy (pxy-3) with pxl1 and pxl2, generate a more severe vascular phenotype than pxy-3 alone, suggesting that these genes act synergistically with PXY in regulating vascular-tissue development in the stem.
AT5G64240	*-1,107*	METACASPASE 3 (MC3)	Encodes a type I metacaspase. Two Arabidopsis metacaspases, AT1G02170 (MC1) and AT4G25110 (MC2) antagonistically control programmed cell death in Arabidopsis. MC1 is a positive regulator of cell death and requires conserved caspase-like putative catalytic residues for its function. MC2 negatively regulates cell death. This function is independent of the putative catalytic residues. A third type I Arabidopsis metacaspase is MC3 (AT5g64240).
AT3G11600	*-1,108*		unknown protein; FUNCTIONS IN: molecular_function unknown; INVOLVED IN: response to karrikin; EXPRESSED IN: 21 plant structures; EXPRESSED DURING: 12 growth stages; BEST Arabidopsis thaliana protein match is: unknown protein (TAIR:AT5G06270.1)
AT2G22800	*-1,109*	(HAT9)	Encodes homeobox protein HAT9.
AT1G73330	*-1,111*	DROUGHT-REPRESSED 4 (DR4)	encodes a plant-specific protease inhibitor-like protein whose transcript level in root disappears in response to progressive drought stress. The decrease in transcript level is independent from abscisic acid level.
AT2G45960	*-1,112*	PLASMA MEMBRANE INTRINSIC PROTEIN 1B (PIP1B)	a member of the plasma membrane intrinsic protein subfamily PIP1. localizes to the plasma membrane and exhibits water transport activity in Xenopus oocyte. expressed ubiquitously and protein level decreases slightly during leaf development.

AT5G02600	*-1,114*	SODIUM POTASSIUM ROOT DEFECTIVE 1 (NAKR1)	Heavy metal transport/detoxification superfamily protein ; FUNCTIONS IN: metal ion binding; INVOLVED IN: metal ion transport; LOCATED IN: chloroplast; EXPRESSED IN: 22 plant structures; EXPRESSED DURING: 12 growth stages; CONTAINS InterPro DOMAIN/s: Heavy metal transport/detoxification protein (InterPro:IPR006121); BEST Arabidopsis thaliana protein match is: Chloroplast-targeted copper chaperone protein (TAIR:AT2G37390.2)
AT4G35160	*-1,115*	N-ACETYLSEROTONIN O-METHYLTRANSFERASE (ASMT)	Encodes a cytosolic N-acetylserotonin O-methyltransferase that can convert N-acetylserotonin to melatonin and serotonin to 5-methoxytryptamine in the process of melatonin synthesis. It does not have caffeic acid O- methyltransferase activity.
AT1G04240	*-1,117*	SHORT HYPOCOTYL 2 (SHY2)	SHY2/IAA3 regulates multiple auxin responses in roots. It is induced rapidly by IAA, and has been shown to be phosphorylated by oat phytochrome A in vitro.
AT1G80920	*-1,118*	(J8)	A nuclear encoded soluble protein found in the chloroplast stroma. Negatively regulated by light and has rapid turnover in darkness.
AT3G62030	*-1,119*	ROTAMASE CYP 4 (ROC4)	nuclear-encoded chloroplast stromal cyclophilin CYP20-3 (also known as ROC4). Protein is tyrosine-phosphorylated and its phosphorylation state is modulated in response to ABA in Arabidopsis thaliana seeds.
AT1G18590	*-1,122*	SULFOTRANSFERASE 17 (SOT17)	encodes a desulfoglucosinolate sulfotransferase, involved in the final step of glucosinolate core structure biosynthesis. Has a broad-substrate specificity with preference with methionine-derived desulfoglucosinolates.
AT1G01190	*-1,123*	CYTOCHROME P450, FAMILY 78, SUBFAMILY A, POLYPEPTIDE 8 (CYP78A8)	member of CYP78A
AT1G29020	*-1,123*		Calcium-binding EF-hand family protein; FUNCTIONS IN: calcium ion binding; LOCATED IN: endomembrane system; EXPRESSED IN: root, callus; CONTAINS InterPro DOMAIN/s: EF-Hand 1, calcium-binding site (InterPro:IPR018247), EF-HAND 2 (InterPro:IPR018249), EF-hand-like domain (InterPro:IPR011992), Calcium-binding EF-hand (InterPro:IPR002048); BEST Arabidopsis thaliana protein match is: Calcium-binding EF-hand family protein (TAIR:AT1G29025.1)
AT5G44410	*-1,124*	(ATBBE27)	FAD-binding Berberine family protein; FUNCTIONS IN: electron carrier activity, oxidoreductase activity, FAD binding, catalytic activity; INVOLVED IN: oxidation reduction; LOCATED IN: endomembrane system; EXPRESSED IN: 22 plant structures; EXPRESSED DURING: 13 growth stages; CONTAINS InterPro DOMAIN/s: FAD-binding, type 2 (InterPro:IPR016166), Oxygen oxidoreductase covalent FAD-binding site (InterPro:IPR006093), Berberine/berberine-like (InterPro:IPR012951), FAD linked oxidase, N-terminal (InterPro:IPR006094); BEST Arabidopsis thaliana protein match is: FAD-binding Berberine family protein (TAIR:AT5G44440.1)
AT4G16980	*-1,126*		arabinogalactan-protein family; INVOLVED IN: biological_process unknown; LOCATED IN: endomembrane system; EXPRESSED IN: 22 plant structures; EXPRESSED DURING: 13 growth stages
AT2G44940	*-1,127*		encodes a member of the DREB subfamily A-4 of ERF/AP2 transcription factor family. The protein contains one AP2 domain. There are 17 members in this subfamily including TINY.
AT1G01070	*-1,128*	USUALLY MULTIPLE ACIDS MOVE IN AND OUT TRANSPORTERS 28 (UMAMIT28)	Encodes a plasma membrane-localized amino acid transporter likely involved in amino acid export in the developing seed.
AT4G31730	*-1,128*	GLUTAMINE DUMPER 1 (GDU1)	Glutamine dumper1 is a putative transmembrane protein. It is involved in glutamine secretion The mRNA is cell-to-cell mobile.
AT2G37750	*-1,132*		unknown protein
AT3G02610	*-1,132*	ACYL-ACYL CARRIER PROTEIN DESATURASE2 (AAD2)	Encodes one of two ∆9 palmitoyl-ACP desaturases responsible for the biosynthesis of ω-7 fatty acids in the maturing endosperm.
AT1G30720	*-1,135*	(ATBBE10)	FAD-binding Berberine family protein; FUNCTIONS IN: electron carrier activity, oxidoreductase activity, FAD binding, catalytic activity; LOCATED IN: endomembrane system; CONTAINS InterPro DOMAIN/s: FAD-binding, type 2 (InterPro:IPR016166), Berberine/berberine-like (InterPro:IPR012951), FAD linked oxidase, N-terminal (InterPro:IPR006094); BEST Arabidopsis thaliana protein match is: FAD-binding Berberine family protein (TAIR:AT1G30730.1)
AT4G09890	*-1,137*		Protein of unknown function (DUF3511); CONTAINS InterPro DOMAIN/s: Protein of unknown function DUF3511 (InterPro:IPR021899); BEST Arabidopsis thaliana protein match is: Protein of unknown function (DUF3511) (TAIR:AT5G11970.1)

AT5G44380	*-1,14*	(ATBBE28)	FAD-binding Berberine family protein; FUNCTIONS IN: electron carrier activity, oxidoreductase activity, FAD binding, catalytic activity; INVOLVED IN: response to oxidative stress; LOCATED IN: cell wall; EXPRESSED IN: shoot apex, hypocotyl, sepal, root, stamen; EXPRESSED DURING: 4 anthesis; CONTAINS InterPro DOMAIN/s: FAD-linked oxidase, FAD-binding, subdomain 2 (InterPro:IPR016168), FAD-binding, type 2 (InterPro:IPR016166), Berberine/berberine-like (InterPro:IPR012951), FAD linked oxidase, N-terminal (InterPro:IPR006094); BEST Arabidopsis thaliana protein match is: FAD-binding Berberine family protein (TAIR:AT5G44390.1)
AT3G01220	*-1,142*	HOMEOBOX PROTEIN 20 (HB20)	Encodes a homeodomain leucine zipper class I (HD-Zip I) protein. Expressed during seed germination in the micropylar endosperm and in the root cap, and increases ABA sensitivity and seed dormancy when mutated. The mRNA is cell-to-cell mobile.
AT2G38400	*-1,145*	ALANINE:GLYOXYLATE AMINOTRANSFERASE 3 (AGT3)	alanine:glyoxylate aminotransferase 2 homolog (AGT3) mRNA,
AT2G43150	*-1,146*		Proline-rich extensin-like family protein; FUNCTIONS IN: structural constituent of cell wall; INVOLVED IN: plant-type cell wall organization; LOCATED IN: endomembrane system; EXPRESSED IN: 16 plant structures; EXPRESSED DURING: 12 growth stages; CONTAINS InterPro DOMAIN/s: Extensin-like repeat (InterPro:IPR006706)
AT1G31885	*-1,147*	NOD26-LIKE INTRINSIC PROTEIN 3;1 (NIP3;1)	NOD26-like intrinsic protein 3;1 (NIP3;1); FUNCTIONS IN: transporter activity; INVOLVED IN: transport, transmembrane transport; LOCATED IN: integral to membrane, membrane; CONTAINS InterPro DOMAIN/s: Major intrinsic protein, conserved site (InterPro:IPR022357), Aquaporin (InterPro:IPR012269), Major intrinsic protein (InterPro:IPR000425); BEST Arabidopsis thaliana protein match is: NOD26-like intrinsic protein 1;2 (TAIR:AT4G18910.1)
AT3G14310	*-1,149*	PECTIN METHYLESTERASE 3 (PME3)	encodes a pectin methylesterase, targeted by a cellulose binding protein (CBP) from the parasitic nematode Heterodera schachtii during parasitism.
AT5G26660	*-1,149*	MYB DOMAIN PROTEIN 86 (MYB86)	myb domain protein 86 (MYB86); CONTAINS InterPro DOMAIN/s: SANT, DNA-binding (InterPro:IPR001005), Homeodomain-like (InterPro:IPR009057), Myb, DNA-binding (InterPro:IPR014778), HTH transcriptional regulator, Myb-type, DNA-binding (InterPro:IPR017930), Homeodomain-related (InterPro:IPR012287), Myb transcription factor (InterPro:IPR015495); BEST Arabidopsis thaliana protein match is: myb domain protein 50 (TAIR:AT1G57560.1)
AT1G04040	*-1,15*		HAD superfamily, subfamily IIIB acid phosphatase ; FUNCTIONS IN: acid phosphatase activity; INVOLVED IN: biological_process unknown; LOCATED IN: cell wall, plasma membrane, vacuole, plant-type cell wall; EXPRESSED IN: 19 plant structures; EXPRESSED DURING: 9 growth stages; CONTAINS InterPro DOMAIN/s: Acid phosphatase (Class B) (InterPro:IPR005519), Vegetative storage protein/acid phosphatase (InterPro:IPR014403), Acid phosphatase, plant (InterPro:IPR010028); BEST Arabidopsis thaliana protein match is: HAD superfamily, subfamily IIIB acid phosphatase (TAIR:AT5G44020.1)
AT1G66200	*-1,152*	GLUTAMINE SYNTHASE CLONE F11 (GSR2)	encodes a cytosolic glutamate synthetase, this enzyme has low affinity with substrate ammonium
AT2G34940	*-1,153*	VACUOLAR SORTING RECEPTOR 5 (VSR5)	VACUOLAR SORTING RECEPTOR 5 (VSR5); FUNCTIONS IN: calcium ion binding; INVOLVED IN: protein targeting to vacuole; LOCATED IN: integral to plasma membrane, Golgi transport complex; EXPRESSED IN: 14 plant structures; EXPRESSED DURING: 11 growth stages; CONTAINS InterPro DOMAIN/s: Protease-associated PA (InterPro:IPR003137), EGF-like calcium-binding, conserved site (InterPro:IPR018097), EGF-like calcium-binding (InterPro:IPR001881), Growth factor, receptor (InterPro:IPR009030); BEST Arabidopsis thaliana protein match is: VACUOLAR SORTING RECEPTOR 6 (TAIR:AT1G30900.1)
AT4G29800	*-1,154*	PATATIN-LIKE PROTEIN 8 (PLP8)	PATATIN-like protein 8 (PLP8); INVOLVED IN: metabolic process, lipid metabolic process; LOCATED IN: cellular_component unknown; EXPRESSED IN: sperm cell, hypocotyl, root; CONTAINS InterPro DOMAIN/s: Acyl transferase/acyl hydrolase/lysophospholipase (InterPro:IPR016035), Patatin (InterPro:IPR002641); BEST Arabidopsis thaliana protein match is: patatin-like protein 6 (TAIR:AT3G54950.1).

AT1G34040	-1,155		Pyridoxal phosphate (PLP)-dependent transferases superfamily protein; FUNCTIONS IN: pyridoxal phosphate binding, carbon-sulfur lyase activity, catalytic activity; INVOLVED IN: biological_process unknown; LOCATED IN: endomembrane system; CONTAINS InterPro DOMAIN/s: Pyridoxal phosphate-dependent transferase, major domain (InterPro:IPR015424), EGF-like, alliinase (InterPro:IPR006947), Pyridoxal phosphate-dependent transferase, major region, subdomain 1 (InterPro:IPR015421), Allinase, C-terminal (InterPro:IPR006948), Pyridoxal phosphate-dependent transferase, major region, subdomain 2 (InterPro:IPR015422); BEST Arabidopsis thaliana protein match is: Pyridoxal phosphate (PLP)-dependent transferases superfamily protein (TAIR:AT1G34060.1)
AT2G40320	-1,157	TRICHOME BIREFRINGENCE-LIKE 33 (TBL33)	Encodes a member of the TBL (TRICHOME BIREFRINGENCE-LIKE) gene family containing a plant-specific DUF231 (domain of unknown function) domain. TBL gene family has 46 members, two of which (TBR/AT5G06700 and TBL3/AT5G01360) have been shown to be involved in the synthesis and deposition of secondary wall cellulose, presumably by influencing the esterification state of pectic polymers. A nomenclature for this gene family has been proposed (Volker Bischoff & Wolf Scheible, 2010, personal communication). Chemical evidence for function comes from xylan NMR analysis. Secondary wall thickening phenotype can be only observed in double or triple mutant with esk1.
AT1G08500	-1,158	EARLY NODULIN-LIKE PROTEIN 18 (ENODL18)	early nodulin-like protein 18 (ENODL18); FUNCTIONS IN: electron carrier activity, copper ion binding; LOCATED IN: anchored to membrane; EXPRESSED IN: 14 plant structures; EXPRESSED DURING: LP.06 six leaves visible, 4 anthesis, petal differentiation and expansion stage, LP.08 eight leaves visible, E expanded cotyledon stage; CONTAINS InterPro DOMAIN/s: Plastocyanin-like (InterPro:IPR003245), Cupredoxin (InterPro:IPR008972); BEST Arabidopsis thaliana protein match is: Cupredoxin superfamily protein (TAIR:AT2G31050.1)
AT2G34770	-1,159	FATTY ACID HYDROXYLASE 1 (FAH1)	encodes a fatty acid hydroxylase, required for the AtBI-1-mediated suppression of programmed cell death.
AT4G02380	-1,159	SENESCENCE-ASSOCIATED GENE 21 (SAG21)	Encodes AtLEA5 (late embryogenesis abundant like protein). Also known as SENESCENCE-ASSOCIATED GENE 21 (SAG21). Has a role on oxidative stress tolerance. mRNA levels are elevated in response to various stresses.
AT3G50660	-1,16	DWARF 4 (DWF4)	Encodes a 22α hydroxylase whose reaction is a rate-limiting step in brassinosteroid biosynthetic pathway. The protein is a member of CYP90B gene family. CLM is an epi-allele with small, compressed rosette, reduced internode length, and reduced fertility, appears in selfed ddm mutant plants possibly due to loss of cytosine methylation. Transcripts accumulate in actively growing tissues, and GUS expression is negatively regulated by brassinosteroids. Localized in the endoplasmic reticulum. The in vitro expressed protein can perform the C-22 hydroxylation of a variety of C27-, C28- and C29-sterols. Cholesterol was the best substrate, followed by campesterol. Sitosterol was a poor substrate.
AT4G27710	-1,16	CYTOCHROME P450, FAMILY 709, SUBFAMILY B, POLYPEPTIDE 3 (CYP709B3)	member of CYP709B The mRNA is cell-to-cell mobile.
AT1G07560	-1,161		Leucine-rich repeat protein kinase family protein; FUNCTIONS IN: kinase activity; INVOLVED IN: protein amino acid phosphorylation; LOCATED IN: endomembrane system; EXPRESSED IN: root; CONTAINS InterPro DOMAIN/s: Protein kinase, ATP binding site (InterPro:IPR017441), Protein kinase, catalytic domain (InterPro:IPR000719), Serine/threonine-protein kinase-like domain (InterPro:IPR017442), Protein kinase-like domain (InterPro:IPR011009), Serine/threonine-protein kinase, active site (InterPro:IPR008271); BEST Arabidopsis thaliana protein match is: Leucine-rich repeat protein kinase family protein (TAIR:AT1G51820.1)
AT4G30460	-1,161		glycine-rich protein; FUNCTIONS IN: molecular_function unknown; INVOLVED IN: biological_process unknown; LOCATED IN: endomembrane system; EXPRESSED IN: 20 plant structures; EXPRESSED DURING: 12 growth stages
AT4G39670	-1,161		Glycolipid transfer protein (GLTP) family protein; FUNCTIONS IN: glycolipid transporter activity, glycolipid binding; INVOLVED IN: glycolipid transport; LOCATED IN: cytoplasm; EXPRESSED IN: 16 plant structures; EXPRESSED DURING: 10 growth stages; CONTAINS InterPro DOMAIN/s: Glycolipid transfer protein, GLTP (InterPro:IPR014830); BEST Arabidopsis thaliana protein match is: Glycolipid transfer protein (GLTP) family protein (TAIR:AT2G34690.1)

AT1G43650	*-1,163*	USUALLY MULTIPLE ACIDS MOVE IN AND OUT TRANSPORTERS 22 (UMAMIT22)	nodulin MtN21-like transporter family protein
AT3G54220	*-1,164*	SCARECROW (SCR)	Encodes a member of a novel family having similarity to DNA binding proteins containing basic-leucine zipper regions; scr is expressed in cortex/endodermal initial cells and in the endodermal cell lineage. Regulates the radial organization of the root. Is required cell-autonomously for distal specification of the quiescent center, which in turn regulates stem cell fate of immediately surrounding cells. SCR appears to be a direct target of SHR. SCR and SCR-LIKE 23 act redundantly in bundle sheath cell fate specification.
AT2G32150	*-1,166*		Haloacid dehalogenase-like hydrolase (HAD) superfamily protein; FUNCTIONS IN: hydrolase activity, catalytic activity; INVOLVED IN: metabolic process; LOCATED IN: cellular_component unknown; EXPRESSED IN: 24 plant structures; EXPRESSED DURING: 13 growth stages; CONTAINS InterPro DOMAIN/s: Haloacid dehalogenase-like hydrolase (InterPro:IPR005834), Pyrimidine 5-nucleotidase (InterPro:IPR010237), HAD-superfamily hydrolase, subfamily IA, variant 3 (InterPro:IPR006402); BEST Arabidopsis thaliana protein match is: Haloacid dehalogenase-like hydrolase (HAD) superfamily protein (TAIR:AT5G02230.2)
AT4G11880	*-1,167*	AGAMOUS-LIKE 14 (AGL14)	AGL12, AGL14, and AGL17 are all preferentially expressed in root tissues and therefore represent the only characterized MADS box genes expressed in roots. The mRNA is cell-to-cell mobile.
AT4G03500	*-1,168*		Ankyrin repeat family protein; CONTAINS InterPro DOMAIN/s: Ankyrin repeat-containing domain (InterPro:IPR020683), Ankyrin repeat (InterPro:IPR002110); BEST Arabidopsis thaliana protein match is: Ankyrin repeat family protein (TAIR:AT4G03460.1)
AT3G10660	*-1,169*	CALMODULIN-DOMAIN PROTEIN KINASE CDPK ISOFORM 2 (CPK2)	predicted to encode calcium-dependent protein kinase and is localized to the ER. Protein is myristoylated in a cell-free extract. Changing the proposed myristoylated site, G residue in the amino terminal, to A prevented the meristoylation . The G to A mutation decreased AtCPK2 membrane association by approximately 50%.
AT2G47140	*-1,172*	SHORT-CHAIN DEHYDROGENASE REDUCTASE 5 (SDR5)	NAD(P)-binding Rossmann-fold superfamily protein; FUNCTIONS IN: oxidoreductase activity, binding, catalytic activity; INVOLVED IN: oxidation reduction, metabolic process; LOCATED IN: cellular_component unknown; EXPRESSED IN: 6 plant structures; EXPRESSED DURING: 4 anthesis, LP.10 ten leaves visible, petal differentiation and expansion stage, LP.08 eight leaves visible, LP.12 twelve leaves visible; CONTAINS InterPro DOMAIN/s: NAD(P)-binding domain (InterPro:IPR016040), Glucose/ribitol dehydrogenase (InterPro:IPR002347), Short-chain dehydrogenase/reductase SDR (InterPro:IPR002198); BEST Arabidopsis thaliana protein match is: NAD(P)-binding Rossmann-fold superfamily protein (TAIR:AT2G47130.1)
AT2G01880	*-1,173*	PURPLE ACID PHOSPHATASE 7 (PAP7)	purple acid phosphatase 7 (PAP7); FUNCTIONS IN: protein serine/threonine phosphatase activity, acid phosphatase activity; LOCATED IN: endomembrane system; EXPRESSED IN: 7 plant structures; CONTAINS InterPro DOMAIN/s: Metallophosphoesterase (InterPro:IPR004843); BEST Arabidopsis thaliana protein match is: purple acid phosphatase 17 (TAIR:AT3G17790.1)
AT5G44160	*-1,18*	NUTCRACKER (NUC)	nutcracker (NUC); FUNCTIONS IN: sequence-specific DNA binding transcription factor activity, zinc ion binding, nucleic acid binding; INVOLVED IN: regulation of transcription; LOCATED IN: intracellular; EXPRESSED IN: primary root apical meristem, stem vascular system, embryo, root, embryonic root; CONTAINS InterPro DOMAIN/s: Zinc finger, C2H2-like (InterPro:IPR015880), Zinc finger, C2H2-type (InterPro:IPR007087); BEST Arabidopsis thaliana protein match is: C2H2 and C2HC zinc fingers superfamily protein (TAIR:AT1G03840.1)
AT5G24910	*-1,182*	CYTOCHROME P450, FAMILY 714, SUBFAMILY A, POLYPEPTIDE 1 (CYP714A1)	Member of CYP714A. Encodes one of the two tandemly duplicated gene pair ELA1 (CYP714A1) and ELA2 (CYP714A2), homologs of the rice cytochrome P450 monooxygenase gene EUI1. Double mutation of ELA1 and ELA2 results in increased biomass and enlarged organs.
AT5G46090	*-1,187*		Protein of unknown function (DUF679); FUNCTIONS IN: molecular_function unknown; INVOLVED IN: biological_process unknown; LOCATED IN: chloroplast; CONTAINS InterPro DOMAIN/s: Protein of unknown function DUF679 (InterPro:IPR007770); BEST Arabidopsis thaliana protein match is: Protein of unknown function (DUF679) (TAIR:AT4G18425.1)
AT2G40900	*-1,19*	USUALLY MULTIPLE ACIDS MOVE IN AND OUT TRANSPORTERS 11 (UMAMIT11)	Encodes a plasma membrane-localized amino acid transporter likely involved in amino acid export in the developing seed.

AT1G13970	*-1,191*		Protein of unknown function (DUF1336); FUNCTIONS IN: molecular_function unknown; INVOLVED IN: N-terminal protein myristoylation; EXPRESSED IN: 20 plant structures; EXPRESSED DURING: 10 growth stages; CONTAINS InterPro DOMAIN/s: Protein of unknown function DUF1336 (InterPro:IPR009769); BEST Arabidopsis thaliana protein match is: Protein of unknown function (DUF1336) (TAIR:AT3G29180.2)
AT4G29700	*-1,195*		Alkaline-phosphatase-like family protein; FUNCTIONS IN: hydrolase activity, catalytic activity; INVOLVED IN: metabolic process, nucleotide metabolic process; LOCATED IN: vacuole; EXPRESSED IN: 21 plant structures; EXPRESSED DURING: 12 growth stages; CONTAINS InterPro DOMAIN/s: Alkaline phosphatase-like, alpha/beta/alpha (InterPro:IPR017849), Type I phosphodiesterase/nucleotide pyrophosphatase/phosphate transferase (InterPro:IPR002591), Alkaline-phosphatase-like, core domain (InterPro:IPR017850); BEST Arabidopsis thaliana protein match is: Alkaline-phosphatase-like family protein (TAIR:AT4G29690.1)
AT5G01870	*-1,195*		Predicted to encode a PR (pathogenesis-related) protein. Belongs to the lipid transfer protein (PR-14) family with the following members: At2g38540/LTP1, At2g38530/LTP2, At5g59320/LTP3, At5g59310/LTP4, At3g51600/LTP5, At3g08770/LTP6, At2g15050/LTP7, At2g18370/LTP8, At2g15325/LTP9, At5g01870/LTP10, At4g33355/LTP11, At3g51590/LTP12, At5g44265/LTP13, At5g62065/LTP14, At4g08530/LTP15.
AT5G24800	*-1,195*	BASIC LEUCINE ZIPPER 9	Encodes bZIP protein BZO2H2.
AT4G14060	*-1,196*		Polyketide cyclase/dehydrase and lipid transport superfamily protein; FUNCTIONS IN: molecular_function unknown; INVOLVED IN: response to biotic stimulus, defense response; LOCATED IN: cellular_component unknown; EXPRESSED IN: cotyledon, root; CONTAINS InterPro DOMAIN/s: Bet v I allergen (InterPro:IPR000916); BEST Arabidopsis thaliana protein match is: MLP-like protein 328 (TAIR:AT2G01520.1)
AT1G29180	*-1,198*		Cysteine/Histidine-rich C1 domain family protein; CONTAINS InterPro DOMAIN/s: DC1 (InterPro:IPR004146), C1-like (InterPro:IPR011424); BEST Arabidopsis thaliana protein match is: Cysteine/Histidine-rich C1 domain family protein (TAIR:AT5G48320.1)
AT4G23980	*-1,199*	AUXIN RESPONSE FACTOR 9 (ARF9)	Encodes auxin response factor 9 (ARF9). The mRNA is cell-to-cell mobile.
AT1G48690	*-1,202*		Auxin-responsive GH3 family protein; CONTAINS InterPro DOMAIN/s: GH3 auxin-responsive promoter (InterPro:IPR004993); BEST Arabidopsis thaliana protein match is: Auxin-responsive GH3 family protein (TAIR:AT5G51470.1)
AT1G74770	*-1,204*		zinc ion binding; FUNCTIONS IN: zinc ion binding; INVOLVED IN: biological_process unknown; LOCATED IN: cellular_component unknown; EXPRESSED IN: hypocotyl, root; CONTAINS InterPro DOMAIN/s: Zinc finger, CTCHY-type (InterPro:IPR017921), Zinc finger, CHY-type (InterPro:IPR008913), Zinc finger, RING-type (InterPro:IPR001841), Haemerythrin/HHE cation-binding motif (InterPro:IPR012312); BEST Arabidopsis thaliana protein match is: zinc ion binding;zinc ion binding (TAIR:AT1G18910.1)
AT3G46130	*-1,204*	MYB DOMAIN PROTEIN 48 (MYB48)	Encodes a putative transcription factor (MYB48) that functions to regulate flavonol biosynthesis primarily in cotyledons.
AT5G63410	*-1,207*		Leucine-rich repeat protein kinase family protein; FUNCTIONS IN: protein serine/threonine kinase activity, protein kinase activity, ATP binding; INVOLVED IN: transmembrane receptor protein tyrosine kinase signaling pathway, protein amino acid phosphorylation; LOCATED IN: endomembrane system; EXPRESSED IN: 22 plant structures; EXPRESSED DURING: 13 growth stages; CONTAINS InterPro DOMAIN/s: Protein kinase, catalytic domain (InterPro:IPR000719), Leucine-rich repeat (InterPro:IPR001611), Serine-threonine/tyrosine-protein kinase (InterPro:IPR001245), Protein kinase-like domain (InterPro:IPR011009); BEST Arabidopsis thaliana protein match is: Leucine-rich repeat protein kinase family protein (TAIR:AT5G14210.1)
AT4G34600	*-1,211*		unknown protein; FUNCTIONS IN: molecular_function unknown; INVOLVED IN: pollen tube growth; LOCATED IN: endomembrane system; EXPRESSED IN: 11 plant structures; EXPRESSED DURING: M germinated pollen stage, LP.04 four leaves visible, 4 anthesis, petal differentiation and expansion stage; BEST Arabidopsis thaliana protein match is: unknown protein (TAIR:AT2G16385.1)
AT2G25090	*-1,213*	CBL-INTERACTING PROTEIN KINASE 16 (CIPK16)	Encodes a member of the SNF1-related kinase (SnRK) gene family (SnRK3.18), which has also been reported as a member of the CBL-interacting protein kinases (CIPK16) and is involved in salinity tolerance.

AT1G11450	*-1,214*	USUALLY MULTIPLE ACIDS MOVE IN AND OUT TRANSPORTERS 27 (UMAMIT27)	nodulin MtN21-like transporter family protein
AT4G15396	*-1,215*	CYTOCHROME P450, FAMILY 702, SUBFAMILY A, POLYPEPTIDE 6 (CYP702A6)	a member of the cytochrome P450 family. molecular function unknown.
AT5G09978	*-1,216*	ELICITOR PEPTIDE 7 PRECURSOR (PEP7)	elicitor peptide 7 precursor (PEP7)
AT1G18940	*-1,22*		Nodulin-like / Major Facilitator Superfamily protein; INVOLVED IN: transmembrane transport; LOCATED IN: endomembrane system; EXPRESSED IN: sperm cell, male gametophyte; EXPRESSED DURING: L mature pollen stage, M germinated pollen stage; CONTAINS InterPro DOMAIN/s: Nodulin-like (InterPro:IPR010658), Major facilitator superfamily MFS-1 (InterPro:IPR011701), Major facilitator superfamily, general substrate transporter (InterPro:IPR016196); BEST Arabidopsis thaliana protein match is: Nodulin-like / Major Facilitator Superfamily protein (TAIR:AT1G74780.1)
AT1G21110	*-1,221*	INDOLE GLUCOSINOLATE O-METHYLTRANSFERASE 3 (IGMT3)	O-methyltransferase family protein; FUNCTIONS IN: methyltransferase activity, O-methyltransferase activity, protein dimerization activity; LOCATED IN: cytosol; CONTAINS InterPro DOMAIN/s: Winged helix-turn-helix transcription repressor DNA-binding (InterPro:IPR011991), Plant methyltransferase dimerisation (InterPro:IPR012967), O-methyltransferase, family 2 (InterPro:IPR001077), O-methyltransferase, COMT, eukaryota (InterPro:IPR016461); BEST Arabidopsis thaliana protein match is: O-methyltransferase family protein (TAIR:AT1G21120.1)
AT5G44190	*-1,221*	GOLDEN2-LIKE 2 (GLK2)	Encodes GLK2, Golden2-like 2, one of a pair of partially redundant nuclear transcription factors that regulate chloroplast development in a cell-autonomous manner. GLK1, Golden2-like 1, is encoded by At2g20570. GLK1 and GLK2 regulate the expression of the photosynthetic apparatus.
AT1G30510	*-1,222*	ROOT FNR 2 (RFNR2)	Encodes a root-type ferredoxin:NADP(H) oxidoreductase.
AT1G60160	*-1,228*		Potassium transporter family protein; FUNCTIONS IN: potassium ion transmembrane transporter activity; INVOLVED IN: potassium ion transport; LOCATED IN: chloroplast; EXPRESSED IN: 21 plant structures; EXPRESSED DURING: 13 growth stages; CONTAINS InterPro DOMAIN/s: Potassium uptake protein, kup (InterPro:IPR018519), K+ potassium transporter (InterPro:IPR003855); BEST Arabidopsis thaliana protein match is: K+ uptake permease 7 (TAIR:AT5G09400.1)
AT1G51860	*-1,229*		Leucine-rich repeat protein kinase family protein; FUNCTIONS IN: kinase activity; INVOLVED IN: protein amino acid phosphorylation; LOCATED IN: endomembrane system; EXPRESSED IN: stem, root, stamen; EXPRESSED DURING: 4 anthesis; CONTAINS InterPro DOMAIN/s: Protein kinase, ATP binding site (InterPro:IPR017441), Protein kinase, catalytic domain (InterPro:IPR000719), Leucine-rich repeat (InterPro:IPR001611), Serine-threonine/tyrosine-protein kinase (InterPro:IPR001245), Protein kinase-like domain (InterPro:IPR011009), Serine/threonine-protein kinase, active site (InterPro:IPR008271); BEST Arabidopsis thaliana protein match is: root hair specific 6 (TAIR:AT1G51880.1)
AT2G26480	*-1,229*	UDP-GLUCOSYL TRANSFERASE 76D1 (UGT76D1)	UDP-glucosyl transferase 76D1 (UGT76D1); FUNCTIONS IN: UDP-glycosyltransferase activity, quercetin 7-O-glucosyltransferase activity, transferase activity, transferring glycosyl groups; INVOLVED IN: metabolic process; EXPRESSED IN: 10 plant structures; EXPRESSED DURING: 4 anthesis, LP.02 two leaves visible, petal differentiation and expansion stage, E expanded cotyledon stage, D bilateral stage; CONTAINS InterPro DOMAIN/s: UDP-glucuronosyl/UDP-glucosyltransferase (InterPro:IPR002213); BEST Arabidopsis thaliana protein match is: UDP-glucosyl transferase 76E1 (TAIR:AT5G59580.1)
AT5G50200	*-1,229*	WOUND-RESPONSIVE 3 (WR3)	Wound-responsive gene 3 (WR3). Encodes a high-affinity nitrate transporter. Up-regulated by nitrate. Involved in jasmonic acid-independent wound signal transduction.
AT3G01350	*-1,231*		Major facilitator superfamily protein; FUNCTIONS IN: transporter activity; INVOLVED IN: oligopeptide transport; LOCATED IN: membrane; EXPRESSED IN: 12 plant structures; EXPRESSED DURING: 6 growth stages; CONTAINS InterPro DOMAIN/s: Oligopeptide transporter (InterPro:IPR000109), Major facilitator superfamily, general substrate transporter (InterPro:IPR016196); BEST Arabidopsis thaliana protein match is: Major facilitator superfamily protein (TAIR:AT5G14940.1)
AT5G53550	*-1,232*	YELLOW STRIPE LIKE 3 (YSL3)	YELLOW STRIPE like 3 (YSL3); CONTAINS InterPro DOMAIN/s: Oligopeptide transporter OPT superfamily (InterPro:IPR004813); BEST Arabidopsis thaliana protein match is: YELLOW STRIPE like 2 (TAIR:AT5G24380.1)

AT1G13300	*-1,233*	HYPERSENSITIVITY TO LOW PI-ELICITED PRIMARY ROOT SHORTENING 1 (HRS1)	Encodes a nuclear localized member of the GARP family of transcription factors. Involved in nitrate/phosphate signaling in roots. It is transcriptionally regulated by nitrate and post transcriptionally by phospate and functions to integrate these two nutrient signaling pathways in the root.
AT1G26680	*-1,233*		transcriptional factor B3 family protein; FUNCTIONS IN: DNA binding, sequence-specific DNA binding transcription factor activity; INVOLVED IN: regulation of transcription, DNA-dependent; LOCATED IN: chloroplast; EXPRESSED IN: ovule, embryo; EXPRESSED DURING: D bilateral stage; CONTAINS InterPro DOMAIN/s: Transcriptional factor B3 (InterPro:IPR003340); BEST Arabidopsis thaliana protein match is: Transcriptional factor B3 family protein (TAIR:AT2G24645.1)
AT5G38550	*-1,235*		Mannose-binding lectin superfamily protein; CONTAINS InterPro DOMAIN/s: Mannose-binding lectin (InterPro:IPR001229); BEST Arabidopsis thaliana protein match is: Mannose-binding lectin superfamily protein (TAIR:AT5G38540.1)
AT3G50740	*-1,236*	UDP-GLUCOSYL TRANSFERASE 72E1 (UGT72E1)	UGT72E1 is an UDPG:coniferyl alcohol glucosyltransferase which specifically glucosylates sinapyl- and coniferyl aldehydes. The enzyme is thought to be involved in lignin metabolism.
AT3G55990	*-1,238*	ESKIMO 1 (ESK1)	Encodes ESK1 (Eskimo1). A member of a large gene family of DUF231 domain proteins whose members encode a total of 45 proteins of unknown function. ESK1 functions as a negative regulator of cold acclimation. Mutations in the ESK1 gene provides strong freezing tolerance. A member of the TBL (TRICHOME BIREFRINGENCE-LIKE) gene family containing a plant-specific DUF231 (domain of unknown function) domain. TBL gene family has 46 members, two of which (TBR/AT5G06700 and TBL3/AT5G01360) have been shown to be involved in the synthesis and deposition of secondary wall cellulose, presumably by influencing the esterification state of pectic polymers. A nomenclature for this gene family has been proposed (Volker Bischoff & Wolf Scheible, 2010, personal communication). The mRNA is cell-to-cell mobile.
AT2G36430	*-1,242*		Plant protein of unknown function (DUF247); CONTAINS InterPro DOMAIN/s: Protein of unknown function DUF247, plant (InterPro:IPR004158); BEST Arabidopsis thaliana protein match is: Plant protein of unknown function (DUF247) (TAIR:AT5G22550.2)
AT4G26220	*-1,244*	CAFFEOYL COENZYME A ESTER O-METHYLTRANSFERASE 7 (CCOAOMT7)	Encodes a caffeoyl-coenzyme A O-methyltransferase (CCoAOMT)-like protein with a strong preference for methylating the para position of flavanones and dihydroflavonols, whereas flavones and flavonols are methylated in the meta-position.
AT5G02640	*-1,244*		unknown protein; BEST Arabidopsis thaliana protein match is: unknown protein (TAIR:AT3G46300.1)
AT4G35030	*-1,246*		Protein kinase superfamily protein; FUNCTIONS IN: protein serine/threonine kinase activity, protein kinase activity, kinase activity, ATP binding; INVOLVED IN: protein amino acid phosphorylation; LOCATED IN: cellular_component unknown; EXPRESSED IN: 20 plant structures; EXPRESSED DURING: 12 growth stages; CONTAINS InterPro DOMAIN/s: Protein kinase, catalytic domain (InterPro:IPR000719), Serine/threonine-protein kinase domain (InterPro:IPR002290), Tyrosine-protein kinase, catalytic domain (InterPro:IPR020635), Serine/threonine-protein kinase-like domain (InterPro:IPR017442), Serine/threonine-protein kinase, active site (InterPro:IPR008271), Protein kinase-like domain (InterPro:IPR011009); BEST Arabidopsis thaliana protein match is: Protein kinase protein with adenine nucleotide alpha hydrolases-like domain (TAIR:AT2G16750.1).
AT5G64700	*-1,248*	USUALLY MULTIPLE ACIDS MOVE IN AND OUT TRANSPORTERS 21 (UMAMIT21)	nodulin MtN21-like transporter family protein
AT1G64210	*-1,252*		Leucine-rich repeat protein kinase family protein; FUNCTIONS IN: protein serine/threonine kinase activity, kinase activity, ATP binding; INVOLVED IN: transmembrane receptor protein tyrosine kinase signaling pathway, protein amino acid phosphorylation; LOCATED IN: mitochondrion; EXPRESSED IN: flower; EXPRESSED DURING: petal differentiation and expansion stage; CONTAINS InterPro DOMAIN/s: Protein kinase, ATP binding site (InterPro:IPR017441), Protein kinase, catalytic domain (InterPro:IPR000719), Leucine-rich repeat-containing N-terminal domain, type 2 (InterPro:IPR013210), Leucine-rich repeat (InterPro:IPR001611), Serine/threonine-protein kinase-like domain (InterPro:IPR017442), Protein kinase-like domain (InterPro:IPR011009); BEST Arabidopsis thaliana protein match is: Leucine-rich repeat protein kinase family protein (TAIR:AT4G23740.1)

AT2G16740	*-1,252*	UBIQUITIN-CONJUGATING ENZYME 29 (UBC29)	ubiquitin-conjugating enzyme 29 (UBC29); CONTAINS InterPro DOMAIN/s: Ubiquitin-conjugating enzyme/RWD-like (InterPro:IPR016135), Ubiquitin-conjugating enzyme, E2 (InterPro:IPR000608); BEST Arabidopsis thaliana protein match is: ubiquitin-conjugating enzyme 10 (TAIR:AT5G53300.4)
AT1G08340	*-1,253*		Rho GTPase activating protein with PAK-box/P21-Rho-binding domain; FUNCTIONS IN: Rac GTPase activator activity; INVOLVED IN: signal transduction; LOCATED IN: intracellular; EXPRESSED IN: 23 plant structures; EXPRESSED DURING: 13 growth stages; CONTAINS InterPro DOMAIN/s: PAK-box/P21-Rho-binding (InterPro:IPR000095), Rho GTPase activation protein (InterPro:IPR008936), RhoGAP (InterPro:IPR000198); BEST Arabidopsis thaliana protein match is: Rho GTPase activating protein with PAK-box/P21-Rho-binding domain (TAIR:AT5G22400.1)
AT5G56100	*-1,253*		glycine-rich protein / oleosin; FUNCTIONS IN: molecular_function unknown; INVOLVED IN: lipid storage; LOCATED IN: monolayer-surrounded lipid storage body, integral to membrane, membrane; EXPRESSED IN: 21 plant structures; EXPRESSED DURING: 12 growth stages; CONTAINS InterPro DOMAIN/s: Oleosin (InterPro:IPR000136)
AT3G26330	*-1,257*	CYTOCHROME P450, FAMILY 71, SUBFAMILY B, POLYPEPTIDE 37 (CYP71B37)	putative cytochrome P450
AT4G38830	*-1,257*	CYSTEINE-RICH RLK (RECEPTOR-LIKE PROTEIN KINASE) 26 (CRK26)	Encodes a cysteine-rich receptor-like protein kinase.
AT3G16560	*-1,258*		Protein phosphatase 2C family protein; FUNCTIONS IN: protein serine/threonine phosphatase activity, catalytic activity; EXPRESSED IN: 24 plant structures; EXPRESSED DURING: 15 growth stages; CONTAINS InterPro DOMAIN/s: Protein phosphatase 2C-related (InterPro:IPR001932), Protein phosphatase 2C (InterPro:IPR015655), Protein phosphatase 2C, N-terminal (InterPro:IPR014045); BEST Arabidopsis thaliana protein match is: pol-like 5 (TAIR:AT1G07630.1)
AT5G25810	*-1,258*	TINY (tny)	encodes a member of the DREB subfamily A-4 of ERF/AP2 transcription factor family (TINY). The protein contains one AP2 domain. There are 17 members in this subfamily including TINY. Ectopic or overexpression of this gene in a Ds tagged line has reduced cell expansion. The expression of this gene is induced by ethylene and light and appears to stimulate cytokinin biosynthesis.
AT1G77530	*-1,259*		O-methyltransferase family protein; CONTAINS InterPro DOMAIN/s: Winged helix-turn-helix transcription repressor DNA-binding (InterPro:IPR011991), Plant methyltransferase dimerisation (InterPro:IPR012967), O-methyltransferase, family 2 (InterPro:IPR001077), O-methyltransferase, COMT, eukaryota (InterPro:IPR016461); BEST Arabidopsis thaliana protein match is: O-methyltransferase family protein (TAIR:AT1G77520.1)
AT4G20390	*-1,26*	CASP-LIKE PROTEIN 1B2 (CASPL1B2)	Uncharacterised protein family (UPF0497); CONTAINS InterPro DOMAIN/s: Uncharacterised protein family UPF0497, trans-membrane plant (InterPro:IPR006702), Uncharacterised protein family UPF0497, trans-membrane plant subgroup (InterPro:IPR006459); BEST Arabidopsis thaliana protein match is: Uncharacterised protein family (UPF0497) (TAIR:AT5G44550.1)
AT4G37220	*-1,267*		Cold acclimation protein WCOR413 family; CONTAINS InterPro DOMAIN/s: Cold acclimation WCOR413 (InterPro:IPR008892); BEST Arabidopsis thaliana protein match is: cold-regulated 413-plasma membrane 2 (TAIR:AT3G50830.1)
AT5G64120	*-1,268*	PEROXIDASE 71 (PRX71)	Encodes a cell wall bound peroxidase that is induced by hypo-osmolarity and is involved in the lignification of cell walls.
AT1G64530	*-1,269*	NIN-LIKE PROTEIN 6 (NLP6)	Plant regulator RWP-RK family protein; CONTAINS InterPro DOMAIN/s: Octicosapeptide/Phox/Bem1p (InterPro:IPR000270), Plant regulator RWP-RK (InterPro:IPR003035); BEST Arabidopsis thaliana protein match is: NIN like protein 7 (TAIR:AT4G24020.1)
AT5G65210	*-1,269*	TGACG SEQUENCE-SPECIFIC BINDING PROTEIN 1 (TGA1)	Encodes TGA1, a redox-controlled regulator of systemic acquired resistance. TGA1 targets the activation sequence-1 (as-1) element of the promoter region of defense proteins. TGA1 are S-nitrosylated.
AT3G05890	*-1,27*	RARE-COLD-INDUCIBLE 2B (RCI2B)	RARE-COLD-INDUCIBLE 2B (RCI2B); CONTAINS InterPro DOMAIN/s: Uncharacterised protein family UPF0057 (InterPro:IPR000612); BEST Arabidopsis thaliana protein match is: Low temperature and salt responsive protein family (TAIR:AT3G05880.1)

AT5G07080	*-1,274*		HXXXD-type acyl-transferase family protein; FUNCTIONS IN: transferase activity, transferring acyl groups other than amino-acyl groups, transferase activity; INVOLVED IN: biological_process unknown; LOCATED IN: cellular_component unknown; EXPRESSED IN: 18 plant structures; EXPRESSED DURING: 9 growth stages; CONTAINS InterPro DOMAIN/s: Transferase (InterPro:IPR003480); BEST Arabidopsis thaliana protein match is: HXXXD-type acyl-transferase family protein (TAIR:AT3G47170.1)
AT1G49390	*-1,275*		2-oxoglutarate (2OG) and Fe(II)-dependent oxygenase superfamily protein; FUNCTIONS IN: oxidoreductase activity, acting on paired donors, with incorporation or reduction of molecular oxygen, 2-oxoglutarate as one donor, and incorporation of one atom each of oxygen into both donors, oxidoreductase activity; INVOLVED IN: flavonoid biosynthetic process; LOCATED IN: cellular_component unknown; EXPRESSED IN: hypocotyl, root, flower; EXPRESSED DURING: petal differentiation and expansion stage; CONTAINS InterPro DOMAIN/s: Oxoglutarate/iron-dependent oxygenase (InterPro:IPR005123); BEST Arabidopsis thaliana protein match is: 2-oxoglutarate (2OG) and Fe(II)-dependent oxygenase superfamily protein (TAIR:AT5G20400.1)
AT4G04830	*-1,276*	METHIONINE SULFOXIDE REDUCTASE B5 (MSRB5)	methionine sulfoxide reductase B5 (MSRB5); FUNCTIONS IN: peptide-methionine-(S)-S-oxide reductase activity; INVOLVED IN: oxidation reduction; LOCATED IN: cytosol; EXPRESSED IN: 24 plant structures; EXPRESSED DURING: 14 growth stages; CONTAINS InterPro DOMAIN/s: Methionine sulphoxide reductase B (InterPro:IPR002579), Mss4-like (InterPro:IPR011057); BEST Arabidopsis thaliana protein match is: methionine sulfoxide reductase B4 (TAIR:AT4G04810.1)
AT5G06930	*-1,276*		LOCATED IN: chloroplast; EXPRESSED IN: 15 plant structures; EXPRESSED DURING: 7 growth stages; BEST Arabidopsis thaliana protein match is: nucleolar protein gar2-related (TAIR:AT2G42320.2)
AT1G35250	*-1,279*	ACYL-LIPID THIOESTERASE 2 (ALT2)	Thioesterase superfamily protein; CONTAINS InterPro DOMAIN/s: Thioesterase superfamily (InterPro:IPR006683); BEST Arabidopsis thaliana protein match is: Thioesterase superfamily protein (TAIR:AT1G35290.1)
AT5G28640	*-1,282*	ANGUSTIFOLIA 3 (AN3)	Encodes a protein with similarity to mammalian transcriptional coactivator that is involved in cell proliferation during leaf and flower development. Loss of function mutations have narrow, pointed leaves and narrow floral organs. AN3 interacts with members of the growth regulating factor (GRF) family of transcription factors.
AT5G48940	*-1,287*	(RGFR2)	RGFR2 is a leucine--rich repeat receptor kinase that, together with RGFR1 and RGFR3, binds ROOT GROWTH FACTORS and is required for establishing the gradient of PLETHORA1 and PLETHORA2 essential for proper root growth and development.
AT1G61590	*-1,289*		Protein kinase superfamily protein; FUNCTIONS IN: protein serine/threonine kinase activity, protein kinase activity, kinase activity, ATP binding; INVOLVED IN: protein amino acid phosphorylation; LOCATED IN: chloroplast; EXPRESSED IN: 6 plant structures; EXPRESSED DURING: 4 anthesis, petal differentiation and expansion stage; CONTAINS InterPro DOMAIN/s: Protein kinase, ATP binding site (InterPro:IPR017441), Protein kinase, catalytic domain (InterPro:IPR000719), Serine-threonine/tyrosine-protein kinase (InterPro:IPR001245), Protein kinase-like domain (InterPro:IPR011009), Serine/threonine-protein kinase, active site (InterPro:IPR008271); BEST Arabidopsis thaliana protein match is: Protein kinase superfamily protein (TAIR:AT2G05940.1)
AT4G30450	*-1,289*		glycine-rich protein
AT5G19040	*-1,289*	ISOPENTENYLTRANSFERASE 5 (IPT5)	Encodes cytokinin synthase.
AT1G35580	*-1,291*	CYTOSOLIC INVERTASE 1 (CINV1)	CINV1 / A/N-InvG is an alkaline/neutral invertase that breaks sucrose down into fructose and glucose (GH100). The exact localization of CINV1 remains under investigation but there is evidence that fluorescently-tagged CINV1 localizes to the cytoplasm. atinvg mutants have reduced root growth, reduced invertase activity, and increased expression of antioxidant genes under basal conditions. The levels of CINV1 / A/N-InvG transcripts rise in response to a hydrogen peroxide treatment. The protein has been shown to interact with PIP5K9.
AT2G26250	*-1,295*	3-KETOACYL-COA SYNTHASE 10 (KCS10)	epidermis-specific, encodes KCS10, a putative 3-ketoacyl-CoA synthase. probably involved in the synthesis of long-chain lipids found in the cuticle.

AT2G37130	*-1,296*		Peroxidase superfamily protein; FUNCTIONS IN: peroxidase activity, heme binding; INVOLVED IN: defense response to fungus; LOCATED IN: endomembrane system; EXPRESSED IN: 15 plant structures; EXPRESSED DURING: 7 growth stages; CONTAINS InterPro DOMAIN/s: Haem peroxidase (InterPro:IPR010255), Plant peroxidase (InterPro:IPR000823), Haem peroxidase, plant/fungal/bacterial (InterPro:IPR002016); BEST Arabidopsis thaliana protein match is: Peroxidase superfamily protein (TAIR:AT4G21960.1)
AT1G75680	*-1,297*	GLYCOSYL HYDROLASE 9B7 (GH9B7)	glycosyl hydrolase 9B7 (GH9B7); FUNCTIONS IN: hydrolase activity, hydrolyzing O-glycosyl compounds, catalytic activity; INVOLVED IN: carbohydrate metabolic process; LOCATED IN: plasma membrane; EXPRESSED IN: 23 plant structures; EXPRESSED DURING: 13 growth stages; CONTAINS InterPro DOMAIN/s: Six-hairpin glycosidase (InterPro:IPR012341), Glycoside hydrolase, family 9, active site (InterPro:IPR018221), Six-hairpin glycosidase-like (InterPro:IPR008928), Glycoside hydrolase, family 9 (InterPro:IPR001701); BEST Arabidopsis thaliana protein match is: glycosyl hydrolase 9B5 (TAIR:AT1G19940.1)
AT5G62720	*-1,297*		Integral membrane HPP family protein; FUNCTIONS IN: molecular_function unknown; INVOLVED IN: biological_process unknown; LOCATED IN: integral to membrane, chloroplast inner membrane, chloroplast envelope; EXPRESSED IN: 22 plant structures; EXPRESSED DURING: 13 growth stages; CONTAINS InterPro DOMAIN/s: HPP (InterPro:IPR007065); BEST Arabidopsis thaliana protein match is: Integral membrane HPP family protein (TAIR:AT3G47980.1)
AT5G65980	*-1,297*	PIN-LIKES 7 (PILS7)	Auxin efflux carrier family protein; FUNCTIONS IN: auxin:hydrogen symporter activity; INVOLVED IN: auxin polar transport, transmembrane transport; LOCATED IN: endomembrane system, integral to membrane; CONTAINS InterPro DOMAIN/s: Auxin efflux carrier (InterPro:IPR004776); BEST Arabidopsis thaliana protein match is: Auxin efflux carrier family protein (TAIR:AT2G17500.4)
AT2G26820	*-1,303*	PHLOEM PROTEIN 2-A3 (PP2-A3)	phloem protein 2-A3 (PP2-A3); CONTAINS InterPro DOMAIN/s: AIG1 (InterPro:IPR006703); BEST Arabidopsis thaliana protein match is: P-loop containing nucleoside triphosphate hydrolases superfamily protein (TAIR:AT1G33900.1)
AT3G61830	*-1,303*	AUXIN RESPONSE FACTOR 18 (ARF18)	auxin response factor 18 (ARF18); FUNCTIONS IN: sequence-specific DNA binding transcription factor activity; INVOLVED IN: response to hormone stimulus, regulation of transcription, DNA-dependent, regulation of transcription; LOCATED IN: nucleus; EXPRESSED IN: 22 plant structures; EXPRESSED DURING: 13 growth stages; CONTAINS InterPro DOMAIN/s: Aux/IAA-ARF-dimerisation (InterPro:IPR011525), Transcriptional factor B3 (InterPro:IPR003340), AUX/IAA protein (InterPro:IPR003311), Auxin response factor (InterPro:IPR010525); BEST Arabidopsis thaliana protein match is: auxin response factor 11 (TAIR:AT2G46530.1)
AT3G18660	*-1,307*	PLANT GLYCOGENIN-LIKE STARCH INITIATION PROTEIN 1 (PGSIP1)	Plants expressing an RNAi construct specifically targeting PGSIP1 was shown to have a dramatically reduced amount of starch. Encodes a glucuronyltransferase responsible for the addition of GlcA residues onto xylan and for secondary wall deposition.
AT1G63530	*-1,311*		BEST Arabidopsis thaliana protein match is: hydroxyproline-rich glycoprotein family protein (TAIR:AT1G63540.1)
AT1G19790	*-1,312*	SHI-RELATED SEQUENCE 7 (SRS7)	A member of SHI gene family. Arabidopsis thaliana has ten members that encode proteins with a RING finger-like zinc finger motif. Despite being highly divergent in sequence, many of the SHI-related genes are partially redundant in function and synergistically promote gynoecium, stamen and leaf development in Arabidopsis.
AT5G61420	*-1,32*	MYB DOMAIN PROTEIN 28 (MYB28)	Encodes a nuclear localized member of the MYB transcription factor family. Involved in positive regulation of aliphatic glucosinolate biosynthesis.Expression is induced by touch, wounding and glucose.
AT4G00880	*-1,323*	SMALL AUXIN UPREGULATED RNA 31 (SAUR31)	SAUR-like auxin-responsive protein family ; CONTAINS InterPro DOMAIN/s: Auxin responsive SAUR protein (InterPro:IPR003676); BEST Arabidopsis thaliana protein match is: SAUR-like auxin-responsive protein family (TAIR:AT2G46690.1)
AT1G07610	*-1,324*	METALLOTHIONEIN 1C (MT1C)	one of the five metallothioneins (MTs) genes identified in Arabidopsis. MTs are cysteine-rich proteins required for heavy metal tolerance. The mRNA is cell-to-cell mobile.
AT2G01530	*-1,325*	MLP-LIKE PROTEIN 329 (MLP329)	MLP-like protein 329 (MLP329); FUNCTIONS IN: copper ion binding; INVOLVED IN: response to biotic stimulus, defense response; LOCATED IN: cellular_component unknown; EXPRESSED IN: hypocotyl, root; CONTAINS InterPro DOMAIN/s: Bet v I allergen (InterPro:IPR000916); BEST Arabidopsis thaliana protein match is: MLP-like protein 328 (TAIR:AT2G01520.1)

AT1G05260	*-1,326*	RARE COLD INDUCIBLE GENE 3 (RCI3)	Encodes a cold-inducible cationic peroxidase that is involved in the stress response. In response to low temperature, RCI3 transcripts accumulate in the aerial part and in roots of etiolated seedlings but only in roots of light-grown seedlings. The mRNA is cell-to-cell mobile.
AT1G78320	*-1,326*	GLUTATHIONE S-TRANSFERASE TAU 23	Encodes glutathione transferase belonging to the tau class of GSTs. Naming convention according to Wagner et al. (2002).
AT5G01550	*-1,326*	L-TYPE LECTIN RECEPTOR KINASE VI.3 (LECRK-VI.3)	Encodes LecRKA4.2, a member of the lectin receptor kinase subfamily A4 (LecRKA4.1 At5g01540; LecRKA4.2 At5g01550; LecRKA4.3 At5g01560). Together with other members of the subfamily, functions redundantly in the negative regulation of ABA response in seed germination.
AT2G21560	*-1,327*		unknown protein; BEST Arabidopsis thaliana protein match is: unknown protein (TAIR:AT4G39190.1)
AT5G45380	*-1,327*	DEGRADATION OF UREA 3 (DUR3)	DEGRADATION OF UREA 3 (DUR3); CONTAINS InterPro DOMAIN/s: Sodium/solute symporter, subgroup (InterPro:IPR019900), Sodium/solute symporter (InterPro:IPR001734)
AT1G52200	*-1,329*		PLAC8 family protein; FUNCTIONS IN: molecular_function unknown; INVOLVED IN: response to oxidative stress; LOCATED IN: plasma membrane; EXPRESSED IN: 10 plant structures; EXPRESSED DURING: 6 growth stages; CONTAINS InterPro DOMAIN/s: Protein of unknown function Cys-rich (InterPro:IPR006461); BEST Arabidopsis thaliana protein match is: PLAC8 family protein (TAIR:AT3G18450.1)
AT2G27000	*-1,329*	CYTOCHROME P450, FAMILY 705, SUBFAMILY A, POLYPEPTIDE 8 (CYP705A8)	member of CYP705A
AT1G29670	*-1,331*		GDSL-motif esterase/acyltransferase/lipase. Enzyme group with broad substrate specificity that may catalyze acyltransfer or hydrolase reactions with lipid and non-lipid substrates. The mRNA is cell-to-cell mobile.
AT2G28160	*-1,331*	FER-LIKE REGULATOR OF IRON UPTAKE (FRU)	Encodes a putative transcription factor that regulates iron uptake responses. mRNA is detected in the outer cell layers of the root and accumulates in response to iron deficiency. The expression of many iron-regulated genes is dependent on FIT1. It specifically regulates FRO2 at the level of mRNA accumulation and IRT1 at the level of protein accumulation.Similar to FER in tomato and is a regulator of iron uptake. It is post-transcriptionally controlled.
AT2G26410	*-1,345*	IQ-DOMAIN 4 (Iqd4)	IQ-domain 4 (Iqd4); CONTAINS InterPro DOMAIN/s: IQ calmodulin-binding region (InterPro:IPR000048); BEST Arabidopsis thaliana protein match is: IQ-domain 2 (TAIR:AT5G03040.3)
AT4G40070	*-1,346*		RING/U-box superfamily protein; FUNCTIONS IN: zinc ion binding; EXPRESSED IN: 9 plant structures; EXPRESSED DURING: 4 anthesis, petal differentiation and expansion stage; CONTAINS InterPro DOMAIN/s: Zinc finger, RING-type (InterPro:IPR001841), Zinc finger, C3HC4 RING-type (InterPro:IPR018957); BEST Arabidopsis thaliana protein match is: RING/U-box superfamily protein (TAIR:AT3G05200.1)
AT5G60020	*-1,346*	LACCASE 17 (LAC17)	LAC17 appears to have laccase activity based on enzyme assays performed using lac17 mutants. Notably, these mutants appear to have a reduced deposition of G lignin units. LAC17 is expressed in interfascicular fibers and likely contributes to lignin biosynthesis, and hence, cell wall biosynthesis, there.
AT2G26980	*-1,347*	CBL-INTERACTING PROTEIN KINASE 3 (CIPK3)	encodes a serine-threonine protein kinase whose expression increases in response to abscisic acid, cold, drought, high salt, and wounding conditions. The gene is expressed in developing seeds and seedlings. Lines carrying a T-DNA insertions have reduced germination efficiency and expression of cold, high-salt, and abscisic acid marker genes are altered, but not drought-response markers.
AT5G10210	*-1,348*		CONTAINS InterPro DOMAIN/s: C2 calcium-dependent membrane targeting (InterPro:IPR000008); BEST Arabidopsis thaliana protein match is: unknown protein (TAIR:AT5G65030.1)
AT1G49450	*-1,349*		Transducin/WD40 repeat-like superfamily protein; CONTAINS InterPro DOMAIN/s: WD40 repeat 2 (InterPro:IPR019782), WD40 repeat-like-containing domain (InterPro:IPR011046), WD40-repeat-containing domain (InterPro:IPR017986), WD40/YVTN repeat-like-containing domain (InterPro:IPR015943), WD40 repeat (InterPro:IPR001680), WD40 repeat, subgroup (InterPro:IPR019781), G-protein beta WD-40 repeat, region (InterPro:IPR020472); BEST Arabidopsis thaliana protein match is: Transducin/WD40 repeat-like superfamily protein (TAIR:AT3G18950.1)

AT1G73300	*-1,349*	SERINE CARBOXYPEPTIDASE-LIKE 2 (scpl2)	serine carboxypeptidase-like 2 (scpl2); FUNCTIONS IN: serine-type carboxypeptidase activity; INVOLVED IN: proteolysis; LOCATED IN: endomembrane system; CONTAINS InterPro DOMAIN/s: Peptidase S10, serine carboxypeptidase (InterPro:IPR001563); BEST Arabidopsis thaliana protein match is: serine carboxypeptidase-like 1 (TAIR:AT5G36180.1)
AT1G26450	*-1,351*		Carbohydrate-binding X8 domain superfamily protein; CONTAINS InterPro DOMAIN/s: X8 (InterPro:IPR012946); BEST Arabidopsis thaliana protein match is: plasmodesmata callose-binding protein 3 (TAIR:AT1G18650.1)
AT5G61250	*-1,354*	GLUCURONIDASE 1 (GUS1)	Belongs to the plant glycoside hydrolase family 79. Encodes a protein with several posttranslational modification sites including O-β-GlcNAc attachment sites and serine-, threonine- and tyrosine-phosphorylation sites, suggesting that this protein is extensively modified posttranslationally. The protein is predicted (WoLF PSORT program) to be secreted.
AT1G21210	*-1,356*	WALL ASSOCIATED KINASE 4 (WAK4)	cell wall-associated ser/thr kinase involved in cell elongation and lateral root development
AT1G01180	*-1,359*		S-adenosyl-L-methionine-dependent methyltransferases superfamily protein; FUNCTIONS IN: methyltransferase activity; INVOLVED IN: lipid biosynthetic process; EXPRESSED IN: sperm cell, hypocotyl; CONTAINS InterPro DOMAIN/s: Rhamnosyl O-methyltransferase/Cephalosporin hydroxylase (InterPro:IPR007072)
AT1G58270	*-1,361*	(ZW9)	ZW9 mRNA, complete cds The mRNA is cell-to-cell mobile.
AT1G21440	*-1,363*		Phosphoenolpyruvate carboxylase family protein; FUNCTIONS IN: isocitrate lyase activity, catalytic activity; INVOLVED IN: metabolic process; LOCATED IN: chloroplast, chloroplast stroma; EXPRESSED IN: 22 plant structures; EXPRESSED DURING: 14 growth stages; CONTAINS InterPro DOMAIN/s: Pyruvate/Phosphoenolpyruvate kinase, catalytic core (InterPro:IPR015813), Isocitrate lyase/phosphorylmutase, conserved site (InterPro:IPR018523), Isocitrate lyase/phosphorylmutase (InterPro:IPR000918); BEST Arabidopsis thaliana protein match is: Phosphoenolpyruvate carboxylase family protein (TAIR:AT1G77060.1)
AT2G31790	*-1,363*		UDP-Glycosyltransferase superfamily protein; FUNCTIONS IN: UDP-glycosyltransferase activity, transferase activity, transferring glycosyl groups; INVOLVED IN: metabolic process; LOCATED IN: cellular_component unknown; EXPRESSED IN: 22 plant structures; EXPRESSED DURING: 13 growth stages; CONTAINS InterPro DOMAIN/s: UDP-glucuronosyl/UDP-glucosyltransferase (InterPro:IPR002213); BEST Arabidopsis thaliana protein match is: Uridine diphosphate glycosyltransferase 74E2 (TAIR:AT1G05680.1)
AT2G47160	*-1,371*	REQUIRES HIGH BORON 1 (BOR1)	Boron transporter. Protein accumulates in shoots and roots under conditions of boron deficiency and is degraded within several hours of restoring boron supply. Localized to the plasma membrane under B limitation, and to the cytoplasm after B application before degradation. Protein is transferred via the endosomes to the vacuole for degradation. Localized to the inner plasma membrane domain in the columella, lateral root cap, epidermis, and endodermis in the root tip region, and in the epidermis and endodermis in the elongation zone. Under high-boron is transported to the vacuole for degradation.
AT3G21550	*-1,373*	DUF679 DOMAIN MEMBRANE PROTEIN 2 (DMP2)	DUF679 domain membrane protein 2 (DMP2); CONTAINS InterPro DOMAIN/s: Protein of unknown function DUF679 (InterPro:IPR007770); BEST Arabidopsis thaliana protein match is: DUF679 domain membrane protein 1 (TAIR:AT3G21520.1)
AT1G75520	*-1,375*	SHI-RELATED SEQUENCE 5 (SRS5)	A member of SHI gene family. Arabidopsis thaliana has ten members that encode proteins with a RING finger-like zinc finger motif. Despite being highly divergent in sequence, many of the SHI-related genes are partially redundant in function and synergistically promote gynoecium, stamen and leaf development in Arabidopsis.
AT4G32000	*-1,376*		Protein kinase superfamily protein; FUNCTIONS IN: protein serine/threonine kinase activity, protein kinase activity, kinase activity, ATP binding; INVOLVED IN: protein amino acid phosphorylation; LOCATED IN: endomembrane system; CONTAINS InterPro DOMAIN/s: Protein kinase, ATP binding site (InterPro:IPR017441), Serine/threonine-protein kinase domain (InterPro:IPR002290), Serine/threonine-protein kinase-like domain (InterPro:IPR017442), Protein kinase-like domain (InterPro:IPR011009), Serine/threonine-protein kinase, active site (InterPro:IPR008271), Protein kinase, catalytic domain (InterPro:IPR000719), Tyrosine-protein kinase, catalytic domain (InterPro:IPR020635); BEST Arabidopsis thaliana protein match is: Protein kinase superfamily protein (TAIR:AT2G25220.1).
AT5G51890	*-1,377*		encodes peroxidase involved in the lignification of tracheary elements (TE) in roots

AT2G28970	*-1,378*		Leucine-rich repeat protein kinase family protein; FUNCTIONS IN: protein serine/threonine kinase activity, kinase activity, ATP binding; INVOLVED IN: transmembrane receptor protein tyrosine kinase signaling pathway, protein amino acid phosphorylation; LOCATED IN: endomembrane system; EXPRESSED IN: root; CONTAINS InterPro DOMAIN/s: Protein kinase, ATP binding site (InterPro:IPR017441), Protein kinase, catalytic domain (InterPro:IPR000719), Leucine-rich repeat (InterPro:IPR001611), Serine/threonine-protein kinase-like domain (InterPro:IPR017442), Protein kinase-like domain (InterPro:IPR011009), Serine/threonine-protein kinase, active site (InterPro:IPR008271); BEST Arabidopsis thaliana protein match is: Leucine-rich repeat protein kinase family protein (TAIR:AT2G28990.1)
AT5G36180	*-1,38*	SERINE CARBOXYPEPTIDASE-LIKE 1 (scpl1)	serine carboxypeptidase-like 1 (scpl1); FUNCTIONS IN: serine-type carboxypeptidase activity; INVOLVED IN: proteolysis; LOCATED IN: endomembrane system; CONTAINS InterPro DOMAIN/s: Peptidase S10, serine carboxypeptidase (InterPro:IPR001563); BEST Arabidopsis thaliana protein match is: serine carboxypeptidase-like 2 (TAIR:AT1G73300.1)
AT1G20840	*-1,381*	TONOPLAST MONOSACCHARIDE TRANSPORTER1 (TMT1)	The protein encoded by this gene is found in the tonoplast (vacuole membrane) of Arabidopsis cells. The gene is expressed at highest levels in juvenile (sink) and adult (source) leaves, followed by flower tissues.
AT3G16920	*-1,383*	CHITINASE-LIKE PROTEIN 2 (CTL2)	Encodes a chitinase-like protein expressed predominantly in stems. Mutants accumulate ligning in etiolated hypocotyls.
AT1G14190	*-1,385*		Glucose-methanol-choline (GMC) oxidoreductase family protein; FUNCTIONS IN: aldehyde-lyase activity, oxidoreductase activity, acting on CH-OH group of donors, FAD binding; INVOLVED IN: alcohol metabolic process; LOCATED IN: cellular_component unknown; CONTAINS InterPro DOMAIN/s: Glucose-methanol-choline oxidoreductase, N-terminal (InterPro:IPR000172), Glucose-methanol-choline oxidoreductase (InterPro:IPR012132), Glucose-methanol-choline oxidoreductase, C-terminal (InterPro:IPR007867); BEST Arabidopsis thaliana protein match is: Glucose-methanol-choline (GMC) oxidoreductase family protein (TAIR:AT1G14185.1)
AT2G15620	*-1,387*	NITRITE REDUCTASE 1 (NIR1)	Involved in the second step of nitrate assimilation. Its expression is induced by nitrate. The mRNA is cell-to-cell mobile.
AT5G56870	*-1,389*	BETA-GALACTOSIDASE 4 (BGAL4)	beta-galactosidase 4 (BGAL4); FUNCTIONS IN: beta-galactosidase activity; INVOLVED IN: lactose catabolic process, using glucoside 3-dehydrogenase, carbohydrate metabolic process, lactose catabolic process via UDP-galactose, lactose catabolic process; LOCATED IN: endomembrane system; EXPRESSED IN: 25 plant structures; EXPRESSED DURING: 12 growth stages; CONTAINS InterPro DOMAIN/s: Glycoside hydrolase, family 35, conserved site (InterPro:IPR019801), Glycoside hydrolase, family 35 (InterPro:IPR001944), Glycoside hydrolase, catalytic core (InterPro:IPR017853), Glycoside hydrolase, subgroup, catalytic core (InterPro:IPR013781), Galactose-binding domain-like (InterPro:IPR008979); BEST Arabidopsis thaliana protein match is: beta-galactosidase 12 (TAIR:AT4G26140.1)
AT2G22170	*-1,391*	PLAT DOMAIN PROTEIN 2 (PLAT2)	Lipase/lipooxygenase, PLAT/LH2 family protein; FUNCTIONS IN: molecular_function unknown; INVOLVED IN: biological_process unknown; LOCATED IN: thylakoid, chloroplast, membrane; EXPRESSED IN: 19 plant structures; EXPRESSED DURING: 13 growth stages; CONTAINS InterPro DOMAIN/s: Lipoxygenase, LH2 (InterPro:IPR001024), Lipase/lipooxygenase, PLAT/LH2 (InterPro:IPR008976); BEST Arabidopsis thaliana protein match is: Lipase/lipooxygenase, PLAT/LH2 family protein (TAIR:AT4G39730.1)
AT3G48920	*-1,391*	MYB DOMAIN PROTEIN 45 (MYB45)	Member of the R2R3 factor gene family.
AT5G39000	*-1,392*		Malectin/receptor-like protein kinase family protein; FUNCTIONS IN: kinase activity; INVOLVED IN: protein amino acid phosphorylation; LOCATED IN: endomembrane system; CONTAINS InterPro DOMAIN/s: Protein kinase, ATP binding site (InterPro:IPR017441), Malectin/receptor-like protein kinase (InterPro:IPR021720), Protein kinase, catalytic domain (InterPro:IPR000719), Serine-threonine/tyrosine-protein kinase (InterPro:IPR001245), Protein kinase-like domain (InterPro:IPR011009), Serine/threonine-protein kinase, active site (InterPro:IPR008271); BEST Arabidopsis thaliana protein match is: Malectin/receptor-like protein kinase family protein (TAIR:AT5G38990.1)

AT4G12270	*-1,393*		Copper amine oxidase family protein; FUNCTIONS IN: quinone binding, primary amine oxidase activity, copper ion binding; INVOLVED IN: oxidation reduction, amine metabolic process; LOCATED IN: endomembrane system; CONTAINS InterPro DOMAIN/s: Copper amine oxidase, N-terminal (InterPro:IPR016182), Copper amine oxidase, N2-terminal (InterPro:IPR015800), Copper amine oxidase, N2/N3-terminal (InterPro:IPR015801), Copper amine oxidase, N3-terminal (InterPro:IPR015802), Copper amine oxidase (InterPro:IPR000269), Copper amine oxidase, C-terminal (InterPro:IPR015798); BEST Arabidopsis thaliana protein match is: Copper amine oxidase family protein (TAIR:AT4G12290.1)
AT1G70880	*-1,396*		Polyketide cyclase/dehydrase and lipid transport superfamily protein; FUNCTIONS IN: molecular_function unknown; INVOLVED IN: response to biotic stimulus, defense response; LOCATED IN: cellular_component unknown; EXPRESSED IN: root; CONTAINS InterPro DOMAIN/s: Bet v I allergen (InterPro:IPR000916); BEST Arabidopsis thaliana protein match is: MLP-like protein 28 (TAIR:AT1G70830.5)
AT4G30420	*-1,396*	USUALLY MULTIPLE ACIDS MOVE IN AND OUT TRANSPORTERS 34 (UMAMIT34)	nodulin MtN21-like transporter family protein
AT5G14070	*-1,398*	(ROXY2)	Encodes glutaredoxin ROXY2. ROXY2, together with ROXY1 (AT3G02000), controls anther development. roxy1 roxy2 double mutants are sterile and do not produce pollen.
AT4G36740	*-1,4*	HOMEOBOX PROTEIN 40 (HB40)	Encodes a homeodomain leucine zipper class I (HD-Zip I) protein.
AT3G51360	*-1,401*		Eukaryotic aspartyl protease family protein; FUNCTIONS IN: aspartic-type endopeptidase activity; INVOLVED IN: proteolysis; LOCATED IN: endomembrane system; EXPRESSED IN: root; CONTAINS InterPro DOMAIN/s: Peptidase aspartic (InterPro:IPR021109), Peptidase aspartic, catalytic (InterPro:IPR009007), Peptidase A1 (InterPro:IPR001461), Peptidase aspartic, active site (InterPro:IPR001969); BEST Arabidopsis thaliana protein match is: Eukaryotic aspartyl protease family protein (TAIR:AT2G17760.1)
AT4G15380	*-1,401*	CYTOCHROME P450, FAMILY 705, SUBFAMILY A, POLYPEPTIDE 4 (CYP705A4)	member of CYP705A
AT2G39370	*-1,402*	MEMBRANE-ASSOCIATED KINASE REGULATOR 4 (MAKR4)	Encodes a member of the MAKR (MEMBRANE-ASSOCIATED KINASE REGULATOR) gene family. MAKRs have putative kinase interacting motifs and membrane localization signals. Known members include: AT5G26230 (MAKR1), AT1G64080 (MAKR2), AT2G37380 (MAKR3), AT2G39370 (MAKR4), AT5G52870 (MAKR5) and AT5G52900 (MAKR6).
AT1G12110	*-1,407*	NRT1/ PTR FAMILY 6.3 (NPF6.3)	Encodes NRT1.1 (CHL1), a dual-affinity nitrate transporter. The protein is expressed in guard cells and function in stomatal opening. Mutants have less transpiration and are more tolerant to drought. Expressed in lateral roots. Involved in nitrate signaling which enables the plant root system to detect and exploit nitrate-rich soil patches. Comparing to the wild type, the mutant displays a strongly decreased lateral root proliferation phenotype in nitrate rich patches on growth medium.
AT1G21050	*-1,407*		Protein of unknown function, DUF617; CONTAINS InterPro DOMAIN/s: Protein of unknown function DUF617, plant (InterPro:IPR006460); BEST Arabidopsis thaliana protein match is: Protein of unknown function, DUF617 (TAIR:AT1G76610.1)
AT5G60890	*-1,409*	MYB DOMAIN PROTEIN 34 (MYB34)	Myb-like transcription factor that modulates expression of ASA1, a key point of control in the tryptophan pathway; mutant has deregulated expression of ASA1 in dominant allele. Loss of function allele suggests ATR1 also functions at a control point for regulating indole glucosinolate homeostasis.
AT1G09155	*-1,41*	PHLOEM PROTEIN 2-B15 (PP2-B15)	phloem protein 2-B15 (PP2-B15); FUNCTIONS IN: carbohydrate binding; LOCATED IN: endomembrane system; EXPRESSED IN: 6 plant structures; EXPRESSED DURING: 4 anthesis, C globular stage, petal differentiation and expansion stage; BEST Arabidopsis thaliana protein match is: phloem protein 2-B13 (TAIR:AT1G56240.1)
AT1G18140	*-1,41*	LACCASE 1 (LAC1)	putative laccase, a member of laccase family of genes (with 17 members in Arabidopsis).
AT5G14120	*-1,412*		Major facilitator superfamily protein; LOCATED IN: plasma membrane, vacuole; EXPRESSED IN: 26 plant structures; EXPRESSED DURING: 15 growth stages; CONTAINS InterPro DOMAIN/s: Nodulin-like (InterPro:IPR010658), Major facilitator superfamily, general substrate transporter (InterPro:IPR016196); BEST Arabidopsis thaliana protein match is: Major facilitator superfamily protein (TAIR:AT3G01930.2)

AT1G48600	*-1,414*	PHOSPHOETHANOLAMINE N-METHYLTRANSFERASE (PMEAMT)	Encodes a phosphoethanolamine N-methyltransferase that catalyses the last two methylation steps of the three sequential methylations of phosphoethanolamine (PEA) that are required for the synthesis of phosphocholine (PCho) in plants.
AT2G14960	*-1,415*	(GH3.1)	encodes a protein similar to IAA-amido synthases. Lines carrying an insertion in this gene are hypersensitive to auxin.
AT4G23130	*-1,417*	CYSTEINE-RICH RLK (RECEPTOR-LIKE PROTEIN	Encodes a receptor-like protein kinase. Naming convention from Chen et al 2003 (PMID 14756307)
AT1G02640	*-1,419*	BETA-XYLOSIDASE 2 (BXL2)	encodes a protein similar to a beta-xylosidase located in the extracellular matrix. This is a member of glycosyl hydrolase family 3 and has six other closely related members.
AT2G19060	*-1,421*		SGNH hydrolase-type esterase superfamily protein; FUNCTIONS IN: lipase activity, hydrolase activity, acting on ester bonds; INVOLVED IN: lipid metabolic process; LOCATED IN: endomembrane system; EXPRESSED IN: root; CONTAINS InterPro DOMAIN/s: Lipase, GDSL (InterPro:IPR001087), Esterase, SGNH hydrolase-type (InterPro:IPR013830); BEST Arabidopsis thaliana protein match is: GDSL-like Lipase/Acylhydrolase superfamily protein (TAIR:AT2G19050.1)
AT5G18170	*-1,422*	GLUTAMATE DEHYDROGENASE 1 (GDH1)	Encodes the 43 kDa alpha-subunit of the glutamate dehydrogenase with a putative mitochondrial transit polypeptide and NAD(H)- and alpha-ketoglutarate-binding domains. Mitochondrial localization confirmed by subcellular fractionation. Combines in several ratios with GDH2 protein (GDH-beta) to form seven isoenzymes. Catalyzes the cleavage of glycine residues. May be involved in ammonia assimilation under conditions of inorganic nitrogen excess. The enzyme is almost exclusively found in the mitochondria of stem and leaf companion cells.
AT2G22200	*-1,423*		encodes a member of the DREB subfamily A-6 of ERF/AP2 transcription factor family. The protein contains one AP2 domain. There are 8 members in this subfamily including RAP2.4.
AT3G61430	*-1,423*	PLASMA MEMBRANE INTRINSIC PROTEIN 1A (PIP1A)	a member of the plasma membrane intrinsic protein subfamily PIP1. localizes to the plasma membrane and exhibits water transport activity in Xenopus oocyte. expressed ubiquitously and protein level decreases slightly during leaf development. The mRNA is cell-to-cell mobile.
AT5G03570	*-1,425*	IRON REGULATED 2 (IREG2)	Encodes FPN2, a tonoplast localized nickel transport protein. FPN2 is one of the Arabidopsis orthologs (AT2G38460/IREG1/FPN1 and AT5G03570/IREG2/FPN2) the iron efflux transporter ferroportin (FPN) identified in animals.
AT5G24410	*-1,425*	6-PHOSPHOGLUCONOLACTONASE 4 (PGL4)	Encodes a cytosolic 6-phosphogluconolactonase (PGL) thought to be involved in the oxidative pentose-phosphate pathway (OPPP).
AT5G50120	*-1,428*		Transducin/WD40 repeat-like superfamily protein; CONTAINS InterPro DOMAIN/s: WD40 repeat 2 (InterPro:IPR019782), WD40 repeat, conserved site (InterPro:IPR019775), WD40 repeat (InterPro:IPR001680), G-protein beta WD-40 repeat, region (InterPro:IPR020472), WD40 repeat-like-containing domain (InterPro:IPR011046), WD40-repeat-containing domain (InterPro:IPR017986), WD40/YVTN repeat-like-containing domain (InterPro:IPR015943), WD40 repeat, subgroup (InterPro:IPR019781); BEST Arabidopsis thaliana protein match is: Transducin/WD40 repeat-like superfamily protein (TAIR:AT1G24130.1)
AT4G31910	*-1,431*	BR-RELATED ACYLTRANSFERASE1 (BAT1)	Encodes an acyltransferase that can modify brassinosteroids (BRs) by acylation and may modulate endogenous BR levels.
AT1G24620	*-1,436*	CALMODULIN LIKE 25 (CML25)	Encodes a EF-hand calcium-binding protein family member. Mutants exhibit longer root hairs under phosphate-deficient conditions.
AT1G75820	*-1,437*	CLAVATA 1 (CLV1)	Putative receptor kinase with an extracellular leucine-rich domain. Controls shoot and floral meristem size, and contributes to establish and maintain floral meristem identity. Negatively regulated by KAPP (kinase-associated protein phosphatase). CLV3 peptide binds directly CLV1 ectodomain.
AT1G64900	*-1,438*	CYTOCHROME P450, FAMILY 89, SUBFAMILY A, POLYPEPTIDE 2 (CYP89A2)	Encodes cytochrome P450 (CYP89A2). The mRNA is cell-to-cell mobile.
AT4G01630	*-1,438*	EXPANSIN A17 (EXPA17)	member of Alpha-Expansin Gene Family. Naming convention from the Expansin Working Group (Kende et al, 2004. Plant Mol Bio)

AT2G25680	*-1,439*	MOLYBDATE TRANSPORTER 1 (MOT1)	Encodes a high-affinity molybdate transporter. Mutant has reduced concentrations of molybdate in roots and shoots, and reduced shoot and root length when growing on Mo-limited medium.
AT4G35900	*-1,442*	(FD)	bZIP protein required for positive regulation of flowering. Mutants are late flowering. FD interacts with FT to promote flowering.Expressed in the shoot apex in floral anlagen, then declines in floral primordia.
AT3G55150	*-1,45*	EXOCYST SUBUNIT EXO70 FAMILY PROTEIN H1 (EXO70H1)	A member of EXO70 gene family, putative exocyst subunits, conserved in land plants. Arabidopsis thaliana contains 23 putative EXO70 genes, which can be classified into eight clusters on the phylogenetic tree.
AT5G42590	*-1,451*	CYTOCHROME P450, FAMILY 71, SUBFAMILY A, POLYPEPTIDE 16 (CYP71A16)	putative cytochrome P450
AT2G38600	*-1,454*		HAD superfamily, subfamily IIIB acid phosphatase ; FUNCTIONS IN: acid phosphatase activity; INVOLVED IN: biological_process unknown; LOCATED IN: endomembrane system; EXPRESSED IN: 15 plant structures; EXPRESSED DURING: 6 growth stages; CONTAINS InterPro DOMAIN/s: Acid phosphatase (Class B) (InterPro:IPR005519), Vegetative storage protein/acid phosphatase (InterPro:IPR014403), Acid phosphatase, plant (InterPro:IPR010028); BEST Arabidopsis thaliana protein match is: HAD superfamily, subfamily IIIB acid phosphatase (TAIR:AT4G29260.1)
AT1G12740	*-1,456*	CYTOCHROME P450, FAMILY 87, SUBFAMILY A, POLYPEPTIDE 2 (CYP87A2)	encodes a protein with cytochrome P450 domain
AT1G64400	*-1,464*	LONG-CHAIN ACYL-COA SYNTHETASE 3 (LACS3)	long-chain acyl-CoA synthetase 3 (LACS3); FUNCTIONS IN: catalytic activity; INVOLVED IN: fatty acid biosynthetic process; EXPRESSED IN: 22 plant structures; EXPRESSED DURING: 13 growth stages; CONTAINS InterPro DOMAIN/s: AMP-binding, conserved site (InterPro:IPR020845), AMP-dependent synthetase/ligase (InterPro:IPR000873); BEST Arabidopsis thaliana protein match is: AMP-dependent synthetase and ligase family protein (TAIR:AT4G23850.1)
AT3G02000	*-1,47*	(ROXY1)	Roxy1 encodes a glutaredoxin belonging to a subgroup specific to higher plants. It is required for proper petal initiation and organogenesis. It is likely to function in the temporal and spatial expression regulation of AGAMOUS in the first and second whorl. It's function is dependent on the Cysteine 49 residue and its nuclear localization. ROXY1 interacts in vitro and in vivo with members of the TGA family of transcription factors (e.g. TGA2, TGA3, TGA7 and PAN).
AT5G17820	*-1,472*	PEROXIDASE 57 (PER57)	Peroxidase superfamily protein; FUNCTIONS IN: peroxidase activity, heme binding; INVOLVED IN: oxidation reduction, response to oxidative stress; LOCATED IN: cell wall, membrane, plant-type cell wall; EXPRESSED IN: root, leaf; CONTAINS InterPro DOMAIN/s: Haem peroxidase (InterPro:IPR010255), Plant peroxidase (InterPro:IPR000823), Peroxidases heam-ligand binding site (InterPro:IPR019793), Peroxidase, active site (InterPro:IPR019794), Haem peroxidase, plant/fungal/bacterial (InterPro:IPR002016); BEST Arabidopsis thaliana protein match is: Peroxidase superfamily protein (TAIR:AT3G03670.1)
AT5G23010	*-1,473*	METHYLTHIOALKYLMALATE SYNTHASE 1 (MAM1)	Encodes a methylthioalkylmalate synthase, catalyzes the condensation reactions of the first two rounds of methionine chain elongation in the biosynthesis of methionine-derived glucosinolates. The mRNA is cell-to-cell mobile.
AT5G48430	*-1,475*		Eukaryotic aspartyl protease family protein; FUNCTIONS IN: aspartic-type endopeptidase activity; INVOLVED IN: proteolysis; LOCATED IN: endomembrane system; EXPRESSED IN: shoot apex, hypocotyl, root, leaf; CONTAINS InterPro DOMAIN/s: Peptidase aspartic (InterPro:IPR021109), Peptidase aspartic, catalytic (InterPro:IPR009007), Peptidase A1 (InterPro:IPR001461); BEST Arabidopsis thaliana protein match is: Eukaryotic aspartyl protease family protein (TAIR:AT1G03220.1)
AT3G48360	*-1,478*	BTB AND TAZ DOMAIN PROTEIN 2 (bt2)	encodes a protein (BT2) that is an essential component of the TAC1-mediated telomerase activation pathway. Acts redundantly with BT3 and BT1 during female gametophyte development and with BT3 during male gametophyte development. BT2 also mediates multiple responses to nutrients, stresses, and hormones.
AT1G10380	*-1,481*		Putative membrane lipoprotein; FUNCTIONS IN: molecular_function unknown; INVOLVED IN: biological_process unknown; LOCATED IN: endomembrane system; EXPRESSED IN: 19 plant structures; EXPRESSED DURING: 10 growth stages; BEST Arabidopsis thaliana protein match is: unknown protein (TAIR:AT3G17350.1)
AT4G37700	*-1,482*		unknown protein
AT1G49860	*-1,483*	GLUTATHIONE S-TRANSFERASE (CLASS PHI) 14	Encodes glutathione transferase belonging to the phi class of GSTs. Naming convention according to Wagner et al. (2002). The mRNA is cell-to-cell mobile.

AT5G18560	*-1,485*	(PUCHI)	Encodes PUCHI, a member of the ERF (ethylene response factor) subfamily B-1 of ERF/AP2 transcription factor family. The protein contains one AP2 domain. There are 15 members in this subfamily including ATERF-3, ATERF-4, ATERF-7, and leafy petiole. PUCHI is required for morphogenesis in the early lateral root primordium of Arabidopsis. Expressed in early floral meristem (stage 1 to 2). Required for early floral meristem growth and for bract suppression. Triple mutant with bop1 and bop2 displays a strong defect in the determination of floral meristem identity with reduced LFY expression and the lack of AP1 expression.
AT3G10912	*-1,486*	CONSERVED PEPTIDE UPSTREAM OPEN READING FRAME 63 (CPuORF63)	Upstream open reading frames (uORFs) are small open reading frames found in the 5' UTR of a mature mRNA, and can potentially mediate translational regulation of the largest, or major, ORF (mORF). CPuORF63 represents a conserved upstream opening reading frame relative to major ORF AT3G10910.1
AT5G57800	*-1,487*	ECERIFERUM 3 (CER3)	encodes a transmembrane protein with similarity to the sterol desaturase family at the N-terminus and to the short-chain dehydrogenase/reductase family at the C-terminus. Mutant analyses indicate this protein is involved in cuticle membrane and wax biosynthesis. The mRNA is cell-to-cell mobile.
AT4G12350	*-1,488*	MYB DOMAIN PROTEIN 42 (MYB42)	Encodes a putative transcription factor (MYB42).
AT1G67870	*-1,49*		glycine-rich protein
AT5G28020	*-1,49*	CYSTEINE SYNTHASE D2 (CYSD2)	Encodes cysteine synthase CysD2.
AT4G14130	*-1,492*	XYLOGLUCAN ENDOTRANSGLUCOSYLASE/HYDROLASE 15 (XTH15)	xyloglucan endotransglycosylase-related protein (XTR7)
AT4G25420	*-1,493*	(GA20OX1)	Encodes gibberellin 20-oxidase that is involved in the later steps of the gibberellin biosynthetic pathway. Regulated by a circadian clock. Weak expression response to far red light.
AT2G21540	*-1,495*	SEC14-LIKE 3 (SFH3)	SEC14-like 3 (SFH3); FUNCTIONS IN: phosphatidylinositol transporter activity; INVOLVED IN: flower development, transport; LOCATED IN: intracellular; EXPRESSED IN: 25 plant structures; EXPRESSED DURING: 17 growth stages; CONTAINS InterPro DOMAIN/s: Cellular retinaldehyde-binding/triple function, C-terminal (InterPro:IPR001251), Cellular retinaldehyde-binding/triple function, N-terminal (InterPro:IPR008273), Cellular retinaldehyde binding/alpha-tocopherol transport (InterPro:IPR001071), Phosphatidylinositol transfer protein-like, N-terminal (InterPro:IPR011074); BEST Arabidopsis thaliana protein match is: Sec14p-like phosphatidylinositol transfer family protein (TAIR:AT4G39180.2)
AT5G41300	*-1,496*		Receptor-like protein kinase-related family protein; CONTAINS InterPro DOMAIN/s: Protein of unknown function DUF26 (InterPro:IPR002902); BEST Arabidopsis thaliana protein match is: Receptor-like protein kinase-related family protein (TAIR:AT5G41290.1)
AT5G66700	*-1,497*	HOMEOBOX 53 (HB53)	Encodes a homeodomain protein. Member of HD-ZIP 1 family, most closely related to HB5. AtHB53 is auxin-inducible and its induction is inhibited by cytokinin, especially in roots therefore may be involved in root development.
AT1G31710	*-1,498*		Copper amine oxidase family protein; FUNCTIONS IN: primary amine oxidase activity, quinone binding, copper ion binding; INVOLVED IN: oxidation reduction, amine metabolic process; LOCATED IN: endomembrane system; EXPRESSED IN: 13 plant structures; EXPRESSED DURING: 8 growth stages; CONTAINS InterPro DOMAIN/s: Copper amine oxidase, N-terminal (InterPro:IPR016182), Copper amine oxidase, N2-terminal (InterPro:IPR015800), Copper amine oxidase, N2/N3-terminal (InterPro:IPR015801), Copper amine oxidase, N3-terminal (InterPro:IPR015802), Copper amine oxidase (InterPro:IPR000269), Copper amine oxidase, C-terminal (InterPro:IPR015798); BEST Arabidopsis thaliana protein match is: Copper amine oxidase family protein (TAIR:AT1G31690.1)
AT4G23870	*-1,498*		unknown protein; BEST Arabidopsis thaliana protein match is: unknown protein (TAIR:AT4G11020.1)

AT5G42250	*-1,501*		Zinc-binding alcohol dehydrogenase family protein; FUNCTIONS IN: oxidoreductase activity, zinc ion binding; INVOLVED IN: oxidation reduction; EXPRESSED IN: 11 plant structures; EXPRESSED DURING: 6 growth stages; CONTAINS InterPro DOMAIN/s: GroES-like (InterPro:IPR011032), Alcohol dehydrogenase GroES-like (InterPro:IPR013154), Alcohol dehydrogenase, zinc-containing, conserved site (InterPro:IPR002328), Alcohol dehydrogenase, C-terminal (InterPro:IPR013149), Alcohol dehydrogenase superfamily, zinc-containing (InterPro:IPR002085); BEST Arabidopsis thaliana protein match is: GroES-like zinc-binding dehydrogenase family protein (TAIR:AT4G22110.2)
AT2G29340	*-1,502*		NAD-dependent epimerase/dehydratase family protein; FUNCTIONS IN: oxidoreductase activity, binding, catalytic activity; INVOLVED IN: oxidation reduction, metabolic process; EXPRESSED IN: 22 plant structures; EXPRESSED DURING: 13 growth stages; CONTAINS InterPro DOMAIN/s: NAD(P)-binding domain (InterPro:IPR016040), Glucose/ribitol dehydrogenase (InterPro:IPR002347), Short-chain dehydrogenase/reductase SDR (InterPro:IPR002198); BEST Arabidopsis thaliana protein match is: NAD(P)-binding Rossmann-fold superfamily protein (TAIR:AT2G29320.1)
AT5G01250	*-1,509*		alpha 1,4-glycosyltransferase family protein; FUNCTIONS IN: transferase activity, transferring glycosyl groups, transferase activity; INVOLVED IN: biological_process unknown; LOCATED IN: Golgi stack; CONTAINS InterPro DOMAIN/s: Alpha 1,4-glycosyltransferase conserved region (InterPro:IPR007652), Glycosyltransferase, DXD sugar-binding region (InterPro:IPR007577); BEST Arabidopsis thaliana protein match is: alpha 1,4-glycosyltransferase family protein (TAIR:AT3G09020.1)
AT1G70890	*-1,511*	MLP-LIKE PROTEIN 43 (MLP43)	MLP-like protein 43 (MLP43); FUNCTIONS IN: molecular_function unknown; INVOLVED IN: response to biotic stimulus, defense response; LOCATED IN: chloroplast; EXPRESSED IN: 17 plant structures; EXPRESSED DURING: 10 growth stages; CONTAINS InterPro DOMAIN/s: Bet v I allergen (InterPro:IPR000916); BEST Arabidopsis thaliana protein match is: MLP-like protein 28 (TAIR:AT1G70830.5)
AT2G22000	*-1,511*	ELICITOR PEPTIDE 6 PRECURSOR (PROPEP6)	elicitor peptide 6 precursor (PROPEP6)
AT5G15830	*-1,511*	BASIC LEUCINE-ZIPPER 3 (bZIP3)	basic leucine-zipper 3 (bZIP3); FUNCTIONS IN: DNA binding, sequence-specific DNA binding transcription factor activity; INVOLVED IN: regulation of transcription, DNA-dependent; EXPRESSED IN: 9 plant structures; EXPRESSED DURING: 6 growth stages; CONTAINS InterPro DOMAIN/s: Basic-leucine zipper (bZIP) transcription factor (InterPro:IPR004827), bZIP transcription factor, bZIP-1 (InterPro:IPR011616); BEST Arabidopsis thaliana protein match is: basic leucine-zipper 42 (TAIR:AT3G30530.1)
AT5G03150	*-1,516*	JACKDAW (JKD)	JKD is a nuclear-localized putative transcription factor with three zinc finger domains. jkd mutants show a number of root patterning defects including ectopic periclinal divisions in the cortex, increased cell numbers in the cortical and epidermal layers, a disrupted QC marker expression pattern, and disorganized QC and columella cells. jkd mutants also have a reduced number of meristematic cells in their roots. JKD can interact with the SCR and SHR proteins implicated in root patterning, as well as another zinc finger transcription factor, MAGPIE. All of these interactions require the first zinc finger in JKD according to a Y2H assay. There are also transcriptional interactions among these proteins. The initiation of JKD transcription does not appear to depend on SCR and SHR, but later expression in the post-embryonic QC cells and ground tissue initials is reduced in scr and shr mutants. JKD also appears to be required for SCR transcription beginning in the embryo. There is also some evidence that JKD plays a role in promoting the movement of SHR into the nucleus, particularly in QC cells, but this may be indirect. The mRNA is cell-to-cell mobile.
AT1G60110	*-1,519*		Mannose-binding lectin superfamily protein; EXPRESSED IN: cultured cell; CONTAINS InterPro DOMAIN/s: Mannose-binding lectin (InterPro:IPR001229); BEST Arabidopsis thaliana protein match is: Mannose-binding lectin superfamily protein (TAIR:AT1G60130.1)
AT3G24300	*-1,519*	AMMONIUM TRANSPORTER 1;3 (AMT1;3)	Encodes a plasma membrane localized ammonium transporter.
AT1G79130	*-1,521*	SMALL AUXIN UPREGULATED 40 (SAUR40)	SAUR-like auxin-responsive protein family ; CONTAINS InterPro DOMAIN/s: Auxin responsive SAUR protein (InterPro:IPR003676); BEST Arabidopsis thaliana protein match is: SAUR-like auxin-responsive protein family (TAIR:AT1G16510.1)

AT5G19530	*-1,523*	ACAULIS 5 (ACL5)	Encodes a spermine synthase. Required for internode elongation and vascular development, specifically in the mechanism that defines the boundaries between veins and nonvein regions. This mechanism may be mediated by polar auxin transport. Though ACL5 has been shown to function as a spermine synthase in E. coli, an ACL5 knockout has no effect on the endogenous levels of free and conjugated polyamines in Arabidopsis, suggesting that ACL5 may have a very specific or altogether different in vivo function.
AT5G25610	*-1,526*	RESPONSIVE TO DESICCATION 22 (RD22)	responsive to dehydration 22 (RD22) mediated by ABA
AT1G63410	*-1,53*		Protein of unknown function (DUF567); CONTAINS InterPro DOMAIN/s: Protein of unknown function DUF567 (InterPro:IPR007612); BEST Arabidopsis thaliana protein match is: Protein of unknown function (DUF567) (TAIR:AT3G14260.1)
AT4G23400	*-1,535*	PLASMA MEMBRANE INTRINSIC PROTEIN 1;5 (PIP1;5)	plasma membrane intrinsic protein 1;5 (PIP1;5); FUNCTIONS IN: water channel activity; INVOLVED IN: response to salt stress, transport; LOCATED IN: plasma membrane, membrane; EXPRESSED IN: 22 plant structures; EXPRESSED DURING: 14 growth stages; CONTAINS InterPro DOMAIN/s: Major intrinsic protein, conserved site (InterPro:IPR022357), Aquaporin (InterPro:IPR012269), Major intrinsic protein (InterPro:IPR000425); BEST Arabidopsis thaliana protein match is: plasma membrane intrinsic protein 1;4 (TAIR:AT4G00430.1)
AT5G43150	*-1,537*		unknown protein; FUNCTIONS IN: molecular_function unknown; INVOLVED IN: biological_process unknown; LOCATED IN: mitochondrion; EXPRESSED IN: 22 plant structures; EXPRESSED DURING: 13 growth stages
AT5G60760	*-1,537*		P-loop containing nucleoside triphosphate hydrolases superfamily protein; BEST Arabidopsis thaliana protein match is: P-loop containing nucleoside triphosphate hydrolases superfamily protein (TAIR:AT3G45090.1)
AT1G64480	*-1,538*	CALCINEURIN B-LIKE PROTEIN 8 (CBL8)	calcineurin B-like protein 8, member of plant-specific family of calcium sensor proteins containing 3 EF-hand motifs
AT5G67450	*-1,54*	ZINC-FINGER PROTEIN 1 (ZF1)	Encodes zinc-finger protein. mRNA levels are elevated in response to low temperature, cold temperatures and high salt. The protein is localized to the nucleus and acts as a transcriptional repressor.
AT3G26290	*-1,543*	CYTOCHROME P450, FAMILY 71, SUBFAMILY B, POLYPEPTIDE 26 (CYP71B26)	putative cytochrome P450
AT2G29320	*-1,55*		NAD(P)-binding Rossmann-fold superfamily protein; FUNCTIONS IN: oxidoreductase activity, binding, catalytic activity; INVOLVED IN: oxidation reduction, metabolic process; LOCATED IN: cellular_component unknown; EXPRESSED IN: 21 plant structures; EXPRESSED DURING: 13 growth stages; CONTAINS InterPro DOMAIN/s: NAD(P)-binding domain (InterPro:IPR016040), Glucose/ribitol dehydrogenase (InterPro:IPR002347), Short-chain dehydrogenase/reductase SDR (InterPro:IPR002198); BEST Arabidopsis thaliana protein match is: NAD(P)-binding Rossmann-fold superfamily protein (TAIR:AT2G29300.1)
AT3G63200	*-1,551*	PATATIN-LIKE PROTEIN 9 (PLP9)	PATATIN-like protein 9 (PLP9); FUNCTIONS IN: nutrient reservoir activity; INVOLVED IN: metabolic process, lipid metabolic process; LOCATED IN: cellular_component unknown; EXPRESSED IN: 18 plant structures; EXPRESSED DURING: 13 growth stages; CONTAINS InterPro DOMAIN/s: Acyl transferase/acyl hydrolase/lysophospholipase (InterPro:IPR016035), Patatin (InterPro:IPR002641); BEST Arabidopsis thaliana protein match is: PATATIN-like protein 6 (TAIR:AT2G39220.1)
AT1G80380	*-1,552*		encodes a glycerate kinase which catalyzes the last step of photorespiration C2 cycle.
AT5G23020	*-1,552*	2-ISOPROPYLMALATE SYNTHASE 2 (IMS2)	methylthioalkymalate synthase-like. Also known as 2-isopropylmalate synthase (IMS2). encodes a methylthioalkylmalate synthase involved in the biosynthesis of aliphatic glucosinolates which accepts all the omega-methylthio-2-oxoalkanoic acids needed to form the known C3 to C8 glucosinolates in Arabidopsis. The mRNA is cell-to-cell mobile.
AT1G43790	*-1,555*	TRACHEARY ELEMENT DIFFERENTIATION-RELATED 6 (TED6)	tracheary element differentiation-related 6 (TED6); BEST Arabidopsis thaliana protein match is: tracheary element differentiation-related 7 (TAIR:AT5G48920.1)

AT4G01380	*-1,561*		plastocyanin-like domain-containing protein; FUNCTIONS IN: electron carrier activity, copper ion binding; INVOLVED IN: biological_process unknown; LOCATED IN: cellular_component unknown; CONTAINS InterPro DOMAIN/s: Plastocyanin-like (InterPro:IPR003245), Cupredoxin (InterPro:IPR008972); BEST Arabidopsis thaliana protein match is: copper ion binding;electron carriers (TAIR:AT1G45063.2)
AT1G19900	*-1,562*		glyoxal oxidase-related protein; FUNCTIONS IN: molecular_function unknown; INVOLVED IN: biological_process unknown; LOCATED IN: endomembrane system; EXPRESSED IN: root; CONTAINS InterPro DOMAIN/s: Galactose oxidase/kelch, beta-propeller (InterPro:IPR011043), Galactose oxidase, beta-propeller (InterPro:IPR015916), Immunoglobulin E-set (InterPro:IPR014756), Glyoxal oxidase, N-terminal (InterPro:IPR009880), Domain of unknown function DUF1929 (InterPro:IPR015202); BEST Arabidopsis thaliana protein match is: glyoxal oxidase-related protein (TAIR:AT1G75620.1)
AT5G50560	*-1,572*		Protein of unknown function (DUF 3339); FUNCTIONS IN: molecular_function unknown; INVOLVED IN: biological_process unknown; LOCATED IN: endomembrane system; CONTAINS InterPro DOMAIN/s: Protein of unknown function DUF3339 (InterPro:IPR021775); BEST Arabidopsis thaliana protein match is: Protein of unknown function (DUF 3339) (TAIR:AT5G50660.1)
AT1G65570	*-1,573*	ROOT CAP POLYGALACTURONASE (RCPG)	Encodes a glycosyl hydrolase 28 (GH28) family polygalacturonase (PG) protein. Involved in root cap development.
AT4G20240	*-1,578*	CYTOCHROME P450, FAMILY 71, SUBFAMILY A, POLYPEPTIDE 27 (CYP71A27)	putative cytochrome P450
AT1G75640	*-1,581*	MUSTACHES (MUS)	Encodes a Leucine-Rich Repeat Receptor-Like Kinase MUSTACHES (MUS). Regulates stomatal bilateral symmetry.
AT2G02020	*-1,586*	NRT1/ PTR FAMILY 8.4 (NPF8.4)	Major facilitator superfamily protein; FUNCTIONS IN: transporter activity; INVOLVED IN: oligopeptide transport; LOCATED IN: membrane; EXPRESSED IN: 6 plant structures; EXPRESSED DURING: LP.06 six leaves visible; CONTAINS InterPro DOMAIN/s: PTR2 family proton/oligopeptide symporter, conserved site (InterPro:IPR018456), Oligopeptide transporter (InterPro:IPR000109), Major facilitator superfamily, general substrate transporter (InterPro:IPR016196); BEST Arabidopsis thaliana protein match is: peptide transporter 2 (TAIR:AT2G02040.1)
AT1G70860	*-1,587*		Polyketide cyclase/dehydrase and lipid transport superfamily protein; FUNCTIONS IN: molecular_function unknown; INVOLVED IN: response to biotic stimulus, defense response; LOCATED IN: cellular_component unknown; CONTAINS InterPro DOMAIN/s: Bet v I allergen (InterPro:IPR000916); BEST Arabidopsis thaliana protein match is: Polyketide cyclase/dehydrase and lipid transport superfamily protein (TAIR:AT1G70880.1).
AT5G48900	*-1,593*		Pectin lyase-like superfamily protein; FUNCTIONS IN: lyase activity, pectate lyase activity; LOCATED IN: endomembrane system; EXPRESSED IN: 22 plant structures; EXPRESSED DURING: 14 growth stages; CONTAINS InterPro DOMAIN/s: Pectin lyase fold/virulence factor (InterPro:IPR011050), AmbAllergen (InterPro:IPR018082), Pectate lyase/Amb allergen (InterPro:IPR002022), Pectin lyase fold (InterPro:IPR012334), Parallel beta-helix repeat (InterPro:IPR006626); BEST Arabidopsis thaliana protein match is: Pectin lyase-like superfamily protein (TAIR:AT3G07010.1)
AT5G60660	*-1,601*	PLASMA MEMBRANE INTRINSIC PROTEIN 2;4 (PIP2;4)	A member of the plasma membrane intrinsic protein subfamily PIP2.When expressed in yeast cells can conduct hydrogen peroxide into those cells. Mutants exhibit longer root hairs.
AT3G29430	*-1,606*		Terpenoid synthases superfamily protein; INVOLVED IN: isoprenoid biosynthetic process; EXPRESSED IN: root; CONTAINS InterPro DOMAIN/s: Polyprenyl synthetase-related (InterPro:IPR017446), Terpenoid synthase (InterPro:IPR008949), Polyprenyl synthetase (InterPro:IPR000092); BEST Arabidopsis thaliana protein match is: Terpenoid synthases superfamily protein (TAIR:AT3G32040.1)
AT2G29060	*-1,609*		GRAS family transcription factor; CONTAINS InterPro DOMAIN/s: Transcription factor GRAS (InterPro:IPR005202); BEST Arabidopsis thaliana protein match is: SCARECROW-like 14 (TAIR:AT1G07530.1)
AT4G01820	*-1,609*	ATP-BINDING CASSETTE B3 (ABCB3)	member of MDR subfamily
AT5G17430	*-1,611*	BABY BOOM (BBM)	Encodes an AP2-domain containing protein similar to ANT. Expressed in embryos and lateral root primordium.

AT1G08090	*-1,613*	NITRATE TRANSPORTER 2:1 (NRT2:1)	High-affinity nitrate transporter. Up-regulated by nitrate. Functions as a repressor of lateral root initiation independently of nitrate uptake.
AT2G34000	*-1,613*		RING/U-box superfamily protein; FUNCTIONS IN: zinc ion binding; LOCATED IN: endomembrane system; CONTAINS InterPro DOMAIN/s: Zinc finger, RING-type (InterPro:IPR001841), Zinc finger, C3HC4 RING-type (InterPro:IPR018957); BEST Arabidopsis thaliana protein match is: RING/U-box superfamily protein (TAIR:AT3G05200.1)
AT4G15290	*-1,614*	(ATCSLB05)	Encodes a gene similar to cellulose synthase. Mutants exhibit shorter root hairs under phosphate-deficient conditions.
AT2G16970	*-1,615*	MATERNAL EFFECT EMBRYO ARREST 15 (MEE15)	maternal effect embryo arrest 15 (MEE15); FUNCTIONS IN: tetracycline transporter activity; INVOLVED IN: embryo development ending in seed dormancy; LOCATED IN: integral to membrane; EXPRESSED IN: 22 plant structures; EXPRESSED DURING: 13 growth stages; CONTAINS InterPro DOMAIN/s: Tetracycline resistance protein, TetA (InterPro:IPR001958), Major facilitator superfamily MFS-1 (InterPro:IPR011701), Major facilitator superfamily, general substrate transporter (InterPro:IPR016196); BEST Arabidopsis thaliana protein match is: Major facilitator superfamily protein (TAIR:AT2G16980.2)
AT1G33870	*-1,617*		P-loop containing nucleoside triphosphate hydrolases superfamily protein; FUNCTIONS IN: GTP binding; INVOLVED IN: response to bacterium; LOCATED IN: cellular_component unknown; EXPRESSED IN: stem, root, seed; EXPRESSED DURING: F mature embryo stage; CONTAINS InterPro DOMAIN/s: AIG1 (InterPro:IPR006703); BEST Arabidopsis thaliana protein match is: P-loop containing nucleoside triphosphate hydrolases superfamily protein (TAIR:AT1G33900.1)
AT3G44990	*-1,618*	XYLOGLUCAN ENDOTRANSGLUCOSYLASE/HYDROLASE 31 (XTH31)	Encodes a xyloglucan endotransglycosylase/hydrolase. Protein sequence and phylogenetic analysis indicates that this enzyme resides in Group III-A of the XTH family, with high similarity to Tropaeolum majus (nasturtium) xyloglucanase 1 (TmNXG1).Enzyme kinetic analysis indicates predominant xyloglucan endo-hydrolase activity (EC 3.2.1.151) with only limited potential to act as a xyloglucan endo-transglycosylase (EC 2.4.1.207).
AT3G23430	*-1,622*	PHOSPHATE 1 (PHO1)	Encodes a protein with the mainly hydrophilic N-terminal and the C-terminal containing 6 potential membrane-spanning domains. The mutant is deficient in the transfer of phosphate from root epidermal and cortical cells to the xylem. Its expression is repressed by phosphate (Pi) in shoots, and transiently induced by phosphite (Phi) in roots and shoots.
AT3G03190	*-1,627*	GLUTATHIONE S-TRANSFERASE F11 (GSTF11)	Encodes glutathione transferase belonging to the phi class of GSTs. Naming convention according to Wagner et al. (2002).
AT3G58990	*-1,629*	ISOPROPYLMALATE ISOMERASE 1 (IPMI1)	isopropylmalate isomerase 1 (IPMI1); FUNCTIONS IN: hydro-lyase activity, 3-isopropylmalate dehydratase activity; INVOLVED IN: leucine biosynthetic process, metabolic process; LOCATED IN: plastid; EXPRESSED IN: 19 plant structures; EXPRESSED DURING: 9 growth stages; CONTAINS InterPro DOMAIN/s: 3-isopropylmalate dehydratase, small subunit-like (InterPro:IPR012305), Aconitase A/isopropylmalate dehydratase small subunit, swivel (InterPro:IPR000573), Aconitase/3-isopropylmalate dehydratase, swivel (InterPro:IPR015928), Aconitase-like core (InterPro:IPR015937); BEST Arabidopsis thaliana protein match is: Aconitase/3-isopropylmalate dehydratase protein (TAIR:AT2G43090.1)
AT5G04730	*-1,631*		Ankyrin-repeat containing protein; BEST Arabidopsis thaliana protein match is: Ankyrin repeat family protein (TAIR:AT5G04700.1)
AT5G07030	*-1,632*		Eukaryotic aspartyl protease family protein; FUNCTIONS IN: aspartic-type endopeptidase activity; INVOLVED IN: proteolysis; LOCATED IN: cell wall, plant-type cell wall; EXPRESSED IN: 23 plant structures; EXPRESSED DURING: 13 growth stages; CONTAINS InterPro DOMAIN/s: Peptidase aspartic, catalytic (InterPro:IPR009007), Peptidase aspartic (InterPro:IPR021109), Peptidase A1 (InterPro:IPR001461); BEST Arabidopsis thaliana protein match is: Eukaryotic aspartyl protease family protein (TAIR:AT3G54400.1)
AT5G61290	*-1,634*		Flavin-binding monooxygenase family protein; FUNCTIONS IN: NADP or NADPH binding, monooxygenase activity, FAD binding, flavin-containing monooxygenase activity; INVOLVED IN: oxidation reduction; LOCATED IN: cellular_component unknown; EXPRESSED IN: 11 plant structures; EXPRESSED DURING: 4 anthesis, C globular stage; CONTAINS InterPro DOMAIN/s: Flavin-containing monooxygenase FMO (InterPro:IPR000960), Flavin-containing monooxygenase-like (InterPro:IPR020946); BEST Arabidopsis thaliana protein match is: Flavin-binding monooxygenase family protein (TAIR:AT5G07800.1)
AT3G02493	*-1,636*	ROTUNDIFOLIA LIKE 19 (RTFL19)	ROTUNDIFOLIA like 19 (RTFL19); CONTAINS InterPro DOMAIN/s: DVL (InterPro:IPR012552); BEST Arabidopsis thaliana protein match is: ROTUNDIFOLIA like 18 (TAIR:AT5G16023.1)

AT1G06160	*-1,637*	OCTADECANOID-RESPONSIVE ARABIDOPSIS AP2/ERF 59 (ORA59)	encodes a member of the ERF (ethylene response factor) subfamily B-3 of ERF/AP2 transcription factor family. The protein contains one AP2 domain. There are 18 members in this subfamily including ATERF-1, ATERF-2, AND ATERF-5.
AT3G01190	*-1,637*		Peroxidase superfamily protein; FUNCTIONS IN: peroxidase activity, heme binding; INVOLVED IN: response to oxidative stress, oxidation reduction; LOCATED IN: endomembrane system; EXPRESSED IN: hypocotyl, root; CONTAINS InterPro DOMAIN/s: Haem peroxidase (InterPro:IPR010255), Plant peroxidase (InterPro:IPR000823), Peroxidases heam-ligand binding site (InterPro:IPR019793), Haem peroxidase, plant/fungal/bacterial (InterPro:IPR002016), Peroxidase, active site (InterPro:IPR019794); BEST Arabidopsis thaliana protein match is: Peroxidase superfamily protein (TAIR:AT5G15180.1)
AT5G42500	*-1,637*		Disease resistance-responsive (dirigent-like protein) family protein; FUNCTIONS IN: molecular_function unknown; INVOLVED IN: defense response; LOCATED IN: endomembrane system; CONTAINS InterPro DOMAIN/s: Plant disease resistance response protein (InterPro:IPR004265); BEST Arabidopsis thaliana protein match is: Disease resistance-responsive (dirigent-like protein) family protein (TAIR:AT5G42510.1)
AT2G45360	*-1,65*		Protein of unknown function (DUF1442); CONTAINS InterPro DOMAIN/s: Protein of unknown function DUF1442 (InterPro:IPR009902); BEST Arabidopsis thaliana protein match is: Protein of unknown function (DUF1442) (TAIR:AT3G60780.1)
AT3G04030	*-1,652*	(MYR2)	Homeodomain-like superfamily protein; FUNCTIONS IN: sequence-specific DNA binding transcription factor activity; INVOLVED IN: regulation of transcription; EXPRESSED IN: 9 plant structures; EXPRESSED DURING: 4 anthesis, 4 leaf senescence stage, petal differentiation and expansion stage; CONTAINS InterPro DOMAIN/s: Homeodomain-like (InterPro:IPR009057), Myb, DNA-binding (InterPro:IPR014778), Myb-like DNA-binding domain, SHAQKYF class (InterPro:IPR006447), HTH transcriptional regulator, Myb-type, DNA-binding (InterPro:IPR017930), Homeodomain-related (InterPro:IPR012287); BEST Arabidopsis thaliana protein match is: myb-related protein 1 (TAIR:AT5G18240.4)
AT5G59260	*-1,652*	L-TYPE LECTIN RECEPTOR KINASE II.1 (LECRK-II.1)	Concanavalin A-like lectin protein kinase family protein; FUNCTIONS IN: protein serine/threonine kinase activity, binding, protein kinase activity, kinase activity, ATP binding; INVOLVED IN: protein amino acid phosphorylation; LOCATED IN: endomembrane system; EXPRESSED IN: hypocotyl, root, stamen; EXPRESSED DURING: 4 anthesis; CONTAINS InterPro DOMAIN/s: Legume lectin, beta chain (InterPro:IPR001220), Protein kinase, ATP binding site (InterPro:IPR017441), Serine/threonine-protein kinase-like domain (InterPro:IPR017442), Concanavalin A-like lectin/glucanase, subgroup (InterPro:IPR013320), Protein kinase-like domain (InterPro:IPR011009), Serine/threonine-protein kinase, active site (InterPro:IPR008271), Protein kinase, catalytic domain (InterPro:IPR000719), Concanavalin A-like lectin/glucanase (InterPro:IPR008985); BEST Arabidopsis thaliana protein match is: Concanavalin A-like lectin protein kinase family protein (TAIR:AT5G59270.1)
AT1G05310	*-1,657*		Pectin lyase-like superfamily protein; FUNCTIONS IN: pectinesterase activity; INVOLVED IN: cell wall modification; LOCATED IN: endomembrane system, cell wall, plant-type cell wall; EXPRESSED IN: 12 plant structures; EXPRESSED DURING: 6 growth stages; CONTAINS InterPro DOMAIN/s: Pectinesterase, active site (InterPro:IPR018040), Pectin lyase fold/virulence factor (InterPro:IPR011050), Pectinesterase, catalytic (InterPro:IPR000070); BEST Arabidopsis thaliana protein match is: Pectin lyase-like superfamily protein (TAIR:AT2G36710.1)
AT1G72200	*-1,658*		RING/U-box superfamily protein; FUNCTIONS IN: zinc ion binding; EXPRESSED IN: 13 plant structures; EXPRESSED DURING: LP.04 four leaves visible, 4 anthesis, petal differentiation and expansion stage, LP.08 eight leaves visible; CONTAINS InterPro DOMAIN/s: Zinc finger, RING-type (InterPro:IPR001841), Zinc finger, C3HC4 RING-type (InterPro:IPR018957); BEST Arabidopsis thaliana protein match is: RING/U-box superfamily protein (TAIR:AT1G22500.1)
AT1G80100	*-1,661*	HISTIDINE PHOSPHOTRANSFER PROTEIN 6 (HP6)	AHP6 lacks the conserved histidine residue (Asn83 in AHP6b), which is required for phosphotransfer, present in the other AHPs. AHP6 does not appear to have phosphotransfer activity. Acts as an inhibitor of cytokinin signaling by interacting with the phosphorelay machinery. Expressed in developing protoxylem and associated pericycle cell files. Negative regulator of cytokinin signaling. Expression is down-regulated by cytokinins. There are two alternatively spliced genes for this locus, AHP6a and AHP6b, differing in the length of the first exon. In ahp6-2 seedlings, only the AHP6a transcript is present. Members of the AHP gene family include: AT3G21510 (AHP1), AT3G29350 (AHP2), AT5G39340 (AHP3), AT3G16360 (AHP4), AT1G03430 (AHP5) and AT1G80100 (AHP6).

AT2G42850	*-1,662*	CYTOCHROME P450, FAMILY 718 (CYP718)	member of CYP718
AT4G28250	*-1,662*	EXPANSIN B3 (EXPB3)	putative beta-expansin/allergen protein. Naming convention from the Expansin Working Group (Kende et al, 2004. Plant Mol Bio). Involved in the formation of nematode-induced syncytia in roots of Arabidopsis thaliana.
AT4G40010	*-1,662*	SNF1-RELATED PROTEIN KINASE 2.7 (SNRK2.7)	encodes a member of SNF1-related protein kinases (SnRK2) whose activity is activated by ionic (salt) and non-ionic (mannitol) osmotic stress.
AT2G29130	*-1,663*	LACCASE 2 (LAC2)	putative laccase, knockout mutant had reduced root elongation under PEG-induced dehydration
AT4G00670	*-1,663*		Remorin family protein; CONTAINS InterPro DOMAIN/s: Remorin, C-terminal (InterPro:IPR005516); BEST Arabidopsis thaliana protein match is: Remorin family protein (TAIR:AT3G61260.1)
AT4G15100	*-1,663*	SERINE CARBOXYPEPTIDASE-LIKE 30 (scpl30)	serine carboxypeptidase-like 30 (scpl30); FUNCTIONS IN: serine-type carboxypeptidase activity; INVOLVED IN: proteolysis; LOCATED IN: endomembrane system; EXPRESSED IN: root, flower; EXPRESSED DURING: petal differentiation and expansion stage; CONTAINS InterPro DOMAIN/s: Peptidase S10, serine carboxypeptidase (InterPro:IPR001563), Peptidase S10, serine carboxypeptidase, active site (InterPro:IPR018202); BEST Arabidopsis thaliana protein match is: serine carboxypeptidase-like 31 (TAIR:AT1G11080.2)
AT1G06090	*-1,665*		Fatty acid desaturase family protein; FUNCTIONS IN: oxidoreductase activity, oxidoreductase activity, acting on paired donors, with oxidation of a pair of donors resulting in the reduction of molecular oxygen to two molecules of water; INVOLVED IN: oxidation reduction, lipid metabolic process; LOCATED IN: cellular_component unknown; CONTAINS InterPro DOMAIN/s: Fatty acid desaturase, type 1, core (InterPro:IPR015876), Fatty acid desaturase, type 1 (InterPro:IPR005804); BEST Arabidopsis thaliana protein match is: Fatty acid desaturase family protein (TAIR:AT1G06120.1)
AT2G19150	*-1,667*		Pectin lyase-like superfamily protein; FUNCTIONS IN: pectinesterase activity; INVOLVED IN: cell wall modification; LOCATED IN: endomembrane system, cell wall, plant-type cell wall; EXPRESSED IN: 6 plant structures; EXPRESSED DURING: LP.04 four leaves visible; CONTAINS InterPro DOMAIN/s: Pectin lyase fold/virulence factor (InterPro:IPR011050), Pectinesterase, catalytic (InterPro:IPR000070), Pectin lyase fold (InterPro:IPR012334); BEST Arabidopsis thaliana protein match is: Pectin lyase-like superfamily protein (TAIR:AT2G47280.1)
AT1G52820	*-1,671*		2-oxoglutarate (2OG) and Fe(II)-dependent oxygenase superfamily protein; FUNCTIONS IN: oxidoreductase activity; EXPRESSED IN: root; CONTAINS InterPro DOMAIN/s: Oxoglutarate/iron-dependent oxygenase (InterPro:IPR005123); BEST Arabidopsis thaliana protein match is: 2-oxoglutarate (2OG) and Fe(II)-dependent oxygenase superfamily protein (TAIR:AT4G03070.1)
AT1G60095	*-1,672*		Mannose-binding lectin superfamily protein; CONTAINS InterPro DOMAIN/s: Mannose-binding lectin (InterPro:IPR001229); BEST Arabidopsis thaliana protein match is: Mannose-binding lectin superfamily protein (TAIR:AT1G60110.1)
AT5G60060	*-1,672*		CONTAINS InterPro DOMAIN/s: F-box domain, cyclin-like (InterPro:IPR001810), Protein of unknown function DUF295 (InterPro:IPR005174); BEST Arabidopsis thaliana protein match is: F-box family protein with a domain of unknown function (DUF295) (TAIR:AT2G17030.1)
AT4G27250	*-1,674*		NAD(P)-binding Rossmann-fold superfamily protein; FUNCTIONS IN: cinnamyl-alcohol dehydrogenase activity; INVOLVED IN: cellular metabolic process, metabolic process; LOCATED IN: cellular_component unknown; EXPRESSED IN: 13 plant structures; EXPRESSED DURING: 6 growth stages; CONTAINS InterPro DOMAIN/s: NAD-dependent epimerase/dehydratase (InterPro:IPR001509), NAD(P)-binding domain (InterPro:IPR016040); BEST Arabidopsis thaliana protein match is: dihydroflavonol 4-reductase (TAIR:AT5G42800.1)
AT1G03840	*-1,676*	MAGPIE (MGP)	MGP is a nuclear-localized putative transcription factor with three zinc finger domains. MGP can interact with three proteins implicated in root patterning: SCR, SHR, and JKD in Y2H assays, and these interactions depend on the first zinc finger in MGP. MGP appears to be a direct transcriptional target of SHR and SCR, based on promoter binding assays, though it is not expressed in the QC, based on in situ hybridizations.

AT4G33770	*-1,676*	INOSITOL 1,3,4-TRISPHOSPHATE 5/6 KINASE 2 (ITPK2)	Inositol 1,3,4-trisphosphate 5/6-kinase family protein; FUNCTIONS IN: inositol or phosphatidylinositol kinase activity, magnesium ion binding, inositol-1,3,4-trisphosphate 5/6-kinase activity, inositol tetrakisphosphate 1-kinase activity, ATP binding; INVOLVED IN: inositol trisphosphate metabolic process; LOCATED IN: intracellular; EXPRESSED IN: 21 plant structures; EXPRESSED DURING: 9 growth stages; CONTAINS InterPro DOMAIN/s: Inositol-tetrakisphosphate 1-kinase (InterPro:IPR017427), Inositol 1, 3, 4-trisphosphate 56-kinase (InterPro:IPR008656); BEST Arabidopsis thaliana protein match is: Inositol 1,3,4-trisphosphate 5/6-kinase family protein (TAIR:AT4G08170.2)
AT4G12500	*-1,679*		Bifunctional inhibitor/lipid-transfer protein/seed storage 2S albumin superfamily protein; FUNCTIONS IN: lipid binding; INVOLVED IN: lipid transport; EXPRESSED IN: hypocotyl, leaf; EXPRESSED DURING: LP.04 four leaves visible; CONTAINS InterPro DOMAIN/s: Bifunctional inhibitor/plant lipid transfer protein/seed storage (InterPro:IPR016140), Plant lipid transfer protein/seed storage/trypsin-alpha amylase inhibitor (InterPro:IPR003612), Plant lipid transfer protein/hydrophobic protein, helical domain (InterPro:IPR013770); BEST Arabidopsis thaliana protein match is: Bifunctional inhibitor/lipid-transfer protein/seed storage 2S albumin superfamily protein (TAIR:AT4G12490.1)
AT1G31050	*-1,683*		basic helix-loop-helix (bHLH) DNA-binding superfamily protein; FUNCTIONS IN: sequence-specific DNA binding transcription factor activity; INVOLVED IN: regulation of transcription; LOCATED IN: nucleus; EXPRESSED IN: sperm cell, hypocotyl, root; CONTAINS InterPro DOMAIN/s: Helix-loop-helix DNA-binding (InterPro:IPR011598); BEST Arabidopsis thaliana protein match is: basic helix-loop-helix (bHLH) DNA-binding superfamily protein (TAIR:AT1G61660.3)
AT1G66930	*-1,684*	LEAF RUST 10 DISEASE-RESISTANCE LOCUS RECEPTOR- LIKE PROTEIN KINASE-LIKE2 (LRK10L2)	Protein kinase superfamily protein; FUNCTIONS IN: kinase activity; INVOLVED IN: protein amino acid phosphorylation; LOCATED IN: cellular_component unknown; EXPRESSED IN: root; CONTAINS InterPro DOMAIN/s: Protein kinase, ATP binding site (InterPro:IPR017441), Serine/threonine-protein kinase domain (InterPro:IPR002290), Serine-threonine/tyrosine-protein kinase (InterPro:IPR001245), Protein kinase-like domain (InterPro:IPR011009), Serine/threonine-protein kinase, active site (InterPro:IPR008271), Protein kinase, catalytic domain (InterPro:IPR000719), Tyrosine-protein kinase, catalytic domain (InterPro:IPR020635); BEST Arabidopsis thaliana protein match is: Protein kinase superfamily protein (TAIR:AT1G67000.1)
AT5G45670	*-1,685*		GDSL-motif esterase/acyltransferase/lipase. Enzyme group with broad substrate specificity that may catalyze acyltransfer or hydrolase reactions with lipid and non-lipid substrates.
AT1G09090	*-1,69*	RESPIRATORY BURST OXIDASE HOMOLOG B (RBOHB)	NADPH-oxidase AtrbohB plays a role in seed after-ripening. Major producer of superoxide in germinating seeds. AtrbohB pre-mRNA is alternatively spliced in seeds in a hormonally and developmentally regulated manner. ABA caused accumulation of AtrbohB-? mRNA and prevented prevented AtrbohB-a mRNA expression in fresh seeds.
AT2G39200	*-1,692*	MILDEW RESISTANCE LOCUS O 12 (MLO12)	A member of a large family of seven-transmembrane domain proteins specific to plants, homologs of the barley mildew resistance locus o (MLO) protein. The Arabidopsis genome contains 15 genes encoding MLO proteins, with localization in plasma membrane. Phylogenetic analysis revealed four clades of closely-related AtMLO genes. ATMLO6 belongs to the clade IV, with AtMLO2, AtMLO3 and AtMLO12. The gene is expressed during early seedling growth, in root tips and cotyledon vascular system, in floral organs (anthers and stigma), and in fruit abscission zone, as shown by GUS activity patterns. The expression of several phylogenetically closely-related AtMLO genes showed similar or overlapping tissue specificity and analogous responsiveness to external stimuli, suggesting functional redundancy, co-function, or antagonistic function(s).
AT1G17190	*-1,693*	GLUTATHIONE S-TRANSFERASE TAU 26	Encodes glutathione transferase belonging to the tau class of GSTs. Naming convention according to Wagner et al. (2002).
AT4G11320	*-1,695*	CYSTEINE PROTEASE 2 (CP2)	Papain family cysteine protease; FUNCTIONS IN: cysteine-type endopeptidase activity, cysteine-type peptidase activity; INVOLVED IN: proteolysis; LOCATED IN: endomembrane system; CONTAINS InterPro DOMAIN/s: Proteinase inhibitor I29, cathepsin propeptide (InterPro:IPR013201), Peptidase C1A, papain (InterPro:IPR013128), Peptidase C1A, papain C-terminal (InterPro:IPR000668), Peptidase, cysteine peptidase active site (InterPro:IPR000169); BEST Arabidopsis thaliana protein match is: Papain family cysteine protease (TAIR:AT4G11310.1)
AT1G58320	*-1,703*		PLAC8 family protein; CONTAINS InterPro DOMAIN/s: Protein of unknown function Cys-rich (InterPro:IPR006461); BEST Arabidopsis thaliana protein match is: PLAC8 family protein (TAIR:AT1G68630.1)
AT4G19980	*-1,714*		unknown protein; FUNCTIONS IN: molecular_function unknown; INVOLVED IN: biological_process unknown; LOCATED IN: chloroplast; EXPRESSED IN: root, pedicel; EXPRESSED DURING: 4 anthesis

AT4G15370	-1,715	BARUOL SYNTHASE 1 (BARS1)	Encodes an oxidosqualene cyclase that primarily produces the tetracyclic triterpene baruol in vitro and when expressed in yeast. It can also make 22 other minor triterpenoid products with varying numbers of rings.
AT2G43050	-1,716	(ATPMEPCRD)	ATPMEPCRD; FUNCTIONS IN: enzyme inhibitor activity, pectinesterase activity; INVOLVED IN: cell wall modification; LOCATED IN: cell wall, plant-type cell wall; EXPRESSED IN: 9 plant structures; EXPRESSED DURING: 4 anthesis, petal differentiation and expansion stage; CONTAINS InterPro DOMAIN/s: Pectinesterase, active site (InterPro:IPR018040), Pectin lyase fold/virulence factor (InterPro:IPR011050), Pectinesterase, catalytic (InterPro:IPR000070), Pectinesterase inhibitor (InterPro:IPR006501), Pectin lyase fold (InterPro:IPR012334); BEST Arabidopsis thaliana protein match is: pectin methylesterase 61 (TAIR:AT3G59010.1)
AT2G36830	-1,724	GAMMA TONOPLAST INTRINSIC PROTEIN (GAMMA-TIP)	Encodes a tonoplast intrinsic protein, which functions as water channel. It has also been shown to be able to facilitate the transport of urea and hydrogen peroxide. Highly expressed in vascular tissues of the root, stem, cauline leaves and flowers but not in the apical meristems. The mRNA is cell-to-cell mobile.
AT5G58780	-1,727	CIS -PRENYLTRANSFERASE 5 (CPT5)	Encodes a novel Z,E-mixed heptaprenyl diphosphate (Z,E-HepPP) synthase, which may be responsible for short-chain betulaprenols. It catalyzes the formation of C 35 short-chain polyisoprenoids in which the optimal allylic substrate was E,E-FPP. It may have a role in response to cold stress in root.
AT1G71030	-1,729	MYB-LIKE 2 (MYBL2)	Encodes a putative myb family transcription factor. In contrast to most other myb-like proteins its myb domain consists of a single repeat. A proline-rich region potentially involved in transactivation is found in the C-terminal part of the protein. Its transcript accumulates mainly in leaves.
AT2G16980	-1,734		Major facilitator superfamily protein; FUNCTIONS IN: tetracycline transporter activity; INVOLVED IN: response to antibiotic, tetracycline transport, transmembrane transport; LOCATED IN: endomembrane system, integral to membrane; EXPRESSED IN: 6 plant structures; EXPRESSED DURING: L mature pollen stage, 4 anthesis; CONTAINS InterPro DOMAIN/s: Tetracycline resistance protein, TetA (InterPro:IPR001958), Major facilitator superfamily MFS-1 (InterPro:IPR011701), Major facilitator superfamily, general substrate transporter (InterPro:IPR016196); BEST Arabidopsis thaliana protein match is: Major facilitator superfamily protein (TAIR:AT2G16970.1)
AT3G51060	-1,74	STYLISH 1 (STY1)	A member of SHI gene family. Arabidopsis thaliana has ten members that encode proteins with a RING finger-like zinc finger motif. Despite being highly divergent in sequence, many of the SHI-related genes are partially redundant in function and synergistically promote gynoecium, stamen and leaf development in Arabidopsis. STY1/STY2 double mutants showed defective style, stigma as well as serrated leaves. Binds to the promoter of YUC4 and YUC8 (binding site ACTCTAC)
AT1G71692	-1,741	AGAMOUS-LIKE 12 (AGL12)	Encodes a member of the MADS box family of transcription factors. Involved in root cell differentiation and flowering time. Loss of function mutations have abnormal cellular differentiation in the roots and are late flowering. AGL12 along with AGL14, and AGL17 is preferentially expressed in root tissues and represent the only characterized MADS box genes expressed in roots.
AT5G56795	-1,741	METALLOTHIONEIN 1B (MT1B)	one of the five metallothioneins (MTs) genes identified in Arabidopsis. MTs are cysteine-rich proteins required for heavy metal tolerance. The MT1b gene, however, is indicated to be inactive.
AT5G59930	-1,743		Cysteine/Histidine-rich C1 domain family protein; FUNCTIONS IN: zinc ion binding; INVOLVED IN: intracellular signaling pathway; EXPRESSED IN: root; CONTAINS InterPro DOMAIN/s: Protein kinase C-like, phorbol ester/diacylglycerol binding (InterPro:IPR002219), DC1 (InterPro:IPR004146), Zinc finger, PHD-type (InterPro:IPR001965), Zinc finger, ZZ-type (InterPro:IPR000433), C1-like (InterPro:IPR011424); BEST Arabidopsis thaliana protein match is: Cysteine/Histidine-rich C1 domain family protein (TAIR:AT5G59940.1)
AT5G20470	-1,744	HEADLESS DERIVATIVE OF MYOSIN XI-K (HDK)	Encodes a headless derivative of myosin XI-K, which likely arose from a partial duplication of the XI-K gene and is developmentally regulated.
AT1G73340	-1,746	ABIETANE DITERPENE OXIDASE 1 (ADTO1)	ADTO1 is required for the activation of systemic acquired resistance.

AT5G47240	*-1,746*	NUDIX HYDROLASE HOMOLOG 8 (NUDT8)	nudix hydrolase homolog 8 (NUDT8); FUNCTIONS IN: hydrolase activity; INVOLVED IN: response to wounding; LOCATED IN: cytosol; EXPRESSED IN: 22 plant structures; EXPRESSED DURING: 13 growth stages; CONTAINS InterPro DOMAIN/s: NUDIX hydrolase domain-like (InterPro:IPR015797), NUDIX hydrolase, conserved site (InterPro:IPR020084), Nudix hydrolase 6-like (InterPro:IPR003293), NUDIX hydrolase domain (InterPro:IPR000086); BEST Arabidopsis thaliana protein match is: nudix hydrolase homolog 2 (TAIR:AT5G47650.1)
AT1G53940	*-1,748*	GDSL-MOTIF LIPASE 2 (GLIP2)	Encodes a lipase, has in vitro lipase activity with p-nitrophenyl acetate and p-nitrophenyl butyrate, gene expression induced by hormones, negatively regulates auxin signaling, involved in disease resistance
AT5G06270	*-1,748*		unknown protein; FUNCTIONS IN: molecular_function unknown; INVOLVED IN: biological_process unknown; LOCATED IN: chloroplast; EXPRESSED IN: 21 plant structures; EXPRESSED DURING: 11 growth stages; BEST Arabidopsis thaliana protein match is: unknown protein (TAIR:AT3G11600.1)
AT5G64110	*-1,748*		Peroxidase superfamily protein; FUNCTIONS IN: peroxidase activity, heme binding; INVOLVED IN: response to oxidative stress, oxidation reduction; LOCATED IN: membrane; EXPRESSED IN: shoot apex, shoot, stamen, leaf; EXPRESSED DURING: 4 anthesis; CONTAINS InterPro DOMAIN/s: Haem peroxidase (InterPro:IPR010255), Plant peroxidase (InterPro:IPR000823), Peroxidases heam-ligand binding site (InterPro:IPR019793), Haem peroxidase, plant/fungal/bacterial (InterPro:IPR002016), Peroxidase, active site (InterPro:IPR019794); BEST Arabidopsis thaliana protein match is: Peroxidase superfamily protein (TAIR:AT5G64100.1)
AT2G37180	*-1,758*	RESPONSIVE TO DESICCATION 28 (RD28)	a member of the plasma membrane intrinsic protein PIP2. functions as aquaporin and is involved in desiccation.
AT3G61390	*-1,762*		RING/U-box superfamily protein; FUNCTIONS IN: ubiquitin-protein ligase activity; INVOLVED IN: protein ubiquitination; LOCATED IN: ubiquitin ligase complex; EXPRESSED IN: 17 plant structures; EXPRESSED DURING: 11 growth stages; CONTAINS InterPro DOMAIN/s: U box domain (InterPro:IPR003613); BEST Arabidopsis thaliana protein match is: U-box domain-containing protein (TAIR:AT2G45920.1)
AT4G18340	*-1,764*		Glycosyl hydrolase superfamily protein; FUNCTIONS IN: cation binding, hydrolase activity, hydrolyzing O-glycosyl compounds, catalytic activity; INVOLVED IN: carbohydrate metabolic process; LOCATED IN: endomembrane system; EXPRESSED IN: 21 plant structures; EXPRESSED DURING: 13 growth stages; CONTAINS InterPro DOMAIN/s: Glycoside hydrolase, catalytic core (InterPro:IPR017853), Glycoside hydrolase, family 17 (InterPro:IPR000490), Glycoside hydrolase, subgroup, catalytic core (InterPro:IPR013781); BEST Arabidopsis thaliana protein match is: Glycosyl hydrolase superfamily protein (TAIR:AT1G30080.1)
AT1G33320	*-1,768*		Pyridoxal phosphate (PLP)-dependent transferases superfamily protein; FUNCTIONS IN: pyridoxal phosphate binding, cystathionine gamma-synthase activity, catalytic activity; INVOLVED IN: methionine biosynthetic process, cellular amino acid metabolic process; EXPRESSED IN: root; CONTAINS InterPro DOMAIN/s: Pyridoxal phosphate-dependent transferase, major domain (InterPro:IPR015424), Cys/Met metabolism, pyridoxal phosphate-dependent enzyme (InterPro:IPR000277), Pyridoxal phosphate-dependent transferase, major region, subdomain 1 (InterPro:IPR015421), Pyridoxal phosphate-dependent transferase, major region, subdomain 2 (InterPro:IPR015422); BEST Arabidopsis thaliana protein match is: Pyridoxal phosphate (PLP)-dependent transferases superfamily protein (TAIR:AT3G01120.1)
AT5G14880	*-1,775*	POTASSIUM UPTAKE 8 (KUP8)	Potassium transporter family protein; FUNCTIONS IN: potassium ion transmembrane transporter activity; INVOLVED IN: potassium ion transport; LOCATED IN: membrane; EXPRESSED IN: 22 plant structures; EXPRESSED DURING: 13 growth stages; CONTAINS InterPro DOMAIN/s: Potassium uptake protein, kup (InterPro:IPR018519), K+ potassium transporter (InterPro:IPR003855); BEST Arabidopsis thaliana protein match is: K+ uptake permease 6 (TAIR:AT1G70300.1)
AT2G02130	*-1,788*	LOW-MOLECULAR-WEIGHT CYSTEINE-RICH 68 (LCR68)	Predicted to encode a PR (pathogenesis-related) protein. Belongs to the plant defensin (PDF) family with the following members: At1g75830/PDF1.1, At5g44420/PDF1.2a, At2g26020/PDF1.2b, At5g44430/PDF1.2c, At2g26010/PDF1.3, At1g19610/PDF1.4, At1g55010/PDF1.5, At2g02120/PDF2.1, At2g02100/PDF2.2, At2g02130/PDF2.3, At1g61070/PDF2.4, At5g63660/PDF2.5, At2g02140/PDF2.6, At5g38330/PDF3.1 and At4g30070/PDF3.2. The mRNA is cell-to-cell mobile.

AT5G64100	*-1,788*		Peroxidase superfamily protein; FUNCTIONS IN: peroxidase activity, heme binding; INVOLVED IN: oxidation reduction, response to oxidative stress; LOCATED IN: cell wall; EXPRESSED IN: hypocotyl, root, callus; EXPRESSED DURING: seedling growth; CONTAINS InterPro DOMAIN/s: Haem peroxidase (InterPro:IPR010255), Plant peroxidase (InterPro:IPR000823), Peroxidases heam-ligand binding site (InterPro:IPR019793), Haem peroxidase, plant/fungal/bacterial (InterPro:IPR002016), Peroxidase, active site (InterPro:IPR019794); BEST Arabidopsis thaliana protein match is: Peroxidase superfamily protein (TAIR:AT5G64110.1)
AT5G44020	*-1,789*		HAD superfamily, subfamily IIIB acid phosphatase ; FUNCTIONS IN: acid phosphatase activity; INVOLVED IN: biological_process unknown; LOCATED IN: chloroplast, vacuole, membrane; EXPRESSED IN: 23 plant structures; EXPRESSED DURING: 13 growth stages; CONTAINS InterPro DOMAIN/s: Acid phosphatase (Class B) (InterPro:IPR005519), Vegetative storage protein/acid phosphatase (InterPro:IPR014403), Acid phosphatase, plant (InterPro:IPR010028); BEST Arabidopsis thaliana protein match is: HAD superfamily, subfamily IIIB acid phosphatase (TAIR:AT1G04040.1)
AT1G11460	*-1,791*	USUALLY MULTIPLE ACIDS MOVE IN AND OUT TRANSPORTERS 26 (UMAMIT26)	nodulin MtN21-like transporter family protein
AT3G60550	*-1,791*	CYCLIN P3;2 (CYCP3;2)	cyclin p3;2 (CYCP3;2); CONTAINS InterPro DOMAIN/s: Negative regulatory factor PREG (InterPro:IPR012389), Cyclin-like (InterPro:IPR011028), Cyclin-related 2 (InterPro:IPR013922); BEST Arabidopsis thaliana protein match is: cyclin p3;1 (TAIR:AT2G45080.1)
AT1G18870	*-1,796*	ISOCHORISMATE SYNTHASE 2 (ICS2)	Encodes a protein with isochorismate synthase activity involved in phylloquinone biosynthesis. Mutant studies of this gene's function suggest that its function is redundant with that of ICS1 (AT1G7410).
AT4G12030	*-1,8*	BILE ACID TRANSPORTER 5 (BAT5)	Required for the biosynthesis of methionine-derived glucosinolates. Involved in the transport of 2-keto acids between chloroplasts and the cytosol.
AT1G80760	*-1,801*	NOD26-LIKE INTRINSIC PROTEIN 6;1 (NIP6;1)	Encodes a protein with boron transporter activity. It helps to preferentially direct boron to young developing tissues in the shoot, such as immature leaves, under low boron conditions. This boron channel appears to be impermeable to water, unlike the closely related NIP5;1 boron transporter. This protein also allows the transport of glycerol, urea, and formimide but not larger uncharged solutes such as arabitol and sucrose when it is expressed heterologously.
AT1G21310	*-1,805*	EXTENSIN 3 (EXT3)	Encodes extensin 3.
AT4G26050	*-1,805*	PLANT INTRACELLULAR RAS GROUP-RELATED LRR 8 (PIRL8)	Encodes PIRL8, a member of the Plant Intracellular Ras-group-related LRRs (Leucine rich repeat proteins). PIRLs are a distinct, plant-specific class of intracellular LRRs that likely mediate protein interactions, possibly in the context of signal transduction. The mRNA is cell-to-cell mobile.
AT1G51880	*-1,808*	ROOT HAIR SPECIFIC 6 (RHS6)	root hair specific 6 (RHS6); FUNCTIONS IN: kinase activity; INVOLVED IN: protein amino acid phosphorylation; LOCATED IN: endomembrane system; EXPRESSED IN: root hair; CONTAINS InterPro DOMAIN/s: Protein kinase, ATP binding site (InterPro:IPR017441), Serine/threonine-protein kinase domain (InterPro:IPR002290), Leucine-rich repeat (InterPro:IPR001611), Serine-threonine/tyrosine-protein kinase (InterPro:IPR001245), Protein kinase-like domain (InterPro:IPR011009), Serine/threonine-protein kinase, active site (InterPro:IPR008271), Protein kinase, catalytic domain (InterPro:IPR000719), Tyrosine-protein kinase, catalytic domain (InterPro:IPR020635); BEST Arabidopsis thaliana protein match is: Leucine-rich repeat protein kinase family protein (TAIR:AT1G51860.1)
AT4G35060	*-1,808*	HEAVY METAL ASSOCIATED ISOPRENYLATED PLANT PROTEIN 25 (HIPP25)	Heavy metal transport/detoxification superfamily protein ; FUNCTIONS IN: metal ion binding; INVOLVED IN: metal ion transport; LOCATED IN: cellular_component unknown; EXPRESSED IN: 14 plant structures; EXPRESSED DURING: LP.06 six leaves visible, LP.04 four leaves visible, 4 anthesis, C globular stage, petal differentiation and expansion stage; CONTAINS InterPro DOMAIN/s: Heavy metal transport/detoxification protein (InterPro:IPR006121); BEST Arabidopsis thaliana protein match is: farnesylated protein 6 (TAIR:AT4G38580.1)

AT1G20380	*-1,811*		Prolyl oligopeptidase family protein; FUNCTIONS IN: serine-type peptidase activity, serine-type endopeptidase activity; INVOLVED IN: proteolysis; LOCATED IN: cellular_component unknown; EXPRESSED IN: 19 plant structures; EXPRESSED DURING: 9 growth stages; CONTAINS InterPro DOMAIN/s: Peptidase S9, prolyl oligopeptidase, catalytic domain (InterPro:IPR001375), Peptidase S9A, oligopeptidase, N-terminal beta-propeller (InterPro:IPR004106), Peptidase S9A, prolyl oligopeptidase (InterPro:IPR002470); BEST Arabidopsis thaliana protein match is: Prolyl oligopeptidase family protein (TAIR:AT1G76140.1)
AT2G29330	*-1,813*	TROPINONE REDUCTASE (TRI)	tropinone reductase (TRI); FUNCTIONS IN: oxidoreductase activity, binding, catalytic activity; INVOLVED IN: oxidation reduction, metabolic process; EXPRESSED IN: embryo, hypocotyl, root; EXPRESSED DURING: C globular stage; CONTAINS InterPro DOMAIN/s: NAD(P)-binding domain (InterPro:IPR016040), Glucose/ribitol dehydrogenase (InterPro:IPR002347), Short-chain dehydrogenase/reductase SDR (InterPro:IPR002198); BEST Arabidopsis thaliana protein match is: NAD(P)-binding Rossmann-fold superfamily protein (TAIR:AT2G30670.1)
AT5G59270	*-1,814*	L-TYPE LECTIN RECEPTOR KINASE II.2 (LECRK-II.2)	Concanavalin A-like lectin protein kinase family protein; FUNCTIONS IN: kinase activity; INVOLVED IN: protein amino acid phosphorylation; LOCATED IN: endomembrane system; CONTAINS InterPro DOMAIN/s: Legume lectin, beta chain (InterPro:IPR001220), Protein kinase, ATP binding site (InterPro:IPR017441), Serine/threonine-protein kinase domain (InterPro:IPR002290), Serine/threonine-protein kinase-like domain (InterPro:IPR017442), Concanavalin A-like lectin/glucanase, subgroup (InterPro:IPR013320), Serine/threonine-protein kinase, active site (InterPro:IPR008271), Protein kinase-like domain (InterPro:IPR011009), Protein kinase, catalytic domain (InterPro:IPR000719), Tyrosine-protein kinase, catalytic domain (InterPro:IPR020635), Concanavalin A-like lectin/glucanase (InterPro:IPR008985), Legume lectin, beta chain, Mn/Ca-binding site (InterPro:IPR019825); BEST Arabidopsis thaliana protein match is: Concanavalin A-like lectin protein kinase family protein (TAIR:AT5G59260.1)
AT2G30080	*-1,816*	(ZIP6)	member of Fe(II) transporter isolog family. Gene expression is not regulated by iron, copper, or zinc deficiency or excess.
AT5G57770	*-1,817*		CONTAINS InterPro DOMAIN/s: Pleckstrin-like, plant (InterPro:IPR013666), Protein of unknown function DUF828 (InterPro:IPR008546); BEST Arabidopsis thaliana protein match is: Plant protein of unknown function (DUF828) with plant pleckstrin homology-like region (TAIR:AT3G22810.1)
AT3G05140	*-1,818*	ROP BINDING PROTEIN KINASES 2 (RBK2)	ROP binding protein kinases 2 (RBK2); FUNCTIONS IN: protein serine/threonine kinase activity, protein kinase activity, kinase activity, ATP binding; INVOLVED IN: protein amino acid phosphorylation; LOCATED IN: nucleus, cytoplasm; EXPRESSED IN: 8 plant structures; EXPRESSED DURING: L mature pollen stage, M germinated pollen stage, 4 anthesis; CONTAINS InterPro DOMAIN/s: Protein kinase, ATP binding site (InterPro:IPR017441), Protein kinase, catalytic domain (InterPro:IPR000719), Serine-threonine/tyrosine-protein kinase (InterPro:IPR001245), Protein kinase-like domain (InterPro:IPR011009), Serine/threonine-protein kinase, active site (InterPro:IPR008271); BEST Arabidopsis thaliana protein match is: Protein kinase superfamily protein (TAIR:AT5G18910.1)
AT1G52060	*-1,822*		Mannose-binding lectin superfamily protein; FUNCTIONS IN: molecular_function unknown; INVOLVED IN: biological_process unknown; LOCATED IN: endomembrane system; EXPRESSED IN: root; CONTAINS InterPro DOMAIN/s: Mannose-binding lectin (InterPro:IPR001229); BEST Arabidopsis thaliana protein match is: Mannose-binding lectin superfamily protein (TAIR:AT1G52070.1)
AT2G38460	*-1,832*	IRON REGULATED 1 (IREG1)	Encodes IRON REGULATED1 (IREG1/FPN1), one of the Arabidopsis orthologs (AT2G38460/IREG1/FPN1 and AT5G03570/IREG2/FPN2) the iron efflux transporter ferroportin (FPN) identified in animals.
AT5G52790	*-1,832*		FUNCTIONS IN: molecular_function unknown; INVOLVED IN: biological_process unknown; LOCATED IN: endomembrane system; CONTAINS InterPro DOMAIN/s: Protein of unknown function DUF21 (InterPro:IPR002550), Cystathionine beta-synthase, core (InterPro:IPR000644); BEST Arabidopsis thaliana protein match is: CBS domain-containing protein with a domain of unknown function (DUF21) (TAIR:AT2G14520.1)
AT4G01440	*-1,837*	USUALLY MULTIPLE ACIDS MOVE IN AND OUT TRANSPORTERS 31 (UMAMIT31)	nodulin MtN21-like transporter family protein

AT2G35380	*-1,845*		Peroxidase superfamily protein; FUNCTIONS IN: peroxidase activity, heme binding; INVOLVED IN: oxidation reduction, response to oxidative stress; LOCATED IN: endomembrane system; EXPRESSED IN: hypocotyl, root; CONTAINS InterPro DOMAIN/s: Haem peroxidase (InterPro:IPR010255), Plant peroxidase (InterPro:IPR000823), Peroxidases heam-ligand binding site (InterPro:IPR019793), Haem peroxidase, plant/fungal/bacterial (InterPro:IPR002016), Peroxidase, active site (InterPro:IPR019794); BEST Arabidopsis thaliana protein match is: Peroxidase superfamily protein (TAIR:AT2G18150.1)
AT2G43590	*-1,852*		Chitinase family protein; FUNCTIONS IN: chitin binding, chitinase activity; INVOLVED IN: carbohydrate metabolic process, cell wall macromolecule catabolic process; LOCATED IN: endomembrane system; EXPRESSED IN: 7 plant structures; EXPRESSED DURING: LP.04 four leaves visible, 4 anthesis, C globular stage, LP.02 two leaves visible; CONTAINS InterPro DOMAIN/s: Chitin-binding, type 1, conserved site (InterPro:IPR018371), Glycoside hydrolase, family 19 (InterPro:IPR016283), Chitin-binding, type 1 (InterPro:IPR001002), Glycoside hydrolase, family 19, catalytic (InterPro:IPR000726); BEST Arabidopsis thaliana protein match is: Chitinase family protein (TAIR:AT2G43580.1)
AT4G12470	*-1,852*	AZELAIC ACID INDUCED 1 (AZI1)	Encodes AZI1 (AZELAIC ACID INDUCED 1). Involved in the priming of salicylic acid induction and systemic immunity triggered by pathogen or azelaic acid. Targeting if AZI1 to chloroplasts is increased during SAR induction and that localization requires the PRR domain.It is involved in the uptake and movement of the azelaic acid signal.
AT2G36690	*-1,854*		2-oxoglutarate (2OG) and Fe(II)-dependent oxygenase superfamily protein; FUNCTIONS IN: oxidoreductase activity, acting on paired donors, with incorporation or reduction of molecular oxygen, 2-oxoglutarate as one donor, and incorporation of one atom each of oxygen into both donors, oxidoreductase activity; INVOLVED IN: biosynthetic process; LOCATED IN: cellular_component unknown; EXPRESSED IN: hypocotyl, root; CONTAINS InterPro DOMAIN/s: Oxoglutarate/iron-dependent oxygenase (InterPro:IPR005123); BEST Arabidopsis thaliana protein match is: 2-oxoglutarate (2OG) and Fe(II)-dependent oxygenase superfamily protein (TAIR:AT3G60290.1)
AT5G54700	*-1,863*		Ankyrin repeat family protein; CONTAINS InterPro DOMAIN/s: Ankyrin repeat-containing domain (InterPro:IPR020683), Ankyrin repeat (InterPro:IPR002110); BEST Arabidopsis thaliana protein match is: Ankyrin repeat family protein (TAIR:AT5G54710.1)
AT5G24780	*-1,864*	VEGETATIVE STORAGE PROTEIN 1 (VSP1)	encodes an acid phosphatase similar to soybean vegetative storage proteins. Gene expression is induced by wounding and jasmonic acid.
AT1G70850	*-1,865*	MLP-LIKE PROTEIN 34 (MLP34)	MLP-like protein 34 (MLP34); FUNCTIONS IN: molecular_function unknown; INVOLVED IN: response to biotic stimulus, defense response; LOCATED IN: cellular_component unknown; EXPRESSED IN: 6 plant structures; EXPRESSED DURING: 4 anthesis, petal differentiation and expansion stage; CONTAINS InterPro DOMAIN/s: Bet v I allergen (InterPro:IPR000916); BEST Arabidopsis thaliana protein match is: MLP-like protein 28 (TAIR:AT1G70830.1)
AT1G15550	*-1,866*	GIBBERELLIN 3-OXIDASE 1 (GA3OX1)	Involved in later steps of the gibberellic acid biosynthetic pathway. Activated by AGAMOUS in a cal-1, ap1-1 background. Deletion of 208 bp from -1016 to -809 (Δ-808) resulted in loss of GA-negative feedback (this sequence, which contains a 43-bp sequence GNFEI, was shown to be sufficient for GA-negative feedback).
AT3G23000	*-1,871*	CBL-INTERACTING PROTEIN KINASE 7 (CIPK7)	Encodes a serine/threonine protein kinase with similarities to CBL-interacting protein kinases, SNF1 and SOS2. The mRNA is cell-to-cell mobile.
AT4G17340	*-1,874*	TONOPLAST INTRINSIC PROTEIN 2;2 (TIP2;2)	tonoplast intrinsic protein 2;2 (TIP2;2); FUNCTIONS IN: water channel activity; INVOLVED IN: response to salt stress, transport; LOCATED IN: in 6 components; EXPRESSED IN: 25 plant structures; EXPRESSED DURING: 10 growth stages; CONTAINS InterPro DOMAIN/s: Major intrinsic protein, conserved site (InterPro:IPR022357), Aquaporin (InterPro:IPR012269), Major intrinsic protein (InterPro:IPR000425); BEST Arabidopsis thaliana protein match is: tonoplast intrinsic protein 2;3 (TAIR:AT5G47450.1)
AT2G37170	*-1,882*	PLASMA MEMBRANE INTRINSIC PROTEIN 2 (PIP2B)	a member of the plasma membrane intrinsic protein subfamily PIP2. localizes to the plasma membrane and exhibits water transport activity in Xenopus oocyte. expressed specifically in the vascular bundles and protein level increases slightly during leaf dev

AT2G43100	*-1,886*	ISOPROPYLMALATE ISOMERASE 2 (IPMI2)	isopropylmalate isomerase 2 (IPMI2); FUNCTIONS IN: hydro-lyase activity, 3-isopropylmalate dehydratase activity; INVOLVED IN: leucine biosynthetic process, metabolic process; LOCATED IN: plastid; EXPRESSED IN: 20 plant structures; EXPRESSED DURING: 10 growth stages; CONTAINS InterPro DOMAIN/s: 3-isopropylmalate dehydratase, small subunit-like (InterPro:IPR012305), Aconitase A/isopropylmalate dehydratase small subunit, swivel (InterPro:IPR000573), Aconitase/3-isopropylmalate dehydratase, swivel (InterPro:IPR015928), Aconitase-like core (InterPro:IPR015937); BEST Arabidopsis thaliana protein match is: Aconitase/3-isopropylmalate dehydratase protein (TAIR:AT2G43090.1)
AT5G60530	*-1,893*		late embryogenesis abundant protein-related / LEA protein-related; FUNCTIONS IN: molecular_function unknown; LOCATED IN: endomembrane system; EXPRESSED IN: root; CONTAINS InterPro DOMAIN/s: Root cap (InterPro:IPR009646); BEST Arabidopsis thaliana protein match is: Late embryogenesis abundant (LEA) protein-related (TAIR:AT5G60520.1)
AT1G70210	*-1,894*	CYCLIN D1;1 (CYCD1;1)	Encodes a D-type cyclin that physically interacts with CDC2A. Its expression is upregulated early during germination.
AT5G43540	*-1,897*		C2H2 and C2HC zinc fingers superfamily protein; FUNCTIONS IN: sequence-specific DNA binding transcription factor activity, zinc ion binding, nucleic acid binding; INVOLVED IN: regulation of transcription; LOCATED IN: intracellular, chloroplast; CONTAINS InterPro DOMAIN/s: Zinc finger, C2H2-like (InterPro:IPR015880), Zinc finger, C2H2-type (InterPro:IPR007087); BEST Arabidopsis thaliana protein match is: C2H2 and C2HC zinc fingers superfamily protein (TAIR:AT3G53820.1)
AT1G53610	*-1,899*		unknown protein; FUNCTIONS IN: molecular_function unknown; INVOLVED IN: biological_process unknown; LOCATED IN: endomembrane system; BEST Arabidopsis thaliana protein match is: unknown protein (TAIR:AT1G53620.1)
AT2G01520	*-1,901*	MLP-LIKE PROTEIN 328 (MLP328)	Encodes a cis-cinnamic acid responsive gene that is a member of the major latex protein-like gene family and plays a role in promoting vegetative growth and delaying ﬂowering. The mRNA is cell-to-cell mobile.
AT4G33790	*-1,901*	ECERIFERUM 4 (CER4)	Encodes an alcohol-forming fatty acyl-CoA reductase, involved in cuticular wax biosynthesis. Lines carrying recessive mutations are deficient in primary alcohol and have glossy stem surfaces.
AT5G09220	*-1,906*	AMINO ACID PERMEASE 2 (AAP2)	member of AAAP family The mRNA is cell-to-cell mobile.
AT1G22500	*-1,908*	ARABIDOPSIS TOXICOS EN LEVADURA 15 (ATL15)	Gene encodes a putative C3HC4-type RING zinc finger factor. it is induced in response to light and ascorbate stimulus.
AT1G78090	*-1,915*	TREHALOSE-6-PHOSPHATE PHOSPHATASE B (TPPB)	homologous to the C-terminal part of microbial trehalose-6-phosphate phosphatases
AT1G19390	*-1,917*		Wall-associated kinase family protein; FUNCTIONS IN: kinase activity; INVOLVED IN: protein amino acid phosphorylation; LOCATED IN: endomembrane system, integral to membrane; CONTAINS InterPro DOMAIN/s: Wall-associated kinase (InterPro:IPR013695), EGF-like calcium-binding, conserved site (InterPro:IPR018097), Protein kinase, catalytic domain (InterPro:IPR000719), Serine/threonine-protein kinase-like domain (InterPro:IPR017442), Protein kinase-like domain (InterPro:IPR011009), Serine/threonine-protein kinase, active site (InterPro:IPR008271); BEST Arabidopsis thaliana protein match is: wall-associated kinase, putative (TAIR:AT4G31100.1)
AT2G16750	*-1,918*		Protein kinase protein with adenine nucleotide alpha hydrolases-like domain; FUNCTIONS IN: protein serine/threonine kinase activity, protein kinase activity, kinase activity, ATP binding; INVOLVED IN: protein amino acid phosphorylation; LOCATED IN: endomembrane system; EXPRESSED IN: 18 plant structures; EXPRESSED DURING: 11 growth stages; CONTAINS InterPro DOMAIN/s: Protein kinase, catalytic domain (InterPro:IPR000719), Serine/threonine-protein kinase-like domain (InterPro:IPR017442), Protein kinase-like domain (InterPro:IPR011009), Serine/threonine-protein kinase, active site (InterPro:IPR008271); BEST Arabidopsis thaliana protein match is: Protein kinase superfamily protein (TAIR:AT4G35030.3)
AT1G50060	*-1,922*		CAP (Cysteine-rich secretory proteins, Antigen 5, and Pathogenesis-related 1 protein) superfamily protein; FUNCTIONS IN: molecular_function unknown; INVOLVED IN: biological_process unknown; LOCATED IN: endomembrane system, extracellular region; EXPRESSED IN: root; CONTAINS InterPro DOMAIN/s: Allergen V5/Tpx-1 related (InterPro:IPR001283), Ves allergen (InterPro:IPR002413), SCP-like extracellular (InterPro:IPR014044); BEST Arabidopsis thaliana protein match is: CAP (Cysteine-rich secretory proteins, Antigen 5, and Pathogenesis-related 1 protein) superfamily protein (TAIR:AT1G50050.1)

AT5G10580	-1,926		Protein of unknown function, DUF599; FUNCTIONS IN: molecular_function unknown; INVOLVED IN: biological_process unknown; LOCATED IN: endomembrane system; EXPRESSED IN: hypocotyl, root, flower; EXPRESSED DURING: petal differentiation and expansion stage; CONTAINS InterPro DOMAIN/s: Protein of unknown function DUF599 (InterPro:IPR006747); BEST Arabidopsis thaliana protein match is: Protein of unknown function, DUF599 (TAIR:AT4G31330.1)
AT1G77520	-1,927		O-methyltransferase family protein; CONTAINS InterPro DOMAIN/s: Winged helix-turn-helix transcription repressor DNA-binding (InterPro:IPR011991), Plant methyltransferase dimerisation (InterPro:IPR012967), O-methyltransferase, family 2 (InterPro:IPR001077), O-methyltransferase, COMT, eukaryota (InterPro:IPR016461); BEST Arabidopsis thaliana protein match is: O-methyltransferase family protein (TAIR:AT1G77530.1)
AT3G02850	-1,93	STELAR K+ OUTWARD RECTIFIER (SKOR)	Encodes SKOR, a member of Shaker family potassium ion (K+) channel. This family includes five groups based on phylogenetic analysis (FEBS Letters (2007) 581: 2357): I (inward rectifying channel): AKT1 (AT2G26650), AKT5 (AT4G32500) and SPIK (also known as AKT6, AT2G25600); II (inward rectifying channel): KAT1 (AT5G46240) and KAT2 (AT4G18290); III (weakly inward rectifying channel): AKT2 (AT4G22200); IV (regulatory subunit involved in inwardly rectifying conductance formation): KAT3 (also known as AtKC1, AT4G32650); V (outward rectifying channel): SKOR (AT3G02850) and GORK (AT5G37500). Mediates the delivery of K+ from stelar cells to the xylem in the roots towards the shoot. mRNA accumulation is modulated by abscisic acid. K+ gating activity is modulated by external and internal K+. Involved in response to low potassium.
AT4G37580	-1,933	HOOKLESS 1 (HLS1)	involved in apical hook development. putative N-acetyltransferase
AT5G62420	-1,951		NAD(P)-linked oxidoreductase superfamily protein; FUNCTIONS IN: oxidoreductase activity; INVOLVED IN: oxidation reduction; EXPRESSED IN: root; CONTAINS InterPro DOMAIN/s: Aldo/keto reductase (InterPro:IPR001395), Aldo/keto reductase subgroup (InterPro:IPR020471), Aldo/keto reductase, conserved site (InterPro:IPR018170); BEST Arabidopsis thaliana protein match is: NAD(P)-linked oxidoreductase superfamily protein (TAIR:AT2G37770.2)
AT5G14200	-1,972	ISOPROPYLMALATE DEHYDROGENASE 1 (IMD1)	The AtIMD1 is one out of 3 genes encoding the enzyme 3-isopropylmalate dehydrogenase involved in leucine biosynthesis in Arabidopsis. Its subcellular location has been targeted to plastids. Encodes methylthioalkylmalate dehydrogenase. Involved in glucosinolate biosynthesis, in methionine chain elongation. The mRNA is cell-to-cell mobile.
AT1G76800	-1,976	VACUOLAR IRON TRANSPORTER-LIKE 2 (VTL2)	The gene encodes nodulin-like2 whose transcript abundance was repressed under conditions of Fe-deficient growth.
AT3G53420	-1,982	PLASMA MEMBRANE INTRINSIC PROTEIN 2A (PIP2A)	a member of the plasma membrane intrinsic protein subfamily PIP2. localizes to the plasma membrane and exhibits water transport activity in Xenopus oocyte. expressed specifically in the vascular bundles and protein level increases slightly during leaf dev. When expressed in yeast cells can conduct hydrogen peroxide into those cells.
AT4G15390	-1,982		HXXXD-type acyl-transferase family protein; FUNCTIONS IN: transferase activity, transferring acyl groups other than amino-acyl groups, transferase activity; INVOLVED IN: biological_process unknown; LOCATED IN: cellular_component unknown; EXPRESSED IN: hypocotyl, root; CONTAINS InterPro DOMAIN/s: Transferase (InterPro:IPR003480); BEST Arabidopsis thaliana protein match is: HXXXD-type acyl-transferase family protein (TAIR:AT3G30280.1)
AT5G59680	-1,985		Leucine-rich repeat protein kinase family protein; FUNCTIONS IN: kinase activity; INVOLVED IN: protein amino acid phosphorylation; LOCATED IN: endomembrane system; EXPRESSED IN: 7 plant structures; EXPRESSED DURING: 6 growth stages; CONTAINS InterPro DOMAIN/s: Protein kinase, ATP binding site (InterPro:IPR017441), Protein kinase, catalytic domain (InterPro:IPR000719), Leucine-rich repeat (InterPro:IPR001611), Serine/threonine-protein kinase-like domain (InterPro:IPR017442), Protein kinase-like domain (InterPro:IPR011009), Serine/threonine-protein kinase, active site (InterPro:IPR008271); BEST Arabidopsis thaliana protein match is: Leucine-rich repeat protein kinase family protein (TAIR:AT5G59650.1)

AT3G62740	*-1,989*	BETA GLUCOSIDASE 7 (BGLU7)	beta glucosidase 7 (BGLU7); FUNCTIONS IN: cation binding, hydrolase activity, hydrolyzing O-glycosyl compounds, catalytic activity; INVOLVED IN: carbohydrate metabolic process; LOCATED IN: endomembrane system; EXPRESSED IN: 7 plant structures; EXPRESSED DURING: petal differentiation and expansion stage, E expanded cotyledon stage, D bilateral stage; CONTAINS InterPro DOMAIN/s: Glycoside hydrolase, family 1 (InterPro:IPR001360), Glycoside hydrolase, family 1, active site (InterPro:IPR018120), Glycoside hydrolase, catalytic core (InterPro:IPR017853), Glycoside hydrolase, subgroup, catalytic core (InterPro:IPR013781); BEST Arabidopsis thaliana protein match is: beta glucosidase 8 (TAIR:AT3G62750.1)
AT2G02000	*-1,993*	GLUTAMATE DECARBOXYLASE 3 (GAD3)	glutamate decarboxylase 3 (GAD3); FUNCTIONS IN: calmodulin binding; INVOLVED IN: carboxylic acid metabolic process, glutamate metabolic process, glutamate decarboxylation to succinate; LOCATED IN: cellular_component unknown; CONTAINS InterPro DOMAIN/s: Pyridoxal phosphate-dependent transferase, major domain (InterPro:IPR015424), Pyridoxal phosphate-dependent decarboxylase (InterPro:IPR002129), Glutamate decarboxylase (InterPro:IPR010107), Pyridoxal phosphate-dependent transferase, major region, subdomain 1 (InterPro:IPR015421); BEST Arabidopsis thaliana protein match is: glutamate decarboxylase 4 (TAIR:AT2G02010.1)
AT5G54370	*-1,998*		Late embryogenesis abundant (LEA) protein-related; FUNCTIONS IN: molecular_function unknown; INVOLVED IN: biological_process unknown; LOCATED IN: endomembrane system; EXPRESSED IN: root; CONTAINS InterPro DOMAIN/s: Root cap (InterPro:IPR009646); BEST Arabidopsis thaliana protein match is: Late embryogenesis abundant (LEA) protein-related (TAIR:AT4G27400.1)
AT5G19970	*-2,001*		unknown protein
AT2G45220	*-2,003*	PECTIN METHYLESTERASE 17 (PME17)	Plant invertase/pectin methylesterase inhibitor superfamily; FUNCTIONS IN: enzyme inhibitor activity, pectinesterase activity; INVOLVED IN: cell wall modification; LOCATED IN: membrane, plant-type cell wall; EXPRESSED IN: 9 plant structures; EXPRESSED DURING: 4 anthesis, C globular stage, petal differentiation and expansion stage; CONTAINS InterPro DOMAIN/s: Carbohydrate-binding/sugar hydrolysis domain (InterPro:IPR006633), Pectinesterase, active site (InterPro:IPR018040), Pectin lyase fold/virulence factor (InterPro:IPR011050), Pectinesterase, catalytic (InterPro:IPR000070), Pectinesterase inhibitor (InterPro:IPR006501), Pectin lyase fold (InterPro:IPR012334); BEST Arabidopsis thaliana protein match is: Plant invertase/pectin methylesterase inhibitor superfamily (TAIR:AT5G51490.1)
AT5G03640	*-2,003*		Protein kinase superfamily protein; FUNCTIONS IN: kinase activity; INVOLVED IN: protein amino acid phosphorylation, N-terminal protein myristoylation; LOCATED IN: cellular_component unknown; EXPRESSED IN: stem, inflorescence meristem, male gametophyte; CONTAINS InterPro DOMAIN/s: Protein kinase, catalytic domain (InterPro:IPR000719), Serine/threonine-protein kinase domain (InterPro:IPR002290), Serine/threonine-protein kinase-like domain (InterPro:IPR017442), Protein kinase-like domain (InterPro:IPR011009), Serine/threonine-protein kinase, active site (InterPro:IPR008271); BEST Arabidopsis thaliana protein match is: KCBP-interacting protein kinase (TAIR:AT3G52890.2)
AT5G50820	*-2,003*	NAC DOMAIN CONTAINING PROTEIN 97 (NAC097)	NAC domain containing protein 97 (NAC097); FUNCTIONS IN: sequence-specific DNA binding transcription factor activity; INVOLVED IN: multicellular organismal development, regulation of transcription; LOCATED IN: cellular_component unknown; EXPRESSED IN: 9 plant structures; EXPRESSED DURING: 6 growth stages; CONTAINS InterPro DOMAIN/s: No apical meristem (NAM) protein (InterPro:IPR003441); BEST Arabidopsis thaliana protein match is: NAC domain containing protein 83 (TAIR:AT5G13180.1)
AT1G06080	*-2,004*	DELTA 9 DESATURASE 1 (ADS1)	Encodes a protein homologous to delta 9 acyl-lipid desaturases of cyanobacteria and acyl-CoA desaturases of yeast and mammals. expression down-regulated by cold temperature. It is involved in the desaturation of VLCFAs to make monounsaturated VLCFAs.
AT1G33840	*-2,021*		Protein of unknown function (DUF567); CONTAINS InterPro DOMAIN/s: Protein of unknown function DUF567 (InterPro:IPR007612); BEST Arabidopsis thaliana protein match is: Protein of unknown function (DUF567) (TAIR:AT5G01750.2)
AT3G19710	*-2,031*	BRANCHED-CHAIN AMINOTRANSFERASE4 (BCAT4)	Belongs to the branched-chain amino acid aminotransferase gene family. Encodes a methionine-oxo-acid transaminase. Involved in the methionine chain elongation pathway that leads to the ultimate biosynthesis of methionine-derived glucosinolates.

AT5G26200	*-2,041*		Mitochondrial substrate carrier family protein; FUNCTIONS IN: binding; INVOLVED IN: transport, mitochondrial transport, transmembrane transport; LOCATED IN: mitochondrial inner membrane, membrane; EXPRESSED IN: 7 plant structures; EXPRESSED DURING: 7 growth stages; CONTAINS InterPro DOMAIN/s: Mitochondrial carrier protein (InterPro:IPR002067), Mitochondrial substrate carrier (InterPro:IPR001993), Mitochondrial substrate/solute carrier (InterPro:IPR018108); BEST Arabidopsis thaliana protein match is: Mitochondrial substrate carrier family protein (TAIR:AT1G72820.1)
AT4G15393	*-2,044*	CYTOCHROME P450, FAMILY 702, SUBFAMILY A, POLYPEPTIDE 5 (CYP702A5)	a member of the cytochrome P450 gene family. molecular function unknown.
AT4G01970	*-2,053*	STACHYOSE SYNTHASE (STS)	Encodes a a raffinose and high affinity stachyose synthase as well as a stachyose and Gol specific galactosylhydrolase enzyme activity.AtRS4 is a sequential multifunctional RafS and StaS as well as a high affinity StaS, accepting only Raf and Gol for Sta product formation. AtRS4 possesses a Sta and Gol specific galactosylhydrolase enzyme activity.
AT4G01430	*-2,061*	USUALLY MULTIPLE ACIDS MOVE IN AND OUT TRANSPORTERS 29 (UMAMIT29)	Encodes a plasma membrane-localized amino acid transporter likely involved in amino acid export in the developing seed.
AT1G73220	*-2,07*	ORGANIC CATION/CARNITINE TRANSPORTER1 (OCT1)	Encodes Organic Cation Transporter 1 (OCT1), likely to be involved in polyamine transport.
AT4G01390	*-2,07*		TRAF-like family protein; CONTAINS InterPro DOMAIN/s: TRAF-like (InterPro:IPR008974), MATH (InterPro:IPR002083); BEST Arabidopsis thaliana protein match is: TRAF-like family protein (TAIR:AT4G00780.1)
AT5G19600	*-2,07*	SULFATE TRANSPORTER 3;5 (SULTR3;5)	Encodes sulfate transporter Sultr3;5.
AT1G64780	*-2,071*	AMMONIUM TRANSPORTER 1;2 (AMT1;2)	encodes an ammonium transporter protein believed to act as a high affinity transporter. It is expressed in the root, primarily in endodermal and cortical cells, and contributes to ammonium uptake in the root.
AT2G28470	*-2,075*	BETA-GALACTOSIDASE 8 (BGAL8)	putative beta-galactosidase (BGAL8 gene)
AT4G21250	*-2,076*		Sulfite exporter TauE/SafE family protein; FUNCTIONS IN: molecular_function unknown; INVOLVED IN: biological_process unknown; LOCATED IN: endomembrane system, integral to membrane; CONTAINS InterPro DOMAIN/s: Protein of unknown function DUF81 (InterPro:IPR002781); BEST Arabidopsis thaliana protein match is: Sulfite exporter TauE/SafE family protein (TAIR:AT4G21260.1)
AT1G08100	*-2,078*	NITRATE TRANSPORTER 2.2 (NRT2.2)	Encodes a high-affinity nitrate transporter.
AT5G24860	*-2,079*	FLOWERING PROMOTING FACTOR 1 (FPF1)	encodes a small protein of 12.6 kDa that regulates flowering and is involved in gibberellin signalling pathway. It is expressed in apical meristems immediately after the photoperiodic induction of flowering. Genetic interactions with flowering time and floral organ identity genes suggest that this gene may be involved in modulating the competence to flower. There are two other genes similar to FPF1, FLP1 (At4g31380) and FLP2 (no locus name yet, on BAC F8F16 on chr 4). This is so far a plant-specific gene and is only found in long-day mustard, arabidopsis, and rice.
AT4G08290	*-2,088*	USUALLY MULTIPLE ACIDS MOVE IN AND OUT TRANSPORTERS 20 (UMAMIT20)	nodulin MtN21-like transporter family protein
AT2G41480	*-2,107*	PEROXIDASE 25 (PRX25)	Encodes a cationic cell-wall-bound peroxidase homolog that is involved in the lignification of cell walls.
AT1G77760	*-2,11*	NITRATE REDUCTASE 1 (NIA1)	Encodes the cytosolic minor isoform of nitrate reductase (NR). Involved in the first step of nitrate assimilation, it contributes about 15% of the nitrate reductase activity in shoots. Similar to molybdopterin oxidoreductases at the N-terminus, and to FAD/NAD-binding cytochrome reductases at the C-terminus. Cofactors: FAD, heme iron (cytochrome B-557), and molybdenum-pterin.

AT1G16400	*-2,114*	CYTOCHROME P450, FAMILY 79, SUBFAMILY F, POLYPEPTIDE 2 (CYP79F2)	Encodes cytochrome P450 CYP79F2.
AT5G38200	*-2,134*		Class I glutamine amidotransferase-like superfamily protein; FUNCTIONS IN: hydrolase activity; INVOLVED IN: glutamine metabolic process; LOCATED IN: cellular_component unknown; CONTAINS InterPro DOMAIN/s: Peptidase C26 (InterPro:IPR011697), Glutamine amidotransferase type 1 (InterPro:IPR017926); BEST Arabidopsis thaliana protein match is: Class I glutamine amidotransferase-like superfamily protein (TAIR:AT1G66860.1)
AT4G39770	*-2,142*	TREHALOSE-6-PHOSPHATE PHOSPHATASE H (TPPH)	Haloacid dehalogenase-like hydrolase (HAD) superfamily protein; FUNCTIONS IN: catalytic activity, trehalose-phosphatase activity; INVOLVED IN: trehalose biosynthetic process, metabolic process; LOCATED IN: cellular_component unknown; EXPRESSED IN: 10 plant structures; EXPRESSED DURING: 8 growth stages; CONTAINS InterPro DOMAIN/s: HAD-superfamily hydrolase, subfamily IIB (InterPro:IPR006379), Trehalose-phosphatase (InterPro:IPR003337); BEST Arabidopsis thaliana protein match is: Haloacid dehalogenase-like hydrolase (HAD) superfamily protein (TAIR:AT2G22190.1)
AT4G15160	*-2,145*		Bifunctional inhibitor/lipid-transfer protein/seed storage 2S albumin superfamily protein; FUNCTIONS IN: lipid binding; INVOLVED IN: lipid transport; LOCATED IN: endomembrane system; EXPRESSED IN: 18 plant structures; EXPRESSED DURING: 9 growth stages; CONTAINS InterPro DOMAIN/s: Bifunctional inhibitor/plant lipid transfer protein/seed storage (InterPro:IPR016140), Plant lipid transfer protein/hydrophobic protein, helical domain (InterPro:IPR013770); BEST Arabidopsis thaliana protein match is: cell wall-plasma membrane linker protein (TAIR:AT3G22120.1)
AT2G22330	*-2,159*	CYTOCHROME P450, FAMILY 79, SUBFAMILY B, POLYPEPTIDE 3 (CYP79B3)	Encodes a cytochrome P450. Involved in tryptophan metabolism. Converts Trp to indole-3-acetaldoxime (IAOx), a precursor to IAA and indole glucosinolates. The mRNA is cell-to-cell mobile.
AT4G25960	*-2,165*	ATP-BINDING CASSETTE B2 (ABCB2)	P-glycoprotein 2 (PGP2); FUNCTIONS IN: ATPase activity, coupled to transmembrane movement of substances; INVOLVED IN: transport, transmembrane transport; LOCATED IN: membrane; EXPRESSED IN: 22 plant structures; EXPRESSED DURING: 13 growth stages; CONTAINS InterPro DOMAIN/s: ATPase, AAA+ type, core (InterPro:IPR003593), ABC transporter-like (InterPro:IPR003439), ABC transporter, transmembrane domain, type 1 (InterPro:IPR011527), ABC transporter integral membrane type 1 (InterPro:IPR017940), ABC transporter, transmembrane domain (InterPro:IPR001140), ABC transporter, conserved site (InterPro:IPR017871); BEST Arabidopsis thaliana protein match is: P-glycoprotein 10 (TAIR:AT1G10680.1)
AT5G43180	*-2,167*		Protein of unknown function, DUF599; FUNCTIONS IN: molecular_function unknown; INVOLVED IN: biological_process unknown; LOCATED IN: endomembrane system; EXPRESSED IN: 10 plant structures; EXPRESSED DURING: LP.04 four leaves visible, 4 anthesis, petal differentiation and expansion stage; CONTAINS InterPro DOMAIN/s: Protein of unknown function DUF599 (InterPro:IPR006747); BEST Arabidopsis thaliana protein match is: Protein of unknown function, DUF599 (TAIR:AT4G31330.1)
AT1G11600	*-2,191*	CYTOCHROME P450, FAMILY 77, SUBFAMILY B, POLYPEPTIDE 1 (CYP77B1)	member of CYP77B
AT1G30840	*-2,192*	PURINE PERMEASE 4 (PUP4)	Member of a family of proteins related to PUP1, a purine transporter. May be involved in the transport of purine and purine derivatives such as cytokinins, across the plasma membrane.
AT4G24670	*-2,192*	TRYPTOPHAN AMINOTRANSFERASE RELATED 2 (TAR2)	Encodes a protein with similarity to the TAA1 trytophan aminotransferase involved in IAA biosynthesis. Double mutant analyses suggest that this protein is involved in regulating many aspects of plant growth and development from embryogenesis to flower formation and plays a role in ethylene-mediated signaling.TAR2 is required for reprogramming root architecture in response to low nitrogen conditions.
AT5G19730	*-2,193*		Pectin lyase-like superfamily protein; FUNCTIONS IN: pectinesterase activity; INVOLVED IN: cell wall modification; LOCATED IN: cell wall, plant-type cell wall, cytoplasm; EXPRESSED IN: 17 plant structures; EXPRESSED DURING: 8 growth stages; CONTAINS InterPro DOMAIN/s: Pectinesterase, active site (InterPro:IPR018040), Pectin lyase fold/virulence factor (InterPro:IPR011050), Pectinesterase, catalytic (InterPro:IPR000070), Pectin lyase fold (InterPro:IPR012334); BEST Arabidopsis thaliana protein match is: Pectin lyase-like superfamily protein (TAIR:AT2G36710.1)

AT3G25820	*-2,202*	TERPENE SYNTHASE-LIKE SEQUENCE-1,8-CINEOLE (TPS-CIN)	Encodes the monoterpene 1,8-cineole synthase, atTPS-Cin. This polypeptide was also shown to synthesize other monoterpenes albeit in minor quantities. The same polypeptide is encoded at two different loci, the result of gene duplication: at3g25820 and at3g25830.
AT5G43520	*-2,204*		Cysteine/Histidine-rich C1 domain family protein; CONTAINS InterPro DOMAIN/s: DC1 (InterPro:IPR004146), C1-like (InterPro:IPR011424); BEST Arabidopsis thaliana protein match is: Cysteine/Histidine-rich C1 domain family protein (TAIR:AT2G44380.1)
AT5G55250	*-2,209*	IAA CARBOXYLMETHYLTRANSFERASE 1 (IAMT1)	Encodes an enzyme which specifically converts IAA to its methyl ester form MeIIAA. This gene belongs to the family of carboxyl methyltransferases whose members catalyze the transfer of the methyl group from S-adenosyl-L-methionine to carboxylic acid-containing substrates to form small molecule methyl esters. Expression of TCP genes is downregulated in mutant iamt1-D.
AT2G13810	*-2,21*	AGD2-LIKE DEFENSE RESPONSE PROTEIN 1 (ALD1)	AGD2-like defense response protein 1 (ALD1); FUNCTIONS IN: transferase activity, transferring nitrogenous groups, pyridoxal phosphate binding, transaminase activity, catalytic activity; INVOLVED IN: asparagine catabolic process, biosynthetic process, glutamate catabolic process to oxaloacetate, aspartate transamidation; LOCATED IN: chloroplast; EXPRESSED IN: sepal, root, stamen; EXPRESSED DURING: 4 anthesis; CONTAINS InterPro DOMAIN/s: LL-diaminopimelate aminotransferase, plant-related (InterPro:IPR019942), Aminotransferase, class I/classII (InterPro:IPR004839), Pyridoxal phosphate-dependent transferase, major domain (InterPro:IPR015424), Pyridoxal phosphate-dependent transferase, major region, subdomain 1 (InterPro:IPR015421), Pyridoxal phosphate-dependent transferase, major region, subdomain 2 (InterPro:IPR015422); BEST Arabidopsis thaliana protein match is: Pyridoxal phosphate (PLP)-dependent transferases superfamily protein (TAIR:AT4G33680.1)
AT5G37990	*-2,214*	(CIMT1)	S-adenosyl-L-methionine-dependent methyltransferases superfamily protein; FUNCTIONS IN: S-adenosylmethionine-dependent methyltransferase activity, methyltransferase activity; INVOLVED IN: biological_process unknown; LOCATED IN: endomembrane system; EXPRESSED IN: hypocotyl, root; CONTAINS InterPro DOMAIN/s: SAM dependent carboxyl methyltransferase (InterPro:IPR005299); BEST Arabidopsis thaliana protein match is: S-adenosyl-L-methionine-dependent methyltransferases superfamily protein (TAIR:AT5G37970.1)
AT2G01900	*-2,216*	INOSITOL POLYPHOSPHATE PHOSPHATIDYLINOSITOL 5-PHOSPHATASE9 (T5PTASE9)	Encodes an inositol polyphosphate phosphatidylinositol 5-phosphatase that is expressed in roots and is involved in mediating salt tolerance through endocytosis.
AT3G08770	*-2,219*	LIPID TRANSFER PROTEIN 6 (LTP6)	Predicted to encode a PR (pathogenesis-related) protein. Belongs to the lipid transfer protein (PR-14) family with the following members: At2g38540/LTP1, At2g38530/LTP2, At5g59320/LTP3, At5g59310/LTP4, At3g51600/LTP5, At3g08770/LTP6, At2g15050/LTP7, At2g18370/LTP8, At2g15325/LTP9, At5g01870/LTP10, At4g33355/LTP11, At3g51590/LTP12, At5g44265/LTP13, At5g62065/LTP14, At4g08530/LTP15.
AT3G51350	*-2,243*		Eukaryotic aspartyl protease family protein; FUNCTIONS IN: aspartic-type endopeptidase activity; INVOLVED IN: proteolysis; LOCATED IN: anchored to membrane; CONTAINS InterPro DOMAIN/s: Peptidase aspartic, catalytic (InterPro:IPR009007), Peptidase aspartic (InterPro:IPR021109), Peptidase A1 (InterPro:IPR001461), Peptidase aspartic, active site (InterPro:IPR001969); BEST Arabidopsis thaliana protein match is: Eukaryotic aspartyl protease family protein (TAIR:AT3G51330.1)
AT1G30370	*-2,244*	DAD1-LIKE ACYLHYDROLASE (DLAH)	Encodes a mitochondria-localized class III phospholipase A1 that plays a role in seed viability.
AT5G10230	*-2,257*	ANNEXIN 7 (ANNAT7)	Encodes a calcium-binding protein annexin (AnnAt7).
AT1G15460	*-2,285*	REQUIRES HIGH BORON 4 (BOR4)	Encodes a efflux-type boron transporter. Over-expression improved plant growth under B toxic conditions.
AT5G36870	*-2,286*	GLUCAN SYNTHASE-LIKE 9 (GSL09)	encodes a gene similar to callose synthase
AT4G17785	*-2,291*	MYB DOMAIN PROTEIN 39 (MYB39)	Encodes a putative transcription factor (MYB39).

AT5G43770	*-2,31*		proline-rich family protein; FUNCTIONS IN: molecular_function unknown; INVOLVED IN: biological_process unknown; LOCATED IN: chloroplast
AT5G22390	*-2,317*		Protein of unknown function (DUF3049); CONTAINS InterPro DOMAIN/s: Protein of unknown function DUF3049 (InterPro:IPR021410); BEST Arabidopsis thaliana protein match is: Protein of unknown function (DUF3049) (TAIR:AT1G54740.1)
AT4G08570	*-2,321*		Heavy metal transport/detoxification superfamily protein ; FUNCTIONS IN: metal ion binding; INVOLVED IN: metal ion transport; LOCATED IN: cellular_component unknown; CONTAINS InterPro DOMAIN/s: Heavy metal transport/detoxification protein (InterPro:IPR006121), Heavy-metal-associated, conserved site (InterPro:IPR017969); BEST Arabidopsis thaliana protein match is: Heavy metal transport/detoxification superfamily protein (TAIR:AT1G22990.1)
AT4G39330	*-2,322*	CINNAMYL ALCOHOL DEHYDROGENASE 9 (CAD9)	cinnamyl alcohol dehydrogenase 9 (CAD9); FUNCTIONS IN: oxidoreductase activity, zinc ion binding; INVOLVED IN: oxidation reduction; LOCATED IN: apoplast; EXPRESSED IN: 25 plant structures; EXPRESSED DURING: 15 growth stages; CONTAINS InterPro DOMAIN/s: GroES-like (InterPro:IPR011032), Polyketide synthase, enoylreductase (InterPro:IPR020843), Alcohol dehydrogenase, zinc-containing, conserved site (InterPro:IPR002328), Alcohol dehydrogenase GroES-like (InterPro:IPR013154), Alcohol dehydrogenase, C-terminal (InterPro:IPR013149), Alcohol dehydrogenase superfamily, zinc-containing (InterPro:IPR002085); BEST Arabidopsis thaliana protein match is: cinnamyl alcohol dehydrogenase homolog 2 (TAIR:AT2G21730.1)
AT4G31710	*-2,362*	GLUTAMATE RECEPTOR 2.4 (GLR2.4)	member of Putative ligand-gated ion channel subunit family
AT2G21210	*-2,396*	SMALL AUXIN UPREGULATED RNA 6 (SAUR6)	Putative auxin-regulated protein whose expression is downregulated in response to chitin oligomers.
AT3G49760	*-2,406*	BASIC LEUCINE-ZIPPER 5 (bZIP5)	basic leucine-zipper 5 (bZIP5); FUNCTIONS IN: DNA binding, sequence-specific DNA binding transcription factor activity; INVOLVED IN: regulation of transcription, DNA-dependent; LOCATED IN: nucleus; EXPRESSED IN: root stele, pericycle, root, primary root elongation zone, primary root differentiation zone; CONTAINS InterPro DOMAIN/s: Basic-leucine zipper (bZIP) transcription factor (InterPro:IPR004827), Basic leucine zipper (InterPro:IPR011700); BEST Arabidopsis thaliana protein match is: basic leucine-zipper 6 (TAIR:AT2G22850.2)
AT4G20230	*-2,434*	TERPENE SYNTHASE 30 (TPS30)	Terpenoid cyclases/Protein prenyltransferases superfamily protein; FUNCTIONS IN: lyase activity, magnesium ion binding; INVOLVED IN: metabolic process; EXPRESSED IN: hypocotyl, root; CONTAINS InterPro DOMAIN/s: Terpene synthase, metal-binding domain (InterPro:IPR005630), Terpenoid synthase (InterPro:IPR008949), Terpenoid cylases/protein prenyltransferase alpha-alpha toroid (InterPro:IPR008930), Terpene synthase-like (InterPro:IPR001906); BEST Arabidopsis thaliana protein match is: Terpenoid cyclases/Protein prenyltransferases superfamily protein (TAIR:AT4G20200.1)
AT2G47560	*-2,458*		RING/U-box superfamily protein; FUNCTIONS IN: zinc ion binding; EXPRESSED IN: root, pedicel; EXPRESSED DURING: 4 anthesis; CONTAINS InterPro DOMAIN/s: Zinc finger, RING-type (InterPro:IPR001841), Zinc finger, C3HC4 RING-type (InterPro:IPR018957); BEST Arabidopsis thaliana protein match is: AtL5 (TAIR:AT3G62690.1)
AT3G02020	*-2,461*	ASPARTATE KINASE 3 (AK3)	encodes a monofunctional aspartate kinase
AT1G61130	*-2,466*	SERINE CARBOXYPEPTIDASE-LIKE 32 (SCPL32)	serine carboxypeptidase-like 32 (SCPL32); FUNCTIONS IN: serine-type carboxypeptidase activity; INVOLVED IN: proteolysis; LOCATED IN: endomembrane system; EXPRESSED IN: male gametophyte, root, flower; EXPRESSED DURING: 4 anthesis; CONTAINS InterPro DOMAIN/s: Peptidase S10, serine carboxypeptidase (InterPro:IPR001563), Peptidase S10, serine carboxypeptidase, active site (InterPro:IPR018202); BEST Arabidopsis thaliana protein match is: serine carboxypeptidase-like 31 (TAIR:AT1G11080.2)
AT1G62280	*-2,484*	SLAC1 HOMOLOGUE 1 (SLAH1)	Encodes a protein with ten predicted transmembrane helices. The SLAH1 protein has similarity to the SLAC1 protein involved in ion homeostasis in guard cells. Although it is not expressed in guard cells, it can complement a slac1-2 mutant suggesting that it performs a similar function. SLAH1:GFP localizes to the plasma membrane.
AT4G20070	*-2,488*	ALLANTOATE AMIDOHYDROLASE (AAH)	The gene encoding Arabidopsis thaliana Allantoate Amidohydrolase (AtAAH)which catalyzes the allantoate deiminase reaction (EC 3.5.3.9)is expressed in all parts of the plant being consistent with a function in purine turnover in Arabidopsis. The mRNA is cell-to-cell mobile.

AT3G25190	*-2,496*	VACUOLAR IRON TRANSPORTER-LIKE 5 (VTL5)	The gene encodes nodulin-like21 whose transcript abundance was repressed under conditions of Fe-deficient growth.
AT1G71870	*-2,528*	(BIGE1A)	MATE efflux family protein; FUNCTIONS IN: antiporter activity, drug transmembrane transporter activity, transporter activity; INVOLVED IN: drug transmembrane transport, transmembrane transport; LOCATED IN: membrane; EXPRESSED IN: 16 plant structures; EXPRESSED DURING: 6 growth stages; CONTAINS InterPro DOMAIN/s: Multi antimicrobial extrusion protein MatE (InterPro:IPR002528); BEST Arabidopsis thaliana protein match is: MATE efflux family protein (TAIR:AT4G29140.1)
AT1G49470	*-2,536*		Family of unknown function (DUF716) ; FUNCTIONS IN: molecular_function unknown; INVOLVED IN: biological_process unknown; LOCATED IN: endomembrane system; EXPRESSED IN: 20 plant structures; EXPRESSED DURING: 10 growth stages; CONTAINS InterPro DOMAIN/s: Protein of unknown function DUF716 (InterPro:IPR006904); BEST Arabidopsis thaliana protein match is: Family of unknown function (DUF716) (TAIR:AT1G55230.1)
AT2G22190	*-2,539*	TREHALOSE-6-PHOSPHATE PHOSPHATASE E (TPPE)	Haloacid dehalogenase-like hydrolase (HAD) superfamily protein; FUNCTIONS IN: catalytic activity, trehalose-phosphatase activity; INVOLVED IN: trehalose biosynthetic process, metabolic process; LOCATED IN: cellular_component unknown; EXPRESSED IN: 23 plant structures; EXPRESSED DURING: 12 growth stages; CONTAINS InterPro DOMAIN/s: HAD-superfamily hydrolase, subfamily IIB (InterPro:IPR006379), Trehalose-phosphatase (InterPro:IPR003337); BEST Arabidopsis thaliana protein match is: Haloacid dehalogenase-like hydrolase (HAD) superfamily protein (TAIR:AT4G39770.1)
AT1G68740	*-2,561*	(PHO1;H1)	Encodes PHO1;H1, a member of the PHO1 family. Involved in inorganic phosphate (Pi) transport and homeostasis. Complements pho1 mutation. Its expression is responsive to both phosphate (Pi) and phosphite (Phi) in shoots.
AT1G15380	*-2,572*	GLYOXYLASE I 4 (GLYI4)	Lactoylglutathione lyase / glyoxalase I family protein; CONTAINS InterPro DOMAIN/s: Glyoxalase/bleomycin resistance protein/dioxygenase (InterPro:IPR004360); BEST Arabidopsis thaliana protein match is: Lactoylglutathione lyase / glyoxalase I family protein (TAIR:AT1G80160.1)
AT4G13770	*-2,579*	CYTOCHROME P450, FAMILY 83, SUBFAMILY A, POLYPEPTIDE 1 (CYP83A1)	Encodes a cytochrome p450 enzyme that catalyzes the initial conversion of aldoximes to thiohydroximates in the synthesis of glucosinolates not derived from tryptophan. Also has a role in auxin homeostasis.
AT1G48670	*-2,592*		auxin-responsive GH3 family protein; CONTAINS InterPro DOMAIN/s: GH3 auxin-responsive promoter (InterPro:IPR004993); BEST Arabidopsis thaliana protein match is: Auxin-responsive GH3 family protein (TAIR:AT1G48660.1)
AT1G44800	*-2,634*	SILIQUES ARE RED 1 (SIAR1)	Encodes Siliques Are Red 1 (SIAR1). Functions as a bidirectional amino acid transporter that is crucial for the amino acid homeostasis of siliques. Member of nodulin MtN21-like transporter family.
AT5G13930	*-2,676*	TRANSPARENT TESTA 4 (TT4)	Encodes chalcone synthase (CHS), a key enzyme involved in the biosynthesis of flavonoids. Required for the accumulation of purple anthocyanins in leaves and stems. Also involved in the regulation of auxin transport and the modulation of root gravitropism. The mRNA is cell-to-cell mobile.
AT4G36060	*-2,829*	BASIC HELIX-LOOP-HELIX 11 (bHLH11)	basic Helix-Loop-Helix 11 (bHLH11); FUNCTIONS IN: DNA binding, sequence-specific DNA binding transcription factor activity; INVOLVED IN: regulation of transcription; LOCATED IN: nucleus, chloroplast, cytoplasm; CONTAINS InterPro DOMAIN/s: Helix-loop-helix DNA-binding domain (InterPro:IPR001092), Helix-loop-helix DNA-binding (InterPro:IPR011598); BEST Arabidopsis thaliana protein match is: basic helix-loop-helix (bHLH) DNA-binding superfamily protein (TAIR:AT3G19860.1)
AT2G37460	*-2,857*	USUALLY MULTIPLE ACIDS MOVE IN AND OUT TRANSPORTERS 12 (UMAMIT12)	nodulin MtN21-like transporter family protein
AT5G59320	*-2,89*	LIPID TRANSFER PROTEIN 3 (LTP3)	Predicted to encode a PR (pathogenesis-related) protein. Belongs to the lipid transfer protein (PR-14) family with the following members: At2g38540/LTP1, At2g38530/LTP2, At5g59320/LTP3, At5g59310/LTP4, At3g51600/LTP5, At3g08770/LTP6, At2g15050/LTP7, At2g18370/LTP8, At2g15325/LTP9, At5g01870/LTP10, At4g33355/LTP11, At3g51590/LTP12, At5g44265/LTP13, At5g62065/LTP14, At4g08530/LTP15. The mRNA is cell-to-cell mobile.
AT5G58360	*-3,009*	OVATE FAMILY PROTEIN 3 (OFP3)	ovate family protein 3 (OFP3); CONTAINS InterPro DOMAIN/s: Protein of unknown function DUF623 (InterPro:IPR006458); BEST Arabidopsis thaliana protein match is: ovate family protein 4 (TAIR:AT1G06920.1)

AT1G14160	*-3,047*	CASP-LIKE PROTEIN 1A1 (CASPL1A1)	Uncharacterised protein family (UPF0497); CONTAINS InterPro DOMAIN/s: Uncharacterised protein family UPF0497, trans-membrane plant (InterPro:IPR006702), Uncharacterised protein family UPF0497, trans-membrane plant subgroup (InterPro:IPR006459); BEST Arabidopsis thaliana protein match is: Uncharacterised protein family (UPF0497) (TAIR:AT5G15290.1)
AT1G04180	*-3,12*	YUCCA 9 (YUC9)	YUCCA 9 (YUC9); FUNCTIONS IN: NADP or NADPH binding, oxidoreductase activity, monooxygenase activity, FAD binding, flavin-containing monooxygenase activity; INVOLVED IN: oxidation reduction; EXPRESSED IN: root; CONTAINS InterPro DOMAIN/s: Pyridine nucleotide-disulphide oxidoreductase, class-II (InterPro:IPR000103), FAD-dependent pyridine nucleotide-disulphide oxidoreductase (InterPro:IPR013027), Flavin-containing monooxygenase-like (InterPro:IPR020946); BEST Arabidopsis thaliana protein match is: Flavin-binding monooxygenase family protein (TAIR:AT5G43890.1)
AT4G08300	*-3,15*	USUALLY MULTIPLE ACIDS MOVE IN AND OUT TRANSPORTERS 17 (UMAMIT17)	nodulin MtN21-like transporter family protein
AT1G32450	*-3,232*	NRT1/ PTR FAMILY 7.3 (NPF7.3)	Transmembrane nitrate transporter. Involved in xylem transport of nitrate from root to shoot. Induced in response to high and low concentrations of nitrate. Not involved in nitrate uptake. Expressed in root pericycle cells.
AT1G11080	*-3,313*	SERINE CARBOXYPEPTIDASE-LIKE 31 (scpl31)	serine carboxypeptidase-like 31 (scpl31); FUNCTIONS IN: serine-type carboxypeptidase activity; INVOLVED IN: proteolysis; LOCATED IN: endomembrane system; EXPRESSED IN: 17 plant structures; EXPRESSED DURING: 4 anthesis, C globular stage, petal differentiation and expansion stage, E expanded cotyledon stage, D bilateral stage; CONTAINS InterPro DOMAIN/s: Peptidase S10, serine carboxypeptidase (InterPro:IPR001563), Peptidase S10, serine carboxypeptidase, active site (InterPro:IPR018202); BEST Arabidopsis thaliana protein match is: serine carboxypeptidase-like 30 (TAIR:AT4G15100.1).
AT3G01260	*-3,382*		Galactose mutarotase-like superfamily protein; FUNCTIONS IN: carbohydrate binding, isomerase activity, aldose 1-epimerase activity, catalytic activity; INVOLVED IN: galactose metabolic process, hexose metabolic process, carbohydrate metabolic process; LOCATED IN: endomembrane system; EXPRESSED IN: root; CONTAINS InterPro DOMAIN/s: Aldose 1-epimerase (InterPro:IPR008183), Glycoside hydrolase-type carbohydrate-binding (InterPro:IPR011013), Aldose 1-epimerase, subgroup (InterPro:IPR015443), Glycoside hydrolase-type carbohydrate-binding, subgroup (InterPro:IPR014718); BEST Arabidopsis thaliana protein match is: Galactose mutarotase-like superfamily protein (TAIR:AT5G15140.1)
AT2G28780	*-3,413*		unknown protein; INVOLVED IN: biological_process unknown; LOCATED IN: mitochondrion; EXPRESSED IN: inflorescence meristem, root, flower; EXPRESSED DURING: petal differentiation and expansion stage; CONTAINS InterPro DOMAIN/s: Protein of unknown function DUF939, bacterial (InterPro:IPR010343); BEST Arabidopsis thaliana protein match is: unknown protein (TAIR:AT3G09450.1)
AT1G80340	*-3,838*	GIBBERELLIN 3-OXIDASE 2 (GA3OX2)	Encodes a protein with gibberellin 3 β-hydroxylase activity. The protein was heterologously expressed in E. coli and shown to catalyze the hydroxylation of both GA9 and GA20.
AT4G23496	*-4,141*	SPIRAL1-LIKE5 (SP1L5)	Belongs to a six-member gene family in Arabidopsis; all members share high sequence similarity in amino- and carboxy-terminal regions. Regulates cortical microtubule organization. Mutant plants exhibit altered patterns of root, leaf and petal growth as a result of defective anisotropic cell expansion.
AT5G01740	*-4,315*		Nuclear transport factor 2 (NTF2) family protein; CONTAINS InterPro DOMAIN/s: Wound-induced protein, Wun1 (InterPro:IPR009798); BEST Arabidopsis thaliana protein match is: senescence associated gene 20 (TAIR:AT3G10985.1)
