# Supplementary material for: The Role of Programmed Cell Death Regulator LSD1 in Nematode-Induced Syncytium Formation
Source: Front Plant Sci. 2018 Mar 19;9:314. doi: 10.3389/fpls.2018.00314 (PMC5868158; doi:10.3389/fpls.2018.00314)

**Supplementary Figure 1** – Mapman analysis on Col0 differentially expressed genes (DEGs) involved in biotic stress pathway (*Thimm et al., 2004*).

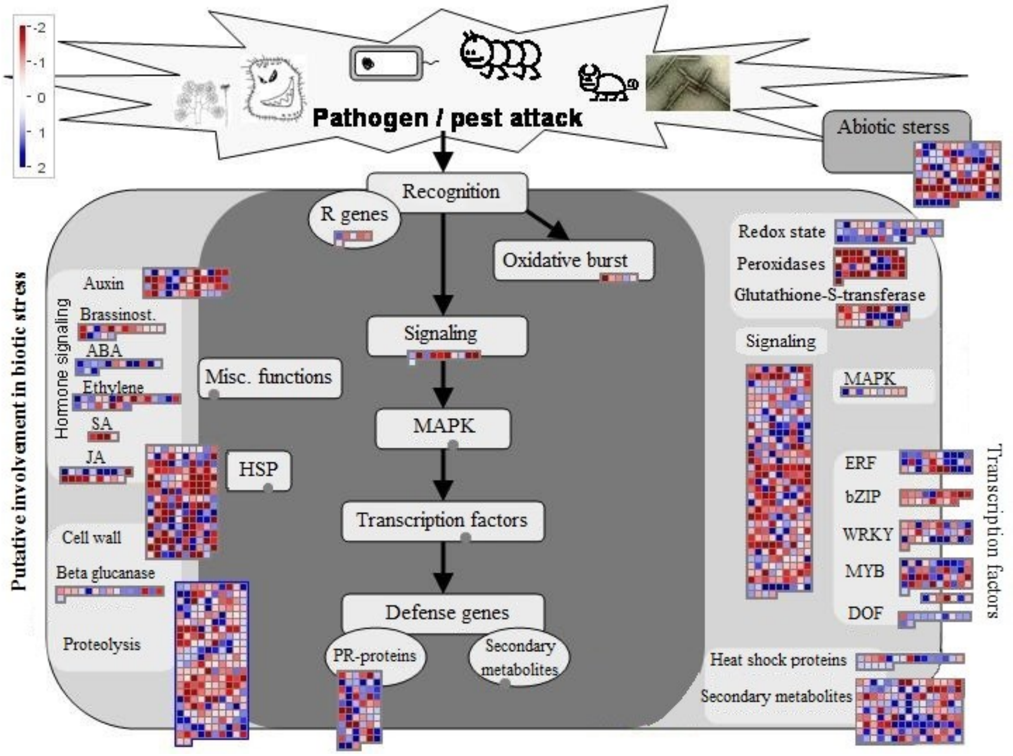


**Supplementary Figure 2** – Mapman analysis on *lsd1(Col0)* differentially expressed genes (DEGs) involved in biotic stress pathway (*Thimm et al., 2004*).

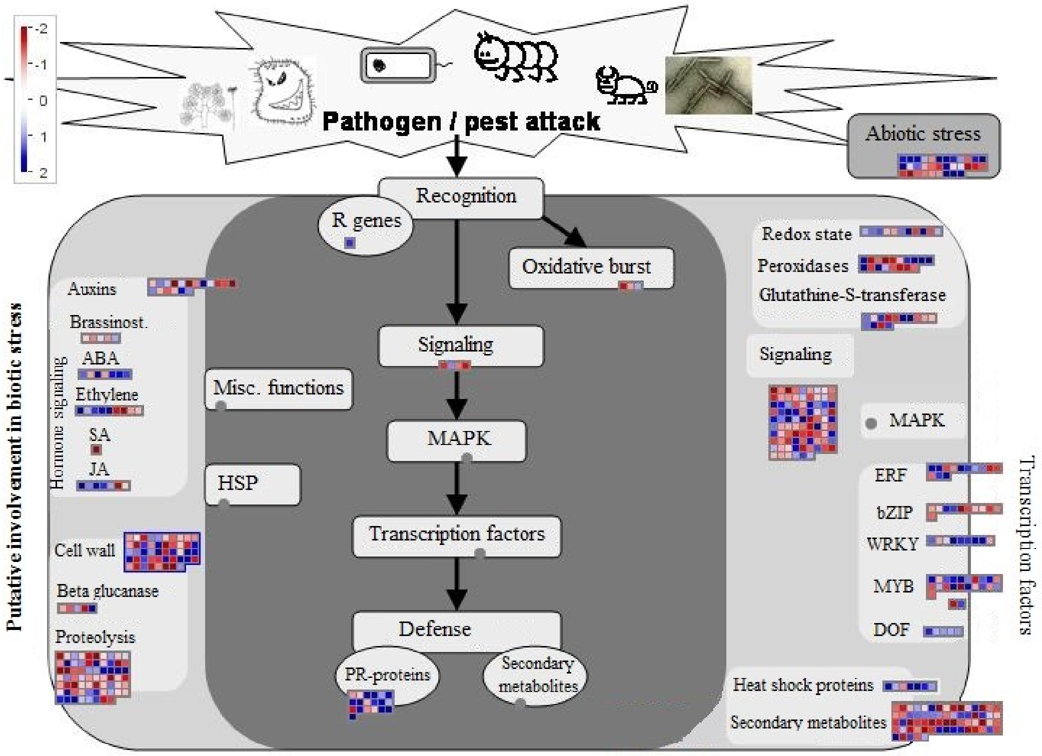

**Supplementary Figure 3** – Gene Ontology enRIchment anaLysis and visuaLizAtion tool (Gorilla; *Eden et al. 2009*) DEGs from Col0 involved in (A) biological processes, (B) molecular functioning, and responsible for (C) cellular components. DEGs from *lsd1(Col0)* involved in (D) biological processes, (E) molecular functioning, and responsible for (F) cellular components.

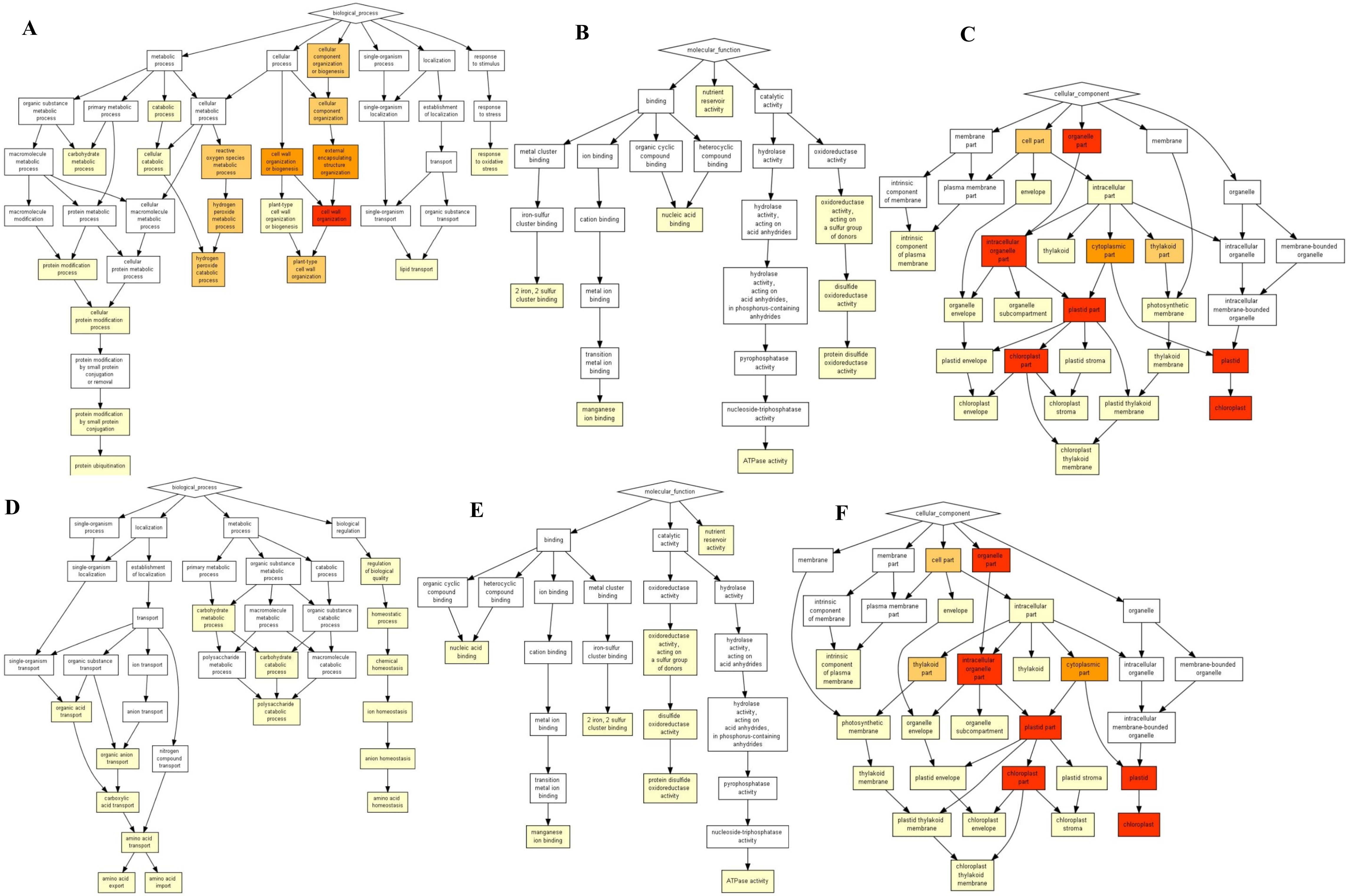

**Supplementary Figure 4** – Growth dynamic of *H. schahtii* on Col0 and *lsd1(Col0)*. Data represent 2 independent experiments – 10 plants per genotype (means ± SEM). Data were analyzed using t-test ($p < 0,05$). Asterisks indicate $p < 0,05$ compared to wild-type.

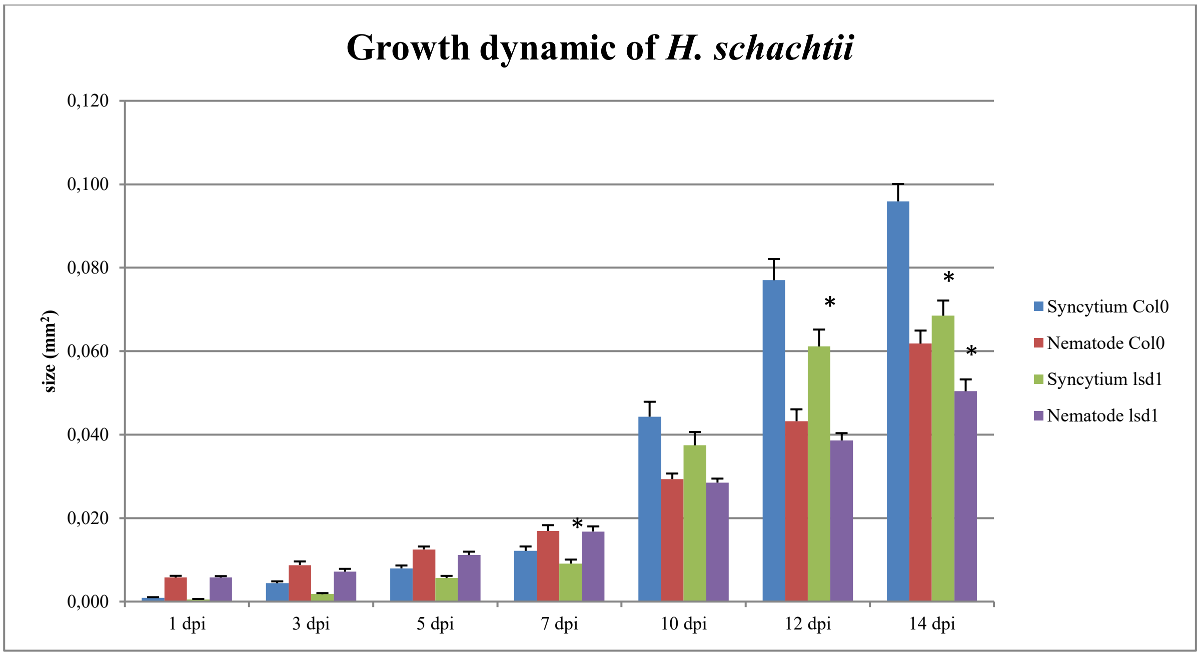

**Supplementary Figure 5** – List of primers used in this study

| Primer list | | |
|---|---|---|
| **LP lol2** | *Forward* | TGGAACAATCTTGGAATGCTC |
| **RP lol2** | *Reverse* | TGACAAGTTGGTTCTTCACCC |
| **Sail LB3** | *Forward* | TAGCATCTGAATTTCATAACCAATCTCGATACA |
| **Act2.Fwd** | *Forward* | TTCACCACAACAGCAGAGCGGG |
| **Act2.Rev** | *Reverse* | CGTGATGACTTGCCCATCGGGT |
| **RbohD_Fw** | *Forward* | CGATGAAAATGAGACGAGGCA |
| **RbohD_Rev** | *Reverse* | TCGTCGGCGAATCTTGCGTT |
| **MC1_Fwd** | *Forward* | TCCACATGGTGTCAAGCTCC |
| **MC1_Rev** | *Reverse* | TGCCCAGCTCTGTTCATTCT |
| **MC2_Fwd** | *Forward* | CAGAAGCGAGCGGTGATAGT |
| **MC2_Rev** | *Reverse* | TCCGCTTCTTCTTCGGTGAG |
| **MC4_Fwd** | *Forward* | GCGACATGAATCTGATTACTGATGA |
| **MC4_Rev** | *Reverse* | ATCTGCTCCTTGGCTTCGTC |
| **MC6_Fwd** | *Forward* | TGAACCGGCTAAATCGGGTG |
| **MC6_Rev** | *Reverse* | CAAGATCCCTGAAATCATCATCGGT |
| **MC9_Fwd** | *Forward* | TCCAGCAACATATCTCCGGC |
| **MC9_Rev** | *Reverse* | TCAAGTGGTCAAGGACTGCC |
| **AtTIR_Fwd** | *Forward* | AAAGTTCTTAAATGGAGGCAAGCA |
| **AtTIR_Rev** | *Reverse* | AGCTTCGAGTCATCATCACCTGA |
| **AtLRR_Fwd** | *Forward* | CTGCTCTTCTCTTAAATTGTTAGTTTGTCTC |
| **AtLRR_Rev** | *Reverse* | GCTTATTAGCCTCATGCTTTAAAATCTTGA |
| **CAT3_Fwd** | *Forward* | ACACCAGAGAGGGAAACTTTGATCT |
| **CAT3_Rev** | *Reverse* | TCCCATCACGGATGAAGAACA |
| **EDR10_Fwd** | *Forward* | AGCTCTTCTTCCTCTTCGAGTGATG |
| **EDR10_Rev** | *Reverse* | CCACTGTTTTCACATGATCTCCTTC |
| **PDF1.2_Fwd** | *Forward* | CGGCAATGGTGGAAGCACAGAA |
| **PDF1.2_Rev** | *Reverse* | TGCATGATCCATGTTTGGCTCC |

**Supplementary Figure 6 –** Comparison of expression levels of differentially expressed genes quantified by qRT-PCR and RNA-seq analysis. The genes *metacaspases* (*MC1*,*MC2, MC4, MC6 and MC9)*, ROS signaling *respiratory burst oxidase homolog D* (*RBOH D*), *catalase 3* (*CAT3*), two R-genes *AtTIR* and *AtLRR* and stress related markers *PDF1.2* and *ERD10* were selected for analysis.

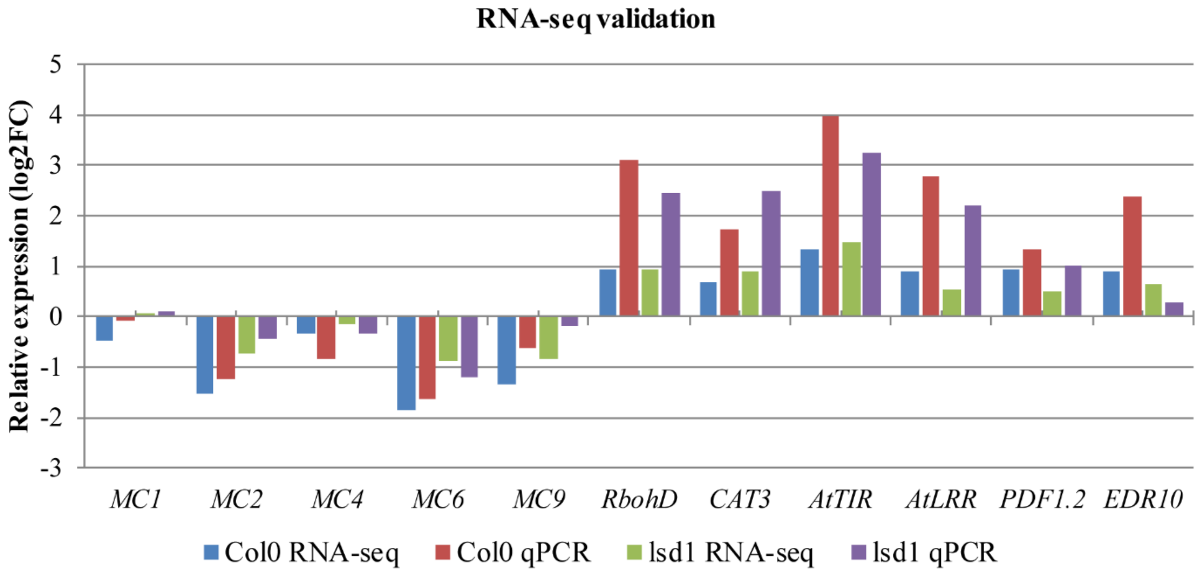

**Supplementary Figure 7** – The nematode induced changes in jasmonic acid (JA), salicylic acid (SA), ethylene (ET) related genes in 12 dpi syncytia of Col0 and *lsd1(Col0)* plants. Genes were divided into three functional groups: marker genes, signaling, biosynthesis. Genes differentially expressed in transcriptome data present bolded.

| **Function** | **Gene** | **Accession no.** | **Log2FC** | | **Log2FC** | |
|---|---|---|---|---|---|---|
| | | | **Col0** | **p-value** | **lsd1(Col0)** | **p-value** |
| Jasmonic acid (JA) | | | | | | |
| Marker | *PDF1.2* | At5g44420 | 0,933 | 0,1308422 | 0,522 | 0,2412968 |
| | *PDF2.1* | At2g02120 | **0,639** | **0,0183975** | **1,232** | **4,06E-05** |
| | *PR3* | At3g12500 | -0,200 | 0,345067 | **0,699** | **0,0014728** |
| | *PR4/HEL* | At3g04720 | -0,128 | 0,5965495 | 0,234 | 0,2527143 |
| | *THI2.1* | At1g72260 | 0,216 | 0,4981015 | 0,495 | 0,2065587 |
| Signalling | *COI1* | At2g39940 | -0,247 | 0,2438883 | -0,264 | 0,2063272 |
| | *JAR1* | At2g46370 | -0,192 | 0,3980509 | 0,144 | 0,7027459 |
| | *JAZ1* | At1g19180 | **0,966** | **0,0018641** | 0,62 | 0,1137719 |
| | *JAZ5* | At1g17380 | **2,209** | **3,19E-20** | **1,351** | **0,0038343** |
| | *JAZ6* | At1g72450 | **1,550** | **1,21E-09** | **1,351** | **0,0038343** |
| | *JAZ8* | At1g30135 | **1,435** | **0,0002534** | **1,422** | **0,0080657** |
| | *JAZ10* | At5g13220 | **3,621** | **3,45E-29** | **2,455** | **2,34E-16** |
| Biosynthesis | *AOC1* | At3g25760 | **3,143** | **8,92E-13** | 0,842 | 0,1126373 |
| | *AOC3* | At3g25780 | **1,765** | **2,65E-11** | **1,487** | **4,69E-06** |
| | *AOC4* | At1g13280 | -0,112 | 0,6237259 | -0,361 | 0,1612409 |
| | *DDE2* | At5g42650 | **0,818** | **0,0022679** | 0,301 | 0,3296245 |
| | *LOX2* | At3g45140 | 0,753 | 0,1130193 | 0,402 | 0,3595181 |
| | *LOX3* | At1g17420 | **2,641** | **2,96E-24** | **2,738** | **5,45E-10** |
| | *LOX4* | At1g72520 | **2,766** | **1,88E-37** | **1,818** | **5,07E-05** |
| | *LOX5* | At3g22400 | -0,426 | 0,0812774 | -0,504 | 0,1028233 |
| | *LOX6* | At1g67560 | **0,956** | **1,90E-05** | 1,123 | 0,0007534 |
| Salicylic acid (SA) | | | | | | |
| Marker | *PR1* | At2g14610 | -0,740 | 0,2004696 | **1,629** | **2,51E-05** |
| | *PR2* | At3g57260 | 0,661 | 0,1685574 | **1,969** | **0,0002115** |
| | *PR5* | At1g75040 | **1,290** | **0,0026548** | **2,394** | **7,20E-06** |
| Signalling | *EDS1* | At3g48090 | -0,235 | 0,281574 | 0,105 | 0,6852671 |
| | *EDS5* | At4g39030 | **1,182** | **2,99E-06** | **0,725** | **0,008045** |
| | *NPR1* | At4g26120 | -0,060 | 0,8440157 | 0,003 | 0,9940736 |
| | *NPR3* | At5g45110 | **0,644** | **0,0214997** | 0,452 | 0,2426548 |
| | *NPR4* | At4g19660 | -0,425 | 0,0663996 | -0,283 | 0,2569571 |
| | *PBS3* | At5g13320 | 0,315 | 0,4254392 | 0,791 | 0,1242446 |
| Biosynthesis | *EPS1* | At5g67160 | **0,788** | **0,0054264** | 0,303 | 0,3189124 |
| | *ICS1/EDS16* | At1g74710 | -0,315 | 0,36478 | 0,456 | 0,1233578 |
| | *ICS2* | At1g18870 | **-0,818** | **0,0060831** | **-1,796** | **8,40E-05** |
| | *PAL1* | At2g37040 | **0,899** | **5,99E-05** | **1,32** | **0,0024843** |
| | *PAL2* | At3g53260 | **0,472** | **0,0408146** | **1,248** | **0,0028396** |
| | *PAL3* | At5g04230 | -0,265 | 0,5967316 | -0,25 | 0,6329567 |

| | | | | | | |
|---|---|---|---|---|---|---|
| | *PAL4* | At3g10340 | **-0,442** | **0,0409101** | -0,006 | 0,9855601 |
| Ethylene (ET) | | | | | | |
| Marker | *PDF1.2* | At5g44420 | 0,933 | 0,1308422 | 0,522 | 0,2412968 |
| | *PR3* | At3g12500 | -0,200 | 0,345067 | **0,699** | **0,0014728** |
| | *PR4/HEL* | At3g04720 | -0,128 | 0,5965495 | 0,234 | 0,2527143 |
| Signalling | *EIL1* | At2g27050 | -0,188 | 0,4376982 | -0,32 | 0,1774092 |
| | *EIN2* | At5g03280 | -0,176 | 0,4708317 | **-0,471** | **0,0466092** |
| | *EIN3* | At3g20770 | -0,025 | 0,9019934 | -0,042 | 0,857345 |
| | *EER4* | At1g17440 | 0,204 | 0,4140033 | 0,227 | 0,3418443 |
| | *EER5* | At2g19560 | -0,336 | 0,1376128 | -0,179 | 0,4661075 |
| | *ERF1* | At3g23240 | **1,688** | **2,98E-06** | 0,49 | 0,2641865 |
| | *ERF3* | At1g50640 | -0,146 | 0,511132 | 0,01 | 0,9622668 |
| | *ERF4* | At3g15210 | 0,037 | 0,8889884 | -0,095 | 0,7634813 |
| | *ERF5* | At5g47230 | -0,338 | 0,3029006 | -0,705 | 0,0568453 |
| | *ERF6* | At4g17490 | -0,340 | 0,340005 | **-0,893** | **0,0490702** |
| | *ERF7* | At3g20310 | -0,364 | 0,1259982 | -0,078 | 0,7271611 |
| | *ERF13* | At2g44840 | **1,159** | **0,0010723** | 0,153 | 0,7477557 |
| Biosynthesis | *ACS2* | At1g01480 | 0,573 | 0,0927995 | **1,475** | **0,0003515** |
| | *ACS4* | At2g22810 | **1,229** | **0,0478245** | 0,5 | 0,1905501 |
| | *ACS6* | At4g11280 | -0,067 | 0,9095863 | -0,211 | 0,6874027 |
| | *ACS7* | At4g26200 | 0,581 | 0,0920129 | **1,704** | **1,47E-09** |
| | *ACS8* | At4g37770 | **1,776** | **0,0042864** | **2,219** | **1,55E-05** |
| | *ACS9/ETO3* | At3g49700 | -0,586 | 0,2164371 | -0,15 | 0,5209781 |
| | *ACS11* | At4g08040 | **-1,223** | **0,0223648** | -0,687 | 0,1989541 |
| | *ETO1* | At3g51770 | -0,212 | 0,355245 | 0,013 | 0,9511515 |
| | *ETO2/ACS5* | At5g65800 | **-2,110** | **1,37E-07** | -0,449 | 0,3273597 |

**Supplementary Figure 8** – Anatomy of syncytia induced in wild-type (Col0) and *lsd1* (Col0) mutant roots. Light microscopy images of cross-sections of syncytia at 12 dpi induced in Col0 (A-C) and *lsd1* (Col0) (D-E). Cross-sections are taken through the widest part of syncytia, remote from the nematode head. Scalebars - 20 µm.

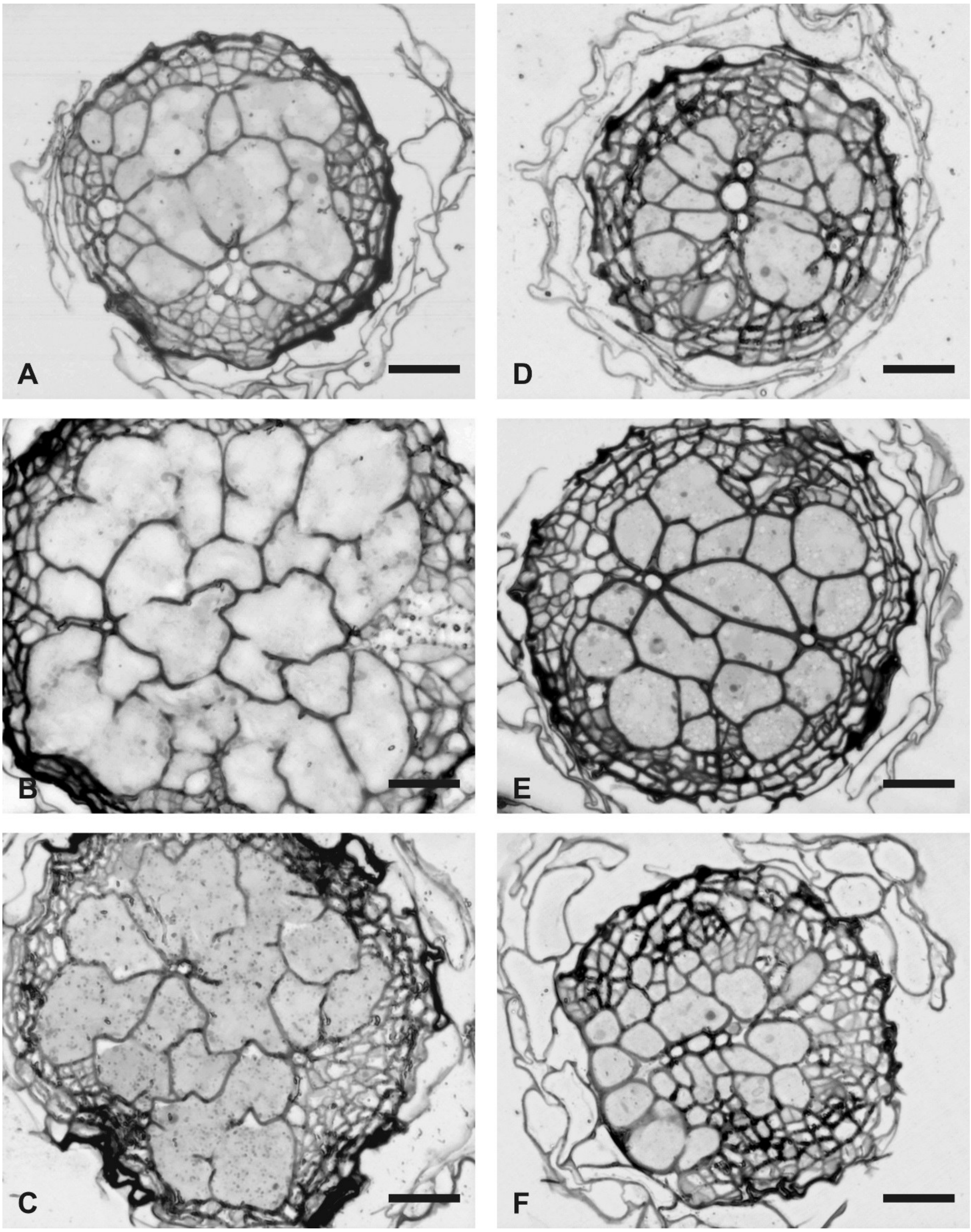

Supplement: Supplementary file 3 [file Image_1.PDF]
